# Regulation of gene expression by MF63, a selective inhibitor of microsomal PGE synthase 1 (mPGES1) in human osteoarthritic chondrocytes

**DOI:** 10.1111/bph.15142

**Published:** 2020-08-10

**Authors:** Lauri Tuure, Antti Pemmari, Mari Hämäläinen, Teemu Moilanen, Eeva Moilanen

**Affiliations:** ^1^ The Immunopharmacology Research Group, Faculty of Medicine and Health Technology Tampere University and Tampere University Hospital Tampere Finland; ^2^ Coxa Hospital for Joint Replacement Tampere Finland

**Keywords:** mPGES‐1, prostaglandins, gene expression, osteoarthritis, chondrocytes

## Abstract

**Background and Purpose:**

mPGES1 catalyses the production of PGE_2_, the most abundant prostanoid related to inflammation and pain in arthritis. mPGES1 is suggested to be a safer and more selective drug target in inflammatory conditions compared to the COX enzymes inhibited by NSAIDs. In the present study, we investigated the effects of the selective mPGES1 inhibitor MF63 on gene expression in primary human chondrocytes from patients with osteoarthritis (OA).

**Experimental Approach:**

Chondrocytes were isolated from articular cartilage obtained from osteoarthritis patients undergoing knee replacement surgery. The effects of MF63 were studied in the primary chondrocytes with RNA‐sequencing based genome‐wide expression analysis. The main results were confirmed with qRT‐PCR and compared with the effects of the NSAID ibuprofen. Functional analysis was performed with the GO database and interactions between the genes were studied with STRING.

**Key Results:**

MF63 enhanced the expression of multiple metallothionein 1 (MT1) isoforms as well as endogenous antagonists of IL‐1 and IL‐36. The expression of IL‐6, by contrast, was down‐regulated. These genes were also essential in functional and interaction network analyses. The effects of MF63 were consistent in qRT‐PCR analysis, whereas the effects of ibuprofen overlapped only partly with MF63. There were no evident findings of catabolic effects by MF63.

**Conclusion and Implications:**

Metallothionein 1 has been suggested to have anti‐inflammatory and protective effects in cartilage. Up‐regulation of the antagonists of IL‐1 superfamily and down‐regulation of the pro‐inflammatory cytokine IL‐6 also support novel anti‐inflammatory and possibly disease‐modifying effects of mPGES1 inhibitors in arthritis.

**Abbreviations**
ADAMTS6: a disintegrin and metalloproteinase with thrombospondin motif 6
COX: cyclooxygenase
FC: fold change
GDF5: growth differentiation factor 5
GO: gene ontology
HAS1: hyaluronan synthase 1
IGFBP 4: insulin‐like growth factor binding protein 4
mPGES1: microsomal PGE synthase1
MT: metallothionein
NGS: next‐generation sequencing
NSAID: non‐steroidal anti‐inflammatory drug
OA: osteoarthritis
PG: prostaglandin
RA: rheumatoid arthritis
RIPK4: receptor interacting serine/threonine kinase 4

**What is already known**

- mPGES1 is a novel target for treating inflammation and pain with improved tolerability compared to NSAIDs.
- Disease‐modifying effects of mPGES1 inhibitors in osteoarthritic cartilage remain unknown and those of NSAIDs inconsistent.

**What does this study add**

- Genome‐wide expression analysis of the effects of the mPGES‐1 inhibitor MF63 in osteoarthritic chondrocytes is shown.
- MF63 enhanced the expression of anti‐inflammatory metallothioneins, and endogenous IL‐1 and IL‐36 antagonists while it suppressed pro‐inflammatory IL‐6.

**What is the clinical significance**

- mPGES1 inhibitors appear not to risk the maintenance of cartilage in arthritis.
- mPGES1 inhibitors may have disease‐modifying effects in osteoarthritis.

## 1 INTRODUCTION

Non‐steroidal anti‐inflammatory drugs (NSAIDs) have been used for decades to treat inflammatory pain related to arthritis and various other inflammatory conditions. They are among the most frequently prescribed and consumed drugs worldwide (Wongrakpanich, Wongrakpanich, Melhado, & Rangaswami, 2018) and have a key role in the recommended drug therapy for osteoarthritis (OA) (Majeed, Sherazi, Bacon, & Bajwa, 2018). The mechanism of action of NSAIDs is based on inhibition of COX‐1 and COX‐2 enzymes, which leads to reduced production of prostaglandins (PGs; Warner & Mitchell, 2004). In general, therapeutic effects of NSAIDs (both non‐selective and COX‐2 selective) are mainly considered to result from decreased production of PGE_2_ which is the most important prostaglandin mediating inflammation and inflammatory pain (Kawahara, Hohjoh, Inazumi, Tsuchiya, & Sugimoto, 2015). Unfortunately, the adverse effects of these drugs result from the same mechanism, that is, inhibition of COX enzymes and consequently reduced production of physiologically important PGs other than PGE_2_ (Patrono & Baigent, 2017). COX‐2 selective NSAIDs (coxibs) were developed in order to avoid the gastrointestinal side effects related to traditional NSAIDs. However, they were shown to increase the risk of cardiovascular hazards (Baron et al., 2008), which was later discovered to concern also traditional NSAIDs depending on the drug and dosage (Nissen et al., 2016).

Microsomal prostaglandin E synthase 1 (mPGES1) is an inducible enzyme that specifically catalyses the production of PGE_2_ in various cells (Koeberle & Werz, 2015; Samuelsson, Morgenstern, & Jakobsson, 2007). In inflammation, the expression of mPGES1 is increased in many tissues, such as synovium in rheumatoid arthritis (RA) patients (Westman et al., 2004) and cartilage in osteoarthritis (OA) patients (Li et al., 2005). Therefore, mPGES1 has been evaluated as an interesting drug target for various inflammatory diseases in order to decrease or even circumvent the typical adverse effects of NSAIDs. Selective mPGES1 inhibitors have been characterised (Koeberle & Werz, 2015; Korotkova & Jakobsson, 2014; Nikolaou, Kokotou, Limnios, & Kokotos, 2017) and some of these, particularly the phenanthrene imidazole MF63, have been investigated in inflammatory models. For instance, MF63 has been shown to attenuate monosodium iodoacetate‐induced arthritis and hyperalgesia in guinea pigs without gastrointestinal toxicity typical for NSAIDs (Xu et al., 2008).

Osteoarthritis is a chronic inflammatory disease with high prevalence as well as individual and economic burden (Glyn‐Jones et al., 2015). Inflammatory and degradative processes in cartilage are characteristic for osteoarthritis. Cytokines, such as IL‐1β, IL‐6 and TNF‐α, enhance the production of inflammatory factors and cartilage‐degrading enzymes in chondrocytes (Haseeb & Haqqi, 2013). On the other hand, osteoarthritic chondrocytes also produce anti‐inflammatory mediators and anabolic factors which preserves the cartilage from pathology. The progression of cartilage degradation in osteoarthritis may thus depend on the balance between the responses of chondrocytes and synoviocytes to pro‐ and anti‐inflammatory mediators and oxidative stress in the joint (Berenbaum, 2013; Wojdasiewicz, Poniatowski, & Szukiewicz, 2014). The molecular pathophysiology of osteoarthritis is poorly known in its complexity and in spite of intensive research, pharmacological therapies with confirmed disease‐modifying properties are not available.

In addition to their pain‐relieving effects, NSAIDs have been suggested to have disease‐modifying effects in osteoarthritic cartilage. Some chondroprotective effects have been reported (Nakata et al., 2018), whereas detrimental effects on cartilage have also been presented. These include, for instance, increased chondrocyte apoptosis in animals receiving long‐term ibuprofen or indomethacin treatment (Ou et al., 2012). This indicates that the data concerning the disease‐modifying potential of NSAIDs in osteoarthritis is controversial.

mPGES1 has primarily been investigated as a safer option (as compared to NSAIDs) for treating inflammation and inflammatory pain (Korotkova & Jakobsson, 2014). Whether selective inhibitors of mPGES‐1 affect inflammation, metabolism and/or maintenance in human cartilage is not known. Therefore, we investigated the effects of the selective mPGES1 inhibitor MF63 on gene expression in human osteoarthritic chondrocytes by carrying out a genome‐wide expression analysis.

## 2 METHODS

### 2.1 Cartilage and cell culture

Cartilage was obtained from patients with osteoarthritis (two series of 10 patients) undergoing knee replacement surgery at Coxa Hospital for Joint Replacement, Tampere, Finland. Patients with diagnosis of diabetes were excluded from this study in order to avoid confounding metabolic changes related to diabetes (Louati, Vidal, Berenbaum, & Sellam, 2015). The patients fulfilled the American College of Rheumatology classification criteria for osteoarthritis (Altman et al., 1986). The patient characteristics are presented in Table 1. The study was approved by the Ethics Committee of Tampere University Hospital, Tampere, Finland and carried out in accordance with the Declaration of Helsinki. Written informed consent was obtained from the patients.

**TABLE 1:** Characteristics of the OA patients whose cartilage was used in the next‐generation sequencing (NGS) and in the quantitative reverse transcription polymerase chain reaction (qRT‐PCR) experiments

NGS			qRT‐PCR		
Age	Sex	BMI (kg·m^−2^)	Age	Sex	BMI (kg·m^−2^)
78	F	26.4	64	F	27.2
71	F	24.9	69	F	24.2
69	M	40.6	59	M	28.7
55	F	26.4	53	F	33.1
60	M	24.0	57	F	24.1
51	F	41.7	76	F	19.0
57	F	41.4	73	F	24.2
78	F	25.2	76	F	25.5
87	M	23.5	85	M	23.8
81	M	25.9	51	F	42.3

Abbreviations: BMI, body mass index; F, female; M, male.

Full‐thickness pieces of articular cartilage from femoral condyles, tibial plateaus and patellar surfaces were removed aseptically from subchondral bone with a scalpel, cut into small pieces and washed with PBS. Chondrocytes were then isolated by enzyme digestion for 16 h at 37°C in a shaker by using a collagenase enzyme blend (0.25 mg·ml^−1^ Liberase™ Research Grade medium; Roche, Mannheim, Germany). Isolated chondrocytes were plated on 24‐well plates (2.0 × 10^5^ cells·ml^−1^) in the culture medium DMEM (Sigma‐Aldrich, St. Louis, MO, USA) supplemented with penicillin (100 U·ml^−1^), streptomycin (100 μg·ml^−1^) and amphotericin B (250 ng·ml^−1^) (all three from Gibco/Life Technologies, Carlsbad, CA, USA), containing 10% fetal bovine serum (FBS, Lonza, Verviers, Belgium) and cultured for 24 h before conducting the experiments. During the experiments, the cells from cartilage samples were treated with and without IL‐1β (R&D Systems Europe Ltd, Abingdon, UK) together with MF63 (Cayman Chemical, Ann Arbor, MI, USA) or ibuprofen (Sigma‐Aldrich).

### 2.2 Next‐generation sequencing and data analysis

After incubation for 24 h, the culture medium was removed, the cells were homogenised and RNA was extracted using RNAeasy Mini Kit (Qiagen, Hilden, Germany) with on‐column DNase digestion according to the manufacturer's instructions. The concentration and integrity of RNA were confirmed with the 2100 Bioanalyzer (Agilent, Santa Clara, CA, USA). Sequencing of the samples from 10 patients (*n* = 10) was performed in the Biomedicum Functional Genomics Unit, University of Helsinki, Finland, using the Illumina NextSeq 500 system (RRID:SCR_014983). Sequencing depth was 15 million single‐end reads 75 bp in length. Read quality was first assessed using FastQC (Andrews, 2010; RRID:SCR_014583) and the reads were trimmed using Trimmomatic (Bolger, Lohse, & Usadel, 2014; RRID:SCR_011848). Trimmed reads were aligned to reference human genome with STAR (Dobin et al., 2013; RRID:SCR_015899). Count matrices were prepared with the featureCounts program (Liao, Smyth, & Shi, 2014) and differential expression was assessed using a generalised linear model implemented in edgeR (Robinson, McCarthy, & Smyth, 2010; McCarthy, Chen, & Smyth, 2012; RRID:SCR_012919) using patient number and treatment as experimental factors.

Normalised gene expression levels are represented by trimmed mean of M‐values (TMM). Functional gene analysis was performed by using the Gene Ontology (GO) database (Ashburner et al., 2000; The Gene Ontology Consortium, 2017) with the DAVID tool (da Huang, Sherman, & Lempicki, 2009; RRID:SCR_001881). Interactions between differentially expressed genes were analysed with the STRING database (Szklarczyk et al., 2015; RRID:SCR_005223).

### 2.3 Quantitative reverse transcription polymerase chain reaction (qRT‐PCR)

RNA was extracted as described above and reverse transcribed to cDNA with Maxima First Strand cDNA Synthesis Kit (Thermo Fisher Scientific, Waltham, MA, USA). Quantitative PCR was performed using TaqMan Universal Master Mix and ABI Prism 7500 sequence detection system (Applied Biosystems, Foster City, CA, USA). The PCR cycling parameters were incubation at 50°C for 2 min, incubation at 95°C for 10 min and thereafter 40 cycles of denaturation at 95°C for 15 s and annealing and extension at 60°C for 1 min. Primers and probes for GAPDH and IL‐6 were purchased from Metabion (Martinsried, Germany). Their sequences were optimised according to the manufacturer's guidelines in TaqMan Universal PCR Master Mix Protocol part number 4304449 revision C (Applied Biosystems) and are presented in Table 2. Expressions of GAPDH and IL‐6 were quantified using the standard curve method as described in the Applied Biosystems User Bulletin. The mRNA levels of metallothionein 1 (MT1) subtypes, IL‐1 receptor antagonist and IL‐36 receptor antagonist were determined with TaqMan Gene Expression assays (Thermo Fisher Scientific; Table 3) by using the 2^−ΔΔCt^ method. When calculating results, the mRNA expression levels were first normalised against GAPDH.

**TABLE 2:** Primer and probe sequences used in the qRT‐PCR experiments

Gene		Sequence
*IL‐6*	Forward	TACCCCCAGGAGAAGATTCCA
	Reverse	CCGTCGAGGATGTACCGAATT
	Probe	CGCCCCACACAGACAGCCACTC
*GAPDH*	Forward	AAGGTCGGAGTCAACGGATTT
	Reverse	GCAACAATATCCACTTTACCAGAGTTAA
	Probe	CGCCTGGTCACCAGGGCTGC

**TABLE 3:** TaqMan gene expression assays used in the qRT‐PCR experiments

Gene	Assay number
*IL1RN*	Hs00893626_m1
*IL36RN*	Hs01104220_g1
*MT1F*	Hs00744661_sH
*MT1X*	Hs00745167_sH
*MT1M*	Hs00828387_g1
*MT1H*	Hs00823168_g1
*MT1G*	Hs01584215_g1
*MT1B*	Hs00538861_m1

### 2.4 ELISA

Enzyme linked immunosorbent assay (ELISA) was used to detect prostaglandin levels in the cell culture medium samples. All ELISA kits were purchased from Cayman Chemical and the measurement protocols were carried out in accordance with the manufacturer's instructions.

### 2.5 Statistical analysis

The data and statistical analysis comply with the recommendations of the *British Journal of Pharmacology* on experimental design and analysis in pharmacology (Curtis et al., 2018). For Next‐Generation Sequencing (NGS) data analysis, normalisation was performed and differential expression was studied using a generalised linear model implemented in edgeR (McCarthy et al., 2012; Robinson et al., 2010) using patient number and treatment as experimental factors. Elsewhere, repeated measures ANOVA or Wilcoxon matched‐pairs signed‐ranks test with Bonferroni's post‐test was performed using GraphPad InStat version 3.10 for Windows (RRID:SCR_000306). Post hoc tests were only run if *F* achieved <0.05. The declared group sizes (*n* = 10) are the numbers of independent values (different patients). Results are presented as mean + SEM. Differences were considered significant at *P* < 0.05.

### 2.6 Nomenclature of targets and ligands

Key protein targets and ligands in this article are hyperlinked to corresponding entries in http://www.guidetopharmacology.org, the common portal for data from the IUPHAR/BPS Guide to PHARMACOLOGY (Harding et al., 2018) and are permanently archived in the Concise Guide to PHARMACOLOGY 2019/20 (Alexander et al., 2019).

## 3 RESULTS

**FIGURE 1:**
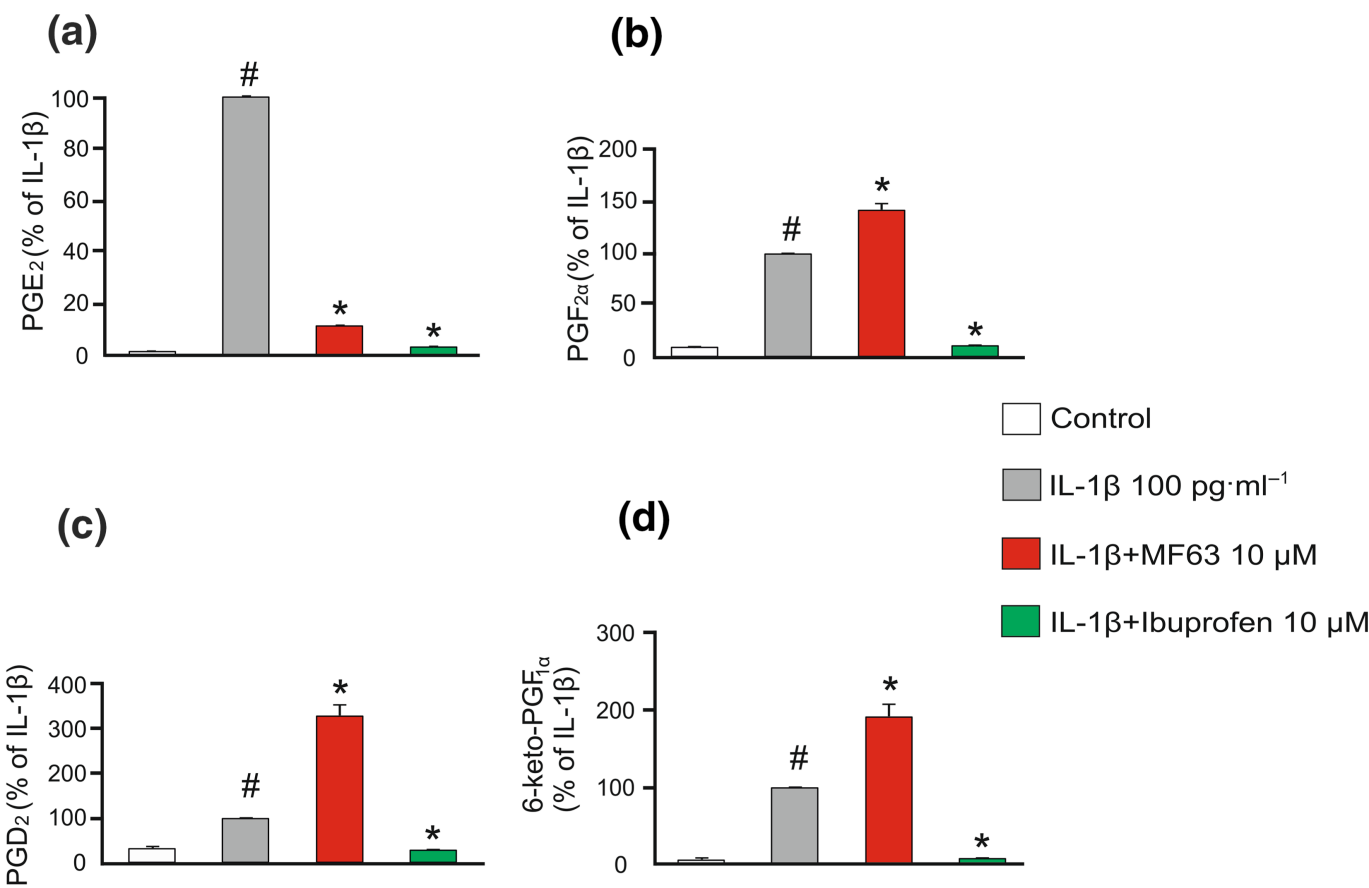
Effects of the mPGES‐1 inhibitor MF63 and the NSAID ibuprofen on the production of PGE_2_ (a), PGF_2α_ (b), PGD_2_ (c) and 6‐keto‐PGF_1α_, (d) in primary human osteoarthritic chondrocytes. Cells were treated with the compounds under investigation for 24 h. The PG levels were determined by ELISA. The PG level in IL‐1β‐treated cells was set as 100% and the other values were related to that. The results from 10 patients (*n* = 10) were combined and expressed as mean + SEM. Wilcoxon matched‐pairs signed‐ranks test with Bonferroni's post‐test was performed and statistical significance is indicated as ^*#*^ *P* < 0.05 as compared to control and ^*^ *P* < 0.05 as compared to the IL‐1β‐stimulated cells

In order to confirm the differential effects of MF63 and ibuprofen on prostaglandin production, we compared the effects of MF63 and ibuprofen on the production of PGE_2_, PGF_2α_, PGD_2_ and 6‐keto‐PGF_1α_ (a metabolite of prostacyclin) in osteoarthritic chondrocytes. Both MF63 and ibuprofen significantly inhibited PGE_2_ production as expected (Figure 1a). While ibuprofen duly decreased the production of the three other PGs as well, MF63 oppositely enhanced the production of PGF_2α_, PGD_2_ and 6‐keto‐PGF_1α_ (Figure 1b–d).

In the genome‐wide expression analysis, 39 protein‐coding genes were found to be up‐ or down‐regulated with fold change (FC) ≥ 2.0 into either direction by the mPGES‐1 inhibitor MF63 in chondrocytes stimulated with the osteoarthritis related cytokine IL‐1β. Top 25 most up‐ and down‐regulated protein‐coding genes by MF63 with their known functions are presented in Tables 4 and 5, respectively.

**TABLE 4:** The 25 most strongly up‐regulated genes by MF63 in IL‐1β‐stimulated primary OA chondrocytes

Gene	Name	Function	FC	Mean (IL‐1β)	Mean (IL‐1β + MF63)	FDR‐adjusted *P*‐value
*MT1B*	Metallothionein 1B	Regulation of metal homeostasis	**3.68**	0.9	3.5	<0.0001
*XIRP1*	Xin actin binding repeat containing 1	Striated muscle protein	**3.43**	5.6	18.8	<0.0001
*MT1G*	Metallothionein 1G	Regulation of metal homeostasis	**3.20**	2,106.6	6,716.4	<0.0001
*MT1H*	Metallothionein 1H	Regulation of metal homeostasis	**2.90**	208.7	609.2	<0.0001
*MT1M*	Metallothionein 1 M	Regulation of metal homeostasis	**2.73**	291.7	793.8	<0.0001
*MT1X*	Metallothionein 1X	Regulation of metal homeostasis	**2.57**	429.8	1,109.7	<0.0001
*SLC30A2*	Solute carrier family 30 member 2	Zinc transporter	**2.70**	16.1	42.1	<0.0001
*SYT7*	Synaptotagmin 7	Exocytosis in synaptic membranes	**2.39**	0.3	0.7	<0.0001
*MT1F*	Metallothionein 1F	Regulation of metal homeostasis	**2.35**	178.8	425.3	<0.0001
*C2CD4B*	C2 calcium‐dependent domain containing 4B	Unknown	**2.34**	0.4	0.9	<0.0001
*PCDH8*	Protocadherin 8	CNS‐specific cell adhesion	**2.30**	0.2	0.5	0.00173
*PTPRR*	Protein tyrosine phosphatase, receptor type R	Cell growth and differentiation	**2.27**	0.3	0.7	<0.0001
*BEAN1*	Brain expressed associated with NEDD4 1	Purkinje cell development	**2.20**	0.2	0.6	0.00057
*SLAMF7*	SLAM family member 7	Immune response	**2.18**	0.6	1.3	<0.0001
*IL36RN*	IL‐36 receptor antagonist	Anti‐inflammatory	**2.15**	116.8	240.4	<0.0001
*P2RY1*	Purinergic receptor P2Y1	Platelet aggregation	**2.12**	0.3	0.6	0.0004
*VSTM2A*	V‐set and transmembrane domain containing 2A	Adipogenesis	**2.09**	0.8	1.6	<0.0001
R*P1*	RP1, axonemal microtubule associated	Morphogenesis of retinal rod photoreceptors	**2.07**	0.3	0.7	0.00087
*FAM189A2*	Family with sequence similarity 189 member A2	Unknown	**2.04**	4.1	8.4	<0.0001
*TCHH*	Trichohyalin	Structural protein in hair follicles and filiform papillae of the tongue	**2.04**	1.8	3.8	<0.0001
*DSCAML1*	DS cell adhesion molecule like 1	Cell adhesion and neuronal differentiation	**2.02**	0.3	0.5	0.0009
*MT1A*	Metallothionein 1A	Regulation of metal homeostasis	**2.02**	51.2	102.3	<0.0001
*IL1RN*	IL‐1 receptor antagonist	Anti‐inflammatory	**2.01**	23.3	45.4	<0.0001
*MT1E*	Metallothionein 1E	Regulation of metal homeostasis	**2.00**	1,365.0	2,729.0	<0.0001
*CHST8*	carbohydrate sulfotransferase 8	Protein sulfation	**1.96**	0.5	1.0	<0.0001

*Note*: Gene expression levels in chondrocytes are given as mean in TMM‐normalised values and the differences between the groups are given as fold change (FC) values. The *P*‐values are adjusted by the false discovery rate (FDR). Functions of the genes were obtained from NCBI Gene database.

**TABLE 5:** The 25 most strongly down‐regulated genes by MF63 in IL‐1β‐stimulated primary OA chondrocytes

Gene	Name	Function	FC	Mean (IL‐1β)	Mean (IL‐1β + MF63)	*FDR‐adjusted P‐value*
*CYP1B1*	Cytochrome P450 family 1 subfamily B member 1	Drug metabolism and lipoprotein synthesis	**0.34**	779.6	280.5	<0.0001
*IGFBP4*	Insulin‐like growth factor binding protein 4	Regulation of insulin‐like growth factors	**0.34**	209.2	70.8	<0.0001
*FADS2*	Fatty acid desaturase 2	Lipid metabolism	**0.36**	19.6	6.7	<0.0001
*ALDH3A1*	Aldehyde dehydrogenase 3 Family member A1	Metabolism of aldehyde substrates	**0.38**	5.2	2.1	<0.0001
*CYP1A1*	Cytochrome P450 family 1 subfamily A member 1	Metabolism of drugs, xenobiotics and lipoproteins	**0.38**	0.6	0.2	0.0025
*LDLR*	Low density lipoprotein receptor	Lipid metabolism	**0.40**	65.4	26.4	<0.0001
*DHCR7*	7‐dehydrocholesterol reductase	Lipid metabolism	**0.41**	39.8	16.0	<0.0001
*GDF5*	Growth differentiation factor 5	Development of cartilage, bone, brown fat and neuronal tissue	**0.42**	7.6	3.2	<0.0001
*SHISA3*	Shisa family member 3	Suppressor of FGF and WNT signalling	**0.44**	2.1	0.9	<0.0001
*HRCT1*	Histidine rich carboxyl terminus 1	Unknown	**0.46**	0.6	0.3	0.0004
*TMEM97*	Transmembrane protein 97	Lipid metabolism	**0.46**	10.3	4.4	<0.0001
*MSMO1*	Methylsterol monooxygenase 1	Lipid metabolism	**0.46**	98.3	44.8	<0.0001
*RIPK4*	Receptor interacting serine/threonine kinase 4	Intracellular signalling and keratinocyte differentiation	**0.47**	6.7	3.1	<0.0001
*CIDEA*	Cell death‐inducing DFFA‐like effector A	Regulation of apoptosis, thermogenesis and lipolysis	**0.47**	1.2	0.6	<0.0001
*GGT5*	Gamma‐glutamyltransferase 5	Leukotriene metabolism	**0.49**	0.7	0.3	0.0010
*HAL*	Histidine ammonia‐lyase	Histidine catabolism	**0.52**	1.7	0.9	<0.0001
*SLC14A1*	Solute carrier family 14 member 1 (Kidd Blood Group)	Urea transporter	**0.53**	1.5	0.8	<0.0001
*CDA*	Cytidine deaminase	Pyrimidine metabolism	**0.53**	4.5	2.4	<0.0001
*IER3*	Immediate early response 3	Regulation of apoptosis	**0.53**	1.3	0.7	<0.0001
*PACSIN3*	Protein kinase C and casein kinase substrate in neurons 3	Neuron development	**0.54**	5.0	2.6	<0.0001
*IL6*	IL‐6	Immune response	**0.54**	938.0	519.5	<0.0001
*HAS1*	Hyaluronan synthase 1	ECM component	**0.54**	0.7	0.4	0.01238
*NDC80*	NDC80, kinetochore complex component	Chromosome segregation	**0.54**	0.9	0.5	0.00017
*ADAMTS6*	ADAM metallopeptidase with thrombospondin type 1 motif 6	ECM degradation	**0.55**	10.2	5.8	<0.0001
*CPNE5*	Copine 5	Mediator of calcium‐dependent intracellular processes	**0.56**	0.6	0.3	0.00856

*Note*: Gene expression levels in chondrocytes are given as mean in TMM‐normalised values and the differences between the groups are given as fold change (FC) values. The *P*‐values are adjusted by the false discovery rate (FDR). Functions of the genes were obtained from NCBI Gene database.

Next, we studied the functional gene categories that were enriched among the genes that were most differently expressed between the IL‐1β‐stimulated chondrocytes treated with or without MF63. We identified three GO terms related to these genes, namely, “cellular response to zinc ion,” “negative regulation of growth,” and “cellular response to cadmium ion,” as shown in Table 6. All of the genes picked up by the GO analysis to include in the three functional categories were subtypes of metallothionein 1 (MT1), which were all significantly up‐regulated by MF‐63 (Table 4).

**TABLE 6:** GO terms covering the 25 most strongly up‐regulated and the 25 most strongly down‐regulated genes in IL‐1 β‐stimulated primary human osteoarthritis chondrocytes treated with the mPGES‐1 inhibitor MF63 as compared to the cells without MF63

GO term	Number of altered genes	Altered genes	Number of genes in the GO term	FDR‐adjusted *P*‐value
Cellular response to zinc ion	8	*MT1M, MT1A, MT1E, MT1B, MT1H, MT1X, MT1G, MT1F*	19	4.19E‐11
Negative regulation of growth	8	*MT1M, MT1A, MT1E, MT1B, MT1H, MT1X, MT1G, MT1F*	19	4.19E‐09
Cellular response to cadmium ion	6	*MT1A, MT1E, MT1H, MT1X, MT1G, MT1F*	17	9.29E‐07

*Note*: The genes were analysed with DAVID tools using Gene Ontology (GO) database. The *P*‐values are adjusted by the false discovery rate (FDR). The gene names and functions are presented in Tables 3 and 4.

We also investigated the effects of MF63 on gene expression in chondrocytes in the absence of added IL‐1β. The number of significantly altered genes by MF63 was smaller as compared to the cells cultured with IL‐1β and none of the genes was altered by fold change ≥ 2.0 into either direction. Metallothionein 1 subtypes were widely up‐regulated, whereas the down‐regulated genes were mainly those associated with lipid metabolism. Comprehensive lists of the differentially expressed genes by MF63 in cells cultured with and without IL‐1β are provided in Tables S1 and S2, respectively. Moreover, a list of genes whose expression was significantly changed by the IL‐1β stimulation *per se* and a list of effects of MF63 on selected genes involved in the pathogenesis of osteoarthritis are available in Tables S3 and S4.

**FIGURE 2:**
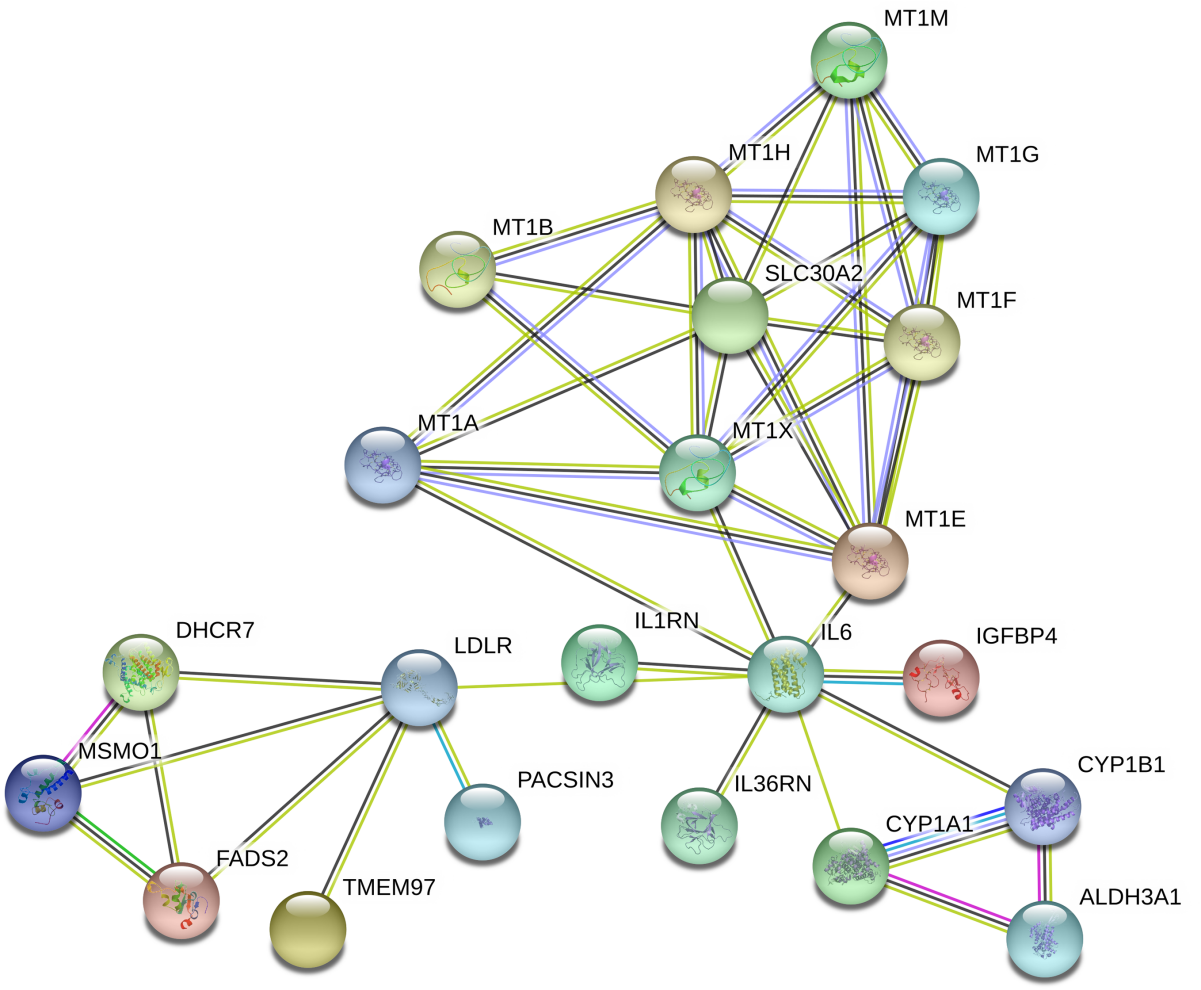
Interactions among the 25 most strongly up‐regulated and 25 most strongly down‐regulated genes in IL‐1β‐stimulated human osteoarthritic chondrocytes by MF63. The gene names and functions are presented in Tables 3 and 4. Genes with no interactions are excluded from the graph. Colours of the edges: green, activation; blue, binding; black, chemical reaction; red, inhibition; violet, catalysis; pink, posttranslational modification

Among the genes whose expression was significantly affected in IL‐1β‐stimulated chondrocytes by MF63, we found some interactions using the STRING database. There were a few genes identified to have a junctional position, as indicated by many interactions with others. Particularly *IL6* (encoding the cytokine IL‐6) was among the genes most strongly down‐regulated by MF63 and seemed to interact with both down‐regulated genes, such as LDL receptor and other genes associated with lipid metabolism and up‐regulated genes, such as metallothionein 1 subtypes (Figure 2).

With reference to Tables 4, 5 and S2, we noticed that several subtypes of metallothionein 1, which have been suggested to have a protective role in inflammatory arthritis (Kim et al., 2014; Sun et al., 2018), were significantly up‐regulated by MF63 in a manner independent of IL‐1β. In addition, Tables 4 and 5 of up‐ and down‐regulated genes included some other genes that have previously been associated with arthritis and inflammation in general. The up‐regulation of *IL36RN* (encoding IL‐36 receptor antagonist) and *IL1RN* (encoding IL‐1 receptor antagonist ) (Table 4), as well as the down‐regulation of *IL6* (Table 5), were particularly interesting in this context. Therefore, we wanted to confirm these effects by qRT‐PCR analysis and to contrast whether the commonly used NSAID, ibuprofen, also had an effect on these genes. To do this, we incubated human osteoarthritic chondrocytes from another group of patients (*n* = 10) with MF63 and ibuprofen and measured their effects on the mRNA production of the genes of interest.

**FIGURE 3:**
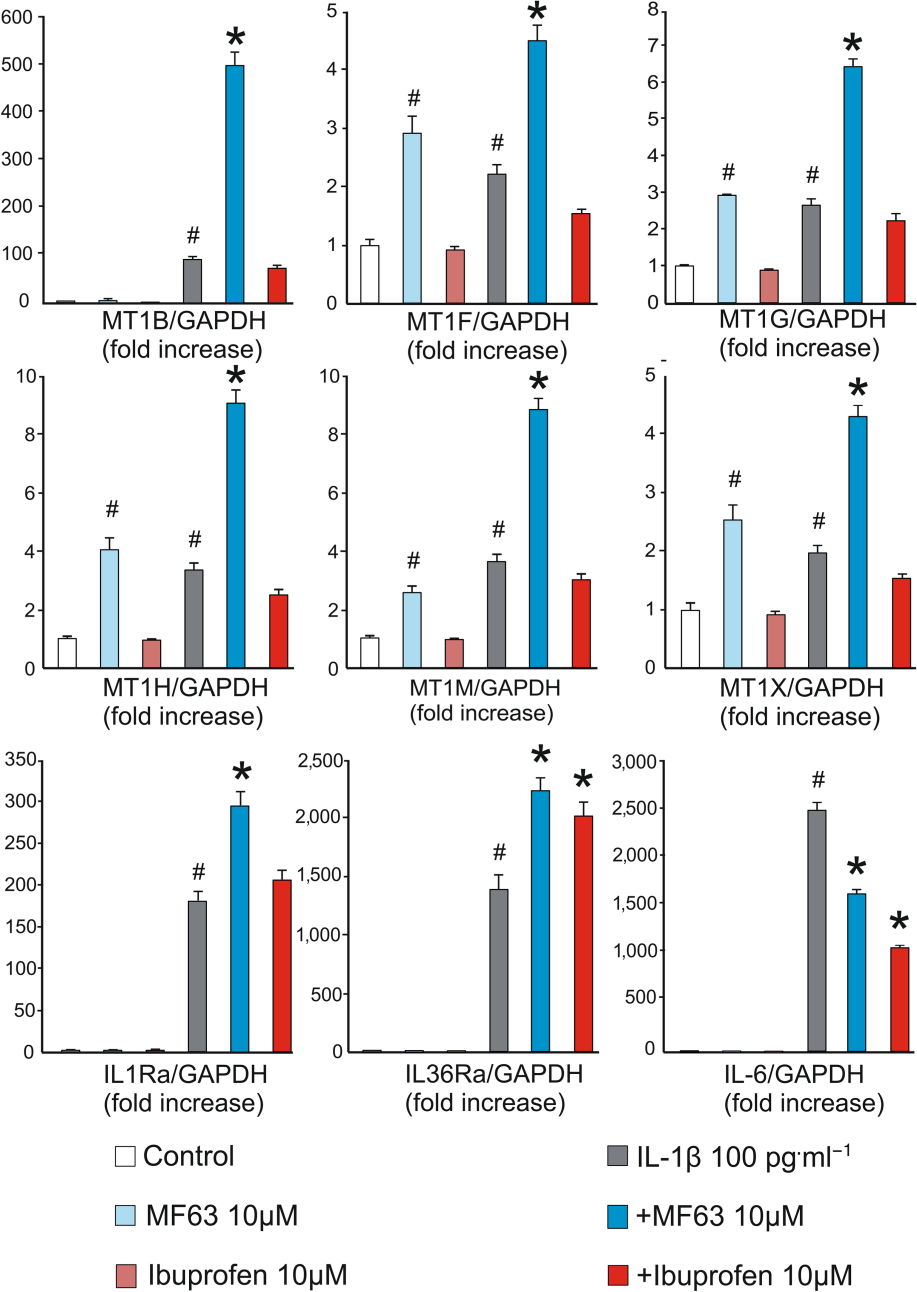
Effects of the mPGES1 inhibitor MF63 and the NSAID ibuprofen on the mRNA expression levels of MT1 subtypes, IL‐6, IL1 receptor antagonist and IL36 receptor antagonist in primary human osteoarthritic chondrocytes as determined by qRT‐PCR. Cells were treated with or without IL‐1β and the compounds under investigation for 24 h. mRNA expression of the genes investigated was measured by quantitative RT‐PCR and normalised against GAPDH mRNA levels. Results are expressed as mean + SEM in arbitrary units, mRNA expression levels in unstimulated control cells were set as 1 and the other values were related to that. The results from 10 patients (*n* = 10) were combined and the experiments were carried out in duplicate. Repeated measures ANOVA with Bonferroni's post‐test was performed and statistical significance is indicated as ^#^ *P* < 0.05 as compared to control and ^*^ *P* < 0.05 as compared to the IL‐1β‐stimulated cells

MF63 significantly increased the expression of metallothionein 1 subtypes, IL‐1 receptor antagonist and IL‐36 receptor antagonist, in the IL‐1β‐stimulated chondrocytes, while IL‐6 was down‐regulated (Figure 3). These results were consistent with the fold change values in the NGS analysis (Tables 4 and 5). Accordingly, MT1 subtypes F, G, H, M and X were up‐regulated by MF63 also in the cells cultured without IL‐1β. In contrast, ibuprofen had no effect on metallothionein 1 subtypes in the unstimulated cells and rather inhibited the expression of the metallothionein 1 subtypes in the IL‐1β‐stimulated chondrocytes (Figure 3). Ibuprofen did not affect the expression of IL‐1 receptor antagonist either, but IL‐36 receptor antagonist was moderately up‐regulated. IL‐6 expression was also similarly decreased by ibuprofen as by MF63.

## 4 DISCUSSION AND CONCLUSIONS

The purpose of the present study was to investigate the effects of the mPGES‐1 inhibitor MF63 on human osteoarthritic chondrocytes by using genome‐wide expression analysis. When evaluating both fold change and expression levels, up‐regulation of metallothionein 1 subtypes, IL‐1 and IL‐36 receptor antagonists, as well as down‐regulation of *IL‐6* were highlighted among the genes altered by MF63 (Tables 4 and 5). The main gene expression changes caused by MF63 were confirmed with qRT‐PCR in samples from another group of patients (Figure 3). The same genes were represented in the STRING analysis showing interactions between the other affected genes and metallothionein 1 subtypes were present under the three GO terms enriched in the analysis of functional gene categories (Table 6).

Metallothioneins are small cysteine‐rich proteins which are ubiquitously expressed in eukaryotic cells. They function as intracellular antioxidants and participate in detoxification and intracellular transportation of heavy metal ions (Ziller & Fraissinet‐Tachet, 2018). These known functions explain the high counts of genes related to cellular responses to zinc and cadmium seen among the detected GO terms (Table 6). Human MTs exist in several isoforms (Inoue, Takano, Shimada, & Satoh, 2009): There are four major groups (MT1–4), of which metallothionein 1 is further divided into eight subgroups. The specific functions of these metallothionein 1 subtypes are not, however, well known. Mice have only a single gene encoding metallothionein 1, indicating a close structural and possibly functional homogeneity among the metallothionein 1 subgroups. This is also suggested by the STRING analysis, where protein homology and co‐expression between the metallothionein 1 subtypes was indicated (Figure 2).

Metallothionein 1 and metallothionein 2 are induced by inflammatory stress factors (Coyle, Philcox, & Rofe, 1995). Accordingly, increased metallothionein 1 levels have been reported in plasma from rheumatoid arthritis patients (Sun et al., 2018), as well as in synovial fluid and cartilage in experimental mouse models of arthritis (Sun et al., 2018; Won et al., 2016). Interestingly, exogenous metallothionein 1 has been reported to suppress inflammation and cytokine expression in experimental models of arthritis (Sun et al., 2018). The hypothesis of metallothionein 1 as a protective factor in arthritis is further supported by the finding that cartilage destruction was enhanced in mice deficient of metallothionein 1 (Kim et al., 2014).

The connection between mPGES1 and metallothioneins has not been previously addressed. It is therefore interesting that many of the human metallothionein 1 subtypes were represented among the genes that were most significantly up‐regulated by MF63 in both IL‐1β‐stimulated and unstimulated chondrocytes, whereas that effect was not shared by ibuprofen. Moreover, the expression levels (counts) of metallothionein 1 subtypes were among the highest in the list of the genes most strongly up‐regulated by MF63 (Table 4). *SLC30A2*, which encodes a zinc exporter protein ZnT2, was also up‐regulated by MF63 and interacts with metallothionein 1 (Figure 2). Zinc influx is accordingly associated with the severity of osteoarthritis (Kim et al., 2014), which suggests that up‐regulation of the efflux protein might be beneficial for cartilage.

The results concerning the metallothioneins with highest fold change values were confirmed by qRT‐PCR analysis using cartilage samples from another group of osteoarthritis patients (Figure 3). As stated above, metallothionein 1 potentially protects from cartilage degradation that is typical for chronic arthritis. Thus, it might be suggested that mPGES1 inhibitors might inhibit arthritis progression in addition to their effects (based on reduced PGE_2_ production) in inflammation and pain in general. Up‐regulation of endogenous IL‐1 and IL‐36 receptor antagonists, as well as down‐regulation of *IL‐6*, support this view. The effects of MF63 on the up‐regulation of IL‐36 receptor antagonist and down‐regulation of IL‐6 were shared by ibuprofen, suggesting a mechanism based on decreased PGE_2_ production.

MF63 was found to enhance the production of PGs other than PGE_2_ (Figure 1). mPGES‐1 inhibition has also previously been associated with increased production of PG metabolites other than PGE_2_, possibly due to substrate shunting (Koeberle & Werz, 2015). It could be hypothesised that this perturbation in PG production could explain the observed differences in gene expression between MF63 and ibuprofen. This is an intriguing avenue of future study.

IL‐1 promotes the degradation of cartilage in osteoarthritis by enhancing the production of proteolytic enzymes and by enhancing the production of other inflammatory cytokines (Daheshia & Yao, 2008; Kapoor, Martel‐Pelletier, Lajeunesse, Pelletier, & Fahmi, 2011). IL‐1 receptor antagonist (encoded by *IL1RN*) effectively counteracts these effects (Arend & Dayer, 1990). It is therefore interesting that the expression of IL1‐antagonist was significantly increased by MF63 in the present study. Recombinant IL1‐antagonist (anakinra) is used in the treatment of RA. It has also been investigated as a disease‐modifying treatment for osteoarthritis, but the studies have not demonstrated sustained beneficial effects. This might be due to poor bioavailability (Chevalier et al., 2009). The pro‐inflammatory cytokine IL‐36 belongs to the IL‐1 family (Bassoy, Towne, & Gabay, 2018). It has mainly been associated with inflammatory diseases of epithelial tissues, but it also enhances the production of other pro‐inflammatory cytokines in human chondrocytes (Magne et al., 2006). IL‐36Ra prevents the binding of IL‐36 to its receptor and therefore prevents its effects, possibly contributing to anti‐inflammatory signalling in cartilage.

The expression of *IL‐6* was decreased in the IL‐1β‐stimulated chondrocytes treated by MF63 and the effect was shared by ibuprofen (Figure 3). IL‐6 also had a central position in the STRING network analysis (Figure 2). IL‐6 is a well‐described cytokine with diverse functions in innate and adaptive immunity (Tanaka, Narazaki, & Kishimoto, 2014). It is also a specific drug target in RA and plays a role in the pathophysiology of many other inflammatory diseases, including osteoarthritis (Schett, 2018). In cartilage, it promotes the production of degradative enzymes, such as MMP‐1 and MMP‐13 (Haseeb & Haqqi, 2013). As an interesting detail, increased levels of MT1 (especially that of MT1G) have been proposed as a factor to predict favourable therapeutic response to anti‐IL‐6 treatment (tocilizumab) in RA patients (Sanayama et al., 2014).

The gene expression between healthy and osteoarthritic cartilage has been compared in different settings to detect genes that might be associated with the pathogenesis of osteoarthritis. Karlsson et al. compared gene expression profiles between osteoarthritis‐affected and healthy cartilage samples with a genome‐wide microarray analysis (Karlsson et al., 2010). Among the up‐regulated genes in OA cartilage, *IGFBP 4* (insulin‐like growth factor binding protein 4) matches the list of genes down‐regulated by MF63 in the present study. In another genome‐wide microarray‐based analysis comparing gene expression between osteoarthritis‐affected and preserved cartilage, 19 genes were found to be differentially expressed with at least twofold shift, including *PTGES* which encodes mPGES‐1 (Ramos et al., 2014). There were no similarities between the results of differentially expressed genes in the osteoarthritis‐affected versus preserved cartilage and our results concerning the most significant effects of MF63 on gene expression. This suggests that MF63 does not have harmful effects on cartilage through up‐regulating the expression of osteoarthritis‐affected genes in chondrocytes. On the contrary, expression of *ADAMTS6* (a disintegrin and metalloproteinase with thrombospondin motif 6), which is overexpressed in osteoarthritis cartilage (Ramos et al., 2014) and might contribute to cartilage degradation (Yang, Chanalaris, & Troeberg, 2017), was down‐regulated by MF63 (Table 5).

Genes related to cholesterol metabolism were widely down‐regulated by MF63. This is an interesting finding, as osteoarthritis has been associated with increased cholesterol levels in serum (de Munter, van der Kraan, van den Berg, & van Lent, 2016) and in chondrocytes (Ali et al., 2016). Of the other genes down‐regulated by MF63 in the present study, growth differentiation factor 5 (*GDF5*) might possibly contribute to the pathogenesis of osteoarthritis as it has been shown to inhibit cartilage degradation in a rat osteoarthritis model (Parrish et al., 2017). In addition, growth differentiation factor 5 polymorphisms have been associated with an increased risk for knee osteoarthritis (Ozcan et al., 2017). HAS1 (hyaluronan synthase 1) is one of the three enzymes recognised to be responsible for the synthesis of hyaluronan, the most abundant polysaccharide in extracellular matrix of cartilage tissues. *HAS1* expression is induced by pro‐inflammatory cytokines, is overexpressed in osteoarthritic joints (David‐Raoudi et al., 2009) and its deficiency has been shown to exacerbate injury‐induced cartilage damage and joint inflammation in mice (Chan et al., 2015). Therefore, it cannot be ruled out that down‐regulation of *HAS1* by MF63 could result in reduced cartilage regeneration. On the other hand, receptor interacting serine/threonine kinase 4 (*RIPK4*) was found among the down‐regulated genes. It has been associated with the regulation of chondrocyte proliferation, apoptosis and disease progression in osteoarthritic cartilage (Zou, Liu, & Lu, 2018). Silencing of *RIPK4* with siRNA was found to enhance the proliferation and inhibit the apoptosis of chondrocytes in vitro. Altogether, the expression levels of *GDF5*, *RIPK4* and especially that of *HAS1* were relatively low (Table 5), questioning the clinical significance of the observed drug effects on the expression of these genes. It is overall important to notice that although numerous differentially expressed genes between the chondrocytes treated with and without MF63 were found (Tables S1 and S2), the vast majority of them was altered with relatively small fold change and/or expressed at very low levels, which necessarily has to be acknowledged when interpreting the clinical relevance of these changes.

In conclusion, our results introduce new potential anti‐inflammatory mechanisms associated with mPGES1 inhibitors. In the genome‐wide expression analysis, we identified that metallothionein 1 subtypes, and IL‐1 and IL‐36 receptor antagonists, were among the most strongly up‐regulated genes in human osteoarthritic chondrocytes treated with the mPGES1 inhibitor MF63. These genes also showed high levels of expression. There were no distinct evidence of increased expression of cartilage‐degrading enzymes or catabolic factors by MF63. However, *IL‐6* expression was significantly down‐regulated. According to our analysis, we suggest that mPGES1 might not only be an interesting drug target for novel “NSAID‐like” drugs, but inhibition of mPGES1 might also possess disease‐modifying properties in arthritis.

## AUTHOR CONTRIBUTIONS

L.T., A.P., M.H., T.M. and E.M. contributed to the design of the study and to the acquisition, analysis and interpretation of the data. E.M. conceived and supervised the study. L.T. drafted the manuscript and all authors revised the manuscript critically for important intellectual content and have approved the final version of the manuscript for submission.

## CONFLICT OF INTEREST

The authors declare no conflicts of interest.

## DECLARATION OF TRANSPARENCY AND SCIENTIFIC RIGOUR

This Declaration acknowledges that this paper adheres to the principles for transparent reporting and scientific rigour of preclinical research as stated in the *BJP* guidelines for Design and Analysis and as recommended by funding agencies, publishers and other organisations engaged with supporting research.

## Supporting information

Table S1. Supporting information

Table S2. Supporting information

Table S3. Supporting information

Table S4. Supporting information
