# Supplementary material for: Regulation of gene expression by MF63, a selective inhibitor of microsomal PGE synthase 1 (mPGES1) in human osteoarthritic chondrocytes
Source: Br J Pharmacol. 2020 Aug 10;177(18):4134–46. doi: 10.1111/bph.15142 (PMC7443472; doi:10.1111/bph.15142)
Supplement: Supplementary file 1 — Table S1. Supporting information [file BPH-177-4134-s001.pdf]

| Gene | Description | FC | adj. P | Mean (IL1) | Mean (IL1+MF) |
|---|---|---|---|---|---|
| MT1B | metallothionein 1B | **3,676** | 0 | 0,332 | 1,852 |
| MT1HL1 | metallothionein 1H like 1 | **3,608** | 0 | 0,055 | 0,511 |
| XIRP1 | xin actin binding repeat containing 1 | **3,428** | 0 | 4,597 | 15,196 |
| MT1G | metallothionein 1G | **3,197** | 0 | 1802,230 | 5608,510 |
| MT1H | metallothionein 1H | **2,897** | 0 | 186,831 | 485,131 |
| MT1JP | metallothionein 1J, pseudogene | **2,800** | 0 | 1,052 | 2,490 |
| MT1M | metallothionein 1M | **2,727** | 0 | 246,375 | 576,309 |
| SLC30A2 | solute carrier family 30 member 2 | **2,696** | 0 | 21,214 | 49,867 |
| MT1X | metallothionein 1X | **2,575** | 0 | 361,919 | 976,839 |
| MT1XP1 | metallothionein 1X pseudogene 1 | **2,491** | 0 | 3,933 | 11,685 |
| SYT7 | synaptotagmin 7 | **2,388** | 1,00E-06 | 0,166 | 0,128 |
| MT1L | metallothionein 1L, pseudogene | **2,382** | 0 | 87,240 | 112,503 |
| MT1DP | metallothionein 1D, pseudogene | **2,369** | 0 | 2,271 | 3,065 |
| MT1F | metallothionein 1F | **2,353** | 0 | 170,103 | 311,268 |
| C2CD4B | C2 calcium dependent domain containing 4B | **2,342** | 2,00E-06 | 0,277 | 1,788 |
| PCDH8 | protocadherin 8 | **2,299** | 0,000228 | 0,388 | 0,638 |
| PTPRR | protein tyrosine phosphatase, receptor type R | **2,273** | 5,00E-06 | 0,332 | 0,511 |
| LINC00944 | long intergenic non-protein coding RNA 944 | **2,214** | 0,00015 | 0,222 | 0,447 |
| BEAN1 | brain expressed associated with NEDD4 1 | **2,204** | 6,20E-05 | 0,332 | 0,319 |
| SLAMF7 | SLAM family member 7 | **2,181** | 0 | 1,163 | 0,638 |
| IL36RN | interleukin 36 receptor antagonist | **2,150** | 0 | 168,331 | 289,304 |
| LINC02257 | long intergenic non-protein coding RNA 2257 | **2,124** | 9,00E-06 | 0,388 | 0,638 |
| P2RY1 | purinergic receptor P2Y1 | **2,124** | 4,70E-05 | 0,277 | 0,575 |
| HES7 | hes family bHLH transcription factor 7 | **2,119** | 0,00016 | 0,222 | 0,447 |
| MT1P3 | metallothionein 1 pseudogene 3 | **2,098** | 0 | 1,662 | 2,362 |
| VSTM2A | V-set and transmembrane domain containing 2A | **2,095** | 0 | 0,665 | 2,299 |
| RP1 | RP1, axonemal microtubule associated | **2,070** | 0,000102 | 0,554 | 0,638 |
| FAM189A2 | family with sequence similarity 189 member A2 | **2,045** | 0 | 2,936 | 7,343 |
| TCHH | trichohyalin | **2,042** | 0 | 1,163 | 4,469 |
| DSCAML1 | DS cell adhesion molecule like 1 | **2,022** | 0,000116 | 0,111 | 0,702 |
| LINC02392 | long intergenic non-protein coding RNA 2392 | **2,021** | 0,000402 | 0,332 | 0,383 |

| | | | | | |
|---|---|---|---|---|---|
| MT1A | metallothionein 1A | **2,016** | 0 | 38,552 | 86,644 |
| IL1RN | interleukin 1 receptor antagonist | **2,012** | 0 | 39,549 | 52,804 |
| DNM1P35 | dynamin 1 pseudogene 35 | **2,005** | 4,40E-05 | 0,166 | 0,638 |
| MT1E | metallothionein 1E | **1,997** | 0 | 1100,381 | 2091,467 |
| CHST8 | carbohydrate sulfotransferase 8 | **1,961** | 1,00E-06 | 0,222 | 0,383 |
| EGR4 | early growth response 4 | **1,954** | 0,000286 | 0,277 | 0,638 |
| PEG10 | paternally expressed 10 | **1,949** | 0 | 24,039 | 48,973 |
| RAPGEFL1 | Rap guanine nucleotide exchange factor like 1 | **1,944** | 0 | 7,035 | 7,981 |
| AIRE | autoimmune regulator | **1,937** | 0 | 2,493 | 8,300 |
| CCRL2 | C-C motif chemokine receptor like 2 | **1,935** | 0 | 21,768 | 28,732 |
| ANKRD1 | ankyrin repeat domain 1 | **1,934** | 0,000582 | 0,665 | 0,319 |
| IL36B | interleukin 36, beta | **1,929** | 0 | 50,737 | 40,353 |
| SGK2 | SGK2, serine/threonine kinase 2 | **1,927** | 0,000579 | 0,111 | 1,341 |
| DIRC3 | disrupted in renal carcinoma 3 | **1,927** | 0,00869 | 0,000 | 0,447 |
| TAGLN3 | transgelin 3 | **1,925** | 6,20E-05 | 0,222 | 0,638 |
| POU3F1 | POU class 3 homeobox 1 | **1,923** | 0 | 1,496 | 4,406 |
| RASGRP1 | RAS guanyl releasing protein 1 | **1,923** | 0 | 7,921 | 18,389 |
| ADRA2B | adrenoceptor alpha 2B | **1,920** | 0,000574 | 0,166 | 0,192 |
| MSANTD1 | Myb/SANT DNA binding domain containing 1 | **1,915** | 0,006335 | 0,055 | 0,575 |
| SBSN | suprabasin | **1,890** | 0 | 17,503 | 45,589 |
| IL36G | interleukin 36, gamma | **1,878** | 0 | 53,396 | 63,722 |
| CLIC5 | chloride intracellular channel 5 | **1,876** | 0 | 1,163 | 1,405 |
| SNAP91 | synaptosome associated protein 91 | **1,871** | 0,005216 | 0,332 | 0,830 |
| SLFNL1-AS1 | SLFNL1 antisense RNA 1 | **1,859** | 0 | 0,277 | 1,724 |
| ICAM5 | intercellular adhesion molecule 5 | **1,847** | 0 | 4,320 | 6,513 |
| HMOX1 | heme oxygenase 1 | **1,844** | 0 | 1150,398 | 1510,050 |
| CECR6 | cat eye syndrome chromosome region, candidate 6 | **1,841** | 0,000539 | 0,332 | 0,575 |
| PIF1 | PIF1 5'-to-3' DNA helicase | **1,833** | 0 | 3,434 | 4,278 |
| MAP1LC3C | microtubule associated protein 1 light chain 3 gamma | **1,828** | 1,00E-06 | 0,443 | 1,341 |
| DOK7 | docking protein 7 | **1,825** | 0 | 0,886 | 2,107 |
| ARC | activity regulated cytoskeleton associated protein | **1,817** | 0 | 54,947 | 134,532 |
| GPR158 | G protein-coupled receptor 158 | **1,815** | 0 | 0,609 | 3,576 |
| FLG | filaggrin | **1,810** | 0 | 1,108 | 2,235 |

| | | | | | |
|---|---|---|---|---|---|
| BRINP1 | BMP/retinoic acid inducible neural specific 1 | **1,808** | 0,002144 | 0,443 | 0,511 |
| PLPPR4 | phospholipid phosphatase related 4 | **1,807** | 0,005807 | 0,055 | 0,575 |
| ABCG1 | ATP binding cassette subfamily G member 1 | **1,804** | 0,000672 | 0,277 | 1,660 |
| PCDH19 | protocadherin 19 | **1,800** | 0 | 2,991 | 9,194 |
| VIPR1 | vasoactive intestinal peptide receptor 1 | **1,799** | 0,000127 | 0,222 | 1,085 |
| KCNMB1 | potassium calcium-activated channel subfamily M regulatory beta subunit 1 | **1,798** | 0 | 9,416 | 22,156 |
| LRFN5 | leucine rich repeat and fibronectin type III domain containing 5 | **1,796** | 0 | 0,775 | 2,235 |
| RGS4 | regulator of G protein signaling 4 | **1,796** | 1,50E-05 | 1,440 | 0,894 |
| PIP5K1B | phosphatidylinositol-4-phosphate 5-kinase type 1 beta | **1,788** | 0,00349 | 0,166 | 0,575 |
| CNTN4 | contactin 4 | **1,783** | 2,00E-06 | 0,443 | 1,532 |
| SERPINB2 | serpin family B member 2 | **1,781** | 0 | 3,379 | 9,833 |
| TMC6 | transmembrane channel like 6 | **1,773** | 0,004559 | 0,222 | 0,702 |
| SYT9 | synaptotagmin 9 | **1,769** | 0,000535 | 0,554 | 1,149 |
| SPINK1 | serine peptidase inhibitor, Kazal type 1 | **1,769** | 1,00E-06 | 1,108 | 2,554 |
| CAMK2A | calcium/calmodulin dependent protein kinase II alpha | **1,768** | 0 | 0,388 | 4,980 |
| PPP1R36 | protein phosphatase 1 regulatory subunit 36 | **1,766** | 0,000671 | 0,166 | 0,511 |
| DUSP2 | dual specificity phosphatase 2 | **1,765** | 0 | 2,049 | 6,577 |
| PLIN1 | perilipin 1 | **1,763** | 3,90E-05 | 0,499 | 0,319 |
| SULT1C2 | sulfotransferase family 1C member 2 | **1,762** | 0 | 0,997 | 1,277 |
| FAM186B | family with sequence similarity 186 member B | **1,758** | 0,000463 | 0,388 | 0,511 |
| LCE1C | late cornified envelope 1C | **1,757** | 1,50E-05 | 0,332 | 1,149 |
| PPARGC1B | PPARG coactivator 1 beta | **1,750** | 0,000165 | 0,554 | 0,638 |
| RHOV | ras homolog family member V | **1,746** | 0,007862 | 0,166 | 0,702 |
| LINC01705 | long intergenic non-protein coding RNA 1705 | **1,732** | 0 | 1,662 | 1,915 |
| CD80 | CD80 molecule | **1,723** | 0,005058 | 0,332 | 0,766 |
| RPLP0P2 | ribosomal protein lateral stalk subunit P0 pseudogene 2 | **1,721** | 0,000404 | 0,499 | 0,511 |
| THSD7A | thrombospondin type 1 domain containing 7A | **1,716** | 0 | 1,828 | 2,809 |
| ARHGAP25 | Rho GTPase activating protein 25 | **1,715** | 7,80E-05 | 0,388 | 1,085 |
| STK32A | serine/threonine kinase 32A | **1,708** | 1,00E-06 | 0,554 | 1,469 |
| KRT38 | keratin 38 | **1,707** | 0,033811 | 0,000 | 0,192 |
| KRT16 | keratin 16 | **1,705** | 0 | 276,618 | 1117,245 |
| AKAP6 | A-kinase anchoring protein 6 | **1,705** | 0 | 2,493 | 4,980 |
| MT1P1 | metallothionein 1 pseudogene 1 | **1,704** | 6,00E-05 | 0,775 | 0,894 |

| | | | | | |
|---|---|---|---|---|---|
| TRIM63 | tripartite motif containing 63 | **1,701** | 2,00E-06 | 2,049 | 5,108 |
| ACTN2 | actinin alpha 2 | **1,699** | 8,00E-06 | 0,222 | 2,171 |
| RSAD2 | radical S-adenosyl methionine domain containing 2 | **1,694** | 0 | 1,606 | 3,192 |
| LRRC39 | leucine rich repeat containing 39 | **1,694** | 0,027647 | 0,277 | 0,958 |
| CYP4F22 | cytochrome P450 family 4 subfamily F member 22 | **1,692** | 0,001095 | 0,166 | 0,383 |
| ITGAX | integrin subunit alpha X | **1,690** | 0 | 10,358 | 21,390 |
| LINC00702 | long intergenic non-protein coding RNA 702 | **1,687** | 0 | 2,936 | 3,959 |
| DLL4 | delta like canonical Notch ligand 4 | **1,684** | 4,00E-06 | 1,274 | 1,596 |
| PLEK | pleckstrin | **1,682** | 1,00E-06 | 0,665 | 1,405 |
| DISP3 | dispatched RND transporter family member 3 | **1,680** | 0,001905 | 0,332 | 0,255 |
| CGN | cingulin | **1,678** | 0,005369 | 0,332 | 0,511 |
| MYH16 | myosin heavy chain 16 pseudogene | **1,677** | 0 | 1,274 | 3,895 |
| KCNK3 | potassium two pore domain channel subfamily K member 3 | **1,677** | 2,10E-05 | 0,720 | 1,405 |
| PLIN4 | perilipin 4 | **1,673** | 0,003142 | 0,388 | 0,511 |
| MTFR2 | mitochondrial fission regulator 2 | **1,672** | 0,006936 | 0,443 | 0,447 |
| HSP90AA2P | heat shock protein 90 alpha family class A member 2, pseudogene | **1,668** | 0,019278 | 0,055 | 0,383 |
| TAS2R5 | taste 2 receptor member 5 | **1,667** | 0,000805 | 0,332 | 0,894 |
| PRR9 | proline rich 9 | **1,665** | 6,90E-05 | 1,828 | 0,830 |
| LINC00504 | long intergenic non-protein coding RNA 504 | **1,662** | 0,003465 | 0,222 | 0,511 |
| ICAM4 | intercellular adhesion molecule 4 (Landsteiner-Wiener blood group) | **1,653** | 0 | 39,105 | 61,743 |
| EIF3FP3 | eukaryotic translation initiation factor 3 subunit F pseudogene 3 | **1,653** | 0,012974 | 0,388 | 0,064 |
| OSBP2 | oxysterol binding protein 2 | **1,652** | 0 | 3,157 | 7,534 |
| NCF2 | neutrophil cytosolic factor 2 | **1,651** | 1,10E-05 | 0,443 | 2,490 |
| NEFM | neurofilament medium | **1,647** | 0 | 7,367 | 22,156 |
| RIMS3 | regulating synaptic membrane exocytosis 3 | **1,644** | 2,30E-05 | 0,886 | 0,830 |
| ALK | ALK receptor tyrosine kinase | **1,642** | 0,004954 | 0,388 | 0,766 |
| MTSS1 | MTSS1, I-BAR domain containing | **1,640** | 0 | 5,761 | 7,470 |
| KCNA1 | potassium voltage-gated channel subfamily A member 1 | **1,638** | 0 | 1,939 | 2,682 |
| NOS2 | nitric oxide synthase 2 | **1,638** | 0 | 929,502 | 1925,840 |
| IL10RA | interleukin 10 receptor subunit alpha | **1,637** | 0 | 0,665 | 1,660 |
| NPIPB13 | nuclear pore complex interacting protein family, member B13 | **1,637** | 0,019587 | 0,277 | 0,319 |
| LINC01252 | long intergenic non-protein coding RNA 1252 | **1,630** | 0,000645 | 0,443 | 0,447 |
| GRIN2A | glutamate ionotropic receptor NMDA type subunit 2A | **1,629** | 0 | 3,988 | 8,364 |

| | | | | | |
|---|---|---|---|---|---|
| CXCL10 | C-X-C motif chemokine ligand 10 | **1,627** | 0 | 5,650 | 9,386 |
| SCNN1B | sodium channel epithelial 1 beta subunit | **1,626** | 0,010462 | 0,000 | 0,830 |
| PATL2 | PAT1 homolog 2 | **1,624** | 0,001407 | 0,277 | 0,830 |
| SLC30A1 | solute carrier family 30 member 1 | **1,622** | 0 | 300,381 | 395,678 |
| EXTL3-AS1 | EXTL3 antisense RNA 1 | **1,619** | 0,000722 | 0,332 | 1,022 |
| CSF2 | colony stimulating factor 2 | **1,616** | 0 | 7,145 | 7,854 |
| SPNS2 | sphingolipid transporter 2 | **1,610** | 0 | 1,551 | 1,852 |
| KCNK2 | potassium two pore domain channel subfamily K member 2 | **1,609** | 0 | 1,662 | 1,852 |
| SLC6A16 | solute carrier family 6 member 16 | **1,608** | 0,00783 | 0,277 | 0,128 |
| STK33 | serine/threonine kinase 33 | **1,608** | 0,004071 | 0,388 | 0,319 |
| PLIN5 | perilipin 5 | **1,607** | 0,014906 | 0,609 | 0,958 |
| CCNA1 | cyclin A1 | **1,603** | 0 | 1,496 | 3,703 |
| ARHGAP27P1-BP | ARHGAP27P1-BPTFP1-KPNA2P3 readthrough, transcribed pseudogene | **1,600** | 0 | 1,662 | 2,107 |
| EBI3 | Epstein-Barr virus induced 3 | **1,598** | 7,00E-06 | 0,831 | 2,043 |
| IL15 | interleukin 15 | **1,595** | 0,00171 | 0,831 | 0,766 |
| FAM49A | family with sequence similarity 49 member A | **1,593** | 0 | 11,964 | 25,476 |
| MT3 | metallothionein 3 | **1,590** | 0 | 1,440 | 2,235 |
| NAT8L | N-acetyltransferase 8 like | **1,590** | 0,018663 | 0,277 | 0,383 |
| TDRD12 | tudor domain containing 12 | **1,589** | 0,045608 | 0,055 | 0,575 |
| DENND3 | DENN domain containing 3 | **1,588** | 0 | 13,183 | 27,711 |
| KCNJ15 | potassium voltage-gated channel subfamily J member 15 | **1,588** | 0 | 1,496 | 1,405 |
| LINC01359 | long intergenic non-protein coding RNA 1359 | **1,587** | 0,006255 | 0,222 | 0,766 |
| CABP7 | calcium binding protein 7 | **1,586** | 0,022235 | 0,332 | 0,958 |
| RHPN2 | rhophilin Rho GTPase binding protein 2 | **1,580** | 9,00E-06 | 0,831 | 0,766 |
| KYNU | kynureninase | **1,580** | 0 | 12,684 | 10,088 |
| AFDN-AS1 | AFDN antisense RNA 1 (head to head) | **1,576** | 0,000302 | 0,388 | 1,085 |
| RBM24 | RNA binding motif protein 24 | **1,575** | 0,004657 | 0,111 | 0,383 |
| IL19 | interleukin 19 | **1,574** | 0,000121 | 0,443 | 1,213 |
| CSPG4P13 | chondroitin sulfate proteoglycan 4 pseudogene 13 | **1,573** | 0 | 3,157 | 6,321 |
| MAFA | MAF bZIP transcription factor A | **1,572** | 0 | 2,714 | 5,044 |
| CNTNAP3B | contactin associated protein-like 3B | **1,570** | 0,010702 | 0,388 | 0,958 |
| PITPNM3 | PITPNM family member 3 | **1,570** | 0,001001 | 0,831 | 1,341 |
| MT2A | metallothionein 2A | **1,565** | 0 | 6784,630 | 11407,164 |

| | | | | | |
|---|---|---|---|---|---|
| RASSF5 | Ras association domain family member 5 | **1,564** | 0 | 7,921 | 12,834 |
| GEM | GTP binding protein overexpressed in skeletal muscle | **1,562** | 0 | 75,829 | 130,509 |
| CLEC2D | C-type lectin domain family 2 member D | **1,562** | 0 | 19,220 | 32,883 |
| A2MP1 | alpha-2-macroglobulin pseudogene 1 | **1,559** | 0,011653 | 0,222 | 0,575 |
| WNT7B | Wnt family member 7B | **1,558** | 0 | 5,262 | 4,342 |
| CYLD | CYLD lysine 63 deubiquitinase | **1,558** | 0 | 212,089 | 287,644 |
| LSMEM1 | leucine rich single-pass membrane protein 1 | **1,557** | 0 | 1,385 | 3,703 |
| DMGDH | dimethylglycine dehydrogenase | **1,557** | 0,006037 | 0,332 | 1,788 |
| DACT1 | dishevelled binding antagonist of beta catenin 1 | **1,555** | 0 | 2,991 | 4,980 |
| KHDRBS3 | KH RNA binding domain containing, signal transduction associated 3 | **1,552** | 0 | 2,603 | 3,384 |
| LGALSL | galectin like | **1,551** | 0 | 24,704 | 65,063 |
| RGS2 | regulator of G protein signaling 2 | **1,551** | 0 | 58,381 | 43,737 |
| ASIC3 | acid sensing ion channel subunit 3 | **1,550** | 0,000586 | 0,443 | 0,830 |
| PYGM | glycogen phosphorylase, muscle associated | **1,549** | 0,001777 | 0,886 | 1,660 |
| DAPK1 | death associated protein kinase 1 | **1,548** | 0 | 6,591 | 6,193 |
| ARHGAP20 | Rho GTPase activating protein 20 | **1,547** | 0 | 4,376 | 5,746 |
| SYTL2 | synaptotagmin like 2 | **1,547** | 0 | 2,991 | 5,810 |
| USP32P1 | ubiquitin specific peptidase 32 pseudogene 1 | **1,546** | 0,031225 | 0,222 | 1,149 |
| FNIP2 | folliculin interacting protein 2 | **1,546** | 0 | 55,501 | 54,911 |
| KITLG | KIT ligand | **1,545** | 0 | 18,113 | 30,329 |
| MFSD4A | major facilitator superfamily domain containing 4A | **1,545** | 0,039732 | 0,000 | 0,702 |
| HCK | HCK proto-oncogene, Src family tyrosine kinase | **1,544** | 0,000879 | 1,385 | 0,447 |
| PMAIP1 | phorbol-12-myristate-13-acetate-induced protein 1 | **1,540** | 0 | 7,478 | 15,835 |
| SPAG8 | sperm associated antigen 8 | **1,539** | 0,009423 | 0,222 | 0,766 |
| RYBP | RING1 and YY1 binding protein | **1,537** | 0,000101 | 1,052 | 2,235 |
| KIAA1324L | KIAA1324 like | **1,537** | 0 | 7,201 | 10,599 |
| PWAR6 | Prader Willi/Angelman region RNA 6 | **1,536** | 0,003787 | 0,609 | 0,894 |
| KCNC1 | potassium voltage-gated channel subfamily C member 1 | **1,533** | 0,004777 | 0,609 | 0,830 |
| ADAMTSL4-AS1 | ADAMTSL4 antisense RNA 1 | **1,532** | 6,00E-06 | 1,551 | 4,469 |
| ANGPT2 | angiopoietin 2 | **1,532** | 0,009805 | 0,554 | 0,830 |
| NDUFB4P11 | NADH:ubiquinone oxidoreductase subunit B4 pseudogene 11 | **1,532** | 0,024046 | 0,277 | 0,255 |
| LRRC75A | leucine rich repeat containing 75A | **1,532** | 3,00E-05 | 1,108 | 1,915 |
| GFOD1 | glucose-fructose oxidoreductase domain containing 1 | **1,530** | 0 | 14,346 | 29,882 |

| | | | | | |
|---|---|---|---|---|---|
| GABRR2 | gamma-aminobutyric acid type A receptor rho2 subunit | **1,526** | 0,001949 | 0,554 | 1,532 |
| RENBP | renin binding protein | **1,526** | 0,040109 | 0,111 | 0,319 |
| ZNF763 | zinc finger protein 763 | **1,525** | 0,002899 | 0,720 | 0,830 |
| PCDH17 | protocadherin 17 | **1,523** | 0,000134 | 1,052 | 1,724 |
| NPPC | natriuretic peptide C | **1,522** | 0 | 2,160 | 3,129 |
| NUP210 | nucleoporin 210 | **1,521** | 0,016682 | 0,443 | 0,638 |
| ICOSLG | inducible T-cell costimulator ligand | **1,514** | 0 | 10,801 | 0,958 |
| AC110814.1 | Neuron-specific protein family member 1 | **1,514** | 0,043379 | 0,277 | 0,447 |
| TDRD10 | tudor domain containing 10 | **1,512** | 0,043587 | 0,332 | 0,383 |
| REEP1 | receptor accessory protein 1 | **1,512** | 0,000456 | 0,499 | 1,724 |
| DLX3 | distal-less homeobox 3 | **1,511** | 0 | 8,198 | 22,411 |
| KIF5C | kinesin family member 5C | **1,510** | 0,016551 | 0,166 | 0,638 |
| NCKAP1L | NCK associated protein 1 like | **1,510** | 0,000799 | 0,277 | 1,149 |
| MCM10 | minichromosome maintenance 10 replication initiation factor | **1,510** | 0,008689 | 1,274 | 0,702 |
| ANKRD33B | ankyrin repeat domain 33B | **1,508** | 0,001972 | 0,720 | 0,575 |
| GRIN1 | glutamate ionotropic receptor NMDA type subunit 1 | **1,505** | 0,018297 | 0,332 | 0,447 |
| FTCD | formimidoyltransferase cyclodeaminase | **1,505** | 0,020474 | 0,277 | 0,830 |
| HADH | hydroxyacyl-CoA dehydrogenase | **1,504** | 0 | 14,512 | 18,133 |
| FAM81A | family with sequence similarity 81 member A | **1,502** | 0,013389 | 0,443 | 0,830 |
| LGI2 | leucine rich repeat LGI family member 2 | **1,502** | 0,001141 | 0,665 | 1,788 |
| SLC6A17 | solute carrier family 6 member 17 | **1,501** | 0,000252 | 0,942 | 2,171 |
| SLC1A2 | solute carrier family 1 member 2 | **1,501** | 0,037944 | 0,277 | 0,958 |
| EPS8L1 | EPS8 like 1 | **1,501** | 0,030414 | 0,166 | 0,511 |
| PCDHGC5 | protocadherin gamma subfamily C, 5 | **1,501** | 0,001054 | 1,052 | 1,469 |
| CAMK1G | calcium/calmodulin dependent protein kinase IG | **1,501** | 0,065271 | 0,111 | 0,383 |
| BCL2L11 | BCL2 like 11 | **1,500** | 0 | 9,582 | 16,856 |
| COBL | cordon-bleu WH2 repeat protein | **1,500** | 0,000144 | 0,886 | 2,618 |
| CCND2-AS2 | CCND2 antisense RNA 2 | **1,499** | 0,005013 | 0,277 | 0,830 |
| STON2 | stonin 2 | **1,498** | 2,00E-06 | 1,274 | 2,299 |
| SYNE3 | spectrin repeat containing nuclear envelope family member 3 | **1,498** | 0 | 7,145 | 7,087 |
| TAC1 | tachykinin precursor 1 | **1,498** | 0 | 6,259 | 18,261 |
| AKNAD1 | AKNA domain containing 1 | **1,498** | 0,002103 | 0,499 | 0,766 |
| SEMA6A-AS1 | SEMA6A antisense RNA 1 | **1,496** | 0,046735 | 0,111 | 0,447 |

| | | | | | |
|---|---|---|---|---|---|
| TNFRSF10C | TNF receptor superfamily member 10c | **1,495** | 0,0154 | 0,720 | 0,894 |
| KIAA1683 | KIAA1683 | **1,492** | 0 | 6,813 | 6,960 |
| MOXD1 | monooxygenase DBH like 1 | **1,491** | 0 | 111,944 | 133,765 |
| ULBP1 | UL16 binding protein 1 | **1,489** | 0 | 27,252 | 36,905 |
| MEX3B | mex-3 RNA binding family member B | **1,486** | 0,001485 | 1,052 | 1,277 |
| ODF3L1 | outer dense fiber of sperm tails 3 like 1 | **1,485** | 0 | 1,052 | 3,639 |
| NPL | N-acetylneuraminate pyruvate lyase | **1,483** | 0 | 3,323 | 4,916 |
| NOD2 | nucleotide binding oligomerization domain containing 2 | **1,482** | 0 | 65,194 | 107,395 |
| SUSD5 | sushi domain containing 5 | **1,481** | 0 | 113,162 | 103,245 |
| LRRC37B | leucine rich repeat containing 37B | **1,478** | 0 | 7,035 | 10,344 |
| SPOCK2 | SPARC/osteonectin, cwcv and kazal like domains proteoglycan 2 | **1,477** | 0,085393 | 0,388 | 0,638 |
| WIPF3 | WAS/WASL interacting protein family member 3 | **1,476** | 0 | 4,487 | 8,428 |
| WSCD2 | WSC domain containing 2 | **1,476** | 2,80E-05 | 1,274 | 2,873 |
| DUSP15 | dual specificity phosphatase 15 | **1,475** | 6,00E-05 | 1,329 | 1,341 |
| PRSS54 | protease, serine 54 | **1,472** | 0,015837 | 0,332 | 1,405 |
| C3orf80 | chromosome 3 open reading frame 80 | **1,472** | 0,011838 | 0,720 | 0,511 |
| AZGP1 | alpha-2-glycoprotein 1, zinc-binding | **1,472** | 0,006136 | 0,277 | 1,660 |
| RPTN | repetin | **1,472** | 0,000242 | 0,332 | 1,532 |
| IFFO2 | intermediate filament family orphan 2 | **1,472** | 0 | 5,262 | 9,514 |
| CCDC102B | coiled-coil domain containing 102B | **1,471** | 0,046584 | 0,166 | 0,638 |
| HIST1H2BE | histone cluster 1 H2B family member e | **1,471** | 0,000212 | 1,717 | 4,214 |
| KRT8P46 | keratin 8 pseudogene 46 | **1,468** | 0,04766 | 0,388 | 0,511 |
| MMP9 | matrix metallopeptidase 9 | **1,467** | 0,000876 | 0,609 | 2,171 |
| GAS2L3 | growth arrest specific 2 like 3 | **1,465** | 0,019595 | 2,216 | 1,341 |
| BCO2 | beta-carotene oxygenase 2 | **1,462** | 0,003493 | 0,443 | 0,511 |
| HS3ST2 | heparan sulfate-glucosamine 3-sulfotransferase 2 | **1,462** | 0,05296 | 0,166 | 0,319 |
| SELE | selectin E | **1,462** | 0 | 41,875 | 38,310 |
| FMO5 | flavin containing monooxygenase 5 | **1,461** | 0,005308 | 1,052 | 0,255 |
| CMYA5 | cardiomyopathy associated 5 | **1,461** | 1,60E-05 | 8,253 | 16,793 |
| CEBPB-AS1 | CEBPB antisense RNA 1 | **1,460** | 0 | 2,493 | 4,789 |
| SHISA2 | shisa family member 2 | **1,459** | 0 | 2,991 | 11,429 |
| F11R | F11 receptor | **1,458** | 0,02195 | 0,443 | 0,319 |
| TGFBR3 | transforming growth factor beta receptor 3 | **1,458** | 0 | 109,340 | 154,517 |

| | | | | | |
|---|---|---|---|---|---|
| CRABP2 | cellular retinoic acid binding protein 2 | **1,458** | 0,004237 | 0,499 | 0,638 |
| PELI1 | pellino E3 ubiquitin protein ligase 1 | **1,457** | 0 | 55,778 | 94,945 |
| BCL2 | BCL2, apoptosis regulator | **1,456** | 0 | 74,611 | 106,310 |
| NPTX1 | neuronal pentraxin 1 | **1,455** | 0,000211 | 0,609 | 1,788 |
| KIT | KIT proto-oncogene receptor tyrosine kinase | **1,455** | 0,01564 | 0,499 | 0,383 |
| TRIM9 | tripartite motif containing 9 | **1,454** | 0 | 4,043 | 7,981 |
| CLIC2 | chloride intracellular channel 2 | **1,452** | 0 | 5,705 | 14,813 |
| TFCP2L1 | transcription factor CP2 like 1 | **1,451** | 0 | 1,274 | 4,597 |
| FAM110C | family with sequence similarity 110 member C | **1,450** | 0,000166 | 1,440 | 3,192 |
| PPP2R2B | protein phosphatase 2 regulatory subunit Bbeta | **1,449** | 0,012747 | 0,443 | 0,255 |
| NDRG4 | NDRG family member 4 | **1,449** | 0,001365 | 1,052 | 1,341 |
| RANBP3L | RAN binding protein 3 like | **1,448** | 0,018027 | 0,609 | 0,702 |
| SLC43A2 | solute carrier family 43 member 2 | **1,447** | 3,00E-06 | 2,493 | 2,554 |
| HSD17B14 | hydroxysteroid 17-beta dehydrogenase 14 | **1,447** | 2,30E-05 | 2,271 | 2,362 |
| NOV | nephroblastoma overexpressed | **1,447** | 0 | 5,539 | 6,513 |
| GADD45B | growth arrest and DNA damage inducible beta | **1,447** | 0 | 70,069 | 104,011 |
| SOCS1 | suppressor of cytokine signaling 1 | **1,446** | 0 | 5,428 | 8,747 |
| LINC00472 | long intergenic non-protein coding RNA 472 | **1,446** | 0,000171 | 1,163 | 2,299 |
| ABCB1 | ATP binding cassette subfamily B member 1 | **1,445** | 2,00E-06 | 1,385 | 5,363 |
| CDKN1C | cyclin dependent kinase inhibitor 1C | **1,445** | 0,001837 | 0,554 | 1,022 |
| CEBPA | CCAAT/enhancer binding protein alpha | **1,444** | 0,004728 | 0,720 | 1,915 |
| CCNE2 | cyclin E2 | **1,444** | 0,000351 | 1,329 | 1,341 |
| ATP6V1C2 | ATPase H+ transporting V1 subunit C2 | **1,444** | 4,80E-05 | 2,714 | 3,192 |
| HIST1H4I | histone cluster 1 H4 family member i | **1,443** | 0,05411 | 0,443 | 0,511 |
| S1PR1 | sphingosine-1-phosphate receptor 1 | **1,443** | 0 | 4,210 | 13,472 |
| ALOX12P2 | arachidonate 12-lipoxygenase pseudogene 2 | **1,443** | 0,006584 | 0,277 | 1,085 |
| OGFRL1 | opioid growth factor receptor like 1 | **1,443** | 0 | 146,673 | 122,273 |
| GCNT4 | glucosaminyl (N-acetyl) transferase 4, core 2 | **1,443** | 0 | 7,422 | 13,025 |
| MTCO1P12 | mitochondrially encoded cytochrome c oxidase I pseudogene 12 | **1,442** | 0,000138 | 1,496 | 1,788 |
| LAMC2 | laminin subunit gamma 2 | **1,442** | 9,00E-06 | 24,593 | 21,709 |
| PLEKHM1P1 | pleckstrin homology and RUN domain containing M1 pseudogene 1 | **1,442** | 0 | 12,851 | 21,262 |
| CA8 | carbonic anhydrase 8 | **1,440** | 0,060959 | 0,111 | 0,383 |
| SOX17 | SRY-box 17 | **1,439** | 1,00E-06 | 3,877 | 2,171 |

| | | | | | |
|---|---|---|---|---|---|
| PDE11A | phosphodiesterase 11A | **1,439** | 0,042593 | 0,222 | 0,702 |
| ADRA2C | adrenoceptor alpha 2C | **1,438** | 0,006992 | 0,554 | 0,894 |
| RSPH1 | radial spoke head 1 homolog | **1,438** | 0,023594 | 0,554 | 0,511 |
| PCDHGC4 | protocadherin gamma subfamily C, 4 | **1,437** | 0,031375 | 0,831 | 0,447 |
| KRT14 | keratin 14 | **1,436** | 4,10E-05 | 1,994 | 7,215 |
| HSP90B3P | heat shock protein 90 beta family member 3, pseudogene | **1,436** | 0,030702 | 0,388 | 0,128 |
| IFIT2 | interferon induced protein with tetratricopeptide repeats 2 | **1,435** | 0 | 6,314 | 10,216 |
| POLN | DNA polymerase nu | **1,432** | 0,002026 | 0,997 | 0,958 |
| IGSF11 | immunoglobulin superfamily member 11 | **1,432** | 0,016222 | 0,720 | 1,532 |
| DYNLRB2 | dynein light chain roadblock-type 2 | **1,432** | 0,021147 | 0,222 | 0,447 |
| GDF15 | growth differentiation factor 15 | **1,431** | 0 | 71,952 | 159,561 |
| SVILP1 | supervillin pseudogene 1 | **1,431** | 0,034904 | 0,554 | 1,979 |
| CDS1 | CDP-diacylglycerol synthase 1 | **1,431** | 0,004062 | 0,665 | 1,085 |
| LINC00921 | long intergenic non-protein coding RNA 921 | **1,430** | 1,00E-05 | 1,662 | 1,788 |
| MYEF2 | myelin expression factor 2 | **1,430** | 0,072766 | 0,222 | 0,575 |
| ZFP36 | ZFP36 ring finger protein | **1,429** | 0 | 24,815 | 32,755 |
| SCX | scleraxis bHLH transcription factor | **1,429** | 1,00E-06 | 1,108 | 3,256 |
| RHOB | ras homolog family member B | **1,429** | 0 | 87,018 | 183,760 |
| RAB3B | RAB3B, member RAS oncogene family | **1,428** | 0,018264 | 0,609 | 0,319 |
| ZDHHC11B | zinc finger DHHC-type containing 11B | **1,427** | 0,017464 | 0,554 | 1,532 |
| PXDNL | peroxidasin like | **1,426** | 0,007761 | 1,551 | 0,192 |
| LINGO1 | leucine rich repeat and Ig domain containing 1 | **1,426** | 7,00E-06 | 2,603 | 4,278 |
| FYB | FYN binding protein | **1,426** | 0 | 3,379 | 4,086 |
| ISM2 | isthmin 2 | **1,425** | 2,40E-05 | 1,662 | 1,788 |
| GAS7 | growth arrest specific 7 | **1,424** | 0 | 101,364 | 125,210 |
| MATN4 | matrilin 4 | **1,424** | 0,004302 | 3,102 | 1,341 |
| NLRC5 | NLR family CARD domain containing 5 | **1,423** | 3,10E-05 | 2,216 | 4,278 |
| ZNF385C | zinc finger protein 385C | **1,423** | 0 | 12,352 | 26,817 |
| SLCO4A1 | solute carrier organic anion transporter family member 4A1 | **1,422** | 0 | 5,539 | 17,750 |
| RASL12 | RAS like family 12 | **1,422** | 0,006117 | 0,665 | 0,575 |
| MSH4 | mutS homolog 4 | **1,421** | 0,043682 | 0,222 | 0,638 |
| SHE | Src homology 2 domain containing E | **1,420** | 0,002014 | 0,886 | 2,554 |
| ANKRD10 | ankyrin repeat domain 10 | **1,420** | 0 | 44,977 | 67,106 |

| | | | | | |
|---|---|---|---|---|---|
| KBTBD12 | kelch repeat and BTB domain containing 12 | **1,420** | 0,000198 | 0,665 | 2,682 |
| NALCN | sodium leak channel, non-selective | **1,419** | 0,05738 | 0,277 | 0,255 |
| CPT1B | carnitine palmitoyltransferase 1B | **1,419** | 0,070038 | 0,166 | 0,702 |
| SLC28A3 | solute carrier family 28 member 3 | **1,419** | 0 | 1,883 | 23,944 |
| EFHB | EF-hand domain family member B | **1,418** | 0,059507 | 0,720 | 0,511 |
| OLFM2 | olfactomedin 2 | **1,417** | 0,103349 | 0,720 | 0,638 |
| SCNN1D | sodium channel epithelial 1 delta subunit | **1,417** | 0 | 3,213 | 7,407 |
| TUBB3 | tubulin beta 3 class III | **1,416** | 0,085551 | 0,222 | 0,319 |
| ATF3 | activating transcription factor 3 | **1,415** | 0 | 24,759 | 41,758 |
| RAG1 | recombination activating 1 | **1,415** | 0,053778 | 0,222 | 0,575 |
| DNAH9 | dynein axonemal heavy chain 9 | **1,415** | 1,60E-05 | 1,717 | 2,235 |
| SLITRK4 | SLIT and NTRK like family member 4 | **1,414** | 1,50E-05 | 5,262 | 3,384 |
| ONECUT2 | one cut homeobox 2 | **1,413** | 0,000195 | 1,440 | 1,596 |
| CD83 | CD83 molecule | **1,412** | 0 | 25,036 | 60,083 |
| SORCS2 | sortilin related VPS10 domain containing receptor 2 | **1,411** | 0,000157 | 2,326 | 1,979 |
| ICAM1 | intercellular adhesion molecule 1 | **1,410** | 0 | 4935,984 | 6547,741 |
| UPB1 | beta-ureidopropionase 1 | **1,410** | 0,001251 | 0,997 | 2,043 |
| LYST | lysosomal trafficking regulator | **1,409** | 0 | 26,698 | 40,353 |
| LINC01105 | long intergenic non-protein coding RNA 1105 | **1,409** | 7,00E-06 | 1,606 | 4,980 |
| IL17RD | interleukin 17 receptor D | **1,407** | 0 | 2,271 | 3,959 |
| FHDC1 | FH2 domain containing 1 | **1,407** | 0 | 17,614 | 30,712 |
| LINC01353 | long intergenic non-protein coding RNA 1353 | **1,407** | 0,042802 | 0,277 | 0,511 |
| TNFAIP8 | TNF alpha induced protein 8 | **1,407** | 0 | 103,192 | 175,013 |
| NGFR | nerve growth factor receptor | **1,406** | 0 | 9,139 | 12,515 |
| ANKRD10-IT1 | ANKRD10 intronic transcript 1 | **1,405** | 6,60E-05 | 2,659 | 3,831 |
| TDRD6 | tudor domain containing 6 | **1,405** | 0,023805 | 0,166 | 0,702 |
| LINC01750 | long intergenic non-protein coding RNA 1750 | **1,405** | 0,006237 | 0,554 | 1,788 |
| SOCS7 | suppressor of cytokine signaling 7 | **1,405** | 0,007508 | 1,772 | 1,852 |
| COLEC10 | collectin subfamily member 10 | **1,404** | 0,021806 | 0,443 | 0,575 |
| FAM229A | family with sequence similarity 229 member A | **1,403** | 0,001847 | 0,831 | 1,149 |
| ASCL2 | achaete-scute family bHLH transcription factor 2 | **1,402** | 0 | 2,382 | 2,043 |
| LINC01336 | long intergenic non-protein coding RNA 1336 | **1,401** | 0,028093 | 0,443 | 1,852 |
| LINC00598 | long intergenic non-protein coding RNA 598 | **1,400** | 0,084595 | 0,111 | 0,511 |

| | | | | | |
|---|---|---|---|---|---|
| CD300E | CD300e molecule | **1,399** | 0 | 3,490 | 15,324 |
| FOS | Fos proto-oncogene, AP-1 transcription factor subunit | **1,399** | 0 | 15,398 | 34,926 |
| ACVR1C | activin A receptor type 1C | **1,398** | 0,005309 | 0,665 | 0,958 |
| ITGB4 | integrin subunit beta 4 | **1,398** | 0,00515 | 0,554 | 0,958 |
| FGR | FGR proto-oncogene, Src family tyrosine kinase | **1,398** | 5,00E-06 | 1,108 | 6,321 |
| ZNF540 | zinc finger protein 540 | **1,397** | 3,70E-05 | 1,219 | 2,682 |
| SAMD5 | sterile alpha motif domain containing 5 | **1,397** | 0 | 6,702 | 6,768 |
| GNAL | G protein subunit alpha L | **1,397** | 0 | 3,434 | 4,533 |
| RAB40B | RAB40B, member RAS oncogene family | **1,397** | 0 | 10,192 | 6,960 |
| RBM47 | RNA binding motif protein 47 | **1,394** | 0 | 10,081 | 3,576 |
| IFIT3 | interferon induced protein with tetratricopeptide repeats 3 | **1,394** | 0 | 41,598 | 43,418 |
| SYPL2 | synaptophysin like 2 | **1,394** | 0,060387 | 0,609 | 0,383 |
| SGPP2 | sphingosine-1-phosphate phosphatase 2 | **1,393** | 0 | 51,845 | 78,599 |
| BICDL1 | BICD family like cargo adaptor 1 | **1,392** | 0,022197 | 0,222 | 0,766 |
| EML5 | echinoderm microtubule associated protein like 5 | **1,392** | 0,001731 | 1,274 | 1,469 |
| FAM117A | family with sequence similarity 117 member A | **1,392** | 0 | 9,306 | 12,770 |
| DSC3 | desmocollin 3 | **1,391** | 0 | 14,291 | 5,874 |
| EGR1 | early growth response 1 | **1,390** | 0 | 53,673 | 143,151 |
| EGR3 | early growth response 3 | **1,390** | 0 | 3,323 | 13,600 |
| LGMNP1 | legumain pseudogene 1 | **1,389** | 0,004268 | 1,163 | 1,469 |
| HIVEP1 | human immunodeficiency virus type I enhancer binding protein 1 | **1,389** | 0 | 47,746 | 62,126 |
| LINC01239 | long intergenic non-protein coding RNA 1239 | **1,388** | 3,00E-06 | 1,551 | 1,979 |
| NKAIN1 | sodium/potassium transporting ATPase interacting 1 | **1,387** | 0,103723 | 0,388 | 0,638 |
| TMCC2 | transmembrane and coiled-coil domain family 2 | **1,387** | 0 | 3,490 | 6,193 |
| SYNM | synemin | **1,387** | 0 | 172,208 | 202,149 |
| HEY2 | hes related family bHLH transcription factor with YRPW motif 2 | **1,387** | 0,019039 | 2,049 | 1,915 |
| WSB1 | WD repeat and SOCS box containing 1 | **1,387** | 0 | 70,346 | 101,074 |
| RASGEF1A | RasGEF domain family member 1A | **1,387** | 0,030824 | 0,720 | 0,958 |
| GIPR | gastric inhibitory polypeptide receptor | **1,386** | 0 | 7,865 | 6,768 |
| HIVEP3 | human immunodeficiency virus type I enhancer binding protein 3 | **1,385** | 0 | 5,317 | 8,173 |
| ABCC8 | ATP binding cassette subfamily C member 8 | **1,385** | 0,071105 | 0,443 | 0,638 |
| LHFPL3-AS2 | LHFPL3 antisense RNA 2 | **1,385** | 0,000648 | 2,049 | 1,660 |
| CSPG4 | chondroitin sulfate proteoglycan 4 | **1,384** | 0 | 874,943 | 1309,944 |

| | | | | | |
|---|---|---|---|---|---|
| RASGRF2 | Ras protein specific guanine nucleotide releasing factor 2 | **1,383** | 0,155068 | 0,332 | 0,255 |
| BARD1 | BRCA1 associated RING domain 1 | **1,383** | 4,60E-05 | 5,761 | 4,916 |
| AMN | amnion associated transmembrane protein | **1,382** | 0,093652 | 0,111 | 0,383 |
| RNF150 | ring finger protein 150 | **1,381** | 0,077936 | 0,499 | 0,511 |
| AHNAK2 | AHNAK nucleoprotein 2 | **1,380** | 0 | 138,531 | 187,399 |
| RAD21-AS1 | RAD21 antisense RNA 1 | **1,380** | 0,077597 | 0,554 | 1,149 |
| LINC01558 | long intergenic non-protein coding RNA 1558 | **1,379** | 0,037274 | 0,222 | 1,277 |
| CORO6 | coronin 6 | **1,379** | 0,058006 | 0,332 | 0,192 |
| CAMK4 | calcium/calmodulin dependent protein kinase IV | **1,378** | 0,065236 | 0,388 | 0,575 |
| IVL | involucrin | **1,378** | 0,000238 | 1,108 | 2,426 |
| NRIP2 | nuclear receptor interacting protein 2 | **1,377** | 0,176018 | 0,222 | 1,022 |
| RNF128 | ring finger protein 128, E3 ubiquitin protein ligase | **1,377** | 0,007641 | 2,271 | 0,638 |
| DGKH | diacylglycerol kinase eta | **1,376** | 0 | 18,279 | 27,775 |
| LFNG | LFNG O-fucosylpeptide 3-beta-N-acetylglucosaminyltransferase | **1,375** | 0 | 8,198 | 20,687 |
| DACT3 | dishevelled binding antagonist of beta catenin 3 | **1,375** | 0 | 7,810 | 8,364 |
| HPCAL4 | hippocalcin like 4 | **1,374** | 0,029884 | 0,665 | 0,894 |
| GPER1 | G protein-coupled estrogen receptor 1 | **1,374** | 4,00E-06 | 3,046 | 3,192 |
| RETREG1 | reticulophagy regulator 1 | **1,374** | 0 | 97,874 | 112,503 |
| NDRG1 | N-myc downstream regulated 1 | **1,373** | 0 | 867,798 | 1210,721 |
| TCAF2 | TRPM8 channel associated factor 2 | **1,373** | 0,00156 | 0,942 | 1,979 |
| GATAD2B | GATA zinc finger domain containing 2B | **1,372** | 0,050374 | 0,554 | 1,085 |
| IRX2 | iroquois homeobox 2 | **1,372** | 3,00E-06 | 1,551 | 3,895 |
| IRF5 | interferon regulatory factor 5 | **1,372** | 0,013221 | 0,443 | 0,447 |
| TNFRSF21 | TNF receptor superfamily member 21 | **1,371** | 1,50E-05 | 5,705 | 6,513 |
| SERTAD2 | SERTA domain containing 2 | **1,371** | 0 | 256,623 | 406,660 |
| PMP22 | peripheral myelin protein 22 | **1,371** | 0 | 329,350 | 417,067 |
| HS6ST3 | heparan sulfate 6-O-sulfotransferase 3 | **1,371** | 0,015572 | 0,443 | 1,277 |
| C4orf32 | chromosome 4 open reading frame 32 | **1,371** | 0 | 10,136 | 18,325 |
| NPIPB11 | nuclear pore complex interacting protein family member B11 | **1,371** | 0,033128 | 0,499 | 0,575 |
| PTGES2-AS1 | PTGES2 antisense RNA 1 (head to head) | **1,370** | 0,000493 | 1,440 | 3,512 |
| KIAA1324 | KIAA1324 | **1,370** | 0,001326 | 0,665 | 1,596 |
| CNKSR3 | CNKSR family member 3 | **1,370** | 0 | 16,395 | 16,218 |
| PCLO | piccolo presynaptic cytomatrix protein | **1,370** | 5,00E-06 | 3,711 | 6,960 |

| | | | | | |
|---|---|---|---|---|---|
| FAM46C | family with sequence similarity 46 member C | **1,369** | 0 | 13,127 | 16,090 |
| SNORA33 | small nucleolar RNA, H/ACA box 33 | **1,369** | 0,052221 | 0,388 | 0,575 |
| CXCL14 | C-X-C motif chemokine ligand 14 | **1,369** | 7,00E-06 | 4,154 | 8,620 |
| AQP3 | aquaporin 3 (Gill blood group) | **1,369** | 6,00E-06 | 8,862 | 5,363 |
| ADAP2 | ArfGAP with dual PH domains 2 | **1,368** | 0,046118 | 0,277 | 0,830 |
| TRIM72 | tripartite motif containing 72 | **1,368** | 0,007519 | 0,443 | 0,894 |
| DDIT4L | DNA damage inducible transcript 4 like | **1,367** | 0 | 50,516 | 90,986 |
| FGF22 | fibroblast growth factor 22 | **1,366** | 0,017966 | 0,775 | 2,107 |
| SEMA6A | semaphorin 6A | **1,366** | 0,032892 | 0,942 | 0,447 |
| LPP | LIM domain containing preferred translocation partner in lipoma | **1,365** | 0 | 432,542 | 513,736 |
| ESYT3 | extended synaptotagmin 3 | **1,365** | 1,00E-06 | 3,545 | 5,108 |
| CLSTN2 | calsyntenin 2 | **1,365** | 3,30E-05 | 0,886 | 2,937 |
| SOX8 | SRY-box 8 | **1,363** | 0 | 9,804 | 20,432 |
| PAPPA2 | pappalysin 2 | **1,362** | 0 | 48,079 | 32,627 |
| AC003002.1 | Trafficking protein particle complex subunit 2B | **1,360** | 4,00E-05 | 1,883 | 3,256 |
| KDSR | 3-ketodihydrosphingosine reductase | **1,360** | 0 | 118,535 | 161,796 |
| OXTR | oxytocin receptor | **1,360** | 5,00E-06 | 1,606 | 4,853 |
| CCDC153 | coiled-coil domain containing 153 | **1,360** | 0,091497 | 0,332 | 0,702 |
| RNF175 | ring finger protein 175 | **1,359** | 0,071062 | 0,609 | 0,702 |
| RAP1GAP | RAP1 GTPase activating protein | **1,359** | 1,90E-05 | 2,437 | 3,129 |
| MSC-AS1 | MSC antisense RNA 1 | **1,358** | 0 | 12,020 | 12,962 |
| BAIAP3 | BAI1 associated protein 3 | **1,358** | 1,30E-05 | 4,542 | 7,981 |
| VGLL2 | vestigial like family member 2 | **1,358** | 0,139472 | 0,166 | 0,255 |
| RCAN2 | regulator of calcineurin 2 | **1,358** | 0,009325 | 1,219 | 1,149 |
| GABRQ | gamma-aminobutyric acid type A receptor theta subunit | **1,358** | 0,022504 | 0,332 | 0,894 |
| WNT9A | Wnt family member 9A | **1,357** | 1,70E-05 | 6,868 | 14,239 |
| EFNB2 | ephrin B2 | **1,357** | 0 | 22,655 | 27,264 |
| RPL34P18 | ribosomal protein L34 pseudogene 18 | **1,357** | 0,137188 | 0,055 | 0,511 |
| PPP1R3E | protein phosphatase 1 regulatory subunit 3E | **1,356** | 0 | 3,767 | 7,981 |
| KCNMB4 | potassium calcium-activated channel subfamily M regulatory beta subunit 4 | **1,356** | 0,098562 | 1,108 | 0,511 |
| RAPGEF4 | Rap guanine nucleotide exchange factor 4 | **1,356** | 0,044474 | 0,775 | 0,958 |
| DOCK10 | dedicator of cytokinesis 10 | **1,356** | 0,000112 | 1,052 | 3,065 |
| YPEL1 | yippee like 1 | **1,355** | 0 | 4,265 | 6,385 |

| | | | | | |
|---|---|---|---|---|---|
| CDH2 | cadherin 2 | **1,355** | 0,00033 | 1,440 | 1,213 |
| N4BP2L1 | NEDD4 binding protein 2 like 1 | **1,355** | 0 | 6,647 | 9,577 |
| INHBB | inhibin beta B subunit | **1,355** | 0,115448 | 0,277 | 0,575 |
| MICALL2 | MICAL like 2 | **1,355** | 0 | 15,454 | 31,733 |
| HEY1 | hes related family bHLH transcription factor with YRPW motif 1 | **1,354** | 0,09172 | 0,554 | 0,383 |
| RBM43 | RNA binding motif protein 43 | **1,352** | 0 | 41,875 | 61,679 |
| RTN4R | reticulon 4 receptor | **1,352** | 0,001345 | 1,772 | 1,660 |
| ZCCHC2 | zinc finger CCHC-type containing 2 | **1,352** | 0 | 12,574 | 19,155 |
| HBA2 | hemoglobin subunit alpha 2 | **1,352** | 0,07429 | 0,997 | 0,511 |
| L1CAM | L1 cell adhesion molecule | **1,352** | 0,046953 | 0,499 | 0,192 |
| CEMIP | cell migration inducing hyaluronan binding protein | **1,352** | 0 | 29,080 | 62,956 |
| LRRC37A3 | leucine rich repeat containing 37 member A3 | **1,351** | 0 | 3,711 | 6,193 |
| CELSR1 | cadherin EGF LAG seven-pass G-type receptor 1 | **1,351** | 0 | 46,362 | 77,961 |
| AZIN2 | antizyme inhibitor 2 | **1,351** | 0 | 8,364 | 11,685 |
| SCUBE3 | signal peptide, CUB domain and EGF like domain containing 3 | **1,350** | 0 | 38,108 | 70,363 |
| ACTA2 | actin, alpha 2, smooth muscle, aorta | **1,349** | 5,10E-05 | 0,886 | 3,576 |
| RGS16 | regulator of G protein signaling 16 | **1,349** | 0 | 55,002 | 69,533 |
| GPRC5B | G protein-coupled receptor class C group 5 member B | **1,349** | 0 | 13,404 | 6,832 |
| CLTCL1 | clathrin heavy chain like 1 | **1,348** | 4,00E-06 | 6,979 | 4,533 |
| NEAT1 | nuclear paraspeckle assembly transcript 1 (non-protein coding) | **1,348** | 0 | 455,418 | 984,310 |
| DIO2 | iodothyronine deiodinase 2 | **1,348** | 0 | 35,173 | 45,333 |
| EFR3B | EFR3 homolog B | **1,348** | 0 | 9,306 | 16,984 |
| TLR2 | toll like receptor 2 | **1,347** | 0 | 96,767 | 92,072 |
| C11orf96 | chromosome 11 open reading frame 96 | **1,347** | 0 | 125,126 | 299,584 |
| MTMR10 | myotubularin related protein 10 | **1,345** | 0,039691 | 0,388 | 1,213 |
| GRM2 | glutamate metabotropic receptor 2 | **1,345** | 0,037526 | 0,499 | 1,022 |
| RRAGD | Ras related GTP binding D | **1,345** | 0 | 21,214 | 21,262 |
| CCNE1 | cyclin E1 | **1,344** | 0,006598 | 2,271 | 1,788 |
| MTMR7 | myotubularin related protein 7 | **1,344** | 0 | 17,448 | 23,816 |
| ZNF658B | zinc finger protein 658B (pseudogene) | **1,344** | 0,103072 | 0,388 | 0,383 |
| LINC00324 | long intergenic non-protein coding RNA 324 | **1,344** | 0,030242 | 0,609 | 1,277 |
| FSD1L | fibronectin type III and SPRY domain containing 1 like | **1,343** | 3,00E-06 | 4,764 | 6,257 |
| NAP1L4P1 | nucleosome assembly protein 1 like 4 pseudogene 1 | **1,343** | 0,168842 | 0,111 | 0,766 |

| | | | | | |
|---|---|---|---|---|---|
| MTTP | microsomal triglyceride transfer protein | **1,343** | 0,014365 | 0,443 | 0,766 |
| BTBD11 | BTB domain containing 11 | **1,342** | 0 | 5,650 | 11,365 |
| RASL11B | RAS like family 11 member B | **1,342** | 0,007581 | 0,720 | 1,085 |
| JAG2 | jagged 2 | **1,341** | 0,00131 | 1,994 | 2,746 |
| AP3B2 | adaptor related protein complex 3 beta 2 subunit | **1,340** | 6,60E-05 | 2,326 | 3,767 |
| GPRIN1 | G protein regulated inducer of neurite outgrowth 1 | **1,340** | 0,000176 | 4,320 | 4,150 |
| C17orf113 | chromosome 17 open reading frame 113 | **1,340** | 0,00183 | 1,108 | 3,129 |
| UAP1L1 | UDP-N-acetylglucosamine pyrophosphorylase 1 like 1 | **1,339** | 0 | 5,927 | 4,980 |
| TMEM200C | transmembrane protein 200C | **1,339** | 0,023543 | 2,659 | 1,085 |
| TNP1 | transition protein 1 | **1,339** | 0,084719 | 0,997 | 1,277 |
| GAREM1 | GRB2 associated regulator of MAPK1 subtype 1 | **1,338** | 0 | 25,479 | 32,691 |
| 5.syys | septin 5 | **1,337** | 0,044235 | 0,554 | 0,511 |
| PARD3B | par-3 family cell polarity regulator beta | **1,337** | 5,10E-05 | 2,603 | 3,320 |
| ALCAM | activated leukocyte cell adhesion molecule | **1,336** | 0 | 43,426 | 45,270 |
| CREBRF | CREB3 regulatory factor | **1,336** | 0 | 72,063 | 91,497 |
| SMPD3 | sphingomyelin phosphodiesterase 3 | **1,335** | 0,000602 | 2,770 | 3,384 |
| FTH1P16 | ferritin heavy chain 1 pseudogene 16 | **1,335** | 0,018792 | 0,665 | 0,830 |
| PKMP3 | pyruvate kinase, muscle pseudogene 3 | **1,335** | 0,003787 | 0,720 | 1,788 |
| ESPN | espin | **1,335** | 0,003506 | 2,714 | 3,384 |
| ARHGAP30 | Rho GTPase activating protein 30 | **1,335** | 0,202718 | 0,222 | 0,894 |
| PLB1 | phospholipase B1 | **1,335** | 0,081492 | 0,277 | 0,766 |
| HOXD11 | homeobox D11 | **1,335** | 0,019414 | 0,665 | 0,702 |
| OSR2 | odd-skipped related transciption factor 2 | **1,335** | 1,00E-06 | 4,099 | 5,746 |
| GRAMD2 | GRAM domain containing 2 | **1,335** | 0,060555 | 0,499 | 0,638 |
| B3GNT5 | UDP-GlcNAc:betaGal beta-1,3-N-acetylglucosaminyltransferase 5 | **1,335** | 0 | 24,981 | 32,500 |
| ROR2 | receptor tyrosine kinase like orphan receptor 2 | **1,335** | 0 | 47,359 | 42,460 |
| CTSV | cathepsin V | **1,334** | 7,40E-05 | 1,828 | 2,362 |
| FBXO32 | F-box protein 32 | **1,333** | 0 | 158,028 | 260,635 |
| MMP15 | matrix metallopeptidase 15 | **1,333** | 2,00E-06 | 3,877 | 4,980 |
| ASPRV1 | aspartic peptidase, retroviral-like 1 | **1,333** | 0 | 3,157 | 4,916 |
| AMDHD2 | amidohydrolase domain containing 2 | **1,332** | 0 | 9,859 | 12,068 |
| PSD2 | pleckstrin and Sec7 domain containing 2 | **1,331** | 0,152101 | 0,166 | 0,830 |
| CCDC148 | coiled-coil domain containing 148 | **1,331** | 0,112349 | 0,222 | 0,192 |

| | | | | | |
|---|---|---|---|---|---|
| NFIL3 | nuclear factor, interleukin 3 regulated | **1,331** | 0 | 52,510 | 76,301 |
| FKBP5 | FK506 binding protein 5 | **1,329** | 0 | 12,241 | 16,473 |
| KLHL24 | kelch like family member 24 | **1,329** | 0 | 40,767 | 44,184 |
| RHEBL1 | Ras homolog enriched in brain like 1 | **1,329** | 0,002409 | 1,551 | 1,724 |
| COLQ | collagen like tail subunit of asymmetric acetylcholinesterase | **1,329** | 0,01419 | 0,388 | 1,405 |
| SEMA5A | semaphorin 5A | **1,329** | 0,000665 | 1,385 | 3,384 |
| CUZD1 | CUB and zona pellucida like domains 1 | **1,329** | 0,13278 | 0,222 | 0,255 |
| LDB3 | LIM domain binding 3 | **1,328** | 0,049274 | 0,554 | 1,149 |
| PAMR1 | peptidase domain containing associated with muscle regeneration 1 | **1,328** | 0,002511 | 5,373 | 2,235 |
| RBPJ | recombination signal binding protein for immunoglobulin kappa J region | **1,328** | 0 | 116,652 | 202,340 |
| BACH2 | BTB domain and CNC homolog 2 | **1,327** | 0,001486 | 1,717 | 2,682 |
| FAM43A | family with sequence similarity 43 member A | **1,327** | 0 | 22,156 | 55,805 |
| ARHGAP24 | Rho GTPase activating protein 24 | **1,327** | 0 | 28,304 | 28,477 |
| GPR63 | G protein-coupled receptor 63 | **1,327** | 0,135389 | 0,166 | 0,575 |
| CISH | cytokine inducible SH2 containing protein | **1,327** | 0,058334 | 0,554 | 0,638 |
| WTAPP1 | Wilms tumor 1 associated protein pseudogene 1 | **1,326** | 0,001353 | 42,429 | 78,408 |
| PLCD1 | phospholipase C delta 1 | **1,325** | 0 | 48,688 | 50,122 |
| CAPN5 | calpain 5 | **1,324** | 0 | 9,859 | 13,792 |
| CNR1 | cannabinoid receptor 1 | **1,324** | 0,003594 | 0,831 | 0,766 |
| ACSS1 | acyl-CoA synthetase short-chain family member 1 | **1,324** | 0,005233 | 0,388 | 1,915 |
| LRRC2 | leucine rich repeat containing 2 | **1,324** | 0,003098 | 1,219 | 7,087 |
| FGF9 | fibroblast growth factor 9 | **1,323** | 0,150269 | 0,277 | 0,319 |
| TRAF1 | TNF receptor associated factor 1 | **1,322** | 0 | 250,640 | 405,447 |
| QRICH2 | glutamine rich 2 | **1,322** | 0 | 4,597 | 6,960 |
| HPSE | heparanase | **1,322** | 0,08747 | 0,443 | 0,894 |
| GRB7 | growth factor receptor bound protein 7 | **1,321** | 0,000163 | 1,108 | 2,937 |
| KCNK12 | potassium two pore domain channel subfamily K member 12 | **1,321** | 0,068509 | 0,499 | 1,085 |
| MYLK | myosin light chain kinase | **1,321** | 0,003555 | 1,994 | 3,831 |
| IFIH1 | interferon induced with helicase C domain 1 | **1,320** | 0 | 13,626 | 20,368 |
| HBEGF | heparin binding EGF like growth factor | **1,320** | 0 | 51,236 | 93,795 |
| CAPN10-AS1 | CAPN10 antisense RNA 1 (head to head) | **1,320** | 0,000187 | 2,105 | 4,916 |
| PPARGC1A | PPARG coactivator 1 alpha | **1,320** | 0,00401 | 0,997 | 1,724 |
| KCNK5 | potassium two pore domain channel subfamily K member 5 | **1,319** | 0 | 48,522 | 119,208 |

| | | | | | |
|---|---|---|---|---|---|
| WNT11 | Wnt family member 11 | **1,319** | 0,016716 | 0,942 | 1,724 |
| PLCG2 | phospholipase C gamma 2 | **1,319** | 0 | 25,479 | 39,395 |
| HEXA-AS1 | HEXA antisense RNA 1 | **1,319** | 0,168631 | 0,277 | 0,319 |
| PRKG1 | protein kinase, cGMP-dependent, type I | **1,319** | 3,00E-05 | 4,708 | 7,151 |
| PRDM16 | PR/SET domain 16 | **1,318** | 0,007871 | 2,216 | 2,426 |
| TPPP | tubulin polymerization promoting protein | **1,317** | 0,000776 | 3,822 | 4,789 |
| GPR153 | G protein-coupled receptor 153 | **1,317** | 2,90E-05 | 10,192 | 5,874 |
| IRF8 | interferon regulatory factor 8 | **1,317** | 0,002387 | 0,886 | 2,426 |
| KLHL23 | kelch like family member 23 | **1,317** | 0,000265 | 2,991 | 4,725 |
| KANK1 | KN motif and ankyrin repeat domains 1 | **1,316** | 0 | 148,003 | 180,504 |
| LONRF2 | LON peptidase N-terminal domain and ring finger 2 | **1,316** | 0 | 8,862 | 20,815 |
| ANKRD6 | ankyrin repeat domain 6 | **1,315** | 0 | 24,206 | 28,669 |
| ST6GALNAC2 | ST6 N-acetylgalactosaminide alpha-2,6-sialyltransferase 2 | **1,315** | 0,000273 | 3,767 | 1,979 |
| USP32P3 | ubiquitin specific peptidase 32 pseudogene 3 | **1,314** | 0,059777 | 0,720 | 0,383 |
| TMEM91 | transmembrane protein 91 | **1,314** | 0,000113 | 1,496 | 2,554 |
| SORBS1 | sorbin and SH3 domain containing 1 | **1,314** | 0 | 15,842 | 20,432 |
| METTL7A | methyltransferase like 7A | **1,314** | 0 | 53,618 | 115,057 |
| TOB1-AS1 | TOB1 antisense RNA 1 | **1,313** | 0,061273 | 0,665 | 0,702 |
| ITGB7 | integrin subunit beta 7 | **1,313** | 5,00E-06 | 2,326 | 4,214 |
| KLHL21 | kelch like family member 21 | **1,312** | 0 | 501,281 | 594,570 |
| KCNQ1OT1 | KCNQ1 opposite strand/antisense transcript 1 (non-protein coding) | **1,311** | 0,001746 | 11,577 | 22,028 |
| CEP295NL | CEP295 N-terminal like | **1,311** | 0,080572 | 0,332 | 0,575 |
| MT-TQ | mitochondrially encoded tRNA glutamine | **1,311** | 0,116637 | 0,554 | 0,511 |
| BSN | bassoon presynaptic cytomatrix protein | **1,311** | 7,80E-05 | 4,043 | 9,131 |
| RAB11FIP4 | RAB11 family interacting protein 4 | **1,311** | 0 | 33,123 | 49,484 |
| SLC39A10 | solute carrier family 39 member 10 | **1,311** | 0 | 10,413 | 12,195 |
| LRRC66 | leucine rich repeat containing 66 | **1,311** | 0,118138 | 0,222 | 0,383 |
| IL1A | interleukin 1 alpha | **1,310** | 4,20E-05 | 3,323 | 3,256 |
| 1.maalis | mitochondrial amidoxime reducing component 1 | **1,310** | 0,05982 | 0,388 | 0,894 |
| PWAR5 | Prader Willi/Angelman region RNA 5 | **1,309** | 0,146092 | 0,720 | 1,532 |
| TNFSF18 | TNF superfamily member 18 | **1,309** | 2,00E-06 | 14,623 | 20,560 |
| CLDN12 | claudin 12 | **1,308** | 0 | 89,067 | 102,224 |
| CBARP | CACN beta subunit associated regulatory protein | **1,308** | 3,30E-05 | 4,708 | 14,685 |

| | | | | | |
|---|---|---|---|---|---|
| TNFAIP2 | TNF alpha induced protein 2 | **1,308** | 0 | 821,768 | 1326,162 |
| PTX3 | pentraxin 3 | **1,308** | 0 | 670,775 | 495,922 |
| VCAM1 | vascular cell adhesion molecule 1 | **1,308** | 0 | 1797,078 | 2925,346 |
| C10orf105 | chromosome 10 open reading frame 105 | **1,307** | 1,00E-05 | 1,274 | 4,278 |
| ALAS1 | 5'-aminolevulinate synthase 1 | **1,307** | 0 | 34,896 | 35,373 |
| CD24 | CD24 molecule | **1,307** | 0 | 5,262 | 7,598 |
| IRF4 | interferon regulatory factor 4 | **1,307** | 0 | 9,416 | 24,135 |
| TP53INP1 | tumor protein p53 inducible nuclear protein 1 | **1,306** | 0 | 13,072 | 16,218 |
| ATP9A | ATPase phospholipid transporting 9A (putative) | **1,306** | 0 | 26,255 | 27,647 |
| VAT1L | vesicle amine transport 1 like | **1,306** | 0,136233 | 0,388 | 0,638 |
| HNRNPA1P65 | heterogeneous nuclear ribonucleoprotein A1 pseudogene 65 | **1,306** | 0,079034 | 0,222 | 0,638 |
| LINC00571 | long intergenic non-protein coding RNA 571 | **1,306** | 0,083074 | 0,277 | 0,319 |
| NEMP1 | nuclear envelope integral membrane protein 1 | **1,305** | 0 | 24,759 | 29,946 |
| ALDH1A2 | aldehyde dehydrogenase 1 family member A2 | **1,305** | 0 | 30,852 | 60,274 |
| KLF5 | Kruppel like factor 5 | **1,305** | 0 | 84,304 | 122,400 |
| SPRR2A | small proline rich protein 2A | **1,305** | 0,002441 | 0,499 | 2,043 |
| APLP1 | amyloid beta precursor like protein 1 | **1,304** | 0,166436 | 0,277 | 0,447 |
| BANK1 | B-cell scaffold protein with ankyrin repeats 1 | **1,304** | 0 | 6,702 | 7,790 |
| KLHL28 | kelch like family member 28 | **1,304** | 0 | 21,824 | 31,286 |
| MFSD2B | major facilitator superfamily domain containing 2B | **1,304** | 0,103122 | 0,443 | 0,766 |
| STBD1 | starch binding domain 1 | **1,303** | 0,002151 | 1,551 | 2,362 |
| UGT8 | UDP glycosyltransferase 8 | **1,303** | 0,015068 | 1,662 | 1,788 |
| SPP1 | secreted phosphoprotein 1 | **1,303** | 0 | 2150,246 | 808,850 |
| TENM3 | teneurin transmembrane protein 3 | **1,303** | 0 | 16,839 | 15,835 |
| MEIG1 | meiosis/spermiogenesis associated 1 | **1,303** | 0,057216 | 0,554 | 0,575 |
| UBE2Q2P1 | ubiquitin conjugating enzyme E2 Q2 pseudogene 1 | **1,302** | 0,099478 | 0,886 | 1,149 |
| YJEFN3 | YjeF N-terminal domain containing 3 | **1,302** | 0,001672 | 1,385 | 2,490 |
| FAM149A | family with sequence similarity 149 member A | **1,302** | 0,129291 | 0,332 | 0,830 |
| HOXA-AS2 | HOXA cluster antisense RNA 2 | **1,301** | 0,190141 | 0,277 | 0,447 |
| PPP1R14C | protein phosphatase 1 regulatory inhibitor subunit 14C | **1,301** | 0 | 60,541 | 63,211 |
| CYP2U1 | cytochrome P450 family 2 subfamily U member 1 | **1,301** | 0 | 18,777 | 19,155 |
| CYP7A1 | cytochrome P450 family 7 subfamily A member 1 | **1,301** | 0,022702 | 1,108 | 2,299 |
| RASGEF1B | RasGEF domain family member 1B | **1,301** | 0 | 13,404 | 18,325 |

| | | | | | |
|---|---|---|---|---|---|
| IL10RB | interleukin 10 receptor subunit beta | **1,300** | 0,005563 | 1,772 | 1,852 |
| ANK2 | ankyrin 2 | **1,300** | 0 | 4,985 | 6,193 |
| PTCH2 | patched 2 | **1,300** | 0,039818 | 0,720 | 1,213 |
| CACNA2D2 | calcium voltage-gated channel auxiliary subunit alpha2delta 2 | **1,300** | 0,032153 | 0,554 | 1,660 |
| IL20 | interleukin 20 | **1,300** | 0,014264 | 1,662 | 2,426 |
| HOMER1 | homer scaffolding protein 1 | **1,300** | 0 | 6,979 | 11,110 |
| YY2 | YY2 transcription factor | **1,300** | 0,042197 | 0,499 | 1,022 |
| SERPINB7 | serpin family B member 7 | **1,299** | 0 | 160,355 | 231,775 |
| WBP1LP2 | WW domain binding protein 1-like pseudogene 2 | **1,299** | 0,148813 | 0,443 | 0,255 |
| PLCL1 | phospholipase C like 1 | **1,299** | 0,187925 | 0,720 | 0,575 |
| AMH | anti-Mullerian hormone | **1,299** | 0,172678 | 0,499 | 0,511 |
| VGF | VGF nerve growth factor inducible | **1,299** | 2,00E-05 | 10,635 | 10,535 |
| C9orf66 | chromosome 9 open reading frame 66 | **1,299** | 0,11985 | 0,277 | 0,447 |
| RAB3A | RAB3A, member RAS oncogene family | **1,299** | 0,002728 | 1,772 | 4,406 |
| FAM53C | family with sequence similarity 53 member C | **1,298** | 0 | 39,826 | 55,486 |
| TEX22 | testis expressed 22 | **1,298** | 0,033931 | 1,052 | 1,660 |
| KLF6 | Kruppel like factor 6 | **1,297** | 0 | 248,646 | 278,385 |
| ADGRF3 | adhesion G protein-coupled receptor F3 | **1,297** | 0,161744 | 0,055 | 0,511 |
| TTC9 | tetratricopeptide repeat domain 9 | **1,297** | 3,00E-06 | 6,370 | 6,002 |
| ZNF483 | zinc finger protein 483 | **1,296** | 0,035016 | 0,720 | 1,277 |
| PKIA | cAMP-dependent protein kinase inhibitor alpha | **1,296** | 0,211492 | 0,665 | 0,255 |
| IL7 | interleukin 7 | **1,296** | 0,075263 | 0,388 | 0,319 |
| EPHA3 | EPH receptor A3 | **1,296** | 0,085276 | 0,665 | 0,766 |
| GPR146 | G protein-coupled receptor 146 | **1,296** | 0 | 4,930 | 5,555 |
| DNAJC6 | DnaJ heat shock protein family (Hsp40) member C6 | **1,295** | 0 | 12,130 | 15,516 |
| SUZ12P1 | SUZ12 polycomb repressive complex 2 subunit pseudogene 1 | **1,295** | 0,015786 | 2,714 | 3,129 |
| MED14OS | MED14 opposite strand | **1,295** | 0,189433 | 0,499 | 0,255 |
| ATRNL1 | attractin like 1 | **1,295** | 0,001681 | 2,049 | 1,660 |
| ELF3 | E74 like ETS transcription factor 3 | **1,295** | 0 | 81,257 | 118,952 |
| RPL4P6 | ribosomal protein L4 pseudogene 6 | **1,295** | 0,047131 | 0,997 | 1,341 |
| CEP192 | centrosomal protein 192 | **1,294** | 2,00E-06 | 21,658 | 29,052 |
| GXYLT2 | glucoside xylosyltransferase 2 | **1,294** | 0,054911 | 1,329 | 1,660 |
| ZNF382 | zinc finger protein 382 | **1,294** | 0,160691 | 0,277 | 0,447 |

| | | | | | |
|---|---|---|---|---|---|
| PLEKHA8P1 | pleckstrin homology domain containing A8 pseudogene 1 | **1,294** | 0,014429 | 0,886 | 1,213 |
| CNNM4 | cyclin and CBS domain divalent metal cation transport mediator 4 | **1,294** | 0 | 40,823 | 68,192 |
| AIF1L | allograft inflammatory factor 1 like | **1,294** | 0,138488 | 0,554 | 0,638 |
| TSTD1 | thiosulfate sulfurtransferase like domain containing 1 | **1,293** | 0,247796 | 0,609 | 0,447 |
| NR1D1 | nuclear receptor subfamily 1 group D member 1 | **1,293** | 0 | 117,649 | 206,618 |
| EFNA3 | ephrin A3 | **1,293** | 0,010872 | 1,108 | 0,447 |
| PHOSPHO2 | phosphatase, orphan 2 | **1,293** | 0,028209 | 1,662 | 1,915 |
| PPM1L | protein phosphatase, Mg2+/Mn2+ dependent 1L | **1,292** | 1,00E-05 | 8,862 | 17,686 |
| DDN | dendrin | **1,292** | 0,076789 | 0,332 | 0,575 |
| MAP1LC3B2 | microtubule associated protein 1 light chain 3 beta 2 | **1,292** | 0,131116 | 0,166 | 0,383 |
| ZMIZ1-AS1 | ZMIZ1 antisense RNA 1 | **1,292** | 0 | 13,515 | 28,860 |
| LEPR | leptin receptor | **1,292** | 1,00E-06 | 5,262 | 5,172 |
| ASF1B | anti-silencing function 1B histone chaperone | **1,292** | 7,00E-05 | 3,490 | 3,831 |
| MLLT11 | myeloid/lymphoid or mixed-lineage leukemia; translocated to, 11 | **1,292** | 0 | 37,610 | 51,782 |
| TMEM38B | transmembrane protein 38B | **1,291** | 0 | 31,683 | 42,907 |
| MXD1 | MAX dimerization protein 1 | **1,290** | 0 | 181,846 | 238,607 |
| SH3BGRL2 | SH3 domain binding glutamate rich protein like 2 | **1,290** | 0,00042 | 2,548 | 3,767 |
| LINC01176 | long intergenic non-protein coding RNA 1176 | **1,290** | 0,040154 | 0,388 | 0,830 |
| RIPOR2 | RHO family interacting cell polarization regulator 2 | **1,290** | 0,11555 | 0,831 | 0,255 |
| KAT2B | lysine acetyltransferase 2B | **1,290** | 3,00E-06 | 10,303 | 9,833 |
| PSMD6-AS2 | PSMD6 antisense RNA 2 | **1,289** | 0,090676 | 0,388 | 1,405 |
| SAPCD2 | suppressor APC domain containing 2 | **1,289** | 0,002493 | 3,711 | 4,853 |
| PRKN | parkin RBR E3 ubiquitin protein ligase | **1,289** | 0,100803 | 0,332 | 0,638 |
| BCL6 | B-cell CLL/lymphoma 6 | **1,288** | 0 | 260,611 | 314,205 |
| PRSS35 | protease, serine 35 | **1,288** | 0,152928 | 0,554 | 1,277 |
| S100A14 | S100 calcium binding protein A14 | **1,287** | 0,141403 | 0,222 | 0,383 |
| CRISPLD2 | cysteine rich secretory protein LCCL domain containing 2 | **1,287** | 0 | 17,891 | 26,562 |
| LGALS3BP | galectin 3 binding protein | **1,287** | 0,100551 | 0,443 | 1,213 |
| KRT8P33 | keratin 8 pseudogene 33 | **1,287** | 0,009462 | 1,219 | 2,107 |
| MEGF9 | multiple EGF like domains 9 | **1,287** | 0 | 16,395 | 18,453 |
| RALGPS1 | Ral GEF with PH domain and SH3 binding motif 1 | **1,286** | 4,10E-05 | 2,770 | 4,853 |
| KIF1A | kinesin family member 1A | **1,286** | 4,20E-05 | 11,687 | 18,453 |
| KLF8 | Kruppel like factor 8 | **1,286** | 0,000715 | 2,603 | 2,937 |

| | | | | | |
|---|---|---|---|---|---|
| NEURL3 | neuralized E3 ubiquitin protein ligase 3 | **1,285** | 0,280202 | 0,443 | 0,958 |
| COL4A3BP | collagen type IV alpha 3 binding protein | **1,285** | 0 | 43,980 | 52,676 |
| PPFIA3 | PTPRF interacting protein alpha 3 | **1,285** | 0,031959 | 0,443 | 0,894 |
| SLC6A12 | solute carrier family 6 member 12 | **1,285** | 0,001656 | 1,662 | 3,959 |
| ZNF718 | zinc finger protein 718 | **1,285** | 6,00E-06 | 6,093 | 5,683 |
| PKD2 | polycystin 2, transient receptor potential cation channel | **1,284** | 0 | 116,098 | 120,868 |
| DLEC1 | deleted in lung and esophageal cancer 1 | **1,284** | 0,090702 | 0,443 | 0,830 |
| ELOVL3 | ELOVL fatty acid elongase 3 | **1,284** | 0,010161 | 1,219 | 1,915 |
| KIAA0319 | KIAA0319 | **1,284** | 0,126972 | 0,443 | 0,511 |
| IGFBPL1 | insulin like growth factor binding protein like 1 | **1,284** | 0,178809 | 0,332 | 0,447 |
| FMNL1 | formin like 1 | **1,284** | 0,009211 | 2,493 | 2,618 |
| BMP8B | bone morphogenetic protein 8b | **1,284** | 0,031472 | 0,665 | 0,894 |
| ADCY10P1 | adenylate cyclase 10, soluble pseudogene 1 | **1,283** | 2,50E-05 | 3,490 | 4,533 |
| SYNE2 | spectrin repeat containing nuclear envelope protein 2 | **1,283** | 0,001463 | 9,306 | 10,663 |
| NPTX2 | neuronal pentraxin 2 | **1,283** | 8,00E-06 | 11,023 | 26,753 |
| FTH1P7 | ferritin heavy chain 1 pseudogene 7 | **1,283** | 0,182865 | 0,388 | 0,383 |
| SNN | stannin | **1,282** | 0 | 16,562 | 22,922 |
| FAM84A | family with sequence similarity 84 member A | **1,282** | 0,001164 | 2,382 | 1,341 |
| KCNN4 | potassium calcium-activated channel subfamily N member 4 | **1,282** | 1,00E-06 | 6,148 | 5,427 |
| B4GALT5 | beta-1,4-galactosyltransferase 5 | **1,281** | 0 | 32,902 | 33,202 |
| NPIPA1 | nuclear pore complex interacting protein family member A1 | **1,281** | 0,063535 | 0,665 | 0,511 |
| CCSAP | centriole, cilia and spindle associated protein | **1,280** | 0 | 6,536 | 11,748 |
| DEPTOR | DEP domain containing MTOR interacting protein | **1,280** | 0,012355 | 1,385 | 2,426 |
| ZNF844 | zinc finger protein 844 | **1,280** | 0 | 20,716 | 24,199 |
| NCK1 | NCK adaptor protein 1 | **1,280** | 0 | 71,287 | 106,119 |
| ADTRP | androgen dependent TFPI regulating protein | **1,280** | 0 | 2,659 | 48,526 |
| C1orf54 | chromosome 1 open reading frame 54 | **1,280** | 0,19478 | 0,166 | 0,447 |
| SERPINE1 | serpin family E member 1 | **1,279** | 0 | 475,192 | 467,892 |
| MEIS3 | Meis homeobox 3 | **1,279** | 0,094751 | 0,942 | 0,511 |
| IL20RB | interleukin 20 receptor subunit beta | **1,279** | 0,018011 | 0,720 | 1,596 |
| HPX | hemopexin | **1,279** | 0,20067 | 0,388 | 0,383 |
| FZD7 | frizzled class receptor 7 | **1,279** | 0 | 45,918 | 44,248 |
| CDKL3 | cyclin dependent kinase like 3 | **1,279** | 0,038283 | 0,997 | 1,596 |

| | | | | | |
|---|---|---|---|---|---|
| CADM4 | cell adhesion molecule 4 | **1,279** | 0,179763 | 0,443 | 1,022 |
| STX3 | syntaxin 3 | **1,279** | 3,00E-06 | 8,530 | 6,832 |
| SLC9B1 | solute carrier family 9 member B1 | **1,278** | 0,242763 | 0,277 | 0,511 |
| FKBP4 | FK506 binding protein 4 | **1,278** | 0 | 77,048 | 85,750 |
| RGS7 | regulator of G protein signaling 7 | **1,278** | 0,144241 | 0,277 | 0,447 |
| B3GALT1 | beta-1,3-galactosyltransferase 1 | **1,276** | 0,000321 | 3,490 | 4,533 |
| MGEA5 | meningioma expressed antigen 5 (hyaluronidase) | **1,276** | 0 | 120,917 | 150,047 |
| BARX2 | BARX homeobox 2 | **1,275** | 0 | 28,415 | 58,678 |
| ERV3-1 | endogenous retrovirus group 3 member 1, envelope | **1,275** | 0,000508 | 4,764 | 5,363 |
| SOCS2-AS1 | SOCS2 antisense RNA 1 | **1,275** | 0,000397 | 3,323 | 2,873 |
| ARRDC4 | arrestin domain containing 4 | **1,275** | 0 | 34,342 | 57,720 |
| CCDC121 | coiled-coil domain containing 121 | **1,275** | 0,018526 | 2,216 | 2,873 |
| CCDC68 | coiled-coil domain containing 68 | **1,275** | 0,194154 | 0,388 | 0,255 |
| PGBD5 | piggyBac transposable element derived 5 | **1,274** | 0,000122 | 4,043 | 4,853 |
| ARHGEF37 | Rho guanine nucleotide exchange factor 37 | **1,274** | 0,01692 | 1,108 | 2,618 |
| CYP46A1 | cytochrome P450 family 46 subfamily A member 1 | **1,274** | 0,008195 | 1,939 | 3,576 |
| RAC2 | ras-related C3 botulinum toxin substrate 2 (rho family, small GTP binding protein Rac2) | **1,274** | 2,70E-05 | 4,708 | 7,917 |
| FMNL3 | formin like 3 | **1,274** | 0 | 50,239 | 56,316 |
| SENCR | smooth muscle and endothelial cell enriched migration/differentiation-associated long no | **1,274** | 0,040433 | 0,831 | 1,341 |
| REL | REL proto-oncogene, NF-kB subunit | **1,273** | 0,021746 | 6,259 | 11,238 |
| GBP1P1 | guanylate binding protein 1 pseudogene 1 | **1,273** | 0,022225 | 1,551 | 2,426 |
| CNTRL | centriolin | **1,273** | 1,00E-06 | 15,731 | 21,070 |
| LEF1 | lymphoid enhancer binding factor 1 | **1,273** | 0,091529 | 0,775 | 0,447 |
| MT-ND5 | mitochondrially encoded NADH:ubiquinone oxidoreductase core subunit 5 | **1,273** | 0,000168 | 774,853 | 1126,887 |
| PROX1 | prospero homeobox 1 | **1,272** | 0,078911 | 1,052 | 0,702 |
| C3orf58 | chromosome 3 open reading frame 58 | **1,272** | 0 | 33,566 | 42,141 |
| RAPH1 | Ras association (RalGDS/AF-6) and pleckstrin homology domains 1 | **1,272** | 1,00E-06 | 133,490 | 181,142 |
| TMEM2 | transmembrane protein 2 | **1,272** | 0 | 59,711 | 97,818 |
| ZNF547 | zinc finger protein 547 | **1,271** | 0,045879 | 0,720 | 1,085 |
| TMEM140 | transmembrane protein 140 | **1,271** | 1,00E-06 | 7,588 | 9,386 |
| CDK5R1 | cyclin dependent kinase 5 regulatory subunit 1 | **1,271** | 0,000114 | 2,936 | 3,767 |
| ITGA6 | integrin subunit alpha 6 | **1,271** | 0 | 21,935 | 34,734 |
| ABTB2 | ankyrin repeat and BTB domain containing 2 | **1,271** | 0 | 112,054 | 174,949 |

| | | | | | |
|---|---|---|---|---|---|
| NOS1AP | nitric oxide synthase 1 adaptor protein | **1,270** | 0,059948 | 1,163 | 1,979 |
| NOXRED1 | NADP dependent oxidoreductase domain containing 1 | **1,270** | 0,126992 | 0,332 | 1,724 |
| ENPP2 | ectonucleotide pyrophosphatase/phosphodiesterase 2 | **1,270** | 0 | 10,524 | 8,684 |
| FAM83D | family with sequence similarity 83 member D | **1,270** | 0,008757 | 3,213 | 3,129 |
| CD276 | CD276 molecule | **1,270** | 0 | 113,107 | 97,690 |
| PPP1R12A-AS1 | PPP1R12A antisense RNA 1 | **1,270** | 0,040638 | 0,665 | 1,022 |
| C1QTNF1-AS1 | C1QTNF1 antisense RNA 1 | **1,269** | 0,238974 | 0,222 | 0,255 |
| RN7SL124P | RNA, 7SL, cytoplasmic 124, pseudogene | **1,269** | 0,108996 | 0,886 | 0,702 |
| HIST1H2AE | histone cluster 1 H2A family member e | **1,269** | 0,331407 | 0,332 | 0,702 |
| LIFR | LIF receptor alpha | **1,268** | 0 | 48,799 | 52,357 |
| EFCAB12 | EF-hand calcium binding domain 12 | **1,268** | 0,246931 | 0,111 | 0,383 |
| CDNF | cerebral dopamine neurotrophic factor | **1,268** | 0,074806 | 0,609 | 1,469 |
| ST3GAL6 | ST3 beta-galactoside alpha-2,3-sialyltransferase 6 | **1,267** | 0,028649 | 1,385 | 1,149 |
| HAVCR2 | hepatitis A virus cellular receptor 2 | **1,267** | 0,086209 | 0,388 | 0,638 |
| ETV5 | ETS variant 5 | **1,267** | 0 | 80,870 | 102,479 |
| RASSF2 | Ras association domain family member 2 | **1,267** | 0,272517 | 0,166 | 0,511 |
| LPAR6 | lysophosphatidic acid receptor 6 | **1,267** | 0,330296 | 0,111 | 0,255 |
| GRIK3 | glutamate ionotropic receptor kainate type subunit 3 | **1,267** | 0,017187 | 1,496 | 2,490 |
| GRAMD4 | GRAM domain containing 4 | **1,266** | 0 | 21,602 | 30,456 |
| ZBTB2 | zinc finger and BTB domain containing 2 | **1,266** | 0 | 12,740 | 13,153 |
| LINC00877 | long intergenic non-protein coding RNA 877 | **1,266** | 0,131284 | 0,388 | 0,447 |
| TTN | titin | **1,266** | 0,02546 | 2,548 | 4,406 |
| TRPV3 | transient receptor potential cation channel subfamily V member 3 | **1,265** | 0,003117 | 2,160 | 4,342 |
| GRIN2C | glutamate ionotropic receptor NMDA type subunit 2C | **1,265** | 0,000547 | 0,554 | 2,299 |
| CAMK2D | calcium/calmodulin dependent protein kinase II delta | **1,265** | 0 | 71,896 | 80,259 |
| PCF11-AS1 | PCF11 antisense RNA 1 | **1,265** | 0,095774 | 0,720 | 0,958 |
| GABRB3 | gamma-aminobutyric acid type A receptor beta3 subunit | **1,265** | 0,224672 | 0,222 | 0,511 |
| NKD1 | naked cuticle homolog 1 | **1,264** | 0,008257 | 6,536 | 6,640 |
| SLC22A1 | solute carrier family 22 member 1 | **1,264** | 0,014181 | 1,274 | 1,149 |
| LY75 | lymphocyte antigen 75 | **1,264** | 0,030472 | 0,831 | 2,618 |
| GOLGA2P5 | golgin A2 pseudogene 5 | **1,264** | 0,023595 | 1,329 | 1,724 |
| SLC15A2 | solute carrier family 15 member 2 | **1,264** | 0,03604 | 0,720 | 1,405 |
| LCP2 | lymphocyte cytosolic protein 2 | **1,264** | 0,104117 | 0,222 | 0,830 |

| | | | | | |
|---|---|---|---|---|---|
| PAX8-AS1 | PAX8 antisense RNA 1 | **1,263** | 0 | 65,527 | 20,943 |
| CCNG2 | cyclin G2 | **1,263** | 0 | 59,655 | 56,954 |
| FAM221A | family with sequence similarity 221 member A | **1,263** | 0,000637 | 1,828 | 3,256 |
| CFAP70 | cilia and flagella associated protein 70 | **1,263** | 0,021687 | 1,329 | 2,426 |
| LBX2-AS1 | LBX2 antisense RNA 1 | **1,263** | 5,60E-05 | 5,373 | 5,491 |
| LRRC8C | leucine rich repeat containing 8 family member C | **1,263** | 0,012954 | 8,696 | 8,939 |
| ITPR3 | inositol 1,4,5-trisphosphate receptor type 3 | **1,263** | 0 | 323,589 | 366,754 |
| BPI | bactericidal/permeability-increasing protein | **1,262** | 3,40E-05 | 7,367 | 4,469 |
| MAB21L3 | mab-21 like 3 | **1,262** | 0,248976 | 0,332 | 0,702 |
| ARID5A | AT-rich interaction domain 5A | **1,262** | 0 | 8,641 | 12,962 |
| STRIP2 | striatin interacting protein 2 | **1,262** | 0 | 18,390 | 24,646 |
| PALM3 | paralemmin 3 | **1,262** | 0,194112 | 0,443 | 0,319 |
| GPR37L1 | G protein-coupled receptor 37 like 1 | **1,262** | 0 | 19,830 | 27,008 |
| ENOX1 | ecto-NOX disulfide-thiol exchanger 1 | **1,261** | 0,001121 | 2,382 | 3,512 |
| MMP7 | matrix metallopeptidase 7 | **1,261** | 0,287563 | 0,166 | 0,128 |
| RFESD | Rieske Fe-S domain containing | **1,261** | 0,026985 | 0,997 | 0,702 |
| HSPA12A | heat shock protein family A (Hsp70) member 12A | **1,261** | 3,00E-06 | 10,746 | 12,706 |
| FOXN3 | forkhead box N3 | **1,261** | 0 | 28,747 | 27,902 |
| ST6GALNAC3 | ST6 N-acetylgalactosaminide alpha-2,6-sialyltransferase 3 | **1,261** | 0,140788 | 0,277 | 0,447 |
| ACKR3 | atypical chemokine receptor 3 | **1,261** | 0 | 80,648 | 185,229 |
| ITGA9 | integrin subunit alpha 9 | **1,260** | 0,002065 | 2,548 | 3,639 |
| AURKC | aurora kinase C | **1,260** | 0,23857 | 0,166 | 0,575 |
| HK2 | hexokinase 2 | **1,260** | 0 | 96,268 | 152,857 |
| HSPA8P4 | heat shock protein family A (Hsp70) member 8 pseudogene 4 | **1,260** | 0,08364 | 0,499 | 0,894 |
| ELF1 | E74 like ETS transcription factor 1 | **1,260** | 0 | 53,064 | 74,066 |
| CACNB1 | calcium voltage-gated channel auxiliary subunit beta 1 | **1,260** | 1,00E-06 | 5,594 | 7,662 |
| SORBS2 | sorbin and SH3 domain containing 2 | **1,260** | 8,20E-05 | 6,481 | 8,173 |
| AVIL | advillin | **1,259** | 1,00E-06 | 8,530 | 12,834 |
| VAMP1 | vesicle associated membrane protein 1 | **1,259** | 1,10E-05 | 7,145 | 16,026 |
| LYRM9 | LYR motif containing 9 | **1,259** | 0 | 5,041 | 7,790 |
| FLCN | folliculin | **1,259** | 0 | 41,487 | 54,400 |
| EPB41L4A | erythrocyte membrane protein band 4.1 like 4A | **1,259** | 5,90E-05 | 3,711 | 4,789 |
| C1orf162 | chromosome 1 open reading frame 162 | **1,259** | 0,139274 | 0,388 | 1,277 |

| | | | | | |
|---|---|---|---|---|---|
| FN3K | fructosamine 3 kinase | **1,259** | 0,000886 | 3,213 | 2,235 |
| MYLIP | myosin regulatory light chain interacting protein | **1,259** | 0 | 10,690 | 22,220 |
| ASIC1 | acid sensing ion channel subunit 1 | **1,259** | 0,136961 | 0,443 | 0,511 |
| MT-CO1 | mitochondrially encoded cytochrome c oxidase I | **1,258** | 0 | 1629,135 | 1416,701 |
| OLFM1 | olfactomedin 1 | **1,258** | 0 | 34,508 | 38,821 |
| LYPD5 | LY6/PLAUR domain containing 5 | **1,258** | 0,173626 | 0,222 | 0,383 |
| PI3 | peptidase inhibitor 3 | **1,258** | 0,000307 | 10,746 | 12,387 |
| ZNF441 | zinc finger protein 441 | **1,258** | 5,20E-05 | 6,813 | 10,024 |
| RAB29 | RAB29, member RAS oncogene family | **1,258** | 0 | 51,014 | 52,165 |
| MT-CYB | mitochondrially encoded cytochrome b | **1,258** | 4,00E-05 | 773,634 | 858,653 |
| HOTAIRM1 | HOXA transcript antisense RNA, myeloid-specific 1 | **1,258** | 0,051383 | 0,665 | 1,469 |
| WDR97 | WD repeat domain 97 | **1,258** | 0,020459 | 0,775 | 2,107 |
| SPOCK3 | SPARC/osteonectin, cwcv and kazal like domains proteoglycan 3 | **1,257** | 0,048397 | 2,936 | 0,128 |
| PUS10 | pseudouridylate synthase 10 | **1,257** | 2,00E-06 | 5,871 | 8,428 |
| ZCCHC14 | zinc finger CCHC-type containing 14 | **1,257** | 0 | 64,419 | 81,153 |
| OSBPL1A | oxysterol binding protein like 1A | **1,257** | 0 | 11,300 | 11,174 |
| CYP2E1 | cytochrome P450 family 2 subfamily E member 1 | **1,257** | 0,400954 | 0,111 | 0,192 |
| SAMD4A | sterile alpha motif domain containing 4A | **1,257** | 0 | 101,364 | 96,924 |
| ADGRG1 | adhesion G protein-coupled receptor G1 | **1,257** | 0 | 13,515 | 21,517 |
| TRIM22 | tripartite motif containing 22 | **1,256** | 0,192313 | 0,443 | 0,830 |
| CAPS2 | calcyphosine 2 | **1,256** | 0,033731 | 0,831 | 1,469 |
| QPCT | glutaminyl-peptide cyclotransferase | **1,256** | 0,002598 | 2,271 | 2,362 |
| PCBP4 | poly(rC) binding protein 4 | **1,256** | 0,007349 | 3,600 | 1,724 |
| ZCCHC24 | zinc finger CCHC-type containing 24 | **1,256** | 0 | 31,849 | 38,885 |
| DSG2 | desmoglein 2 | **1,256** | 0 | 35,948 | 27,455 |
| RYR1 | ryanodine receptor 1 | **1,256** | 0,084199 | 0,609 | 0,702 |
| MMP1 | matrix metallopeptidase 1 | **1,255** | 0 | 12148,835 | 11637,024 |
| SPRY1 | sprouty RTK signaling antagonist 1 | **1,255** | 0,143085 | 0,388 | 1,532 |
| RNF32 | ring finger protein 32 | **1,255** | 0,044668 | 0,997 | 1,341 |
| AHCYL2 | adenosylhomocysteinase like 2 | **1,255** | 0 | 155,037 | 198,126 |
| TNFSF14 | TNF superfamily member 14 | **1,255** | 0,179921 | 0,277 | 0,766 |
| GAL | galanin and GMAP prepropeptide | **1,255** | 0,040569 | 0,831 | 0,958 |
| GDF11 | growth differentiation factor 11 | **1,254** | 0,000922 | 4,320 | 3,831 |

| | | | | | |
|---|---|---|---|---|---|
| FOXA3 | forkhead box A3 | **1,254** | 3,40E-05 | 3,434 | 3,448 |
| RGS5 | regulator of G protein signaling 5 | **1,254** | 0,00491 | 2,160 | 3,384 |
| DND1 | DND microRNA-mediated repression inhibitor 1 | **1,253** | 0,230699 | 0,388 | 1,149 |
| SPTBN4 | spectrin beta, non-erythrocytic 4 | **1,253** | 0,04703 | 0,831 | 1,532 |
| NEO1 | neogenin 1 | **1,253** | 0 | 40,878 | 69,022 |
| PIWIL4 | piwi like RNA-mediated gene silencing 4 | **1,253** | 0 | 11,466 | 15,579 |
| MAP3K7CL | MAP3K7 C-terminal like | **1,253** | 0 | 16,506 | 15,643 |
| SOX13 | SRY-box 13 | **1,253** | 0,000113 | 3,711 | 5,491 |
| FAR2P2 | fatty acyl-CoA reductase 2 pseudogene 2 | **1,253** | 0,090059 | 0,499 | 1,213 |
| SSTR2 | somatostatin receptor 2 | **1,252** | 0 | 40,158 | 84,920 |
| TMPRSS5 | transmembrane protease, serine 5 | **1,252** | 0,145156 | 0,554 | 0,638 |
| UBR5-AS1 | UBR5 antisense RNA 1 | **1,251** | 0,115334 | 0,665 | 0,766 |
| FNIP1 | folliculin interacting protein 1 | **1,251** | 0 | 53,119 | 66,212 |
| HIST1H2BG | histone cluster 1 H2B family member g | **1,251** | 0,004245 | 2,160 | 2,618 |
| TMEM65 | transmembrane protein 65 | **1,251** | 0 | 10,912 | 14,494 |
| PIK3IP1 | phosphoinositide-3-kinase interacting protein 1 | **1,251** | 0 | 41,155 | 38,693 |
| RET | ret proto-oncogene | **1,250** | 0,003661 | 1,939 | 2,554 |
| MTATP6P1 | mitochondrially encoded ATP synthase 6 pseudogene 1 | **1,250** | 0 | 168,386 | 186,122 |
| DNAJC27-AS1 | DNAJC27 antisense RNA 1 | **1,250** | 0,008091 | 0,997 | 2,171 |
| GCNA | germ cell nuclear acidic peptidase | **1,250** | 0,006884 | 2,548 | 3,320 |
| TTLL9 | tubulin tyrosine ligase like 9 | **1,249** | 0,213591 | 0,332 | 0,064 |
| TUBB4B | tubulin beta 4B class IVb | **1,249** | 0 | 300,769 | 572,670 |
| WDFY3-AS2 | WDFY3 antisense RNA 2 | **1,249** | 6,30E-05 | 4,597 | 6,257 |
| ZBTB7C | zinc finger and BTB domain containing 7C | **1,249** | 0 | 6,647 | 18,261 |
| ZNF548 | zinc finger protein 548 | **1,249** | 3,00E-06 | 6,758 | 6,513 |
| SYCE1L | synaptonemal complex central element protein 1 like | **1,249** | 0,24055 | 0,332 | 0,447 |
| ZNF256 | zinc finger protein 256 | **1,249** | 0,027394 | 1,163 | 1,277 |
| GET4 | golgi to ER traffic protein 4 | **1,248** | 0,002302 | 4,265 | 6,130 |
| HMCN1 | hemicentin 1 | **1,248** | 0,002015 | 9,472 | 6,704 |
| RNF19A | ring finger protein 19A, RBR E3 ubiquitin protein ligase | **1,248** | 0 | 29,745 | 34,224 |
| MINOS1P3 | mitochondrial inner membrane organizing system 1 pseudogene 3 | **1,248** | 0,271992 | 0,111 | 0,447 |
| RELL1 | RELT like 1 | **1,248** | 0,000145 | 11,687 | 9,705 |
| BTN3A3 | butyrophilin subfamily 3 member A3 | **1,248** | 0,005487 | 3,268 | 4,342 |

| | | | | | |
|---|---|---|---|---|---|
| BTN2A3P | butyrophilin subfamily 2 member A3, pseudogene | **1,248** | 0,001005 | 5,927 | 4,789 |
| HIST1H2BN | histone cluster 1 H2B family member n | **1,248** | 0,037148 | 0,554 | 1,660 |
| HOXD10 | homeobox D10 | **1,248** | 0,16674 | 0,332 | 1,341 |
| RNF112 | ring finger protein 112 | **1,248** | 0,172349 | 0,277 | 0,255 |
| TMEM88 | transmembrane protein 88 | **1,248** | 0,231178 | 0,111 | 0,319 |
| PLEKHG1 | pleckstrin homology and RhoGEF domain containing G1 | **1,247** | 0,030354 | 3,213 | 1,213 |
| NOTCH2 | notch 2 | **1,247** | 1,00E-06 | 970,214 | 1136,272 |
| EDA | ectodysplasin A | **1,247** | 0,030603 | 2,991 | 2,554 |
| EPHB3 | EPH receptor B3 | **1,247** | 0 | 15,509 | 22,986 |
| CGNL1 | cingulin like 1 | **1,247** | 0 | 61,815 | 67,553 |
| C8orf48 | chromosome 8 open reading frame 48 | **1,247** | 0,000103 | 3,988 | 3,001 |
| SPDYE18 | speedy/RINGO cell cycle regulator family member E18 | **1,247** | 0,329531 | 0,388 | 0,319 |
| APOBEC3G | apolipoprotein B mRNA editing enzyme catalytic subunit 3G | **1,247** | 0,062049 | 1,440 | 2,618 |
| GOLGA7B | golgin A7 family member B | **1,247** | 0,005265 | 2,160 | 2,171 |
| TTC32 | tetratricopeptide repeat domain 32 | **1,246** | 0,066495 | 1,052 | 1,788 |
| MDGA1 | MAM domain containing glycosylphosphatidylinositol anchor 1 | **1,246** | 0 | 7,035 | 13,153 |
| BRICD5 | BRICHOS domain containing 5 | **1,246** | 0,003416 | 1,939 | 2,873 |
| RN7SKP16 | RNA, 7SK small nuclear pseudogene 16 | **1,246** | 0,295246 | 0,222 | 0,255 |
| NRBP2 | nuclear receptor binding protein 2 | **1,246** | 0,000203 | 10,358 | 6,704 |
| DNAJB1 | DnaJ heat shock protein family (Hsp40) member B1 | **1,246** | 0 | 78,654 | 115,185 |
| PPP1R3C | protein phosphatase 1 regulatory subunit 3C | **1,245** | 0 | 149,110 | 270,915 |
| ADRA2A | adrenoceptor alpha 2A | **1,245** | 0,002418 | 1,828 | 3,001 |
| PFKFB1 | 6-phosphofructo-2-kinase/fructose-2,6-biphosphatase 1 | **1,245** | 0,290041 | 0,222 | 0,383 |
| SMG1P5 | SMG1P5, nonsense mediated mRNA decay associated PI3K related kinase pseudogene 5 | **1,245** | 0,167844 | 0,332 | 0,638 |
| KANSL1L | KAT8 regulatory NSL complex subunit 1 like | **1,245** | 0 | 30,298 | 35,692 |
| HS6ST1 | heparan sulfate 6-O-sulfotransferase 1 | **1,245** | 0 | 28,027 | 37,608 |
| VGLL4 | vestigial like family member 4 | **1,244** | 0 | 95,437 | 126,678 |
| TOB1 | transducer of ERBB2, 1 | **1,244** | 0 | 48,079 | 71,512 |
| LINC02062 | long intergenic non-protein coding RNA 2062 | **1,244** | 0,126527 | 0,332 | 1,022 |
| PARP14 | poly(ADP-ribose) polymerase family member 14 | **1,244** | 2,00E-06 | 42,817 | 66,915 |
| SLCO2B1 | solute carrier organic anion transporter family member 2B1 | **1,244** | 0,046512 | 1,329 | 2,554 |
| MAP3K8 | mitogen-activated protein kinase kinase kinase 8 | **1,244** | 0 | 84,027 | 93,987 |
| RCAN3 | RCAN family member 3 | **1,244** | 0 | 14,291 | 14,941 |

| | | | | | |
|---|---|---|---|---|---|
| PIM1 | Pim-1 proto-oncogene, serine/threonine kinase | **1,244** | 0 | 48,300 | 76,237 |
| ARNTL | aryl hydrocarbon receptor nuclear translocator like | **1,244** | 4,30E-05 | 8,918 | 15,324 |
| TUBB2A | tubulin beta 2A class IIa | **1,244** | 4,10E-05 | 12,075 | 16,793 |
| SPAG9 | sperm associated antigen 9 | **1,244** | 0 | 193,921 | 215,685 |
| LINC01277 | long intergenic non-protein coding RNA 1277 | **1,243** | 0,227223 | 0,277 | 0,575 |
| SLC13A3 | solute carrier family 13 member 3 | **1,243** | 0 | 10,801 | 18,133 |
| COL9A1 | collagen type IX alpha 1 chain | **1,243** | 0 | 17,282 | 6,002 |
| VWC2 | von Willebrand factor C domain containing 2 | **1,243** | 0 | 25,424 | 17,495 |
| MEF2A | myocyte enhancer factor 2A | **1,243** | 0 | 69,182 | 81,281 |
| CDKN2B | cyclin dependent kinase inhibitor 2B | **1,243** | 0 | 548,806 | 578,416 |
| PCSK5 | proprotein convertase subtilisin/kexin type 5 | **1,243** | 0,002467 | 12,463 | 10,918 |
| BTG2 | BTG anti-proliferation factor 2 | **1,243** | 0 | 113,882 | 165,180 |
| EFCC1 | EF-hand and coiled-coil domain containing 1 | **1,243** | 0,272305 | 0,166 | 0,511 |
| KCTD12 | potassium channel tetramerization domain containing 12 | **1,243** | 0 | 37,278 | 60,657 |
| AMPH | amphiphysin | **1,243** | 0,00583 | 2,548 | 5,555 |
| KLF13 | Kruppel like factor 13 | **1,243** | 0,004544 | 3,988 | 1,660 |
| ADAM22 | ADAM metallopeptidase domain 22 | **1,242** | 0,011287 | 1,108 | 2,873 |
| UBXN10 | UBX domain protein 10 | **1,242** | 0,006753 | 1,108 | 1,532 |
| DNM1 | dynamin 1 | **1,242** | 0 | 11,687 | 24,774 |
| PCDH9 | protocadherin 9 | **1,242** | 0 | 25,867 | 23,050 |
| ITGA7 | integrin subunit alpha 7 | **1,242** | 0,04514 | 1,385 | 2,490 |
| KCNC3 | potassium voltage-gated channel subfamily C member 3 | **1,242** | 3,70E-05 | 14,845 | 15,579 |
| ZNF362 | zinc finger protein 362 | **1,241** | 0 | 14,845 | 12,706 |
| BACH1 | BTB domain and CNC homolog 1 | **1,241** | 0 | 93,111 | 94,242 |
| NOCT | nocturnin | **1,241** | 0 | 16,949 | 17,303 |
| LRCH2 | leucine rich repeats and calponin homology domain containing 2 | **1,241** | 0,103827 | 0,332 | 1,405 |
| SPATA1 | spermatogenesis associated 1 | **1,241** | 0,085682 | 0,609 | 1,341 |
| SLC45A3 | solute carrier family 45 member 3 | **1,241** | 0,248506 | 0,499 | 0,766 |
| DDIT3 | DNA damage inducible transcript 3 | **1,241** | 0 | 53,285 | 73,810 |
| JPH1 | junctophilin 1 | **1,241** | 0,013404 | 3,323 | 2,043 |
| CPAMD8 | C3 and PZP like, alpha-2-macroglobulin domain containing 8 | **1,241** | 0 | 113,328 | 101,330 |
| CXCR4 | C-X-C motif chemokine receptor 4 | **1,241** | 0,068772 | 0,886 | 0,766 |
| C1orf228 | chromosome 1 open reading frame 228 | **1,241** | 0,12974 | 0,499 | 0,447 |

| | | | | | |
|---|---|---|---|---|---|
| FABP3 | fatty acid binding protein 3 | **1,241** | 0,011899 | 3,102 | 2,937 |
| TNFRSF18 | TNF receptor superfamily member 18 | **1,241** | 0,139471 | 0,111 | 0,383 |
| ZC3H12C | zinc finger CCCH-type containing 12C | **1,241** | 0 | 30,908 | 42,716 |
| FAM102B | family with sequence similarity 102 member B | **1,240** | 0,002526 | 3,988 | 5,300 |
| NRROS | negative regulator of reactive oxygen species | **1,240** | 0,02484 | 1,717 | 1,469 |
| ZIK1 | zinc finger protein interacting with K protein 1 | **1,240** | 0,005671 | 1,883 | 2,043 |
| STK4-AS1 | STK4 antisense RNA 1 (head to head) | **1,240** | 0,196168 | 0,222 | 0,830 |
| CFAP54 | cilia and flagella associated protein 54 | **1,240** | 0,021719 | 1,551 | 1,915 |
| SP4 | Sp4 transcription factor | **1,240** | 0 | 13,515 | 19,155 |
| CHRNA10 | cholinergic receptor nicotinic alpha 10 subunit | **1,240** | 0,081888 | 0,388 | 1,022 |
| LIPE-AS1 | LIPE antisense RNA 1 | **1,240** | 0,305802 | 0,388 | 0,638 |
| CD72 | CD72 molecule | **1,239** | 0,086937 | 0,499 | 0,830 |
| MCOLN3 | mucolipin 3 | **1,239** | 9,80E-05 | 6,093 | 6,066 |
| RBM38 | RNA binding motif protein 38 | **1,239** | 2,00E-05 | 5,041 | 8,811 |
| PEX13 | peroxisomal biogenesis factor 13 | **1,239** | 0 | 28,304 | 35,947 |
| MAOB | monoamine oxidase B | **1,239** | 0 | 141,910 | 201,957 |
| LRIG3 | leucine rich repeats and immunoglobulin like domains 3 | **1,239** | 1,00E-06 | 19,664 | 30,712 |
| XKR9 | XK related 9 | **1,238** | 0,057123 | 1,108 | 0,255 |
| AFF1 | AF4/FMR2 family member 1 | **1,238** | 6,80E-05 | 57,495 | 63,211 |
| PON3 | paraoxonase 3 | **1,238** | 0,002475 | 1,828 | 3,129 |
| ITPR1 | inositol 1,4,5-trisphosphate receptor type 1 | **1,238** | 5,00E-06 | 18,777 | 32,116 |
| SLAMF8 | SLAM family member 8 | **1,238** | 0,000536 | 2,714 | 5,363 |
| IDO1 | indoleamine 2,3-dioxygenase 1 | **1,238** | 0,049056 | 1,274 | 1,915 |
| APOL3 | apolipoprotein L3 | **1,238** | 0 | 100,644 | 249,525 |
| IL4I1 | interleukin 4 induced 1 | **1,238** | 0 | 15,066 | 18,197 |
| GPR84 | G protein-coupled receptor 84 | **1,238** | 0 | 13,183 | 19,921 |
| TSC22D1 | TSC22 domain family member 1 | **1,238** | 0 | 196,524 | 204,767 |
| HES6 | hes family bHLH transcription factor 6 | **1,237** | 0,000817 | 10,026 | 17,495 |
| KCND3 | potassium voltage-gated channel subfamily D member 3 | **1,237** | 0 | 7,145 | 11,812 |
| GADD45A | growth arrest and DNA damage inducible alpha | **1,237** | 0 | 55,058 | 85,942 |
| ZFP14 | ZFP14 zinc finger protein | **1,237** | 4,10E-05 | 5,096 | 5,683 |
| PACSIN2 | protein kinase C and casein kinase substrate in neurons 2 | **1,237** | 0 | 161,019 | 202,468 |
| LBX2 | ladybird homeobox 2 | **1,237** | 0,108945 | 1,052 | 1,213 |

| | | | | | |
|---|---|---|---|---|---|
| AQP7P2 | aquaporin 7 pseudogene 2 | **1,236** | 0,090336 | 0,111 | 0,192 |
| CARD14 | caspase recruitment domain family member 14 | **1,236** | 0,116806 | 0,443 | 1,213 |
| MIR99AHG | mir-99a-let-7c cluster host gene | **1,236** | 0,009084 | 1,662 | 2,682 |
| IRAK2 | interleukin 1 receptor associated kinase 2 | **1,236** | 0 | 172,208 | 209,683 |
| GPC4 | glypican 4 | **1,236** | 0,074361 | 1,883 | 1,277 |
| NTSR2 | neurotensin receptor 2 | **1,236** | 0,231509 | 0,277 | 0,511 |
| MT-ATP8 | mitochondrially encoded ATP synthase 8 | **1,236** | 3,00E-06 | 76,660 | 101,202 |
| ZNF136 | zinc finger protein 136 | **1,235** | 1,00E-06 | 7,588 | 10,727 |
| WDR93 | WD repeat domain 93 | **1,235** | 0,201216 | 0,277 | 0,383 |
| IL17C | interleukin 17C | **1,235** | 0,019864 | 1,939 | 2,554 |
| VAV3 | vav guanine nucleotide exchange factor 3 | **1,235** | 0,000221 | 7,533 | 6,321 |
| PIK3C2B | phosphatidylinositol-4-phosphate 3-kinase catalytic subunit type 2 beta | **1,235** | 0,249293 | 0,665 | 0,511 |
| ANKDD1A | ankyrin repeat and death domain containing 1A | **1,235** | 0,002972 | 2,216 | 3,256 |
| ACRBP | acrosin binding protein | **1,235** | 0,07487 | 0,554 | 0,830 |
| SH2D3C | SH2 domain containing 3C | **1,234** | 0,129579 | 0,499 | 1,469 |
| HDAC9 | histone deacetylase 9 | **1,234** | 0 | 15,786 | 19,347 |
| SEMA3G | semaphorin 3G | **1,234** | 0,008012 | 0,997 | 1,660 |
| N4BP3 | NEDD4 binding protein 3 | **1,234** | 9,80E-05 | 4,930 | 5,619 |
| TSPAN14 | tetraspanin 14 | **1,234** | 5,00E-06 | 11,909 | 10,471 |
| FANCD2 | Fanconi anemia complementation group D2 | **1,233** | 8,60E-05 | 7,699 | 8,364 |
| CNNM2 | cyclin and CBS domain divalent metal cation transport mediator 2 | **1,233** | 0 | 16,617 | 20,496 |
| COL5A3 | collagen type V alpha 3 chain | **1,232** | 0,012358 | 2,271 | 3,895 |
| ZNF836 | zinc finger protein 836 | **1,231** | 0,000687 | 2,825 | 2,873 |
| SMAD9 | SMAD family member 9 | **1,231** | 0,007325 | 4,265 | 4,661 |
| OSBPL6 | oxysterol binding protein like 6 | **1,231** | 1,30E-05 | 6,093 | 8,684 |
| SLC25A34 | solute carrier family 25 member 34 | **1,231** | 0,095453 | 1,662 | 3,512 |
| WASH3P | WAS protein family homolog 3 pseudogene | **1,231** | 0,036181 | 1,662 | 2,554 |
| RNF144A | ring finger protein 144A | **1,231** | 0,020824 | 2,493 | 2,299 |
| ZFP28 | ZFP28 zinc finger protein | **1,231** | 0,000326 | 6,702 | 8,747 |
| LINC01004 | long intergenic non-protein coding RNA 1004 | **1,230** | 0,001375 | 4,542 | 5,683 |
| SPHK1 | sphingosine kinase 1 | **1,230** | 0 | 22,932 | 33,777 |
| GULP1 | GULP, engulfment adaptor PTB domain containing 1 | **1,230** | 0,000273 | 37,665 | 67,936 |
| LINC01963 | long intergenic non-protein coding RNA 1963 | **1,230** | 0,011444 | 3,877 | 3,831 |

| | | | | | |
|---|---|---|---|---|---|
| KIAA1522 | KIAA1522 | **1,230** | 0,002079 | 8,087 | 6,002 |
| RPL15P3 | ribosomal protein L15 pseudogene 3 | **1,230** | 0,115913 | 0,720 | 0,830 |
| ARL6IP6 | ADP ribosylation factor like GTPase 6 interacting protein 6 | **1,230** | 0 | 26,366 | 34,160 |
| PSD4 | pleckstrin and Sec7 domain containing 4 | **1,229** | 0 | 74,721 | 79,046 |
| WDR19 | WD repeat domain 19 | **1,229** | 0 | 31,129 | 39,778 |
| CFAP53 | cilia and flagella associated protein 53 | **1,229** | 0,015147 | 1,551 | 2,746 |
| NTRK2 | neurotrophic receptor tyrosine kinase 2 | **1,229** | 3,00E-06 | 13,072 | 31,223 |
| FLT1 | fms related tyrosine kinase 1 | **1,229** | 0 | 22,211 | 33,585 |
| CACNA1G | calcium voltage-gated channel subunit alpha1 G | **1,229** | 0,095995 | 0,609 | 1,277 |
| FAM222A | family with sequence similarity 222 member A | **1,228** | 0 | 16,229 | 24,710 |
| ABCA12 | ATP binding cassette subfamily A member 12 | **1,228** | 0,014207 | 2,049 | 5,172 |
| CDKL1 | cyclin dependent kinase like 1 | **1,228** | 0,000368 | 4,043 | 8,045 |
| BEX5 | brain expressed X-linked 5 | **1,228** | 0,014276 | 3,213 | 4,278 |
| CRB1 | crumbs 1, cell polarity complex component | **1,228** | 0,285665 | 0,332 | 0,575 |
| PLEKHA6 | pleckstrin homology domain containing A6 | **1,228** | 0,141268 | 0,388 | 0,894 |
| FAM214A | family with sequence similarity 214 member A | **1,228** | 0 | 17,836 | 18,836 |
| LINC02035 | long intergenic non-protein coding RNA 2035 | **1,228** | 0,000453 | 3,268 | 4,023 |
| AFDN | afadin, adherens junction formation factor | **1,228** | 0 | 139,860 | 226,986 |
| HID1 | HID1 domain containing | **1,227** | 0,112054 | 0,388 | 0,192 |
| AFF2 | AF4/FMR2 family member 2 | **1,227** | 0,28427 | 0,831 | 0,511 |
| RHOQ | ras homolog family member Q | **1,227** | 0 | 54,005 | 45,908 |
| PLA2G4C | phospholipase A2 group IVC | **1,227** | 0 | 17,780 | 26,689 |
| PFKFB2 | 6-phosphofructo-2-kinase/fructose-2,6-biphosphatase 2 | **1,227** | 0,000294 | 7,201 | 7,790 |
| ZC3H12B | zinc finger CCCH-type containing 12B | **1,227** | 0,060929 | 1,274 | 1,469 |
| DEPDC1B | DEP domain containing 1B | **1,227** | 0,151732 | 0,775 | 0,894 |
| CRYBG1 | crystallin beta-gamma domain containing 1 | **1,227** | 0,057562 | 1,772 | 1,149 |
| RCBTB1 | RCC1 and BTB domain containing protein 1 | **1,227** | 1,00E-06 | 16,949 | 14,302 |
| RNF145 | ring finger protein 145 | **1,227** | 0 | 66,690 | 68,319 |
| CSPG5 | chondroitin sulfate proteoglycan 5 | **1,227** | 0,007381 | 2,326 | 3,001 |
| ZC3HAV1L | zinc finger CCCH-type containing, antiviral 1 like | **1,227** | 0,089279 | 1,108 | 1,596 |
| LINC01011 | long intergenic non-protein coding RNA 1011 | **1,226** | 0,045585 | 0,997 | 1,596 |
| CERS3 | ceramide synthase 3 | **1,226** | 0,207734 | 0,277 | 0,766 |
| RPS6KA5 | ribosomal protein S6 kinase A5 | **1,226** | 0,0011 | 5,041 | 5,874 |

| | | | | | |
|---|---|---|---|---|---|
| HAND2-AS1 | HAND2 antisense RNA 1 (head to head) | **1,226** | 0,138758 | 2,770 | 1,213 |
| SERTAD1 | SERTA domain containing 1 | **1,226** | 0 | 47,248 | 71,831 |
| RGS3 | regulator of G protein signaling 3 | **1,226** | 0 | 62,203 | 74,385 |
| IP6K3 | inositol hexakisphosphate kinase 3 | **1,226** | 0,04221 | 0,720 | 0,383 |
| CATSPERG | cation channel sperm associated auxiliary subunit gamma | **1,226** | 0,014444 | 1,329 | 2,235 |
| RUBCNL | RUN and cysteine rich domain containing beclin 1 interacting protein like | **1,226** | 0,011948 | 1,717 | 2,362 |
| MAGI2 | membrane associated guanylate kinase, WW and PDZ domain containing 2 | **1,226** | 0,00017 | 6,591 | 10,471 |
| KIAA1161 | KIAA1161 | **1,226** | 7,00E-06 | 9,416 | 9,705 |
| IFNGR2 | interferon gamma receptor 2 | **1,225** | 0,003874 | 9,361 | 7,790 |
| CBX4 | chromobox 4 | **1,225** | 0 | 20,882 | 25,923 |
| GPR156 | G protein-coupled receptor 156 | **1,225** | 0,206493 | 0,554 | 0,638 |
| CPEB4 | cytoplasmic polyadenylation element binding protein 4 | **1,225** | 2,00E-06 | 76,715 | 92,902 |
| ENPP6 | ectonucleotide pyrophosphatase/phosphodiesterase 6 | **1,225** | 0,305654 | 0,388 | 0,638 |
| SLC25A35 | solute carrier family 25 member 35 | **1,225** | 0,136245 | 0,775 | 1,341 |
| SYN1 | synapsin I | **1,225** | 0,00061 | 3,767 | 7,023 |
| CPNE2 | copine 2 | **1,224** | 0 | 16,229 | 15,707 |
| MSX1 | msh homeobox 1 | **1,224** | 0,010277 | 3,822 | 7,215 |
| CCT6P1 | chaperonin containing TCP1 subunit 6 pseudogene 1 | **1,224** | 0,066159 | 0,775 | 1,852 |
| CSRNP2 | cysteine and serine rich nuclear protein 2 | **1,224** | 0 | 39,272 | 51,910 |
| AMZ1 | archaelysin family metallopeptidase 1 | **1,224** | 0,124422 | 0,886 | 0,511 |
| CBLB | Cbl proto-oncogene B | **1,224** | 0 | 21,547 | 25,412 |
| PGR | progesterone receptor | **1,224** | 0,210754 | 0,886 | 0,383 |
| HCG11 | HLA complex group 11 (non-protein coding) | **1,224** | 0 | 27,640 | 32,372 |
| FERMT1 | fermitin family member 1 | **1,224** | 0,00471 | 30,409 | 48,015 |
| NPTN-IT1 | NPTN intronic transcript 1 | **1,223** | 0,098797 | 1,606 | 3,767 |
| LINC01006 | long intergenic non-protein coding RNA 1006 | **1,223** | 0,001818 | 2,714 | 3,001 |
| CDC42SE1 | CDC42 small effector 1 | **1,223** | 0 | 138,309 | 186,059 |
| CLCN5 | chloride voltage-gated channel 5 | **1,223** | 0,00043 | 8,807 | 9,514 |
| C1orf226 | chromosome 1 open reading frame 226 | **1,223** | 0,011739 | 1,662 | 2,873 |
| FAM160A1 | family with sequence similarity 160 member A1 | **1,223** | 0,000507 | 21,768 | 28,605 |
| C14orf132 | chromosome 14 open reading frame 132 | **1,223** | 0 | 32,680 | 43,035 |
| KANK3 | KN motif and ankyrin repeat domains 3 | **1,223** | 0,189435 | 0,388 | 0,447 |
| LYVE1 | lymphatic vessel endothelial hyaluronan receptor 1 | **1,223** | 0,000163 | 8,585 | 5,300 |

| | | | | | |
|---|---|---|---|---|---|
| CDH11 | cadherin 11 | **1,223** | 0 | 9,029 | 11,238 |
| HNRNPKP4 | heterogeneous nuclear ribonucleoprotein K pseudogene 4 | **1,223** | 0,204161 | 0,443 | 0,447 |
| RRAGC | Ras related GTP binding C | **1,223** | 3,00E-06 | 18,445 | 18,453 |
| CCDC126 | coiled-coil domain containing 126 | **1,223** | 3,30E-05 | 6,813 | 7,470 |
| ENDOD1 | endonuclease domain containing 1 | **1,222** | 0 | 47,636 | 59,955 |
| PAPD5 | poly(A) RNA polymerase D5, non-canonical | **1,222** | 0 | 23,098 | 30,265 |
| FAM111A | family with sequence similarity 111 member A | **1,222** | 0 | 35,284 | 43,482 |
| STX1B | syntaxin 1B | **1,222** | 0,093414 | 0,443 | 1,469 |
| KCNS3 | potassium voltage-gated channel modifier subfamily S member 3 | **1,222** | 0,064262 | 0,942 | 1,213 |
| ABLIM1 | actin binding LIM protein 1 | **1,221** | 0,000631 | 7,478 | 10,216 |
| HSPB7 | heat shock protein family B (small) member 7 | **1,221** | 0,049042 | 0,831 | 1,022 |
| PAQR8 | progestin and adipoQ receptor family member 8 | **1,221** | 0,000317 | 6,813 | 6,385 |
| CYP4F11 | cytochrome P450 family 4 subfamily F member 11 | **1,221** | 0,004089 | 5,539 | 0,447 |
| CHST3 | carbohydrate sulfotransferase 3 | **1,221** | 0 | 130,832 | 198,126 |
| ZNF469 | zinc finger protein 469 | **1,221** | 3,00E-06 | 24,926 | 13,919 |
| BBC3 | BCL2 binding component 3 | **1,221** | 1,00E-06 | 10,247 | 12,834 |
| SPON1 | spondin 1 | **1,221** | 0,000303 | 2,714 | 3,512 |
| CCDC112 | coiled-coil domain containing 112 | **1,221** | 0,0507 | 3,213 | 3,895 |
| PNRC1 | proline rich nuclear receptor coactivator 1 | **1,220** | 0 | 363,803 | 388,016 |
| HIST1H2BD | histone cluster 1 H2B family member d | **1,220** | 5,00E-06 | 14,124 | 20,368 |
| LPAL2 | lipoprotein(a) like 2, pseudogene | **1,220** | 0,209253 | 0,166 | 0,766 |
| LRP1B | LDL receptor related protein 1B | **1,220** | 0,004887 | 5,816 | 1,149 |
| L3MBTL4 | l(3)mbt-like 4 (Drosophila) | **1,220** | 0,001077 | 10,026 | 6,704 |
| DZIP1 | DAZ interacting zinc finger protein 1 | **1,220** | 0 | 18,611 | 19,602 |
| ZNF772 | zinc finger protein 772 | **1,220** | 0,003644 | 2,493 | 2,107 |
| ESPL1 | extra spindle pole bodies like 1, separase | **1,220** | 0 | 9,582 | 10,535 |
| VPS13C | vacuolar protein sorting 13 homolog C | **1,219** | 0,000138 | 33,123 | 40,034 |
| AFF3 | AF4/FMR2 family member 3 | **1,219** | 0 | 16,451 | 33,777 |
| NBPF26 | NBPF member 26 | **1,219** | 0,000455 | 6,204 | 7,534 |
| MAP1A | microtubule associated protein 1A | **1,219** | 1,00E-05 | 22,544 | 21,262 |
| DAGLA | diacylglycerol lipase alpha | **1,219** | 0,05341 | 0,886 | 1,532 |
| GALNT13 | polypeptide N-acetylgalactosaminyltransferase 13 | **1,219** | 0,003401 | 2,548 | 2,490 |
| SDK1 | sidekick cell adhesion molecule 1 | **1,219** | 0,000292 | 7,478 | 9,514 |

| | | | | | |
|---|---|---|---|---|---|
| TFPI2 | tissue factor pathway inhibitor 2 | **1,219** | 4,00E-06 | 405,567 | 258,209 |
| EPPK1 | epiplakin 1 | **1,219** | 0,024081 | 0,942 | 1,979 |
| LENG8 | leukocyte receptor cluster member 8 | **1,219** | 0,355017 | 0,388 | 0,064 |
| ZSCAN2 | zinc finger and SCAN domain containing 2 | **1,219** | 0,001085 | 4,099 | 8,045 |
| C5orf30 | chromosome 5 open reading frame 30 | **1,218** | 0,016651 | 2,437 | 2,107 |
| ADORA2A | adenosine A2a receptor | **1,218** | 0,000423 | 3,490 | 9,194 |
| LINC01605 | long intergenic non-protein coding RNA 1605 | **1,218** | 0,010649 | 2,880 | 2,554 |
| PAG1 | phosphoprotein membrane anchor with glycosphingolipid microdomains 1 | **1,218** | 2,00E-06 | 9,693 | 11,940 |
| FNDC1 | fibronectin type III domain containing 1 | **1,218** | 0 | 115,212 | 153,942 |
| BAIAP2 | BAI1 associated protein 2 | **1,217** | 1,00E-06 | 17,060 | 23,624 |
| RAET1L | retinoic acid early transcript 1L | **1,217** | 0,002291 | 2,991 | 6,768 |
| SMG1P3 | SMG1P3, nonsense mediated mRNA decay associated PI3K related kinase pseudogene 3 | **1,217** | 0,376052 | 0,499 | 0,575 |
| UST | uronyl 2-sulfotransferase | **1,217** | 0 | 40,379 | 34,479 |
| CORO1A | coronin 1A | **1,217** | 0,190293 | 0,332 | 0,766 |
| DNAH12 | dynein axonemal heavy chain 12 | **1,217** | 0,327713 | 0,222 | 0,511 |
| NPAS1 | neuronal PAS domain protein 1 | **1,217** | 0,336152 | 0,332 | 0,255 |
| GMPR | guanosine monophosphate reductase | **1,217** | 0,124005 | 0,775 | 0,638 |
| CMTM8 | CKLF like MARVEL transmembrane domain containing 8 | **1,217** | 0,022238 | 1,496 | 2,043 |
| PLEKHH1 | pleckstrin homology, MyTH4 and FERM domain containing H1 | **1,217** | 0,029647 | 1,052 | 2,299 |
| GNG2 | G protein subunit gamma 2 | **1,217** | 1,60E-05 | 8,309 | 13,089 |
| CELSR2 | cadherin EGF LAG seven-pass G-type receptor 2 | **1,216** | 0,013148 | 1,606 | 3,703 |
| ZNF846 | zinc finger protein 846 | **1,216** | 0,005717 | 2,548 | 3,831 |
| FLI1 | Fli-1 proto-oncogene, ETS transcription factor | **1,216** | 1,00E-05 | 11,687 | 12,834 |
| UBALD2 | UBA like domain containing 2 | **1,216** | 0 | 57,440 | 84,154 |
| DNAH14 | dynein axonemal heavy chain 14 | **1,216** | 0,033108 | 2,326 | 2,554 |
| SLC2A1 | solute carrier family 2 member 1 | **1,216** | 0 | 461,622 | 695,261 |
| STEAP4 | STEAP4 metalloreductase | **1,216** | 0 | 35,173 | 27,902 |
| LIN37 | lin-37 DREAM MuvB core complex component | **1,216** | 0,069465 | 0,831 | 1,213 |
| CSRP2 | cysteine and glycine rich protein 2 | **1,215** | 0,271125 | 0,831 | 0,958 |
| SDC4 | syndecan 4 | **1,215** | 0 | 1280,953 | 2215,335 |
| SNHG10 | small nucleolar RNA host gene 10 | **1,215** | 0,028876 | 1,662 | 3,959 |
| COL22A1 | collagen type XXII alpha 1 chain | **1,215** | 0,037396 | 1,883 | 0,958 |
| TMPRSS4 | transmembrane protease, serine 4 | **1,215** | 0,018653 | 0,499 | 1,660 |

| | | | | | |
|---|---|---|---|---|---|
| STX11 | syntaxin 11 | **1,215** | 0 | 28,194 | 30,648 |
| KLHDC9 | kelch domain containing 9 | **1,215** | 0,264626 | 0,775 | 0,447 |
| ZFP30 | ZFP30 zinc finger protein | **1,215** | 0,001005 | 4,431 | 5,363 |
| MYO5B | myosin VB | **1,215** | 0 | 75,995 | 76,109 |
| RTCA-AS1 | RTCA antisense RNA 1 | **1,214** | 0,328053 | 0,499 | 1,022 |
| PPP1R3G | protein phosphatase 1 regulatory subunit 3G | **1,214** | 0,031517 | 1,329 | 1,277 |
| LYN | LYN proto-oncogene, Src family tyrosine kinase | **1,214** | 0 | 48,245 | 71,703 |
| OTUD3 | OTU deubiquitinase 3 | **1,214** | 1,40E-05 | 9,084 | 11,812 |
| RAP2A | RAP2A, member of RAS oncogene family | **1,214** | 0 | 24,593 | 25,476 |
| HHEX | hematopoietically expressed homeobox | **1,214** | 0,039348 | 2,271 | 1,724 |
| LOR | loricrin | **1,214** | 0,305018 | 0,720 | 0,894 |
| UPK3B | uroplakin 3B | **1,214** | 0,28513 | 0,332 | 0,766 |
| GABRB1 | gamma-aminobutyric acid type A receptor beta1 subunit | **1,214** | 0,295597 | 0,332 | 0,766 |
| OSBPL8 | oxysterol binding protein like 8 | **1,214** | 1,00E-06 | 77,103 | 90,922 |
| NT5C2 | 5'-nucleotidase, cytosolic II | **1,214** | 0 | 48,854 | 49,037 |
| TRIM62 | tripartite motif containing 62 | **1,213** | 0,000783 | 10,746 | 12,131 |
| C16orf59 | chromosome 16 open reading frame 59 | **1,213** | 0,072963 | 0,720 | 1,660 |
| FXYD6 | FXYD domain containing ion transport regulator 6 | **1,213** | 0 | 82,587 | 55,102 |
| ZNF204P | zinc finger protein 204, pseudogene | **1,213** | 0,084994 | 1,163 | 1,469 |
| DNM3 | dynamin 3 | **1,213** | 0,224668 | 0,720 | 1,213 |
| SPTBN2 | spectrin beta, non-erythrocytic 2 | **1,213** | 0,266614 | 0,554 | 0,830 |
| ZNF181 | zinc finger protein 181 | **1,213** | 1,00E-06 | 17,503 | 22,284 |
| TONSL | tonsoku like, DNA repair protein | **1,212** | 0,021492 | 1,828 | 1,405 |
| PPP3CB-AS1 | PPP3CB antisense RNA 1 (head to head) | **1,212** | 1,10E-05 | 5,816 | 8,045 |
| MAPK8IP2 | mitogen-activated protein kinase 8 interacting protein 2 | **1,212** | 3,10E-05 | 8,087 | 6,640 |
| TCF7 | transcription factor 7 (T-cell specific, HMG-box) | **1,212** | 0,024227 | 1,883 | 2,873 |
| CNIH3 | cornichon family AMPA receptor auxiliary protein 3 | **1,212** | 0,030655 | 1,329 | 1,469 |
| MEGF6 | multiple EGF like domains 6 | **1,212** | 0 | 23,762 | 18,516 |
| DNM1P46 | dynamin 1 pseudogene 46 | **1,212** | 0,157637 | 1,496 | 1,405 |
| FHIT | fragile histidine triad | **1,212** | 0,164804 | 0,499 | 0,447 |
| COL14A1 | collagen type XIV alpha 1 chain | **1,212** | 1,70E-05 | 12,851 | 17,878 |
| SQSTM1 | sequestosome 1 | **1,212** | 0 | 277,615 | 238,990 |
| NACA3P | NACA family member 3 pseudogene | **1,212** | 0,183811 | 0,443 | 0,319 |

| | | | | | |
|---|---|---|---|---|---|
| MISP3 | MISP family member 3 | **1,212** | 0,132351 | 1,163 | 1,213 |
| NFKBIZ | NFKB inhibitor zeta | **1,212** | 0 | 219,123 | 379,588 |
| ITGB3 | integrin subunit beta 3 | **1,212** | 0,008091 | 6,758 | 7,151 |
| ZNF90 | zinc finger protein 90 | **1,211** | 0,239469 | 0,499 | 0,383 |
| NIPSNAP3B | nipsnap homolog 3B | **1,211** | 0,11286 | 0,775 | 1,022 |
| C9orf16 | chromosome 9 open reading frame 16 | **1,211** | 2,00E-06 | 99,592 | 106,693 |
| SPCS2P4 | signal peptidase complex subunit 2 homolog (S. cerevisiae) pseudogene 4 | **1,211** | 0,291955 | 0,277 | 0,255 |
| BEST1 | bestrophin 1 | **1,211** | 0,033184 | 85,301 | 123,166 |
| CACNA1H | calcium voltage-gated channel subunit alpha1 H | **1,211** | 0,05823 | 2,880 | 0,894 |
| TTYH2 | tweety family member 2 | **1,211** | 0,020837 | 2,493 | 2,618 |
| SLC16A10 | solute carrier family 16 member 10 | **1,211** | 0,003926 | 5,982 | 6,193 |
| CMTM1 | CKLF like MARVEL transmembrane domain containing 1 | **1,211** | 0,25213 | 0,609 | 0,702 |
| SEMA3F | semaphorin 3F | **1,211** | 0,007056 | 6,314 | 7,534 |
| MT-TP | mitochondrially encoded tRNA proline | **1,211** | 0,250288 | 0,388 | 0,383 |
| MAFG | MAF bZIP transcription factor G | **1,210** | 0 | 100,367 | 101,394 |
| TNKS | tankyrase | **1,210** | 0,109234 | 0,775 | 1,596 |
| PYCARD-AS1 | PYCARD antisense RNA 1 | **1,210** | 0,251435 | 0,443 | 0,447 |
| PCSK6 | proprotein convertase subtilisin/kexin type 6 | **1,210** | 0,020951 | 1,717 | 0,894 |
| AUTS2 | AUTS2, activator of transcription and developmental regulator | **1,210** | 0 | 91,394 | 103,373 |
| ATP8A1 | ATPase phospholipid transporting 8A1 | **1,210** | 0,009749 | 1,274 | 3,512 |
| HAS3 | hyaluronan synthase 3 | **1,210** | 0,001523 | 6,702 | 7,981 |
| MAPKBP1 | mitogen-activated protein kinase binding protein 1 | **1,210** | 0 | 48,743 | 58,167 |
| ANK3 | ankyrin 3 | **1,209** | 0,006322 | 3,102 | 8,173 |
| NEDD4L | neural precursor cell expressed, developmentally down-regulated 4-like, E3 ubiquitin prot | **1,209** | 0 | 24,039 | 30,520 |
| PDLIM5 | PDZ and LIM domain 5 | **1,209** | 0 | 171,266 | 177,183 |
| INTS6L | integrator complex subunit 6 like | **1,209** | 0,000262 | 11,244 | 11,046 |
| FTH1P3 | ferritin heavy chain 1 pseudogene 3 | **1,209** | 0,129573 | 0,665 | 0,575 |
| DDI2 | DNA damage inducible 1 homolog 2 | **1,209** | 2,20E-05 | 32,403 | 46,419 |
| PHF6 | PHD finger protein 6 | **1,209** | 2,60E-05 | 16,174 | 19,219 |
| MT-ATP6 | mitochondrially encoded ATP synthase 6 | **1,208** | 9,00E-06 | 732,535 | 787,333 |
| GINS3 | GINS complex subunit 3 | **1,208** | 8,60E-05 | 9,195 | 11,046 |
| PM20D2 | peptidase M20 domain containing 2 | **1,208** | 1,00E-06 | 15,454 | 21,454 |
| PCDHGA3 | protocadherin gamma subfamily A, 3 | **1,208** | 0,002217 | 3,434 | 5,938 |

| | | | | | |
|---|---|---|---|---|---|
| OTUD1 | OTU deubiquitinase 1 | **1,208** | 0 | 16,174 | 18,644 |
| PRDM2 | PR/SET domain 2 | **1,208** | 3,00E-06 | 95,382 | 99,861 |
| DSP | desmoplakin | **1,208** | 3,00E-06 | 33,345 | 48,909 |
| EFTUD1P1 | elongation factor Tu GTP binding domain containing 1 pseudogene 1 | **1,208** | 0,251018 | 0,277 | 0,894 |
| RAP1B | RAP1B, member of RAS oncogene family | **1,208** | 0 | 50,294 | 62,892 |
| SCNN1A | sodium channel epithelial 1 alpha subunit | **1,208** | 0,005931 | 1,883 | 3,703 |
| MAK | male germ cell associated kinase | **1,208** | 0,161799 | 0,831 | 0,830 |
| FTH1P10 | ferritin heavy chain 1 pseudogene 10 | **1,208** | 0,2606 | 0,277 | 0,319 |
| SLC2A1-AS1 | SLC2A1 antisense RNA 1 | **1,208** | 0,053122 | 0,831 | 1,213 |
| APC2 | APC2, WNT signaling pathway regulator | **1,207** | 0,25744 | 0,388 | 1,341 |
| TRIM68 | tripartite motif containing 68 | **1,207** | 2,00E-06 | 10,912 | 14,749 |
| SORT1 | sortilin 1 | **1,207** | 0 | 49,242 | 71,129 |
| NAP1L1P1 | nucleosome assembly protein 1 like 1 pseudogene 1 | **1,207** | 0,307618 | 0,332 | 0,447 |
| ZFYVE16 | zinc finger FYVE-type containing 16 | **1,207** | 1,00E-06 | 79,208 | 79,685 |
| CD44-AS1 | CD44 antisense RNA 1 | **1,207** | 0,432968 | 0,222 | 1,022 |
| C3orf52 | chromosome 3 open reading frame 52 | **1,206** | 0,010983 | 2,825 | 3,192 |
| HMGA1P2 | high mobility group AT-hook 1 pseudogene 2 | **1,206** | 0,274302 | 0,166 | 0,447 |
| KCNAB2 | potassium voltage-gated channel subfamily A regulatory beta subunit 2 | **1,206** | 0,000224 | 5,539 | 5,236 |
| LAMA2 | laminin subunit alpha 2 | **1,206** | 0,040079 | 1,496 | 2,362 |
| ZNF585A | zinc finger protein 585A | **1,206** | 0,001453 | 6,425 | 9,641 |
| FGFR3 | fibroblast growth factor receptor 3 | **1,206** | 8,20E-05 | 36,170 | 36,650 |
| CHKA | choline kinase alpha | **1,206** | 0 | 16,340 | 22,347 |
| C20orf204 | chromosome 20 open reading frame 204 | **1,206** | 0,18708 | 0,720 | 2,043 |
| SYP | synaptophysin | **1,206** | 0,0032 | 4,099 | 7,917 |
| B4GALNT4 | beta-1,4-N-acetyl-galactosaminyltransferase 4 | **1,206** | 0,417807 | 0,443 | 0,383 |
| PRSS53 | protease, serine 53 | **1,205** | 0,070597 | 0,886 | 1,405 |
| PANO1 | proapoptotic nucleolar protein 1 | **1,205** | 0,219233 | 0,222 | 0,255 |
| STX7 | syntaxin 7 | **1,205** | 0 | 51,790 | 47,313 |
| GRHL1 | grainyhead like transcription factor 1 | **1,205** | 0,000514 | 3,545 | 7,726 |
| CCDC141 | coiled-coil domain containing 141 | **1,205** | 0,007706 | 2,049 | 3,639 |
| APTR | Alu-mediated CDKN1A/p21 transcriptional regulator (non-protein coding) | **1,205** | 0,002314 | 4,320 | 5,427 |
| NBL1 | neuroblastoma 1, DAN family BMP antagonist | **1,205** | 0 | 21,658 | 27,519 |
| SLC25A27 | solute carrier family 25 member 27 | **1,205** | 0,075026 | 2,271 | 2,235 |

| | | | | | |
|---|---|---|---|---|---|
| SLC16A6 | solute carrier family 16 member 6 | **1,205** | 0,167001 | 1,329 | 0,766 |
| STEAP2 | STEAP2 metalloreductase | **1,205** | 0 | 78,931 | 95,839 |
| EDIL3 | EGF like repeats and discoidin domains 3 | **1,205** | 0 | 591,235 | 448,354 |
| FAM21FP | family with sequence similarity 21 member F, pseudogene | **1,204** | 0,221629 | 0,332 | 0,447 |
| RGS17 | regulator of G protein signaling 17 | **1,204** | 0,048332 | 2,326 | 1,085 |
| SH3BP5 | SH3 domain binding protein 5 | **1,204** | 0 | 67,521 | 91,944 |
| ENPP1 | ectonucleotide pyrophosphatase/phosphodiesterase 1 | **1,204** | 1,00E-06 | 126,899 | 108,992 |
| CHST7 | carbohydrate sulfotransferase 7 | **1,204** | 0,002564 | 7,035 | 9,131 |
| ANXA4 | annexin A4 | **1,204** | 0 | 38,496 | 33,330 |
| NEB | nebulin | **1,203** | 0,189032 | 0,997 | 1,405 |
| ELMSAN1 | ELM2 and Myb/SANT domain containing 1 | **1,203** | 0,000323 | 26,920 | 45,078 |
| PCDHGA4 | protocadherin gamma subfamily A, 4 | **1,203** | 0,020155 | 1,662 | 1,660 |
| FAM83B | family with sequence similarity 83 member B | **1,203** | 0,023124 | 4,154 | 2,618 |
| EDNRA | endothelin receptor type A | **1,203** | 0,134114 | 1,052 | 0,383 |
| ZNF296 | zinc finger protein 296 | **1,202** | 0,294215 | 0,554 | 0,447 |
| PDXP | pyridoxal phosphatase | **1,202** | 0,008457 | 3,102 | 2,873 |
| PHLPP1 | PH domain and leucine rich repeat protein phosphatase 1 | **1,202** | 0,000219 | 9,970 | 12,387 |
| FZD5 | frizzled class receptor 5 | **1,202** | 0 | 17,669 | 21,390 |
| ERN1 | endoplasmic reticulum to nucleus signaling 1 | **1,202** | 0 | 242,886 | 397,976 |
| CLDN4 | claudin 4 | **1,202** | 0,182609 | 0,886 | 1,341 |
| CNTFR | ciliary neurotrophic factor receptor | **1,202** | 0,001682 | 3,988 | 6,577 |
| CKB | creatine kinase B | **1,202** | 0,00035 | 11,466 | 18,836 |
| FMO4 | flavin containing monooxygenase 4 | **1,201** | 0,000407 | 5,207 | 6,002 |
| HECTD2 | HECT domain E3 ubiquitin protein ligase 2 | **1,201** | 2,40E-05 | 13,626 | 23,178 |
| MT-ND4L | mitochondrially encoded NADH:ubiquinone oxidoreductase core subunit 4L | **1,201** | 0,000297 | 208,156 | 283,366 |
| CRISPLD1 | cysteine rich secretory protein LCCL domain containing 1 | **1,201** | 0,017651 | 4,099 | 4,023 |
| TLL2 | tolloid like 2 | **1,201** | 0,058247 | 1,440 | 2,171 |
| TPP1 | tripeptidyl peptidase 1 | **1,201** | 0 | 91,560 | 112,440 |
| C1RL-AS1 | C1RL antisense RNA 1 | **1,201** | 0,002217 | 8,807 | 11,429 |
| ANO6 | anoctamin 6 | **1,201** | 1,00E-06 | 55,390 | 57,529 |
| SLC7A8 | solute carrier family 7 member 8 | **1,201** | 6,90E-05 | 6,591 | 6,385 |
| ADHFE1 | alcohol dehydrogenase, iron containing 1 | **1,201** | 0,076888 | 0,775 | 1,596 |
| MMP10 | matrix metallopeptidase 10 | **1,201** | 0,000567 | 19,830 | 25,285 |

| | | | | | |
|---|---|---|---|---|---|
| ARHGAP31 | Rho GTPase activating protein 31 | **1,201** | 6,00E-06 | 35,117 | 44,056 |
| DBIL5P | diazepam binding inhibitor-like 5, pseudogene | **1,200** | 0,398431 | 0,111 | 0,575 |
| ATP10D | ATPase phospholipid transporting 10D (putative) | **1,200** | 1,80E-05 | 19,830 | 27,519 |
| PSD3 | pleckstrin and Sec7 domain containing 3 | **1,200** | 0,000165 | 307,138 | 315,865 |
| MT-RNR2 | mitochondrially encoded 16S RNA | **1,200** | 6,00E-06 | 2316,749 | 2318,070 |
| MIR34AHG | MIR34A host gene | **1,200** | 0,086999 | 1,219 | 2,362 |
| ITPKB | inositol-trisphosphate 3-kinase B | **1,200** | 0,005198 | 3,822 | 5,810 |
| DRC3 | dynein regulatory complex subunit 3 | **1,200** | 0,003772 | 3,268 | 5,236 |
| GCHFR | GTP cyclohydrolase I feedback regulator | **1,200** | 0,252342 | 0,609 | 0,830 |
| TRIB1 | tribbles pseudokinase 1 | **1,200** | 0 | 87,073 | 133,893 |
| DNAJC22 | DnaJ heat shock protein family (Hsp40) member C22 | **1,200** | 0,056073 | 1,717 | 2,171 |
| DIXDC1 | DIX domain containing 1 | **1,200** | 0,001446 | 17,448 | 18,580 |
| NR6A1 | nuclear receptor subfamily 6 group A member 1 | **1,199** | 0,149572 | 0,775 | 0,830 |
| POLR3G | RNA polymerase III subunit G | **1,199** | 0,243352 | 0,886 | 0,702 |
| STAG3L2 | stromal antigen 3-like 2 (pseudogene) | **1,199** | 0,013051 | 2,493 | 2,873 |
| GNPDA1 | glucosamine-6-phosphate deaminase 1 | **1,199** | 0 | 48,356 | 71,256 |
| NRGN | neurogranin | **1,199** | 0,011068 | 3,877 | 5,619 |
| CLK1 | CDC like kinase 1 | **1,198** | 0 | 88,181 | 129,104 |
| ZNF23 | zinc finger protein 23 | **1,198** | 0,143113 | 0,942 | 1,277 |
| CLEC18B | C-type lectin domain family 18 member B | **1,198** | 0,382233 | 0,332 | 0,319 |
| EPS8 | epidermal growth factor receptor pathway substrate 8 | **1,198** | 0 | 387,233 | 499,051 |
| ZNF57 | zinc finger protein 57 | **1,198** | 0,034816 | 2,216 | 2,299 |
| KIAA1614-AS1 | KIAA1614 antisense RNA 1 | **1,198** | 0,155937 | 0,554 | 1,469 |
| HAPLN1 | hyaluronan and proteoglycan link protein 1 | **1,198** | 0 | 669,722 | 549,428 |
| DYRK3 | dual specificity tyrosine phosphorylation regulated kinase 3 | **1,197** | 0 | 31,185 | 42,716 |
| ZC2HC1A | zinc finger C2HC-type containing 1A | **1,197** | 0,010499 | 5,650 | 3,959 |
| NBPF19 | NBPF member 19 | **1,197** | 0,000104 | 8,807 | 9,577 |
| RAD9B | RAD9 checkpoint clamp component B | **1,197** | 0,143185 | 0,997 | 0,830 |
| AMPD3 | adenosine monophosphate deaminase 3 | **1,197** | 0 | 126,567 | 145,705 |
| HAND2 | heart and neural crest derivatives expressed 2 | **1,197** | 0,141143 | 1,606 | 1,788 |
| DIRAS1 | DIRAS family GTPase 1 | **1,196** | 0,029363 | 3,545 | 2,937 |
| GPR137B | G protein-coupled receptor 137B | **1,196** | 1,80E-05 | 11,023 | 15,069 |
| USP49 | ubiquitin specific peptidase 49 | **1,196** | 0,000509 | 5,428 | 11,876 |

| | | | | | |
|---|---|---|---|---|---|
| BEND5 | BEN domain containing 5 | **1,196** | 0,11892 | 0,609 | 0,575 |
| HIST2H2BF | histone cluster 2 H2B family member f | **1,196** | 0,058212 | 1,274 | 2,682 |
| RLF | rearranged L-myc fusion | **1,196** | 0 | 31,240 | 42,396 |
| ATP6V1B2 | ATPase H+ transporting V1 subunit B2 | **1,196** | 0 | 134,709 | 181,589 |
| ZNRF2 | zinc and ring finger 2 | **1,195** | 0,000149 | 11,909 | 11,174 |
| CYP3A5 | cytochrome P450 family 3 subfamily A member 5 | **1,195** | 0 | 28,194 | 140,278 |
| IKZF1 | IKAROS family zinc finger 1 | **1,195** | 0,26936 | 0,831 | 1,149 |
| FBXL3 | F-box and leucine rich repeat protein 3 | **1,195** | 0 | 75,829 | 94,115 |
| CACNA1E | calcium voltage-gated channel subunit alpha1 E | **1,195** | 0,451078 | 0,388 | 0,638 |
| KATNAL2 | katanin catalytic subunit A1 like 2 | **1,195** | 0,149755 | 0,665 | 1,213 |
| BHLHE40 | basic helix-loop-helix family member e40 | **1,195** | 0 | 131,552 | 205,724 |
| RNF19B | ring finger protein 19B | **1,195** | 0 | 111,500 | 197,615 |
| FOSB | FosB proto-oncogene, AP-1 transcription factor subunit | **1,195** | 0,003451 | 3,822 | 9,961 |
| PLXNC1 | plexin C1 | **1,195** | 0,207227 | 0,554 | 1,405 |
| FOXL1 | forkhead box L1 | **1,195** | 0,001838 | 2,880 | 3,448 |
| ACOT7 | acyl-CoA thioesterase 7 | **1,195** | 1,00E-06 | 44,478 | 67,042 |
| TRIM47 | tripartite motif containing 47 | **1,195** | 0 | 26,809 | 28,030 |
| MAGI2-AS3 | MAGI2 antisense RNA 3 | **1,194** | 0 | 35,560 | 33,138 |
| GXYLT1 | glucoside xylosyltransferase 1 | **1,194** | 0 | 38,552 | 48,271 |
| KLF3 | Kruppel like factor 3 | **1,194** | 1,00E-06 | 82,753 | 137,405 |
| LINC01629 | long intergenic non-protein coding RNA 1629 | **1,194** | 0,37836 | 0,000 | 0,575 |
| PHF10 | PHD finger protein 10 | **1,194** | 0 | 31,295 | 33,521 |
| CCDC96 | coiled-coil domain containing 96 | **1,194** | 0,011547 | 2,160 | 3,256 |
| TNFSF4 | TNF superfamily member 4 | **1,194** | 0,134047 | 0,665 | 0,766 |
| ENKUR | enkurin, TRPC channel interacting protein | **1,194** | 1,20E-05 | 7,921 | 14,941 |
| BMP6 | bone morphogenetic protein 6 | **1,194** | 0 | 130,444 | 248,121 |
| DNAH17 | dynein axonemal heavy chain 17 | **1,194** | 0,11767 | 0,886 | 3,512 |
| PATJ | PATJ, crumbs cell polarity complex component | **1,193** | 0,001534 | 6,924 | 8,109 |
| SARDH | sarcosine dehydrogenase | **1,193** | 0,373769 | 0,720 | 0,894 |
| RABEP1 | rabaptin, RAB GTPase binding effector protein 1 | **1,193** | 0 | 54,338 | 61,807 |
| ZKSCAN8 | zinc finger with KRAB and SCAN domains 8 | **1,193** | 0,000924 | 42,041 | 54,655 |
| CHD5 | chromodomain helicase DNA binding protein 5 | **1,193** | 0,228297 | 0,055 | 0,511 |
| SRGAP2B | SLIT-ROBO Rho GTPase activating protein 2B | **1,192** | 0,032839 | 1,994 | 2,235 |

| | | | | | |
|---|---|---|---|---|---|
| FRY | FRY microtubule binding protein | **1,192** | 0 | 44,478 | 67,553 |
| KIF26A | kinesin family member 26A | **1,192** | 0,006751 | 4,708 | 4,342 |
| ZNF845 | zinc finger protein 845 | **1,192** | 0,000351 | 7,090 | 8,364 |
| MAML2 | mastermind like transcriptional coactivator 2 | **1,192** | 0,000103 | 98,650 | 133,893 |
| HIST1H2BJ | histone cluster 1 H2B family member j | **1,192** | 0,002585 | 5,428 | 7,087 |
| CHRNB2 | cholinergic receptor nicotinic beta 2 subunit | **1,192** | 0,174201 | 0,388 | 0,830 |
| NET1 | neuroepithelial cell transforming 1 | **1,192** | 1,20E-05 | 17,171 | 19,538 |
| RMI2 | RecQ mediated genome instability 2 | **1,192** | 0,1092 | 1,219 | 1,213 |
| CYTH3 | cytohesin 3 | **1,192** | 1,20E-05 | 17,116 | 21,390 |
| NBPF10 | NBPF member 10 | **1,192** | 0,00042 | 5,982 | 4,789 |
| CCL28 | C-C motif chemokine ligand 28 | **1,192** | 0,00074 | 6,038 | 6,768 |
| ACTL10 | actin like 10 | **1,191** | 0,093679 | 0,942 | 1,213 |
| MRAP2 | melanocortin 2 receptor accessory protein 2 | **1,191** | 0,125814 | 0,942 | 2,299 |
| NUDT10 | nudix hydrolase 10 | **1,191** | 0,170053 | 0,609 | 0,894 |
| ANXA5 | annexin A5 | **1,191** | 0 | 921,028 | 976,648 |
| NUDT14 | nudix hydrolase 14 | **1,191** | 0,016903 | 5,151 | 2,937 |
| CCDC62 | coiled-coil domain containing 62 | **1,191** | 0,351866 | 0,332 | 0,192 |
| FTH1P12 | ferritin heavy chain 1 pseudogene 12 | **1,191** | 0,046624 | 1,385 | 1,022 |
| ZBTB46 | zinc finger and BTB domain containing 46 | **1,191** | 4,00E-06 | 12,463 | 14,366 |
| ZNF117 | zinc finger protein 117 | **1,191** | 0,017323 | 6,536 | 8,875 |
| TNKS2-AS1 | TNKS2 antisense RNA 1 (head to head) | **1,191** | 0,217313 | 0,277 | 1,213 |
| LRRC55 | leucine rich repeat containing 55 | **1,191** | 0,198842 | 0,499 | 1,405 |
| NR3C2 | nuclear receptor subfamily 3 group C member 2 | **1,191** | 0,011193 | 3,213 | 4,597 |
| SWT1 | SWT1, RNA endoribonuclease homolog | **1,191** | 0,001934 | 5,207 | 6,321 |
| CD82 | CD82 molecule | **1,190** | 0 | 527,037 | 626,495 |
| FRRS1 | ferric chelate reductase 1 | **1,190** | 0,161397 | 1,052 | 1,469 |
| DCAF12L2 | DDB1 and CUL4 associated factor 12 like 2 | **1,190** | 0,13785 | 1,329 | 1,596 |
| FGF13 | fibroblast growth factor 13 | **1,190** | 0,049414 | 3,988 | 1,405 |
| TRANK1 | tetratricopeptide repeat and ankyrin repeat containing 1 | **1,190** | 0,000124 | 11,466 | 13,855 |
| NMB | neuromedin B | **1,190** | 1,40E-05 | 12,352 | 21,134 |
| FLVCR2 | feline leukemia virus subgroup C cellular receptor family member 2 | **1,190** | 0,002457 | 5,927 | 5,810 |
| TEC | tec protein tyrosine kinase | **1,190** | 0,179843 | 0,332 | 1,085 |
| RPARP-AS1 | RPARP antisense RNA 1 | **1,190** | 0,177273 | 0,609 | 0,830 |

| | | | | | |
|---|---|---|---|---|---|
| SAV1 | salvador family WW domain containing protein 1 | **1,190** | 0 | 79,983 | 83,963 |
| APOL1 | apolipoprotein L1 | **1,189** | 0 | 33,345 | 29,371 |
| MIR222HG | MIR222 host gene | **1,189** | 0,022717 | 3,102 | 4,725 |
| RAMP2 | receptor activity modifying protein 2 | **1,189** | 0,364245 | 0,499 | 0,192 |
| DOPEY2 | dopey family member 2 | **1,189** | 0,000535 | 14,235 | 15,196 |
| GPD1L | glycerol-3-phosphate dehydrogenase 1-like | **1,189** | 0,003484 | 4,597 | 5,746 |
| CHM | CHM, Rab escort protein 1 | **1,189** | 1,00E-06 | 40,712 | 49,101 |
| SPRR2E | small proline rich protein 2E | **1,189** | 0,010926 | 1,219 | 3,703 |
| GNRH1 | gonadotropin releasing hormone 1 | **1,189** | 0,192604 | 0,942 | 1,213 |
| AP1AR | adaptor related protein complex 1 associated regulatory protein | **1,189** | 2,90E-05 | 14,124 | 16,856 |
| RFX3-AS1 | RFX3 antisense RNA 1 | **1,189** | 0,149268 | 0,443 | 1,085 |
| ASPHD1 | aspartate beta-hydroxylase domain containing 1 | **1,189** | 0,223917 | 0,609 | 0,511 |
| FARP2 | FERM, ARH/RhoGEF and pleckstrin domain protein 2 | **1,188** | 0,00033 | 20,771 | 23,305 |
| RAB30 | RAB30, member RAS oncogene family | **1,188** | 0,00011 | 19,054 | 16,920 |
| SLF2 | SMC5-SMC6 complex localization factor 2 | **1,188** | 3,00E-06 | 26,753 | 37,544 |
| NOTCH3 | notch 3 | **1,188** | 0,000954 | 11,577 | 3,576 |
| CIT | citron rho-interacting serine/threonine kinase | **1,188** | 0,183465 | 4,265 | 0,383 |
| USP2 | ubiquitin specific peptidase 2 | **1,188** | 0,176157 | 1,219 | 1,149 |
| SNHG19 | small nucleolar RNA host gene 19 | **1,188** | 0,001744 | 6,591 | 11,046 |
| ZEB1-AS1 | ZEB1 antisense RNA 1 | **1,188** | 0,000525 | 7,755 | 9,897 |
| ATP2A3 | ATPase sarcoplasmic/endoplasmic reticulum Ca2+ transporting 3 | **1,187** | 0,249788 | 1,219 | 1,341 |
| TCEA1P2 | transcription elongation factor A1 pseudogene 2 | **1,187** | 0,356401 | 0,443 | 0,128 |
| STARD9 | StAR related lipid transfer domain containing 9 | **1,187** | 0,003784 | 9,416 | 10,024 |
| ZNF773 | zinc finger protein 773 | **1,187** | 0,009894 | 2,382 | 4,150 |
| FOXO3 | forkhead box O3 | **1,187** | 0 | 86,852 | 94,945 |
| BTBD19 | BTB domain containing 19 | **1,187** | 0,005118 | 7,533 | 9,003 |
| KIAA0825 | KIAA0825 | **1,187** | 0,079013 | 1,496 | 2,171 |
| LINC01384 | long intergenic non-protein coding RNA 1384 | **1,186** | 0,41991 | 0,111 | 0,575 |
| PARP12 | poly(ADP-ribose) polymerase family member 12 | **1,186** | 0 | 18,666 | 24,646 |
| ITGAV | integrin subunit alpha V | **1,186** | 9,00E-06 | 199,737 | 187,910 |
| FYTTD1 | forty-two-three domain containing 1 | **1,186** | 0 | 65,028 | 74,768 |
| ZNF365 | zinc finger protein 365 | **1,186** | 0,206843 | 1,385 | 1,213 |
| YPEL2 | yippee like 2 | **1,186** | 0 | 20,827 | 23,624 |

| | | | | | |
|---|---|---|---|---|---|
| ZBTB10 | zinc finger and BTB domain containing 10 | **1,186** | 1,00E-06 | 22,544 | 25,604 |
| ACSL3 | acyl-CoA synthetase long-chain family member 3 | **1,186** | 0 | 358,596 | 410,363 |
| SLC25A36 | solute carrier family 25 member 36 | **1,186** | 5,60E-05 | 41,543 | 45,972 |
| ZFP36L1 | ZFP36 ring finger protein like 1 | **1,186** | 0 | 293,512 | 353,664 |
| GPR137C | G protein-coupled receptor 137C | **1,186** | 0,255248 | 0,665 | 0,255 |
| FCMR | Fc fragment of IgM receptor | **1,186** | 2,30E-05 | 9,804 | 12,898 |
| HEXIM1 | hexamethylene bisacetamide inducible 1 | **1,186** | 0 | 84,636 | 129,232 |
| KLHL11 | kelch like family member 11 | **1,186** | 0,041007 | 5,428 | 6,321 |
| ATP1B2 | ATPase Na+/K+ transporting subunit beta 2 | **1,185** | 0,009159 | 3,213 | 4,597 |
| LAMB3 | laminin subunit beta 3 | **1,185** | 0 | 2255,377 | 3900,206 |
| YWHAH | tyrosine 3-monooxygenase/tryptophan 5-monooxygenase activation protein eta | **1,185** | 0 | 114,768 | 146,025 |
| MYO18A | myosin XVIIIA | **1,185** | 0,000179 | 24,039 | 35,628 |
| MAL2 | mal, T-cell differentiation protein 2 (gene/pseudogene) | **1,185** | 0,260861 | 0,111 | 0,638 |
| TPT1P9 | tumor protein, translationally-controlled 1 pseudogene 9 | **1,185** | 0,463249 | 0,222 | 0,128 |
| GATM | glycine amidinotransferase | **1,185** | 0,038046 | 4,708 | 4,597 |
| XRCC3 | X-ray repair cross complementing 3 | **1,185** | 0,001312 | 5,151 | 8,109 |
| ZNF107 | zinc finger protein 107 | **1,185** | 0,125357 | 1,994 | 1,788 |
| STAG3L5P | stromal antigen 3-like 5 pseudogene | **1,184** | 0,101164 | 1,274 | 1,915 |
| CDYL | chromodomain Y like | **1,184** | 0 | 21,491 | 27,455 |
| ADGRV1 | adhesion G protein-coupled receptor V1 | **1,184** | 0,132103 | 1,219 | 1,788 |
| AQP7 | aquaporin 7 | **1,184** | 0,244028 | 0,443 | 0,319 |
| TPK1 | thiamin pyrophosphokinase 1 | **1,184** | 0,330099 | 0,609 | 0,511 |
| HILPDA | hypoxia inducible lipid droplet associated | **1,184** | 0,000125 | 15,897 | 19,283 |
| ZNF563 | zinc finger protein 563 | **1,184** | 0,048796 | 1,662 | 2,171 |
| S100A16 | S100 calcium binding protein A16 | **1,184** | 0 | 33,622 | 73,172 |
| ERRFI1 | ERBB receptor feedback inhibitor 1 | **1,184** | 2,00E-06 | 614,222 | 826,154 |
| HECTD4 | HECT domain E3 ubiquitin protein ligase 4 | **1,184** | 0,000498 | 34,951 | 44,567 |
| ITGA5 | integrin subunit alpha 5 | **1,184** | 0 | 501,724 | 757,834 |
| RNF157 | ring finger protein 157 | **1,183** | 0,128746 | 1,274 | 1,852 |
| IL2RG | interleukin 2 receptor subunit gamma | **1,183** | 5,00E-05 | 5,594 | 7,726 |
| INA | internexin neuronal intermediate filament protein alpha | **1,183** | 0,138846 | 0,609 | 1,149 |
| ARHGAP5-AS1 | ARHGAP5 antisense RNA 1 (head to head) | **1,183** | 0,006445 | 2,049 | 4,278 |
| BTBD8 | BTB domain containing 8 | **1,183** | 0,033373 | 3,767 | 3,256 |

| | | | | | |
|---|---|---|---|---|---|
| SOWAHC | sosondowah ankyrin repeat domain family member C | **1,183** | 0 | 42,263 | 37,480 |
| FTH1P20 | ferritin heavy chain 1 pseudogene 20 | **1,183** | 0,10356 | 0,997 | 0,702 |
| TBX19 | T-box 19 | **1,183** | 0,02032 | 2,493 | 2,873 |
| ZFHX2 | zinc finger homeobox 2 | **1,183** | 0,175235 | 0,720 | 1,022 |
| PNLIPRP3 | pancreatic lipase related protein 3 | **1,183** | 0,391882 | 0,332 | 0,511 |
| TP53I11 | tumor protein p53 inducible protein 11 | **1,183** | 0,067493 | 1,606 | 1,724 |
| TNS3 | tensin 3 | **1,183** | 0 | 260,001 | 305,011 |
| KIF21A | kinesin family member 21A | **1,182** | 0,000332 | 13,515 | 23,433 |
| FZD4 | frizzled class receptor 4 | **1,182** | 0,003834 | 10,247 | 13,855 |
| JAK1 | Janus kinase 1 | **1,182** | 0 | 194,863 | 212,492 |
| SSH2 | slingshot protein phosphatase 2 | **1,182** | 0,007952 | 4,210 | 6,577 |
| H3F3C | H3 histone family member 3C | **1,182** | 0,383052 | 0,277 | 0,383 |
| HSPB8 | heat shock protein family B (small) member 8 | **1,182** | 1,00E-06 | 36,447 | 42,652 |
| ASNS | asparagine synthetase (glutamine-hydrolyzing) | **1,182** | 0,008939 | 8,752 | 10,727 |
| ST6GAL1 | ST6 beta-galactoside alpha-2,6-sialyltransferase 1 | **1,182** | 0 | 50,682 | 53,953 |
| FBLN2 | fibulin 2 | **1,182** | 0,022613 | 1,385 | 1,277 |
| SDHAP1 | succinate dehydrogenase complex flavoprotein subunit A pseudogene 1 | **1,182** | 0,006952 | 3,102 | 3,129 |
| SLC4A3 | solute carrier family 4 member 3 | **1,181** | 0,098845 | 1,163 | 0,702 |
| GSKIP | GSK3B interacting protein | **1,181** | 0 | 25,978 | 28,094 |
| EGLN3 | egl-9 family hypoxia inducible factor 3 | **1,181** | 1,00E-06 | 18,833 | 28,285 |
| WDR47 | WD repeat domain 47 | **1,181** | 2,00E-06 | 30,465 | 32,308 |
| SLCO3A1 | solute carrier organic anion transporter family member 3A1 | **1,181** | 0 | 58,547 | 73,108 |
| LINC00704 | long intergenic non-protein coding RNA 704 | **1,181** | 0,469153 | 0,388 | 0,319 |
| PCF11 | PCF11 cleavage and polyadenylation factor subunit | **1,181** | 0 | 60,154 | 86,325 |
| FOXO1 | forkhead box O1 | **1,181** | 0 | 30,908 | 45,397 |
| FLNC | filamin C | **1,181** | 0,023499 | 1,828 | 3,001 |
| RAPGEF5 | Rap guanine nucleotide exchange factor 5 | **1,181** | 0,022761 | 1,052 | 2,043 |
| TTL | tubulin tyrosine ligase | **1,181** | 0 | 56,498 | 64,169 |
| KDM7A | lysine demethylase 7A | **1,181** | 0,000242 | 129,558 | 284,324 |
| ANKRD31 | ankyrin repeat domain 31 | **1,181** | 0,34635 | 0,222 | 0,638 |
| DDX43 | DEAD-box helicase 43 | **1,180** | 0,204193 | 1,108 | 0,128 |
| ARL6IP1 | ADP ribosylation factor like GTPase 6 interacting protein 1 | **1,180** | 0 | 51,956 | 52,293 |
| SERPINE2 | serpin family E member 2 | **1,180** | 0 | 10393,189 | 7581,024 |

| | | | | | |
|---|---|---|---|---|---|
| SMPDL3A | sphingomyelin phosphodiesterase acid like 3A | **1,180** | 5,00E-04 | 6,868 | 6,704 |
| GLI2 | GLI family zinc finger 2 | **1,180** | 0,033946 | 4,099 | 4,086 |
| TET3 | tet methylcytosine dioxygenase 3 | **1,180** | 0,00537 | 14,457 | 20,368 |
| ACBD7 | acyl-CoA binding domain containing 7 | **1,180** | 0,238308 | 0,831 | 1,213 |
| EGLN2 | egl-9 family hypoxia inducible factor 2 | **1,180** | 0,017046 | 3,545 | 4,278 |
| DCLK2 | doublecortin like kinase 2 | **1,180** | 0,153953 | 1,772 | 1,341 |
| ZNF280C | zinc finger protein 280C | **1,179** | 0,007059 | 3,988 | 6,385 |
| TMEM107 | transmembrane protein 107 | **1,179** | 0,040306 | 3,046 | 2,809 |
| MAP1LC3B | microtubule associated protein 1 light chain 3 beta | **1,179** | 0 | 189,933 | 198,126 |
| KIAA1841 | KIAA1841 | **1,179** | 0,004546 | 5,982 | 4,661 |
| ZNF84 | zinc finger protein 84 | **1,179** | 1,00E-06 | 23,873 | 30,520 |
| ING1 | inhibitor of growth family member 1 | **1,179** | 0 | 18,777 | 25,221 |
| CEP68 | centrosomal protein 68 | **1,179** | 0,000555 | 14,955 | 12,068 |
| YAF2 | YY1 associated factor 2 | **1,179** | 0,000257 | 8,530 | 12,323 |
| DTX3L | deltex E3 ubiquitin ligase 3L | **1,179** | 0 | 28,083 | 32,372 |
| WDR17 | WD repeat domain 17 | **1,179** | 0,060299 | 1,717 | 3,256 |
| ZNF615 | zinc finger protein 615 | **1,179** | 0,001503 | 8,032 | 9,897 |
| CSTB | cystatin B | **1,179** | 0 | 151,769 | 127,444 |
| ZNF554 | zinc finger protein 554 | **1,179** | 0,036965 | 4,320 | 4,214 |
| KCNIP3 | potassium voltage-gated channel interacting protein 3 | **1,179** | 0,226963 | 0,665 | 0,894 |
| C1orf198 | chromosome 1 open reading frame 198 | **1,179** | 0 | 73,669 | 86,708 |
| KIAA0040 | KIAA0040 | **1,178** | 1,00E-06 | 87,184 | 117,548 |
| SESN3 | sestrin 3 | **1,178** | 1,00E-06 | 140,857 | 145,322 |
| AGT | angiotensinogen | **1,178** | 2,90E-05 | 7,201 | 6,768 |
| PCAT6 | prostate cancer associated transcript 6 (non-protein coding) | **1,178** | 0,382869 | 0,166 | 0,128 |
| ZNF254 | zinc finger protein 254 | **1,178** | 0,06185 | 2,437 | 2,490 |
| MAN2A2 | mannosidase alpha class 2A member 2 | **1,178** | 3,00E-06 | 32,846 | 41,630 |
| ZMYND12 | zinc finger MYND-type containing 12 | **1,178** | 0,053261 | 1,496 | 2,490 |
| CABLES2 | Cdk5 and Abl enzyme substrate 2 | **1,178** | 0,000178 | 11,023 | 13,025 |
| LINC01285 | long intergenic non-protein coding RNA 1285 | **1,178** | 0,443966 | 0,388 | 0,255 |
| ICA1L | islet cell autoantigen 1 like | **1,177** | 0,036057 | 1,828 | 2,937 |
| ABCC5 | ATP binding cassette subfamily C member 5 | **1,177** | 0,000157 | 10,524 | 18,836 |
| CRYBG3 | crystallin beta-gamma domain containing 3 | **1,177** | 0,002336 | 38,552 | 46,419 |

| | | | | | |
|---|---|---|---|---|---|
| WFIKKN1 | WAP, follistatin/kazal, immunoglobulin, kunitz and netrin domain containing 1 | **1,177** | 0,223627 | 0,554 | 0,830 |
| RGN | regucalcin | **1,177** | 0,505772 | 0,499 | 0,830 |
| SAA4 | serum amyloid A4, constitutive | **1,177** | 0,424751 | 0,277 | 0,000 |
| SCN4B | sodium voltage-gated channel beta subunit 4 | **1,177** | 0,024923 | 1,496 | 2,171 |
| GDNF | glial cell derived neurotrophic factor | **1,177** | 0,049927 | 1,939 | 5,363 |
| CHCHD7 | coiled-coil-helix-coiled-coil-helix domain containing 7 | **1,177** | 3,00E-06 | 33,123 | 40,353 |
| CROCCP3 | ciliary rootlet coiled-coil, rootletin pseudogene 3 | **1,177** | 0,157748 | 1,329 | 1,341 |
| MEGF10 | multiple EGF like domains 10 | **1,176** | 0,000402 | 14,124 | 18,133 |
| GNG7 | G protein subunit gamma 7 | **1,176** | 0,022662 | 1,717 | 3,831 |
| TNNC1 | troponin C1, slow skeletal and cardiac type | **1,176** | 0,443565 | 0,277 | 0,447 |
| CLVS2 | clavesin 2 | **1,176** | 0,007192 | 3,600 | 2,873 |
| ZNF155 | zinc finger protein 155 | **1,176** | 0,021282 | 3,046 | 3,576 |
| SDAD1P1 | SDA1 domain containing 1 pseudogene 1 | **1,176** | 0,000173 | 10,967 | 6,577 |
| PIK3R3 | phosphoinositide-3-kinase regulatory subunit 3 | **1,176** | 6,00E-06 | 14,678 | 24,710 |
| RSRP1 | arginine and serine rich protein 1 | **1,176** | 0 | 53,119 | 64,361 |
| CBWD6 | COBW domain containing 6 | **1,176** | 0,434831 | 0,665 | 0,511 |
| ZNF354C | zinc finger protein 354C | **1,176** | 0,001007 | 10,247 | 10,280 |
| APCDD1L-AS1 | APCDD1L antisense RNA 1 (head to head) | **1,176** | 0,148503 | 1,496 | 2,107 |
| PLAG1 | PLAG1 zinc finger | **1,176** | 0,00614 | 9,582 | 5,427 |
| LINC01152 | long intergenic non-protein coding RNA 1152 | **1,175** | 0,120814 | 1,052 | 1,532 |
| CHSY3 | chondroitin sulfate synthase 3 | **1,175** | 0,001792 | 13,571 | 21,134 |
| ZNF586 | zinc finger protein 586 | **1,175** | 0,007485 | 3,877 | 3,639 |
| SAXO2 | stabilizer of axonemal microtubules 2 | **1,175** | 0,289058 | 0,332 | 0,702 |
| DUSP18 | dual specificity phosphatase 18 | **1,175** | 0,00096 | 8,807 | 9,450 |
| TMEM64 | transmembrane protein 64 | **1,175** | 0,000314 | 9,638 | 10,599 |
| SIRPA | signal regulatory protein alpha | **1,175** | 0 | 283,155 | 346,194 |
| RAB8B | RAB8B, member RAS oncogene family | **1,175** | 3,80E-05 | 36,779 | 34,351 |
| OVGP1 | oviductal glycoprotein 1 | **1,175** | 0,322946 | 0,443 | 0,192 |
| NPTXR | neuronal pentraxin receptor | **1,175** | 0,003255 | 20,882 | 19,283 |
| SH2B2 | SH2B adaptor protein 2 | **1,175** | 0,074339 | 1,496 | 2,426 |
| MED14 | mediator complex subunit 14 | **1,174** | 0,000151 | 43,094 | 43,482 |
| KLF12 | Kruppel like factor 12 | **1,174** | 0,000339 | 36,391 | 33,904 |
| COCH | cochlin | **1,174** | 0,1047 | 2,493 | 0,766 |

| | | | | | |
|---|---|---|---|---|---|
| PIMREG | PICALM interacting mitotic regulator | **1,174** | 0,073155 | 3,988 | 2,299 |
| SGSM1 | small G protein signaling modulator 1 | **1,174** | 0,037774 | 1,496 | 2,043 |
| PDLIM3 | PDZ and LIM domain 3 | **1,174** | 0,00553 | 2,271 | 4,342 |
| KLF9 | Kruppel like factor 9 | **1,174** | 6,00E-06 | 23,319 | 27,519 |
| DLG2 | discs large MAGUK scaffold protein 2 | **1,174** | 0,087772 | 1,440 | 1,469 |
| MT-CO3 | mitochondrially encoded cytochrome c oxidase III | **1,174** | 1,20E-05 | 597,937 | 441,905 |
| SNX25 | sorting nexin 25 | **1,174** | 3,00E-06 | 27,252 | 23,241 |
| MXI1 | MAX interactor 1, dimerization protein | **1,174** | 2,00E-06 | 30,631 | 34,862 |
| HERC5 | HECT and RLD domain containing E3 ubiquitin protein ligase 5 | **1,174** | 0,003279 | 4,985 | 9,705 |
| TMEM60 | transmembrane protein 60 | **1,174** | 2,10E-05 | 12,241 | 13,664 |
| ZNF385B | zinc finger protein 385B | **1,174** | 0,003228 | 6,093 | 4,469 |
| TRIM36 | tripartite motif containing 36 | **1,174** | 1,10E-05 | 17,393 | 17,942 |
| ZNF93 | zinc finger protein 93 | **1,174** | 0,176035 | 0,942 | 1,085 |
| LRRC58 | leucine rich repeat containing 58 | **1,173** | 0 | 58,437 | 74,896 |
| MYO3A | myosin IIIA | **1,173** | 0,40295 | 0,443 | 0,958 |
| ABI1 | abl interactor 1 | **1,173** | 0 | 90,064 | 97,499 |
| LINC01315 | long intergenic non-protein coding RNA 1315 | **1,173** | 0,437761 | 0,111 | 0,383 |
| PRADC1 | protease associated domain containing 1 | **1,173** | 0,001473 | 5,927 | 8,173 |
| RICTOR | RPTOR independent companion of MTOR complex 2 | **1,173** | 5,70E-05 | 49,685 | 55,294 |
| CD9 | CD9 molecule | **1,173** | 0 | 81,922 | 64,552 |
| PDE2A | phosphodiesterase 2A | **1,173** | 0,108624 | 1,108 | 1,277 |
| C5AR2 | complement component 5a receptor 2 | **1,173** | 0,001131 | 18,057 | 31,797 |
| ZNF302 | zinc finger protein 302 | **1,173** | 0 | 38,773 | 45,972 |
| HS1BP3 | HCLS1 binding protein 3 | **1,173** | 1,00E-06 | 15,066 | 17,942 |
| CTSL | cathepsin L | **1,172** | 0 | 393,879 | 408,895 |
| MROH9 | maestro heat like repeat family member 9 | **1,172** | 0,360962 | 0,443 | 0,575 |
| ZNF333 | zinc finger protein 333 | **1,172** | 4,60E-05 | 14,401 | 19,857 |
| WASHC4 | WASH complex subunit 4 | **1,172** | 8,00E-06 | 71,841 | 68,128 |
| PALB2 | partner and localizer of BRCA2 | **1,172** | 0,000128 | 7,921 | 10,024 |
| MAST4 | microtubule associated serine/threonine kinase family member 4 | **1,172** | 6,00E-06 | 188,105 | 386,292 |
| UBE2H | ubiquitin conjugating enzyme E2 H | **1,172** | 0 | 177,692 | 206,044 |
| HYLS1 | HYLS1, centriolar and ciliogenesis associated | **1,172** | 0,022514 | 3,988 | 3,320 |
| IQGAP1 | IQ motif containing GTPase activating protein 1 | **1,172** | 5,00E-06 | 534,404 | 485,131 |

| | | | | | |
|---|---|---|---|---|---|
| PCDH10 | protocadherin 10 | **1,172** | 0,009715 | 3,213 | 7,023 |
| BAALC-AS1 | BAALC antisense RNA 1 | **1,172** | 0,275071 | 0,720 | 0,894 |
| NINJ1 | ninjurin 1 | **1,172** | 0 | 125,348 | 104,395 |
| UNC5B | unc-5 netrin receptor B | **1,172** | 1,00E-06 | 102,638 | 172,267 |
| CRYGS | crystallin gamma S | **1,171** | 0,156021 | 1,108 | 1,405 |
| STRA6 | stimulated by retinoic acid 6 | **1,171** | 0,165846 | 0,499 | 2,107 |
| FAM161A | family with sequence similarity 161 member A | **1,171** | 0,038195 | 2,160 | 2,618 |
| ANKAR | ankyrin and armadillo repeat containing | **1,171** | 0,026156 | 2,216 | 3,703 |
| SESTD1 | SEC14 and spectrin domain containing 1 | **1,171** | 0 | 50,294 | 59,444 |
| RIMS4 | regulating synaptic membrane exocytosis 4 | **1,171** | 0,349712 | 0,111 | 0,766 |
| CAPRIN2 | caprin family member 2 | **1,171** | 0,00083 | 10,247 | 12,131 |
| ERICH6-AS1 | ERICH6 antisense RNA 1 | **1,171** | 0,339702 | 0,166 | 0,638 |
| OAT | ornithine aminotransferase | **1,171** | 0 | 118,590 | 126,614 |
| CCDC93 | coiled-coil domain containing 93 | **1,170** | 1,30E-05 | 53,064 | 60,785 |
| GNA13 | G protein subunit alpha 13 | **1,170** | 1,00E-06 | 58,991 | 59,827 |
| DUSP22 | dual specificity phosphatase 22 | **1,170** | 0 | 38,330 | 51,271 |
| PAWR | pro-apoptotic WT1 regulator | **1,170** | 0,051029 | 4,376 | 2,043 |
| LRRC38 | leucine rich repeat containing 38 | **1,170** | 0,000221 | 5,428 | 28,669 |
| RTP4 | receptor transporter protein 4 | **1,170** | 0,344589 | 0,443 | 0,830 |
| SPON2 | spondin 2 | **1,170** | 0,097836 | 4,210 | 3,192 |
| EEF1A1P6 | eukaryotic translation elongation factor 1 alpha 1 pseudogene 6 | **1,170** | 0,281994 | 0,499 | 0,447 |
| HIF1A-AS1 | HIF1A antisense RNA 1 | **1,170** | 0,349321 | 0,111 | 0,766 |
| C7orf25 | chromosome 7 open reading frame 25 | **1,170** | 0,448202 | 0,388 | 0,319 |
| INPP4B | inositol polyphosphate-4-phosphatase type II B | **1,169** | 0,012639 | 12,906 | 9,386 |
| GLDC | glycine decarboxylase | **1,169** | 0,264644 | 0,720 | 0,638 |
| SH3BP2 | SH3 domain binding protein 2 | **1,169** | 1,00E-06 | 40,601 | 47,249 |
| GPCPD1 | glycerophosphocholine phosphodiesterase 1 | **1,169** | 1,00E-06 | 21,658 | 27,647 |
| CAV1 | caveolin 1 | **1,169** | 0 | 545,150 | 734,337 |
| CHIC1 | cysteine rich hydrophobic domain 1 | **1,169** | 0,000748 | 10,856 | 12,004 |
| LACTB | lactamase beta | **1,169** | 8,30E-05 | 16,395 | 20,624 |
| CDC42EP4 | CDC42 effector protein 4 | **1,169** | 1,00E-06 | 31,406 | 32,308 |
| KL | klotho | **1,169** | 0,138986 | 0,886 | 2,171 |
| ARL15 | ADP ribosylation factor like GTPase 15 | **1,169** | 0,010767 | 4,154 | 5,491 |

| | | | | | |
|---|---|---|---|---|---|
| MSC | musculin | **1,169** | 1,40E-05 | 53,507 | 51,591 |
| RNF217-AS1 | RNF217 antisense RNA 1 (head to head) | **1,168** | 0,407164 | 0,277 | 0,511 |
| ERMP1 | endoplasmic reticulum metallopeptidase 1 | **1,168** | 0,000557 | 12,740 | 14,302 |
| SLC25A21 | solute carrier family 25 member 21 | **1,168** | 0,079924 | 0,942 | 1,660 |
| LINC01770 | long intergenic non-protein coding RNA 1770 | **1,168** | 0,416779 | 0,166 | 0,511 |
| RUFY3 | RUN and FYVE domain containing 3 | **1,168** | 0,000743 | 15,509 | 13,345 |
| C5orf56 | chromosome 5 open reading frame 56 | **1,168** | 0,001688 | 6,093 | 12,387 |
| THRA | thyroid hormone receptor, alpha | **1,168** | 0 | 25,369 | 26,562 |
| RPS6KA3 | ribosomal protein S6 kinase A3 | **1,168** | 1,00E-06 | 115,599 | 88,304 |
| NR1D2 | nuclear receptor subfamily 1 group D member 2 | **1,168** | 0 | 137,146 | 133,574 |
| PPDPF | pancreatic progenitor cell differentiation and proliferation factor | **1,168** | 1,00E-06 | 153,652 | 246,780 |
| DDX60L | DEAD-box helicase 60 like | **1,168** | 0,015545 | 3,767 | 4,278 |
| ATP1A1-AS1 | ATP1A1 antisense RNA 1 | **1,167** | 0,149561 | 1,163 | 1,724 |
| ASB16-AS1 | ASB16 antisense RNA 1 | **1,167** | 0,000498 | 6,647 | 8,428 |
| MGAT4A | mannosyl (alpha-1,3-)-glycoprotein beta-1,4-N-acetylglucosaminyltransferase, isozyme A | **1,167** | 0,001972 | 15,565 | 14,941 |
| MED13L | mediator complex subunit 13 like | **1,167** | 0,00053 | 66,247 | 96,030 |
| ZNF710-AS1 | ZNF710 antisense RNA 1 | **1,167** | 0,230892 | 0,775 | 0,766 |
| TEP1 | telomerase associated protein 1 | **1,167** | 4,00E-06 | 35,394 | 42,971 |
| IRF1 | interferon regulatory factor 1 | **1,167** | 0 | 81,313 | 104,395 |
| CLCN4 | chloride voltage-gated channel 4 | **1,167** | 0,000163 | 18,500 | 17,048 |
| ZFYVE1 | zinc finger FYVE-type containing 1 | **1,167** | 4,00E-06 | 24,427 | 28,285 |
| HMGB2 | high mobility group box 2 | **1,167** | 5,00E-06 | 66,413 | 70,107 |
| NOTCH1 | notch 1 | **1,167** | 3,10E-05 | 33,788 | 48,334 |
| NEK7 | NIMA related kinase 7 | **1,167** | 0 | 63,920 | 65,893 |
| DUSP6 | dual specificity phosphatase 6 | **1,167** | 0 | 380,309 | 503,903 |
| RPS23P8 | ribosomal protein S23 pseudogene 8 | **1,167** | 0,430127 | 0,831 | 0,511 |
| CCL25 | C-C motif chemokine ligand 25 | **1,167** | 0,50923 | 0,055 | 0,383 |
| SFRP1 | secreted frizzled related protein 1 | **1,166** | 0,006482 | 3,323 | 3,320 |
| ABCA10 | ATP binding cassette subfamily A member 10 | **1,166** | 0,144816 | 0,942 | 1,532 |
| ZNF607 | zinc finger protein 607 | **1,166** | 0,176896 | 1,551 | 2,490 |
| AGAP3 | ArfGAP with GTPase domain, ankyrin repeat and PH domain 3 | **1,166** | 1,60E-05 | 24,759 | 27,072 |
| FAM83G | family with sequence similarity 83 member G | **1,166** | 1,00E-06 | 46,195 | 58,870 |
| RASL11A | RAS like family 11 member A | **1,166** | 0,014024 | 4,099 | 4,597 |

| | | | | | |
|---|---|---|---|---|---|
| ZNF649 | zinc finger protein 649 | **1,166** | 0,002421 | 7,256 | 8,620 |
| CBFA2T2 | CBFA2/RUNX1 translocation partner 2 | **1,166** | 0,000167 | 23,153 | 27,583 |
| STAT5A | signal transducer and activator of transcription 5A | **1,166** | 1,00E-06 | 34,508 | 47,121 |
| PPP1R13B | protein phosphatase 1 regulatory subunit 13B | **1,166** | 5,00E-06 | 17,226 | 28,158 |
| EMP1 | epithelial membrane protein 1 | **1,166** | 3,00E-06 | 337,437 | 590,420 |
| FAM19A2 | family with sequence similarity 19 member A2, C-C motif chemokine like | **1,166** | 0,108916 | 1,108 | 1,532 |
| FBXL7 | F-box and leucine rich repeat protein 7 | **1,166** | 0,000226 | 27,750 | 36,394 |
| BEND6 | BEN domain containing 6 | **1,165** | 0,00369 | 5,373 | 4,342 |
| PDGFRL | platelet derived growth factor receptor like | **1,165** | 8,70E-05 | 21,270 | 24,838 |
| ALS2CR12 | amyotrophic lateral sclerosis 2 chromosome region 12 | **1,165** | 0,409673 | 0,332 | 0,511 |
| ANXA2P2 | annexin A2 pseudogene 2 | **1,165** | 0,07155 | 2,493 | 2,107 |
| HIST1H4H | histone cluster 1 H4 family member h | **1,165** | 3,90E-05 | 10,303 | 14,813 |
| NDST2 | N-deacetylase and N-sulfotransferase 2 | **1,165** | 0,399952 | 0,332 | 0,830 |
| ST5 | suppression of tumorigenicity 5 | **1,165** | 0 | 22,156 | 34,543 |
| FOXC2 | forkhead box C2 | **1,165** | 0 | 42,983 | 56,507 |
| FAM217B | family with sequence similarity 217 member B | **1,165** | 0,001391 | 16,340 | 14,622 |
| ADAMTS4 | ADAM metallopeptidase with thrombospondin type 1 motif 4 | **1,165** | 0,089227 | 2,493 | 4,278 |
| CLGN | calmegin | **1,165** | 0,001487 | 7,035 | 7,662 |
| EN2 | engrailed homeobox 2 | **1,165** | 0,294611 | 0,554 | 0,766 |
| PAQR4 | progestin and adipoQ receptor family member 4 | **1,165** | 0,020074 | 6,979 | 3,639 |
| TWSG1 | twisted gastrulation BMP signaling modulator 1 | **1,164** | 0 | 291,186 | 186,186 |
| SKI | SKI proto-oncogene | **1,164** | 1,00E-06 | 141,799 | 165,243 |
| MAP3K6 | mitogen-activated protein kinase kinase kinase 6 | **1,164** | 0,010038 | 7,533 | 5,236 |
| TBXA2R | thromboxane A2 receptor | **1,164** | 0,217091 | 1,052 | 0,702 |
| FRG1BP | FSHD region gene 1 family member B, pseudogene | **1,164** | 0,115258 | 1,440 | 0,958 |
| PDPN | podoplanin | **1,164** | 0 | 626,518 | 459,208 |
| CBR3 | carbonyl reductase 3 | **1,164** | 0,000127 | 31,628 | 46,930 |
| NUPR1 | nuclear protein 1, transcriptional regulator | **1,164** | 0,000453 | 171,156 | 175,140 |
| PCMTD1 | protein-L-isoaspartate (D-aspartate) O-methyltransferase domain containing 1 | **1,164** | 8,00E-06 | 34,065 | 26,817 |
| VAT1 | vesicle amine transport 1 | **1,164** | 1,00E-06 | 101,807 | 80,962 |
| PKD1L1 | polycystin 1 like 1, transient receptor potential channel interacting | **1,164** | 0,18671 | 0,554 | 1,277 |
| PRRG4 | proline rich and Gla domain 4 | **1,164** | 0,221221 | 1,163 | 0,638 |
| GHR | growth hormone receptor | **1,164** | 0,004399 | 10,303 | 7,917 |

| | | | | | |
|---|---|---|---|---|---|
| SATB2 | SATB homeobox 2 | **1,164** | 0,034644 | 3,600 | 2,873 |
| ATL1 | atlastin GTPase 1 | **1,163** | 0,010392 | 7,312 | 7,726 |
| FNBP1P1 | formin binding protein 1 pseudogene 1 | **1,163** | 0,126404 | 1,219 | 1,660 |
| ADGRA2 | adhesion G protein-coupled receptor A2 | **1,163** | 0,123595 | 1,385 | 1,149 |
| HNRNPCP1 | heterogeneous nuclear ribonucleoprotein C pseudogene 1 | **1,163** | 0,428229 | 0,332 | 0,702 |
| FAM124A | family with sequence similarity 124 member A | **1,163** | 0,084468 | 2,160 | 1,724 |
| EZH2 | enhancer of zeste 2 polycomb repressive complex 2 subunit | **1,163** | 0,013146 | 5,871 | 5,619 |
| SMG1 | SMG1, nonsense mediated mRNA decay associated PI3K related kinase | **1,163** | 0,001248 | 109,119 | 125,210 |
| DNAH11 | dynein axonemal heavy chain 11 | **1,163** | 0,018029 | 4,874 | 12,706 |
| ZNF699 | zinc finger protein 699 | **1,162** | 0,0365 | 3,656 | 4,278 |
| OSTM1 | osteopetrosis associated transmembrane protein 1 | **1,162** | 1,00E-06 | 78,322 | 96,605 |
| CYSTM1 | cysteine rich transmembrane module containing 1 | **1,162** | 4,00E-06 | 41,432 | 35,820 |
| PLEKHO2 | pleckstrin homology domain containing O2 | **1,162** | 0 | 59,655 | 63,914 |
| ZNF597 | zinc finger protein 597 | **1,162** | 0,000261 | 6,758 | 9,769 |
| C2orf72 | chromosome 2 open reading frame 72 | **1,162** | 0,051171 | 2,770 | 2,746 |
| ARVCF | armadillo repeat gene deleted in velocardiofacial syndrome | **1,162** | 0,036045 | 3,767 | 3,703 |
| ARHGAP10 | Rho GTPase activating protein 10 | **1,162** | 0,000253 | 16,617 | 17,750 |
| FURIN | furin, paired basic amino acid cleaving enzyme | **1,162** | 3,50E-05 | 140,691 | 131,786 |
| SPAG6 | sperm associated antigen 6 | **1,162** | 0,091356 | 1,440 | 2,618 |
| STK17B | serine/threonine kinase 17b | **1,162** | 0,008465 | 7,035 | 3,639 |
| ZFC3H1 | zinc finger C3H1-type containing | **1,162** | 0,00173 | 27,030 | 32,372 |
| SCARNA13 | small Cajal body-specific RNA 13 | **1,161** | 0,337218 | 0,332 | 0,638 |
| GLMP | glycosylated lysosomal membrane protein | **1,161** | 1,60E-05 | 25,479 | 23,178 |
| RECK | reversion inducing cysteine rich protein with kazal motifs | **1,161** | 0 | 27,695 | 28,669 |
| SSPN | sarcospan | **1,161** | 0,000928 | 10,746 | 11,110 |
| NBPF14 | NBPF member 14 | **1,161** | 0,001342 | 10,690 | 15,643 |
| KLF11 | Kruppel like factor 11 | **1,161** | 0,000229 | 13,460 | 9,641 |
| PCDH1 | protocadherin 1 | **1,161** | 4,60E-05 | 15,454 | 28,349 |
| ZCWPW2 | zinc finger CW-type and PWWP domain containing 2 | **1,161** | 0,401352 | 0,388 | 0,575 |
| SERINC5 | serine incorporator 5 | **1,161** | 0,000576 | 33,289 | 36,267 |
| ZNF274 | zinc finger protein 274 | **1,161** | 7,40E-05 | 12,961 | 15,962 |
| CHD2 | chromodomain helicase DNA binding protein 2 | **1,161** | 4,00E-06 | 96,268 | 83,579 |
| MANBA | mannosidase beta | **1,161** | 3,00E-06 | 44,700 | 36,394 |

| | | | | | |
|---|---|---|---|---|---|
| ZNF367 | zinc finger protein 367 | **1,161** | 0,00286 | 12,574 | 6,577 |
| FTH1 | ferritin heavy chain 1 | **1,161** | 1,50E-05 | 9195,321 | 6225,427 |
| LPCAT2 | lysophosphatidylcholine acyltransferase 2 | **1,161** | 0,050781 | 0,997 | 2,426 |
| HMGN5 | high mobility group nucleosome binding domain 5 | **1,161** | 0,052766 | 6,259 | 8,173 |
| FERMT2 | fermitin family member 2 | **1,161** | 1,70E-05 | 130,499 | 144,939 |
| TXNDC16 | thioredoxin domain containing 16 | **1,161** | 0,000983 | 13,460 | 11,301 |
| KIAA1211 | KIAA1211 | **1,160** | 0,057034 | 3,102 | 4,597 |
| ACVR2B-AS1 | ACVR2B antisense RNA 1 | **1,160** | 0,418466 | 0,332 | 0,447 |
| CCDC154 | coiled-coil domain containing 154 | **1,160** | 0,121492 | 0,886 | 1,277 |
| GBP4 | guanylate binding protein 4 | **1,160** | 0,002118 | 7,644 | 13,536 |
| ZNF160 | zinc finger protein 160 | **1,160** | 8,00E-06 | 27,030 | 30,265 |
| TNS1 | tensin 1 | **1,160** | 0,000367 | 159,911 | 150,303 |
| AKAP13 | A-kinase anchoring protein 13 | **1,160** | 0,000428 | 62,702 | 81,536 |
| PEX3 | peroxisomal biogenesis factor 3 | **1,160** | 0,00183 | 6,481 | 10,088 |
| SWAP70 | SWAP switching B-cell complex subunit 70 | **1,160** | 0 | 118,036 | 100,053 |
| ARL8B | ADP ribosylation factor like GTPase 8B | **1,160** | 0 | 96,268 | 116,654 |
| MFSD2A | major facilitator superfamily domain containing 2A | **1,160** | 3,00E-06 | 17,891 | 25,859 |
| SIGLEC15 | sialic acid binding Ig like lectin 15 | **1,160** | 0,091039 | 1,828 | 1,979 |
| MAPK8IP3 | mitogen-activated protein kinase 8 interacting protein 3 | **1,160** | 2,00E-05 | 50,128 | 63,722 |
| TNRC6C | trinucleotide repeat containing 6C | **1,159** | 2,20E-05 | 27,529 | 39,587 |
| NCS1 | neuronal calcium sensor 1 | **1,159** | 0,00065 | 11,133 | 9,258 |
| ACAP2 | ArfGAP with coiled-coil, ankyrin repeat and PH domains 2 | **1,159** | 1,00E-06 | 60,431 | 66,404 |
| WDR78 | WD repeat domain 78 | **1,159** | 0,003238 | 5,705 | 7,598 |
| FTH1P8 | ferritin heavy chain 1 pseudogene 8 | **1,159** | 0,002344 | 7,644 | 4,469 |
| BHLHE41 | basic helix-loop-helix family member e41 | **1,159** | 4,00E-06 | 84,858 | 82,813 |
| HSPBAP1 | HSPB1 associated protein 1 | **1,159** | 0,003835 | 11,133 | 11,174 |
| SEMA6C | semaphorin 6C | **1,159** | 0,000285 | 9,250 | 12,004 |
| MARCKSL1 | MARCKS like 1 | **1,159** | 0,009231 | 9,970 | 7,854 |
| LAMA4 | laminin subunit alpha 4 | **1,159** | 2,40E-05 | 54,061 | 63,978 |
| HMGN4 | high mobility group nucleosomal binding domain 4 | **1,159** | 0,000151 | 20,882 | 21,645 |
| ZNF470 | zinc finger protein 470 | **1,159** | 0,009397 | 7,588 | 7,151 |
| ZNF550 | zinc finger protein 550 | **1,159** | 0,027114 | 4,210 | 5,874 |
| DNAJC27 | DnaJ heat shock protein family (Hsp40) member C27 | **1,159** | 0,004736 | 4,708 | 6,321 |

| | | | | | |
|---|---|---|---|---|---|
| TOLLIP | toll interacting protein | **1,159** | 1,00E-06 | 75,885 | 83,771 |
| RAD51B | RAD51 paralog B | **1,159** | 0,010515 | 2,825 | 4,214 |
| ZFYVE26 | zinc finger FYVE-type containing 26 | **1,159** | 1,40E-05 | 31,517 | 42,396 |
| SLC9A5 | solute carrier family 9 member A5 | **1,159** | 0,184009 | 1,163 | 1,979 |
| ZNF793-AS1 | ZNF793 antisense RNA 1 (head to head) | **1,158** | 0,463341 | 0,720 | 0,255 |
| SLFN5 | schlafen family member 5 | **1,158** | 2,00E-06 | 113,494 | 135,234 |
| FGFR4 | fibroblast growth factor receptor 4 | **1,158** | 0,257152 | 0,388 | 0,958 |
| FTH1P11 | ferritin heavy chain 1 pseudogene 11 | **1,158** | 0,400915 | 0,277 | 0,702 |
| CD74 | CD74 molecule | **1,158** | 0,013039 | 1,329 | 4,980 |
| ZBTB1 | zinc finger and BTB domain containing 1 | **1,158** | 9,00E-06 | 47,248 | 51,144 |
| HIST1H1E | histone cluster 1 H1 family member e | **1,158** | 0,286272 | 1,274 | 2,746 |
| SLC25A29 | solute carrier family 25 member 29 | **1,158** | 0,000468 | 10,303 | 9,003 |
| DMRT2 | doublesex and mab-3 related transcription factor 2 | **1,158** | 0,518657 | 0,332 | 0,894 |
| LINC00641 | long intergenic non-protein coding RNA 641 | **1,158** | 0,005102 | 8,475 | 11,429 |
| ZNF273 | zinc finger protein 273 | **1,157** | 0,124254 | 1,274 | 1,596 |
| EMC3-AS1 | EMC3 antisense RNA 1 | **1,157** | 0,155996 | 1,662 | 2,043 |
| IGF2R | insulin like growth factor 2 receptor | **1,157** | 6,00E-06 | 364,190 | 415,982 |
| HACD1 | 3-hydroxyacyl-CoA dehydratase 1 | **1,157** | 0,060244 | 2,659 | 1,405 |
| MIR155HG | MIR155 host gene | **1,157** | 0,124043 | 2,825 | 3,320 |
| SEMA3E | semaphorin 3E | **1,157** | 5,00E-06 | 24,206 | 18,963 |
| CDHR1 | cadherin related family member 1 | **1,157** | 0,313393 | 1,994 | 0,575 |
| CSRNP1 | cysteine and serine rich nuclear protein 1 | **1,157** | 1,00E-05 | 53,507 | 87,219 |
| FILIP1 | filamin A interacting protein 1 | **1,157** | 0,284229 | 0,720 | 1,149 |
| MIPOL1 | mirror-image polydactyly 1 | **1,157** | 0,008982 | 8,198 | 11,429 |
| RAD9A | RAD9 checkpoint clamp component A | **1,157** | 0,019494 | 5,484 | 4,980 |
| MT-CO2 | mitochondrially encoded cytochrome c oxidase II | **1,157** | 0,000246 | 1006,938 | 799,337 |
| UBL3 | ubiquitin like 3 | **1,157** | 2,00E-06 | 95,160 | 102,607 |
| RNF24 | ring finger protein 24 | **1,157** | 2,10E-05 | 107,069 | 127,764 |
| LINC00475 | long intergenic non-protein coding RNA 475 | **1,157** | 0,4111 | 0,499 | 0,128 |
| PICART1 | p53-inducible cancer-associated RNA transcript 1 | **1,156** | 0,403362 | 0,222 | 0,383 |
| DCLK3 | doublecortin like kinase 3 | **1,156** | 0,22795 | 1,662 | 1,085 |
| DCK | deoxycytidine kinase | **1,156** | 0,000138 | 10,690 | 14,239 |
| FRMD8 | FERM domain containing 8 | **1,156** | 0 | 39,493 | 48,845 |

| | | | | | |
|---|---|---|---|---|---|
| RAB17 | RAB17, member RAS oncogene family | **1,156** | 0,23005 | 0,554 | 1,022 |
| HAS2 | hyaluronan synthase 2 | **1,156** | 0,011045 | 36,724 | 27,200 |
| S1PR3 | sphingosine-1-phosphate receptor 3 | **1,156** | 0,002674 | 12,020 | 13,345 |
| TRPM7 | transient receptor potential cation channel subfamily M member 7 | **1,156** | 0,000185 | 90,784 | 90,795 |
| CACNA2D1 | calcium voltage-gated channel auxiliary subunit alpha2delta 1 | **1,156** | 0,000512 | 19,664 | 12,323 |
| KDM4A-AS1 | KDM4A antisense RNA 1 | **1,156** | 0,24503 | 0,720 | 0,894 |
| RNF111 | ring finger protein 111 | **1,156** | 6,50E-05 | 34,840 | 40,672 |
| KAT6A | lysine acetyltransferase 6A | **1,156** | 0,003167 | 47,414 | 70,363 |
| CERCAM | cerebral endothelial cell adhesion molecule | **1,156** | 9,60E-05 | 33,068 | 21,901 |
| PKNOX1 | PBX/knotted 1 homeobox 1 | **1,155** | 1,60E-05 | 16,783 | 17,814 |
| SERINC4 | serine incorporator 4 | **1,155** | 0,373061 | 0,665 | 0,958 |
| TTC33 | tetratricopeptide repeat domain 33 | **1,155** | 9,90E-05 | 12,961 | 15,388 |
| PPAT | phosphoribosyl pyrophosphate amidotransferase | **1,155** | 0,009517 | 9,306 | 12,323 |
| AFF4 | AF4/FMR2 family member 4 | **1,155** | 0,002497 | 155,148 | 169,713 |
| GARNL3 | GTPase activating Rap/RanGAP domain like 3 | **1,155** | 0,364126 | 0,942 | 0,958 |
| ZBTB33 | zinc finger and BTB domain containing 33 | **1,155** | 8,70E-05 | 18,500 | 21,134 |
| KLF15 | Kruppel like factor 15 | **1,155** | 0,007559 | 6,370 | 8,747 |
| LRRIQ1 | leucine rich repeats and IQ motif containing 1 | **1,155** | 0,371444 | 0,997 | 0,638 |
| LINC02367 | long intergenic non-protein coding RNA 2367 | **1,155** | 0,380855 | 0,166 | 0,575 |
| SGK3 | serum/glucocorticoid regulated kinase family member 3 | **1,154** | 0,000295 | 16,008 | 21,709 |
| KIF1B | kinesin family member 1B | **1,154** | 1,00E-05 | 45,531 | 49,867 |
| MYLK-AS1 | MYLK antisense RNA 1 | **1,154** | 0,393584 | 0,443 | 0,447 |
| ZNF385A | zinc finger protein 385A | **1,154** | 0 | 102,860 | 131,722 |
| PTGES3P1 | prostaglandin E synthase 3 pseudogene 1 | **1,154** | 0,022111 | 4,542 | 4,597 |
| NEFL | neurofilament light | **1,154** | 0,485403 | 0,166 | 0,511 |
| SRPK2 | SRSF protein kinase 2 | **1,154** | 2,00E-06 | 32,736 | 38,501 |
| SEC14L2 | SEC14 like lipid binding 2 | **1,154** | 0,004378 | 9,527 | 9,897 |
| AMY2B | amylase, alpha 2B (pancreatic) | **1,154** | 0,074991 | 1,219 | 2,426 |
| SLC5A3 | solute carrier family 5 member 3 | **1,154** | 7,40E-05 | 498,733 | 584,801 |
| PERP | PERP, TP53 apoptosis effector | **1,154** | 5,70E-05 | 37,056 | 44,567 |
| RNF38 | ring finger protein 38 | **1,154** | 3,90E-05 | 24,538 | 23,178 |
| ZNF224 | zinc finger protein 224 | **1,154** | 0,000826 | 9,970 | 11,748 |
| MYO1E | myosin IE | **1,154** | 1,30E-05 | 50,460 | 85,048 |

| | | | | | |
|---|---|---|---|---|---|
| C10orf107 | chromosome 10 open reading frame 107 | **1,154** | 0,476149 | 0,554 | 1,469 |
| CD109 | CD109 molecule | **1,154** | 5,00E-06 | 191,705 | 156,751 |
| TYW5 | tRNA-yW synthesizing protein 5 | **1,153** | 0,002844 | 6,979 | 7,343 |
| HSF2 | heat shock transcription factor 2 | **1,153** | 5,00E-05 | 17,503 | 20,687 |
| ATP6V1C1 | ATPase H+ transporting V1 subunit C1 | **1,153** | 6,00E-06 | 54,282 | 61,807 |
| MCF2L2 | MCF.2 cell line derived transforming sequence-like 2 | **1,153** | 0,218316 | 0,942 | 1,341 |
| PABPC5 | poly(A) binding protein cytoplasmic 5 | **1,153** | 0,06287 | 4,265 | 4,086 |
| TBX3 | T-box 3 | **1,153** | 0,01429 | 7,810 | 10,216 |
| ZMAT1 | zinc finger matrin-type 1 | **1,153** | 0,017565 | 3,711 | 9,514 |
| CORO2B | coronin 2B | **1,153** | 0,000545 | 9,527 | 11,621 |
| CPNE8 | copine 8 | **1,153** | 0,000385 | 24,095 | 23,050 |
| NCAM2 | neural cell adhesion molecule 2 | **1,153** | 0,074468 | 2,548 | 1,596 |
| MT-TE | mitochondrially encoded tRNA glutamic acid | **1,153** | 0,448934 | 0,499 | 0,383 |
| PEG13 | paternally expressed 13 | **1,153** | 0,451249 | 0,388 | 0,511 |
| PARD6G | par-6 family cell polarity regulator gamma | **1,153** | 0,213204 | 0,886 | 0,894 |
| KANTR | KDM5C adjacent transcript | **1,153** | 0,069995 | 3,490 | 2,426 |
| FAM26F | family with sequence similarity 26 member F | **1,152** | 0,326297 | 0,775 | 0,958 |
| INPP5J | inositol polyphosphate-5-phosphatase J | **1,152** | 0,169794 | 0,609 | 1,788 |
| SLFN12 | schlafen family member 12 | **1,152** | 0,305187 | 1,163 | 1,277 |
| SEC24B-AS1 | SEC24B antisense RNA 1 | **1,152** | 0,454381 | 0,332 | 0,638 |
| TP53BP2 | tumor protein p53 binding protein 2 | **1,152** | 0 | 56,886 | 58,040 |
| BTBD3 | BTB domain containing 3 | **1,152** | 3,00E-05 | 15,565 | 16,346 |
| KPNA2 | karyopherin subunit alpha 2 | **1,152** | 0,000567 | 26,532 | 21,326 |
| MTND2P28 | mitochondrially encoded NADH:ubiquinone oxidoreductase core subunit 2 pseudogene 28 | **1,152** | 0,176 | 22,987 | 33,777 |
| ATP11C | ATPase phospholipid transporting 11C | **1,152** | 8,00E-06 | 21,104 | 18,516 |
| GPRASP1 | G protein-coupled receptor associated sorting protein 1 | **1,152** | 0,000398 | 14,845 | 20,113 |
| MSI2 | musashi RNA binding protein 2 | **1,152** | 3,80E-05 | 85,633 | 80,004 |
| CENPN | centromere protein N | **1,152** | 0,010142 | 9,859 | 10,791 |
| LARGE1 | LARGE xylosyl- and glucuronyltransferase 1 | **1,152** | 0,001117 | 11,687 | 14,175 |
| GALNT4 | polypeptide N-acetylgalactosaminyltransferase 4 | **1,152** | 0,04457 | 2,216 | 3,256 |
| ERO1B | endoplasmic reticulum oxidoreductase 1 beta | **1,152** | 2,80E-05 | 22,710 | 24,071 |
| CHTF18 | chromosome transmission fidelity factor 18 | **1,152** | 0,006399 | 7,035 | 11,812 |
| SH3BGR | SH3 domain binding glutamate rich protein | **1,152** | 0,068828 | 2,271 | 3,703 |

| | | | | | |
|---|---|---|---|---|---|
| ANKRD49 | ankyrin repeat domain 49 | **1,152** | 0,001148 | 7,256 | 7,279 |
| PGF | placental growth factor | **1,152** | 0,069948 | 3,933 | 3,320 |
| ST20-AS1 | ST20 antisense RNA 1 | **1,152** | 0,019243 | 3,545 | 4,023 |
| PARM1 | prostate androgen-regulated mucin-like protein 1 | **1,152** | 2,00E-06 | 108,509 | 110,205 |
| FAM219A | family with sequence similarity 219 member A | **1,151** | 1,00E-06 | 39,161 | 44,248 |
| ZNF678 | zinc finger protein 678 | **1,151** | 0,005056 | 5,761 | 5,619 |
| TEX2 | testis expressed 2 | **1,151** | 0,000806 | 37,610 | 54,783 |
| FBXO31 | F-box protein 31 | **1,151** | 1,30E-05 | 19,996 | 28,605 |
| MMP12 | matrix metallopeptidase 12 | **1,151** | 0,065243 | 1,828 | 2,873 |
| ARHGAP21 | Rho GTPase activating protein 21 | **1,151** | 0,000291 | 74,223 | 70,746 |
| LRRC3 | leucine rich repeat containing 3 | **1,151** | 0,461265 | 1,385 | 0,319 |
| KCTD7 | potassium channel tetramerization domain containing 7 | **1,151** | 0,000169 | 16,285 | 21,326 |
| SLC7A2 | solute carrier family 7 member 2 | **1,151** | 0,000193 | 9241,018 | 8870,600 |
| FMR1 | fragile X mental retardation 1 | **1,151** | 1,00E-04 | 27,861 | 31,350 |
| MBOAT1 | membrane bound O-acyltransferase domain containing 1 | **1,151** | 0,017373 | 5,151 | 7,023 |
| RPL24P4 | ribosomal protein L24 pseudogene 4 | **1,151** | 0,640618 | 0,332 | 0,447 |
| ABCA9 | ATP binding cassette subfamily A member 9 | **1,151** | 0,177338 | 0,775 | 1,724 |
| ID4 | inhibitor of DNA binding 4, HLH protein | **1,151** | 0,000225 | 28,803 | 21,964 |
| PLEKHF1 | pleckstrin homology and FYVE domain containing 1 | **1,151** | 0,00162 | 11,743 | 12,387 |
| MT-ND4 | mitochondrially encoded NADH:ubiquinone oxidoreductase core subunit 4 | **1,150** | 0,000639 | 2218,542 | 2599,009 |
| ZNF300P1 | zinc finger protein 300 pseudogene 1 | **1,150** | 0,009207 | 4,597 | 9,769 |
| CASP8AP2 | caspase 8 associated protein 2 | **1,150** | 0,002295 | 15,011 | 19,793 |
| PARGP1 | poly(ADP-ribose) glycohydrolase pseudogene 1 | **1,150** | 0,294945 | 0,665 | 1,277 |
| TCEAL9 | transcription elongation factor A like 9 | **1,150** | 0,00013 | 41,155 | 32,819 |
| ALPK3 | alpha kinase 3 | **1,150** | 0,005248 | 7,588 | 11,429 |
| ASB3 | ankyrin repeat and SOCS box containing 3 | **1,150** | 0,318783 | 0,665 | 0,958 |
| LINC02246 | long intergenic non-protein coding RNA 2246 | **1,150** | 0,541071 | 0,055 | 0,447 |
| BRI3BP | BRI3 binding protein | **1,150** | 0,000217 | 17,226 | 22,794 |
| OTUD7A | OTU deubiquitinase 7A | **1,150** | 0,416157 | 0,166 | 0,511 |
| ALKBH1 | alkB homolog 1, histone H2A dioxygenase | **1,150** | 0,000349 | 9,306 | 10,408 |
| RAB21 | RAB21, member RAS oncogene family | **1,150** | 2,00E-06 | 106,017 | 128,147 |
| POLE | DNA polymerase epsilon, catalytic subunit | **1,150** | 0,000867 | 11,909 | 16,856 |
| ABCA3 | ATP binding cassette subfamily A member 3 | **1,150** | 0,001755 | 11,632 | 21,134 |

| | | | | | |
|---|---|---|---|---|---|
| MET | MET proto-oncogene, receptor tyrosine kinase | **1,149** | 5,70E-05 | 294,509 | 343,640 |
| ACVR1 | activin A receptor type 1 | **1,149** | 0 | 222,004 | 242,821 |
| TESK2 | testis-specific kinase 2 | **1,149** | 0,000851 | 11,853 | 13,536 |
| ZNF330 | zinc finger protein 330 | **1,149** | 4,00E-06 | 18,666 | 23,433 |
| TPT1-AS1 | TPT1 antisense RNA 1 | **1,149** | 0,007297 | 6,979 | 9,258 |
| SIK2 | salt inducible kinase 2 | **1,149** | 3,70E-05 | 63,200 | 82,111 |
| ZNF200 | zinc finger protein 200 | **1,149** | 0,011877 | 6,314 | 5,491 |
| SNX16 | sorting nexin 16 | **1,148** | 0,001803 | 9,749 | 11,812 |
| UGCG | UDP-glucose ceramide glucosyltransferase | **1,148** | 9,00E-06 | 116,818 | 121,506 |
| SNRK | SNF related kinase | **1,148** | 1,80E-05 | 23,652 | 26,242 |
| ZNF721 | zinc finger protein 721 | **1,148** | 2,40E-05 | 12,075 | 18,197 |
| DDX58 | DExD/H-box helicase 58 | **1,148** | 2,00E-05 | 18,666 | 31,733 |
| ZNF490 | zinc finger protein 490 | **1,148** | 0,315541 | 1,274 | 0,958 |
| VPS26A | VPS26, retromer complex component A | **1,148** | 0 | 65,693 | 72,342 |
| LRRN1 | leucine rich repeat neuronal 1 | **1,148** | 0,07194 | 3,767 | 2,299 |
| ZNF229 | zinc finger protein 229 | **1,148** | 0,046075 | 3,600 | 3,703 |
| ZBTB34 | zinc finger and BTB domain containing 34 | **1,148** | 0,002388 | 10,967 | 10,280 |
| CDKN1A | cyclin dependent kinase inhibitor 1A | **1,148** | 2,10E-05 | 367,846 | 434,882 |
| SLC15A4 | solute carrier family 15 member 4 | **1,148** | 7,00E-06 | 78,876 | 48,654 |
| RNF180 | ring finger protein 180 | **1,148** | 0,130216 | 2,105 | 1,852 |
| ZNF782 | zinc finger protein 782 | **1,148** | 0,014121 | 5,871 | 7,790 |
| CDCA2 | cell division cycle associated 2 | **1,148** | 0,049207 | 4,043 | 3,256 |
| KCNJ2 | potassium voltage-gated channel subfamily J member 2 | **1,148** | 0,004001 | 2,991 | 6,002 |
| SIRPB1 | signal regulatory protein beta 1 | **1,148** | 0,176341 | 4,320 | 2,426 |
| AFG1L | AFG1 like ATPase | **1,148** | 0,035375 | 2,326 | 2,746 |
| LAMP2 | lysosomal associated membrane protein 2 | **1,148** | 1,00E-06 | 137,922 | 109,439 |
| ICK | intestinal cell kinase | **1,148** | 0,000493 | 13,183 | 15,005 |
| DCXR | dicarbonyl and L-xylulose reductase | **1,148** | 0,006695 | 42,429 | 41,630 |
| ACAD11 | acyl-CoA dehydrogenase family member 11 | **1,147** | 0,207073 | 1,052 | 2,107 |
| AKIRIN2 | akirin 2 | **1,147** | 0 | 51,734 | 66,276 |
| DISP2 | dispatched RND transporter family member 2 | **1,147** | 0,099841 | 1,717 | 3,192 |
| SFXN5 | sideroflexin 5 | **1,147** | 0,000107 | 12,186 | 16,090 |
| BAMBI | BMP and activin membrane bound inhibitor | **1,147** | 0,000118 | 44,921 | 47,313 |

| | | | | | |
|---|---|---|---|---|---|
| NFIX | nuclear factor I X | **1,147** | 0 | 209,984 | 258,273 |
| GALNT12 | polypeptide N-acetylgalactosaminyltransferase 12 | **1,147** | 0,000115 | 16,451 | 28,988 |
| WDFY1 | WD repeat and FYVE domain containing 1 | **1,147** | 1,30E-05 | 47,580 | 46,036 |
| FBXO48 | F-box protein 48 | **1,147** | 0,061397 | 3,490 | 3,576 |
| ZNF530 | zinc finger protein 530 | **1,147** | 0,163103 | 1,606 | 1,022 |
| RTL5 | retrotransposon Gag like 5 | **1,147** | 0,002456 | 8,696 | 9,833 |
| ARL5B | ADP ribosylation factor like GTPase 5B | **1,147** | 0,000161 | 31,185 | 33,585 |
| DDO | D-aspartate oxidase | **1,147** | 0,384003 | 0,831 | 0,447 |
| SEL1L3 | SEL1L family member 3 | **1,147** | 0,218551 | 0,388 | 1,149 |
| SRD5A3-AS1 | SRD5A3 antisense RNA 1 | **1,147** | 0,454322 | 0,609 | 0,383 |
| N4BP2L2-IT2 | N4BPL2 intronic transcript 2 | **1,147** | 0,393225 | 0,665 | 0,702 |
| RRN3P1 | RRN3 homolog, RNA polymerase I transcription factor pseudogene 1 | **1,147** | 0,271231 | 0,609 | 0,511 |
| ZFAND6 | zinc finger AN1-type containing 6 | **1,147** | 1,00E-06 | 60,708 | 76,492 |
| DOCK11 | dedicator of cytokinesis 11 | **1,146** | 0,002614 | 8,032 | 11,748 |
| LYG1 | lysozyme g1 | **1,146** | 0,366132 | 0,499 | 0,383 |
| ZNF445 | zinc finger protein 445 | **1,146** | 0,005815 | 14,401 | 20,177 |
| MITD1 | microtubule interacting and trafficking domain containing 1 | **1,146** | 0,000294 | 11,355 | 18,900 |
| CETN4P | centrin 4, pseudogene | **1,146** | 0,172133 | 0,665 | 1,596 |
| BCORL1 | BCL6 corepressor like 1 | **1,146** | 0,006151 | 8,253 | 9,577 |
| LINC01125 | long intergenic non-protein coding RNA 1125 | **1,146** | 0,177757 | 1,108 | 1,979 |
| FAM13A-AS1 | FAM13A antisense RNA 1 | **1,146** | 0,354484 | 0,332 | 0,766 |
| LAMTOR3 | late endosomal/lysosomal adaptor, MAPK and MTOR activator 3 | **1,146** | 0,000866 | 32,902 | 38,374 |
| SLC25A21-AS1 | SLC25A21 antisense RNA 1 | **1,146** | 0,041442 | 3,379 | 5,491 |
| ARHGAP5 | Rho GTPase activating protein 5 | **1,146** | 0,003116 | 75,552 | 82,749 |
| THAP8 | THAP domain containing 8 | **1,146** | 0,009969 | 4,431 | 5,746 |
| MSL1 | male specific lethal 1 homolog | **1,146** | 1,00E-06 | 79,596 | 102,607 |
| GSTA4 | glutathione S-transferase alpha 4 | **1,146** | 0,000882 | 8,364 | 10,216 |
| ZNF814 | zinc finger protein 814 | **1,146** | 0,271003 | 0,886 | 1,022 |
| MATN2 | matrilin 2 | **1,146** | 0,000791 | 16,617 | 17,431 |
| TBC1D2B | TBC1 domain family member 2B | **1,146** | 0,000647 | 23,319 | 26,625 |
| RAP1A | RAP1A, member of RAS oncogene family | **1,146** | 1,00E-06 | 54,615 | 66,532 |
| VMO1 | vitelline membrane outer layer 1 homolog | **1,145** | 0,27647 | 1,219 | 0,638 |
| ZFP3 | ZFP3 zinc finger protein | **1,145** | 0,00157 | 10,358 | 11,429 |

| | | | | | |
|---|---|---|---|---|---|
| RNF103 | ring finger protein 103 | **1,145** | 1,20E-05 | 57,052 | 55,549 |
| ZNF174 | zinc finger protein 174 | **1,145** | 0,065989 | 3,988 | 6,385 |
| IGF1R | insulin like growth factor 1 receptor | **1,145** | 0,000647 | 58,935 | 57,018 |
| ZNF44 | zinc finger protein 44 | **1,145** | 0,000572 | 9,139 | 11,493 |
| C12orf49 | chromosome 12 open reading frame 49 | **1,145** | 2,00E-06 | 44,977 | 49,037 |
| AVL9 | AVL9 cell migration associated | **1,145** | 0,000175 | 25,036 | 26,945 |
| CD44 | CD44 molecule (Indian blood group) | **1,145** | 0 | 1542,948 | 1593,182 |
| RBBP5 | RB binding protein 5, histone lysine methyltransferase complex subunit | **1,145** | 7,60E-05 | 27,861 | 40,609 |
| MICAL3 | microtubule associated monooxygenase, calponin and LIM domain containing 3 | **1,145** | 0,03126 | 18,500 | 27,008 |
| NRP1 | neuropilin 1 | **1,145** | 0,000337 | 25,646 | 26,562 |
| TRIM52 | tripartite motif containing 52 | **1,145** | 0,016035 | 7,755 | 11,301 |
| CTNS | cystinosin, lysosomal cystine transporter | **1,145** | 1,00E-06 | 27,972 | 36,203 |
| CPEB2 | cytoplasmic polyadenylation element binding protein 2 | **1,145** | 1,70E-05 | 57,606 | 54,719 |
| USP28 | ubiquitin specific peptidase 28 | **1,145** | 0,000456 | 15,122 | 16,154 |
| LRRC8B | leucine rich repeat containing 8 family member B | **1,145** | 0,075703 | 4,099 | 5,938 |
| DESI2 | desumoylating isopeptidase 2 | **1,145** | 7,00E-06 | 40,933 | 42,205 |
| TRPV1 | transient receptor potential cation channel subfamily V member 1 | **1,145** | 0,039531 | 4,487 | 4,661 |
| BBX | BBX, HMG-box containing | **1,145** | 0,000376 | 62,868 | 69,596 |
| MTUS1 | microtubule associated scaffold protein 1 | **1,144** | 5,00E-06 | 34,619 | 36,969 |
| DIP2B | disco interacting protein 2 homolog B | **1,144** | 8,80E-05 | 32,459 | 43,737 |
| LINC01139 | long intergenic non-protein coding RNA 1139 | **1,144** | 0,04018 | 4,708 | 4,342 |
| SLBP | stem-loop binding protein | **1,144** | 2,30E-05 | 38,718 | 52,485 |
| FAM78A | family with sequence similarity 78 member A | **1,144** | 0,415449 | 1,052 | 0,511 |
| SLC8A3 | solute carrier family 8 member A3 | **1,144** | 0,394244 | 0,831 | 0,830 |
| RGMA | repulsive guidance molecule family member a | **1,144** | 0,000125 | 15,122 | 16,729 |
| SKIL | SKI like proto-oncogene | **1,144** | 7,10E-05 | 75,054 | 70,043 |
| SNX10 | sorting nexin 10 | **1,144** | 0,317298 | 0,554 | 0,575 |
| FTX | FTX transcript, XIST regulator (non-protein coding) | **1,144** | 0,007301 | 16,728 | 22,284 |
| NKAPL | NFKB activating protein like | **1,144** | 0,037819 | 4,487 | 4,725 |
| TSPOAP1-AS1 | TSPOAP1 antisense RNA 1 | **1,144** | 0,426063 | 0,222 | 0,638 |
| USP46-AS1 | USP46 antisense RNA 1 | **1,144** | 0,159462 | 2,049 | 3,320 |
| ORC6 | origin recognition complex subunit 6 | **1,144** | 0,065858 | 3,268 | 3,512 |
| FAS | Fas cell surface death receptor | **1,144** | 0,003288 | 7,588 | 14,685 |

| | | | | | |
|---|---|---|---|---|---|
| LRRC17 | leucine rich repeat containing 17 | **1,144** | 0,48201 | 0,443 | 0,383 |
| MAP3K5 | mitogen-activated protein kinase kinase kinase 5 | **1,144** | 5,80E-05 | 43,426 | 42,652 |
| ZBTB18 | zinc finger and BTB domain containing 18 | **1,144** | 0,00015 | 15,232 | 22,922 |
| CEP112 | centrosomal protein 112 | **1,144** | 0,000455 | 14,180 | 23,880 |
| MAPK6 | mitogen-activated protein kinase 6 | **1,143** | 8,60E-05 | 104,023 | 109,183 |
| PYCARD | PYD and CARD domain containing | **1,143** | 0,008648 | 5,761 | 6,449 |
| SOX9-AS1 | SOX9 antisense RNA 1 | **1,143** | 0,071989 | 2,493 | 3,192 |
| PTGIR | prostaglandin I2 (prostacyclin) receptor (IP) | **1,143** | 0,023849 | 7,422 | 11,238 |
| PHACTR1 | phosphatase and actin regulator 1 | **1,143** | 0,000803 | 11,964 | 16,346 |
| OR7E12P | olfactory receptor family 7 subfamily E member 12 pseudogene | **1,143** | 0,499663 | 0,332 | 0,958 |
| HAUS3 | HAUS augmin like complex subunit 3 | **1,143** | 0,000522 | 12,463 | 15,132 |
| SIRT4 | sirtuin 4 | **1,143** | 0,450153 | 0,332 | 0,447 |
| FN1 | fibronectin 1 | **1,143** | 0,001098 | 79223,000 | 61454,105 |
| HSDL1 | hydroxysteroid dehydrogenase like 1 | **1,143** | 8,00E-06 | 31,683 | 38,693 |
| ZNF577 | zinc finger protein 577 | **1,143** | 0,010209 | 4,043 | 6,130 |
| PRICKLE2 | prickle planar cell polarity protein 2 | **1,143** | 0,002382 | 46,029 | 69,213 |
| CFHR3 | complement factor H related 3 | **1,143** | 0,44723 | 0,332 | 0,638 |
| SCUBE2 | signal peptide, CUB domain and EGF like domain containing 2 | **1,143** | 0,034973 | 2,714 | 5,300 |
| MREG | melanoregulin | **1,143** | 0,001975 | 12,684 | 15,132 |
| LIMD1 | LIM domains containing 1 | **1,143** | 1,00E-06 | 40,047 | 36,714 |
| CHRNA5 | cholinergic receptor nicotinic alpha 5 subunit | **1,143** | 0,139262 | 1,385 | 2,490 |
| TRIM25 | tripartite motif containing 25 | **1,143** | 1,10E-05 | 84,027 | 117,548 |
| ENO2 | enolase 2 | **1,143** | 0 | 81,036 | 73,044 |
| SNPH | syntaphilin | **1,143** | 0,249431 | 2,326 | 2,171 |
| ZNF10 | zinc finger protein 10 | **1,143** | 0,012175 | 4,985 | 7,470 |
| DCP2 | decapping mRNA 2 | **1,142** | 3,00E-06 | 19,664 | 24,646 |
| P2RX7 | purinergic receptor P2X 7 | **1,142** | 0,033884 | 2,770 | 2,362 |
| XPO1 | exportin 1 | **1,142** | 8,00E-06 | 112,497 | 107,012 |
| ABL2 | ABL proto-oncogene 2, non-receptor tyrosine kinase | **1,142** | 0,00056 | 125,016 | 120,804 |
| FBXO33 | F-box protein 33 | **1,142** | 3,00E-06 | 23,430 | 23,241 |
| SLC25A33 | solute carrier family 25 member 33 | **1,142** | 0,001331 | 9,250 | 11,812 |
| EPHX1 | epoxide hydrolase 1 | **1,142** | 0,001334 | 29,080 | 40,098 |
| SECISBP2L | SECIS binding protein 2 like | **1,142** | 0,001039 | 71,287 | 66,723 |

| | | | | | |
|---|---|---|---|---|---|
| C5orf51 | chromosome 5 open reading frame 51 | **1,142** | 0,000336 | 37,333 | 40,928 |
| EPB41 | erythrocyte membrane protein band 4.1 | **1,142** | 0,018168 | 5,539 | 4,023 |
| OPHN1 | oligophrenin 1 | **1,142** | 0,011156 | 7,090 | 7,151 |
| TAS1R3 | taste 1 receptor member 3 | **1,142** | 0,124644 | 1,219 | 1,596 |
| DTNB | dystrobrevin beta | **1,142** | 0,06157 | 4,099 | 3,767 |
| STARD8 | StAR related lipid transfer domain containing 8 | **1,142** | 0,021094 | 4,154 | 4,469 |
| LINC00863 | long intergenic non-protein coding RNA 863 | **1,142** | 0,066686 | 1,274 | 3,320 |
| CMTR2 | cap methyltransferase 2 | **1,142** | 0,35478 | 0,499 | 0,702 |
| PNRC2 | proline rich nuclear receptor coactivator 2 | **1,142** | 2,00E-06 | 92,058 | 116,334 |
| PPFIBP1 | PPFIA binding protein 1 | **1,141** | 9,40E-05 | 170,879 | 197,679 |
| ATP11B | ATPase phospholipid transporting 11B (putative) | **1,141** | 0,00014 | 58,824 | 63,211 |
| CROCC | ciliary rootlet coiled-coil, rootletin | **1,141** | 0,001234 | 8,364 | 13,600 |
| KIAA2026 | KIAA2026 | **1,141** | 6,00E-06 | 46,417 | 51,846 |
| TCAF1 | TRPM8 channel associated factor 1 | **1,141** | 0,079607 | 3,545 | 3,384 |
| RUBCN | RUN and cysteine rich domain containing beclin 1 interacting protein | **1,141** | 9,50E-05 | 17,614 | 21,837 |
| MICU1 | mitochondrial calcium uptake 1 | **1,141** | 3,50E-05 | 32,071 | 33,521 |
| MAP3K1 | mitogen-activated protein kinase kinase kinase 1 | **1,141** | 0,079435 | 4,930 | 5,300 |
| LINC01686 | long intergenic non-protein coding RNA 1686 | **1,141** | 0,131424 | 1,717 | 2,746 |
| ZNF774 | zinc finger protein 774 | **1,141** | 0,016823 | 8,530 | 8,875 |
| HSD3B7 | hydroxy-delta-5-steroid dehydrogenase, 3 beta- and steroid delta-isomerase 7 | **1,141** | 0,000393 | 9,029 | 10,535 |
| FAM46A | family with sequence similarity 46 member A | **1,141** | 1,00E-06 | 271,356 | 239,692 |
| EFCAB13 | EF-hand calcium binding domain 13 | **1,141** | 0,256674 | 1,163 | 1,596 |
| USP2-AS1 | USP2 antisense RNA 1 (head to head) | **1,141** | 0,533614 | 0,166 | 0,447 |
| NORAD | non-coding RNA activated by DNA damage | **1,141** | 1,10E-05 | 402,520 | 386,228 |
| TNFRSF11B | TNF receptor superfamily member 11b | **1,140** | 1,10E-05 | 4169,384 | 4454,742 |
| SMPD1 | sphingomyelin phosphodiesterase 1 | **1,140** | 5,00E-06 | 122,301 | 103,118 |
| CAVIN4 | caveolae associated protein 4 | **1,140** | 0,292245 | 1,052 | 1,277 |
| SESN1 | sestrin 1 | **1,140** | 0,001121 | 11,853 | 13,983 |
| PIK3R1 | phosphoinositide-3-kinase regulatory subunit 1 | **1,140** | 7,40E-05 | 71,121 | 73,172 |
| LNX2 | ligand of numb-protein X 2 | **1,140** | 0,000523 | 12,795 | 12,962 |
| ZNF416 | zinc finger protein 416 | **1,140** | 0,080811 | 2,548 | 2,107 |
| ZMAT3 | zinc finger matrin-type 3 | **1,140** | 0,001877 | 20,605 | 17,495 |
| TSHZ1 | teashirt zinc finger homeobox 1 | **1,140** | 0,00206 | 10,690 | 14,941 |

| | | | | | |
|---|---|---|---|---|---|
| NMT2 | N-myristoyltransferase 2 | **1,140** | 5,40E-05 | 17,282 | 17,814 |
| NRP2 | neuropilin 2 | **1,140** | 2,00E-05 | 904,577 | 1261,163 |
| KIAA0355 | KIAA0355 | **1,140** | 0,013273 | 11,189 | 12,515 |
| TIGD3 | tigger transposable element derived 3 | **1,140** | 0,477324 | 0,388 | 0,575 |
| PMS2P4 | PMS1 homolog 2, mismatch repair system component pseudogene 4 | **1,140** | 0,468649 | 0,554 | 0,319 |
| DDX60 | DExD/H-box helicase 60 | **1,140** | 0,08412 | 3,877 | 4,342 |
| SMG1P2 | SMG1P2, nonsense mediated mRNA decay associated PI3K related kinase pseudogene 2 | **1,140** | 0,417355 | 0,554 | 0,830 |
| SPINT1 | serine peptidase inhibitor, Kunitz type 1 | **1,140** | 0,046444 | 2,160 | 2,554 |
| SLC2A5 | solute carrier family 2 member 5 | **1,140** | 0,086728 | 0,831 | 1,532 |
| COL6A1 | collagen type VI alpha 1 chain | **1,140** | 1,40E-05 | 2296,864 | 2152,635 |
| BDNF-AS | BDNF antisense RNA | **1,140** | 0,042863 | 2,326 | 3,065 |
| CCDC173 | coiled-coil domain containing 173 | **1,140** | 0,356611 | 0,775 | 1,277 |
| ARFGEF2 | ADP ribosylation factor guanine nucleotide exchange factor 2 | **1,139** | 0,001331 | 86,630 | 114,994 |
| IPMK | inositol polyphosphate multikinase | **1,139** | 0,00464 | 8,087 | 9,067 |
| DOPEY1 | dopey family member 1 | **1,139** | 0,025616 | 9,195 | 10,918 |
| KIDINS220 | kinase D interacting substrate 220 | **1,139** | 0,001328 | 84,692 | 99,478 |
| TMEM132B | transmembrane protein 132B | **1,139** | 0,391838 | 0,277 | 0,447 |
| SCPEP1 | serine carboxypeptidase 1 | **1,139** | 6,10E-05 | 61,982 | 49,675 |
| CX3CL1 | C-X3-C motif chemokine ligand 1 | **1,139** | 0,001465 | 75,441 | 103,373 |
| CALM1 | calmodulin 1 | **1,139** | 1,00E-06 | 248,314 | 262,551 |
| DLGAP1-AS2 | DLGAP1 antisense RNA 2 | **1,139** | 0,000354 | 17,060 | 22,411 |
| ADORA2BP1 | adenosine A2b receptor pseudogene 1 | **1,139** | 0,019176 | 4,320 | 4,086 |
| LEAP2 | liver enriched antimicrobial peptide 2 | **1,139** | 0,24335 | 0,665 | 1,979 |
| ZNF17 | zinc finger protein 17 | **1,139** | 0,082634 | 2,493 | 3,639 |
| LINC00310 | long intergenic non-protein coding RNA 310 | **1,139** | 0,167379 | 1,163 | 1,915 |
| ACVR2A | activin A receptor type 2A | **1,139** | 0,000614 | 12,352 | 15,005 |
| MEF2D | myocyte enhancer factor 2D | **1,139** | 4,00E-06 | 69,515 | 85,878 |
| ZNF292 | zinc finger protein 292 | **1,139** | 0,001382 | 31,129 | 40,545 |
| GUCY1B3 | guanylate cyclase 1 soluble subunit beta | **1,139** | 0,262791 | 0,443 | 1,596 |
| GTF2IRD2P1 | GTF2I repeat domain containing 2 pseudogene 1 | **1,139** | 0,480023 | 0,388 | 0,192 |
| CDH13 | cadherin 13 | **1,139** | 0,004502 | 9,416 | 9,641 |
| ZNFX1 | zinc finger NFX1-type containing 1 | **1,138** | 1,50E-05 | 158,028 | 183,505 |
| USP6NL | USP6 N-terminal like | **1,138** | 0,000298 | 38,496 | 41,694 |

| | | | | | |
|---|---|---|---|---|---|
| FHL3 | four and a half LIM domains 3 | **1,138** | 0,157645 | 2,326 | 2,809 |
| RAB4B | RAB4B, member RAS oncogene family | **1,138** | 0,135826 | 1,939 | 3,384 |
| PPP2R5D | protein phosphatase 2 regulatory subunit B'delta | **1,138** | 9,00E-06 | 31,351 | 30,456 |
| FAM117B | family with sequence similarity 117 member B | **1,138** | 0,154677 | 3,102 | 3,767 |
| TPPP3 | tubulin polymerization promoting protein family member 3 | **1,138** | 0,386495 | 0,443 | 1,022 |
| NEBL | nebulette | **1,138** | 0,000163 | 66,191 | 85,495 |
| KLHDC10 | kelch domain containing 10 | **1,138** | 0,00035 | 53,119 | 55,486 |
| NUTM2D | NUT family member 2D | **1,138** | 0,28304 | 0,942 | 1,341 |
| TCN2 | transcobalamin 2 | **1,138** | 0,220642 | 1,662 | 1,149 |
| DENND5B | DENN domain containing 5B | **1,138** | 0,015869 | 12,407 | 12,131 |
| ZSCAN12 | zinc finger and SCAN domain containing 12 | **1,138** | 0,016999 | 5,761 | 6,832 |
| CSRP1 | cysteine and glycine rich protein 1 | **1,138** | 2,00E-06 | 141,356 | 154,836 |
| EMILIN3 | elastin microfibril interfacer 3 | **1,138** | 0,288766 | 0,942 | 2,107 |
| NECTIN4 | nectin cell adhesion molecule 4 | **1,138** | 0,336316 | 0,554 | 0,830 |
| RPL32P3 | ribosomal protein L32 pseudogene 3 | **1,138** | 0,047425 | 4,154 | 5,555 |
| CCDC92 | coiled-coil domain containing 92 | **1,138** | 0,136872 | 1,274 | 1,724 |
| DNAJB14 | DnaJ heat shock protein family (Hsp40) member B14 | **1,137** | 6,70E-05 | 53,064 | 50,122 |
| DLC1 | DLC1 Rho GTPase activating protein | **1,137** | 0,016124 | 10,746 | 15,132 |
| TMEM200A | transmembrane protein 200A | **1,137** | 0,226648 | 2,105 | 0,766 |
| CHMP2B | charged multivesicular body protein 2B | **1,137** | 1,00E-05 | 45,088 | 56,635 |
| STRADA | STE20-related kinase adaptor alpha | **1,137** | 0,125649 | 1,883 | 4,278 |
| IGF1 | insulin like growth factor 1 | **1,137** | 0,005601 | 6,259 | 17,112 |
| MIR9-3HG | MIR9-3 host gene | **1,137** | 0,501424 | 0,388 | 0,638 |
| AMD1 | adenosylmethionine decarboxylase 1 | **1,137** | 4,00E-06 | 143,350 | 161,476 |
| KDM3A | lysine demethylase 3A | **1,137** | 6,00E-06 | 49,408 | 63,722 |
| ARRDC3 | arrestin domain containing 3 | **1,137** | 0 | 498,179 | 691,750 |
| SMG9 | SMG9, nonsense mediated mRNA decay factor | **1,137** | 3,20E-05 | 31,849 | 52,548 |
| HAR1A | highly accelerated region 1A (non-protein coding) | **1,137** | 0,398786 | 0,222 | 0,638 |
| FGD5 | FYVE, RhoGEF and PH domain containing 5 | **1,137** | 2,10E-05 | 24,538 | 47,887 |
| HOXA13 | homeobox A13 | **1,137** | 0,038889 | 4,043 | 5,236 |
| GLUD1P3 | glutamate dehydrogenase 1 pseudogene 3 | **1,137** | 0,365846 | 0,554 | 0,894 |
| MOB1B | MOB kinase activator 1B | **1,137** | 0,001039 | 27,917 | 32,372 |
| PRELID3A | PRELI domain containing 3A | **1,137** | 0,172204 | 1,551 | 1,915 |

| | | | | | |
|---|---|---|---|---|---|
| ANKRD46 | ankyrin repeat domain 46 | **1,137** | 0,00081 | 13,460 | 18,580 |
| PTPRM | protein tyrosine phosphatase, receptor type M | **1,136** | 0,000358 | 218,847 | 270,532 |
| MBNL1 | muscleblind like splicing regulator 1 | **1,136** | 0,00142 | 206,771 | 216,579 |
| SPAG5-AS1 | SPAG5 antisense RNA 1 | **1,136** | 0,245957 | 1,052 | 2,107 |
| SPECC1 | sperm antigen with calponin homology and coiled-coil domains 1 | **1,136** | 0,000124 | 46,915 | 49,739 |
| MAP3K3 | mitogen-activated protein kinase kinase kinase 3 | **1,136** | 5,50E-05 | 50,017 | 50,888 |
| CD247 | CD247 molecule | **1,136** | 0,590827 | 0,277 | 0,575 |
| C5 | complement C5 | **1,136** | 0,03707 | 3,046 | 4,853 |
| MT-ND1 | mitochondrially encoded NADH:ubiquinone oxidoreductase core subunit 1 | **1,136** | 0,007407 | 394,323 | 584,418 |
| RAPGEF1 | Rap guanine nucleotide exchange factor 1 | **1,136** | 2,50E-05 | 58,049 | 50,186 |
| OSER1 | oxidative stress responsive serine rich 1 | **1,136** | 4,00E-06 | 60,541 | 65,765 |
| SMIM27 | small integral membrane protein 27 | **1,136** | 0,145041 | 1,385 | 1,788 |
| DDX47 | DEAD-box helicase 47 | **1,136** | 0,503872 | 0,222 | 0,319 |
| SOS2 | SOS Ras/Rho guanine nucleotide exchange factor 2 | **1,136** | 9,40E-05 | 38,607 | 37,224 |
| CCNYL1 | cyclin Y like 1 | **1,136** | 2,00E-06 | 29,523 | 33,968 |
| INE1 | inactivation escape 1 (non-protein coding) | **1,135** | 0,365552 | 0,665 | 0,830 |
| MORF4L2-AS1 | MORF4L2 antisense RNA 1 | **1,135** | 0,439837 | 0,499 | 0,958 |
| KLHL3 | kelch like family member 3 | **1,135** | 0,223152 | 1,717 | 1,213 |
| TMEM81 | transmembrane protein 81 | **1,135** | 0,07003 | 2,770 | 3,703 |
| PDE1B | phosphodiesterase 1B | **1,135** | 0,009566 | 8,862 | 4,214 |
| C21orf91 | chromosome 21 open reading frame 91 | **1,135** | 0,004142 | 11,133 | 14,239 |
| SLC38A7 | solute carrier family 38 member 7 | **1,135** | 3,50E-05 | 21,547 | 18,772 |
| ALAD | aminolevulinate dehydratase | **1,135** | 0,002594 | 14,678 | 14,685 |
| ZC3H6 | zinc finger CCCH-type containing 6 | **1,135** | 8,40E-05 | 23,873 | 29,499 |
| RORA | RAR related orphan receptor A | **1,135** | 0,002696 | 49,463 | 59,572 |
| E2F1 | E2F transcription factor 1 | **1,135** | 0,018457 | 6,259 | 4,469 |
| SAMD11 | sterile alpha motif domain containing 11 | **1,135** | 0,466031 | 0,277 | 0,638 |
| C8B | complement C8 beta chain | **1,135** | 0,25363 | 0,388 | 0,575 |
| BACE1 | beta-secretase 1 | **1,135** | 8,30E-05 | 28,858 | 28,924 |
| HR | HR, lysine demethylase and nuclear receptor corepressor | **1,135** | 0,024278 | 9,029 | 10,599 |
| OTUD4 | OTU deubiquitinase 4 | **1,135** | 0,003321 | 37,887 | 43,290 |
| DGKE | diacylglycerol kinase epsilon | **1,135** | 0,00554 | 9,859 | 14,430 |
| MMAA | methylmalonic aciduria (cobalamin deficiency) cblA type | **1,134** | 0,008959 | 10,690 | 10,854 |

| | | | | | |
|---|---|---|---|---|---|
| TECTA | tectorin alpha | **1,134** | 0,330613 | 0,499 | 1,213 |
| SEMA4A | semaphorin 4A | **1,134** | 0,380788 | 0,609 | 0,830 |
| PLEKHF2 | pleckstrin homology and FYVE domain containing 2 | **1,134** | 0,003548 | 11,355 | 11,940 |
| TG | thyroglobulin | **1,134** | 0,21221 | 1,163 | 1,915 |
| PCGF5 | polycomb group ring finger 5 | **1,134** | 0,000112 | 69,238 | 76,492 |
| DPP4 | dipeptidyl peptidase 4 | **1,134** | 0,001707 | 23,430 | 28,860 |
| ZSCAN30 | zinc finger and SCAN domain containing 30 | **1,134** | 0,006932 | 15,565 | 19,857 |
| ABHD3 | abhydrolase domain containing 3 | **1,134** | 0,000444 | 9,361 | 14,494 |
| DIRAS3 | DIRAS family GTPase 3 | **1,134** | 0,129152 | 0,499 | 2,235 |
| TMEM86A | transmembrane protein 86A | **1,134** | 0,143112 | 1,440 | 1,788 |
| PTMS | parathymosin | **1,134** | 0,000263 | 81,700 | 72,150 |
| ZEB1 | zinc finger E-box binding homeobox 1 | **1,134** | 0,005644 | 63,699 | 74,960 |
| TMEM106B | transmembrane protein 106B | **1,134** | 0,000261 | 50,017 | 55,869 |
| ANKRD44 | ankyrin repeat domain 44 | **1,134** | 0,040029 | 4,376 | 4,086 |
| UMAD1 | UBAP1-MVB12-associated (UMA) domain containing 1 | **1,134** | 0,000445 | 20,605 | 23,305 |
| SCN3B | sodium voltage-gated channel beta subunit 3 | **1,134** | 0,299314 | 1,108 | 1,022 |
| CDH19 | cadherin 19 | **1,134** | 0,008445 | 24,482 | 11,685 |
| RRM2B | ribonucleotide reductase regulatory TP53 inducible subunit M2B | **1,134** | 0,000552 | 64,973 | 76,684 |
| TFEB | transcription factor EB | **1,134** | 0,051087 | 2,991 | 4,597 |
| XIST | X inactive specific transcript (non-protein coding) | **1,134** | 0,035447 | 226,933 | 328,444 |
| PTPRD | protein tyrosine phosphatase, receptor type D | **1,134** | 0,01199 | 30,742 | 49,931 |
| TIAM2 | T-cell lymphoma invasion and metastasis 2 | **1,134** | 3,80E-05 | 256,955 | 330,040 |
| LHFPL2 | lipoma HMGIC fusion partner-like 2 | **1,133** | 6,20E-05 | 135,263 | 151,197 |
| ZNF322 | zinc finger protein 322 | **1,133** | 0,01935 | 3,490 | 6,193 |
| CENPU | centromere protein U | **1,133** | 0,100528 | 1,606 | 2,426 |
| TRPS1 | transcriptional repressor GATA binding 1 | **1,133** | 0,004154 | 78,488 | 67,617 |
| SMIM13 | small integral membrane protein 13 | **1,133** | 0,000369 | 18,002 | 19,857 |
| TCF7L2 | transcription factor 7 like 2 | **1,133** | 5,70E-05 | 45,143 | 49,292 |
| MRAS | muscle RAS oncogene homolog | **1,133** | 0,000942 | 14,568 | 12,259 |
| SPATA13 | spermatogenesis associated 13 | **1,133** | 0,000547 | 25,646 | 30,073 |
| PPTC7 | PTC7 protein phosphatase homolog | **1,133** | 0,000849 | 26,366 | 28,030 |
| BCAT1 | branched chain amino acid transaminase 1 | **1,133** | 8,00E-06 | 440,518 | 619,088 |
| ZNF24 | zinc finger protein 24 | **1,133** | 0,000132 | 81,645 | 100,117 |

| | | | | | |
|---|---|---|---|---|---|
| SLC5A10 | solute carrier family 5 member 10 | **1,133** | 0,4723 | 0,277 | 0,638 |
| MYADM | myeloid associated differentiation marker | **1,133** | 3,00E-06 | 114,325 | 88,049 |
| 5.maalis | membrane associated ring-CH-type finger 5 | **1,133** | 1,90E-05 | 30,852 | 34,224 |
| SLC44A2 | solute carrier family 44 member 2 | **1,133** | 3,30E-05 | 70,622 | 51,016 |
| PTPRH | protein tyrosine phosphatase, receptor type H | **1,133** | 0,306789 | 0,388 | 2,171 |
| NRIP1 | nuclear receptor interacting protein 1 | **1,133** | 0,002456 | 44,312 | 67,042 |
| JMY | junction mediating and regulatory protein, p53 cofactor | **1,133** | 0,000555 | 37,111 | 39,651 |
| FAM171A2 | family with sequence similarity 171 member A2 | **1,133** | 0,260274 | 0,388 | 1,022 |
| LINC00174 | long intergenic non-protein coding RNA 174 | **1,133** | 0,057435 | 5,871 | 9,131 |
| ZNF304 | zinc finger protein 304 | **1,132** | 0,010514 | 6,370 | 7,662 |
| CHN1 | chimerin 1 | **1,132** | 0,436279 | 0,222 | 1,660 |
| SLC40A1 | solute carrier family 40 member 1 | **1,132** | 0,000274 | 18,999 | 18,900 |
| STARD5 | StAR related lipid transfer domain containing 5 | **1,132** | 0,230333 | 1,939 | 2,043 |
| NR4A1 | nuclear receptor subfamily 4 group A member 1 | **1,132** | 0,00734 | 5,539 | 8,556 |
| CLK4 | CDC like kinase 4 | **1,132** | 0,000287 | 15,731 | 19,730 |
| PTPRS | protein tyrosine phosphatase, receptor type S | **1,132** | 0,000197 | 62,591 | 98,967 |
| KLF4 | Kruppel like factor 4 | **1,132** | 4,00E-06 | 113,882 | 157,135 |
| ITGA10 | integrin subunit alpha 10 | **1,132** | 2,80E-05 | 289,746 | 218,877 |
| CD274 | CD274 molecule | **1,132** | 0,003326 | 29,246 | 36,905 |
| ZFPM2 | zinc finger protein, FOG family member 2 | **1,132** | 0,047023 | 4,708 | 5,746 |
| DUSP7 | dual specificity phosphatase 7 | **1,132** | 0,000937 | 22,045 | 26,625 |
| QKI | QKI, KH domain containing RNA binding | **1,132** | 4,90E-05 | 131,385 | 132,999 |
| TIAM1 | T-cell lymphoma invasion and metastasis 1 | **1,132** | 0,021811 | 7,478 | 10,152 |
| ZNF461 | zinc finger protein 461 | **1,132** | 0,016373 | 5,096 | 5,683 |
| UBE2L6 | ubiquitin conjugating enzyme E2 L6 | **1,131** | 0,019508 | 8,752 | 7,662 |
| GPATCH2L | G-patch domain containing 2 like | **1,131** | 0,001585 | 37,444 | 46,036 |
| TRIM33 | tripartite motif containing 33 | **1,131** | 7,20E-05 | 38,108 | 46,547 |
| BCOR | BCL6 corepressor | **1,131** | 0,002013 | 31,295 | 37,416 |
| ARSB | arylsulfatase B | **1,131** | 3,60E-05 | 274,514 | 203,873 |
| ZNF654 | zinc finger protein 654 | **1,131** | 0,001841 | 22,710 | 28,030 |
| GRAMD3 | GRAM domain containing 3 | **1,131** | 9,90E-05 | 25,535 | 24,008 |
| PAIP2B | poly(A) binding protein interacting protein 2B | **1,131** | 0,032164 | 3,656 | 6,768 |
| NEIL1 | nei like DNA glycosylase 1 | **1,131** | 0,173901 | 1,329 | 1,979 |

| | | | | | |
|---|---|---|---|---|---|
| GALM | galactose mutarotase | **1,131** | 0,117919 | 2,049 | 2,299 |
| DYNC1H1 | dynein cytoplasmic 1 heavy chain 1 | **1,131** | 0,001377 | 381,306 | 380,545 |
| IQSEC1 | IQ motif and Sec7 domain 1 | **1,131** | 4,30E-05 | 41,820 | 51,846 |
| ATP6V0A1 | ATPase H+ transporting V0 subunit a1 | **1,131** | 1,00E-06 | 73,669 | 115,568 |
| TUBD1 | tubulin delta 1 | **1,131** | 0,016113 | 5,761 | 8,045 |
| PIM3 | Pim-3 proto-oncogene, serine/threonine kinase | **1,131** | 1,00E-05 | 150,163 | 146,855 |
| TSPAN33 | tetraspanin 33 | **1,131** | 0,06313 | 3,434 | 5,044 |
| RSPH9 | radial spoke head 9 homolog | **1,131** | 0,431337 | 0,720 | 0,575 |
| BST1 | bone marrow stromal cell antigen 1 | **1,131** | 0,517494 | 0,499 | 0,319 |
| AK9 | adenylate kinase 9 | **1,131** | 0,00135 | 8,585 | 8,173 |
| TBC1D15 | TBC1 domain family member 15 | **1,131** | 5,50E-05 | 60,098 | 62,637 |
| FAM133B | family with sequence similarity 133 member B | **1,131** | 0,031742 | 6,979 | 10,599 |
| ACAN | aggrecan | **1,131** | 5,80E-05 | 4128,173 | 3863,812 |
| HSPA4L | heat shock protein family A (Hsp70) member 4 like | **1,131** | 0,00295 | 9,915 | 17,495 |
| AR | androgen receptor | **1,130** | 0,04397 | 6,425 | 7,598 |
| TMOD1 | tropomodulin 1 | **1,130** | 0,024359 | 10,026 | 6,960 |
| C1orf167 | chromosome 1 open reading frame 167 | **1,130** | 0,561037 | 0,166 | 0,128 |
| ZNF568 | zinc finger protein 568 | **1,130** | 0,008136 | 6,868 | 8,045 |
| MSANTD2 | Myb/SANT DNA binding domain containing 2 | **1,130** | 0,006579 | 8,419 | 10,408 |
| ZMAT4 | zinc finger matrin-type 4 | **1,130** | 0,441527 | 0,277 | 0,638 |
| HDAC5 | histone deacetylase 5 | **1,130** | 0,000147 | 48,356 | 49,803 |
| COL6A3 | collagen type VI alpha 3 chain | **1,130** | 0,000889 | 1596,788 | 1204,592 |
| GTF2A1 | general transcription factor IIA subunit 1 | **1,130** | 0,000189 | 75,497 | 82,430 |
| ITIH6 | inter-alpha-trypsin inhibitor heavy chain family member 6 | **1,130** | 0,000285 | 92,834 | 58,550 |
| GDF7 | growth differentiation factor 7 | **1,130** | 0,517814 | 0,111 | 0,766 |
| RAB23 | RAB23, member RAS oncogene family | **1,130** | 0,000606 | 21,713 | 21,198 |
| LONRF3 | LON peptidase N-terminal domain and ring finger 3 | **1,130** | 0,026053 | 4,985 | 8,620 |
| CDR2L | cerebellar degeneration related protein 2 like | **1,130** | 0,037059 | 10,303 | 8,492 |
| CASC15 | cancer susceptibility 15 (non-protein coding) | **1,130** | 0,316323 | 0,831 | 1,724 |
| MGLL | monoglyceride lipase | **1,130** | 0,000464 | 124,296 | 106,246 |
| ZDHHC17 | zinc finger DHHC-type containing 17 | **1,130** | 0,000344 | 17,116 | 21,326 |
| RND3 | Rho family GTPase 3 | **1,130** | 8,30E-05 | 863,533 | 575,160 |
| PARP9 | poly(ADP-ribose) polymerase family member 9 | **1,130** | 0,003066 | 13,737 | 11,748 |

| | | | | | |
|---|---|---|---|---|---|
| ACSL1 | acyl-CoA synthetase long-chain family member 1 | **1,130** | 2,60E-05 | 74,666 | 66,659 |
| IGF2 | insulin like growth factor 2 | **1,130** | 0,32313 | 2,714 | 2,937 |
| COL4A1 | collagen type IV alpha 1 chain | **1,130** | 0,018372 | 6,314 | 7,598 |
| FTH1P4 | ferritin heavy chain 1 pseudogene 4 | **1,130** | 0,515321 | 0,775 | 0,447 |
| SPAG1 | sperm associated antigen 1 | **1,129** | 0,013552 | 9,859 | 8,747 |
| SCAMP1-AS1 | SCAMP1 antisense RNA 1 | **1,129** | 0,136059 | 2,049 | 4,916 |
| HPS1 | HPS1, biogenesis of lysosomal organelles complex 3 subunit 1 | **1,129** | 0,000882 | 31,794 | 25,029 |
| IL24 | interleukin 24 | **1,129** | 0,229027 | 2,770 | 2,682 |
| CALCA | calcitonin related polypeptide alpha | **1,129** | 0,377813 | 0,499 | 1,277 |
| REEP2 | receptor accessory protein 2 | **1,129** | 0,181932 | 1,440 | 2,171 |
| GCLM | glutamate-cysteine ligase modifier subunit | **1,129** | 8,50E-05 | 135,983 | 116,845 |
| PKP1 | plakophilin 1 | **1,129** | 0,251021 | 4,210 | 2,809 |
| PLEKHH2 | pleckstrin homology, MyTH4 and FERM domain containing H2 | **1,129** | 0,022037 | 6,591 | 15,069 |
| IL6R | interleukin 6 receptor | **1,129** | 5,90E-05 | 35,339 | 30,137 |
| EFNA1 | ephrin A1 | **1,129** | 0,006805 | 15,675 | 9,769 |
| SCAND2P | SCAN domain containing 2 pseudogene | **1,129** | 0,002195 | 8,198 | 12,131 |
| CPHL1P | ceruloplasmin and hephaestin-like 1 pseudogene | **1,129** | 0,618319 | 0,332 | 0,255 |
| LRP4 | LDL receptor related protein 4 | **1,128** | 0,059023 | 7,478 | 8,492 |
| FTH1P23 | ferritin heavy chain 1 pseudogene 23 | **1,128** | 0,007447 | 7,644 | 5,746 |
| ZBTB43 | zinc finger and BTB domain containing 43 | **1,128** | 0,006989 | 25,812 | 26,625 |
| CBX2 | chromobox 2 | **1,128** | 0,314264 | 1,108 | 1,405 |
| LIME1 | Lck interacting transmembrane adaptor 1 | **1,128** | 0,245907 | 0,886 | 1,469 |
| CTDSP2 | CTD small phosphatase 2 | **1,128** | 4,40E-05 | 221,173 | 232,924 |
| ERAP1 | endoplasmic reticulum aminopeptidase 1 | **1,128** | 5,60E-05 | 119,477 | 110,013 |
| ZNF471 | zinc finger protein 471 | **1,128** | 0,085445 | 9,306 | 10,982 |
| IZUMO4 | IZUMO family member 4 | **1,128** | 0,25564 | 1,219 | 1,532 |
| ZNF75D | zinc finger protein 75D | **1,128** | 0,024236 | 8,198 | 8,556 |
| CLSTN3 | calsyntenin 3 | **1,128** | 0,004936 | 18,223 | 15,643 |
| LGALS8 | galectin 8 | **1,128** | 1,60E-05 | 53,728 | 66,532 |
| CFAP43 | cilia and flagella associated protein 43 | **1,128** | 0,275106 | 0,997 | 1,341 |
| SLC44A1 | solute carrier family 44 member 1 | **1,128** | 0,001933 | 19,830 | 21,581 |
| CBL | Cbl proto-oncogene | **1,128** | 0,006133 | 18,833 | 20,049 |
| CPQ | carboxypeptidase Q | **1,128** | 9,00E-06 | 38,829 | 27,200 |

| | | | | | |
|---|---|---|---|---|---|
| BFSP1 | beaded filament structural protein 1 | **1,128** | 0,072383 | 2,880 | 3,320 |
| ATP2B1 | ATPase plasma membrane Ca2+ transporting 1 | **1,127** | 8,00E-06 | 768,539 | 752,407 |
| HINFP | histone H4 transcription factor | **1,127** | 0,000941 | 13,737 | 15,132 |
| C18orf25 | chromosome 18 open reading frame 25 | **1,127** | 9,20E-05 | 25,313 | 32,180 |
| ZNF596 | zinc finger protein 596 | **1,127** | 0,038783 | 5,317 | 6,513 |
| CDC42SE2 | CDC42 small effector 2 | **1,127** | 0,000656 | 22,932 | 28,158 |
| FAM175A | family with sequence similarity 175 member A | **1,127** | 0,001683 | 9,749 | 12,515 |
| RTN4RL1 | reticulon 4 receptor like 1 | **1,127** | 0,262412 | 1,329 | 1,341 |
| MROH8 | maestro heat like repeat family member 8 | **1,127** | 0,23094 | 1,274 | 1,915 |
| SOD2 | superoxide dismutase 2 | **1,127** | 1,60E-05 | 18997,497 | 23370,908 |
| ZNF677 | zinc finger protein 677 | **1,127** | 0,051447 | 7,533 | 9,833 |
| SLC2A13 | solute carrier family 2 member 13 | **1,127** | 0,002318 | 18,999 | 24,901 |
| GNS | glucosamine (N-acetyl)-6-sulfatase | **1,127** | 1,40E-05 | 147,449 | 129,296 |
| HIVEP2 | human immunodeficiency virus type I enhancer binding protein 2 | **1,127** | 0,024989 | 219,179 | 293,518 |
| WDCP | WD repeat and coiled coil containing | **1,127** | 0,020787 | 5,816 | 8,173 |
| LRRFIP1P1 | LRR binding FLII interacting protein 1 pseudogene 1 | **1,127** | 0,500751 | 0,388 | 1,277 |
| ZNF468 | zinc finger protein 468 | **1,127** | 0,004875 | 9,029 | 10,216 |
| KIAA0895 | KIAA0895 | **1,127** | 0,101275 | 3,877 | 5,363 |
| TP53I3 | tumor protein p53 inducible protein 3 | **1,127** | 0,031769 | 5,539 | 4,916 |
| EPB41L5 | erythrocyte membrane protein band 4.1 like 5 | **1,127** | 0,00777 | 13,737 | 13,919 |
| FAM118B | family with sequence similarity 118 member B | **1,126** | 0,004741 | 11,244 | 15,962 |
| TANC1 | tetratricopeptide repeat, ankyrin repeat and coiled-coil containing 1 | **1,126** | 0,000867 | 29,191 | 26,945 |
| NFYB | nuclear transcription factor Y subunit beta | **1,126** | 0,002854 | 14,789 | 17,942 |
| FILIP1L | filamin A interacting protein 1 like | **1,126** | 0,219914 | 1,496 | 2,426 |
| MARK1 | microtubule affinity regulating kinase 1 | **1,126** | 0,023121 | 6,204 | 6,577 |
| NXF1 | nuclear RNA export factor 1 | **1,126** | 4,00E-06 | 70,733 | 98,201 |
| GUCA1B | guanylate cyclase activator 1B | **1,126** | 0,289775 | 0,332 | 1,405 |
| PHLDA1 | pleckstrin homology like domain family A member 1 | **1,126** | 1,70E-05 | 393,159 | 491,516 |
| ZYG11B | zyg-11 family member B, cell cycle regulator | **1,126** | 0,000634 | 29,911 | 32,947 |
| ADSS | adenylosuccinate synthase | **1,126** | 0,000207 | 98,705 | 155,411 |
| PLXNB1 | plexin B1 | **1,126** | 5,30E-05 | 87,350 | 54,464 |
| SEMA7A | semaphorin 7A (John Milton Hagen blood group) | **1,126** | 0,014696 | 25,036 | 22,603 |
| SERPINI1 | serpin family I member 1 | **1,126** | 0,001715 | 34,453 | 26,753 |

| | | | | | |
|---|---|---|---|---|---|
| RMND5A | required for meiotic nuclear division 5 homolog A | **1,126** | 8,00E-06 | 37,942 | 50,250 |
| TNFRSF19 | TNF receptor superfamily member 19 | **1,126** | 0,232789 | 1,717 | 1,341 |
| MPZL2 | myelin protein zero like 2 | **1,126** | 0,008002 | 7,256 | 16,026 |
| ZNF767P | zinc finger family member 767, pseudogene | **1,126** | 0,021862 | 5,484 | 7,470 |
| CDK19 | cyclin dependent kinase 19 | **1,126** | 0,00038 | 24,427 | 21,581 |
| FAM103A1 | family with sequence similarity 103 member A1 | **1,126** | 0,029929 | 6,979 | 6,768 |
| SHMT1 | serine hydroxymethyltransferase 1 | **1,126** | 0,268508 | 1,329 | 1,149 |
| USP9X | ubiquitin specific peptidase 9, X-linked | **1,125** | 0,001007 | 142,464 | 136,511 |
| SLC25A4 | solute carrier family 25 member 4 | **1,125** | 0,002132 | 13,958 | 20,113 |
| ZNF543 | zinc finger protein 543 | **1,125** | 0,322104 | 1,662 | 2,043 |
| MYO1D | myosin ID | **1,125** | 0,000107 | 177,415 | 200,680 |
| FAM110B | family with sequence similarity 110 member B | **1,125** | 8,60E-05 | 48,189 | 55,932 |
| TMEM170B | transmembrane protein 170B | **1,125** | 0,01578 | 8,530 | 8,428 |
| ZEB2 | zinc finger E-box binding homeobox 2 | **1,125** | 0,000673 | 91,006 | 96,669 |
| CPNE3 | copine 3 | **1,125** | 4,70E-05 | 65,471 | 62,381 |
| LIFR-AS1 | LIFR antisense RNA 1 | **1,125** | 0,087037 | 2,936 | 3,576 |
| SAMD9L | sterile alpha motif domain containing 9 like | **1,125** | 0,005144 | 34,176 | 34,032 |
| PAXBP1 | PAX3 and PAX7 binding protein 1 | **1,125** | 0,001781 | 16,229 | 22,539 |
| CHAF1A | chromatin assembly factor 1 subunit A | **1,125** | 0,059189 | 6,425 | 6,193 |
| ZFAND5 | zinc finger AN1-type containing 5 | **1,125** | 1,30E-05 | 211,313 | 178,524 |
| PLAGL1 | PLAG1 like zinc finger 1 | **1,125** | 0,000309 | 36,059 | 48,462 |
| SUGT1P3 | SGT1 homolog, MIS12 kinetochore complex assembly cochaperone pseudogene 3 | **1,125** | 0,509032 | 0,332 | 0,447 |
| NCOA1 | nuclear receptor coactivator 1 | **1,125** | 0,004556 | 35,893 | 40,609 |
| ZNRF2P2 | zinc and ring finger 2 pseudogene 2 | **1,125** | 0,322157 | 0,831 | 1,341 |
| IER5 | immediate early response 5 | **1,125** | 2,30E-05 | 143,405 | 186,569 |
| MSX2 | msh homeobox 2 | **1,125** | 0,080877 | 1,772 | 3,831 |
| RIC3 | RIC3 acetylcholine receptor chaperone | **1,124** | 0,004465 | 6,204 | 8,747 |
| SCOC | short coiled-coil protein | **1,124** | 6,10E-05 | 64,585 | 76,939 |
| IFNLR1 | interferon lambda receptor 1 | **1,124** | 0,150646 | 2,382 | 2,809 |
| AXIN2 | axin 2 | **1,124** | 5,70E-05 | 68,185 | 105,991 |
| LAMC1 | laminin subunit gamma 1 | **1,124** | 0,000244 | 184,615 | 161,540 |
| BMS1P10 | BMS1, ribosome biogenesis factor pseudogene 10 | **1,124** | 0,292559 | 1,219 | 0,702 |
| TYRO3 | TYRO3 protein tyrosine kinase | **1,124** | 0,016502 | 12,352 | 9,961 |

| | | | | | |
|---|---|---|---|---|---|
| ZNF124 | zinc finger protein 124 | **1,124** | 0,254896 | 1,163 | 2,618 |
| PEBP4 | phosphatidylethanolamine binding protein 4 | **1,124** | 0,398288 | 0,443 | 0,192 |
| DHRS2 | dehydrogenase/reductase 2 | **1,124** | 0,030581 | 17,946 | 44,056 |
| NAV2 | neuron navigator 2 | **1,124** | 0,004443 | 73,614 | 89,709 |
| THSD4 | thrombospondin type 1 domain containing 4 | **1,124** | 0,008667 | 9,527 | 9,322 |
| SIRPAP1 | signal regulatory protein alpha pseudogene 1 | **1,124** | 0,130111 | 5,982 | 3,767 |
| C11orf21 | chromosome 11 open reading frame 21 | **1,124** | 0,39827 | 0,720 | 0,702 |
| SLC9A1 | solute carrier family 9 member A1 | **1,124** | 1,90E-05 | 70,567 | 99,414 |
| SLC35D2 | solute carrier family 35 member D2 | **1,124** | 0,131781 | 2,880 | 2,299 |
| ESR1 | estrogen receptor 1 | **1,124** | 0,086001 | 5,428 | 4,278 |
| GBP2 | guanylate binding protein 2 | **1,124** | 8,00E-06 | 82,753 | 105,288 |
| TEX9 | testis expressed 9 | **1,124** | 0,074014 | 4,043 | 4,342 |
| SBNO2 | strawberry notch homolog 2 | **1,124** | 0,473214 | 0,665 | 0,638 |
| VPS37B | VPS37B, ESCRT-I subunit | **1,124** | 2,00E-06 | 58,547 | 79,493 |
| EPS8L2 | EPS8 like 2 | **1,124** | 7,00E-06 | 111,611 | 150,813 |
| TNFRSF4 | TNF receptor superfamily member 4 | **1,124** | 0,477365 | 0,277 | 0,192 |
| HIST1H3H | histone cluster 1 H3 family member h | **1,123** | 0,36452 | 1,052 | 1,405 |
| AFAP1L2 | actin filament associated protein 1 like 2 | **1,123** | 0,198576 | 1,440 | 2,299 |
| GLIPR2 | GLI pathogenesis related 2 | **1,123** | 0,043846 | 2,825 | 5,491 |
| ZNF92 | zinc finger protein 92 | **1,123** | 0,018806 | 3,822 | 5,427 |
| PCDHGC3 | protocadherin gamma subfamily C, 3 | **1,123** | 0,025233 | 59,655 | 62,701 |
| UXS1 | UDP-glucuronate decarboxylase 1 | **1,123** | 1,30E-05 | 98,705 | 90,731 |
| SEC61A2 | Sec61 translocon alpha 2 subunit | **1,123** | 0,041905 | 7,145 | 9,322 |
| TCP11L2 | t-complex 11 like 2 | **1,123** | 0,074267 | 7,976 | 6,768 |
| CARNS1 | carnosine synthase 1 | **1,123** | 0,582654 | 0,166 | 0,255 |
| ATP2B4 | ATPase plasma membrane Ca2+ transporting 4 | **1,123** | 0,000421 | 129,890 | 112,057 |
| TDRKH | tudor and KH domain containing | **1,123** | 0,009753 | 8,807 | 9,258 |
| PHTF2 | putative homeodomain transcription factor 2 | **1,123** | 0,000414 | 31,406 | 37,544 |
| JOSD1 | Josephin domain containing 1 | **1,123** | 4,70E-05 | 58,824 | 58,614 |
| NKX3-1 | NK3 homeobox 1 | **1,123** | 1,80E-05 | 175,864 | 192,954 |
| DMRTA1 | DMRT like family A1 | **1,123** | 0,000924 | 15,177 | 14,302 |
| ISG15 | ISG15 ubiquitin-like modifier | **1,123** | 0,127769 | 3,545 | 5,427 |
| ACAP3 | ArfGAP with coiled-coil, ankyrin repeat and PH domains 3 | **1,123** | 0,009491 | 10,081 | 8,939 |

| | | | | | |
|---|---|---|---|---|---|
| GM2A | GM2 ganglioside activator | **1,123** | 0,007176 | 19,996 | 18,006 |
| LHX2 | LIM homeobox 2 | **1,123** | 0,54688 | 0,277 | 0,447 |
| DIP2C | disco interacting protein 2 homolog C | **1,123** | 0,000679 | 25,203 | 22,986 |
| STAM | signal transducing adaptor molecule | **1,123** | 9,10E-05 | 30,409 | 38,182 |
| TSPAN11 | tetraspanin 11 | **1,123** | 0,217345 | 0,886 | 1,596 |
| ZNF234 | zinc finger protein 234 | **1,123** | 0,069596 | 5,816 | 4,916 |
| RPP25 | ribonuclease P and MRP subunit p25 | **1,122** | 0,009002 | 11,687 | 16,218 |
| NFE2L1 | nuclear factor, erythroid 2 like 1 | **1,122** | 1,30E-05 | 1383,314 | 1349,467 |
| JMJD1C | jumonji domain containing 1C | **1,122** | 0,033829 | 89,566 | 117,356 |
| UHMK1 | U2AF homology motif kinase 1 | **1,122** | 0,001176 | 90,286 | 91,241 |
| SNX30 | sorting nexin family member 30 | **1,122** | 0,003942 | 15,232 | 20,240 |
| HPCAL1 | hippocalcin like 1 | **1,122** | 0,002304 | 21,270 | 25,732 |
| FERMT3 | fermitin family member 3 | **1,122** | 0,453782 | 0,886 | 1,022 |
| KIAA1551 | KIAA1551 | **1,122** | 0,013722 | 8,253 | 9,131 |
| AQP7P1 | aquaporin 7 pseudogene 1 | **1,122** | 0,215323 | 0,332 | 0,958 |
| EIF5A2 | eukaryotic translation initiation factor 5A2 | **1,122** | 0,036114 | 6,481 | 7,215 |
| RAB7A | RAB7A, member RAS oncogene family | **1,122** | 4,00E-06 | 218,514 | 202,468 |
| SVIL | supervillin | **1,122** | 3,60E-05 | 101,641 | 107,076 |
| ZNF835 | zinc finger protein 835 | **1,122** | 0,591232 | 0,443 | 0,766 |
| UNG | uracil DNA glycosylase | **1,122** | 0,002558 | 13,072 | 13,408 |
| AUH | AU RNA binding methylglutaconyl-CoA hydratase | **1,122** | 0,041692 | 7,035 | 7,854 |
| ARHGAP12 | Rho GTPase activating protein 12 | **1,122** | 0,001662 | 25,756 | 28,285 |
| TLE4 | transducin like enhancer of split 4 | **1,122** | 0,001419 | 19,719 | 20,560 |
| CYP7B1 | cytochrome P450 family 7 subfamily B member 1 | **1,121** | 0,009391 | 25,036 | 27,902 |
| USP27X-AS1 | USP27X antisense RNA 1 (head to head) | **1,121** | 0,173186 | 1,939 | 2,171 |
| MDM2 | MDM2 proto-oncogene | **1,121** | 4,60E-05 | 162,293 | 251,249 |
| HES4 | hes family bHLH transcription factor 4 | **1,121** | 0,436587 | 0,443 | 0,638 |
| DCUN1D1 | defective in cullin neddylation 1 domain containing 1 | **1,121** | 0,002597 | 23,375 | 22,284 |
| PGLYRP2 | peptidoglycan recognition protein 2 | **1,121** | 0,048571 | 3,877 | 1,213 |
| RAB10 | RAB10, member RAS oncogene family | **1,121** | 1,70E-05 | 123,022 | 129,679 |
| LINC00346 | long intergenic non-protein coding RNA 346 | **1,121** | 0,006753 | 10,690 | 11,557 |
| ABCB9 | ATP binding cassette subfamily B member 9 | **1,121** | 3,80E-05 | 67,410 | 49,101 |
| ZNF555 | zinc finger protein 555 | **1,121** | 0,03364 | 5,096 | 6,193 |

| | | | | | |
|---|---|---|---|---|---|
| OSBPL3 | oxysterol binding protein like 3 | **1,121** | 0,025122 | 5,871 | 9,194 |
| FBXO30 | F-box protein 30 | **1,121** | 0,000315 | 64,308 | 62,509 |
| WTAP | WT1 associated protein | **1,121** | 0,000111 | 399,806 | 443,565 |
| RELT | RELT, TNF receptor | **1,121** | 0,00339 | 17,669 | 23,241 |
| CKS2 | CDC28 protein kinase regulatory subunit 2 | **1,121** | 0,019702 | 5,484 | 6,130 |
| WNK3 | WNK lysine deficient protein kinase 3 | **1,121** | 0,118333 | 3,046 | 3,959 |
| BMPR2 | bone morphogenetic protein receptor type 2 | **1,121** | 0,001379 | 44,368 | 38,374 |
| LINC01003 | long intergenic non-protein coding RNA 1003 | **1,121** | 0,02091 | 4,154 | 7,087 |
| SDC2 | syndecan 2 | **1,121** | 1,70E-05 | 227,432 | 207,065 |
| ZNF429 | zinc finger protein 429 | **1,121** | 0,091728 | 3,545 | 5,236 |
| FHOD1 | formin homology 2 domain containing 1 | **1,121** | 0,024732 | 5,871 | 7,917 |
| AP1S2 | adaptor related protein complex 1 sigma 2 subunit | **1,120** | 0,000671 | 19,110 | 20,624 |
| REST | RE1 silencing transcription factor | **1,120** | 0,000413 | 35,228 | 38,118 |
| ZNF425 | zinc finger protein 425 | **1,120** | 0,015187 | 6,647 | 9,577 |
| PKN2 | protein kinase N2 | **1,120** | 0,00028 | 30,631 | 37,991 |
| HAUS7 | HAUS augmin like complex subunit 7 | **1,120** | 0,574929 | 0,443 | 0,064 |
| CASC10 | cancer susceptibility 10 | **1,120** | 0,363614 | 0,997 | 0,702 |
| CENPE | centromere protein E | **1,120** | 0,332764 | 4,320 | 1,979 |
| SEC31B | SEC31 homolog B, COPII coat complex component | **1,120** | 0,403497 | 0,665 | 0,830 |
| RABGAP1L | RAB GTPase activating protein 1 like | **1,120** | 0,006414 | 16,839 | 15,388 |
| P3H2-AS1 | P3H2 antisense RNA 1 | **1,120** | 0,528752 | 0,443 | 0,702 |
| CHD9 | chromodomain helicase DNA binding protein 9 | **1,120** | 0,029802 | 56,276 | 68,702 |
| TBC1D1 | TBC1 domain family member 1 | **1,120** | 0,000472 | 71,343 | 57,018 |
| WASHC2A | WASH complex subunit 2A | **1,120** | 0,0094 | 19,220 | 18,070 |
| PIAS1 | protein inhibitor of activated STAT 1 | **1,120** | 0,000552 | 28,304 | 35,564 |
| 8.maalis | membrane associated ring-CH-type finger 8 | **1,120** | 0,241809 | 3,822 | 3,065 |
| ZNF19 | zinc finger protein 19 | **1,120** | 0,127296 | 2,216 | 2,362 |
| VPS4B | vacuolar protein sorting 4 homolog B | **1,120** | 4,40E-05 | 57,384 | 56,124 |
| ZNF506 | zinc finger protein 506 | **1,120** | 0,027218 | 7,865 | 9,386 |
| DENND4C | DENN domain containing 4C | **1,120** | 0,009874 | 31,018 | 29,435 |
| POLR3K | RNA polymerase III subunit K | **1,120** | 0,049982 | 7,976 | 11,621 |
| SSBP2 | single stranded DNA binding protein 2 | **1,120** | 0,053361 | 7,145 | 7,023 |
| ENPP5 | ectonucleotide pyrophosphatase/phosphodiesterase 5 (putative) | **1,120** | 0,405596 | 1,385 | 0,128 |

| | | | | | |
|---|---|---|---|---|---|
| SPIRE2 | spire type actin nucleation factor 2 | **1,120** | 0,265708 | 2,326 | 2,809 |
| PAPLN | papilin, proteoglycan like sulfated glycoprotein | **1,120** | 0,096039 | 2,493 | 4,150 |
| SCAMP1 | secretory carrier membrane protein 1 | **1,120** | 0,000134 | 76,549 | 64,552 |
| FAM227B | family with sequence similarity 227 member B | **1,120** | 0,102406 | 3,600 | 5,172 |
| C10orf111 | chromosome 10 open reading frame 111 | **1,119** | 0,580738 | 0,111 | 0,575 |
| LINC00882 | long intergenic non-protein coding RNA 882 | **1,119** | 0,552411 | 0,332 | 0,638 |
| BBS12 | Bardet-Biedl syndrome 12 | **1,119** | 0,000597 | 16,894 | 22,794 |
| SPAG17 | sperm associated antigen 17 | **1,119** | 0,327574 | 0,775 | 1,852 |
| EBF3 | early B-cell factor 3 | **1,119** | 0,50145 | 0,443 | 0,447 |
| SPATA41 | spermatogenesis associated 41 (non-protein coding) | **1,119** | 0,44052 | 0,499 | 0,830 |
| AASS | aminoadipate-semialdehyde synthase | **1,119** | 0,189003 | 6,259 | 9,386 |
| NBPF3 | NBPF member 3 | **1,119** | 0,236687 | 1,551 | 3,065 |
| TSIX | TSIX transcript, XIST antisense RNA | **1,119** | 0,52528 | 1,551 | 2,809 |
| FBXO45 | F-box protein 45 | **1,119** | 0,003918 | 11,743 | 11,621 |
| TFAP2E | transcription factor AP-2 epsilon | **1,119** | 0,322016 | 0,720 | 1,660 |
| CGGBP1 | CGG triplet repeat binding protein 1 | **1,119** | 3,20E-05 | 72,783 | 70,363 |
| SSBP3 | single stranded DNA binding protein 3 | **1,119** | 7,90E-05 | 21,768 | 26,051 |
| EGLN1 | egl-9 family hypoxia inducible factor 1 | **1,119** | 0,000513 | 130,776 | 131,914 |
| HECA | hdc homolog, cell cycle regulator | **1,119** | 0,000658 | 38,441 | 35,564 |
| INCENP | inner centromere protein | **1,119** | 0,009293 | 11,632 | 11,238 |
| RAP2B | RAP2B, member of RAS oncogene family | **1,119** | 0,00125 | 12,906 | 16,729 |
| LRRN2 | leucine rich repeat neuronal 2 | **1,119** | 0,286308 | 2,936 | 1,915 |
| CNBP | CCHC-type zinc finger nucleic acid binding protein | **1,119** | 3,00E-06 | 325,473 | 412,343 |
| POLH | DNA polymerase eta | **1,119** | 0,025143 | 8,419 | 11,940 |
| C15orf39 | chromosome 15 open reading frame 39 | **1,119** | 7,60E-05 | 25,258 | 26,945 |
| TROVE2 | TROVE domain family member 2 | **1,118** | 0,000773 | 34,730 | 40,162 |
| LCORL | ligand dependent nuclear receptor corepressor like | **1,118** | 0,053138 | 7,256 | 7,790 |
| TBX18 | T-box 18 | **1,118** | 0,002072 | 14,180 | 18,836 |
| CALHM2 | calcium homeostasis modulator 2 | **1,118** | 0,039522 | 5,207 | 4,916 |
| LMLN | leishmanolysin like peptidase | **1,118** | 0,009469 | 18,334 | 20,815 |
| GLP2R | glucagon like peptide 2 receptor | **1,118** | 0,010876 | 6,591 | 5,746 |
| SLC6A8 | solute carrier family 6 member 8 | **1,118** | 0,004588 | 86,741 | 84,793 |
| GPD1 | glycerol-3-phosphate dehydrogenase 1 | **1,118** | 0,491961 | 0,277 | 0,702 |

| | | | | | |
|---|---|---|---|---|---|
| NCOA7 | nuclear receptor coactivator 7 | **1,118** | 0,000743 | 55,002 | 62,892 |
| CHSY1 | chondroitin sulfate synthase 1 | **1,118** | 0,000522 | 51,901 | 59,636 |
| ANO1 | anoctamin 1 | **1,118** | 0,501501 | 0,720 | 1,532 |
| SCARB2 | scavenger receptor class B member 2 | **1,118** | 2,00E-06 | 230,368 | 214,983 |
| IPP | intracisternal A particle-promoted polypeptide | **1,118** | 0,008988 | 12,851 | 10,663 |
| AGO4 | argonaute 4, RISC catalytic component | **1,118** | 0,024113 | 10,635 | 9,194 |
| TMEM47 | transmembrane protein 47 | **1,118** | 0,000113 | 113,107 | 107,459 |
| GZF1 | GDNF inducible zinc finger protein 1 | **1,118** | 0,00052 | 28,138 | 27,519 |
| NR2C2 | nuclear receptor subfamily 2 group C member 2 | **1,117** | 0,001331 | 76,161 | 75,981 |
| ZBTB44 | zinc finger and BTB domain containing 44 | **1,117** | 0,003755 | 27,806 | 33,649 |
| UCP2 | uncoupling protein 2 | **1,117** | 0,018153 | 13,294 | 7,981 |
| CRACR2B | calcium release activated channel regulator 2B | **1,117** | 0,245413 | 1,385 | 2,490 |
| TAOK3 | TAO kinase 3 | **1,117** | 0,001366 | 23,929 | 24,391 |
| MINPP1 | multiple inositol-polyphosphate phosphatase 1 | **1,117** | 0,001568 | 48,023 | 60,083 |
| UBAP1L | ubiquitin associated protein 1 like | **1,117** | 0,221533 | 2,382 | 3,576 |
| KLF2 | Kruppel like factor 2 | **1,117** | 0,006194 | 23,652 | 37,097 |
| FOXP4 | forkhead box P4 | **1,117** | 0,000274 | 44,201 | 54,209 |
| HSF4 | heat shock transcription factor 4 | **1,117** | 0,252511 | 1,163 | 1,149 |
| INTU | inturned planar cell polarity protein | **1,117** | 0,022705 | 8,641 | 15,388 |
| ZNF566 | zinc finger protein 566 | **1,117** | 0,01602 | 8,364 | 9,067 |
| SCUBE1 | signal peptide, CUB domain and EGF like domain containing 1 | **1,117** | 0,001125 | 50,017 | 24,135 |
| FBXO36 | F-box protein 36 | **1,117** | 0,065388 | 3,046 | 5,108 |
| ZBTB49 | zinc finger and BTB domain containing 49 | **1,116** | 0,030402 | 4,099 | 7,279 |
| SOCS2 | suppressor of cytokine signaling 2 | **1,116** | 0,002105 | 23,319 | 14,239 |
| OGDH | oxoglutarate dehydrogenase | **1,116** | 4,40E-05 | 185,834 | 209,045 |
| MGAT5 | mannosyl (alpha-1,6-)-glycoprotein beta-1,6-N-acetyl-glucosaminyltransferase | **1,116** | 0,000697 | 73,447 | 78,408 |
| ICA1 | islet cell autoantigen 1 | **1,116** | 0,084905 | 3,379 | 2,235 |
| PCDHGB5 | protocadherin gamma subfamily B, 5 | **1,116** | 0,040127 | 9,859 | 7,790 |
| CCDC168 | coiled-coil domain containing 168 | **1,116** | 0,485778 | 0,388 | 1,149 |
| ZNF529-AS1 | ZNF529 antisense RNA 1 | **1,116** | 0,409134 | 1,440 | 0,766 |
| RNF213 | ring finger protein 213 | **1,116** | 0,025635 | 109,673 | 103,884 |
| ZDHHC11 | zinc finger DHHC-type containing 11 | **1,116** | 0,385858 | 1,052 | 1,405 |
| EML2-AS1 | EML2 antisense RNA 1 | **1,116** | 0,385699 | 0,388 | 0,511 |

| | | | | | |
|---|---|---|---|---|---|
| SEPT7P2 | septin 7 pseudogene 2 | **1,116** | 0,137925 | 1,883 | 2,618 |
| ITGAM | integrin subunit alpha M | **1,116** | 0,154314 | 1,994 | 3,895 |
| SOBP | sine oculis binding protein homolog | **1,116** | 0,001025 | 26,975 | 28,541 |
| UBR4 | ubiquitin protein ligase E3 component n-recognin 4 | **1,116** | 0,046177 | 210,538 | 228,646 |
| DPY19L2 | dpy-19 like 2 | **1,116** | 0,333867 | 1,052 | 2,554 |
| SLC16A9 | solute carrier family 16 member 9 | **1,116** | 0,281183 | 1,163 | 2,809 |
| HELLS | helicase, lymphoid-specific | **1,116** | 0,251121 | 2,382 | 1,532 |
| CITED4 | Cbp/p300 interacting transactivator with Glu/Asp rich carboxy-terminal domain 4 | **1,115** | 0,003745 | 47,636 | 35,564 |
| FEM1B | fem-1 homolog B | **1,115** | 0,000241 | 58,991 | 56,316 |
| FYN | FYN proto-oncogene, Src family tyrosine kinase | **1,115** | 0,001089 | 41,930 | 34,096 |
| SLC36A1 | solute carrier family 36 member 1 | **1,115** | 0,004916 | 16,340 | 18,389 |
| AP3M2 | adaptor related protein complex 3 mu 2 subunit | **1,115** | 0,000476 | 16,285 | 17,239 |
| CAMTA1 | calmodulin binding transcription activator 1 | **1,115** | 0,000173 | 17,559 | 19,985 |
| FAM102A | family with sequence similarity 102 member A | **1,115** | 0,000401 | 30,188 | 25,476 |
| BMT2 | base methyltransferase of 25S rRNA 2 homolog | **1,115** | 0,004946 | 12,851 | 10,982 |
| C15orf62 | chromosome 15 open reading frame 62 | **1,115** | 0,36381 | 0,499 | 1,149 |
| PHF13 | PHD finger protein 13 | **1,115** | 0,000277 | 19,664 | 18,580 |
| RBPMS2 | RNA binding protein with multiple splicing 2 | **1,115** | 0,012416 | 11,577 | 12,515 |
| RSRC1 | arginine and serine rich coiled-coil 1 | **1,115** | 0,013621 | 11,466 | 10,152 |
| PLS1 | plastin 1 | **1,115** | 0,188134 | 1,329 | 2,362 |
| NPR3 | natriuretic peptide receptor 3 | **1,115** | 0,413339 | 0,942 | 2,171 |
| SARAF | store-operated calcium entry associated regulatory factor | **1,115** | 6,60E-05 | 208,599 | 205,980 |
| ALPK1 | alpha kinase 1 | **1,115** | 0,001678 | 18,556 | 22,986 |
| IGF2BP2 | insulin like growth factor 2 mRNA binding protein 2 | **1,114** | 0,020926 | 10,635 | 10,344 |
| RBM14 | RNA binding motif protein 14 | **1,114** | 0,000338 | 61,982 | 75,279 |
| EIF4EBP2 | eukaryotic translation initiation factor 4E binding protein 2 | **1,114** | 7,30E-05 | 121,360 | 147,238 |
| TMEM170A | transmembrane protein 170A | **1,114** | 0,001366 | 15,897 | 17,942 |
| KMT2E-AS1 | KMT2E antisense RNA 1 (head to head) | **1,114** | 0,176378 | 2,770 | 3,065 |
| ZNF675 | zinc finger protein 675 | **1,114** | 0,13072 | 2,936 | 2,937 |
| AIDA | axin interactor, dorsalization associated | **1,114** | 0,012586 | 12,851 | 11,365 |
| NECTIN1 | nectin cell adhesion molecule 1 | **1,114** | 0,002093 | 32,957 | 50,569 |
| FAM89B | family with sequence similarity 89 member B | **1,114** | 0,058649 | 5,428 | 5,044 |
| GLIDR | glioblastoma down-regulated RNA | **1,114** | 0,372886 | 1,496 | 1,213 |

| | | | | | |
|---|---|---|---|---|---|
| TICAM2 | toll like receptor adaptor molecule 2 | **1,114** | 0,535161 | 0,499 | 0,319 |
| KIAA1462 | KIAA1462 | **1,114** | 0,003222 | 28,803 | 38,821 |
| CMTM6 | CKLF like MARVEL transmembrane domain containing 6 | **1,114** | 4,60E-05 | 47,082 | 57,337 |
| BTN2A2 | butyrophilin subfamily 2 member A2 | **1,114** | 0,003947 | 14,900 | 10,854 |
| TNFSF13B | TNF superfamily member 13b | **1,114** | 0,45668 | 0,443 | 0,830 |
| JADE2 | jade family PHD finger 2 | **1,114** | 0,003179 | 29,468 | 38,246 |
| TNFSF9 | TNF superfamily member 9 | **1,114** | 0,026024 | 6,204 | 7,470 |
| OGT | O-linked N-acetylglucosamine (GlcNAc) transferase | **1,114** | 0,003321 | 119,864 | 130,509 |
| KIF24 | kinesin family member 24 | **1,114** | 0,26696 | 2,160 | 1,852 |
| DNM1P47 | dynamin 1 pseudogene 47 | **1,114** | 0,310303 | 0,886 | 1,596 |
| GATS | GATS, stromal antigen 3 opposite strand | **1,113** | 0,100101 | 4,764 | 6,002 |
| CTNNB1 | catenin beta 1 | **1,113** | 2,10E-05 | 214,194 | 231,264 |
| WNT4 | Wnt family member 4 | **1,113** | 0,561259 | 0,332 | 0,447 |
| CCDC69 | coiled-coil domain containing 69 | **1,113** | 0,402821 | 0,775 | 1,596 |
| MKNK2 | MAP kinase interacting serine/threonine kinase 2 | **1,113** | 0,000633 | 115,710 | 178,716 |
| ZNF79 | zinc finger protein 79 | **1,113** | 0,057188 | 7,145 | 9,194 |
| RIMKLA | ribosomal modification protein rimK like family member A | **1,113** | 0,018298 | 7,644 | 7,854 |
| C1orf50 | chromosome 1 open reading frame 50 | **1,113** | 0,085547 | 2,714 | 3,256 |
| GCH1 | GTP cyclohydrolase 1 | **1,113** | 4,90E-05 | 248,480 | 303,925 |
| ZIC4 | Zic family member 4 | **1,113** | 0,549262 | 0,277 | 0,192 |
| TP53TG1 | TP53 target 1 (non-protein coding) | **1,113** | 0,241165 | 1,662 | 3,512 |
| SVIP | small VCP interacting protein | **1,113** | 0,035293 | 11,909 | 18,261 |
| PTPDC1 | protein tyrosine phosphatase domain containing 1 | **1,113** | 0,016708 | 9,804 | 10,216 |
| DYNLT3 | dynein light chain Tctex-type 3 | **1,113** | 0,001233 | 58,769 | 58,614 |
| SLK | STE20 like kinase | **1,113** | 0,000586 | 88,237 | 91,816 |
| KCNC4 | potassium voltage-gated channel subfamily C member 4 | **1,113** | 0,038125 | 14,845 | 12,195 |
| C12orf76 | chromosome 12 open reading frame 76 | **1,112** | 0,093291 | 4,431 | 4,023 |
| LRRFIP1 | LRR binding FLII interacting protein 1 | **1,112** | 0,000534 | 149,166 | 173,416 |
| LAMA3 | laminin subunit alpha 3 | **1,112** | 0,54146 | 0,388 | 0,766 |
| CREB1 | cAMP responsive element binding protein 1 | **1,112** | 0,003042 | 44,534 | 53,378 |
| MINDY3 | MINDY lysine 48 deubiquitinase 3 | **1,112** | 0,000897 | 21,935 | 20,496 |
| ALOX12-AS1 | ALOX12 antisense RNA 1 | **1,112** | 0,147304 | 3,767 | 4,278 |
| KIAA1755 | KIAA1755 | **1,112** | 0,000338 | 30,077 | 50,314 |

| | | | | | |
|---|---|---|---|---|---|
| OCSTAMP | osteoclast stimulatory transmembrane protein | **1,112** | 0,423834 | 0,775 | 1,277 |
| SERPINB1 | serpin family B member 1 | **1,112** | 0,000389 | 44,644 | 52,293 |
| RELL2 | RELT like 2 | **1,112** | 0,194692 | 2,991 | 2,171 |
| ARHGAP45 | Rho GTPase activating protein 45 | **1,112** | 0,252848 | 0,554 | 0,383 |
| B4GALNT3 | beta-1,4-N-acetyl-galactosaminyltransferase 3 | **1,112** | 0,000241 | 50,017 | 76,748 |
| EIF4G3 | eukaryotic translation initiation factor 4 gamma 3 | **1,112** | 0,001766 | 100,367 | 91,880 |
| SLC25A30 | solute carrier family 25 member 30 | **1,112** | 0,012639 | 14,124 | 15,899 |
| TMEM51 | transmembrane protein 51 | **1,112** | 0,233517 | 2,216 | 1,085 |
| ZNF449 | zinc finger protein 449 | **1,112** | 0,037531 | 11,521 | 9,131 |
| MFSD14A | major facilitator superfamily domain containing 14A | **1,112** | 0,338688 | 1,274 | 1,788 |
| CRIP2 | cysteine rich protein 2 | **1,112** | 0,003426 | 36,779 | 26,817 |
| FHL1 | four and a half LIM domains 1 | **1,112** | 0,00176 | 32,126 | 30,648 |
| AMTN | amelotin | **1,112** | 0,05551 | 62,314 | 44,312 |
| ATP6V1H | ATPase H+ transporting V1 subunit H | **1,112** | 0,00011 | 61,428 | 59,572 |
| ETS2 | ETS proto-oncogene 2, transcription factor | **1,112** | 0,000377 | 70,456 | 88,113 |
| RREB1 | ras responsive element binding protein 1 | **1,112** | 0,004323 | 28,969 | 35,692 |
| CEP152 | centrosomal protein 152 | **1,112** | 0,086373 | 3,988 | 3,895 |
| RNF138 | ring finger protein 138 | **1,112** | 0,012239 | 8,032 | 8,364 |
| CATSPER3 | cation channel sperm associated 3 | **1,111** | 0,537027 | 0,111 | 0,575 |
| SPTBN1 | spectrin beta, non-erythrocytic 1 | **1,111** | 0,00997 | 190,542 | 207,576 |
| PRR16 | proline rich 16 | **1,111** | 0,569926 | 0,609 | 0,511 |
| SH2D4A | SH2 domain containing 4A | **1,111** | 6,50E-05 | 36,114 | 43,993 |
| STXBP1 | syntaxin binding protein 1 | **1,111** | 0,001153 | 86,021 | 105,416 |
| BHLHB9 | basic helix-loop-helix family member b9 | **1,111** | 0,035664 | 5,816 | 6,257 |
| DMXL1 | Dmx like 1 | **1,111** | 0,012245 | 30,686 | 34,160 |
| USP51 | ubiquitin specific peptidase 51 | **1,111** | 0,129105 | 4,487 | 5,172 |
| SOX9 | SRY-box 9 | **1,111** | 0,000222 | 309,243 | 295,370 |
| ITGB2-AS1 | ITGB2 antisense RNA 1 | **1,111** | 0,141733 | 2,493 | 3,129 |
| HAPLN3 | hyaluronan and proteoglycan link protein 3 | **1,111** | 0,002044 | 24,704 | 25,029 |
| SLC25A19 | solute carrier family 25 member 19 | **1,111** | 0,050078 | 5,041 | 6,385 |
| CDKN2A | cyclin dependent kinase inhibitor 2A | **1,111** | 0,050814 | 3,933 | 4,789 |
| GTF2IP20 | general transcription factor IIi pseudogene 20 | **1,111** | 0,12344 | 3,711 | 4,086 |
| SENP7 | SUMO1/sentrin specific peptidase 7 | **1,111** | 0,058605 | 10,413 | 9,450 |

| | | | | | |
|---|---|---|---|---|---|
| RNPC3 | RNA binding region (RNP1, RRM) containing 3 | **1,111** | 0,040578 | 5,594 | 10,152 |
| SPIRE1 | spire type actin nucleation factor 1 | **1,111** | 0,002294 | 49,685 | 44,823 |
| CCNT2 | cyclin T2 | **1,111** | 0,002706 | 26,698 | 34,287 |
| N4BP2L2 | NEDD4 binding protein 2 like 2 | **1,111** | 0,001031 | 43,758 | 49,994 |
| HMGCL | 3-hydroxymethyl-3-methylglutaryl-CoA lyase | **1,111** | 0,000543 | 37,998 | 42,460 |
| FAM76B | family with sequence similarity 76 member B | **1,111** | 0,029378 | 11,687 | 13,153 |
| SNX7 | sorting nexin 7 | **1,111** | 0,014139 | 16,949 | 10,918 |
| SERPINB9P1 | serpin family B member 9 pseudogene 1 | **1,111** | 0,553995 | 0,443 | 0,447 |
| SYTL4 | synaptotagmin like 4 | **1,110** | 0,028605 | 5,428 | 5,427 |
| ZKSCAN1 | zinc finger with KRAB and SCAN domains 1 | **1,110** | 0,002631 | 77,491 | 82,494 |
| VAMP4 | vesicle associated membrane protein 4 | **1,110** | 0,029235 | 8,696 | 9,322 |
| ZSCAN26 | zinc finger and SCAN domain containing 26 | **1,110** | 0,024044 | 13,792 | 15,069 |
| VSIG10L | V-set and immunoglobulin domain containing 10 like | **1,110** | 0,277375 | 1,329 | 0,958 |
| JHDM1D-AS1 | JHDM1D antisense RNA 1 (head to head) | **1,110** | 0,000219 | 134,266 | 270,340 |
| UNC13D | unc-13 homolog D | **1,110** | 0,050994 | 3,157 | 5,363 |
| TXNIP | thioredoxin interacting protein | **1,110** | 2,10E-05 | 224,441 | 317,015 |
| RCOR1 | REST corepressor 1 | **1,110** | 0,001767 | 29,191 | 28,669 |
| MAP3K14 | mitogen-activated protein kinase kinase kinase 14 | **1,110** | 0,025703 | 6,979 | 7,917 |
| VPS41 | VPS41, HOPS complex subunit | **1,110** | 0,001525 | 31,295 | 33,266 |
| ARHGAP22 | Rho GTPase activating protein 22 | **1,110** | 0,00061 | 27,252 | 21,517 |
| NOS3 | nitric oxide synthase 3 | **1,110** | 0,523493 | 0,609 | 0,830 |
| LVRN | laeverin | **1,110** | 0,21525 | 1,828 | 1,852 |
| ZNF682 | zinc finger protein 682 | **1,110** | 0,362191 | 0,942 | 1,788 |
| ARL10 | ADP ribosylation factor like GTPase 10 | **1,110** | 0,028578 | 27,141 | 21,581 |
| KMT2E | lysine methyltransferase 2E | **1,110** | 0,009323 | 161,573 | 212,748 |
| HCN2 | hyperpolarization activated cyclic nucleotide gated potassium channel 2 | **1,110** | 0,576297 | 0,222 | 0,447 |
| PROSER2-AS1 | PROSER2 antisense RNA 1 | **1,110** | 0,648518 | 0,111 | 0,064 |
| PAOX | polyamine oxidase | **1,110** | 0,189906 | 2,770 | 2,107 |
| ALDH4A1 | aldehyde dehydrogenase 4 family member A1 | **1,110** | 0,066475 | 6,038 | 7,343 |
| TPD52 | tumor protein D52 | **1,110** | 0,007321 | 21,935 | 21,134 |
| RASA2 | RAS p21 protein activator 2 | **1,110** | 0,011837 | 11,355 | 13,025 |
| BRCA1 | BRCA1, DNA repair associated | **1,109** | 0,079295 | 6,591 | 5,108 |
| KIFC2 | kinesin family member C2 | **1,109** | 0,23925 | 3,213 | 1,788 |

| | | | | | |
|---|---|---|---|---|---|
| HHIPL2 | HHIP like 2 | **1,109** | 0,018729 | 11,853 | 12,387 |
| HMCN2 | hemicentin 2 | **1,109** | 0,425402 | 0,332 | 0,702 |
| CD58 | CD58 molecule | **1,109** | 0,018018 | 16,285 | 19,027 |
| HRAT92 | heart tissue-associated transcript 92 | **1,109** | 0,474058 | 0,720 | 1,149 |
| CCDC18-AS1 | CCDC18 antisense RNA 1 | **1,109** | 0,056715 | 5,761 | 6,640 |
| CADM1 | cell adhesion molecule 1 | **1,109** | 0,106327 | 4,708 | 5,810 |
| TMEM132E | transmembrane protein 132E | **1,109** | 0,419555 | 0,222 | 0,766 |
| PLD4 | phospholipase D family member 4 | **1,109** | 0,40396 | 0,443 | 1,469 |
| NREP | neuronal regeneration related protein | **1,109** | 0,016118 | 17,226 | 10,471 |
| BDNF | brain derived neurotrophic factor | **1,109** | 0,100255 | 4,653 | 6,193 |
| USF2 | upstream transcription factor 2, c-fos interacting | **1,109** | 0,00269 | 56,609 | 43,035 |
| IRS2 | insulin receptor substrate 2 | **1,109** | 0,000483 | 125,846 | 92,518 |
| RPS6KA6 | ribosomal protein S6 kinase A6 | **1,109** | 0,140776 | 3,767 | 4,214 |
| CDH23 | cadherin related 23 | **1,109** | 0,007095 | 11,521 | 22,922 |
| CNPPD1 | cyclin Pas1/PHO80 domain containing 1 | **1,109** | 0,000995 | 47,580 | 48,654 |
| PPM1B | protein phosphatase, Mg2+/Mn2+ dependent 1B | **1,109** | 0,002223 | 29,357 | 34,479 |
| NECTIN3 | nectin cell adhesion molecule 3 | **1,108** | 0,001148 | 28,194 | 30,712 |
| PPP1R2 | protein phosphatase 1 regulatory inhibitor subunit 2 | **1,108** | 8,00E-05 | 36,281 | 40,800 |
| ZNF141 | zinc finger protein 141 | **1,108** | 0,041201 | 4,210 | 6,321 |
| ZNF135 | zinc finger protein 135 | **1,108** | 0,114399 | 3,490 | 3,576 |
| SNTB2 | syntrophin beta 2 | **1,108** | 0,010259 | 16,063 | 15,452 |
| KLHL7-AS1 | KLHL7 antisense RNA 1 (head to head) | **1,108** | 0,622467 | 1,772 | 0,511 |
| PLCD4 | phospholipase C delta 4 | **1,108** | 0,067787 | 4,099 | 4,086 |
| RPL10P16 | ribosomal protein L10 pseudogene 16 | **1,108** | 0,569933 | 0,111 | 0,192 |
| CPEB3 | cytoplasmic polyadenylation element binding protein 3 | **1,108** | 0,066886 | 4,930 | 8,875 |
| BTAF1 | B-TFIID TATA-box binding protein associated factor 1 | **1,108** | 0,01223 | 35,560 | 41,502 |
| ALG1L9P | asparagine-linked glycosylation 1-like 9, pseudogene | **1,108** | 0,613641 | 0,222 | 0,638 |
| ZNF419 | zinc finger protein 419 | **1,108** | 0,151409 | 5,041 | 5,363 |
| SDK2 | sidekick cell adhesion molecule 2 | **1,108** | 0,00985 | 73,558 | 73,044 |
| LRP3 | LDL receptor related protein 3 | **1,108** | 0,010442 | 11,466 | 7,279 |
| CGRRF1 | cell growth regulator with ring finger domain 1 | **1,108** | 0,004125 | 14,734 | 18,900 |
| SSSCA1-AS1 | SSSCA1 antisense RNA 1 (head to head) | **1,108** | 0,249235 | 1,385 | 2,618 |
| MT-ND2 | mitochondrially encoded NADH:ubiquinone oxidoreductase core subunit 2 | **1,108** | 0,121638 | 546,479 | 812,043 |

| | | | | | |
|---|---|---|---|---|---|
| PLA2G6 | phospholipase A2 group VI | **1,108** | 0,048092 | 5,373 | 6,321 |
| PI4K2A | phosphatidylinositol 4-kinase type 2 alpha | **1,108** | 0,000425 | 46,417 | 48,271 |
| NAPB | NSF attachment protein beta | **1,108** | 0,01329 | 6,924 | 8,237 |
| EZH1 | enhancer of zeste 1 polycomb repressive complex 2 subunit | **1,108** | 3,40E-05 | 39,382 | 44,950 |
| SLC30A4 | solute carrier family 30 member 4 | **1,108** | 0,201145 | 4,708 | 5,108 |
| RFX8 | RFX family member 8, lacking RFX DNA binding domain | **1,108** | 0,032097 | 4,930 | 10,280 |
| PIP4K2C | phosphatidylinositol-5-phosphate 4-kinase type 2 gamma | **1,108** | 0,000764 | 35,505 | 35,054 |
| ARPP19 | cAMP regulated phosphoprotein 19 | **1,108** | 0,001065 | 79,651 | 71,001 |
| RPL23AP7 | ribosomal protein L23a pseudogene 7 | **1,108** | 0,23476 | 1,717 | 2,235 |
| ETV7 | ETS variant 7 | **1,108** | 0,575035 | 0,554 | 0,128 |
| TRIM28 | tripartite motif containing 28 | **1,108** | 0,000108 | 154,040 | 164,477 |
| PPP2R5A | protein phosphatase 2 regulatory subunit B'alpha | **1,108** | 0,001291 | 38,330 | 36,011 |
| SLC16A5 | solute carrier family 16 member 5 | **1,107** | 0,302401 | 0,942 | 1,532 |
| FAM129A | family with sequence similarity 129 member A | **1,107** | 0,005521 | 136,703 | 133,574 |
| REEP6 | receptor accessory protein 6 | **1,107** | 0,157431 | 3,877 | 3,448 |
| CLDN15 | claudin 15 | **1,107** | 0,048178 | 4,043 | 4,725 |
| GALNT7 | polypeptide N-acetylgalactosaminyltransferase 7 | **1,107** | 0,005005 | 42,152 | 32,947 |
| GTF2B | general transcription factor IIB | **1,107** | 0,000302 | 29,191 | 34,990 |
| TRPC1 | transient receptor potential cation channel subfamily C member 1 | **1,107** | 0,044885 | 8,918 | 7,662 |
| CARD6 | caspase recruitment domain family member 6 | **1,107** | 0,088468 | 5,373 | 4,725 |
| NRF1 | nuclear respiratory factor 1 | **1,107** | 0,01071 | 12,684 | 13,728 |
| LINC01772 | long intergenic non-protein coding RNA 1772 | **1,107** | 0,414566 | 0,222 | 0,958 |
| CD164 | CD164 molecule | **1,107** | 0,000643 | 257,453 | 226,731 |
| GNAZ | G protein subunit alpha z | **1,107** | 0,114727 | 3,490 | 3,129 |
| CRTC3 | CREB regulated transcription coactivator 3 | **1,107** | 0,026971 | 22,987 | 20,496 |
| BICD1 | BICD cargo adaptor 1 | **1,107** | 0,019373 | 12,795 | 16,154 |
| PEAK1 | pseudopodium enriched atypical kinase 1 | **1,107** | 0,042522 | 31,849 | 36,586 |
| MEF2C | myocyte enhancer factor 2C | **1,107** | 0,021282 | 8,862 | 9,577 |
| ABHD5 | abhydrolase domain containing 5 | **1,107** | 0,008713 | 11,410 | 11,046 |
| CDK6 | cyclin dependent kinase 6 | **1,107** | 0,054807 | 31,295 | 29,562 |
| FRS2 | fibroblast growth factor receptor substrate 2 | **1,107** | 0,016792 | 21,436 | 22,986 |
| RPL23AP53 | ribosomal protein L23a pseudogene 53 | **1,107** | 0,255201 | 1,440 | 1,277 |
| WDFY3 | WD repeat and FYVE domain containing 3 | **1,107** | 0,042719 | 47,580 | 58,423 |

| | | | | | |
|---|---|---|---|---|---|
| TRNP1 | TMF1-regulated nuclear protein 1 | **1,107** | 0,032745 | 8,364 | 11,110 |
| ZNF81 | zinc finger protein 81 | **1,107** | 0,197334 | 5,207 | 6,002 |
| RETSAT | retinol saturase | **1,107** | 0,000947 | 34,010 | 34,032 |
| CCNL1 | cyclin L1 | **1,106** | 0,004303 | 37,388 | 48,654 |
| MINDY2 | MINDY lysine 48 deubiquitinase 2 | **1,106** | 0,000974 | 43,038 | 46,483 |
| EGFLAM | EGF like, fibronectin type III and laminin G domains | **1,106** | 0,013804 | 18,168 | 13,025 |
| YPEL5 | yippee like 5 | **1,106** | 8,00E-05 | 95,659 | 98,520 |
| PKD1P5 | polycystin 1, transient receptor potential channel interacting pseudogene 5 | **1,106** | 0,328421 | 0,000 | 0,064 |
| LINC00630 | long intergenic non-protein coding RNA 630 | **1,106** | 0,285122 | 1,662 | 2,746 |
| HEATR5B | HEAT repeat containing 5B | **1,106** | 0,052307 | 22,322 | 27,264 |
| CLCN6 | chloride voltage-gated channel 6 | **1,106** | 0,006142 | 28,027 | 36,458 |
| PRRC2C | proline rich coiled-coil 2C | **1,106** | 0,009798 | 236,738 | 273,022 |
| COL17A1 | collagen type XVII alpha 1 chain | **1,106** | 0,463814 | 0,831 | 0,830 |
| GPSM2 | G protein signaling modulator 2 | **1,106** | 0,014483 | 16,174 | 14,047 |
| SH3PXD2A | SH3 and PX domains 2A | **1,106** | 0,075663 | 16,783 | 16,154 |
| DICER1 | dicer 1, ribonuclease III | **1,106** | 0,008241 | 54,892 | 55,549 |
| CCDC163 | coiled-coil domain containing 163 | **1,106** | 0,063955 | 3,822 | 4,853 |
| G0S2 | G0/G1 switch 2 | **1,106** | 0,004984 | 849,076 | 999,187 |
| C4BPA | complement component 4 binding protein alpha | **1,106** | 0,385836 | 0,277 | 0,319 |
| PDE4B | phosphodiesterase 4B | **1,106** | 0,000762 | 341,979 | 413,939 |
| SNX18 | sorting nexin 18 | **1,106** | 0,000749 | 33,456 | 37,352 |
| ZNF778 | zinc finger protein 778 | **1,105** | 0,006318 | 12,463 | 15,899 |
| RNF185 | ring finger protein 185 | **1,105** | 0,001012 | 39,493 | 38,246 |
| CDYL2 | chromodomain Y like 2 | **1,105** | 0,285004 | 3,933 | 6,449 |
| C16orf72 | chromosome 16 open reading frame 72 | **1,105** | 0,001406 | 68,407 | 62,828 |
| ZNF134 | zinc finger protein 134 | **1,105** | 0,015403 | 12,241 | 11,110 |
| S100A9 | S100 calcium binding protein A9 | **1,105** | 0,064701 | 7,312 | 16,856 |
| JPT1 | Jupiter microtubule associated homolog 1 | **1,105** | 0,032541 | 11,632 | 12,770 |
| UBE2V1 | ubiquitin conjugating enzyme E2 V1 | **1,105** | 0,509757 | 0,720 | 1,085 |
| NIPAL3 | NIPA like domain containing 3 | **1,105** | 0,001765 | 27,695 | 27,008 |
| ZNF12 | zinc finger protein 12 | **1,105** | 0,011695 | 25,313 | 25,923 |
| FRMD4A | FERM domain containing 4A | **1,105** | 0,043384 | 14,124 | 9,386 |
| MAPKAPK5-AS1 | MAPKAPK5 antisense RNA 1 | **1,105** | 0,05802 | 3,600 | 4,023 |

| | | | | | |
|---|---|---|---|---|---|
| CTSB | cathepsin B | **1,105** | 0,000495 | 175,642 | 122,464 |
| MBTD1 | mbt domain containing 1 | **1,105** | 0,026561 | 8,475 | 10,471 |
| PRPF39 | pre-mRNA processing factor 39 | **1,105** | 0,002451 | 13,848 | 15,962 |
| TCEANC | transcription elongation factor A N-terminal and central domain containing | **1,105** | 0,153119 | 2,603 | 2,746 |
| CASP3 | caspase 3 | **1,105** | 0,001326 | 19,719 | 17,623 |
| FUT1 | fucosyltransferase 1 (H blood group) | **1,105** | 0,652986 | 0,388 | 0,319 |
| ZMYM5 | zinc finger MYM-type containing 5 | **1,105** | 0,031815 | 9,195 | 10,727 |
| HSP90B2P | heat shock protein 90 beta family member 2, pseudogene | **1,105** | 0,390504 | 0,942 | 0,958 |
| GPS2 | G protein pathway suppressor 2 | **1,105** | 0,334881 | 2,105 | 3,703 |
| IFIT1 | interferon induced protein with tetratricopeptide repeats 1 | **1,105** | 0,16132 | 2,936 | 4,023 |
| CRYAB | crystallin alpha B | **1,105** | 0,012414 | 15,177 | 17,112 |
| BAZ2B | bromodomain adjacent to zinc finger domain 2B | **1,104** | 0,025842 | 15,509 | 20,049 |
| APOL2 | apolipoprotein L2 | **1,104** | 0,000373 | 54,338 | 57,720 |
| FBXO34 | F-box protein 34 | **1,104** | 0,004528 | 14,845 | 13,025 |
| TNN | tenascin N | **1,104** | 0,240925 | 2,880 | 3,129 |
| IFIT5 | interferon induced protein with tetratricopeptide repeats 5 | **1,104** | 0,004228 | 20,051 | 20,177 |
| KCTD13 | potassium channel tetramerization domain containing 13 | **1,104** | 0,006051 | 12,961 | 19,219 |
| TLE3 | transducin like enhancer of split 3 | **1,104** | 0,01397 | 23,929 | 38,246 |
| ZNF614 | zinc finger protein 614 | **1,104** | 0,072006 | 6,536 | 7,917 |
| UBTD2 | ubiquitin domain containing 2 | **1,104** | 5,40E-05 | 125,902 | 139,193 |
| SLC43A3 | solute carrier family 43 member 3 | **1,104** | 0,000666 | 99,647 | 102,288 |
| TMEM135 | transmembrane protein 135 | **1,104** | 0,161214 | 5,761 | 4,023 |
| ZNF225 | zinc finger protein 225 | **1,104** | 0,133236 | 4,819 | 3,384 |
| ZNF572 | zinc finger protein 572 | **1,104** | 0,215948 | 1,440 | 2,171 |
| MT-ND3 | mitochondrially encoded NADH:ubiquinone oxidoreductase core subunit 3 | **1,104** | 0,003894 | 261,220 | 300,158 |
| GATA2 | GATA binding protein 2 | **1,104** | 0,545884 | 1,163 | 0,575 |
| CRYZL1 | crystallin zeta like 1 | **1,104** | 0,040795 | 11,687 | 11,748 |
| CCDC85A | coiled-coil domain containing 85A | **1,104** | 0,252604 | 3,822 | 1,852 |
| ABHD12 | abhydrolase domain containing 12 | **1,104** | 0,000372 | 44,755 | 48,781 |
| LINC01128 | long intergenic non-protein coding RNA 1128 | **1,104** | 0,008855 | 15,731 | 12,770 |
| KCNQ5 | potassium voltage-gated channel subfamily Q member 5 | **1,104** | 0,009134 | 71,952 | 79,876 |
| ABCC2 | ATP binding cassette subfamily C member 2 | **1,103** | 0,307661 | 1,052 | 1,788 |
| XPR1 | xenotropic and polytropic retrovirus receptor 1 | **1,103** | 0,003028 | 39,604 | 50,888 |

| | | | | | |
|---|---|---|---|---|---|
| ZNF606 | zinc finger protein 606 | **1,103** | 0,074474 | 5,317 | 4,086 |
| MLF1 | myeloid leukemia factor 1 | **1,103** | 0,060386 | 5,317 | 6,768 |
| ERI2 | ERI1 exoribonuclease family member 2 | **1,103** | 0,087801 | 5,927 | 6,002 |
| FCAMR | Fc fragment of IgA and IgM receptor | **1,103** | 0,007943 | 32,569 | 35,117 |
| GLRX2 | glutaredoxin 2 | **1,103** | 0,010054 | 17,337 | 24,008 |
| TSPAN15 | tetraspanin 15 | **1,103** | 0,604591 | 0,277 | 0,255 |
| SERINC1 | serine incorporator 1 | **1,103** | 0,00013 | 511,030 | 505,563 |
| KBTBD2 | kelch repeat and BTB domain containing 2 | **1,103** | 0,000622 | 40,269 | 33,904 |
| ZFR | zinc finger RNA binding protein | **1,103** | 0,001858 | 132,327 | 128,466 |
| ATF7 | activating transcription factor 7 | **1,103** | 0,014196 | 26,366 | 31,159 |
| SNHG12 | small nucleolar RNA host gene 12 | **1,103** | 0,02862 | 7,201 | 12,642 |
| CREB5 | cAMP responsive element binding protein 5 | **1,103** | 0,04285 | 23,707 | 33,330 |
| SDCBP | syndecan binding protein | **1,103** | 3,00E-04 | 304,424 | 331,636 |
| STRN | striatin | **1,103** | 0,036313 | 30,465 | 30,648 |
| TBC1D20 | TBC1 domain family member 20 | **1,103** | 0,000431 | 55,501 | 55,677 |
| ACTR6 | ARP6 actin-related protein 6 homolog | **1,103** | 0,005631 | 16,894 | 20,240 |
| MMP24 | matrix metallopeptidase 24 | **1,103** | 0,438396 | 1,496 | 1,532 |
| ALOX12B | arachidonate 12-lipoxygenase, 12R type | **1,103** | 0,643704 | 0,277 | 0,830 |
| STIL | SCL/TAL1 interrupting locus | **1,103** | 0,339267 | 3,379 | 2,426 |
| VEGFC | vascular endothelial growth factor C | **1,103** | 0,035812 | 12,740 | 11,557 |
| PTPN13 | protein tyrosine phosphatase, non-receptor type 13 | **1,103** | 0,062465 | 13,571 | 15,388 |
| INPP5A | inositol polyphosphate-5-phosphatase A | **1,103** | 0,004262 | 10,856 | 13,472 |
| TMEM202-AS1 | TMEM202 antisense RNA 1 | **1,103** | 0,333545 | 0,886 | 1,469 |
| WDR27 | WD repeat domain 27 | **1,103** | 0,031009 | 8,419 | 15,324 |
| DAB2 | DAB2, clathrin adaptor protein | **1,103** | 0,000759 | 131,053 | 110,780 |
| PAQR3 | progestin and adipoQ receptor family member 3 | **1,102** | 0,009548 | 10,967 | 13,345 |
| LINC02001 | long intergenic non-protein coding RNA 2001 | **1,102** | 0,209279 | 1,883 | 3,448 |
| HNRNPA1P16 | heterogeneous nuclear ribonucleoprotein A1 pseudogene 16 | **1,102** | 0,61372 | 0,499 | 0,575 |
| ABCA4 | ATP binding cassette subfamily A member 4 | **1,102** | 0,2095 | 1,828 | 2,682 |
| ZNF432 | zinc finger protein 432 | **1,102** | 0,05168 | 6,591 | 6,704 |
| TMEM159 | transmembrane protein 159 | **1,102** | 0,112151 | 7,644 | 5,300 |
| CELF2 | CUGBP Elav-like family member 2 | **1,102** | 0,487697 | 0,609 | 0,702 |
| PRKAA2 | protein kinase AMP-activated catalytic subunit alpha 2 | **1,102** | 0,129019 | 2,936 | 5,938 |

| | | | | | |
|---|---|---|---|---|---|
| RALGPS2 | Ral GEF with PH domain and SH3 binding motif 2 | **1,102** | 0,195872 | 2,382 | 2,235 |
| SLC9A3 | solute carrier family 9 member A3 | **1,102** | 0,540419 | 0,499 | 0,702 |
| MAPK1 | mitogen-activated protein kinase 1 | **1,102** | 9,20E-05 | 96,490 | 107,651 |
| FAM53B | family with sequence similarity 53 member B | **1,102** | 0,038184 | 6,813 | 10,280 |
| FAM198A | family with sequence similarity 198 member A | **1,102** | 0,585339 | 0,166 | 0,702 |
| ATAD2B | ATPase family, AAA domain containing 2B | **1,102** | 0,117694 | 7,035 | 9,577 |
| RAB6B | RAB6B, member RAS oncogene family | **1,102** | 0,236564 | 2,493 | 2,682 |
| TBC1D12 | TBC1 domain family member 12 | **1,102** | 0,010888 | 17,171 | 13,153 |
| MGA | MGA, MAX dimerization protein | **1,102** | 0,042934 | 57,606 | 64,744 |
| THBS2 | thrombospondin 2 | **1,102** | 0,039508 | 6,979 | 12,515 |
| GPC1 | glypican 1 | **1,102** | 0,000751 | 166,115 | 77,195 |
| LITAF | lipopolysaccharide induced TNF factor | **1,102** | 0,000202 | 80,593 | 95,264 |
| MIR4697HG | MIR4697 host gene | **1,102** | 0,035899 | 7,145 | 7,279 |
| UBXN7 | UBX domain protein 7 | **1,102** | 0,050318 | 37,942 | 35,501 |
| ETV2 | ETS variant 2 | **1,102** | 0,62234 | 0,443 | 0,000 |
| PGAP1 | post-GPI attachment to proteins 1 | **1,101** | 0,062546 | 9,859 | 11,301 |
| FOXD1 | forkhead box D1 | **1,101** | 0,002307 | 27,474 | 35,628 |
| CACHD1 | cache domain containing 1 | **1,101** | 0,002422 | 43,315 | 49,931 |
| SOS1 | SOS Ras/Rac guanine nucleotide exchange factor 1 | **1,101** | 0,041943 | 30,908 | 30,456 |
| ZNF180 | zinc finger protein 180 | **1,101** | 0,070058 | 6,038 | 5,619 |
| MTHFR | methylenetetrahydrofolate reductase | **1,101** | 0,0019 | 27,474 | 29,116 |
| MORC3 | MORC family CW-type zinc finger 3 | **1,101** | 0,001553 | 57,329 | 67,489 |
| NIPBL | NIPBL, cohesin loading factor | **1,101** | 0,017389 | 61,206 | 76,748 |
| FOXRED2 | FAD dependent oxidoreductase domain containing 2 | **1,101** | 0,015741 | 14,291 | 17,495 |
| THEMIS2 | thymocyte selection associated family member 2 | **1,101** | 0,569599 | 0,332 | 0,447 |
| TLR5 | toll like receptor 5 | **1,101** | 0,495003 | 0,720 | 1,149 |
| LINC01117 | long intergenic non-protein coding RNA 1117 | **1,101** | 0,52535 | 0,665 | 0,958 |
| TCEAL4 | transcription elongation factor A like 4 | **1,101** | 0,031261 | 40,379 | 35,373 |
| ABCA2 | ATP binding cassette subfamily A member 2 | **1,101** | 0,00272 | 30,963 | 26,753 |
| TAF1 | TATA-box binding protein associated factor 1 | **1,101** | 0,031054 | 33,123 | 37,863 |
| UBE2R2 | ubiquitin conjugating enzyme E2 R2 | **1,101** | 0,000265 | 39,493 | 41,949 |
| PCDHGB2 | protocadherin gamma subfamily B, 2 | **1,101** | 0,323348 | 1,274 | 1,405 |
| ROGDI | rogdi homolog | **1,101** | 0,05912 | 6,481 | 10,727 |

| | | | | | |
|---|---|---|---|---|---|
| NID1 | nidogen 1 | **1,101** | 0,275712 | 3,213 | 1,979 |
| APBB1IP | amyloid beta precursor protein binding family B member 1 interacting protein | **1,101** | 0,317796 | 0,720 | 1,788 |
| ZSWIM5 | zinc finger SWIM-type containing 5 | **1,101** | 0,172727 | 3,213 | 3,767 |
| ZNF510 | zinc finger protein 510 | **1,101** | 0,013705 | 9,195 | 12,898 |
| IER5L | immediate early response 5 like | **1,101** | 0,003077 | 51,901 | 57,593 |
| ZNF83 | zinc finger protein 83 | **1,101** | 0,005851 | 17,836 | 22,284 |
| RASD1 | ras related dexamethasone induced 1 | **1,101** | 0,005541 | 43,592 | 35,628 |
| POPDC2 | popeye domain containing 2 | **1,101** | 0,332336 | 0,997 | 0,830 |
| ZNF442 | zinc finger protein 442 | **1,101** | 0,17042 | 3,213 | 3,703 |
| TLR1 | toll like receptor 1 | **1,101** | 0,045892 | 4,874 | 7,279 |
| ARHGAP32 | Rho GTPase activating protein 32 | **1,101** | 0,079595 | 10,580 | 10,280 |
| DIAPH2 | diaphanous related formin 2 | **1,100** | 0,033855 | 11,078 | 6,896 |
| B3GNT8 | UDP-GlcNAc:betaGal beta-1,3-N-acetylglucosaminyltransferase 8 | **1,100** | 0,646964 | 0,332 | 0,064 |
| PDGFD | platelet derived growth factor D | **1,100** | 0,163622 | 3,656 | 7,215 |
| AFTPH | aftiphilin | **1,100** | 0,000613 | 52,288 | 57,529 |
| CTSD | cathepsin D | **1,100** | 0,004124 | 459,018 | 288,027 |
| CLSPN | claspin | **1,100** | 0,151471 | 4,320 | 4,597 |
| TONSL-AS1 | TONSL antisense RNA 1 | **1,100** | 0,578006 | 0,443 | 0,383 |
| CEP295 | centrosomal protein 295 | **1,100** | 0,043462 | 10,690 | 12,195 |
| SP6 | Sp6 transcription factor | **1,100** | 0,057783 | 6,702 | 6,193 |
| HNRNPH3 | heterogeneous nuclear ribonucleoprotein H3 | **1,100** | 0,000282 | 105,851 | 144,875 |
| ZNF383 | zinc finger protein 383 | **1,100** | 0,083783 | 7,699 | 7,087 |
| HMGN2P46 | high mobility group nucleosomal binding domain 2 pseudogene 46 | **1,100** | 0,002494 | 15,731 | 16,090 |
| SPEN | spen family transcriptional repressor | **1,100** | 0,062791 | 106,349 | 138,873 |
| SHISA4 | shisa family member 4 | **1,100** | 0,033561 | 14,124 | 9,705 |
| RETREG2 | reticulophagy regulator family member 2 | **1,100** | 0,00047 | 113,771 | 107,779 |
| ZNF888 | zinc finger protein 888 | **1,100** | 0,31128 | 2,548 | 2,362 |
| KIF3C | kinesin family member 3C | **1,100** | 0,158337 | 6,204 | 4,533 |
| SENP8 | SUMO/sentrin peptidase family member, NEDD8 specific | **1,100** | 0,018914 | 8,309 | 10,982 |
| ZNF791 | zinc finger protein 791 | **1,100** | 0,000739 | 34,231 | 43,993 |
| ASH1L | ASH1 like histone lysine methyltransferase | **1,100** | 0,032986 | 96,157 | 117,165 |
| FAM71F2 | family with sequence similarity 71 member F2 | **1,100** | 0,399013 | 1,163 | 0,575 |
| PAX8 | paired box 8 | **1,100** | 0,015024 | 18,500 | 13,217 |

| | | | | | |
|---|---|---|---|---|---|
| CCDC88A | coiled-coil domain containing 88A | **1,100** | 0,002234 | 58,991 | 54,847 |
| ZNF37BP | zinc finger protein 37B, pseudogene | **1,100** | 0,080285 | 5,927 | 8,364 |
| PPIEL | peptidylprolyl isomerase E like pseudogene | **1,100** | 0,285311 | 2,991 | 3,320 |
| ZBTB11 | zinc finger and BTB domain containing 11 | **1,100** | 0,002314 | 34,453 | 42,588 |
| XIAP | X-linked inhibitor of apoptosis | **1,100** | 0,000378 | 73,724 | 77,450 |
| ZNF354B | zinc finger protein 354B | **1,100** | 0,145979 | 3,490 | 3,831 |
| ZNF430 | zinc finger protein 430 | **1,100** | 0,078835 | 4,985 | 5,300 |
| ORAI3 | ORAI calcium release-activated calcium modulator 3 | **1,100** | 0,090634 | 7,422 | 8,492 |
| POLG | DNA polymerase gamma, catalytic subunit | **1,099** | 0,001051 | 60,818 | 78,791 |
| DSCR3 | DSCR3 arrestin fold containing | **1,099** | 0,000806 | 48,079 | 45,461 |
| TSHZ3 | teashirt zinc finger homeobox 3 | **1,099** | 0,15539 | 6,758 | 3,959 |
| ZC3HAV1 | zinc finger CCCH-type containing, antiviral 1 | **1,099** | 0,000428 | 63,809 | 64,744 |
| NPHP3 | nephrocystin 3 | **1,099** | 0,046786 | 5,761 | 6,768 |
| LRCH1 | leucine rich repeats and calponin homology domain containing 1 | **1,099** | 0,011463 | 21,325 | 22,156 |
| LNPK | lunapark, ER junction formation factor | **1,099** | 0,001182 | 30,575 | 32,436 |
| GTF2IP4 | general transcription factor IIi pseudogene 4 | **1,099** | 0,255823 | 2,382 | 2,682 |
| CBLN1 | cerebellin 1 precursor | **1,099** | 0,462178 | 0,665 | 0,830 |
| CDC7 | cell division cycle 7 | **1,099** | 0,059545 | 9,749 | 9,577 |
| CYBA | cytochrome b-245 alpha chain | **1,099** | 0,017269 | 47,580 | 54,975 |
| RBM12B | RNA binding motif protein 12B | **1,099** | 0,029584 | 18,666 | 24,774 |
| RAB20 | RAB20, member RAS oncogene family | **1,099** | 0,08294 | 4,043 | 3,576 |
| WHRN | whirlin | **1,099** | 0,057065 | 5,207 | 7,662 |
| TEAD1 | TEA domain transcription factor 1 | **1,099** | 0,05459 | 49,962 | 51,399 |
| HOMER2 | homer scaffolding protein 2 | **1,099** | 0,287065 | 2,382 | 2,362 |
| SLC3A2 | solute carrier family 3 member 2 | **1,099** | 0,001215 | 584,477 | 559,963 |
| MED12 | mediator complex subunit 12 | **1,099** | 0,102312 | 30,298 | 35,437 |
| VPS35 | VPS35, retromer complex component | **1,099** | 0,000229 | 76,771 | 79,749 |
| INSR | insulin receptor | **1,099** | 0,00307 | 52,565 | 53,889 |
| OGN | osteoglycin | **1,099** | 0,001285 | 76,438 | 69,277 |
| ZNF112 | zinc finger protein 112 | **1,099** | 0,095525 | 4,265 | 3,831 |
| FAM167B | family with sequence similarity 167 member B | **1,099** | 0,667064 | 0,222 | 0,319 |
| SMAD5 | SMAD family member 5 | **1,099** | 0,002314 | 37,665 | 48,334 |
| NEDD9 | neural precursor cell expressed, developmentally down-regulated 9 | **1,099** | 0,09194 | 7,090 | 10,535 |

| | | | | | |
|---|---|---|---|---|---|
| ATG14 | autophagy related 14 | **1,099** | 0,022762 | 14,124 | 16,154 |
| FMNL2 | formin like 2 | **1,098** | 0,027841 | 31,794 | 38,501 |
| WDR44 | WD repeat domain 44 | **1,098** | 0,002332 | 17,780 | 17,239 |
| ADPRH | ADP-ribosylarginine hydrolase | **1,098** | 0,039946 | 8,530 | 9,194 |
| E2F3 | E2F transcription factor 3 | **1,098** | 0,025263 | 18,390 | 15,707 |
| USP27X | ubiquitin specific peptidase 27, X-linked | **1,098** | 0,158934 | 4,874 | 4,342 |
| CCSER2 | coiled-coil serine rich protein 2 | **1,098** | 0,003488 | 53,064 | 54,975 |
| PHLPP2 | PH domain and leucine rich repeat protein phosphatase 2 | **1,098** | 0,062828 | 9,582 | 10,727 |
| ZFYVE21 | zinc finger FYVE-type containing 21 | **1,098** | 0,00095 | 38,219 | 35,947 |
| NUDT4 | nudix hydrolase 4 | **1,098** | 0,004235 | 23,707 | 28,285 |
| ZMYM2 | zinc finger MYM-type containing 2 | **1,098** | 0,009997 | 36,558 | 36,905 |
| SRC | SRC proto-oncogene, non-receptor tyrosine kinase | **1,098** | 0,00544 | 25,036 | 29,435 |
| GTF2IRD2 | GTF2I repeat domain containing 2 | **1,098** | 0,228262 | 1,994 | 1,469 |
| ZNF214 | zinc finger protein 214 | **1,098** | 0,185014 | 3,600 | 3,831 |
| ZADH2 | zinc binding alcohol dehydrogenase domain containing 2 | **1,098** | 0,005442 | 20,051 | 23,433 |
| PAQR5 | progestin and adipoQ receptor family member 5 | **1,098** | 0,555898 | 0,332 | 0,255 |
| RUNX1 | runt related transcription factor 1 | **1,098** | 0,000495 | 218,847 | 266,956 |
| ENHO | energy homeostasis associated | **1,098** | 0,432792 | 1,163 | 1,277 |
| DMTF1 | cyclin D binding myb like transcription factor 1 | **1,098** | 0,008407 | 27,030 | 34,670 |
| SLC2A8 | solute carrier family 2 member 8 | **1,097** | 0,02342 | 12,906 | 14,239 |
| ANXA2R | annexin A2 receptor | **1,097** | 0,524569 | 0,388 | 0,894 |
| MAP3K12 | mitogen-activated protein kinase kinase kinase 12 | **1,097** | 0,179455 | 3,434 | 3,065 |
| LINC01426 | long intergenic non-protein coding RNA 1426 | **1,097** | 0,001927 | 12,961 | 16,537 |
| PHYHD1 | phytanoyl-CoA dioxygenase domain containing 1 | **1,097** | 0,280358 | 1,551 | 1,852 |
| SCML1 | sex comb on midleg like 1 (Drosophila) | **1,097** | 0,024046 | 10,967 | 12,642 |
| RABGAP1 | RAB GTPase activating protein 1 | **1,097** | 0,009572 | 34,730 | 34,415 |
| JADE3 | jade family PHD finger 3 | **1,097** | 0,373459 | 2,880 | 2,362 |
| MED20 | mediator complex subunit 20 | **1,097** | 0,012956 | 10,635 | 11,174 |
| WDR62 | WD repeat domain 62 | **1,097** | 0,29109 | 3,323 | 1,979 |
| DNASE1 | deoxyribonuclease 1 | **1,097** | 0,053847 | 10,690 | 16,026 |
| ZNF18 | zinc finger protein 18 | **1,097** | 0,064898 | 7,478 | 10,535 |
| OTUD7B | OTU deubiquitinase 7B | **1,097** | 0,002063 | 47,026 | 52,676 |
| ITSN2 | intersectin 2 | **1,097** | 0,003243 | 55,446 | 67,042 |

| | | | | | |
|---|---|---|---|---|---|
| FAM45A | family with sequence similarity 45 member A | **1,097** | 0,110316 | 5,594 | 7,279 |
| ZNF879 | zinc finger protein 879 | **1,097** | 0,143195 | 3,656 | 4,278 |
| NUFIP2 | NUFIP2, FMR1 interacting protein 2 | **1,097** | 0,010564 | 103,524 | 106,565 |
| AFAP1 | actin filament associated protein 1 | **1,097** | 0,018555 | 31,683 | 39,715 |
| COL27A1 | collagen type XXVII alpha 1 chain | **1,097** | 0,030187 | 25,978 | 43,673 |
| GPR35 | G protein-coupled receptor 35 | **1,097** | 0,33604 | 0,997 | 1,469 |
| A2M | alpha-2-macroglobulin | **1,097** | 0,001883 | 357,322 | 196,083 |
| ENOPH1 | enolase-phosphatase 1 | **1,097** | 0,012168 | 27,030 | 27,328 |
| C11orf91 | chromosome 11 open reading frame 91 | **1,097** | 0,554723 | 0,499 | 0,702 |
| KCTD3 | potassium channel tetramerization domain containing 3 | **1,097** | 0,001634 | 29,689 | 20,432 |
| FNDC3A | fibronectin type III domain containing 3A | **1,097** | 0,011745 | 86,630 | 91,816 |
| DIP2A | disco interacting protein 2 homolog A | **1,097** | 0,018983 | 24,095 | 31,478 |
| ANTXR2 | anthrax toxin receptor 2 | **1,097** | 0,009991 | 93,332 | 115,441 |
| MPP7 | membrane palmitoylated protein 7 | **1,097** | 0,167532 | 3,157 | 5,172 |
| LIMK2 | LIM domain kinase 2 | **1,097** | 0,062614 | 10,413 | 9,514 |
| FUS | FUS RNA binding protein | **1,096** | 0,001188 | 244,437 | 375,565 |
| PLXNA2 | plexin A2 | **1,096** | 0,014641 | 30,575 | 44,056 |
| AGO2 | argonaute 2, RISC catalytic component | **1,096** | 0,10486 | 73,780 | 87,857 |
| DNAJB6 | DnaJ heat shock protein family (Hsp40) member B6 | **1,096** | 0,000784 | 95,936 | 97,180 |
| KCP | kielin/chordin-like protein | **1,096** | 0,770534 | 0,499 | 0,638 |
| WARS | tryptophanyl-tRNA synthetase | **1,096** | 0,000186 | 409,001 | 547,002 |
| PLK4 | polo like kinase 4 | **1,096** | 0,584876 | 1,606 | 0,319 |
| TRAPPC8 | trafficking protein particle complex 8 | **1,096** | 0,011746 | 37,111 | 41,183 |
| HUWE1 | HECT, UBA and WWE domain containing 1, E3 ubiquitin protein ligase | **1,096** | 0,03629 | 278,169 | 292,432 |
| GALNT15 | polypeptide N-acetylgalactosaminyltransferase 15 | **1,096** | 0,002047 | 145,565 | 191,358 |
| API5 | apoptosis inhibitor 5 | **1,096** | 0,00114 | 74,001 | 84,346 |
| KCNJ12 | potassium voltage-gated channel subfamily J member 12 | **1,096** | 0,390543 | 2,991 | 2,235 |
| ALDH1L1 | aldehyde dehydrogenase 1 family member L1 | **1,096** | 0,407224 | 0,886 | 1,277 |
| MXD3 | MAX dimerization protein 3 | **1,096** | 0,256489 | 2,603 | 2,937 |
| ZNF137P | zinc finger protein 137, pseudogene | **1,096** | 0,417521 | 1,163 | 1,596 |
| LUZP1 | leucine zipper protein 1 | **1,096** | 0,013627 | 34,896 | 39,268 |
| ZNF397 | zinc finger protein 397 | **1,096** | 0,001938 | 26,643 | 34,798 |
| BBS2 | Bardet-Biedl syndrome 2 | **1,096** | 0,007565 | 19,830 | 24,901 |

| | | | | | |
|---|---|---|---|---|---|
| PGBD2 | piggyBac transposable element derived 2 | **1,096** | 0,084457 | 5,539 | 6,832 |
| ZFP36L2 | ZFP36 ring finger protein like 2 | **1,096** | 0,000545 | 99,370 | 106,565 |
| CXorf57 | chromosome X open reading frame 57 | **1,096** | 0,479558 | 0,942 | 1,022 |
| PALLD | palladin, cytoskeletal associated protein | **1,096** | 0,008246 | 28,581 | 36,267 |
| CD22 | CD22 molecule | **1,095** | 0,456283 | 2,493 | 1,149 |
| PHOSPHO1 | phosphoethanolamine/phosphocholine phosphatase | **1,095** | 0,423463 | 0,222 | 0,575 |
| TNKS1BP1 | tankyrase 1 binding protein 1 | **1,095** | 0,001559 | 254,185 | 339,745 |
| SETD2 | SET domain containing 2 | **1,095** | 0,024826 | 44,977 | 53,762 |
| KREMEN1 | kringle containing transmembrane protein 1 | **1,095** | 0,023217 | 20,550 | 14,941 |
| SPRED2 | sprouty related EVH1 domain containing 2 | **1,095** | 0,015043 | 45,088 | 42,396 |
| TBL1X | transducin beta like 1X-linked | **1,095** | 0,013445 | 47,248 | 57,593 |
| ANGPTL7 | angiopoietin like 7 | **1,095** | 0,254224 | 2,160 | 2,554 |
| KIF3A | kinesin family member 3A | **1,095** | 0,01235 | 18,223 | 24,455 |
| OXR1 | oxidation resistance 1 | **1,095** | 0,008426 | 20,937 | 24,646 |
| LIN52 | lin-52 DREAM MuvB core complex component | **1,095** | 0,006496 | 15,011 | 19,027 |
| SVEP1 | sushi, von Willebrand factor type A, EGF and pentraxin domain containing 1 | **1,095** | 0,121725 | 8,530 | 8,364 |
| TRIM21 | tripartite motif containing 21 | **1,095** | 0,020999 | 12,130 | 14,749 |
| DDIT4 | DNA damage inducible transcript 4 | **1,095** | 0,001669 | 228,651 | 274,874 |
| RNF170 | ring finger protein 170 | **1,095** | 0,017532 | 16,617 | 16,665 |
| SEL1L | SEL1L ERAD E3 ligase adaptor subunit | **1,095** | 0,017997 | 246,985 | 240,586 |
| HOXC-AS2 | HOXC cluster antisense RNA 2 | **1,095** | 0,586985 | 0,554 | 0,319 |
| CNOT4 | CCR4-NOT transcription complex subunit 4 | **1,095** | 0,006757 | 32,514 | 33,649 |
| EIF1 | eukaryotic translation initiation factor 1 | **1,095** | 0,001468 | 755,023 | 941,658 |
| THAP5 | THAP domain containing 5 | **1,095** | 0,00078 | 38,053 | 46,163 |
| PHACTR2 | phosphatase and actin regulator 2 | **1,095** | 0,07427 | 19,054 | 20,751 |
| ZNF529 | zinc finger protein 529 | **1,095** | 0,053011 | 8,253 | 10,663 |
| GPNMB | glycoprotein nmb | **1,095** | 0,036279 | 4,043 | 12,515 |
| RALGAPA2 | Ral GTPase activating protein catalytic alpha subunit 2 | **1,095** | 0,057359 | 10,192 | 10,854 |
| CYP4V2 | cytochrome P450 family 4 subfamily V member 2 | **1,095** | 0,009728 | 19,165 | 33,266 |
| CABYR | calcium binding tyrosine phosphorylation regulated | **1,095** | 0,491918 | 0,831 | 0,638 |
| ZXDA | zinc finger, X-linked, duplicated A | **1,095** | 0,255563 | 3,822 | 2,746 |
| VSTM4 | V-set and transmembrane domain containing 4 | **1,095** | 0,00955 | 33,733 | 30,712 |
| MFHAS1 | malignant fibrous histiocytoma amplified sequence 1 | **1,095** | 0,489335 | 0,997 | 0,894 |

| | | | | | |
|---|---|---|---|---|---|
| MATN3 | matrilin 3 | **1,095** | 0,010209 | 77,989 | 47,696 |
| HERC1 | HECT and RLD domain containing E3 ubiquitin protein ligase family member 1 | **1,095** | 0,050875 | 59,655 | 69,149 |
| SH3BP5-AS1 | SH3BP5 antisense RNA 1 | **1,095** | 0,153431 | 5,982 | 9,003 |
| PSEN1 | presenilin 1 | **1,095** | 0,001047 | 126,123 | 127,572 |
| MAPRE2 | microtubule associated protein RP/EB family member 2 | **1,095** | 0,00287 | 43,592 | 48,143 |
| JADE1 | jade family PHD finger 1 | **1,094** | 0,004157 | 20,882 | 30,137 |
| GALNT9 | polypeptide N-acetylgalactosaminyltransferase 9 | **1,094** | 0,070057 | 15,454 | 15,324 |
| BAHD1 | bromo adjacent homology domain containing 1 | **1,094** | 0,002396 | 35,782 | 33,585 |
| HIPK3 | homeodomain interacting protein kinase 3 | **1,094** | 0,011687 | 72,838 | 85,240 |
| INHBA-AS1 | INHBA antisense RNA 1 | **1,094** | 0,412537 | 0,942 | 1,596 |
| AK4 | adenylate kinase 4 | **1,094** | 0,002302 | 58,658 | 46,738 |
| UVRAG | UV radiation resistance associated | **1,094** | 0,002755 | 17,946 | 20,751 |
| SUSD2 | sushi domain containing 2 | **1,094** | 0,618264 | 1,385 | 0,575 |
| RRAD | RRAD, Ras related glycolysis inhibitor and calcium channel regulator | **1,094** | 0,074735 | 18,777 | 13,600 |
| ZCCHC12 | zinc finger CCHC-type containing 12 | **1,094** | 0,66363 | 0,111 | 0,383 |
| ZNF284 | zinc finger protein 284 | **1,094** | 0,570265 | 0,554 | 0,638 |
| PIKFYVE | phosphoinositide kinase, FYVE-type zinc finger containing | **1,094** | 0,053356 | 35,560 | 43,865 |
| ADGRA3 | adhesion G protein-coupled receptor A3 | **1,094** | 0,022461 | 27,972 | 24,071 |
| MFAP3L | microfibrillar associated protein 3 like | **1,094** | 0,028865 | 16,894 | 15,835 |
| C15orf48 | chromosome 15 open reading frame 48 | **1,094** | 0,003533 | 284,705 | 359,219 |
| MYH10 | myosin heavy chain 10 | **1,094** | 0,054655 | 19,830 | 26,051 |
| IQUB | IQ motif and ubiquitin domain containing | **1,094** | 0,54606 | 0,443 | 0,638 |
| CTCF | CCCTC-binding factor | **1,094** | 0,001987 | 52,122 | 54,209 |
| ASAH1 | N-acylsphingosine amidohydrolase 1 | **1,094** | 0,00504 | 89,123 | 78,344 |
| NCKAP1 | NCK associated protein 1 | **1,094** | 0,00704 | 173,039 | 164,797 |
| DHRS13 | dehydrogenase/reductase 13 | **1,094** | 0,070731 | 6,093 | 8,364 |
| PALM | paralemmin | **1,094** | 0,422612 | 0,499 | 0,383 |
| HS3ST1 | heparan sulfate-glucosamine 3-sulfotransferase 1 | **1,094** | 0,118092 | 3,046 | 5,172 |
| PTAR1 | protein prenyltransferase alpha subunit repeat containing 1 | **1,094** | 0,050651 | 45,641 | 52,293 |
| HIST1H2BK | histone cluster 1 H2B family member k | **1,094** | 0,03077 | 21,658 | 38,118 |
| RBM33 | RNA binding motif protein 33 | **1,094** | 0,03447 | 34,176 | 44,056 |
| COPS8 | COP9 signalosome subunit 8 | **1,094** | 0,002092 | 47,912 | 57,848 |
| ZNF230 | zinc finger protein 230 | **1,094** | 0,253335 | 3,046 | 2,299 |

| | | | | | |
|---|---|---|---|---|---|
| SNHG14 | small nucleolar RNA host gene 14 | **1,093** | 0,074434 | 18,722 | 18,644 |
| RSRC2 | arginine and serine rich coiled-coil 2 | **1,093** | 0,00154 | 50,239 | 65,957 |
| MECP2 | methyl-CpG binding protein 2 | **1,093** | 0,004076 | 70,512 | 79,302 |
| ADNP-AS1 | ADNP antisense RNA 1 | **1,093** | 0,340785 | 1,163 | 1,852 |
| SS18 | SS18, nBAF chromatin remodeling complex subunit | **1,093** | 0,001011 | 74,611 | 84,026 |
| GRM4 | glutamate metabotropic receptor 4 | **1,093** | 0,513508 | 0,499 | 0,638 |
| ZMYND11 | zinc finger MYND-type containing 11 | **1,093** | 0,002717 | 67,133 | 67,489 |
| USP38 | ubiquitin specific peptidase 38 | **1,093** | 0,018792 | 22,488 | 25,476 |
| KATNAL1 | katanin catalytic subunit A1 like 1 | **1,093** | 0,015025 | 22,433 | 16,154 |
| ENTPD1-AS1 | ENTPD1 antisense RNA 1 | **1,093** | 0,340785 | 1,828 | 2,171 |
| PPM1K | protein phosphatase, Mg2+/Mn2+ dependent 1K | **1,093** | 0,037961 | 9,859 | 10,344 |
| MAP7D3 | MAP7 domain containing 3 | **1,093** | 0,008197 | 24,926 | 25,348 |
| SLC9A8 | solute carrier family 9 member A8 | **1,093** | 0,005336 | 35,173 | 48,271 |
| SNX2 | sorting nexin 2 | **1,093** | 0,005355 | 38,108 | 41,822 |
| NR3C1 | nuclear receptor subfamily 3 group C member 1 | **1,093** | 0,005011 | 272,132 | 255,591 |
| ZNF582-AS1 | ZNF582 antisense RNA 1 (head to head) | **1,093** | 0,303692 | 1,717 | 2,809 |
| MTSS1L | MTSS1L, I-BAR domain containing | **1,093** | 0,006572 | 24,649 | 17,686 |
| BRMS1L | breast cancer metastasis-suppressor 1 like | **1,093** | 0,044751 | 8,807 | 7,726 |
| C1orf115 | chromosome 1 open reading frame 115 | **1,093** | 0,042068 | 7,810 | 9,705 |
| ANKMY1 | ankyrin repeat and MYND domain containing 1 | **1,093** | 0,074657 | 8,032 | 10,599 |
| CYB5RL | cytochrome b5 reductase like | **1,093** | 0,177538 | 3,490 | 2,746 |
| LINC01578 | long intergenic non-protein coding RNA 1578 | **1,093** | 0,008649 | 53,452 | 88,049 |
| CPED1 | cadherin like and PC-esterase domain containing 1 | **1,093** | 0,011437 | 47,746 | 50,505 |
| HIST4H4 | histone cluster 4 H4 | **1,093** | 0,487678 | 0,499 | 1,149 |
| TRPV2 | transient receptor potential cation channel subfamily V member 2 | **1,093** | 0,392111 | 4,431 | 1,660 |
| SLC25A13 | solute carrier family 25 member 13 | **1,093** | 0,029503 | 16,340 | 16,218 |
| ZNF823 | zinc finger protein 823 | **1,093** | 0,095084 | 4,154 | 5,427 |
| AHDC1 | AT-hook DNA binding motif containing 1 | **1,093** | 0,02566 | 24,039 | 30,967 |
| ZNF800 | zinc finger protein 800 | **1,093** | 0,014539 | 19,719 | 21,709 |
| USP22 | ubiquitin specific peptidase 22 | **1,093** | 0,000774 | 136,371 | 142,832 |
| MEMO1 | mediator of cell motility 1 | **1,093** | 0,516014 | 1,329 | 0,766 |
| TNKS2 | tankyrase 2 | **1,092** | 0,013093 | 65,083 | 68,064 |
| IFI44 | interferon induced protein 44 | **1,092** | 0,62318 | 0,388 | 0,830 |

| | | | | | |
|---|---|---|---|---|---|
| SRR | serine racemase | **1,092** | 0,083606 | 8,087 | 6,193 |
| PEG3 | paternally expressed 3 | **1,092** | 0,040771 | 9,638 | 13,600 |
| BAG3 | BCL2 associated athanogene 3 | **1,092** | 0,000571 | 78,266 | 99,414 |
| DEAF1 | DEAF1, transcription factor | **1,092** | 0,005706 | 19,553 | 20,177 |
| TET2 | tet methylcytosine dioxygenase 2 | **1,092** | 0,246876 | 12,629 | 15,771 |
| CBLN3 | cerebellin 3 precursor | **1,092** | 0,181724 | 3,711 | 7,023 |
| CSMD2 | CUB and Sushi multiple domains 2 | **1,092** | 0,06996 | 16,617 | 18,580 |
| LYPD6 | LY6/PLAUR domain containing 6 | **1,092** | 0,609384 | 0,277 | 0,511 |
| B3GNT2 | UDP-GlcNAc:betaGal beta-1,3-N-acetylglucosaminyltransferase 2 | **1,092** | 0,03786 | 23,818 | 26,625 |
| ATP2B1-AS1 | ATP2B1 antisense RNA 1 | **1,092** | 0,007997 | 19,664 | 21,326 |
| KDM5A | lysine demethylase 5A | **1,092** | 0,004642 | 52,676 | 60,210 |
| ELMO2 | engulfment and cell motility 2 | **1,092** | 0,005024 | 40,490 | 38,182 |
| RHBDF2 | rhomboid 5 homolog 2 | **1,092** | 0,001287 | 45,365 | 66,085 |
| FAM160B1 | family with sequence similarity 160 member B1 | **1,092** | 0,036578 | 22,488 | 22,411 |
| PKN3 | protein kinase N3 | **1,092** | 0,001655 | 31,129 | 38,118 |
| MBLAC2 | metallo-beta-lactamase domain containing 2 | **1,092** | 0,140073 | 4,154 | 5,108 |
| VPS37A | VPS37A, ESCRT-I subunit | **1,092** | 0,001549 | 63,089 | 69,660 |
| RHPN1 | rhophilin Rho GTPase binding protein 1 | **1,092** | 0,396564 | 1,496 | 2,043 |
| STRN3 | striatin 3 | **1,092** | 0,008751 | 27,861 | 28,796 |
| TSLP | thymic stromal lymphopoietin | **1,092** | 0,231427 | 2,160 | 2,809 |
| SLC41A1 | solute carrier family 41 member 1 | **1,092** | 0,014773 | 62,812 | 90,475 |
| ASB7 | ankyrin repeat and SOCS box containing 7 | **1,092** | 0,022342 | 13,072 | 14,111 |
| SAMD8 | sterile alpha motif domain containing 8 | **1,092** | 0,01293 | 47,137 | 46,802 |
| GALNT1 | polypeptide N-acetylgalactosaminyltransferase 1 | **1,092** | 0,00211 | 91,615 | 94,817 |
| SFPQ | splicing factor proline and glutamine rich | **1,092** | 0,003812 | 138,309 | 147,238 |
| FAM168B | family with sequence similarity 168 member B | **1,092** | 0,001137 | 153,154 | 198,254 |
| CKMT2-AS1 | CKMT2 antisense RNA 1 | **1,092** | 0,138843 | 4,708 | 5,044 |
| ZFP62 | ZFP62 zinc finger protein | **1,092** | 0,02011 | 12,740 | 16,026 |
| MATN1-AS1 | MATN1 antisense RNA 1 | **1,091** | 0,315345 | 0,886 | 1,724 |
| APPBP2 | amyloid beta precursor protein binding protein 2 | **1,091** | 0,00245 | 35,671 | 39,651 |
| IQCA1 | IQ motif containing with AAA domain 1 | **1,091** | 0,097636 | 4,542 | 5,300 |
| COQ8A | coenzyme Q8A | **1,091** | 0,016262 | 17,337 | 23,944 |
| RFTN2 | raftlin family member 2 | **1,091** | 0,083434 | 16,451 | 12,195 |

| | | | | | |
|---|---|---|---|---|---|
| ERICH1 | glutamate rich 1 | **1,091** | 0,082678 | 9,804 | 9,769 |
| ABCA5 | ATP binding cassette subfamily A member 5 | **1,091** | 0,024258 | 28,249 | 35,884 |
| RNF146 | ring finger protein 146 | **1,091** | 0,002698 | 46,362 | 46,419 |
| ERBIN | erbb2 interacting protein | **1,091** | 0,027829 | 45,697 | 51,144 |
| B4GALNT1 | beta-1,4-N-acetyl-galactosaminyltransferase 1 | **1,091** | 0,190264 | 8,087 | 5,683 |
| ZFX | zinc finger protein, X-linked | **1,091** | 0,009888 | 43,703 | 44,950 |
| APCDD1L | APC down-regulated 1 like | **1,091** | 0,186754 | 13,127 | 9,258 |
| NFYA | nuclear transcription factor Y subunit alpha | **1,091** | 0,021235 | 36,281 | 46,930 |
| MYH3 | myosin heavy chain 3 | **1,091** | 0,279486 | 1,551 | 3,767 |
| WASF3 | WAS protein family member 3 | **1,091** | 0,024731 | 14,900 | 26,562 |
| EXOC8 | exocyst complex component 8 | **1,091** | 0,01809 | 27,529 | 28,732 |
| VCPIP1 | valosin containing protein interacting protein 1 | **1,091** | 0,005933 | 36,447 | 31,286 |
| ECHDC2 | enoyl-CoA hydratase domain containing 2 | **1,091** | 0,019144 | 15,177 | 13,408 |
| NDUFS4 | NADH:ubiquinone oxidoreductase subunit S4 | **1,091** | 0,003266 | 18,888 | 22,284 |
| POU3F3 | POU class 3 homeobox 3 | **1,091** | 0,127592 | 3,379 | 3,320 |
| DCUN1D4 | defective in cullin neddylation 1 domain containing 4 | **1,091** | 0,022436 | 18,168 | 18,644 |
| RALB | RAS like proto-oncogene B | **1,091** | 0,000625 | 64,585 | 74,321 |
| NFKBIA | NFKB inhibitor alpha | **1,091** | 0,001252 | 523,769 | 636,200 |
| SLC39A11 | solute carrier family 39 member 11 | **1,091** | 0,03771 | 10,801 | 13,408 |
| WWTR1 | WW domain containing transcription regulator 1 | **1,091** | 0,003901 | 95,493 | 83,707 |
| YWHAG | tyrosine 3-monooxygenase/tryptophan 5-monooxygenase activation protein gamma | **1,091** | 0,00263 | 184,006 | 184,654 |
| ZNF595 | zinc finger protein 595 | **1,091** | 0,123745 | 4,597 | 5,300 |
| PCDHGB7 | protocadherin gamma subfamily B, 7 | **1,091** | 0,090833 | 7,256 | 8,109 |
| RNF11 | ring finger protein 11 | **1,091** | 0,000927 | 93,000 | 94,242 |
| TRAF5 | TNF receptor associated factor 5 | **1,091** | 0,484444 | 1,662 | 1,596 |
| C8G | complement C8 gamma chain | **1,091** | 0,645367 | 0,332 | 0,319 |
| ANKRD12 | ankyrin repeat domain 12 | **1,091** | 0,050367 | 159,745 | 184,782 |
| COL1A1 | collagen type I alpha 1 chain | **1,090** | 0,239333 | 17,282 | 7,343 |
| FGD5-AS1 | FGD5 antisense RNA 1 | **1,090** | 0,003206 | 139,528 | 146,152 |
| MED12L | mediator complex subunit 12 like | **1,090** | 0,659139 | 0,332 | 0,511 |
| ASB8 | ankyrin repeat and SOCS box containing 8 | **1,090** | 0,006646 | 29,135 | 30,967 |
| ADGRD1 | adhesion G protein-coupled receptor D1 | **1,090** | 0,37049 | 2,216 | 1,788 |
| PIP4K2A | phosphatidylinositol-5-phosphate 4-kinase type 2 alpha | **1,090** | 0,011935 | 25,756 | 27,200 |

| | | | | | |
|---|---|---|---|---|---|
| SNED1 | sushi, nidogen and EGF like domains 1 | **1,090** | 0,015791 | 45,974 | 28,477 |
| GABPA | GA binding protein transcription factor alpha subunit | **1,090** | 0,021233 | 27,363 | 28,222 |
| ZNF571 | zinc finger protein 571 | **1,090** | 0,237005 | 2,271 | 3,065 |
| ZNF738 | zinc finger protein 738 | **1,090** | 0,318601 | 1,385 | 2,554 |
| TRIM24 | tripartite motif containing 24 | **1,090** | 0,038614 | 15,288 | 13,089 |
| SHOC2 | SHOC2, leucine rich repeat scaffold protein | **1,090** | 0,004145 | 49,574 | 50,441 |
| MOB1A | MOB kinase activator 1A | **1,090** | 0,000503 | 60,375 | 66,532 |
| HMGB1P31 | high mobility group box 1 pseudogene 31 | **1,090** | 0,562921 | 0,554 | 0,447 |
| CDKN2AIP | CDKN2A interacting protein | **1,090** | 0,002239 | 23,652 | 34,798 |
| TRIM23 | tripartite motif containing 23 | **1,090** | 0,002739 | 32,625 | 38,821 |
| YOD1 | YOD1 deubiquitinase | **1,090** | 0,048967 | 8,807 | 8,428 |
| SERPINB8 | serpin family B member 8 | **1,090** | 0,015718 | 33,289 | 40,864 |
| STK10 | serine/threonine kinase 10 | **1,090** | 0,002621 | 39,272 | 48,462 |
| PCDHGA8 | protocadherin gamma subfamily A, 8 | **1,090** | 0,617501 | 0,332 | 0,447 |
| KDM4C | lysine demethylase 4C | **1,090** | 0,026463 | 24,372 | 26,434 |
| 4.syys | septin 4 | **1,090** | 0,362783 | 1,385 | 3,001 |
| RSBN1 | round spermatid basic protein 1 | **1,090** | 0,018076 | 18,057 | 25,412 |
| ARRB1 | arrestin beta 1 | **1,090** | 0,210043 | 2,770 | 2,299 |
| ZNF696 | zinc finger protein 696 | **1,090** | 0,056227 | 7,644 | 8,684 |
| LZTS3 | leucine zipper tumor suppressor family member 3 | **1,090** | 0,149102 | 7,699 | 9,514 |
| LIN54 | lin-54 DREAM MuvB core complex component | **1,089** | 0,033557 | 18,666 | 17,048 |
| B3GLCT | beta 3-glucosyltransferase | **1,089** | 0,14303 | 11,078 | 7,726 |
| B4GALT6 | beta-1,4-galactosyltransferase 6 | **1,089** | 0,029052 | 30,631 | 27,328 |
| SNAI1 | snail family transcriptional repressor 1 | **1,089** | 0,095665 | 7,533 | 8,620 |
| RAB3IP | RAB3A interacting protein | **1,089** | 0,139803 | 4,431 | 5,300 |
| MIR3945HG | MIR3945 host gene | **1,089** | 0,639515 | 1,052 | 0,447 |
| IFRD1 | interferon related developmental regulator 1 | **1,089** | 0,004795 | 74,334 | 78,535 |
| CTC1 | CST telomere replication complex component 1 | **1,089** | 0,009795 | 13,404 | 18,516 |
| SEC24B | SEC24 homolog B, COPII coat complex component | **1,089** | 0,002687 | 42,540 | 49,101 |
| TGFBR1 | transforming growth factor beta receptor 1 | **1,089** | 0,005302 | 141,633 | 140,150 |
| KAT7 | lysine acetyltransferase 7 | **1,089** | 0,00455 | 31,351 | 33,010 |
| HECTD1 | HECT domain E3 ubiquitin protein ligase 1 | **1,089** | 0,039698 | 123,908 | 143,535 |
| ABCA13 | ATP binding cassette subfamily A member 13 | **1,089** | 0,04141 | 22,156 | 13,536 |

| | | | | | |
|---|---|---|---|---|---|
| RTKN | rhotekin | **1,089** | 0,028588 | 11,189 | 10,535 |
| ANKRA2 | ankyrin repeat family A member 2 | **1,089** | 0,048724 | 10,358 | 10,791 |
| AHNAK | AHNAK nucleoprotein | **1,089** | 0,035271 | 1374,175 | 1162,132 |
| CLMP | CXADR like membrane protein | **1,089** | 0,002014 | 743,890 | 734,210 |
| MYBPH | myosin binding protein H | **1,089** | 0,344375 | 1,551 | 2,618 |
| STXBP5 | syntaxin binding protein 5 | **1,089** | 0,031051 | 29,578 | 21,198 |
| ACYP1 | acylphosphatase 1 | **1,089** | 0,217129 | 2,603 | 2,554 |
| CTBP2 | C-terminal binding protein 2 | **1,089** | 0,003375 | 33,179 | 34,351 |
| CLIC6 | chloride intracellular channel 6 | **1,089** | 0,004311 | 32,957 | 55,805 |
| EXOC1 | exocyst complex component 1 | **1,089** | 0,00115 | 39,050 | 41,183 |
| GPC5 | glypican 5 | **1,089** | 0,079701 | 4,043 | 8,364 |
| NFAT5 | nuclear factor of activated T-cells 5 | **1,088** | 0,17791 | 60,320 | 65,063 |
| TP53RK | TP53 regulating kinase | **1,088** | 0,001339 | 30,963 | 42,396 |
| TAF7 | TATA-box binding protein associated factor 7 | **1,088** | 0,000568 | 126,788 | 132,042 |
| PILRB | paired immunoglobin-like type 2 receptor beta | **1,088** | 0,417864 | 1,385 | 2,107 |
| HCAR1 | hydroxycarboxylic acid receptor 1 | **1,088** | 0,590286 | 0,609 | 0,255 |
| DIABLO | diablo IAP-binding mitochondrial protein | **1,088** | 0,39822 | 1,108 | 1,660 |
| VWDE | von Willebrand factor D and EGF domains | **1,088** | 0,402574 | 2,049 | 0,766 |
| UBE2O | ubiquitin conjugating enzyme E2 O | **1,088** | 0,001496 | 56,609 | 69,341 |
| PTMAP4 | prothymosin, alpha pseudogene 4 | **1,088** | 0,581131 | 0,775 | 0,766 |
| RNF125 | ring finger protein 125 | **1,088** | 0,546328 | 0,499 | 0,638 |
| TMEM255B | transmembrane protein 255B | **1,088** | 0,014274 | 21,602 | 18,070 |
| DDHD1 | DDHD domain containing 1 | **1,088** | 0,114778 | 9,859 | 10,471 |
| F13A1 | coagulation factor XIII A chain | **1,088** | 0,011485 | 103,303 | 74,513 |
| DSC2 | desmocollin 2 | **1,088** | 0,292014 | 5,207 | 2,171 |
| TGFB1 | transforming growth factor beta 1 | **1,088** | 0,080429 | 141,965 | 108,098 |
| MCU | mitochondrial calcium uniporter | **1,088** | 0,023069 | 16,783 | 16,856 |
| KIAA0513 | KIAA0513 | **1,088** | 0,058828 | 9,416 | 11,940 |
| TMEM55B | transmembrane protein 55B | **1,088** | 0,008956 | 35,616 | 34,032 |
| AMFR | autocrine motility factor receptor | **1,088** | 0,00111 | 149,000 | 137,405 |
| ETS1 | ETS proto-oncogene 1, transcription factor | **1,088** | 0,00255 | 240,393 | 294,539 |
| DUSP28 | dual specificity phosphatase 28 | **1,088** | 0,242297 | 2,659 | 3,256 |
| HABP4 | hyaluronan binding protein 4 | **1,088** | 0,018575 | 23,652 | 26,625 |

| | | | | | |
|---|---|---|---|---|---|
| NAA16 | N(alpha)-acetyltransferase 16, NatA auxiliary subunit | **1,088** | 0,150391 | 5,761 | 7,407 |
| AGAP4 | ArfGAP with GTPase domain, ankyrin repeat and PH domain 4 | **1,088** | 0,571647 | 0,554 | 0,958 |
| PPM1D | protein phosphatase, Mg2+/Mn2+ dependent 1D | **1,088** | 0,020022 | 18,611 | 21,454 |
| IFNAR1 | interferon alpha and beta receptor subunit 1 | **1,088** | 0,002188 | 146,950 | 146,663 |
| RHEB | Ras homolog enriched in brain | **1,088** | 0,007861 | 99,204 | 98,265 |
| ZNF138 | zinc finger protein 138 | **1,087** | 0,437421 | 1,385 | 1,277 |
| PCBP1-AS1 | PCBP1 antisense RNA 1 | **1,087** | 0,013161 | 15,675 | 23,624 |
| NNT | nicotinamide nucleotide transhydrogenase | **1,087** | 0,023921 | 15,897 | 11,110 |
| KIF5B | kinesin family member 5B | **1,087** | 0,001793 | 180,683 | 206,618 |
| CHST11 | carbohydrate sulfotransferase 11 | **1,087** | 0,00964 | 85,356 | 71,767 |
| RFFL | ring finger and FYVE like domain containing E3 ubiquitin protein ligase | **1,087** | 0,013974 | 25,424 | 31,861 |
| MLH3 | mutL homolog 3 | **1,087** | 0,012551 | 19,940 | 17,686 |
| CAMK2N2 | calcium/calmodulin dependent protein kinase II inhibitor 2 | **1,087** | 0,554034 | 0,443 | 0,766 |
| DOK4 | docking protein 4 | **1,087** | 0,046087 | 14,401 | 15,069 |
| HK1 | hexokinase 1 | **1,087** | 0,002597 | 208,710 | 166,137 |
| DPF3 | double PHD fingers 3 | **1,087** | 0,476894 | 0,997 | 0,830 |
| SHB | SH2 domain containing adaptor protein B | **1,087** | 0,0622 | 6,038 | 6,066 |
| CYBRD1 | cytochrome b reductase 1 | **1,087** | 0,002798 | 143,627 | 113,270 |
| WNK1 | WNK lysine deficient protein kinase 1 | **1,087** | 0,060162 | 137,645 | 139,129 |
| SMIM8 | small integral membrane protein 8 | **1,087** | 0,233353 | 3,767 | 5,363 |
| MAP7 | microtubule associated protein 7 | **1,087** | 0,125483 | 4,154 | 6,193 |
| ELOVL7 | ELOVL fatty acid elongase 7 | **1,087** | 0,039054 | 44,091 | 43,290 |
| CAMK1D | calcium/calmodulin dependent protein kinase ID | **1,087** | 0,011699 | 23,098 | 36,075 |
| KLHL8 | kelch like family member 8 | **1,087** | 0,048462 | 13,238 | 15,707 |
| UBR3 | ubiquitin protein ligase E3 component n-recognin 3 (putative) | **1,087** | 0,011001 | 43,149 | 38,821 |
| ISLR2 | immunoglobulin superfamily containing leucine rich repeat 2 | **1,087** | 0,661737 | 0,443 | 1,149 |
| DNHD1 | dynein heavy chain domain 1 | **1,087** | 0,115774 | 10,469 | 17,814 |
| POGK | pogo transposable element derived with KRAB domain | **1,087** | 0,016827 | 44,312 | 42,205 |
| RIPK2 | receptor interacting serine/threonine kinase 2 | **1,087** | 0,003026 | 104,798 | 119,910 |
| IL1RAP | interleukin 1 receptor accessory protein | **1,087** | 0,024522 | 24,704 | 26,434 |
| IGSF9B | immunoglobulin superfamily member 9B | **1,087** | 0,234384 | 6,979 | 9,641 |
| PHF7 | PHD finger protein 7 | **1,087** | 0,270673 | 3,600 | 3,384 |
| KCNJ14 | potassium voltage-gated channel subfamily J member 14 | **1,087** | 0,355317 | 1,052 | 2,043 |

| | | | | | |
|---|---|---|---|---|---|
| GYPE | glycophorin E (MNS blood group) | **1,086** | 0,723447 | 0,388 | 0,638 |
| PTOV1-AS2 | PTOV1 antisense RNA 2 | **1,086** | 0,290302 | 1,606 | 3,384 |
| DAAM1 | dishevelled associated activator of morphogenesis 1 | **1,086** | 0,014546 | 21,381 | 27,328 |
| GOLGA8B | golgin A8 family member B | **1,086** | 0,105076 | 6,148 | 6,960 |
| ROS1 | ROS proto-oncogene 1, receptor tyrosine kinase | **1,086** | 0,472398 | 1,163 | 1,405 |
| MAGI3 | membrane associated guanylate kinase, WW and PDZ domain containing 3 | **1,086** | 0,206099 | 3,379 | 3,512 |
| CAPZA2 | capping actin protein of muscle Z-line alpha subunit 2 | **1,086** | 0,003654 | 86,132 | 83,133 |
| TM4SF1 | transmembrane 4 L six family member 1 | **1,086** | 0,000509 | 666,399 | 653,631 |
| RPS27L | ribosomal protein S27 like | **1,086** | 0,020398 | 50,350 | 71,193 |
| MKLN1-AS | MKLN1 antisense RNA | **1,086** | 0,203932 | 2,326 | 3,192 |
| SOCS4 | suppressor of cytokine signaling 4 | **1,086** | 0,023234 | 25,812 | 26,881 |
| ZNF789 | zinc finger protein 789 | **1,086** | 0,150086 | 5,207 | 6,640 |
| ASAH2 | N-acylsphingosine amidohydrolase 2 | **1,086** | 0,553207 | 1,052 | 0,575 |
| HIST1H2BC | histone cluster 1 H2B family member c | **1,086** | 0,095178 | 10,967 | 16,856 |
| DGKQ | diacylglycerol kinase theta | **1,086** | 0,118889 | 13,460 | 18,836 |
| SPSB1 | splA/ryanodine receptor domain and SOCS box containing 1 | **1,086** | 0,00394 | 40,546 | 29,946 |
| LINC00894 | long intergenic non-protein coding RNA 894 | **1,086** | 0,567371 | 0,775 | 1,213 |
| STK35 | serine/threonine kinase 35 | **1,086** | 0,028064 | 33,179 | 33,904 |
| WDR26 | WD repeat domain 26 | **1,086** | 0,009333 | 79,429 | 75,279 |
| LINC00632 | long intergenic non-protein coding RNA 632 | **1,086** | 0,612574 | 0,720 | 0,766 |
| PFKFB3 | 6-phosphofructo-2-kinase/fructose-2,6-biphosphatase 3 | **1,086** | 0,004319 | 120,474 | 131,403 |
| TMBIM1 | transmembrane BAX inhibitor motif containing 1 | **1,086** | 0,000991 | 236,073 | 213,833 |
| ZNF622 | zinc finger protein 622 | **1,086** | 0,00836 | 51,347 | 60,338 |
| TRAPPC10 | trafficking protein particle complex 10 | **1,086** | 0,100965 | 18,833 | 18,900 |
| SLC22A15 | solute carrier family 22 member 15 | **1,086** | 0,093848 | 22,433 | 22,475 |
| CSN1S1 | casein alpha s1 | **1,086** | 0,519646 | 2,160 | 0,830 |
| HIP1 | huntingtin interacting protein 1 | **1,085** | 0,071644 | 12,518 | 20,368 |
| LAMA5 | laminin subunit alpha 5 | **1,085** | 0,156269 | 3,213 | 5,172 |
| EFCAB6 | EF-hand calcium binding domain 6 | **1,085** | 0,396706 | 1,440 | 1,852 |
| PLA2G15 | phospholipase A2 group XV | **1,085** | 0,048157 | 18,999 | 17,239 |
| ZNF347 | zinc finger protein 347 | **1,085** | 0,048782 | 9,084 | 13,664 |
| NUCB1-AS1 | NUCB1 antisense RNA 1 | **1,085** | 0,743344 | 0,443 | 0,830 |
| BMP2 | bone morphogenetic protein 2 | **1,085** | 0,009887 | 1617,227 | 1048,415 |

| | | | | | |
|---|---|---|---|---|---|
| PURB | purine rich element binding protein B | **1,085** | 0,036466 | 47,137 | 52,165 |
| TEX30 | testis expressed 30 | **1,085** | 0,152037 | 7,588 | 7,981 |
| ATP6V1A | ATPase H+ transporting V1 subunit A | **1,085** | 0,004033 | 40,823 | 46,547 |
| ZNF714 | zinc finger protein 714 | **1,085** | 0,459264 | 1,883 | 1,596 |
| ZNF644 | zinc finger protein 644 | **1,085** | 0,013657 | 39,604 | 44,567 |
| SUCO | SUN domain containing ossification factor | **1,085** | 0,009832 | 102,693 | 116,845 |
| TAF9B | TATA-box binding protein associated factor 9b | **1,085** | 0,044094 | 8,198 | 8,747 |
| ETNK1 | ethanolamine kinase 1 | **1,085** | 0,009485 | 43,426 | 41,822 |
| TOPORS | TOP1 binding arginine/serine rich protein | **1,085** | 0,002594 | 39,936 | 42,779 |
| KIF25-AS1 | KIF25 antisense RNA 1 | **1,085** | 0,730244 | 0,222 | 0,447 |
| NEU3 | neuraminidase 3 | **1,085** | 0,076802 | 9,527 | 13,217 |
| MAP6 | microtubule associated protein 6 | **1,085** | 0,222561 | 3,323 | 2,937 |
| LINC00909 | long intergenic non-protein coding RNA 909 | **1,085** | 0,048974 | 9,306 | 10,471 |
| NFASC | neurofascin | **1,085** | 0,111016 | 10,967 | 4,597 |
| ZMIZ2 | zinc finger MIZ-type containing 2 | **1,085** | 0,016273 | 65,028 | 75,407 |
| RNF122 | ring finger protein 122 | **1,085** | 0,518462 | 0,554 | 1,022 |
| WAC | WW domain containing adaptor with coiled-coil | **1,085** | 0,028351 | 114,436 | 126,934 |
| DST | dystonin | **1,084** | 0,16722 | 279,222 | 310,757 |
| LINC00511 | long intergenic non-protein coding RNA 511 | **1,084** | 0,009179 | 34,065 | 36,331 |
| UBE2D1 | ubiquitin conjugating enzyme E2 D1 | **1,084** | 0,02873 | 13,460 | 16,793 |
| GTF2IRD1 | GTF2I repeat domain containing 1 | **1,084** | 0,013921 | 15,731 | 24,008 |
| CD4 | CD4 molecule | **1,084** | 0,487659 | 0,388 | 0,894 |
| ZBTB40 | zinc finger and BTB domain containing 40 | **1,084** | 0,017585 | 42,097 | 51,463 |
| STAC3 | SH3 and cysteine rich domain 3 | **1,084** | 0,507422 | 0,443 | 0,830 |
| RIT1 | Ras like without CAAX 1 | **1,084** | 0,026752 | 14,900 | 12,770 |
| PEX1 | peroxisomal biogenesis factor 1 | **1,084** | 0,033552 | 15,177 | 17,112 |
| RAI2 | retinoic acid induced 2 | **1,084** | 0,13027 | 5,761 | 5,491 |
| NUDT21 | nudix hydrolase 21 | **1,084** | 0,002927 | 69,127 | 91,305 |
| ZNF235 | zinc finger protein 235 | **1,084** | 0,432533 | 1,939 | 2,746 |
| LINC01619 | long intergenic non-protein coding RNA 1619 | **1,084** | 0,460015 | 0,277 | 1,022 |
| ADPRM | ADP-ribose/CDP-alcohol diphosphatase, manganese dependent | **1,084** | 0,074326 | 6,204 | 8,620 |
| APP | amyloid beta precursor protein | **1,084** | 0,001434 | 668,504 | 536,403 |
| CKAP5 | cytoskeleton associated protein 5 | **1,084** | 0,012152 | 61,926 | 64,425 |

| | | | | | |
|---|---|---|---|---|---|
| ZNF549 | zinc finger protein 549 | **1,084** | 0,099855 | 5,151 | 7,215 |
| C22orf29 | chromosome 22 open reading frame 29 | **1,084** | 0,045811 | 16,174 | 17,495 |
| TGFBRAP1 | transforming growth factor beta receptor associated protein 1 | **1,084** | 0,036303 | 20,605 | 21,454 |
| MICU3 | mitochondrial calcium uptake family member 3 | **1,084** | 0,211085 | 4,653 | 6,193 |
| SP1 | Sp1 transcription factor | **1,084** | 0,065032 | 86,796 | 95,775 |
| EPS15 | epidermal growth factor receptor pathway substrate 15 | **1,084** | 0,028163 | 19,497 | 20,624 |
| CCDC149 | coiled-coil domain containing 149 | **1,084** | 0,00878 | 29,191 | 21,262 |
| BNIP3 | BCL2 interacting protein 3 | **1,084** | 0,003851 | 136,149 | 133,893 |
| HIST1H2AC | histone cluster 1 H2A family member c | **1,084** | 0,007704 | 38,829 | 51,463 |
| ISCA1 | iron-sulfur cluster assembly 1 | **1,084** | 0,018798 | 21,990 | 19,921 |
| POLK | DNA polymerase kappa | **1,084** | 0,015779 | 28,914 | 33,010 |
| ZNF567 | zinc finger protein 567 | **1,084** | 0,156103 | 4,154 | 4,406 |
| PRKAG2-AS1 | PRKAG2 antisense RNA 1 | **1,084** | 0,273065 | 2,049 | 3,320 |
| CHD7 | chromodomain helicase DNA binding protein 7 | **1,084** | 0,09624 | 13,072 | 17,750 |
| RALGAPA1 | Ral GTPase activating protein catalytic alpha subunit 1 | **1,084** | 0,159344 | 16,949 | 19,666 |
| WDR7 | WD repeat domain 7 | **1,084** | 0,029742 | 22,599 | 26,881 |
| ZNF862 | zinc finger protein 862 | **1,084** | 0,02271 | 15,565 | 18,197 |
| WIPF2 | WAS/WASL interacting protein family member 2 | **1,083** | 0,00253 | 51,457 | 49,164 |
| UBE2B | ubiquitin conjugating enzyme E2 B | **1,083** | 0,007396 | 81,645 | 94,179 |
| AGAP2 | ArfGAP with GTPase domain, ankyrin repeat and PH domain 2 | **1,083** | 0,673022 | 0,554 | 0,128 |
| CRYZ | crystallin zeta | **1,083** | 0,015153 | 14,955 | 11,876 |
| MAT2B | methionine adenosyltransferase 2B | **1,083** | 0,002772 | 61,871 | 68,958 |
| RAB11FIP1 | RAB11 family interacting protein 1 | **1,083** | 0,332982 | 2,271 | 2,937 |
| NOVA1 | NOVA alternative splicing regulator 1 | **1,083** | 0,005775 | 56,332 | 35,309 |
| MERTK | MER proto-oncogene, tyrosine kinase | **1,083** | 0,257471 | 2,880 | 3,703 |
| SYT12 | synaptotagmin 12 | **1,083** | 0,073764 | 11,189 | 15,899 |
| POPDC3 | popeye domain containing 3 | **1,083** | 0,112121 | 10,524 | 4,469 |
| MED26 | mediator complex subunit 26 | **1,083** | 0,142385 | 6,425 | 6,832 |
| BTG1 | BTG anti-proliferation factor 1 | **1,083** | 0,000789 | 501,946 | 843,138 |
| SLC6A6 | solute carrier family 6 member 6 | **1,083** | 0,020442 | 27,030 | 21,581 |
| TUG1 | taurine up-regulated 1 (non-protein coding) | **1,083** | 0,042835 | 130,001 | 137,405 |
| LOXL4 | lysyl oxidase like 4 | **1,083** | 0,009895 | 270,470 | 357,368 |
| RCHY1 | ring finger and CHY zinc finger domain containing 1 | **1,083** | 0,035718 | 12,740 | 14,302 |

| | | | | | |
|---|---|---|---|---|---|
| GPLD1 | glycosylphosphatidylinositol specific phospholipase D1 | **1,083** | 0,337647 | 1,274 | 2,362 |
| FAR1 | fatty acyl-CoA reductase 1 | **1,083** | 0,028992 | 27,418 | 25,157 |
| ZNF184 | zinc finger protein 184 | **1,083** | 0,162456 | 8,475 | 8,684 |
| FCHO2 | FCH domain only 2 | **1,083** | 0,076404 | 23,541 | 24,327 |
| EML6 | echinoderm microtubule associated protein like 6 | **1,083** | 0,268233 | 2,049 | 3,448 |
| PSD | pleckstrin and Sec7 domain containing | **1,083** | 0,365109 | 1,274 | 2,554 |
| PSMD5-AS1 | PSMD5 antisense RNA 1 (head to head) | **1,083** | 0,088812 | 11,355 | 19,219 |
| CCDC82 | coiled-coil domain containing 82 | **1,083** | 0,005172 | 34,397 | 38,246 |
| AHCTF1 | AT-hook containing transcription factor 1 | **1,083** | 0,02885 | 37,776 | 45,206 |
| GAPLINC | gastric adenocarcinoma associated, positive CD44 regulator, long intergenic non-coding RI | **1,083** | 0,633179 | 0,277 | 0,319 |
| CDAN1 | codanin 1 | **1,083** | 0,013364 | 15,565 | 16,920 |
| ZNF75A | zinc finger protein 75a | **1,083** | 0,035513 | 9,139 | 11,429 |
| CBX7 | chromobox 7 | **1,083** | 0,001664 | 50,848 | 54,528 |
| KIAA0907 | KIAA0907 | **1,083** | 0,008486 | 33,289 | 43,482 |
| HECW1 | HECT, C2 and WW domain containing E3 ubiquitin protein ligase 1 | **1,083** | 0,424625 | 2,437 | 2,490 |
| DNAH6 | dynein axonemal heavy chain 6 | **1,083** | 0,510717 | 0,720 | 1,341 |
| RAB3IL1 | RAB3A interacting protein like 1 | **1,083** | 0,019937 | 12,906 | 10,663 |
| FAM91A1 | family with sequence similarity 91 member A1 | **1,083** | 0,020097 | 39,272 | 40,162 |
| RPS28P7 | ribosomal protein S28 pseudogene 7 | **1,083** | 0,64662 | 0,388 | 0,319 |
| MIER1 | MIER1 transcriptional regulator | **1,083** | 0,043728 | 41,321 | 43,226 |
| CHGB | chromogranin B | **1,083** | 0,618953 | 0,277 | 0,575 |
| GADD45G | growth arrest and DNA damage inducible gamma | **1,083** | 0,064686 | 7,478 | 14,494 |
| FOXD2-AS1 | FOXD2 antisense RNA 1 (head to head) | **1,082** | 0,143685 | 3,157 | 7,470 |
| SEC14L1P1 | SEC14 like 1 pseudogene 1 | **1,082** | 0,576232 | 0,554 | 1,085 |
| R3HCC1L | R3H domain and coiled-coil containing 1 like | **1,082** | 0,024967 | 17,559 | 20,177 |
| RIC1 | RIC1 homolog, RAB6A GEF complex partner 1 | **1,082** | 0,160153 | 21,214 | 22,794 |
| NUMBL | NUMB like, endocytic adaptor protein | **1,082** | 0,043603 | 14,512 | 12,004 |
| TPRN | taperin | **1,082** | 0,188059 | 13,127 | 13,728 |
| WDPCP | WD repeat containing planar cell polarity effector | **1,082** | 0,194591 | 2,880 | 4,533 |
| TLE1 | transducin like enhancer of split 1 | **1,082** | 0,015526 | 31,351 | 27,583 |
| TTBK2 | tau tubulin kinase 2 | **1,082** | 0,055936 | 24,704 | 24,327 |
| ZNF770 | zinc finger protein 770 | **1,082** | 0,024172 | 50,073 | 50,825 |
| GRIP1 | glutamate receptor interacting protein 1 | **1,082** | 0,559678 | 0,831 | 0,575 |

| | | | | | |
|---|---|---|---|---|---|
| ZNF585B | zinc finger protein 585B | **1,082** | 0,247868 | 5,262 | 6,321 |
| CSNK1G3 | casein kinase 1 gamma 3 | **1,082** | 0,004861 | 36,170 | 37,224 |
| ZNF211 | zinc finger protein 211 | **1,082** | 0,19047 | 3,268 | 4,278 |
| SEMA3D | semaphorin 3D | **1,082** | 0,202008 | 18,113 | 14,047 |
| TLN2 | talin 2 | **1,082** | 0,071102 | 8,752 | 13,281 |
| COL12A1 | collagen type XII alpha 1 chain | **1,082** | 0,039375 | 260,334 | 167,734 |
| ULBP3 | UL16 binding protein 3 | **1,082** | 0,31249 | 2,326 | 3,256 |
| VNN1 | vanin 1 | **1,082** | 0,013105 | 64,031 | 94,881 |
| EPC2 | enhancer of polycomb homolog 2 | **1,082** | 0,011687 | 30,354 | 37,608 |
| NUMB | NUMB, endocytic adaptor protein | **1,082** | 0,007994 | 130,499 | 139,767 |
| HIST1H2AG | histone cluster 1 H2A family member g | **1,082** | 0,36939 | 1,772 | 3,256 |
| ARL13B | ADP ribosylation factor like GTPase 13B | **1,081** | 0,042143 | 11,410 | 14,111 |
| COPRS | coordinator of PRMT5 and differentiation stimulator | **1,081** | 0,055717 | 31,462 | 28,158 |
| ZNF674 | zinc finger protein 674 | **1,081** | 0,175297 | 4,985 | 5,938 |
| KAZN | kazrin, periplakin interacting protein | **1,081** | 0,471311 | 0,831 | 1,915 |
| PHYH | phytanoyl-CoA 2-hydroxylase | **1,081** | 0,082841 | 12,961 | 12,131 |
| ZNF532 | zinc finger protein 532 | **1,081** | 0,092673 | 56,941 | 54,272 |
| EBLN2 | endogenous Bornavirus-like nucleoprotein 2 | **1,081** | 0,741709 | 0,277 | 0,064 |
| ORM1 | orosomucoid 1 | **1,081** | 0,462277 | 4,597 | 1,341 |
| SCD5 | stearoyl-CoA desaturase 5 | **1,081** | 0,626102 | 0,886 | 0,319 |
| SNX4 | sorting nexin 4 | **1,081** | 0,115347 | 15,675 | 13,153 |
| PDLIM7 | PDZ and LIM domain 7 | **1,081** | 0,012612 | 95,825 | 101,649 |
| PEA15 | phosphoprotein enriched in astrocytes 15 | **1,081** | 0,001309 | 137,312 | 144,748 |
| PCYOX1 | prenylcysteine oxidase 1 | **1,081** | 0,012364 | 75,885 | 60,402 |
| UBE2E2 | ubiquitin conjugating enzyme E2 E2 | **1,081** | 0,084254 | 15,842 | 18,963 |
| PPP1R12A | protein phosphatase 1 regulatory subunit 12A | **1,081** | 0,006979 | 47,802 | 49,803 |
| MIER3 | MIER family member 3 | **1,081** | 0,040005 | 17,116 | 19,347 |
| FKBP15 | FK506 binding protein 15 | **1,081** | 0,009757 | 43,758 | 51,974 |
| ZNF28 | zinc finger protein 28 | **1,081** | 0,145343 | 7,644 | 8,747 |
| KIAA0586 | KIAA0586 | **1,081** | 0,17287 | 9,139 | 10,535 |
| FGFR1OP2 | FGFR1 oncogene partner 2 | **1,081** | 0,017901 | 31,406 | 35,373 |
| CUBN | cubilin | **1,081** | 0,355831 | 2,382 | 2,107 |
| LTN1 | listerin E3 ubiquitin protein ligase 1 | **1,081** | 0,04288 | 32,071 | 40,545 |

| | | | | | |
|---|---|---|---|---|---|
| KLF3-AS1 | KLF3 antisense RNA 1 | **1,081** | 0,218076 | 1,662 | 2,554 |
| ZBTB26 | zinc finger and BTB domain containing 26 | **1,081** | 0,112624 | 10,967 | 10,408 |
| EIF1AX | eukaryotic translation initiation factor 1A, X-linked | **1,081** | 0,022617 | 44,811 | 58,231 |
| BOD1L1 | biorientation of chromosomes in cell division 1 like 1 | **1,081** | 0,022233 | 90,009 | 105,288 |
| GPD2 | glycerol-3-phosphate dehydrogenase 2 | **1,081** | 0,048324 | 65,360 | 51,208 |
| YBX1P1 | Y-box binding protein 1 pseudogene 1 | **1,081** | 0,619312 | 0,332 | 0,638 |
| PAXIP1-AS2 | PAXIP1 antisense RNA 2 | **1,081** | 0,248173 | 6,259 | 7,598 |
| DAG1 | dystroglycan 1 | **1,081** | 0,004803 | 102,139 | 97,052 |
| TYMP | thymidine phosphorylase | **1,081** | 0,012001 | 220,287 | 218,814 |
| ATXN1L | ataxin 1 like | **1,081** | 0,069985 | 35,062 | 39,651 |
| ELOA-AS1 | ELOA antisense RNA 1 | **1,081** | 0,36653 | 2,049 | 3,192 |
| MED7 | mediator complex subunit 7 | **1,081** | 0,055065 | 16,728 | 16,218 |
| ZNF761 | zinc finger protein 761 | **1,080** | 0,077503 | 6,038 | 7,917 |
| SLC4A11 | solute carrier family 4 member 11 | **1,080** | 0,306761 | 3,822 | 2,171 |
| CREBL2 | cAMP responsive element binding protein like 2 | **1,080** | 0,004934 | 41,875 | 40,545 |
| MRE11 | MRE11 homolog, double strand break repair nuclease | **1,080** | 0,111343 | 8,752 | 10,535 |
| FGFR2 | fibroblast growth factor receptor 2 | **1,080** | 0,046212 | 21,491 | 33,777 |
| MSI1 | musashi RNA binding protein 1 | **1,080** | 0,614203 | 1,052 | 0,255 |
| ERAP2 | endoplasmic reticulum aminopeptidase 2 | **1,080** | 0,100331 | 3,656 | 50,186 |
| MXD4 | MAX dimerization protein 4 | **1,080** | 0,017555 | 46,140 | 39,715 |
| CHML | CHM like, Rab escort protein 2 | **1,080** | 0,060538 | 9,416 | 12,004 |
| SCAI | suppressor of cancer cell invasion | **1,080** | 0,19565 | 4,043 | 4,661 |
| TCF4 | transcription factor 4 | **1,080** | 0,013736 | 56,276 | 55,805 |
| HSBP1L1 | heat shock factor binding protein 1 like 1 | **1,080** | 0,425614 | 1,052 | 1,405 |
| CENPL | centromere protein L | **1,080** | 0,243316 | 4,431 | 4,469 |
| CNST | consortin, connexin sorting protein | **1,080** | 0,062323 | 22,765 | 24,455 |
| MZF1-AS1 | MZF1 antisense RNA 1 | **1,080** | 0,381866 | 1,717 | 2,937 |
| H3F3B | H3 histone family member 3B | **1,080** | 0,003521 | 451,596 | 602,041 |
| NLGN1 | neuroligin 1 | **1,080** | 0,578823 | 0,443 | 0,830 |
| ATP10A | ATPase phospholipid transporting 10A (putative) | **1,080** | 0,087476 | 11,466 | 14,622 |
| CCDC14 | coiled-coil domain containing 14 | **1,080** | 0,11069 | 8,585 | 11,876 |
| CFH | complement factor H | **1,080** | 0,001306 | 478,128 | 667,870 |
| PPP3CC | protein phosphatase 3 catalytic subunit gamma | **1,080** | 0,014497 | 33,289 | 34,415 |

| | | | | | |
|---|---|---|---|---|---|
| RPL23AP42 | ribosomal protein L23a pseudogene 42 | **1,080** | 0,679877 | 0,222 | 0,383 |
| SAMHD1 | SAM and HD domain containing deoxynucleoside triphosphate triphosphohydrolase 1 | **1,080** | 0,3028 | 3,268 | 2,746 |
| EPC1 | enhancer of polycomb homolog 1 | **1,080** | 0,021304 | 33,400 | 41,183 |
| PODXL | podocalyxin like | **1,080** | 0,188758 | 19,276 | 12,004 |
| SLC41A2 | solute carrier family 41 member 2 | **1,080** | 0,016273 | 54,172 | 39,587 |
| TIFA | TRAF interacting protein with forkhead associated domain | **1,080** | 0,008167 | 64,474 | 69,596 |
| RAD18 | RAD18, E3 ubiquitin protein ligase | **1,079** | 0,139516 | 7,367 | 7,343 |
| ANXA2 | annexin A2 | **1,079** | 0,00389 | 634,217 | 598,018 |
| COL19A1 | collagen type XIX alpha 1 chain | **1,079** | 0,649898 | 0,166 | 0,575 |
| LYSMD4 | LysM domain containing 4 | **1,079** | 0,180465 | 5,705 | 7,854 |
| TSPAN12 | tetraspanin 12 | **1,079** | 0,316375 | 2,936 | 3,384 |
| MIPEP | mitochondrial intermediate peptidase | **1,079** | 0,069473 | 6,481 | 8,364 |
| BTC | betacellulin | **1,079** | 0,185026 | 6,813 | 7,726 |
| TBCEL | tubulin folding cofactor E like | **1,079** | 0,082552 | 13,571 | 13,664 |
| ZSWIM6 | zinc finger SWIM-type containing 6 | **1,079** | 0,080294 | 22,544 | 35,628 |
| DCDC1 | doublecortin domain containing 1 | **1,079** | 0,608697 | 0,388 | 0,830 |
| FBXO10 | F-box protein 10 | **1,079** | 0,358205 | 2,714 | 2,682 |
| H2AFZ | H2A histone family member Z | **1,079** | 0,01363 | 81,977 | 94,562 |
| CILP | cartilage intermediate layer protein | **1,079** | 0,005104 | 545,482 | 1222,981 |
| STAT6 | signal transducer and activator of transcription 6 | **1,079** | 0,003291 | 159,911 | 179,674 |
| NCOA2 | nuclear receptor coactivator 2 | **1,079** | 0,042899 | 31,351 | 30,839 |
| FRG1HP | FSHD region gene 1 family member H, pseudogene | **1,079** | 0,171326 | 5,317 | 6,513 |
| CCDC30 | coiled-coil domain containing 30 | **1,079** | 0,302343 | 1,496 | 2,746 |
| PROB1 | proline rich basic protein 1 | **1,079** | 0,386453 | 2,049 | 2,362 |
| PKIB | cAMP-dependent protein kinase inhibitor beta | **1,079** | 0,627941 | 0,388 | 0,702 |
| PTPRJ | protein tyrosine phosphatase, receptor type J | **1,079** | 0,020883 | 49,408 | 36,203 |
| PPP1R3D | protein phosphatase 1 regulatory subunit 3D | **1,079** | 0,077298 | 11,300 | 11,940 |
| VPS8 | VPS8, CORVET complex subunit | **1,078** | 0,021219 | 21,547 | 25,859 |
| ACOT4 | acyl-CoA thioesterase 4 | **1,078** | 0,30494 | 2,991 | 3,320 |
| CERS6 | ceramide synthase 6 | **1,078** | 0,027207 | 28,581 | 31,989 |
| MTM1 | myotubularin 1 | **1,078** | 0,046948 | 14,568 | 13,345 |
| KDM2B | lysine demethylase 2B | **1,078** | 0,043001 | 12,297 | 11,429 |
| GAB3 | GRB2 associated binding protein 3 | **1,078** | 0,56949 | 0,775 | 1,213 |

| | | | | | |
|---|---|---|---|---|---|
| ZNF473 | zinc finger protein 473 | **1,078** | 0,109473 | 9,306 | 11,557 |
| ADAM17 | ADAM metallopeptidase domain 17 | **1,078** | 0,004656 | 45,365 | 49,037 |
| ZBTB24 | zinc finger and BTB domain containing 24 | **1,078** | 0,063465 | 8,973 | 10,088 |
| FBXO6 | F-box protein 6 | **1,078** | 0,187404 | 6,148 | 6,193 |
| TMEM246 | transmembrane protein 246 | **1,078** | 0,014852 | 16,562 | 20,624 |
| ABI3BP | ABI family member 3 binding protein | **1,078** | 0,049017 | 483,667 | 386,739 |
| CAMSAP2 | calmodulin regulated spectrin associated protein family member 2 | **1,078** | 0,150206 | 60,763 | 54,847 |
| NRBP1 | nuclear receptor binding protein 1 | **1,078** | 0,003779 | 74,223 | 77,514 |
| PNISR | PNN interacting serine and arginine rich protein | **1,078** | 0,026906 | 43,315 | 56,890 |
| TUBGCP6 | tubulin gamma complex associated protein 6 | **1,078** | 0,016737 | 32,182 | 35,884 |
| ZNF148 | zinc finger protein 148 | **1,078** | 0,032602 | 42,429 | 41,247 |
| LRRC8A | leucine rich repeat containing 8 family member A | **1,078** | 0,006401 | 66,524 | 70,235 |
| WASF2 | WAS protein family member 2 | **1,078** | 0,003748 | 188,770 | 210,960 |
| RAB14 | RAB14, member RAS oncogene family | **1,078** | 0,004773 | 118,092 | 136,064 |
| ICE1 | interactor of little elongation complex ELL subunit 1 | **1,078** | 0,061683 | 38,939 | 46,100 |
| THUMPD3-AS1 | THUMPD3 antisense RNA 1 | **1,078** | 0,120792 | 7,921 | 13,281 |
| FAM162A | family with sequence similarity 162 member A | **1,078** | 0,054071 | 20,882 | 22,667 |
| ZBTB5 | zinc finger and BTB domain containing 5 | **1,077** | 0,040464 | 17,171 | 20,240 |
| M6PR | mannose-6-phosphate receptor, cation dependent | **1,077** | 0,007071 | 63,422 | 59,125 |
| SIM2 | single-minded family bHLH transcription factor 2 | **1,077** | 0,05101 | 11,909 | 12,834 |
| C14orf37 | chromosome 14 open reading frame 37 | **1,077** | 0,232503 | 9,472 | 7,981 |
| ZNF630 | zinc finger protein 630 | **1,077** | 0,352163 | 2,437 | 2,107 |
| IDNK | IDNK, gluconokinase | **1,077** | 0,299827 | 2,216 | 2,362 |
| DENND1B | DENN domain containing 1B | **1,077** | 0,383043 | 2,659 | 2,171 |
| BPTF | bromodomain PHD finger transcription factor | **1,077** | 0,058794 | 55,058 | 66,085 |
| SEMA4F | ssemaphorin 4F | **1,077** | 0,053128 | 20,605 | 22,858 |
| SERPINB4 | serpin family B member 4 | **1,077** | 0,504549 | 0,720 | 2,107 |
| KIAA0232 | KIAA0232 | **1,077** | 0,034301 | 56,166 | 56,379 |
| PTPN1 | protein tyrosine phosphatase, non-receptor type 1 | **1,077** | 0,007638 | 50,460 | 59,061 |
| SRSF4 | serine and arginine rich splicing factor 4 | **1,077** | 0,006324 | 50,183 | 61,232 |
| CARF | calcium responsive transcription factor | **1,077** | 0,322925 | 5,041 | 4,533 |
| MYO9A | myosin IXA | **1,077** | 0,025549 | 41,210 | 47,313 |
| MFSD6 | major facilitator superfamily domain containing 6 | **1,077** | 0,107519 | 8,530 | 13,408 |

| | | | | | |
|---|---|---|---|---|---|
| ACADL | acyl-CoA dehydrogenase, long chain | **1,077** | 0,598347 | 0,720 | 0,511 |
| ZNF37A | zinc finger protein 37A | **1,077** | 0,042312 | 18,888 | 21,198 |
| MME | membrane metalloendopeptidase | **1,077** | 0,274754 | 3,434 | 9,833 |
| ADAMTSL5 | ADAMTS like 5 | **1,077** | 0,704526 | 0,332 | 0,128 |
| C6orf106 | chromosome 6 open reading frame 106 | **1,077** | 0,010876 | 87,406 | 90,986 |
| ZNF736 | zinc finger protein 736 | **1,077** | 0,201667 | 7,921 | 2,873 |
| ZNF408 | zinc finger protein 408 | **1,077** | 0,080982 | 12,020 | 12,834 |
| RHBDD2 | rhomboid domain containing 2 | **1,077** | 0,012704 | 39,272 | 49,803 |
| ZNF45 | zinc finger protein 45 | **1,077** | 0,09514 | 8,198 | 8,684 |
| PAPD4 | poly(A) RNA polymerase D4, non-canonical | **1,077** | 0,038281 | 24,261 | 25,604 |
| POC1B | POC1 centriolar protein B | **1,077** | 0,043908 | 13,294 | 13,792 |
| CDCA7L | cell division cycle associated 7 like | **1,077** | 0,150937 | 4,431 | 5,555 |
| ZNF319 | zinc finger protein 319 | **1,077** | 0,025525 | 16,119 | 17,559 |
| GOLGA8A | golgin A8 family member A | **1,077** | 0,14908 | 8,530 | 12,451 |
| SCYL2 | SCY1 like pseudokinase 2 | **1,077** | 0,027853 | 43,149 | 40,672 |
| CARD8 | caspase recruitment domain family member 8 | **1,077** | 0,073181 | 12,574 | 13,472 |
| MIGA1 | mitoguardin 1 | **1,077** | 0,0698 | 21,824 | 20,560 |
| RPS7P1 | ribosomal protein S7 pseudogene 1 | **1,077** | 0,469448 | 1,108 | 0,894 |
| NETO2 | neuropilin and tolloid like 2 | **1,077** | 0,598752 | 1,108 | 1,149 |
| CCND3 | cyclin D3 | **1,077** | 0,00799 | 36,447 | 45,014 |
| RAB39B | RAB39B, member RAS oncogene family | **1,076** | 0,433496 | 1,994 | 2,043 |
| EAPP | E2F associated phosphoprotein | **1,076** | 0,03815 | 22,544 | 23,561 |
| SVIL-AS1 | SVIL antisense RNA 1 | **1,076** | 0,031368 | 27,030 | 22,731 |
| RNF20 | ring finger protein 20 | **1,076** | 0,014415 | 39,161 | 46,227 |
| GCC1 | GRIP and coiled-coil domain containing 1 | **1,076** | 0,008095 | 42,263 | 46,483 |
| GPR19 | G protein-coupled receptor 19 | **1,076** | 0,391561 | 1,939 | 3,001 |
| TBC1D8 | TBC1 domain family member 8 | **1,076** | 0,177313 | 7,145 | 9,833 |
| PLBD1-AS1 | PLBD1 antisense RNA 1 | **1,076** | 0,707397 | 0,609 | 0,702 |
| UNC13A | unc-13 homolog A | **1,076** | 0,118297 | 6,702 | 14,877 |
| CLDND1 | claudin domain containing 1 | **1,076** | 0,013238 | 64,363 | 59,827 |
| YY1 | YY1 transcription factor | **1,076** | 0,005751 | 78,045 | 76,301 |
| CLIC4 | chloride intracellular channel 4 | **1,076** | 0,008464 | 828,914 | 853,673 |
| MED13 | mediator complex subunit 13 | **1,076** | 0,108614 | 74,167 | 73,938 |

| | | | | | |
|---|---|---|---|---|---|
| MCM6 | minichromosome maintenance complex component 6 | **1,076** | 0,024708 | 21,547 | 21,837 |
| KLKB1 | kallikrein B1 | **1,076** | 0,517851 | 0,665 | 1,532 |
| MST1R | macrophage stimulating 1 receptor | **1,076** | 0,159559 | 3,600 | 5,427 |
| PPP1R15A | protein phosphatase 1 regulatory subunit 15A | **1,076** | 0,001998 | 388,230 | 448,290 |
| USP37 | ubiquitin specific peptidase 37 | **1,076** | 0,053033 | 13,238 | 19,538 |
| TJP1 | tight junction protein 1 | **1,076** | 0,655417 | 0,388 | 0,638 |
| LPAR1 | lysophosphatidic acid receptor 1 | **1,076** | 0,008473 | 246,652 | 133,063 |
| CUL3 | cullin 3 | **1,076** | 0,004043 | 61,594 | 69,277 |
| VWA5A | von Willebrand factor A domain containing 5A | **1,076** | 0,060665 | 8,973 | 9,961 |
| HP1BP3 | heterochromatin protein 1 binding protein 3 | **1,076** | 0,015234 | 124,240 | 111,354 |
| RAB43 | RAB43, member RAS oncogene family | **1,076** | 0,440793 | 2,160 | 1,405 |
| CROT | carnitine O-octanoyltransferase | **1,076** | 0,086534 | 10,192 | 9,386 |
| CCNB3 | cyclin B3 | **1,076** | 0,653849 | 0,499 | 0,383 |
| SCARA3 | scavenger receptor class A member 3 | **1,076** | 0,008693 | 199,958 | 253,101 |
| LRP5L | LDL receptor related protein 5 like | **1,076** | 0,356897 | 1,329 | 2,682 |
| KDM3B | lysine demethylase 3B | **1,076** | 0,041927 | 50,959 | 51,655 |
| PSAP | prosaposin | **1,076** | 0,005268 | 1843,052 | 1888,679 |
| MGARP | mitochondria localized glutamic acid rich protein | **1,076** | 0,69906 | 0,332 | 0,255 |
| SLC25A53 | solute carrier family 25 member 53 | **1,076** | 0,346178 | 3,157 | 2,618 |
| AGO3 | argonaute 3, RISC catalytic component | **1,076** | 0,063077 | 19,442 | 21,645 |
| KIAA1614 | KIAA1614 | **1,076** | 0,233767 | 5,594 | 5,236 |
| ZNF25 | zinc finger protein 25 | **1,076** | 0,07959 | 13,848 | 13,345 |
| RAD51AP1 | RAD51 associated protein 1 | **1,076** | 0,589104 | 0,997 | 0,319 |
| SLC12A6 | solute carrier family 12 member 6 | **1,076** | 0,077974 | 71,398 | 74,832 |
| SNRNP48 | small nuclear ribonucleoprotein U11/U12 subunit 48 | **1,076** | 0,089398 | 10,192 | 9,194 |
| PRKXP1 | protein kinase, X-linked, pseudogene 1 | **1,075** | 0,700794 | 0,443 | 0,702 |
| TTC13 | tetratricopeptide repeat domain 13 | **1,075** | 0,071826 | 12,906 | 15,516 |
| MRPL38 | mitochondrial ribosomal protein L38 | **1,075** | 0,438381 | 1,385 | 2,235 |
| PLXND1 | plexin D1 | **1,075** | 0,117644 | 11,577 | 8,045 |
| LINC01376 | long intergenic non-protein coding RNA 1376 | **1,075** | 0,577297 | 0,443 | 0,958 |
| GYS1 | glycogen synthase 1 | **1,075** | 0,021836 | 28,747 | 31,350 |
| TRIM38 | tripartite motif containing 38 | **1,075** | 0,020816 | 27,197 | 30,903 |
| PDK4 | pyruvate dehydrogenase kinase 4 | **1,075** | 0,35442 | 3,434 | 3,639 |

| | | | | | |
|---|---|---|---|---|---|
| WEE1 | WEE1 G2 checkpoint kinase | **1,075** | 0,068399 | 13,626 | 7,790 |
| STAU2 | staufen double-stranded RNA binding protein 2 | **1,075** | 0,152192 | 8,198 | 6,896 |
| NDFIP1 | Nedd4 family interacting protein 1 | **1,075** | 0,006327 | 181,735 | 196,785 |
| TSC1 | tuberous sclerosis 1 | **1,075** | 0,043165 | 38,330 | 44,631 |
| COL9A2 | collagen type IX alpha 2 chain | **1,075** | 0,037483 | 178,522 | 88,496 |
| SERPINA1 | serpin family A member 1 | **1,075** | 0,057529 | 1346,258 | 979,138 |
| CCNL2 | cyclin L2 | **1,075** | 0,009659 | 63,256 | 78,472 |
| BICRAL | BRD4 interacting chromatin remodelling complex associated protein like | **1,075** | 0,172026 | 19,608 | 22,794 |
| PTMA | prothymosin, alpha | **1,075** | 0,042739 | 153,265 | 155,219 |
| ATP6V0E1 | ATPase H+ transporting V0 subunit e1 | **1,075** | 0,024106 | 105,740 | 98,903 |
| LRP1 | LDL receptor related protein 1 | **1,075** | 0,057005 | 1629,634 | 1586,670 |
| VPS13A | vacuolar protein sorting 13 homolog A | **1,075** | 0,167716 | 40,878 | 44,312 |
| SRSF5 | serine and arginine rich splicing factor 5 | **1,075** | 0,004561 | 76,217 | 101,713 |
| ARID4A | AT-rich interaction domain 4A | **1,075** | 0,08676 | 20,661 | 24,327 |
| HAUS4 | HAUS augmin like complex subunit 4 | **1,075** | 0,274447 | 5,373 | 4,597 |
| MIR193BHG | MIR193B host gene | **1,075** | 0,224963 | 4,265 | 3,448 |
| MYH7B | myosin heavy chain 7B | **1,075** | 0,622868 | 0,222 | 1,341 |
| PHF3 | PHD finger protein 3 | **1,075** | 0,048268 | 88,292 | 81,600 |
| FANCF | Fanconi anemia complementation group F | **1,075** | 0,136348 | 8,585 | 9,897 |
| TTLL7 | tubulin tyrosine ligase like 7 | **1,075** | 0,186813 | 9,250 | 7,470 |
| CHST2 | carbohydrate sulfotransferase 2 | **1,075** | 0,012648 | 114,935 | 76,939 |
| GAN | gigaxonin | **1,075** | 0,247784 | 7,865 | 10,216 |
| NAALADL2 | N-acetylated alpha-linked acidic dipeptidase like 2 | **1,075** | 0,561801 | 2,049 | 1,213 |
| ATF7IP2 | activating transcription factor 7 interacting protein 2 | **1,075** | 0,355535 | 2,770 | 3,384 |
| SPESP1 | sperm equatorial segment protein 1 | **1,074** | 0,487658 | 1,274 | 1,085 |
| SPSB2 | splA/ryanodine receptor domain and SOCS box containing 2 | **1,074** | 0,14553 | 7,921 | 4,853 |
| TF | transferrin | **1,074** | 0,004354 | 76,605 | 154,325 |
| CCDC191 | coiled-coil domain containing 191 | **1,074** | 0,245161 | 4,320 | 3,959 |
| PDZD4 | PDZ domain containing 4 | **1,074** | 0,541826 | 0,609 | 0,894 |
| LRP12 | LDL receptor related protein 12 | **1,074** | 0,021516 | 36,114 | 42,907 |
| CDK14 | cyclin dependent kinase 14 | **1,074** | 0,037296 | 19,110 | 21,326 |
| NCAM1 | neural cell adhesion molecule 1 | **1,074** | 0,328787 | 6,038 | 4,916 |
| ZNF420 | zinc finger protein 420 | **1,074** | 0,109656 | 8,087 | 9,897 |

| | | | | | |
|---|---|---|---|---|---|
| SYK | spleen associated tyrosine kinase | **1,074** | 0,609435 | 0,277 | 0,702 |
| SMIM4 | small integral membrane protein 4 | **1,074** | 0,320703 | 3,711 | 4,916 |
| CLCN7 | chloride voltage-gated channel 7 | **1,074** | 0,00646 | 66,025 | 71,193 |
| ADARB2 | adenosine deaminase, RNA specific B2 (inactive) | **1,074** | 0,647764 | 0,277 | 0,000 |
| METTL14 | methyltransferase like 14 | **1,074** | 0,025619 | 19,664 | 24,008 |
| PDPR | pyruvate dehydrogenase phosphatase regulatory subunit | **1,074** | 0,058604 | 47,746 | 47,377 |
| SEC14L6 | SEC14 like lipid binding 6 | **1,074** | 0,556258 | 1,052 | 1,788 |
| NPHP1 | nephrocystin 1 | **1,074** | 0,192003 | 4,764 | 5,236 |
| GPATCH2 | G-patch domain containing 2 | **1,074** | 0,075517 | 13,183 | 17,942 |
| RTEL1 | regulator of telomere elongation helicase 1 | **1,074** | 0,688547 | 0,166 | 0,575 |
| POU6F1 | POU class 6 homeobox 1 | **1,074** | 0,093335 | 8,973 | 10,024 |
| MESP1 | mesoderm posterior bHLH transcription factor 1 | **1,074** | 0,686951 | 0,499 | 0,383 |
| CRY1 | cryptochrome circadian clock 1 | **1,074** | 0,011918 | 34,896 | 44,312 |
| LINC01258 | long intergenic non-protein coding RNA 1258 | **1,074** | 0,708156 | 0,000 | 0,319 |
| KCTD1 | potassium channel tetramerization domain containing 1 | **1,074** | 0,325509 | 3,545 | 5,427 |
| PRKAB2 | protein kinase AMP-activated non-catalytic subunit beta 2 | **1,074** | 0,047557 | 19,442 | 24,327 |
| PPP6R3 | protein phosphatase 6 regulatory subunit 3 | **1,074** | 0,006673 | 62,314 | 70,235 |
| TSPAN3 | tetraspanin 3 | **1,074** | 0,007596 | 148,556 | 118,314 |
| DCP1A | decapping mRNA 1A | **1,074** | 0,008769 | 34,508 | 45,397 |
| FAM122B | family with sequence similarity 122B | **1,074** | 0,053788 | 16,672 | 16,346 |
| HMGN3-AS1 | HMGN3 antisense RNA 1 | **1,074** | 0,513292 | 1,717 | 2,299 |
| STOX2 | storkhead box 2 | **1,074** | 0,316417 | 1,828 | 3,767 |
| FAM76A | family with sequence similarity 76 member A | **1,074** | 0,116363 | 7,367 | 7,917 |
| SSX2IP | SSX family member 2 interacting protein | **1,074** | 0,3207 | 4,376 | 4,725 |
| SERPINB9 | serpin family B member 9 | **1,074** | 0,213514 | 7,145 | 8,428 |
| MT-ND6 | mitochondrially encoded NADH:ubiquinone oxidoreductase core subunit 6 | **1,074** | 0,406242 | 24,482 | 30,839 |
| CHD1 | chromodomain helicase DNA binding protein 1 | **1,074** | 0,090058 | 44,866 | 50,122 |
| PTER | phosphotriesterase related | **1,074** | 0,126785 | 6,647 | 8,109 |
| ALS2 | ALS2, alsin Rho guanine nucleotide exchange factor | **1,074** | 0,046055 | 19,331 | 23,624 |
| SLC45A4 | solute carrier family 45 member 4 | **1,074** | 0,109175 | 8,419 | 9,577 |
| KDM6A | lysine demethylase 6A | **1,073** | 0,053758 | 37,278 | 46,483 |
| CSTF3 | cleavage stimulation factor subunit 3 | **1,073** | 0,040629 | 16,174 | 21,709 |
| TMEM184C | transmembrane protein 184C | **1,073** | 0,048152 | 36,336 | 39,012 |

| | | | | | |
|---|---|---|---|---|---|
| PUM2 | pumilio RNA binding family member 2 | **1,073** | 0,016861 | 99,204 | 107,842 |
| ANKRD40 | ankyrin repeat domain 40 | **1,073** | 0,037461 | 36,336 | 36,458 |
| PTBP3 | polypyrimidine tract binding protein 3 | **1,073** | 0,053617 | 55,390 | 44,184 |
| TFRC | transferrin receptor | **1,073** | 0,024157 | 391,498 | 377,800 |
| HTR7P1 | 5-hydroxytryptamine receptor 7 pseudogene 1 | **1,073** | 0,275596 | 8,032 | 9,514 |
| PCOLCE2 | procollagen C-endopeptidase enhancer 2 | **1,073** | 0,003787 | 1239,743 | 1004,550 |
| FYCO1 | FYVE and coiled-coil domain containing 1 | **1,073** | 0,138936 | 33,234 | 38,629 |
| SLC26A1 | solute carrier family 26 member 1 | **1,073** | 0,466772 | 1,883 | 1,979 |
| ZNF317 | zinc finger protein 317 | **1,073** | 0,012355 | 26,421 | 31,542 |
| NAB2 | NGFI-A binding protein 2 | **1,073** | 0,019898 | 52,011 | 53,059 |
| OXCT1 | 3-oxoacid CoA-transferase 1 | **1,073** | 0,129289 | 23,098 | 19,283 |
| TBCC | tubulin folding cofactor C | **1,073** | 0,010366 | 29,800 | 36,075 |
| MAPKAPK2 | mitogen-activated protein kinase-activated protein kinase 2 | **1,073** | 0,009298 | 129,114 | 129,679 |
| GDAP1 | ganglioside induced differentiation associated protein 1 | **1,073** | 0,053193 | 17,836 | 24,774 |
| ZNF727 | zinc finger protein 727 | **1,073** | 0,733594 | 0,388 | 0,575 |
| TMC7 | transmembrane channel like 7 | **1,073** | 0,262896 | 8,918 | 7,726 |
| GIGYF2 | GRB10 interacting GYF protein 2 | **1,073** | 0,023604 | 67,631 | 76,428 |
| KIF27 | kinesin family member 27 | **1,073** | 0,292087 | 3,877 | 6,066 |
| MMD | monocyte to macrophage differentiation associated | **1,073** | 0,25691 | 6,038 | 3,320 |
| LRRFIP2 | LRR binding FLII interacting protein 2 | **1,073** | 0,010522 | 44,589 | 38,501 |
| TTI1 | TELO2 interacting protein 1 | **1,073** | 0,050166 | 14,845 | 16,729 |
| TPI1P1 | triosephosphate isomerase 1 pseudogene 1 | **1,073** | 0,614586 | 0,499 | 0,383 |
| PAXBP1-AS1 | PAXBP1 antisense RNA 1 | **1,073** | 0,405603 | 1,329 | 2,235 |
| KIF2A | kinesin family member 2A | **1,073** | 0,052462 | 21,935 | 21,517 |
| SH3GLB2 | SH3 domain containing GRB2 like, endophilin B2 | **1,073** | 0,080588 | 19,553 | 21,454 |
| KCNG1 | potassium voltage-gated channel modifier subfamily G member 1 | **1,073** | 0,200977 | 4,985 | 8,556 |
| USP12 | ubiquitin specific peptidase 12 | **1,073** | 0,01251 | 83,584 | 74,066 |
| APOBEC3F | apolipoprotein B mRNA editing enzyme catalytic subunit 3F | **1,073** | 0,612975 | 0,942 | 1,341 |
| GABARAPL1 | GABA type A receptor associated protein like 1 | **1,073** | 0,007899 | 101,530 | 97,116 |
| STIP1 | stress induced phosphoprotein 1 | **1,073** | 0,010774 | 92,003 | 107,459 |
| CIPC | CLOCK interacting pacemaker | **1,073** | 0,080997 | 10,912 | 12,259 |
| MRPL50 | mitochondrial ribosomal protein L50 | **1,073** | 0,074024 | 19,220 | 22,539 |
| USP32 | ubiquitin specific peptidase 32 | **1,073** | 0,053992 | 33,068 | 36,331 |

| | | | | | |
|---|---|---|---|---|---|
| TM9SF2 | transmembrane 9 superfamily member 2 | **1,073** | 0,010563 | 216,520 | 196,211 |
| 7.syys | septin 7 | **1,073** | 0,009161 | 115,932 | 116,526 |
| EIF4A3 | eukaryotic translation initiation factor 4A3 | **1,073** | 0,016011 | 94,329 | 117,548 |
| SLC9B2 | solute carrier family 9 member B2 | **1,073** | 0,030774 | 33,511 | 31,414 |
| TMX4 | thioredoxin related transmembrane protein 4 | **1,072** | 0,015658 | 209,098 | 180,057 |
| RIF1 | replication timing regulatory factor 1 | **1,072** | 0,037358 | 53,064 | 67,042 |
| TMEM70 | transmembrane protein 70 | **1,072** | 0,041298 | 20,384 | 23,816 |
| VIT | vitrin | **1,072** | 0,450094 | 1,274 | 0,894 |
| CCDC186 | coiled-coil domain containing 186 | **1,072** | 0,026957 | 24,649 | 31,223 |
| SNX8 | sorting nexin 8 | **1,072** | 0,035389 | 41,487 | 44,376 |
| MARS2 | methionyl-tRNA synthetase 2, mitochondrial | **1,072** | 0,179337 | 4,542 | 6,704 |
| STAG2 | stromal antigen 2 | **1,072** | 0,023387 | 121,304 | 134,212 |
| EP300 | E1A binding protein p300 | **1,072** | 0,163591 | 101,530 | 117,931 |
| PABPC1L | poly(A) binding protein cytoplasmic 1 like | **1,072** | 0,199701 | 5,373 | 6,385 |
| ZFP90 | ZFP90 zinc finger protein | **1,072** | 0,088431 | 17,116 | 19,347 |
| VTI1A | vesicle transport through interaction with t-SNAREs 1A | **1,072** | 0,036064 | 21,270 | 26,178 |
| CERNA1 | competing endogenous lncRNA 1 for miR-4707-5p and miR-4767 | **1,072** | 0,665283 | 0,554 | 0,447 |
| ZNF766 | zinc finger protein 766 | **1,072** | 0,041965 | 12,574 | 15,069 |
| LAMP1 | lysosomal associated membrane protein 1 | **1,072** | 0,002829 | 406,398 | 348,301 |
| HTRA3 | HtrA serine peptidase 3 | **1,072** | 0,172003 | 17,060 | 13,600 |
| ORAOV1 | oral cancer overexpressed 1 | **1,072** | 0,062563 | 12,186 | 15,516 |
| NANOS1 | nanos C2HC-type zinc finger 1 | **1,072** | 0,053748 | 24,261 | 22,092 |
| LRIF1 | ligand dependent nuclear receptor interacting factor 1 | **1,072** | 0,0733 | 22,932 | 21,007 |
| ANKRD13A | ankyrin repeat domain 13A | **1,072** | 0,067633 | 19,276 | 21,134 |
| SDHAF4 | succinate dehydrogenase complex assembly factor 4 | **1,072** | 0,24682 | 4,376 | 4,916 |
| CDV3 | CDV3 homolog | **1,072** | 0,007296 | 147,061 | 152,729 |
| GPR27 | G protein-coupled receptor 27 | **1,072** | 0,265605 | 3,877 | 8,109 |
| POLR2M | RNA polymerase II subunit M | **1,072** | 0,116084 | 19,719 | 18,516 |
| DBF4 | DBF4 zinc finger | **1,072** | 0,265517 | 4,154 | 5,044 |
| CDC42EP3 | CDC42 effector protein 3 | **1,072** | 0,030013 | 45,863 | 60,083 |
| SLFN11 | schlafen family member 11 | **1,072** | 0,162009 | 2,825 | 5,427 |
| TRIM45 | tripartite motif containing 45 | **1,072** | 0,455379 | 1,219 | 1,915 |
| ABCA6 | ATP binding cassette subfamily A member 6 | **1,072** | 0,588203 | 0,554 | 1,085 |

| | | | | | |
|---|---|---|---|---|---|
| RPL7P9 | ribosomal protein L7 pseudogene 9 | **1,072** | 0,501972 | 2,493 | 1,788 |
| CTR9 | CTR9 homolog, Paf1/RNA polymerase II complex component | **1,072** | 0,01077 | 59,434 | 73,044 |
| UTRN | utrophin | **1,072** | 0,195873 | 43,647 | 40,225 |
| THAP12 | THAP domain containing 12 | **1,072** | 0,023157 | 24,261 | 25,795 |
| FAM199X | family with sequence similarity 199, X-linked | **1,072** | 0,092383 | 17,946 | 16,026 |
| DCAF5 | DDB1 and CUL4 associated factor 5 | **1,071** | 0,038054 | 37,333 | 41,630 |
| KTN1-AS1 | KTN1 antisense RNA 1 | **1,071** | 0,492888 | 1,052 | 1,915 |
| B2M | beta-2-microglobulin | **1,071** | 0,024543 | 59,157 | 57,465 |
| TMOD2 | tropomodulin 2 | **1,071** | 0,318285 | 8,087 | 5,746 |
| CCAR1 | cell division cycle and apoptosis regulator 1 | **1,071** | 0,03874 | 57,661 | 63,275 |
| FSTL3 | follistatin like 3 | **1,071** | 0,265604 | 15,011 | 6,257 |
| MAPK7 | mitogen-activated protein kinase 7 | **1,071** | 0,013187 | 26,144 | 28,477 |
| TIMP1 | TIMP metallopeptidase inhibitor 1 | **1,071** | 0,131241 | 1493,042 | 1147,191 |
| PATL1 | PAT1 homolog 1, processing body mRNA decay factor | **1,071** | 0,065851 | 41,543 | 43,035 |
| XRN1 | 5'-3' exoribonuclease 1 | **1,071** | 0,186477 | 40,878 | 43,737 |
| C3orf38 | chromosome 3 open reading frame 38 | **1,071** | 0,02 | 19,110 | 20,177 |
| VPS13B | vacuolar protein sorting 13 homolog B | **1,071** | 0,14681 | 25,424 | 29,946 |
| TEN1-CDK3 | TEN1-CDK3 readthrough (NMD candidate) | **1,071** | 0,716654 | 1,163 | 0,575 |
| TNK2 | tyrosine kinase non receptor 2 | **1,071** | 0,014206 | 41,653 | 47,824 |
| MAP3K20 | mitogen-activated protein kinase kinase kinase 20 | **1,071** | 0,072085 | 29,911 | 27,966 |
| SKP2 | S-phase kinase associated protein 2 | **1,071** | 0,202742 | 5,262 | 6,896 |
| SPRED3 | sprouty related EVH1 domain containing 3 | **1,071** | 0,193774 | 9,804 | 10,599 |
| TMEM54 | transmembrane protein 54 | **1,071** | 0,167984 | 13,349 | 10,791 |
| TOGARAM1 | TOG array regulator of axonemal microtubules 1 | **1,071** | 0,09572 | 22,765 | 25,795 |
| ABTB1 | ankyrin repeat and BTB domain containing 1 | **1,071** | 0,03754 | 35,948 | 33,713 |
| WASHC5 | WASH complex subunit 5 | **1,071** | 0,018193 | 32,902 | 34,160 |
| LINC00998 | long intergenic non-protein coding RNA 998 | **1,071** | 0,217251 | 12,241 | 11,812 |
| ERBB3 | erb-b2 receptor tyrosine kinase 3 | **1,071** | 0,737191 | 0,443 | 0,830 |
| ZNF354A | zinc finger protein 354A | **1,071** | 0,126356 | 5,761 | 7,534 |
| MANEA | mannosidase endo-alpha | **1,071** | 0,155942 | 10,856 | 11,238 |
| ARFGEF3 | ARFGEF family member 3 | **1,071** | 0,376977 | 2,216 | 4,214 |
| PRKX | protein kinase, X-linked | **1,071** | 0,411541 | 2,880 | 1,213 |
| BLOC1S5 | biogenesis of lysosomal organelles complex 1 subunit 5 | **1,071** | 0,063331 | 16,672 | 17,303 |

| | | | | | |
|---|---|---|---|---|---|
| SYNE1 | spectrin repeat containing nuclear envelope protein 1 | **1,071** | 0,205056 | 142,796 | 254,761 |
| NCR3LG1 | natural killer cell cytotoxicity receptor 3 ligand 1 | **1,071** | 0,363702 | 5,096 | 6,704 |
| LINC00959 | long intergenic non-protein coding RNA 959 | **1,071** | 0,243682 | 2,659 | 4,789 |
| NUP58 | nucleoporin 58 | **1,071** | 0,046722 | 53,839 | 68,894 |
| PMEL | premelanosome protein | **1,071** | 0,636707 | 1,108 | 0,766 |
| GAL3ST1 | galactose-3-O-sulfotransferase 1 | **1,071** | 0,671645 | 0,332 | 0,894 |
| C1QTNF3 | C1q and TNF related 3 | **1,071** | 0,175721 | 7,035 | 1,596 |
| SLC35A3 | solute carrier family 35 member A3 | **1,071** | 0,06474 | 18,999 | 22,858 |
| ZNF451 | zinc finger protein 451 | **1,071** | 0,054455 | 31,295 | 34,862 |
| THNSL1 | threonine synthase like 1 | **1,071** | 0,058806 | 13,183 | 17,112 |
| SLC38A3 | solute carrier family 38 member 3 | **1,070** | 0,159475 | 7,699 | 7,981 |
| CEP126 | centrosomal protein 126 | **1,070** | 0,125677 | 8,696 | 12,004 |
| APLF | aprataxin and PNKP like factor | **1,070** | 0,212752 | 5,262 | 4,278 |
| DRAM2 | DNA damage regulated autophagy modulator 2 | **1,070** | 0,045451 | 15,786 | 12,770 |
| CBX6 | chromobox 6 | **1,070** | 0,030168 | 60,874 | 52,102 |
| SCAPER | S-phase cyclin A associated protein in the ER | **1,070** | 0,217533 | 6,425 | 5,363 |
| AMMECR1 | Alport syndrome, mental retardation, midface hypoplasia and elliptocytosis chromosomal | **1,070** | 0,096841 | 10,967 | 12,706 |
| TSPAN2 | tetraspanin 2 | **1,070** | 0,349764 | 3,157 | 2,618 |
| SEC14L1 | SEC14 like lipid binding 1 | **1,070** | 0,021604 | 74,112 | 69,724 |
| TYW1B | tRNA-yW synthesizing protein 1 homolog B | **1,070** | 0,354356 | 3,988 | 1,149 |
| SPRY2 | sprouty RTK signaling antagonist 2 | **1,070** | 0,021417 | 113,993 | 100,117 |
| STK32C | serine/threonine kinase 32C | **1,070** | 0,238726 | 5,428 | 6,513 |
| EIF5 | eukaryotic translation initiation factor 5 | **1,070** | 0,009133 | 227,432 | 244,737 |
| KPNA3 | karyopherin subunit alpha 3 | **1,070** | 0,051273 | 32,403 | 26,434 |
| TTN-AS1 | TTN antisense RNA 1 | **1,070** | 0,153641 | 3,545 | 8,109 |
| CFI | complement factor I | **1,070** | 0,619142 | 0,388 | 0,511 |
| ZNF701 | zinc finger protein 701 | **1,070** | 0,207802 | 5,594 | 7,151 |
| MINDY1 | MINDY lysine 48 deubiquitinase 1 | **1,070** | 0,196994 | 5,650 | 5,938 |
| TADA1 | transcriptional adaptor 1 | **1,070** | 0,208084 | 7,090 | 7,598 |
| INAFM2 | InaF motif containing 2 | **1,070** | 0,108342 | 11,798 | 11,685 |
| TBCCD1 | TBCC domain containing 1 | **1,070** | 0,226815 | 6,868 | 9,322 |
| TPRG1 | tumor protein p63 regulated 1 | **1,070** | 0,49241 | 1,052 | 1,532 |
| RBM5 | RNA binding motif protein 5 | **1,070** | 0,014836 | 62,148 | 74,130 |

| | | | | | |
|---|---|---|---|---|---|
| ZNF703 | zinc finger protein 703 | **1,070** | 0,024165 | 30,963 | 33,521 |
| FAM212B-AS1 | FAM212B antisense RNA 1 | **1,070** | 0,701221 | 0,388 | 0,383 |
| CPD | carboxypeptidase D | **1,070** | 0,020669 | 310,517 | 221,176 |
| FGFRL1 | fibroblast growth factor receptor-like 1 | **1,070** | 0,019021 | 370,394 | 262,231 |
| UTP23 | UTP23, small subunit processome component | **1,069** | 0,028702 | 18,556 | 21,070 |
| NXPH4 | neurexophilin 4 | **1,069** | 0,095796 | 24,870 | 17,176 |
| FAM177B | family with sequence similarity 177 member B | **1,069** | 0,728828 | 0,222 | 0,638 |
| EP400NL | EP400 N-terminal like | **1,069** | 0,2361 | 10,192 | 14,749 |
| SNX13 | sorting nexin 13 | **1,069** | 0,05053 | 41,155 | 42,077 |
| OPTN | optineurin | **1,069** | 0,010226 | 154,428 | 113,206 |
| ATP6V0D1 | ATPase H+ transporting V0 subunit d1 | **1,069** | 0,047375 | 50,128 | 50,633 |
| SDE2 | SDE2 telomere maintenance homolog | **1,069** | 0,019211 | 30,465 | 36,203 |
| RPL10P6 | ribosomal protein L10 pseudogene 6 | **1,069** | 0,658228 | 0,499 | 0,447 |
| MVP | major vault protein | **1,069** | 0,021726 | 136,814 | 96,860 |
| TBC1D4 | TBC1 domain family member 4 | **1,069** | 0,143261 | 14,235 | 13,792 |
| PLAGL2 | PLAG1 like zinc finger 2 | **1,069** | 0,135645 | 17,503 | 20,432 |
| E2F6 | E2F transcription factor 6 | **1,069** | 0,165041 | 7,035 | 8,684 |
| ZDHHC21 | zinc finger DHHC-type containing 21 | **1,069** | 0,115301 | 16,728 | 16,409 |
| SRSF10 | serine and arginine rich splicing factor 10 | **1,069** | 0,016878 | 50,793 | 68,894 |
| DCLRE1B | DNA cross-link repair 1B | **1,069** | 0,145227 | 5,816 | 6,130 |
| KDM4A | lysine demethylase 4A | **1,069** | 0,075758 | 14,235 | 13,664 |
| DEF6 | DEF6, guanine nucleotide exchange factor | **1,069** | 0,568193 | 0,720 | 1,022 |
| DTNBP1 | dystrobrevin binding protein 1 | **1,069** | 0,151346 | 10,026 | 8,237 |
| FRYL | FRY like transcription coactivator | **1,069** | 0,146644 | 50,239 | 54,209 |
| LINC00674 | long intergenic non-protein coding RNA 674 | **1,069** | 0,363636 | 3,046 | 3,959 |
| POU2F1 | POU class 2 homeobox 1 | **1,069** | 0,10731 | 16,119 | 18,516 |
| WLS | wntless Wnt ligand secretion mediator | **1,069** | 0,056085 | 28,526 | 29,499 |
| LAMTOR4 | late endosomal/lysosomal adaptor, MAPK and MTOR activator 4 | **1,069** | 0,066554 | 24,593 | 24,391 |
| C15orf40 | chromosome 15 open reading frame 40 | **1,069** | 0,088968 | 11,853 | 13,281 |
| PRR11 | proline rich 11 | **1,069** | 0,64918 | 4,210 | 0,575 |
| CBLL1 | Cbl proto-oncogene like 1 | **1,069** | 0,081328 | 15,398 | 17,686 |
| EFHD1 | EF-hand domain family member D1 | **1,069** | 0,136888 | 11,964 | 10,535 |
| PDS5B | PDS5 cohesin associated factor B | **1,069** | 0,042006 | 35,450 | 42,013 |

| | | | | | |
|---|---|---|---|---|---|
| MLLT3 | MLLT3, super elongation complex subunit | **1,069** | 0,337547 | 6,314 | 7,087 |
| ZNF799 | zinc finger protein 799 | **1,069** | 0,320284 | 2,770 | 3,129 |
| SNIP1 | Smad nuclear interacting protein 1 | **1,069** | 0,016049 | 28,194 | 30,009 |
| RBM26-AS1 | RBM26 antisense RNA 1 | **1,069** | 0,319299 | 2,548 | 4,406 |
| GCAT | glycine C-acetyltransferase | **1,069** | 0,07237 | 17,780 | 19,538 |
| MOB3B | MOB kinase activator 3B | **1,069** | 0,195025 | 4,985 | 6,704 |
| THBS4 | thrombospondin 4 | **1,069** | 0,065411 | 43,814 | 67,170 |
| CBFB | core-binding factor beta subunit | **1,069** | 0,029484 | 39,216 | 36,458 |
| AAMDC | adipogenesis associated Mth938 domain containing | **1,069** | 0,20819 | 8,973 | 9,641 |
| INO80B | INO80 complex subunit B | **1,069** | 0,642058 | 0,499 | 0,447 |
| SHTN1 | shootin 1 | **1,068** | 0,142786 | 14,955 | 11,493 |
| RGPD8 | RANBP2-like and GRIP domain containing 8 | **1,068** | 0,499899 | 1,440 | 1,788 |
| GGA2 | golgi associated, gamma adaptin ear containing, ARF binding protein 2 | **1,068** | 0,018716 | 34,342 | 30,967 |
| TNFAIP3 | TNF alpha induced protein 3 | **1,068** | 0,023455 | 177,027 | 181,589 |
| DHRS7 | dehydrogenase/reductase 7 | **1,068** | 0,04058 | 30,520 | 29,052 |
| LIMS1 | LIM zinc finger domain containing 1 | **1,068** | 0,04694 | 51,014 | 45,653 |
| NAP1L2 | nucleosome assembly protein 1 like 2 | **1,068** | 0,214672 | 6,702 | 7,598 |
| ANKZF1 | ankyrin repeat and zinc finger domain containing 1 | **1,068** | 0,039751 | 14,512 | 15,132 |
| NCKAP5L | NCK associated protein 5 like | **1,068** | 0,083367 | 18,888 | 15,324 |
| PDPK1 | 3-phosphoinositide dependent protein kinase 1 | **1,068** | 0,070875 | 38,773 | 37,991 |
| RC3H2 | ring finger and CCCH-type domains 2 | **1,068** | 0,110962 | 40,546 | 37,608 |
| CRTAC1 | cartilage acidic protein 1 | **1,068** | 0,014727 | 1586,928 | 1750,572 |
| DUSP3 | dual specificity phosphatase 3 | **1,068** | 0,02332 | 49,463 | 53,123 |
| CEP70 | centrosomal protein 70 | **1,068** | 0,2161 | 6,148 | 5,427 |
| MPP1 | membrane palmitoylated protein 1 | **1,068** | 0,1452 | 8,198 | 9,322 |
| PRKCQ-AS1 | PRKCQ antisense RNA 1 | **1,068** | 0,536255 | 0,942 | 1,469 |
| CASTOR2 | cytosolic arginine sensor for mTORC1 subunit 2 | **1,068** | 0,540459 | 2,160 | 2,746 |
| WDR92 | WD repeat domain 92 | **1,068** | 0,314482 | 3,545 | 3,512 |
| IL11RA | interleukin 11 receptor subunit alpha | **1,068** | 0,460392 | 1,496 | 1,341 |
| RGL1 | ral guanine nucleotide dissociation stimulator like 1 | **1,068** | 0,186255 | 6,813 | 9,067 |
| HIST2H4A | histone cluster 2 H4 family member a | **1,068** | 0,580585 | 0,720 | 1,724 |
| DVL2 | dishevelled segment polarity protein 2 | **1,068** | 0,018385 | 33,013 | 34,224 |
| SNHG21 | small nucleolar RNA host gene 21 | **1,068** | 0,761711 | 0,332 | 0,192 |

| | | | | | |
|---|---|---|---|---|---|
| ANKRD65 | ankyrin repeat domain 65 | **1,068** | 0,627966 | 1,219 | 0,702 |
| LMO4 | LIM domain only 4 | **1,068** | 0,008962 | 72,284 | 61,040 |
| PRMT2 | protein arginine methyltransferase 2 | **1,068** | 0,014729 | 58,658 | 64,744 |
| PBX3 | PBX homeobox 3 | **1,068** | 0,175106 | 10,358 | 7,470 |
| ZNF175 | zinc finger protein 175 | **1,068** | 0,073874 | 13,460 | 16,409 |
| KIAA1109 | KIAA1109 | **1,067** | 0,236237 | 44,811 | 46,802 |
| PNMA1 | paraneoplastic Ma antigen 1 | **1,067** | 0,021993 | 72,340 | 53,059 |
| CHD6 | chromodomain helicase DNA binding protein 6 | **1,067** | 0,150749 | 24,482 | 25,795 |
| HMGA1 | high mobility group AT-hook 1 | **1,067** | 0,024343 | 681,742 | 1018,406 |
| KCTD5 | potassium channel tetramerization domain containing 5 | **1,067** | 0,016306 | 51,679 | 45,461 |
| ZNF350 | zinc finger protein 350 | **1,067** | 0,105278 | 8,752 | 9,258 |
| GPAT3 | glycerol-3-phosphate acyltransferase 3 | **1,067** | 0,05739 | 19,054 | 24,071 |
| PUM1 | pumilio RNA binding family member 1 | **1,067** | 0,03818 | 72,063 | 79,493 |
| DMPK | dystrophia myotonica protein kinase | **1,067** | 0,101867 | 16,063 | 12,323 |
| PRPF38B | pre-mRNA processing factor 38B | **1,067** | 0,047988 | 45,752 | 51,655 |
| TERF2IP | TERF2 interacting protein | **1,067** | 0,00718 | 105,241 | 117,420 |
| RIC8B | RIC8 guanine nucleotide exchange factor B | **1,067** | 0,125559 | 9,306 | 13,025 |
| ZSWIM8 | zinc finger SWIM-type containing 8 | **1,067** | 0,016304 | 81,202 | 91,433 |
| RAB18 | RAB18, member RAS oncogene family | **1,067** | 0,026572 | 76,328 | 76,684 |
| TBCA | tubulin folding cofactor A | **1,067** | 0,063793 | 45,032 | 48,973 |
| PTPRG | protein tyrosine phosphatase, receptor type G | **1,067** | 0,167946 | 42,595 | 41,183 |
| ZNF253 | zinc finger protein 253 | **1,067** | 0,304898 | 2,548 | 3,129 |
| COL2A1 | collagen type II alpha 1 chain | **1,067** | 0,049601 | 3781,154 | 1217,936 |
| PTN | pleiotrophin | **1,067** | 0,554976 | 3,157 | 1,149 |
| ARFGEF1 | ADP ribosylation factor guanine nucleotide exchange factor 1 | **1,067** | 0,079526 | 49,851 | 49,484 |
| MIB1 | mindbomb E3 ubiquitin protein ligase 1 | **1,067** | 0,098608 | 48,743 | 53,251 |
| ZNF300 | zinc finger protein 300 | **1,067** | 0,123474 | 9,361 | 13,281 |
| PLEKHA8 | pleckstrin homology domain containing A8 | **1,067** | 0,178875 | 9,638 | 9,705 |
| PIH1D2 | PIH1 domain containing 2 | **1,067** | 0,442221 | 1,772 | 1,532 |
| MTMR6 | myotubularin related protein 6 | **1,067** | 0,072123 | 41,210 | 45,908 |
| B4GALT4 | beta-1,4-galactosyltransferase 4 | **1,067** | 0,034572 | 31,683 | 32,053 |
| LINC00482 | long intergenic non-protein coding RNA 482 | **1,067** | 0,753926 | 0,332 | 0,064 |
| DHX58 | DExH-box helicase 58 | **1,066** | 0,217629 | 5,207 | 5,427 |

| | | | | | |
|---|---|---|---|---|---|
| DLGAP4 | DLG associated protein 4 | **1,066** | 0,026657 | 132,382 | 148,132 |
| TTC21B | tetratricopeptide repeat domain 21B | **1,066** | 0,112286 | 13,958 | 19,602 |
| AHI1 | Abelson helper integration site 1 | **1,066** | 0,05328 | 38,164 | 50,250 |
| UBR5 | ubiquitin protein ligase E3 component n-recognin 5 | **1,066** | 0,090745 | 87,904 | 87,985 |
| CALCOCO1 | calcium binding and coiled-coil domain 1 | **1,066** | 0,0242 | 75,386 | 75,981 |
| RNASE4 | ribonuclease A family member 4 | **1,066** | 0,714775 | 0,332 | 0,128 |
| TGM2 | transglutaminase 2 | **1,066** | 0,01833 | 371,668 | 901,050 |
| LATS1 | large tumor suppressor kinase 1 | **1,066** | 0,064869 | 36,558 | 36,841 |
| SMOC2 | SPARC related modular calcium binding 2 | **1,066** | 0,028377 | 336,108 | 181,270 |
| INTS6-AS1 | INTS6 antisense RNA 1 | **1,066** | 0,421938 | 1,994 | 2,235 |
| PLXNA4 | plexin A4 | **1,066** | 0,585823 | 0,886 | 1,022 |
| ZBTB37 | zinc finger and BTB domain containing 37 | **1,066** | 0,395955 | 7,865 | 8,684 |
| RPRD1B | regulation of nuclear pre-mRNA domain containing 1B | **1,066** | 0,022741 | 38,219 | 49,101 |
| SBNO1 | strawberry notch homolog 1 | **1,066** | 0,025567 | 59,101 | 62,317 |
| TTC23 | tetratricopeptide repeat domain 23 | **1,066** | 0,064702 | 15,122 | 16,218 |
| MYSM1 | Myb like, SWIRM and MPN domains 1 | **1,066** | 0,136981 | 17,946 | 22,156 |
| STXBP6 | syntaxin binding protein 6 | **1,066** | 0,577402 | 1,994 | 0,894 |
| KIF14 | kinesin family member 14 | **1,066** | 0,473897 | 3,877 | 1,341 |
| NASP | nuclear autoantigenic sperm protein | **1,066** | 0,053702 | 27,861 | 25,157 |
| STEAP3 | STEAP3 metalloreductase | **1,066** | 0,024806 | 121,803 | 81,728 |
| C2orf74 | chromosome 2 open reading frame 74 | **1,066** | 0,741032 | 0,388 | 0,383 |
| LUCAT1 | lung cancer associated transcript 1 (non-protein coding) | **1,066** | 0,296445 | 7,865 | 19,410 |
| MPZL3 | myelin protein zero like 3 | **1,066** | 0,132853 | 9,306 | 10,344 |
| POC1B-AS1 | POC1B antisense RNA 1 | **1,066** | 0,433651 | 1,662 | 2,235 |
| C1QTNF6 | C1q and TNF related 6 | **1,066** | 0,39241 | 2,049 | 3,895 |
| FZD3 | frizzled class receptor 3 | **1,066** | 0,403128 | 2,160 | 2,554 |
| RPS2P32 | ribosomal protein S2 pseudogene 32 | **1,066** | 0,6253 | 0,831 | 0,766 |
| SCN4A | sodium voltage-gated channel alpha subunit 4 | **1,066** | 0,712636 | 0,277 | 0,511 |
| PRKCA | protein kinase C alpha | **1,066** | 0,038637 | 88,347 | 96,158 |
| CTNNBIP1 | catenin beta interacting protein 1 | **1,066** | 0,183897 | 14,235 | 14,111 |
| PNN | pinin, desmosome associated protein | **1,066** | 0,039692 | 49,186 | 58,614 |
| BNC2 | basonuclin 2 | **1,066** | 0,266959 | 6,148 | 5,810 |
| CNOT6 | CCR4-NOT transcription complex subunit 6 | **1,065** | 0,067835 | 22,765 | 27,008 |

| | | | | | |
|---|---|---|---|---|---|
| CHAF1B | chromatin assembly factor 1 subunit B | **1,065** | 0,334658 | 2,991 | 2,937 |
| IPO13 | importin 13 | **1,065** | 0,017071 | 30,465 | 33,202 |
| ARMCX6 | armadillo repeat containing, X-linked 6 | **1,065** | 0,185373 | 8,585 | 6,002 |
| CNTD1 | cyclin N-terminal domain containing 1 | **1,065** | 0,754607 | 0,388 | 0,255 |
| GLCE | glucuronic acid epimerase | **1,065** | 0,185653 | 23,319 | 20,943 |
| HLA-B | major histocompatibility complex, class I, B | **1,065** | 0,115222 | 15,897 | 105,735 |
| SIRT1 | sirtuin 1 | **1,065** | 0,027733 | 37,444 | 42,971 |
| TMEM242 | transmembrane protein 242 | **1,065** | 0,110643 | 12,574 | 14,239 |
| LINC00899 | long intergenic non-protein coding RNA 899 | **1,065** | 0,490058 | 1,551 | 2,809 |
| NCAPH | non-SMC condensin I complex subunit H | **1,065** | 0,624771 | 1,717 | 0,830 |
| ERVMER34-1 | endogenous retrovirus group MER34 member 1, envelope | **1,065** | 0,625893 | 0,775 | 1,213 |
| SRSF12 | serine and arginine rich splicing factor 12 | **1,065** | 0,483366 | 1,828 | 1,915 |
| PAN3 | PAN3 poly(A) specific ribonuclease subunit | **1,065** | 0,21802 | 18,888 | 18,836 |
| ADAMTS17 | ADAM metallopeptidase with thrombospondin type 1 motif 17 | **1,065** | 0,080202 | 12,352 | 16,346 |
| GNG12 | G protein subunit gamma 12 | **1,065** | 0,075698 | 203,891 | 225,901 |
| PPP5D1 | PPP5 tetratricopeptide repeat domain containing 1 | **1,065** | 0,624361 | 0,443 | 1,277 |
| TFDP2 | transcription factor Dp-2 | **1,065** | 0,062413 | 16,617 | 16,601 |
| ACP5 | acid phosphatase 5, tartrate resistant | **1,065** | 0,687378 | 0,222 | 0,575 |
| VPS18 | VPS18, CORVET/HOPS core subunit | **1,065** | 0,066332 | 30,520 | 24,582 |
| ASXL2 | additional sex combs like 2, transcriptional regulator | **1,065** | 0,192168 | 46,639 | 55,294 |
| IKZF5 | IKAROS family zinc finger 5 | **1,065** | 0,12238 | 13,404 | 17,303 |
| MYCBP2 | MYC binding protein 2, E3 ubiquitin protein ligase | **1,065** | 0,239714 | 56,221 | 67,872 |
| SS18L2 | SS18 like 2 | **1,065** | 0,15357 | 7,644 | 8,109 |
| TAF4B | TATA-box binding protein associated factor 4b | **1,065** | 0,197966 | 7,312 | 8,620 |
| PHF14 | PHD finger protein 14 | **1,065** | 0,017435 | 26,476 | 30,648 |
| TCFL5 | transcription factor like 5 | **1,065** | 0,178859 | 9,029 | 11,621 |
| NKTR | natural killer cell triggering receptor | **1,065** | 0,071161 | 44,811 | 49,994 |
| PLCXD2 | phosphatidylinositol specific phospholipase C X domain containing 2 | **1,065** | 0,766197 | 0,609 | 0,766 |
| C10orf35 | chromosome 10 open reading frame 35 | **1,065** | 0,139734 | 4,930 | 7,534 |
| MFGE8 | milk fat globule-EGF factor 8 protein | **1,065** | 0,018915 | 1165,464 | 845,500 |
| SOX5 | SRY-box 5 | **1,065** | 0,120831 | 82,144 | 79,940 |
| ATF2 | activating transcription factor 2 | **1,065** | 0,105628 | 37,887 | 32,116 |
| HEXB | hexosaminidase subunit beta | **1,065** | 0,018967 | 121,637 | 95,392 |

| | | | | | |
|---|---|---|---|---|---|
| RPL18AP3 | ribosomal protein L18a pseudogene 3 | **1,065** | 0,667984 | 0,554 | 0,383 |
| MKRN1 | makorin ring finger protein 1 | **1,065** | 0,01642 | 68,019 | 79,940 |
| DPM1 | dolichyl-phosphate mannosyltransferase subunit 1, catalytic | **1,065** | 0,082549 | 28,083 | 34,798 |
| TRIM13 | tripartite motif containing 13 | **1,065** | 0,051663 | 27,030 | 27,136 |
| WRB | tryptophan rich basic protein | **1,065** | 0,089956 | 21,768 | 21,390 |
| NFX1 | nuclear transcription factor, X-box binding 1 | **1,065** | 0,032698 | 42,318 | 44,312 |
| EPOR | erythropoietin receptor | **1,064** | 0,479591 | 2,049 | 1,724 |
| POM121C | POM121 transmembrane nucleoporin C | **1,064** | 0,058818 | 17,891 | 20,496 |
| HMGN1 | high mobility group nucleosome binding domain 1 | **1,064** | 0,040019 | 46,472 | 35,117 |
| SMARCA2 | SWI/SNF related, matrix associated, actin dependent regulator of chromatin, subfamily a, | **1,064** | 0,032565 | 79,429 | 77,769 |
| PNMA2 | paraneoplastic Ma antigen 2 | **1,064** | 0,169449 | 5,262 | 2,682 |
| SPPL3 | signal peptide peptidase like 3 | **1,064** | 0,019808 | 63,089 | 60,913 |
| SDCBP2-AS1 | SDCBP2 antisense RNA 1 | **1,064** | 0,453489 | 2,271 | 1,915 |
| ATAD2 | ATPase family, AAA domain containing 2 | **1,064** | 0,097396 | 18,777 | 14,494 |
| SLC25A51 | solute carrier family 25 member 51 | **1,064** | 0,244171 | 3,877 | 4,725 |
| LRP2BP | LRP2 binding protein | **1,064** | 0,498178 | 1,052 | 2,107 |
| SUSD6 | sushi domain containing 6 | **1,064** | 0,038752 | 148,556 | 165,754 |
| ZNF493 | zinc finger protein 493 | **1,064** | 0,312913 | 3,213 | 4,916 |
| FAM206A | family with sequence similarity 206 member A | **1,064** | 0,140132 | 11,410 | 15,132 |
| ULK2 | unc-51 like autophagy activating kinase 2 | **1,064** | 0,220814 | 13,460 | 10,088 |
| CCDC6 | coiled-coil domain containing 6 | **1,064** | 0,037526 | 40,047 | 47,824 |
| RHOBTB2 | Rho related BTB domain containing 2 | **1,064** | 0,150817 | 14,900 | 12,834 |
| SLC25A22 | solute carrier family 25 member 22 | **1,064** | 0,035738 | 29,745 | 34,862 |
| LUC7L2 | LUC7 like 2, pre-mRNA splicing factor | **1,064** | 0,033948 | 23,762 | 29,754 |
| RPRD1A | regulation of nuclear pre-mRNA domain containing 1A | **1,064** | 0,054759 | 35,837 | 37,544 |
| RBM48 | RNA binding motif protein 48 | **1,064** | 0,08495 | 14,789 | 18,836 |
| ASB13 | ankyrin repeat and SOCS box containing 13 | **1,064** | 0,147843 | 11,687 | 11,429 |
| GIT2 | GIT ArfGAP 2 | **1,064** | 0,029911 | 33,566 | 35,564 |
| LAPTM4A | lysosomal protein transmembrane 4 alpha | **1,064** | 0,035958 | 296,116 | 299,456 |
| CHD1L | chromodomain helicase DNA binding protein 1 like | **1,064** | 0,030456 | 25,812 | 26,562 |
| ATP6V1G1 | ATPase H+ transporting V1 subunit G1 | **1,064** | 0,069474 | 77,546 | 85,750 |
| ELF2 | E74 like ETS transcription factor 2 | **1,064** | 0,078053 | 27,197 | 24,774 |
| IDS | iduronate 2-sulfatase | **1,064** | 0,028281 | 117,926 | 91,114 |

| | | | | | |
|---|---|---|---|---|---|
| MORC2 | MORC family CW-type zinc finger 2 | **1,064** | 0,019232 | 47,026 | 48,143 |
| GPALPP1 | GPALPP motifs containing 1 | **1,064** | 0,114795 | 13,460 | 16,090 |
| CXorf38 | chromosome X open reading frame 38 | **1,064** | 0,087906 | 14,235 | 16,984 |
| ARHGEF6 | Rac/Cdc42 guanine nucleotide exchange factor 6 | **1,064** | 0,33687 | 8,641 | 5,236 |
| CRBN | cereblon | **1,064** | 0,120698 | 17,725 | 17,495 |
| CDC73 | cell division cycle 73 | **1,063** | 0,046595 | 53,396 | 59,444 |
| GSN | gelsolin | **1,063** | 0,031994 | 238,012 | 169,394 |
| CD79A | CD79a molecule | **1,063** | 0,578572 | 1,219 | 0,447 |
| SPATA18 | spermatogenesis associated 18 | **1,063** | 0,17715 | 5,317 | 9,641 |
| CNOT6L | CCR4-NOT transcription complex subunit 6 like | **1,063** | 0,247145 | 14,623 | 12,578 |
| MALAT1 | metastasis associated lung adenocarcinoma transcript 1 (non-protein coding) | **1,063** | 0,458287 | 712,041 | 1052,629 |
| LSM11 | LSM11, U7 small nuclear RNA associated | **1,063** | 0,347242 | 4,487 | 4,853 |
| ZNF404 | zinc finger protein 404 | **1,063** | 0,648312 | 0,942 | 0,192 |
| NXN | nucleoredoxin | **1,063** | 0,177458 | 9,527 | 8,747 |
| MFSD14B | major facilitator superfamily domain containing 14B | **1,063** | 0,048546 | 61,594 | 54,528 |
| HSF5 | heat shock transcription factor 5 | **1,063** | 0,829918 | 0,443 | 0,447 |
| MROH1 | maestro heat like repeat family member 1 | **1,063** | 0,108273 | 22,987 | 23,305 |
| TBC1D30 | TBC1 domain family member 30 | **1,063** | 0,587332 | 1,052 | 1,213 |
| SMS | spermine synthase | **1,063** | 0,051673 | 61,538 | 61,104 |
| BCR | BCR, RhoGEF and GTPase activating protein | **1,063** | 0,062431 | 44,755 | 56,379 |
| LRRC49 | leucine rich repeat containing 49 | **1,063** | 0,116275 | 25,978 | 23,624 |
| ARRDC3-AS1 | ARRDC3 antisense RNA 1 | **1,063** | 0,694781 | 0,499 | 0,511 |
| TPST2 | tyrosylprotein sulfotransferase 2 | **1,063** | 0,155987 | 14,401 | 16,090 |
| CRKL | CRK like proto-oncogene, adaptor protein | **1,063** | 0,040143 | 94,551 | 98,840 |
| UPP1 | uridine phosphorylase 1 | **1,063** | 0,034271 | 92,612 | 125,720 |
| TTC28-AS1 | TTC28 antisense RNA 1 | **1,063** | 0,248707 | 3,988 | 6,385 |
| PDE12 | phosphodiesterase 12 | **1,063** | 0,027346 | 24,482 | 29,435 |
| VCL | vinculin | **1,063** | 0,027482 | 91,505 | 94,562 |
| ZSCAN9 | zinc finger and SCAN domain containing 9 | **1,063** | 0,22282 | 6,038 | 7,981 |
| CPSF6 | cleavage and polyadenylation specific factor 6 | **1,063** | 0,019078 | 51,291 | 57,529 |
| SRGAP3 | SLIT-ROBO Rho GTPase activating protein 3 | **1,063** | 0,489166 | 1,052 | 2,618 |
| AMN1 | antagonist of mitotic exit network 1 homolog | **1,063** | 0,481841 | 2,105 | 1,852 |
| OSBPL9 | oxysterol binding protein like 9 | **1,063** | 0,080757 | 18,611 | 29,243 |

| | | | | | |
|---|---|---|---|---|---|
| MINOS1 | mitochondrial inner membrane organizing system 1 | **1,063** | 0,159521 | 7,921 | 10,088 |
| SGF29 | SAGA complex associated factor 29 | **1,063** | 0,162117 | 6,204 | 8,300 |
| GLIPR1L2 | GLI pathogenesis related 1 like 2 | **1,063** | 0,530821 | 0,222 | 3,703 |
| HIPK1 | homeodomain interacting protein kinase 1 | **1,063** | 0,174187 | 65,471 | 76,237 |
| NBPF11 | NBPF member 11 | **1,063** | 0,395468 | 3,323 | 3,703 |
| CDKL5 | cyclin dependent kinase like 5 | **1,063** | 0,217813 | 12,629 | 14,302 |
| CASP7 | caspase 7 | **1,063** | 0,051716 | 14,124 | 15,516 |
| PCDHB15 | protocadherin beta 15 | **1,063** | 0,525015 | 3,157 | 2,490 |
| CEP135 | centrosomal protein 135 | **1,063** | 0,209443 | 9,416 | 9,961 |
| PCDHGA2 | protocadherin gamma subfamily A, 2 | **1,062** | 0,116499 | 16,894 | 16,729 |
| ALG10B | ALG10B, alpha-1,2-glucosyltransferase | **1,062** | 0,210917 | 9,306 | 12,259 |
| SLCO2A1 | solute carrier organic anion transporter family member 2A1 | **1,062** | 0,755424 | 0,055 | 0,575 |
| LRRCC1 | leucine rich repeat and coiled-coil centrosomal protein 1 | **1,062** | 0,154042 | 7,644 | 10,024 |
| PANK3 | pantothenate kinase 3 | **1,062** | 0,085137 | 56,996 | 57,784 |
| LRIG2 | leucine rich repeats and immunoglobulin like domains 2 | **1,062** | 0,167417 | 16,617 | 23,880 |
| MAFB | MAF bZIP transcription factor B | **1,062** | 0,026488 | 201,399 | 329,210 |
| ZNF551 | zinc finger protein 551 | **1,062** | 0,397687 | 2,548 | 3,192 |
| SLC7A5P1 | solute carrier family 7 member 5 pseudogene 1 | **1,062** | 0,738226 | 0,277 | 0,575 |
| RAB35 | RAB35, member RAS oncogene family | **1,062** | 0,037667 | 55,778 | 54,783 |
| UBE2W | ubiquitin conjugating enzyme E2 W | **1,062** | 0,050804 | 20,882 | 26,115 |
| MRPS30 | mitochondrial ribosomal protein S30 | **1,062** | 0,034143 | 21,436 | 28,605 |
| TMED8 | transmembrane p24 trafficking protein family member 8 | **1,062** | 0,12253 | 28,138 | 26,817 |
| RAP1GDS1 | Rap1 GTPase-GDP dissociation stimulator 1 | **1,062** | 0,067964 | 19,996 | 17,303 |
| COX19 | COX19, cytochrome c oxidase assembly factor | **1,062** | 0,077632 | 10,635 | 14,685 |
| C6orf203 | chromosome 6 open reading frame 203 | **1,062** | 0,124039 | 11,300 | 13,472 |
| SMURF1 | SMAD specific E3 ubiquitin protein ligase 1 | **1,062** | 0,065932 | 48,910 | 49,356 |
| PRELID3B | PRELI domain containing 3B | **1,062** | 0,030453 | 39,272 | 35,884 |
| SPOPL | speckle type BTB/POZ protein like | **1,062** | 0,090449 | 28,249 | 27,839 |
| ERFE | erythroferrone | **1,062** | 0,305102 | 10,635 | 6,960 |
| HIC2 | HIC ZBTB transcriptional repressor 2 | **1,062** | 0,407066 | 3,268 | 3,959 |
| LINC00847 | long intergenic non-protein coding RNA 847 | **1,062** | 0,267657 | 5,816 | 5,683 |
| GMNN | geminin, DNA replication inhibitor | **1,062** | 0,295 | 6,536 | 6,193 |
| PHF20L1 | PHD finger protein 20-like 1 | **1,062** | 0,032614 | 35,339 | 41,566 |

| | | | | | |
|---|---|---|---|---|---|
| FAM135A | family with sequence similarity 135 member A | **1,062** | 0,213374 | 10,303 | 11,812 |
| RUFY2 | RUN and FYVE domain containing 2 | **1,062** | 0,180907 | 12,518 | 13,600 |
| C9orf78 | chromosome 9 open reading frame 78 | **1,062** | 0,080989 | 46,528 | 50,122 |
| TMEM50A | transmembrane protein 50A | **1,062** | 0,027224 | 118,590 | 88,241 |
| LRRC20 | leucine rich repeat containing 20 | **1,062** | 0,344016 | 2,382 | 4,342 |
| H2AFX | H2A histone family member X | **1,062** | 0,126547 | 40,490 | 37,288 |
| TINCR | tissue differentiation-inducing non-protein coding RNA | **1,062** | 0,591608 | 0,499 | 1,469 |
| WASL | Wiskott-Aldrich syndrome like | **1,062** | 0,056695 | 34,896 | 38,565 |
| UNC5C | unc-5 netrin receptor C | **1,062** | 0,507881 | 7,145 | 6,257 |
| RIMS1 | regulating synaptic membrane exocytosis 1 | **1,062** | 0,672503 | 0,665 | 0,192 |
| RABGEF1 | RAB guanine nucleotide exchange factor 1 | **1,062** | 0,241003 | 3,102 | 4,789 |
| ZNF525 | zinc finger protein 525 | **1,062** | 0,282773 | 4,708 | 7,279 |
| BMP1 | bone morphogenetic protein 1 | **1,062** | 0,04187 | 74,057 | 50,122 |
| OFD1 | OFD1, centriole and centriolar satellite protein | **1,062** | 0,087237 | 18,833 | 20,815 |
| ITGB3BP | integrin subunit beta 3 binding protein | **1,061** | 0,298128 | 7,090 | 4,853 |
| ZDHHC7 | zinc finger DHHC-type containing 7 | **1,061** | 0,044893 | 29,855 | 31,414 |
| PABPC1P4 | poly(A) binding protein cytoplasmic 1 pseudogene 4 | **1,061** | 0,486371 | 3,490 | 3,256 |
| ZNF582 | zinc finger protein 582 | **1,061** | 0,301967 | 3,600 | 4,533 |
| HERPUD2 | HERPUD family member 2 | **1,061** | 0,066443 | 36,004 | 37,288 |
| EML4 | echinoderm microtubule associated protein like 4 | **1,061** | 0,084431 | 17,060 | 19,155 |
| TSC22D2 | TSC22 domain family member 2 | **1,061** | 0,115784 | 27,086 | 32,180 |
| NHLRC2 | NHL repeat containing 2 | **1,061** | 0,103613 | 35,893 | 41,758 |
| MMGT1 | membrane magnesium transporter 1 | **1,061** | 0,050335 | 57,606 | 55,166 |
| CELF1 | CUGBP Elav-like family member 1 | **1,061** | 0,03623 | 49,186 | 45,653 |
| MOSPD1 | motile sperm domain containing 1 | **1,061** | 0,257826 | 13,903 | 15,452 |
| CFLAR | CASP8 and FADD like apoptosis regulator | **1,061** | 0,070845 | 58,769 | 62,317 |
| TFAP2A | transcription factor AP-2 alpha | **1,061** | 0,756761 | 0,277 | 0,319 |
| LANCL1 | LanC like 1 | **1,061** | 0,094075 | 29,301 | 37,544 |
| NBPF15 | NBPF member 15 | **1,061** | 0,302846 | 6,868 | 7,981 |
| SSTR5-AS1 | SSTR5 antisense RNA 1 | **1,061** | 0,553122 | 1,329 | 2,235 |
| MON2 | MON2 homolog, regulator of endosome-to-Golgi trafficking | **1,061** | 0,208002 | 51,347 | 52,421 |
| SH3BGRL | SH3 domain binding glutamate rich protein like | **1,061** | 0,059802 | 40,823 | 35,309 |
| CEP350 | centrosomal protein 350 | **1,061** | 0,261341 | 61,428 | 58,423 |

| | | | | | |
|---|---|---|---|---|---|
| TAX1BP1 | Tax1 binding protein 1 | **1,061** | 0,022689 | 131,385 | 128,466 |
| ENPEP | glutamyl aminopeptidase | **1,061** | 0,488003 | 1,662 | 2,618 |
| C3orf14 | chromosome 3 open reading frame 14 | **1,061** | 0,389063 | 4,154 | 4,086 |
| ANAPC10 | anaphase promoting complex subunit 10 | **1,061** | 0,135389 | 6,924 | 11,812 |
| HERPUD1 | homocysteine inducible ER protein with ubiquitin like domain 1 | **1,061** | 0,068675 | 163,456 | 172,459 |
| DSTYK | dual serine/threonine and tyrosine protein kinase | **1,061** | 0,057852 | 47,359 | 41,949 |
| MCUB | mitochondrial calcium uniporter dominant negative beta subunit | **1,061** | 0,13312 | 10,580 | 16,537 |
| FBXL5 | F-box and leucine rich repeat protein 5 | **1,061** | 0,031082 | 32,736 | 32,755 |
| ZSCAN25 | zinc finger and SCAN domain containing 25 | **1,061** | 0,11814 | 11,189 | 15,899 |
| ZNRF2P1 | zinc and ring finger 2 pseudogene 1 | **1,060** | 0,663418 | 0,775 | 1,532 |
| SEC22C | SEC22 homolog C, vesicle trafficking protein | **1,060** | 0,098525 | 27,917 | 25,157 |
| SON | SON DNA binding protein | **1,060** | 0,056627 | 256,733 | 311,971 |
| LXN | latexin | **1,060** | 0,536775 | 2,659 | 1,596 |
| TRMO | tRNA methyltransferase O | **1,060** | 0,105913 | 12,574 | 19,857 |
| KIAA0922 | KIAA0922 | **1,060** | 0,274554 | 6,314 | 6,768 |
| XDH | xanthine dehydrogenase | **1,060** | 0,489332 | 1,385 | 2,490 |
| ATXN7L3B | ataxin 7 like 3B | **1,060** | 0,043625 | 62,203 | 66,212 |
| ABCB4 | ATP binding cassette subfamily B member 4 | **1,060** | 0,754443 | 0,942 | 0,766 |
| SLC25A44 | solute carrier family 25 member 44 | **1,060** | 0,076406 | 21,104 | 28,349 |
| ZNF267 | zinc finger protein 267 | **1,060** | 0,093496 | 16,894 | 21,709 |
| PCDHB4 | protocadherin beta 4 | **1,060** | 0,35449 | 9,139 | 8,237 |
| TRIM4 | tripartite motif containing 4 | **1,060** | 0,042029 | 42,152 | 41,630 |
| RSF1 | remodeling and spacing factor 1 | **1,060** | 0,024259 | 60,763 | 64,616 |
| MSRB3 | methionine sulfoxide reductase B3 | **1,060** | 0,04777 | 40,269 | 31,031 |
| ERO1A | endoplasmic reticulum oxidoreductase 1 alpha | **1,060** | 0,029162 | 96,379 | 68,383 |
| HERC2P2 | hect domain and RLD 2 pseudogene 2 | **1,060** | 0,380955 | 3,656 | 3,767 |
| ZNF286B | zinc finger protein 286B | **1,060** | 0,563131 | 1,163 | 1,852 |
| PPM1J | protein phosphatase, Mg2+/Mn2+ dependent 1J | **1,060** | 0,605256 | 1,440 | 2,299 |
| CSAD | cysteine sulfinic acid decarboxylase | **1,060** | 0,212147 | 7,699 | 9,003 |
| YAP1 | Yes associated protein 1 | **1,060** | 0,06352 | 115,378 | 112,248 |
| TSPYL5 | TSPY like 5 | **1,060** | 0,054471 | 47,026 | 35,245 |
| ZNF776 | zinc finger protein 776 | **1,060** | 0,345373 | 8,087 | 7,215 |
| NRARP | NOTCH-regulated ankyrin repeat protein | **1,060** | 0,738289 | 0,443 | 0,638 |

| | | | | | |
|---|---|---|---|---|---|
| TMEM254 | transmembrane protein 254 | **1,060** | 0,15867 | 7,478 | 7,598 |
| CLN5 | ceroid-lipofuscinosis, neuronal 5 | **1,060** | 0,030622 | 37,721 | 33,521 |
| FEM1C | fem-1 homolog C | **1,060** | 0,056904 | 60,154 | 51,782 |
| CHMP3 | charged multivesicular body protein 3 | **1,060** | 0,06185 | 48,189 | 42,269 |
| HIBADH | 3-hydroxyisobutyrate dehydrogenase | **1,059** | 0,110261 | 20,273 | 18,261 |
| LLGL2 | LLGL2, scribble cell polarity complex component | **1,059** | 0,567597 | 0,942 | 1,085 |
| CEP131 | centrosomal protein 131 | **1,059** | 0,236648 | 7,644 | 7,343 |
| SERINC2 | serine incorporator 2 | **1,059** | 0,088487 | 26,310 | 11,621 |
| PTPN20 | protein tyrosine phosphatase, non-receptor type 20 | **1,059** | 0,396707 | 4,099 | 1,469 |
| TIGD1 | tigger transposable element derived 1 | **1,059** | 0,352932 | 2,603 | 5,044 |
| PCMTD2 | protein-L-isoaspartate (D-aspartate) O-methyltransferase domain containing 2 | **1,059** | 0,32423 | 4,210 | 5,108 |
| CREBZF | CREB/ATF bZIP transcription factor | **1,059** | 0,103839 | 29,966 | 28,413 |
| C5orf24 | chromosome 5 open reading frame 24 | **1,059** | 0,106324 | 57,384 | 50,888 |
| SIN3A | SIN3 transcription regulator family member A | **1,059** | 0,145582 | 48,466 | 47,057 |
| SEMA4D | semaphorin 4D | **1,059** | 0,142395 | 12,186 | 17,942 |
| NLRP3 | NLR family pyrin domain containing 3 | **1,059** | 0,744944 | 0,332 | 0,702 |
| SLU7 | SLU7 homolog, splicing factor | **1,059** | 0,033265 | 64,419 | 62,062 |
| ZNF431 | zinc finger protein 431 | **1,059** | 0,281537 | 5,484 | 6,257 |
| ZNF26 | zinc finger protein 26 | **1,059** | 0,171786 | 9,693 | 12,259 |
| ARL6IP5 | ADP ribosylation factor like GTPase 6 interacting protein 5 | **1,059** | 0,078958 | 131,441 | 114,611 |
| FBXW4 | F-box and WD repeat domain containing 4 | **1,059** | 0,130622 | 16,506 | 16,473 |
| NRG4 | neuregulin 4 | **1,059** | 0,723834 | 0,166 | 0,575 |
| GEMIN8P4 | gem nuclear organelle associated protein 8 pseudogene 4 | **1,059** | 0,65965 | 0,332 | 0,766 |
| PCMT1 | protein-L-isoaspartate (D-aspartate) O-methyltransferase | **1,059** | 0,052865 | 57,772 | 59,380 |
| PIGK | phosphatidylinositol glycan anchor biosynthesis class K | **1,059** | 0,085118 | 40,490 | 35,117 |
| EMSY | EMSY, BRCA2 interacting transcriptional repressor | **1,059** | 0,211358 | 22,710 | 26,945 |
| TNFAIP6 | TNF alpha induced protein 6 | **1,059** | 0,038259 | 2125,653 | 990,759 |
| NRG1 | neuregulin 1 | **1,059** | 0,657737 | 0,775 | 0,766 |
| PCBP3 | poly(rC) binding protein 3 | **1,059** | 0,45464 | 3,268 | 2,043 |
| PRICKLE1 | prickle planar cell polarity protein 1 | **1,059** | 0,259863 | 7,090 | 10,663 |
| NADK | NAD kinase | **1,059** | 0,038791 | 28,747 | 25,795 |
| EEF1D | eukaryotic translation elongation factor 1 delta | **1,059** | 0,547268 | 1,329 | 1,341 |
| MKLN1 | muskelin 1 | **1,059** | 0,122324 | 73,780 | 80,770 |

| | | | | | |
|---|---|---|---|---|---|
| NEK10 | NIMA related kinase 10 | **1,059** | 0,599449 | 1,108 | 1,469 |
| ABHD18 | abhydrolase domain containing 18 | **1,059** | 0,329264 | 5,927 | 6,385 |
| CENPJ | centromere protein J | **1,059** | 0,397882 | 4,265 | 3,959 |
| IQCG | IQ motif containing G | **1,058** | 0,354581 | 4,320 | 5,300 |
| AGBL3 | ATP/GTP binding protein like 3 | **1,058** | 0,520275 | 0,997 | 1,915 |
| OLFML2B | olfactomedin like 2B | **1,058** | 0,726985 | 0,775 | 0,638 |
| VEZT | vezatin, adherens junctions transmembrane protein | **1,058** | 0,110813 | 44,977 | 37,863 |
| GMFB | glia maturation factor beta | **1,058** | 0,066233 | 60,431 | 56,252 |
| TMEM201 | transmembrane protein 201 | **1,058** | 0,175947 | 13,737 | 19,921 |
| RUNX3 | runt related transcription factor 3 | **1,058** | 0,521557 | 4,320 | 1,469 |
| PHF11 | PHD finger protein 11 | **1,058** | 0,212902 | 11,687 | 13,728 |
| AHSA2 | activator of HSP90 ATPase homolog 2 | **1,058** | 0,221482 | 10,801 | 16,984 |
| MIR600HG | MIR600 host gene | **1,058** | 0,652973 | 0,775 | 1,277 |
| ENGASE | endo-beta-N-acetylglucosaminidase | **1,058** | 0,13721 | 17,005 | 24,199 |
| PLEKHB2 | pleckstrin homology domain containing B2 | **1,058** | 0,029112 | 110,559 | 95,136 |
| LINC01569 | long intergenic non-protein coding RNA 1569 | **1,058** | 0,503742 | 1,828 | 1,596 |
| POM121 | POM121 transmembrane nucleoporin | **1,058** | 0,21295 | 19,497 | 22,156 |
| LRRC8E | leucine rich repeat containing 8 family member E | **1,058** | 0,506217 | 1,274 | 1,788 |
| PHLDA3 | pleckstrin homology like domain family A member 3 | **1,058** | 0,12411 | 28,194 | 26,753 |
| HINT3 | histidine triad nucleotide binding protein 3 | **1,058** | 0,081075 | 19,719 | 19,921 |
| LZTS1 | leucine zipper tumor suppressor 1 | **1,058** | 0,466175 | 2,326 | 4,406 |
| C14orf93 | chromosome 14 open reading frame 93 | **1,058** | 0,520104 | 2,659 | 1,915 |
| AQP11 | aquaporin 11 | **1,058** | 0,687806 | 0,609 | 0,575 |
| TMEM117 | transmembrane protein 117 | **1,058** | 0,515698 | 1,883 | 1,852 |
| ZKSCAN5 | zinc finger with KRAB and SCAN domains 5 | **1,058** | 0,092048 | 23,873 | 27,519 |
| KRAS | KRAS proto-oncogene, GTPase | **1,058** | 0,193358 | 27,252 | 27,583 |
| LINC00960 | long intergenic non-protein coding RNA 960 | **1,058** | 0,736533 | 0,000 | 0,447 |
| WDR90 | WD repeat domain 90 | **1,058** | 0,226638 | 9,029 | 10,280 |
| COL10A1 | collagen type X alpha 1 chain | **1,058** | 0,054276 | 404,957 | 46,866 |
| SGCE | sarcoglycan epsilon | **1,058** | 0,207944 | 8,032 | 8,492 |
| NOP56 | NOP56 ribonucleoprotein | **1,058** | 0,052739 | 82,088 | 144,365 |
| SRSF11 | serine and arginine rich splicing factor 11 | **1,058** | 0,061544 | 95,991 | 99,542 |
| KCNN3 | potassium calcium-activated channel subfamily N member 3 | **1,058** | 0,478908 | 4,265 | 7,023 |

| | | | | | |
|---|---|---|---|---|---|
| ABCC3 | ATP binding cassette subfamily C member 3 | **1,058** | 0,071954 | 36,779 | 26,242 |
| ADGRE5 | adhesion G protein-coupled receptor E5 | **1,058** | 0,103246 | 27,750 | 51,718 |
| SLC25A25 | solute carrier family 25 member 25 | **1,058** | 0,075594 | 18,722 | 27,072 |
| MGST1 | microsomal glutathione S-transferase 1 | **1,058** | 0,055354 | 31,351 | 23,369 |
| PABPC3 | poly(A) binding protein cytoplasmic 3 | **1,058** | 0,777538 | 0,000 | 0,383 |
| EAF1 | ELL associated factor 1 | **1,058** | 0,082626 | 37,001 | 38,055 |
| STK36 | serine/threonine kinase 36 | **1,058** | 0,18894 | 13,294 | 15,899 |
| CEP85 | centrosomal protein 85 | **1,058** | 0,173605 | 17,171 | 19,730 |
| SETDB2 | SET domain bifurcated 2 | **1,058** | 0,305026 | 5,982 | 6,257 |
| HIST2H2BC | histone cluster 2 H2B family member c (pseudogene) | **1,058** | 0,780645 | 0,609 | 1,085 |
| RND1 | Rho family GTPase 1 | **1,058** | 0,156801 | 32,459 | 38,055 |
| SFMBT2 | Scm-like with four mbt domains 2 | **1,058** | 0,163926 | 6,370 | 7,534 |
| PIK3C2A | phosphatidylinositol-4-phosphate 3-kinase catalytic subunit type 2 alpha | **1,058** | 0,198334 | 40,490 | 44,503 |
| RERE | arginine-glutamic acid dipeptide repeats | **1,057** | 0,098967 | 63,533 | 73,044 |
| PLBD2 | phospholipase B domain containing 2 | **1,057** | 0,045749 | 137,146 | 83,133 |
| RBM15 | RNA binding motif protein 15 | **1,057** | 0,215843 | 6,314 | 7,279 |
| CEP250 | centrosomal protein 250 | **1,057** | 0,142794 | 15,509 | 21,390 |
| CRAMP1 | cramped chromatin regulator homolog 1 | **1,057** | 0,165778 | 14,955 | 17,367 |
| LEMD3 | LEM domain containing 3 | **1,057** | 0,112543 | 48,743 | 46,100 |
| AP5M1 | adaptor related protein complex 5 mu 1 subunit | **1,057** | 0,137821 | 24,538 | 23,369 |
| ZFP82 | ZFP82 zinc finger protein | **1,057** | 0,423475 | 3,213 | 3,831 |
| FTLP3 | ferritin light chain pseudogene 3 | **1,057** | 0,473404 | 2,326 | 1,660 |
| ZNF275 | zinc finger protein 275 | **1,057** | 0,115218 | 25,036 | 28,158 |
| ATG2A | autophagy related 2A | **1,057** | 0,065591 | 40,823 | 43,737 |
| PPM1A | protein phosphatase, Mg2+/Mn2+ dependent 1A | **1,057** | 0,037837 | 55,390 | 52,995 |
| PFKL | phosphofructokinase, liver type | **1,057** | 0,043448 | 185,114 | 181,972 |
| MTDH | metadherin | **1,057** | 0,050256 | 142,685 | 124,954 |
| RHOT1 | ras homolog family member T1 | **1,057** | 0,175357 | 20,716 | 16,856 |
| CEP290 | centrosomal protein 290 | **1,057** | 0,125081 | 19,940 | 21,198 |
| TTC37 | tetratricopeptide repeat domain 37 | **1,057** | 0,092157 | 44,368 | 40,162 |
| SCAF4 | SR-related CTD associated factor 4 | **1,057** | 0,040731 | 32,902 | 40,672 |
| ARID4B | AT-rich interaction domain 4B | **1,057** | 0,045281 | 48,300 | 60,083 |
| TARSL2 | threonyl-tRNA synthetase like 2 | **1,057** | 0,226901 | 9,416 | 12,259 |

| | | | | | |
|---|---|---|---|---|---|
| BRI3 | brain protein I3 | **1,057** | 0,091522 | 155,757 | 121,698 |
| SCAF11 | SR-related CTD associated factor 11 | **1,057** | 0,056255 | 77,214 | 81,089 |
| ACADSB | acyl-CoA dehydrogenase, short/branched chain | **1,057** | 0,283035 | 11,078 | 11,238 |
| SFSWAP | splicing factor SWAP homolog | **1,057** | 0,088577 | 28,138 | 39,332 |
| L3MBTL3 | l(3)mbt-like 3 (Drosophila) | **1,057** | 0,379579 | 5,373 | 6,066 |
| RNF13 | ring finger protein 13 | **1,057** | 0,03264 | 90,951 | 86,644 |
| ADAM9 | ADAM metallopeptidase domain 9 | **1,057** | 0,108883 | 52,178 | 31,925 |
| STAB1 | stabilin 1 | **1,057** | 0,508204 | 4,043 | 1,596 |
| SMIM10L2B | small integral membrane protein 10 like 2B | **1,057** | 0,577737 | 2,493 | 2,426 |
| IST1 | IST1, ESCRT-III associated factor | **1,057** | 0,030049 | 111,334 | 135,553 |
| EBF1 | early B-cell factor 1 | **1,057** | 0,525559 | 3,046 | 2,362 |
| 10.syys | septin 10 | **1,057** | 0,051199 | 69,681 | 61,998 |
| RALY | RALY heterogeneous nuclear ribonucleoprotein | **1,057** | 0,063077 | 69,792 | 74,385 |
| PEPD | peptidase D | **1,057** | 0,083355 | 41,044 | 40,034 |
| NIPA1 | non imprinted in Prader-Willi/Angelman syndrome 1 | **1,057** | 0,132914 | 23,929 | 21,390 |
| SMYD3 | SET and MYND domain containing 3 | **1,056** | 0,500202 | 1,994 | 2,043 |
| ZBED4 | zinc finger BED-type containing 4 | **1,056** | 0,13146 | 14,789 | 15,771 |
| TRH | thyrotropin releasing hormone | **1,056** | 0,798567 | 0,166 | 0,702 |
| HPS4 | HPS4, biogenesis of lysosomal organelles complex 3 subunit 2 | **1,056** | 0,054163 | 30,520 | 29,690 |
| PRCP | prolylcarboxypeptidase | **1,056** | 0,038399 | 47,303 | 34,479 |
| FAM171B | family with sequence similarity 171 member B | **1,056** | 0,436939 | 4,320 | 4,342 |
| TNFSF13 | TNF superfamily member 13 | **1,056** | 0,366165 | 3,268 | 4,853 |
| PRPF38A | pre-mRNA processing factor 38A | **1,056** | 0,080972 | 33,123 | 45,525 |
| GALC | galactosylceramidase | **1,056** | 0,209914 | 10,967 | 9,131 |
| ATP13A3 | ATPase 13A3 | **1,056** | 0,21006 | 308,025 | 284,068 |
| LINC01001 | long intergenic non-protein coding RNA 1001 | **1,056** | 0,763244 | 0,332 | 0,447 |
| SRD5A1 | steroid 5 alpha-reductase 1 | **1,056** | 0,112812 | 10,469 | 11,876 |
| ITPR2 | inositol 1,4,5-trisphosphate receptor type 2 | **1,056** | 0,211679 | 76,438 | 74,257 |
| BRD1 | bromodomain containing 1 | **1,056** | 0,075624 | 27,750 | 33,330 |
| GAB1 | GRB2 associated binding protein 1 | **1,056** | 0,241853 | 14,457 | 11,876 |
| ATP7A | ATPase copper transporting alpha | **1,056** | 0,286378 | 20,827 | 20,751 |
| FLJ20021 | uncharacterized LOC90024 | **1,056** | 0,235638 | 17,614 | 22,667 |
| CNBD2 | cyclic nucleotide binding domain containing 2 | **1,056** | 0,52444 | 1,662 | 1,852 |

| | | | | | |
|---|---|---|---|---|---|
| TOR1AIP1 | torsin 1A interacting protein 1 | **1,056** | 0,038275 | 117,538 | 118,186 |
| HMGXB4 | HMG-box containing 4 | **1,056** | 0,148057 | 28,083 | 30,584 |
| ERCC6L2 | ERCC excision repair 6 like 2 | **1,056** | 0,17886 | 14,291 | 13,345 |
| IMPG2 | interphotoreceptor matrix proteoglycan 2 | **1,056** | 0,709131 | 1,274 | 1,341 |
| CEP104 | centrosomal protein 104 | **1,056** | 0,059179 | 23,430 | 23,816 |
| SFXN3 | sideroflexin 3 | **1,056** | 0,160125 | 20,716 | 14,941 |
| TRIM56 | tripartite motif containing 56 | **1,056** | 0,040221 | 97,099 | 117,292 |
| TWF1 | twinfilin actin binding protein 1 | **1,056** | 0,130433 | 39,549 | 36,394 |
| ARNTL2 | aryl hydrocarbon receptor nuclear translocator like 2 | **1,056** | 0,223069 | 16,562 | 12,323 |
| PPARD | peroxisome proliferator activated receptor delta | **1,056** | 0,069758 | 49,574 | 52,612 |
| MIR210HG | MIR210 host gene | **1,056** | 0,733863 | 1,329 | 0,000 |
| LPAR2 | lysophosphatidic acid receptor 2 | **1,056** | 0,731488 | 0,499 | 0,447 |
| SGO2 | shugoshin 2 | **1,056** | 0,359469 | 5,151 | 3,703 |
| PJA2 | praja ring finger ubiquitin ligase 2 | **1,056** | 0,086774 | 204,279 | 192,699 |
| COMMD8 | COMM domain containing 8 | **1,056** | 0,252854 | 8,807 | 10,471 |
| WARS2 | tryptophanyl tRNA synthetase 2, mitochondrial | **1,056** | 0,203532 | 8,419 | 12,898 |
| COX10-AS1 | COX10 antisense RNA 1 | **1,055** | 0,512932 | 1,828 | 3,384 |
| RELA | RELA proto-oncogene, NF-kB subunit | **1,055** | 0,033566 | 209,319 | 228,391 |
| 6.maalis | membrane associated ring-CH-type finger 6 | **1,055** | 0,060808 | 103,635 | 104,203 |
| RNF114 | ring finger protein 114 | **1,055** | 0,051267 | 56,941 | 58,103 |
| DYNC1I1 | dynein cytoplasmic 1 intermediate chain 1 | **1,055** | 0,180661 | 12,851 | 9,514 |
| CFAP97 | cilia and flagella associated protein 97 | **1,055** | 0,162134 | 33,677 | 43,673 |
| ZNF578 | zinc finger protein 578 | **1,055** | 0,696784 | 0,665 | 1,149 |
| AMBRA1 | autophagy and beclin 1 regulator 1 | **1,055** | 0,117664 | 21,491 | 24,327 |
| HOOK3 | hook microtubule tethering protein 3 | **1,055** | 0,087327 | 48,300 | 46,163 |
| RNF14 | ring finger protein 14 | **1,055** | 0,084889 | 27,252 | 25,029 |
| BLOC1S6 | biogenesis of lysosomal organelles complex 1 subunit 6 | **1,055** | 0,054997 | 82,421 | 79,876 |
| ADGRG6 | adhesion G protein-coupled receptor G6 | **1,055** | 0,155104 | 31,074 | 41,055 |
| AMMECR1L | AMMECR1 like | **1,055** | 0,113566 | 43,592 | 36,650 |
| PTAFR | platelet activating factor receptor | **1,055** | 0,787997 | 0,111 | 0,575 |
| TUBGCP5 | tubulin gamma complex associated protein 5 | **1,055** | 0,753647 | 0,499 | 0,638 |
| MTMR9 | myotubularin related protein 9 | **1,055** | 0,582574 | 2,437 | 2,299 |
| ESCO1 | establishment of sister chromatid cohesion N-acetyltransferase 1 | **1,055** | 0,16553 | 13,626 | 15,452 |

| | | | | | |
|---|---|---|---|---|---|
| SH3TC2 | SH3 domain and tetratricopeptide repeats 2 | **1,055** | 0,474402 | 5,207 | 8,684 |
| LINC00205 | long intergenic non-protein coding RNA 205 | **1,055** | 0,103348 | 18,279 | 25,668 |
| ASAP1 | ArfGAP with SH3 domain, ankyrin repeat and PH domain 1 | **1,055** | 0,146504 | 31,351 | 33,393 |
| AASDH | aminoadipate-semialdehyde dehydrogenase | **1,055** | 0,207295 | 8,530 | 11,812 |
| ZNF627 | zinc finger protein 627 | **1,055** | 0,155666 | 12,629 | 12,515 |
| PHC2 | polyhomeotic homolog 2 | **1,055** | 0,039108 | 132,715 | 158,220 |
| MRPL34 | mitochondrial ribosomal protein L34 | **1,055** | 0,125492 | 15,620 | 16,346 |
| FNDC3B | fibronectin type III domain containing 3B | **1,055** | 0,127161 | 660,085 | 637,541 |
| ANGEL2 | angel homolog 2 | **1,055** | 0,175406 | 18,390 | 18,070 |
| CENPP | centromere protein P | **1,055** | 0,514736 | 2,991 | 2,809 |
| BBS4 | Bardet-Biedl syndrome 4 | **1,055** | 0,144048 | 24,150 | 34,224 |
| GTF2E1 | general transcription factor IIE subunit 1 | **1,055** | 0,280447 | 11,244 | 11,429 |
| WWTR1-AS1 | WWTR1 antisense RNA 1 | **1,055** | 0,674853 | 0,499 | 0,830 |
| FER | FER tyrosine kinase | **1,055** | 0,37136 | 17,836 | 23,050 |
| DDX17 | DEAD-box helicase 17 | **1,055** | 0,109142 | 340,650 | 371,606 |
| SEMA6D | semaphorin 6D | **1,055** | 0,319697 | 31,960 | 59,572 |
| ZNF517 | zinc finger protein 517 | **1,055** | 0,308876 | 5,761 | 7,534 |
| OSBPL11 | oxysterol binding protein like 11 | **1,055** | 0,114755 | 17,836 | 15,962 |
| ZNF337 | zinc finger protein 337 | **1,055** | 0,189465 | 9,139 | 9,450 |
| BAZ2A | bromodomain adjacent to zinc finger domain 2A | **1,055** | 0,097658 | 58,714 | 63,658 |
| TCAP | titin-cap | **1,054** | 0,763008 | 0,443 | 0,383 |
| ZNF484 | zinc finger protein 484 | **1,054** | 0,278553 | 6,204 | 8,300 |
| ORC4 | origin recognition complex subunit 4 | **1,054** | 0,1366 | 23,042 | 25,412 |
| ZBTB41 | zinc finger and BTB domain containing 41 | **1,054** | 0,218199 | 21,491 | 18,006 |
| HPS3 | HPS3, biogenesis of lysosomal organelles complex 2 subunit 1 | **1,054** | 0,120835 | 24,593 | 22,092 |
| SPEF2 | sperm flagellar 2 | **1,054** | 0,395752 | 3,323 | 6,130 |
| CTDSPL2 | CTD small phosphatase like 2 | **1,054** | 0,233046 | 20,716 | 21,645 |
| CCDC28A | coiled-coil domain containing 28A | **1,054** | 0,19646 | 11,798 | 11,365 |
| ARID1B | AT-rich interaction domain 1B | **1,054** | 0,129546 | 61,262 | 57,401 |
| RANBP10 | RAN binding protein 10 | **1,054** | 0,119378 | 23,264 | 27,519 |
| EPS15L1 | epidermal growth factor receptor pathway substrate 15 like 1 | **1,054** | 0,077516 | 30,465 | 34,607 |
| ST3GAL1 | ST3 beta-galactoside alpha-2,3-sialyltransferase 1 | **1,054** | 0,08883 | 506,100 | 496,944 |
| ZSWIM3 | zinc finger SWIM-type containing 3 | **1,054** | 0,419973 | 4,265 | 3,703 |

| | | | | | |
|---|---|---|---|---|---|
| SH3KBP1 | SH3 domain containing kinase binding protein 1 | **1,054** | 0,134976 | 43,537 | 38,501 |
| IFFO1 | intermediate filament family orphan 1 | **1,054** | 0,224214 | 9,638 | 7,981 |
| FBXL4 | F-box and leucine rich repeat protein 4 | **1,054** | 0,114622 | 15,122 | 15,516 |
| DCUN1D2 | defective in cullin neddylation 1 domain containing 2 | **1,054** | 0,236733 | 11,355 | 12,642 |
| DNAJC8 | DnaJ heat shock protein family (Hsp40) member C8 | **1,054** | 0,093362 | 60,486 | 72,725 |
| TERF2 | telomeric repeat binding factor 2 | **1,054** | 0,079218 | 29,135 | 34,862 |
| BANP | BTG3 associated nuclear protein | **1,054** | 0,166974 | 11,133 | 14,047 |
| KCNT2 | potassium sodium-activated channel subfamily T member 2 | **1,054** | 0,530186 | 2,603 | 2,618 |
| BROX | BRO1 domain and CAAX motif containing | **1,054** | 0,067434 | 50,959 | 52,357 |
| ZNF189 | zinc finger protein 189 | **1,054** | 0,135674 | 15,288 | 16,920 |
| TOB2 | transducer of ERBB2, 2 | **1,054** | 0,061045 | 32,403 | 36,011 |
| ATG12 | autophagy related 12 | **1,054** | 0,085004 | 44,644 | 47,440 |
| SUZ12 | SUZ12 polycomb repressive complex 2 subunit | **1,054** | 0,170508 | 24,095 | 23,433 |
| KMT2C | lysine methyltransferase 2C | **1,054** | 0,323984 | 43,426 | 46,994 |
| S100A11 | S100 calcium binding protein A11 | **1,054** | 0,082287 | 231,586 | 239,756 |
| TNIP3 | TNFAIP3 interacting protein 3 | **1,054** | 0,675243 | 1,329 | 1,788 |
| RAB3GAP1 | RAB3 GTPase activating protein catalytic subunit 1 | **1,053** | 0,056563 | 66,080 | 75,024 |
| TYW1 | tRNA-yW synthesizing protein 1 homolog | **1,053** | 0,197009 | 10,635 | 11,685 |
| UBAP2 | ubiquitin associated protein 2 | **1,053** | 0,164201 | 17,393 | 20,560 |
| GAS8 | growth arrest specific 8 | **1,053** | 0,226833 | 9,250 | 10,727 |
| MAP3K2 | mitogen-activated protein kinase kinase kinase 2 | **1,053** | 0,179073 | 51,125 | 47,185 |
| STK4 | serine/threonine kinase 4 | **1,053** | 0,185247 | 24,759 | 30,903 |
| SCO2 | SCO2, cytochrome c oxidase assembly protein | **1,053** | 0,483743 | 2,603 | 1,469 |
| WDR60 | WD repeat domain 60 | **1,053** | 0,273834 | 18,113 | 19,219 |
| IL6ST | interleukin 6 signal transducer | **1,053** | 0,17862 | 643,523 | 685,173 |
| TCF12 | transcription factor 12 | **1,053** | 0,206437 | 39,050 | 36,905 |
| LIPH | lipase H | **1,053** | 0,657539 | 0,886 | 0,766 |
| NIM1K | NIM1 serine/threonine protein kinase | **1,053** | 0,373437 | 6,647 | 7,534 |
| SORL1 | sortilin related receptor 1 | **1,053** | 0,443316 | 3,656 | 5,044 |
| SH3PXD2B | SH3 and PX domains 2B | **1,053** | 0,126922 | 83,750 | 90,348 |
| TXLNG | taxilin gamma | **1,053** | 0,155247 | 18,334 | 22,220 |
| SPPL2A | signal peptide peptidase like 2A | **1,053** | 0,028115 | 271,467 | 238,671 |
| KMT2A | lysine methyltransferase 2A | **1,053** | 0,336156 | 71,786 | 91,880 |

| | | | | | |
|---|---|---|---|---|---|
| SEMA4B | semaphorin 4B | **1,053** | 0,069833 | 29,855 | 31,478 |
| MOSPD2 | motile sperm domain containing 2 | **1,053** | 0,176743 | 19,276 | 18,261 |
| DNAJC3-AS1 | DNAJC3 antisense RNA 1 (head to head) | **1,053** | 0,392837 | 3,157 | 4,278 |
| TSPY26P | testis specific protein, Y-linked 26, pseudogene | **1,053** | 0,284416 | 8,530 | 6,960 |
| RABIF | RAB interacting factor | **1,053** | 0,175667 | 16,783 | 14,047 |
| METTL4 | methyltransferase like 4 | **1,053** | 0,257348 | 9,638 | 6,704 |
| MLPH | melanophilin | **1,053** | 0,494761 | 2,880 | 1,660 |
| CCDC66 | coiled-coil domain containing 66 | **1,053** | 0,228173 | 9,472 | 10,663 |
| OMA1 | OMA1 zinc metallopeptidase | **1,053** | 0,369993 | 7,865 | 10,599 |
| PLA2R1 | phospholipase A2 receptor 1 | **1,053** | 0,20447 | 20,771 | 19,347 |
| LIMA1 | LIM domain and actin binding 1 | **1,053** | 0,162683 | 46,915 | 45,397 |
| MMP2 | matrix metallopeptidase 2 | **1,053** | 0,136917 | 42,650 | 72,533 |
| EIF3C | eukaryotic translation initiation factor 3 subunit C | **1,053** | 0,611767 | 0,831 | 1,469 |
| DCAF7 | DDB1 and CUL4 associated factor 7 | **1,053** | 0,059478 | 63,256 | 67,362 |
| LIMCH1 | LIM and calponin homology domains 1 | **1,053** | 0,095354 | 33,068 | 43,035 |
| ABLIM2 | actin binding LIM protein family member 2 | **1,053** | 0,665159 | 0,665 | 0,894 |
| MDM1 | Mdm1 nuclear protein | **1,053** | 0,355433 | 5,041 | 6,130 |
| TTLL5 | tubulin tyrosine ligase like 5 | **1,053** | 0,348231 | 10,746 | 10,982 |
| PTGER4 | prostaglandin E receptor 4 | **1,053** | 0,279579 | 10,303 | 13,025 |
| SETX | senataxin | **1,053** | 0,16314 | 71,066 | 80,642 |
| BCAP29 | B-cell receptor associated protein 29 | **1,053** | 0,161456 | 15,731 | 15,899 |
| NBPF20 | NBPF member 20 | **1,053** | 0,479482 | 2,659 | 2,746 |
| LINC01481 | long intergenic non-protein coding RNA 1481 | **1,053** | 0,722409 | 0,499 | 1,085 |
| CAHM | colon adenocarcinoma hypermethylated (non-protein coding) | **1,053** | 0,738817 | 0,222 | 0,766 |
| JPX | JPX transcript, XIST activator (non-protein coding) | **1,053** | 0,205187 | 19,276 | 26,753 |
| ZNF646 | zinc finger protein 646 | **1,053** | 0,16076 | 21,436 | 23,241 |
| ZFHX2-AS1 | ZFHX2 antisense RNA 1 | **1,053** | 0,687573 | 0,886 | 1,660 |
| IGFBP5 | insulin like growth factor binding protein 5 | **1,053** | 0,3324 | 157,363 | 191,422 |
| CSPP1 | centrosome and spindle pole associated protein 1 | **1,053** | 0,249789 | 11,023 | 10,408 |
| NUDT11 | nudix hydrolase 11 | **1,052** | 0,497323 | 4,099 | 2,746 |
| DPYSL4 | dihydropyrimidinase like 4 | **1,052** | 0,395751 | 2,603 | 3,703 |
| DNAJC16 | DnaJ heat shock protein family (Hsp40) member C16 | **1,052** | 0,189593 | 42,706 | 40,417 |
| PLCE1 | phospholipase C epsilon 1 | **1,052** | 0,653894 | 2,714 | 2,490 |

| | | | | | |
|---|---|---|---|---|---|
| UBR2 | ubiquitin protein ligase E3 component n-recognin 2 | **1,052** | 0,129337 | 45,586 | 45,525 |
| SHC4 | SHC adaptor protein 4 | **1,052** | 0,595993 | 1,994 | 2,490 |
| TLK1 | tousled like kinase 1 | **1,052** | 0,153771 | 33,788 | 34,607 |
| ZNF616 | zinc finger protein 616 | **1,052** | 0,298754 | 6,481 | 7,726 |
| TRIM3 | tripartite motif containing 3 | **1,052** | 0,308482 | 8,142 | 5,300 |
| SLC39A14 | solute carrier family 39 member 14 | **1,052** | 0,064403 | 5894,898 | 5946,659 |
| SLC29A1 | solute carrier family 29 member 1 (Augustine blood group) | **1,052** | 0,071354 | 70,512 | 74,832 |
| BAG5 | BCL2 associated athanogene 5 | **1,052** | 0,080901 | 54,725 | 55,805 |
| PIK3C3 | phosphatidylinositol 3-kinase catalytic subunit type 3 | **1,052** | 0,13862 | 28,858 | 28,541 |
| MFAP3 | microfibrillar associated protein 3 | **1,052** | 0,227147 | 25,978 | 23,688 |
| NIPAL2 | NIPA like domain containing 2 | **1,052** | 0,359606 | 7,699 | 5,427 |
| GNAI3 | G protein subunit alpha i3 | **1,052** | 0,055516 | 78,045 | 79,493 |
| CHST15 | carbohydrate sulfotransferase 15 | **1,052** | 0,131491 | 20,827 | 34,798 |
| C2CD4D | C2 calcium dependent domain containing 4D | **1,052** | 0,704401 | 0,499 | 1,149 |
| CDO1 | cysteine dioxygenase type 1 | **1,052** | 0,113287 | 40,989 | 52,038 |
| GCNT3 | glucosaminyl (N-acetyl) transferase 3, mucin type | **1,052** | 0,523784 | 4,154 | 4,214 |
| ULBP2 | UL16 binding protein 2 | **1,052** | 0,204647 | 19,331 | 27,392 |
| COQ10B | coenzyme Q10B | **1,052** | 0,072826 | 51,291 | 52,421 |
| C1QTNF1 | C1q and TNF related 1 | **1,052** | 0,084343 | 245,988 | 172,012 |
| FGF14-AS2 | FGF14 antisense RNA 2 | **1,052** | 0,651872 | 2,271 | 2,873 |
| PLEKHA7 | pleckstrin homology domain containing A7 | **1,052** | 0,440185 | 4,043 | 5,300 |
| CEBPG | CCAAT/enhancer binding protein gamma | **1,052** | 0,037812 | 66,080 | 71,895 |
| ZNF222 | zinc finger protein 222 | **1,052** | 0,532755 | 3,213 | 2,554 |
| JARID2 | jumonji and AT-rich interaction domain containing 2 | **1,052** | 0,252342 | 12,795 | 16,218 |
| ISOC1 | isochorismatase domain containing 1 | **1,052** | 0,112601 | 19,553 | 29,243 |
| ZNF619 | zinc finger protein 619 | **1,052** | 0,310295 | 6,148 | 6,321 |
| AC024270.1 | Hepatoma-derived growth factor-related protein 3 | **1,052** | 0,156271 | 27,141 | 32,436 |
| SPAG5 | sperm associated antigen 5 | **1,052** | 0,397269 | 6,868 | 4,725 |
| ZNF486 | zinc finger protein 486 | **1,052** | 0,366032 | 4,930 | 6,130 |
| SNHG1 | small nucleolar RNA host gene 1 | **1,052** | 0,105135 | 24,870 | 42,141 |
| TSGA10 | testis specific 10 | **1,052** | 0,461786 | 2,049 | 3,320 |
| TXNL4B | thioredoxin like 4B | **1,052** | 0,164391 | 15,842 | 23,369 |
| ZNF562 | zinc finger protein 562 | **1,052** | 0,202392 | 19,664 | 25,285 |

| | | | | | |
|---|---|---|---|---|---|
| ZNF281 | zinc finger protein 281 | **1,052** | 0,150811 | 19,442 | 18,900 |
| MYH9 | myosin heavy chain 9 | **1,052** | 0,132419 | 518,673 | 510,863 |
| AGRN | agrin | **1,052** | 0,074319 | 120,252 | 101,074 |
| CDC6 | cell division cycle 6 | **1,052** | 0,238435 | 11,632 | 9,258 |
| TDG | thymine DNA glycosylase | **1,052** | 0,271198 | 7,976 | 7,917 |
| TUBG2 | tubulin gamma 2 | **1,052** | 0,300827 | 8,309 | 8,620 |
| SHOX2 | short stature homeobox 2 | **1,052** | 0,440352 | 3,545 | 4,597 |
| TMEM173 | transmembrane protein 173 | **1,052** | 0,068557 | 27,252 | 29,562 |
| CACUL1 | CDK2 associated cullin domain 1 | **1,052** | 0,058088 | 65,416 | 84,920 |
| FIG4 | FIG4 phosphoinositide 5-phosphatase | **1,052** | 0,276759 | 11,577 | 7,598 |
| RCOR3 | REST corepressor 3 | **1,052** | 0,223566 | 15,952 | 15,771 |
| TAOK1 | TAO kinase 1 | **1,052** | 0,30889 | 77,989 | 73,747 |
| SLX4IP | SLX4 interacting protein | **1,051** | 0,254797 | 6,481 | 8,237 |
| AP2A2 | adaptor related protein complex 2 alpha 2 subunit | **1,051** | 0,58391 | 1,108 | 1,405 |
| VPS50 | VPS50, EARP/GARPII complex subunit | **1,051** | 0,218831 | 12,518 | 12,323 |
| RBBP6 | RB binding protein 6, ubiquitin ligase | **1,051** | 0,063633 | 63,200 | 73,044 |
| ST13 | ST13, Hsp70 interacting protein | **1,051** | 0,084581 | 61,926 | 55,677 |
| DDX5 | DEAD-box helicase 5 | **1,051** | 0,02765 | 417,586 | 463,550 |
| ARMC1 | armadillo repeat containing 1 | **1,051** | 0,186043 | 20,107 | 20,368 |
| MYOC | myocilin | **1,051** | 0,524434 | 0,499 | 1,532 |
| GHRLOS | ghrelin opposite strand/antisense RNA | **1,051** | 0,679324 | 0,720 | 0,575 |
| ISYNA1 | inositol-3-phosphate synthase 1 | **1,051** | 0,440964 | 2,936 | 4,342 |
| CD47 | CD47 molecule | **1,051** | 0,076782 | 68,407 | 72,278 |
| KMT5C | lysine methyltransferase 5C | **1,051** | 0,670321 | 2,216 | 2,171 |
| C10orf10 | chromosome 10 open reading frame 10 | **1,051** | 0,16771 | 61,040 | 44,056 |
| NPAT | nuclear protein, coactivator of histone transcription | **1,051** | 0,282132 | 17,171 | 19,155 |
| ELF3-AS1 | ELF3 antisense RNA 1 | **1,051** | 0,4428 | 3,767 | 3,065 |
| HTRA1 | HtrA serine peptidase 1 | **1,051** | 0,053129 | 824,095 | 432,838 |
| CEBPD | CCAAT/enhancer binding protein delta | **1,051** | 0,118026 | 313,398 | 313,184 |
| RAD52 | RAD52 homolog, DNA repair protein | **1,051** | 0,207993 | 6,425 | 10,471 |
| PLEKHM3 | pleckstrin homology domain containing M3 | **1,051** | 0,324311 | 17,725 | 20,624 |
| PTP4A1 | protein tyrosine phosphatase type IVA, member 1 | **1,051** | 0,093734 | 196,524 | 144,109 |
| KDM2A | lysine demethylase 2A | **1,051** | 0,135285 | 125,680 | 163,136 |

| | | | | | |
|---|---|---|---|---|---|
| PCDHGB6 | protocadherin gamma subfamily B, 6 | **1,051** | 0,267154 | 6,425 | 9,194 |
| TRIM66 | tripartite motif containing 66 | **1,051** | 0,286903 | 12,130 | 20,751 |
| SPAST | spastin | **1,051** | 0,172706 | 14,291 | 14,941 |
| GOLGA2P7 | golgin A2 pseudogene 7 | **1,051** | 0,716936 | 0,554 | 0,447 |
| CEL | carboxyl ester lipase | **1,051** | 0,732837 | 0,609 | 1,085 |
| TTLL3 | tubulin tyrosine ligase like 3 | **1,051** | 0,529271 | 3,046 | 3,703 |
| HSP90AA1 | heat shock protein 90 alpha family class A member 1 | **1,051** | 0,103454 | 525,874 | 493,368 |
| CREBBP | CREB binding protein | **1,051** | 0,254313 | 97,099 | 119,591 |
| CXorf23 | chromosome X open reading frame 23 | **1,051** | 0,364402 | 8,253 | 6,066 |
| NDUFA2 | NADH:ubiquinone oxidoreductase subunit A2 | **1,051** | 0,251689 | 14,457 | 17,048 |
| CPOX | coproporphyrinogen oxidase | **1,051** | 0,221888 | 15,675 | 11,301 |
| AZIN1 | antizyme inhibitor 1 | **1,050** | 0,043943 | 101,032 | 132,361 |
| MAF | MAF bZIP transcription factor | **1,050** | 0,14791 | 18,611 | 16,473 |
| LRIG1 | leucine rich repeats and immunoglobulin like domains 1 | **1,050** | 0,248913 | 37,555 | 35,628 |
| ADCY9 | adenylate cyclase 9 | **1,050** | 0,197648 | 13,183 | 12,578 |
| FIBCD1 | fibrinogen C domain containing 1 | **1,050** | 0,556258 | 0,609 | 0,128 |
| DNPH1 | 2'-deoxynucleoside 5'-phosphate N-hydrolase 1 | **1,050** | 0,412751 | 6,314 | 5,236 |
| VTA1 | vesicle trafficking 1 | **1,050** | 0,111405 | 45,420 | 51,463 |
| PHF20 | PHD finger protein 20 | **1,050** | 0,082503 | 64,862 | 68,383 |
| CMAHP | cytidine monophospho-N-acetylneuraminic acid hydroxylase, pseudogene | **1,050** | 0,190087 | 28,747 | 74,385 |
| EHD4 | EH domain containing 4 | **1,050** | 0,139723 | 26,476 | 29,307 |
| ARIH2OS | ariadne homolog 2 opposite strand | **1,050** | 0,643766 | 0,775 | 1,149 |
| ARHGEF40 | Rho guanine nucleotide exchange factor 40 | **1,050** | 0,109672 | 84,692 | 64,488 |
| IMMP1L | inner mitochondrial membrane peptidase subunit 1 | **1,050** | 0,494327 | 1,828 | 2,809 |
| ZNF33A | zinc finger protein 33A | **1,050** | 0,167202 | 23,596 | 25,476 |
| SPIN4 | spindlin family member 4 | **1,050** | 0,559122 | 3,268 | 2,426 |
| NHLRC3 | NHL repeat containing 3 | **1,050** | 0,273066 | 11,189 | 11,748 |
| SP140L | SP140 nuclear body protein like | **1,050** | 0,351441 | 8,253 | 9,194 |
| RAB9A | RAB9A, member RAS oncogene family | **1,050** | 0,28059 | 17,669 | 16,026 |
| CERK | ceramide kinase | **1,050** | 0,085059 | 60,763 | 48,845 |
| TOP3B | topoisomerase (DNA) III beta | **1,050** | 0,296508 | 8,641 | 9,386 |
| FOXK1 | forkhead box K1 | **1,050** | 0,116057 | 54,282 | 51,718 |
| ARMC2 | armadillo repeat containing 2 | **1,050** | 0,682773 | 1,329 | 2,235 |

| | | | | | |
|---|---|---|---|---|---|
| PLA2G12A | phospholipase A2 group XIIA | **1,050** | 0,249995 | 7,201 | 6,768 |
| ARHGEF10L | Rho guanine nucleotide exchange factor 10 like | **1,050** | 0,149997 | 26,421 | 35,054 |
| CACNB3 | calcium voltage-gated channel auxiliary subunit beta 3 | **1,050** | 0,659268 | 1,329 | 1,979 |
| FAM122A | family with sequence similarity 122A | **1,050** | 0,159852 | 13,294 | 12,770 |
| LINC01096 | long intergenic non-protein coding RNA 1096 | **1,050** | 0,536762 | 2,437 | 2,426 |
| TNRC6B | trinucleotide repeat containing 6B | **1,050** | 0,270376 | 35,117 | 41,949 |
| RTF1 | RTF1 homolog, Paf1/RNA polymerase II complex component | **1,050** | 0,105276 | 43,094 | 42,779 |
| TMEM169 | transmembrane protein 169 | **1,050** | 0,653636 | 1,219 | 1,341 |
| PIPSL | PIP5K1A and PSMD4-like, pseudogene | **1,050** | 0,81305 | 0,388 | 0,128 |
| DKFZP434I0714 | uncharacterized protein DKFZP434I0714 | **1,050** | 0,411712 | 5,816 | 7,726 |
| TOP1 | topoisomerase (DNA) I | **1,050** | 0,083839 | 72,173 | 85,367 |
| DLGAP1-AS1 | DLGAP1 antisense RNA 1 | **1,050** | 0,144573 | 20,494 | 18,197 |
| LRCH4 | leucine rich repeats and calponin homology domain containing 4 | **1,050** | 0,402675 | 3,822 | 5,044 |
| ZNF2 | zinc finger protein 2 | **1,050** | 0,367015 | 4,930 | 6,896 |
| THRB | thyroid hormone receptor beta | **1,050** | 0,212888 | 16,229 | 25,732 |
| TMTC2 | transmembrane and tetratricopeptide repeat containing 2 | **1,050** | 0,392402 | 5,317 | 7,981 |
| THUMPD1 | THUMP domain containing 1 | **1,049** | 0,086459 | 31,351 | 35,564 |
| MEG8 | maternally expressed 8, small nucleolar RNA host gene | **1,049** | 0,558419 | 3,545 | 1,596 |
| WIF1 | WNT inhibitory factor 1 | **1,049** | 0,246188 | 6,758 | 4,725 |
| CCNK | cyclin K | **1,049** | 0,075688 | 49,020 | 60,147 |
| PRKRA | protein activator of interferon induced protein kinase EIF2AK2 | **1,049** | 0,211717 | 10,303 | 9,258 |
| GALNT11 | polypeptide N-acetylgalactosaminyltransferase 11 | **1,049** | 0,094337 | 44,811 | 41,119 |
| APOBEC3B | apolipoprotein B mRNA editing enzyme catalytic subunit 3B | **1,049** | 0,715079 | 3,157 | 0,447 |
| FAXDC2 | fatty acid hydroxylase domain containing 2 | **1,049** | 0,371916 | 2,382 | 2,682 |
| DDX3X | DEAD-box helicase 3, X-linked | **1,049** | 0,129114 | 358,042 | 381,439 |
| TBC1D16 | TBC1 domain family member 16 | **1,049** | 0,168052 | 30,742 | 27,583 |
| ATP6V0E2 | ATPase H+ transporting V0 subunit e2 | **1,049** | 0,510722 | 3,268 | 3,384 |
| AASDHPPT | aminoadipate-semialdehyde dehydrogenase-phosphopantetheinyl transferase | **1,049** | 0,223982 | 22,987 | 20,815 |
| PCNA | proliferating cell nuclear antigen | **1,049** | 0,089453 | 55,667 | 67,745 |
| COLGALT2 | collagen beta(1-O)galactosyltransferase 2 | **1,049** | 0,196658 | 144,458 | 159,561 |
| BAHCC1 | BAH domain and coiled-coil containing 1 | **1,049** | 0,327008 | 10,746 | 12,068 |
| VHL | von Hippel-Lindau tumor suppressor | **1,049** | 0,110326 | 51,236 | 41,630 |
| MAN1A2 | mannosidase alpha class 1A member 2 | **1,049** | 0,169174 | 65,360 | 72,661 |

| | | | | | |
|---|---|---|---|---|---|
| ANKRD26 | ankyrin repeat domain 26 | **1,049** | 0,309958 | 8,585 | 9,961 |
| YWHAQ | tyrosine 3-monooxygenase/tryptophan 5-monooxygenase activation protein theta | **1,049** | 0,052528 | 252,468 | 225,518 |
| ROCK1 | Rho associated coiled-coil containing protein kinase 1 | **1,049** | 0,094621 | 61,649 | 64,361 |
| C9orf40 | chromosome 9 open reading frame 40 | **1,049** | 0,548279 | 3,157 | 2,171 |
| RAB11FIP2 | RAB11 family interacting protein 2 | **1,049** | 0,336987 | 17,171 | 18,389 |
| IFI16 | interferon gamma inducible protein 16 | **1,049** | 0,335166 | 4,764 | 8,045 |
| R3HDM2 | R3H domain containing 2 | **1,049** | 0,102247 | 19,996 | 22,539 |
| FAM105A | family with sequence similarity 105 member A | **1,049** | 0,757757 | 0,997 | 0,575 |
| RAB2A | RAB2A, member RAS oncogene family | **1,049** | 0,066467 | 142,464 | 138,554 |
| TBC1D9B | TBC1 domain family member 9B | **1,049** | 0,244127 | 24,926 | 20,815 |
| PPFIA4 | PTPRF interacting protein alpha 4 | **1,049** | 0,182727 | 12,297 | 10,088 |
| PKD1P6 | polycystin 1, transient receptor potential channel interacting pseudogene 6 | **1,049** | 0,80472 | 0,277 | 0,319 |
| SCART1 | scavenger receptor family member expressed on T-cells 1 | **1,049** | 0,728981 | 0,886 | 0,766 |
| FAM220A | family with sequence similarity 220 member A | **1,049** | 0,124146 | 14,069 | 17,367 |
| TRPV4 | transient receptor potential cation channel subfamily V member 4 | **1,049** | 0,106929 | 150,163 | 196,083 |
| ATP6V1E1 | ATPase H+ transporting V1 subunit E1 | **1,049** | 0,094978 | 112,553 | 122,656 |
| LNPEP | leucyl and cystinyl aminopeptidase | **1,049** | 0,238294 | 69,515 | 57,784 |
| PPP2R5E | protein phosphatase 2 regulatory subunit B'epsilon | **1,049** | 0,119983 | 33,843 | 32,755 |
| WWP1 | WW domain containing E3 ubiquitin protein ligase 1 | **1,048** | 0,179127 | 25,867 | 24,710 |
| ADAMTSL4 | ADAMTS like 4 | **1,048** | 0,475334 | 3,046 | 4,342 |
| HEXA | hexosaminidase subunit alpha | **1,048** | 0,081052 | 82,698 | 64,680 |
| ULK1 | unc-51 like autophagy activating kinase 1 | **1,048** | 0,158394 | 40,435 | 33,840 |
| COL18A1 | collagen type XVIII alpha 1 chain | **1,048** | 0,36773 | 13,958 | 14,047 |
| AK2 | adenylate kinase 2 | **1,048** | 0,085017 | 57,052 | 71,193 |
| SLC20A1 | solute carrier family 20 member 1 | **1,048** | 0,096904 | 318,050 | 468,850 |
| REV1 | REV1, DNA directed polymerase | **1,048** | 0,196467 | 21,658 | 24,518 |
| ANKMY2 | ankyrin repeat and MYND domain containing 2 | **1,048** | 0,232099 | 16,783 | 18,325 |
| PLSCR4 | phospholipid scramblase 4 | **1,048** | 0,309765 | 14,180 | 13,664 |
| RB1CC1 | RB1 inducible coiled-coil 1 | **1,048** | 0,127839 | 102,306 | 95,200 |
| USP53 | ubiquitin specific peptidase 53 | **1,048** | 0,177706 | 119,421 | 143,343 |
| AGAP1 | ArfGAP with GTPase domain, ankyrin repeat and PH domain 1 | **1,048** | 0,211493 | 21,325 | 23,816 |
| MRM3 | mitochondrial rRNA methyltransferase 3 | **1,048** | 0,304751 | 11,687 | 15,324 |
| ZNF260 | zinc finger protein 260 | **1,048** | 0,251776 | 12,851 | 10,791 |

| | | | | | |
|---|---|---|---|---|---|
| USP46 | ubiquitin specific peptidase 46 | **1,048** | 0,175966 | 15,454 | 13,281 |
| PTPRA | protein tyrosine phosphatase, receptor type A | **1,048** | 0,090486 | 62,868 | 56,571 |
| ZNF264 | zinc finger protein 264 | **1,048** | 0,303988 | 14,623 | 15,260 |
| RAB22A | RAB22A, member RAS oncogene family | **1,048** | 0,084762 | 50,017 | 48,398 |
| HAR1B | highly accelerated region 1B (non-protein coding) | **1,048** | 0,774009 | 0,609 | 0,192 |
| C3 | complement C3 | **1,048** | 0,140109 | 142,076 | 163,903 |
| BSDC1 | BSD domain containing 1 | **1,048** | 0,098943 | 76,715 | 74,002 |
| C1orf27 | chromosome 1 open reading frame 27 | **1,048** | 0,19934 | 25,036 | 27,519 |
| P3H2 | prolyl 3-hydroxylase 2 | **1,048** | 0,074575 | 694,094 | 511,054 |
| LTF | lactotransferrin | **1,048** | 0,699924 | 0,166 | 0,383 |
| ITGB8 | integrin subunit beta 8 | **1,048** | 0,154991 | 291,352 | 250,547 |
| HARS2 | histidyl-tRNA synthetase 2, mitochondrial | **1,048** | 0,137635 | 23,375 | 27,775 |
| ATG2B | autophagy related 2B | **1,048** | 0,207576 | 29,191 | 36,331 |
| AHCYL1 | adenosylhomocysteinase like 1 | **1,048** | 0,062635 | 78,765 | 105,161 |
| CRLF3 | cytokine receptor like factor 3 | **1,048** | 0,310302 | 11,133 | 13,536 |
| HCFC2 | host cell factor C2 | **1,048** | 0,354 | 10,413 | 9,833 |
| FOXA2 | forkhead box A2 | **1,048** | 0,597858 | 1,939 | 1,085 |
| GALNT10 | polypeptide N-acetylgalactosaminyltransferase 10 | **1,047** | 0,144228 | 87,295 | 83,452 |
| FAM126B | family with sequence similarity 126 member B | **1,047** | 0,367379 | 13,737 | 12,962 |
| C6orf62 | chromosome 6 open reading frame 62 | **1,047** | 0,054124 | 318,272 | 329,785 |
| C17orf49 | chromosome 17 open reading frame 49 | **1,047** | 0,645124 | 1,329 | 1,596 |
| PAK1 | p21 (RAC1) activated kinase 1 | **1,047** | 0,435675 | 6,481 | 4,789 |
| UBE2D2 | ubiquitin conjugating enzyme E2 D2 | **1,047** | 0,092822 | 69,570 | 73,747 |
| WWOX | WW domain containing oxidoreductase | **1,047** | 0,502354 | 3,323 | 4,023 |
| PXN | paxillin | **1,047** | 0,118005 | 77,879 | 79,812 |
| TGFB3 | transforming growth factor beta 3 | **1,047** | 0,516831 | 2,326 | 2,554 |
| GPRC5A | G protein-coupled receptor class C group 5 member A | **1,047** | 0,131454 | 68,850 | 121,059 |
| PER1 | period circadian clock 1 | **1,047** | 0,141126 | 35,450 | 32,691 |
| ARL8A | ADP ribosylation factor like GTPase 8A | **1,047** | 0,072426 | 44,478 | 38,885 |
| SLC11A2 | solute carrier family 11 member 2 | **1,047** | 0,070226 | 263,048 | 274,235 |
| RAB33B | RAB33B, member RAS oncogene family | **1,047** | 0,231429 | 18,888 | 23,688 |
| NDEL1 | nudE neurodevelopment protein 1 like 1 | **1,047** | 0,09754 | 59,157 | 56,124 |
| GTPBP10 | GTP binding protein 10 | **1,047** | 0,29379 | 14,955 | 17,623 |

| | | | | | |
|---|---|---|---|---|---|
| UBE2I | ubiquitin conjugating enzyme E2 I | **1,047** | 0,080377 | 68,573 | 79,812 |
| UBXN2A | UBX domain protein 2A | **1,047** | 0,177543 | 15,232 | 14,175 |
| GSAP | gamma-secretase activating protein | **1,047** | 0,206914 | 22,655 | 28,796 |
| AMZ2P1 | archaelysin family metallopeptidase 2 pseudogene 1 | **1,047** | 0,430463 | 5,207 | 6,193 |
| LRRC46 | leucine rich repeat containing 46 | **1,047** | 0,695456 | 1,108 | 1,341 |
| EIF4EBP3 | eukaryotic translation initiation factor 4E binding protein 3 | **1,047** | 0,829966 | 0,332 | 0,830 |
| ARHGEF7 | Rho guanine nucleotide exchange factor 7 | **1,047** | 0,085532 | 33,954 | 35,564 |
| RORC | RAR related orphan receptor C | **1,047** | 0,674969 | 0,332 | 1,149 |
| SNW1 | SNW domain containing 1 | **1,047** | 0,061997 | 59,046 | 66,915 |
| TACC1 | transforming acidic coiled-coil containing protein 1 | **1,047** | 0,127241 | 101,752 | 120,165 |
| ZNF518A | zinc finger protein 518A | **1,047** | 0,283514 | 13,958 | 14,685 |
| ATP8B2 | ATPase phospholipid transporting 8B2 | **1,047** | 0,124431 | 189,102 | 157,582 |
| ZNF638 | zinc finger protein 638 | **1,047** | 0,127339 | 92,502 | 107,779 |
| RAB24 | RAB24, member RAS oncogene family | **1,047** | 0,472242 | 3,988 | 5,300 |
| TPT1 | tumor protein, translationally-controlled 1 | **1,047** | 0,089023 | 1120,377 | 1169,538 |
| ABI2 | abl interactor 2 | **1,047** | 0,205698 | 29,800 | 30,648 |
| PRPSAP1 | phosphoribosyl pyrophosphate synthetase associated protein 1 | **1,047** | 0,147813 | 26,033 | 25,668 |
| SMIM29 | small integral membrane protein 29 | **1,047** | 0,184409 | 34,342 | 38,501 |
| TK1 | thymidine kinase 1 | **1,047** | 0,523713 | 6,868 | 2,043 |
| CCDC174 | coiled-coil domain containing 174 | **1,047** | 0,095126 | 21,879 | 23,816 |
| KIAA1328 | KIAA1328 | **1,047** | 0,49602 | 3,877 | 4,469 |
| KIF1BP | KIF1 binding protein | **1,047** | 0,108145 | 45,254 | 43,226 |
| IREB2 | iron responsive element binding protein 2 | **1,047** | 0,273126 | 28,914 | 33,010 |
| LINC00963 | long intergenic non-protein coding RNA 963 | **1,047** | 0,089781 | 36,558 | 34,734 |
| DICER1-AS1 | DICER1 antisense RNA 1 | **1,047** | 0,679914 | 0,665 | 1,022 |
| ZNF324 | zinc finger protein 324 | **1,047** | 0,310556 | 8,309 | 7,598 |
| GUCY1A3 | guanylate cyclase 1 soluble subunit alpha | **1,047** | 0,788466 | 0,222 | 1,788 |
| KLHL9 | kelch like family member 9 | **1,047** | 0,113088 | 73,060 | 70,746 |
| LRRK1 | leucine rich repeat kinase 1 | **1,046** | 0,290035 | 12,574 | 13,025 |
| ETHE1 | ETHE1, persulfide dioxygenase | **1,046** | 0,280883 | 18,390 | 15,643 |
| ESF1 | ESF1 nucleolar pre-rRNA processing protein homolog | **1,046** | 0,319326 | 28,360 | 34,670 |
| VRK3 | vaccinia related kinase 3 | **1,046** | 0,111347 | 23,541 | 25,987 |
| ADAM23 | ADAM metallopeptidase domain 23 | **1,046** | 0,805616 | 0,609 | 0,511 |

| | | | | | |
|---|---|---|---|---|---|
| MST1 | macrophage stimulating 1 | **1,046** | 0,448225 | 3,490 | 4,150 |
| CCL8 | C-C motif chemokine ligand 8 | **1,046** | 0,787977 | 0,388 | 0,766 |
| TRAPPC6B | trafficking protein particle complex 6B | **1,046** | 0,183687 | 30,742 | 32,563 |
| GTF3C3 | general transcription factor IIIC subunit 3 | **1,046** | 0,215612 | 30,188 | 31,861 |
| NUDT16L1 | nudix hydrolase 16 like 1 | **1,046** | 0,219777 | 16,285 | 19,474 |
| IGSF3 | immunoglobulin superfamily member 3 | **1,046** | 0,18663 | 33,511 | 35,117 |
| EPB41L2 | erythrocyte membrane protein band 4.1 like 2 | **1,046** | 0,237046 | 43,703 | 45,908 |
| ZNF583 | zinc finger protein 583 | **1,046** | 0,437372 | 5,705 | 4,789 |
| LSM14B | LSM family member 14B | **1,046** | 0,123614 | 29,523 | 30,776 |
| C18orf8 | chromosome 18 open reading frame 8 | **1,046** | 0,139603 | 17,226 | 19,347 |
| PPP1R32 | protein phosphatase 1 regulatory subunit 32 | **1,046** | 0,712313 | 0,775 | 0,575 |
| ATG10 | autophagy related 10 | **1,046** | 0,434583 | 3,877 | 4,789 |
| TDRD9 | tudor domain containing 9 | **1,046** | 0,558234 | 10,912 | 1,532 |
| PCDHB9 | protocadherin beta 9 | **1,046** | 0,660391 | 1,329 | 0,830 |
| NOMO2 | NODAL modulator 2 | **1,046** | 0,308753 | 35,117 | 24,646 |
| WDR33 | WD repeat domain 33 | **1,046** | 0,101794 | 53,507 | 56,188 |
| KPNA4 | karyopherin subunit alpha 4 | **1,046** | 0,108535 | 62,425 | 63,084 |
| CFDP1 | craniofacial development protein 1 | **1,046** | 0,251481 | 12,795 | 12,323 |
| LINC00476 | long intergenic non-protein coding RNA 476 | **1,046** | 0,454139 | 4,985 | 4,853 |
| EFCAB14 | EF-hand calcium binding domain 14 | **1,046** | 0,084684 | 96,767 | 108,289 |
| SHPK | sedoheptulokinase | **1,046** | 0,580359 | 1,939 | 1,469 |
| RAP2C | RAP2C, member of RAS oncogene family | **1,046** | 0,125766 | 32,126 | 22,858 |
| CASC4 | cancer susceptibility 4 | **1,046** | 0,134055 | 126,788 | 97,626 |
| TOMM5 | translocase of outer mitochondrial membrane 5 | **1,046** | 0,752993 | 0,554 | 0,638 |
| TTC8 | tetratricopeptide repeat domain 8 | **1,046** | 0,171849 | 16,617 | 13,919 |
| CEP162 | centrosomal protein 162 | **1,046** | 0,421068 | 4,653 | 6,513 |
| IL27RA | interleukin 27 receptor subunit alpha | **1,046** | 0,536024 | 5,041 | 4,214 |
| DAZAP2 | DAZ associated protein 2 | **1,046** | 0,104181 | 152,877 | 158,412 |
| ABHD17B | abhydrolase domain containing 17B | **1,046** | 0,275401 | 8,309 | 9,641 |
| PAIP1 | poly(A) binding protein interacting protein 1 | **1,046** | 0,186524 | 25,313 | 23,178 |
| CFAP46 | cilia and flagella associated protein 46 | **1,046** | 0,787453 | 0,554 | 0,447 |
| ARSK | arylsulfatase family member K | **1,046** | 0,410844 | 6,924 | 7,981 |
| PFN4 | profilin family member 4 | **1,045** | 0,784607 | 0,609 | 0,830 |

| | | | | | |
|---|---|---|---|---|---|
| PPP3CA | protein phosphatase 3 catalytic subunit alpha | **1,045** | 0,108908 | 222,613 | 199,467 |
| CCNB1IP1 | cyclin B1 interacting protein 1 | **1,045** | 0,379702 | 7,256 | 7,023 |
| C10orf90 | chromosome 10 open reading frame 90 | **1,045** | 0,85666 | 0,388 | 0,383 |
| KLF10 | Kruppel like factor 10 | **1,045** | 0,106353 | 55,446 | 75,215 |
| SLC29A4 | solute carrier family 29 member 4 | **1,045** | 0,737053 | 0,775 | 1,213 |
| MAP3K7 | mitogen-activated protein kinase kinase kinase 7 | **1,045** | 0,123718 | 35,284 | 36,394 |
| ZNF487 | zinc finger protein 487 | **1,045** | 0,59737 | 1,772 | 2,809 |
| CHUK | conserved helix-loop-helix ubiquitous kinase | **1,045** | 0,215564 | 18,113 | 17,431 |
| MOCS3 | molybdenum cofactor synthesis 3 | **1,045** | 0,201201 | 11,466 | 13,855 |
| ACACB | acetyl-CoA carboxylase beta | **1,045** | 0,402448 | 7,921 | 9,322 |
| SBF2-AS1 | SBF2 antisense RNA 1 | **1,045** | 0,491119 | 3,323 | 3,256 |
| CAMSAP1 | calmodulin regulated spectrin associated protein 1 | **1,045** | 0,255779 | 23,319 | 25,540 |
| STIM2 | stromal interaction molecule 2 | **1,045** | 0,224048 | 12,629 | 19,857 |
| PPFIBP2 | PPFIA binding protein 2 | **1,045** | 0,255047 | 32,348 | 29,690 |
| CRK | CRK proto-oncogene, adaptor protein | **1,045** | 0,301307 | 9,472 | 8,875 |
| MIS18A | MIS18 kinetochore protein A | **1,045** | 0,391833 | 6,425 | 6,768 |
| TGFBR3L | transforming growth factor beta receptor 3 like | **1,045** | 0,856168 | 0,111 | 0,383 |
| ZNF580 | zinc finger protein 580 | **1,045** | 0,319037 | 9,693 | 12,323 |
| IGHMBP2 | immunoglobulin mu binding protein 2 | **1,045** | 0,165409 | 21,935 | 23,114 |
| WSB2 | WD repeat and SOCS box containing 2 | **1,045** | 0,08186 | 113,771 | 92,710 |
| UBE2G1 | ubiquitin conjugating enzyme E2 G1 | **1,045** | 0,138294 | 27,750 | 24,838 |
| ITPRIPL2 | inositol 1,4,5-trisphosphate receptor interacting protein like 2 | **1,045** | 0,170765 | 194,918 | 158,028 |
| NRAV | negative regulator of antiviral response (non-protein coding) | **1,045** | 0,3645 | 6,813 | 8,109 |
| RANBP2 | RAN binding protein 2 | **1,045** | 0,132938 | 94,108 | 105,416 |
| RDX | radixin | **1,045** | 0,165003 | 46,251 | 45,908 |
| UBN1 | ubinuclein 1 | **1,045** | 0,122888 | 54,615 | 68,000 |
| METTL3 | methyltransferase like 3 | **1,045** | 0,272148 | 13,404 | 12,642 |
| RALBP1 | ralA binding protein 1 | **1,045** | 0,123348 | 74,721 | 64,808 |
| HOMEZ | homeobox and leucine zipper encoding | **1,045** | 0,320045 | 8,918 | 8,620 |
| DHX15 | DEAH-box helicase 15 | **1,045** | 0,127967 | 94,883 | 104,522 |
| HEBP1 | heme binding protein 1 | **1,045** | 0,222455 | 31,905 | 28,349 |
| PPP1CB | protein phosphatase 1 catalytic subunit beta | **1,045** | 0,140904 | 176,861 | 166,520 |
| MTFMT | mitochondrial methionyl-tRNA formyltransferase | **1,045** | 0,327732 | 10,358 | 10,152 |

| | | | | | |
|---|---|---|---|---|---|
| CRTAP | cartilage associated protein | **1,045** | 0,076906 | 216,299 | 154,772 |
| CDC23 | cell division cycle 23 | **1,045** | 0,238962 | 17,060 | 17,942 |
| TMEM231 | transmembrane protein 231 | **1,045** | 0,410133 | 6,148 | 6,257 |
| GRINA | glutamate ionotropic receptor NMDA type subunit associated protein 1 | **1,045** | 0,112286 | 138,697 | 125,273 |
| DZIP3 | DAZ interacting zinc finger protein 3 | **1,045** | 0,28645 | 10,635 | 13,153 |
| ZBTB6 | zinc finger and BTB domain containing 6 | **1,045** | 0,207717 | 18,888 | 20,815 |
| SMCHD1 | structural maintenance of chromosomes flexible hinge domain containing 1 | **1,045** | 0,188326 | 36,336 | 40,225 |
| S100B | S100 calcium binding protein B | **1,044** | 0,350492 | 16,949 | 12,004 |
| MPPE1 | metallophosphoesterase 1 | **1,044** | 0,345252 | 9,527 | 11,557 |
| C17orf51 | chromosome 17 open reading frame 51 | **1,044** | 0,309568 | 8,198 | 7,981 |
| ABLIM3 | actin binding LIM protein family member 3 | **1,044** | 0,428286 | 7,367 | 3,320 |
| MTCL1 | microtubule crosslinking factor 1 | **1,044** | 0,379456 | 10,247 | 14,494 |
| MED28 | mediator complex subunit 28 | **1,044** | 0,169602 | 27,030 | 27,966 |
| STAU1 | staufen double-stranded RNA binding protein 1 | **1,044** | 0,080492 | 83,584 | 77,067 |
| ZSCAN21 | zinc finger and SCAN domain containing 21 | **1,044** | 0,292909 | 9,084 | 9,769 |
| SELENOP | selenoprotein P | **1,044** | 0,193054 | 8,198 | 10,982 |
| ZNF507 | zinc finger protein 507 | **1,044** | 0,296411 | 24,482 | 25,859 |
| GOPC | golgi associated PDZ and coiled-coil motif containing | **1,044** | 0,191995 | 40,656 | 40,034 |
| PCM1 | pericentriolar material 1 | **1,044** | 0,108789 | 84,525 | 99,925 |
| PTPRD-AS1 | PTPRD antisense RNA 1 | **1,044** | 0,769375 | 0,609 | 0,447 |
| ACAT1 | acetyl-CoA acetyltransferase 1 | **1,044** | 0,289411 | 15,731 | 15,643 |
| WNK2 | WNK lysine deficient protein kinase 2 | **1,044** | 0,594838 | 2,105 | 4,150 |
| ZDHHC20 | zinc finger DHHC-type containing 20 | **1,044** | 0,172082 | 52,399 | 53,953 |
| MAN2B1 | mannosidase alpha class 2B member 1 | **1,044** | 0,180497 | 40,823 | 31,989 |
| UBE2FP1 | ubiquitin conjugating enzyme E2 F (putative) pseudogene 1 | **1,044** | 0,789441 | 0,665 | 1,085 |
| TMSB4X | thymosin beta 4, X-linked | **1,044** | 0,258024 | 72,118 | 40,417 |
| C2CD2 | C2 calcium dependent domain containing 2 | **1,044** | 0,157297 | 21,658 | 20,943 |
| RAD21 | RAD21 cohesin complex component | **1,044** | 0,090169 | 115,045 | 115,568 |
| PCBP2 | poly(rC) binding protein 2 | **1,044** | 0,130917 | 80,870 | 93,668 |
| DNAJC13 | DnaJ heat shock protein family (Hsp40) member C13 | **1,044** | 0,215612 | 48,799 | 49,867 |
| THAP6 | THAP domain containing 6 | **1,044** | 0,301325 | 19,664 | 20,560 |
| HECTD3 | HECT domain E3 ubiquitin protein ligase 3 | **1,044** | 0,181162 | 31,960 | 28,349 |
| TIMP2 | TIMP metallopeptidase inhibitor 2 | **1,044** | 0,10517 | 408,281 | 355,005 |

| | | | | | |
|---|---|---|---|---|---|
| ZNF417 | zinc finger protein 417 | **1,044** | 0,47838 | 4,210 | 4,661 |
| PSPC1 | paraspeckle component 1 | **1,044** | 0,172974 | 20,494 | 23,433 |
| C1GALT1C1L | C1GALT1-specific chaperone 1 like | **1,044** | 0,467627 | 4,320 | 3,959 |
| BIRC3 | baculoviral IAP repeat containing 3 | **1,044** | 0,081073 | 1013,418 | 1231,409 |
| ITFG1 | integrin alpha FG-GAP repeat containing 1 | **1,044** | 0,131825 | 89,012 | 59,253 |
| HOXD1 | homeobox D1 | **1,044** | 0,792329 | 0,166 | 0,958 |
| LINC01278 | long intergenic non-protein coding RNA 1278 | **1,044** | 0,252719 | 11,798 | 12,387 |
| NIPSNAP3A | nipsnap homolog 3A | **1,043** | 0,380049 | 8,696 | 5,108 |
| CMKLR1 | chemerin chemokine-like receptor 1 | **1,043** | 0,412333 | 14,568 | 11,876 |
| MTR | 5-methyltetrahydrofolate-homocysteine methyltransferase | **1,043** | 0,290449 | 30,631 | 36,905 |
| PIWIL2 | piwi like RNA-mediated gene silencing 2 | **1,043** | 0,54525 | 3,434 | 7,981 |
| PI4KAP1 | phosphatidylinositol 4-kinase alpha pseudogene 1 | **1,043** | 0,733107 | 0,388 | 0,766 |
| CSGALNACT2 | chondroitin sulfate N-acetylgalactosaminyltransferase 2 | **1,043** | 0,165502 | 109,451 | 77,131 |
| DKK3 | dickkopf WNT signaling pathway inhibitor 3 | **1,043** | 0,193066 | 20,661 | 20,687 |
| ORMDL1 | ORMDL sphingolipid biosynthesis regulator 1 | **1,043** | 0,16684 | 26,366 | 27,839 |
| CIDECP | cell death-inducing DFFA-like effector c pseudogene | **1,043** | 0,341175 | 8,087 | 9,705 |
| ZNF343 | zinc finger protein 343 | **1,043** | 0,425276 | 6,924 | 6,193 |
| PAFAH1B2 | platelet activating factor acetylhydrolase 1b catalytic subunit 2 | **1,043** | 0,089623 | 81,479 | 71,001 |
| UPF1 | UPF1, RNA helicase and ATPase | **1,043** | 0,090651 | 104,300 | 124,890 |
| MAPK12 | mitogen-activated protein kinase 12 | **1,043** | 0,635562 | 3,434 | 1,788 |
| POLDIP3 | DNA polymerase delta interacting protein 3 | **1,043** | 0,078423 | 120,584 | 150,430 |
| MID2 | midline 2 | **1,043** | 0,462597 | 10,192 | 8,811 |
| BAZ1A | bromodomain adjacent to zinc finger domain 1A | **1,043** | 0,150282 | 37,665 | 39,012 |
| SDHAP3 | succinate dehydrogenase complex flavoprotein subunit A pseudogene 3 | **1,043** | 0,690485 | 2,770 | 0,638 |
| ODF3B | outer dense fiber of sperm tails 3B | **1,043** | 0,288263 | 11,023 | 13,600 |
| KALRN | kalirin, RhoGEF kinase | **1,043** | 0,253171 | 13,515 | 36,458 |
| RBM26 | RNA binding motif protein 26 | **1,043** | 0,12765 | 42,429 | 58,486 |
| TAB2 | TGF-beta activated kinase 1/MAP3K7 binding protein 2 | **1,043** | 0,21426 | 83,251 | 70,554 |
| USF3 | upstream transcription factor family member 3 | **1,043** | 0,537541 | 17,725 | 16,409 |
| PPP1R8 | protein phosphatase 1 regulatory subunit 8 | **1,043** | 0,101169 | 43,204 | 49,164 |
| GLYR1 | glyoxylate reductase 1 homolog | **1,043** | 0,129252 | 58,160 | 72,150 |
| DYNC1LI1 | dynein cytoplasmic 1 light intermediate chain 1 | **1,043** | 0,133942 | 41,820 | 39,012 |
| MIS12 | MIS12, kinetochore complex component | **1,043** | 0,172131 | 30,908 | 33,138 |

| | | | | | |
|---|---|---|---|---|---|
| ZNF711 | zinc finger protein 711 | **1,043** | 0,773502 | 1,329 | 0,894 |
| DNA2 | DNA replication helicase/nuclease 2 | **1,043** | 0,697372 | 1,994 | 1,724 |
| KDM1B | lysine demethylase 1B | **1,043** | 0,341034 | 15,565 | 15,707 |
| MMP3 | matrix metallopeptidase 3 | **1,043** | 0,171576 | 102644,786 | 108413,201 |
| CHST1 | carbohydrate sulfotransferase 1 | **1,043** | 0,274871 | 9,195 | 8,364 |
| SYTL3 | synaptotagmin like 3 | **1,043** | 0,714727 | 1,496 | 1,469 |
| C9orf72 | chromosome 9 open reading frame 72 | **1,043** | 0,47164 | 9,084 | 8,300 |
| MICALL1 | MICAL like 1 | **1,043** | 0,227249 | 56,553 | 52,804 |
| CAPG | capping actin protein, gelsolin like | **1,043** | 0,192176 | 70,789 | 55,422 |
| SNAPC5 | small nuclear RNA activating complex polypeptide 5 | **1,043** | 0,348781 | 10,912 | 11,174 |
| BRD8 | bromodomain containing 8 | **1,043** | 0,149255 | 45,365 | 51,144 |
| RPSAP52 | ribosomal protein SA pseudogene 52 | **1,043** | 0,761075 | 1,274 | 0,255 |
| MANEAL | mannosidase endo-alpha like | **1,043** | 0,633938 | 3,545 | 4,214 |
| CRADD | CASP2 and RIPK1 domain containing adaptor with death domain | **1,043** | 0,39301 | 7,699 | 7,279 |
| PTCH1 | patched 1 | **1,043** | 0,378382 | 10,967 | 10,471 |
| UBE2A | ubiquitin conjugating enzyme E2 A | **1,043** | 0,118793 | 61,594 | 62,828 |
| U2SURP | U2 snRNP associated SURP domain containing | **1,043** | 0,155593 | 51,291 | 66,915 |
| RFX1 | regulatory factor X1 | **1,043** | 0,214088 | 17,946 | 21,007 |
| ATP9B | ATPase phospholipid transporting 9B (putative) | **1,043** | 0,280228 | 14,014 | 14,111 |
| SLC25A37 | solute carrier family 25 member 37 | **1,043** | 0,138004 | 381,084 | 435,392 |
| ESD | esterase D | **1,043** | 0,199904 | 50,073 | 43,482 |
| ANKFY1 | ankyrin repeat and FYVE domain containing 1 | **1,042** | 0,24272 | 34,730 | 34,032 |
| CERS4 | ceramide synthase 4 | **1,042** | 0,414134 | 8,087 | 4,406 |
| APAF1 | apoptotic peptidase activating factor 1 | **1,042** | 0,274515 | 14,069 | 12,962 |
| CCT6B | chaperonin containing TCP1 subunit 6B | **1,042** | 0,657008 | 1,551 | 1,660 |
| KIAA1671 | KIAA1671 | **1,042** | 0,31922 | 13,958 | 11,940 |
| GTDC1 | glycosyltransferase like domain containing 1 | **1,042** | 0,150749 | 20,162 | 21,007 |
| LAMB2 | laminin subunit beta 2 | **1,042** | 0,175496 | 295,174 | 158,284 |
| ZNF528 | zinc finger protein 528 | **1,042** | 0,444747 | 7,478 | 7,407 |
| DYNLL2 | dynein light chain LC8-type 2 | **1,042** | 0,187432 | 96,600 | 90,795 |
| RNF141 | ring finger protein 141 | **1,042** | 0,238655 | 15,565 | 17,176 |
| SHF | Src homology 2 domain containing F | **1,042** | 0,716557 | 0,886 | 0,766 |
| TPRG1L | tumor protein p63 regulated 1 like | **1,042** | 0,144387 | 88,292 | 84,665 |

| | | | | | |
|---|---|---|---|---|---|
| ZNF140 | zinc finger protein 140 | **1,042** | 0,30908 | 8,641 | 9,641 |
| KIF21B | kinesin family member 21B | **1,042** | 0,591998 | 2,548 | 2,235 |
| ZNF700 | zinc finger protein 700 | **1,042** | 0,408626 | 6,647 | 4,980 |
| ZFAND3 | zinc finger AN1-type containing 3 | **1,042** | 0,110989 | 105,407 | 113,653 |
| SNX15 | sorting nexin 15 | **1,042** | 0,842317 | 0,277 | 0,447 |
| MPV17 | MPV17, mitochondrial inner membrane protein | **1,042** | 0,21261 | 18,390 | 18,325 |
| DMXL2 | Dmx like 2 | **1,042** | 0,468034 | 71,841 | 78,727 |
| ZNF287 | zinc finger protein 287 | **1,042** | 0,50137 | 4,487 | 5,108 |
| MCM7 | minichromosome maintenance complex component 7 | **1,042** | 0,17872 | 43,869 | 44,886 |
| TUFT1 | tuftelin 1 | **1,042** | 0,431787 | 6,702 | 8,811 |
| H2AFV | H2A histone family member V | **1,042** | 0,092476 | 90,175 | 89,326 |
| MAPK14 | mitogen-activated protein kinase 14 | **1,042** | 0,161909 | 33,068 | 34,479 |
| DLST | dihydrolipoamide S-succinyltransferase | **1,042** | 0,109028 | 83,916 | 80,962 |
| ARFGAP3 | ADP ribosylation factor GTPase activating protein 3 | **1,042** | 0,101195 | 124,240 | 132,808 |
| SRRM1 | serine and arginine repetitive matrix 1 | **1,042** | 0,181026 | 34,730 | 41,630 |
| NCOA5 | nuclear receptor coactivator 5 | **1,042** | 0,240335 | 32,514 | 38,693 |
| TIAF1 | TGFB1-induced anti-apoptotic factor 1 | **1,042** | 0,763613 | 1,772 | 1,724 |
| CLEC16A | C-type lectin domain containing 16A | **1,042** | 0,277091 | 13,903 | 17,367 |
| FAHD2B | fumarylacetoacetate hydrolase domain containing 2B | **1,042** | 0,541676 | 3,046 | 3,320 |
| OMD | osteomodulin | **1,042** | 0,330144 | 5,373 | 2,682 |
| IQCK | IQ motif containing K | **1,042** | 0,277949 | 9,306 | 9,003 |
| FAAH | fatty acid amide hydrolase | **1,042** | 0,547715 | 2,603 | 2,490 |
| LRGUK | leucine rich repeats and guanylate kinase domain containing | **1,042** | 0,796448 | 0,443 | 0,575 |
| MAT2A | methionine adenosyltransferase 2A | **1,042** | 0,186845 | 177,359 | 206,171 |
| ETV1 | ETS variant 1 | **1,042** | 0,501912 | 4,874 | 3,192 |
| FAP | fibroblast activation protein alpha | **1,042** | 0,219324 | 184,338 | 107,140 |
| ODC1 | ornithine decarboxylase 1 | **1,042** | 0,203252 | 336,828 | 384,121 |
| CASC2 | cancer susceptibility 2 (non-protein coding) | **1,041** | 0,582284 | 2,603 | 2,490 |
| XRCC6P1 | X-ray repair cross complementing 6 pseudogene 1 | **1,041** | 0,8084 | 0,388 | 1,341 |
| SPG11 | SPG11, spatacsin vesicle trafficking associated | **1,041** | 0,24649 | 44,091 | 48,015 |
| MTMR11 | myotubularin related protein 11 | **1,041** | 0,465008 | 6,259 | 5,300 |
| NUP188 | nucleoporin 188 | **1,041** | 0,183286 | 38,552 | 57,848 |
| RPL39L | ribosomal protein L39 like | **1,041** | 0,486493 | 3,157 | 3,831 |

| | | | | | |
|---|---|---|---|---|---|
| DUSP10 | dual specificity phosphatase 10 | **1,041** | 0,412741 | 16,506 | 9,897 |
| DDX28 | DEAD-box helicase 28 | **1,041** | 0,349403 | 7,312 | 10,727 |
| GALNT8 | polypeptide N-acetylgalactosaminyltransferase 8 | **1,041** | 0,447883 | 4,154 | 5,810 |
| KPNA1 | karyopherin subunit alpha 1 | **1,041** | 0,15409 | 51,790 | 56,954 |
| TNFRSF10B | TNF receptor superfamily member 10b | **1,041** | 0,127236 | 214,526 | 234,840 |
| TRDC | T-cell receptor delta constant | **1,041** | 0,827165 | 0,388 | 0,575 |
| SDR42E1 | short chain dehydrogenase/reductase family 42E, member 1 | **1,041** | 0,330828 | 15,786 | 14,685 |
| SERAC1 | serine active site containing 1 | **1,041** | 0,400278 | 9,749 | 8,811 |
| HNMT | histamine N-methyltransferase | **1,041** | 0,238845 | 47,857 | 60,083 |
| NKIRAS1 | NFKB inhibitor interacting Ras like 1 | **1,041** | 0,176079 | 32,292 | 42,205 |
| TRAPPC6A | trafficking protein particle complex 6A | **1,041** | 0,528953 | 2,936 | 2,107 |
| NDRG2 | NDRG family member 2 | **1,041** | 0,532442 | 3,988 | 4,469 |
| RBAK | RB associated KRAB zinc finger | **1,041** | 0,437072 | 13,848 | 14,302 |
| SETBP1 | SET binding protein 1 | **1,041** | 0,542468 | 15,122 | 17,303 |
| PPME1 | protein phosphatase methylesterase 1 | **1,041** | 0,144985 | 39,382 | 44,440 |
| CLU | clusterin | **1,041** | 0,202156 | 6965,424 | 9888,239 |
| SYT11 | synaptotagmin 11 | **1,041** | 0,243817 | 31,517 | 23,433 |
| RRAGB | Ras related GTP binding B | **1,041** | 0,469029 | 4,210 | 4,980 |
| MAPK8 | mitogen-activated protein kinase 8 | **1,041** | 0,249073 | 34,176 | 35,564 |
| DCAF17 | DDB1 and CUL4 associated factor 17 | **1,041** | 0,366163 | 13,737 | 13,408 |
| PCDHGA11 | protocadherin gamma subfamily A, 11 | **1,041** | 0,488123 | 7,256 | 5,044 |
| SPTLC1 | serine palmitoyltransferase long chain base subunit 1 | **1,041** | 0,113623 | 72,118 | 68,319 |
| PHKG1 | phosphorylase kinase catalytic subunit gamma 1 | **1,041** | 0,684217 | 0,831 | 1,660 |
| S100A3 | S100 calcium binding protein A3 | **1,041** | 0,450752 | 6,647 | 9,067 |
| KLHL36 | kelch like family member 36 | **1,041** | 0,2255 | 25,036 | 26,242 |
| C8orf76 | chromosome 8 open reading frame 76 | **1,041** | 0,623896 | 1,883 | 1,660 |
| TMEM116 | transmembrane protein 116 | **1,041** | 0,403789 | 5,317 | 5,683 |
| RB1 | RB transcriptional corepressor 1 | **1,041** | 0,215445 | 38,385 | 35,820 |
| EDRF1 | erythroid differentiation regulatory factor 1 | **1,041** | 0,365778 | 10,580 | 16,729 |
| IPO7 | importin 7 | **1,041** | 0,202425 | 94,939 | 90,922 |
| HELZ2 | helicase with zinc finger 2 | **1,041** | 0,355918 | 19,220 | 25,476 |
| BMPR1B | bone morphogenetic protein receptor type 1B | **1,041** | 0,530113 | 11,521 | 5,236 |
| MYO5C | myosin VC | **1,041** | 0,604474 | 2,493 | 2,362 |

| | | | | | |
|---|---|---|---|---|---|
| CFAP161 | cilia and flagella associated protein 161 | **1,041** | 0,786369 | 0,609 | 0,958 |
| ADAR | adenosine deaminase, RNA specific | **1,041** | 0,193555 | 194,475 | 213,450 |
| PAM | peptidylglycine alpha-amidating monooxygenase | **1,040** | 0,129821 | 303,040 | 199,850 |
| PIK3CA | phosphatidylinositol-4,5-bisphosphate 3-kinase catalytic subunit alpha | **1,040** | 0,274562 | 32,071 | 31,350 |
| ANO10 | anoctamin 10 | **1,040** | 0,202661 | 60,375 | 44,248 |
| KRCC1 | lysine rich coiled-coil 1 | **1,040** | 0,226707 | 31,683 | 26,625 |
| SYT17 | synaptotagmin 17 | **1,040** | 0,608097 | 2,493 | 1,341 |
| TSPAN32 | tetraspanin 32 | **1,040** | 0,869369 | 0,499 | 0,511 |
| SNAP29 | synaptosome associated protein 29 | **1,040** | 0,195954 | 46,362 | 44,184 |
| RPA1 | replication protein A1 | **1,040** | 0,119164 | 58,658 | 56,890 |
| ITGB5 | integrin subunit beta 5 | **1,040** | 0,11776 | 934,210 | 725,015 |
| ZNF624 | zinc finger protein 624 | **1,040** | 0,536393 | 4,099 | 3,384 |
| SPATS2L | spermatogenesis associated serine rich 2 like | **1,040** | 0,139471 | 54,116 | 46,738 |
| PPP2R1B | protein phosphatase 2 scaffold subunit Abeta | **1,040** | 0,163133 | 39,493 | 47,313 |
| SRRM2 | serine/arginine repetitive matrix 2 | **1,040** | 0,328894 | 322,038 | 392,741 |
| ZNF777 | zinc finger protein 777 | **1,040** | 0,216628 | 15,620 | 16,920 |
| TMTC3 | transmembrane and tetratricopeptide repeat containing 3 | **1,040** | 0,241186 | 33,068 | 29,818 |
| PLXDC2 | plexin domain containing 2 | **1,040** | 0,205595 | 165,118 | 151,069 |
| SENP5 | SUMO1/sentrin specific peptidase 5 | **1,040** | 0,249663 | 31,517 | 35,628 |
| NAA30 | N(alpha)-acetyltransferase 30, NatC catalytic subunit | **1,040** | 0,321219 | 20,384 | 17,048 |
| KCTD21 | potassium channel tetramerization domain containing 21 | **1,040** | 0,243571 | 13,072 | 11,940 |
| TMX3 | thioredoxin related transmembrane protein 3 | **1,040** | 0,19615 | 69,847 | 69,022 |
| LINC00654 | long intergenic non-protein coding RNA 654 | **1,040** | 0,728649 | 1,496 | 1,149 |
| RASA1 | RAS p21 protein activator 1 | **1,040** | 0,232472 | 60,763 | 58,103 |
| HSPA6 | heat shock protein family A (Hsp70) member 6 | **1,040** | 0,580317 | 0,942 | 7,151 |
| ZNF454 | zinc finger protein 454 | **1,040** | 0,726373 | 1,108 | 1,405 |
| CCDC127 | coiled-coil domain containing 127 | **1,040** | 0,205484 | 41,875 | 30,839 |
| MAP4K3 | mitogen-activated protein kinase kinase kinase kinase 3 | **1,040** | 0,357975 | 13,460 | 12,706 |
| P4HA2 | prolyl 4-hydroxylase subunit alpha 2 | **1,040** | 0,182585 | 120,252 | 67,362 |
| MFSD5 | major facilitator superfamily domain containing 5 | **1,040** | 0,26242 | 24,815 | 20,560 |
| WDR48 | WD repeat domain 48 | **1,040** | 0,17917 | 35,339 | 35,309 |
| FBXO8 | F-box protein 8 | **1,040** | 0,328688 | 12,463 | 12,642 |
| ISPD | isoprenoid synthase domain containing | **1,040** | 0,807237 | 0,665 | 0,766 |

| | | | | | |
|---|---|---|---|---|---|
| DPYSL3 | dihydropyrimidinase like 3 | **1,040** | 0,238213 | 28,526 | 33,138 |
| GRIA3 | glutamate ionotropic receptor AMPA type subunit 3 | **1,040** | 0,484022 | 8,641 | 4,406 |
| COMTD1 | catechol-O-methyltransferase domain containing 1 | **1,040** | 0,785956 | 0,554 | 0,575 |
| COBLL1 | cordon-bleu WH2 repeat protein like 1 | **1,040** | 0,282393 | 51,734 | 41,375 |
| LPIN2 | lipin 2 | **1,040** | 0,219514 | 126,123 | 175,332 |
| KTI12 | KTI12 chromatin associated homolog | **1,040** | 0,361059 | 7,921 | 9,577 |
| CLP1 | cleavage and polyadenylation factor I subunit 1 | **1,040** | 0,314585 | 11,078 | 12,195 |
| MOB4 | MOB family member 4, phocein | **1,040** | 0,348172 | 12,684 | 13,025 |
| MTERF2 | mitochondrial transcription termination factor 2 | **1,040** | 0,57173 | 2,049 | 4,023 |
| CFAP57 | cilia and flagella associated protein 57 | **1,040** | 0,834136 | 0,554 | 0,319 |
| HLF | HLF, PAR bZIP transcription factor | **1,040** | 0,545218 | 6,148 | 5,683 |
| ZDHHC2 | zinc finger DHHC-type containing 2 | **1,040** | 0,190015 | 65,416 | 47,440 |
| EFNB1 | ephrin B1 | **1,040** | 0,32494 | 29,301 | 29,754 |
| RPS27 | ribosomal protein S27 | **1,039** | 0,283721 | 183,175 | 186,186 |
| C4orf46 | chromosome 4 open reading frame 46 | **1,039** | 0,486882 | 4,043 | 3,448 |
| ZNF226 | zinc finger protein 226 | **1,039** | 0,335209 | 8,032 | 9,833 |
| NOC2LP1 | NOC2 like nucleolar associated transcriptional repressor pseudogene 1 | **1,039** | 0,734304 | 2,659 | 1,788 |
| MC1R | melanocortin 1 receptor | **1,039** | 0,710801 | 1,329 | 0,830 |
| CMPK2 | cytidine/uridine monophosphate kinase 2 | **1,039** | 0,785208 | 1,496 | 1,213 |
| FBXL20 | F-box and leucine rich repeat protein 20 | **1,039** | 0,333237 | 21,713 | 21,709 |
| MTPN | myotrophin | **1,039** | 0,248822 | 143,571 | 126,487 |
| FZD1 | frizzled class receptor 1 | **1,039** | 0,130324 | 52,067 | 77,003 |
| SELENOT | selenoprotein T | **1,039** | 0,209155 | 66,080 | 74,194 |
| TBC1D2 | TBC1 domain family member 2 | **1,039** | 0,345501 | 10,303 | 10,854 |
| NAPA | NSF attachment protein alpha | **1,039** | 0,160155 | 33,622 | 36,714 |
| PDSS1 | decaprenyl diphosphate synthase subunit 1 | **1,039** | 0,595339 | 2,603 | 4,086 |
| SAA2 | serum amyloid A2 | **1,039** | 0,459141 | 22,710 | 10,471 |
| ZSCAN32 | zinc finger and SCAN domain containing 32 | **1,039** | 0,309558 | 10,856 | 11,046 |
| CSTF2T | cleavage stimulation factor subunit 2 tau variant | **1,039** | 0,137676 | 33,289 | 38,885 |
| NOM1 | nucleolar protein with MIF4G domain 1 | **1,039** | 0,346741 | 11,632 | 16,984 |
| BLOC1S2 | biogenesis of lysosomal organelles complex 1 subunit 2 | **1,039** | 0,202487 | 28,027 | 28,732 |
| CLIP4 | CAP-Gly domain containing linker protein family member 4 | **1,039** | 0,370925 | 9,915 | 10,280 |
| PRDM10 | PR/SET domain 10 | **1,039** | 0,40794 | 9,804 | 14,558 |

| | | | | | |
|---|---|---|---|---|---|
| PDCD6IP | programmed cell death 6 interacting protein | **1,039** | 0,170969 | 107,734 | 102,671 |
| ZDHHC23 | zinc finger DHHC-type containing 23 | **1,039** | 0,492098 | 3,988 | 4,150 |
| RNMT | RNA guanine-7 methyltransferase | **1,039** | 0,155812 | 35,560 | 41,630 |
| HNRNPH1 | heterogeneous nuclear ribonucleoprotein H1 | **1,039** | 0,498451 | 7,755 | 9,897 |
| MRPL42 | mitochondrial ribosomal protein L42 | **1,039** | 0,228168 | 33,234 | 36,203 |
| EIF3A | eukaryotic translation initiation factor 3 subunit A | **1,039** | 0,173011 | 228,817 | 245,247 |
| KRBA1 | KRAB-A domain containing 1 | **1,039** | 0,33718 | 8,364 | 9,897 |
| UBE2D3 | ubiquitin conjugating enzyme E2 D3 | **1,039** | 0,142095 | 270,027 | 280,620 |
| LIPT1 | lipoyltransferase 1 | **1,039** | 0,547399 | 3,102 | 2,937 |
| SEC1P | secretory blood group 1, pseudogene | **1,038** | 0,805482 | 0,443 | 0,319 |
| RFK | riboflavin kinase | **1,038** | 0,276778 | 21,602 | 27,583 |
| SCIN | scinderin | **1,038** | 0,171978 | 57,938 | 56,826 |
| DNAJB2 | DnaJ heat shock protein family (Hsp40) member B2 | **1,038** | 0,216244 | 87,627 | 99,414 |
| ZNF816 | zinc finger protein 816 | **1,038** | 0,559554 | 2,659 | 3,767 |
| ZNF542P | zinc finger protein 542, pseudogene | **1,038** | 0,418558 | 7,533 | 8,109 |
| CTBS | chitobiase | **1,038** | 0,21245 | 47,525 | 44,184 |
| PMS2 | PMS1 homolog 2, mismatch repair system component | **1,038** | 0,537828 | 5,317 | 5,619 |
| SDC1 | syndecan 1 | **1,038** | 0,508482 | 16,506 | 8,747 |
| RNF44 | ring finger protein 44 | **1,038** | 0,311293 | 22,433 | 25,604 |
| GAPDHP65 | glyceraldehyde 3 phosphate dehydrogenase pseudogene 65 | **1,038** | 0,719899 | 1,052 | 0,830 |
| SEPHS2 | selenophosphate synthetase 2 | **1,038** | 0,22907 | 51,457 | 48,398 |
| SIAH1 | siah E3 ubiquitin protein ligase 1 | **1,038** | 0,321566 | 16,617 | 13,919 |
| LLPH | LLP homolog, long-term synaptic facilitation | **1,038** | 0,327374 | 15,509 | 18,325 |
| RETREG3 | reticulophagy regulator family member 3 | **1,038** | 0,186716 | 47,525 | 44,376 |
| CDKN3 | cyclin dependent kinase inhibitor 3 | **1,038** | 0,540538 | 4,764 | 3,703 |
| KLHL7 | kelch like family member 7 | **1,038** | 0,169805 | 26,587 | 25,157 |
| NTN1 | netrin 1 | **1,038** | 0,192226 | 187,330 | 177,375 |
| ABCB10 | ATP binding cassette subfamily B member 10 | **1,038** | 0,506826 | 6,425 | 5,683 |
| CAPN2 | calpain 2 | **1,038** | 0,121798 | 141,799 | 160,774 |
| TMOD3 | tropomodulin 3 | **1,038** | 0,202579 | 125,957 | 110,588 |
| SLC4A7 | solute carrier family 4 member 7 | **1,038** | 0,347751 | 48,300 | 55,166 |
| EVI5L | ecotropic viral integration site 5 like | **1,038** | 0,458806 | 10,635 | 10,663 |
| INPP4A | inositol polyphosphate-4-phosphatase type I A | **1,038** | 0,337631 | 21,325 | 21,390 |

| | | | | | |
|---|---|---|---|---|---|
| PIGG | phosphatidylinositol glycan anchor biosynthesis class G | **1,038** | 0,203058 | 48,189 | 43,226 |
| SIPA1L2 | signal induced proliferation associated 1 like 2 | **1,038** | 0,356851 | 70,567 | 78,025 |
| RASSF8 | Ras association domain family member 8 | **1,038** | 0,310095 | 141,799 | 119,272 |
| EXOC5 | exocyst complex component 5 | **1,038** | 0,268919 | 30,575 | 27,264 |
| RNF4 | ring finger protein 4 | **1,038** | 0,142866 | 36,779 | 39,268 |
| ZNF169 | zinc finger protein 169 | **1,038** | 0,738863 | 1,329 | 1,724 |
| POSTN | periostin | **1,038** | 0,453562 | 71,619 | 6,449 |
| MTFR1 | mitochondrial fission regulator 1 | **1,038** | 0,249976 | 21,325 | 20,496 |
| TIGD6 | tigger transposable element derived 6 | **1,038** | 0,380806 | 8,198 | 6,704 |
| PRUNE2 | prune homolog 2 | **1,038** | 0,373538 | 93,942 | 48,143 |
| MTMR12 | myotubularin related protein 12 | **1,038** | 0,193095 | 28,249 | 30,265 |
| ARHGAP19 | Rho GTPase activating protein 19 | **1,038** | 0,643138 | 2,271 | 1,788 |
| FBXL17 | F-box and leucine rich repeat protein 17 | **1,038** | 0,353552 | 19,608 | 17,750 |
| DYNC1LI2 | dynein cytoplasmic 1 light intermediate chain 2 | **1,038** | 0,285885 | 70,235 | 76,811 |
| DBF4B | DBF4 zinc finger B | **1,038** | 0,599005 | 2,770 | 2,873 |
| CENPC | centromere protein C | **1,038** | 0,365353 | 10,303 | 11,812 |
| FBXO3 | F-box protein 3 | **1,038** | 0,248192 | 22,267 | 22,539 |
| MIEN1 | migration and invasion enhancer 1 | **1,038** | 0,255296 | 29,191 | 25,795 |
| STX16 | syntaxin 16 | **1,038** | 0,229375 | 20,661 | 25,540 |
| CDK8 | cyclin dependent kinase 8 | **1,038** | 0,380825 | 8,309 | 7,790 |
| RHOG | ras homolog family member G | **1,038** | 0,208574 | 41,930 | 46,802 |
| MAD2L1BP | MAD2L1 binding protein | **1,038** | 0,286305 | 19,774 | 20,049 |
| DHX36 | DEAH-box helicase 36 | **1,038** | 0,261594 | 29,523 | 27,711 |
| PPFIA1 | PTPRF interacting protein alpha 1 | **1,037** | 0,242816 | 80,094 | 83,133 |
| SACM1L | SAC1 suppressor of actin mutations 1 like (yeast) | **1,037** | 0,209143 | 53,728 | 48,271 |
| SNHG6 | small nucleolar RNA host gene 6 | **1,037** | 0,27511 | 35,560 | 40,353 |
| SBDS | SBDS, ribosome assembly guanine nucleotide exchange factor | **1,037** | 0,182241 | 81,479 | 84,218 |
| ATRX | ATRX, chromatin remodeler | **1,037** | 0,229733 | 60,929 | 59,700 |
| ETV3 | ETS variant 3 | **1,037** | 0,349202 | 24,372 | 23,688 |
| CHMP4B | charged multivesicular body protein 4B | **1,037** | 0,228564 | 158,139 | 173,927 |
| NUB1 | negative regulator of ubiquitin like proteins 1 | **1,037** | 0,178092 | 52,842 | 58,870 |
| JRKL | JRK-like | **1,037** | 0,281423 | 23,929 | 25,540 |
| RNF216P1 | ring finger protein 216 pseudogene 1 | **1,037** | 0,317291 | 11,078 | 9,961 |

| | | | | | |
|---|---|---|---|---|---|
| AOC3 | amine oxidase, copper containing 3 | **1,037** | 0,399615 | 18,833 | 13,792 |
| C2CD2L | C2CD2 like | **1,037** | 0,41439 | 9,361 | 7,981 |
| MAP3K21 | mitogen-activated protein kinase kinase kinase 21 | **1,037** | 0,684959 | 1,883 | 2,107 |
| CWF19L2 | CWF19 like 2, cell cycle control (S. pombe) | **1,037** | 0,340992 | 17,946 | 22,092 |
| AREL1 | apoptosis resistant E3 ubiquitin protein ligase 1 | **1,037** | 0,210164 | 36,281 | 33,585 |
| CDHR2 | cadherin related family member 2 | **1,037** | 0,791496 | 0,831 | 0,383 |
| DCLRE1C | DNA cross-link repair 1C | **1,037** | 0,485295 | 7,976 | 7,279 |
| UBE2Z | ubiquitin conjugating enzyme E2 Z | **1,037** | 0,133253 | 165,949 | 165,499 |
| HSD11B1 | hydroxysteroid 11-beta dehydrogenase 1 | **1,037** | 0,199843 | 429,218 | 629,879 |
| GMEB2 | glucocorticoid modulatory element binding protein 2 | **1,037** | 0,247498 | 21,159 | 25,285 |
| WWC3 | WWC family member 3 | **1,037** | 0,360937 | 37,499 | 34,351 |
| CEP164 | centrosomal protein 164 | **1,037** | 0,239501 | 16,174 | 21,007 |
| WNT2B | Wnt family member 2B | **1,037** | 0,843898 | 0,499 | 0,575 |
| DDX11L2 | DEAD/H-box helicase 11 like 2 | **1,037** | 0,81586 | 0,332 | 0,511 |
| GRK4 | G protein-coupled receptor kinase 4 | **1,037** | 0,621055 | 2,714 | 2,937 |
| HELZ | helicase with zinc finger | **1,037** | 0,459664 | 24,206 | 23,561 |
| LGMN | legumain | **1,037** | 0,238128 | 193,256 | 192,507 |
| SIDT2 | SID1 transmembrane family member 2 | **1,037** | 0,209952 | 27,197 | 29,818 |
| PPIP5K2 | diphosphoinositol pentakisphosphate kinase 2 | **1,037** | 0,372726 | 30,908 | 27,902 |
| DOCK8 | dedicator of cytokinesis 8 | **1,037** | 0,487363 | 12,740 | 35,501 |
| CRLF1 | cytokine receptor like factor 1 | **1,037** | 0,329443 | 59,766 | 25,732 |
| AP1G2 | adaptor related protein complex 1 gamma 2 subunit | **1,037** | 0,450886 | 9,804 | 7,534 |
| E2F7 | E2F transcription factor 7 | **1,037** | 0,845738 | 0,942 | 0,447 |
| NSUN3 | NOP2/Sun RNA methyltransferase family member 3 | **1,037** | 0,458624 | 10,912 | 10,918 |
| GAB2 | GRB2 associated binding protein 2 | **1,037** | 0,263876 | 25,867 | 18,325 |
| PRKACB | protein kinase cAMP-activated catalytic subunit beta | **1,037** | 0,299323 | 22,488 | 18,453 |
| CEP128 | centrosomal protein 128 | **1,037** | 0,762986 | 1,440 | 1,085 |
| TRAF6 | TNF receptor associated factor 6 | **1,037** | 0,287471 | 13,294 | 14,622 |
| GGPS1 | geranylgeranyl diphosphate synthase 1 | **1,037** | 0,283556 | 12,297 | 14,558 |
| STAT1 | signal transducer and activator of transcription 1 | **1,037** | 0,216292 | 72,561 | 66,148 |
| GPATCH11 | G-patch domain containing 11 | **1,037** | 0,399405 | 11,909 | 16,090 |
| PYROXD1 | pyridine nucleotide-disulphide oxidoreductase domain 1 | **1,037** | 0,313627 | 36,391 | 28,413 |
| PRKAR2B | protein kinase cAMP-dependent type II regulatory subunit beta | **1,037** | 0,404689 | 25,313 | 13,025 |

| | | | | | |
|---|---|---|---|---|---|
| GOLPH3L | golgi phosphoprotein 3 like | **1,037** | 0,234834 | 33,954 | 31,159 |
| LINC01547 | long intergenic non-protein coding RNA 1547 | **1,037** | 0,707354 | 1,440 | 2,873 |
| HAUS8 | HAUS augmin like complex subunit 8 | **1,037** | 0,48022 | 5,151 | 5,300 |
| USP34 | ubiquitin specific peptidase 34 | **1,037** | 0,517416 | 80,980 | 82,813 |
| RALGAPB | Ral GTPase activating protein non-catalytic beta subunit | **1,037** | 0,275773 | 45,808 | 52,102 |
| EOGT | EGF domain specific O-linked N-acetylglucosamine transferase | **1,037** | 0,360277 | 17,393 | 16,537 |
| ZBTB20 | zinc finger and BTB domain containing 20 | **1,037** | 0,576937 | 10,524 | 18,708 |
| BCL2L2 | BCL2 like 2 | **1,037** | 0,184921 | 42,817 | 41,055 |
| ARSE | arylsulfatase E (chondrodysplasia punctata 1) | **1,037** | 0,820453 | 0,665 | 0,255 |
| DNAL4 | dynein axonemal light chain 4 | **1,037** | 0,322083 | 14,346 | 15,579 |
| DCTN5 | dynactin subunit 5 | **1,037** | 0,224511 | 46,915 | 53,187 |
| CCDC40 | coiled-coil domain containing 40 | **1,037** | 0,71276 | 1,108 | 1,277 |
| NGEF | neuronal guanine nucleotide exchange factor | **1,037** | 0,307376 | 52,233 | 33,840 |
| C2orf69 | chromosome 2 open reading frame 69 | **1,037** | 0,43051 | 13,903 | 12,323 |
| DEK | DEK proto-oncogene | **1,036** | 0,160319 | 105,574 | 112,312 |
| FBXW11 | F-box and WD repeat domain containing 11 | **1,036** | 0,214877 | 61,040 | 57,337 |
| S100A13 | S100 calcium binding protein A13 | **1,036** | 0,296467 | 23,042 | 25,859 |
| RECQL | RecQ like helicase | **1,036** | 0,209515 | 75,054 | 75,279 |
| CDKN2D | cyclin dependent kinase inhibitor 2D | **1,036** | 0,227086 | 20,716 | 16,409 |
| LAMA1 | laminin subunit alpha 1 | **1,036** | 0,567657 | 10,856 | 10,727 |
| SCAF8 | SR-related CTD associated factor 8 | **1,036** | 0,358155 | 42,927 | 44,823 |
| EMD | emerin | **1,036** | 0,218791 | 37,610 | 38,374 |
| DRAM1 | DNA damage regulated autophagy modulator 1 | **1,036** | 0,17401 | 130,499 | 155,028 |
| BTN2A1 | butyrophilin subfamily 2 member A1 | **1,036** | 0,23015 | 54,116 | 37,608 |
| CDK17 | cyclin dependent kinase 17 | **1,036** | 0,298661 | 27,086 | 29,499 |
| TIGD4 | tigger transposable element derived 4 | **1,036** | 0,803475 | 0,443 | 0,766 |
| MEX3D | mex-3 RNA binding family member D | **1,036** | 0,435413 | 9,970 | 6,960 |
| ARF6 | ADP ribosylation factor 6 | **1,036** | 0,171056 | 100,644 | 113,206 |
| 3.maalis | membrane associated ring-CH-type finger 3 | **1,036** | 0,238274 | 34,730 | 39,842 |
| ARIH1 | ariadne RBR E3 ubiquitin protein ligase 1 | **1,036** | 0,269403 | 65,693 | 59,253 |
| RDH14 | retinol dehydrogenase 14 (all-trans/9-cis/11-cis) | **1,036** | 0,438517 | 9,195 | 8,620 |
| ZNF250 | zinc finger protein 250 | **1,036** | 0,603574 | 4,320 | 5,044 |
| ZNF667-AS1 | ZNF667 antisense RNA 1 (head to head) | **1,036** | 0,434498 | 8,918 | 10,791 |

| | | | | | |
|---|---|---|---|---|---|
| MICAL2 | microtubule associated monooxygenase, calponin and LIM domain containing 2 | **1,036** | 0,315019 | 31,018 | 27,072 |
| IPPK | inositol-pentakisphosphate 2-kinase | **1,036** | 0,354055 | 11,410 | 10,535 |
| C9orf116 | chromosome 9 open reading frame 116 | **1,036** | 0,79591 | 0,665 | 0,766 |
| SLC12A2 | solute carrier family 12 member 2 | **1,036** | 0,318335 | 24,372 | 21,007 |
| SRI | sorcin | **1,036** | 0,296424 | 24,759 | 28,285 |
| UBAC2-AS1 | UBAC2 antisense RNA 1 | **1,036** | 0,718565 | 1,219 | 2,235 |
| ZNF212 | zinc finger protein 212 | **1,036** | 0,42241 | 12,740 | 12,770 |
| CDK20 | cyclin dependent kinase 20 | **1,036** | 0,565063 | 5,373 | 5,810 |
| BDH2 | 3-hydroxybutyrate dehydrogenase 2 | **1,036** | 0,453823 | 6,813 | 11,429 |
| OVCH1-AS1 | OVCH1 antisense RNA 1 | **1,036** | 0,819215 | 0,277 | 0,255 |
| TSPYL4 | TSPY like 4 | **1,036** | 0,212531 | 27,086 | 29,690 |
| MAP3K9 | mitogen-activated protein kinase kinase kinase 9 | **1,036** | 0,665564 | 4,431 | 1,979 |
| C1orf131 | chromosome 1 open reading frame 131 | **1,036** | 0,372491 | 7,422 | 7,790 |
| MID1IP1 | MID1 interacting protein 1 | **1,036** | 0,291926 | 50,959 | 42,141 |
| ZNF684 | zinc finger protein 684 | **1,036** | 0,709467 | 1,606 | 1,915 |
| NOL4L | nucleolar protein 4 like | **1,036** | 0,590522 | 5,262 | 4,980 |
| HNRNPC | heterogeneous nuclear ribonucleoprotein C (C1/C2) | **1,036** | 0,176832 | 260,168 | 275,895 |
| RFX5 | regulatory factor X5 | **1,036** | 0,388842 | 16,783 | 12,834 |
| GDF9 | growth differentiation factor 9 | **1,036** | 0,799024 | 0,554 | 1,213 |
| MPP6 | membrane palmitoylated protein 6 | **1,036** | 0,422164 | 13,183 | 16,537 |
| INPP5K | inositol polyphosphate-5-phosphatase K | **1,036** | 0,227678 | 40,102 | 37,991 |
| NEK11 | NIMA related kinase 11 | **1,036** | 0,442091 | 6,591 | 7,790 |
| DHTKD1 | dehydrogenase E1 and transketolase domain containing 1 | **1,036** | 0,516543 | 6,425 | 4,342 |
| ANKRD28 | ankyrin repeat domain 28 | **1,036** | 0,273763 | 89,898 | 76,237 |
| PRPF4B | pre-mRNA processing factor 4B | **1,036** | 0,304413 | 49,907 | 60,338 |
| COMMD9 | COMM domain containing 9 | **1,036** | 0,368013 | 9,472 | 11,174 |
| ZDHHC15 | zinc finger DHHC-type containing 15 | **1,036** | 0,677243 | 2,271 | 3,639 |
| ZFP1 | ZFP1 zinc finger protein | **1,036** | 0,670589 | 2,493 | 3,192 |
| DYNC1I2 | dynein cytoplasmic 1 intermediate chain 2 | **1,036** | 0,16659 | 87,295 | 87,027 |
| FBXO2 | F-box protein 2 | **1,036** | 0,326 | 35,671 | 30,201 |
| SUPT20H | SPT20 homolog, SAGA complex component | **1,036** | 0,379191 | 18,556 | 19,027 |
| L3MBTL1 | l(3)mbt-like 1 (Drosophila) | **1,036** | 0,669099 | 2,548 | 2,746 |
| SLC25A40 | solute carrier family 25 member 40 | **1,036** | 0,483425 | 8,475 | 9,450 |

| | | | | | |
|---|---|---|---|---|---|
| RASSF8-AS1 | RASSF8 antisense RNA 1 | **1,035** | 0,225016 | 16,063 | 20,560 |
| ARHGAP26 | Rho GTPase activating protein 26 | **1,035** | 0,473138 | 9,472 | 12,515 |
| ME2 | malic enzyme 2 | **1,035** | 0,294696 | 18,666 | 18,006 |
| RBMXL1 | RNA binding motif protein, X-linked like 1 | **1,035** | 0,372931 | 17,448 | 16,282 |
| GABARAPL2 | GABA type A receptor associated protein like 2 | **1,035** | 0,26492 | 59,157 | 55,932 |
| GNG5 | G protein subunit gamma 5 | **1,035** | 0,243408 | 40,213 | 39,076 |
| MR1 | major histocompatibility complex, class I-related | **1,035** | 0,307866 | 16,395 | 18,836 |
| ZNF558 | zinc finger protein 558 | **1,035** | 0,464942 | 15,288 | 15,388 |
| PACERR | PTGS2 antisense NFKB1 complex-mediated expression regulator RNA | **1,035** | 0,846336 | 0,388 | 0,575 |
| BDKRB2 | bradykinin receptor B2 | **1,035** | 0,274846 | 65,028 | 78,982 |
| TRIB2 | tribbles pseudokinase 2 | **1,035** | 0,396453 | 12,906 | 18,197 |
| PDZD2 | PDZ domain containing 2 | **1,035** | 0,411499 | 11,466 | 17,431 |
| MAPK8IP1 | mitogen-activated protein kinase 8 interacting protein 1 | **1,035** | 0,412169 | 12,407 | 12,131 |
| TBP | TATA-box binding protein | **1,035** | 0,439451 | 11,355 | 11,876 |
| FRZB | frizzled-related protein | **1,035** | 0,252562 | 106,958 | 87,538 |
| BTN3A1 | butyrophilin subfamily 3 member A1 | **1,035** | 0,432167 | 14,235 | 19,283 |
| ZFP37 | ZFP37 zinc finger protein | **1,035** | 0,43886 | 6,481 | 10,535 |
| HNRNPF | heterogeneous nuclear ribonucleoprotein F | **1,035** | 0,199563 | 139,306 | 126,742 |
| GLB1 | galactosidase beta 1 | **1,035** | 0,224517 | 44,866 | 33,968 |
| SIAE | sialic acid acetylesterase | **1,035** | 0,375828 | 11,244 | 7,534 |
| C11orf63 | chromosome 11 open reading frame 63 | **1,035** | 0,546761 | 3,434 | 3,959 |
| ANKK1 | ankyrin repeat and kinase domain containing 1 | **1,035** | 0,863044 | 0,222 | 0,319 |
| NMI | N-myc and STAT interactor | **1,035** | 0,312835 | 20,771 | 16,920 |
| CD81 | CD81 molecule | **1,035** | 0,238651 | 337,492 | 209,172 |
| ARGLU1 | arginine and glutamate rich 1 | **1,035** | 0,233436 | 39,604 | 43,801 |
| BIRC6 | baculoviral IAP repeat containing 6 | **1,035** | 0,488008 | 68,130 | 68,447 |
| HOXC6 | homeobox C6 | **1,035** | 0,507876 | 7,588 | 5,810 |
| NEDD1 | neural precursor cell expressed, developmentally down-regulated 1 | **1,035** | 0,479113 | 10,635 | 11,365 |
| GK | glycerol kinase | **1,035** | 0,587181 | 6,370 | 4,086 |
| ZNRF3 | zinc and ring finger 3 | **1,035** | 0,446118 | 7,810 | 8,939 |
| ZNF765 | zinc finger protein 765 | **1,035** | 0,511694 | 7,921 | 6,193 |
| MOB2 | MOB kinase activator 2 | **1,035** | 0,323356 | 22,101 | 23,816 |
| MPPED2 | metallophosphoesterase domain containing 2 | **1,035** | 0,721255 | 1,329 | 2,426 |

| | | | | | |
|---|---|---|---|---|---|
| TUSC3 | tumor suppressor candidate 3 | **1,035** | 0,200819 | 85,855 | 65,063 |
| HMGN3 | high mobility group nucleosomal binding domain 3 | **1,035** | 0,475145 | 10,635 | 11,748 |
| CEP44 | centrosomal protein 44 | **1,035** | 0,540941 | 6,924 | 7,470 |
| PPP2R5C | protein phosphatase 2 regulatory subunit B'gamma | **1,035** | 0,200782 | 45,309 | 40,864 |
| PHC3 | polyhomeotic homolog 3 | **1,035** | 0,475256 | 52,731 | 61,871 |
| TBC1D7 | TBC1 domain family member 7 | **1,035** | 0,537885 | 6,647 | 6,513 |
| PRIMPOL | primase and DNA directed polymerase | **1,035** | 0,478621 | 6,591 | 6,257 |
| NUDT13 | nudix hydrolase 13 | **1,034** | 0,748465 | 0,499 | 0,830 |
| LRRN4CL | LRRN4 C-terminal like | **1,034** | 0,586702 | 3,822 | 5,172 |
| ZNF570 | zinc finger protein 570 | **1,034** | 0,520035 | 5,096 | 5,555 |
| ZNF621 | zinc finger protein 621 | **1,034** | 0,436659 | 36,059 | 38,182 |
| ERLIN2 | ER lipid raft associated 2 | **1,034** | 0,164831 | 65,527 | 70,873 |
| PHF21A | PHD finger protein 21A | **1,034** | 0,328152 | 27,141 | 27,136 |
| KCTD6 | potassium channel tetramerization domain containing 6 | **1,034** | 0,580198 | 3,988 | 3,959 |
| PLD3 | phospholipase D family member 3 | **1,034** | 0,230979 | 209,042 | 140,917 |
| TGIF1 | TGFB induced factor homeobox 1 | **1,034** | 0,163593 | 141,577 | 169,330 |
| MRPL45P2 | mitochondrial ribosomal protein L45 pseudogene 2 | **1,034** | 0,786451 | 0,720 | 1,405 |
| RPL39 | ribosomal protein L39 | **1,034** | 0,540869 | 5,871 | 7,917 |
| BICD2 | BICD cargo adaptor 2 | **1,034** | 0,334347 | 32,846 | 28,796 |
| WBP4 | WW domain binding protein 4 | **1,034** | 0,40613 | 23,652 | 24,135 |
| DYRK1A | dual specificity tyrosine phosphorylation regulated kinase 1A | **1,034** | 0,275751 | 51,402 | 54,400 |
| LHPP | phospholysine phosphohistidine inorganic pyrophosphate phosphatase | **1,034** | 0,539554 | 3,988 | 4,278 |
| TRIM2 | tripartite motif containing 2 | **1,034** | 0,338512 | 20,993 | 20,751 |
| SCLT1 | sodium channel and clathrin linker 1 | **1,034** | 0,579228 | 4,819 | 3,576 |
| HPS5 | HPS5, biogenesis of lysosomal organelles complex 2 subunit 2 | **1,034** | 0,323277 | 25,147 | 36,586 |
| RAET1G | retinoic acid early transcript 1G | **1,034** | 0,631728 | 3,268 | 3,001 |
| WHAMMP3 | WAS protein homolog associated with actin, golgi membranes and microtubules pseudoge | **1,034** | 0,647387 | 2,216 | 2,107 |
| NDST1 | N-deacetylase and N-sulfotransferase 1 | **1,034** | 0,317549 | 63,865 | 32,308 |
| BCAS3 | BCAS3, microtubule associated cell migration factor | **1,034** | 0,427719 | 11,355 | 9,769 |
| CTSF | cathepsin F | **1,034** | 0,360001 | 47,580 | 42,077 |
| ANKRD54 | ankyrin repeat domain 54 | **1,034** | 0,431665 | 15,177 | 15,899 |
| AP1S3 | adaptor related protein complex 1 sigma 3 subunit | **1,034** | 0,783773 | 0,831 | 0,894 |
| KLHL18 | kelch like family member 18 | **1,034** | 0,390294 | 22,433 | 25,923 |

| | | | | | |
|---|---|---|---|---|---|
| CC2D1B | coiled-coil and C2 domain containing 1B | **1,034** | 0,206466 | 41,100 | 35,756 |
| ZNF611 | zinc finger protein 611 | **1,034** | 0,507496 | 8,918 | 11,110 |
| PLCB4 | phospholipase C beta 4 | **1,034** | 0,803196 | 1,274 | 1,085 |
| XAB2 | XPA binding protein 2 | **1,034** | 0,284927 | 40,601 | 43,929 |
| WDFY4 | WDFY family member 4 | **1,034** | 0,859807 | 0,388 | 0,383 |
| NUP50-AS1 | NUP50 antisense RNA 1 (head to head) | **1,034** | 0,563626 | 4,487 | 3,831 |
| GRTP1 | growth hormone regulated TBC protein 1 | **1,034** | 0,804175 | 1,440 | 0,894 |
| RAB31 | RAB31, member RAS oncogene family | **1,034** | 0,293473 | 30,575 | 23,816 |
| IP6K1 | inositol hexakisphosphate kinase 1 | **1,034** | 0,225985 | 72,284 | 66,723 |
| PTHLH | parathyroid hormone like hormone | **1,034** | 0,712515 | 2,216 | 2,682 |
| NSUN5P1 | NOP2/Sun RNA methyltransferase family member 5 pseudogene 1 | **1,034** | 0,733009 | 1,662 | 1,724 |
| PTK7 | protein tyrosine kinase 7 (inactive) | **1,034** | 0,377359 | 21,104 | 18,133 |
| GABARAP | GABA type A receptor-associated protein | **1,034** | 0,509618 | 9,139 | 9,641 |
| EXD3 | exonuclease 3'-5' domain containing 3 | **1,034** | 0,6166 | 3,988 | 5,236 |
| SBF1 | SET binding factor 1 | **1,034** | 0,235051 | 116,652 | 129,871 |
| D2HGDH | D-2-hydroxyglutarate dehydrogenase | **1,034** | 0,454581 | 8,364 | 12,898 |
| ARID1A | AT-rich interaction domain 1A | **1,034** | 0,381757 | 63,643 | 66,340 |
| ARSJ | arylsulfatase family member J | **1,034** | 0,292262 | 78,654 | 61,168 |
| SLC35E2B | solute carrier family 35 member E2B | **1,034** | 0,285487 | 29,135 | 42,716 |
| HACD2 | 3-hydroxyacyl-CoA dehydratase 2 | **1,034** | 0,285049 | 27,474 | 33,777 |
| PRKCQ | protein kinase C theta | **1,034** | 0,75513 | 1,219 | 2,299 |
| PAXIP1 | PAX interacting protein 1 | **1,034** | 0,45821 | 6,979 | 8,300 |
| HSPA13 | heat shock protein family A (Hsp70) member 13 | **1,034** | 0,291487 | 166,724 | 151,005 |
| SUPT5H | SPT5 homolog, DSIF elongation factor subunit | **1,033** | 0,243721 | 91,006 | 100,500 |
| MEX3C | mex-3 RNA binding family member C | **1,033** | 0,340615 | 44,146 | 37,735 |
| KLHL29 | kelch like family member 29 | **1,033** | 0,323067 | 63,865 | 71,959 |
| MAP2 | microtubule associated protein 2 | **1,033** | 0,738465 | 1,662 | 1,469 |
| VPS13D | vacuolar protein sorting 13 homolog D | **1,033** | 0,49417 | 53,396 | 63,658 |
| ADAM19 | ADAM metallopeptidase domain 19 | **1,033** | 0,873034 | 0,554 | 0,511 |
| CLEC2B | C-type lectin domain family 2 member B | **1,033** | 0,712266 | 3,268 | 1,469 |
| FXR1 | FMR1 autosomal homolog 1 | **1,033** | 0,190053 | 84,636 | 75,024 |
| FGD4 | FYVE, RhoGEF and PH domain containing 4 | **1,033** | 0,372977 | 24,206 | 14,111 |
| ARL3 | ADP ribosylation factor like GTPase 3 | **1,033** | 0,298954 | 39,936 | 46,227 |

| | | | | | |
|---|---|---|---|---|---|
| CMTR1 | cap methyltransferase 1 | **1,033** | 0,22936 | 36,170 | 45,461 |
| LCOR | ligand dependent nuclear receptor corepressor | **1,033** | 0,528079 | 19,774 | 19,730 |
| LUM | lumican | **1,033** | 0,255285 | 7972,306 | 5169,989 |
| SUGP2 | SURP and G-patch domain containing 2 | **1,033** | 0,2708 | 41,487 | 38,885 |
| LINC01679 | long intergenic non-protein coding RNA 1679 | **1,033** | 0,775375 | 0,443 | 0,830 |
| SOD3 | superoxide dismutase 3 | **1,033** | 0,344177 | 848,799 | 806,488 |
| UBOX5 | U-box domain containing 5 | **1,033** | 0,463851 | 7,976 | 9,322 |
| ZHX1 | zinc fingers and homeoboxes 1 | **1,033** | 0,368825 | 19,719 | 19,410 |
| KTN1 | kinectin 1 | **1,033** | 0,248996 | 173,427 | 164,924 |
| LINC01137 | long intergenic non-protein coding RNA 1137 | **1,033** | 0,505752 | 9,527 | 5,683 |
| RPL7AP6 | ribosomal protein L7a pseudogene 6 | **1,033** | 0,890688 | 0,332 | 0,128 |
| SGMS1 | sphingomyelin synthase 1 | **1,033** | 0,289218 | 35,117 | 30,967 |
| ZNF30 | zinc finger protein 30 | **1,033** | 0,785133 | 1,329 | 1,405 |
| TFAP4 | transcription factor AP-4 | **1,033** | 0,566405 | 3,933 | 6,193 |
| SIRT5 | sirtuin 5 | **1,033** | 0,499389 | 6,536 | 5,236 |
| TULP4 | tubby like protein 4 | **1,033** | 0,490123 | 16,229 | 15,132 |
| SRPK1 | SRSF protein kinase 1 | **1,033** | 0,200749 | 56,941 | 62,509 |
| CYTH1 | cytohesin 1 | **1,033** | 0,330495 | 32,680 | 31,414 |
| ATP6AP2 | ATPase H+ transporting accessory protein 2 | **1,033** | 0,241968 | 156,422 | 109,822 |
| PNPLA7 | patatin like phospholipase domain containing 7 | **1,033** | 0,722887 | 1,329 | 2,299 |
| IFNGR1 | interferon gamma receptor 1 | **1,033** | 0,241855 | 66,690 | 70,043 |
| OSMR | oncostatin M receptor | **1,033** | 0,331213 | 344,361 | 272,384 |
| MT-RNR1 | mitochondrially encoded 12S RNA | **1,033** | 0,816462 | 146,950 | 151,133 |
| RHBDL2 | rhomboid like 2 | **1,033** | 0,650156 | 2,049 | 1,596 |
| TBRG1 | transforming growth factor beta regulator 1 | **1,032** | 0,315473 | 34,231 | 33,713 |
| NBR2 | neighbor of BRCA1 gene 2 (non-protein coding) | **1,032** | 0,654634 | 2,548 | 2,937 |
| FGD6 | FYVE, RhoGEF and PH domain containing 6 | **1,032** | 0,687625 | 18,002 | 3,959 |
| ZNF609 | zinc finger protein 609 | **1,032** | 0,483263 | 36,225 | 35,884 |
| FAM47E | family with sequence similarity 47 member E | **1,032** | 0,81098 | 0,720 | 0,575 |
| THAP9 | THAP domain containing 9 | **1,032** | 0,664606 | 4,597 | 5,683 |
| RADIL | Rap associating with DIL domain | **1,032** | 0,731367 | 0,997 | 1,596 |
| F8 | coagulation factor VIII | **1,032** | 0,450008 | 7,865 | 6,513 |
| FTL | ferritin light chain | **1,032** | 0,329187 | 1702,417 | 1206,252 |

| | | | | | |
|---|---|---|---|---|---|
| C9orf85 | chromosome 9 open reading frame 85 | **1,032** | 0,530351 | 7,699 | 9,833 |
| LINC01554 | long intergenic non-protein coding RNA 1554 | **1,032** | 0,473966 | 8,585 | 9,131 |
| P2RX5 | purinergic receptor P2X 5 | **1,032** | 0,851214 | 0,388 | 0,511 |
| XPO5 | exportin 5 | **1,032** | 0,279993 | 33,234 | 38,246 |
| MGST3 | microsomal glutathione S-transferase 3 | **1,032** | 0,431574 | 36,059 | 32,947 |
| MAGED2 | MAGE family member D2 | **1,032** | 0,266527 | 196,967 | 146,727 |
| CLIP2 | CAP-Gly domain containing linker protein 2 | **1,032** | 0,399007 | 90,286 | 77,258 |
| MRPL41 | mitochondrial ribosomal protein L41 | **1,032** | 0,43592 | 20,937 | 16,537 |
| NRAS | NRAS proto-oncogene, GTPase | **1,032** | 0,293657 | 43,315 | 38,118 |
| ITGB1 | integrin subunit beta 1 | **1,032** | 0,388165 | 715,364 | 501,669 |
| ISLR | immunoglobulin superfamily containing leucine rich repeat | **1,032** | 0,235659 | 122,855 | 97,626 |
| GCC2-AS1 | GCC2 antisense RNA 1 | **1,032** | 0,876446 | 0,222 | 0,575 |
| FUBP3 | far upstream element binding protein 3 | **1,032** | 0,301605 | 47,359 | 45,908 |
| FLOT2 | flotillin 2 | **1,032** | 0,302353 | 52,122 | 43,609 |
| IKBKB | inhibitor of nuclear factor kappa B kinase subunit beta | **1,032** | 0,258309 | 26,144 | 28,924 |
| UBE4B | ubiquitination factor E4B | **1,032** | 0,33186 | 43,094 | 44,184 |
| DNMT3B | DNA methyltransferase 3 beta | **1,032** | 0,829587 | 0,720 | 0,575 |
| SCN1B | sodium voltage-gated channel beta subunit 1 | **1,032** | 0,572014 | 7,976 | 3,512 |
| OCIAD1 | OCIA domain containing 1 | **1,032** | 0,236234 | 97,044 | 100,883 |
| C11orf65 | chromosome 11 open reading frame 65 | **1,032** | 0,827115 | 0,332 | 1,022 |
| GRIK5 | glutamate ionotropic receptor kainate type subunit 5 | **1,032** | 0,728194 | 1,496 | 3,639 |
| ARMCX3 | armadillo repeat containing, X-linked 3 | **1,032** | 0,332863 | 49,131 | 36,905 |
| ZFAND2A | zinc finger AN1-type containing 2A | **1,032** | 0,371767 | 20,882 | 24,391 |
| LARP4 | La ribonucleoprotein domain family member 4 | **1,032** | 0,396199 | 31,018 | 30,520 |
| LSM12 | LSM12 homolog | **1,032** | 0,642203 | 2,493 | 2,235 |
| NFXL1 | nuclear transcription factor, X-box binding like 1 | **1,032** | 0,294109 | 16,728 | 18,580 |
| MMRN2 | multimerin 2 | **1,032** | 0,64036 | 1,052 | 3,129 |
| C4orf47 | chromosome 4 open reading frame 47 | **1,032** | 0,74959 | 0,942 | 1,277 |
| ZNF574 | zinc finger protein 574 | **1,032** | 0,341511 | 19,497 | 22,858 |
| STAT3 | signal transducer and activator of transcription 3 | **1,032** | 0,215299 | 249,034 | 259,422 |
| SGK1 | serum/glucocorticoid regulated kinase 1 | **1,032** | 0,257402 | 491,865 | 254,442 |
| DNAJA1 | DnaJ heat shock protein family (Hsp40) member A1 | **1,032** | 0,239474 | 131,496 | 134,149 |
| ACAA1 | acetyl-CoA acyltransferase 1 | **1,032** | 0,394975 | 29,080 | 29,371 |

| | | | | | |
|---|---|---|---|---|---|
| AGTPBP1 | ATP/GTP binding protein 1 | **1,032** | 0,455151 | 9,527 | 8,492 |
| MBD6 | methyl-CpG binding domain protein 6 | **1,032** | 0,296275 | 33,123 | 39,715 |
| VCPKMT | valosin containing protein lysine methyltransferase | **1,031** | 0,61185 | 5,041 | 7,215 |
| FBXO21 | F-box protein 21 | **1,031** | 0,348336 | 25,424 | 26,945 |
| DNAJC9 | DnaJ heat shock protein family (Hsp40) member C9 | **1,031** | 0,476268 | 11,687 | 12,004 |
| C16orf70 | chromosome 16 open reading frame 70 | **1,031** | 0,436293 | 10,801 | 12,834 |
| MKNK1 | MAP kinase interacting serine/threonine kinase 1 | **1,031** | 0,387965 | 12,020 | 13,153 |
| PXK | PX domain containing serine/threonine kinase like | **1,031** | 0,303617 | 48,965 | 52,165 |
| TRAF3IP2-AS1 | TRAF3IP2 antisense RNA 1 | **1,031** | 0,757707 | 1,219 | 1,213 |
| FBXO28 | F-box protein 28 | **1,031** | 0,338834 | 36,447 | 34,287 |
| PGBD4 | piggyBac transposable element derived 4 | **1,031** | 0,61374 | 7,865 | 10,791 |
| BTBD9 | BTB domain containing 9 | **1,031** | 0,459328 | 15,565 | 17,878 |
| WDR25 | WD repeat domain 25 | **1,031** | 0,425378 | 10,580 | 14,111 |
| UBE3A | ubiquitin protein ligase E3A | **1,031** | 0,211506 | 72,229 | 77,705 |
| USP8 | ubiquitin specific peptidase 8 | **1,031** | 0,339605 | 49,076 | 45,653 |
| PHLDB2 | pleckstrin homology like domain family B member 2 | **1,031** | 0,708153 | 2,936 | 3,448 |
| PNPLA8 | patatin like phospholipase domain containing 8 | **1,031** | 0,287162 | 59,101 | 58,614 |
| MSH6 | mutS homolog 6 | **1,031** | 0,31321 | 47,359 | 46,483 |
| MGME1 | mitochondrial genome maintenance exonuclease 1 | **1,031** | 0,454154 | 11,189 | 10,535 |
| ALG11 | ALG11, alpha-1,2-mannosyltransferase | **1,031** | 0,5137 | 9,970 | 11,685 |
| DDX6 | DEAD-box helicase 6 | **1,031** | 0,310922 | 125,016 | 130,828 |
| MRPS22 | mitochondrial ribosomal protein S22 | **1,031** | 0,330566 | 29,689 | 37,927 |
| ZNF790-AS1 | ZNF790 antisense RNA 1 | **1,031** | 0,819769 | 0,443 | 0,766 |
| 11.syys | septin 11 | **1,031** | 0,30791 | 48,965 | 42,077 |
| FOXD2 | forkhead box D2 | **1,031** | 0,438626 | 8,032 | 11,110 |
| NFATC3 | nuclear factor of activated T-cells 3 | **1,031** | 0,284748 | 21,935 | 25,285 |
| YEATS4 | YEATS domain containing 4 | **1,031** | 0,545373 | 9,859 | 8,173 |
| NCOA6 | nuclear receptor coactivator 6 | **1,031** | 0,479356 | 23,873 | 24,391 |
| ANKS1B | ankyrin repeat and sterile alpha motif domain containing 1B | **1,031** | 0,808432 | 1,163 | 0,702 |
| WAPL | WAPL cohesin release factor | **1,031** | 0,311395 | 48,854 | 51,527 |
| HDX | highly divergent homeobox | **1,031** | 0,825492 | 0,942 | 0,319 |
| RNF169 | ring finger protein 169 | **1,031** | 0,491845 | 20,107 | 21,773 |
| MTF1 | metal regulatory transcription factor 1 | **1,031** | 0,403726 | 38,496 | 32,627 |

| | | | | | |
|---|---|---|---|---|---|
| LRPPRC | leucine rich pentatricopeptide repeat containing | **1,031** | 0,294258 | 53,618 | 54,017 |
| BACE1-AS | BACE1 antisense RNA | **1,031** | 0,813183 | 0,499 | 0,575 |
| KIN | Kin17 DNA and RNA binding protein | **1,031** | 0,405909 | 10,413 | 10,663 |
| STX17 | syntaxin 17 | **1,031** | 0,533604 | 14,678 | 17,623 |
| IKZF4 | IKAROS family zinc finger 4 | **1,031** | 0,507996 | 10,469 | 9,322 |
| CDC37L1 | cell division cycle 37 like 1 | **1,031** | 0,381402 | 19,054 | 18,644 |
| DTX2 | deltex E3 ubiquitin ligase 2 | **1,031** | 0,436905 | 18,611 | 15,005 |
| SLC23A3 | solute carrier family 23 member 3 | **1,031** | 0,864599 | 0,388 | 0,319 |
| SS18L1 | SS18L1, nBAF chromatin remodeling complex subunit | **1,031** | 0,392405 | 14,291 | 17,495 |
| RBM6 | RNA binding motif protein 6 | **1,031** | 0,283428 | 41,598 | 48,143 |
| EXO5 | exonuclease 5 | **1,031** | 0,588609 | 6,038 | 6,896 |
| ZNF205 | zinc finger protein 205 | **1,031** | 0,541602 | 8,530 | 5,427 |
| SIKE1 | suppressor of IKBKE 1 | **1,031** | 0,350688 | 30,132 | 32,883 |
| BAG4 | BCL2 associated athanogene 4 | **1,031** | 0,364821 | 24,649 | 22,475 |
| CDK11A | cyclin dependent kinase 11A | **1,031** | 0,783143 | 1,052 | 1,341 |
| ZHX2 | zinc fingers and homeoboxes 2 | **1,031** | 0,279013 | 68,351 | 74,257 |
| KIF9 | kinesin family member 9 | **1,031** | 0,616763 | 3,434 | 3,895 |
| MRPL30 | mitochondrial ribosomal protein L30 | **1,031** | 0,383309 | 13,460 | 13,983 |
| ADD1 | adducin 1 | **1,031** | 0,245267 | 109,285 | 97,818 |
| RC3H1 | ring finger and CCCH-type domains 1 | **1,031** | 0,499391 | 22,599 | 20,624 |
| USP15 | ubiquitin specific peptidase 15 | **1,031** | 0,309751 | 37,665 | 43,035 |
| HMG20B | high mobility group 20B | **1,030** | 0,342774 | 38,607 | 40,992 |
| DNAAF4 | dynein axonemal assembly factor 4 | **1,030** | 0,856183 | 0,609 | 0,702 |
| USP20 | ubiquitin specific peptidase 20 | **1,030** | 0,326979 | 19,996 | 24,263 |
| WBP2 | WW domain binding protein 2 | **1,030** | 0,261421 | 145,565 | 143,088 |
| STK3 | serine/threonine kinase 3 | **1,030** | 0,358993 | 19,165 | 20,879 |
| PAPOLG | poly(A) polymerase gamma | **1,030** | 0,506907 | 13,626 | 14,685 |
| PRG4 | proteoglycan 4 | **1,030** | 0,425431 | 649,727 | 415,727 |
| ZBTB39 | zinc finger and BTB domain containing 39 | **1,030** | 0,548429 | 11,078 | 12,259 |
| ATF7IP | activating transcription factor 7 interacting protein | **1,030** | 0,427243 | 20,051 | 22,794 |
| LAMP3 | lysosomal associated membrane protein 3 | **1,030** | 0,681972 | 2,880 | 6,321 |
| NPC2 | NPC intracellular cholesterol transporter 2 | **1,030** | 0,289502 | 105,186 | 83,005 |
| SLC9A3R1 | SLC9A3 regulator 1 | **1,030** | 0,443195 | 39,272 | 49,739 |

| | | | | | |
|---|---|---|---|---|---|
| PDHX | pyruvate dehydrogenase complex component X | **1,030** | 0,466778 | 13,072 | 18,133 |
| HEIH | hepatocellular carcinoma up-regulated EZH2-associated long non-coding RNA | **1,030** | 0,420106 | 17,226 | 22,411 |
| RBSN | rabenosyn, RAB effector | **1,030** | 0,386 | 26,033 | 32,053 |
| DYSF | dysferlin | **1,030** | 0,536054 | 11,133 | 12,131 |
| RFX7 | regulatory factor X7 | **1,030** | 0,573217 | 10,192 | 8,556 |
| AGTR1 | angiotensin II receptor type 1 | **1,030** | 0,663954 | 2,382 | 1,532 |
| CBR4 | carbonyl reductase 4 | **1,030** | 0,453138 | 9,139 | 12,323 |
| CCDC17 | coiled-coil domain containing 17 | **1,030** | 0,794855 | 0,775 | 1,277 |
| APH1B | aph-1 homolog B, gamma-secretase subunit | **1,030** | 0,412145 | 26,587 | 23,369 |
| C1orf132 | chromosome 1 open reading frame 132 | **1,030** | 0,757469 | 6,702 | 6,385 |
| ATRIP | ATR interacting protein | **1,030** | 0,615156 | 5,207 | 6,130 |
| STX2 | syntaxin 2 | **1,030** | 0,474685 | 17,559 | 9,641 |
| SGCB | sarcoglycan beta | **1,030** | 0,3575 | 69,847 | 51,846 |
| RNF166 | ring finger protein 166 | **1,030** | 0,537139 | 6,702 | 8,300 |
| ZC3H13 | zinc finger CCCH-type containing 13 | **1,030** | 0,349078 | 70,733 | 84,537 |
| GOLGB1 | golgin B1 | **1,030** | 0,534493 | 145,178 | 142,130 |
| CD27-AS1 | CD27 antisense RNA 1 | **1,030** | 0,582893 | 5,428 | 6,321 |
| ASCC3 | activating signal cointegrator 1 complex subunit 3 | **1,030** | 0,39112 | 33,345 | 42,588 |
| GNAQ | G protein subunit alpha q | **1,030** | 0,345714 | 39,382 | 33,904 |
| PITPNB | phosphatidylinositol transfer protein beta | **1,030** | 0,277605 | 51,845 | 50,888 |
| ZNF746 | zinc finger protein 746 | **1,030** | 0,345197 | 19,719 | 18,963 |
| TIPRL | TOR signaling pathway regulator | **1,030** | 0,308774 | 39,382 | 46,100 |
| DCAF10 | DDB1 and CUL4 associated factor 10 | **1,030** | 0,370096 | 26,255 | 26,817 |
| SP110 | SP110 nuclear body protein | **1,030** | 0,444736 | 12,130 | 13,664 |
| SUN2 | Sad1 and UNC84 domain containing 2 | **1,030** | 0,241031 | 163,512 | 168,244 |
| PLEKHA3 | pleckstrin homology domain containing A3 | **1,030** | 0,337555 | 46,805 | 41,822 |
| ATM | ATM serine/threonine kinase | **1,030** | 0,53713 | 53,839 | 52,102 |
| 7.maalis | membrane associated ring-CH-type finger 7 | **1,030** | 0,376324 | 58,824 | 53,634 |
| BCL2L13 | BCL2 like 13 | **1,030** | 0,299979 | 54,005 | 65,063 |
| SF1 | splicing factor 1 | **1,030** | 0,219952 | 122,080 | 148,451 |
| NSMF | NMDA receptor synaptonuclear signaling and neuronal migration factor | **1,030** | 0,414613 | 16,451 | 14,877 |
| ZNF629 | zinc finger protein 629 | **1,030** | 0,362519 | 22,821 | 23,816 |
| SLC19A2 | solute carrier family 19 member 2 | **1,030** | 0,462756 | 23,707 | 19,921 |

| | | | | | |
|---|---|---|---|---|---|
| INF2 | inverted formin, FH2 and WH2 domain containing | **1,030** | 0,324621 | 153,375 | 132,808 |
| PANK2 | pantothenate kinase 2 | **1,030** | 0,290821 | 33,345 | 38,693 |
| ABHD4 | abhydrolase domain containing 4 | **1,029** | 0,312786 | 53,064 | 38,948 |
| CHMP1B | charged multivesicular body protein 1B | **1,029** | 0,334236 | 71,398 | 58,614 |
| PC | pyruvate carboxylase | **1,029** | 0,346912 | 84,636 | 100,627 |
| SBDSP1 | SBDS, ribosome assembly guanine nucleotide exchange factor pseudogene 1 | **1,029** | 0,357087 | 14,124 | 16,346 |
| ATP2C2-AS1 | ATP2C2 antisense RNA 1 | **1,029** | 0,832187 | 0,277 | 0,575 |
| TNFRSF10D | TNF receptor superfamily member 10d | **1,029** | 0,291954 | 218,514 | 290,325 |
| SLC25A46 | solute carrier family 25 member 46 | **1,029** | 0,339635 | 20,993 | 23,050 |
| ELOC | elongin C | **1,029** | 0,310443 | 35,062 | 37,863 |
| RCBTB2 | RCC1 and BTB domain containing protein 2 | **1,029** | 0,498343 | 9,804 | 8,300 |
| FNBP1 | formin binding protein 1 | **1,029** | 0,479946 | 31,129 | 20,304 |
| FDXR | ferredoxin reductase | **1,029** | 0,589414 | 4,376 | 5,108 |
| AZIN1-AS1 | AZIN1 antisense RNA 1 | **1,029** | 0,755257 | 1,440 | 1,596 |
| SCAMP5 | secretory carrier membrane protein 5 | **1,029** | 0,726979 | 1,496 | 1,724 |
| PARP6 | poly(ADP-ribose) polymerase family member 6 | **1,029** | 0,351637 | 21,547 | 22,092 |
| WDR81 | WD repeat domain 81 | **1,029** | 0,741112 | 3,157 | 2,107 |
| SUCLG1 | succinate-CoA ligase alpha subunit | **1,029** | 0,422953 | 19,276 | 20,624 |
| MCUR1 | mitochondrial calcium uniporter regulator 1 | **1,029** | 0,333057 | 24,815 | 23,497 |
| DDX19B | DEAD-box helicase 19B | **1,029** | 0,518527 | 6,204 | 7,407 |
| C16orf86 | chromosome 16 open reading frame 86 | **1,029** | 0,807708 | 1,385 | 1,022 |
| PLAT | plasminogen activator, tissue type | **1,029** | 0,416339 | 35,616 | 48,462 |
| ETFBKMT | electron transfer flavoprotein beta subunit lysine methyltransferase | **1,029** | 0,631569 | 3,600 | 3,703 |
| IL17RA | interleukin 17 receptor A | **1,029** | 0,368476 | 17,669 | 13,153 |
| ZNF316 | zinc finger protein 316 | **1,029** | 0,440031 | 24,372 | 25,923 |
| HMGB1 | high mobility group box 1 | **1,029** | 0,326392 | 89,234 | 78,599 |
| SPOCK1 | SPARC/osteonectin, cwcv and kazal like domains proteoglycan 1 | **1,029** | 0,437448 | 83,196 | 56,316 |
| LPCAT4 | lysophosphatidylcholine acyltransferase 4 | **1,029** | 0,606137 | 4,043 | 4,150 |
| PLRG1 | pleiotropic regulator 1 | **1,029** | 0,277801 | 71,010 | 74,002 |
| ZC2HC1C | zinc finger C2HC-type containing 1C | **1,029** | 0,694892 | 2,049 | 1,596 |
| BUB3 | BUB3, mitotic checkpoint protein | **1,029** | 0,278418 | 63,920 | 59,827 |
| ALDH6A1 | aldehyde dehydrogenase 6 family member A1 | **1,029** | 0,479471 | 16,063 | 17,559 |
| ANKH | ANKH inorganic pyrophosphate transport regulator | **1,029** | 0,345175 | 159,025 | 159,625 |

| | | | | | |
|---|---|---|---|---|---|
| PDS5A | PDS5 cohesin associated factor A | **1,029** | 0,392875 | 85,966 | 79,876 |
| ZRANB2 | zinc finger RANBP2-type containing 2 | **1,029** | 0,290321 | 47,414 | 67,170 |
| IGIP | IgA inducing protein | **1,029** | 0,499828 | 11,964 | 11,876 |
| USP1 | ubiquitin specific peptidase 1 | **1,029** | 0,307858 | 44,811 | 48,462 |
| USP14 | ubiquitin specific peptidase 14 | **1,029** | 0,30972 | 91,228 | 90,795 |
| TMEM128 | transmembrane protein 128 | **1,029** | 0,414061 | 28,415 | 35,947 |
| WASHC2C | WASH complex subunit 2C | **1,029** | 0,483568 | 15,731 | 13,536 |
| ZNF192P1 | zinc finger protein 192 pseudogene 1 | **1,029** | 0,866773 | 0,332 | 0,894 |
| ASCC1 | activating signal cointegrator 1 complex subunit 1 | **1,029** | 0,477384 | 18,833 | 21,134 |
| PAK2 | p21 (RAC1) activated kinase 2 | **1,029** | 0,332168 | 70,179 | 74,066 |
| TDP2 | tyrosyl-DNA phosphodiesterase 2 | **1,028** | 0,316808 | 44,755 | 48,909 |
| RGS9 | regulator of G protein signaling 9 | **1,028** | 0,857353 | 0,665 | 0,830 |
| RRP1B | ribosomal RNA processing 1B | **1,028** | 0,349564 | 28,637 | 30,393 |
| RUNDC1 | RUN domain containing 1 | **1,028** | 0,308667 | 30,797 | 30,520 |
| CPB2-AS1 | CPB2 antisense RNA 1 | **1,028** | 0,872959 | 0,332 | 0,383 |
| TCEA1 | transcription elongation factor A1 | **1,028** | 0,335362 | 82,254 | 89,581 |
| DNAH1 | dynein axonemal heavy chain 1 | **1,028** | 0,72033 | 7,145 | 7,407 |
| NBEAL1 | neurobeachin like 1 | **1,028** | 0,696725 | 9,361 | 11,301 |
| SACS | sacsin molecular chaperone | **1,028** | 0,544915 | 36,668 | 46,355 |
| PPP2R3A | protein phosphatase 2 regulatory subunit B''alpha | **1,028** | 0,552663 | 33,733 | 25,157 |
| ATE1 | arginyltransferase 1 | **1,028** | 0,414736 | 34,508 | 37,480 |
| PLEKHA4 | pleckstrin homology domain containing A4 | **1,028** | 0,44588 | 28,083 | 24,582 |
| HYI | hydroxypyruvate isomerase (putative) | **1,028** | 0,570368 | 7,810 | 6,832 |
| NUP54 | nucleoporin 54 | **1,028** | 0,413233 | 19,774 | 26,370 |
| BLID | BH3-like motif containing, cell death inducer | **1,028** | 0,909724 | 0,277 | 0,383 |
| TMEM209 | transmembrane protein 209 | **1,028** | 0,417926 | 23,430 | 27,455 |
| PIEZO1 | piezo type mechanosensitive ion channel component 1 | **1,028** | 0,276733 | 782,386 | 731,464 |
| FAM8A1 | family with sequence similarity 8 member A1 | **1,028** | 0,387895 | 20,384 | 20,113 |
| FBLN1 | fibulin 1 | **1,028** | 0,563331 | 5,428 | 5,874 |
| MCM2 | minichromosome maintenance complex component 2 | **1,028** | 0,505746 | 13,127 | 15,324 |
| GNPTAB | N-acetylglucosamine-1-phosphate transferase alpha and beta subunits | **1,028** | 0,372751 | 39,050 | 33,138 |
| ERMAP | erythroblast membrane associated protein (Scianna blood group) | **1,028** | 0,443491 | 22,599 | 16,984 |
| ACTR2 | ARP2 actin related protein 2 homolog | **1,028** | 0,314499 | 176,805 | 166,010 |

| | | | | | |
|---|---|---|---|---|---|
| ELK4 | ELK4, ETS transcription factor | **1,028** | 0,549766 | 28,969 | 32,244 |
| FDX1 | ferredoxin 1 | **1,028** | 0,542289 | 10,303 | 10,727 |
| SIRT7 | sirtuin 7 | **1,028** | 0,428172 | 15,288 | 14,813 |
| FOXJ3 | forkhead box J3 | **1,028** | 0,407495 | 40,933 | 43,993 |
| PGP | phosphoglycolate phosphatase | **1,028** | 0,535168 | 10,358 | 9,131 |
| STYX | serine/threonine/tyrosine interacting protein | **1,028** | 0,507375 | 16,672 | 14,558 |
| ZC3H14 | zinc finger CCCH-type containing 14 | **1,028** | 0,388579 | 37,222 | 38,501 |
| TCAIM | T-cell activation inhibitor, mitochondrial | **1,028** | 0,511455 | 10,081 | 10,280 |
| ATP1B3 | ATPase Na+/K+ transporting subunit beta 3 | **1,028** | 0,364147 | 86,132 | 80,387 |
| UBE3B | ubiquitin protein ligase E3B | **1,028** | 0,413606 | 39,604 | 41,119 |
| AMIGO1 | adhesion molecule with Ig like domain 1 | **1,028** | 0,609576 | 9,306 | 7,854 |
| ATP6V0A2 | ATPase H+ transporting V0 subunit a2 | **1,028** | 0,365723 | 20,827 | 27,583 |
| GLB1L2 | galactosidase beta 1 like 2 | **1,028** | 0,653861 | 9,029 | 2,746 |
| IER2 | immediate early response 2 | **1,028** | 0,355492 | 76,549 | 106,246 |
| SAP130 | Sin3A associated protein 130 | **1,028** | 0,525027 | 16,174 | 15,643 |
| USP31 | ubiquitin specific peptidase 31 | **1,028** | 0,62351 | 10,690 | 12,770 |
| DHX8 | DEAH-box helicase 8 | **1,028** | 0,424161 | 45,863 | 52,868 |
| RIOK3 | RIO kinase 3 | **1,028** | 0,295643 | 98,373 | 101,968 |
| FASTKD5 | FAST kinase domains 5 | **1,028** | 0,491502 | 16,174 | 14,749 |
| C17orf80 | chromosome 17 open reading frame 80 | **1,028** | 0,32901 | 21,381 | 22,092 |
| MAP2K4 | mitogen-activated protein kinase kinase 4 | **1,028** | 0,477606 | 15,177 | 13,153 |
| C14orf2 | chromosome 14 open reading frame 2 | **1,028** | 0,438247 | 25,036 | 25,285 |
| RECQL5 | RecQ like helicase 5 | **1,028** | 0,463544 | 18,057 | 19,538 |
| UCN | urocortin | **1,027** | 0,888925 | 0,554 | 0,638 |
| HOXD8 | homeobox D8 | **1,027** | 0,578999 | 11,521 | 13,281 |
| CYB5R2 | cytochrome b5 reductase 2 | **1,027** | 0,392379 | 47,857 | 46,547 |
| EBLN3P | endogenous Bornavirus-like nucleoprotein 3, pseudogene | **1,027** | 0,390804 | 66,579 | 60,147 |
| MGAT3 | mannosyl (beta-1,4-)-glycoprotein beta-1,4-N-acetylglucosaminyltransferase | **1,027** | 0,800641 | 0,388 | 0,766 |
| CAST | calpastatin | **1,027** | 0,286973 | 131,109 | 129,935 |
| METTL15 | methyltransferase like 15 | **1,027** | 0,626712 | 9,029 | 10,982 |
| PLEKHA1 | pleckstrin homology domain containing A1 | **1,027** | 0,43683 | 38,053 | 42,716 |
| PTGS1 | prostaglandin-endoperoxide synthase 1 | **1,027** | 0,574297 | 3,988 | 3,512 |
| MACF1 | microtubule-actin crosslinking factor 1 | **1,027** | 0,571299 | 160,465 | 173,289 |

| | | | | | |
|---|---|---|---|---|---|
| EIF1B-AS1 | EIF1B antisense RNA 1 | **1,027** | 0,862973 | 0,665 | 1,469 |
| TCF7L1 | transcription factor 7 like 1 | **1,027** | 0,793801 | 1,662 | 1,532 |
| SOCS6 | suppressor of cytokine signaling 6 | **1,027** | 0,376405 | 30,797 | 24,838 |
| DPY19L2P3 | DPY19L2 pseudogene 3 | **1,027** | 0,87073 | 0,443 | 0,255 |
| PCNP | PEST proteolytic signal containing nuclear protein | **1,027** | 0,378872 | 93,388 | 92,263 |
| RNF8 | ring finger protein 8 | **1,027** | 0,574009 | 10,303 | 9,514 |
| FARS2 | phenylalanyl-tRNA synthetase 2, mitochondrial | **1,027** | 0,559774 | 7,699 | 8,428 |
| FOXI2 | forkhead box I2 | **1,027** | 0,879517 | 0,166 | 0,702 |
| NAAA | N-acylethanolamine acid amidase | **1,027** | 0,461061 | 19,553 | 10,344 |
| COL9A3 | collagen type IX alpha 3 chain | **1,027** | 0,439115 | 507,928 | 370,585 |
| ZMYM1 | zinc finger MYM-type containing 1 | **1,027** | 0,557845 | 9,416 | 7,279 |
| KCMF1 | potassium channel modulatory factor 1 | **1,027** | 0,315456 | 56,166 | 53,634 |
| STK40 | serine/threonine kinase 40 | **1,027** | 0,313934 | 69,182 | 109,886 |
| PPP4R3B | protein phosphatase 4 regulatory subunit 3B | **1,027** | 0,369472 | 79,429 | 82,302 |
| CHFR | checkpoint with forkhead and ring finger domains | **1,027** | 0,434013 | 19,830 | 21,709 |
| AP002495.2 | Putative short transient receptor potential channel 2-like protein | **1,027** | 0,891931 | 0,609 | 0,383 |
| WDR11 | WD repeat domain 11 | **1,027** | 0,3305 | 72,838 | 65,382 |
| PCDHAC2 | protocadherin alpha subfamily C, 2 | **1,027** | 0,876571 | 0,554 | 0,511 |
| CDADC1 | cytidine and dCMP deaminase domain containing 1 | **1,027** | 0,612619 | 4,043 | 5,810 |
| GINS4 | GINS complex subunit 4 | **1,027** | 0,671542 | 4,431 | 3,129 |
| PLCXD3 | phosphatidylinositol specific phospholipase C X domain containing 3 | **1,027** | 0,827913 | 2,603 | 0,766 |
| ASB6 | ankyrin repeat and SOCS box containing 6 | **1,027** | 0,339866 | 45,254 | 45,014 |
| BRAP | BRCA1 associated protein | **1,027** | 0,38116 | 24,427 | 24,071 |
| ZNF788 | zinc finger family member 788 | **1,027** | 0,670931 | 4,376 | 5,300 |
| NAALADL1 | N-acetylated alpha-linked acidic dipeptidase like 1 | **1,027** | 0,6707 | 4,708 | 4,278 |
| RNF6 | ring finger protein 6 | **1,027** | 0,364507 | 41,930 | 36,969 |
| PTPN12 | protein tyrosine phosphatase, non-receptor type 12 | **1,027** | 0,380429 | 223,444 | 245,247 |
| GTF2H1 | general transcription factor IIH subunit 1 | **1,027** | 0,317603 | 33,289 | 44,823 |
| TACC2 | transforming acidic coiled-coil containing protein 2 | **1,027** | 0,363751 | 20,550 | 21,837 |
| SPG21 | SPG21, maspardin | **1,027** | 0,357779 | 45,198 | 38,438 |
| ADAMTSL3 | ADAMTS like 3 | **1,027** | 0,471911 | 38,108 | 29,754 |
| ZSCAN29 | zinc finger and SCAN domain containing 29 | **1,027** | 0,570501 | 15,897 | 14,430 |
| SOGA1 | suppressor of glucose, autophagy associated 1 | **1,027** | 0,515182 | 26,975 | 18,963 |

| | | | | | |
|---|---|---|---|---|---|
| DAB2IP | DAB2 interacting protein | **1,027** | 0,399508 | 26,864 | 24,965 |
| CNMD | chondromodulin | **1,027** | 0,394919 | 23,984 | 17,303 |
| MAP3K4 | mitogen-activated protein kinase kinase kinase 4 | **1,027** | 0,478179 | 43,038 | 25,029 |
| MBIP | MAP3K12 binding inhibitory protein 1 | **1,027** | 0,542365 | 12,407 | 11,110 |
| USP45 | ubiquitin specific peptidase 45 | **1,027** | 0,605619 | 6,702 | 5,363 |
| PRR5 | proline rich 5 | **1,027** | 0,684077 | 2,493 | 1,915 |
| RHOC | ras homolog family member C | **1,027** | 0,35213 | 179,353 | 153,367 |
| BMPR1A | bone morphogenetic protein receptor type 1A | **1,027** | 0,538528 | 15,232 | 14,302 |
| KPNA5 | karyopherin subunit alpha 5 | **1,027** | 0,606297 | 9,306 | 10,663 |
| C6orf120 | chromosome 6 open reading frame 120 | **1,026** | 0,370961 | 36,391 | 35,692 |
| SEZ6L | seizure related 6 homolog like | **1,026** | 0,814392 | 0,332 | 1,085 |
| IPO9 | importin 9 | **1,026** | 0,339527 | 42,207 | 51,335 |
| KIAA0556 | KIAA0556 | **1,026** | 0,464822 | 24,150 | 24,199 |
| ID2-AS1 | ID2 antisense RNA 1 (head to head) | **1,026** | 0,755039 | 2,160 | 3,831 |
| RCSD1 | RCSD domain containing 1 | **1,026** | 0,723515 | 2,105 | 2,554 |
| EDC4 | enhancer of mRNA decapping 4 | **1,026** | 0,509518 | 18,279 | 19,921 |
| MAP6D1 | MAP6 domain containing 1 | **1,026** | 0,886374 | 0,332 | 0,383 |
| LRTOMT | leucine rich transmembrane and O-methyltransferase domain containing | **1,026** | 0,688735 | 3,933 | 4,214 |
| MUT | methylmalonyl-CoA mutase | **1,026** | 0,422974 | 29,191 | 26,370 |
| MFSD14C | major facilitator superfamily domain containing 14C | **1,026** | 0,55913 | 9,638 | 8,492 |
| NECAP2 | NECAP endocytosis associated 2 | **1,026** | 0,354504 | 52,454 | 49,611 |
| ZNF667 | zinc finger protein 667 | **1,026** | 0,651322 | 4,154 | 5,619 |
| EIF2AK2 | eukaryotic translation initiation factor 2 alpha kinase 2 | **1,026** | 0,474813 | 28,969 | 26,881 |
| ADCY5 | adenylate cyclase 5 | **1,026** | 0,80438 | 1,551 | 2,299 |
| KLHL42 | kelch like family member 42 | **1,026** | 0,501035 | 22,655 | 18,453 |
| AKR7A2 | aldo-keto reductase family 7 member A2 | **1,026** | 0,506538 | 22,710 | 28,477 |
| APLP2 | amyloid beta precursor like protein 2 | **1,026** | 0,33062 | 795,957 | 753,173 |
| TMEM41A | transmembrane protein 41A | **1,026** | 0,546814 | 17,005 | 16,409 |
| NPLOC4 | NPL4 homolog, ubiquitin recognition factor | **1,026** | 0,313276 | 107,623 | 97,307 |
| NKAPP1 | NFKB activating protein pseudogene 1 | **1,026** | 0,878157 | 0,609 | 0,702 |
| COQ10A | coenzyme Q10A | **1,026** | 0,61142 | 6,259 | 7,407 |
| MTMR4 | myotubularin related protein 4 | **1,026** | 0,514754 | 16,949 | 19,410 |
| REXO5 | RNA exonuclease 5 | **1,026** | 0,841894 | 1,219 | 0,702 |

| | | | | | |
|---|---|---|---|---|---|
| EEA1 | early endosome antigen 1 | **1,026** | 0,562658 | 28,637 | 26,817 |
| PDE6D | phosphodiesterase 6D | **1,026** | 0,453715 | 18,722 | 16,026 |
| MRFAP1L1 | Morf4 family associated protein 1 like 1 | **1,026** | 0,331763 | 68,518 | 78,408 |
| WNT5A | Wnt family member 5A | **1,026** | 0,430764 | 86,741 | 53,698 |
| SLC27A4 | solute carrier family 27 member 4 | **1,026** | 0,386242 | 33,733 | 38,629 |
| FPGT | fucose-1-phosphate guanylyltransferase | **1,026** | 0,578344 | 7,976 | 7,598 |
| SPIN1 | spindlin 1 | **1,026** | 0,422687 | 55,002 | 53,634 |
| MIA3 | MIA family member 3, ER export factor | **1,026** | 0,373707 | 78,488 | 88,432 |
| G2E3 | G2/M-phase specific E3 ubiquitin protein ligase | **1,026** | 0,649499 | 8,862 | 8,939 |
| ZNF592 | zinc finger protein 592 | **1,026** | 0,405877 | 39,493 | 46,036 |
| CNIH4 | cornichon family AMPA receptor auxiliary protein 4 | **1,026** | 0,45832 | 28,526 | 28,349 |
| RIPOR1 | RHO family interacting cell polarization regulator 1 | **1,026** | 0,474329 | 42,263 | 37,352 |
| CCNF | cyclin F | **1,026** | 0,672923 | 5,151 | 5,363 |
| TMEM192 | transmembrane protein 192 | **1,026** | 0,549288 | 15,398 | 16,346 |
| BRF2 | BRF2, RNA polymerase III transcription initiation factor subunit | **1,026** | 0,435337 | 32,846 | 39,651 |
| PIP5K1C | phosphatidylinositol-4-phosphate 5-kinase type 1 gamma | **1,026** | 0,415207 | 41,155 | 36,905 |
| ZNF16 | zinc finger protein 16 | **1,026** | 0,632848 | 6,425 | 7,598 |
| ADAM15 | ADAM metallopeptidase domain 15 | **1,026** | 0,440034 | 101,530 | 89,071 |
| SPRYD7 | SPRY domain containing 7 | **1,026** | 0,574825 | 12,407 | 13,089 |
| ROBO1 | roundabout guidance receptor 1 | **1,026** | 0,497917 | 73,337 | 84,154 |
| TTC9C | tetratricopeptide repeat domain 9C | **1,026** | 0,462876 | 11,909 | 10,663 |
| GUSB | glucuronidase beta | **1,026** | 0,320476 | 49,519 | 40,162 |
| PDZD8 | PDZ domain containing 8 | **1,026** | 0,443181 | 56,609 | 55,422 |
| POLA2 | DNA polymerase alpha 2, accessory subunit | **1,026** | 0,558056 | 9,472 | 12,451 |
| AGAP6 | ArfGAP with GTPase domain, ankyrin repeat and PH domain 6 | **1,026** | 0,750214 | 2,271 | 3,384 |
| ANP32E | acidic nuclear phosphoprotein 32 family member E | **1,026** | 0,338266 | 54,061 | 57,018 |
| KLHL15 | kelch like family member 15 | **1,026** | 0,36937 | 31,351 | 36,841 |
| TERF1 | telomeric repeat binding factor 1 | **1,026** | 0,460243 | 20,937 | 24,071 |
| RUSC1-AS1 | RUSC1 antisense RNA 1 | **1,026** | 0,859986 | 1,274 | 1,085 |
| COL6A2 | collagen type VI alpha 2 chain | **1,026** | 0,435955 | 2776,266 | 2642,619 |
| C1QBP | complement C1q binding protein | **1,026** | 0,366729 | 61,262 | 65,638 |
| GCLC | glutamate-cysteine ligase catalytic subunit | **1,025** | 0,454518 | 35,173 | 27,072 |
| AAED1 | AhpC/TSA antioxidant enzyme domain containing 1 | **1,025** | 0,575135 | 9,527 | 7,726 |

| | | | | | |
|---|---|---|---|---|---|
| ADAMTS2 | ADAM metallopeptidase with thrombospondin type 1 motif 2 | **1,025** | 0,628349 | 17,503 | 7,279 |
| GLS | glutaminase | **1,025** | 0,366592 | 109,008 | 82,941 |
| ZNF740 | zinc finger protein 740 | **1,025** | 0,396616 | 24,759 | 27,902 |
| CLN6 | ceroid-lipofuscinosis, neuronal 6, late infantile, variant | **1,025** | 0,597172 | 7,533 | 6,896 |
| FAM193B | family with sequence similarity 193 member B | **1,025** | 0,443042 | 20,882 | 24,391 |
| FLJ37035 | uncharacterized LOC399821 | **1,025** | 0,871283 | 0,443 | 0,511 |
| UBE2Q2 | ubiquitin conjugating enzyme E2 Q2 | **1,025** | 0,420651 | 45,752 | 30,648 |
| BPGM | bisphosphoglycerate mutase | **1,025** | 0,38948 | 55,335 | 43,354 |
| NPIPB2 | nuclear pore complex interacting protein family, member B2 | **1,025** | 0,71065 | 3,434 | 6,321 |
| EZR | ezrin | **1,025** | 0,317378 | 63,311 | 56,763 |
| PCNX1 | pecanex homolog 1 (Drosophila) | **1,025** | 0,515053 | 34,120 | 32,500 |
| GTSE1 | G2 and S-phase expressed 1 | **1,025** | 0,835559 | 4,099 | 0,830 |
| FAM19A5 | family with sequence similarity 19 member A5, C-C motif chemokine like | **1,025** | 0,869526 | 0,720 | 0,383 |
| NADSYN1 | NAD synthetase 1 | **1,025** | 0,431205 | 19,110 | 16,154 |
| TUBA1A | tubulin alpha 1a | **1,025** | 0,606673 | 27,363 | 19,985 |
| ADNP2 | ADNP homeobox 2 | **1,025** | 0,484456 | 18,279 | 18,644 |
| PPP1R12B | protein phosphatase 1 regulatory subunit 12B | **1,025** | 0,579977 | 9,859 | 13,153 |
| NT5C3A | 5'-nucleotidase, cytosolic IIIA | **1,025** | 0,524713 | 9,582 | 10,791 |
| PCNT | pericentrin | **1,025** | 0,595317 | 25,701 | 24,391 |
| PEX11A | peroxisomal biogenesis factor 11 alpha | **1,025** | 0,618392 | 5,816 | 7,534 |
| MTERF4 | mitochondrial transcription termination factor 4 | **1,025** | 0,401184 | 19,387 | 22,667 |
| LUC7L3 | LUC7 like 3 pre-mRNA splicing factor | **1,025** | 0,494693 | 63,699 | 69,724 |
| ZNF613 | zinc finger protein 613 | **1,025** | 0,63132 | 5,484 | 5,236 |
| PRKAR1A | protein kinase cAMP-dependent type I regulatory subunit alpha | **1,025** | 0,394833 | 205,331 | 196,721 |
| FLNA | filamin A | **1,025** | 0,488496 | 1549,207 | 1284,596 |
| WFDC3 | WAP four-disulfide core domain 3 | **1,025** | 0,7393 | 1,551 | 3,831 |
| SQOR | sulfide quinone oxidoreductase | **1,025** | 0,615338 | 29,468 | 24,071 |
| UNC13B | unc-13 homolog B | **1,025** | 0,547417 | 34,730 | 32,819 |
| RPGR | retinitis pigmentosa GTPase regulator | **1,025** | 0,549723 | 9,915 | 8,939 |
| PIGA | phosphatidylinositol glycan anchor biosynthesis class A | **1,025** | 0,647296 | 14,235 | 12,770 |
| TMEM136 | transmembrane protein 136 | **1,025** | 0,558054 | 10,856 | 11,365 |
| RAC1 | ras-related C3 botulinum toxin substrate 1 (rho family, small GTP binding protein Rac1) | **1,025** | 0,436472 | 171,266 | 161,412 |
| RLIM | ring finger protein, LIM domain interacting | **1,024** | 0,476469 | 68,462 | 70,682 |

| | | | | | |
|---|---|---|---|---|---|
| PPP1R3B | protein phosphatase 1 regulatory subunit 3B | **1,024** | 0,893677 | 0,388 | 0,447 |
| SPTAN1 | spectrin alpha, non-erythrocytic 1 | **1,024** | 0,52027 | 178,744 | 158,412 |
| MTHFD2 | methylenetetrahydrofolate dehydrogenase (NADP+ dependent) 2, methenyltetrahydrofol | **1,024** | 0,395312 | 202,506 | 182,738 |
| ITM2B | integral membrane protein 2B | **1,024** | 0,330479 | 458,797 | 389,612 |
| DYNC2H1 | dynein cytoplasmic 2 heavy chain 1 | **1,024** | 0,668256 | 32,237 | 33,457 |
| FUCA1 | fucosidase, alpha-L- 1, tissue | **1,024** | 0,420184 | 23,375 | 20,432 |
| WBP1L | WW domain binding protein 1 like | **1,024** | 0,346697 | 68,351 | 51,910 |
| HAS2-AS1 | HAS2 antisense RNA 1 | **1,024** | 0,867519 | 1,329 | 1,022 |
| CLTA | clathrin light chain A | **1,024** | 0,439434 | 91,892 | 79,110 |
| TBC1D5 | TBC1 domain family member 5 | **1,024** | 0,567802 | 37,222 | 29,882 |
| MCM4 | minichromosome maintenance complex component 4 | **1,024** | 0,420332 | 21,491 | 18,389 |
| RIPK1 | receptor interacting serine/threonine kinase 1 | **1,024** | 0,368158 | 40,767 | 35,947 |
| PIGL | phosphatidylinositol glycan anchor biosynthesis class L | **1,024** | 0,625996 | 5,317 | 8,109 |
| PRDM4 | PR/SET domain 4 | **1,024** | 0,377453 | 45,641 | 42,971 |
| SGMS1-AS1 | SGMS1 antisense RNA 1 | **1,024** | 0,71563 | 3,988 | 6,704 |
| LAMTOR5-AS1 | LAMTOR5 antisense RNA 1 | **1,024** | 0,79719 | 0,886 | 1,277 |
| CTTNBP2 | cortactin binding protein 2 | **1,024** | 0,811207 | 1,496 | 1,660 |
| MAVS | mitochondrial antiviral signaling protein | **1,024** | 0,463957 | 76,328 | 63,020 |
| LINC02280 | long intergenic non-protein coding RNA 2280 | **1,024** | 0,874938 | 0,388 | 0,830 |
| GBAP1 | glucosylceramidase beta pseudogene 1 | **1,024** | 0,882993 | 0,332 | 0,319 |
| ATP6AP1L | ATPase H+ transporting accessory protein 1 like | **1,024** | 0,738394 | 2,049 | 3,065 |
| HOOK2 | hook microtubule tethering protein 2 | **1,024** | 0,597652 | 9,416 | 9,067 |
| RBMS1 | RNA binding motif single stranded interacting protein 1 | **1,024** | 0,402933 | 63,754 | 75,471 |
| GCDH | glutaryl-CoA dehydrogenase | **1,024** | 0,569701 | 19,885 | 24,327 |
| NPEPL1 | aminopeptidase-like 1 | **1,024** | 0,828349 | 1,662 | 0,958 |
| EPM2AIP1 | EPM2A interacting protein 1 | **1,024** | 0,514326 | 34,397 | 40,864 |
| STK39 | serine/threonine kinase 39 | **1,024** | 0,516021 | 20,107 | 15,516 |
| GFPT2 | glutamine-fructose-6-phosphate transaminase 2 | **1,024** | 0,386347 | 393,215 | 431,561 |
| C2orf68 | chromosome 2 open reading frame 68 | **1,024** | 0,470689 | 20,162 | 17,750 |
| TIGD7 | tigger transposable element derived 7 | **1,024** | 0,682561 | 7,921 | 6,385 |
| FCF1 | FCF1 rRNA-processing protein | **1,024** | 0,416737 | 51,070 | 59,061 |
| MARK2 | microtubule affinity regulating kinase 2 | **1,024** | 0,397578 | 32,126 | 32,500 |
| FBXW7 | F-box and WD repeat domain containing 7 | **1,024** | 0,582884 | 12,352 | 14,239 |

| | | | | | |
|---|---|---|---|---|---|
| WDR66 | WD repeat domain 66 | **1,024** | 0,634927 | 5,317 | 7,662 |
| NPPA-AS1 | NPPA antisense RNA 1 | **1,024** | 0,878791 | 0,554 | 1,341 |
| SRPX | sushi repeat containing protein, X-linked | **1,024** | 0,819208 | 1,551 | 0,575 |
| EPAS1 | endothelial PAS domain protein 1 | **1,023** | 0,410033 | 663,962 | 680,512 |
| FAHD2CP | fumarylacetoacetate hydrolase domain containing 2C, pseudogene | **1,023** | 0,768877 | 1,828 | 2,809 |
| FMN1 | formin 1 | **1,023** | 0,767242 | 4,874 | 7,726 |
| PRRC2B | proline rich coiled-coil 2B | **1,023** | 0,519988 | 149,830 | 142,194 |
| TASP1 | taspase 1 | **1,023** | 0,710918 | 4,043 | 4,661 |
| RAD23B | RAD23 homolog B, nucleotide excision repair protein | **1,023** | 0,388301 | 134,321 | 132,808 |
| GPAM | glycerol-3-phosphate acyltransferase, mitochondrial | **1,023** | 0,614664 | 9,804 | 10,408 |
| RNF219 | ring finger protein 219 | **1,023** | 0,611888 | 10,413 | 10,982 |
| SPICE1 | spindle and centriole associated protein 1 | **1,023** | 0,647471 | 5,428 | 7,470 |
| ZNF519 | zinc finger protein 519 | **1,023** | 0,774306 | 1,606 | 1,532 |
| RALA | RAS like proto-oncogene A | **1,023** | 0,512203 | 13,238 | 12,515 |
| STAM-AS1 | STAM antisense RNA 1 (head to head) | **1,023** | 0,893371 | 0,166 | 0,511 |
| SEMA3B | semaphorin 3B | **1,023** | 0,612439 | 7,478 | 10,152 |
| KRT8P12 | keratin 8 pseudogene 12 | **1,023** | 0,71373 | 4,597 | 5,683 |
| PDCD7 | programmed cell death 7 | **1,023** | 0,43547 | 18,002 | 18,900 |
| GRPEL1 | GrpE like 1, mitochondrial | **1,023** | 0,395673 | 40,989 | 47,121 |
| DAGLB | diacylglycerol lipase beta | **1,023** | 0,491807 | 15,952 | 17,686 |
| DIAPH1 | diaphanous related formin 1 | **1,023** | 0,402583 | 110,725 | 126,550 |
| PCDHGB1 | protocadherin gamma subfamily B, 1 | **1,023** | 0,874746 | 0,388 | 0,958 |
| HKR1 | HKR1, GLI-Kruppel zinc finger family member | **1,023** | 0,591202 | 10,580 | 11,876 |
| THOC2 | THO complex 2 | **1,023** | 0,375193 | 41,155 | 45,461 |
| COMMD10 | COMM domain containing 10 | **1,023** | 0,645551 | 8,309 | 7,726 |
| PPP2R2A | protein phosphatase 2 regulatory subunit Balpha | **1,023** | 0,402934 | 46,472 | 47,377 |
| TPR | translocated promoter region, nuclear basket protein | **1,023** | 0,557302 | 103,967 | 113,014 |
| SGK494 | uncharacterized serine/threonine-protein kinase SgK494 | **1,023** | 0,756938 | 2,105 | 2,618 |
| GLUL | glutamate-ammonia ligase | **1,023** | 0,428843 | 95,603 | 78,982 |
| SLC35E3 | solute carrier family 35 member E3 | **1,023** | 0,476328 | 33,733 | 35,884 |
| RNF208 | ring finger protein 208 | **1,023** | 0,851669 | 1,606 | 1,022 |
| GMCL1 | germ cell-less, spermatogenesis associated 1 | **1,023** | 0,46144 | 31,794 | 32,436 |
| MAD2L1 | mitotic arrest deficient 2 like 1 | **1,023** | 0,756006 | 3,656 | 2,618 |

| | | | | | |
|---|---|---|---|---|---|
| SRGAP2 | SLIT-ROBO Rho GTPase activating protein 2 | **1,023** | 0,451108 | 22,987 | 18,900 |
| MUC1 | mucin 1, cell surface associated | **1,023** | 0,658732 | 11,355 | 5,236 |
| TMEM9B | TMEM9 domain family member B | **1,023** | 0,443642 | 35,117 | 35,245 |
| MPHOSPH9 | M-phase phosphoprotein 9 | **1,023** | 0,6664 | 12,684 | 13,536 |
| ZSCAN16-AS1 | ZSCAN16 antisense RNA 1 | **1,023** | 0,742188 | 2,659 | 2,618 |
| PREPL | prolyl endopeptidase-like | **1,023** | 0,423097 | 84,193 | 113,014 |
| ACSF2 | acyl-CoA synthetase family member 2 | **1,023** | 0,803521 | 2,880 | 1,915 |
| CBR3-AS1 | CBR3 antisense RNA 1 | **1,023** | 0,685897 | 3,877 | 4,533 |
| RPLP1 | ribosomal protein lateral stalk subunit P1 | **1,023** | 0,523686 | 597,161 | 635,626 |
| TMEM126B | transmembrane protein 126B | **1,023** | 0,613773 | 12,851 | 13,664 |
| PPIG | peptidylprolyl isomerase G | **1,023** | 0,509128 | 50,239 | 56,124 |
| ORC3 | origin recognition complex subunit 3 | **1,023** | 0,477555 | 27,861 | 33,393 |
| PLEKHM2 | pleckstrin homology and RUN domain containing M2 | **1,023** | 0,385592 | 99,924 | 91,433 |
| ETFDH | electron transfer flavoprotein dehydrogenase | **1,023** | 0,576507 | 16,008 | 15,132 |
| LINC01089 | long intergenic non-protein coding RNA 1089 | **1,023** | 0,784957 | 2,603 | 3,895 |
| HGH1 | HGH1 homolog | **1,023** | 0,595805 | 11,410 | 13,345 |
| GPATCH8 | G-patch domain containing 8 | **1,023** | 0,571897 | 54,559 | 57,720 |
| ARFIP1 | ADP ribosylation factor interacting protein 1 | **1,023** | 0,402518 | 34,231 | 38,565 |
| PPP3R1 | protein phosphatase 3 regulatory subunit B, alpha | **1,023** | 0,486177 | 51,402 | 52,293 |
| NUP98 | nucleoporin 98 | **1,023** | 0,468283 | 82,144 | 92,838 |
| HMGN2 | high mobility group nucleosomal binding domain 2 | **1,023** | 0,426334 | 68,241 | 61,807 |
| USP30 | ubiquitin specific peptidase 30 | **1,023** | 0,65267 | 9,582 | 9,003 |
| WDR37 | WD repeat domain 37 | **1,023** | 0,52541 | 17,005 | 15,771 |
| FBF1 | Fas binding factor 1 | **1,023** | 0,77125 | 1,772 | 2,362 |
| GINS1 | GINS complex subunit 1 | **1,023** | 0,878258 | 1,274 | 0,638 |
| RPS29 | ribosomal protein S29 | **1,023** | 0,500811 | 85,689 | 80,195 |
| FAM13B | family with sequence similarity 13 member B | **1,023** | 0,526994 | 39,549 | 34,862 |
| CLK3 | CDC like kinase 3 | **1,023** | 0,428011 | 34,287 | 43,226 |
| MASTL | microtubule associated serine/threonine kinase like | **1,023** | 0,565869 | 17,725 | 18,261 |
| VEZF1 | vascular endothelial zinc finger 1 | **1,023** | 0,480582 | 49,851 | 51,910 |
| DPP7 | dipeptidyl peptidase 7 | **1,023** | 0,473412 | 64,197 | 41,758 |
| SARS | seryl-tRNA synthetase | **1,023** | 0,403127 | 272,409 | 310,630 |
| DCAF8 | DDB1 and CUL4 associated factor 8 | **1,023** | 0,504822 | 25,923 | 29,052 |

| | | | | | |
|---|---|---|---|---|---|
| AKAP10 | A-kinase anchoring protein 10 | **1,023** | 0,445657 | 27,086 | 29,562 |
| DEF8 | differentially expressed in FDCP 8 homolog | **1,023** | 0,402155 | 32,957 | 29,371 |
| PPP4R1L | protein phosphatase 4 regulatory subunit 1 like (pseudogene) | **1,022** | 0,70293 | 5,096 | 5,427 |
| FAM104A | family with sequence similarity 104 member A | **1,022** | 0,399364 | 31,572 | 32,883 |
| ATXN7L1 | ataxin 7 like 1 | **1,022** | 0,710214 | 5,428 | 5,427 |
| DCLRE1A | DNA cross-link repair 1A | **1,022** | 0,614088 | 10,469 | 12,195 |
| TMEM53 | transmembrane protein 53 | **1,022** | 0,727618 | 5,207 | 5,427 |
| TREML3P | triggering receptor expressed on myeloid cells like 3, pseudogene | **1,022** | 0,75844 | 5,262 | 3,129 |
| FBXO22 | F-box protein 22 | **1,022** | 0,545601 | 17,448 | 24,199 |
| SUPT16H | SPT16 homolog, facilitates chromatin remodeling subunit | **1,022** | 0,429749 | 82,033 | 82,686 |
| FGFR1 | fibroblast growth factor receptor 1 | **1,022** | 0,39022 | 260,943 | 248,759 |
| MYO15B | myosin XVB | **1,022** | 0,66184 | 9,693 | 16,090 |
| TAF1A-AS1 | TAF1A antisense RNA 1 | **1,022** | 0,831271 | 0,665 | 1,596 |
| EXOC6B | exocyst complex component 6B | **1,022** | 0,56999 | 41,764 | 36,139 |
| CATSPERE | catsper channel auxiliary subunit epsilon | **1,022** | 0,882369 | 1,052 | 1,022 |
| TIMELESS | timeless circadian clock | **1,022** | 0,596449 | 14,401 | 13,217 |
| PDRG1 | p53 and DNA damage regulated 1 | **1,022** | 0,493736 | 17,116 | 23,369 |
| ZNF227 | zinc finger protein 227 | **1,022** | 0,560932 | 10,635 | 12,131 |
| DNAJA4 | DnaJ heat shock protein family (Hsp40) member A4 | **1,022** | 0,612476 | 5,428 | 9,067 |
| IFNAR2 | interferon alpha and beta receptor subunit 2 | **1,022** | 0,439486 | 36,336 | 25,157 |
| PPP6C | protein phosphatase 6 catalytic subunit | **1,022** | 0,495333 | 51,014 | 50,314 |
| RAB2B | RAB2B, member RAS oncogene family | **1,022** | 0,542064 | 22,544 | 20,560 |
| BIRC2 | baculoviral IAP repeat containing 2 | **1,022** | 0,409506 | 209,818 | 169,138 |
| SIAH2 | siah E3 ubiquitin protein ligase 2 | **1,022** | 0,43463 | 41,155 | 37,224 |
| DHX34 | DExH-box helicase 34 | **1,022** | 0,597336 | 19,497 | 22,667 |
| MCC | mutated in colorectal cancers | **1,022** | 0,568789 | 46,860 | 31,861 |
| FGF18 | fibroblast growth factor 18 | **1,022** | 0,618988 | 27,474 | 25,157 |
| NUDT7 | nudix hydrolase 7 | **1,022** | 0,829678 | 1,385 | 1,915 |
| SFT2D3 | SFT2 domain containing 3 | **1,022** | 0,58948 | 10,413 | 10,854 |
| PLP2 | proteolipid protein 2 | **1,022** | 0,368092 | 114,270 | 74,385 |
| MED1 | mediator complex subunit 1 | **1,022** | 0,706849 | 37,998 | 39,076 |
| FRAT1 | FRAT1, WNT signaling pathway regulator | **1,022** | 0,828176 | 2,105 | 1,979 |
| MDM4 | MDM4, p53 regulator | **1,022** | 0,504239 | 20,771 | 22,220 |

| | | | | | |
|---|---|---|---|---|---|
| CKS1B | CDC28 protein kinase regulatory subunit 1B | **1,022** | 0,726945 | 5,539 | 5,363 |
| TANGO2 | transport and golgi organization 2 homolog | **1,022** | 0,479463 | 20,771 | 18,708 |
| ZNF384 | zinc finger protein 384 | **1,022** | 0,46762 | 42,650 | 45,333 |
| C19orf66 | chromosome 19 open reading frame 66 | **1,022** | 0,663868 | 10,247 | 10,280 |
| MRPL44 | mitochondrial ribosomal protein L44 | **1,022** | 0,548054 | 21,214 | 22,220 |
| TRAF3IP3 | TRAF3 interacting protein 3 | **1,022** | 0,889549 | 1,329 | 0,958 |
| NPHP4 | nephrocystin 4 | **1,022** | 0,545202 | 13,183 | 18,133 |
| TMEM164 | transmembrane protein 164 | **1,022** | 0,606097 | 16,174 | 14,430 |
| RFC5 | replication factor C subunit 5 | **1,022** | 0,646695 | 6,536 | 6,513 |
| C5orf42 | chromosome 5 open reading frame 42 | **1,022** | 0,770689 | 11,189 | 8,684 |
| ZNF512 | zinc finger protein 512 | **1,022** | 0,657862 | 8,198 | 8,045 |
| SRFBP1 | serum response factor binding protein 1 | **1,022** | 0,588657 | 9,915 | 12,962 |
| SOX11 | SRY-box 11 | **1,022** | 0,722746 | 7,145 | 4,853 |
| TMEM199 | transmembrane protein 199 | **1,022** | 0,571737 | 17,337 | 20,560 |
| FXR2 | FMR1 autosomal homolog 2 | **1,022** | 0,42961 | 65,637 | 63,339 |
| KIAA0391 | KIAA0391 | **1,022** | 0,836405 | 1,828 | 3,448 |
| JUND | JunD proto-oncogene, AP-1 transcription factor subunit | **1,022** | 0,510501 | 95,382 | 106,182 |
| C19orf12 | chromosome 19 open reading frame 12 | **1,022** | 0,535581 | 21,768 | 19,155 |
| TGDS | TDP-glucose 4,6-dehydratase | **1,022** | 0,556638 | 14,734 | 15,005 |
| TTC21A | tetratricopeptide repeat domain 21A | **1,022** | 0,792061 | 2,770 | 1,852 |
| ZNF394 | zinc finger protein 394 | **1,022** | 0,585176 | 17,116 | 18,325 |
| PURA | purine rich element binding protein A | **1,021** | 0,420861 | 67,133 | 65,957 |
| PCDHGA9 | protocadherin gamma subfamily A, 9 | **1,021** | 0,840251 | 1,772 | 2,235 |
| MAN2A1 | mannosidase alpha class 2A member 1 | **1,021** | 0,468496 | 55,833 | 60,402 |
| KDM4B | lysine demethylase 4B | **1,021** | 0,475705 | 29,689 | 32,819 |
| SP3 | Sp3 transcription factor | **1,021** | 0,534149 | 65,748 | 62,701 |
| HS2ST1 | heparan sulfate 2-O-sulfotransferase 1 | **1,021** | 0,477227 | 20,550 | 20,815 |
| SLC9A9 | solute carrier family 9 member A9 | **1,021** | 0,593379 | 9,582 | 10,663 |
| ZNF34 | zinc finger protein 34 | **1,021** | 0,775456 | 2,382 | 1,852 |
| EMC7 | ER membrane protein complex subunit 7 | **1,021** | 0,442703 | 87,849 | 76,684 |
| PITHD1 | PITH domain containing 1 | **1,021** | 0,501097 | 28,637 | 33,138 |
| PAIP2 | poly(A) binding protein interacting protein 2 | **1,021** | 0,41041 | 64,862 | 71,320 |
| LIG1 | DNA ligase 1 | **1,021** | 0,713012 | 5,096 | 5,427 |

| | | | | | |
|---|---|---|---|---|---|
| SELENOI | selenoprotein I | **1,021** | 0,538991 | 41,321 | 39,970 |
| SPATA20 | spermatogenesis associated 20 | **1,021** | 0,570642 | 19,331 | 17,623 |
| NBEAL2 | neurobeachin like 2 | **1,021** | 0,711746 | 4,487 | 7,151 |
| RAVER2 | ribonucleoprotein, PTB binding 2 | **1,021** | 0,787808 | 2,105 | 2,618 |
| NBEA | neurobeachin | **1,021** | 0,712096 | 9,139 | 13,855 |
| PPIL6 | peptidylprolyl isomerase like 6 | **1,021** | 0,82518 | 2,326 | 1,469 |
| PDK3 | pyruvate dehydrogenase kinase 3 | **1,021** | 0,667144 | 7,478 | 5,619 |
| RAB6A | RAB6A, member RAS oncogene family | **1,021** | 0,444207 | 124,794 | 113,589 |
| TMEM147-AS1 | TMEM147 antisense RNA 1 | **1,021** | 0,737068 | 3,767 | 5,619 |
| ATP6AP1 | ATPase H+ transporting accessory protein 1 | **1,021** | 0,44963 | 133,823 | 98,393 |
| SLMAP | sarcolemma associated protein | **1,021** | 0,52598 | 42,041 | 35,373 |
| PSIP1 | PC4 and SFRS1 interacting protein 1 | **1,021** | 0,473632 | 38,662 | 44,567 |
| MRPL46 | mitochondrial ribosomal protein L46 | **1,021** | 0,782777 | 2,382 | 3,639 |
| CCNT1 | cyclin T1 | **1,021** | 0,586454 | 38,552 | 40,481 |
| MCTP1 | multiple C2 and transmembrane domain containing 1 | **1,021** | 0,907745 | 0,443 | 0,511 |
| TPRKB | TP53RK binding protein | **1,021** | 0,579193 | 13,626 | 18,006 |
| HMG20A | high mobility group 20A | **1,021** | 0,525099 | 27,917 | 31,095 |
| GLT8D2 | glycosyltransferase 8 domain containing 2 | **1,021** | 0,618501 | 11,909 | 11,365 |
| CHPT1 | choline phosphotransferase 1 | **1,021** | 0,500483 | 35,616 | 29,818 |
| PCDHGB4 | protocadherin gamma subfamily B, 4 | **1,021** | 0,75562 | 6,148 | 3,767 |
| FAAP24 | Fanconi anemia core complex associated protein 24 | **1,021** | 0,771423 | 2,880 | 3,001 |
| MARK3 | microtubule affinity regulating kinase 3 | **1,021** | 0,451582 | 53,784 | 58,040 |
| MBD5 | methyl-CpG binding domain protein 5 | **1,021** | 0,633052 | 12,020 | 12,515 |
| AMOT | angiomotin | **1,021** | 0,781822 | 4,431 | 4,916 |
| ING4 | inhibitor of growth family member 4 | **1,021** | 0,551391 | 12,241 | 12,578 |
| DOK5 | docking protein 5 | **1,021** | 0,74452 | 4,265 | 4,278 |
| ANKRD17 | ankyrin repeat domain 17 | **1,021** | 0,577504 | 79,928 | 86,261 |
| FANCM | Fanconi anemia complementation group M | **1,021** | 0,723945 | 4,819 | 4,661 |
| PPCS | phosphopantothenoylcysteine synthetase | **1,021** | 0,536382 | 20,162 | 22,667 |
| ARNT | aryl hydrocarbon receptor nuclear translocator | **1,021** | 0,536356 | 32,569 | 29,116 |
| ESRRA | estrogen related receptor alpha | **1,021** | 0,60859 | 22,599 | 20,624 |
| GTPBP2 | GTP binding protein 2 | **1,021** | 0,475416 | 101,973 | 114,419 |
| RNASEL | ribonuclease L | **1,021** | 0,645172 | 10,358 | 9,833 |

| | | | | | |
|---|---|---|---|---|---|
| CECR7 | cat eye syndrome chromosome region, candidate 7 (non-protein coding) | **1,021** | 0,881683 | 1,163 | 0,447 |
| STEAP1 | STEAP family member 1 | **1,021** | 0,514549 | 59,877 | 51,080 |
| RNF139 | ring finger protein 139 | **1,021** | 0,501413 | 36,834 | 39,715 |
| RPL3 | ribosomal protein L3 | **1,021** | 0,498864 | 550,024 | 493,815 |
| LEPROT | leptin receptor overlapping transcript | **1,021** | 0,458948 | 133,269 | 117,803 |
| TFIP11 | tuftelin interacting protein 11 | **1,021** | 0,506386 | 50,904 | 51,016 |
| SENP2 | SUMO1/sentrin/SMT3 specific peptidase 2 | **1,020** | 0,499967 | 35,727 | 36,331 |
| SETD6 | SET domain containing 6 | **1,020** | 0,64903 | 6,924 | 8,045 |
| RTN3 | reticulon 3 | **1,020** | 0,42517 | 137,589 | 112,887 |
| BGN | biglycan | **1,020** | 0,445118 | 923,077 | 572,478 |
| SRGAP2C | SLIT-ROBO Rho GTPase activating protein 2C | **1,020** | 0,75745 | 3,323 | 2,937 |
| UBAP1 | ubiquitin associated protein 1 | **1,020** | 0,424488 | 70,567 | 68,256 |
| DCBLD2 | discoidin, CUB and LCCL domain containing 2 | **1,020** | 0,482467 | 45,254 | 42,396 |
| GGA3 | golgi associated, gamma adaptin ear containing, ARF binding protein 3 | **1,020** | 0,525154 | 28,747 | 28,477 |
| KATNBL1 | katanin regulatory subunit B1 like 1 | **1,020** | 0,611033 | 14,623 | 10,599 |
| TMPPE | transmembrane protein with metallophosphoesterase domain | **1,020** | 0,851923 | 2,049 | 1,277 |
| SLC26A11 | solute carrier family 26 member 11 | **1,020** | 0,54946 | 19,331 | 18,453 |
| NUCB1 | nucleobindin 1 | **1,020** | 0,456827 | 238,953 | 154,453 |
| PPP1R3F | protein phosphatase 1 regulatory subunit 3F | **1,020** | 0,590551 | 13,903 | 14,494 |
| C12orf60 | chromosome 12 open reading frame 60 | **1,020** | 0,801957 | 11,078 | 9,003 |
| ZNF569 | zinc finger protein 569 | **1,020** | 0,714091 | 5,041 | 4,853 |
| TRIP12 | thyroid hormone receptor interactor 12 | **1,020** | 0,565723 | 165,727 | 154,134 |
| GJB2 | gap junction protein beta 2 | **1,020** | 0,601062 | 19,165 | 14,558 |
| CTNND1 | catenin delta 1 | **1,020** | 0,483355 | 111,168 | 104,905 |
| MTMR3 | myotubularin related protein 3 | **1,020** | 0,593521 | 42,983 | 43,163 |
| TBK1 | TANK binding kinase 1 | **1,020** | 0,557701 | 33,677 | 32,308 |
| CPE | carboxypeptidase E | **1,020** | 0,482881 | 133,546 | 94,881 |
| OS9 | OS9, endoplasmic reticulum lectin | **1,020** | 0,47012 | 429,661 | 318,036 |
| PSMB10 | proteasome subunit beta 10 | **1,020** | 0,665735 | 10,690 | 8,875 |
| ZMYM6 | zinc finger MYM-type containing 6 | **1,020** | 0,645275 | 8,198 | 9,194 |
| PTBP1 | polypyrimidine tract binding protein 1 | **1,020** | 0,421575 | 176,362 | 193,657 |
| ZNF277 | zinc finger protein 277 | **1,020** | 0,710891 | 9,084 | 7,854 |
| CRNKL1 | crooked neck pre-mRNA splicing factor 1 | **1,020** | 0,495462 | 29,412 | 31,031 |

| | | | | | |
|---|---|---|---|---|---|
| SRP54-AS1 | SRP54 antisense RNA 1 (head to head) | **1,020** | 0,926562 | 0,277 | 0,702 |
| SNX3 | sorting nexin 3 | **1,020** | 0,471007 | 84,359 | 87,538 |
| CFAP126 | cilia and flagella associated protein 126 | **1,020** | 0,921585 | 0,111 | 0,766 |
| ITGA2 | integrin subunit alpha 2 | **1,020** | 0,804594 | 19,774 | 13,408 |
| TXNDC9 | thioredoxin domain containing 9 | **1,020** | 0,541579 | 35,893 | 39,332 |
| POR | cytochrome p450 oxidoreductase | **1,020** | 0,533895 | 56,055 | 51,399 |
| SLC25A28 | solute carrier family 25 member 28 | **1,020** | 0,455415 | 57,218 | 66,021 |
| CD55 | CD55 molecule (Cromer blood group) | **1,020** | 0,475842 | 1533,754 | 2108,004 |
| ACTR10 | actin-related protein 10 homolog | **1,020** | 0,488487 | 53,064 | 58,103 |
| CYB5R1 | cytochrome b5 reductase 1 | **1,020** | 0,499006 | 44,700 | 43,801 |
| PRKAR1B | protein kinase cAMP-dependent type I regulatory subunit beta | **1,020** | 0,781784 | 3,213 | 2,937 |
| LRR1 | leucine rich repeat protein 1 | **1,020** | 0,762946 | 4,265 | 3,703 |
| CEPT1 | choline/ethanolamine phosphotransferase 1 | **1,020** | 0,512496 | 27,917 | 29,052 |
| ATXN1 | ataxin 1 | **1,020** | 0,610591 | 56,110 | 53,378 |
| ITGA3 | integrin subunit alpha 3 | **1,020** | 0,43306 | 146,950 | 118,697 |
| ZNF329 | zinc finger protein 329 | **1,020** | 0,709139 | 4,597 | 5,108 |
| CXXC5 | CXXC finger protein 5 | **1,020** | 0,537267 | 38,219 | 26,945 |
| C1orf35 | chromosome 1 open reading frame 35 | **1,020** | 0,668241 | 7,533 | 7,790 |
| ZNF252P | zinc finger protein 252, pseudogene | **1,020** | 0,634883 | 19,331 | 20,815 |
| USP39 | ubiquitin specific peptidase 39 | **1,020** | 0,55858 | 22,765 | 23,816 |
| ASPH | aspartate beta-hydroxylase | **1,020** | 0,553331 | 303,649 | 172,139 |
| VAMP3 | vesicle associated membrane protein 3 | **1,019** | 0,477244 | 114,547 | 94,242 |
| AKAP11 | A-kinase anchoring protein 11 | **1,019** | 0,65595 | 62,259 | 62,764 |
| MED23 | mediator complex subunit 23 | **1,019** | 0,576909 | 24,261 | 24,263 |
| CAMKK2 | calcium/calmodulin dependent protein kinase kinase 2 | **1,019** | 0,649193 | 17,725 | 14,813 |
| FNTA | farnesyltransferase, CAAX box, alpha | **1,019** | 0,542436 | 25,369 | 26,370 |
| ASPHD2 | aspartate beta-hydroxylase domain containing 2 | **1,019** | 0,85126 | 1,772 | 1,277 |
| TUBE1 | tubulin epsilon 1 | **1,019** | 0,664034 | 20,882 | 23,369 |
| SLC15A3 | solute carrier family 15 member 3 | **1,019** | 0,657832 | 10,746 | 13,664 |
| USP24 | ubiquitin specific peptidase 24 | **1,019** | 0,595908 | 52,454 | 46,163 |
| NEFH | neurofilament heavy | **1,019** | 0,792908 | 4,431 | 5,746 |
| PAPSS2 | 3'-phosphoadenosine 5'-phosphosulfate synthase 2 | **1,019** | 0,577108 | 124,849 | 119,591 |
| WRN | Werner syndrome RecQ like helicase | **1,019** | 0,668431 | 11,300 | 16,537 |

| | | | | | |
|---|---|---|---|---|---|
| KIAA1217 | KIAA1217 | **1,019** | 0,5836 | 16,229 | 26,562 |
| LIN7C | lin-7 homolog C, crumbs cell polarity complex component | **1,019** | 0,550336 | 27,917 | 30,903 |
| STX6 | syntaxin 6 | **1,019** | 0,525031 | 32,846 | 35,437 |
| MPV17L2 | MPV17 mitochondrial inner membrane protein like 2 | **1,019** | 0,668194 | 18,113 | 14,749 |
| NPTN | neuroplastin | **1,019** | 0,439495 | 229,371 | 200,616 |
| CILP2 | cartilage intermediate layer protein 2 | **1,019** | 0,633671 | 89,234 | 92,965 |
| RBM27 | RNA binding motif protein 27 | **1,019** | 0,644595 | 11,466 | 12,898 |
| SNHG7 | small nucleolar RNA host gene 7 | **1,019** | 0,622054 | 8,918 | 14,047 |
| TSC22D3 | TSC22 domain family member 3 | **1,019** | 0,568675 | 55,778 | 60,338 |
| BNIP2 | BCL2 interacting protein 2 | **1,019** | 0,505826 | 67,521 | 68,064 |
| NT5DC3 | 5'-nucleotidase domain containing 3 | **1,019** | 0,768486 | 8,973 | 6,449 |
| S100PBP | S100P binding protein | **1,019** | 0,573479 | 18,168 | 13,536 |
| TNPO3 | transportin 3 | **1,019** | 0,520019 | 42,761 | 43,929 |
| CCDC144CP | coiled-coil domain containing 144C, pseudogene | **1,019** | 0,898805 | 0,443 | 0,383 |
| KRIT1 | KRIT1, ankyrin repeat containing | **1,019** | 0,627712 | 25,147 | 24,965 |
| TRAF3IP1 | TRAF3 interacting protein 1 | **1,019** | 0,664967 | 16,672 | 13,664 |
| CNRIP1 | cannabinoid receptor interacting protein 1 | **1,019** | 0,560819 | 33,289 | 36,139 |
| STK38 | serine/threonine kinase 38 | **1,019** | 0,550562 | 28,415 | 32,691 |
| RTN1 | reticulon 1 | **1,019** | 0,81968 | 2,548 | 3,959 |
| MBD1 | methyl-CpG binding domain protein 1 | **1,019** | 0,52653 | 51,956 | 57,273 |
| SRP19 | signal recognition particle 19 | **1,019** | 0,646708 | 13,848 | 16,729 |
| BRWD1 | bromodomain and WD repeat domain containing 1 | **1,019** | 0,617652 | 33,899 | 38,310 |
| DENND6B | DENN domain containing 6B | **1,019** | 0,769719 | 4,653 | 5,044 |
| GOLGA7 | golgin A7 | **1,019** | 0,542356 | 46,362 | 43,099 |
| DOK3 | docking protein 3 | **1,019** | 0,859319 | 1,496 | 1,469 |
| LARP4B | La ribonucleoprotein domain family member 4B | **1,019** | 0,515758 | 42,263 | 47,568 |
| ZNF14 | zinc finger protein 14 | **1,019** | 0,709031 | 7,312 | 8,300 |
| CWC22 | CWC22 spliceosome associated protein homolog | **1,019** | 0,624633 | 27,584 | 30,393 |
| C9orf64 | chromosome 9 open reading frame 64 | **1,019** | 0,582606 | 14,512 | 12,642 |
| RDH10 | retinol dehydrogenase 10 | **1,019** | 0,591504 | 33,456 | 16,154 |
| SUPT4H1 | SPT4 homolog, DSIF elongation factor subunit | **1,019** | 0,484219 | 51,457 | 51,527 |
| RAF1 | Raf-1 proto-oncogene, serine/threonine kinase | **1,019** | 0,459008 | 135,540 | 125,976 |
| IQSEC2 | IQ motif and Sec7 domain 2 | **1,019** | 0,647058 | 18,500 | 14,685 |

| | | | | | |
|---|---|---|---|---|---|
| SOX12 | SRY-box 12 | **1,018** | 0,869265 | 3,157 | 0,447 |
| HSPA9 | heat shock protein family A (Hsp70) member 9 | **1,018** | 0,472185 | 368,123 | 422,750 |
| INPP5B | inositol polyphosphate-5-phosphatase B | **1,018** | 0,580087 | 18,556 | 19,410 |
| MYCBPAP | MYCBP associated protein | **1,018** | 0,902445 | 0,886 | 1,149 |
| TRAPPC5 | trafficking protein particle complex 5 | **1,018** | 0,896491 | 0,609 | 0,511 |
| SAT2 | spermidine/spermine N1-acetyltransferase family member 2 | **1,018** | 0,59702 | 21,602 | 20,687 |
| N4BP1 | NEDD4 binding protein 1 | **1,018** | 0,574007 | 28,858 | 25,668 |
| SLC27A1 | solute carrier family 27 member 1 | **1,018** | 0,598656 | 21,214 | 17,176 |
| CBX5 | chromobox 5 | **1,018** | 0,640162 | 216,132 | 226,859 |
| GPBP1L1 | GC-rich promoter binding protein 1 like 1 | **1,018** | 0,512469 | 75,774 | 72,023 |
| TNXB | tenascin XB | **1,018** | 0,876499 | 0,831 | 0,766 |
| UBA6-AS1 | UBA6 antisense RNA 1 (head to head) | **1,018** | 0,626354 | 9,859 | 12,195 |
| DYNLL1 | dynein light chain LC8-type 1 | **1,018** | 0,515401 | 77,712 | 79,174 |
| C12orf66 | chromosome 12 open reading frame 66 | **1,018** | 0,730471 | 4,930 | 6,449 |
| FAM234B | family with sequence similarity 234 member B | **1,018** | 0,736636 | 3,379 | 5,810 |
| EMC3 | ER membrane protein complex subunit 3 | **1,018** | 0,523909 | 79,097 | 65,574 |
| UBXN2B | UBX domain protein 2B | **1,018** | 0,615554 | 19,220 | 19,219 |
| WDR1 | WD repeat domain 1 | **1,018** | 0,479038 | 239,729 | 228,199 |
| TAPBPL | TAP binding protein like | **1,018** | 0,717605 | 15,565 | 11,365 |
| TNFRSF1B | TNF receptor superfamily member 1B | **1,018** | 0,493224 | 193,921 | 245,822 |
| NLK | nemo like kinase | **1,018** | 0,723779 | 9,804 | 10,024 |
| KLHL12 | kelch like family member 12 | **1,018** | 0,575172 | 26,200 | 27,455 |
| NUP50 | nucleoporin 50 | **1,018** | 0,556533 | 32,348 | 28,349 |
| SLC37A2 | solute carrier family 37 member 2 | **1,018** | 0,635614 | 6,093 | 22,284 |
| UCHL5 | ubiquitin C-terminal hydrolase L5 | **1,018** | 0,592208 | 13,238 | 17,176 |
| TAB3 | TGF-beta activated kinase 1 and MAP3K7 binding protein 3 | **1,018** | 0,703442 | 31,517 | 28,605 |
| ZNF664 | zinc finger protein 664 | **1,018** | 0,544391 | 70,290 | 64,552 |
| CLTC | clathrin heavy chain | **1,018** | 0,52646 | 417,476 | 362,348 |
| TSNARE1 | t-SNARE domain containing 1 | **1,018** | 0,738692 | 7,090 | 5,874 |
| ST20 | suppressor of tumorigenicity 20 | **1,018** | 0,819007 | 1,939 | 2,043 |
| ZNF345 | zinc finger protein 345 | **1,018** | 0,865215 | 1,662 | 2,362 |
| ALG9 | ALG9, alpha-1,2-mannosyltransferase | **1,018** | 0,743395 | 7,201 | 6,513 |
| TRMT44 | tRNA methyltransferase 44 homolog (S. cerevisiae) | **1,018** | 0,763352 | 5,373 | 6,449 |

| | | | | | |
|---|---|---|---|---|---|
| TOM1L1 | target of myb1 like 1 membrane trafficking protein | **1,018** | 0,657102 | 14,014 | 12,770 |
| SLC7A6OS | solute carrier family 7 member 6 opposite strand | **1,018** | 0,617211 | 17,171 | 19,027 |
| TBX15 | T-box 15 | **1,018** | 0,520904 | 116,707 | 119,463 |
| KIF9-AS1 | KIF9 antisense RNA 1 | **1,018** | 0,852956 | 1,939 | 2,554 |
| ADAM10 | ADAM metallopeptidase domain 10 | **1,018** | 0,498959 | 98,816 | 79,876 |
| GLTP | glycolipid transfer protein | **1,018** | 0,469582 | 89,289 | 89,837 |
| CAPN7 | calpain 7 | **1,018** | 0,515857 | 48,910 | 51,655 |
| UBAP2L | ubiquitin associated protein 2 like | **1,018** | 0,542895 | 88,015 | 80,068 |
| IMMP2L | inner mitochondrial membrane peptidase subunit 2 | **1,018** | 0,779564 | 5,041 | 4,980 |
| ZBTB21 | zinc finger and BTB domain containing 21 | **1,018** | 0,593179 | 37,721 | 40,800 |
| FANK1 | fibronectin type III and ankyrin repeat domains 1 | **1,018** | 0,861526 | 1,329 | 1,022 |
| TST | thiosulfate sulfurtransferase | **1,018** | 0,737997 | 10,635 | 8,300 |
| SOD1 | superoxide dismutase 1 | **1,018** | 0,581264 | 140,580 | 134,085 |
| TOR1B | torsin family 1 member B | **1,018** | 0,548763 | 43,481 | 47,568 |
| PER3 | period circadian clock 3 | **1,018** | 0,671919 | 36,945 | 35,117 |
| EFR3A | EFR3 homolog A | **1,017** | 0,57951 | 51,679 | 53,187 |
| KLHL25 | kelch like family member 25 | **1,017** | 0,70979 | 5,871 | 8,237 |
| SPATS2 | spermatogenesis associated serine rich 2 | **1,017** | 0,693469 | 9,361 | 9,131 |
| PPIF | peptidylprolyl isomerase F | **1,017** | 0,648682 | 46,528 | 48,079 |
| FAM193A | family with sequence similarity 193 member A | **1,017** | 0,644061 | 23,485 | 29,818 |
| GATD1 | glutamine amidotransferase like class 1 domain containing 1 | **1,017** | 0,635775 | 33,677 | 33,904 |
| CYFIP1 | cytoplasmic FMR1 interacting protein 1 | **1,017** | 0,593224 | 36,668 | 32,436 |
| AGPAT4 | 1-acylglycerol-3-phosphate O-acyltransferase 4 | **1,017** | 0,680971 | 21,713 | 14,749 |
| CTNNA1 | catenin alpha 1 | **1,017** | 0,493089 | 373,717 | 357,368 |
| STRADB | STE20-related kinase adaptor beta | **1,017** | 0,795419 | 3,656 | 3,384 |
| MAN2B2 | mannosidase alpha class 2B member 2 | **1,017** | 0,59453 | 72,340 | 52,485 |
| ADAT1 | adenosine deaminase, tRNA specific 1 | **1,017** | 0,688273 | 12,075 | 14,430 |
| TOR1AIP2 | torsin 1A interacting protein 2 | **1,017** | 0,555151 | 67,354 | 69,724 |
| COL11A1 | collagen type XI alpha 1 chain | **1,017** | 0,589685 | 295,340 | 102,032 |
| ZC3H18 | zinc finger CCCH-type containing 18 | **1,017** | 0,61727 | 34,896 | 38,821 |
| HBS1L | HBS1 like translational GTPase | **1,017** | 0,534623 | 48,245 | 58,550 |
| CRELD1 | cysteine rich with EGF like domains 1 | **1,017** | 0,547134 | 30,852 | 18,453 |
| LRRC24 | leucine rich repeat containing 24 | **1,017** | 0,871179 | 1,385 | 1,277 |

| | | | | | |
|---|---|---|---|---|---|
| NRDC | nardilysin convertase | **1,017** | 0,545479 | 83,750 | 87,985 |
| NCOR1 | nuclear receptor corepressor 1 | **1,017** | 0,618668 | 80,980 | 87,666 |
| TAF1A | TATA-box binding protein associated factor, RNA polymerase I subunit A | **1,017** | 0,620211 | 14,623 | 20,049 |
| STAG3L4 | stromal antigen 3-like 4 (pseudogene) | **1,017** | 0,771167 | 4,431 | 4,214 |
| PCDHGA5 | protocadherin gamma subfamily A, 5 | **1,017** | 0,820159 | 4,542 | 2,746 |
| BPNT1 | 3'(2'), 5'-bisphosphate nucleotidase 1 | **1,017** | 0,704958 | 10,469 | 10,982 |
| TMEM161B | transmembrane protein 161B | **1,017** | 0,726369 | 9,250 | 11,110 |
| CHAMP1 | chromosome alignment maintaining phosphoprotein 1 | **1,017** | 0,598926 | 19,774 | 20,624 |
| ANGPTL1 | angiopoietin like 1 | **1,017** | 0,689287 | 15,232 | 20,815 |
| SRSF6 | serine and arginine rich splicing factor 6 | **1,017** | 0,51359 | 66,191 | 75,534 |
| TMTC1 | transmembrane and tetratricopeptide repeat containing 1 | **1,017** | 0,61225 | 52,454 | 65,765 |
| ZNF385D | zinc finger protein 385D | **1,017** | 0,634949 | 44,977 | 50,761 |
| RAP2C-AS1 | RAP2C antisense RNA 1 | **1,017** | 0,884158 | 1,329 | 0,702 |
| SEPSECS | Sep (O-phosphoserine) tRNA:Sec (selenocysteine) tRNA synthase | **1,017** | 0,668699 | 9,638 | 11,493 |
| CDS2 | CDP-diacylglycerol synthase 2 | **1,017** | 0,61078 | 47,137 | 38,885 |
| TOPBP1 | topoisomerase (DNA) II binding protein 1 | **1,017** | 0,686142 | 14,845 | 14,749 |
| CNOT7 | CCR4-NOT transcription complex subunit 7 | **1,017** | 0,567046 | 45,143 | 46,738 |
| TPTEP1 | transmembrane phosphatase with tensin homology pseudogene 1 | **1,017** | 0,910794 | 0,332 | 0,894 |
| HAGH | hydroxyacylglutathione hydrolase | **1,017** | 0,626465 | 29,634 | 24,965 |
| PIGM | phosphatidylinositol glycan anchor biosynthesis class M | **1,017** | 0,63241 | 22,267 | 27,966 |
| ARHGAP1 | Rho GTPase activating protein 1 | **1,017** | 0,524706 | 49,242 | 48,973 |
| UBA3 | ubiquitin like modifier activating enzyme 3 | **1,017** | 0,544892 | 31,129 | 34,479 |
| PUS3 | pseudouridylate synthase 3 | **1,017** | 0,707233 | 7,201 | 11,174 |
| UBXN4 | UBX domain protein 4 | **1,017** | 0,539204 | 138,032 | 129,041 |
| AP3M1 | adaptor related protein complex 3 mu 1 subunit | **1,017** | 0,584622 | 54,005 | 53,634 |
| CENPF | centromere protein F | **1,017** | 0,771594 | 16,728 | 3,001 |
| NUCKS1 | nuclear casein kinase and cyclin dependent kinase substrate 1 | **1,017** | 0,517379 | 124,129 | 133,255 |
| PCED1A | PC-esterase domain containing 1A | **1,017** | 0,650703 | 14,014 | 11,110 |
| RPS20P22 | ribosomal protein S20 pseudogene 22 | **1,017** | 0,930336 | 0,443 | 0,638 |
| ZGRF1 | zinc finger GRF-type containing 1 | **1,017** | 0,856145 | 2,049 | 2,490 |
| SETD7 | SET domain containing lysine methyltransferase 7 | **1,017** | 0,657587 | 161,573 | 127,125 |
| BAZ1B | bromodomain adjacent to zinc finger domain 1B | **1,017** | 0,609473 | 88,680 | 89,007 |
| ZDHHC13 | zinc finger DHHC-type containing 13 | **1,017** | 0,72331 | 9,306 | 7,662 |

| | | | | | |
|---|---|---|---|---|---|
| SMARCD2 | SWI/SNF related, matrix associated, actin dependent regulator of chromatin, subfamily d, | **1,017** | 0,629374 | 26,698 | 23,114 |
| RWDD3 | RWD domain containing 3 | **1,017** | 0,915057 | 0,942 | 0,830 |
| NDUFA1 | NADH:ubiquinone oxidoreductase subunit A1 | **1,017** | 0,709545 | 31,295 | 27,264 |
| AFG3L1P | AFG3 like matrix AAA peptidase subunit 1, pseudogene | **1,017** | 0,756362 | 6,591 | 9,258 |
| AKIRIN1 | akirin 1 | **1,017** | 0,525557 | 47,082 | 56,188 |
| RBL1 | RB transcriptional corepressor like 1 | **1,017** | 0,7449 | 6,370 | 5,172 |
| NPIPB4 | nuclear pore complex interacting protein family member B4 | **1,016** | 0,89979 | 0,831 | 0,702 |
| WWP2 | WW domain containing E3 ubiquitin protein ligase 2 | **1,016** | 0,558995 | 127,951 | 156,688 |
| DDX11 | DEAD/H-box helicase 11 | **1,016** | 0,664058 | 10,746 | 15,771 |
| SERINC3 | serine incorporator 3 | **1,016** | 0,509985 | 199,460 | 174,949 |
| CASC3 | cancer susceptibility 3 | **1,016** | 0,541843 | 99,536 | 114,674 |
| ARSG | arylsulfatase G | **1,016** | 0,798296 | 3,157 | 5,491 |
| CDON | cell adhesion associated, oncogene regulated | **1,016** | 0,674992 | 85,135 | 93,285 |
| NUP62 | nucleoporin 62 | **1,016** | 0,541411 | 39,826 | 38,118 |
| GTPBP1 | GTP binding protein 1 | **1,016** | 0,603652 | 33,899 | 40,289 |
| EIF5AL1 | eukaryotic translation initiation factor 5A-like 1 | **1,016** | 0,911471 | 0,332 | 0,958 |
| EXOC4 | exocyst complex component 4 | **1,016** | 0,578644 | 37,610 | 34,990 |
| ACD | ACD, shelterin complex subunit and telomerase recruitment factor | **1,016** | 0,629537 | 10,635 | 13,025 |
| WHAMM | WAS protein homolog associated with actin, golgi membranes and microtubules | **1,016** | 0,64887 | 21,104 | 28,605 |
| G3BP2 | G3BP stress granule assembly factor 2 | **1,016** | 0,524556 | 116,209 | 116,781 |
| CSNK2A2 | casein kinase 2 alpha 2 | **1,016** | 0,543737 | 35,062 | 36,905 |
| PIGN | phosphatidylinositol glycan anchor biosynthesis class N | **1,016** | 0,572538 | 24,039 | 22,922 |
| NR2F2 | nuclear receptor subfamily 2 group F member 2 | **1,016** | 0,835726 | 5,594 | 2,490 |
| RAB3GAP2 | RAB3 GTPase activating non-catalytic protein subunit 2 | **1,016** | 0,638113 | 52,510 | 47,887 |
| UTP11 | UTP11, small subunit processome component homolog (S. cerevisiae) | **1,016** | 0,603417 | 23,208 | 31,095 |
| ACOT2 | acyl-CoA thioesterase 2 | **1,016** | 0,743198 | 6,481 | 5,300 |
| APLN | apelin | **1,016** | 0,892504 | 0,609 | 0,702 |
| BRMS1 | breast cancer metastasis suppressor 1 | **1,016** | 0,621225 | 19,996 | 19,155 |
| YIPF4 | Yip1 domain family member 4 | **1,016** | 0,648925 | 54,393 | 59,636 |
| GLB1L | galactosidase beta 1 like | **1,016** | 0,719603 | 10,856 | 6,385 |
| FAM160A2 | family with sequence similarity 160 member A2 | **1,016** | 0,656285 | 19,497 | 25,157 |
| RNF34 | ring finger protein 34 | **1,016** | 0,636813 | 20,439 | 18,772 |
| FBXL18 | F-box and leucine rich repeat protein 18 | **1,016** | 0,733103 | 9,804 | 9,961 |

| | | | | | |
|---|---|---|---|---|---|
| NPEPPS | aminopeptidase puromycin sensitive | **1,016** | 0,522573 | 65,028 | 50,505 |
| SBSPON | somatomedin B and thrombospondin type 1 domain containing | **1,016** | 0,80972 | 1,329 | 3,384 |
| NRIP3 | nuclear receptor interacting protein 3 | **1,016** | 0,811136 | 3,545 | 2,299 |
| GSTCD | glutathione S-transferase C-terminal domain containing | **1,016** | 0,800912 | 5,373 | 5,938 |
| ZNF318 | zinc finger protein 318 | **1,016** | 0,732207 | 24,870 | 22,347 |
| EEPD1 | endonuclease/exonuclease/phosphatase family domain containing 1 | **1,016** | 0,88701 | 2,493 | 1,405 |
| GAA | glucosidase alpha, acid | **1,016** | 0,607841 | 71,398 | 49,356 |
| RHBDD1 | rhomboid domain containing 1 | **1,016** | 0,610045 | 28,581 | 31,670 |
| ZNF687 | zinc finger protein 687 | **1,016** | 0,643434 | 29,966 | 27,328 |
| VMP1 | vacuole membrane protein 1 | **1,016** | 0,560133 | 94,052 | 99,287 |
| TIA1 | TIA1 cytotoxic granule associated RNA binding protein | **1,016** | 0,727569 | 21,602 | 21,517 |
| SLC45A1 | solute carrier family 45 member 1 | **1,016** | 0,803014 | 2,936 | 3,384 |
| PCCA | propionyl-CoA carboxylase alpha subunit | **1,016** | 0,775329 | 5,041 | 5,874 |
| GBP1 | guanylate binding protein 1 | **1,016** | 0,605606 | 21,325 | 24,263 |
| CANT1 | calcium activated nucleotidase 1 | **1,016** | 0,595928 | 71,232 | 67,617 |
| PCGF3 | polycomb group ring finger 3 | **1,016** | 0,662015 | 34,065 | 26,945 |
| SNHG8 | small nucleolar RNA host gene 8 | **1,016** | 0,752061 | 16,728 | 22,858 |
| SNX29 | sorting nexin 29 | **1,016** | 0,636184 | 41,820 | 46,738 |
| GPRASP2 | G protein-coupled receptor associated sorting protein 2 | **1,016** | 0,684985 | 19,276 | 18,963 |
| CHMP5 | charged multivesicular body protein 5 | **1,016** | 0,585086 | 88,624 | 88,751 |
| STOM | stomatin | **1,016** | 0,599997 | 52,676 | 47,760 |
| STX12 | syntaxin 12 | **1,015** | 0,57671 | 31,129 | 30,584 |
| NMT1 | N-myristoyltransferase 1 | **1,015** | 0,539502 | 107,125 | 117,292 |
| ZNF326 | zinc finger protein 326 | **1,015** | 0,593798 | 21,270 | 25,285 |
| SREK1 | splicing regulatory glutamic acid and lysine rich protein 1 | **1,015** | 0,637761 | 52,731 | 58,040 |
| FADS3 | fatty acid desaturase 3 | **1,015** | 0,594924 | 61,926 | 40,098 |
| KCTD9 | potassium channel tetramerization domain containing 9 | **1,015** | 0,608444 | 42,484 | 37,927 |
| MINCR | MYC-induced long noncoding RNA | **1,015** | 0,874886 | 0,997 | 1,852 |
| NBPF25P | NBPF member 25, pseudogene | **1,015** | 0,914294 | 0,997 | 0,511 |
| AARS | alanyl-tRNA synthetase | **1,015** | 0,545097 | 221,727 | 237,458 |
| TICAM1 | toll like receptor adaptor molecule 1 | **1,015** | 0,621819 | 30,686 | 38,310 |
| SNAP47 | synaptosome associated protein 47 | **1,015** | 0,677862 | 23,042 | 20,624 |
| C3orf62 | chromosome 3 open reading frame 62 | **1,015** | 0,806326 | 3,767 | 3,576 |

| | | | | | |
|---|---|---|---|---|---|
| RHOF | ras homolog family member F, filopodia associated | **1,015** | 0,692967 | 9,084 | 8,684 |
| ACOT1 | acyl-CoA thioesterase 1 | **1,015** | 0,897015 | 0,665 | 2,043 |
| PQLC2 | PQ loop repeat containing 2 | **1,015** | 0,760604 | 10,912 | 9,258 |
| CCPG1 | cell cycle progression 1 | **1,015** | 0,582111 | 74,500 | 74,768 |
| FBXW2 | F-box and WD repeat domain containing 2 | **1,015** | 0,634916 | 52,178 | 46,866 |
| ZNF91 | zinc finger protein 91 | **1,015** | 0,751624 | 8,862 | 10,854 |
| PWWP2A | PWWP domain containing 2A | **1,015** | 0,660175 | 14,512 | 12,706 |
| FAM210B | family with sequence similarity 210 member B | **1,015** | 0,61114 | 30,409 | 25,540 |
| PML | promyelocytic leukemia | **1,015** | 0,703031 | 53,341 | 57,976 |
| NCOA4 | nuclear receptor coactivator 4 | **1,015** | 0,581607 | 108,786 | 89,134 |
| CHURC1 | churchill domain containing 1 | **1,015** | 0,63382 | 26,643 | 60,274 |
| BAK1 | BCL2 antagonist/killer 1 | **1,015** | 0,775387 | 4,542 | 3,384 |
| C11orf58 | chromosome 11 open reading frame 58 | **1,015** | 0,603442 | 121,969 | 128,338 |
| MED6 | mediator complex subunit 6 | **1,015** | 0,671658 | 15,343 | 17,495 |
| GTF3C4 | general transcription factor IIIC subunit 4 | **1,015** | 0,645386 | 34,674 | 36,394 |
| GBA2 | glucosylceramidase beta 2 | **1,015** | 0,627551 | 30,465 | 31,286 |
| COPS5 | COP9 signalosome subunit 5 | **1,015** | 0,64686 | 27,640 | 31,414 |
| LARP1B | La ribonucleoprotein domain family member 1B | **1,015** | 0,715022 | 12,684 | 14,175 |
| CPSF2 | cleavage and polyadenylation specific factor 2 | **1,015** | 0,578201 | 51,568 | 60,977 |
| ZCCHC6 | zinc finger CCHC-type containing 6 | **1,015** | 0,701366 | 37,831 | 39,906 |
| CEP120 | centrosomal protein 120 | **1,015** | 0,697217 | 17,780 | 19,602 |
| DOCK7 | dedicator of cytokinesis 7 | **1,015** | 0,677075 | 50,294 | 38,310 |
| SEC62 | SEC62 homolog, preprotein translocation factor | **1,015** | 0,673383 | 160,133 | 159,114 |
| STPG1 | sperm tail PG-rich repeat containing 1 | **1,015** | 0,767342 | 8,585 | 6,768 |
| FN3KRP | fructosamine 3 kinase related protein | **1,015** | 0,726561 | 16,839 | 24,135 |
| PICALM | phosphatidylinositol binding clathrin assembly protein | **1,015** | 0,623307 | 189,933 | 173,033 |
| GAPVD1 | GTPase activating protein and VPS9 domains 1 | **1,015** | 0,682869 | 45,365 | 53,570 |
| CUL4A | cullin 4A | **1,015** | 0,570395 | 55,002 | 57,720 |
| PPP1R15B | protein phosphatase 1 regulatory subunit 15B | **1,015** | 0,633315 | 55,446 | 62,317 |
| PYGO2 | pygopus family PHD finger 2 | **1,014** | 0,657123 | 29,412 | 32,116 |
| HIF1A-AS2 | HIF1A antisense RNA 2 | **1,014** | 0,850045 | 1,163 | 4,278 |
| ATG4B | autophagy related 4B cysteine peptidase | **1,014** | 0,608124 | 37,665 | 40,417 |
| C5orf22 | chromosome 5 open reading frame 22 | **1,014** | 0,689351 | 17,226 | 21,517 |

| | | | | | |
|---|---|---|---|---|---|
| ARHGEF3 | Rho guanine nucleotide exchange factor 3 | **1,014** | 0,7993 | 5,207 | 4,661 |
| ITM2A | integral membrane protein 2A | **1,014** | 0,713158 | 48,300 | 7,470 |
| EID2 | EP300 interacting inhibitor of differentiation 2 | **1,014** | 0,736933 | 14,955 | 11,685 |
| PIGH | phosphatidylinositol glycan anchor biosynthesis class H | **1,014** | 0,621894 | 21,381 | 25,412 |
| KIAA0368 | KIAA0368 | **1,014** | 0,604583 | 116,209 | 100,627 |
| TMCC1 | transmembrane and coiled-coil domain family 1 | **1,014** | 0,741402 | 12,130 | 9,194 |
| PHAX | phosphorylated adaptor for RNA export | **1,014** | 0,578702 | 46,583 | 51,399 |
| HNRNPH2 | heterogeneous nuclear ribonucleoprotein H2 | **1,014** | 0,600066 | 75,054 | 64,616 |
| CTTNBP2NL | CTTNBP2 N-terminal like | **1,014** | 0,77576 | 32,016 | 28,349 |
| EPYC | epiphycan | **1,014** | 0,923969 | 1,163 | 0,766 |
| ACOX1 | acyl-CoA oxidase 1 | **1,014** | 0,62499 | 44,866 | 38,693 |
| UBR1 | ubiquitin protein ligase E3 component n-recognin 1 | **1,014** | 0,724779 | 42,595 | 44,950 |
| HIST1H3E | histone cluster 1 H3 family member e | **1,014** | 0,931457 | 0,443 | 0,319 |
| TRAPPC9 | trafficking protein particle complex 9 | **1,014** | 0,773376 | 9,804 | 10,535 |
| ATXN3 | ataxin 3 | **1,014** | 0,715297 | 17,226 | 15,771 |
| DCAF6 | DDB1 and CUL4 associated factor 6 | **1,014** | 0,64028 | 33,013 | 31,733 |
| H2AFJ | H2A histone family member J | **1,014** | 0,678936 | 47,359 | 49,164 |
| HERC2 | HECT and RLD domain containing E3 ubiquitin protein ligase 2 | **1,014** | 0,862413 | 10,192 | 10,791 |
| AAK1 | AP2 associated kinase 1 | **1,014** | 0,763795 | 43,814 | 43,673 |
| PRPF4 | pre-mRNA processing factor 4 | **1,014** | 0,659909 | 30,132 | 32,691 |
| RFWD3 | ring finger and WD repeat domain 3 | **1,014** | 0,719924 | 22,821 | 18,580 |
| SMC4 | structural maintenance of chromosomes 4 | **1,014** | 0,684249 | 32,459 | 25,029 |
| B4GALT1-AS1 | B4GALT1 antisense RNA 1 | **1,014** | 0,922692 | 0,554 | 0,638 |
| NPC1 | NPC intracellular cholesterol transporter 1 | **1,014** | 0,619581 | 239,562 | 207,576 |
| LRFN4 | leucine rich repeat and fibronectin type III domain containing 4 | **1,014** | 0,677807 | 49,962 | 42,716 |
| ZNF826P | zinc finger protein 826, pseudogene | **1,014** | 0,83634 | 3,877 | 3,703 |
| KIF13B | kinesin family member 13B | **1,014** | 0,634471 | 86,575 | 79,812 |
| SMC3 | structural maintenance of chromosomes 3 | **1,014** | 0,607373 | 46,472 | 49,675 |
| GIN1 | gypsy retrotransposon integrase 1 | **1,014** | 0,757805 | 5,927 | 8,173 |
| NCK2 | NCK adaptor protein 2 | **1,014** | 0,637405 | 76,494 | 66,787 |
| TYSND1 | trypsin domain containing 1 | **1,014** | 0,818336 | 5,151 | 6,321 |
| FOXJ2 | forkhead box J2 | **1,014** | 0,699716 | 27,750 | 30,137 |
| RAB5B | RAB5B, member RAS oncogene family | **1,014** | 0,648628 | 104,909 | 85,623 |

| | | | | | |
|---|---|---|---|---|---|
| SLC37A3 | solute carrier family 37 member 3 | **1,013** | 0,651145 | 35,394 | 48,207 |
| OGFR | opioid growth factor receptor | **1,013** | 0,703722 | 28,249 | 29,179 |
| AP5Z1 | adaptor related protein complex 5 zeta 1 subunit | **1,013** | 0,636661 | 31,905 | 34,926 |
| AIG1 | androgen induced 1 | **1,013** | 0,678808 | 26,920 | 27,455 |
| TXN | thioredoxin | **1,013** | 0,715401 | 240,670 | 173,544 |
| LRCH3 | leucine rich repeats and calponin homology domain containing 3 | **1,013** | 0,645889 | 28,692 | 26,625 |
| ZNF697 | zinc finger protein 697 | **1,013** | 0,687327 | 22,932 | 28,988 |
| ZNF232 | zinc finger protein 232 | **1,013** | 0,785634 | 4,708 | 5,108 |
| EIF1B | eukaryotic translation initiation factor 1B | **1,013** | 0,65551 | 36,945 | 36,267 |
| CCDC183-AS1 | CCDC183 antisense RNA 1 | **1,013** | 0,843805 | 2,936 | 5,172 |
| BLVRB | biliverdin reductase B | **1,013** | 0,705132 | 21,214 | 15,962 |
| NBPF1 | NBPF member 1 | **1,013** | 0,831871 | 13,127 | 11,365 |
| CCNDBP1 | cyclin D1 binding protein 1 | **1,013** | 0,621399 | 47,026 | 42,332 |
| VDAC1 | voltage dependent anion channel 1 | **1,013** | 0,612624 | 93,886 | 79,749 |
| SLC25A38 | solute carrier family 25 member 38 | **1,013** | 0,66484 | 26,643 | 28,158 |
| SLC23A2 | solute carrier family 23 member 2 | **1,013** | 0,700401 | 113,993 | 88,624 |
| SPA17 | sperm autoantigenic protein 17 | **1,013** | 0,765093 | 7,367 | 7,023 |
| REV3L | REV3 like, DNA directed polymerase zeta catalytic subunit | **1,013** | 0,767337 | 27,640 | 32,308 |
| ZNF720 | zinc finger protein 720 | **1,013** | 0,755106 | 11,798 | 12,004 |
| YWHAZ | tyrosine 3-monooxygenase/tryptophan 5-monooxygenase activation protein zeta | **1,013** | 0,608038 | 290,300 | 233,371 |
| NUDT16P1 | nudix hydrolase 16 pseudogene 1 | **1,013** | 0,86375 | 2,991 | 2,235 |
| INIP | INTS3 and NABP interacting protein | **1,013** | 0,675479 | 27,640 | 30,201 |
| TRERF1 | transcriptional regulating factor 1 | **1,013** | 0,717565 | 29,024 | 24,008 |
| UPF2 | UPF2 regulator of nonsense transcripts homolog (yeast) | **1,013** | 0,62947 | 58,547 | 55,996 |
| MRC2 | mannose receptor C type 2 | **1,013** | 0,701655 | 312,234 | 243,268 |
| EIF3L | eukaryotic translation initiation factor 3 subunit L | **1,013** | 0,677474 | 61,040 | 58,359 |
| PLGRKT | plasminogen receptor with a C-terminal lysine | **1,013** | 0,732559 | 20,384 | 15,771 |
| PIGX | phosphatidylinositol glycan anchor biosynthesis class X | **1,013** | 0,740235 | 14,124 | 14,558 |
| MYNN | myoneurin | **1,013** | 0,73577 | 18,722 | 21,390 |
| ADAMTSL1 | ADAMTS like 1 | **1,013** | 0,871571 | 2,825 | 4,342 |
| UNC119B | unc-119 lipid binding chaperone B | **1,013** | 0,618753 | 92,003 | 107,587 |
| MCOLN1 | mucolipin 1 | **1,013** | 0,645745 | 21,602 | 23,241 |
| BTBD1 | BTB domain containing 1 | **1,013** | 0,66372 | 40,102 | 32,819 |

| | | | | | |
|---|---|---|---|---|---|
| MPC1 | mitochondrial pyruvate carrier 1 | **1,013** | 0,685515 | 15,232 | 15,579 |
| VPS54 | VPS54, GARP complex subunit | **1,013** | 0,701855 | 23,652 | 22,539 |
| TRAF3IP2 | TRAF3 interacting protein 2 | **1,013** | 0,61398 | 121,914 | 118,952 |
| VAPA | VAMP associated protein A | **1,013** | 0,643224 | 98,428 | 93,157 |
| PPP4R2 | protein phosphatase 4 regulatory subunit 2 | **1,013** | 0,642159 | 56,332 | 53,315 |
| GIT1 | GIT ArfGAP 1 | **1,013** | 0,673111 | 27,086 | 25,093 |
| SKAP2 | src kinase associated phosphoprotein 2 | **1,013** | 0,762221 | 19,996 | 6,960 |
| MPC2 | mitochondrial pyruvate carrier 2 | **1,013** | 0,72555 | 35,893 | 41,566 |
| ANKRD27 | ankyrin repeat domain 27 | **1,013** | 0,721595 | 33,234 | 20,687 |
| SMARCA5 | SWI/SNF related, matrix associated, actin dependent regulator of chromatin, subfamily a, | **1,013** | 0,642335 | 57,218 | 67,489 |
| ITCH | itchy E3 ubiquitin protein ligase | **1,013** | 0,699632 | 53,507 | 50,186 |
| DPP8 | dipeptidyl peptidase 8 | **1,013** | 0,707082 | 32,071 | 27,647 |
| SKP1 | S-phase kinase associated protein 1 | **1,013** | 0,661301 | 104,854 | 99,670 |
| TBCD | tubulin folding cofactor D | **1,013** | 0,789154 | 10,967 | 8,045 |
| DENND4A | DENN domain containing 4A | **1,013** | 0,737409 | 22,322 | 24,774 |
| SLC20A2 | solute carrier family 20 member 2 | **1,013** | 0,674236 | 18,334 | 23,880 |
| MELTF | melanotransferrin | **1,013** | 0,698082 | 402,576 | 254,633 |
| RPL7 | ribosomal protein L7 | **1,013** | 0,7285 | 296,116 | 322,378 |
| TBCK | TBC1 domain containing kinase | **1,013** | 0,757166 | 14,457 | 15,707 |
| WASH2P | WAS protein family homolog 2 pseudogene | **1,013** | 0,882657 | 1,772 | 2,554 |
| NEK3 | NIMA related kinase 3 | **1,013** | 0,839086 | 4,708 | 5,044 |
| LTBP3 | latent transforming growth factor beta binding protein 3 | **1,013** | 0,670648 | 458,852 | 433,158 |
| SLC25A25-AS1 | SLC25A25 antisense RNA 1 | **1,013** | 0,765411 | 6,370 | 8,620 |
| SETD5 | SET domain containing 5 | **1,012** | 0,740777 | 72,450 | 78,855 |
| C1S | complement C1s | **1,012** | 0,621243 | 953,099 | 806,296 |
| ZNF223 | zinc finger protein 223 | **1,012** | 0,945814 | 0,554 | 0,447 |
| RMI1 | RecQ mediated genome instability 1 | **1,012** | 0,833301 | 5,927 | 4,725 |
| VOPP1 | vesicular, overexpressed in cancer, prosurvival protein 1 | **1,012** | 0,713557 | 24,427 | 21,645 |
| PLK3 | polo like kinase 3 | **1,012** | 0,634877 | 48,633 | 58,040 |
| METRN | meteorin, glial cell differentiation regulator | **1,012** | 0,787035 | 21,768 | 20,049 |
| MSANTD4 | Myb/SANT DNA binding domain containing 4 with coiled-coils | **1,012** | 0,68774 | 24,538 | 25,157 |
| ZBTB25 | zinc finger and BTB domain containing 25 | **1,012** | 0,778787 | 15,731 | 17,814 |
| DUSP5 | dual specificity phosphatase 5 | **1,012** | 0,727151 | 47,026 | 60,083 |

| | | | | | |
|---|---|---|---|---|---|
| CAMLG | calcium modulating ligand | **1,012** | 0,696472 | 25,424 | 28,413 |
| SSFA2 | sperm specific antigen 2 | **1,012** | 0,723189 | 82,587 | 72,087 |
| ZNF217 | zinc finger protein 217 | **1,012** | 0,728128 | 63,588 | 70,171 |
| LMX1B | LIM homeobox transcription factor 1 beta | **1,012** | 0,899036 | 1,994 | 2,235 |
| FRA10AC1 | FRA10A associated CGG repeat 1 | **1,012** | 0,756738 | 13,681 | 18,197 |
| EP400 | E1A binding protein p400 | **1,012** | 0,750793 | 49,242 | 55,549 |
| F5 | coagulation factor V | **1,012** | 0,786515 | 19,719 | 20,815 |
| TAF4 | TATA-box binding protein associated factor 4 | **1,012** | 0,864493 | 2,770 | 3,703 |
| BRAF | B-Raf proto-oncogene, serine/threonine kinase | **1,012** | 0,794833 | 28,138 | 30,329 |
| ZFYVE27 | zinc finger FYVE-type containing 27 | **1,012** | 0,658077 | 31,794 | 36,650 |
| CLASRP | CLK4 associating serine/arginine rich protein | **1,012** | 0,736246 | 14,568 | 19,219 |
| KCNJ2-AS1 | KCNJ2 antisense RNA 1 (head to head) | **1,012** | 0,910041 | 1,496 | 1,724 |
| GNAS | GNAS complex locus | **1,012** | 0,668435 | 354,774 | 225,901 |
| HINT1 | histidine triad nucleotide binding protein 1 | **1,012** | 0,682106 | 102,970 | 93,668 |
| RXRA | retinoid X receptor alpha | **1,012** | 0,677885 | 38,662 | 31,095 |
| EID2B | EP300 interacting inhibitor of differentiation 2B | **1,012** | 0,841524 | 4,431 | 3,512 |
| DPH7 | diphthamide biosynthesis 7 | **1,012** | 0,752338 | 11,300 | 13,728 |
| MTRF1 | mitochondrial translation release factor 1 | **1,012** | 0,856905 | 3,877 | 4,278 |
| TRIB3 | tribbles pseudokinase 3 | **1,012** | 0,643172 | 114,159 | 92,135 |
| CYHR1 | cysteine and histidine rich 1 | **1,012** | 0,709919 | 29,689 | 30,520 |
| EDA2R | ectodysplasin A2 receptor | **1,012** | 0,889725 | 3,268 | 4,342 |
| OCRL | OCRL, inositol polyphosphate-5-phosphatase | **1,012** | 0,692671 | 33,179 | 31,733 |
| ZC3H12A | zinc finger CCCH-type containing 12A | **1,012** | 0,663736 | 152,932 | 175,013 |
| ACBD5 | acyl-CoA binding domain containing 5 | **1,012** | 0,7103 | 23,153 | 20,304 |
| ACVR1B | activin A receptor type 1B | **1,012** | 0,742718 | 15,232 | 13,025 |
| FAM149B1 | family with sequence similarity 149 member B1 | **1,012** | 0,746616 | 19,054 | 16,282 |
| ETV6 | ETS variant 6 | **1,012** | 0,750978 | 16,451 | 17,239 |
| PABPC4 | poly(A) binding protein cytoplasmic 4 | **1,012** | 0,684942 | 127,287 | 108,226 |
| CCDC157 | coiled-coil domain containing 157 | **1,012** | 0,854661 | 3,323 | 4,023 |
| SLC35F6 | solute carrier family 35 member F6 | **1,012** | 0,686711 | 37,499 | 39,587 |
| 2.maalis | membrane associated ring-CH-type finger 2 | **1,012** | 0,750576 | 18,057 | 16,729 |
| SPTLC2 | serine palmitoyltransferase long chain base subunit 2 | **1,012** | 0,739254 | 23,596 | 17,814 |
| PACS2 | phosphofurin acidic cluster sorting protein 2 | **1,012** | 0,73969 | 35,727 | 36,394 |

| | | | | | |
|---|---|---|---|---|---|
| SELENBP1 | selenium binding protein 1 | **1,012** | 0,863408 | 3,988 | 4,789 |
| PCYT1A | phosphate cytidylyltransferase 1, choline, alpha | **1,012** | 0,692357 | 71,730 | 66,595 |
| C15orf61 | chromosome 15 open reading frame 61 | **1,012** | 0,862608 | 4,708 | 2,809 |
| AGL | amylo-alpha-1, 6-glucosidase, 4-alpha-glucanotransferase | **1,012** | 0,81916 | 7,865 | 6,193 |
| DPYD | dihydropyrimidine dehydrogenase | **1,011** | 0,727295 | 18,057 | 19,793 |
| SMAD1 | SMAD family member 1 | **1,011** | 0,854278 | 4,985 | 3,895 |
| TRA2B | transformer 2 beta homolog | **1,011** | 0,670594 | 55,723 | 80,962 |
| TDRP | testis development related protein | **1,011** | 0,832327 | 4,819 | 5,108 |
| MAN1C1 | mannosidase alpha class 1C member 1 | **1,011** | 0,884624 | 5,262 | 2,618 |
| EPB41L4A-AS1 | EPB41L4A antisense RNA 1 | **1,011** | 0,74964 | 15,177 | 17,495 |
| SPTBN5 | spectrin beta, non-erythrocytic 5 | **1,011** | 0,913577 | 0,499 | 2,362 |
| RP2 | RP2, ARL3 GTPase activating protein | **1,011** | 0,705006 | 24,704 | 20,368 |
| PDXK | pyridoxal kinase | **1,011** | 0,677064 | 81,257 | 81,983 |
| RPS18P9 | ribosomal protein S18 pseudogene 9 | **1,011** | 0,899093 | 1,496 | 1,341 |
| TBC1D23 | TBC1 domain family member 23 | **1,011** | 0,698965 | 42,484 | 37,416 |
| IL10RB-AS1 | IL10RB antisense RNA 1 (head to head) | **1,011** | 0,941803 | 0,942 | 0,894 |
| GLG1 | golgi glycoprotein 1 | **1,011** | 0,681936 | 390,667 | 369,052 |
| CAP1 | adenylate cyclase associated protein 1 | **1,011** | 0,642163 | 169,715 | 144,492 |
| SCP2 | sterol carrier protein 2 | **1,011** | 0,732399 | 34,619 | 29,243 |
| NAXE | NAD(P)HX epimerase | **1,011** | 0,77074 | 18,390 | 20,560 |
| GJC1 | gap junction protein gamma 1 | **1,011** | 0,901153 | 2,382 | 1,405 |
| LINC02454 | long intergenic non-protein coding RNA 2454 | **1,011** | 0,927317 | 1,385 | 0,702 |
| MICU2 | mitochondrial calcium uptake 2 | **1,011** | 0,716325 | 30,465 | 30,584 |
| GPX4 | glutathione peroxidase 4 | **1,011** | 0,767758 | 160,355 | 184,335 |
| EXOSC1 | exosome component 1 | **1,011** | 0,769688 | 10,413 | 12,706 |
| ADAM8 | ADAM metallopeptidase domain 8 | **1,011** | 0,908223 | 1,551 | 1,915 |
| TBC1D10A | TBC1 domain family member 10A | **1,011** | 0,773542 | 10,303 | 10,024 |
| ATP6V0B | ATPase H+ transporting V0 subunit b | **1,011** | 0,721827 | 65,139 | 68,000 |
| NRBF2 | nuclear receptor binding factor 2 | **1,011** | 0,77347 | 26,310 | 24,008 |
| ZNF324B | zinc finger protein 324B | **1,011** | 0,89099 | 1,772 | 1,724 |
| FAM172A | family with sequence similarity 172 member A | **1,011** | 0,77425 | 20,439 | 18,836 |
| UBN2 | ubinuclein 2 | **1,011** | 0,872093 | 21,048 | 22,794 |
| CDC25B | cell division cycle 25B | **1,011** | 0,713225 | 23,153 | 23,497 |

| | | | | | |
|---|---|---|---|---|---|
| ZMAT2 | zinc finger matrin-type 2 | **1,011** | 0,722997 | 56,996 | 54,400 |
| ZNF605 | zinc finger protein 605 | **1,011** | 0,823377 | 9,306 | 9,258 |
| GANAB | glucosidase II alpha subunit | **1,011** | 0,661214 | 469,210 | 358,709 |
| TMLHE | trimethyllysine hydroxylase, epsilon | **1,011** | 0,876106 | 2,880 | 2,171 |
| NBN | nibrin | **1,011** | 0,704848 | 54,393 | 63,786 |
| CRY2 | cryptochrome circadian clock 2 | **1,011** | 0,717876 | 36,890 | 46,163 |
| CDCA4 | cell division cycle associated 4 | **1,011** | 0,765922 | 14,124 | 12,068 |
| CDC5L | cell division cycle 5 like | **1,011** | 0,702193 | 45,752 | 44,823 |
| ZER1 | zyg-11 related cell cycle regulator | **1,011** | 0,722077 | 38,330 | 34,607 |
| TP63 | tumor protein p63 | **1,011** | 0,93039 | 0,720 | 1,405 |
| NEK4 | NIMA related kinase 4 | **1,011** | 0,739498 | 19,940 | 19,091 |
| TMEM131 | transmembrane protein 131 | **1,011** | 0,782015 | 75,331 | 76,428 |
| TMEM234 | transmembrane protein 234 | **1,011** | 0,810307 | 10,524 | 8,939 |
| FKTN | fukutin | **1,011** | 0,809712 | 17,559 | 18,070 |
| MBTPS1 | membrane bound transcription factor peptidase, site 1 | **1,011** | 0,669408 | 196,414 | 180,631 |
| PQLC3 | PQ loop repeat containing 3 | **1,011** | 0,81938 | 8,309 | 7,790 |
| SYNJ1 | synaptojanin 1 | **1,011** | 0,843501 | 17,282 | 17,112 |
| C12orf29 | chromosome 12 open reading frame 29 | **1,011** | 0,809228 | 16,728 | 21,517 |
| NEURL4 | neuralized E3 ubiquitin protein ligase 4 | **1,011** | 0,789479 | 13,017 | 15,707 |
| EXOC6 | exocyst complex component 6 | **1,010** | 0,890741 | 2,326 | 2,618 |
| SGMS2 | sphingomyelin synthase 2 | **1,010** | 0,721787 | 77,269 | 66,787 |
| STXBP3 | syntaxin binding protein 3 | **1,010** | 0,749476 | 60,375 | 57,784 |
| UHRF1BP1L | UHRF1 binding protein 1 like | **1,010** | 0,770353 | 57,218 | 47,632 |
| LAG3 | lymphocyte activating 3 | **1,010** | 0,777124 | 14,789 | 15,388 |
| RASGRP3 | RAS guanyl releasing protein 3 | **1,010** | 0,943116 | 1,440 | 1,277 |
| YTHDC2 | YTH domain containing 2 | **1,010** | 0,807668 | 17,503 | 20,113 |
| GLCCI1 | glucocorticoid induced 1 | **1,010** | 0,835052 | 7,810 | 6,193 |
| DDX39A | DExD-box helicase 39A | **1,010** | 0,705683 | 39,327 | 37,735 |
| PLCG1 | phospholipase C gamma 1 | **1,010** | 0,783684 | 33,234 | 25,795 |
| RAB32 | RAB32, member RAS oncogene family | **1,010** | 0,77041 | 35,228 | 29,818 |
| RAB12 | RAB12, member RAS oncogene family | **1,010** | 0,754525 | 22,876 | 21,964 |
| KCTD18 | potassium channel tetramerization domain containing 18 | **1,010** | 0,740492 | 18,611 | 18,389 |
| RPL15 | ribosomal protein L15 | **1,010** | 0,724767 | 327,356 | 330,551 |

| | | | | | |
|---|---|---|---|---|---|
| MRPS18A | mitochondrial ribosomal protein S18A | **1,010** | 0,753799 | 23,375 | 25,093 |
| NBPF12 | NBPF member 12 | **1,010** | 0,843258 | 5,539 | 6,513 |
| ZNF655 | zinc finger protein 655 | **1,010** | 0,76648 | 37,610 | 43,801 |
| CAND1 | cullin associated and neddylation dissociated 1 | **1,010** | 0,714639 | 87,627 | 82,239 |
| HESX1 | HESX homeobox 1 | **1,010** | 0,952066 | 0,388 | 0,575 |
| MUL1 | mitochondrial E3 ubiquitin protein ligase 1 | **1,010** | 0,701103 | 52,565 | 56,890 |
| ARHGEF11 | Rho guanine nucleotide exchange factor 11 | **1,010** | 0,78237 | 50,516 | 52,676 |
| RPL3P4 | ribosomal protein L3 pseudogene 4 | **1,010** | 0,886737 | 2,160 | 2,107 |
| ZNF101 | zinc finger protein 101 | **1,010** | 0,832679 | 5,207 | 6,321 |
| PSMD7 | proteasome 26S subunit, non-ATPase 7 | **1,010** | 0,710641 | 85,910 | 80,962 |
| CHD8 | chromodomain helicase DNA binding protein 8 | **1,010** | 0,790766 | 55,944 | 58,231 |
| MSTO2P | misato family member 2, pseudogene | **1,010** | 0,921928 | 1,219 | 1,660 |
| MOV10 | Mov10 RISC complex RNA helicase | **1,010** | 0,744193 | 21,325 | 20,240 |
| SUFU | SUFU negative regulator of hedgehog signaling | **1,010** | 0,791956 | 13,404 | 13,345 |
| C2orf49 | chromosome 2 open reading frame 49 | **1,010** | 0,766528 | 24,926 | 24,518 |
| TTC39B | tetratricopeptide repeat domain 39B | **1,010** | 0,823512 | 12,518 | 12,578 |
| ZBTB3 | zinc finger and BTB domain containing 3 | **1,010** | 0,904332 | 3,379 | 2,873 |
| PBXIP1 | PBX homeobox interacting protein 1 | **1,010** | 0,789778 | 77,546 | 45,142 |
| VPS45 | vacuolar protein sorting 45 homolog | **1,010** | 0,779539 | 11,632 | 12,387 |
| ANKRD42 | ankyrin repeat domain 42 | **1,010** | 0,777369 | 17,337 | 19,347 |
| RPP38 | ribonuclease P/MRP subunit p38 | **1,010** | 0,83823 | 8,641 | 10,408 |
| GNPTG | N-acetylglucosamine-1-phosphate transferase gamma subunit | **1,010** | 0,756838 | 52,233 | 44,695 |
| CDC16 | cell division cycle 16 | **1,010** | 0,750525 | 31,849 | 30,265 |
| ATP2C1 | ATPase secretory pathway Ca2+ transporting 1 | **1,010** | 0,744664 | 64,031 | 54,081 |
| SFMBT1 | Scm-like with four mbt domains 1 | **1,010** | 0,796382 | 14,180 | 11,174 |
| PAPD7 | poly(A) RNA polymerase D7, non-canonical | **1,010** | 0,774198 | 25,203 | 27,264 |
| SH3BGRL3 | SH3 domain binding glutamate rich protein like 3 | **1,010** | 0,731618 | 599,432 | 582,694 |
| HOXA-AS3 | HOXA cluster antisense RNA 3 | **1,010** | 0,959945 | 0,332 | 0,702 |
| TSPYL1 | TSPY like 1 | **1,010** | 0,711816 | 111,279 | 88,113 |
| TTC1 | tetratricopeptide repeat domain 1 | **1,010** | 0,74004 | 61,594 | 71,767 |
| GABRA4 | gamma-aminobutyric acid type A receptor alpha4 subunit | **1,010** | 0,882718 | 4,985 | 8,939 |
| APOL6 | apolipoprotein L6 | **1,010** | 0,803769 | 24,150 | 30,009 |
| UBC | ubiquitin C | **1,010** | 0,727558 | 705,837 | 710,330 |

| | | | | | |
|---|---|---|---|---|---|
| UMPS | uridine monophosphate synthetase | **1,009** | 0,791525 | 19,774 | 19,027 |
| FBXO44 | F-box protein 44 | **1,009** | 0,84784 | 8,253 | 5,683 |
| ALS2CL | ALS2 C-terminal like | **1,009** | 0,79859 | 18,445 | 24,646 |
| C15orf41 | chromosome 15 open reading frame 41 | **1,009** | 0,923599 | 1,717 | 1,660 |
| CCZ1 | CCZ1 homolog, vacuolar protein trafficking and biogenesis associated | **1,009** | 0,880132 | 5,871 | 1,532 |
| UBL7 | ubiquitin like 7 | **1,009** | 0,791871 | 24,095 | 29,690 |
| FAM208A | family with sequence similarity 208 member A | **1,009** | 0,766693 | 43,592 | 38,885 |
| STAT5B | signal transducer and activator of transcription 5B | **1,009** | 0,779579 | 21,935 | 24,391 |
| APPL1 | adaptor protein, phosphotyrosine interacting with PH domain and leucine zipper 1 | **1,009** | 0,747445 | 32,459 | 31,989 |
| TP53 | tumor protein p53 | **1,009** | 0,806052 | 25,756 | 34,862 |
| GRSF1 | G-rich RNA sequence binding factor 1 | **1,009** | 0,751689 | 56,886 | 52,421 |
| NUCB2 | nucleobindin 2 | **1,009** | 0,739676 | 150,440 | 92,135 |
| WDR82 | WD repeat domain 82 | **1,009** | 0,75629 | 84,802 | 81,919 |
| CDC14A | cell division cycle 14A | **1,009** | 0,854281 | 6,204 | 5,555 |
| NOSIP | nitric oxide synthase interacting protein | **1,009** | 0,789681 | 34,120 | 36,522 |
| FAM161B | family with sequence similarity 161 member B | **1,009** | 0,764877 | 21,325 | 26,178 |
| ANOS1 | anosmin 1 | **1,009** | 0,833183 | 26,698 | 19,857 |
| GDE1 | glycerophosphodiester phosphodiesterase 1 | **1,009** | 0,751861 | 59,600 | 45,780 |
| GALNS | galactosamine (N-acetyl)-6-sulfatase | **1,009** | 0,758241 | 33,899 | 27,775 |
| BLCAP | bladder cancer associated protein | **1,009** | 0,763765 | 38,496 | 41,949 |
| PLBD1 | phospholipase B domain containing 1 | **1,009** | 0,815239 | 18,445 | 12,259 |
| EVA1C | eva-1 homolog C | **1,009** | 0,874372 | 3,157 | 3,512 |
| TECPR2 | tectonin beta-propeller repeat containing 2 | **1,009** | 0,788761 | 26,200 | 29,818 |
| TRIP4 | thyroid hormone receptor interactor 4 | **1,009** | 0,783222 | 15,122 | 14,366 |
| TSEN54 | tRNA splicing endonuclease subunit 54 | **1,009** | 0,891961 | 5,207 | 6,066 |
| RNF130 | ring finger protein 130 | **1,009** | 0,743207 | 74,389 | 62,062 |
| HLA-A | major histocompatibility complex, class I, A | **1,009** | 0,876199 | 8,475 | 5,172 |
| SMIM5 | small integral membrane protein 5 | **1,009** | 0,9524 | 0,443 | 0,255 |
| BBS5 | Bardet-Biedl syndrome 5 | **1,009** | 0,877498 | 5,041 | 6,577 |
| C11orf1 | chromosome 11 open reading frame 1 | **1,009** | 0,890847 | 4,265 | 6,321 |
| NATD1 | N-acetyltransferase domain containing 1 | **1,009** | 0,797501 | 15,454 | 12,898 |
| BRD3 | bromodomain containing 3 | **1,009** | 0,756455 | 32,902 | 33,138 |
| NAXD | NAD(P)HX dehydratase | **1,009** | 0,757509 | 35,560 | 32,691 |

| | | | | | |
|---|---|---|---|---|---|
| SELENOW | selenoprotein W | **1,009** | 0,804978 | 23,707 | 19,538 |
| MFSD12 | major facilitator superfamily domain containing 12 | **1,009** | 0,799026 | 24,538 | 25,285 |
| GRN | granulin precursor | **1,009** | 0,781869 | 264,045 | 158,859 |
| TMEM59 | transmembrane protein 59 | **1,009** | 0,753824 | 252,856 | 188,485 |
| CMTM4 | CKLF like MARVEL transmembrane domain containing 4 | **1,009** | 0,834439 | 30,686 | 31,159 |
| LINC01560 | long intergenic non-protein coding RNA 1560 | **1,009** | 0,920392 | 1,994 | 1,405 |
| LMBR1 | limb development membrane protein 1 | **1,009** | 0,824354 | 22,544 | 21,326 |
| GPS2P1 | G protein pathway suppressor 2 pseudogene 1 | **1,009** | 0,961665 | 0,499 | 0,319 |
| FLYWCH1 | FLYWCH-type zinc finger 1 | **1,009** | 0,794086 | 34,619 | 35,309 |
| EHHADH | enoyl-CoA hydratase and 3-hydroxyacyl CoA dehydrogenase | **1,009** | 0,881884 | 5,927 | 4,661 |
| ARL14EP | ADP ribosylation factor like GTPase 14 effector protein | **1,009** | 0,777273 | 22,488 | 18,261 |
| NUP85 | nucleoporin 85 | **1,009** | 0,839055 | 13,460 | 14,430 |
| SMARCC2 | SWI/SNF related, matrix associated, actin dependent regulator of chromatin subfamily c m | **1,009** | 0,802152 | 40,989 | 33,393 |
| RANBP3 | RAN binding protein 3 | **1,009** | 0,769573 | 38,219 | 38,885 |
| STK26 | serine/threonine kinase 26 | **1,008** | 0,787939 | 53,839 | 36,522 |
| TTC39C | tetratricopeptide repeat domain 39C | **1,008** | 0,831094 | 9,472 | 10,535 |
| LRRC57 | leucine rich repeat containing 57 | **1,008** | 0,82369 | 16,894 | 15,579 |
| PHKB | phosphorylase kinase regulatory subunit beta | **1,008** | 0,792106 | 26,809 | 26,498 |
| HIST1H1C | histone cluster 1 H1 family member c | **1,008** | 0,835438 | 51,070 | 68,128 |
| ELAVL1 | ELAV like RNA binding protein 1 | **1,008** | 0,754009 | 55,999 | 53,378 |
| TGOLN2 | trans-golgi network protein 2 | **1,008** | 0,756676 | 308,191 | 282,216 |
| TCTN1 | tectonic family member 1 | **1,008** | 0,764347 | 29,468 | 17,942 |
| L2HGDH | L-2-hydroxyglutarate dehydrogenase | **1,008** | 0,900871 | 4,154 | 4,086 |
| UHRF2 | ubiquitin like with PHD and ring finger domains 2 | **1,008** | 0,797086 | 37,942 | 37,799 |
| LDLRAD4 | low density lipoprotein receptor class A domain containing 4 | **1,008** | 0,850276 | 8,142 | 8,556 |
| RAB11B-AS1 | RAB11B antisense RNA 1 | **1,008** | 0,919179 | 2,160 | 2,299 |
| TMEM189 | transmembrane protein 189 | **1,008** | 0,80269 | 21,768 | 20,049 |
| SUN1 | Sad1 and UNC84 domain containing 1 | **1,008** | 0,787806 | 129,059 | 127,636 |
| OSMR-AS1 | OSMR antisense RNA 1 (head to head) | **1,008** | 0,929927 | 1,551 | 1,724 |
| PARG | poly(ADP-ribose) glycohydrolase | **1,008** | 0,836152 | 10,801 | 12,642 |
| PON2 | paraoxonase 2 | **1,008** | 0,806225 | 25,313 | 17,623 |
| PDGFA | platelet derived growth factor subunit A | **1,008** | 0,822679 | 21,658 | 24,199 |
| DCTN4 | dynactin subunit 4 | **1,008** | 0,764022 | 79,485 | 80,132 |

| | | | | | |
|---|---|---|---|---|---|
| HS3ST3B1 | heparan sulfate-glucosamine 3-sulfotransferase 3B1 | **1,008** | 0,813077 | 44,644 | 31,286 |
| BCDIN3D | BCDIN3 domain containing RNA methyltransferase | **1,008** | 0,823679 | 10,580 | 13,600 |
| SH3GLB1 | SH3 domain containing GRB2 like, endophilin B1 | **1,008** | 0,748402 | 72,395 | 60,977 |
| IGFBP2 | insulin like growth factor binding protein 2 | **1,008** | 0,877405 | 2,493 | 5,108 |
| CACNB4 | calcium voltage-gated channel auxiliary subunit beta 4 | **1,008** | 0,960115 | 0,554 | 0,894 |
| CAAP1 | caspase activity and apoptosis inhibitor 1 | **1,008** | 0,807686 | 13,349 | 14,047 |
| CLUAP1 | clusterin associated protein 1 | **1,008** | 0,813075 | 13,958 | 15,388 |
| PGGT1B | protein geranylgeranyltransferase type I subunit beta | **1,008** | 0,820521 | 15,454 | 18,644 |
| TAF3 | TATA-box binding protein associated factor 3 | **1,008** | 0,813667 | 20,273 | 31,031 |
| TMEM267 | transmembrane protein 267 | **1,008** | 0,845587 | 15,454 | 15,260 |
| PLAUR | plasminogen activator, urokinase receptor | **1,008** | 0,796634 | 221,893 | 210,130 |
| CNNM3 | cyclin and CBS domain divalent metal cation transport mediator 3 | **1,008** | 0,818689 | 29,634 | 27,966 |
| TBC1D22B | TBC1 domain family member 22B | **1,008** | 0,849704 | 10,690 | 11,365 |
| NPW | neuropeptide W | **1,008** | 0,967833 | 0,222 | 0,192 |
| PPID | peptidylprolyl isomerase D | **1,008** | 0,81253 | 17,337 | 19,857 |
| HCLS1 | hematopoietic cell-specific Lyn substrate 1 | **1,008** | 0,931972 | 1,551 | 1,660 |
| KANSL2 | KAT8 regulatory NSL complex subunit 2 | **1,008** | 0,834441 | 18,943 | 20,496 |
| MRPL53 | mitochondrial ribosomal protein L53 | **1,008** | 0,942969 | 1,662 | 1,852 |
| PTPRU | protein tyrosine phosphatase, receptor type U | **1,008** | 0,83041 | 29,689 | 26,498 |
| SUPT6H | SPT6 homolog, histone chaperone | **1,008** | 0,796713 | 111,279 | 134,787 |
| DPF2 | double PHD fingers 2 | **1,008** | 0,788019 | 41,210 | 40,417 |
| SFR1 | SWI5 dependent homologous recombination repair protein 1 | **1,008** | 0,85014 | 13,626 | 16,154 |
| PSMG4 | proteasome assembly chaperone 4 | **1,008** | 0,885226 | 4,653 | 5,236 |
| ADGRL2 | adhesion G protein-coupled receptor L2 | **1,008** | 0,907548 | 8,142 | 4,725 |
| PSKH1 | protein serine kinase H1 | **1,008** | 0,817139 | 23,375 | 25,412 |
| PODXL2 | podocalyxin like 2 | **1,008** | 0,933067 | 1,385 | 1,469 |
| C2CD3 | C2 calcium dependent domain containing 3 | **1,008** | 0,863529 | 20,439 | 25,476 |
| IFI6 | interferon alpha inducible protein 6 | **1,008** | 0,847387 | 12,574 | 12,387 |
| H1F0 | H1 histone family member 0 | **1,008** | 0,788497 | 231,531 | 221,623 |
| LINC00933 | long intergenic non-protein coding RNA 933 | **1,007** | 0,967633 | 0,388 | 0,383 |
| ITM2C | integral membrane protein 2C | **1,007** | 0,80447 | 162,293 | 87,794 |
| ARL6 | ADP ribosylation factor like GTPase 6 | **1,007** | 0,862141 | 8,641 | 10,471 |
| DLGAP1 | DLG associated protein 1 | **1,007** | 0,931407 | 0,886 | 1,724 |

| | | | | | |
|---|---|---|---|---|---|
| NLGN4X | neuroligin 4, X-linked | **1,007** | 0,937086 | 1,329 | 1,724 |
| LIG4 | DNA ligase 4 | **1,007** | 0,835971 | 18,611 | 18,261 |
| NDUFV3 | NADH:ubiquinone oxidoreductase subunit V3 | **1,007** | 0,813238 | 22,710 | 26,434 |
| ZBTB17 | zinc finger and BTB domain containing 17 | **1,007** | 0,800153 | 23,485 | 26,306 |
| ZXDC | ZXD family zinc finger C | **1,007** | 0,794337 | 30,021 | 32,947 |
| CDC42BPB | CDC42 binding protein kinase beta | **1,007** | 0,826155 | 99,647 | 98,010 |
| RAB11A | RAB11A, member RAS oncogene family | **1,007** | 0,805522 | 46,029 | 43,163 |
| PRRT3 | proline rich transmembrane protein 3 | **1,007** | 0,901623 | 4,542 | 3,129 |
| KAT5 | lysine acetyltransferase 5 | **1,007** | 0,776441 | 49,408 | 58,359 |
| FUT8-AS1 | FUT8 antisense RNA 1 | **1,007** | 0,95657 | 0,554 | 1,085 |
| MEGF8 | multiple EGF like domains 8 | **1,007** | 0,837728 | 106,017 | 90,156 |
| CPXM1 | carboxypeptidase X, M14 family member 1 | **1,007** | 0,933665 | 7,533 | 2,043 |
| DCAF1 | DDB1 and CUL4 associated factor 1 | **1,007** | 0,810865 | 37,942 | 36,267 |
| TMEM5 | transmembrane protein 5 | **1,007** | 0,840727 | 17,614 | 16,473 |
| GON4L | gon-4 like | **1,007** | 0,849017 | 24,593 | 26,753 |
| PAN2 | PAN2 poly(A) specific ribonuclease subunit | **1,007** | 0,830803 | 32,403 | 46,547 |
| KATNA1 | katanin catalytic subunit A1 | **1,007** | 0,857638 | 15,398 | 17,176 |
| ZNF527 | zinc finger protein 527 | **1,007** | 0,925034 | 3,434 | 3,065 |
| GIPC1 | GIPC PDZ domain containing family member 1 | **1,007** | 0,832166 | 34,730 | 30,073 |
| SKOR1 | SKI family transcriptional corepressor 1 | **1,007** | 0,970073 | 0,111 | 0,447 |
| NIPAL1 | NIPA like domain containing 1 | **1,007** | 0,922957 | 3,711 | 4,342 |
| GGH | gamma-glutamyl hydrolase | **1,007** | 0,892041 | 10,026 | 4,661 |
| RCN2 | reticulocalbin 2 | **1,007** | 0,807891 | 115,599 | 91,369 |
| NQO2 | N-ribosyldihydronicotinamide:quinone reductase 2 | **1,007** | 0,810523 | 47,192 | 17,814 |
| ZNF263 | zinc finger protein 263 | **1,007** | 0,821356 | 24,372 | 22,603 |
| RBBP4 | RB binding protein 4, chromatin remodeling factor | **1,007** | 0,832261 | 60,375 | 49,931 |
| WFS1 | wolframin ER transmembrane glycoprotein | **1,007** | 0,841741 | 64,917 | 63,275 |
| NTNG2 | netrin G2 | **1,007** | 0,955575 | 0,609 | 1,532 |
| TMEM254-AS1 | TMEM254 antisense RNA 1 | **1,007** | 0,940532 | 1,108 | 1,213 |
| DNAJA2 | DnaJ heat shock protein family (Hsp40) member A2 | **1,007** | 0,805986 | 56,276 | 51,144 |
| PYROXD2 | pyridine nucleotide-disulphide oxidoreductase domain 2 | **1,007** | 0,93853 | 1,219 | 1,341 |
| KLHL20 | kelch like family member 20 | **1,007** | 0,821056 | 26,255 | 24,838 |
| TSG101 | tumor susceptibility 101 | **1,007** | 0,800544 | 83,971 | 82,430 |

| | | | | | |
|---|---|---|---|---|---|
| MACROD1 | MACRO domain containing 1 | **1,007** | 0,938575 | 2,216 | 1,596 |
| SP2 | Sp2 transcription factor | **1,007** | 0,827723 | 19,996 | 18,133 |
| LINC00607 | long intergenic non-protein coding RNA 607 | **1,007** | 0,942344 | 1,606 | 1,915 |
| LRRC15 | leucine rich repeat containing 15 | **1,007** | 0,894448 | 53,285 | 21,326 |
| ELAC1 | elaC ribonuclease Z 1 | **1,007** | 0,878779 | 6,647 | 7,151 |
| PRKCI | protein kinase C iota | **1,007** | 0,848572 | 17,836 | 17,239 |
| ZNF670 | zinc finger protein 670 | **1,007** | 0,92676 | 3,323 | 3,384 |
| CENPO | centromere protein O | **1,007** | 0,90995 | 7,367 | 4,533 |
| NUDT8 | nudix hydrolase 8 | **1,007** | 0,951794 | 1,052 | 0,894 |
| FMOD | fibromodulin | **1,007** | 0,789446 | 2349,152 | 2072,056 |
| KCNMB3 | potassium calcium-activated channel subfamily M regulatory beta subunit 3 | **1,007** | 0,965605 | 1,052 | 1,405 |
| SLC38A1 | solute carrier family 38 member 1 | **1,006** | 0,857018 | 268,088 | 243,013 |
| TAF5 | TATA-box binding protein associated factor 5 | **1,006** | 0,899808 | 8,918 | 9,258 |
| PTPN9 | protein tyrosine phosphatase, non-receptor type 9 | **1,006** | 0,834161 | 32,902 | 25,859 |
| LARP7 | La ribonucleoprotein domain family member 7 | **1,006** | 0,877783 | 35,671 | 37,352 |
| ARID2 | AT-rich interaction domain 2 | **1,006** | 0,906061 | 16,008 | 17,303 |
| FAM98B | family with sequence similarity 98 member B | **1,006** | 0,854997 | 21,658 | 23,688 |
| SUCLA2 | succinate-CoA ligase ADP-forming beta subunit | **1,006** | 0,860054 | 21,159 | 18,006 |
| PGS1 | phosphatidylglycerophosphate synthase 1 | **1,006** | 0,873948 | 14,069 | 12,962 |
| RPL26 | ribosomal protein L26 | **1,006** | 0,884601 | 66,856 | 76,109 |
| FAM151B | family with sequence similarity 151 member B | **1,006** | 0,960221 | 2,271 | 1,213 |
| SF3A1 | splicing factor 3a subunit 1 | **1,006** | 0,826885 | 77,989 | 90,156 |
| BMP2K | BMP2 inducible kinase | **1,006** | 0,920913 | 11,355 | 8,237 |
| PAQR9 | progestin and adipoQ receptor family member 9 | **1,006** | 0,952599 | 1,163 | 1,915 |
| ST7L | suppression of tumorigenicity 7 like | **1,006** | 0,849834 | 16,229 | 17,623 |
| HAT1 | histone acetyltransferase 1 | **1,006** | 0,839946 | 38,607 | 38,246 |
| MGC16275 | uncharacterized protein MGC16275 | **1,006** | 0,973535 | 0,277 | 0,511 |
| HAO1 | hydroxyacid oxidase 1 | **1,006** | 0,97261 | 0,277 | 0,447 |
| CHI3L1 | chitinase 3 like 1 | **1,006** | 0,827774 | 2465,859 | 1413,381 |
| IDE | insulin degrading enzyme | **1,006** | 0,852919 | 32,071 | 29,499 |
| SPATA2L | spermatogenesis associated 2 like | **1,006** | 0,909628 | 5,041 | 4,533 |
| NEURL2 | neuralized E3 ubiquitin protein ligase 2 | **1,006** | 0,95608 | 1,219 | 1,660 |
| PIAS4 | protein inhibitor of activated STAT 4 | **1,006** | 0,84216 | 19,830 | 20,304 |

| | | | | | |
|---|---|---|---|---|---|
| MAD2L2 | mitotic arrest deficient 2 like 2 | **1,006** | 0,875187 | 18,556 | 15,899 |
| KMT5B | lysine methyltransferase 5B | **1,006** | 0,834143 | 40,989 | 40,800 |
| SLF1 | SMC5-SMC6 complex localization factor 1 | **1,006** | 0,931535 | 2,825 | 4,086 |
| PLD6 | phospholipase D family member 6 | **1,006** | 0,933987 | 2,714 | 2,554 |
| MAST2 | microtubule associated serine/threonine kinase 2 | **1,006** | 0,85769 | 44,312 | 53,570 |
| UGGT2 | UDP-glucose glycoprotein glucosyltransferase 2 | **1,006** | 0,861075 | 61,372 | 47,185 |
| METTL8 | methyltransferase like 8 | **1,006** | 0,917073 | 6,204 | 5,427 |
| LDLRAP1 | low density lipoprotein receptor adaptor protein 1 | **1,006** | 0,864604 | 26,089 | 17,623 |
| CIC | capicua transcriptional repressor | **1,006** | 0,870565 | 74,500 | 70,426 |
| ISCU | iron-sulfur cluster assembly enzyme | **1,006** | 0,849671 | 42,484 | 43,482 |
| SH3D21 | SH3 domain containing 21 | **1,006** | 0,889118 | 17,669 | 25,859 |
| EGF | epidermal growth factor | **1,006** | 0,964546 | 1,108 | 0,638 |
| STARD7 | StAR related lipid transfer domain containing 7 | **1,006** | 0,850391 | 56,443 | 64,233 |
| SSBP4 | single stranded DNA binding protein 4 | **1,006** | 0,89347 | 12,961 | 13,728 |
| CNOT3 | CCR4-NOT transcription complex subunit 3 | **1,006** | 0,951381 | 1,994 | 2,107 |
| SOX4 | SRY-box 4 | **1,006** | 0,887117 | 44,977 | 42,524 |
| GABPB1 | GA binding protein transcription factor beta subunit 1 | **1,006** | 0,876491 | 14,291 | 13,536 |
| COQ9 | coenzyme Q9 | **1,006** | 0,848314 | 20,439 | 23,752 |
| CALCOCO2 | calcium binding and coiled-coil domain 2 | **1,005** | 0,838652 | 70,013 | 63,339 |
| EFHC2 | EF-hand domain containing 2 | **1,005** | 0,966733 | 0,886 | 0,830 |
| ZFAND1 | zinc finger AN1-type containing 1 | **1,005** | 0,878249 | 13,792 | 17,176 |
| KAT8 | lysine acetyltransferase 8 | **1,005** | 0,848187 | 21,547 | 23,688 |
| NCOR2 | nuclear receptor corepressor 2 | **1,005** | 0,892359 | 69,016 | 79,621 |
| SAP30 | Sin3A associated protein 30 | **1,005** | 0,904558 | 16,506 | 14,111 |
| ARHGEF18 | Rho/Rac guanine nucleotide exchange factor 18 | **1,005** | 0,9444 | 2,382 | 2,426 |
| NIFK-AS1 | NIFK antisense RNA 1 | **1,005** | 0,933867 | 3,988 | 3,576 |
| PNP | purine nucleoside phosphorylase | **1,005** | 0,862097 | 84,858 | 73,044 |
| DNAJC21 | DnaJ heat shock protein family (Hsp40) member C21 | **1,005** | 0,858576 | 31,960 | 26,498 |
| SHQ1 | SHQ1, H/ACA ribonucleoprotein assembly factor | **1,005** | 0,91298 | 13,404 | 11,748 |
| TADA2B | transcriptional adaptor 2B | **1,005** | 0,864793 | 27,252 | 24,455 |
| ALDOA | aldolase, fructose-bisphosphate A | **1,005** | 0,915553 | 9,416 | 13,281 |
| ABCD1 | ATP binding cassette subfamily D member 1 | **1,005** | 0,924592 | 14,124 | 9,577 |
| TMF1 | TATA element modulatory factor 1 | **1,005** | 0,867479 | 104,300 | 108,992 |

| | | | | | |
|---|---|---|---|---|---|
| C1QL1 | complement C1q like 1 | **1,005** | 0,943319 | 3,379 | 2,171 |
| ELAC2 | elaC ribonuclease Z 2 | **1,005** | 0,893114 | 30,243 | 28,796 |
| ELMOD3 | ELMO domain containing 3 | **1,005** | 0,896828 | 10,413 | 10,918 |
| FAM66C | family with sequence similarity 66 member C | **1,005** | 0,954579 | 1,274 | 1,724 |
| MAPK11 | mitogen-activated protein kinase 11 | **1,005** | 0,947902 | 5,096 | 3,384 |
| KMT2D | lysine methyltransferase 2D | **1,005** | 0,923224 | 88,126 | 104,586 |
| MKRN2 | makorin ring finger protein 2 | **1,005** | 0,857013 | 38,995 | 31,606 |
| KCTD10 | potassium channel tetramerization domain containing 10 | **1,005** | 0,85643 | 73,004 | 75,854 |
| GALK2 | galactokinase 2 | **1,005** | 0,909522 | 9,693 | 8,811 |
| YTHDF3 | YTH N6-methyladenosine RNA binding protein 3 | **1,005** | 0,870968 | 81,922 | 79,174 |
| DUSP8P5 | dual specificity phosphatase 8 pseudogene 5 | **1,005** | 0,969874 | 0,775 | 0,894 |
| CCDC117 | coiled-coil domain containing 117 | **1,005** | 0,86793 | 24,538 | 20,368 |
| ERG | ERG, ETS transcription factor | **1,005** | 0,891261 | 10,358 | 15,516 |
| KIAA2013 | KIAA2013 | **1,005** | 0,876501 | 36,281 | 26,370 |
| UBQLN2 | ubiquilin 2 | **1,005** | 0,861432 | 42,650 | 34,734 |
| CARHSP1 | calcium regulated heat stable protein 1 | **1,005** | 0,87632 | 73,226 | 60,338 |
| KIF13A | kinesin family member 13A | **1,005** | 0,887694 | 34,176 | 29,882 |
| CREG1 | cellular repressor of E1A stimulated genes 1 | **1,005** | 0,857594 | 80,980 | 66,979 |
| ETFRF1 | electron transfer flavoprotein regulatory factor 1 | **1,005** | 0,923046 | 6,370 | 9,003 |
| KLC1 | kinesin light chain 1 | **1,005** | 0,858754 | 43,260 | 37,927 |
| NADK2 | NAD kinase 2, mitochondrial | **1,005** | 0,876186 | 20,605 | 18,006 |
| MAU2 | MAU2 sister chromatid cohesion factor | **1,005** | 0,863012 | 50,460 | 59,189 |
| DIO3OS | DIO3 opposite strand/antisense RNA (head to head) | **1,005** | 0,916265 | 6,813 | 7,917 |
| CUL1 | cullin 1 | **1,005** | 0,856371 | 63,477 | 69,213 |
| PDZRN3 | PDZ domain containing ring finger 3 | **1,005** | 0,878314 | 117,372 | 68,894 |
| ZUFSP | zinc finger with UFM1 specific peptidase domain | **1,005** | 0,915948 | 6,868 | 9,322 |
| EDEM1 | ER degradation enhancing alpha-mannosidase like protein 1 | **1,005** | 0,889148 | 66,745 | 57,720 |
| IMPAD1 | inositol monophosphatase domain containing 1 | **1,005** | 0,856444 | 195,029 | 162,689 |
| MLEC | malectin | **1,005** | 0,862309 | 181,901 | 181,270 |
| IL15RA | interleukin 15 receptor subunit alpha | **1,005** | 0,884644 | 31,462 | 45,844 |
| MIA | melanoma inhibitory activity | **1,005** | 0,917404 | 20,661 | 14,047 |
| ATPAF2 | ATP synthase mitochondrial F1 complex assembly factor 2 | **1,005** | 0,932586 | 4,985 | 3,959 |
| LYRM7 | LYR motif containing 7 | **1,005** | 0,878576 | 16,728 | 21,645 |

| | | | | | |
|---|---|---|---|---|---|
| JAM2 | junctional adhesion molecule 2 | **1,005** | 0,945528 | 3,545 | 4,533 |
| TTC41P | tetratricopeptide repeat domain 41, pseudogene | **1,005** | 0,968316 | 0,886 | 1,085 |
| SNRNP70 | small nuclear ribonucleoprotein U1 subunit 70 | **1,005** | 0,880466 | 95,659 | 108,672 |
| ARL2BP | ADP ribosylation factor like GTPase 2 binding protein | **1,005** | 0,859437 | 97,044 | 81,409 |
| ZNF691 | zinc finger protein 691 | **1,005** | 0,915655 | 7,256 | 6,896 |
| MATR3 | matrin 3 | **1,005** | 0,968706 | 1,274 | 1,469 |
| ACAD8 | acyl-CoA dehydrogenase family member 8 | **1,004** | 0,88901 | 19,664 | 19,857 |
| FAM109A | family with sequence similarity 109 member A | **1,004** | 0,931746 | 10,469 | 9,705 |
| BCAR3 | breast cancer anti-estrogen resistance 3 | **1,004** | 0,926445 | 10,247 | 8,492 |
| PHIP | pleckstrin homology domain interacting protein | **1,004** | 0,870861 | 48,799 | 51,463 |
| TCTEX1D2 | Tctex1 domain containing 2 | **1,004** | 0,959066 | 1,606 | 1,213 |
| UBE4A | ubiquitination factor E4A | **1,004** | 0,88332 | 47,912 | 56,124 |
| GORAB | golgin, RAB6 interacting | **1,004** | 0,891588 | 25,369 | 28,413 |
| GJC2 | gap junction protein gamma 2 | **1,004** | 0,965392 | 1,385 | 1,022 |
| HDAC4 | histone deacetylase 4 | **1,004** | 0,907303 | 15,509 | 18,836 |
| SNX33 | sorting nexin 33 | **1,004** | 0,872994 | 91,726 | 90,220 |
| PDE1C | phosphodiesterase 1C | **1,004** | 0,950385 | 3,046 | 0,958 |
| LBR | lamin B receptor | **1,004** | 0,931028 | 10,026 | 8,875 |
| KBTBD3 | kelch repeat and BTB domain containing 3 | **1,004** | 0,964383 | 2,770 | 2,107 |
| SPIDR | scaffolding protein involved in DNA repair | **1,004** | 0,892574 | 18,500 | 18,389 |
| CYTL1 | cytokine like 1 | **1,004** | 0,901918 | 115,212 | 45,461 |
| ACADVL | acyl-CoA dehydrogenase, very long chain | **1,004** | 0,887558 | 198,574 | 123,677 |
| AEBP2 | AE binding protein 2 | **1,004** | 0,889842 | 32,680 | 28,924 |
| ARPC5 | actin related protein 2/3 complex subunit 5 | **1,004** | 0,870432 | 74,832 | 69,979 |
| ACLY | ATP citrate lyase | **1,004** | 0,903523 | 147,061 | 127,572 |
| TBC1D14 | TBC1 domain family member 14 | **1,004** | 0,907943 | 24,372 | 19,857 |
| TEPSIN | TEPSIN, adaptor related protein complex 4 accessory protein | **1,004** | 0,911657 | 12,463 | 12,323 |
| DNAH7 | dynein axonemal heavy chain 7 | **1,004** | 0,972577 | 1,219 | 1,022 |
| EYA3 | EYA transcriptional coactivator and phosphatase 3 | **1,004** | 0,903117 | 20,882 | 21,964 |
| SNRNP200 | small nuclear ribonucleoprotein U5 subunit 200 | **1,004** | 0,896827 | 137,866 | 156,879 |
| BBS10 | Bardet-Biedl syndrome 10 | **1,004** | 0,896471 | 24,150 | 30,967 |
| SLC43A1 | solute carrier family 43 member 1 | **1,004** | 0,957658 | 1,994 | 2,107 |
| HIF1AN | hypoxia inducible factor 1 alpha subunit inhibitor | **1,004** | 0,887688 | 55,889 | 62,956 |

| | | | | | |
|---|---|---|---|---|---|
| GIGYF1 | GRB10 interacting GYF protein 1 | **1,004** | 0,880602 | 59,157 | 64,680 |
| AXIN1 | axin 1 | **1,004** | 0,89643 | 24,704 | 24,518 |
| LRRC14 | leucine rich repeat containing 14 | **1,004** | 0,902362 | 23,042 | 23,114 |
| CHAD | chondroadherin | **1,004** | 0,889088 | 353,611 | 272,000 |
| SPIN3 | spindlin family member 3 | **1,004** | 0,939879 | 8,696 | 10,216 |
| SUMO3 | small ubiquitin-like modifier 3 | **1,004** | 0,886949 | 53,285 | 42,652 |
| IMPA1 | inositol monophosphatase 1 | **1,004** | 0,916291 | 33,511 | 23,624 |
| NUBPL | nucleotide binding protein like | **1,004** | 0,93578 | 3,213 | 7,981 |
| SPTSSA | serine palmitoyltransferase small subunit A | **1,004** | 0,913881 | 24,538 | 22,858 |
| RPE | ribulose-5-phosphate-3-epimerase | **1,004** | 0,906942 | 20,771 | 20,751 |
| OTUD5 | OTU deubiquitinase 5 | **1,004** | 0,889098 | 47,857 | 38,757 |
| ZDHHC5 | zinc finger DHHC-type containing 5 | **1,004** | 0,884716 | 98,705 | 97,754 |
| KANSL3 | KAT8 regulatory NSL complex subunit 3 | **1,004** | 0,907584 | 82,864 | 84,856 |
| C15orf59 | chromosome 15 open reading frame 59 | **1,004** | 0,977104 | 0,942 | 0,702 |
| REXO1 | RNA exonuclease 1 homolog | **1,004** | 0,890531 | 25,036 | 30,009 |
| RTN4IP1 | reticulon 4 interacting protein 1 | **1,004** | 0,958378 | 2,603 | 3,576 |
| SNAPC4 | small nuclear RNA activating complex polypeptide 4 | **1,004** | 0,932473 | 8,973 | 12,387 |
| C5orf34 | chromosome 5 open reading frame 34 | **1,004** | 0,967758 | 2,216 | 2,809 |
| CNOT1 | CCR4-NOT transcription complex subunit 1 | **1,004** | 0,913703 | 109,119 | 118,952 |
| PSMD12 | proteasome 26S subunit, non-ATPase 12 | **1,004** | 0,894369 | 71,509 | 77,258 |
| ABHD10 | abhydrolase domain containing 10 | **1,004** | 0,924691 | 19,497 | 22,794 |
| RPS3A | ribosomal protein S3A | **1,004** | 0,913786 | 113,827 | 124,124 |
| RAB5C | RAB5C, member RAS oncogene family | **1,004** | 0,904677 | 25,923 | 24,008 |
| MTX3 | metaxin 3 | **1,004** | 0,914718 | 22,488 | 26,753 |
| CDC37 | cell division cycle 37 | **1,004** | 0,895258 | 111,113 | 116,781 |
| SLC26A2 | solute carrier family 26 member 2 | **1,004** | 0,924631 | 56,443 | 54,209 |
| YTHDF2 | YTH N6-methyladenosine RNA binding protein 2 | **1,004** | 0,893872 | 51,347 | 47,249 |
| SRP14 | signal recognition particle 14 | **1,004** | 0,897287 | 90,120 | 92,391 |
| PPP4R1 | protein phosphatase 4 regulatory subunit 1 | **1,004** | 0,909593 | 45,420 | 35,437 |
| KCTD11 | potassium channel tetramerization domain containing 11 | **1,004** | 0,910342 | 24,482 | 21,709 |
| UBA2 | ubiquitin like modifier activating enzyme 2 | **1,004** | 0,891327 | 58,049 | 54,400 |
| THAP7 | THAP domain containing 7 | **1,004** | 0,914968 | 26,421 | 27,455 |
| PIAS2 | protein inhibitor of activated STAT 2 | **1,004** | 0,935137 | 9,859 | 10,216 |

| | | | | | |
|---|---|---|---|---|---|
| NNT-AS1 | NNT antisense RNA 1 | **1,004** | 0,938741 | 13,737 | 9,705 |
| SLC35F5 | solute carrier family 35 member F5 | **1,004** | 0,896557 | 66,634 | 60,210 |
| APOOL | apolipoprotein O like | **1,004** | 0,919421 | 17,780 | 20,113 |
| PRDX6 | peroxiredoxin 6 | **1,004** | 0,897675 | 118,590 | 68,511 |
| PSMB1 | proteasome subunit beta 1 | **1,004** | 0,909385 | 136,814 | 156,368 |
| ILDR2 | immunoglobulin like domain containing receptor 2 | **1,004** | 0,987471 | 0,111 | 0,383 |
| SYF2 | SYF2 pre-mRNA splicing factor | **1,003** | 0,910485 | 38,829 | 37,352 |
| DLEU2 | deleted in lymphocytic leukemia 2 (non-protein coding) | **1,003** | 0,969226 | 1,828 | 1,405 |
| PLEC | plectin | **1,003** | 0,933636 | 1115,669 | 947,149 |
| ABHD14B | abhydrolase domain containing 14B | **1,003** | 0,930455 | 14,124 | 18,261 |
| NAP1L5 | nucleosome assembly protein 1 like 5 | **1,003** | 0,943532 | 5,151 | 6,449 |
| LPCAT3 | lysophosphatidylcholine acyltransferase 3 | **1,003** | 0,94081 | 11,244 | 10,854 |
| SMYD4 | SET and MYND domain containing 4 | **1,003** | 0,927192 | 13,571 | 16,601 |
| TMEM33 | transmembrane protein 33 | **1,003** | 0,914829 | 50,904 | 52,293 |
| MON1B | MON1 homolog B, secretory trafficking associated | **1,003** | 0,90576 | 58,270 | 54,847 |
| TRAPPC11 | trafficking protein particle complex 11 | **1,003** | 0,911555 | 32,403 | 27,392 |
| IDH3A | isocitrate dehydrogenase 3 (NAD(+)) alpha | **1,003** | 0,906202 | 29,634 | 31,223 |
| TTC17 | tetratricopeptide repeat domain 17 | **1,003** | 0,915087 | 69,238 | 68,256 |
| GALNT14 | polypeptide N-acetylgalactosaminyltransferase 14 | **1,003** | 0,97892 | 0,499 | 0,830 |
| TPM3 | tropomyosin 3 | **1,003** | 0,892144 | 159,302 | 142,066 |
| SAMD4B | sterile alpha motif domain containing 4B | **1,003** | 0,900681 | 66,690 | 65,191 |
| CCDC115 | coiled-coil domain containing 115 | **1,003** | 0,934345 | 13,349 | 14,111 |
| OIP5-AS1 | OIP5 antisense RNA 1 | **1,003** | 0,924216 | 76,882 | 79,174 |
| TM7SF3 | transmembrane 7 superfamily member 3 | **1,003** | 0,908259 | 37,610 | 31,989 |
| TNPO1 | transportin 1 | **1,003** | 0,916447 | 157,530 | 146,982 |
| MALL | mal, T-cell differentiation protein like | **1,003** | 0,948865 | 6,924 | 6,130 |
| UQCRHL | ubiquinol-cytochrome c reductase hinge protein like | **1,003** | 0,975444 | 1,440 | 1,852 |
| MBLAC1 | metallo-beta-lactamase domain containing 1 | **1,003** | 0,970534 | 1,717 | 1,596 |
| GLRX | glutaredoxin | **1,003** | 0,913537 | 122,911 | 80,259 |
| NRN1 | neuritin 1 | **1,003** | 0,928423 | 100,810 | 68,064 |
| RPL30 | ribosomal protein L30 | **1,003** | 0,924522 | 257,343 | 286,175 |
| KLHL13 | kelch like family member 13 | **1,003** | 0,972314 | 1,939 | 4,853 |
| MTERF3 | mitochondrial transcription termination factor 3 | **1,003** | 0,945422 | 11,410 | 15,388 |

| | | | | | |
|---|---|---|---|---|---|
| CRTC2 | CREB regulated transcription coactivator 2 | **1,003** | 0,922456 | 23,707 | 26,753 |
| ST7 | suppression of tumorigenicity 7 | **1,003** | 0,927397 | 14,734 | 14,366 |
| HDHD3 | haloacid dehalogenase like hydrolase domain containing 3 | **1,003** | 0,964092 | 5,262 | 4,853 |
| KIAA1468 | KIAA1468 | **1,003** | 0,934193 | 19,774 | 21,262 |
| ASAP2 | ArfGAP with SH3 domain, ankyrin repeat and PH domain 2 | **1,003** | 0,934701 | 83,362 | 73,044 |
| HAUS6 | HAUS augmin like complex subunit 6 | **1,003** | 0,960443 | 8,641 | 6,960 |
| NFATC2IP | nuclear factor of activated T-cells 2 interacting protein | **1,003** | 0,927013 | 22,488 | 24,965 |
| ZZEF1 | zinc finger ZZ-type and EF-hand domain containing 1 | **1,003** | 0,933045 | 39,936 | 43,163 |
| FBLN7 | fibulin 7 | **1,003** | 0,955607 | 12,352 | 4,023 |
| PPT1 | palmitoyl-protein thioesterase 1 | **1,003** | 0,913602 | 102,915 | 85,303 |
| KCTD2 | potassium channel tetramerization domain containing 2 | **1,003** | 0,917698 | 26,864 | 30,520 |
| BCLAF1 | BCL2 associated transcription factor 1 | **1,003** | 0,913038 | 95,493 | 97,180 |
| SAMD9 | sterile alpha motif domain containing 9 | **1,003** | 0,956077 | 12,684 | 14,175 |
| SF3B1 | splicing factor 3b subunit 1 | **1,003** | 0,921935 | 211,147 | 207,895 |
| NUP205 | nucleoporin 205 | **1,003** | 0,932547 | 36,281 | 38,693 |
| SNAPIN | SNAP associated protein | **1,003** | 0,940611 | 17,614 | 16,920 |
| B3GAT2 | beta-1,3-glucuronyltransferase 2 | **1,003** | 0,96808 | 4,653 | 4,597 |
| UBTF | upstream binding transcription factor, RNA polymerase I | **1,003** | 0,932801 | 44,478 | 40,800 |
| PPP2CA | protein phosphatase 2 catalytic subunit alpha | **1,003** | 0,926343 | 95,603 | 92,263 |
| NCBP3 | nuclear cap binding subunit 3 | **1,003** | 0,93661 | 31,295 | 32,053 |
| ZBED5 | zinc finger BED-type containing 5 | **1,003** | 0,936789 | 35,284 | 32,053 |
| DNMT3A | DNA methyltransferase 3 alpha | **1,003** | 0,967071 | 7,145 | 7,215 |
| GMEB1 | glucocorticoid modulatory element binding protein 1 | **1,003** | 0,939323 | 19,276 | 20,815 |
| MAPK1IP1L | mitogen-activated protein kinase 1 interacting protein 1 like | **1,002** | 0,927922 | 64,253 | 57,529 |
| CLOCK | clock circadian regulator | **1,002** | 0,95313 | 28,360 | 27,455 |
| USP33 | ubiquitin specific peptidase 33 | **1,002** | 0,939401 | 31,960 | 29,946 |
| RAD51D | RAD51 paralog D | **1,002** | 0,963037 | 6,591 | 6,640 |
| TMCO3 | transmembrane and coiled-coil domains 3 | **1,002** | 0,92233 | 153,375 | 88,687 |
| C1orf43 | chromosome 1 open reading frame 43 | **1,002** | 0,924362 | 196,192 | 190,081 |
| SNHG5 | small nucleolar RNA host gene 5 | **1,002** | 0,941677 | 72,395 | 32,116 |
| ZDHHC6 | zinc finger DHHC-type containing 6 | **1,002** | 0,943842 | 26,532 | 23,497 |
| SYPL1 | synaptophysin like 1 | **1,002** | 0,940442 | 81,867 | 68,319 |
| CYP20A1 | cytochrome P450 family 20 subfamily A member 1 | **1,002** | 0,952998 | 15,509 | 15,388 |

| | | | | | |
|---|---|---|---|---|---|
| GPHN | gephyrin | **1,002** | 0,964384 | 5,207 | 5,491 |
| GMFG | glia maturation factor gamma | **1,002** | 0,986344 | 0,831 | 0,702 |
| ZNF768 | zinc finger protein 768 | **1,002** | 0,951373 | 27,141 | 24,582 |
| CENPBD1 | CENPB DNA-binding domain containing 1 | **1,002** | 0,954382 | 14,678 | 12,898 |
| BTD | biotinidase | **1,002** | 0,948115 | 13,958 | 16,920 |
| UFL1 | UFM1 specific ligase 1 | **1,002** | 0,943819 | 38,330 | 39,651 |
| XPNPEP3 | X-prolyl aminopeptidase 3 | **1,002** | 0,959781 | 11,466 | 12,706 |
| ZNF131 | zinc finger protein 131 | **1,002** | 0,954763 | 18,500 | 17,048 |
| RARRES3 | retinoic acid receptor responder 3 | **1,002** | 0,976178 | 1,883 | 3,959 |
| SNTB1 | syntrophin beta 1 | **1,002** | 0,957667 | 17,337 | 19,474 |
| FBXL14 | F-box and leucine rich repeat protein 14 | **1,002** | 0,962499 | 8,973 | 9,897 |
| LATS2 | large tumor suppressor kinase 2 | **1,002** | 0,951413 | 72,617 | 65,063 |
| MAGOH | mago homolog, exon junction complex core component | **1,002** | 0,951855 | 22,267 | 27,200 |
| PGK1 | phosphoglycerate kinase 1 | **1,002** | 0,934291 | 470,761 | 337,894 |
| ZNF749 | zinc finger protein 749 | **1,002** | 0,981934 | 2,160 | 2,107 |
| EXOC2 | exocyst complex component 2 | **1,002** | 0,951821 | 24,095 | 22,986 |
| ZBED6 | zinc finger BED-type containing 6 | **1,002** | 0,989748 | 1,274 | 1,979 |
| SUCLG2-AS1 | SUCLG2 antisense RNA 1 (head to head) | **1,002** | 0,990522 | 0,942 | 0,638 |
| DNAL1 | dynein axonemal light chain 1 | **1,002** | 0,959907 | 19,442 | 20,815 |
| PLEKHB1 | pleckstrin homology domain containing B1 | **1,002** | 0,989251 | 1,052 | 0,638 |
| CYTH2 | cytohesin 2 | **1,002** | 0,964906 | 21,214 | 19,155 |
| ZCCHC8 | zinc finger CCHC-type containing 8 | **1,002** | 0,954646 | 27,252 | 28,669 |
| PEX5 | peroxisomal biogenesis factor 5 | **1,002** | 0,952843 | 40,712 | 38,310 |
| PITPNA | phosphatidylinositol transfer protein alpha | **1,002** | 0,951559 | 76,438 | 73,938 |
| DDIAS | DNA damage induced apoptosis suppressor | **1,002** | 0,987387 | 2,160 | 2,235 |
| SMU1 | DNA replication regulator and spliceosomal factor | **1,002** | 0,955401 | 45,863 | 43,290 |
| MIATNB | MIAT neighbor (non-protein coding) | **1,002** | 0,986222 | 1,108 | 1,724 |
| EML2 | echinoderm microtubule associated protein like 2 | **1,002** | 0,965984 | 20,605 | 18,006 |
| RSAD1 | radical S-adenosyl methionine domain containing 1 | **1,002** | 0,957014 | 22,710 | 22,539 |
| ZNF681 | zinc finger protein 681 | **1,002** | 0,990308 | 1,440 | 0,958 |
| FLNB | filamin B | **1,002** | 0,968988 | 20,882 | 27,328 |
| GPR162 | G protein-coupled receptor 162 | **1,002** | 0,991956 | 0,997 | 0,255 |
| YY1AP1 | YY1 associated protein 1 | **1,001** | 0,963825 | 24,206 | 24,327 |

| | | | | | |
|---|---|---|---|---|---|
| UFM1 | ubiquitin fold modifier 1 | **1,001** | 0,962383 | 92,502 | 104,458 |
| SLC9A7 | solute carrier family 9 member A7 | **1,001** | 0,971003 | 24,759 | 17,559 |
| CCDC34 | coiled-coil domain containing 34 | **1,001** | 0,989137 | 3,379 | 2,873 |
| MED29 | mediator complex subunit 29 | **1,001** | 0,959828 | 40,269 | 45,333 |
| ZNF106 | zinc finger protein 106 | **1,001** | 0,975054 | 49,297 | 54,655 |
| KIF16B | kinesin family member 16B | **1,001** | 0,975045 | 12,352 | 11,238 |
| THRAP3 | thyroid hormone receptor associated protein 3 | **1,001** | 0,965602 | 128,948 | 129,679 |
| CTDSPL | CTD small phosphatase like | **1,001** | 0,965075 | 26,366 | 28,796 |
| PASK | PAS domain containing serine/threonine kinase | **1,001** | 0,988147 | 2,714 | 3,001 |
| ZNF266 | zinc finger protein 266 | **1,001** | 0,973814 | 10,690 | 15,132 |
| RBL2 | RB transcriptional corepressor like 2 | **1,001** | 0,968843 | 37,111 | 35,117 |
| PDCD4 | programmed cell death 4 | **1,001** | 0,962913 | 61,372 | 55,039 |
| CSK | CSK, non-receptor tyrosine kinase | **1,001** | 0,966476 | 53,175 | 55,230 |
| NBR1 | NBR1, autophagy cargo receptor | **1,001** | 0,964 | 219,954 | 207,448 |
| ZNF771 | zinc finger protein 771 | **1,001** | 0,98332 | 7,865 | 6,704 |
| ANKRD16 | ankyrin repeat domain 16 | **1,001** | 0,986433 | 2,493 | 3,320 |
| SELL | selectin L | **1,001** | 0,9911 | 0,775 | 1,149 |
| ZNF589 | zinc finger protein 589 | **1,001** | 0,985145 | 3,933 | 6,896 |
| UQCRB | ubiquinol-cytochrome c reductase binding protein | **1,001** | 0,973696 | 79,042 | 81,919 |
| MAN2C1 | mannosidase alpha class 2C member 1 | **1,001** | 0,970103 | 56,553 | 56,635 |
| USP36 | ubiquitin specific peptidase 36 | **1,001** | 0,970656 | 51,679 | 64,169 |
| COG8 | component of oligomeric golgi complex 8 | **1,001** | 0,983883 | 6,204 | 7,726 |
| ECE1 | endothelin converting enzyme 1 | **1,001** | 0,975504 | 76,549 | 84,473 |
| CCDC80 | coiled-coil domain containing 80 | **1,001** | 0,968423 | 410,441 | 268,808 |
| IFI35 | interferon induced protein 35 | **1,001** | 0,98042 | 11,632 | 9,961 |
| PEX16 | peroxisomal biogenesis factor 16 | **1,001** | 0,97977 | 20,328 | 20,240 |
| PRIM1 | primase (DNA) subunit 1 | **1,001** | 0,991065 | 1,828 | 2,746 |
| SOCS3 | suppressor of cytokine signaling 3 | **1,001** | 0,974099 | 105,352 | 105,863 |
| CSNK1D | casein kinase 1 delta | **1,001** | 0,973587 | 81,424 | 94,306 |
| DHX9 | DExH-box helicase 9 | **1,001** | 0,972114 | 100,644 | 112,759 |
| IRF2 | interferon regulatory factor 2 | **1,001** | 0,980285 | 26,532 | 24,008 |
| VPS29 | VPS29, retromer complex component | **1,001** | 0,981327 | 38,385 | 30,329 |
| IBSP | integrin binding sialoprotein | **1,001** | 0,986666 | 39,216 | 1,852 |

| | | | | | |
|---|---|---|---|---|---|
| UHRF1BP1 | UHRF1 binding protein 1 | **1,001** | 0,989822 | 13,294 | 12,578 |
| ZNF41 | zinc finger protein 41 | **1,001** | 0,990721 | 9,361 | 7,343 |
| AP1G1 | adaptor related protein complex 1 gamma 1 subunit | **1,001** | 0,9838 | 82,864 | 81,025 |
| LAMTOR5 | late endosomal/lysosomal adaptor, MAPK and MTOR activator 5 | **1,001** | 0,985986 | 52,399 | 49,994 |
| ANKRD52 | ankyrin repeat domain 52 | **1,001** | 0,986308 | 39,382 | 32,563 |
| TMEM220 | transmembrane protein 220 | **1,001** | 0,99046 | 3,046 | 6,066 |
| NIT1 | nitrilase 1 | **1,001** | 0,984336 | 19,442 | 17,239 |
| EPHX2 | epoxide hydrolase 2 | **1,001** | 0,99306 | 2,548 | 5,619 |
| FGL2 | fibrinogen like 2 | **1,001** | 0,996369 | 0,499 | 0,894 |
| ARHGAP33 | Rho GTPase activating protein 33 | **1,001** | 0,996127 | 1,496 | 0,830 |
| ATF5 | activating transcription factor 5 | **1,001** | 0,98815 | 38,330 | 26,370 |
| PRKAR2A | protein kinase cAMP-dependent type II regulatory subunit alpha | **1,001** | 0,9854 | 46,140 | 46,291 |
| INPP5F | inositol polyphosphate-5-phosphatase F | **1,001** | 0,986411 | 30,797 | 31,861 |
| TRIM44 | tripartite motif containing 44 | **1,001** | 0,984835 | 89,787 | 96,286 |
| DYNC2LI1 | dynein cytoplasmic 2 light intermediate chain 1 | **1,000** | 0,99187 | 7,422 | 10,152 |
| GSTO1 | glutathione S-transferase omega 1 | **1,000** | 0,990137 | 130,610 | 115,057 |
| MCMBP | minichromosome maintenance complex binding protein | **1,000** | 0,987989 | 46,195 | 51,718 |
| RPS12 | ribosomal protein S12 | **1,000** | 0,990491 | 442,180 | 496,177 |
| AAR2 | AAR2 splicing factor homolog | **1,000** | 0,989549 | 32,071 | 38,693 |
| SNX19 | sorting nexin 19 | **1,000** | 0,991029 | 68,296 | 72,661 |
| USP42 | ubiquitin specific peptidase 42 | **1,000** | 0,994346 | 14,124 | 14,941 |
| PI4KA | phosphatidylinositol 4-kinase alpha | **1,000** | 0,993633 | 40,823 | 44,886 |
| SPATA33 | spermatogenesis associated 33 | **1,000** | 0,996135 | 5,096 | 5,172 |
| SMIM2-AS1 | SMIM2 antisense RNA 1 | **1,000** | 0,998713 | 0,277 | 0,958 |
| TEX10 | testis expressed 10 | **1,000** | 0,994956 | 21,214 | 24,327 |
| RPL10A | ribosomal protein L10a | **1,000** | 0,994659 | 149,498 | 144,620 |
| ABHD2 | abhydrolase domain containing 2 | **1,000** | 0,994417 | 282,767 | 286,303 |
| FAM168A | family with sequence similarity 168 member A | **1,000** | 0,996652 | 35,893 | 33,266 |
| KYAT3 | kynurenine aminotransferase 3 | **1,000** | 0,996394 | 24,593 | 17,303 |
| SPG7 | SPG7, paraplegin matrix AAA peptidase subunit | **1,000** | 0,9961 | 35,837 | 45,142 |
| WDHD1 | WD repeat and HMG-box DNA binding protein 1 | **1,000** | 0,998591 | 3,656 | 3,129 |
| NUDT19 | nudix hydrolase 19 | **1,000** | 0,997453 | 10,469 | 11,685 |
| TUSC2 | tumor suppressor candidate 2 | **1,000** | 0,997613 | 24,593 | 24,646 |

| | | | | | |
|---|---|---|---|---|---|
| PTK2 | protein tyrosine kinase 2 | **1,000** | 0,997926 | 52,344 | 45,780 |
| CPNE1 | copine 1 | **1,000** | 0,998468 | 35,948 | 24,263 |
| MAP4K4 | mitogen-activated protein kinase kinase kinase kinase 4 | **1,000** | 0,999248 | 277,172 | 258,081 |
| TCEAL1 | transcription elongation factor A like 1 | **1,000** | 0,999652 | 7,478 | 7,087 |
| NAF1 | nuclear assembly factor 1 ribonucleoprotein | **1,000** | 0,999833 | 11,798 | 9,961 |
| FES | FES proto-oncogene, tyrosine kinase | **1,000** | 0,999801 | 0,720 | 1,277 |
| C5AR1 | complement C5a receptor 1 | **1,000** | 0,999353 | 1,163 | 0,383 |
| SMG7 | SMG7, nonsense mediated mRNA decay factor | **1,000** | 0,996888 | 43,592 | 44,503 |
| THEM4 | thioesterase superfamily member 4 | **1,000** | 0,997879 | 7,588 | 9,322 |
| HNRNPLL | heterogeneous nuclear ribonucleoprotein L like | **1,000** | 0,99612 | 30,409 | 31,350 |
| MDH1 | malate dehydrogenase 1 | **1,000** | 0,996092 | 90,064 | 95,392 |
| RABL2A | RAB, member of RAS oncogene family like 2A | **1,000** | 0,997423 | 2,603 | 3,767 |
| PTGR1 | prostaglandin reductase 1 | **1,000** | 0,992727 | 23,929 | 21,964 |
| COIL | coilin | **1,000** | 0,993885 | 11,909 | 14,558 |
| ARRDC1 | arrestin domain containing 1 | **1,000** | 0,995822 | 9,139 | 6,321 |
| C1orf216 | chromosome 1 open reading frame 216 | **1,000** | 0,992378 | 26,200 | 18,006 |
| CCDC97 | coiled-coil domain containing 97 | **1,000** | 0,990768 | 35,394 | 36,139 |
| MSH2 | mutS homolog 2 | **1,000** | 0,992034 | 16,728 | 18,389 |
| TMEM222 | transmembrane protein 222 | **1,000** | 0,987895 | 36,945 | 46,802 |
| GNB1 | G protein subunit beta 1 | **1,000** | 0,985365 | 324,642 | 265,360 |
| USP54 | ubiquitin specific peptidase 54 | **1,000** | 0,991389 | 13,072 | 15,324 |
| CDC14B | cell division cycle 14B | **1,000** | 0,995076 | 3,600 | 3,512 |
| TSNAXIP1 | translin associated factor X interacting protein 1 | **1,000** | 0,995793 | 1,606 | 2,235 |
| BORA | bora, aurora kinase A activator | **1,000** | 0,996269 | 2,216 | 1,405 |
| CYS1 | cystin 1 | **1,000** | 0,995799 | 3,379 | 1,341 |
| TTC5 | tetratricopeptide repeat domain 5 | **1,000** | 0,990044 | 12,075 | 15,324 |
| AKAP8L | A-kinase anchoring protein 8 like | **0,999** | 0,98561 | 34,951 | 34,862 |
| HERC2P3 | hect domain and RLD 2 pseudogene 3 | **0,999** | 0,996 | 2,548 | 4,150 |
| GRB2 | growth factor receptor bound protein 2 | **0,999** | 0,983416 | 95,271 | 92,710 |
| RPS23 | ribosomal protein S23 | **0,999** | 0,988515 | 137,423 | 132,169 |
| PSME4 | proteasome activator subunit 4 | **0,999** | 0,986009 | 56,720 | 61,807 |
| LGALS3 | galectin 3 | **0,999** | 0,985943 | 348,626 | 270,085 |
| NECAP1 | NECAP endocytosis associated 1 | **0,999** | 0,984364 | 32,514 | 43,163 |

| | | | | | |
|---|---|---|---|---|---|
| RBM18 | RNA binding motif protein 18 | **0,999** | 0,985816 | 32,292 | 33,457 |
| CFAP20 | cilia and flagella associated protein 20 | **0,999** | 0,983218 | 31,739 | 31,606 |
| ZZZ3 | zinc finger ZZ-type containing 3 | **0,999** | 0,983376 | 30,908 | 34,926 |
| TMIE | transmembrane inner ear | **0,999** | 0,994713 | 1,551 | 2,809 |
| ACER2 | alkaline ceramidase 2 | **0,999** | 0,995318 | 0,775 | 1,341 |
| CRYBB2P1 | crystallin beta B2 pseudogene 1 | **0,999** | 0,985888 | 9,416 | 9,514 |
| DGCR2 | DiGeorge syndrome critical region gene 2 | **0,999** | 0,977335 | 82,365 | 86,006 |
| SPRED1 | sprouty related EVH1 domain containing 1 | **0,999** | 0,977062 | 51,568 | 49,867 |
| TMEM8A | transmembrane protein 8A | **0,999** | 0,979488 | 47,469 | 39,715 |
| ERCC6L | ERCC excision repair 6 like, spindle assembly checkpoint helicase | **0,999** | 0,996129 | 1,772 | 0,383 |
| LINC01759 | long intergenic non-protein coding RNA 1759 | **0,999** | 0,995935 | 0,499 | 0,958 |
| ARL4C | ADP ribosylation factor like GTPase 4C | **0,999** | 0,982556 | 18,999 | 14,941 |
| TIGD2 | tigger transposable element derived 2 | **0,999** | 0,98603 | 7,422 | 7,279 |
| ILKAP | ILK associated serine/threonine phosphatase | **0,999** | 0,97904 | 16,008 | 17,112 |
| NUP160 | nucleoporin 160 | **0,999** | 0,983812 | 10,746 | 16,473 |
| CDKN1B | cyclin dependent kinase inhibitor 1B | **0,999** | 0,977144 | 35,782 | 31,861 |
| NR4A3 | nuclear receptor subfamily 4 group A member 3 | **0,999** | 0,980865 | 18,500 | 19,027 |
| AP4E1 | adaptor related protein complex 4 epsilon 1 subunit | **0,999** | 0,983801 | 16,949 | 18,580 |
| DDHD2 | DDHD domain containing 2 | **0,999** | 0,978951 | 13,183 | 13,919 |
| TOM1 | target of myb1 membrane trafficking protein | **0,999** | 0,974926 | 112,664 | 111,482 |
| NUAK2 | NUAK family kinase 2 | **0,999** | 0,994458 | 0,665 | 0,638 |
| PTPRE | protein tyrosine phosphatase, receptor type E | **0,999** | 0,979043 | 62,757 | 70,043 |
| GPR89B | G protein-coupled receptor 89B | **0,999** | 0,993124 | 1,440 | 1,213 |
| SLC2A3 | solute carrier family 2 member 3 | **0,999** | 0,974919 | 28,692 | 39,906 |
| AACS | acetoacetyl-CoA synthetase | **0,999** | 0,978986 | 19,996 | 17,176 |
| CTSA | cathepsin A | **0,999** | 0,966359 | 109,174 | 77,195 |
| ICMT | isoprenylcysteine carboxyl methyltransferase | **0,999** | 0,962021 | 69,459 | 59,061 |
| UBE2L3 | ubiquitin conjugating enzyme E2 L3 | **0,999** | 0,964074 | 46,860 | 50,250 |
| SPATA6L | spermatogenesis associated 6 like | **0,999** | 0,990236 | 1,606 | 2,235 |
| SAP18 | Sin3A associated protein 18 | **0,999** | 0,968847 | 89,898 | 100,244 |
| CUX1 | cut like homeobox 1 | **0,999** | 0,969204 | 73,060 | 64,680 |
| COMMD6 | COMM domain containing 6 | **0,999** | 0,976192 | 21,879 | 19,538 |
| ASF1A | anti-silencing function 1A histone chaperone | **0,999** | 0,974314 | 13,460 | 13,664 |

| | | | | | |
|---|---|---|---|---|---|
| CDK9 | cyclin dependent kinase 9 | **0,999** | 0,96375 | 41,653 | 37,991 |
| NAPG | NSF attachment protein gamma | **0,999** | 0,965817 | 51,845 | 55,549 |
| DUBR | DPPA2 upstream binding RNA | **0,999** | 0,988683 | 2,160 | 2,554 |
| POU2F2 | POU class 2 homeobox 2 | **0,999** | 0,970658 | 85,356 | 91,688 |
| DNM2 | dynamin 2 | **0,999** | 0,959905 | 67,299 | 61,168 |
| NF1 | neurofibromin 1 | **0,999** | 0,980266 | 51,125 | 49,484 |
| RPIA | ribose 5-phosphate isomerase A | **0,999** | 0,977314 | 7,810 | 10,663 |
| C4orf3 | chromosome 4 open reading frame 3 | **0,999** | 0,962978 | 108,288 | 95,839 |
| ZNF830 | zinc finger protein 830 | **0,999** | 0,969924 | 13,737 | 15,132 |
| PLSCR1 | phospholipid scramblase 1 | **0,999** | 0,964234 | 21,270 | 19,474 |
| TREM1 | triggering receptor expressed on myeloid cells 1 | **0,999** | 0,968797 | 38,552 | 37,033 |
| WDR53 | WD repeat domain 53 | **0,998** | 0,973516 | 6,924 | 6,768 |
| DDR2 | discoidin domain receptor tyrosine kinase 2 | **0,998** | 0,969945 | 206,495 | 192,380 |
| LTBP2 | latent transforming growth factor beta binding protein 2 | **0,998** | 0,960251 | 462,397 | 572,861 |
| UTP15 | UTP15, small subunit processome component | **0,998** | 0,974767 | 12,851 | 13,919 |
| THBS3 | thrombospondin 3 | **0,998** | 0,975098 | 15,066 | 8,428 |
| HFE | hemochromatosis | **0,998** | 0,975823 | 6,813 | 6,257 |
| NDUFV1 | NADH:ubiquinone oxidoreductase core subunit V1 | **0,998** | 0,961279 | 39,936 | 46,291 |
| RPAIN | RPA interacting protein | **0,998** | 0,967911 | 15,842 | 18,963 |
| HSP90B1 | heat shock protein 90 beta family member 1 | **0,998** | 0,950675 | 1620,938 | 1142,594 |
| EMC2 | ER membrane protein complex subunit 2 | **0,998** | 0,952779 | 35,616 | 36,011 |
| LARP6 | La ribonucleoprotein domain family member 6 | **0,998** | 0,950671 | 78,543 | 66,659 |
| MCM8 | minichromosome maintenance 8 homologous recombination repair factor | **0,998** | 0,977428 | 6,259 | 5,555 |
| CLCN3 | chloride voltage-gated channel 3 | **0,998** | 0,954067 | 69,958 | 71,576 |
| NIT2 | nitrilase family member 2 | **0,998** | 0,960975 | 20,661 | 20,560 |
| WBP11 | WW domain binding protein 11 | **0,998** | 0,948012 | 52,676 | 65,318 |
| RAB28 | RAB28, member RAS oncogene family | **0,998** | 0,958195 | 19,885 | 18,900 |
| RNF181 | ring finger protein 181 | **0,998** | 0,955331 | 41,155 | 40,481 |
| TRIM65 | tripartite motif containing 65 | **0,998** | 0,969399 | 9,306 | 4,150 |
| STK38L | serine/threonine kinase 38 like | **0,998** | 0,958529 | 52,676 | 42,524 |
| NEMF | nuclear export mediator factor | **0,998** | 0,962634 | 36,558 | 35,437 |
| TMEM106C | transmembrane protein 106C | **0,998** | 0,956687 | 35,117 | 32,691 |
| MTIF2 | mitochondrial translational initiation factor 2 | **0,998** | 0,95427 | 19,553 | 17,048 |

| | | | | | |
|---|---|---|---|---|---|
| NOC4L | nucleolar complex associated 4 homolog | **0,998** | 0,964039 | 7,810 | 6,960 |
| MYO5A | myosin VA | **0,998** | 0,961323 | 55,224 | 34,479 |
| UBE2E1 | ubiquitin conjugating enzyme E2 E1 | **0,998** | 0,94898 | 71,232 | 65,382 |
| ITGA11 | integrin subunit alpha 11 | **0,998** | 0,956258 | 56,166 | 28,860 |
| CNEP1R1 | CTD nuclear envelope phosphatase 1 regulatory subunit 1 | **0,998** | 0,957686 | 17,060 | 16,856 |
| AP2B1 | adaptor related protein complex 2 beta 1 subunit | **0,998** | 0,942521 | 105,020 | 88,624 |
| MFAP1 | microfibrillar associated protein 1 | **0,998** | 0,944077 | 36,834 | 34,543 |
| MRPS31 | mitochondrial ribosomal protein S31 | **0,998** | 0,95802 | 9,693 | 10,727 |
| GPBP1 | GC-rich promoter binding protein 1 | **0,998** | 0,93272 | 51,236 | 48,909 |
| POGZ | pogo transposable element derived with ZNF domain | **0,998** | 0,956407 | 39,105 | 39,842 |
| PLXNA1 | plexin A1 | **0,998** | 0,94221 | 103,358 | 92,582 |
| SDC3 | syndecan 3 | **0,998** | 0,936532 | 99,315 | 84,346 |
| ATXN2 | ataxin 2 | **0,998** | 0,943176 | 32,126 | 29,562 |
| TBC1D13 | TBC1 domain family member 13 | **0,998** | 0,943952 | 30,797 | 29,307 |
| WDTC1 | WD and tetratricopeptide repeats 1 | **0,998** | 0,946651 | 52,399 | 44,440 |
| SNTA1 | syntrophin alpha 1 | **0,998** | 0,970626 | 3,600 | 2,618 |
| IL13RA1 | interleukin 13 receptor subunit alpha 1 | **0,998** | 0,934951 | 160,410 | 150,813 |
| ATP6V1D | ATPase H+ transporting V1 subunit D | **0,998** | 0,940465 | 40,047 | 32,627 |
| CNOT8 | CCR4-NOT transcription complex subunit 8 | **0,998** | 0,929136 | 33,954 | 36,011 |
| NUP214 | nucleoporin 214 | **0,998** | 0,948376 | 37,665 | 40,609 |
| ZNF271P | zinc finger protein 271, pseudogene | **0,998** | 0,936847 | 30,797 | 27,264 |
| PTMAP5 | prothymosin, alpha pseudogene 5 | **0,998** | 0,979604 | 2,659 | 1,213 |
| EEF2 | eukaryotic translation elongation factor 2 | **0,997** | 0,930318 | 1771,267 | 1738,824 |
| OSGEPL1 | O-sialoglycoprotein endopeptidase like 1 | **0,997** | 0,968907 | 2,049 | 3,831 |
| WIPI2 | WD repeat domain, phosphoinositide interacting 2 | **0,997** | 0,925247 | 72,672 | 65,127 |
| LINC02005 | long intergenic non-protein coding RNA 2005 | **0,997** | 0,981791 | 1,329 | 1,979 |
| ENTPD7 | ectonucleoside triphosphate diphosphohydrolase 7 | **0,997** | 0,946006 | 79,153 | 81,217 |
| KLHL5 | kelch like family member 5 | **0,997** | 0,943529 | 42,318 | 27,966 |
| TMEM79 | transmembrane protein 79 | **0,997** | 0,96244 | 3,877 | 4,597 |
| RBM39 | RNA binding motif protein 39 | **0,997** | 0,922457 | 125,957 | 128,274 |
| NCOA3 | nuclear receptor coactivator 3 | **0,997** | 0,947091 | 55,556 | 51,782 |
| EIF2S1 | eukaryotic translation initiation factor 2 subunit alpha | **0,997** | 0,923222 | 78,765 | 85,112 |
| MLLT10 | myeloid/lymphoid or mixed-lineage leukemia; translocated to, 10 | **0,997** | 0,953389 | 11,743 | 13,089 |

| | | | | | |
|---|---|---|---|---|---|
| BCAS2 | BCAS2, pre-mRNA processing factor | **0,997** | 0,924799 | 34,785 | 37,735 |
| FAM185BP | family with sequence similarity 185 member B, pseudogene | **0,997** | 0,988504 | 0,222 | 0,192 |
| DHX38 | DEAH-box helicase 38 | **0,997** | 0,931369 | 53,784 | 65,446 |
| ING3 | inhibitor of growth family member 3 | **0,997** | 0,944042 | 13,294 | 16,601 |
| DRG2 | developmentally regulated GTP binding protein 2 | **0,997** | 0,92828 | 18,002 | 21,517 |
| AGK | acylglycerol kinase | **0,997** | 0,966545 | 3,988 | 4,661 |
| PTPN23 | protein tyrosine phosphatase, non-receptor type 23 | **0,997** | 0,930602 | 53,839 | 49,739 |
| ZFAT | zinc finger and AT-hook domain containing | **0,997** | 0,958447 | 9,804 | 8,939 |
| RAPGEF6 | Rap guanine nucleotide exchange factor 6 | **0,997** | 0,958278 | 6,204 | 5,810 |
| PRKCZ | protein kinase C zeta | **0,997** | 0,947773 | 9,804 | 7,151 |
| TSPAN17 | tetraspanin 17 | **0,997** | 0,940548 | 18,057 | 14,558 |
| ZNF439 | zinc finger protein 439 | **0,997** | 0,959758 | 4,265 | 3,639 |
| RPS27A | ribosomal protein S27a | **0,997** | 0,932233 | 260,832 | 291,283 |
| ZNF341-AS1 | ZNF341 antisense RNA 1 | **0,997** | 0,978295 | 1,883 | 1,915 |
| RAB37 | RAB37, member RAS oncogene family | **0,997** | 0,962591 | 1,385 | 2,426 |
| VPS37C | VPS37C, ESCRT-I subunit | **0,997** | 0,917851 | 49,519 | 42,971 |
| TYW3 | tRNA-yW synthesizing protein 3 homolog | **0,997** | 0,921221 | 20,384 | 23,497 |
| UBQLN1 | ubiquilin 1 | **0,997** | 0,918741 | 117,261 | 118,505 |
| COL15A1 | collagen type XV alpha 1 chain | **0,997** | 0,932917 | 94,385 | 256,357 |
| NOXA1 | NADPH oxidase activator 1 | **0,997** | 0,973184 | 1,163 | 3,192 |
| ERI1 | exoribonuclease 1 | **0,997** | 0,971869 | 1,883 | 2,235 |
| BLOC1S1 | biogenesis of lysosomal organelles complex 1 subunit 1 | **0,997** | 0,943528 | 9,139 | 7,917 |
| ZNF396 | zinc finger protein 396 | **0,997** | 0,95402 | 5,650 | 6,193 |
| PGPEP1 | pyroglutamyl-peptidase I | **0,997** | 0,95263 | 9,084 | 5,746 |
| SASS6 | SAS-6 centriolar assembly protein | **0,997** | 0,961254 | 3,656 | 3,448 |
| VDAC2 | voltage dependent anion channel 2 | **0,997** | 0,922031 | 33,068 | 32,691 |
| VAPB | VAMP associated protein B and C | **0,997** | 0,905849 | 66,690 | 60,274 |
| ANKRD35 | ankyrin repeat domain 35 | **0,997** | 0,962211 | 3,157 | 3,320 |
| RAB1A | RAB1A, member RAS oncogene family | **0,997** | 0,906053 | 220,564 | 184,079 |
| ZBTB16 | zinc finger and BTB domain containing 16 | **0,997** | 0,976814 | 1,385 | 1,724 |
| DMKN | dermokine | **0,997** | 0,92399 | 22,987 | 18,963 |
| JAG1 | jagged 1 | **0,997** | 0,914611 | 115,544 | 139,576 |
| JSRP1 | junctional sarcoplasmic reticulum protein 1 | **0,997** | 0,983339 | 0,443 | 0,447 |

| | | | | | |
|---|---|---|---|---|---|
| C19orf25 | chromosome 19 open reading frame 25 | **0,997** | 0,913949 | 20,384 | 21,773 |
| MPHOSPH8 | M-phase phosphoprotein 8 | **0,997** | 0,907332 | 50,959 | 58,167 |
| POM121B | POM121 transmembrane nucleoporin B (pseudogene) | **0,997** | 0,985516 | 0,111 | 0,064 |
| PRR14L | proline rich 14 like | **0,997** | 0,941816 | 24,759 | 27,264 |
| SNORD104 | small nucleolar RNA, C/D box 104 | **0,997** | 0,975406 | 1,108 | 1,405 |
| RNASEH1-AS1 | RNASEH1 antisense RNA 1 | **0,996** | 0,957089 | 5,096 | 4,661 |
| MTG1 | mitochondrial ribosome associated GTPase 1 | **0,996** | 0,955098 | 3,046 | 4,342 |
| GSK3B | glycogen synthase kinase 3 beta | **0,996** | 0,890662 | 93,277 | 79,621 |
| MCM5 | minichromosome maintenance complex component 5 | **0,996** | 0,916268 | 19,331 | 12,451 |
| POLM | DNA polymerase mu | **0,996** | 0,920844 | 11,964 | 15,260 |
| ZBED8 | zinc finger BED-type containing 8 | **0,996** | 0,954052 | 4,431 | 4,342 |
| DIDO1 | death inducer-obliterator 1 | **0,996** | 0,912696 | 52,731 | 47,121 |
| ARFGAP2 | ADP ribosylation factor GTPase activating protein 2 | **0,996** | 0,914808 | 41,100 | 48,973 |
| GALE | UDP-galactose-4-epimerase | **0,996** | 0,900273 | 49,020 | 57,082 |
| RARG | retinoic acid receptor gamma | **0,996** | 0,894851 | 97,597 | 116,909 |
| TMEM19 | transmembrane protein 19 | **0,996** | 0,932254 | 13,681 | 7,790 |
| NDUFV2 | NADH:ubiquinone oxidoreductase core subunit V2 | **0,996** | 0,98301 | 0,332 | 0,319 |
| DENND4B | DENN domain containing 4B | **0,996** | 0,899766 | 31,185 | 29,946 |
| USPL1 | ubiquitin specific peptidase like 1 | **0,996** | 0,928608 | 17,393 | 14,877 |
| GID4 | GID complex subunit 4 homolog | **0,996** | 0,916162 | 20,328 | 17,750 |
| LTBP1 | latent transforming growth factor beta binding protein 1 | **0,996** | 0,922147 | 138,198 | 53,442 |
| PSMD14 | proteasome 26S subunit, non-ATPase 14 | **0,996** | 0,892209 | 67,354 | 67,809 |
| OSBP | oxysterol binding protein | **0,996** | 0,896624 | 88,846 | 84,920 |
| BNIP3L | BCL2 interacting protein 3 like | **0,996** | 0,886092 | 260,389 | 237,202 |
| COPS3 | COP9 signalosome subunit 3 | **0,996** | 0,889512 | 27,474 | 32,691 |
| MSN | moesin | **0,996** | 0,904828 | 523,105 | 465,338 |
| STX18-AS1 | STX18 antisense RNA 1 (head to head) | **0,996** | 0,980911 | 0,609 | 0,638 |
| PIGQ | phosphatidylinositol glycan anchor biosynthesis class Q | **0,996** | 0,917433 | 19,664 | 19,666 |
| FOSL2 | FOS like 2, AP-1 transcription factor subunit | **0,996** | 0,898956 | 212,532 | 202,021 |
| KHNYN | KH and NYN domain containing | **0,996** | 0,895879 | 69,293 | 66,468 |
| COMP | cartilage oligomeric matrix protein | **0,996** | 0,912118 | 3044,963 | 3728,194 |
| MED9 | mediator complex subunit 9 | **0,996** | 0,915123 | 12,130 | 9,705 |
| PRR13 | proline rich 13 | **0,996** | 0,910293 | 29,966 | 25,732 |

| | | | | | |
|---|---|---|---|---|---|
| SYNJ2BP | synaptojanin 2 binding protein | **0,996** | 0,908567 | 26,920 | 28,669 |
| CEP95 | centrosomal protein 95 | **0,996** | 0,915836 | 12,352 | 15,707 |
| KHSRP | KH-type splicing regulatory protein | **0,996** | 0,877336 | 89,898 | 92,774 |
| NUP107 | nucleoporin 107 | **0,996** | 0,910603 | 23,208 | 24,135 |
| DNPEP | aspartyl aminopeptidase | **0,996** | 0,905128 | 28,027 | 20,879 |
| CCDC122 | coiled-coil domain containing 122 | **0,996** | 0,947518 | 4,764 | 5,172 |
| DCTN2 | dynactin subunit 2 | **0,996** | 0,874531 | 70,733 | 57,146 |
| C22orf39 | chromosome 22 open reading frame 39 | **0,996** | 0,91579 | 9,582 | 9,577 |
| DPH6-AS1 | DPH6 antisense RNA 1 (head to head) | **0,996** | 0,98343 | 0,111 | 0,000 |
| REEP3 | receptor accessory protein 3 | **0,996** | 0,882745 | 76,383 | 69,852 |
| PDCD10 | programmed cell death 10 | **0,996** | 0,905241 | 28,858 | 28,094 |
| CDC27 | cell division cycle 27 | **0,996** | 0,897783 | 47,746 | 43,290 |
| SART3 | squamous cell carcinoma antigen recognized by T-cells 3 | **0,996** | 0,877546 | 46,639 | 54,145 |
| WDR20 | WD repeat domain 20 | **0,996** | 0,907663 | 21,547 | 20,240 |
| TFAM | transcription factor A, mitochondrial | **0,996** | 0,907129 | 23,707 | 27,775 |
| SELENOO | selenoprotein O | **0,996** | 0,913786 | 10,303 | 10,024 |
| TTC26 | tetratricopeptide repeat domain 26 | **0,996** | 0,912757 | 13,238 | 15,771 |
| ELOF1 | elongation factor 1 homolog | **0,996** | 0,905608 | 23,873 | 24,901 |
| RITA1 | RBPJ interacting and tubulin associated 1 | **0,996** | 0,907001 | 10,413 | 10,408 |
| SLC25A45 | solute carrier family 25 member 45 | **0,996** | 0,924194 | 8,918 | 14,941 |
| RNASEH2C | ribonuclease H2 subunit C | **0,996** | 0,911735 | 32,459 | 27,839 |
| PCED1B | PC-esterase domain containing 1B | **0,996** | 0,958101 | 3,434 | 2,554 |
| CNTROB | centrobin, centriole duplication and spindle assembly protein | **0,996** | 0,909379 | 19,664 | 18,261 |
| SBF2 | SET binding factor 2 | **0,996** | 0,909192 | 38,053 | 39,778 |
| TSN | translin | **0,996** | 0,867696 | 86,464 | 115,760 |
| TECR | trans-2,3-enoyl-CoA reductase | **0,996** | 0,886128 | 43,703 | 43,673 |
| GS1-124K5.4 | uncharacterized LOC100289098 | **0,996** | 0,976899 | 0,499 | 0,894 |
| COG8 | component of oligomeric golgi complex 8 | **0,996** | 0,942968 | 5,761 | 6,513 |
| EFCAB2 | EF-hand calcium binding domain 2 | **0,996** | 0,915326 | 9,361 | 12,642 |
| RPS15 | ribosomal protein S15 | **0,996** | 0,895886 | 148,723 | 169,266 |
| ZMIZ1 | zinc finger MIZ-type containing 1 | **0,996** | 0,88968 | 218,791 | 205,724 |
| MAF1 | MAF1 homolog, negative regulator of RNA polymerase III | **0,995** | 0,860617 | 69,736 | 58,678 |
| FBXO16 | F-box protein 16 | **0,995** | 0,969317 | 0,720 | 0,638 |

| | | | | | |
|---|---|---|---|---|---|
| CD63 | CD63 molecule | **0,995** | 0,876681 | 1063,657 | 763,708 |
| SLC39A8 | solute carrier family 39 member 8 | **0,995** | 0,897731 | 161,185 | 100,819 |
| GTF2IRD1P1 | GTF2I repeat domain containing 1 pseudogene 1 | **0,995** | 0,981887 | 0,499 | 0,383 |
| CEP89 | centrosomal protein 89 | **0,995** | 0,891567 | 18,833 | 15,835 |
| CST4 | cystatin S | **0,995** | 0,956916 | 0,886 | 1,022 |
| PRKRIP1 | PRKR interacting protein 1 (IL11 inducible) | **0,995** | 0,900517 | 15,786 | 19,985 |
| OXSR1 | oxidative stress responsive 1 | **0,995** | 0,881345 | 52,178 | 42,077 |
| NPAS3 | neuronal PAS domain protein 3 | **0,995** | 0,964754 | 1,219 | 1,532 |
| SLC25A16 | solute carrier family 25 member 16 | **0,995** | 0,938203 | 6,868 | 6,193 |
| MED21 | mediator complex subunit 21 | **0,995** | 0,886214 | 27,307 | 28,094 |
| SETD3 | SET domain containing 3 | **0,995** | 0,860683 | 41,487 | 42,332 |
| VPS37D | VPS37D, ESCRT-I subunit | **0,995** | 0,970523 | 0,942 | 0,447 |
| CLPX | caseinolytic mitochondrial matrix peptidase chaperone subunit | **0,995** | 0,869267 | 30,520 | 27,072 |
| DANCR | differentiation antagonizing non-protein coding RNA | **0,995** | 0,918848 | 10,856 | 11,174 |
| RAI14 | retinoic acid induced 14 | **0,995** | 0,8924 | 48,411 | 41,183 |
| ITGB1BP1 | integrin subunit beta 1 binding protein 1 | **0,995** | 0,865065 | 46,140 | 39,842 |
| ZNF213 | zinc finger protein 213 | **0,995** | 0,871531 | 21,325 | 21,454 |
| KIAA1524 | KIAA1524 | **0,995** | 0,948685 | 5,650 | 2,746 |
| HNRNPU | heterogeneous nuclear ribonucleoprotein U | **0,995** | 0,849567 | 321,485 | 329,146 |
| NUP153 | nucleoporin 153 | **0,995** | 0,907313 | 58,381 | 58,103 |
| HELQ | helicase, POLQ-like | **0,995** | 0,886037 | 15,066 | 16,537 |
| GALT | galactose-1-phosphate uridylyltransferase | **0,995** | 0,953953 | 3,157 | 2,107 |
| ZRANB1 | zinc finger RANBP2-type containing 1 | **0,995** | 0,861269 | 40,656 | 38,693 |
| EXOSC3 | exosome component 3 | **0,995** | 0,893953 | 11,853 | 13,664 |
| UBA7 | ubiquitin like modifier activating enzyme 7 | **0,995** | 0,929917 | 6,093 | 4,278 |
| MLYCD | malonyl-CoA decarboxylase | **0,995** | 0,903539 | 9,582 | 9,194 |
| NAMPTP1 | nicotinamide phosphoribosyltransferase pseudogene 1 | **0,995** | 0,905523 | 22,433 | 27,775 |
| ANKRD11 | ankyrin repeat domain 11 | **0,995** | 0,865599 | 143,571 | 145,769 |
| GTF3C1 | general transcription factor IIIC subunit 1 | **0,995** | 0,855356 | 60,486 | 58,806 |
| CRELD2 | cysteine rich with EGF like domains 2 | **0,995** | 0,90593 | 45,198 | 34,032 |
| IPO8 | importin 8 | **0,995** | 0,89603 | 37,167 | 32,947 |
| ANKRD24 | ankyrin repeat domain 24 | **0,995** | 0,92839 | 2,770 | 4,278 |
| OXCT2P1 | 3-oxoacid CoA-transferase 2 pseudogene 1 | **0,995** | 0,978314 | 0,277 | 0,766 |

| | | | | | |
|---|---|---|---|---|---|
| GSDMB | gasdermin B | **0,995** | 0,9387 | 3,656 | 3,639 |
| CIRBP | cold inducible RNA binding protein | **0,995** | 0,864764 | 198,075 | 241,927 |
| TCHP | trichoplein keratin filament binding | **0,995** | 0,898944 | 13,571 | 14,941 |
| CAV2 | caveolin 2 | **0,995** | 0,861446 | 105,075 | 76,811 |
| NEK9 | NIMA related kinase 9 | **0,995** | 0,865988 | 51,014 | 54,719 |
| APOD | apolipoprotein D | **0,995** | 0,884223 | 111,002 | 25,093 |
| CSNK1G1 | casein kinase 1 gamma 1 | **0,995** | 0,909283 | 15,232 | 14,175 |
| PTCD3 | pentatricopeptide repeat domain 3 | **0,995** | 0,856739 | 29,301 | 35,564 |
| TRMT10B | tRNA methyltransferase 10B | **0,995** | 0,936559 | 3,711 | 3,384 |
| TIMM8A | translocase of inner mitochondrial membrane 8A | **0,995** | 0,928674 | 4,487 | 7,151 |
| KDM4D | lysine demethylase 4D | **0,995** | 0,941453 | 1,828 | 3,384 |
| WDR91 | WD repeat domain 91 | **0,995** | 0,900527 | 8,918 | 11,110 |
| ZNF285 | zinc finger protein 285 | **0,995** | 0,957294 | 1,883 | 1,405 |
| NSD1 | nuclear receptor binding SET domain protein 1 | **0,995** | 0,889387 | 47,802 | 50,186 |
| CABIN1 | calcineurin binding protein 1 | **0,995** | 0,881372 | 28,747 | 29,690 |
| MMP16 | matrix metallopeptidase 16 | **0,995** | 0,938019 | 7,035 | 3,767 |
| JUNB | JunB proto-oncogene, AP-1 transcription factor subunit | **0,995** | 0,87132 | 175,310 | 182,419 |
| KIFAP3 | kinesin associated protein 3 | **0,995** | 0,872879 | 27,307 | 22,156 |
| YBEY | ybeY metallopeptidase (putative) | **0,995** | 0,900855 | 6,314 | 9,577 |
| PBLD | phenazine biosynthesis like protein domain containing | **0,995** | 0,94176 | 2,437 | 2,107 |
| MTOR | mechanistic target of rapamycin | **0,995** | 0,887014 | 46,472 | 44,759 |
| OTULIN | OTU deubiquitinase with linear linkage specificity | **0,995** | 0,905394 | 18,168 | 16,154 |
| NXT2 | nuclear transport factor 2 like export factor 2 | **0,995** | 0,882165 | 22,987 | 24,901 |
| UFSP2 | UFM1 specific peptidase 2 | **0,995** | 0,857581 | 25,424 | 24,518 |
| UEVLD | UEV and lactate/malate dehyrogenase domains | **0,994** | 0,887647 | 18,279 | 15,835 |
| SYNPO2 | synaptopodin 2 | **0,994** | 0,925219 | 5,207 | 5,363 |
| XPC | XPC complex subunit, DNA damage recognition and repair factor | **0,994** | 0,849805 | 39,936 | 44,056 |
| MRPS18C | mitochondrial ribosomal protein S18C | **0,994** | 0,890265 | 15,011 | 17,750 |
| TPP2 | tripeptidyl peptidase 2 | **0,994** | 0,880689 | 27,640 | 27,328 |
| PDHA1 | pyruvate dehydrogenase (lipoamide) alpha 1 | **0,994** | 0,839806 | 42,373 | 43,163 |
| ELP4 | elongator acetyltransferase complex subunit 4 | **0,994** | 0,90552 | 7,699 | 8,492 |
| TNIP2 | TNFAIP3 interacting protein 2 | **0,994** | 0,846082 | 28,360 | 31,925 |
| CCDC189 | coiled-coil domain containing 189 | **0,994** | 0,942457 | 2,493 | 2,490 |

| | | | | | |
|---|---|---|---|---|---|
| ATP5O | ATP synthase, H+ transporting, mitochondrial F1 complex, O subunit | **0,994** | 0,889414 | 14,069 | 15,452 |
| CUL9 | cullin 9 | **0,994** | 0,871823 | 20,550 | 21,709 |
| LPIN1 | lipin 1 | **0,994** | 0,824198 | 41,210 | 44,120 |
| ZNF514 | zinc finger protein 514 | **0,994** | 0,916575 | 8,198 | 5,555 |
| BOP1 | block of proliferation 1 | **0,994** | 0,896281 | 21,325 | 28,541 |
| CCNT2-AS1 | CCNT2 antisense RNA 1 | **0,994** | 0,96285 | 0,554 | 0,894 |
| DGCR8 | DGCR8, microprocessor complex subunit | **0,994** | 0,846161 | 33,622 | 25,540 |
| ABCD3 | ATP binding cassette subfamily D member 3 | **0,994** | 0,841125 | 32,182 | 33,521 |
| TNRC18 | trinucleotide repeat containing 18 | **0,994** | 0,878571 | 72,173 | 71,959 |
| BET1 | Bet1 golgi vesicular membrane trafficking protein | **0,994** | 0,879621 | 22,378 | 25,540 |
| RMND1 | required for meiotic nuclear division 1 homolog | **0,994** | 0,895976 | 14,014 | 15,069 |
| EHBP1L1 | EH domain binding protein 1 like 1 | **0,994** | 0,856013 | 94,939 | 82,749 |
| RARS2 | arginyl-tRNA synthetase 2, mitochondrial | **0,994** | 0,846025 | 19,996 | 18,516 |
| UBXN1 | UBX domain protein 1 | **0,994** | 0,842293 | 45,420 | 45,780 |
| EEF1AKMT2 | EEF1A lysine methyltransferase 2 | **0,994** | 0,932366 | 2,825 | 2,937 |
| SAYSD1 | SAYSVFN motif domain containing 1 | **0,994** | 0,872652 | 11,853 | 10,791 |
| CDCA8 | cell division cycle associated 8 | **0,994** | 0,94469 | 4,099 | 2,107 |
| LINC00662 | long intergenic non-protein coding RNA 662 | **0,994** | 0,904742 | 8,419 | 7,854 |
| LMTK2 | lemur tyrosine kinase 2 | **0,994** | 0,871507 | 30,021 | 35,309 |
| PLXNB2 | plexin B2 | **0,994** | 0,825876 | 350,454 | 355,133 |
| RPL23 | ribosomal protein L23 | **0,994** | 0,869933 | 264,765 | 253,612 |
| CEP72 | centrosomal protein 72 | **0,994** | 0,941679 | 1,662 | 2,746 |
| MFN1 | mitofusin 1 | **0,994** | 0,860639 | 27,086 | 22,220 |
| WAC-AS1 | WAC antisense RNA 1 (head to head) | **0,994** | 0,865579 | 17,891 | 12,195 |
| INO80 | INO80 complex subunit | **0,994** | 0,843016 | 41,653 | 46,163 |
| SMIM12 | small integral membrane protein 12 | **0,994** | 0,877849 | 21,713 | 23,561 |
| POLA1 | DNA polymerase alpha 1, catalytic subunit | **0,994** | 0,887569 | 9,029 | 10,918 |
| GLOD4 | glyoxalase domain containing 4 | **0,994** | 0,862682 | 29,855 | 26,115 |
| PIAS3 | protein inhibitor of activated STAT 3 | **0,994** | 0,824113 | 26,421 | 26,370 |
| RGS12 | regulator of G protein signaling 12 | **0,994** | 0,913894 | 8,364 | 4,725 |
| SPTY2D1 | SPT2 chromatin protein domain containing 1 | **0,994** | 0,831373 | 45,420 | 58,550 |
| IVNS1ABP | influenza virus NS1A binding protein | **0,994** | 0,859902 | 286,312 | 333,807 |
| USP47 | ubiquitin specific peptidase 47 | **0,994** | 0,841007 | 76,051 | 68,383 |

| | | | | | |
|---|---|---|---|---|---|
| SRP68 | signal recognition particle 68 | **0,994** | 0,796975 | 80,593 | 83,324 |
| DECR1 | 2,4-dienoyl-CoA reductase 1 | **0,994** | 0,84836 | 20,716 | 26,625 |
| HNRNPDL | heterogeneous nuclear ribonucleoprotein D like | **0,994** | 0,81361 | 113,550 | 148,387 |
| RABL2B | RAB, member of RAS oncogene family like 2B | **0,994** | 0,880407 | 11,189 | 11,301 |
| PYGO1 | pygopus family PHD finger 1 | **0,994** | 0,895405 | 6,370 | 5,810 |
| HAGLR | HOXD antisense growth-associated long non-coding RNA | **0,994** | 0,94593 | 1,883 | 1,788 |
| CSDC2 | cold shock domain containing C2 | **0,994** | 0,943275 | 1,717 | 0,958 |
| EIF4E3 | eukaryotic translation initiation factor 4E family member 3 | **0,994** | 0,879887 | 10,469 | 8,747 |
| FAF2 | Fas associated factor family member 2 | **0,994** | 0,829298 | 71,232 | 74,002 |
| POT1 | protection of telomeres 1 | **0,994** | 0,873059 | 14,401 | 17,112 |
| BCAT2 | branched chain amino acid transaminase 2 | **0,994** | 0,87889 | 8,641 | 10,216 |
| RSPH3 | radial spoke 3 homolog | **0,994** | 0,864183 | 17,337 | 14,047 |
| UBL7-AS1 | UBL7 antisense RNA 1 (head to head) | **0,994** | 0,955877 | 1,551 | 0,766 |
| SRP9 | signal recognition particle 9 | **0,994** | 0,837721 | 53,285 | 57,465 |
| UBE2J2 | ubiquitin conjugating enzyme E2 J2 | **0,994** | 0,840408 | 29,301 | 31,542 |
| CEP57 | centrosomal protein 57 | **0,993** | 0,838877 | 26,200 | 24,263 |
| ZNF418 | zinc finger protein 418 | **0,993** | 0,960511 | 0,831 | 1,405 |
| FUT4 | fucosyltransferase 4 | **0,993** | 0,810808 | 32,902 | 29,179 |
| RWDD2A | RWD domain containing 2A | **0,993** | 0,892005 | 10,967 | 8,237 |
| EED | embryonic ectoderm development | **0,993** | 0,860452 | 11,023 | 15,835 |
| ADAMTS9-AS2 | ADAMTS9 antisense RNA 2 | **0,993** | 0,930144 | 1,496 | 2,809 |
| SLC7A1 | solute carrier family 7 member 1 | **0,993** | 0,829771 | 158,804 | 172,395 |
| RPLP2 | ribosomal protein lateral stalk subunit P2 | **0,993** | 0,860383 | 199,349 | 216,898 |
| LLGL1 | LLGL1, scribble cell polarity complex component | **0,993** | 0,858446 | 20,494 | 19,410 |
| MORN1 | MORN repeat containing 1 | **0,993** | 0,89642 | 5,982 | 7,215 |
| OGG1 | 8-oxoguanine DNA glycosylase | **0,993** | 0,868779 | 11,964 | 12,834 |
| VKORC1 | vitamin K epoxide reductase complex subunit 1 | **0,993** | 0,866773 | 17,559 | 11,110 |
| NDOR1 | NADPH dependent diflavin oxidoreductase 1 | **0,993** | 0,866503 | 9,527 | 11,301 |
| CYB561D1 | cytochrome b561 family member D1 | **0,993** | 0,860563 | 10,192 | 10,280 |
| RPL37 | ribosomal protein L37 | **0,993** | 0,829294 | 193,810 | 212,684 |
| TRAK2 | trafficking kinesin protein 2 | **0,993** | 0,849572 | 24,759 | 22,284 |
| CDK5RAP3 | CDK5 regulatory subunit associated protein 3 | **0,993** | 0,894929 | 5,373 | 6,130 |
| SLC9A2 | solute carrier family 9 member A2 | **0,993** | 0,961786 | 0,332 | 0,638 |

| | | | | | |
|---|---|---|---|---|---|
| CASD1 | CAS1 domain containing 1 | **0,993** | 0,871006 | 17,005 | 19,474 |
| NCMAP | non-compact myelin associated protein | **0,993** | 0,96247 | 0,332 | 0,638 |
| ATRN | attractin | **0,993** | 0,820065 | 111,057 | 98,265 |
| TLR6 | toll like receptor 6 | **0,993** | 0,89648 | 9,195 | 10,152 |
| GOLIM4 | golgi integral membrane protein 4 | **0,993** | 0,818797 | 101,863 | 95,711 |
| NANP | N-acetylneuraminic acid phosphatase | **0,993** | 0,889811 | 6,813 | 6,832 |
| CHP1 | calcineurin like EF-hand protein 1 | **0,993** | 0,819596 | 62,037 | 50,761 |
| HMBOX1 | homeobox containing 1 | **0,993** | 0,896185 | 9,749 | 10,280 |
| SDHB | succinate dehydrogenase complex iron sulfur subunit B | **0,993** | 0,813706 | 32,459 | 33,649 |
| DARS-AS1 | DARS antisense RNA 1 | **0,993** | 0,912554 | 2,603 | 2,362 |
| ZNF440 | zinc finger protein 440 | **0,993** | 0,896553 | 4,819 | 4,533 |
| SMAD4 | SMAD family member 4 | **0,993** | 0,801834 | 49,574 | 42,460 |
| SLC30A6 | solute carrier family 30 member 6 | **0,993** | 0,800638 | 39,050 | 36,011 |
| XPO4 | exportin 4 | **0,993** | 0,848869 | 15,343 | 17,239 |
| BRPF1 | bromodomain and PHD finger containing 1 | **0,993** | 0,856135 | 16,174 | 17,303 |
| SEZ6L2 | seizure related 6 homolog like 2 | **0,993** | 0,926175 | 2,105 | 0,383 |
| NELFA | negative elongation factor complex member A | **0,993** | 0,811578 | 23,596 | 27,264 |
| FOXO4 | forkhead box O4 | **0,992** | 0,867849 | 11,521 | 9,961 |
| ARPC1A | actin related protein 2/3 complex subunit 1A | **0,992** | 0,857478 | 11,521 | 8,492 |
| ZNF587 | zinc finger protein 587 | **0,992** | 0,891832 | 8,309 | 7,726 |
| TMEM161B-AS1 | TMEM161B antisense RNA 1 | **0,992** | 0,90563 | 1,994 | 3,384 |
| ZNF669 | zinc finger protein 669 | **0,992** | 0,905803 | 4,210 | 3,639 |
| CRYL1 | crystallin lambda 1 | **0,992** | 0,865418 | 6,148 | 7,151 |
| RNGTT | RNA guanylyltransferase and 5'-phosphatase | **0,992** | 0,834683 | 20,937 | 22,028 |
| REEP5 | receptor accessory protein 5 | **0,992** | 0,816733 | 113,827 | 89,581 |
| ANXA7 | annexin A7 | **0,992** | 0,753796 | 213,197 | 201,063 |
| LPGAT1 | lysophosphatidylglycerol acyltransferase 1 | **0,992** | 0,787443 | 48,189 | 43,226 |
| EXD2 | exonuclease 3'-5' domain containing 2 | **0,992** | 0,834665 | 15,509 | 11,046 |
| DR1 | down-regulator of transcription 1 | **0,992** | 0,775194 | 47,912 | 49,611 |
| TCP1 | t-complex 1 | **0,992** | 0,7759 | 108,731 | 116,462 |
| TOMM20 | translocase of outer mitochondrial membrane 20 | **0,992** | 0,756568 | 103,635 | 105,735 |
| FAM120AOS | family with sequence similarity 120A opposite strand | **0,992** | 0,765532 | 53,175 | 48,334 |
| ST3GAL2 | ST3 beta-galactoside alpha-2,3-sialyltransferase 2 | **0,992** | 0,814246 | 22,765 | 18,772 |

| | | | | | |
|---|---|---|---|---|---|
| DNAJC15 | DnaJ heat shock protein family (Hsp40) member C15 | **0,992** | 0,809837 | 19,608 | 29,818 |
| ANKRD50 | ankyrin repeat domain 50 | **0,992** | 0,855737 | 38,330 | 28,094 |
| HYPK | huntingtin interacting protein K | **0,992** | 0,953285 | 0,720 | 0,830 |
| RIOX1 | ribosomal oxygenase 1 | **0,992** | 0,870363 | 8,309 | 10,024 |
| YBX1 | Y-box binding protein 1 | **0,992** | 0,770288 | 374,105 | 329,210 |
| ZNF662 | zinc finger protein 662 | **0,992** | 0,883971 | 5,761 | 5,874 |
| DPH3 | diphthamide biosynthesis 3 | **0,992** | 0,77683 | 36,281 | 39,778 |
| ASS1 | argininosuccinate synthase 1 | **0,992** | 0,768999 | 226,435 | 167,861 |
| CALM2 | calmodulin 2 | **0,992** | 0,765896 | 384,685 | 361,326 |
| RBM4 | RNA binding motif protein 4 | **0,992** | 0,923324 | 2,659 | 3,512 |
| NIN | ninein | **0,992** | 0,844062 | 67,687 | 62,956 |
| PIN4 | peptidylprolyl cis/trans isomerase, NIMA-interacting 4 | **0,992** | 0,859138 | 11,410 | 14,430 |
| NME6 | NME/NM23 nucleoside diphosphate kinase 6 | **0,992** | 0,843493 | 16,839 | 21,454 |
| DEXI | Dexi homolog | **0,992** | 0,804704 | 22,765 | 21,581 |
| COA7 | cytochrome c oxidase assembly factor 7 (putative) | **0,992** | 0,852487 | 12,297 | 16,282 |
| RBMX | RNA binding motif protein, X-linked | **0,992** | 0,744134 | 51,845 | 66,723 |
| ADORA2A-AS1 | ADORA2A antisense RNA 1 | **0,992** | 0,948271 | 0,997 | 0,766 |
| ACTR3B | ARP3 actin related protein 3 homolog B | **0,992** | 0,891076 | 3,933 | 4,597 |
| ZNF415 | zinc finger protein 415 | **0,992** | 0,929345 | 2,271 | 2,299 |
| TMEM9B-AS1 | TMEM9B antisense RNA 1 | **0,992** | 0,909989 | 2,603 | 2,490 |
| SNORD3B-2 | small nucleolar RNA, C/D box 3B-2 | **0,992** | 0,96809 | 0,277 | 0,830 |
| TK2 | thymidine kinase 2, mitochondrial | **0,992** | 0,778348 | 21,713 | 22,028 |
| NDUFA10 | NADH:ubiquinone oxidoreductase subunit A10 | **0,992** | 0,771494 | 41,487 | 38,948 |
| MUTYH | mutY DNA glycosylase | **0,992** | 0,833133 | 11,355 | 13,855 |
| GUCD1 | guanylyl cyclase domain containing 1 | **0,992** | 0,818304 | 43,647 | 32,308 |
| C2CD5 | C2 calcium dependent domain containing 5 | **0,992** | 0,7913 | 15,952 | 18,006 |
| LIAS | lipoic acid synthetase | **0,992** | 0,872808 | 5,539 | 7,470 |
| ZSCAN22 | zinc finger and SCAN domain containing 22 | **0,992** | 0,901214 | 5,096 | 4,597 |
| BLZF1 | basic leucine zipper nuclear factor 1 | **0,992** | 0,756858 | 48,910 | 49,611 |
| LCMT2 | leucine carboxyl methyltransferase 2 | **0,992** | 0,773755 | 22,378 | 25,540 |
| PSMA3 | proteasome subunit alpha 3 | **0,992** | 0,78963 | 60,597 | 55,613 |
| PRKAG2 | protein kinase AMP-activated non-catalytic subunit gamma 2 | **0,992** | 0,765835 | 40,712 | 43,418 |
| NR2C1 | nuclear receptor subfamily 2 group C member 1 | **0,992** | 0,835296 | 11,466 | 12,642 |

| | | | | | |
|---|---|---|---|---|---|
| OPA1 | OPA1, mitochondrial dynamin like GTPase | **0,992** | 0,781963 | 31,406 | 32,180 |
| RBM23 | RNA binding motif protein 23 | **0,992** | 0,774186 | 50,627 | 52,868 |
| C16orf87 | chromosome 16 open reading frame 87 | **0,992** | 0,871179 | 5,428 | 6,257 |
| TTI2 | TELO2 interacting protein 2 | **0,991** | 0,812204 | 13,127 | 14,558 |
| YME1L1 | YME1 like 1 ATPase | **0,991** | 0,734651 | 182,067 | 178,780 |
| IL1R1 | interleukin 1 receptor type 1 | **0,991** | 0,821407 | 46,306 | 56,571 |
| STRBP | spermatid perinuclear RNA binding protein | **0,991** | 0,891064 | 6,924 | 8,556 |
| CIAO1 | cytosolic iron-sulfur assembly component 1 | **0,991** | 0,747412 | 39,438 | 44,503 |
| PIBF1 | progesterone immunomodulatory binding factor 1 | **0,991** | 0,820522 | 11,410 | 14,366 |
| EVI2A | ecotropic viral integration site 2A | **0,991** | 0,837122 | 11,853 | 15,579 |
| ZNF213-AS1 | ZNF213 antisense RNA 1 (head to head) | **0,991** | 0,89678 | 3,323 | 6,577 |
| SP100 | SP100 nuclear antigen | **0,991** | 0,751908 | 51,457 | 46,419 |
| ASAH2B | N-acylsphingosine amidohydrolase 2B | **0,991** | 0,90615 | 1,772 | 2,362 |
| GPAT4 | glycerol-3-phosphate acyltransferase 4 | **0,991** | 0,751916 | 74,721 | 66,276 |
| ATF4 | activating transcription factor 4 | **0,991** | 0,759343 | 502,998 | 534,998 |
| FRMD3 | FERM domain containing 3 | **0,991** | 0,960576 | 0,332 | 0,702 |
| PPIP5K1 | diphosphoinositol pentakisphosphate kinase 1 | **0,991** | 0,860332 | 7,145 | 9,705 |
| CIART | circadian associated repressor of transcription | **0,991** | 0,851538 | 5,594 | 5,555 |
| UBE2V2 | ubiquitin conjugating enzyme E2 V2 | **0,991** | 0,743863 | 43,537 | 50,250 |
| CDC42 | cell division cycle 42 | **0,991** | 0,731718 | 100,201 | 91,305 |
| PTPN18 | protein tyrosine phosphatase, non-receptor type 18 | **0,991** | 0,798091 | 31,185 | 26,498 |
| NKAP | NFKB activating protein | **0,991** | 0,807959 | 19,331 | 19,857 |
| RBM25 | RNA binding motif protein 25 | **0,991** | 0,759719 | 59,323 | 63,978 |
| NME3 | NME/NM23 nucleoside diphosphate kinase 3 | **0,991** | 0,885109 | 5,096 | 3,576 |
| FBXO38 | F-box protein 38 | **0,991** | 0,794909 | 26,089 | 21,773 |
| SHROOM4 | shroom family member 4 | **0,991** | 0,879564 | 10,912 | 11,429 |
| NBPF9 | NBPF member 9 | **0,991** | 0,879887 | 3,656 | 6,832 |
| ATG5 | autophagy related 5 | **0,991** | 0,809028 | 25,590 | 28,222 |
| LRWD1 | leucine rich repeats and WD repeat domain containing 1 | **0,991** | 0,783326 | 19,110 | 21,709 |
| SLC35A4 | solute carrier family 35 member A4 | **0,991** | 0,723557 | 65,250 | 73,619 |
| DYNLT1 | dynein light chain Tctex-type 1 | **0,991** | 0,779258 | 32,791 | 30,009 |
| CD68 | CD68 molecule | **0,991** | 0,893006 | 4,487 | 1,915 |
| SMC5 | structural maintenance of chromosomes 5 | **0,991** | 0,827889 | 23,596 | 27,519 |

| | | | | | |
|---|---|---|---|---|---|
| RNASEK | ribonuclease K | **0,991** | 0,920583 | 1,939 | 1,277 |
| PMM1 | phosphomannomutase 1 | **0,991** | 0,800949 | 19,054 | 15,835 |
| MAGT1 | magnesium transporter 1 | **0,991** | 0,767859 | 104,687 | 72,853 |
| SOAT1 | sterol O-acyltransferase 1 | **0,991** | 0,760555 | 33,788 | 31,989 |
| PI4KB | phosphatidylinositol 4-kinase beta | **0,991** | 0,725168 | 58,991 | 51,335 |
| COX4I1 | cytochrome c oxidase subunit 4I1 | **0,991** | 0,793945 | 81,811 | 75,854 |
| NUDC | nuclear distribution C, dynein complex regulator | **0,991** | 0,811898 | 54,061 | 60,721 |
| MRI1 | methylthioribose-1-phosphate isomerase 1 | **0,991** | 0,853456 | 10,746 | 8,939 |
| SENP1 | SUMO1/sentrin specific peptidase 1 | **0,991** | 0,805779 | 20,993 | 24,135 |
| CBX1 | chromobox 1 | **0,991** | 0,748913 | 56,332 | 50,186 |
| LBH | limb bud and heart development | **0,991** | 0,824711 | 37,998 | 39,204 |
| PSMC6 | proteasome 26S subunit, ATPase 6 | **0,991** | 0,722655 | 40,379 | 45,206 |
| EID3 | EP300 interacting inhibitor of differentiation 3 | **0,991** | 0,900937 | 0,886 | 2,299 |
| NAT1 | N-acetyltransferase 1 | **0,991** | 0,867237 | 4,487 | 3,639 |
| ANGPTL5 | angiopoietin like 5 | **0,991** | 0,926259 | 3,157 | 1,979 |
| ZC4H2 | zinc finger C4H2-type containing | **0,991** | 0,922522 | 1,385 | 1,660 |
| MADCAM1 | mucosal vascular addressin cell adhesion molecule 1 | **0,991** | 0,959441 | 0,277 | 0,383 |
| ACAD10 | acyl-CoA dehydrogenase family member 10 | **0,991** | 0,807657 | 9,416 | 12,068 |
| C5orf15 | chromosome 5 open reading frame 15 | **0,991** | 0,759488 | 92,114 | 73,300 |
| CLASP1 | cytoplasmic linker associated protein 1 | **0,991** | 0,83765 | 40,269 | 37,735 |
| C9orf50 | chromosome 9 open reading frame 50 | **0,991** | 0,958943 | 0,388 | 0,255 |
| SNRNP27 | small nuclear ribonucleoprotein U4/U6.U5 subunit 27 | **0,991** | 0,78642 | 24,815 | 24,071 |
| NUMA1 | nuclear mitotic apparatus protein 1 | **0,991** | 0,753155 | 161,795 | 163,839 |
| ARIH2 | ariadne RBR E3 ubiquitin protein ligase 2 | **0,991** | 0,725534 | 41,432 | 40,800 |
| PROS1 | protein S (alpha) | **0,990** | 0,751414 | 22,433 | 19,538 |
| SERPING1 | serpin family G member 1 | **0,990** | 0,752162 | 123,908 | 115,441 |
| ZNF821 | zinc finger protein 821 | **0,990** | 0,90283 | 1,939 | 1,724 |
| TMBIM6 | transmembrane BAX inhibitor motif containing 6 | **0,990** | 0,691556 | 538,946 | 478,810 |
| C5orf63 | chromosome 5 open reading frame 63 | **0,990** | 0,896892 | 3,102 | 3,703 |
| C14orf159 | chromosome 14 open reading frame 159 | **0,990** | 0,915337 | 2,326 | 1,660 |
| ARSD | arylsulfatase D | **0,990** | 0,771445 | 16,395 | 18,453 |
| NINL | ninein like | **0,990** | 0,894062 | 2,880 | 3,512 |
| SIPA1L1 | signal induced proliferation associated 1 like 1 | **0,990** | 0,803952 | 49,851 | 43,354 |

| | | | | | |
|---|---|---|---|---|---|
| CCDC116 | coiled-coil domain containing 116 | **0,990** | 0,95767 | 0,166 | 0,319 |
| FAM229B | family with sequence similarity 229 member B | **0,990** | 0,819474 | 6,758 | 7,662 |
| PRUNE1 | prune exopolyphosphatase 1 | **0,990** | 0,752482 | 18,611 | 21,645 |
| RPS6KA2-IT1 | RPS6KA2 intronic transcript 1 | **0,990** | 0,960552 | 0,277 | 0,575 |
| TSPAN10 | tetraspanin 10 | **0,990** | 0,884137 | 2,936 | 5,236 |
| SH3RF3-AS1 | SH3RF3 antisense RNA 1 | **0,990** | 0,925272 | 0,942 | 2,107 |
| DCAF12 | DDB1 and CUL4 associated factor 12 | **0,990** | 0,744959 | 31,018 | 26,242 |
| APBB2 | amyloid beta precursor protein binding family B member 2 | **0,990** | 0,813744 | 30,298 | 38,374 |
| ASNSD1 | asparagine synthetase domain containing 1 | **0,990** | 0,726072 | 44,866 | 51,016 |
| ZNF599 | zinc finger protein 599 | **0,990** | 0,878385 | 2,382 | 4,916 |
| WBP1 | WW domain binding protein 1 | **0,990** | 0,911554 | 2,326 | 3,576 |
| ADK | adenosine kinase | **0,990** | 0,712386 | 81,091 | 71,129 |
| BCL7A | BCL tumor suppressor 7A | **0,990** | 0,923107 | 1,052 | 1,277 |
| TBC1D8-AS1 | TBC1 domain family member 8 antisense RNA 1 | **0,990** | 0,929477 | 0,775 | 0,958 |
| STX4 | syntaxin 4 | **0,990** | 0,74286 | 40,102 | 34,287 |
| PEX11B | peroxisomal biogenesis factor 11 beta | **0,990** | 0,824485 | 9,416 | 6,577 |
| DDX50 | DExD-box helicase 50 | **0,990** | 0,707974 | 31,351 | 31,606 |
| FBXL12 | F-box and leucine rich repeat protein 12 | **0,990** | 0,7954 | 12,851 | 13,855 |
| CEP170B | centrosomal protein 170B | **0,990** | 0,758445 | 43,537 | 35,820 |
| PRKD3 | protein kinase D3 | **0,990** | 0,780031 | 29,966 | 27,839 |
| ALKBH6 | alkB homolog 6 | **0,990** | 0,932411 | 1,329 | 1,149 |
| GDAP2 | ganglioside induced differentiation associated protein 2 | **0,990** | 0,824081 | 13,903 | 11,493 |
| ATF1 | activating transcription factor 1 | **0,990** | 0,764526 | 23,208 | 25,029 |
| RNF149 | ring finger protein 149 | **0,990** | 0,712613 | 43,315 | 38,948 |
| MZF1 | myeloid zinc finger 1 | **0,990** | 0,808972 | 15,122 | 16,984 |
| CD99L2 | CD99 molecule like 2 | **0,990** | 0,715111 | 65,305 | 40,481 |
| WDR59 | WD repeat domain 59 | **0,990** | 0,761914 | 23,984 | 21,262 |
| LINC00667 | long intergenic non-protein coding RNA 667 | **0,990** | 0,708234 | 35,450 | 37,033 |
| KIF3B | kinesin family member 3B | **0,990** | 0,72459 | 85,190 | 74,768 |
| CS | citrate synthase | **0,990** | 0,735796 | 39,936 | 34,287 |
| AQR | aquarius intron-binding spliceosomal factor | **0,990** | 0,760699 | 36,945 | 36,905 |
| RPL37A | ribosomal protein L37a | **0,990** | 0,76222 | 410,552 | 426,006 |
| SLC12A7 | solute carrier family 12 member 7 | **0,990** | 0,934298 | 1,329 | 0,830 |

| | | | | | |
|---|---|---|---|---|---|
| MRPS36 | mitochondrial ribosomal protein S36 | **0,990** | 0,902326 | 3,379 | 2,554 |
| ANPEP | alanyl aminopeptidase, membrane | **0,990** | 0,868956 | 17,725 | 12,578 |
| PAFAH2 | platelet activating factor acetylhydrolase 2 | **0,990** | 0,78698 | 11,853 | 15,707 |
| HERC3 | HECT and RLD domain containing E3 ubiquitin protein ligase 3 | **0,990** | 0,792321 | 18,777 | 12,259 |
| COA5 | cytochrome c oxidase assembly factor 5 | **0,990** | 0,791232 | 10,967 | 10,024 |
| MCRIP1 | MAPK regulated corepressor interacting protein 1 | **0,990** | 0,805783 | 53,008 | 45,206 |
| PRKACA | protein kinase cAMP-activated catalytic subunit alpha | **0,990** | 0,700182 | 41,598 | 40,353 |
| LSM4 | LSM4 homolog, U6 small nuclear RNA and mRNA degradation associated | **0,990** | 0,717186 | 39,826 | 37,735 |
| APC | APC, WNT signaling pathway regulator | **0,990** | 0,838195 | 33,511 | 30,456 |
| RNF168 | ring finger protein 168 | **0,990** | 0,699112 | 23,098 | 30,712 |
| ZNF195 | zinc finger protein 195 | **0,990** | 0,815301 | 9,527 | 10,918 |
| ADCY3 | adenylate cyclase 3 | **0,990** | 0,788869 | 18,113 | 16,920 |
| PLAA | phospholipase A2 activating protein | **0,990** | 0,717729 | 37,056 | 36,331 |
| PSTK | phosphoseryl-tRNA kinase | **0,990** | 0,883473 | 2,271 | 2,682 |
| AKR1B15 | aldo-keto reductase family 1 member B15 | **0,990** | 0,939838 | 1,108 | 0,830 |
| RPP30 | ribonuclease P/MRP subunit p30 | **0,990** | 0,821589 | 11,355 | 13,983 |
| PABPN1 | poly(A) binding protein nuclear 1 | **0,990** | 0,759787 | 31,628 | 28,732 |
| FAM173B | family with sequence similarity 173 member B | **0,990** | 0,789362 | 9,306 | 11,940 |
| NMNAT3 | nicotinamide nucleotide adenylyltransferase 3 | **0,989** | 0,930498 | 0,609 | 1,085 |
| ACTR3 | ARP3 actin related protein 3 homolog | **0,989** | 0,671851 | 213,141 | 175,459 |
| FAM200A | family with sequence similarity 200 member A | **0,989** | 0,816368 | 9,749 | 8,173 |
| C8orf33 | chromosome 8 open reading frame 33 | **0,989** | 0,711411 | 27,917 | 30,073 |
| SNHG9 | small nucleolar RNA host gene 9 | **0,989** | 0,889615 | 1,939 | 2,299 |
| GSTO2 | glutathione S-transferase omega 2 | **0,989** | 0,882345 | 2,437 | 4,278 |
| HSPA8 | heat shock protein family A (Hsp70) member 8 | **0,989** | 0,712818 | 513,024 | 478,108 |
| RPS6KB1 | ribosomal protein S6 kinase B1 | **0,989** | 0,790982 | 16,008 | 15,835 |
| RHCE | Rh blood group CcEe antigens | **0,989** | 0,882656 | 2,049 | 4,533 |
| SMC6 | structural maintenance of chromosomes 6 | **0,989** | 0,76973 | 12,574 | 14,366 |
| PCOLCE | procollagen C-endopeptidase enhancer | **0,989** | 0,720331 | 75,497 | 37,799 |
| NOL7 | nucleolar protein 7 | **0,989** | 0,743091 | 52,953 | 58,933 |
| MIPEPP3 | mitochondrial intermediate peptidase pseudogene 3 | **0,989** | 0,915514 | 0,665 | 1,341 |
| HADHB | hydroxyacyl-CoA dehydrogenase/3-ketoacyl-CoA thiolase/enoyl-CoA hydratase (trifunctio | **0,989** | 0,685849 | 57,495 | 55,166 |
| SLC38A9 | solute carrier family 38 member 9 | **0,989** | 0,83043 | 14,678 | 14,685 |

| | | | | | |
|---|---|---|---|---|---|
| GLE1 | GLE1, RNA export mediator | **0,989** | 0,7513 | 22,987 | 20,560 |
| ATAD1 | ATPase family, AAA domain containing 1 | **0,989** | 0,731942 | 41,653 | 39,076 |
| ACKR2 | atypical chemokine receptor 2 | **0,989** | 0,943707 | 0,499 | 0,638 |
| FAM32A | family with sequence similarity 32 member A | **0,989** | 0,672027 | 42,927 | 41,439 |
| RPL34 | ribosomal protein L34 | **0,989** | 0,757073 | 119,975 | 125,337 |
| CUL5 | cullin 5 | **0,989** | 0,751879 | 29,966 | 27,519 |
| ZNF276 | zinc finger protein 276 | **0,989** | 0,767892 | 13,183 | 13,472 |
| PRPS2 | phosphoribosyl pyrophosphate synthetase 2 | **0,989** | 0,789165 | 7,921 | 7,534 |
| VAC14 | Vac14, PIKFYVE complex component | **0,989** | 0,761867 | 18,500 | 18,772 |
| CHMP4C | charged multivesicular body protein 4C | **0,989** | 0,894989 | 2,437 | 2,426 |
| LYRM1 | LYR motif containing 1 | **0,989** | 0,777001 | 19,608 | 21,709 |
| LAMB1 | laminin subunit beta 1 | **0,989** | 0,903029 | 1,717 | 0,383 |
| ENOX2 | ecto-NOX disulfide-thiol exchanger 2 | **0,989** | 0,816909 | 11,078 | 8,556 |
| RAB11FIP5 | RAB11 family interacting protein 5 | **0,989** | 0,692095 | 152,711 | 154,836 |
| ERAL1 | Era like 12S mitochondrial rRNA chaperone 1 | **0,989** | 0,706898 | 16,672 | 19,921 |
| DIEXF | digestive organ expansion factor homolog (zebrafish) | **0,989** | 0,729291 | 27,917 | 29,818 |
| ABHD13 | abhydrolase domain containing 13 | **0,989** | 0,781513 | 18,611 | 17,112 |
| GTF2IRD2B | GTF2I repeat domain containing 2B | **0,989** | 0,866756 | 2,493 | 3,256 |
| UBE2N | ubiquitin conjugating enzyme E2 N | **0,989** | 0,685746 | 54,892 | 63,786 |
| MTRF1L | mitochondrial translational release factor 1 like | **0,989** | 0,833019 | 6,702 | 7,023 |
| ENY2 | ENY2, transcription and export complex 2 subunit | **0,989** | 0,707741 | 31,628 | 35,756 |
| ADAMTSL2 | ADAMTS like 2 | **0,989** | 0,753627 | 12,851 | 37,033 |
| RPL36A | ribosomal protein L36a | **0,989** | 0,925269 | 1,108 | 1,085 |
| ATAD3C | ATPase family, AAA domain containing 3C | **0,989** | 0,945123 | 1,274 | 1,085 |
| IQCH-AS1 | IQCH antisense RNA 1 | **0,989** | 0,90854 | 1,496 | 0,958 |
| USP48 | ubiquitin specific peptidase 48 | **0,989** | 0,722137 | 28,249 | 27,264 |
| ZNF438 | zinc finger protein 438 | **0,989** | 0,813287 | 5,151 | 5,619 |
| COMMD7 | COMM domain containing 7 | **0,989** | 0,824217 | 10,524 | 8,747 |
| GPN3 | GPN-loop GTPase 3 | **0,989** | 0,751534 | 17,171 | 22,220 |
| ZC3H10 | zinc finger CCCH-type containing 10 | **0,989** | 0,763674 | 14,678 | 12,706 |
| RAB4A | RAB4A, member RAS oncogene family | **0,989** | 0,763796 | 14,734 | 12,131 |
| RNF123 | ring finger protein 123 | **0,989** | 0,793765 | 18,888 | 20,432 |
| HTATSF1 | HIV-1 Tat specific factor 1 | **0,989** | 0,710806 | 56,941 | 61,360 |

| | | | | | |
|---|---|---|---|---|---|
| SMAP1 | small ArfGAP 1 | **0,989** | 0,719196 | 25,036 | 22,603 |
| SNX1 | sorting nexin 1 | **0,989** | 0,668451 | 60,486 | 59,125 |
| GRB10 | growth factor receptor bound protein 10 | **0,989** | 0,703365 | 30,908 | 26,306 |
| HSD17B4 | hydroxysteroid 17-beta dehydrogenase 4 | **0,989** | 0,717687 | 44,091 | 34,607 |
| PAFAH1B1 | platelet activating factor acetylhydrolase 1b regulatory subunit 1 | **0,988** | 0,675837 | 104,466 | 87,474 |
| PEBP1 | phosphatidylethanolamine binding protein 1 | **0,988** | 0,700637 | 85,467 | 71,576 |
| PLOD3 | procollagen-lysine,2-oxoglutarate 5-dioxygenase 3 | **0,988** | 0,691492 | 171,876 | 100,564 |
| IL31RA | interleukin 31 receptor A | **0,988** | 0,95001 | 0,831 | 0,447 |
| DUSP11 | dual specificity phosphatase 11 | **0,988** | 0,715148 | 21,270 | 23,433 |
| PHACTR4 | phosphatase and actin regulator 4 | **0,988** | 0,701194 | 30,963 | 24,582 |
| PPP2CB | protein phosphatase 2 catalytic subunit beta | **0,988** | 0,667897 | 89,511 | 80,132 |
| C11orf54 | chromosome 11 open reading frame 54 | **0,988** | 0,792733 | 9,804 | 11,046 |
| RBM41 | RNA binding motif protein 41 | **0,988** | 0,84515 | 4,708 | 3,959 |
| CSNK1A1 | casein kinase 1 alpha 1 | **0,988** | 0,68806 | 80,980 | 79,046 |
| STAP2 | signal transducing adaptor family member 2 | **0,988** | 0,888296 | 2,603 | 2,107 |
| ZMYND19 | zinc finger MYND-type containing 19 | **0,988** | 0,714444 | 15,454 | 15,962 |
| SLC25A23 | solute carrier family 25 member 23 | **0,988** | 0,84106 | 6,647 | 3,831 |
| TM2D1 | TM2 domain containing 1 | **0,988** | 0,767163 | 20,882 | 19,347 |
| CAB39 | calcium binding protein 39 | **0,988** | 0,626643 | 96,822 | 78,025 |
| REPS1 | RALBP1 associated Eps domain containing 1 | **0,988** | 0,734735 | 17,448 | 21,837 |
| MED27 | mediator complex subunit 27 | **0,988** | 0,797553 | 8,364 | 11,110 |
| TRPA1 | transient receptor potential cation channel subfamily A member 1 | **0,988** | 0,949839 | 0,166 | 0,383 |
| FAM210A | family with sequence similarity 210 member A | **0,988** | 0,715686 | 18,334 | 18,644 |
| PUS7 | pseudouridylate synthase 7 (putative) | **0,988** | 0,734654 | 14,014 | 13,983 |
| C10orf88 | chromosome 10 open reading frame 88 | **0,988** | 0,784001 | 8,087 | 7,981 |
| LPP-AS2 | LPP antisense RNA 2 | **0,988** | 0,882355 | 2,493 | 1,469 |
| SGTB | small glutamine rich tetratricopeptide repeat containing beta | **0,988** | 0,789579 | 15,011 | 13,919 |
| ULK3 | unc-51 like kinase 3 | **0,988** | 0,754469 | 13,349 | 15,771 |
| FTO | FTO, alpha-ketoglutarate dependent dioxygenase | **0,988** | 0,686515 | 27,418 | 32,627 |
| PRX | periaxin | **0,988** | 0,899507 | 1,772 | 2,171 |
| TOX4 | TOX high mobility group box family member 4 | **0,988** | 0,656712 | 48,411 | 44,759 |
| MED31 | mediator complex subunit 31 | **0,988** | 0,824017 | 7,533 | 7,215 |
| GUF1 | GUF1 homolog, GTPase | **0,988** | 0,806787 | 13,571 | 13,536 |

| | | | | | |
|---|---|---|---|---|---|
| ANGPTL6 | angiopoietin like 6 | **0,988** | 0,902914 | 1,772 | 1,660 |
| RPS7 | ribosomal protein S7 | **0,988** | 0,706866 | 123,243 | 135,936 |
| RPL12 | ribosomal protein L12 | **0,988** | 0,72713 | 299,716 | 265,999 |
| LMBR1L | limb development membrane protein 1 like | **0,988** | 0,797663 | 13,848 | 13,472 |
| ADPRHL2 | ADP-ribosylhydrolase like 2 | **0,988** | 0,694345 | 33,677 | 39,842 |
| RUNX2 | runt related transcription factor 2 | **0,988** | 0,940743 | 1,163 | 0,128 |
| IGFBP7 | insulin like growth factor binding protein 7 | **0,988** | 0,680824 | 216,963 | 140,023 |
| GSE1 | Gse1 coiled-coil protein | **0,988** | 0,777354 | 9,693 | 12,515 |
| PLPP3 | phospholipid phosphatase 3 | **0,988** | 0,685974 | 238,621 | 201,702 |
| SRCAP | Snf2 related CREBBP activator protein | **0,988** | 0,874059 | 4,431 | 4,597 |
| MOB3C | MOB kinase activator 3C | **0,988** | 0,79365 | 12,075 | 10,152 |
| 8.syys | septin 8 | **0,988** | 0,7136 | 30,409 | 33,585 |
| RARRES1 | retinoic acid receptor responder 1 | **0,988** | 0,833956 | 6,647 | 4,533 |
| STMN1 | stathmin 1 | **0,988** | 0,814146 | 20,051 | 2,043 |
| MTO1 | mitochondrial tRNA translation optimization 1 | **0,988** | 0,67838 | 19,497 | 25,093 |
| MRS2 | MRS2, magnesium transporter | **0,988** | 0,769067 | 11,687 | 10,599 |
| DSTN | destrin, actin depolymerizing factor | **0,988** | 0,657948 | 235,851 | 205,724 |
| ZNF395 | zinc finger protein 395 | **0,988** | 0,688928 | 40,269 | 40,736 |
| TMEM186 | transmembrane protein 186 | **0,988** | 0,754809 | 12,130 | 15,771 |
| EIF3H | eukaryotic translation initiation factor 3 subunit H | **0,988** | 0,650713 | 85,301 | 78,408 |
| MTERF1 | mitochondrial transcription termination factor 1 | **0,988** | 0,799831 | 8,032 | 7,917 |
| PIGT | phosphatidylinositol glycan anchor biosynthesis class T | **0,988** | 0,627161 | 142,630 | 100,947 |
| GPR155 | G protein-coupled receptor 155 | **0,988** | 0,833543 | 5,373 | 4,023 |
| RPL38 | ribosomal protein L38 | **0,988** | 0,725696 | 147,005 | 152,346 |
| RPLP0P6 | ribosomal protein lateral stalk subunit P0 pseudogene 6 | **0,988** | 0,876113 | 3,046 | 2,809 |
| POLR2G | RNA polymerase II subunit G | **0,988** | 0,712192 | 30,243 | 31,095 |
| PLIN2 | perilipin 2 | **0,988** | 0,771973 | 17,282 | 10,088 |
| C18orf54 | chromosome 18 open reading frame 54 | **0,987** | 0,853468 | 3,988 | 2,171 |
| PPP1R21 | protein phosphatase 1 regulatory subunit 21 | **0,987** | 0,784235 | 11,466 | 9,833 |
| EIF2AK3 | eukaryotic translation initiation factor 2 alpha kinase 3 | **0,987** | 0,704287 | 45,918 | 39,332 |
| MIS18BP1 | MIS18 binding protein 1 | **0,987** | 0,810403 | 8,807 | 7,470 |
| SFT2D2 | SFT2 domain containing 2 | **0,987** | 0,705215 | 31,185 | 24,008 |
| NDUFAF4 | NADH:ubiquinone oxidoreductase complex assembly factor 4 | **0,987** | 0,801005 | 10,192 | 13,281 |

| | | | | | |
|---|---|---|---|---|---|
| DNAJC14 | DnaJ heat shock protein family (Hsp40) member C14 | **0,987** | 0,698485 | 25,535 | 21,645 |
| RPS20 | ribosomal protein S20 | **0,987** | 0,711504 | 294,233 | 321,931 |
| ZNF680 | zinc finger protein 680 | **0,987** | 0,799398 | 5,705 | 5,172 |
| UVSSA | UV stimulated scaffold protein A | **0,987** | 0,764455 | 22,045 | 28,477 |
| GTF2I | general transcription factor IIi | **0,987** | 0,791961 | 18,002 | 12,898 |
| NUP43 | nucleoporin 43 | **0,987** | 0,721685 | 25,092 | 22,858 |
| TCEAL8 | transcription elongation factor A like 8 | **0,987** | 0,702711 | 26,587 | 16,729 |
| TRMT1L | tRNA methyltransferase 1 like | **0,987** | 0,652402 | 22,932 | 25,795 |
| SMIM19 | small integral membrane protein 19 | **0,987** | 0,754451 | 15,343 | 15,388 |
| SCYL3 | SCY1 like pseudokinase 3 | **0,987** | 0,74047 | 12,075 | 13,025 |
| FAHD2A | fumarylacetoacetate hydrolase domain containing 2A | **0,987** | 0,816799 | 5,871 | 5,236 |
| MRPS10 | mitochondrial ribosomal protein S10 | **0,987** | 0,662304 | 22,267 | 25,923 |
| DDX41 | DEAD-box helicase 41 | **0,987** | 0,636695 | 26,033 | 24,582 |
| ZSCAN18 | zinc finger and SCAN domain containing 18 | **0,987** | 0,708796 | 33,013 | 35,820 |
| FAM86JP | family with sequence similarity 86 member J, pseudogene | **0,987** | 0,894906 | 1,219 | 1,979 |
| MAP9 | microtubule associated protein 9 | **0,987** | 0,749289 | 17,614 | 22,539 |
| CA2 | carbonic anhydrase 2 | **0,987** | 0,918119 | 1,883 | 0,830 |
| THNSL2 | threonine synthase like 2 | **0,987** | 0,76422 | 14,678 | 14,877 |
| MMP14 | matrix metallopeptidase 14 | **0,987** | 0,657116 | 865,305 | 648,779 |
| SLC31A1 | solute carrier family 31 member 1 | **0,987** | 0,639235 | 80,759 | 85,240 |
| ERLIN1 | ER lipid raft associated 1 | **0,987** | 0,63309 | 43,980 | 34,926 |
| PRIM2 | primase (DNA) subunit 2 | **0,987** | 0,834706 | 3,323 | 2,809 |
| DCN | decorin | **0,987** | 0,60978 | 7864,517 | 5020,133 |
| ANKS1A | ankyrin repeat and sterile alpha motif domain containing 1A | **0,987** | 0,756716 | 16,451 | 15,260 |
| PSMA5 | proteasome subunit alpha 5 | **0,987** | 0,651204 | 56,332 | 54,272 |
| EEF1A1P11 | eukaryotic translation elongation factor 1 alpha 1 pseudogene 11 | **0,987** | 0,9115 | 0,775 | 1,213 |
| STK11IP | serine/threonine kinase 11 interacting protein | **0,987** | 0,754061 | 10,081 | 12,387 |
| APBB1 | amyloid beta precursor protein binding family B member 1 | **0,987** | 0,705109 | 27,363 | 19,857 |
| SETD4 | SET domain containing 4 | **0,987** | 0,766396 | 10,136 | 10,854 |
| RBCK1 | RANBP2-type and C3HC4-type zinc finger containing 1 | **0,987** | 0,618095 | 54,781 | 53,506 |
| MRPL47 | mitochondrial ribosomal protein L47 | **0,987** | 0,710909 | 22,876 | 26,817 |
| ADGRG2 | adhesion G protein-coupled receptor G2 | **0,987** | 0,76916 | 37,942 | 29,626 |
| RYK | receptor-like tyrosine kinase | **0,987** | 0,629375 | 55,944 | 54,592 |

| | | | | | |
|---|---|---|---|---|---|
| RPL9 | ribosomal protein L9 | **0,987** | 0,693416 | 196,857 | 223,538 |
| WASHC1 | WASH complex subunit 1 | **0,987** | 0,883065 | 2,659 | 2,873 |
| PIN1 | peptidylprolyl cis/trans isomerase, NIMA-interacting 1 | **0,987** | 0,708134 | 26,366 | 25,093 |
| ZC3H8 | zinc finger CCCH-type containing 8 | **0,987** | 0,816995 | 4,985 | 9,577 |
| FAM214B | family with sequence similarity 214 member B | **0,987** | 0,702953 | 34,342 | 28,222 |
| MIR100HG | mir-100-let-7a-2 cluster host gene | **0,987** | 0,656378 | 249,533 | 308,970 |
| EPB41L1 | erythrocyte membrane protein band 4.1 like 1 | **0,987** | 0,679711 | 11,632 | 13,089 |
| ZNF43 | zinc finger protein 43 | **0,987** | 0,850161 | 2,603 | 2,362 |
| KDELC2 | KDEL motif containing 2 | **0,987** | 0,682426 | 58,326 | 40,609 |
| ALMS1 | ALMS1, centrosome and basal body associated protein | **0,987** | 0,843667 | 21,768 | 25,221 |
| ILF3 | interleukin enhancer binding factor 3 | **0,987** | 0,593889 | 126,954 | 140,917 |
| SPSB3 | splA/ryanodine receptor domain and SOCS box containing 3 | **0,987** | 0,683132 | 23,762 | 17,495 |
| EIF4G2 | eukaryotic translation initiation factor 4 gamma 2 | **0,987** | 0,64472 | 774,133 | 667,870 |
| TMEM175 | transmembrane protein 175 | **0,987** | 0,791262 | 9,582 | 10,152 |
| SET | SET nuclear proto-oncogene | **0,987** | 0,583986 | 176,307 | 164,030 |
| IGBP1 | immunoglobulin binding protein 1 | **0,987** | 0,625219 | 33,068 | 30,201 |
| MCM9 | minichromosome maintenance 9 homologous recombination repair factor | **0,987** | 0,805605 | 5,207 | 7,023 |
| SNX6 | sorting nexin 6 | **0,987** | 0,629335 | 41,598 | 32,819 |
| FAM50A | family with sequence similarity 50 member A | **0,987** | 0,684666 | 65,305 | 64,105 |
| OSER1-AS1 | OSER1 antisense RNA 1 (head to head) | **0,987** | 0,736028 | 9,804 | 9,067 |
| CDK5RAP1 | CDK5 regulatory subunit associated protein 1 | **0,987** | 0,715469 | 11,632 | 13,408 |
| ACTN1 | actinin alpha 1 | **0,987** | 0,665322 | 143,184 | 126,742 |
| ST3GAL3 | ST3 beta-galactoside alpha-2,3-sialyltransferase 3 | **0,987** | 0,883427 | 1,828 | 1,915 |
| MCL1 | MCL1, BCL2 family apoptosis regulator | **0,987** | 0,633321 | 339,542 | 367,584 |
| PTPN21 | protein tyrosine phosphatase, non-receptor type 21 | **0,987** | 0,749169 | 29,578 | 28,158 |
| NMD3 | NMD3 ribosome export adaptor | **0,986** | 0,639781 | 39,881 | 44,056 |
| TTLL12 | tubulin tyrosine ligase like 12 | **0,986** | 0,677637 | 24,372 | 21,964 |
| HMGXB3 | HMG-box containing 3 | **0,986** | 0,624559 | 38,552 | 36,331 |
| CD79B | CD79b molecule | **0,986** | 0,924855 | 0,665 | 0,511 |
| CEP97 | centrosomal protein 97 | **0,986** | 0,777721 | 10,192 | 11,621 |
| UPF3A | UPF3 regulator of nonsense transcripts homolog A (yeast) | **0,986** | 0,702274 | 17,337 | 17,686 |
| HSPB6 | heat shock protein family B (small) member 6 | **0,986** | 0,893257 | 1,606 | 0,958 |
| TPD52L1 | tumor protein D52 like 1 | **0,986** | 0,720571 | 37,831 | 36,075 |

| | | | | | |
|---|---|---|---|---|---|
| UPF3B | UPF3 regulator of nonsense transcripts homolog B (yeast) | **0,986** | 0,752456 | 16,229 | 16,473 |
| POLR2B | RNA polymerase II subunit B | **0,986** | 0,636219 | 78,931 | 79,429 |
| DISP1 | dispatched RND transporter family member 1 | **0,986** | 0,810942 | 8,032 | 7,470 |
| TRIM32 | tripartite motif containing 32 | **0,986** | 0,70619 | 23,762 | 20,049 |
| DLG1 | discs large MAGUK scaffold protein 1 | **0,986** | 0,69372 | 50,460 | 36,905 |
| INPP5E | inositol polyphosphate-5-phosphatase E | **0,986** | 0,753402 | 13,460 | 12,195 |
| SMC2 | structural maintenance of chromosomes 2 | **0,986** | 0,739873 | 20,882 | 16,729 |
| LONP2 | lon peptidase 2, peroxisomal | **0,986** | 0,653202 | 39,050 | 39,459 |
| METAP2 | methionyl aminopeptidase 2 | **0,986** | 0,668 | 78,266 | 86,261 |
| RSBN1L | round spermatid basic protein 1 like | **0,986** | 0,688705 | 17,337 | 19,730 |
| CASK | calcium/calmodulin dependent serine protein kinase | **0,986** | 0,62054 | 36,004 | 39,204 |
| ZNF446 | zinc finger protein 446 | **0,986** | 0,805474 | 7,256 | 9,067 |
| MYL5 | myosin light chain 5 | **0,986** | 0,8016 | 6,481 | 9,577 |
| S100A6 | S100 calcium binding protein A6 | **0,986** | 0,648778 | 433,317 | 284,387 |
| ZNF236 | zinc finger protein 236 | **0,986** | 0,848058 | 9,582 | 10,663 |
| THOC7 | THO complex 7 | **0,986** | 0,70497 | 24,206 | 22,220 |
| TUBGCP4 | tubulin gamma complex associated protein 4 | **0,986** | 0,744525 | 10,524 | 9,897 |
| LAMTOR1 | late endosomal/lysosomal adaptor, MAPK and MTOR activator 1 | **0,986** | 0,65689 | 53,285 | 52,868 |
| RPS28 | ribosomal protein S28 | **0,986** | 0,703639 | 104,964 | 129,104 |
| PSMG3-AS1 | PSMG3 antisense RNA 1 (head to head) | **0,986** | 0,74234 | 10,912 | 9,258 |
| COX15 | COX15, cytochrome c oxidase assembly homolog | **0,986** | 0,633503 | 17,171 | 19,091 |
| SAP30L | SAP30 like | **0,986** | 0,692299 | 24,649 | 17,750 |
| LINC01235 | long intergenic non-protein coding RNA 1235 | **0,986** | 0,917103 | 0,665 | 0,128 |
| ALG2 | ALG2, alpha-1,3/1,6-mannosyltransferase | **0,986** | 0,6155 | 61,317 | 67,106 |
| UBLCP1 | ubiquitin like domain containing CTD phosphatase 1 | **0,986** | 0,64831 | 20,882 | 22,922 |
| ATPAF1 | ATP synthase mitochondrial F1 complex assembly factor 1 | **0,986** | 0,596886 | 25,535 | 22,156 |
| KMT5A | lysine methyltransferase 5A | **0,986** | 0,661314 | 14,678 | 11,365 |
| IFT88 | intraflagellar transport 88 | **0,986** | 0,684341 | 11,466 | 13,345 |
| PSPN | persephin | **0,986** | 0,907242 | 0,997 | 1,532 |
| PITRM1 | pitrilysin metallopeptidase 1 | **0,986** | 0,565783 | 118,203 | 112,376 |
| MMS19 | MMS19 homolog, cytosolic iron-sulfur assembly component | **0,986** | 0,620602 | 24,095 | 26,370 |
| MBTPS2 | membrane bound transcription factor peptidase, site 2 | **0,986** | 0,63735 | 36,447 | 37,608 |
| GID8 | GID complex subunit 8 homolog | **0,986** | 0,570496 | 48,577 | 51,080 |

| | | | | | |
|---|---|---|---|---|---|
| PHLDB1 | pleckstrin homology like domain family B member 1 | **0,986** | 0,74623 | 45,863 | 34,032 |
| BEX4 | brain expressed X-linked 4 | **0,986** | 0,671727 | 24,482 | 18,644 |
| DHFR2 | dihydrofolate reductase 2 | **0,986** | 0,725403 | 17,836 | 17,814 |
| ATG13 | autophagy related 13 | **0,986** | 0,584526 | 63,422 | 64,744 |
| RPN2 | ribophorin II | **0,986** | 0,544764 | 533,463 | 351,494 |
| CHPF | chondroitin polymerizing factor | **0,986** | 0,652867 | 368,622 | 297,030 |
| CXorf56 | chromosome X open reading frame 56 | **0,986** | 0,697458 | 14,678 | 15,132 |
| ERCC4 | ERCC excision repair 4, endonuclease catalytic subunit | **0,986** | 0,714874 | 17,946 | 19,410 |
| IRAK4 | interleukin 1 receptor associated kinase 4 | **0,986** | 0,717513 | 12,684 | 10,216 |
| NUDCD3 | NudC domain containing 3 | **0,986** | 0,602142 | 54,116 | 60,274 |
| ZNF706 | zinc finger protein 706 | **0,986** | 0,621525 | 26,698 | 25,540 |
| CD40 | CD40 molecule | **0,986** | 0,793157 | 6,813 | 6,960 |
| TTC31 | tetratricopeptide repeat domain 31 | **0,986** | 0,713601 | 13,792 | 15,132 |
| SZT2 | seizure threshold 2 homolog (mouse) | **0,986** | 0,749166 | 43,094 | 49,803 |
| GBE1 | 1,4-alpha-glucan branching enzyme 1 | **0,986** | 0,578288 | 280,440 | 177,183 |
| C5orf66 | chromosome 5 open reading frame 66 | **0,986** | 0,896406 | 1,219 | 1,724 |
| GAK | cyclin G associated kinase | **0,985** | 0,611097 | 42,152 | 51,144 |
| FAM86C2P | family with sequence similarity 86 member C2, pseudogene | **0,985** | 0,886462 | 0,997 | 1,277 |
| SAFB2 | scaffold attachment factor B2 | **0,985** | 0,63693 | 48,466 | 55,166 |
| NAA50 | N(alpha)-acetyltransferase 50, NatE catalytic subunit | **0,985** | 0,600264 | 82,199 | 69,852 |
| RAB1B | RAB1B, member RAS oncogene family | **0,985** | 0,592961 | 114,381 | 99,606 |
| ARMT1 | acidic residue methyltransferase 1 | **0,985** | 0,736536 | 26,587 | 21,326 |
| ERP44 | endoplasmic reticulum protein 44 | **0,985** | 0,546698 | 93,055 | 85,303 |
| EFL1 | elongation factor like GTPase 1 | **0,985** | 0,619662 | 48,189 | 43,354 |
| EIF4H | eukaryotic translation initiation factor 4H | **0,985** | 0,559566 | 116,209 | 122,273 |
| CKAP2 | cytoskeleton associated protein 2 | **0,985** | 0,813793 | 12,518 | 5,172 |
| HACD4 | 3-hydroxyacyl-CoA dehydratase 4 | **0,985** | 0,888463 | 2,216 | 0,830 |
| WDR35 | WD repeat domain 35 | **0,985** | 0,752303 | 14,512 | 18,644 |
| YTHDF1 | YTH N6-methyladenosine RNA binding protein 1 | **0,985** | 0,633122 | 39,327 | 39,012 |
| MRFAP1 | Morf4 family associated protein 1 | **0,985** | 0,590592 | 282,490 | 297,732 |
| GOLGA4 | golgin A4 | **0,985** | 0,567571 | 134,044 | 131,850 |
| FAM84B | family with sequence similarity 84 member B | **0,985** | 0,807251 | 2,714 | 3,320 |
| LSM8 | LSM8 homolog, U6 small nuclear RNA associated | **0,985** | 0,670009 | 13,626 | 14,175 |

| | | | | | |
|---|---|---|---|---|---|
| PAF1 | PAF1 homolog, Paf1/RNA polymerase II complex component | **0,985** | 0,593747 | 39,272 | 36,714 |
| HRH1 | histamine receptor H1 | **0,985** | 0,598851 | 173,648 | 250,611 |
| RHOA | ras homolog family member A | **0,985** | 0,595555 | 368,345 | 362,157 |
| RMDN1 | regulator of microtubule dynamics 1 | **0,985** | 0,666138 | 20,273 | 17,112 |
| ARHGEF2 | Rho/Rac guanine nucleotide exchange factor 2 | **0,985** | 0,575927 | 301,378 | 348,556 |
| CIR1 | corepressor interacting with RBPJ, 1 | **0,985** | 0,685708 | 26,532 | 25,732 |
| HAUS5 | HAUS augmin like complex subunit 5 | **0,985** | 0,756571 | 5,761 | 5,363 |
| PPP3CB | protein phosphatase 3 catalytic subunit beta | **0,985** | 0,58376 | 47,414 | 47,249 |
| HSPB1 | heat shock protein family B (small) member 1 | **0,985** | 0,820872 | 7,699 | 7,790 |
| CD3EAP | CD3e molecule associated protein | **0,985** | 0,723569 | 7,810 | 9,450 |
| BMP4 | bone morphogenetic protein 4 | **0,985** | 0,864799 | 1,883 | 1,724 |
| MDN1 | midasin AAA ATPase 1 | **0,985** | 0,761594 | 29,689 | 42,652 |
| TATDN1 | TatD DNase domain containing 1 | **0,985** | 0,731254 | 8,862 | 10,408 |
| GINM1 | glycoprotein integral membrane 1 | **0,985** | 0,580764 | 74,389 | 52,804 |
| H3F3A | H3 histone family member 3A | **0,985** | 0,840217 | 2,493 | 2,043 |
| ABHD6 | abhydrolase domain containing 6 | **0,985** | 0,801156 | 6,868 | 4,916 |
| PSMD3 | proteasome 26S subunit, non-ATPase 3 | **0,985** | 0,563725 | 97,764 | 103,820 |
| PAPOLA | poly(A) polymerase alpha | **0,985** | 0,587582 | 113,605 | 115,632 |
| ARID3B | AT-rich interaction domain 3B | **0,985** | 0,864038 | 1,994 | 1,979 |
| EPDR1 | ependymin related 1 | **0,985** | 0,781272 | 5,262 | 3,384 |
| CUL2 | cullin 2 | **0,985** | 0,666869 | 20,605 | 19,666 |
| DENND2C | DENN domain containing 2C | **0,985** | 0,835595 | 2,991 | 3,576 |
| C11orf57 | chromosome 11 open reading frame 57 | **0,985** | 0,624158 | 22,433 | 19,091 |
| MAD1L1 | MAD1 mitotic arrest deficient like 1 | **0,985** | 0,731661 | 14,789 | 15,643 |
| TUBB6 | tubulin beta 6 class V | **0,985** | 0,584265 | 116,486 | 98,010 |
| SLAIN2 | SLAIN motif family member 2 | **0,985** | 0,663713 | 51,568 | 50,314 |
| FAM96A | family with sequence similarity 96 member A | **0,985** | 0,620902 | 16,119 | 13,345 |
| CTTN | cortactin | **0,985** | 0,561862 | 147,227 | 112,376 |
| TMEM176B | transmembrane protein 176B | **0,985** | 0,939371 | 0,332 | 0,192 |
| HOXB2 | homeobox B2 | **0,985** | 0,802124 | 5,594 | 8,620 |
| POMP | proteasome maturation protein | **0,985** | 0,608781 | 52,621 | 51,591 |
| RCE1 | Ras converting CAAX endopeptidase 1 | **0,985** | 0,674668 | 10,303 | 12,068 |
| ARSA | arylsulfatase A | **0,985** | 0,631583 | 42,872 | 25,476 |

| | | | | | |
|---|---|---|---|---|---|
| PRPS1 | phosphoribosyl pyrophosphate synthetase 1 | **0,985** | 0,612708 | 25,978 | 31,286 |
| ATP6V0E2-AS1 | ATP6V0E2 antisense RNA 1 | **0,985** | 0,873055 | 1,219 | 1,532 |
| SPG20 | spastic paraplegia 20 (Troyer syndrome) | **0,985** | 0,624718 | 89,843 | 87,474 |
| EWSR1 | EWS RNA binding protein 1 | **0,985** | 0,53224 | 119,754 | 135,681 |
| POLR3C | RNA polymerase III subunit C | **0,985** | 0,645438 | 19,830 | 20,113 |
| CSNK2A1 | casein kinase 2 alpha 1 | **0,985** | 0,565931 | 57,329 | 60,274 |
| REPIN1 | replication initiator 1 | **0,985** | 0,617839 | 46,417 | 42,907 |
| IFT74 | intraflagellar transport 74 | **0,985** | 0,720884 | 13,183 | 11,812 |
| PDIK1L | PDLIM1 interacting kinase 1 like | **0,985** | 0,770345 | 6,591 | 6,768 |
| CPSF7 | cleavage and polyadenylation specific factor 7 | **0,985** | 0,585372 | 49,076 | 54,783 |
| PYGL | glycogen phosphorylase L | **0,985** | 0,671973 | 24,095 | 19,091 |
| A4GALT | alpha 1,4-galactosyltransferase (P blood group) | **0,985** | 0,622602 | 154,095 | 164,733 |
| PHF5A | PHD finger protein 5A | **0,985** | 0,655746 | 21,381 | 21,390 |
| UACA | uveal autoantigen with coiled-coil domains and ankyrin repeats | **0,984** | 0,600463 | 33,068 | 46,610 |
| ZNF503-AS1 | ZNF503 antisense RNA 1 | **0,984** | 0,917531 | 0,997 | 0,575 |
| GPATCH3 | G-patch domain containing 3 | **0,984** | 0,700777 | 12,906 | 12,642 |
| TULP3 | tubby like protein 3 | **0,984** | 0,582929 | 47,137 | 47,504 |
| ZNF737 | zinc finger protein 737 | **0,984** | 0,868612 | 1,994 | 1,660 |
| CISD2 | CDGSH iron sulfur domain 2 | **0,984** | 0,699622 | 16,451 | 12,259 |
| SETDB1 | SET domain bifurcated 1 | **0,984** | 0,603794 | 15,897 | 18,261 |
| LINS1 | lines homolog 1 | **0,984** | 0,753591 | 6,148 | 8,811 |
| ZNF433 | zinc finger protein 433 | **0,984** | 0,83328 | 1,772 | 1,660 |
| PARD3 | par-3 family cell polarity regulator | **0,984** | 0,696947 | 15,675 | 14,813 |
| WDR70 | WD repeat domain 70 | **0,984** | 0,750534 | 13,238 | 13,983 |
| C1RL | complement C1r subcomponent like | **0,984** | 0,539967 | 41,321 | 30,584 |
| EIF4B | eukaryotic translation initiation factor 4B | **0,984** | 0,534845 | 140,082 | 117,995 |
| PLPP6 | phospholipid phosphatase 6 | **0,984** | 0,708785 | 12,297 | 11,940 |
| PRLR | prolactin receptor | **0,984** | 0,853849 | 1,939 | 1,405 |
| CISD1 | CDGSH iron sulfur domain 1 | **0,984** | 0,743861 | 7,865 | 7,790 |
| SLC35B3 | solute carrier family 35 member B3 | **0,984** | 0,633511 | 21,270 | 21,964 |
| LINC00663 | long intergenic non-protein coding RNA 663 | **0,984** | 0,860023 | 0,942 | 1,852 |
| TMEM171 | transmembrane protein 171 | **0,984** | 0,790988 | 8,364 | 4,342 |
| DNAJC19 | DnaJ heat shock protein family (Hsp40) member C19 | **0,984** | 0,686426 | 12,851 | 15,260 |

| | | | | | |
|---|---|---|---|---|---|
| MAP2K1 | mitogen-activated protein kinase kinase 1 | **0,984** | 0,544772 | 62,148 | 50,697 |
| PARS2 | prolyl-tRNA synthetase 2, mitochondrial (putative) | **0,984** | 0,74822 | 5,317 | 5,427 |
| TCTN2 | tectonic family member 2 | **0,984** | 0,605967 | 12,241 | 17,942 |
| NAA60 | N(alpha)-acetyltransferase 60, NatF catalytic subunit | **0,984** | 0,597695 | 30,575 | 29,690 |
| ZBTB7A | zinc finger and BTB domain containing 7A | **0,984** | 0,539154 | 91,726 | 79,685 |
| TRIO | trio Rho guanine nucleotide exchange factor | **0,984** | 0,70084 | 114,215 | 122,528 |
| EIF4E2 | eukaryotic translation initiation factor 4E family member 2 | **0,984** | 0,564448 | 47,912 | 38,246 |
| RPL19 | ribosomal protein L19 | **0,984** | 0,605092 | 435,145 | 457,995 |
| METTL16 | methyltransferase like 16 | **0,984** | 0,570515 | 31,960 | 43,226 |
| C19orf44 | chromosome 19 open reading frame 44 | **0,984** | 0,707388 | 8,530 | 9,705 |
| ATMIN | ATM interactor | **0,984** | 0,592878 | 51,291 | 42,907 |
| SHROOM1 | shroom family member 1 | **0,984** | 0,713134 | 13,072 | 12,259 |
| LINC00869 | long intergenic non-protein coding RNA 869 | **0,984** | 0,852507 | 1,662 | 1,596 |
| FRMD4B | FERM domain containing 4B | **0,984** | 0,710757 | 13,017 | 10,535 |
| MAPK10 | mitogen-activated protein kinase 10 | **0,984** | 0,88662 | 1,828 | 1,788 |
| IK | IK cytokine, down-regulator of HLA II | **0,984** | 0,561845 | 75,885 | 79,110 |
| METRNL | meteorin like, glial cell differentiation regulator | **0,984** | 0,742477 | 9,859 | 4,086 |
| ATG101 | autophagy related 101 | **0,984** | 0,547027 | 37,998 | 42,460 |
| FAM46B | family with sequence similarity 46 member B | **0,984** | 0,86065 | 1,163 | 1,213 |
| SPATA13 | spermatogenesis associated 13 | **0,984** | 0,941298 | 0,222 | 0,128 |
| ZNF600 | zinc finger protein 600 | **0,984** | 0,775296 | 3,046 | 3,384 |
| RPS6 | ribosomal protein S6 | **0,984** | 0,609523 | 526,428 | 586,589 |
| FAM174A | family with sequence similarity 174 member A | **0,984** | 0,700182 | 13,183 | 12,642 |
| ZNF165 | zinc finger protein 165 | **0,984** | 0,863345 | 1,163 | 1,596 |
| PLOD2 | procollagen-lysine,2-oxoglutarate 5-dioxygenase 2 | **0,984** | 0,589164 | 927,674 | 663,273 |
| FIGN | fidgetin, microtubule severing factor | **0,984** | 0,851645 | 2,603 | 1,213 |
| LZIC | leucine zipper and CTNNBIP1 domain containing | **0,984** | 0,626554 | 20,771 | 26,115 |
| PRPF19 | pre-mRNA processing factor 19 | **0,984** | 0,528864 | 64,031 | 80,387 |
| DOCK1 | dedicator of cytokinesis 1 | **0,984** | 0,622063 | 45,586 | 52,804 |
| CNOT9 | CCR4-NOT transcription complex subunit 9 | **0,984** | 0,559767 | 45,088 | 43,609 |
| NONO | non-POU domain containing, octamer-binding | **0,984** | 0,530418 | 103,801 | 95,839 |
| ZNRF1 | zinc and ring finger 1 | **0,984** | 0,550986 | 33,899 | 39,715 |
| MIGA2 | mitoguardin 2 | **0,984** | 0,604453 | 18,500 | 20,113 |

| | | | | | |
|---|---|---|---|---|---|
| KLHL2 | kelch like family member 2 | **0,984** | 0,55991 | 31,406 | 25,859 |
| NSL1 | NSL1, MIS12 kinetochore complex component | **0,984** | 0,597651 | 24,206 | 24,455 |
| ZSCAN20 | zinc finger and SCAN domain containing 20 | **0,984** | 0,813278 | 5,982 | 5,874 |
| RPS4XP16 | ribosomal protein S4X pseudogene 16 | **0,984** | 0,925308 | 0,277 | 0,383 |
| MCAT | malonyl-CoA-acyl carrier protein transacylase | **0,984** | 0,7086 | 11,743 | 12,387 |
| DNAJB11 | DnaJ heat shock protein family (Hsp40) member B11 | **0,984** | 0,592055 | 43,924 | 33,202 |
| TCEAL2 | transcription elongation factor A like 2 | **0,984** | 0,77391 | 5,705 | 4,916 |
| AQP9 | aquaporin 9 | **0,983** | 0,874336 | 2,714 | 2,299 |
| WDR75 | WD repeat domain 75 | **0,983** | 0,540118 | 28,471 | 33,777 |
| RNLS | renalase, FAD dependent amine oxidase | **0,983** | 0,841027 | 2,105 | 4,086 |
| SLC35A1 | solute carrier family 35 member A1 | **0,983** | 0,667698 | 6,924 | 8,045 |
| SIGMAR1 | sigma non-opioid intracellular receptor 1 | **0,983** | 0,600847 | 32,071 | 30,967 |
| UCP3 | uncoupling protein 3 | **0,983** | 0,897437 | 0,775 | 0,958 |
| DHRS7B | dehydrogenase/reductase 7B | **0,983** | 0,593474 | 16,783 | 15,643 |
| NAE1 | NEDD8 activating enzyme E1 subunit 1 | **0,983** | 0,587065 | 26,753 | 27,966 |
| GATAD1 | GATA zinc finger domain containing 1 | **0,983** | 0,559884 | 32,846 | 37,799 |
| MCCC1 | methylcrotonoyl-CoA carboxylase 1 | **0,983** | 0,754984 | 7,035 | 8,620 |
| DPY19L4 | dpy-19 like 4 (C. elegans) | **0,983** | 0,664918 | 34,840 | 26,498 |
| SMIM10L2A | small integral membrane protein 10 like 2A | **0,983** | 0,867685 | 1,662 | 1,085 |
| GSTM4 | glutathione S-transferase mu 4 | **0,983** | 0,802397 | 2,603 | 2,682 |
| HADHA | hydroxyacyl-CoA dehydrogenase/3-ketoacyl-CoA thiolase/enoyl-CoA hydratase (trifunctio | **0,983** | 0,516159 | 91,615 | 76,301 |
| ODF2 | outer dense fiber of sperm tails 2 | **0,983** | 0,605256 | 28,304 | 30,712 |
| CBWD2 | COBW domain containing 2 | **0,983** | 0,717256 | 8,752 | 5,938 |
| IL23A | interleukin 23 subunit alpha | **0,983** | 0,740999 | 18,223 | 20,943 |
| EID1 | EP300 interacting inhibitor of differentiation 1 | **0,983** | 0,565995 | 159,690 | 159,561 |
| XAF1 | XIAP associated factor 1 | **0,983** | 0,851543 | 2,437 | 1,788 |
| CTSK | cathepsin K | **0,983** | 0,632153 | 55,224 | 39,204 |
| ZNF7 | zinc finger protein 7 | **0,983** | 0,656946 | 12,851 | 13,664 |
| ROBO2 | roundabout guidance receptor 2 | **0,983** | 0,787954 | 4,597 | 1,788 |
| VASH1 | vasohibin 1 | **0,983** | 0,835358 | 1,662 | 2,809 |
| SETD1B | SET domain containing 1B | **0,983** | 0,682665 | 26,643 | 25,348 |
| KIAA1429 | KIAA1429 | **0,983** | 0,59609 | 52,898 | 49,228 |
| MIIP | migration and invasion inhibitory protein | **0,983** | 0,74212 | 5,484 | 6,002 |

| | | | | | |
|---|---|---|---|---|---|
| PNPO | pyridoxamine 5'-phosphate oxidase | **0,983** | 0,676769 | 9,859 | 11,621 |
| FAM89A | family with sequence similarity 89 member A | **0,983** | 0,756539 | 7,256 | 7,215 |
| IRF2BP2 | interferon regulatory factor 2 binding protein 2 | **0,983** | 0,48086 | 71,509 | 70,746 |
| CEP76 | centrosomal protein 76 | **0,983** | 0,767501 | 4,265 | 5,683 |
| CATSPER2 | cation channel sperm associated 2 | **0,983** | 0,830651 | 1,662 | 2,490 |
| LRP10 | LDL receptor related protein 10 | **0,983** | 0,53305 | 741,564 | 581,417 |
| UNKL | unkempt family like zinc finger | **0,983** | 0,513984 | 65,914 | 50,888 |
| CENPQ | centromere protein Q | **0,983** | 0,753465 | 5,650 | 5,619 |
| PPP4R3A | protein phosphatase 4 regulatory subunit 3A | **0,983** | 0,535909 | 51,014 | 51,271 |
| LEO1 | LEO1 homolog, Paf1/RNA polymerase II complex component | **0,983** | 0,618524 | 17,226 | 20,049 |
| RPS15A | ribosomal protein S15a | **0,983** | 0,637449 | 38,330 | 37,416 |
| ENSA | endosulfine alpha | **0,983** | 0,53072 | 89,344 | 95,456 |
| ZNF335 | zinc finger protein 335 | **0,983** | 0,669733 | 22,045 | 24,582 |
| FAHD1 | fumarylacetoacetate hydrolase domain containing 1 | **0,983** | 0,601397 | 24,095 | 21,837 |
| HIGD1A | HIG1 hypoxia inducible domain family member 1A | **0,983** | 0,560901 | 26,421 | 23,688 |
| TNFAIP1 | TNF alpha induced protein 1 | **0,983** | 0,614715 | 25,036 | 20,496 |
| ARAP1 | ArfGAP with RhoGAP domain, ankyrin repeat and PH domain 1 | **0,983** | 0,552171 | 59,378 | 59,380 |
| EIF3D | eukaryotic translation initiation factor 3 subunit D | **0,983** | 0,514183 | 111,667 | 105,225 |
| MTURN | maturin, neural progenitor differentiation regulator homolog | **0,983** | 0,581038 | 42,983 | 37,033 |
| RASSF3 | Ras association domain family member 3 | **0,983** | 0,722237 | 9,029 | 7,726 |
| BLOC1S3 | biogenesis of lysosomal organelles complex 1 subunit 3 | **0,983** | 0,587853 | 16,728 | 13,408 |
| RSL24D1 | ribosomal L24 domain containing 1 | **0,983** | 0,572358 | 76,438 | 77,003 |
| LRRC40 | leucine rich repeat containing 40 | **0,983** | 0,70925 | 10,746 | 10,280 |
| CLINT1 | clathrin interactor 1 | **0,983** | 0,533601 | 114,381 | 120,612 |
| ARMC10 | armadillo repeat containing 10 | **0,982** | 0,745442 | 6,702 | 7,151 |
| GOLPH3 | golgi phosphoprotein 3 | **0,982** | 0,491659 | 101,918 | 101,521 |
| SLC7A11 | solute carrier family 7 member 11 | **0,982** | 0,646276 | 176,473 | 103,054 |
| PDE3B | phosphodiesterase 3B | **0,982** | 0,881098 | 1,828 | 1,149 |
| CAB39L | calcium binding protein 39 like | **0,982** | 0,639199 | 10,967 | 10,727 |
| MFSD9 | major facilitator superfamily domain containing 9 | **0,982** | 0,747224 | 5,982 | 5,810 |
| RASAL2 | RAS protein activator like 2 | **0,982** | 0,605952 | 34,730 | 25,285 |
| ZMYM4 | zinc finger MYM-type containing 4 | **0,982** | 0,608666 | 38,496 | 28,860 |
| WIZ | widely interspaced zinc finger motifs | **0,982** | 0,539645 | 49,519 | 49,548 |

| | | | | | |
|---|---|---|---|---|---|
| SDAD1 | SDA1 domain containing 1 | **0,982** | 0,544331 | 25,424 | 27,839 |
| WDR36 | WD repeat domain 36 | **0,982** | 0,589462 | 18,168 | 23,305 |
| YES1 | YES proto-oncogene 1, Src family tyrosine kinase | **0,982** | 0,56661 | 42,540 | 39,459 |
| UBE2S | ubiquitin conjugating enzyme E2 S | **0,982** | 0,726222 | 18,390 | 14,047 |
| VMA21 | VMA21, vacuolar ATPase assembly factor | **0,982** | 0,616142 | 29,800 | 24,901 |
| EXOSC10 | exosome component 10 | **0,982** | 0,614535 | 37,721 | 35,054 |
| CHRAC1 | chromatin accessibility complex 1 | **0,982** | 0,573335 | 18,223 | 16,090 |
| ELMOD2 | ELMO domain containing 2 | **0,982** | 0,534649 | 23,707 | 21,007 |
| C16orf62 | chromosome 16 open reading frame 62 | **0,982** | 0,586549 | 32,514 | 22,284 |
| ACTR1A | ARP1 actin-related protein 1 homolog A, centractin alpha | **0,982** | 0,52383 | 75,885 | 62,381 |
| TPI1P2 | triosephosphate isomerase 1 pseudogene 2 | **0,982** | 0,871729 | 1,219 | 1,341 |
| PKD1 | polycystin 1, transient receptor potential channel interacting | **0,982** | 0,543697 | 220,564 | 235,159 |
| CCDC15 | coiled-coil domain containing 15 | **0,982** | 0,874298 | 2,105 | 1,788 |
| EVI5 | ecotropic viral integration site 5 | **0,982** | 0,651549 | 16,728 | 16,282 |
| SNRNP25 | small nuclear ribonucleoprotein U11/U12 subunit 25 | **0,982** | 0,731761 | 7,644 | 8,747 |
| HACD3 | 3-hydroxyacyl-CoA dehydratase 3 | **0,982** | 0,503119 | 43,924 | 42,460 |
| VPS16 | VPS16, CORVET/HOPS core subunit | **0,982** | 0,641164 | 11,521 | 9,386 |
| ZNF764 | zinc finger protein 764 | **0,982** | 0,751857 | 6,536 | 5,938 |
| RAD54L2 | RAD54-like 2 (S. cerevisiae) | **0,982** | 0,612731 | 17,614 | 22,731 |
| C4orf26 | chromosome 4 open reading frame 26 | **0,982** | 0,803051 | 7,921 | 2,554 |
| EHD1 | EH domain containing 1 | **0,982** | 0,542262 | 108,565 | 138,235 |
| PEX6 | peroxisomal biogenesis factor 6 | **0,982** | 0,618725 | 26,366 | 17,303 |
| MBL1P | mannose binding lectin 1, pseudogene | **0,982** | 0,932843 | 0,277 | 0,638 |
| MTMR1 | myotubularin related protein 1 | **0,982** | 0,724682 | 7,035 | 7,854 |
| PBRM1 | polybromo 1 | **0,982** | 0,630233 | 47,137 | 40,992 |
| NUDT12 | nudix hydrolase 12 | **0,982** | 0,691687 | 8,530 | 8,428 |
| DOCK4 | dedicator of cytokinesis 4 | **0,982** | 0,697257 | 7,090 | 6,066 |
| MCOLN2 | mucolipin 2 | **0,982** | 0,636192 | 18,113 | 14,239 |
| CRAT | carnitine O-acetyltransferase | **0,982** | 0,642341 | 24,039 | 20,049 |
| TUSC1 | tumor suppressor candidate 1 | **0,982** | 0,603211 | 16,119 | 13,153 |
| R3HDM1 | R3H domain containing 1 | **0,982** | 0,719813 | 10,192 | 9,641 |
| POLD3 | DNA polymerase delta 3, accessory subunit | **0,982** | 0,623392 | 15,122 | 17,495 |
| RASSF4 | Ras association domain family member 4 | **0,982** | 0,742515 | 6,536 | 6,066 |

| | | | | | |
|---|---|---|---|---|---|
| BIVM | basic, immunoglobulin-like variable motif containing | **0,982** | 0,788724 | 3,490 | 3,001 |
| AEBP1 | AE binding protein 1 | **0,982** | 0,578239 | 519,227 | 301,691 |
| TTC7B | tetratricopeptide repeat domain 7B | **0,982** | 0,774423 | 6,536 | 3,639 |
| TMED5 | transmembrane p24 trafficking protein 5 | **0,982** | 0,531988 | 73,724 | 56,316 |
| TRAM1L1 | translocation associated membrane protein 1-like 1 | **0,982** | 0,871984 | 0,831 | 1,341 |
| ZNF618 | zinc finger protein 618 | **0,982** | 0,852448 | 1,440 | 1,532 |
| MITF | melanogenesis associated transcription factor | **0,982** | 0,65571 | 8,309 | 12,323 |
| NR2C2AP | nuclear receptor 2C2 associated protein | **0,982** | 0,702839 | 7,035 | 7,023 |
| PLD2 | phospholipase D2 | **0,982** | 0,782042 | 4,653 | 3,192 |
| IDH2 | isocitrate dehydrogenase (NADP(+)) 2, mitochondrial | **0,981** | 0,624119 | 15,454 | 14,302 |
| ACOT8 | acyl-CoA thioesterase 8 | **0,981** | 0,645346 | 8,862 | 7,790 |
| PIK3R4 | phosphoinositide-3-kinase regulatory subunit 4 | **0,981** | 0,491528 | 26,643 | 26,498 |
| PSMA4 | proteasome subunit alpha 4 | **0,981** | 0,497323 | 84,858 | 81,409 |
| SCARNA15 | small Cajal body-specific RNA 15 | **0,981** | 0,900266 | 0,443 | 0,830 |
| VPS26B | VPS26, retromer complex component B | **0,981** | 0,611096 | 24,482 | 20,687 |
| AKAP9 | A-kinase anchoring protein 9 | **0,981** | 0,583459 | 96,656 | 91,944 |
| GABPB2 | GA binding protein transcription factor beta subunit 2 | **0,981** | 0,608145 | 18,999 | 24,455 |
| GNPDA2 | glucosamine-6-phosphate deaminase 2 | **0,981** | 0,540968 | 36,834 | 36,714 |
| USP11 | ubiquitin specific peptidase 11 | **0,981** | 0,488111 | 72,118 | 58,295 |
| LOX | lysyl oxidase | **0,981** | 0,576632 | 63,311 | 56,316 |
| TAF1B | TATA-box binding protein associated factor, RNA polymerase I subunit B | **0,981** | 0,619433 | 11,909 | 11,557 |
| SLC31A2 | solute carrier family 31 member 2 | **0,981** | 0,510605 | 55,279 | 47,057 |
| TAF2 | TATA-box binding protein associated factor 2 | **0,981** | 0,632259 | 22,765 | 22,475 |
| NDN | necdin, MAGE family member | **0,981** | 0,512093 | 38,164 | 39,970 |
| NUTM2A-AS1 | NUTM2A antisense RNA 1 | **0,981** | 0,667487 | 7,312 | 6,321 |
| ZNF783 | zinc finger family member 783 | **0,981** | 0,657346 | 9,638 | 8,811 |
| MMADHC | methylmalonic aciduria and homocystinuria, cblD type | **0,981** | 0,493313 | 106,681 | 111,035 |
| ZNF132 | zinc finger protein 132 | **0,981** | 0,815236 | 3,213 | 4,278 |
| CCDC85C | coiled-coil domain containing 85C | **0,981** | 0,620524 | 15,731 | 15,579 |
| RTFDC1 | replication termination factor 2 domain containing 1 | **0,981** | 0,466624 | 63,865 | 66,085 |
| KLF16 | Kruppel like factor 16 | **0,981** | 0,638116 | 24,926 | 20,815 |
| RPP25L | ribonuclease P/MRP subunit p25 like | **0,981** | 0,698594 | 10,247 | 12,004 |
| TMED10 | transmembrane p24 trafficking protein 10 | **0,981** | 0,463351 | 382,358 | 285,345 |

| | | | | | |
|---|---|---|---|---|---|
| MTMR8 | myotubularin related protein 8 | **0,981** | 0,916945 | 0,332 | 0,638 |
| TBX5-AS1 | TBX5 antisense RNA 1 | **0,981** | 0,873976 | 0,499 | 1,022 |
| IFT80 | intraflagellar transport 80 | **0,981** | 0,742196 | 7,478 | 6,768 |
| OARD1 | O-acyl-ADP-ribose deacylase 1 | **0,981** | 0,544907 | 18,611 | 24,965 |
| IFITM10 | interferon induced transmembrane protein 10 | **0,981** | 0,898416 | 0,997 | 1,277 |
| ZNF880 | zinc finger protein 880 | **0,981** | 0,631958 | 8,253 | 9,450 |
| PILRA | paired immunoglobin like type 2 receptor alpha | **0,981** | 0,648689 | 33,843 | 32,372 |
| SENP6 | SUMO1/sentrin specific peptidase 6 | **0,981** | 0,50608 | 54,282 | 51,080 |
| FAM175B | family with sequence similarity 175 member B | **0,981** | 0,582364 | 12,407 | 14,749 |
| MDK | midkine (neurite growth-promoting factor 2) | **0,981** | 0,854012 | 1,385 | 0,894 |
| METTL6 | methyltransferase like 6 | **0,981** | 0,625842 | 12,352 | 13,472 |
| AKAP8 | A-kinase anchoring protein 8 | **0,981** | 0,492866 | 20,328 | 20,687 |
| SLC39A6 | solute carrier family 39 member 6 | **0,981** | 0,455812 | 132,826 | 102,351 |
| ATF6 | activating transcription factor 6 | **0,981** | 0,527858 | 61,317 | 52,612 |
| TMEM134 | transmembrane protein 134 | **0,981** | 0,630183 | 9,582 | 10,982 |
| SDHA | succinate dehydrogenase complex flavoprotein subunit A | **0,981** | 0,470914 | 110,393 | 77,769 |
| WDR45B | WD repeat domain 45B | **0,981** | 0,455833 | 63,477 | 58,742 |
| TIAL1 | TIA1 cytotoxic granule associated RNA binding protein like 1 | **0,981** | 0,440008 | 45,309 | 46,419 |
| CYB5R4 | cytochrome b5 reductase 4 | **0,981** | 0,641272 | 8,530 | 9,131 |
| VAMP2 | vesicle associated membrane protein 2 | **0,981** | 0,53049 | 40,823 | 44,759 |
| RAB27B | RAB27B, member RAS oncogene family | **0,981** | 0,870966 | 1,772 | 0,638 |
| SPTSSB | serine palmitoyltransferase small subunit B | **0,981** | 0,858775 | 1,329 | 1,532 |
| MCM3AP | minichromosome maintenance complex component 3 associated protein | **0,981** | 0,548507 | 55,169 | 52,293 |
| HIST2H2BE | histone cluster 2 H2B family member e | **0,980** | 0,608724 | 52,011 | 59,253 |
| CD2AP | CD2 associated protein | **0,980** | 0,622375 | 23,652 | 16,984 |
| PDIA4 | protein disulfide isomerase family A member 4 | **0,980** | 0,486071 | 266,759 | 203,617 |
| GTF2H2 | general transcription factor IIH subunit 2 | **0,980** | 0,914566 | 0,554 | 0,000 |
| ZFHX3 | zinc finger homeobox 3 | **0,980** | 0,646722 | 43,647 | 45,653 |
| AAGAB | alpha- and gamma-adaptin binding protein | **0,980** | 0,476107 | 38,662 | 41,758 |
| PIM2 | Pim-2 proto-oncogene, serine/threonine kinase | **0,980** | 0,569896 | 54,227 | 39,651 |
| SAR1A | secretion associated Ras related GTPase 1A | **0,980** | 0,450834 | 95,160 | 96,349 |
| THAP2 | THAP domain containing 2 | **0,980** | 0,604086 | 9,472 | 9,769 |
| MAP4K5 | mitogen-activated protein kinase kinase kinase kinase 5 | **0,980** | 0,589137 | 42,650 | 38,438 |

| | | | | | |
|---|---|---|---|---|---|
| IQCE | IQ motif containing E | **0,980** | 0,59372 | 18,888 | 20,496 |
| MAFK | MAF bZIP transcription factor K | **0,980** | 0,464567 | 149,830 | 151,324 |
| MAPK9 | mitogen-activated protein kinase 9 | **0,980** | 0,525766 | 21,768 | 20,432 |
| BORCS7 | BLOC-1 related complex subunit 7 | **0,980** | 0,66155 | 14,512 | 13,792 |
| TOM1L2 | target of myb1 like 2 membrane trafficking protein | **0,980** | 0,523655 | 46,251 | 51,974 |
| PGD | phosphogluconate dehydrogenase | **0,980** | 0,589237 | 80,150 | 50,505 |
| BTF3L4 | basic transcription factor 3 like 4 | **0,980** | 0,510867 | 36,391 | 44,248 |
| MRPL40 | mitochondrial ribosomal protein L40 | **0,980** | 0,53837 | 22,378 | 28,413 |
| BCL9 | B-cell CLL/lymphoma 9 | **0,980** | 0,725487 | 7,367 | 9,003 |
| LEMD2 | LEM domain containing 2 | **0,980** | 0,45431 | 57,163 | 59,636 |
| PFDN1 | prefoldin subunit 1 | **0,980** | 0,502425 | 28,304 | 28,158 |
| ZNF207 | zinc finger protein 207 | **0,980** | 0,485128 | 85,301 | 75,151 |
| PPP1R12C | protein phosphatase 1 regulatory subunit 12C | **0,980** | 0,537985 | 62,480 | 69,724 |
| PPHLN1 | periphilin 1 | **0,980** | 0,526134 | 26,643 | 25,157 |
| PRKAA1 | protein kinase AMP-activated catalytic subunit alpha 1 | **0,980** | 0,533846 | 53,784 | 45,014 |
| LURAP1 | leucine rich adaptor protein 1 | **0,980** | 0,815554 | 2,105 | 2,554 |
| HTT | huntingtin | **0,980** | 0,611116 | 45,198 | 47,760 |
| KAT14 | lysine acetyltransferase 14 | **0,980** | 0,757219 | 7,367 | 7,023 |
| GPN2 | GPN-loop GTPase 2 | **0,980** | 0,498018 | 19,110 | 19,027 |
| RAB8A | RAB8A, member RAS oncogene family | **0,980** | 0,455966 | 33,566 | 31,159 |
| CAMK1 | calcium/calmodulin dependent protein kinase I | **0,980** | 0,736298 | 4,764 | 3,895 |
| PNPLA2 | patatin like phospholipase domain containing 2 | **0,980** | 0,569238 | 183,729 | 164,350 |
| ANKRD36C | ankyrin repeat domain 36C | **0,980** | 0,807689 | 2,770 | 3,448 |
| PARP11 | poly(ADP-ribose) polymerase family member 11 | **0,980** | 0,653212 | 8,309 | 8,875 |
| FAM174B | family with sequence similarity 174 member B | **0,980** | 0,676166 | 6,425 | 7,343 |
| SPOUT1 | SPOUT domain containing methyltransferase 1 | **0,980** | 0,56011 | 25,479 | 29,818 |
| P4HTM | prolyl 4-hydroxylase, transmembrane | **0,980** | 0,58542 | 25,535 | 14,749 |
| RANBP6 | RAN binding protein 6 | **0,980** | 0,599372 | 20,550 | 19,219 |
| EDEM3 | ER degradation enhancing alpha-mannosidase like protein 3 | **0,980** | 0,521009 | 108,509 | 90,922 |
| CINP | cyclin dependent kinase 2 interacting protein | **0,980** | 0,513866 | 17,171 | 16,218 |
| RPL35 | ribosomal protein L35 | **0,980** | 0,557237 | 139,639 | 156,177 |
| ORC2 | origin recognition complex subunit 2 | **0,980** | 0,603834 | 16,672 | 17,303 |
| SDHD | succinate dehydrogenase complex subunit D | **0,980** | 0,574567 | 23,208 | 23,816 |

| | | | | | |
|---|---|---|---|---|---|
| INTS2 | integrator complex subunit 2 | **0,980** | 0,60142 | 11,189 | 13,855 |
| TMEM185B | transmembrane protein 185B | **0,980** | 0,477367 | 28,692 | 28,541 |
| PTDSS1 | phosphatidylserine synthase 1 | **0,980** | 0,479849 | 42,706 | 40,417 |
| CAPN10 | calpain 10 | **0,980** | 0,601274 | 13,183 | 16,090 |
| RPS21 | ribosomal protein S21 | **0,980** | 0,611085 | 102,693 | 103,884 |
| ZNF71 | zinc finger protein 71 | **0,980** | 0,723511 | 5,927 | 4,214 |
| POMGNT2 | protein O-linked mannose N-acetylglucosaminyltransferase 2 (beta 1,4-) | **0,980** | 0,612044 | 11,244 | 8,428 |
| LRRC23 | leucine rich repeat containing 23 | **0,980** | 0,655082 | 6,204 | 9,450 |
| ZDHHC3 | zinc finger DHHC-type containing 3 | **0,979** | 0,45346 | 24,704 | 21,709 |
| DDAH1 | dimethylarginine dimethylaminohydrolase 1 | **0,979** | 0,557765 | 27,030 | 24,391 |
| ZNF268 | zinc finger protein 268 | **0,979** | 0,616888 | 17,448 | 14,877 |
| GLDN | gliomedin | **0,979** | 0,583153 | 40,656 | 11,110 |
| PGBD1 | piggyBac transposable element derived 1 | **0,979** | 0,746256 | 4,376 | 2,937 |
| TAPT1 | transmembrane anterior posterior transformation 1 | **0,979** | 0,472723 | 21,491 | 23,178 |
| RGCC | regulator of cell cycle | **0,979** | 0,423957 | 73,447 | 69,022 |
| EEF1A1 | eukaryotic translation elongation factor 1 alpha 1 | **0,979** | 0,476332 | 2349,041 | 1853,498 |
| PGM2 | phosphoglucomutase 2 | **0,979** | 0,506389 | 25,535 | 29,307 |
| NSMCE2 | NSE2/MMS21 homolog, SMC5-SMC6 complex SUMO ligase | **0,979** | 0,636203 | 12,463 | 11,493 |
| GOLM1 | golgi membrane protein 1 | **0,979** | 0,500039 | 196,635 | 139,704 |
| TRAPPC13 | trafficking protein particle complex 13 | **0,979** | 0,606119 | 12,297 | 12,898 |
| CHAC1 | ChaC glutathione specific gamma-glutamylcyclotransferase 1 | **0,979** | 0,517922 | 35,616 | 31,095 |
| CEP85L | centrosomal protein 85 like | **0,979** | 0,659418 | 10,580 | 9,897 |
| NIF3L1 | NGG1 interacting factor 3 like 1 | **0,979** | 0,515385 | 15,842 | 16,793 |
| RNF41 | ring finger protein 41 | **0,979** | 0,47938 | 33,511 | 31,797 |
| PLEKHJ1 | pleckstrin homology domain containing J1 | **0,979** | 0,681056 | 10,081 | 7,917 |
| TRRAP | transformation/transcription domain associated protein | **0,979** | 0,624555 | 45,586 | 46,483 |
| STX5 | syntaxin 5 | **0,979** | 0,42724 | 46,915 | 44,056 |
| UTP4 | UTP4, small subunit processome component | **0,979** | 0,701953 | 7,090 | 7,279 |
| METTL22 | methyltransferase like 22 | **0,979** | 0,563551 | 9,693 | 11,940 |
| CETN3 | centrin 3 | **0,979** | 0,668012 | 9,139 | 9,450 |
| CDC37L1-AS1 | CDC37L1 antisense RNA 1 (head to head) | **0,979** | 0,911245 | 0,277 | 0,255 |
| ADAT3 | adenosine deaminase, tRNA specific 3 | **0,979** | 0,851222 | 0,831 | 0,702 |
| COL5A2 | collagen type V alpha 2 chain | **0,979** | 0,498462 | 166,669 | 108,992 |

| | | | | | |
|---|---|---|---|---|---|
| RAB30-AS1 | RAB30 antisense RNA 1 (head to head) | **0,979** | 0,658708 | 4,265 | 6,002 |
| RPL36 | ribosomal protein L36 | **0,979** | 0,522251 | 120,529 | 144,812 |
| RTL6 | retrotransposon Gag like 6 | **0,979** | 0,48407 | 35,117 | 37,608 |
| TUBA4A | tubulin alpha 4a | **0,979** | 0,595321 | 7,865 | 10,727 |
| PQBP1 | polyglutamine binding protein 1 | **0,979** | 0,51709 | 20,605 | 19,091 |
| SMIM14 | small integral membrane protein 14 | **0,979** | 0,467414 | 186,776 | 134,149 |
| NDUFA5 | NADH:ubiquinone oxidoreductase subunit A5 | **0,979** | 0,514318 | 26,864 | 27,136 |
| LINC01768 | long intergenic non-protein coding RNA 1768 | **0,979** | 0,897883 | 0,166 | 0,447 |
| BATF3 | basic leucine zipper ATF-like transcription factor 3 | **0,979** | 0,867329 | 0,942 | 1,596 |
| KDM6B | lysine demethylase 6B | **0,979** | 0,624152 | 36,945 | 49,867 |
| GTF2IP13 | general transcription factor IIi pseudogene 13 | **0,979** | 0,742597 | 3,268 | 4,980 |
| SLC4A2 | solute carrier family 4 member 2 | **0,979** | 0,458143 | 113,771 | 79,685 |
| CADPS2 | calcium dependent secretion activator 2 | **0,979** | 0,675181 | 9,416 | 9,322 |
| RPS27AP16 | ribosomal protein S27a pseudogene 16 | **0,979** | 0,832576 | 0,831 | 1,469 |
| AP3S2 | adaptor related protein complex 3 sigma 2 subunit | **0,979** | 0,619558 | 9,693 | 11,174 |
| HNRNPA2B1 | heterogeneous nuclear ribonucleoprotein A2/B1 | **0,979** | 0,383917 | 295,617 | 246,014 |
| SARM1 | sterile alpha and TIR motif containing 1 | **0,979** | 0,650848 | 10,690 | 8,556 |
| HNRNPK | heterogeneous nuclear ribonucleoprotein K | **0,979** | 0,399442 | 323,035 | 334,254 |
| DCTD | dCMP deaminase | **0,979** | 0,399521 | 67,188 | 67,489 |
| RNF113A | ring finger protein 113A | **0,979** | 0,586818 | 14,180 | 13,919 |
| AFG3L2 | AFG3 like matrix AAA peptidase subunit 2 | **0,979** | 0,435363 | 42,983 | 45,078 |
| SORBS3 | sorbin and SH3 domain containing 3 | **0,979** | 0,538307 | 77,380 | 68,639 |
| CASP6 | caspase 6 | **0,979** | 0,790409 | 3,157 | 2,746 |
| OXSM | 3-oxoacyl-ACP synthase, mitochondrial | **0,979** | 0,559691 | 8,253 | 9,641 |
| RILPL1 | Rab interacting lysosomal protein like 1 | **0,979** | 0,630116 | 10,303 | 6,130 |
| CEBPZ | CCAAT/enhancer binding protein zeta | **0,979** | 0,441292 | 38,607 | 40,289 |
| CDK12 | cyclin dependent kinase 12 | **0,978** | 0,627682 | 53,452 | 49,228 |
| PACRGL | PARK2 coregulated like | **0,978** | 0,571679 | 11,743 | 10,535 |
| DBR1 | debranching RNA lariats 1 | **0,978** | 0,637597 | 11,853 | 11,046 |
| PIDD1 | p53-induced death domain protein 1 | **0,978** | 0,658201 | 6,259 | 7,917 |
| MEST | mesoderm specific transcript | **0,978** | 0,845802 | 1,440 | 0,894 |
| SCLY | selenocysteine lyase | **0,978** | 0,829312 | 1,329 | 1,469 |
| LUC7L | LUC7 like | **0,978** | 0,475739 | 27,640 | 29,243 |

| | | | | | |
|---|---|---|---|---|---|
| PITPNA-AS1 | PITPNA antisense RNA 1 | **0,978** | 0,753167 | 3,268 | 3,192 |
| SH2B1 | SH2B adaptor protein 1 | **0,978** | 0,471101 | 21,159 | 17,176 |
| SCCPDH | saccharopine dehydrogenase (putative) | **0,978** | 0,583177 | 24,815 | 19,793 |
| PQLC1 | PQ loop repeat containing 1 | **0,978** | 0,570649 | 29,578 | 27,839 |
| KLHL22 | kelch like family member 22 | **0,978** | 0,657679 | 8,530 | 7,726 |
| USP25 | ubiquitin specific peptidase 25 | **0,978** | 0,493565 | 23,762 | 23,880 |
| KIF1C | kinesin family member 1C | **0,978** | 0,507512 | 79,928 | 53,315 |
| HNRNPAB | heterogeneous nuclear ribonucleoprotein A/B | **0,978** | 0,429105 | 77,269 | 78,216 |
| DTX3 | deltex E3 ubiquitin ligase 3 | **0,978** | 0,589963 | 12,020 | 11,748 |
| UBP1 | upstream binding protein 1 (LBP-1a) | **0,978** | 0,492755 | 53,507 | 44,695 |
| TTLL1 | tubulin tyrosine ligase like 1 | **0,978** | 0,770679 | 3,102 | 4,533 |
| SNUPN | snurportin 1 | **0,978** | 0,650694 | 6,868 | 5,491 |
| MAPKAPK5 | mitogen-activated protein kinase-activated protein kinase 5 | **0,978** | 0,58497 | 18,943 | 18,325 |
| ZIC1 | Zic family member 1 | **0,978** | 0,837634 | 0,775 | 0,958 |
| LMBRD2 | LMBR1 domain containing 2 | **0,978** | 0,519513 | 27,695 | 29,499 |
| GMDS-AS1 | GMDS antisense RNA 1 (head to head) | **0,978** | 0,714774 | 3,711 | 3,576 |
| AEN | apoptosis enhancing nuclease | **0,978** | 0,441297 | 41,709 | 63,594 |
| KCNE4 | potassium voltage-gated channel subfamily E regulatory subunit 4 | **0,978** | 0,798454 | 3,434 | 2,682 |
| RNF216 | ring finger protein 216 | **0,978** | 0,388974 | 48,300 | 42,779 |
| SDHC | succinate dehydrogenase complex subunit C | **0,978** | 0,408139 | 34,397 | 30,712 |
| ELP3 | elongator acetyltransferase complex subunit 3 | **0,978** | 0,513817 | 20,384 | 22,922 |
| PSMD6 | proteasome 26S subunit, non-ATPase 6 | **0,978** | 0,454225 | 42,484 | 43,035 |
| EEF1A1P13 | eukaryotic translation elongation factor 1 alpha 1 pseudogene 13 | **0,978** | 0,766573 | 2,160 | 1,596 |
| TBPL1 | TATA-box binding protein like 1 | **0,978** | 0,500066 | 13,127 | 12,578 |
| TLR3 | toll like receptor 3 | **0,978** | 0,657678 | 8,198 | 15,388 |
| FTCDNL1 | formiminotransferase cyclodeaminase N-terminal like | **0,978** | 0,876289 | 1,662 | 0,447 |
| MAP4 | microtubule associated protein 4 | **0,978** | 0,405195 | 248,979 | 195,444 |
| CSDE1 | cold shock domain containing E1 | **0,978** | 0,346899 | 352,725 | 300,286 |
| MRRF | mitochondrial ribosome recycling factor | **0,978** | 0,580225 | 12,130 | 14,558 |
| NDUFS2 | NADH:ubiquinone oxidoreductase core subunit S2 | **0,978** | 0,406227 | 39,050 | 42,652 |
| PRKCE | protein kinase C epsilon | **0,978** | 0,644419 | 6,647 | 8,045 |
| RFWD2 | ring finger and WD repeat domain 2 | **0,978** | 0,478569 | 21,602 | 18,325 |
| APOBEC3C | apolipoprotein B mRNA editing enzyme catalytic subunit 3C | **0,978** | 0,725283 | 4,431 | 2,490 |

| | | | | | |
|---|---|---|---|---|---|
| INHBE | inhibin beta E subunit | **0,977** | 0,845511 | 1,717 | 2,746 |
| KNTC1 | kinetochore associated 1 | **0,977** | 0,731591 | 5,705 | 7,598 |
| RPS19 | ribosomal protein S19 | **0,977** | 0,53947 | 248,093 | 266,509 |
| NDUFB8 | NADH:ubiquinone oxidoreductase subunit B8 | **0,977** | 0,635474 | 10,247 | 9,131 |
| ZNF576 | zinc finger protein 576 | **0,977** | 0,572863 | 9,195 | 9,386 |
| DZANK1 | double zinc ribbon and ankyrin repeat domains 1 | **0,977** | 0,763639 | 3,711 | 4,150 |
| PCID2 | PCI domain containing 2 | **0,977** | 0,462859 | 24,095 | 31,670 |
| UBXN11 | UBX domain protein 11 | **0,977** | 0,604252 | 13,681 | 18,325 |
| TMEM30A | transmembrane protein 30A | **0,977** | 0,400691 | 183,120 | 165,690 |
| PTK2B | protein tyrosine kinase 2 beta | **0,977** | 0,56598 | 8,585 | 9,961 |
| C20orf96 | chromosome 20 open reading frame 96 | **0,977** | 0,805353 | 1,662 | 1,341 |
| ATL2 | atlastin GTPase 2 | **0,977** | 0,551792 | 26,698 | 26,625 |
| RANBP17 | RAN binding protein 17 | **0,977** | 0,852458 | 1,163 | 1,532 |
| NSUN6 | NOP2/Sun RNA methyltransferase family member 6 | **0,977** | 0,671662 | 5,982 | 4,853 |
| NOL11 | nucleolar protein 11 | **0,977** | 0,463229 | 25,424 | 29,882 |
| CIZ1 | CDKN1A interacting zinc finger protein 1 | **0,977** | 0,408444 | 65,194 | 62,190 |
| UNC119 | unc-119 lipid binding chaperone | **0,977** | 0,567322 | 12,518 | 10,216 |
| PRKCD | protein kinase C delta | **0,977** | 0,525645 | 25,923 | 15,260 |
| ZNF133 | zinc finger protein 133 | **0,977** | 0,556294 | 10,358 | 11,493 |
| RPS6KL1 | ribosomal protein S6 kinase like 1 | **0,977** | 0,905806 | 1,052 | 0,192 |
| RNF40 | ring finger protein 40 | **0,977** | 0,454931 | 68,351 | 83,643 |
| CD59 | CD59 molecule (CD59 blood group) | **0,977** | 0,324296 | 1132,895 | 795,123 |
| BTG3 | BTG anti-proliferation factor 3 | **0,977** | 0,460256 | 44,534 | 42,716 |
| EIF5B | eukaryotic translation initiation factor 5B | **0,977** | 0,539597 | 95,326 | 101,394 |
| ATG3 | autophagy related 3 | **0,977** | 0,462578 | 43,703 | 51,208 |
| ZNF717 | zinc finger protein 717 | **0,977** | 0,716186 | 2,991 | 4,086 |
| FBXO7 | F-box protein 7 | **0,977** | 0,40356 | 46,915 | 42,141 |
| ANKIB1 | ankyrin repeat and IBR domain containing 1 | **0,977** | 0,477991 | 40,989 | 34,862 |
| HSPA2 | heat shock protein family A (Hsp70) member 2 | **0,977** | 0,52807 | 39,105 | 46,100 |
| MMP17 | matrix metallopeptidase 17 | **0,977** | 0,903832 | 0,443 | 0,383 |
| PI4K2B | phosphatidylinositol 4-kinase type 2 beta | **0,977** | 0,663338 | 6,591 | 5,108 |
| EXOG | exo/endonuclease G | **0,977** | 0,710297 | 5,428 | 4,469 |
| RPL11 | ribosomal protein L11 | **0,977** | 0,490583 | 303,427 | 307,884 |

| | | | | | |
|---|---|---|---|---|---|
| NUP88 | nucleoporin 88 | **0,977** | 0,441886 | 26,975 | 29,179 |
| TRMT6 | tRNA methyltransferase 6 | **0,977** | 0,456019 | 17,393 | 24,391 |
| MAFF | MAF bZIP transcription factor F | **0,977** | 0,330762 | 379,810 | 418,025 |
| MEAF6 | MYST/Esa1 associated factor 6 | **0,977** | 0,459583 | 26,366 | 27,711 |
| RECQL4 | RecQ like helicase 4 | **0,977** | 0,697318 | 4,874 | 3,895 |
| STX8 | syntaxin 8 | **0,977** | 0,574383 | 9,084 | 8,300 |
| EIF1AD | eukaryotic translation initiation factor 1A domain containing | **0,977** | 0,510953 | 17,946 | 16,409 |
| NTMT1 | N-terminal Xaa-Pro-Lys N-methyltransferase 1 | **0,977** | 0,516701 | 19,885 | 18,070 |
| TDP1 | tyrosyl-DNA phosphodiesterase 1 | **0,977** | 0,702033 | 4,043 | 3,320 |
| DENND5A | DENN domain containing 5A | **0,977** | 0,488802 | 177,913 | 146,408 |
| PHKA2 | phosphorylase kinase regulatory subunit alpha 2 | **0,977** | 0,580342 | 8,973 | 11,046 |
| AP4S1 | adaptor related protein complex 4 sigma 1 subunit | **0,977** | 0,6806 | 4,043 | 5,874 |
| PEX7 | peroxisomal biogenesis factor 7 | **0,977** | 0,611678 | 4,320 | 7,023 |
| RALGDS | ral guanine nucleotide dissociation stimulator | **0,977** | 0,795999 | 1,772 | 2,107 |
| MAP3K13 | mitogen-activated protein kinase kinase kinase 13 | **0,977** | 0,476171 | 13,349 | 13,472 |
| XXYLT1 | xyloside xylosyltransferase 1 | **0,977** | 0,639678 | 7,478 | 7,726 |
| BOD1 | biorientation of chromosomes in cell division 1 | **0,977** | 0,442892 | 23,984 | 29,754 |
| TOMM70 | translocase of outer mitochondrial membrane 70 | **0,977** | 0,400224 | 77,657 | 87,666 |
| ACCS | 1-aminocyclopropane-1-carboxylate synthase homolog (inactive) | **0,977** | 0,796115 | 1,717 | 1,022 |
| OAZ1 | ornithine decarboxylase antizyme 1 | **0,977** | 0,433174 | 428,498 | 338,915 |
| OPN3 | opsin 3 | **0,976** | 0,74105 | 2,936 | 3,065 |
| DHX32 | DEAH-box helicase 32 (putative) | **0,976** | 0,437444 | 18,168 | 15,516 |
| SRPRA | SRP receptor alpha subunit | **0,976** | 0,417775 | 275,234 | 288,921 |
| LRPAP1 | LDL receptor related protein associated protein 1 | **0,976** | 0,433622 | 125,293 | 101,011 |
| NAGLU | N-acetyl-alpha-glucosaminidase | **0,976** | 0,513159 | 34,453 | 22,411 |
| CCDC91 | coiled-coil domain containing 91 | **0,976** | 0,595482 | 18,722 | 16,856 |
| IDH3B | isocitrate dehydrogenase 3 (NAD(+)) beta | **0,976** | 0,422167 | 26,089 | 25,029 |
| LETMD1 | LETM1 domain containing 1 | **0,976** | 0,47926 | 15,509 | 16,537 |
| ATXN2L | ataxin 2 like | **0,976** | 0,360698 | 86,852 | 90,603 |
| ASB1 | ankyrin repeat and SOCS box containing 1 | **0,976** | 0,37177 | 42,540 | 41,247 |
| PPIL4 | peptidylprolyl isomerase like 4 | **0,976** | 0,496109 | 32,126 | 35,309 |
| ANGEL1 | angel homolog 1 | **0,976** | 0,640653 | 8,309 | 7,534 |
| LMNA | lamin A/C | **0,976** | 0,432555 | 314,173 | 218,303 |

| | | | | | |
|---|---|---|---|---|---|
| LZTFL1 | leucine zipper transcription factor like 1 | **0,976** | 0,505109 | 30,797 | 23,369 |
| ECI1 | enoyl-CoA delta isomerase 1 | **0,976** | 0,588357 | 20,051 | 19,027 |
| EMC1 | ER membrane protein complex subunit 1 | **0,976** | 0,339093 | 128,173 | 120,038 |
| POLG2 | DNA polymerase gamma 2, accessory subunit | **0,976** | 0,663249 | 3,600 | 5,427 |
| PSMB2 | proteasome subunit beta 2 | **0,976** | 0,445548 | 62,979 | 65,318 |
| CHCHD1 | coiled-coil-helix-coiled-coil-helix domain containing 1 | **0,976** | 0,548363 | 13,404 | 12,578 |
| ZCCHC4 | zinc finger CCHC-type containing 4 | **0,976** | 0,655402 | 6,425 | 9,067 |
| COG3 | component of oligomeric golgi complex 3 | **0,976** | 0,472423 | 39,549 | 36,522 |
| TRA2A | transformer 2 alpha homolog | **0,976** | 0,429778 | 23,430 | 26,945 |
| RPL27 | ribosomal protein L27 | **0,976** | 0,499542 | 189,988 | 204,830 |
| DGKI | diacylglycerol kinase iota | **0,976** | 0,778454 | 3,933 | 2,171 |
| CLASP2 | cytoplasmic linker associated protein 2 | **0,976** | 0,504087 | 21,381 | 21,007 |
| CYB5D2 | cytochrome b5 domain containing 2 | **0,976** | 0,433847 | 29,024 | 23,624 |
| PXMP4 | peroxisomal membrane protein 4 | **0,976** | 0,698756 | 4,764 | 3,320 |
| SLC35E2 | solute carrier family 35 member E2 | **0,976** | 0,612223 | 10,469 | 5,044 |
| PARP4 | poly(ADP-ribose) polymerase family member 4 | **0,976** | 0,471306 | 32,126 | 21,901 |
| CHERP | calcium homeostasis endoplasmic reticulum protein | **0,976** | 0,427066 | 36,668 | 41,694 |
| TSTD2 | thiosulfate sulfurtransferase like domain containing 2 | **0,976** | 0,503151 | 20,827 | 23,433 |
| KIAA0930 | KIAA0930 | **0,976** | 0,469267 | 30,963 | 23,944 |
| SFTPD | surfactant protein D | **0,976** | 0,913581 | 0,388 | 0,192 |
| ZNF282 | zinc finger protein 282 | **0,976** | 0,440939 | 26,476 | 28,924 |
| POFUT1 | protein O-fucosyltransferase 1 | **0,976** | 0,358619 | 68,407 | 63,148 |
| MZT1 | mitotic spindle organizing protein 1 | **0,976** | 0,421 | 28,526 | 23,561 |
| LZTS2 | leucine zipper tumor suppressor 2 | **0,976** | 0,493573 | 20,162 | 14,941 |
| TAX1BP3 | Tax1 binding protein 3 | **0,976** | 0,652182 | 6,868 | 6,513 |
| ARHGAP4 | Rho GTPase activating protein 4 | **0,976** | 0,762772 | 1,274 | 1,660 |
| FIP1L1 | factor interacting with PAPOLA and CPSF1 | **0,976** | 0,515941 | 18,556 | 23,433 |
| RHNO1 | RAD9-HUS1-RAD1 interacting nuclear orphan 1 | **0,976** | 0,61825 | 10,081 | 7,151 |
| RBBP7 | RB binding protein 7, chromatin remodeling factor | **0,976** | 0,402258 | 50,959 | 41,183 |
| EIF3E | eukaryotic translation initiation factor 3 subunit E | **0,976** | 0,349912 | 111,390 | 116,271 |
| COMMD2 | COMM domain containing 2 | **0,976** | 0,487051 | 15,398 | 15,452 |
| LSP1 | lymphocyte-specific protein 1 | **0,976** | 0,768254 | 3,767 | 2,746 |
| ABAT | 4-aminobutyrate aminotransferase | **0,976** | 0,647893 | 9,250 | 7,343 |

| | | | | | |
|---|---|---|---|---|---|
| SLC35D1 | solute carrier family 35 member D1 | **0,976** | 0,481506 | 28,581 | 26,434 |
| RNF214 | ring finger protein 214 | **0,976** | 0,53545 | 12,020 | 10,918 |
| STXBP5-AS1 | STXBP5 antisense RNA 1 | **0,976** | 0,65349 | 5,151 | 5,172 |
| QRICH1 | glutamine rich 1 | **0,976** | 0,341421 | 57,052 | 52,485 |
| STRAP | serine/threonine kinase receptor associated protein | **0,976** | 0,374979 | 95,493 | 95,072 |
| TRMT10A | tRNA methyltransferase 10A | **0,976** | 0,640705 | 5,207 | 6,193 |
| CA5B | carbonic anhydrase 5B | **0,976** | 0,516357 | 15,398 | 14,622 |
| PELI2 | pellino E3 ubiquitin protein ligase family member 2 | **0,976** | 0,665906 | 3,656 | 5,108 |
| NDUFAF7 | NADH:ubiquinone oxidoreductase complex assembly factor 7 | **0,976** | 0,590816 | 8,752 | 8,300 |
| COX11 | COX11, cytochrome c oxidase copper chaperone | **0,976** | 0,493004 | 19,608 | 19,538 |
| SNX11 | sorting nexin 11 | **0,976** | 0,554071 | 12,795 | 14,111 |
| CCDC159 | coiled-coil domain containing 159 | **0,976** | 0,591066 | 6,259 | 5,619 |
| GLI3 | GLI family zinc finger 3 | **0,976** | 0,580867 | 23,375 | 19,921 |
| CCND1 | cyclin D1 | **0,976** | 0,416493 | 400,859 | 324,102 |
| PIR | pirin | **0,976** | 0,553776 | 13,626 | 11,046 |
| EGFL7 | EGF like domain multiple 7 | **0,976** | 0,807349 | 1,939 | 1,852 |
| SDR39U1 | short chain dehydrogenase/reductase family 39U member 1 | **0,976** | 0,457719 | 19,054 | 17,239 |
| BIN3 | bridging integrator 3 | **0,976** | 0,399722 | 23,430 | 26,689 |
| DUSP12 | dual specificity phosphatase 12 | **0,976** | 0,49184 | 17,337 | 19,283 |
| MRPL32 | mitochondrial ribosomal protein L32 | **0,976** | 0,461761 | 26,476 | 33,393 |
| ZNF497 | zinc finger protein 497 | **0,976** | 0,832917 | 0,775 | 0,702 |
| DVL3 | dishevelled segment polarity protein 3 | **0,976** | 0,396781 | 47,137 | 41,949 |
| POLI | DNA polymerase iota | **0,975** | 0,530531 | 14,291 | 14,941 |
| SMNDC1 | survival motor neuron domain containing 1 | **0,975** | 0,429764 | 19,331 | 20,177 |
| AC008560.1 | Uncharacterized protein FLJ26957 | **0,975** | 0,823402 | 1,163 | 1,469 |
| FRK | fyn related Src family tyrosine kinase | **0,975** | 0,695912 | 8,696 | 5,810 |
| C15orf57 | chromosome 15 open reading frame 57 | **0,975** | 0,512078 | 12,463 | 15,835 |
| NRK | Nik related kinase | **0,975** | 0,637513 | 12,629 | 10,024 |
| DCTN1 | dynactin subunit 1 | **0,975** | 0,470086 | 84,193 | 68,319 |
| NFRKB | nuclear factor related to kappaB binding protein | **0,975** | 0,52355 | 15,011 | 16,026 |
| RPL31 | ribosomal protein L31 | **0,975** | 0,473154 | 222,890 | 222,389 |
| YLPM1 | YLP motif containing 1 | **0,975** | 0,443373 | 50,737 | 50,952 |
| MYO9B | myosin IXB | **0,975** | 0,447307 | 102,472 | 99,861 |

| | | | | | |
|---|---|---|---|---|---|
| LCE3E | late cornified envelope 3E | **0,975** | 0,775649 | 3,767 | 6,896 |
| OSBPL7 | oxysterol binding protein like 7 | **0,975** | 0,838889 | 1,662 | 1,724 |
| FAM200B | family with sequence similarity 200 member B | **0,975** | 0,584098 | 6,647 | 10,088 |
| TFCP2 | transcription factor CP2 | **0,975** | 0,436274 | 20,328 | 20,113 |
| DHFR | dihydrofolate reductase | **0,975** | 0,738308 | 4,819 | 3,895 |
| ZSCAN5A | zinc finger and SCAN domain containing 5A | **0,975** | 0,65157 | 3,157 | 3,639 |
| CAMK2G | calcium/calmodulin dependent protein kinase II gamma | **0,975** | 0,594711 | 14,678 | 11,174 |
| TMEM56 | transmembrane protein 56 | **0,975** | 0,856184 | 1,052 | 0,511 |
| KIAA0895L | KIAA0895 like | **0,975** | 0,718251 | 5,262 | 4,853 |
| MTRR | 5-methyltetrahydrofolate-homocysteine methyltransferase reductase | **0,975** | 0,447192 | 24,704 | 28,988 |
| VIPAS39 | VPS33B interacting protein, apical-basolateral polarity regulator, spe-39 homolog | **0,975** | 0,393911 | 20,661 | 18,197 |
| ZXDB | zinc finger, X-linked, duplicated B | **0,975** | 0,584075 | 15,232 | 9,131 |
| TRMT2A | tRNA methyltransferase 2 homolog A | **0,975** | 0,45065 | 16,839 | 18,644 |
| CYB5R3 | cytochrome b5 reductase 3 | **0,975** | 0,325899 | 423,181 | 372,947 |
| NOA1 | nitric oxide associated 1 | **0,975** | 0,461038 | 16,839 | 15,579 |
| KIAA1191 | KIAA1191 | **0,975** | 0,315858 | 72,617 | 57,656 |
| CBY1 | chibby family member 1, beta catenin antagonist | **0,975** | 0,570056 | 9,749 | 8,811 |
| CA11 | carbonic anhydrase 11 | **0,975** | 0,803115 | 0,997 | 0,958 |
| ALDH3B1 | aldehyde dehydrogenase 3 family member B1 | **0,975** | 0,517854 | 25,590 | 13,153 |
| ANAPC16 | anaphase promoting complex subunit 16 | **0,975** | 0,437153 | 58,935 | 56,954 |
| PDE4DIP | phosphodiesterase 4D interacting protein | **0,975** | 0,363103 | 67,299 | 62,956 |
| INTS14 | integrator complex subunit 14 | **0,975** | 0,426277 | 23,042 | 25,923 |
| ARF5 | ADP ribosylation factor 5 | **0,975** | 0,463464 | 25,812 | 21,517 |
| SEC11A | SEC11 homolog A, signal peptidase complex subunit | **0,975** | 0,320712 | 88,735 | 78,025 |
| PTGES3 | prostaglandin E synthase 3 | **0,975** | 0,309396 | 213,197 | 199,786 |
| CHCHD3 | coiled-coil-helix-coiled-coil-helix domain containing 3 | **0,975** | 0,381016 | 22,211 | 21,134 |
| PSMC3IP | PSMC3 interacting protein | **0,975** | 0,661093 | 9,084 | 5,172 |
| FAM192A | family with sequence similarity 192 member A | **0,975** | 0,367488 | 33,899 | 32,116 |
| P2RY2 | purinergic receptor P2Y2 | **0,975** | 0,735793 | 2,216 | 2,043 |
| NISCH | nischarin | **0,975** | 0,394056 | 36,945 | 42,141 |
| PCDHB10 | protocadherin beta 10 | **0,975** | 0,732357 | 3,213 | 1,341 |
| SUMO1 | small ubiquitin-like modifier 1 | **0,975** | 0,359431 | 53,064 | 50,250 |
| RNASEH1 | ribonuclease H1 | **0,975** | 0,547824 | 11,189 | 11,046 |

| | | | | | |
|---|---|---|---|---|---|
| SPOP | speckle type BTB/POZ protein | **0,975** | 0,418297 | 49,685 | 52,548 |
| ZNF813 | zinc finger protein 813 | **0,975** | 0,683369 | 4,819 | 3,895 |
| KDM5B | lysine demethylase 5B | **0,975** | 0,441207 | 44,755 | 47,057 |
| TNFSF15 | TNF superfamily member 15 | **0,975** | 0,837558 | 0,665 | 0,575 |
| DDRGK1 | DDRGK domain containing 1 | **0,975** | 0,472673 | 49,353 | 45,525 |
| ZC3H4 | zinc finger CCCH-type containing 4 | **0,975** | 0,458352 | 30,354 | 31,478 |
| CTAGE5 | CTAGE family member 5, ER export factor | **0,975** | 0,416466 | 17,282 | 17,750 |
| PMPCB | peptidase, mitochondrial processing beta subunit | **0,975** | 0,390438 | 39,826 | 42,460 |
| VBP1 | VHL binding protein 1 | **0,974** | 0,457273 | 31,406 | 29,243 |
| CALM3 | calmodulin 3 | **0,974** | 0,357493 | 142,685 | 116,654 |
| SLTM | SAFB like transcription modulator | **0,974** | 0,334361 | 85,135 | 86,389 |
| TRMT10C | tRNA methyltransferase 10C, mitochondrial RNase P subunit | **0,974** | 0,394745 | 18,390 | 18,006 |
| DNAJC9-AS1 | DNAJC9 antisense RNA 1 | **0,974** | 0,818038 | 0,831 | 0,766 |
| MYEOV | myeloma overexpressed | **0,974** | 0,653395 | 2,936 | 5,427 |
| HDDC2 | HD domain containing 2 | **0,974** | 0,405533 | 31,074 | 37,097 |
| CHRD | chordin | **0,974** | 0,749056 | 3,434 | 1,596 |
| RASAL2-AS1 | RASAL2 antisense RNA 1 | **0,974** | 0,761774 | 2,382 | 1,405 |
| ITPKA | inositol-trisphosphate 3-kinase A | **0,974** | 0,846895 | 0,775 | 0,511 |
| MARCKS | myristoylated alanine rich protein kinase C substrate | **0,974** | 0,370261 | 317,275 | 218,111 |
| TMEM110 | transmembrane protein 110 | **0,974** | 0,789745 | 1,883 | 1,596 |
| DHPS | deoxyhypusine synthase | **0,974** | 0,349257 | 24,704 | 25,476 |
| SGTA | small glutamine rich tetratricopeptide repeat containing alpha | **0,974** | 0,386458 | 47,026 | 48,143 |
| ESYT2 | extended synaptotagmin 2 | **0,974** | 0,352731 | 100,533 | 81,409 |
| FAM177A1 | family with sequence similarity 177 member A1 | **0,974** | 0,309435 | 69,182 | 64,935 |
| KBTBD7 | kelch repeat and BTB domain containing 7 | **0,974** | 0,522589 | 14,124 | 12,515 |
| AGGF1 | angiogenic factor with G-patch and FHA domains 1 | **0,974** | 0,559582 | 9,416 | 9,067 |
| ZNF841 | zinc finger protein 841 | **0,974** | 0,555093 | 14,180 | 12,770 |
| ELOVL6 | ELOVL fatty acid elongase 6 | **0,974** | 0,563735 | 8,973 | 6,640 |
| EPN2 | epsin 2 | **0,974** | 0,410306 | 24,372 | 25,987 |
| POLR2D | RNA polymerase II subunit D | **0,974** | 0,42363 | 21,436 | 23,624 |
| FNDC10 | fibronectin type III domain containing 10 | **0,974** | 0,848102 | 1,440 | 0,702 |
| PCSK4 | proprotein convertase subtilisin/kexin type 4 | **0,974** | 0,891573 | 0,388 | 0,128 |
| CFL2 | cofilin 2 | **0,974** | 0,422432 | 29,412 | 21,134 |

| | | | | | |
|---|---|---|---|---|---|
| RABL3 | RAB, member of RAS oncogene family like 3 | **0,974** | 0,42565 | 19,276 | 20,815 |
| INAFM1 | InaF motif containing 1 | **0,974** | 0,648159 | 6,204 | 9,705 |
| TRIM35 | tripartite motif containing 35 | **0,974** | 0,450878 | 19,442 | 19,921 |
| RAD17 | RAD17 checkpoint clamp loader component | **0,974** | 0,894826 | 0,332 | 0,255 |
| ARHGEF12 | Rho guanine nucleotide exchange factor 12 | **0,974** | 0,485861 | 102,804 | 95,839 |
| PRNP | prion protein | **0,974** | 0,360678 | 194,696 | 189,251 |
| ACO2 | aconitase 2 | **0,974** | 0,300741 | 83,473 | 83,516 |
| PSMD10 | proteasome 26S subunit, non-ATPase 10 | **0,974** | 0,383688 | 37,887 | 38,438 |
| SMARCD1 | SWI/SNF related, matrix associated, actin dependent regulator of chromatin, subfamily d, | **0,974** | 0,347051 | 35,450 | 30,265 |
| CACTIN | cactin, spliceosome C complex subunit | **0,974** | 0,396352 | 21,824 | 23,050 |
| RPS4X | ribosomal protein S4, X-linked | **0,974** | 0,371448 | 543,433 | 525,548 |
| RNF135 | ring finger protein 135 | **0,974** | 0,552581 | 11,909 | 10,599 |
| ZNF658 | zinc finger protein 658 | **0,974** | 0,769408 | 1,551 | 1,596 |
| RPL10 | ribosomal protein L10 | **0,973** | 0,303473 | 445,005 | 323,974 |
| AGO1 | argonaute 1, RISC catalytic component | **0,973** | 0,437811 | 22,267 | 22,539 |
| LDHAP4 | lactate dehydrogenase A pseudogene 4 | **0,973** | 0,63929 | 3,767 | 6,066 |
| USP7 | ubiquitin specific peptidase 7 | **0,973** | 0,309997 | 79,817 | 76,428 |
| MAPK13 | mitogen-activated protein kinase 13 | **0,973** | 0,809519 | 0,775 | 2,426 |
| CDK13 | cyclin dependent kinase 13 | **0,973** | 0,461123 | 39,881 | 38,438 |
| BTBD10 | BTB domain containing 10 | **0,973** | 0,473073 | 18,500 | 16,090 |
| THAP9-AS1 | THAP9 antisense RNA 1 | **0,973** | 0,429442 | 18,666 | 21,964 |
| ATP2A2 | ATPase sarcoplasmic/endoplasmic reticulum Ca2+ transporting 2 | **0,973** | 0,40755 | 255,736 | 222,708 |
| RPRD2 | regulation of nuclear pre-mRNA domain containing 2 | **0,973** | 0,454423 | 46,860 | 49,739 |
| APTX | aprataxin | **0,973** | 0,481959 | 15,565 | 16,346 |
| DTWD2 | DTW domain containing 2 | **0,973** | 0,749997 | 2,825 | 2,618 |
| TMEM67 | transmembrane protein 67 | **0,973** | 0,390644 | 15,454 | 18,261 |
| C7orf50 | chromosome 7 open reading frame 50 | **0,973** | 0,39105 | 45,752 | 40,800 |
| LIF | LIF, interleukin 6 family cytokine | **0,973** | 0,43609 | 723,562 | 965,346 |
| GBF1 | golgi brefeldin A resistant guanine nucleotide exchange factor 1 | **0,973** | 0,366661 | 93,997 | 97,180 |
| HAUS2 | HAUS augmin like complex subunit 2 | **0,973** | 0,493169 | 14,457 | 16,282 |
| C11orf84 | chromosome 11 open reading frame 84 | **0,973** | 0,58662 | 7,256 | 5,746 |
| AKR1C2 | aldo-keto reductase family 1 member C2 | **0,973** | 0,355694 | 1165,021 | 841,478 |
| BVES | blood vessel epicardial substance | **0,973** | 0,516984 | 16,839 | 7,726 |

| | | | | | |
|---|---|---|---|---|---|
| AIMP1 | aminoacyl tRNA synthetase complex interacting multifunctional protein 1 | **0,973** | 0,414241 | 34,065 | 39,715 |
| DAPK2 | death associated protein kinase 2 | **0,973** | 0,440592 | 20,162 | 18,197 |
| NARF | nuclear prelamin A recognition factor | **0,973** | 0,393017 | 24,316 | 19,602 |
| RPS11 | ribosomal protein S11 | **0,973** | 0,367503 | 462,951 | 400,850 |
| HSPG2 | heparan sulfate proteoglycan 2 | **0,973** | 0,461582 | 1114,893 | 765,879 |
| CYP27B1 | cytochrome P450 family 27 subfamily B member 1 | **0,973** | 0,790609 | 1,939 | 1,022 |
| MTX2 | metaxin 2 | **0,973** | 0,384216 | 19,774 | 23,497 |
| ATP5F1 | ATP synthase, H+ transporting, mitochondrial Fo complex subunit B1 | **0,973** | 0,326431 | 66,690 | 63,339 |
| RNASEH2B | ribonuclease H2 subunit B | **0,973** | 0,61009 | 6,647 | 5,874 |
| VPS39 | VPS39, HOPS complex subunit | **0,973** | 0,309211 | 63,865 | 65,957 |
| RABGGTB | Rab geranylgeranyltransferase beta subunit | **0,973** | 0,372512 | 45,808 | 47,504 |
| H6PD | hexose-6-phosphate dehydrogenase/glucose 1-dehydrogenase | **0,973** | 0,332193 | 87,461 | 79,302 |
| NAMPT | nicotinamide phosphoribosyltransferase | **0,973** | 0,318921 | 767,763 | 453,526 |
| CHI3L2 | chitinase 3 like 2 | **0,973** | 0,319506 | 2480,039 | 1312,370 |
| EIF3J-AS1 | EIF3J antisense RNA 1 (head to head) | **0,973** | 0,518084 | 10,524 | 9,641 |
| SPRTN | SprT-like N-terminal domain | **0,973** | 0,529727 | 11,798 | 11,940 |
| PLCB3 | phospholipase C beta 3 | **0,973** | 0,521369 | 11,466 | 9,322 |
| POLR1D | RNA polymerase I subunit D | **0,973** | 0,396756 | 26,920 | 26,370 |
| EMC8 | ER membrane protein complex subunit 8 | **0,973** | 0,392607 | 16,229 | 19,091 |
| TRAPPC2 | trafficking protein particle complex 2 | **0,973** | 0,541217 | 8,862 | 8,109 |
| KATNB1 | katanin regulatory subunit B1 | **0,973** | 0,476556 | 11,466 | 10,471 |
| SUMO2 | small ubiquitin-like modifier 2 | **0,973** | 0,355827 | 39,770 | 37,352 |
| TJAP1 | tight junction associated protein 1 | **0,973** | 0,323593 | 31,185 | 30,137 |
| RWDD4 | RWD domain containing 4 | **0,973** | 0,509579 | 12,297 | 12,706 |
| MORF4L1P1 | mortality factor 4 like 1 pseudogene 1 | **0,973** | 0,672447 | 8,752 | 7,343 |
| TRMT61B | tRNA methyltransferase 61B | **0,973** | 0,55821 | 7,644 | 5,363 |
| CTPS1 | CTP synthase 1 | **0,973** | 0,401104 | 16,783 | 16,218 |
| CTSS | cathepsin S | **0,973** | 0,341849 | 121,083 | 151,005 |
| CHTF8 | chromosome transmission fidelity factor 8 | **0,973** | 0,319484 | 59,378 | 61,679 |
| SEMA3C | semaphorin 3C | **0,973** | 0,362067 | 1109,133 | 649,481 |
| MRPL49 | mitochondrial ribosomal protein L49 | **0,973** | 0,30307 | 35,450 | 39,076 |
| SHPRH | SNF2 histone linker PHD RING helicase | **0,973** | 0,635287 | 12,020 | 10,599 |
| NEK1 | NIMA related kinase 1 | **0,973** | 0,471429 | 16,008 | 19,602 |

| | | | | | |
|---|---|---|---|---|---|
| CAVIN1 | caveolae associated protein 1 | **0,972** | 0,339848 | 919,587 | 855,588 |
| MED30 | mediator complex subunit 30 | **0,972** | 0,612651 | 6,314 | 8,556 |
| NUDT16 | nudix hydrolase 16 | **0,972** | 0,466691 | 24,372 | 25,668 |
| EXTL3 | exostosin like glycosyltransferase 3 | **0,972** | 0,404529 | 44,700 | 41,822 |
| TTF1 | transcription termination factor 1 | **0,972** | 0,409984 | 15,620 | 15,962 |
| LRRC28 | leucine rich repeat containing 28 | **0,972** | 0,614852 | 5,484 | 4,342 |
| TSFM | Ts translation elongation factor, mitochondrial | **0,972** | 0,53085 | 9,582 | 10,152 |
| ATP6V0C | ATPase H+ transporting V0 subunit c | **0,972** | 0,864305 | 0,499 | 0,447 |
| UCKL1-AS1 | UCKL1 antisense RNA 1 | **0,972** | 0,823219 | 0,997 | 0,958 |
| FGD1 | FYVE, RhoGEF and PH domain containing 1 | **0,972** | 0,61497 | 5,484 | 4,789 |
| RPS8 | ribosomal protein S8 | **0,972** | 0,395403 | 389,504 | 385,398 |
| PIGZ | phosphatidylinositol glycan anchor biosynthesis class Z | **0,972** | 0,660539 | 3,877 | 3,767 |
| GOSR1 | golgi SNAP receptor complex member 1 | **0,972** | 0,303964 | 35,228 | 41,375 |
| CAPZA1 | capping actin protein of muscle Z-line alpha subunit 1 | **0,972** | 0,310198 | 92,169 | 75,854 |
| PGRMC2 | progesterone receptor membrane component 2 | **0,972** | 0,355678 | 50,350 | 46,163 |
| ZNF496 | zinc finger protein 496 | **0,972** | 0,417369 | 21,713 | 21,709 |
| SSTR5 | somatostatin receptor 5 | **0,972** | 0,832792 | 0,942 | 0,638 |
| LIN9 | lin-9 DREAM MuvB core complex component | **0,972** | 0,698267 | 3,323 | 3,001 |
| CACNA1A | calcium voltage-gated channel subunit alpha1 A | **0,972** | 0,755192 | 1,717 | 1,788 |
| C2orf42 | chromosome 2 open reading frame 42 | **0,972** | 0,507267 | 11,300 | 11,046 |
| SLC25A14 | solute carrier family 25 member 14 | **0,972** | 0,59979 | 5,539 | 7,981 |
| S100A1 | S100 calcium binding protein A1 | **0,972** | 0,469624 | 128,671 | 82,366 |
| GSTM5 | glutathione S-transferase mu 5 | **0,972** | 0,869181 | 1,496 | 0,702 |
| CCNO | cyclin O | **0,972** | 0,609995 | 7,755 | 9,131 |
| ADPGK | ADP dependent glucokinase | **0,972** | 0,283877 | 73,614 | 66,340 |
| ZCWPW1 | zinc finger CW-type and PWWP domain containing 1 | **0,972** | 0,54928 | 6,093 | 8,300 |
| KHK | ketohexokinase | **0,972** | 0,823011 | 0,886 | 1,149 |
| LIMS2 | LIM zinc finger domain containing 2 | **0,972** | 0,601214 | 4,376 | 2,937 |
| BCL10 | B-cell CLL/lymphoma 10 | **0,972** | 0,392358 | 28,637 | 34,096 |
| TM2D3 | TM2 domain containing 3 | **0,972** | 0,342922 | 30,963 | 29,243 |
| FBXO25 | F-box protein 25 | **0,972** | 0,407691 | 14,568 | 11,110 |
| SARNP | SAP domain containing ribonucleoprotein | **0,972** | 0,763159 | 0,997 | 1,085 |
| IP6K2 | inositol hexakisphosphate kinase 2 | **0,972** | 0,345653 | 30,409 | 31,031 |

| | | | | | |
|---|---|---|---|---|---|
| RPF1 | ribosome production factor 1 homolog | **0,972** | 0,392808 | 28,471 | 30,265 |
| PTTG1IP | PTTG1 interacting protein | **0,972** | 0,241147 | 1168,400 | 858,781 |
| C19orf68 | chromosome 19 open reading frame 68 | **0,972** | 0,582854 | 8,364 | 6,577 |
| VSIG10 | V-set and immunoglobulin domain containing 10 | **0,972** | 0,437357 | 12,795 | 10,088 |
| LSM5 | LSM5 homolog, U6 small nuclear RNA and mRNA degradation associated | **0,972** | 0,44879 | 14,235 | 14,558 |
| SUGP1 | SURP and G-patch domain containing 1 | **0,972** | 0,470735 | 20,051 | 20,560 |
| NGRN | neugrin, neurite outgrowth associated | **0,972** | 0,34424 | 24,649 | 19,921 |
| FAM228B | family with sequence similarity 228 member B | **0,972** | 0,68064 | 3,490 | 3,001 |
| MSH3 | mutS homolog 3 | **0,972** | 0,48438 | 11,466 | 12,004 |
| AVEN | apoptosis and caspase activation inhibitor | **0,972** | 0,383657 | 14,789 | 12,706 |
| RANBP9 | RAN binding protein 9 | **0,971** | 0,360681 | 57,440 | 46,802 |
| TMEM200B | transmembrane protein 200B | **0,971** | 0,750018 | 3,767 | 1,724 |
| RAB27A | RAB27A, member RAS oncogene family | **0,971** | 0,326216 | 20,993 | 21,007 |
| TTPAL | alpha tocopherol transfer protein like | **0,971** | 0,44617 | 19,664 | 20,815 |
| FAM49B | family with sequence similarity 49 member B | **0,971** | 0,488216 | 13,127 | 14,047 |
| WDR63 | WD repeat domain 63 | **0,971** | 0,826132 | 0,554 | 0,702 |
| USP4 | ubiquitin specific peptidase 4 | **0,971** | 0,283573 | 36,558 | 33,010 |
| DPT | dermatopontin | **0,971** | 0,53656 | 15,288 | 7,854 |
| MTAP | methylthioadenosine phosphorylase | **0,971** | 0,379582 | 25,812 | 27,583 |
| GNB4 | G protein subunit beta 4 | **0,971** | 0,442731 | 19,442 | 15,643 |
| GCA | grancalcin | **0,971** | 0,66968 | 3,656 | 3,448 |
| WISP3 | WNT1 inducible signaling pathway protein 3 | **0,971** | 0,313197 | 31,018 | 17,303 |
| PIK3CB | phosphatidylinositol-4,5-bisphosphate 3-kinase catalytic subunit beta | **0,971** | 0,522647 | 14,346 | 13,025 |
| MICAL1 | microtubule associated monooxygenase, calponin and LIM domain containing 1 | **0,971** | 0,439744 | 18,556 | 13,089 |
| GPC6 | glypican 6 | **0,971** | 0,3543 | 163,290 | 142,768 |
| CST6 | cystatin E/M | **0,971** | 0,528618 | 12,795 | 5,555 |
| NOL3 | nucleolar protein 3 | **0,971** | 0,293482 | 24,316 | 17,431 |
| PRPSAP2 | phosphoribosyl pyrophosphate synthetase associated protein 2 | **0,971** | 0,462175 | 16,672 | 14,941 |
| SPATA17 | spermatogenesis associated 17 | **0,971** | 0,751525 | 0,775 | 1,596 |
| SERGEF | secretion regulating guanine nucleotide exchange factor | **0,971** | 0,692382 | 5,484 | 3,703 |
| CCDC47 | coiled-coil domain containing 47 | **0,971** | 0,240197 | 149,941 | 143,088 |
| GFOD2 | glucose-fructose oxidoreductase domain containing 2 | **0,971** | 0,479191 | 18,168 | 15,835 |
| PGM2L1 | phosphoglucomutase 2 like 1 | **0,971** | 0,425107 | 23,541 | 25,221 |

| | | | | | |
|---|---|---|---|---|---|
| CLPTM1 | CLPTM1, transmembrane protein | **0,971** | 0,265802 | 152,434 | 154,900 |
| PROSER3 | proline and serine rich 3 | **0,971** | 0,648477 | 8,807 | 9,258 |
| ADI1 | acireductone dioxygenase 1 | **0,971** | 0,5686 | 6,425 | 5,491 |
| NFKBIE | NFKB inhibitor epsilon | **0,971** | 0,410261 | 33,677 | 28,732 |
| H19 | H19, imprinted maternally expressed transcript (non-protein coding) | **0,971** | 0,702355 | 2,216 | 2,299 |
| UFD1 | ubiquitin recognition factor in ER associated degradation 1 | **0,971** | 0,24658 | 50,460 | 44,950 |
| ABCA1 | ATP binding cassette subfamily A member 1 | **0,971** | 0,701461 | 2,105 | 5,555 |
| GBP3 | guanylate binding protein 3 | **0,971** | 0,349196 | 45,863 | 96,669 |
| NDUFAF1 | NADH:ubiquinone oxidoreductase complex assembly factor 1 | **0,971** | 0,457149 | 15,509 | 16,729 |
| USP13 | ubiquitin specific peptidase 13 (isopeptidase T-3) | **0,971** | 0,334975 | 28,637 | 29,882 |
| TANC2 | tetratricopeptide repeat, ankyrin repeat and coiled-coil containing 2 | **0,971** | 0,671935 | 7,312 | 5,172 |
| TAF5L | TATA-box binding protein associated factor 5 like | **0,971** | 0,450665 | 13,017 | 11,365 |
| TACSTD2 | tumor associated calcium signal transducer 2 | **0,971** | 0,336474 | 55,390 | 47,568 |
| ATG4C | autophagy related 4C cysteine peptidase | **0,971** | 0,632942 | 5,096 | 4,725 |
| CCT8 | chaperonin containing TCP1 subunit 8 | **0,971** | 0,310162 | 96,047 | 100,117 |
| RIDA | reactive intermediate imine deaminase A homolog | **0,971** | 0,616305 | 5,982 | 5,427 |
| CDKN2C | cyclin dependent kinase inhibitor 2C | **0,971** | 0,667357 | 11,909 | 4,214 |
| VTI1B | vesicle transport through interaction with t-SNAREs 1B | **0,971** | 0,31005 | 37,499 | 32,755 |
| ZNF808 | zinc finger protein 808 | **0,971** | 0,676371 | 4,099 | 3,831 |
| LRRC8D | leucine rich repeat containing 8 family member D | **0,971** | 0,438265 | 14,069 | 10,982 |
| PDK1 | pyruvate dehydrogenase kinase 1 | **0,971** | 0,292312 | 18,445 | 19,283 |
| ITIH5 | inter-alpha-trypsin inhibitor heavy chain family member 5 | **0,971** | 0,425405 | 20,882 | 8,811 |
| NAA40 | N(alpha)-acetyltransferase 40, NatD catalytic subunit | **0,971** | 0,387692 | 12,740 | 12,898 |
| ZBTB42 | zinc finger and BTB domain containing 42 | **0,971** | 0,746623 | 1,828 | 1,341 |
| GPR137 | G protein-coupled receptor 137 | **0,971** | 0,364224 | 27,750 | 23,050 |
| ENOSF1 | enolase superfamily member 1 | **0,971** | 0,520238 | 26,753 | 36,331 |
| SAMD10 | sterile alpha motif domain containing 10 | **0,971** | 0,847964 | 0,720 | 0,192 |
| HGS | hepatocyte growth factor-regulated tyrosine kinase substrate | **0,971** | 0,306847 | 75,718 | 71,256 |
| PEX12 | peroxisomal biogenesis factor 12 | **0,971** | 0,391454 | 21,547 | 22,475 |
| PTPRF | protein tyrosine phosphatase, receptor type F | **0,971** | 0,500244 | 16,506 | 11,621 |
| ELK1 | ELK1, ETS transcription factor | **0,971** | 0,308837 | 35,560 | 34,479 |
| KHDRBS1 | KH RNA binding domain containing, signal transduction associated 1 | **0,970** | 0,226108 | 96,767 | 116,845 |
| UNC5CL | unc-5 family C-terminal like | **0,970** | 0,88401 | 0,166 | 0,511 |

| | | | | | |
|---|---|---|---|---|---|
| ACIN1 | apoptotic chromatin condensation inducer 1 | **0,970** | 0,269607 | 107,346 | 99,606 |
| THBS1 | thrombospondin 1 | **0,970** | 0,489472 | 305,698 | 317,206 |
| ZNF781 | zinc finger protein 781 | **0,970** | 0,877222 | 0,443 | 1,085 |
| MIR497HG | mir-497-195 cluster host gene | **0,970** | 0,790953 | 0,942 | 1,724 |
| LRP11 | LDL receptor related protein 11 | **0,970** | 0,252169 | 48,577 | 34,734 |
| TMEM8B | transmembrane protein 8B | **0,970** | 0,465865 | 11,632 | 16,218 |
| THAP1 | THAP domain containing 1 | **0,970** | 0,496249 | 8,752 | 8,620 |
| BRCC3 | BRCA1/BRCA2-containing complex subunit 3 | **0,970** | 0,498459 | 11,189 | 10,088 |
| MAEA | macrophage erythroblast attacher | **0,970** | 0,253821 | 55,667 | 55,166 |
| CCDC146 | coiled-coil domain containing 146 | **0,970** | 0,755109 | 1,496 | 1,469 |
| CCZ1B | CCZ1 homolog B, vacuolar protein trafficking and biogenesis associated | **0,970** | 0,646691 | 2,049 | 5,172 |
| FAM20B | FAM20B, glycosaminoglycan xylosylkinase | **0,970** | 0,331127 | 74,500 | 64,105 |
| NARS2 | asparaginyl-tRNA synthetase 2, mitochondrial (putative) | **0,970** | 0,515799 | 9,527 | 13,025 |
| BSG | basigin (Ok blood group) | **0,970** | 0,307946 | 302,375 | 198,509 |
| TRIAP1 | TP53 regulated inhibitor of apoptosis 1 | **0,970** | 0,393215 | 12,851 | 12,323 |
| GPR157 | G protein-coupled receptor 157 | **0,970** | 0,541334 | 7,090 | 6,832 |
| GPR88 | G protein-coupled receptor 88 | **0,970** | 0,28788 | 30,077 | 51,208 |
| PCTP | phosphatidylcholine transfer protein | **0,970** | 0,574408 | 7,976 | 7,023 |
| RPAP2 | RNA polymerase II associated protein 2 | **0,970** | 0,494123 | 14,069 | 16,282 |
| SMIM26 | small integral membrane protein 26 | **0,970** | 0,48105 | 7,090 | 8,875 |
| CCDC51 | coiled-coil domain containing 51 | **0,970** | 0,547437 | 7,312 | 7,151 |
| IKBKAP | inhibitor of kappa light polypeptide gene enhancer in B-cells, kinase complex-associated pr | **0,970** | 0,243627 | 36,391 | 44,248 |
| TRO | trophinin | **0,970** | 0,668873 | 4,210 | 3,448 |
| PWWP2B | PWWP domain containing 2B | **0,970** | 0,647471 | 4,985 | 3,320 |
| C1orf174 | chromosome 1 open reading frame 174 | **0,970** | 0,423576 | 12,851 | 12,898 |
| NACA | nascent polypeptide-associated complex alpha subunit | **0,970** | 0,313272 | 210,815 | 214,280 |
| RAE1 | ribonucleic acid export 1 | **0,970** | 0,342054 | 24,704 | 23,305 |
| INTS6 | integrator complex subunit 6 | **0,970** | 0,456443 | 20,217 | 18,580 |
| KPNA6 | karyopherin subunit alpha 6 | **0,970** | 0,336949 | 49,076 | 46,547 |
| CCDC59 | coiled-coil domain containing 59 | **0,970** | 0,482334 | 17,891 | 23,433 |
| AIFM1 | apoptosis inducing factor mitochondria associated 1 | **0,970** | 0,479148 | 15,343 | 14,685 |
| TMEM138 | transmembrane protein 138 | **0,970** | 0,467196 | 16,119 | 17,303 |
| F8A1 | coagulation factor VIII-associated 1 | **0,970** | 0,701576 | 3,268 | 3,192 |

| | | | | | |
|---|---|---|---|---|---|
| IWS1 | IWS1, SUPT6H interacting protein | **0,970** | 0,291554 | 32,292 | 32,116 |
| RAB40C | RAB40C, member RAS oncogene family | **0,970** | 0,375668 | 17,891 | 11,876 |
| ANK1 | ankyrin 1 | **0,970** | 0,866321 | 0,388 | 0,702 |
| SSB | Sjogren syndrome antigen B | **0,970** | 0,37282 | 72,949 | 71,767 |
| GEMIN8 | gem nuclear organelle associated protein 8 | **0,970** | 0,499238 | 6,481 | 6,704 |
| LCA5L | LCA5L, lebercilin like | **0,970** | 0,68981 | 2,936 | 3,065 |
| RGP1 | RGP1 homolog, RAB6A GEF complex partner 1 | **0,970** | 0,357824 | 59,766 | 57,146 |
| NOP14-AS1 | NOP14 antisense RNA 1 | **0,970** | 0,45969 | 11,466 | 8,684 |
| RPA2 | replication protein A2 | **0,970** | 0,237259 | 33,954 | 46,483 |
| DNAJB5 | DnaJ heat shock protein family (Hsp40) member B5 | **0,970** | 0,524631 | 7,976 | 5,491 |
| AARS2 | alanyl-tRNA synthetase 2, mitochondrial | **0,970** | 0,426353 | 11,743 | 13,919 |
| KDM5C | lysine demethylase 5C | **0,970** | 0,293803 | 137,589 | 163,009 |
| PRPF40A | pre-mRNA processing factor 40 homolog A | **0,970** | 0,270594 | 65,416 | 63,403 |
| DENND6A | DENN domain containing 6A | **0,970** | 0,483954 | 8,309 | 11,301 |
| ANKRD37 | ankyrin repeat domain 37 | **0,970** | 0,466515 | 10,635 | 11,301 |
| BCAP31 | B-cell receptor-associated protein 31 | **0,970** | 0,337359 | 119,421 | 84,729 |
| TNS2 | tensin 2 | **0,970** | 0,445362 | 24,372 | 10,918 |
| SATB1 | SATB homeobox 1 | **0,970** | 0,518493 | 21,270 | 16,665 |
| PSMD11 | proteasome 26S subunit, non-ATPase 11 | **0,970** | 0,224092 | 91,006 | 77,067 |
| ZDHHC14 | zinc finger DHHC-type containing 14 | **0,970** | 0,487962 | 8,032 | 9,194 |
| POLR3GL | RNA polymerase III subunit G like | **0,970** | 0,533559 | 7,312 | 6,896 |
| TM2D2 | TM2 domain containing 2 | **0,970** | 0,343679 | 33,788 | 30,584 |
| UGDH-AS1 | UGDH antisense RNA 1 | **0,970** | 0,678316 | 2,049 | 2,362 |
| ANKRD39 | ankyrin repeat domain 39 | **0,970** | 0,724652 | 1,440 | 1,915 |
| LHFP | lipoma HMGIC fusion partner | **0,970** | 0,313461 | 28,803 | 27,455 |
| RFC1 | replication factor C subunit 1 | **0,970** | 0,402383 | 24,870 | 24,008 |
| NSFL1C | NSFL1 cofactor | **0,970** | 0,235563 | 84,193 | 84,026 |
| SEPHS1 | selenophosphate synthetase 1 | **0,970** | 0,268887 | 24,870 | 20,240 |
| HLCS | holocarboxylase synthetase | **0,970** | 0,413291 | 15,288 | 19,857 |
| FAM208B | family with sequence similarity 208 member B | **0,970** | 0,414014 | 68,518 | 77,705 |
| TPMT | thiopurine S-methyltransferase | **0,970** | 0,432655 | 14,789 | 15,899 |
| TLN1 | talin 1 | **0,969** | 0,379446 | 518,341 | 474,149 |
| TCEANC2 | transcription elongation factor A N-terminal and central domain containing 2 | **0,969** | 0,471791 | 7,145 | 9,577 |

| | | | | | |
|---|---|---|---|---|---|
| NAA25 | N(alpha)-acetyltransferase 25, NatB auxiliary subunit | **0,969** | 0,430253 | 22,045 | 25,668 |
| JAM3 | junctional adhesion molecule 3 | **0,969** | 0,319332 | 35,228 | 34,734 |
| IRAK3 | interleukin 1 receptor associated kinase 3 | **0,969** | 0,312687 | 50,682 | 54,975 |
| IDUA | iduronidase, alpha-L- | **0,969** | 0,392263 | 15,620 | 13,600 |
| THTPA | thiamine triphosphatase | **0,969** | 0,624664 | 3,767 | 4,278 |
| CCDC171 | coiled-coil domain containing 171 | **0,969** | 0,704934 | 2,160 | 1,979 |
| XBP1 | X-box binding protein 1 | **0,969** | 0,239858 | 398,532 | 441,522 |
| MTMR14 | myotubularin related protein 14 | **0,969** | 0,346762 | 18,666 | 21,070 |
| SNRPA1 | small nuclear ribonucleoprotein polypeptide A' | **0,969** | 0,441509 | 10,358 | 12,004 |
| TRNAU1AP | tRNA selenocysteine 1 associated protein 1 | **0,969** | 0,575351 | 7,533 | 6,130 |
| LINC01270 | long intergenic non-protein coding RNA 1270 | **0,969** | 0,836589 | 0,886 | 0,958 |
| LDB1 | LIM domain binding 1 | **0,969** | 0,419869 | 19,664 | 19,219 |
| ATP5B | ATP synthase, H+ transporting, mitochondrial F1 complex, beta polypeptide | **0,969** | 0,212814 | 249,699 | 229,668 |
| QSOX2 | quiescin sulfhydryl oxidase 2 | **0,969** | 0,288506 | 57,661 | 54,783 |
| SHCBP1 | SHC binding and spindle associated 1 | **0,969** | 0,760519 | 5,041 | 0,830 |
| WDYHV1 | WDYHV motif containing 1 | **0,969** | 0,58806 | 5,927 | 5,491 |
| GOT1 | glutamic-oxaloacetic transaminase 1 | **0,969** | 0,313583 | 46,140 | 58,870 |
| ZNF829 | zinc finger protein 829 | **0,969** | 0,558317 | 4,653 | 4,661 |
| ZNF407 | zinc finger protein 407 | **0,969** | 0,413624 | 12,574 | 14,494 |
| SCAMP4 | secretory carrier membrane protein 4 | **0,969** | 0,299303 | 48,743 | 41,311 |
| KRR1 | KRR1, small subunit processome component homolog | **0,969** | 0,275893 | 30,797 | 39,842 |
| HMGA2 | high mobility group AT-hook 2 | **0,969** | 0,459112 | 32,237 | 12,004 |
| ZNF76 | zinc finger protein 76 | **0,969** | 0,38831 | 14,845 | 18,325 |
| TOR1A | torsin family 1 member A | **0,969** | 0,292177 | 32,846 | 27,583 |
| COX7A2L | cytochrome c oxidase subunit 7A2 like | **0,969** | 0,274301 | 62,757 | 58,295 |
| PFDN5 | prefoldin subunit 5 | **0,969** | 0,307492 | 90,508 | 78,982 |
| TEFM | transcription elongation factor, mitochondrial | **0,969** | 0,511452 | 7,256 | 9,641 |
| VKORC1L1 | vitamin K epoxide reductase complex subunit 1 like 1 | **0,969** | 0,282555 | 31,129 | 26,370 |
| EBF4 | early B-cell factor 4 | **0,969** | 0,775104 | 1,717 | 0,575 |
| GNPAT | glyceronephosphate O-acyltransferase | **0,969** | 0,247668 | 25,092 | 24,901 |
| SELENON | selenoprotein N | **0,969** | 0,298069 | 26,255 | 21,390 |
| A2M-AS1 | A2M antisense RNA 1 (head to head) | **0,969** | 0,858224 | 0,443 | 0,575 |
| ELF4 | E74 like ETS transcription factor 4 | **0,969** | 0,420511 | 19,774 | 15,196 |

| | | | | | |
|---|---|---|---|---|---|
| CYP11A1 | cytochrome P450 family 11 subfamily A member 1 | **0,969** | 0,81303 | 0,775 | 1,277 |
| STARD10 | StAR related lipid transfer domain containing 10 | **0,969** | 0,354881 | 34,010 | 26,178 |
| MRPL10 | mitochondrial ribosomal protein L10 | **0,969** | 0,29288 | 21,824 | 27,711 |
| RPS3 | ribosomal protein S3 | **0,969** | 0,343469 | 383,521 | 407,937 |
| CCDC7 | coiled-coil domain containing 7 | **0,969** | 0,763442 | 2,160 | 3,192 |
| NCSTN | nicastrin | **0,969** | 0,253277 | 73,060 | 56,826 |
| OSBPL10 | oxysterol binding protein like 10 | **0,969** | 0,274044 | 44,201 | 48,398 |
| ZNF518B | zinc finger protein 518B | **0,969** | 0,538991 | 12,407 | 10,663 |
| DGUOK-AS1 | DGUOK antisense RNA 1 | **0,969** | 0,857149 | 0,499 | 0,638 |
| EGFR | epidermal growth factor receptor | **0,969** | 0,42029 | 174,257 | 222,581 |
| CCNG1 | cyclin G1 | **0,969** | 0,258605 | 30,742 | 24,710 |
| DOHH | deoxyhypusine hydroxylase | **0,969** | 0,337929 | 16,949 | 19,857 |
| BTRC | beta-transducin repeat containing E3 ubiquitin protein ligase | **0,969** | 0,447019 | 21,713 | 20,560 |
| OGFOD1 | 2-oxoglutarate and iron dependent oxygenase domain containing 1 | **0,968** | 0,288082 | 32,791 | 37,352 |
| COL4A2 | collagen type IV alpha 2 chain | **0,968** | 0,506504 | 7,755 | 7,215 |
| MRPL23 | mitochondrial ribosomal protein L23 | **0,968** | 0,806718 | 0,942 | 1,341 |
| COG1 | component of oligomeric golgi complex 1 | **0,968** | 0,296952 | 28,858 | 30,009 |
| CNTLN | centlein | **0,968** | 0,537885 | 7,422 | 5,810 |
| ARHGAP42 | Rho GTPase activating protein 42 | **0,968** | 0,360722 | 44,478 | 49,739 |
| GNA12 | G protein subunit alpha 12 | **0,968** | 0,222632 | 74,001 | 62,317 |
| STX1A | syntaxin 1A | **0,968** | 0,48508 | 10,801 | 9,769 |
| MAATS1 | MYCBP associated and testis expressed 1 | **0,968** | 0,566391 | 3,046 | 6,002 |
| C12orf73 | chromosome 12 open reading frame 73 | **0,968** | 0,706523 | 2,991 | 2,107 |
| HES1 | hes family bHLH transcription factor 1 | **0,968** | 0,614686 | 5,816 | 7,534 |
| MRPL14 | mitochondrial ribosomal protein L14 | **0,968** | 0,360252 | 34,397 | 29,882 |
| NDNF | neuron derived neurotrophic factor | **0,968** | 0,391631 | 19,054 | 18,708 |
| PHF2 | PHD finger protein 2 | **0,968** | 0,336727 | 35,782 | 29,946 |
| ARRDC1-AS1 | ARRDC1 antisense RNA 1 | **0,968** | 0,569517 | 6,481 | 8,492 |
| BAG2 | BCL2 associated athanogene 2 | **0,968** | 0,485669 | 18,168 | 14,749 |
| OTUD6B-AS1 | OTUD6B antisense RNA 1 (head to head) | **0,968** | 0,272659 | 44,755 | 40,417 |
| MTBP | MDM2 binding protein | **0,968** | 0,622388 | 3,213 | 3,512 |
| FOXRED1 | FAD dependent oxidoreductase domain containing 1 | **0,968** | 0,489 | 9,859 | 10,152 |
| ZCCHC10 | zinc finger CCHC-type containing 10 | **0,968** | 0,440938 | 10,247 | 11,493 |

| | | | | | |
|---|---|---|---|---|---|
| CREM | cAMP responsive element modulator | **0,968** | 0,33692 | 24,981 | 25,029 |
| RIOK2 | RIO kinase 2 | **0,968** | 0,269814 | 24,261 | 25,157 |
| CEP41 | centrosomal protein 41 | **0,968** | 0,437606 | 4,764 | 9,705 |
| ABCA17P | ATP binding cassette subfamily A member 17, pseudogene | **0,968** | 0,835869 | 0,332 | 0,702 |
| CROCCP2 | ciliary rootlet coiled-coil, rootletin pseudogene 2 | **0,968** | 0,415988 | 14,789 | 13,664 |
| RPL18A | ribosomal protein L18a | **0,968** | 0,29024 | 164,841 | 156,751 |
| PRDX1 | peroxiredoxin 1 | **0,968** | 0,223699 | 566,364 | 431,178 |
| ZCCHC7 | zinc finger CCHC-type containing 7 | **0,968** | 0,32031 | 18,223 | 21,070 |
| PRTG | protogenin | **0,968** | 0,663769 | 3,379 | 3,831 |
| TBC1D24 | TBC1 domain family member 24 | **0,968** | 0,425148 | 12,352 | 9,833 |
| MRPL58 | mitochondrial ribosomal protein L58 | **0,968** | 0,496426 | 8,752 | 9,961 |
| NCL | nucleolin | **0,968** | 0,246015 | 320,044 | 310,694 |
| ROCK2 | Rho associated coiled-coil containing protein kinase 2 | **0,968** | 0,356956 | 58,049 | 44,376 |
| CNOT6LP1 | CCR4-NOT transcription complex subunit 6-like pseudogene 1 | **0,968** | 0,767641 | 1,662 | 0,830 |
| SRSF8 | serine and arginine rich splicing factor 8 | **0,968** | 0,312772 | 46,860 | 46,610 |
| CLIP3 | CAP-Gly domain containing linker protein 3 | **0,968** | 0,562778 | 14,235 | 5,427 |
| PINK1-AS | PINK1 antisense RNA | **0,968** | 0,37492 | 16,949 | 20,304 |
| SLC4A5 | solute carrier family 4 member 5 | **0,968** | 0,82108 | 0,443 | 0,958 |
| ITSN1 | intersectin 1 | **0,968** | 0,392359 | 29,080 | 28,605 |
| CARS2 | cysteinyl-tRNA synthetase 2, mitochondrial (putative) | **0,968** | 0,347085 | 16,894 | 16,154 |
| SUMF2 | sulfatase modifying factor 2 | **0,968** | 0,217135 | 109,728 | 75,215 |
| WDR31 | WD repeat domain 31 | **0,968** | 0,592615 | 4,874 | 4,342 |
| ASL | argininosuccinate lyase | **0,968** | 0,34566 | 28,526 | 34,798 |
| PRMT3 | protein arginine methyltransferase 3 | **0,968** | 0,482967 | 9,693 | 9,514 |
| UROD | uroporphyrinogen decarboxylase | **0,968** | 0,32341 | 21,824 | 18,070 |
| PRKCSH | protein kinase C substrate 80K-H | **0,968** | 0,302699 | 224,330 | 152,282 |
| PRKDC | protein kinase, DNA-activated, catalytic polypeptide | **0,968** | 0,346152 | 116,098 | 117,995 |
| SPACA6 | sperm acrosome associated 6 | **0,968** | 0,711018 | 1,551 | 1,852 |
| BLOC1S4 | biogenesis of lysosomal organelles complex 1 subunit 4 | **0,968** | 0,336009 | 16,506 | 16,920 |
| ZNF283 | zinc finger protein 283 | **0,968** | 0,666843 | 3,545 | 4,023 |
| MPP5 | membrane palmitoylated protein 5 | **0,968** | 0,278522 | 38,108 | 36,394 |
| MKX | mohawk homeobox | **0,968** | 0,605226 | 4,708 | 5,108 |
| EEF1A1P12 | eukaryotic translation elongation factor 1 alpha 1 pseudogene 12 | **0,968** | 0,774608 | 1,551 | 0,958 |

| | | | | | |
|---|---|---|---|---|---|
| ARMCX4 | armadillo repeat containing, X-linked 4 | **0,968** | 0,482011 | 7,367 | 7,598 |
| ACVR2B | activin A receptor type 2B | **0,968** | 0,574246 | 7,090 | 5,938 |
| IRX5 | iroquois homeobox 5 | **0,968** | 0,655868 | 4,154 | 3,001 |
| KCNK1 | potassium two pore domain channel subfamily K member 1 | **0,968** | 0,278944 | 38,441 | 54,336 |
| MAIP1 | matrix AAA peptidase interacting protein 1 | **0,967** | 0,405833 | 7,921 | 10,471 |
| SMARCAL1 | SWI/SNF related, matrix associated, actin dependent regulator of chromatin, subfamily a li | **0,967** | 0,370287 | 15,731 | 11,621 |
| HDHD5 | haloacid dehalogenase like hydrolase domain containing 5 | **0,967** | 0,259926 | 17,836 | 15,579 |
| CDK16 | cyclin dependent kinase 16 | **0,967** | 0,253499 | 63,920 | 52,740 |
| TMEM181 | transmembrane protein 181 | **0,967** | 0,329762 | 52,787 | 49,292 |
| MFSD8 | major facilitator superfamily domain containing 8 | **0,967** | 0,375 | 14,845 | 15,707 |
| OXA1L | OXA1L, mitochondrial inner membrane protein | **0,967** | 0,218221 | 39,493 | 42,396 |
| CCDC134 | coiled-coil domain containing 134 | **0,967** | 0,602712 | 3,711 | 5,300 |
| VNN3 | vanin 3 | **0,967** | 0,556586 | 8,419 | 14,047 |
| SEPT7-AS1 | SEPT7 antisense RNA 1 (head to head) | **0,967** | 0,786693 | 1,717 | 1,149 |
| SFI1 | SFI1 centrin binding protein | **0,967** | 0,500957 | 9,804 | 13,472 |
| PGM3 | phosphoglucomutase 3 | **0,967** | 0,263272 | 105,629 | 81,217 |
| GPR75 | G protein-coupled receptor 75 | **0,967** | 0,794467 | 1,052 | 1,532 |
| MAGEE1 | MAGE family member E1 | **0,967** | 0,637406 | 2,936 | 2,490 |
| GHITM | growth hormone inducible transmembrane protein | **0,967** | 0,194619 | 177,802 | 175,906 |
| PHF12 | PHD finger protein 12 | **0,967** | 0,292997 | 28,471 | 30,903 |
| ZNF248 | zinc finger protein 248 | **0,967** | 0,405888 | 8,364 | 8,300 |
| LYPLA2 | lysophospholipase II | **0,967** | 0,342439 | 24,815 | 20,496 |
| VWA8 | von Willebrand factor A domain containing 8 | **0,967** | 0,43345 | 10,192 | 11,238 |
| ARL1 | ADP ribosylation factor like GTPase 1 | **0,967** | 0,260008 | 105,241 | 90,411 |
| HSPH1 | heat shock protein family H (Hsp110) member 1 | **0,967** | 0,289605 | 64,585 | 53,059 |
| PLPP1 | phospholipid phosphatase 1 | **0,967** | 0,388889 | 24,482 | 17,176 |
| MKL1 | megakaryoblastic leukemia (translocation) 1 | **0,967** | 0,339442 | 29,191 | 30,265 |
| DFFB | DNA fragmentation factor subunit beta | **0,967** | 0,659493 | 2,216 | 1,852 |
| E4F1 | E4F transcription factor 1 | **0,967** | 0,277301 | 18,666 | 17,176 |
| CEP57L1 | centrosomal protein 57 like 1 | **0,967** | 0,585597 | 4,653 | 5,044 |
| BRD4 | bromodomain containing 4 | **0,967** | 0,194304 | 65,859 | 67,425 |
| C15orf65 | chromosome 15 open reading frame 65 | **0,967** | 0,664501 | 2,326 | 1,724 |
| PARVA | parvin alpha | **0,967** | 0,189796 | 110,891 | 81,856 |

| | | | | | |
|---|---|---|---|---|---|
| PPP1R37 | protein phosphatase 1 regulatory subunit 37 | **0,967** | 0,286203 | 21,547 | 20,240 |
| MAP1S | microtubule associated protein 1S | **0,967** | 0,321567 | 27,640 | 25,859 |
| IFT140 | intraflagellar transport 140 | **0,967** | 0,364284 | 22,987 | 23,241 |
| EHD2 | EH domain containing 2 | **0,967** | 0,185298 | 221,284 | 158,412 |
| DDX55 | DEAD-box helicase 55 | **0,967** | 0,347056 | 14,235 | 15,579 |
| ADRM1 | adhesion regulating molecule 1 | **0,967** | 0,227492 | 85,301 | 87,474 |
| RANGRF | RAN guanine nucleotide release factor | **0,967** | 0,534165 | 5,262 | 4,278 |
| FAM219B | family with sequence similarity 219 member B | **0,967** | 0,204709 | 38,496 | 35,245 |
| SCRN1 | secernin 1 | **0,967** | 0,202804 | 73,337 | 59,955 |
| NFIC | nuclear factor I C | **0,967** | 0,255935 | 109,506 | 83,005 |
| CCS | copper chaperone for superoxide dismutase | **0,967** | 0,382836 | 21,325 | 20,879 |
| ETF1 | eukaryotic translation termination factor 1 | **0,967** | 0,197671 | 98,816 | 101,330 |
| SMIM15 | small integral membrane protein 15 | **0,967** | 0,225894 | 38,275 | 39,906 |
| SAA1 | serum amyloid A1 | **0,967** | 0,534856 | 90,784 | 60,147 |
| C1QTNF12 | C1q and TNF related 12 | **0,967** | 0,840861 | 0,166 | 0,192 |
| MED24 | mediator complex subunit 24 | **0,967** | 0,219197 | 24,704 | 23,369 |
| EXTL2 | exostosin like glycosyltransferase 2 | **0,967** | 0,347674 | 20,605 | 18,133 |
| ISG20 | interferon stimulated exonuclease gene 20 | **0,967** | 0,348883 | 47,691 | 80,004 |
| PIP5K1A | phosphatidylinositol-4-phosphate 5-kinase type 1 alpha | **0,967** | 0,28181 | 43,094 | 42,524 |
| MIOS | meiosis regulator for oocyte development | **0,967** | 0,37292 | 17,116 | 14,111 |
| ROM1 | retinal outer segment membrane protein 1 | **0,967** | 0,676305 | 2,382 | 1,596 |
| PIGC | phosphatidylinositol glycan anchor biosynthesis class C | **0,966** | 0,415211 | 16,451 | 16,154 |
| FAM129B | family with sequence similarity 129 member B | **0,966** | 0,229544 | 649,837 | 725,398 |
| ALKBH5 | alkB homolog 5, RNA demethylase | **0,966** | 0,17832 | 84,525 | 78,599 |
| SEM1 | SEM1, 26S proteasome complex subunit | **0,966** | 0,354284 | 59,378 | 57,720 |
| SERBP1 | SERPINE1 mRNA binding protein 1 | **0,966** | 0,23861 | 129,668 | 144,939 |
| CCDC22 | coiled-coil domain containing 22 | **0,966** | 0,421973 | 14,291 | 13,919 |
| STAT2 | signal transducer and activator of transcription 2 | **0,966** | 0,303195 | 53,341 | 43,801 |
| FH | fumarate hydratase | **0,966** | 0,311502 | 26,200 | 27,583 |
| ZFAS1 | ZNFX1 antisense RNA 1 | **0,966** | 0,291058 | 26,476 | 28,988 |
| CDK4 | cyclin dependent kinase 4 | **0,966** | 0,259004 | 38,884 | 38,118 |
| PMPCA | peptidase, mitochondrial processing alpha subunit | **0,966** | 0,305696 | 23,264 | 22,667 |
| ALG13 | ALG13, UDP-N-acetylglucosaminyltransferase subunit | **0,966** | 0,339823 | 19,054 | 17,750 |

| | | | | | |
|---|---|---|---|---|---|
| GCC2 | GRIP and coiled-coil domain containing 2 | **0,966** | 0,26145 | 60,209 | 61,871 |
| CAPS | calcyphosine | **0,966** | 0,416725 | 21,658 | 15,707 |
| MSRB1 | methionine sulfoxide reductase B1 | **0,966** | 0,405059 | 13,571 | 8,492 |
| CCDC43 | coiled-coil domain containing 43 | **0,966** | 0,366235 | 11,798 | 11,557 |
| ZNF443 | zinc finger protein 443 | **0,966** | 0,748076 | 1,052 | 1,085 |
| C1GALT1C1 | C1GALT1 specific chaperone 1 | **0,966** | 0,262644 | 23,762 | 18,006 |
| BAIAP2-AS1 | BAIAP2 antisense RNA 1 (head to head) | **0,966** | 0,52649 | 8,862 | 5,044 |
| MBD4 | methyl-CpG binding domain 4, DNA glycosylase | **0,966** | 0,290589 | 22,322 | 25,987 |
| FZD6 | frizzled class receptor 6 | **0,966** | 0,304818 | 17,780 | 13,855 |
| STYXL1 | serine/threonine/tyrosine interacting like 1 | **0,966** | 0,361465 | 18,666 | 23,050 |
| ELL | elongation factor for RNA polymerase II | **0,966** | 0,282448 | 21,658 | 24,327 |
| FIGNL1 | fidgetin like 1 | **0,966** | 0,512606 | 11,300 | 9,514 |
| SCRN3 | secernin 3 | **0,966** | 0,367588 | 10,081 | 9,322 |
| GDPD1 | glycerophosphodiester phosphodiesterase domain containing 1 | **0,966** | 0,784188 | 0,665 | 0,894 |
| ATP5E | ATP synthase, H+ transporting, mitochondrial F1 complex, epsilon subunit | **0,966** | 0,307732 | 58,381 | 53,570 |
| RNF2 | ring finger protein 2 | **0,966** | 0,343733 | 13,072 | 11,110 |
| CNDP2 | carnosine dipeptidase 2 | **0,966** | 0,203988 | 49,408 | 50,441 |
| CEP83 | centrosomal protein 83 | **0,966** | 0,600304 | 3,490 | 3,831 |
| AAMP | angio associated migratory cell protein | **0,966** | 0,273761 | 70,069 | 72,278 |
| SETD9 | SET domain containing 9 | **0,966** | 0,617031 | 3,490 | 4,533 |
| PTGES2 | prostaglandin E synthase 2 | **0,966** | 0,217474 | 51,845 | 66,915 |
| TRNT1 | tRNA nucleotidyl transferase 1 | **0,966** | 0,382345 | 16,617 | 18,325 |
| NCBP1 | nuclear cap binding protein subunit 1 | **0,966** | 0,339494 | 24,981 | 30,137 |
| THYN1 | thymocyte nuclear protein 1 | **0,966** | 0,468312 | 9,638 | 11,238 |
| TMEM109 | transmembrane protein 109 | **0,966** | 0,230807 | 54,005 | 55,422 |
| ZNF713 | zinc finger protein 713 | **0,966** | 0,721908 | 1,163 | 1,596 |
| SH3BP4 | SH3 domain binding protein 4 | **0,966** | 0,365436 | 16,949 | 11,940 |
| CNOT10 | CCR4-NOT transcription complex subunit 10 | **0,966** | 0,335372 | 13,626 | 14,047 |
| LCE3D | late cornified envelope 3D | **0,966** | 0,784486 | 0,499 | 1,724 |
| SNX9 | sorting nexin 9 | **0,966** | 0,247301 | 100,755 | 93,476 |
| MAX | MYC associated factor X | **0,966** | 0,241543 | 37,444 | 34,926 |
| DNMBP | dynamin binding protein | **0,966** | 0,359489 | 26,587 | 29,243 |
| ZNF790 | zinc finger protein 790 | **0,966** | 0,615411 | 2,714 | 3,192 |

| | | | | | |
|---|---|---|---|---|---|
| SPATA2 | spermatogenesis associated 2 | **0,966** | 0,267569 | 15,288 | 16,601 |
| EIF4A1 | eukaryotic translation initiation factor 4A1 | **0,966** | 0,68022 | 1,717 | 2,490 |
| FANCI | Fanconi anemia complementation group I | **0,966** | 0,582713 | 8,032 | 2,043 |
| RBM17 | RNA binding motif protein 17 | **0,965** | 0,226824 | 30,686 | 31,031 |
| MIR17HG | miR-17-92a-1 cluster host gene | **0,965** | 0,835315 | 0,332 | 0,702 |
| ARID5B | AT-rich interaction domain 5B | **0,965** | 0,348606 | 30,852 | 27,200 |
| ENAH | enabled homolog (Drosophila) | **0,965** | 0,291923 | 55,446 | 35,373 |
| PAAF1 | proteasomal ATPase associated factor 1 | **0,965** | 0,431426 | 7,035 | 8,684 |
| USF1 | upstream transcription factor 1 | **0,965** | 0,336519 | 18,500 | 17,750 |
| TMEM127 | transmembrane protein 127 | **0,965** | 0,219755 | 89,898 | 79,302 |
| SH2D5 | SH2 domain containing 5 | **0,965** | 0,61683 | 2,326 | 3,320 |
| XRN2 | 5'-3' exoribonuclease 2 | **0,965** | 0,235679 | 60,431 | 55,102 |
| ACBD3 | acyl-CoA binding domain containing 3 | **0,965** | 0,184406 | 73,503 | 61,743 |
| TTLL11 | tubulin tyrosine ligase like 11 | **0,965** | 0,642586 | 2,548 | 1,852 |
| IQCB1 | IQ motif containing B1 | **0,965** | 0,289349 | 16,617 | 15,388 |
| NCBP2 | nuclear cap binding protein subunit 2 | **0,965** | 0,17872 | 48,134 | 53,953 |
| CAT | catalase | **0,965** | 0,351946 | 23,042 | 9,258 |
| JUN | Jun proto-oncogene, AP-1 transcription factor subunit | **0,965** | 0,214784 | 240,726 | 315,865 |
| NUTM2B-AS1 | NUTM2B antisense RNA 1 | **0,965** | 0,497601 | 5,484 | 6,193 |
| OTUD6B | OTU domain containing 6B | **0,965** | 0,532231 | 10,192 | 9,705 |
| TNFRSF1A | TNF receptor superfamily member 1A | **0,965** | 0,168008 | 148,169 | 136,511 |
| GPM6B | glycoprotein M6B | **0,965** | 0,805961 | 0,388 | 0,319 |
| TUFM | Tu translation elongation factor, mitochondrial | **0,965** | 0,230138 | 71,398 | 77,003 |
| YAE1D1 | Yae1 domain containing 1 | **0,965** | 0,445585 | 7,367 | 10,152 |
| ZNF143 | zinc finger protein 143 | **0,965** | 0,296929 | 19,331 | 18,836 |
| FAM3C | family with sequence similarity 3 member C | **0,965** | 0,185004 | 36,004 | 34,926 |
| UGGT1 | UDP-glucose glycoprotein glucosyltransferase 1 | **0,965** | 0,345689 | 121,083 | 113,653 |
| DNTTIP2 | deoxynucleotidyltransferase terminal interacting protein 2 | **0,965** | 0,173975 | 65,083 | 60,785 |
| GOLT1B | golgi transport 1B | **0,965** | 0,235617 | 54,836 | 45,206 |
| SMG8 | SMG8, nonsense mediated mRNA decay factor | **0,965** | 0,316363 | 18,113 | 17,239 |
| RNPEP | arginyl aminopeptidase | **0,965** | 0,250478 | 33,677 | 31,542 |
| RPL23P2 | ribosomal protein L23 pseudogene 2 | **0,965** | 0,848424 | 0,111 | 0,447 |
| ZNF653 | zinc finger protein 653 | **0,965** | 0,55492 | 4,542 | 3,001 |

| | | | | | |
|---|---|---|---|---|---|
| RAPGEF2 | Rap guanine nucleotide exchange factor 2 | **0,965** | 0,41415 | 93,554 | 86,070 |
| FAM13A | family with sequence similarity 13 member A | **0,965** | 0,30983 | 17,060 | 17,495 |
| TMEM184A | transmembrane protein 184A | **0,965** | 0,819447 | 1,163 | 1,341 |
| PDCD6 | programmed cell death 6 | **0,965** | 0,188484 | 61,040 | 52,932 |
| TXNRD1 | thioredoxin reductase 1 | **0,965** | 0,261291 | 200,512 | 135,745 |
| DDX19A | DEAD-box helicase 19A | **0,965** | 0,518306 | 8,087 | 7,726 |
| PYGB | glycogen phosphorylase B | **0,965** | 0,182747 | 395,652 | 287,133 |
| ZP3 | zona pellucida glycoprotein 3 | **0,965** | 0,567344 | 4,542 | 3,384 |
| RPS24 | ribosomal protein S24 | **0,965** | 0,333003 | 180,738 | 191,039 |
| RPS14 | ribosomal protein S14 | **0,965** | 0,318985 | 274,126 | 294,923 |
| CPSF4 | cleavage and polyadenylation specific factor 4 | **0,965** | 0,384526 | 12,130 | 13,728 |
| TMEM14A | transmembrane protein 14A | **0,965** | 0,509825 | 6,591 | 5,491 |
| ELOA | elongin A | **0,965** | 0,16078 | 74,278 | 63,275 |
| RPS16 | ribosomal protein S16 | **0,965** | 0,285639 | 241,224 | 237,841 |
| ALDH1A3 | aldehyde dehydrogenase 1 family member A3 | **0,965** | 0,29357 | 62,037 | 97,626 |
| ZNF594 | zinc finger protein 594 | **0,965** | 0,46099 | 7,256 | 6,960 |
| ARHGEF1 | Rho guanine nucleotide exchange factor 1 | **0,965** | 0,163952 | 50,017 | 45,142 |
| TUB | tubby bipartite transcription factor | **0,964** | 0,608182 | 3,877 | 3,001 |
| TEF | TEF, PAR bZIP transcription factor | **0,964** | 0,171443 | 39,881 | 24,263 |
| FARP1 | FERM, ARH/RhoGEF and pleckstrin domain protein 1 | **0,964** | 0,274466 | 77,380 | 71,065 |
| DBT | dihydrolipoamide branched chain transacylase E2 | **0,964** | 0,349496 | 10,856 | 13,728 |
| CLSTN1 | calsyntenin 1 | **0,964** | 0,18773 | 255,293 | 211,215 |
| TBC1D17 | TBC1 domain family member 17 | **0,964** | 0,201093 | 58,991 | 49,164 |
| FOXN2 | forkhead box N2 | **0,964** | 0,449557 | 23,762 | 22,731 |
| MAN1A1 | mannosidase alpha class 1A member 1 | **0,964** | 0,201672 | 571,183 | 207,576 |
| CNOT2 | CCR4-NOT transcription complex subunit 2 | **0,964** | 0,325712 | 35,394 | 34,670 |
| ELL2 | elongation factor for RNA polymerase II 2 | **0,964** | 0,203845 | 257,564 | 195,508 |
| GATB | glutamyl-tRNA amidotransferase subunit B | **0,964** | 0,384644 | 7,921 | 8,364 |
| MAP2K3 | mitogen-activated protein kinase kinase 3 | **0,964** | 0,194397 | 132,216 | 165,499 |
| TPI1 | triosephosphate isomerase 1 | **0,964** | 0,279578 | 321,152 | 236,947 |
| NGDN | neuroguidin | **0,964** | 0,335987 | 20,993 | 27,072 |
| NDUFB5 | NADH:ubiquinone oxidoreductase subunit B5 | **0,964** | 0,30268 | 36,558 | 34,990 |
| AGTRAP | angiotensin II receptor associated protein | **0,964** | 0,180675 | 91,449 | 92,902 |

| | | | | | |
|---|---|---|---|---|---|
| DCAF4 | DDB1 and CUL4 associated factor 4 | **0,964** | 0,457697 | 8,142 | 7,726 |
| PTCD2 | pentatricopeptide repeat domain 2 | **0,964** | 0,407358 | 9,527 | 12,387 |
| TSNAX | translin associated factor X | **0,964** | 0,36301 | 24,926 | 22,731 |
| PIFO | primary cilia formation | **0,964** | 0,696308 | 2,659 | 2,171 |
| FASTKD2 | FAST kinase domains 2 | **0,964** | 0,312956 | 21,824 | 27,966 |
| EXT2 | exostosin glycosyltransferase 2 | **0,964** | 0,152252 | 288,583 | 201,319 |
| LRRC47 | leucine rich repeat containing 47 | **0,964** | 0,237918 | 36,502 | 34,224 |
| RNF115 | ring finger protein 115 | **0,964** | 0,245459 | 26,975 | 27,966 |
| RPL5 | ribosomal protein L5 | **0,964** | 0,24677 | 271,024 | 299,647 |
| MSANTD3 | Myb/SANT DNA binding domain containing 3 | **0,964** | 0,220112 | 47,248 | 34,798 |
| TATDN2 | TatD DNase domain containing 2 | **0,964** | 0,423376 | 12,407 | 12,131 |
| NSMCE4A | NSE4 homolog A, SMC5-SMC6 complex component | **0,964** | 0,329244 | 10,413 | 11,301 |
| YIPF6 | Yip1 domain family member 6 | **0,964** | 0,263515 | 64,973 | 56,443 |
| SMAD2 | SMAD family member 2 | **0,964** | 0,212424 | 50,017 | 46,100 |
| PPIC | peptidylprolyl isomerase C | **0,964** | 0,289989 | 26,753 | 17,878 |
| HEATR5A | HEAT repeat containing 5A | **0,964** | 0,245832 | 19,442 | 17,814 |
| ATP6V1F | ATPase H+ transporting V1 subunit F | **0,964** | 0,284238 | 44,201 | 37,608 |
| LINC01521 | long intergenic non-protein coding RNA 1521 | **0,964** | 0,826604 | 0,997 | 1,022 |
| PSMD9 | proteasome 26S subunit, non-ATPase 9 | **0,964** | 0,466315 | 7,090 | 5,300 |
| ZCCHC11 | zinc finger CCHC-type containing 11 | **0,964** | 0,353641 | 23,264 | 24,646 |
| WDR41 | WD repeat domain 41 | **0,964** | 0,2827 | 20,605 | 19,283 |
| VPS9D1 | VPS9 domain containing 1 | **0,964** | 0,47787 | 7,367 | 5,874 |
| CEP63 | centrosomal protein 63 | **0,964** | 0,231256 | 37,333 | 33,585 |
| DARS | aspartyl-tRNA synthetase | **0,964** | 0,1961 | 78,709 | 63,722 |
| LAMC3 | laminin subunit gamma 3 | **0,964** | 0,405357 | 8,530 | 2,554 |
| XRCC4 | X-ray repair cross complementing 4 | **0,964** | 0,548923 | 4,154 | 3,831 |
| ATP6V0A4 | ATPase H+ transporting V0 subunit a4 | **0,964** | 0,822569 | 0,554 | 0,447 |
| UNC45A | unc-45 myosin chaperone A | **0,964** | 0,270926 | 36,391 | 26,051 |
| MPP2 | membrane palmitoylated protein 2 | **0,964** | 0,667839 | 2,216 | 0,894 |
| MTCH1 | mitochondrial carrier 1 | **0,964** | 0,215821 | 91,505 | 82,813 |
| THAP10 | THAP domain containing 10 | **0,964** | 0,463204 | 4,653 | 5,746 |
| SERPINA5 | serpin family A member 5 | **0,964** | 0,16857 | 125,957 | 64,488 |
| NUDT9 | nudix hydrolase 9 | **0,964** | 0,271115 | 31,572 | 31,414 |

| | | | | | |
|---|---|---|---|---|---|
| POLR2C | RNA polymerase II subunit C | **0,964** | 0,174312 | 54,892 | 59,189 |
| TOR3A | torsin family 3 member A | **0,963** | 0,388516 | 16,340 | 18,197 |
| ANKLE2 | ankyrin repeat and LEM domain containing 2 | **0,963** | 0,176893 | 76,549 | 56,188 |
| RGS5 | regulator of G-protein signaling 5 | **0,963** | 0,806131 | 0,775 | 0,894 |
| C12orf57 | chromosome 12 open reading frame 57 | **0,963** | 0,211934 | 63,145 | 60,210 |
| CCDC167 | coiled-coil domain containing 167 | **0,963** | 0,471095 | 5,041 | 4,661 |
| MRPS23 | mitochondrial ribosomal protein S23 | **0,963** | 0,272393 | 19,331 | 20,432 |
| TMEM260 | transmembrane protein 260 | **0,963** | 0,45781 | 11,632 | 14,558 |
| ALDH16A1 | aldehyde dehydrogenase 16 family member A1 | **0,963** | 0,357658 | 10,247 | 8,556 |
| PTPN3 | protein tyrosine phosphatase, non-receptor type 3 | **0,963** | 0,464901 | 9,029 | 6,130 |
| EIF3J | eukaryotic translation initiation factor 3 subunit J | **0,963** | 0,205914 | 54,338 | 58,167 |
| LYRM2 | LYR motif containing 2 | **0,963** | 0,273378 | 28,194 | 30,265 |
| EXOSC6 | exosome component 6 | **0,963** | 0,298177 | 19,497 | 22,603 |
| DHX33 | DEAH-box helicase 33 | **0,963** | 0,375768 | 15,343 | 15,643 |
| GDI2 | GDP dissociation inhibitor 2 | **0,963** | 0,171083 | 144,734 | 116,015 |
| SLX4 | SLX4 structure-specific endonuclease subunit | **0,963** | 0,321939 | 11,687 | 13,664 |
| LYZ | lysozyme | **0,963** | 0,588235 | 2,936 | 2,682 |
| DDX42 | DEAD-box helicase 42 | **0,963** | 0,142565 | 75,885 | 80,642 |
| SLC6A15 | solute carrier family 6 member 15 | **0,963** | 0,852856 | 0,886 | 0,255 |
| TBCB | tubulin folding cofactor B | **0,963** | 0,358504 | 47,857 | 44,567 |
| FBXO46 | F-box protein 46 | **0,963** | 0,357707 | 7,865 | 7,151 |
| ZNF22 | zinc finger protein 22 | **0,963** | 0,207303 | 20,273 | 19,027 |
| CHMP7 | charged multivesicular body protein 7 | **0,963** | 0,215781 | 35,117 | 32,947 |
| NYAP1 | neuronal tyrosine phosphorylated phosphoinositide-3-kinase adaptor 1 | **0,963** | 0,486379 | 5,262 | 3,959 |
| TAF10 | TATA-box binding protein associated factor 10 | **0,963** | 0,563936 | 5,871 | 7,407 |
| SAFB | scaffold attachment factor B | **0,963** | 0,181102 | 67,521 | 66,659 |
| FIZ1 | FLT3 interacting zinc finger 1 | **0,963** | 0,384746 | 13,958 | 17,303 |
| TIMM29 | translocase of inner mitochondrial membrane 29 | **0,963** | 0,322199 | 12,352 | 10,599 |
| ZNF337-AS1 | ZNF337 antisense RNA 1 | **0,963** | 0,619826 | 1,662 | 3,001 |
| TSPAN1 | tetraspanin 1 | **0,963** | 0,450188 | 7,644 | 11,748 |
| NUP155 | nucleoporin 155 | **0,963** | 0,225651 | 21,824 | 22,156 |
| ANO8 | anoctamin 8 | **0,963** | 0,656192 | 3,157 | 3,831 |
| CTDP1 | CTD phosphatase subunit 1 | **0,963** | 0,41138 | 8,862 | 8,109 |

| | | | | | |
|---|---|---|---|---|---|
| LDHB | lactate dehydrogenase B | **0,963** | 0,347857 | 57,273 | 46,163 |
| STAC2 | SH3 and cysteine rich domain 2 | **0,963** | 0,730157 | 0,665 | 0,830 |
| HGSNAT | heparan-alpha-glucosaminide N-acetyltransferase | **0,963** | 0,247574 | 42,817 | 25,476 |
| ADIPOR1 | adiponectin receptor 1 | **0,963** | 0,146962 | 119,698 | 101,649 |
| ERP29 | endoplasmic reticulum protein 29 | **0,963** | 0,258565 | 74,998 | 55,869 |
| DOLK | dolichol kinase | **0,963** | 0,317119 | 21,713 | 18,325 |
| TMEM241 | transmembrane protein 241 | **0,963** | 0,509031 | 4,320 | 3,959 |
| SLC35A5 | solute carrier family 35 member A5 | **0,963** | 0,229688 | 62,148 | 55,422 |
| STAM2 | signal transducing adaptor molecule 2 | **0,963** | 0,237766 | 51,236 | 50,186 |
| MUM1L1 | MUM1 like 1 | **0,963** | 0,830672 | 1,052 | 0,511 |
| SNRPD1 | small nuclear ribonucleoprotein D1 polypeptide | **0,963** | 0,211219 | 32,182 | 41,630 |
| AVPR1A | arginine vasopressin receptor 1A | **0,963** | 0,880742 | 0,166 | 0,958 |
| ERF | ETS2 repressor factor | **0,963** | 0,21176 | 44,478 | 40,992 |
| USP19 | ubiquitin specific peptidase 19 | **0,963** | 0,21858 | 36,945 | 36,586 |
| ALG10 | ALG10, alpha-1,2-glucosyltransferase | **0,963** | 0,631253 | 2,603 | 3,576 |
| VAV2 | vav guanine nucleotide exchange factor 2 | **0,963** | 0,222192 | 19,331 | 17,559 |
| MBNL2 | muscleblind like splicing regulator 2 | **0,963** | 0,359053 | 46,472 | 36,458 |
| DNM1L | dynamin 1 like | **0,962** | 0,157932 | 52,122 | 39,778 |
| ANAPC5 | anaphase promoting complex subunit 5 | **0,962** | 0,154778 | 60,818 | 64,935 |
| NARFL | nuclear prelamin A recognition factor like | **0,962** | 0,365807 | 12,020 | 10,152 |
| EIF4ENIF1 | eukaryotic translation initiation factor 4E nuclear import factor 1 | **0,962** | 0,332802 | 15,731 | 10,024 |
| CHTOP | chromatin target of PRMT1 | **0,962** | 0,170738 | 80,039 | 80,068 |
| BCDIN3D-AS1 | BCDIN3D antisense RNA 1 | **0,962** | 0,731995 | 1,828 | 1,724 |
| VPS36 | vacuolar protein sorting 36 homolog | **0,962** | 0,260361 | 17,226 | 21,773 |
| SELENOF | selenoprotein F | **0,962** | 0,187229 | 94,717 | 85,048 |
| DNAJC24 | DnaJ heat shock protein family (Hsp40) member C24 | **0,962** | 0,461566 | 10,690 | 10,280 |
| PDIA3 | protein disulfide isomerase family A member 3 | **0,962** | 0,12644 | 460,181 | 308,842 |
| MIDN | midnolin | **0,962** | 0,228636 | 85,079 | 109,439 |
| SNX27 | sorting nexin family member 27 | **0,962** | 0,32948 | 18,833 | 18,070 |
| PNO1 | partner of NOB1 homolog | **0,962** | 0,173525 | 26,310 | 37,863 |
| ZMYND8 | zinc finger MYND-type containing 8 | **0,962** | 0,261579 | 25,203 | 34,160 |
| NECTIN2 | nectin cell adhesion molecule 2 | **0,962** | 0,24183 | 32,016 | 20,432 |
| ADIRF | adipogenesis regulatory factor | **0,962** | 0,28314 | 25,313 | 40,481 |

| | | | | | |
|---|---|---|---|---|---|
| AIFM2 | apoptosis inducing factor, mitochondria associated 2 | **0,962** | 0,171786 | 43,980 | 39,332 |
| COX7C | cytochrome c oxidase subunit 7C | **0,962** | 0,215124 | 68,351 | 61,424 |
| RAD1 | RAD1 checkpoint DNA exonuclease | **0,962** | 0,201889 | 17,393 | 16,218 |
| PCDHGA12 | protocadherin gamma subfamily A, 12 | **0,962** | 0,716464 | 1,883 | 2,235 |
| GPR135 | G protein-coupled receptor 135 | **0,962** | 0,696055 | 3,434 | 2,299 |
| TIE1 | tyrosine kinase with immunoglobulin like and EGF like domains 1 | **0,962** | 0,658196 | 1,274 | 0,766 |
| EHMT1 | euchromatic histone lysine methyltransferase 1 | **0,962** | 0,263641 | 25,756 | 26,945 |
| ATL3 | atlastin GTPase 3 | **0,962** | 0,15386 | 121,526 | 91,241 |
| GANC | glucosidase alpha, neutral C | **0,962** | 0,4032 | 8,752 | 8,109 |
| ASH2L | ASH2 like histone lysine methyltransferase complex subunit | **0,962** | 0,223136 | 27,917 | 31,286 |
| THUMPD3 | THUMP domain containing 3 | **0,962** | 0,166755 | 29,246 | 27,839 |
| PLOD1 | procollagen-lysine,2-oxoglutarate 5-dioxygenase 1 | **0,962** | 0,17116 | 158,416 | 85,431 |
| TMEM38A | transmembrane protein 38A | **0,962** | 0,66219 | 1,606 | 1,277 |
| RPL32 | ribosomal protein L32 | **0,962** | 0,23466 | 209,541 | 202,787 |
| ENTPD5 | ectonucleoside triphosphate diphosphohydrolase 5 | **0,962** | 0,232958 | 32,348 | 29,179 |
| AMACR | alpha-methylacyl-CoA racemase | **0,962** | 0,753978 | 1,163 | 0,894 |
| TMEM39B | transmembrane protein 39B | **0,962** | 0,305555 | 10,580 | 10,471 |
| MAPRE1 | microtubule associated protein RP/EB family member 1 | **0,962** | 0,141026 | 81,811 | 70,618 |
| POLR2F | RNA polymerase II subunit F | **0,962** | 0,330109 | 28,747 | 29,499 |
| SLC7A5 | solute carrier family 7 member 5 | **0,962** | 0,217662 | 298,941 | 222,070 |
| SPINT2 | serine peptidase inhibitor, Kunitz type 2 | **0,961** | 0,232639 | 34,840 | 16,729 |
| CYB5D1 | cytochrome b5 domain containing 1 | **0,961** | 0,587435 | 3,102 | 2,873 |
| NPDC1 | neural proliferation, differentiation and control 1 | **0,961** | 0,381386 | 24,039 | 17,686 |
| FBXL2 | F-box and leucine rich repeat protein 2 | **0,961** | 0,532649 | 4,431 | 3,001 |
| MOCOS | molybdenum cofactor sulfurase | **0,961** | 0,418239 | 7,976 | 8,428 |
| HEBP2 | heme binding protein 2 | **0,961** | 0,312339 | 13,072 | 15,388 |
| BRD7 | bromodomain containing 7 | **0,961** | 0,176223 | 29,966 | 29,052 |
| REPS2 | RALBP1 associated Eps domain containing 2 | **0,961** | 0,774485 | 0,831 | 0,383 |
| FCGRT | Fc fragment of IgG receptor and transporter | **0,961** | 0,214463 | 46,085 | 25,348 |
| MEIS2 | Meis homeobox 2 | **0,961** | 0,50936 | 7,810 | 5,683 |
| FRMD6-AS1 | FRMD6 antisense RNA 1 | **0,961** | 0,636081 | 1,828 | 3,001 |
| ACSL4 | acyl-CoA synthetase long-chain family member 4 | **0,961** | 0,112534 | 499,342 | 433,349 |
| LYNX1 | Ly6/neurotoxin 1 | **0,961** | 0,24308 | 30,963 | 28,349 |

| | | | | | |
|---|---|---|---|---|---|
| PRMT9 | protein arginine methyltransferase 9 | **0,961** | 0,478119 | 7,035 | 6,960 |
| SMG6 | SMG6, nonsense mediated mRNA decay factor | **0,961** | 0,209292 | 36,281 | 34,287 |
| MRPS14 | mitochondrial ribosomal protein S14 | **0,961** | 0,363973 | 12,297 | 12,770 |
| PRDM8 | PR/SET domain 8 | **0,961** | 0,43728 | 6,370 | 4,725 |
| ABCA7 | ATP binding cassette subfamily A member 7 | **0,961** | 0,506925 | 5,207 | 5,938 |
| PCNX4 | pecanex homolog 4 (Drosophila) | **0,961** | 0,195502 | 66,745 | 60,785 |
| ZNF426 | zinc finger protein 426 | **0,961** | 0,356951 | 9,970 | 9,641 |
| ULK4 | unc-51 like kinase 4 | **0,961** | 0,527948 | 2,770 | 5,427 |
| ZFYVE28 | zinc finger FYVE-type containing 28 | **0,961** | 0,474612 | 5,041 | 4,150 |
| RPS6KC1 | ribosomal protein S6 kinase C1 | **0,961** | 0,208876 | 23,652 | 19,155 |
| SRSF7 | serine and arginine rich splicing factor 7 | **0,961** | 0,162669 | 23,873 | 30,520 |
| GRK6 | G protein-coupled receptor kinase 6 | **0,961** | 0,301403 | 22,322 | 20,751 |
| ABCD4 | ATP binding cassette subfamily D member 4 | **0,961** | 0,26919 | 16,839 | 13,089 |
| PTPN4 | protein tyrosine phosphatase, non-receptor type 4 | **0,961** | 0,389394 | 11,244 | 11,493 |
| FLG-AS1 | FLG antisense RNA 1 | **0,961** | 0,700581 | 2,437 | 1,213 |
| CST3 | cystatin C | **0,961** | 0,229217 | 257,398 | 149,217 |
| TAF12 | TATA-box binding protein associated factor 12 | **0,961** | 0,194518 | 23,818 | 26,242 |
| SPATA5 | spermatogenesis associated 5 | **0,961** | 0,511549 | 5,705 | 6,321 |
| DGCR6L | DiGeorge syndrome critical region gene 6 like | **0,961** | 0,364763 | 16,506 | 18,006 |
| TMED4 | transmembrane p24 trafficking protein 4 | **0,961** | 0,118365 | 85,024 | 67,617 |
| ACYP2 | acylphosphatase 2 | **0,961** | 0,48202 | 8,198 | 6,704 |
| ANP32B | acidic nuclear phosphoprotein 32 family member B | **0,961** | 0,201548 | 132,493 | 120,357 |
| PHF19 | PHD finger protein 19 | **0,961** | 0,23085 | 24,759 | 19,283 |
| THOC6 | THO complex 6 | **0,961** | 0,319303 | 11,798 | 13,153 |
| CRIPT | CXXC repeat containing interactor of PDZ3 domain | **0,961** | 0,204213 | 19,608 | 20,943 |
| TNPO2 | transportin 2 | **0,961** | 0,129854 | 43,038 | 34,798 |
| PAK4 | p21 (RAC1) activated kinase 4 | **0,961** | 0,202547 | 41,266 | 38,565 |
| SMARCC1 | SWI/SNF related, matrix associated, actin dependent regulator of chromatin subfamily c m | **0,961** | 0,202014 | 28,249 | 25,348 |
| PPARA | peroxisome proliferator activated receptor alpha | **0,961** | 0,356624 | 38,496 | 24,455 |
| NENF | neudesin neurotrophic factor | **0,961** | 0,281859 | 32,403 | 26,498 |
| FEZ2 | fasciculation and elongation protein zeta 2 | **0,961** | 0,226426 | 26,144 | 25,668 |
| GTPBP3 | GTP binding protein 3 (mitochondrial) | **0,961** | 0,378238 | 6,259 | 6,321 |
| RHOJ | ras homolog family member J | **0,961** | 0,593311 | 2,991 | 3,767 |

| | | | | | |
|---|---|---|---|---|---|
| MED15 | mediator complex subunit 15 | **0,961** | 0,141497 | 40,878 | 39,268 |
| RNPS1 | RNA binding protein with serine rich domain 1 | **0,961** | 0,145821 | 65,804 | 68,639 |
| ERCC5 | ERCC excision repair 5, endonuclease | **0,961** | 0,711725 | 1,329 | 1,660 |
| RGS14 | regulator of G protein signaling 14 | **0,960** | 0,805015 | 0,665 | 0,383 |
| MTMR2 | myotubularin related protein 2 | **0,960** | 0,168778 | 46,472 | 48,845 |
| NDUFC1 | NADH:ubiquinone oxidoreductase subunit C1 | **0,960** | 0,300402 | 20,328 | 21,964 |
| TMEM167A | transmembrane protein 167A | **0,960** | 0,190135 | 86,907 | 74,257 |
| TIGAR | TP53 induced glycolysis regulatory phosphatase | **0,960** | 0,227174 | 13,958 | 16,729 |
| UQCR10 | ubiquinol-cytochrome c reductase, complex III subunit X | **0,960** | 0,356292 | 21,990 | 20,432 |
| SRRD | SRR1 domain containing | **0,960** | 0,328723 | 16,451 | 14,685 |
| ZNF391 | zinc finger protein 391 | **0,960** | 0,66291 | 2,216 | 1,788 |
| ZNF142 | zinc finger protein 142 | **0,960** | 0,259276 | 23,984 | 22,858 |
| ATN1 | atrophin 1 | **0,960** | 0,182487 | 116,762 | 90,731 |
| TCTN3 | tectonic family member 3 | **0,960** | 0,189733 | 43,204 | 36,714 |
| RPL13A | ribosomal protein L13a | **0,960** | 0,238351 | 541,162 | 549,684 |
| INVS | inversin | **0,960** | 0,360526 | 8,918 | 9,769 |
| TRIM37 | tripartite motif containing 37 | **0,960** | 0,286496 | 27,917 | 24,455 |
| THOC1 | THO complex 1 | **0,960** | 0,254495 | 10,967 | 11,940 |
| CHADL | chondroadherin like | **0,960** | 0,436922 | 9,970 | 6,768 |
| CERS2 | ceramide synthase 2 | **0,960** | 0,082052 | 171,765 | 133,382 |
| SLC25A17 | solute carrier family 25 member 17 | **0,960** | 0,2461 | 14,623 | 14,239 |
| TESK1 | testis-specific kinase 1 | **0,960** | 0,247176 | 27,474 | 23,305 |
| GALNT16 | polypeptide N-acetylgalactosaminyltransferase 16 | **0,960** | 0,562682 | 7,201 | 2,426 |
| NOC3L | NOC3 like DNA replication regulator | **0,960** | 0,255494 | 13,903 | 16,218 |
| ZNF793 | zinc finger protein 793 | **0,960** | 0,656002 | 1,828 | 2,299 |
| TTC38 | tetratricopeptide repeat domain 38 | **0,960** | 0,341173 | 11,133 | 7,534 |
| PCSK1 | proprotein convertase subtilisin/kexin type 1 | **0,960** | 0,545098 | 5,373 | 5,427 |
| MTG2 | mitochondrial ribosome associated GTPase 2 | **0,960** | 0,212639 | 18,556 | 23,050 |
| TMCO1 | transmembrane and coiled-coil domains 1 | **0,960** | 0,172379 | 72,007 | 52,612 |
| SRD5A3 | steroid 5 alpha-reductase 3 | **0,960** | 0,240913 | 16,562 | 11,940 |
| RNPEPL1 | arginyl aminopeptidase like 1 | **0,960** | 0,281373 | 31,295 | 24,327 |
| MRPS25 | mitochondrial ribosomal protein S25 | **0,960** | 0,351794 | 14,291 | 14,111 |
| CBWD5 | COBW domain containing 5 | **0,960** | 0,78893 | 0,720 | 0,511 |

| | | | | | |
|---|---|---|---|---|---|
| SLC9A3-AS1 | SLC9A3 antisense RNA 1 | **0,960** | 0,3369 | 20,661 | 13,600 |
| EIF2D | eukaryotic translation initiation factor 2D | **0,960** | 0,223847 | 14,291 | 12,642 |
| TMPO | thymopoietin | **0,960** | 0,216567 | 26,200 | 22,986 |
| SALL2 | spalt like transcription factor 2 | **0,960** | 0,763347 | 1,551 | 0,511 |
| UBA52 | ubiquitin A-52 residue ribosomal protein fusion product 1 | **0,960** | 0,206399 | 188,105 | 175,523 |
| RAB5A | RAB5A, member RAS oncogene family | **0,960** | 0,145138 | 70,512 | 52,868 |
| C1D | C1D nuclear receptor corepressor | **0,960** | 0,31391 | 8,918 | 10,535 |
| CNPY3 | canopy FGF signaling regulator 3 | **0,960** | 0,304291 | 41,543 | 26,115 |
| USP40 | ubiquitin specific peptidase 40 | **0,960** | 0,134732 | 33,622 | 30,009 |
| KANK2 | KN motif and ankyrin repeat domains 2 | **0,960** | 0,261322 | 48,079 | 34,734 |
| MTF2 | metal response element binding transcription factor 2 | **0,960** | 0,292844 | 8,973 | 10,216 |
| PDCL | phosducin like | **0,960** | 0,301938 | 16,174 | 16,601 |
| ACTRT3 | actin related protein T3 | **0,960** | 0,691929 | 1,496 | 1,277 |
| CYFIP2 | cytoplasmic FMR1 interacting protein 2 | **0,959** | 0,573412 | 3,213 | 2,490 |
| ZNF665 | zinc finger protein 665 | **0,959** | 0,737252 | 1,052 | 0,702 |
| VMAC | vimentin-type intermediate filament associated coiled-coil protein | **0,959** | 0,557626 | 2,714 | 2,746 |
| PLCD3 | phospholipase C delta 3 | **0,959** | 0,15083 | 29,246 | 23,369 |
| LINC01023 | long intergenic non-protein coding RNA 1023 | **0,959** | 0,777116 | 0,609 | 0,638 |
| ODF2L | outer dense fiber of sperm tails 2 like | **0,959** | 0,445641 | 10,358 | 11,557 |
| CCDC114 | coiled-coil domain containing 114 | **0,959** | 0,829694 | 0,388 | 0,447 |
| MAGI1 | membrane associated guanylate kinase, WW and PDZ domain containing 1 | **0,959** | 0,175226 | 25,147 | 26,625 |
| TCF3 | transcription factor 3 | **0,959** | 0,284463 | 19,165 | 18,644 |
| AFMID | arylformamidase | **0,959** | 0,511595 | 2,437 | 4,278 |
| PSMD5 | proteasome 26S subunit, non-ATPase 5 | **0,959** | 0,14688 | 32,569 | 37,671 |
| TMEM245 | transmembrane protein 245 | **0,959** | 0,144804 | 72,118 | 48,334 |
| NUDT18 | nudix hydrolase 18 | **0,959** | 0,447477 | 6,591 | 4,597 |
| CUTC | cutC copper transporter | **0,959** | 0,370306 | 5,539 | 5,044 |
| CIAPIN1 | cytokine induced apoptosis inhibitor 1 | **0,959** | 0,25298 | 17,282 | 19,793 |
| LRP8 | LDL receptor related protein 8 | **0,959** | 0,22369 | 55,944 | 30,393 |
| RSL1D1 | ribosomal L1 domain containing 1 | **0,959** | 0,153726 | 94,606 | 117,931 |
| ZFP41 | ZFP41 zinc finger protein | **0,959** | 0,446583 | 5,594 | 4,789 |
| CCDC50 | coiled-coil domain containing 50 | **0,959** | 0,161383 | 43,426 | 38,118 |
| ATP11A | ATPase phospholipid transporting 11A | **0,959** | 0,298439 | 18,611 | 18,772 |

| | | | | | |
|---|---|---|---|---|---|
| CLEC3A | C-type lectin domain family 3 member A | **0,959** | 0,495851 | 6,314 | 3,767 |
| CPTP | ceramide-1-phosphate transfer protein | **0,959** | 0,15496 | 21,048 | 18,516 |
| ATP13A2 | ATPase 13A2 | **0,959** | 0,231962 | 31,572 | 39,587 |
| LZTR1 | leucine zipper like transcription regulator 1 | **0,959** | 0,339305 | 10,081 | 9,258 |
| SNHG4 | small nucleolar RNA host gene 4 | **0,959** | 0,734539 | 0,554 | 1,405 |
| SNX14 | sorting nexin 14 | **0,959** | 0,158735 | 32,791 | 24,646 |
| ZRSR2 | zinc finger CCCH-type, RNA binding motif and serine/arginine rich 2 | **0,959** | 0,348851 | 8,530 | 7,981 |
| PRR14 | proline rich 14 | **0,959** | 0,182442 | 18,722 | 14,494 |
| HEXDC | hexosaminidase D | **0,959** | 0,413667 | 5,484 | 6,321 |
| TMBIM4 | transmembrane BAX inhibitor motif containing 4 | **0,959** | 0,324859 | 12,740 | 11,365 |
| ERICH2 | glutamate rich 2 | **0,959** | 0,815143 | 1,052 | 0,702 |
| C11orf95 | chromosome 11 open reading frame 95 | **0,959** | 0,418918 | 15,509 | 9,386 |
| OSBPL2 | oxysterol binding protein like 2 | **0,959** | 0,23456 | 19,110 | 17,942 |
| RRP7BP | ribosomal RNA processing 7 homolog B, pseudogene | **0,959** | 0,587629 | 3,046 | 1,660 |
| SF3B4 | splicing factor 3b subunit 4 | **0,959** | 0,124711 | 44,257 | 42,971 |
| HSPD1 | heat shock protein family D (Hsp60) member 1 | **0,959** | 0,156801 | 105,795 | 88,113 |
| DROSHA | drosha ribonuclease III | **0,959** | 0,182166 | 24,759 | 25,859 |
| PDLIM4 | PDZ and LIM domain 4 | **0,959** | 0,242435 | 25,923 | 22,603 |
| MYL12B | myosin light chain 12B | **0,959** | 0,116041 | 130,444 | 105,544 |
| MINDY4 | MINDY lysine 48 deubiquitinase 4 | **0,959** | 0,807671 | 0,277 | 0,128 |
| C12orf4 | chromosome 12 open reading frame 4 | **0,959** | 0,372899 | 7,201 | 8,875 |
| LYSMD3 | LysM domain containing 3 | **0,959** | 0,200118 | 40,324 | 33,713 |
| DGKZ | diacylglycerol kinase zeta | **0,959** | 0,132691 | 35,505 | 33,968 |
| TXLNA | taxilin alpha | **0,959** | 0,13867 | 77,214 | 64,935 |
| CACYBP | calcyclin binding protein | **0,959** | 0,183275 | 30,354 | 30,329 |
| LSM6 | LSM6 homolog, U6 small nuclear RNA and mRNA degradation associated | **0,959** | 0,450839 | 14,568 | 17,303 |
| NAP1L1 | nucleosome assembly protein 1 like 1 | **0,959** | 0,171011 | 191,373 | 165,116 |
| EIF2S3 | eukaryotic translation initiation factor 2 subunit gamma | **0,959** | 0,142737 | 53,673 | 50,378 |
| TOP2B | topoisomerase (DNA) II beta | **0,959** | 0,112557 | 83,584 | 77,769 |
| OAS2 | 2'-5'-oligoadenylate synthetase 2 | **0,959** | 0,82998 | 0,388 | 0,702 |
| TOR2A | torsin family 2 member A | **0,959** | 0,448482 | 4,265 | 4,214 |
| PLA2G16 | phospholipase A2 group XVI | **0,959** | 0,312983 | 6,425 | 8,875 |
| POLR2K | RNA polymerase II subunit K | **0,959** | 0,212748 | 30,631 | 35,437 |

| | | | | | |
|---|---|---|---|---|---|
| IKBKG | inhibitor of nuclear factor kappa B kinase subunit gamma | **0,959** | 0,508472 | 3,379 | 3,384 |
| PRDM5 | PR/SET domain 5 | **0,959** | 0,468733 | 7,865 | 9,067 |
| PSMC1 | proteasome 26S subunit, ATPase 1 | **0,959** | 0,175329 | 34,951 | 35,054 |
| ANXA1 | annexin A1 | **0,959** | 0,102846 | 884,027 | 818,300 |
| FAM78B | family with sequence similarity 78 member B | **0,959** | 0,789238 | 0,554 | 0,638 |
| STARD3 | StAR related lipid transfer domain containing 3 | **0,959** | 0,157732 | 24,815 | 24,646 |
| MUM1 | melanoma associated antigen (mutated) 1 | **0,959** | 0,307681 | 19,387 | 14,941 |
| ENO3 | enolase 3 | **0,959** | 0,707616 | 1,772 | 2,235 |
| EFTUD2 | elongation factor Tu GTP binding domain containing 2 | **0,959** | 0,121187 | 50,073 | 48,334 |
| COQ6 | coenzyme Q6, monooxygenase | **0,959** | 0,287559 | 12,020 | 11,812 |
| AKAP1 | A-kinase anchoring protein 1 | **0,959** | 0,302986 | 10,524 | 13,153 |
| PPWD1 | peptidylprolyl isomerase domain and WD repeat containing 1 | **0,959** | 0,125089 | 26,809 | 31,350 |
| ARHGEF17 | Rho guanine nucleotide exchange factor 17 | **0,959** | 0,174542 | 40,712 | 34,734 |
| PPIL2 | peptidylprolyl isomerase like 2 | **0,959** | 0,197053 | 19,830 | 18,963 |
| SNRNP40 | small nuclear ribonucleoprotein U5 subunit 40 | **0,959** | 0,158774 | 25,590 | 27,966 |
| GATAD2A | GATA zinc finger domain containing 2A | **0,959** | 0,167862 | 37,887 | 32,755 |
| LINC01144 | long intergenic non-protein coding RNA 1144 | **0,959** | 0,758726 | 0,720 | 0,958 |
| ARL4A | ADP ribosylation factor like GTPase 4A | **0,958** | 0,492764 | 3,933 | 2,490 |
| AGPAT4-IT1 | AGPAT4 intronic transcript 1 | **0,958** | 0,727662 | 0,997 | 1,085 |
| ACER3 | alkaline ceramidase 3 | **0,958** | 0,260152 | 12,629 | 9,450 |
| C14orf119 | chromosome 14 open reading frame 119 | **0,958** | 0,173895 | 32,292 | 31,478 |
| RNF10 | ring finger protein 10 | **0,958** | 0,104051 | 116,042 | 93,540 |
| RAD51-AS1 | RAD51 antisense RNA 1 (head to head) | **0,958** | 0,588728 | 2,049 | 2,043 |
| SMC1A | structural maintenance of chromosomes 1A | **0,958** | 0,153116 | 71,232 | 66,404 |
| ARMCX1 | armadillo repeat containing, X-linked 1 | **0,958** | 0,171674 | 59,323 | 39,715 |
| NSRP1 | nuclear speckle splicing regulatory protein 1 | **0,958** | 0,213652 | 37,222 | 36,011 |
| MAP2K7 | mitogen-activated protein kinase kinase 7 | **0,958** | 0,142884 | 42,263 | 41,694 |
| ADAL | adenosine deaminase like | **0,958** | 0,323012 | 8,032 | 8,428 |
| SPCS2 | signal peptidase complex subunit 2 | **0,958** | 0,168437 | 32,403 | 34,862 |
| UBE2J1 | ubiquitin conjugating enzyme E2 J1 | **0,958** | 0,111581 | 65,914 | 52,868 |
| TRIM14 | tripartite motif containing 14 | **0,958** | 0,607559 | 2,105 | 2,235 |
| FBRSL1 | fibrosin like 1 | **0,958** | 0,285091 | 20,162 | 18,006 |
| MKKS | McKusick-Kaufman syndrome | **0,958** | 0,286876 | 12,740 | 10,535 |

| | | | | | |
|---|---|---|---|---|---|
| GTF3C5 | general transcription factor IIIC subunit 5 | **0,958** | 0,170082 | 32,957 | 35,309 |
| ASNA1 | arsA arsenite transporter, ATP-binding, homolog 1 (bacterial) | **0,958** | 0,152188 | 35,228 | 33,585 |
| LAPTM4B | lysosomal protein transmembrane 4 beta | **0,958** | 0,211439 | 33,788 | 36,778 |
| GDPD5 | glycerophosphodiester phosphodiesterase domain containing 5 | **0,958** | 0,300743 | 18,334 | 24,774 |
| STXBP4 | syntaxin binding protein 4 | **0,958** | 0,4912 | 5,207 | 5,108 |
| EIF2B2 | eukaryotic translation initiation factor 2B subunit beta | **0,958** | 0,168238 | 24,150 | 20,751 |
| DNAJC3 | DnaJ heat shock protein family (Hsp40) member C3 | **0,958** | 0,139339 | 248,425 | 184,398 |
| SIMC1 | SUMO interacting motifs containing 1 | **0,958** | 0,585135 | 3,046 | 2,618 |
| DMD | dystrophin | **0,958** | 0,832402 | 0,665 | 0,319 |
| CMAS | cytidine monophosphate N-acetylneuraminic acid synthetase | **0,958** | 0,189281 | 26,033 | 25,412 |
| MLXIP | MLX interacting protein | **0,958** | 0,25001 | 20,439 | 19,091 |
| RBP4 | retinol binding protein 4 | **0,958** | 0,132514 | 132,050 | 30,584 |
| RUSC1 | RUN and SH3 domain containing 1 | **0,958** | 0,270426 | 15,731 | 8,620 |
| PABPC1 | poly(A) binding protein cytoplasmic 1 | **0,958** | 0,080336 | 472,035 | 399,573 |
| DEGS1 | delta 4-desaturase, sphingolipid 1 | **0,958** | 0,108798 | 81,922 | 70,299 |
| SUMF1 | sulfatase modifying factor 1 | **0,958** | 0,196516 | 35,007 | 25,476 |
| FAM86HP | family with sequence similarity 86 member H, pseudogene | **0,958** | 0,753826 | 1,440 | 0,958 |
| GATC | glutamyl-tRNA amidotransferase subunit C | **0,958** | 0,205593 | 19,331 | 26,178 |
| DSCC1 | DNA replication and sister chromatid cohesion 1 | **0,958** | 0,700852 | 1,274 | 0,511 |
| U2AF1L4 | U2 small nuclear RNA auxiliary factor 1 like 4 | **0,958** | 0,617652 | 1,551 | 2,043 |
| C7orf43 | chromosome 7 open reading frame 43 | **0,958** | 0,333648 | 10,635 | 10,918 |
| CIB1 | calcium and integrin binding 1 | **0,958** | 0,183734 | 60,874 | 62,637 |
| HIRA | histone cell cycle regulator | **0,958** | 0,377696 | 6,813 | 4,980 |
| PPOX | protoporphyrinogen oxidase | **0,958** | 0,430575 | 6,370 | 6,066 |
| WDR55 | WD repeat domain 55 | **0,958** | 0,212325 | 18,279 | 18,772 |
| GRK2 | G protein-coupled receptor kinase 2 | **0,958** | 0,177931 | 42,207 | 43,482 |
| ZNF786 | zinc finger protein 786 | **0,958** | 0,36636 | 5,484 | 5,363 |
| GORASP1 | golgi reassembly stacking protein 1 | **0,958** | 0,194366 | 26,033 | 24,455 |
| CDKAL1 | CDK5 regulatory subunit associated protein 1 like 1 | **0,958** | 0,305331 | 7,865 | 8,875 |
| WFDC2 | WAP four-disulfide core domain 2 | **0,957** | 0,689415 | 0,554 | 0,830 |
| ZCCHC9 | zinc finger CCHC-type containing 9 | **0,957** | 0,184474 | 15,122 | 13,345 |
| CEBPZOS | CEBPZ opposite strand | **0,957** | 0,157137 | 22,211 | 18,644 |
| ZNF672 | zinc finger protein 672 | **0,957** | 0,244126 | 27,861 | 21,645 |

| | | | | | |
|---|---|---|---|---|---|
| SMAD7 | SMAD family member 7 | **0,957** | 0,175046 | 67,244 | 66,340 |
| MPZL1 | myelin protein zero like 1 | **0,957** | 0,091316 | 94,994 | 75,279 |
| GSR | glutathione-disulfide reductase | **0,957** | 0,190305 | 31,628 | 20,496 |
| EEF1B2P3 | eukaryotic translation elongation factor 1 beta 2 pseudogene 3 | **0,957** | 0,809419 | 0,443 | 0,255 |
| HDAC3 | histone deacetylase 3 | **0,957** | 0,144259 | 26,698 | 23,369 |
| C1orf52 | chromosome 1 open reading frame 52 | **0,957** | 0,264676 | 10,690 | 10,918 |
| AUP1 | ancient ubiquitous protein 1 | **0,957** | 0,112969 | 53,839 | 58,423 |
| LRRC32 | leucine rich repeat containing 32 | **0,957** | 0,798727 | 0,665 | 0,383 |
| SLC2A4RG | SLC2A4 regulator | **0,957** | 0,150126 | 23,042 | 18,836 |
| CASZ1 | castor zinc finger 1 | **0,957** | 0,632837 | 2,105 | 2,235 |
| C14orf80 | chromosome 14 open reading frame 80 | **0,957** | 0,453396 | 5,650 | 4,342 |
| PTGFRN | prostaglandin F2 receptor inhibitor | **0,957** | 0,195625 | 24,759 | 22,986 |
| PLEKHG4 | pleckstrin homology and RhoGEF domain containing G4 | **0,957** | 0,575133 | 4,210 | 7,598 |
| JAGN1 | jagunal homolog 1 | **0,957** | 0,147041 | 32,403 | 34,032 |
| RSPRY1 | ring finger and SPRY domain containing 1 | **0,957** | 0,11395 | 59,877 | 51,910 |
| UQCC1 | ubiquinol-cytochrome c reductase complex assembly factor 1 | **0,957** | 0,15787 | 21,547 | 18,900 |
| RHOD | ras homolog family member D | **0,957** | 0,202106 | 23,098 | 21,454 |
| CDH24 | cadherin 24 | **0,957** | 0,707583 | 1,385 | 0,830 |
| OCEL1 | occludin/ELL domain containing 1 | **0,957** | 0,427633 | 5,761 | 4,980 |
| LARP1 | La ribonucleoprotein domain family member 1 | **0,957** | 0,101717 | 158,804 | 133,319 |
| KCTD15 | potassium channel tetramerization domain containing 15 | **0,957** | 0,325535 | 12,574 | 11,174 |
| CDK2 | cyclin dependent kinase 2 | **0,957** | 0,288799 | 11,189 | 8,109 |
| NFU1 | NFU1 iron-sulfur cluster scaffold | **0,957** | 0,158195 | 15,842 | 15,324 |
| TGS1 | trimethylguanosine synthase 1 | **0,957** | 0,145166 | 19,110 | 21,454 |
| CCDC90B | coiled-coil domain containing 90B | **0,957** | 0,112513 | 41,376 | 38,693 |
| CMTM3 | CKLF like MARVEL transmembrane domain containing 3 | **0,957** | 0,162462 | 21,879 | 20,687 |
| NSD3 | nuclear receptor binding SET domain protein 3 | **0,957** | 0,260222 | 60,264 | 57,720 |
| RIMKLB | ribosomal modification protein rimK like family member B | **0,957** | 0,487465 | 6,536 | 4,789 |
| DMWD | dystrophia myotonica, WD repeat containing | **0,957** | 0,132336 | 33,456 | 28,669 |
| BCL2A1 | BCL2 related protein A1 | **0,957** | 0,201267 | 99,204 | 126,167 |
| USP44 | ubiquitin specific peptidase 44 | **0,957** | 0,672548 | 1,219 | 1,341 |
| PARP2 | poly(ADP-ribose) polymerase 2 | **0,957** | 0,302681 | 8,585 | 9,386 |
| HDAC11 | histone deacetylase 11 | **0,957** | 0,402823 | 6,647 | 4,150 |

| | | | | | |
|---|---|---|---|---|---|
| ATG9A | autophagy related 9A | **0,957** | 0,260365 | 19,774 | 17,878 |
| PTBP2 | polypyrimidine tract binding protein 2 | **0,957** | 0,269008 | 9,250 | 13,408 |
| DOT1L | DOT1 like histone lysine methyltransferase | **0,957** | 0,227697 | 20,716 | 19,027 |
| UIMC1 | ubiquitin interaction motif containing 1 | **0,957** | 0,127414 | 24,593 | 22,092 |
| ADSSL1 | adenylosuccinate synthase like 1 | **0,957** | 0,385152 | 5,539 | 5,427 |
| OAS3 | 2'-5'-oligoadenylate synthetase 3 | **0,957** | 0,292644 | 8,696 | 10,280 |
| NEPRO | nucleolus and neural progenitor protein | **0,957** | 0,193384 | 26,033 | 24,327 |
| BCL9L | B-cell CLL/lymphoma 9 like | **0,957** | 0,120604 | 96,490 | 82,686 |
| C20orf194 | chromosome 20 open reading frame 194 | **0,957** | 0,338447 | 13,017 | 7,981 |
| CLHC1 | clathrin heavy chain linker domain containing 1 | **0,957** | 0,6789 | 0,831 | 1,149 |
| LAP3 | leucine aminopeptidase 3 | **0,957** | 0,177169 | 33,843 | 30,137 |
| MPLKIP | M-phase specific PLK1 interacting protein | **0,956** | 0,266168 | 13,127 | 13,025 |
| TROAP | trophinin associated protein | **0,956** | 0,750221 | 2,049 | 0,575 |
| C12orf45 | chromosome 12 open reading frame 45 | **0,956** | 0,406961 | 4,431 | 4,725 |
| CTNNBL1 | catenin beta like 1 | **0,956** | 0,170059 | 20,882 | 19,538 |
| RPL22 | ribosomal protein L22 | **0,956** | 0,209773 | 36,613 | 41,502 |
| UCHL1 | ubiquitin C-terminal hydrolase L1 | **0,956** | 0,28435 | 7,921 | 5,363 |
| GTF2E2 | general transcription factor IIE subunit 2 | **0,956** | 0,174974 | 23,098 | 22,539 |
| GNE | glucosamine (UDP-N-acetyl)-2-epimerase/N-acetylmannosamine kinase | **0,956** | 0,105292 | 44,257 | 35,117 |
| COX5B | cytochrome c oxidase subunit 5B | **0,956** | 0,155613 | 55,335 | 56,890 |
| PROSER1 | proline and serine rich 1 | **0,956** | 0,346242 | 16,562 | 14,430 |
| DDX31 | DEAD-box helicase 31 | **0,956** | 0,245906 | 10,136 | 12,195 |
| FKBP3 | FK506 binding protein 3 | **0,956** | 0,226184 | 31,406 | 26,115 |
| ZNF8 | zinc finger protein 8 | **0,956** | 0,650298 | 2,991 | 2,426 |
| EMX2 | empty spiracles homeobox 2 | **0,956** | 0,685755 | 1,385 | 1,149 |
| KHDC1 | KH homology domain containing 1 | **0,956** | 0,667872 | 1,772 | 1,213 |
| LCA5 | LCA5, lebercilin | **0,956** | 0,424205 | 4,985 | 5,172 |
| RPL23AP82 | ribosomal protein L23a pseudogene 82 | **0,956** | 0,514778 | 3,877 | 2,490 |
| TPM3P9 | tropomyosin 3 pseudogene 9 | **0,956** | 0,447024 | 5,207 | 3,448 |
| C16orf91 | chromosome 16 open reading frame 91 | **0,956** | 0,380809 | 7,533 | 6,832 |
| BCL7B | BCL tumor suppressor 7B | **0,956** | 0,169136 | 32,459 | 33,202 |
| ARHGAP28 | Rho GTPase activating protein 28 | **0,956** | 0,698594 | 1,385 | 1,532 |
| RPL28 | ribosomal protein L28 | **0,956** | 0,207295 | 222,890 | 262,168 |

| | | | | | |
|---|---|---|---|---|---|
| USB1 | U6 snRNA biogenesis phosphodiesterase 1 | **0,956** | 0,195616 | 30,963 | 27,902 |
| CDC34 | cell division cycle 34 | **0,956** | 0,176657 | 27,917 | 25,093 |
| SGCD | sarcoglycan delta | **0,956** | 0,323309 | 11,023 | 11,876 |
| DARS2 | aspartyl-tRNA synthetase 2, mitochondrial | **0,956** | 0,192738 | 14,734 | 14,302 |
| ANGPTL4 | angiopoietin like 4 | **0,956** | 0,361145 | 71,509 | 106,565 |
| SNRPG | small nuclear ribonucleoprotein polypeptide G | **0,956** | 0,284739 | 11,743 | 16,473 |
| SMARCAD1 | SWI/SNF-related, matrix-associated actin-dependent regulator of chromatin, subfamily a, | **0,956** | 0,153561 | 27,030 | 28,477 |
| JRK | Jrk helix-turn-helix protein | **0,956** | 0,270922 | 13,737 | 17,176 |
| ICE2 | interactor of little elongation complex ELL subunit 2 | **0,956** | 0,236411 | 17,005 | 15,707 |
| APMAP | adipocyte plasma membrane associated protein | **0,956** | 0,1133 | 104,687 | 91,497 |
| EMC6 | ER membrane protein complex subunit 6 | **0,956** | 0,119459 | 19,774 | 19,602 |
| NDUFB1 | NADH:ubiquinone oxidoreductase subunit B1 | **0,956** | 0,314019 | 12,795 | 12,834 |
| EIF4A2 | eukaryotic translation initiation factor 4A2 | **0,956** | 0,055286 | 155,480 | 183,121 |
| IL18BP | interleukin 18 binding protein | **0,956** | 0,499998 | 3,213 | 3,703 |
| SMAP2 | small ArfGAP2 | **0,956** | 0,248306 | 12,629 | 13,600 |
| EIF4E | eukaryotic translation initiation factor 4E | **0,956** | 0,243097 | 13,127 | 14,941 |
| SAP30BP | SAP30 binding protein | **0,956** | 0,151336 | 28,914 | 32,436 |
| DAD1 | defender against cell death 1 | **0,956** | 0,124466 | 129,170 | 115,824 |
| HIKESHI | Hikeshi, heat shock protein nuclear import factor | **0,956** | 0,306151 | 14,678 | 14,622 |
| ZSWIM1 | zinc finger SWIM-type containing 1 | **0,956** | 0,289747 | 10,136 | 10,471 |
| SSH3 | slingshot protein phosphatase 3 | **0,956** | 0,526369 | 5,982 | 6,193 |
| MALT1 | MALT1 paracaspase | **0,956** | 0,279538 | 21,491 | 19,410 |
| SCMH1 | sex comb on midleg homolog 1 (Drosophila) | **0,955** | 0,244054 | 17,669 | 15,388 |
| CACNA1D | calcium voltage-gated channel subunit alpha1 D | **0,955** | 0,727773 | 0,775 | 0,383 |
| ZNF610 | zinc finger protein 610 | **0,955** | 0,521253 | 2,105 | 3,129 |
| RPL36AL | ribosomal protein L36a like | **0,955** | 0,242745 | 99,093 | 88,304 |
| PSMC2 | proteasome 26S subunit, ATPase 2 | **0,955** | 0,109592 | 85,633 | 70,299 |
| DNAJC5 | DnaJ heat shock protein family (Hsp40) member C5 | **0,955** | 0,100227 | 118,867 | 117,548 |
| MOCS1 | molybdenum cofactor synthesis 1 | **0,955** | 0,305743 | 13,903 | 14,685 |
| DDB2 | damage specific DNA binding protein 2 | **0,955** | 0,263933 | 11,355 | 10,535 |
| GLUD1 | glutamate dehydrogenase 1 | **0,955** | 0,086636 | 55,833 | 49,675 |
| TRIOBP | TRIO and F-actin binding protein | **0,955** | 0,167608 | 39,105 | 34,351 |
| UTP14C | UTP14, small subunit processome component homolog C (S. cerevisiae) | **0,955** | 0,09808 | 42,540 | 39,204 |

| | | | | | |
|---|---|---|---|---|---|
| NARS | asparaginyl-tRNA synthetase | **0,955** | 0,097296 | 173,039 | 154,453 |
| ABCB7 | ATP binding cassette subfamily B member 7 | **0,955** | 0,25037 | 8,807 | 9,897 |
| TMEM104 | transmembrane protein 104 | **0,955** | 0,217685 | 20,162 | 21,007 |
| ZNF565 | zinc finger protein 565 | **0,955** | 0,489538 | 3,600 | 3,576 |
| CARD19 | caspase recruitment domain family member 19 | **0,955** | 0,197296 | 20,328 | 13,345 |
| RPL18 | ribosomal protein L18 | **0,955** | 0,168311 | 240,283 | 244,226 |
| PLS3 | plastin 3 | **0,955** | 0,126117 | 87,516 | 68,702 |
| TMEM30B | transmembrane protein 30B | **0,955** | 0,415421 | 6,979 | 10,408 |
| N6AMT1 | N-6 adenine-specific DNA methyltransferase 1 (putative) | **0,955** | 0,275505 | 10,967 | 8,684 |
| AP5B1 | adaptor related protein complex 5 beta 1 subunit | **0,955** | 0,315004 | 14,124 | 14,302 |
| PLEKHH3 | pleckstrin homology, MyTH4 and FERM domain containing H3 | **0,955** | 0,395313 | 8,696 | 5,938 |
| NMNAT1 | nicotinamide nucleotide adenylyltransferase 1 | **0,955** | 0,344221 | 11,300 | 7,470 |
| FAM122C | family with sequence similarity 122C | **0,955** | 0,674206 | 1,329 | 1,213 |
| CENPBD1P1 | CENPB DNA-binding domains containing 1 pseudogene 1 | **0,955** | 0,222475 | 23,430 | 20,624 |
| PDGFRB | platelet derived growth factor receptor beta | **0,955** | 0,346931 | 5,927 | 9,386 |
| TCEAL3 | transcription elongation factor A like 3 | **0,955** | 0,355343 | 13,072 | 11,876 |
| SPEF1 | sperm flagellar 1 | **0,955** | 0,821396 | 0,111 | 0,192 |
| NPR2 | natriuretic peptide receptor 2 | **0,955** | 0,165128 | 35,007 | 27,519 |
| ABRACL | ABRA C-terminal like | **0,955** | 0,53314 | 6,314 | 5,619 |
| ETFA | electron transfer flavoprotein alpha subunit | **0,955** | 0,158363 | 32,016 | 26,434 |
| MORF4L1 | mortality factor 4 like 1 | **0,955** | 0,081509 | 206,771 | 186,186 |
| MAPK3 | mitogen-activated protein kinase 3 | **0,955** | 0,182777 | 25,258 | 14,302 |
| TMEM62 | transmembrane protein 62 | **0,955** | 0,433949 | 5,151 | 4,597 |
| DENR | density regulated re-initiation and release factor | **0,955** | 0,120637 | 38,829 | 36,011 |
| LIX1L | limb and CNS expressed 1 like | **0,955** | 0,105983 | 23,098 | 21,134 |
| SNAP23 | synaptosome associated protein 23 | **0,955** | 0,119148 | 36,613 | 30,009 |
| AKTIP | AKT interacting protein | **0,955** | 0,285256 | 12,961 | 13,025 |
| UBTD1 | ubiquitin domain containing 1 | **0,955** | 0,240136 | 16,728 | 14,813 |
| MYOM1 | myomesin 1 | **0,955** | 0,588802 | 1,163 | 1,724 |
| AQP1 | aquaporin 1 (Colton blood group) | **0,955** | 0,771758 | 0,277 | 1,277 |
| RPL21 | ribosomal protein L21 | **0,955** | 0,235696 | 104,687 | 105,416 |
| LINC00513 | long intergenic non-protein coding RNA 513 | **0,955** | 0,749218 | 1,163 | 1,469 |
| MBP | myelin basic protein | **0,955** | 0,286329 | 20,217 | 13,345 |

| | | | | | |
|---|---|---|---|---|---|
| CCP110 | centriolar coiled-coil protein 110 | **0,955** | 0,297035 | 19,054 | 16,920 |
| SZRD1 | SUZ RNA binding domain containing 1 | **0,955** | 0,069681 | 118,424 | 112,312 |
| UCK2 | uridine-cytidine kinase 2 | **0,955** | 0,206844 | 18,445 | 16,218 |
| PTRH2 | peptidyl-tRNA hydrolase 2 | **0,955** | 0,264182 | 12,961 | 14,111 |
| NCBP2-AS2 | NCBP2 antisense RNA 2 (head to head) | **0,955** | 0,289218 | 8,807 | 9,897 |
| MRPS2 | mitochondrial ribosomal protein S2 | **0,955** | 0,170869 | 22,488 | 22,092 |
| KYAT1 | kynurenine aminotransferase 1 | **0,955** | 0,637823 | 2,049 | 2,746 |
| TRAPPC3 | trafficking protein particle complex 3 | **0,955** | 0,128578 | 47,968 | 48,845 |
| PMS2CL | PMS2 C-terminal like pseudogene | **0,955** | 0,469034 | 3,157 | 4,150 |
| ETFB | electron transfer flavoprotein beta subunit | **0,955** | 0,395738 | 9,029 | 7,087 |
| TSPAN9 | tetraspanin 9 | **0,955** | 0,158431 | 15,509 | 12,387 |
| SLC35B1 | solute carrier family 35 member B1 | **0,955** | 0,19439 | 41,653 | 42,205 |
| XRCC1 | X-ray repair cross complementing 1 | **0,955** | 0,251278 | 15,786 | 13,089 |
| VRK1 | vaccinia related kinase 1 | **0,955** | 0,385886 | 4,431 | 4,406 |
| IQCH | IQ motif containing H | **0,955** | 0,521139 | 2,770 | 3,065 |
| TMX1 | thioredoxin related transmembrane protein 1 | **0,955** | 0,126669 | 61,760 | 52,102 |
| ARHGEF28 | Rho guanine nucleotide exchange factor 28 | **0,955** | 0,416741 | 9,527 | 5,427 |
| UTP3 | UTP3, small subunit processome component homolog (S. cerevisiae) | **0,954** | 0,140717 | 46,805 | 47,185 |
| GAS2L1 | growth arrest specific 2 like 1 | **0,954** | 0,306713 | 11,244 | 8,747 |
| FANCE | Fanconi anemia complementation group E | **0,954** | 0,42956 | 6,259 | 5,874 |
| PANX1 | pannexin 1 | **0,954** | 0,123965 | 36,668 | 25,412 |
| EXOSC9 | exosome component 9 | **0,954** | 0,20835 | 10,801 | 12,642 |
| GPATCH1 | G-patch domain containing 1 | **0,954** | 0,379866 | 8,807 | 9,641 |
| CCDC71 | coiled-coil domain containing 71 | **0,954** | 0,201062 | 15,897 | 16,729 |
| RDH11 | retinol dehydrogenase 11 (all-trans/9-cis/11-cis) | **0,954** | 0,138534 | 58,381 | 57,082 |
| DFNA5 | DFNA5, deafness associated tumor suppressor | **0,954** | 0,176362 | 27,806 | 14,877 |
| MAN1B1 | mannosidase alpha class 1B member 1 | **0,954** | 0,092462 | 83,971 | 75,918 |
| SEC24C | SEC24 homolog C, COPII coat complex component | **0,954** | 0,099692 | 85,744 | 84,856 |
| TMEM191A | transmembrane protein 191A (pseudogene) | **0,954** | 0,724632 | 0,609 | 1,405 |
| KLC2 | kinesin light chain 2 | **0,954** | 0,149556 | 39,272 | 33,330 |
| CCDC94 | coiled-coil domain containing 94 | **0,954** | 0,196571 | 12,075 | 12,770 |
| NXPE2 | neurexophilin and PC-esterase domain family member 2 | **0,954** | 0,618439 | 4,874 | 4,533 |
| CFAP36 | cilia and flagella associated protein 36 | **0,954** | 0,095771 | 33,179 | 37,288 |

| | | | | | |
|---|---|---|---|---|---|
| LASP1 | LIM and SH3 protein 1 | **0,954** | 0,096263 | 121,304 | 71,256 |
| XPO7 | exportin 7 | **0,954** | 0,0913 | 45,863 | 41,566 |
| RINT1 | RAD50 interactor 1 | **0,954** | 0,198669 | 17,946 | 18,580 |
| FAU | FAU, ubiquitin like and ribosomal protein S30 fusion | **0,954** | 0,192162 | 117,538 | 107,970 |
| CPT1C | carnitine palmitoyltransferase 1C | **0,954** | 0,491039 | 3,767 | 2,171 |
| SLC9A6 | solute carrier family 9 member A6 | **0,954** | 0,213205 | 27,695 | 19,666 |
| PHYHIP | phytanoyl-CoA 2-hydroxylase interacting protein | **0,954** | 0,812111 | 0,554 | 0,638 |
| UBA6 | ubiquitin like modifier activating enzyme 6 | **0,954** | 0,201998 | 33,400 | 31,542 |
| ING5 | inhibitor of growth family member 5 | **0,954** | 0,201049 | 15,786 | 18,900 |
| TRMU | tRNA 5-methylaminomethyl-2-thiouridylate methyltransferase | **0,954** | 0,181739 | 12,186 | 10,727 |
| LRRK2 | leucine rich repeat kinase 2 | **0,954** | 0,606566 | 2,160 | 3,320 |
| C22orf46 | chromosome 22 open reading frame 46 | **0,954** | 0,143109 | 34,287 | 33,904 |
| SELENOS | selenoprotein S | **0,954** | 0,069899 | 121,028 | 105,352 |
| GTF3C2 | general transcription factor IIIC subunit 2 | **0,954** | 0,095309 | 27,695 | 26,817 |
| ARPC1B | actin related protein 2/3 complex subunit 1B | **0,954** | 0,384361 | 6,148 | 4,725 |
| TMEM115 | transmembrane protein 115 | **0,954** | 0,123942 | 50,128 | 36,331 |
| USMG5 | up-regulated during skeletal muscle growth 5 homolog (mouse) | **0,954** | 0,129355 | 38,773 | 36,075 |
| S100A10 | S100 calcium binding protein A10 | **0,954** | 0,188925 | 86,242 | 61,743 |
| APH1A | aph-1 homolog A, gamma-secretase subunit | **0,954** | 0,090919 | 87,073 | 67,042 |
| MCCC2 | methylcrotonoyl-CoA carboxylase 2 | **0,954** | 0,138409 | 15,731 | 18,644 |
| NSD2 | nuclear receptor binding SET domain protein 2 | **0,954** | 0,21155 | 34,785 | 21,198 |
| ZNF557 | zinc finger protein 557 | **0,954** | 0,428743 | 5,428 | 5,427 |
| FLRT3 | fibronectin leucine rich transmembrane protein 3 | **0,954** | 0,664076 | 1,219 | 0,128 |
| C11orf70 | chromosome 11 open reading frame 70 | **0,954** | 0,478081 | 3,933 | 4,916 |
| BOLA3-AS1 | BOLA3 antisense RNA 1 (head to head) | **0,954** | 0,574688 | 2,160 | 1,213 |
| ALDH3A2 | aldehyde dehydrogenase 3 family member A2 | **0,954** | 0,088837 | 69,404 | 54,209 |
| ARMC7 | armadillo repeat containing 7 | **0,954** | 0,405012 | 6,868 | 7,470 |
| GPSM1 | G protein signaling modulator 1 | **0,954** | 0,210792 | 17,171 | 19,347 |
| COX6B1 | cytochrome c oxidase subunit 6B1 | **0,953** | 0,168284 | 59,489 | 57,209 |
| COQ7 | coenzyme Q7, hydroxylase | **0,953** | 0,220221 | 15,011 | 15,835 |
| RBM10 | RNA binding motif protein 10 | **0,953** | 0,094938 | 36,281 | 37,608 |
| AMZ2 | archaelysin family metallopeptidase 2 | **0,953** | 0,128841 | 35,505 | 29,626 |
| ZNF202 | zinc finger protein 202 | **0,953** | 0,251546 | 8,696 | 10,408 |

| | | | | | |
|---|---|---|---|---|---|
| ADAM12 | ADAM metallopeptidase domain 12 | **0,953** | 0,455434 | 19,276 | 15,516 |
| ACTL6A | actin like 6A | **0,953** | 0,142606 | 27,806 | 31,031 |
| ZW10 | zw10 kinetochore protein | **0,953** | 0,182456 | 14,568 | 14,366 |
| CFAP58 | cilia and flagella associated protein 58 | **0,953** | 0,623992 | 1,551 | 2,171 |
| RNF139-AS1 | RNF139 antisense RNA 1 (head to head) | **0,953** | 0,734327 | 1,274 | 2,043 |
| SNX24 | sorting nexin 24 | **0,953** | 0,230921 | 10,358 | 7,981 |
| SLC25A11 | solute carrier family 25 member 11 | **0,953** | 0,111921 | 35,837 | 38,693 |
| HCCS | holocytochrome c synthase | **0,953** | 0,218 | 19,774 | 16,665 |
| CDIPT | CDP-diacylglycerol--inositol 3-phosphatidyltransferase | **0,953** | 0,121938 | 62,868 | 55,486 |
| VIM-AS1 | VIM antisense RNA 1 | **0,953** | 0,396762 | 6,148 | 5,172 |
| SYNCRIP | synaptotagmin binding cytoplasmic RNA interacting protein | **0,953** | 0,047044 | 110,226 | 105,672 |
| RBM22 | RNA binding motif protein 22 | **0,953** | 0,074428 | 32,071 | 30,648 |
| GGT1 | gamma-glutamyltransferase 1 | **0,953** | 0,684211 | 0,665 | 1,341 |
| LGALS1 | galectin 1 | **0,953** | 0,186573 | 194,696 | 48,079 |
| IFT22 | intraflagellar transport 22 | **0,953** | 0,222091 | 18,556 | 17,112 |
| IARS2 | isoleucyl-tRNA synthetase 2, mitochondrial | **0,953** | 0,058046 | 69,958 | 54,783 |
| PCBP1 | poly(rC) binding protein 1 | **0,953** | 0,070609 | 401,357 | 356,985 |
| MED17 | mediator complex subunit 17 | **0,953** | 0,504881 | 2,714 | 3,703 |
| RPS13 | ribosomal protein S13 | **0,953** | 0,134771 | 145,344 | 150,750 |
| PCDHB6 | protocadherin beta 6 | **0,953** | 0,58078 | 1,772 | 1,788 |
| EEF1AKMT1 | EEF1A lysine methyltransferase 1 | **0,953** | 0,323629 | 5,484 | 6,832 |
| FAM184A | family with sequence similarity 184 member A | **0,953** | 0,767441 | 0,609 | 0,766 |
| HSBP1 | heat shock factor binding protein 1 | **0,953** | 0,128715 | 60,874 | 48,462 |
| TANK | TRAF family member associated NFKB activator | **0,953** | 0,124449 | 48,854 | 39,587 |
| RBM7 | RNA binding motif protein 7 | **0,953** | 0,242677 | 19,885 | 19,666 |
| C1GALT1 | core 1 synthase, glycoprotein-N-acetylgalactosamine 3-beta-galactosyltransferase 1 | **0,953** | 0,09542 | 63,809 | 42,779 |
| KCTD20 | potassium channel tetramerization domain containing 20 | **0,953** | 0,10681 | 45,918 | 40,992 |
| DBI | diazepam binding inhibitor, acyl-CoA binding protein | **0,953** | 0,181766 | 68,462 | 52,612 |
| ATP1A1 | ATPase Na+/K+ transporting subunit alpha 1 | **0,953** | 0,074299 | 299,938 | 253,484 |
| TMEM198 | transmembrane protein 198 | **0,953** | 0,368484 | 7,755 | 5,938 |
| FBXO4 | F-box protein 4 | **0,953** | 0,374076 | 4,431 | 5,683 |
| IFT122 | intraflagellar transport 122 | **0,953** | 0,228392 | 12,297 | 9,131 |
| POLR3F | RNA polymerase III subunit F | **0,953** | 0,249529 | 9,084 | 10,280 |

| | | | | | |
|---|---|---|---|---|---|
| CCNB1 | cyclin B1 | **0,953** | 0,496682 | 9,250 | 4,214 |
| FCHSD1 | FCH and double SH3 domains 1 | **0,953** | 0,292073 | 13,460 | 10,344 |
| SELENOK | selenoprotein K | **0,953** | 0,0565 | 86,187 | 66,787 |
| MPND | MPN domain containing | **0,953** | 0,516814 | 5,705 | 4,150 |
| SASH1 | SAM and SH3 domain containing 1 | **0,953** | 0,19733 | 48,633 | 60,785 |
| POLR2J4 | RNA polymerase II subunit J4, pseudogene | **0,953** | 0,63544 | 1,329 | 1,213 |
| PACS1 | phosphofurin acidic cluster sorting protein 1 | **0,953** | 0,201544 | 28,637 | 19,602 |
| EDC3 | enhancer of mRNA decapping 3 | **0,953** | 0,100779 | 23,984 | 25,412 |
| VEGFB | vascular endothelial growth factor B | **0,953** | 0,325106 | 14,124 | 7,790 |
| NCAPD2 | non-SMC condensin I complex subunit D2 | **0,953** | 0,086496 | 49,020 | 43,354 |
| COPS2 | COP9 signalosome subunit 2 | **0,953** | 0,077862 | 58,603 | 50,633 |
| ZNF3 | zinc finger protein 3 | **0,953** | 0,250749 | 14,900 | 12,898 |
| GEN1 | GEN1, Holliday junction 5' flap endonuclease | **0,953** | 0,552561 | 6,591 | 4,278 |
| CPS1 | carbamoyl-phosphate synthase 1 | **0,953** | 0,500789 | 3,157 | 3,959 |
| DHRS12 | dehydrogenase/reductase 12 | **0,953** | 0,777672 | 0,332 | 0,319 |
| BLM | Bloom syndrome RecQ like helicase | **0,953** | 0,678315 | 1,329 | 0,830 |
| TMEM230 | transmembrane protein 230 | **0,952** | 0,079992 | 72,838 | 61,360 |
| PRDM6 | PR/SET domain 6 | **0,952** | 0,62806 | 2,825 | 1,979 |
| CTSZ | cathepsin Z | **0,952** | 0,10641 | 112,664 | 42,588 |
| COG2 | component of oligomeric golgi complex 2 | **0,952** | 0,110778 | 20,107 | 22,858 |
| DGCR14 | DiGeorge syndrome critical region gene 14 | **0,952** | 0,228989 | 14,900 | 16,090 |
| KEAP1 | kelch like ECH associated protein 1 | **0,952** | 0,085026 | 93,776 | 70,746 |
| NDRG3 | NDRG family member 3 | **0,952** | 0,297835 | 6,813 | 5,555 |
| DPY19L1P1 | DPY19L1 pseudogene 1 | **0,952** | 0,804453 | 0,609 | 0,638 |
| HLX | H2.0 like homeobox | **0,952** | 0,404233 | 5,816 | 3,192 |
| UXT | ubiquitously expressed prefoldin like chaperone | **0,952** | 0,2194 | 21,159 | 18,644 |
| C14orf28 | chromosome 14 open reading frame 28 | **0,952** | 0,306108 | 7,312 | 5,044 |
| PSMD2 | proteasome 26S subunit, non-ATPase 2 | **0,952** | 0,063876 | 166,281 | 138,235 |
| STRIP1 | striatin interacting protein 1 | **0,952** | 0,171564 | 28,969 | 33,202 |
| SLC47A1 | solute carrier family 47 member 1 | **0,952** | 0,440909 | 5,428 | 6,130 |
| C21orf58 | chromosome 21 open reading frame 58 | **0,952** | 0,648344 | 1,496 | 0,894 |
| REEP4 | receptor accessory protein 4 | **0,952** | 0,230229 | 10,081 | 8,875 |
| ZBTB48 | zinc finger and BTB domain containing 48 | **0,952** | 0,247714 | 7,921 | 9,705 |

| | | | | | |
|---|---|---|---|---|---|
| RNF144B | ring finger protein 144B | **0,952** | 0,057253 | 126,843 | 100,691 |
| EMC4 | ER membrane protein complex subunit 4 | **0,952** | 0,0656 | 55,169 | 54,145 |
| C6orf89 | chromosome 6 open reading frame 89 | **0,952** | 0,057078 | 137,811 | 117,356 |
| TCP11L1 | t-complex 11 like 1 | **0,952** | 0,232107 | 23,541 | 19,921 |
| ZNF331 | zinc finger protein 331 | **0,952** | 0,201057 | 17,559 | 17,559 |
| SOX6 | SRY-box 6 | **0,952** | 0,431247 | 4,099 | 6,066 |
| PCSK1N | proprotein convertase subtilisin/kexin type 1 inhibitor | **0,952** | 0,489682 | 7,201 | 4,342 |
| CCNC | cyclin C | **0,952** | 0,102124 | 17,448 | 18,580 |
| ZNF641 | zinc finger protein 641 | **0,952** | 0,151148 | 17,669 | 13,536 |
| PRKAB1 | protein kinase AMP-activated non-catalytic subunit beta 1 | **0,952** | 0,134183 | 19,774 | 26,370 |
| 2.syys | septin 2 | **0,952** | 0,056449 | 180,517 | 133,510 |
| ARHGAP39 | Rho GTPase activating protein 39 | **0,952** | 0,29282 | 9,693 | 6,896 |
| WWC2 | WW and C2 domain containing 2 | **0,952** | 0,240275 | 30,465 | 27,711 |
| TIMM21 | translocase of inner mitochondrial membrane 21 | **0,952** | 0,116374 | 16,451 | 20,815 |
| FICD | FIC domain containing | **0,952** | 0,141925 | 78,876 | 75,343 |
| CA5BP1 | carbonic anhydrase 5B pseudogene 1 | **0,952** | 0,257072 | 7,367 | 9,322 |
| ID2 | inhibitor of DNA binding 2, HLH protein | **0,952** | 0,084127 | 454,698 | 534,487 |
| DDX20 | DEAD-box helicase 20 | **0,952** | 0,13361 | 15,122 | 15,132 |
| PEX19 | peroxisomal biogenesis factor 19 | **0,952** | 0,072973 | 32,791 | 31,733 |
| ADARB1 | adenosine deaminase, RNA specific B1 | **0,952** | 0,159898 | 19,830 | 16,601 |
| KDM1A | lysine demethylase 1A | **0,952** | 0,113688 | 32,237 | 27,392 |
| CSTF1 | cleavage stimulation factor subunit 1 | **0,952** | 0,124686 | 16,229 | 12,962 |
| NOP10 | NOP10 ribonucleoprotein | **0,952** | 0,105777 | 38,718 | 34,479 |
| TSPAN5 | tetraspanin 5 | **0,952** | 0,20542 | 24,649 | 21,964 |
| LRRC27 | leucine rich repeat containing 27 | **0,952** | 0,368979 | 5,705 | 5,810 |
| NPM1 | nucleophosmin | **0,952** | 0,111843 | 250,530 | 272,384 |
| LPIN3 | lipin 3 | **0,952** | 0,363879 | 4,930 | 4,406 |
| C17orf75 | chromosome 17 open reading frame 75 | **0,952** | 0,213609 | 10,912 | 9,577 |
| COPS7A | COP9 signalosome subunit 7A | **0,952** | 0,090116 | 54,282 | 46,738 |
| RAET1E-AS1 | RAET1E antisense RNA 1 | **0,952** | 0,787673 | 0,222 | 0,511 |
| MFSD1 | major facilitator superfamily domain containing 1 | **0,952** | 0,141832 | 65,360 | 51,782 |
| HNRNPLP2 | heterogeneous nuclear ribonucleoprotein L pseudogene 2 | **0,951** | 0,802236 | 0,609 | 0,255 |
| HMCES | 5-hydroxymethylcytosine binding, ES cell specific | **0,951** | 0,132983 | 32,182 | 26,051 |

| | | | | | |
|---|---|---|---|---|---|
| SMARCE1 | SWI/SNF related, matrix associated, actin dependent regulator of chromatin, subfamily e, | **0,951** | 0,502423 | 3,767 | 3,703 |
| SPACA9 | sperm acrosome associated 9 | **0,951** | 0,365922 | 5,705 | 5,044 |
| TSPAN6 | tetraspanin 6 | **0,951** | 0,098121 | 35,284 | 23,241 |
| ZC3H15 | zinc finger CCCH-type containing 15 | **0,951** | 0,181749 | 47,414 | 43,482 |
| RPL14 | ribosomal protein L14 | **0,951** | 0,106316 | 215,246 | 187,527 |
| METTL27 | methyltransferase like 27 | **0,951** | 0,62555 | 0,942 | 0,447 |
| PARP8 | poly(ADP-ribose) polymerase family member 8 | **0,951** | 0,363864 | 4,043 | 4,023 |
| AMIGO2 | adhesion molecule with Ig like domain 2 | **0,951** | 0,193327 | 155,646 | 64,233 |
| TRUB1 | TruB pseudouridine synthase family member 1 | **0,951** | 0,248717 | 13,515 | 12,962 |
| WDR83 | WD repeat domain 83 | **0,951** | 0,417591 | 6,979 | 7,598 |
| URI1 | URI1, prefoldin like chaperone | **0,951** | 0,076322 | 44,921 | 37,671 |
| INTS8 | integrator complex subunit 8 | **0,951** | 0,201951 | 13,737 | 14,558 |
| PPP1CC | protein phosphatase 1 catalytic subunit gamma | **0,951** | 0,064591 | 57,273 | 52,421 |
| TIMP3 | TIMP metallopeptidase inhibitor 3 | **0,951** | 0,134313 | 637,430 | 751,130 |
| TARDBP | TAR DNA binding protein | **0,951** | 0,059439 | 59,046 | 57,656 |
| SLC30A9 | solute carrier family 30 member 9 | **0,951** | 0,091071 | 43,537 | 34,415 |
| CD70 | CD70 molecule | **0,951** | 0,695246 | 1,274 | 1,085 |
| SSC5D | scavenger receptor cysteine rich family member with 5 domains | **0,951** | 0,537426 | 5,761 | 2,362 |
| NSA2 | NSA2, ribosome biogenesis homolog | **0,951** | 0,123365 | 36,281 | 36,139 |
| SNRPB2 | small nuclear ribonucleoprotein polypeptide B2 | **0,951** | 0,180981 | 42,983 | 42,205 |
| VPS33A | VPS33A, CORVET/HOPS core subunit | **0,951** | 0,343541 | 7,588 | 6,513 |
| TMEM100 | transmembrane protein 100 | **0,951** | 0,681634 | 0,831 | 1,149 |
| RSPO2 | R-spondin 2 | **0,951** | 0,141964 | 23,596 | 29,882 |
| METAP1 | methionyl aminopeptidase 1 | **0,951** | 0,14161 | 20,550 | 17,112 |
| POP4 | POP4 homolog, ribonuclease P/MRP subunit | **0,951** | 0,190378 | 24,649 | 22,858 |
| DHX40 | DEAH-box helicase 40 | **0,951** | 0,094596 | 36,558 | 28,924 |
| AGFG1 | ArfGAP with FG repeats 1 | **0,951** | 0,078991 | 61,705 | 70,107 |
| C19orf38 | chromosome 19 open reading frame 38 | **0,951** | 0,62858 | 0,997 | 1,022 |
| PARP16 | poly(ADP-ribose) polymerase family member 16 | **0,951** | 0,276728 | 6,425 | 5,172 |
| NUTF2 | nuclear transport factor 2 | **0,951** | 0,124363 | 26,809 | 21,837 |
| NFATC2 | nuclear factor of activated T-cells 2 | **0,951** | 0,471949 | 3,379 | 3,448 |
| TMEM150A | transmembrane protein 150A | **0,951** | 0,353734 | 6,979 | 4,597 |
| ZNF785 | zinc finger protein 785 | **0,951** | 0,253605 | 12,684 | 11,621 |

| | | | | | |
|---|---|---|---|---|---|
| TNRC6A | trinucleotide repeat containing 6A | **0,951** | 0,177645 | 59,046 | 59,636 |
| BPHL | biphenyl hydrolase like | **0,951** | 0,452858 | 6,591 | 7,534 |
| HPS6 | HPS6, biogenesis of lysosomal organelles complex 2 subunit 3 | **0,951** | 0,195149 | 20,494 | 13,664 |
| SLC25A5 | solute carrier family 25 member 5 | **0,951** | 0,120806 | 75,774 | 63,786 |
| PIH1D1 | PIH1 domain containing 1 | **0,951** | 0,096153 | 24,981 | 22,284 |
| METTL23 | methyltransferase like 23 | **0,951** | 0,211818 | 8,530 | 7,917 |
| RBX1 | ring-box 1 | **0,951** | 0,128227 | 25,147 | 20,113 |
| TMEM11 | transmembrane protein 11 | **0,951** | 0,092397 | 18,445 | 19,474 |
| KLHDC3 | kelch domain containing 3 | **0,950** | 0,102426 | 26,366 | 26,051 |
| SRGN | serglycin | **0,950** | 0,14148 | 251,970 | 74,385 |
| ANAPC2 | anaphase promoting complex subunit 2 | **0,950** | 0,077973 | 29,135 | 27,200 |
| NFYC | nuclear transcription factor Y subunit gamma | **0,950** | 0,093248 | 24,538 | 27,008 |
| IDH3G | isocitrate dehydrogenase 3 (NAD(+)) gamma | **0,950** | 0,158703 | 16,894 | 12,578 |
| SRPX2 | sushi repeat containing protein, X-linked 2 | **0,950** | 0,067356 | 138,309 | 69,213 |
| CUL4B | cullin 4B | **0,950** | 0,05564 | 52,454 | 48,590 |
| SF3B6 | splicing factor 3b subunit 6 | **0,950** | 0,13434 | 38,330 | 42,269 |
| MRPS5 | mitochondrial ribosomal protein S5 | **0,950** | 0,107668 | 23,375 | 27,328 |
| NUDT15 | nudix hydrolase 15 | **0,950** | 0,207579 | 10,635 | 12,004 |
| LRRC37A7P | leucine rich repeat containing 37 member A7, pseudogene | **0,950** | 0,77193 | 0,499 | 0,319 |
| NOL10 | nucleolar protein 10 | **0,950** | 0,139381 | 17,559 | 18,133 |
| DNAJB9 | DnaJ heat shock protein family (Hsp40) member B9 | **0,950** | 0,140611 | 208,433 | 181,525 |
| TMEM185A | transmembrane protein 185A | **0,950** | 0,335773 | 8,087 | 5,938 |
| SNRPN | small nuclear ribonucleoprotein polypeptide N | **0,950** | 0,306804 | 5,428 | 4,980 |
| PTGER3 | prostaglandin E receptor 3 | **0,950** | 0,375506 | 9,361 | 4,150 |
| GOLGA1 | golgin A1 | **0,950** | 0,143625 | 27,418 | 22,092 |
| GFM1 | G elongation factor mitochondrial 1 | **0,950** | 0,12015 | 32,237 | 33,777 |
| GAS5-AS1 | GAS5 antisense RNA 1 | **0,950** | 0,810486 | 0,332 | 0,128 |
| CD302 | CD302 molecule | **0,950** | 0,219657 | 10,469 | 11,301 |
| EME2 | essential meiotic structure-specific endonuclease subunit 2 | **0,950** | 0,253202 | 8,142 | 8,045 |
| DLX5 | distal-less homeobox 5 | **0,950** | 0,34931 | 4,985 | 5,044 |
| RMND5B | required for meiotic nuclear division 5 homolog B | **0,950** | 0,083027 | 19,553 | 20,496 |
| TUBB2B | tubulin beta 2B class IIb | **0,950** | 0,515867 | 3,490 | 2,426 |
| ARV1 | ARV1 homolog, fatty acid homeostasis modulator | **0,950** | 0,201046 | 9,139 | 8,300 |

| | | | | | |
|---|---|---|---|---|---|
| UBE2F | ubiquitin conjugating enzyme E2 F (putative) | **0,950** | 0,288273 | 6,425 | 5,236 |
| DIS3L2 | DIS3 like 3'-5' exoribonuclease 2 | **0,950** | 0,217474 | 8,918 | 8,875 |
| FUCA2 | fucosidase, alpha-L- 2, plasma | **0,950** | 0,09243 | 79,873 | 53,953 |
| GAPDH | glyceraldehyde-3-phosphate dehydrogenase | **0,950** | 0,077767 | 2748,626 | 2110,430 |
| IGLV5-52 | immunoglobulin lambda variable 5-52 | **0,950** | 0,669831 | 1,108 | 0,447 |
| CYP2T1P | cytochrome P450 family 2 subfamily T member 1, pseudogene | **0,950** | 0,471135 | 3,323 | 4,342 |
| KIAA0753 | KIAA0753 | **0,950** | 0,188377 | 11,023 | 13,536 |
| TUBA1B | tubulin alpha 1b | **0,950** | 0,084838 | 140,137 | 107,012 |
| TMEM98 | transmembrane protein 98 | **0,950** | 0,114407 | 27,861 | 20,240 |
| FAM118A | family with sequence similarity 118 member A | **0,950** | 0,283013 | 9,915 | 5,619 |
| FBXL8 | F-box and leucine rich repeat protein 8 | **0,950** | 0,587885 | 1,385 | 1,149 |
| FRAT2 | FRAT2, WNT signaling pathway regulator | **0,950** | 0,401402 | 4,320 | 4,789 |
| MCPH1 | microcephalin 1 | **0,950** | 0,193993 | 11,632 | 10,471 |
| CLMN | calmin | **0,950** | 0,280938 | 9,970 | 7,279 |
| SUB1 | SUB1 homolog, transcriptional regulator | **0,950** | 0,118216 | 44,257 | 44,695 |
| HSDL2 | hydroxysteroid dehydrogenase like 2 | **0,950** | 0,092116 | 20,882 | 17,623 |
| NACC2 | NACC family member 2 | **0,949** | 0,168766 | 26,476 | 18,070 |
| NOL8 | nucleolar protein 8 | **0,949** | 0,182119 | 17,836 | 15,516 |
| RHOBTB3 | Rho related BTB domain containing 3 | **0,949** | 0,142687 | 29,745 | 20,240 |
| TTC14 | tetratricopeptide repeat domain 14 | **0,949** | 0,127714 | 21,436 | 23,369 |
| CYB561A3 | cytochrome b561 family member A3 | **0,949** | 0,054924 | 39,659 | 30,009 |
| TSC2 | tuberous sclerosis 2 | **0,949** | 0,179427 | 43,869 | 42,588 |
| LTA4H | leukotriene A4 hydrolase | **0,949** | 0,114204 | 44,091 | 44,440 |
| PLEKHO1 | pleckstrin homology domain containing O1 | **0,949** | 0,370093 | 5,484 | 4,661 |
| FAM222B | family with sequence similarity 222 member B | **0,949** | 0,084389 | 19,220 | 18,453 |
| PHC1 | polyhomeotic homolog 1 | **0,949** | 0,387174 | 5,207 | 3,831 |
| FBLN5 | fibulin 5 | **0,949** | 0,404038 | 11,743 | 2,554 |
| PREP | prolyl endopeptidase | **0,949** | 0,182225 | 22,876 | 17,814 |
| ZNF575 | zinc finger protein 575 | **0,949** | 0,632406 | 1,163 | 0,766 |
| GRIPAP1 | GRIP1 associated protein 1 | **0,949** | 0,084943 | 47,137 | 42,971 |
| C1orf74 | chromosome 1 open reading frame 74 | **0,949** | 0,328321 | 4,930 | 5,363 |
| SPCS1 | signal peptidase complex subunit 1 | **0,949** | 0,066745 | 56,443 | 51,463 |
| TMED7 | transmembrane p24 trafficking protein 7 | **0,949** | 0,139247 | 115,876 | 85,048 |

| | | | | | |
|---|---|---|---|---|---|
| C9orf142 | chromosome 9 open reading frame 142 | **0,949** | 0,308331 | 5,705 | 4,916 |
| C1orf56 | chromosome 1 open reading frame 56 | **0,949** | 0,584971 | 1,606 | 1,532 |
| DHX57 | DExH-box helicase 57 | **0,949** | 0,14118 | 18,168 | 19,793 |
| AARSD1 | alanyl-tRNA synthetase domain containing 1 | **0,949** | 0,748941 | 0,222 | 0,511 |
| MRPL13 | mitochondrial ribosomal protein L13 | **0,949** | 0,164212 | 16,728 | 19,027 |
| ZNF460 | zinc finger protein 460 | **0,949** | 0,612885 | 1,939 | 3,448 |
| ADCY1 | adenylate cyclase 1 | **0,949** | 0,637269 | 0,720 | 1,149 |
| ZNF626 | zinc finger protein 626 | **0,949** | 0,475839 | 2,936 | 2,235 |
| SECISBP2 | SECIS binding protein 2 | **0,949** | 0,070459 | 32,791 | 33,074 |
| ZNF280D | zinc finger protein 280D | **0,949** | 0,365953 | 9,250 | 6,960 |
| TMEM206 | transmembrane protein 206 | **0,949** | 0,398655 | 5,207 | 6,066 |
| TTC30A | tetratricopeptide repeat domain 30A | **0,949** | 0,217139 | 17,393 | 15,196 |
| MOK | MOK protein kinase | **0,949** | 0,260666 | 6,259 | 6,385 |
| ADGRB2 | adhesion G protein-coupled receptor B2 | **0,949** | 0,62418 | 1,052 | 1,149 |
| PET100 | PET100 homolog | **0,949** | 0,501657 | 3,046 | 3,001 |
| EVI2B | ecotropic viral integration site 2B | **0,949** | 0,575854 | 3,157 | 1,660 |
| SLC10A3 | solute carrier family 10 member 3 | **0,949** | 0,083064 | 34,287 | 20,049 |
| TWISTNB | TWIST neighbor | **0,949** | 0,1062 | 22,544 | 23,241 |
| ZNF516 | zinc finger protein 516 | **0,949** | 0,223324 | 9,084 | 7,790 |
| MSTO1 | misato 1, mitochondrial distribution and morphology regulator | **0,949** | 0,426527 | 5,317 | 5,746 |
| ZFP91 | ZFP91 zinc finger protein | **0,949** | 0,060404 | 63,477 | 57,720 |
| RPL7L1 | ribosomal protein L7 like 1 | **0,949** | 0,041283 | 88,403 | 99,095 |
| SERPINA9 | serpin family A member 9 | **0,949** | 0,696535 | 2,271 | 1,596 |
| TUBGCP3 | tubulin gamma complex associated protein 3 | **0,949** | 0,339015 | 10,912 | 12,578 |
| SF3B3 | splicing factor 3b subunit 3 | **0,949** | 0,056093 | 89,123 | 93,668 |
| CETN2 | centrin 2 | **0,949** | 0,153897 | 45,863 | 40,162 |
| DIO3 | iodothyronine deiodinase 3 | **0,949** | 0,263643 | 15,509 | 24,774 |
| DNAJC25 | DnaJ heat shock protein family (Hsp40) member C25 | **0,949** | 0,111521 | 21,658 | 18,197 |
| USP10 | ubiquitin specific peptidase 10 | **0,949** | 0,052539 | 30,797 | 30,201 |
| MGP | matrix Gla protein | **0,949** | 0,090048 | 2052,815 | 829,027 |
| TEX261 | testis expressed 261 | **0,949** | 0,056198 | 77,823 | 68,447 |
| ALDH9A1 | aldehyde dehydrogenase 9 family member A1 | **0,949** | 0,071401 | 40,213 | 41,055 |
| POLR2L | RNA polymerase II subunit L | **0,949** | 0,183933 | 65,970 | 61,551 |

| | | | | | |
|---|---|---|---|---|---|
| RPL24P2 | ribosomal protein L24 pseudogene 2 | **0,949** | 0,736053 | 0,443 | 0,575 |
| LINC01311 | long intergenic non-protein coding RNA 1311 | **0,948** | 0,728509 | 0,222 | 0,319 |
| TMUB2 | transmembrane and ubiquitin like domain containing 2 | **0,948** | 0,091871 | 27,584 | 26,051 |
| EBPL | emopamil binding protein like | **0,948** | 0,523309 | 5,041 | 4,916 |
| LDHA | lactate dehydrogenase A | **0,948** | 0,057217 | 634,162 | 598,848 |
| CIB2 | calcium and integrin binding family member 2 | **0,948** | 0,483501 | 2,271 | 2,362 |
| TSR2 | TSR2, ribosome maturation factor | **0,948** | 0,152389 | 22,433 | 24,646 |
| ZNF559 | zinc finger protein 559 | **0,948** | 0,217727 | 9,970 | 9,450 |
| GNA11 | G protein subunit alpha 11 | **0,948** | 0,064541 | 76,937 | 61,168 |
| DNAJB12 | DnaJ heat shock protein family (Hsp40) member B12 | **0,948** | 0,036076 | 38,995 | 34,543 |
| CEACAM1 | carcinoembryonic antigen related cell adhesion molecule 1 | **0,948** | 0,789505 | 0,720 | 0,319 |
| COMMD4 | COMM domain containing 4 | **0,948** | 0,118319 | 21,381 | 18,836 |
| NUP93 | nucleoporin 93 | **0,948** | 0,143454 | 16,008 | 18,516 |
| EBAG9 | estrogen receptor binding site associated, antigen, 9 | **0,948** | 0,093831 | 19,719 | 20,304 |
| TIMM23 | translocase of inner mitochondrial membrane 23 | **0,948** | 0,093839 | 21,325 | 20,304 |
| HEXIM2 | hexamethylene bisacetamide inducible 2 | **0,948** | 0,483853 | 2,936 | 3,448 |
| RP9P | retinitis pigmentosa 9 pseudogene | **0,948** | 0,364489 | 3,711 | 3,320 |
| TM9SF3 | transmembrane 9 superfamily member 3 | **0,948** | 0,05141 | 194,973 | 170,607 |
| SLC39A9 | solute carrier family 39 member 9 | **0,948** | 0,041206 | 72,118 | 61,871 |
| STIM1 | stromal interaction molecule 1 | **0,948** | 0,077547 | 24,704 | 15,899 |
| EEF1A1P5 | eukaryotic translation elongation factor 1 alpha 1 pseudogene 5 | **0,948** | 0,436531 | 5,041 | 3,895 |
| HLTF | helicase like transcription factor | **0,948** | 0,066521 | 23,707 | 27,072 |
| CPXM2 | carboxypeptidase X, M14 family member 2 | **0,948** | 0,062143 | 38,108 | 45,142 |
| CNP | 2',3'-cyclic nucleotide 3' phosphodiesterase | **0,948** | 0,040007 | 42,650 | 29,946 |
| CMC1 | C-X9-C motif containing 1 | **0,948** | 0,173728 | 12,075 | 15,324 |
| RPL35A | ribosomal protein L35a | **0,948** | 0,109981 | 151,381 | 148,834 |
| SLC26A6 | solute carrier family 26 member 6 | **0,948** | 0,173784 | 17,448 | 16,537 |
| ZKSCAN2 | zinc finger with KRAB and SCAN domains 2 | **0,948** | 0,302886 | 8,752 | 8,045 |
| CASP2 | caspase 2 | **0,948** | 0,262252 | 10,580 | 9,258 |
| RFC2 | replication factor C subunit 2 | **0,948** | 0,233568 | 9,250 | 9,450 |
| TMEM167B | transmembrane protein 167B | **0,948** | 0,07842 | 62,757 | 50,314 |
| CSE1L | chromosome segregation 1 like | **0,948** | 0,048625 | 54,449 | 59,125 |
| SRF | serum response factor | **0,948** | 0,069464 | 48,356 | 42,652 |

| | | | | | |
|---|---|---|---|---|---|
| NCDN | neurochondrin | **0,948** | 0,085623 | 47,746 | 47,951 |
| ZNF573 | zinc finger protein 573 | **0,948** | 0,619311 | 0,942 | 1,469 |
| CCDC18 | coiled-coil domain containing 18 | **0,948** | 0,456388 | 3,268 | 2,937 |
| ATRAID | all-trans retinoic acid induced differentiation factor | **0,948** | 0,031412 | 92,779 | 55,677 |
| TMEM63B | transmembrane protein 63B | **0,948** | 0,085161 | 32,348 | 26,306 |
| RELN | reelin | **0,948** | 0,44111 | 4,154 | 2,554 |
| COA1 | cytochrome c oxidase assembly factor 1 homolog | **0,948** | 0,124854 | 21,990 | 25,540 |
| RPL4 | ribosomal protein L4 | **0,948** | 0,064592 | 400,969 | 341,916 |
| ATG4A | autophagy related 4A cysteine peptidase | **0,948** | 0,238323 | 7,256 | 6,577 |
| TM9SF4 | transmembrane 9 superfamily member 4 | **0,948** | 0,036636 | 76,051 | 61,360 |
| DPY19L1 | dpy-19 like 1 | **0,948** | 0,050926 | 51,568 | 37,991 |
| PIK3CD | phosphatidylinositol-4,5-bisphosphate 3-kinase catalytic subunit delta | **0,948** | 0,274395 | 18,445 | 11,238 |
| PITPNM2 | phosphatidylinositol transfer protein membrane associated 2 | **0,948** | 0,226099 | 12,574 | 13,472 |
| CAPRIN1 | cell cycle associated protein 1 | **0,947** | 0,05801 | 133,158 | 113,142 |
| NUP35 | nucleoporin 35 | **0,947** | 0,121081 | 13,294 | 17,239 |
| KCNMA1 | potassium calcium-activated channel subfamily M alpha 1 | **0,947** | 0,038927 | 286,478 | 259,486 |
| DCAKD | dephospho-CoA kinase domain containing | **0,947** | 0,180795 | 16,340 | 14,558 |
| DEDD | death effector domain containing | **0,947** | 0,177591 | 15,842 | 15,707 |
| CDC42BPA | CDC42 binding protein kinase alpha | **0,947** | 0,144171 | 115,101 | 96,030 |
| RPLP0 | ribosomal protein lateral stalk subunit P0 | **0,947** | 0,09958 | 725,223 | 696,155 |
| UTP6 | UTP6, small subunit processome component | **0,947** | 0,082124 | 32,625 | 35,628 |
| MRPL36 | mitochondrial ribosomal protein L36 | **0,947** | 0,34192 | 8,087 | 7,534 |
| TRIM11 | tripartite motif containing 11 | **0,947** | 0,11524 | 23,873 | 27,200 |
| VPS4A | vacuolar protein sorting 4 homolog A | **0,947** | 0,119842 | 23,153 | 21,134 |
| RPA3 | replication protein A3 | **0,947** | 0,14244 | 15,842 | 19,283 |
| BRK1 | BRICK1, SCAR/WAVE actin nucleating complex subunit | **0,947** | 0,086261 | 104,078 | 90,284 |
| CSF1 | colony stimulating factor 1 | **0,947** | 0,086656 | 4652,497 | 5325,335 |
| JKAMP | JNK1/MAPK8-associated membrane protein | **0,947** | 0,096566 | 45,752 | 43,418 |
| MMP13 | matrix metallopeptidase 13 | **0,947** | 0,04247 | 740,622 | 251,313 |
| 1.maalis | membrane associated ring-CH-type finger 1 | **0,947** | 0,750839 | 0,000 | 0,192 |
| ADM | adrenomedullin | **0,947** | 0,150322 | 202,673 | 120,229 |
| SDCBP2 | syndecan binding protein 2 | **0,947** | 0,471706 | 2,271 | 1,852 |
| DOCK6 | dedicator of cytokinesis 6 | **0,947** | 0,252377 | 9,527 | 7,917 |

| | | | | | |
|---|---|---|---|---|---|
| HAP1 | huntingtin associated protein 1 | **0,947** | 0,645129 | 1,163 | 0,894 |
| MFSD13A | major facilitator superfamily domain containing 13A | **0,947** | 0,656821 | 1,440 | 1,277 |
| SHARPIN | SHANK associated RH domain interactor | **0,947** | 0,149004 | 19,276 | 15,771 |
| MRPL39 | mitochondrial ribosomal protein L39 | **0,947** | 0,210063 | 10,358 | 12,068 |
| ARPC2 | actin related protein 2/3 complex subunit 2 | **0,947** | 0,029584 | 173,593 | 135,681 |
| MFSD7 | major facilitator superfamily domain containing 7 | **0,947** | 0,49347 | 4,154 | 3,895 |
| ZC3H7A | zinc finger CCCH-type containing 7A | **0,947** | 0,050382 | 68,739 | 62,956 |
| NDUFS7 | NADH:ubiquinone oxidoreductase core subunit S7 | **0,947** | 0,192642 | 17,669 | 14,622 |
| CLEC11A | C-type lectin domain containing 11A | **0,947** | 0,403159 | 5,927 | 4,214 |
| SEC22B | SEC22 homolog B, vesicle trafficking protein (gene/pseudogene) | **0,947** | 0,043734 | 86,298 | 76,939 |
| P4HA3 | prolyl 4-hydroxylase subunit alpha 3 | **0,947** | 0,192597 | 11,078 | 11,429 |
| TECPR1 | tectonin beta-propeller repeat containing 1 | **0,947** | 0,205295 | 17,669 | 19,921 |
| LMAN2L | lectin, mannose binding 2 like | **0,947** | 0,102993 | 21,436 | 23,752 |
| SNHG26 | small nucleolar RNA host gene 26 | **0,947** | 0,557746 | 1,606 | 2,043 |
| STT3B | STT3B, catalytic subunit of the oligosaccharyltransferase complex | **0,947** | 0,068971 | 200,679 | 152,282 |
| NFYC-AS1 | NFYC antisense RNA 1 | **0,947** | 0,702488 | 0,499 | 0,702 |
| PDAP1 | PDGFA associated protein 1 | **0,947** | 0,092866 | 86,353 | 86,133 |
| CHDH | choline dehydrogenase | **0,947** | 0,31985 | 6,813 | 5,108 |
| MTA3 | metastasis associated 1 family member 3 | **0,947** | 0,280418 | 11,355 | 7,407 |
| UBE2D4 | ubiquitin conjugating enzyme E2 D4 (putative) | **0,947** | 0,127275 | 18,943 | 17,367 |
| PTRH1 | peptidyl-tRNA hydrolase 1 homolog | **0,947** | 0,243486 | 9,804 | 9,961 |
| METTL5 | methyltransferase like 5 | **0,947** | 0,212032 | 14,457 | 15,005 |
| AHSA1 | activator of HSP90 ATPase activity 1 | **0,947** | 0,095503 | 52,067 | 51,527 |
| ZMPSTE24 | zinc metallopeptidase STE24 | **0,947** | 0,042931 | 61,206 | 44,056 |
| PSMF1 | proteasome inhibitor subunit 1 | **0,947** | 0,02732 | 68,462 | 67,617 |
| CASP8 | caspase 8 | **0,947** | 0,388774 | 5,816 | 7,470 |
| C3orf70 | chromosome 3 open reading frame 70 | **0,947** | 0,379769 | 6,370 | 5,427 |
| ASTE1 | asteroid homolog 1 (Drosophila) | **0,947** | 0,421169 | 3,767 | 4,150 |
| HARS | histidyl-tRNA synthetase | **0,947** | 0,071274 | 38,275 | 40,928 |
| ZNF581 | zinc finger protein 581 | **0,947** | 0,269668 | 6,868 | 4,597 |
| S100P | S100 calcium binding protein P | **0,947** | 0,462699 | 2,659 | 2,299 |
| NAGA | alpha-N-acetylgalactosaminidase | **0,947** | 0,08949 | 20,661 | 10,791 |
| UQCC2 | ubiquinol-cytochrome c reductase complex assembly factor 2 | **0,947** | 0,141804 | 18,390 | 21,581 |

| | | | | | |
|---|---|---|---|---|---|
| GKAP1 | G kinase anchoring protein 1 | **0,947** | 0,500472 | 2,437 | 1,532 |
| LRMDA | leucine rich melanocyte differentiation associated | **0,947** | 0,38732 | 4,708 | 3,895 |
| NDUFAF6 | NADH:ubiquinone oxidoreductase complex assembly factor 6 | **0,947** | 0,376951 | 3,490 | 3,192 |
| SUPT7L | SPT7 like, STAGA complex gamma subunit | **0,947** | 0,078059 | 26,975 | 25,093 |
| CMC2 | C-X9-C motif containing 2 | **0,946** | 0,263707 | 9,361 | 10,599 |
| MSL2 | male-specific lethal 2 homolog (Drosophila) | **0,946** | 0,051661 | 29,080 | 29,052 |
| UCHL3 | ubiquitin C-terminal hydrolase L3 | **0,946** | 0,592511 | 1,772 | 1,788 |
| APPL2 | adaptor protein, phosphotyrosine interacting with PH domain and leucine zipper 2 | **0,946** | 0,068358 | 34,508 | 32,053 |
| LTBR | lymphotoxin beta receptor | **0,946** | 0,035209 | 72,783 | 40,609 |
| XRRA1 | X-ray radiation resistance associated 1 | **0,946** | 0,138484 | 17,669 | 17,239 |
| DOCK9 | dedicator of cytokinesis 9 | **0,946** | 0,250028 | 21,270 | 20,304 |
| DDX10 | DEAD-box helicase 10 | **0,946** | 0,144106 | 12,518 | 17,303 |
| EPHB1 | EPH receptor B1 | **0,946** | 0,584378 | 2,493 | 1,724 |
| CLCC1 | chloride channel CLIC like 1 | **0,946** | 0,094733 | 39,382 | 32,627 |
| IAH1 | isoamyl acetate-hydrolyzing esterase 1 homolog | **0,946** | 0,159934 | 12,684 | 8,237 |
| SLC1A4 | solute carrier family 1 member 4 | **0,946** | 0,050233 | 118,812 | 113,717 |
| SDCCAG8 | serologically defined colon cancer antigen 8 | **0,946** | 0,695656 | 0,942 | 0,702 |
| CD151 | CD151 molecule (Raph blood group) | **0,946** | 0,036861 | 277,560 | 178,333 |
| BLMH | bleomycin hydrolase | **0,946** | 0,204552 | 10,081 | 6,257 |
| KIF2C | kinesin family member 2C | **0,946** | 0,527071 | 5,096 | 1,022 |
| PGAM1 | phosphoglycerate mutase 1 | **0,946** | 0,04943 | 50,350 | 43,354 |
| FAIM | Fas apoptotic inhibitory molecule | **0,946** | 0,345969 | 5,428 | 5,044 |
| POT1-AS1 | POT1 antisense RNA 1 | **0,946** | 0,790854 | 0,222 | 0,255 |
| ALKBH3 | alkB homolog 3, alpha-ketoglutaratedependent dioxygenase | **0,946** | 0,13347 | 15,343 | 15,005 |
| ABCB8 | ATP binding cassette subfamily B member 8 | **0,946** | 0,078157 | 24,316 | 30,839 |
| CCDC28B | coiled-coil domain containing 28B | **0,946** | 0,631846 | 1,329 | 1,022 |
| FEM1A | fem-1 homolog A | **0,946** | 0,090436 | 34,840 | 38,693 |
| ZNF689 | zinc finger protein 689 | **0,946** | 0,241165 | 7,422 | 7,598 |
| SRP54 | signal recognition particle 54 | **0,946** | 0,032757 | 81,091 | 73,364 |
| CCDC3 | coiled-coil domain containing 3 | **0,946** | 0,296033 | 7,699 | 9,003 |
| PRKD1 | protein kinase D1 | **0,946** | 0,206981 | 8,364 | 9,131 |
| HMX3 | H6 family homeobox 3 | **0,946** | 0,793091 | 0,443 | 0,192 |
| METTL13 | methyltransferase like 13 | **0,946** | 0,087109 | 29,523 | 25,732 |

| | | | | | |
|---|---|---|---|---|---|
| GYG1 | glycogenin 1 | **0,946** | 0,033159 | 49,907 | 36,331 |
| EIF3F | eukaryotic translation initiation factor 3 subunit F | **0,946** | 0,038678 | 50,405 | 52,868 |
| NFKB2 | nuclear factor kappa B subunit 2 | **0,946** | 0,048419 | 272,076 | 254,633 |
| AP1B1 | adaptor related protein complex 1 beta 1 subunit | **0,946** | 0,062648 | 46,528 | 39,587 |
| ANKS6 | ankyrin repeat and sterile alpha motif domain containing 6 | **0,946** | 0,080219 | 27,252 | 36,522 |
| TRPM4 | transient receptor potential cation channel subfamily M member 4 | **0,946** | 0,466709 | 3,877 | 2,682 |
| TEX26-AS1 | TEX26 antisense RNA 1 | **0,946** | 0,765556 | 0,111 | 0,319 |
| PDXDC2P-NPIPB1 | nuclear pore complex-interacting protein | **0,946** | 0,71715 | 0,720 | 1,341 |
| MRPS9 | mitochondrial ribosomal protein S9 | **0,945** | 0,135685 | 13,017 | 16,729 |
| CEP19 | centrosomal protein 19 | **0,945** | 0,358928 | 5,871 | 3,576 |
| TRIM8 | tripartite motif containing 8 | **0,945** | 0,029432 | 130,333 | 119,399 |
| PTP4A2 | protein tyrosine phosphatase type IVA, member 2 | **0,945** | 0,024048 | 201,565 | 179,865 |
| 2.maalis | mitochondrial amidoxime reducing component 2 | **0,945** | 0,333754 | 3,600 | 3,384 |
| SLC38A2 | solute carrier family 38 member 2 | **0,945** | 0,101651 | 355,826 | 224,943 |
| ATP5G3 | ATP synthase, H+ transporting, mitochondrial Fo complex subunit C3 (subunit 9) | **0,945** | 0,126982 | 39,826 | 38,885 |
| GNB5 | G protein subunit beta 5 | **0,945** | 0,111468 | 16,285 | 13,600 |
| ANAPC7 | anaphase promoting complex subunit 7 | **0,945** | 0,054762 | 20,937 | 19,027 |
| NAA15 | N(alpha)-acetyltransferase 15, NatA auxiliary subunit | **0,945** | 0,141649 | 31,185 | 31,095 |
| DNAJC1 | DnaJ heat shock protein family (Hsp40) member C1 | **0,945** | 0,073969 | 70,512 | 74,385 |
| MNAT1 | MNAT1, CDK activating kinase assembly factor | **0,945** | 0,17177 | 15,786 | 15,005 |
| ERCC3 | ERCC excision repair 3, TFIIH core complex helicase subunit | **0,945** | 0,07983 | 35,893 | 33,393 |
| WBSCR17 | Williams-Beuren syndrome chromosome region 17 | **0,945** | 0,367898 | 7,145 | 5,746 |
| POLR2J | RNA polymerase II subunit J | **0,945** | 0,219276 | 13,903 | 15,643 |
| NFKB1 | nuclear factor kappa B subunit 1 | **0,945** | 0,037278 | 116,873 | 115,824 |
| GLO1 | glyoxalase I | **0,945** | 0,048009 | 59,489 | 57,656 |
| UBE2Q1 | ubiquitin conjugating enzyme E2 Q1 | **0,945** | 0,034893 | 46,694 | 39,842 |
| TMEM187 | transmembrane protein 187 | **0,945** | 0,390994 | 6,868 | 4,023 |
| YRDC | yrdC N6-threonylcarbamoyltransferase domain containing | **0,945** | 0,137947 | 18,666 | 17,686 |
| JAZF1 | JAZF zinc finger 1 | **0,945** | 0,267366 | 9,582 | 5,044 |
| MYO1C | myosin IC | **0,945** | 0,073414 | 171,266 | 92,582 |
| MFF | mitochondrial fission factor | **0,945** | 0,135019 | 21,159 | 19,793 |
| TOE1 | target of EGR1, member 1 (nuclear) | **0,945** | 0,228315 | 8,198 | 9,386 |
| SUDS3 | SDS3 homolog, SIN3A corepressor complex component | **0,945** | 0,074905 | 17,282 | 13,536 |

| | | | | | |
|---|---|---|---|---|---|
| WDR5 | WD repeat domain 5 | **0,945** | 0,054555 | 22,156 | 26,370 |
| B3GNT7 | UDP-GlcNAc:betaGal beta-1,3-N-acetylglucosaminyltransferase 7 | **0,945** | 0,108632 | 152,212 | 101,841 |
| PRPF3 | pre-mRNA processing factor 3 | **0,945** | 0,06445 | 27,861 | 31,925 |
| TYK2 | tyrosine kinase 2 | **0,945** | 0,060882 | 44,035 | 41,822 |
| TAF8 | TATA-box binding protein associated factor 8 | **0,945** | 0,147928 | 18,057 | 19,027 |
| CFAP44 | cilia and flagella associated protein 44 | **0,945** | 0,426719 | 4,320 | 5,491 |
| FOXK2 | forkhead box K2 | **0,945** | 0,046483 | 39,216 | 35,309 |
| ZNF423 | zinc finger protein 423 | **0,945** | 0,572338 | 1,883 | 2,235 |
| SRSF3 | serine and arginine rich splicing factor 3 | **0,945** | 0,072082 | 76,383 | 78,599 |
| PHYKPL | 5-phosphohydroxy-L-lysine phospho-lyase | **0,945** | 0,11979 | 19,553 | 17,176 |
| CPT1A | carnitine palmitoyltransferase 1A | **0,945** | 0,171605 | 37,111 | 16,856 |
| BACH1-IT1 | BACH1 intronic transcript 1 | **0,945** | 0,673682 | 0,775 | 0,702 |
| TBC1D19 | TBC1 domain family member 19 | **0,945** | 0,465409 | 3,822 | 2,682 |
| MAPRE3 | microtubule associated protein RP/EB family member 3 | **0,945** | 0,113801 | 16,008 | 12,195 |
| STK24 | serine/threonine kinase 24 | **0,945** | 0,036421 | 98,982 | 86,133 |
| TTC28 | tetratricopeptide repeat domain 28 | **0,945** | 0,171067 | 18,611 | 17,431 |
| ASPN | asporin | **0,945** | 0,595343 | 0,166 | 1,915 |
| AADAT | aminoadipate aminotransferase | **0,945** | 0,492553 | 2,049 | 1,532 |
| TMEM129 | transmembrane protein 129 | **0,945** | 0,080644 | 27,252 | 24,518 |
| ACTR1B | ARP1 actin-related protein 1 homolog B, centractin beta | **0,945** | 0,050723 | 42,706 | 39,842 |
| NDUFB9 | NADH:ubiquinone oxidoreductase subunit B9 | **0,945** | 0,123643 | 39,438 | 43,226 |
| CPT2 | carnitine palmitoyltransferase 2 | **0,945** | 0,171379 | 11,355 | 12,578 |
| PPP2R2D | protein phosphatase 2 regulatory subunit Bdelta | **0,945** | 0,088323 | 14,789 | 14,239 |
| MAP3K11 | mitogen-activated protein kinase kinase kinase 11 | **0,944** | 0,103155 | 27,197 | 26,562 |
| SYCP2 | synaptonemal complex protein 2 | **0,944** | 0,444281 | 2,603 | 2,426 |
| ZFYVE19 | zinc finger FYVE-type containing 19 | **0,944** | 0,219909 | 11,133 | 10,854 |
| GSEC | G-quadruplex forming sequence containing lncRNA | **0,944** | 0,772326 | 0,665 | 0,255 |
| PCYT2 | phosphate cytidylyltransferase 2, ethanolamine | **0,944** | 0,288367 | 20,993 | 17,112 |
| ARMCX5-GPRASI | ARMCX5-GPRASP2 readthrough | **0,944** | 0,385207 | 2,271 | 2,362 |
| UBE2T | ubiquitin conjugating enzyme E2 T | **0,944** | 0,46577 | 2,659 | 1,979 |
| AKT2 | AKT serine/threonine kinase 2 | **0,944** | 0,025206 | 78,211 | 63,978 |
| BLVRA | biliverdin reductase A | **0,944** | 0,209157 | 17,669 | 14,494 |
| HEMK1 | HemK methyltransferase family member 1 | **0,944** | 0,123111 | 18,666 | 26,881 |

| | | | | | |
|---|---|---|---|---|---|
| LTBP4 | latent transforming growth factor beta binding protein 4 | **0,944** | 0,073615 | 88,347 | 84,665 |
| SLC30A7 | solute carrier family 30 member 7 | **0,944** | 0,065527 | 37,942 | 34,351 |
| TMEM143 | transmembrane protein 143 | **0,944** | 0,416593 | 2,326 | 2,107 |
| LINC00339 | long intergenic non-protein coding RNA 339 | **0,944** | 0,169247 | 6,979 | 6,768 |
| FGF2 | fibroblast growth factor 2 | **0,944** | 0,104854 | 866,468 | 780,501 |
| RPL8 | ribosomal protein L8 | **0,944** | 0,094427 | 476,743 | 440,820 |
| KRT15 | keratin 15 | **0,944** | 0,589037 | 0,499 | 1,341 |
| LRRC73 | leucine rich repeat containing 73 | **0,944** | 0,752858 | 0,665 | 0,958 |
| TPD52L2 | tumor protein D52 like 2 | **0,944** | 0,023806 | 164,952 | 162,817 |
| AGFG2 | ArfGAP with FG repeats 2 | **0,944** | 0,067048 | 29,246 | 34,351 |
| HDLBP | high density lipoprotein binding protein | **0,944** | 0,06734 | 557,779 | 454,803 |
| IBA57 | IBA57 homolog, iron-sulfur cluster assembly | **0,944** | 0,220533 | 8,641 | 10,663 |
| NUDT5 | nudix hydrolase 5 | **0,944** | 0,05112 | 19,220 | 21,198 |
| SRXN1 | sulfiredoxin 1 | **0,944** | 0,447916 | 4,542 | 3,448 |
| SEC23IP | SEC23 interacting protein | **0,944** | 0,033813 | 46,306 | 42,396 |
| HFM1 | HFM1, ATP dependent DNA helicase homolog | **0,944** | 0,726502 | 0,332 | 0,575 |
| GGCX | gamma-glutamyl carboxylase | **0,944** | 0,061512 | 43,592 | 42,013 |
| PJA1 | praja ring finger ubiquitin ligase 1 | **0,944** | 0,058667 | 22,101 | 18,006 |
| SMG5 | SMG5, nonsense mediated mRNA decay factor | **0,944** | 0,04224 | 78,377 | 77,195 |
| ZCCHC3 | zinc finger CCHC-type containing 3 | **0,944** | 0,072033 | 19,608 | 22,092 |
| NRDE2 | NRDE-2, necessary for RNA interference, domain containing | **0,944** | 0,111005 | 20,937 | 17,623 |
| TMEM44-AS1 | TMEM44 antisense RNA 1 | **0,944** | 0,391118 | 2,437 | 3,065 |
| PRSS27 | protease, serine 27 | **0,944** | 0,625732 | 1,052 | 1,277 |
| METTL18 | methyltransferase like 18 | **0,944** | 0,251114 | 5,816 | 6,513 |
| ARPIN | actin-related protein 2/3 complex inhibitor | **0,944** | 0,271547 | 8,475 | 5,810 |
| PLPBP | pyridoxal phosphate binding protein | **0,944** | 0,060604 | 49,907 | 51,144 |
| LRRC41 | leucine rich repeat containing 41 | **0,944** | 0,037823 | 53,562 | 43,673 |
| CNTNAP1 | contactin associated protein 1 | **0,944** | 0,114395 | 20,051 | 17,495 |
| PEX26 | peroxisomal biogenesis factor 26 | **0,944** | 0,057215 | 32,126 | 28,541 |
| FAM114A2 | family with sequence similarity 114 member A2 | **0,944** | 0,070897 | 21,436 | 24,391 |
| TENM2 | teneurin transmembrane protein 2 | **0,944** | 0,569229 | 1,828 | 3,639 |
| DDX46 | DEAD-box helicase 46 | **0,944** | 0,042545 | 42,761 | 34,479 |
| TMEM240 | transmembrane protein 240 | **0,944** | 0,754809 | 0,332 | 0,575 |

| | | | | | |
|---|---|---|---|---|---|
| ZNF792 | zinc finger protein 792 | **0,944** | 0,71807 | 0,277 | 0,511 |
| CCAR2 | cell cycle and apoptosis regulator 2 | **0,943** | 0,048428 | 47,359 | 44,376 |
| NCAPG2 | non-SMC condensin II complex subunit G2 | **0,943** | 0,281947 | 12,851 | 5,810 |
| YEATS2 | YEATS domain containing 2 | **0,943** | 0,107144 | 24,759 | 22,475 |
| DAZAP1 | DAZ associated protein 1 | **0,943** | 0,035336 | 57,827 | 60,274 |
| RIOK1 | RIO kinase 1 | **0,943** | 0,105055 | 14,734 | 18,580 |
| ITPKC | inositol-trisphosphate 3-kinase C | **0,943** | 0,067743 | 32,569 | 32,500 |
| TMX2 | thioredoxin related transmembrane protein 2 | **0,943** | 0,047596 | 58,492 | 67,553 |
| TSPAN31 | tetraspanin 31 | **0,943** | 0,029589 | 37,278 | 32,244 |
| CFL1 | cofilin 1 | **0,943** | 0,078402 | 273,295 | 231,647 |
| ISM1 | isthmin 1 | **0,943** | 0,037231 | 68,850 | 63,084 |
| GABPB1-AS1 | GABPB1 antisense RNA 1 | **0,943** | 0,098711 | 18,390 | 22,028 |
| FKBP9 | FK506 binding protein 9 | **0,943** | 0,069618 | 65,748 | 33,585 |
| TMEM123 | transmembrane protein 123 | **0,943** | 0,032411 | 89,400 | 63,658 |
| RPS6KB2 | ribosomal protein S6 kinase B2 | **0,943** | 0,078926 | 15,842 | 13,153 |
| CCM2 | CCM2 scaffolding protein | **0,943** | 0,0869 | 47,857 | 33,330 |
| TRAPPC2L | trafficking protein particle complex 2 like | **0,943** | 0,157898 | 14,014 | 16,920 |
| TP53INP2 | tumor protein p53 inducible nuclear protein 2 | **0,943** | 0,049622 | 34,730 | 22,986 |
| C11orf71 | chromosome 11 open reading frame 71 | **0,943** | 0,142777 | 9,250 | 11,493 |
| MKL2 | MKL1/myocardin like 2 | **0,943** | 0,294761 | 6,813 | 6,321 |
| SEC63 | SEC63 homolog, protein translocation regulator | **0,943** | 0,026391 | 128,117 | 120,996 |
| PARP1 | poly(ADP-ribose) polymerase 1 | **0,943** | 0,109925 | 36,281 | 34,670 |
| MRPL19 | mitochondrial ribosomal protein L19 | **0,943** | 0,083317 | 14,512 | 15,132 |
| LINC00467 | long intergenic non-protein coding RNA 467 | **0,943** | 0,267593 | 5,428 | 6,960 |
| AGPS | alkylglycerone phosphate synthase | **0,943** | 0,123493 | 28,083 | 20,177 |
| FUK | fucokinase | **0,943** | 0,276865 | 7,644 | 7,726 |
| GEMIN5 | gem nuclear organelle associated protein 5 | **0,943** | 0,083903 | 19,940 | 20,560 |
| TBC1D3L | TBC1 domain family member 3L | **0,943** | 0,806841 | 0,332 | 0,128 |
| AGPAT3 | 1-acylglycerol-3-phosphate O-acyltransferase 3 | **0,943** | 0,034854 | 46,085 | 38,182 |
| FBXO11 | F-box protein 11 | **0,943** | 0,031391 | 54,504 | 54,336 |
| NUPL2 | nucleoporin like 2 | **0,943** | 0,238326 | 9,416 | 6,960 |
| SRSF1 | serine and arginine rich splicing factor 1 | **0,943** | 0,025278 | 78,876 | 75,151 |
| APCDD1 | APC down-regulated 1 | **0,943** | 0,545966 | 2,382 | 1,277 |

| | | | | | |
|---|---|---|---|---|---|
| UBAC1 | UBA domain containing 1 | **0,943** | 0,203616 | 13,072 | 11,876 |
| PSMD8 | proteasome 26S subunit, non-ATPase 8 | **0,943** | 0,025852 | 116,652 | 115,824 |
| ZCCHC17 | zinc finger CCHC-type containing 17 | **0,943** | 0,044077 | 28,526 | 26,434 |
| TRAM2-AS1 | TRAM2 antisense RNA 1 (head to head) | **0,943** | 0,339335 | 6,813 | 7,023 |
| C19orf43 | chromosome 19 open reading frame 43 | **0,943** | 0,065799 | 84,747 | 79,685 |
| LINC00094 | long intergenic non-protein coding RNA 94 | **0,943** | 0,146099 | 18,279 | 12,770 |
| PROCR | protein C receptor | **0,943** | 0,037355 | 43,980 | 37,288 |
| ASXL1 | additional sex combs like 1, transcriptional regulator | **0,943** | 0,067919 | 57,384 | 54,783 |
| DERA | deoxyribose-phosphate aldolase | **0,943** | 0,124621 | 15,066 | 12,706 |
| RPS5 | ribosomal protein S5 | **0,943** | 0,107316 | 177,249 | 140,470 |
| TIMM8B | translocase of inner mitochondrial membrane 8 homolog B | **0,943** | 0,117565 | 13,404 | 12,642 |
| COA6 | cytochrome c oxidase assembly factor 6 | **0,943** | 0,279317 | 7,921 | 10,663 |
| COX5A | cytochrome c oxidase subunit 5A | **0,943** | 0,08962 | 30,686 | 22,156 |
| ZNF146 | zinc finger protein 146 | **0,943** | 0,032093 | 64,862 | 61,807 |
| PPP1R7 | protein phosphatase 1 regulatory subunit 7 | **0,943** | 0,101479 | 22,599 | 25,285 |
| PER2 | period circadian clock 2 | **0,943** | 0,184547 | 17,226 | 16,537 |
| SETMAR | SET domain and mariner transposase fusion gene | **0,942** | 0,458536 | 3,323 | 2,362 |
| BRCA2 | BRCA2, DNA repair associated | **0,942** | 0,356354 | 7,478 | 6,896 |
| TMEM120A | transmembrane protein 120A | **0,942** | 0,033026 | 22,211 | 17,623 |
| TMEM132A | transmembrane protein 132A | **0,942** | 0,097293 | 79,706 | 57,082 |
| RBFADN | RBFA downstream neighbor (non-protein coding) | **0,942** | 0,711284 | 0,609 | 0,575 |
| ZNF552 | zinc finger protein 552 | **0,942** | 0,438656 | 2,991 | 3,065 |
| RAP1GAP2 | RAP1 GTPase activating protein 2 | **0,942** | 0,049298 | 40,102 | 38,629 |
| MIR4458HG | MIR4458 host gene | **0,942** | 0,493405 | 2,493 | 3,065 |
| PELO | pelota homolog (Drosophila) | **0,942** | 0,030216 | 60,264 | 56,379 |
| MEG3 | maternally expressed 3 (non-protein coding) | **0,942** | 0,195077 | 82,254 | 46,355 |
| MORN4 | MORN repeat containing 4 | **0,942** | 0,227929 | 12,241 | 12,962 |
| UNC50 | unc-50 inner nuclear membrane RNA binding protein | **0,942** | 0,064112 | 32,292 | 31,223 |
| TLK2 | tousled like kinase 2 | **0,942** | 0,130312 | 15,343 | 14,366 |
| STARD3NL | STARD3 N-terminal like | **0,942** | 0,06677 | 14,789 | 13,345 |
| GUK1 | guanylate kinase 1 | **0,942** | 0,054712 | 64,806 | 57,720 |
| UQCRFS1 | ubiquinol-cytochrome c reductase, Rieske iron-sulfur polypeptide 1 | **0,942** | 0,068521 | 30,465 | 30,073 |
| SMG1P7 | SMG1P7, nonsense mediated mRNA decay associated PI3K related kinase pseudogene 7 | **0,942** | 0,54047 | 1,274 | 2,043 |

| | | | | | |
|---|---|---|---|---|---|
| ANGPTL2 | angiopoietin like 2 | **0,942** | 0,063997 | 114,768 | 51,463 |
| ZPLD1 | zona pellucida like domain containing 1 | **0,942** | 0,492429 | 0,665 | 2,043 |
| MTHFSD | methenyltetrahydrofolate synthetase domain containing | **0,942** | 0,125444 | 11,466 | 12,962 |
| IMPDH1 | inosine monophosphate dehydrogenase 1 | **0,942** | 0,170741 | 12,241 | 11,557 |
| COPS9 | COP9 signalosome subunit 9 | **0,942** | 0,133562 | 20,273 | 18,453 |
| PLAC8L1 | PLAC8 like 1 | **0,942** | 0,739745 | 0,332 | 0,575 |
| PTPN11 | protein tyrosine phosphatase, non-receptor type 11 | **0,942** | 0,052785 | 91,283 | 80,706 |
| ZRANB2-AS2 | ZRANB2 antisense RNA 2 (head to head) | **0,942** | 0,671357 | 0,499 | 0,511 |
| LRRC37BP1 | leucine rich repeat containing 37B pseudogene 1 | **0,942** | 0,494702 | 2,160 | 1,341 |
| DDA1 | DET1 and DDB1 associated 1 | **0,942** | 0,042341 | 34,785 | 33,074 |
| PMS1 | PMS1 homolog 1, mismatch repair system component | **0,942** | 0,179915 | 9,859 | 11,429 |
| TVP23C | trans-golgi network vesicle protein 23 homolog C | **0,942** | 0,362025 | 4,265 | 5,300 |
| MMP25-AS1 | MMP25 antisense RNA 1 | **0,942** | 0,514279 | 2,603 | 2,554 |
| LYPLA1 | lysophospholipase I | **0,942** | 0,222185 | 22,932 | 15,516 |
| PSMA1 | proteasome subunit alpha 1 | **0,942** | 0,13109 | 15,675 | 14,047 |
| SAE1 | SUMO1 activating enzyme subunit 1 | **0,942** | 0,04147 | 41,432 | 39,204 |
| UBALD1 | UBA like domain containing 1 | **0,942** | 0,125175 | 12,518 | 13,345 |
| HNRNPA3 | heterogeneous nuclear ribonucleoprotein A3 | **0,942** | 0,02035 | 85,744 | 83,005 |
| TUBGCP2 | tubulin gamma complex associated protein 2 | **0,942** | 0,049135 | 43,426 | 30,329 |
| CDC40 | cell division cycle 40 | **0,942** | 0,083226 | 17,780 | 18,261 |
| NBAS | neuroblastoma amplified sequence | **0,942** | 0,087979 | 30,409 | 25,732 |
| TTC12 | tetratricopeptide repeat domain 12 | **0,942** | 0,17805 | 10,469 | 6,257 |
| COX20 | COX20, cytochrome c oxidase assembly factor | **0,942** | 0,054469 | 47,192 | 47,057 |
| PRRT3-AS1 | PRRT3 antisense RNA 1 | **0,942** | 0,640752 | 1,385 | 1,277 |
| EXOSC8 | exosome component 8 | **0,942** | 0,059811 | 17,171 | 18,516 |
| BAX | BCL2 associated X, apoptosis regulator | **0,942** | 0,05436 | 32,459 | 33,393 |
| MIR762HG | MIR762 host gene | **0,942** | 0,634551 | 0,886 | 0,894 |
| IPO5P1 | importin 5 pseudogene 1 | **0,942** | 0,364223 | 2,991 | 3,576 |
| C8orf44 | chromosome 8 open reading frame 44 | **0,942** | 0,520177 | 1,939 | 1,341 |
| TRIP11 | thyroid hormone receptor interactor 11 | **0,942** | 0,048469 | 81,534 | 75,407 |
| TAF6 | TATA-box binding protein associated factor 6 | **0,942** | 0,091591 | 30,797 | 32,500 |
| C7orf26 | chromosome 7 open reading frame 26 | **0,942** | 0,145978 | 12,629 | 16,154 |
| C4orf33 | chromosome 4 open reading frame 33 | **0,941** | 0,546688 | 1,329 | 0,511 |

| | | | | | |
|---|---|---|---|---|---|
| SLC26A4 | solute carrier family 26 member 4 | **0,941** | 0,462957 | 2,825 | 1,788 |
| ATP5I | ATP synthase, H+ transporting, mitochondrial Fo complex subunit E | **0,941** | 0,184378 | 26,366 | 24,646 |
| DEDD2 | death effector domain containing 2 | **0,941** | 0,038441 | 30,409 | 29,882 |
| BBS7 | Bardet-Biedl syndrome 7 | **0,941** | 0,183833 | 12,241 | 8,300 |
| P4HA1 | prolyl 4-hydroxylase subunit alpha 1 | **0,941** | 0,01428 | 327,633 | 263,381 |
| BHMT2 | betaine--homocysteine S-methyltransferase 2 | **0,941** | 0,66568 | 0,886 | 1,213 |
| RBM8A | RNA binding motif protein 8A | **0,941** | 0,031431 | 52,011 | 58,486 |
| SIRT3 | sirtuin 3 | **0,941** | 0,143977 | 9,029 | 9,258 |
| BRF1 | BRF1, RNA polymerase III transcription initiation factor subunit | **0,941** | 0,098699 | 19,387 | 18,070 |
| RPP14 | ribonuclease P/MRP subunit p14 | **0,941** | 0,128013 | 13,404 | 13,153 |
| SLC37A1 | solute carrier family 37 member 1 | **0,941** | 0,425914 | 2,382 | 2,554 |
| GNAI2 | G protein subunit alpha i2 | **0,941** | 0,034362 | 258,894 | 219,133 |
| XG | Xg blood group | **0,941** | 0,314207 | 3,323 | 5,555 |
| SKIV2L2 | Ski2 like RNA helicase 2 | **0,941** | 0,035716 | 59,544 | 52,740 |
| COMMD1 | copper metabolism domain containing 1 | **0,941** | 0,144833 | 10,136 | 8,939 |
| IQCC | IQ motif containing C | **0,941** | 0,45325 | 1,828 | 2,235 |
| RTEL1-TNFRSF6B | RTEL1-TNFRSF6B readthrough (NMD candidate) | **0,941** | 0,470782 | 1,606 | 2,043 |
| SYDE2 | synapse defective Rho GTPase homolog 2 | **0,941** | 0,493928 | 2,437 | 2,107 |
| PSENEN | presenilin enhancer gamma-secretase subunit | **0,941** | 0,171683 | 17,060 | 9,386 |
| CLCN2 | chloride voltage-gated channel 2 | **0,941** | 0,414593 | 3,656 | 2,618 |
| PHF8 | PHD finger protein 8 | **0,941** | 0,030448 | 30,742 | 32,947 |
| DDX21 | DExD-box helicase 21 | **0,941** | 0,019699 | 105,297 | 115,951 |
| TGIF2 | TGFB induced factor homeobox 2 | **0,941** | 0,376463 | 3,102 | 2,107 |
| TMEM183A | transmembrane protein 183A | **0,941** | 0,14042 | 11,410 | 10,471 |
| MAPKAPK3 | mitogen-activated protein kinase-activated protein kinase 3 | **0,941** | 0,026713 | 22,045 | 18,070 |
| NACC1 | nucleus accumbens associated 1 | **0,941** | 0,04207 | 45,198 | 39,204 |
| C2orf76 | chromosome 2 open reading frame 76 | **0,941** | 0,329659 | 6,979 | 6,768 |
| XRCC6 | X-ray repair cross complementing 6 | **0,941** | 0,022335 | 169,328 | 152,793 |
| FRS3 | fibroblast growth factor receptor substrate 3 | **0,941** | 0,501879 | 1,883 | 2,746 |
| RNF126 | ring finger protein 126 | **0,941** | 0,20437 | 15,842 | 13,153 |
| UBE3C | ubiquitin protein ligase E3C | **0,941** | 0,022419 | 73,115 | 62,381 |
| REXO2 | RNA exonuclease 2 | **0,941** | 0,022142 | 38,164 | 31,733 |
| PSMA6 | proteasome subunit alpha 6 | **0,941** | 0,310261 | 5,650 | 5,683 |

| | | | | | |
|---|---|---|---|---|---|
| BAALC | brain and acute leukemia, cytoplasmic | **0,941** | 0,459533 | 4,985 | 3,320 |
| ZFPM1 | zinc finger protein, FOG family member 1 | **0,941** | 0,190583 | 5,207 | 6,321 |
| VAMP8 | vesicle associated membrane protein 8 | **0,941** | 0,263692 | 5,151 | 4,278 |
| LURAP1L-AS1 | LURAP1L antisense RNA 1 | **0,941** | 0,76658 | 0,111 | 0,511 |
| DLEU1 | deleted in lymphocytic leukemia 1 | **0,941** | 0,554071 | 1,219 | 1,532 |
| TANGO6 | transport and golgi organization 6 homolog | **0,941** | 0,244206 | 7,644 | 10,088 |
| NME5 | NME/NM23 family member 5 | **0,941** | 0,634215 | 0,997 | 0,575 |
| TMEM43 | transmembrane protein 43 | **0,941** | 0,011726 | 128,394 | 108,800 |
| FUT10 | fucosyltransferase 10 | **0,941** | 0,37143 | 3,323 | 3,959 |
| THADA | THADA, armadillo repeat containing | **0,941** | 0,05959 | 21,159 | 23,114 |
| MRPS26 | mitochondrial ribosomal protein S26 | **0,941** | 0,129081 | 12,186 | 12,515 |
| C1orf123 | chromosome 1 open reading frame 123 | **0,941** | 0,037653 | 23,762 | 23,561 |
| ACP1 | acid phosphatase 1, soluble | **0,941** | 0,022922 | 39,327 | 46,100 |
| BUD31 | BUD31 homolog | **0,941** | 0,05663 | 41,321 | 42,843 |
| MEPCE | methylphosphate capping enzyme | **0,941** | 0,013984 | 69,016 | 61,104 |
| RBM12 | RNA binding motif protein 12 | **0,940** | 0,013837 | 46,749 | 43,226 |
| PPP1R26 | protein phosphatase 1 regulatory subunit 26 | **0,940** | 0,115723 | 14,457 | 10,663 |
| TMEM126A | transmembrane protein 126A | **0,940** | 0,215044 | 10,303 | 10,024 |
| FBRS | fibrosin | **0,940** | 0,031865 | 84,248 | 68,064 |
| BRWD3 | bromodomain and WD repeat domain containing 3 | **0,940** | 0,206777 | 11,300 | 12,898 |
| DDX23 | DEAD-box helicase 23 | **0,940** | 0,020804 | 60,985 | 62,445 |
| MORN2 | MORN repeat containing 2 | **0,940** | 0,211371 | 8,585 | 6,832 |
| GPR107 | G protein-coupled receptor 107 | **0,940** | 0,052965 | 125,016 | 100,819 |
| NCAPG | non-SMC condensin I complex subunit G | **0,940** | 0,510338 | 4,930 | 0,575 |
| POMT2 | protein O-mannosyltransferase 2 | **0,940** | 0,075426 | 23,042 | 16,218 |
| B4GALT3 | beta-1,4-galactosyltransferase 3 | **0,940** | 0,095824 | 23,208 | 24,135 |
| MGAT5B | mannosyl (alpha-1,6-)-glycoprotein beta-1,6-N-acetyl-glucosaminyltransferase, isozyme B | **0,940** | 0,668614 | 0,499 | 0,958 |
| PALD1 | phosphatase domain containing, paladin 1 | **0,940** | 0,354131 | 3,379 | 3,448 |
| NOD1 | nucleotide binding oligomerization domain containing 1 | **0,940** | 0,433904 | 2,216 | 3,001 |
| NUDCD2 | NudC domain containing 2 | **0,940** | 0,178639 | 12,518 | 11,365 |
| MSL3 | male-specific lethal 3 homolog (Drosophila) | **0,940** | 0,122588 | 10,912 | 9,833 |
| PTPA | protein phosphatase 2 phosphatase activator | **0,940** | 0,04979 | 53,728 | 67,553 |
| ACADS | acyl-CoA dehydrogenase, C-2 to C-3 short chain | **0,940** | 0,153258 | 12,463 | 10,791 |

| | | | | | |
|---|---|---|---|---|---|
| PKP4 | plakophilin 4 | **0,940** | 0,131745 | 20,661 | 18,325 |
| STAG1 | stromal antigen 1 | **0,940** | 0,063363 | 54,116 | 49,356 |
| KLC4 | kinesin light chain 4 | **0,940** | 0,109173 | 12,463 | 12,642 |
| NSMAF | neutral sphingomyelinase activation associated factor | **0,940** | 0,097372 | 15,786 | 16,729 |
| ZNF639 | zinc finger protein 639 | **0,940** | 0,059653 | 18,002 | 17,303 |
| ITGB2 | integrin subunit beta 2 | **0,940** | 0,077563 | 21,935 | 20,496 |
| MRPS21 | mitochondrial ribosomal protein S21 | **0,940** | 0,075364 | 36,004 | 30,329 |
| NDUFA9 | NADH:ubiquinone oxidoreductase subunit A9 | **0,940** | 0,185375 | 5,761 | 5,683 |
| SLC35E4 | solute carrier family 35 member E4 | **0,940** | 0,163115 | 14,069 | 16,856 |
| SDF2 | stromal cell derived factor 2 | **0,940** | 0,067666 | 37,998 | 32,436 |
| NELFB | negative elongation factor complex member B | **0,940** | 0,040614 | 49,020 | 45,525 |
| SULF2 | sulfatase 2 | **0,940** | 0,048398 | 128,173 | 108,736 |
| UNK | unkempt family zinc finger | **0,940** | 0,026181 | 22,932 | 21,709 |
| GMDS | GDP-mannose 4,6-dehydratase | **0,940** | 0,093066 | 22,267 | 22,411 |
| NDUFAF3 | NADH:ubiquinone oxidoreductase complex assembly factor 3 | **0,940** | 0,042363 | 37,111 | 33,393 |
| UBQLN4 | ubiquilin 4 | **0,940** | 0,111651 | 15,897 | 11,748 |
| UBIAD1 | UbiA prenyltransferase domain containing 1 | **0,940** | 0,039614 | 21,325 | 24,135 |
| BRPF3 | bromodomain and PHD finger containing 3 | **0,940** | 0,178526 | 36,834 | 28,796 |
| ACADM | acyl-CoA dehydrogenase, C-4 to C-12 straight chain | **0,940** | 0,09318 | 17,946 | 15,579 |
| RPUSD4 | RNA pseudouridylate synthase domain containing 4 | **0,940** | 0,116177 | 10,524 | 10,791 |
| RFNG | RFNG O-fucosylpeptide 3-beta-N-acetylglucosaminyltransferase | **0,940** | 0,05682 | 22,211 | 18,197 |
| SKA3 | spindle and kinetochore associated complex subunit 3 | **0,940** | 0,681974 | 2,326 | 0,447 |
| P2RY11 | purinergic receptor P2Y11 | **0,940** | 0,473699 | 1,163 | 1,915 |
| BAIAP2L1 | BAI1 associated protein 2 like 1 | **0,940** | 0,021839 | 53,895 | 46,866 |
| CAPZB | capping actin protein of muscle Z-line beta subunit | **0,940** | 0,014745 | 88,403 | 78,344 |
| PLXNA3 | plexin A3 | **0,940** | 0,049692 | 41,598 | 20,240 |
| UTP18 | UTP18, small subunit processome component | **0,940** | 0,04659 | 28,803 | 26,562 |
| GGT7 | gamma-glutamyltransferase 7 | **0,940** | 0,05067 | 17,669 | 15,516 |
| ZNF598 | zinc finger protein 598 | **0,940** | 0,086792 | 25,867 | 22,667 |
| TAF1C | TATA-box binding protein associated factor, RNA polymerase I subunit C | **0,940** | 0,03262 | 18,500 | 21,198 |
| SPRY4 | sprouty RTK signaling antagonist 4 | **0,939** | 0,1399 | 58,104 | 54,464 |
| DCUN1D3 | defective in cullin neddylation 1 domain containing 3 | **0,939** | 0,164362 | 10,469 | 9,067 |
| GRPEL2 | GrpE like 2, mitochondrial | **0,939** | 0,172252 | 10,856 | 11,876 |

| | | | | | |
|---|---|---|---|---|---|
| ZNF70 | zinc finger protein 70 | **0,939** | 0,200363 | 11,244 | 11,621 |
| CCDC77 | coiled-coil domain containing 77 | **0,939** | 0,272312 | 5,982 | 3,512 |
| CDRT4 | CMT1A duplicated region transcript 4 | **0,939** | 0,778036 | 0,166 | 0,064 |
| GPS1 | G protein pathway suppressor 1 | **0,939** | 0,055711 | 34,896 | 32,116 |
| CCDC25 | coiled-coil domain containing 25 | **0,939** | 0,027013 | 25,978 | 23,433 |
| CCDC152 | coiled-coil domain containing 152 | **0,939** | 0,649472 | 0,831 | 0,575 |
| PPP2R3C | protein phosphatase 2 regulatory subunit B''gamma | **0,939** | 0,072654 | 18,334 | 19,602 |
| ELP2 | elongator acetyltransferase complex subunit 2 | **0,939** | 0,022153 | 40,213 | 39,332 |
| FEN1 | flap structure-specific endonuclease 1 | **0,939** | 0,212406 | 7,921 | 5,491 |
| TMEM237 | transmembrane protein 237 | **0,939** | 0,134557 | 15,565 | 11,685 |
| RPAP3 | RNA polymerase II associated protein 3 | **0,939** | 0,083766 | 16,119 | 16,473 |
| ASCC2 | activating signal cointegrator 1 complex subunit 2 | **0,939** | 0,071385 | 21,935 | 20,560 |
| OSGIN2 | oxidative stress induced growth inhibitor family member 2 | **0,939** | 0,017056 | 150,661 | 142,704 |
| NHSL1 | NHS like 1 | **0,939** | 0,571932 | 2,049 | 1,469 |
| AKT1S1 | AKT1 substrate 1 | **0,939** | 0,02031 | 106,405 | 82,111 |
| SSR4P1 | signal sequence receptor subunit 4 pseudogene 1 | **0,939** | 0,436278 | 1,440 | 2,618 |
| ZNHIT1 | zinc finger HIT-type containing 1 | **0,939** | 0,129654 | 24,316 | 20,751 |
| LACC1 | laccase domain containing 1 | **0,939** | 0,069741 | 26,643 | 25,285 |
| TAF1D | TATA-box binding protein associated factor, RNA polymerase I subunit D | **0,939** | 0,064152 | 24,759 | 26,115 |
| PKIG | cAMP-dependent protein kinase inhibitor gamma | **0,939** | 0,082839 | 22,378 | 17,048 |
| YIPF5 | Yip1 domain family member 5 | **0,939** | 0,01833 | 105,352 | 89,134 |
| INTS11 | integrator complex subunit 11 | **0,939** | 0,0157 | 40,047 | 43,163 |
| LAYN | layilin | **0,939** | 0,280087 | 4,043 | 4,661 |
| EIF2B1 | eukaryotic translation initiation factor 2B subunit alpha | **0,939** | 0,054548 | 21,270 | 22,092 |
| INTS5 | integrator complex subunit 5 | **0,939** | 0,161273 | 20,107 | 19,985 |
| NPAS2 | neuronal PAS domain protein 2 | **0,939** | 0,103976 | 19,442 | 30,073 |
| MRGBP | MRG domain binding protein | **0,939** | 0,234748 | 3,767 | 3,448 |
| GRHPR | glyoxylate and hydroxypyruvate reductase | **0,939** | 0,102205 | 31,517 | 24,071 |
| CEBPB | CCAAT/enhancer binding protein beta | **0,939** | 0,034075 | 295,562 | 246,588 |
| C1QTNF8 | C1q and TNF related 8 | **0,939** | 0,453203 | 1,939 | 0,702 |
| TXNDC5 | thioredoxin domain containing 5 | **0,939** | 0,308141 | 8,364 | 7,343 |
| SNRPC | small nuclear ribonucleoprotein polypeptide C | **0,939** | 0,06952 | 26,366 | 27,647 |
| HNRNPA0 | heterogeneous nuclear ribonucleoprotein A0 | **0,939** | 0,042062 | 94,440 | 90,156 |

| | | | | | |
|---|---|---|---|---|---|
| STUB1 | STIP1 homology and U-box containing protein 1 | **0,939** | 0,082459 | 40,102 | 30,903 |
| BBS1 | Bardet-Biedl syndrome 1 | **0,939** | 0,707679 | 0,665 | 1,022 |
| SUGT1 | SGT1 homolog, MIS12 kinetochore complex assembly cochaperone | **0,939** | 0,019792 | 41,653 | 40,609 |
| MDH2 | malate dehydrogenase 2 | **0,938** | 0,047045 | 89,067 | 66,404 |
| PRKAG1 | protein kinase AMP-activated non-catalytic subunit gamma 1 | **0,938** | 0,026538 | 40,601 | 35,884 |
| PARK7 | Parkinsonism associated deglycase | **0,938** | 0,025099 | 122,246 | 117,228 |
| INPPL1 | inositol polyphosphate phosphatase like 1 | **0,938** | 0,064636 | 44,035 | 31,478 |
| CCT4 | chaperonin containing TCP1 subunit 4 | **0,938** | 0,035968 | 76,826 | 74,960 |
| ERC1 | ELKS/RAB6-interacting/CAST family member 1 | **0,938** | 0,059861 | 56,276 | 44,440 |
| TIRAP | TIR domain containing adaptor protein | **0,938** | 0,17385 | 6,647 | 6,449 |
| IL16 | interleukin 16 | **0,938** | 0,158282 | 22,765 | 32,500 |
| AKT1 | AKT serine/threonine kinase 1 | **0,938** | 0,015955 | 103,081 | 76,045 |
| AKNA | AT-hook transcription factor | **0,938** | 0,142911 | 18,445 | 20,560 |
| RNF207 | ring finger protein 207 | **0,938** | 0,165887 | 6,148 | 7,854 |
| CNOT11 | CCR4-NOT transcription complex subunit 11 | **0,938** | 0,046922 | 29,357 | 22,411 |
| BCL2L12 | BCL2 like 12 | **0,938** | 0,161308 | 6,979 | 5,874 |
| RNF212 | ring finger protein 212 | **0,938** | 0,373572 | 2,271 | 1,852 |
| MTFR1L | mitochondrial fission regulator 1 like | **0,938** | 0,037453 | 29,855 | 23,433 |
| GTF2A2 | general transcription factor IIA subunit 2 | **0,938** | 0,086425 | 29,855 | 30,776 |
| ATE1-AS1 | ATE1 antisense RNA 1 | **0,938** | 0,286754 | 2,880 | 3,831 |
| SLC25A42 | solute carrier family 25 member 42 | **0,938** | 0,160455 | 9,472 | 8,364 |
| IPO11 | importin 11 | **0,938** | 0,125363 | 13,349 | 14,813 |
| CSGALNACT1 | chondroitin sulfate N-acetylgalactosaminyltransferase 1 | **0,938** | 0,032655 | 199,571 | 145,833 |
| CD1D | CD1d molecule | **0,938** | 0,490239 | 1,385 | 0,511 |
| MNT | MAX network transcriptional repressor | **0,938** | 0,048549 | 25,036 | 17,559 |
| DNASE1L2 | deoxyribonuclease 1 like 2 | **0,938** | 0,596339 | 1,108 | 0,383 |
| LINC00638 | long intergenic non-protein coding RNA 638 | **0,938** | 0,376901 | 2,437 | 3,448 |
| ZNF747 | zinc finger protein 747 | **0,938** | 0,273488 | 5,871 | 4,214 |
| INTS7 | integrator complex subunit 7 | **0,938** | 0,169902 | 7,422 | 8,300 |
| KBTBD8 | kelch repeat and BTB domain containing 8 | **0,938** | 0,224926 | 9,029 | 7,726 |
| TLR4 | toll like receptor 4 | **0,938** | 0,287353 | 5,816 | 5,172 |
| SDHAF2 | succinate dehydrogenase complex assembly factor 2 | **0,938** | 0,201548 | 7,865 | 7,215 |
| RRAGA | Ras related GTP binding A | **0,938** | 0,030997 | 62,979 | 47,696 |

| | | | | | |
|---|---|---|---|---|---|
| MB21D1 | Mab-21 domain containing 1 | **0,938** | 0,299538 | 4,542 | 3,831 |
| SIK3 | SIK family kinase 3 | **0,938** | 0,060852 | 71,509 | 94,242 |
| SH3BP1 | SH3 domain binding protein 1 | **0,938** | 0,751236 | 0,554 | 0,447 |
| FAM126A | family with sequence similarity 126 member A | **0,938** | 0,04213 | 66,413 | 61,615 |
| MORF4L2 | mortality factor 4 like 2 | **0,938** | 0,014111 | 232,362 | 209,938 |
| ADCY7 | adenylate cyclase 7 | **0,938** | 0,29091 | 4,376 | 3,831 |
| WRNIP1 | Werner helicase interacting protein 1 | **0,938** | 0,028559 | 40,324 | 36,778 |
| RBMX2 | RNA binding motif protein, X-linked 2 | **0,938** | 0,158591 | 12,130 | 11,174 |
| BOK | BOK, BCL2 family apoptosis regulator | **0,938** | 0,046388 | 33,622 | 15,899 |
| TOMM40L | translocase of outer mitochondrial membrane 40 like | **0,938** | 0,18384 | 6,924 | 8,620 |
| BTN3A2 | butyrophilin subfamily 3 member A2 | **0,938** | 0,212136 | 6,702 | 6,704 |
| VLDLR | very low density lipoprotein receptor | **0,938** | 0,022616 | 86,132 | 69,596 |
| SCNM1 | sodium channel modifier 1 | **0,938** | 0,06344 | 16,063 | 17,750 |
| CCDC130 | coiled-coil domain containing 130 | **0,938** | 0,051919 | 19,940 | 21,326 |
| BDKRB1 | bradykinin receptor B1 | **0,938** | 0,049693 | 27,086 | 35,117 |
| CCNI | cyclin I | **0,938** | 0,009887 | 175,587 | 158,412 |
| LMNB2 | lamin B2 | **0,938** | 0,0163 | 50,350 | 48,398 |
| PDP2 | pyruvate dehyrogenase phosphatase catalytic subunit 2 | **0,938** | 0,138676 | 9,472 | 9,833 |
| RRP8 | ribosomal RNA processing 8, methyltransferase, homolog (yeast) | **0,938** | 0,144115 | 7,478 | 9,450 |
| ATR | ATR serine/threonine kinase | **0,938** | 0,086729 | 20,107 | 22,347 |
| COQ5 | coenzyme Q5, methyltransferase | **0,938** | 0,029227 | 17,559 | 19,091 |
| CYP2R1 | cytochrome P450 family 2 subfamily R member 1 | **0,938** | 0,251574 | 3,656 | 3,959 |
| POMGNT1 | protein O-linked mannose N-acetylglucosaminyltransferase 1 (beta 1,2-) | **0,938** | 0,015175 | 57,329 | 43,290 |
| PCDHGA10 | protocadherin gamma subfamily A, 10 | **0,938** | 0,41878 | 3,323 | 2,809 |
| GTF2H3 | general transcription factor IIH subunit 3 | **0,938** | 0,028487 | 23,652 | 22,986 |
| MPHOSPH10 | M-phase phosphoprotein 10 | **0,938** | 0,045327 | 22,322 | 25,285 |
| NUP133 | nucleoporin 133 | **0,938** | 0,059472 | 22,156 | 17,623 |
| BUD23 | BUD23, rRNA methyltransferase and ribosome maturation factor | **0,938** | 0,088403 | 55,556 | 58,423 |
| DET1 | de-etiolated homolog 1 (Arabidopsis) | **0,938** | 0,157425 | 10,413 | 9,514 |
| C14orf79 | chromosome 14 open reading frame 79 | **0,937** | 0,035575 | 15,343 | 15,324 |
| SYAP1 | synapse associated protein 1 | **0,937** | 0,026807 | 42,817 | 33,010 |
| NEK8 | NIMA related kinase 8 | **0,937** | 0,202283 | 6,924 | 6,704 |
| HSPB11 | heat shock protein family B (small) member 11 | **0,937** | 0,265505 | 7,312 | 7,343 |

| | | | | | |
|---|---|---|---|---|---|
| FGGY | FGGY carbohydrate kinase domain containing | **0,937** | 0,140587 | 6,536 | 7,981 |
| RPL24 | ribosomal protein L24 | **0,937** | 0,080554 | 192,425 | 171,692 |
| KIAA0100 | KIAA0100 | **0,937** | 0,06198 | 95,714 | 87,410 |
| UBASH3B | ubiquitin associated and SH3 domain containing B | **0,937** | 0,057142 | 28,027 | 23,050 |
| NCAPH2 | non-SMC condensin II complex subunit H2 | **0,937** | 0,059884 | 21,935 | 25,859 |
| HOXD9 | homeobox D9 | **0,937** | 0,210106 | 7,145 | 6,640 |
| PDE8A | phosphodiesterase 8A | **0,937** | 0,041109 | 40,767 | 42,716 |
| CCDC9 | coiled-coil domain containing 9 | **0,937** | 0,073263 | 24,427 | 32,947 |
| TBC1D8B | TBC1 domain family member 8B | **0,937** | 0,158485 | 12,574 | 9,450 |
| TFB2M | transcription factor B2, mitochondrial | **0,937** | 0,186261 | 9,029 | 10,535 |
| SEMA4C | semaphorin 4C | **0,937** | 0,082447 | 27,917 | 20,560 |
| SYMPK | symplekin | **0,937** | 0,04039 | 45,918 | 46,291 |
| NRSN2 | neurensin 2 | **0,937** | 0,104421 | 12,851 | 6,002 |
| FAM35A | family with sequence similarity 35 member A | **0,937** | 0,128475 | 10,247 | 11,174 |
| BRAT1 | BRCA1 associated ATM activator 1 | **0,937** | 0,061908 | 17,891 | 22,028 |
| ERGIC3 | ERGIC and golgi 3 | **0,937** | 0,03395 | 150,329 | 86,580 |
| VAMP5 | vesicle associated membrane protein 5 | **0,937** | 0,203838 | 7,422 | 7,023 |
| KXD1 | KxDL motif containing 1 | **0,937** | 0,01409 | 53,008 | 49,739 |
| ABHD15 | abhydrolase domain containing 15 | **0,937** | 0,222424 | 12,297 | 9,131 |
| HOXC9 | homeobox C9 | **0,937** | 0,259805 | 6,259 | 6,513 |
| QSER1 | glutamine and serine rich 1 | **0,937** | 0,129557 | 30,908 | 26,625 |
| TLE1P1 | transducin like enhancer of split 1 pseudogene 1 | **0,937** | 0,717388 | 0,554 | 0,383 |
| FAT3 | FAT atypical cadherin 3 | **0,937** | 0,439305 | 8,142 | 8,556 |
| HACE1 | HECT domain and ankyrin repeat containing E3 ubiquitin protein ligase 1 | **0,937** | 0,217371 | 9,527 | 6,577 |
| WASF1 | WAS protein family member 1 | **0,937** | 0,390608 | 3,157 | 2,107 |
| PTRHD1 | peptidyl-tRNA hydrolase domain containing 1 | **0,937** | 0,114862 | 7,865 | 5,874 |
| DCAF16 | DDB1 and CUL4 associated factor 16 | **0,937** | 0,112415 | 11,853 | 12,515 |
| ARMC8 | armadillo repeat containing 8 | **0,937** | 0,028182 | 24,206 | 22,986 |
| COPZ2 | coatomer protein complex subunit zeta 2 | **0,937** | 0,114236 | 16,894 | 8,556 |
| INTS13 | integrator complex subunit 13 | **0,936** | 0,074862 | 17,226 | 13,345 |
| LRRIQ3 | leucine rich repeats and IQ motif containing 3 | **0,936** | 0,686757 | 0,443 | 0,638 |
| TIMM17A | translocase of inner mitochondrial membrane 17A | **0,936** | 0,030348 | 59,932 | 53,315 |
| NAV1 | neuron navigator 1 | **0,936** | 0,198216 | 76,106 | 60,083 |

| | | | | | |
|---|---|---|---|---|---|
| STX18 | syntaxin 18 | **0,936** | 0,040597 | 19,608 | 18,900 |
| DCTN6 | dynactin subunit 6 | **0,936** | 0,11526 | 17,559 | 16,026 |
| TMEM120B | transmembrane protein 120B | **0,936** | 0,154589 | 9,361 | 10,918 |
| DDX27 | DEAD-box helicase 27 | **0,936** | 0,016078 | 36,668 | 39,842 |
| ARHGEF9 | Cdc42 guanine nucleotide exchange factor 9 | **0,936** | 0,300458 | 5,594 | 5,619 |
| POLL | DNA polymerase lambda | **0,936** | 0,082036 | 14,235 | 12,642 |
| XPOT | exportin for tRNA | **0,936** | 0,030428 | 159,634 | 147,238 |
| BOLA1 | bolA family member 1 | **0,936** | 0,136711 | 11,023 | 14,558 |
| PDIA6 | protein disulfide isomerase family A member 6 | **0,936** | 0,015378 | 475,469 | 285,601 |
| TPM1 | tropomyosin 1 (alpha) | **0,936** | 0,032651 | 105,518 | 93,732 |
| MST1P2 | macrophage stimulating 1 pseudogene 2 | **0,936** | 0,684958 | 0,886 | 0,511 |
| RPS2 | ribosomal protein S2 | **0,936** | 0,084012 | 370,449 | 381,567 |
| NDUFS3 | NADH:ubiquinone oxidoreductase core subunit S3 | **0,936** | 0,11503 | 6,591 | 8,173 |
| IFT172 | intraflagellar transport 172 | **0,936** | 0,092882 | 11,355 | 11,621 |
| GFM2 | G elongation factor mitochondrial 2 | **0,936** | 0,076785 | 16,728 | 18,006 |
| TRAF3 | TNF receptor associated factor 3 | **0,936** | 0,029598 | 69,958 | 55,230 |
| BECN1 | beclin 1 | **0,936** | 0,009768 | 48,633 | 41,502 |
| RAD50 | RAD50 double strand break repair protein | **0,936** | 0,562198 | 3,656 | 2,362 |
| AGA | aspartylglucosaminidase | **0,936** | 0,116902 | 10,303 | 6,704 |
| NUAK1 | NUAK family kinase 1 | **0,936** | 0,349742 | 10,690 | 7,790 |
| COQ8B | coenzyme Q8B | **0,936** | 0,084464 | 15,288 | 13,536 |
| TUT1 | terminal uridylyl transferase 1, U6 snRNA-specific | **0,936** | 0,16721 | 9,139 | 10,280 |
| TMEM184B | transmembrane protein 184B | **0,936** | 0,009235 | 138,586 | 132,872 |
| GTPBP8 | GTP binding protein 8 (putative) | **0,936** | 0,16816 | 7,588 | 8,492 |
| LINC01301 | long intergenic non-protein coding RNA 1301 | **0,936** | 0,680785 | 0,277 | 0,319 |
| GNAI1 | G protein subunit alpha i1 | **0,936** | 0,160323 | 8,253 | 4,789 |
| RP9 | retinitis pigmentosa 9 (autosomal dominant) | **0,936** | 0,240661 | 6,758 | 7,023 |
| VPS72 | vacuolar protein sorting 72 homolog | **0,936** | 0,071577 | 22,876 | 18,325 |
| TXNL1 | thioredoxin like 1 | **0,936** | 0,019829 | 57,661 | 48,526 |
| ELK3 | ELK3, ETS transcription factor | **0,936** | 0,0178 | 100,699 | 104,714 |
| AKR1A1 | aldo-keto reductase family 1 member A1 | **0,936** | 0,083948 | 49,796 | 48,909 |
| SPARC | secreted protein acidic and cysteine rich | **0,936** | 0,022279 | 843,371 | 276,406 |
| CCDC57 | coiled-coil domain containing 57 | **0,936** | 0,232123 | 12,130 | 15,388 |

| | | | | | |
|---|---|---|---|---|---|
| SAR1B | secretion associated Ras related GTPase 1B | **0,936** | 0,017559 | 54,559 | 58,678 |
| DDOST | dolichyl-diphosphooligosaccharide--protein glycosyltransferase non-catalytic subunit | **0,936** | 0,009532 | 276,231 | 168,691 |
| RPS10 | ribosomal protein S10 | **0,936** | 0,211702 | 6,314 | 6,130 |
| NUP62CL | nucleoporin 62 C-terminal like | **0,936** | 0,725593 | 0,332 | 0,255 |
| NPRL2 | NPR2-like, GATOR1 complex subunit | **0,935** | 0,150853 | 7,312 | 7,917 |
| THEM6 | thioesterase superfamily member 6 | **0,935** | 0,152786 | 11,687 | 13,408 |
| SLC16A3 | solute carrier family 16 member 3 | **0,935** | 0,011102 | 183,618 | 116,079 |
| TADA3 | transcriptional adaptor 3 | **0,935** | 0,02845 | 58,658 | 52,421 |
| CTU2 | cytosolic thiouridylase subunit 2 | **0,935** | 0,172935 | 4,154 | 6,130 |
| FUT8 | fucosyltransferase 8 | **0,935** | 0,107812 | 10,967 | 8,875 |
| SEC16A | SEC16 homolog A, endoplasmic reticulum export factor | **0,935** | 0,052836 | 112,608 | 118,505 |
| AKAP3 | A-kinase anchoring protein 3 | **0,935** | 0,320449 | 4,043 | 3,895 |
| CLPB | ClpB homolog, mitochondrial AAA ATPase chaperonin | **0,935** | 0,042531 | 19,442 | 18,516 |
| PPP1R9B | protein phosphatase 1 regulatory subunit 9B | **0,935** | 0,050959 | 25,701 | 22,284 |
| RBBP9 | RB binding protein 9, serine hydrolase | **0,935** | 0,054355 | 18,057 | 19,602 |
| MRM2 | mitochondrial rRNA methyltransferase 2 | **0,935** | 0,083491 | 17,503 | 16,665 |
| COL16A1 | collagen type XVI alpha 1 chain | **0,935** | 0,049648 | 100,976 | 104,842 |
| DONSON | downstream neighbor of SON | **0,935** | 0,266595 | 5,761 | 5,746 |
| BCKDHB | branched chain keto acid dehydrogenase E1 subunit beta | **0,935** | 0,068389 | 10,413 | 10,918 |
| INTS12 | integrator complex subunit 12 | **0,935** | 0,04905 | 11,853 | 12,770 |
| SORD | sorbitol dehydrogenase | **0,935** | 0,613046 | 0,775 | 0,638 |
| CRTC1 | CREB regulated transcription coactivator 1 | **0,935** | 0,08235 | 10,635 | 10,408 |
| PRDX3 | peroxiredoxin 3 | **0,935** | 0,018251 | 67,576 | 54,528 |
| ZNF561 | zinc finger protein 561 | **0,935** | 0,042391 | 16,617 | 16,984 |
| TTC3P1 | tetratricopeptide repeat domain 3 pseudogene 1 | **0,935** | 0,370203 | 4,487 | 4,853 |
| STOX1 | storkhead box 1 | **0,935** | 0,433375 | 1,828 | 1,788 |
| TCEAL6 | transcription elongation factor A like 6 | **0,935** | 0,745029 | 0,222 | 0,192 |
| NDUFA8 | NADH:ubiquinone oxidoreductase subunit A8 | **0,935** | 0,059288 | 29,578 | 26,625 |
| ACTR5 | ARP5 actin-related protein 5 homolog | **0,935** | 0,317041 | 5,041 | 6,385 |
| TXNDC12 | thioredoxin domain containing 12 | **0,935** | 0,021976 | 81,257 | 72,533 |
| DAPK3 | death associated protein kinase 3 | **0,935** | 0,042049 | 28,360 | 20,560 |
| RAVER1 | ribonucleoprotein, PTB binding 1 | **0,935** | 0,06152 | 21,325 | 21,390 |
| THAP11 | THAP domain containing 11 | **0,935** | 0,045314 | 18,445 | 16,920 |

| | | | | | |
|---|---|---|---|---|---|
| KCNB1 | potassium voltage-gated channel subfamily B member 1 | **0,935** | 0,732625 | 0,277 | 0,319 |
| RILPL2 | Rab interacting lysosomal protein like 2 | **0,935** | 0,027203 | 48,965 | 37,480 |
| DLX6 | distal-less homeobox 6 | **0,935** | 0,3707 | 2,714 | 2,171 |
| RPL6 | ribosomal protein L6 | **0,935** | 0,038881 | 154,926 | 141,364 |
| FGFR1OP | FGFR1 oncogene partner | **0,935** | 0,194024 | 8,973 | 8,173 |
| ACSL5 | acyl-CoA synthetase long-chain family member 5 | **0,935** | 0,389406 | 5,317 | 2,682 |
| CALD1 | caldesmon 1 | **0,935** | 0,028096 | 76,826 | 85,750 |
| GLRX5 | glutaredoxin 5 | **0,935** | 0,12127 | 16,174 | 12,962 |
| CRCP | CGRP receptor component | **0,935** | 0,039843 | 21,491 | 20,815 |
| XPA | XPA, DNA damage recognition and repair factor | **0,935** | 0,225549 | 3,877 | 3,767 |
| PTPN6 | protein tyrosine phosphatase, non-receptor type 6 | **0,935** | 0,526321 | 1,772 | 1,660 |
| VNN2 | vanin 2 | **0,935** | 0,121504 | 10,413 | 15,132 |
| SETD1A | SET domain containing 1A | **0,935** | 0,016872 | 26,200 | 25,604 |
| RAN | RAN, member RAS oncogene family | **0,935** | 0,023652 | 171,876 | 146,599 |
| TPGS2 | tubulin polyglutamylase complex subunit 2 | **0,935** | 0,044898 | 24,206 | 15,005 |
| EIF2S2 | eukaryotic translation initiation factor 2 subunit beta | **0,935** | 0,028043 | 103,247 | 93,604 |
| ORMDL3 | ORMDL sphingolipid biosynthesis regulator 3 | **0,935** | 0,017986 | 64,363 | 61,934 |
| CCND2 | cyclin D2 | **0,934** | 0,051507 | 51,125 | 45,142 |
| EXOC3 | exocyst complex component 3 | **0,934** | 0,022908 | 47,968 | 36,331 |
| GDI1 | GDP dissociation inhibitor 1 | **0,934** | 0,011513 | 126,677 | 105,927 |
| PSAT1 | phosphoserine aminotransferase 1 | **0,934** | 0,009616 | 125,182 | 119,782 |
| OXT | oxytocin/neurophysin I prepropeptide | **0,934** | 0,62755 | 0,942 | 0,575 |
| PCGF1 | polycomb group ring finger 1 | **0,934** | 0,045632 | 12,130 | 12,068 |
| TBL1XR1 | transducin beta like 1 X-linked receptor 1 | **0,934** | 0,028232 | 149,830 | 98,584 |
| SPR | sepiapterin reductase (7,8-dihydrobiopterin:NADP+ oxidoreductase) | **0,934** | 0,040556 | 21,768 | 20,240 |
| HNRNPM | heterogeneous nuclear ribonucleoprotein M | **0,934** | 0,028907 | 162,238 | 167,478 |
| AKT3 | AKT serine/threonine kinase 3 | **0,934** | 0,515503 | 0,997 | 1,532 |
| MED4 | mediator complex subunit 4 | **0,934** | 0,025446 | 28,969 | 22,603 |
| ZMYND10 | zinc finger MYND-type containing 10 | **0,934** | 0,630309 | 0,166 | 0,447 |
| CLNS1A | chloride nucleotide-sensitive channel 1A | **0,934** | 0,036411 | 32,736 | 29,818 |
| PPP2R1A | protein phosphatase 2 scaffold subunit Aalpha | **0,934** | 0,01594 | 103,192 | 95,647 |
| PENK | proenkephalin | **0,934** | 0,024359 | 34,453 | 26,115 |
| LINC00680 | long intergenic non-protein coding RNA 680 | **0,934** | 0,303055 | 5,594 | 2,873 |

| | | | | | |
|---|---|---|---|---|---|
| EXOSC7 | exosome component 7 | **0,934** | 0,098406 | 13,792 | 13,664 |
| DIS3 | DIS3 homolog, exosome endoribonuclease and 3'-5' exoribonuclease | **0,934** | 0,021673 | 51,679 | 54,975 |
| PEX14 | peroxisomal biogenesis factor 14 | **0,934** | 0,046795 | 14,346 | 15,643 |
| POLR2I | RNA polymerase II subunit I | **0,934** | 0,143495 | 10,912 | 11,493 |
| WDR45 | WD repeat domain 45 | **0,934** | 0,010797 | 45,475 | 37,735 |
| RTCA | RNA 3'-terminal phosphate cyclase | **0,934** | 0,022646 | 21,325 | 17,367 |
| KLHDC2 | kelch domain containing 2 | **0,934** | 0,024248 | 27,363 | 21,964 |
| ARHGAP17 | Rho GTPase activating protein 17 | **0,934** | 0,034223 | 30,797 | 29,052 |
| XRCC2 | X-ray repair cross complementing 2 | **0,934** | 0,650432 | 1,440 | 0,511 |
| ARCN1 | archain 1 | **0,934** | 0,045565 | 223,555 | 192,890 |
| RRP7A | ribosomal RNA processing 7 homolog A | **0,934** | 0,038266 | 29,412 | 35,054 |
| BICRA | BRD4 interacting chromatin remodelling complex associated protein | **0,934** | 0,163051 | 12,020 | 12,770 |
| ZNHIT2 | zinc finger HIT-type containing 2 | **0,934** | 0,252606 | 7,810 | 8,364 |
| LINC00271 | long intergenic non-protein coding RNA 271 | **0,934** | 0,672656 | 0,332 | 0,447 |
| PLTP | phospholipid transfer protein | **0,934** | 0,3977 | 3,213 | 2,490 |
| MHENCR | melanoma highly expressed competing endogenous lncRNA for miR-425 and miR-489 | **0,934** | 0,534837 | 0,886 | 0,830 |
| ZFYVE9 | zinc finger FYVE-type containing 9 | **0,934** | 0,109038 | 30,631 | 31,797 |
| IFT46 | intraflagellar transport 46 | **0,934** | 0,02718 | 22,211 | 19,793 |
| ZNF32 | zinc finger protein 32 | **0,934** | 0,162998 | 10,469 | 7,917 |
| JMJD6 | arginine demethylase and lysine hydroxylase | **0,934** | 0,02143 | 24,538 | 20,240 |
| SMPD2 | sphingomyelin phosphodiesterase 2 | **0,934** | 0,102806 | 10,413 | 7,215 |
| SRP14-AS1 | SRP14 antisense RNA1 (head to head) | **0,934** | 0,407069 | 1,662 | 2,873 |
| SUOX | sulfite oxidase | **0,934** | 0,125335 | 9,804 | 7,407 |
| TNFRSF8 | TNF receptor superfamily member 8 | **0,934** | 0,726024 | 0,332 | 0,511 |
| DPY30 | dpy-30, histone methyltransferase complex regulatory subunit | **0,934** | 0,059245 | 17,725 | 13,345 |
| PSMA3-AS1 | PSMA3 antisense RNA 1 | **0,934** | 0,055688 | 18,943 | 23,050 |
| ZBTB38 | zinc finger and BTB domain containing 38 | **0,933** | 0,102222 | 66,524 | 55,294 |
| NUDT17 | nudix hydrolase 17 | **0,933** | 0,614565 | 0,609 | 0,319 |
| CYCS | cytochrome c, somatic | **0,933** | 0,036891 | 66,745 | 73,619 |
| ADNP | activity dependent neuroprotector homeobox | **0,933** | 0,044816 | 56,996 | 47,887 |
| ABCA8 | ATP binding cassette subfamily A member 8 | **0,933** | 0,595382 | 0,942 | 2,043 |
| UBA1 | ubiquitin like modifier activating enzyme 1 | **0,933** | 0,017998 | 285,758 | 242,757 |
| DNAJC2 | DnaJ heat shock protein family (Hsp40) member C2 | **0,933** | 0,093934 | 19,442 | 16,729 |

| | | | | | |
|---|---|---|---|---|---|
| TRMT5 | tRNA methyltransferase 5 | **0,933** | 0,024159 | 21,104 | 22,475 |
| POLR1A | RNA polymerase I subunit A | **0,933** | 0,034884 | 37,444 | 42,843 |
| H1FX | H1 histone family member X | **0,933** | 0,035103 | 162,902 | 117,803 |
| BBS9 | Bardet-Biedl syndrome 9 | **0,933** | 0,121784 | 8,142 | 6,385 |
| BORCS8 | BLOC-1 related complex subunit 8 | **0,933** | 0,121264 | 7,256 | 7,279 |
| RRP1 | ribosomal RNA processing 1 | **0,933** | 0,09172 | 19,553 | 20,943 |
| NIPA2 | non imprinted in Prader-Willi/Angelman syndrome 2 | **0,933** | 0,021742 | 53,895 | 45,333 |
| RFC4 | replication factor C subunit 4 | **0,933** | 0,204832 | 5,484 | 6,640 |
| DERL2 | derlin 2 | **0,933** | 0,040414 | 44,423 | 37,799 |
| NDUFB6 | NADH:ubiquinone oxidoreductase subunit B6 | **0,933** | 0,064262 | 21,048 | 22,731 |
| ZSWIM7 | zinc finger SWIM-type containing 7 | **0,933** | 0,162345 | 8,862 | 8,620 |
| PSMG2 | proteasome assembly chaperone 2 | **0,933** | 0,049727 | 26,587 | 26,051 |
| TAZ | tafazzin | **0,933** | 0,080442 | 9,195 | 7,470 |
| FBXO42 | F-box protein 42 | **0,933** | 0,022885 | 21,990 | 21,517 |
| SDF2L1 | stromal cell derived factor 2 like 1 | **0,933** | 0,202299 | 15,897 | 13,408 |
| RFX2 | regulatory factor X2 | **0,933** | 0,195939 | 3,600 | 5,363 |
| HDAC2 | histone deacetylase 2 | **0,933** | 0,008463 | 51,236 | 43,801 |
| PCDHB16 | protocadherin beta 16 | **0,933** | 0,182526 | 8,530 | 2,171 |
| CNIH1 | cornichon family AMPA receptor auxiliary protein 1 | **0,933** | 0,010164 | 121,914 | 118,633 |
| OAZ2 | ornithine decarboxylase antizyme 2 | **0,933** | 0,025192 | 43,703 | 30,456 |
| ADAMTS7 | ADAM metallopeptidase with thrombospondin type 1 motif 7 | **0,933** | 0,762427 | 0,111 | 0,383 |
| PCDHB2 | protocadherin beta 2 | **0,933** | 0,456987 | 2,936 | 1,788 |
| ADO | 2-aminoethanethiol dioxygenase | **0,933** | 0,038068 | 24,926 | 21,070 |
| SF3A3 | splicing factor 3a subunit 3 | **0,933** | 0,007173 | 53,175 | 52,229 |
| L3MBTL2 | L3MBTL2 polycomb repressive complex 1 subunit | **0,933** | 0,061448 | 22,045 | 24,263 |
| RPL22L1 | ribosomal protein L22 like 1 | **0,933** | 0,210417 | 11,410 | 7,981 |
| KMT2B | lysine methyltransferase 2B | **0,933** | 0,054019 | 28,194 | 29,435 |
| RER1 | retention in endoplasmic reticulum sorting receptor 1 | **0,933** | 0,007704 | 109,506 | 99,606 |
| WASHC3 | WASH complex subunit 3 | **0,933** | 0,05881 | 17,337 | 13,089 |
| NAA20 | N(alpha)-acetyltransferase 20, NatB catalytic subunit | **0,933** | 0,018605 | 38,552 | 31,733 |
| QPCTL | glutaminyl-peptide cyclotransferase like | **0,933** | 0,137955 | 17,669 | 12,578 |
| RNASET2 | ribonuclease T2 | **0,933** | 0,274408 | 4,985 | 3,192 |
| DGKD | diacylglycerol kinase delta | **0,933** | 0,126906 | 11,632 | 9,067 |

| | | | | | |
|---|---|---|---|---|---|
| CENPI | centromere protein I | **0,933** | 0,696016 | 2,049 | 0,255 |
| DERL1 | derlin 1 | **0,933** | 0,010994 | 78,377 | 64,105 |
| MPDZ | multiple PDZ domain crumbs cell polarity complex component | **0,933** | 0,15835 | 11,300 | 15,260 |
| DUSP23 | dual specificity phosphatase 23 | **0,933** | 0,260193 | 3,822 | 3,256 |
| LMBRD1 | LMBR1 domain containing 1 | **0,933** | 0,044327 | 58,104 | 43,290 |
| MAPKAP1 | mitogen-activated protein kinase associated protein 1 | **0,933** | 0,015712 | 44,423 | 36,905 |
| SAMD1 | sterile alpha motif domain containing 1 | **0,933** | 0,148377 | 10,303 | 4,342 |
| LYPD3 | LY6/PLAUR domain containing 3 | **0,933** | 0,5041 | 4,708 | 1,852 |
| RRP15 | ribosomal RNA processing 15 homolog | **0,933** | 0,064251 | 16,617 | 15,516 |
| DTL | denticleless E3 ubiquitin protein ligase homolog | **0,933** | 0,538955 | 3,379 | 1,022 |
| SLC46A3 | solute carrier family 46 member 3 | **0,933** | 0,235248 | 4,320 | 4,725 |
| LAS1L | LAS1 like, ribosome biogenesis factor | **0,933** | 0,020649 | 19,276 | 21,645 |
| YARS2 | tyrosyl-tRNA synthetase 2 | **0,933** | 0,134406 | 8,918 | 9,514 |
| CYB5B | cytochrome b5 type B | **0,933** | 0,017297 | 50,571 | 48,845 |
| ERVK13-1 | endogenous retrovirus group K13 member 1 | **0,932** | 0,180119 | 7,367 | 6,130 |
| SLC10A7 | solute carrier family 10 member 7 | **0,932** | 0,203673 | 10,801 | 9,450 |
| PSMA2 | proteasome subunit alpha 2 | **0,932** | 0,343938 | 4,210 | 4,023 |
| NNMT | nicotinamide N-methyltransferase | **0,932** | 0,050358 | 159,579 | 118,633 |
| CTHRC1 | collagen triple helix repeat containing 1 | **0,932** | 0,194453 | 9,250 | 6,002 |
| TP73-AS1 | TP73 antisense RNA 1 | **0,932** | 0,064127 | 17,448 | 17,431 |
| NFS1 | NFS1, cysteine desulfurase | **0,932** | 0,09406 | 9,250 | 7,790 |
| ADCY6 | adenylate cyclase 6 | **0,932** | 0,055161 | 25,923 | 18,006 |
| BNIP3P1 | BCL2 interacting protein 3 pseudogene 1 | **0,932** | 0,694939 | 0,332 | 0,319 |
| GTF2F2 | general transcription factor IIF subunit 2 | **0,932** | 0,068154 | 17,226 | 21,837 |
| SIPA1 | signal-induced proliferation-associated 1 | **0,932** | 0,072369 | 14,678 | 16,346 |
| IMPA2 | inositol monophosphatase 2 | **0,932** | 0,462701 | 1,828 | 2,171 |
| AIMP2 | aminoacyl tRNA synthetase complex interacting multifunctional protein 2 | **0,932** | 0,090362 | 22,211 | 21,581 |
| ZNF850 | zinc finger protein 850 | **0,932** | 0,453622 | 1,440 | 1,915 |
| JMJD7-PLA2G4B | JMJD7-PLA2G4B readthrough | **0,932** | 0,353491 | 1,772 | 2,682 |
| COX6A1 | cytochrome c oxidase subunit 6A1 | **0,932** | 0,08319 | 20,716 | 18,580 |
| TMSB10 | thymosin beta 10 | **0,932** | 0,074114 | 104,632 | 55,741 |
| WDR24 | WD repeat domain 24 | **0,932** | 0,070011 | 15,952 | 14,622 |
| FAM120A | family with sequence similarity 120A | **0,932** | 0,016615 | 81,590 | 61,871 |

| | | | | | |
|---|---|---|---|---|---|
| SUPV3L1 | Suv3 like RNA helicase | **0,932** | 0,122095 | 12,629 | 12,259 |
| SH3RF3 | SH3 domain containing ring finger 3 | **0,932** | 0,095414 | 15,288 | 20,177 |
| SFRP2 | secreted frizzled related protein 2 | **0,932** | 0,562418 | 3,711 | 0,958 |
| DYRK2 | dual specificity tyrosine phosphorylation regulated kinase 2 | **0,932** | 0,038897 | 21,436 | 13,600 |
| KRTCAP2 | keratinocyte associated protein 2 | **0,932** | 0,469515 | 1,385 | 1,213 |
| ANAPC1 | anaphase promoting complex subunit 1 | **0,932** | 0,288728 | 6,979 | 6,704 |
| PHKA1 | phosphorylase kinase regulatory subunit alpha 1 | **0,932** | 0,329915 | 3,877 | 3,384 |
| YARS | tyrosyl-tRNA synthetase | **0,932** | 0,006962 | 122,246 | 123,294 |
| RPL27A | ribosomal protein L27a | **0,932** | 0,036067 | 141,743 | 132,233 |
| ADAMTS10 | ADAM metallopeptidase with thrombospondin type 1 motif 10 | **0,932** | 0,107584 | 11,521 | 8,492 |
| ZNF286A | zinc finger protein 286A | **0,932** | 0,308992 | 3,102 | 2,937 |
| UGDH | UDP-glucose 6-dehydrogenase | **0,932** | 0,018783 | 233,248 | 172,139 |
| ATG7 | autophagy related 7 | **0,932** | 0,020388 | 46,085 | 45,270 |
| DFNB59 | deafness, autosomal recessive 59 | **0,932** | 0,673108 | 1,385 | 1,022 |
| RAD51C | RAD51 paralog C | **0,932** | 0,138072 | 7,145 | 4,853 |
| GOLGA5 | golgin A5 | **0,932** | 0,020568 | 49,353 | 37,927 |
| BEGAIN | brain enriched guanylate kinase associated | **0,932** | 0,396868 | 1,828 | 2,299 |
| MFSD10 | major facilitator superfamily domain containing 10 | **0,932** | 0,019544 | 39,659 | 26,753 |
| ZNF544 | zinc finger protein 544 | **0,932** | 0,618764 | 1,329 | 0,766 |
| NDUFB4 | NADH:ubiquinone oxidoreductase subunit B4 | **0,932** | 0,023667 | 48,965 | 49,164 |
| SLC5A6 | solute carrier family 5 member 6 | **0,932** | 0,043813 | 15,066 | 16,218 |
| ADRB2 | adrenoceptor beta 2 | **0,932** | 0,071275 | 11,355 | 15,324 |
| TMEM68 | transmembrane protein 68 | **0,932** | 0,100843 | 12,961 | 10,599 |
| CHAC2 | ChaC cation transport regulator homolog 2 | **0,932** | 0,232981 | 3,822 | 3,703 |
| RUVBL2 | RuvB like AAA ATPase 2 | **0,932** | 0,038867 | 37,111 | 35,564 |
| TMEM161A | transmembrane protein 161A | **0,932** | 0,124483 | 9,804 | 8,875 |
| FADD | Fas associated via death domain | **0,932** | 0,042889 | 42,650 | 37,991 |
| CDC25A | cell division cycle 25A | **0,932** | 0,398517 | 3,545 | 1,788 |
| PALM2 | paralemmin 2 | **0,932** | 0,462167 | 2,493 | 2,554 |
| CFAP45 | cilia and flagella associated protein 45 | **0,932** | 0,617784 | 0,166 | 0,638 |
| PRTFDC1 | phosphoribosyl transferase domain containing 1 | **0,932** | 0,520312 | 1,717 | 0,702 |
| ARHGDIA | Rho GDP dissociation inhibitor alpha | **0,932** | 0,006978 | 241,113 | 159,433 |
| TTC19 | tetratricopeptide repeat domain 19 | **0,932** | 0,052746 | 19,276 | 20,815 |

| | | | | | |
|---|---|---|---|---|---|
| GSPT1 | G1 to S phase transition 1 | **0,932** | 0,005508 | 109,119 | 105,225 |
| DNAJB4 | DnaJ heat shock protein family (Hsp40) member B4 | **0,932** | 0,023759 | 26,366 | 21,517 |
| ADCY2 | adenylate cyclase 2 | **0,931** | 0,054186 | 13,404 | 13,792 |
| NVL | nuclear VCP-like | **0,931** | 0,050393 | 11,521 | 10,663 |
| TMEM182 | transmembrane protein 182 | **0,931** | 0,288454 | 2,991 | 4,023 |
| MADD | MAP kinase activating death domain | **0,931** | 0,046726 | 27,806 | 25,923 |
| SH3TC1 | SH3 domain and tetratricopeptide repeats 1 | **0,931** | 0,043534 | 12,795 | 13,153 |
| TOMM7 | translocase of outer mitochondrial membrane 7 | **0,931** | 0,070387 | 74,555 | 53,953 |
| PMS2P3 | PMS1 homolog 2, mismatch repair system component pseudogene 3 | **0,931** | 0,477651 | 1,108 | 1,788 |
| TMEM203 | transmembrane protein 203 | **0,931** | 0,029691 | 24,372 | 19,474 |
| RPUSD2 | RNA pseudouridylate synthase domain containing 2 | **0,931** | 0,160061 | 6,038 | 6,449 |
| C8orf59 | chromosome 8 open reading frame 59 | **0,931** | 0,09638 | 12,684 | 12,642 |
| YWHAB | tyrosine 3-monooxygenase/tryptophan 5-monooxygenase activation protein beta | **0,931** | 0,012755 | 131,773 | 113,206 |
| SLC13A4 | solute carrier family 13 member 4 | **0,931** | 0,611138 | 0,554 | 1,405 |
| INTS4 | integrator complex subunit 4 | **0,931** | 0,07632 | 12,629 | 10,088 |
| PRMT7 | protein arginine methyltransferase 7 | **0,931** | 0,121988 | 6,979 | 8,109 |
| LSM1 | LSM1 homolog, mRNA degradation associated | **0,931** | 0,045542 | 21,935 | 22,284 |
| PPP6R1 | protein phosphatase 6 regulatory subunit 1 | **0,931** | 0,007245 | 44,146 | 41,183 |
| UBFD1 | ubiquitin family domain containing 1 | **0,931** | 0,00476 | 62,314 | 47,632 |
| GSTZ1 | glutathione S-transferase zeta 1 | **0,931** | 0,064145 | 13,017 | 12,770 |
| NAA35 | N(alpha)-acetyltransferase 35, NatC auxiliary subunit | **0,931** | 0,030969 | 21,270 | 22,411 |
| NRG2 | neuregulin 2 | **0,931** | 0,631725 | 0,499 | 0,383 |
| HOXD3 | homeobox D3 | **0,931** | 0,707263 | 0,609 | 0,575 |
| SDF4 | stromal cell derived factor 4 | **0,931** | 0,016589 | 333,061 | 234,393 |
| MED19 | mediator complex subunit 19 | **0,931** | 0,107265 | 14,678 | 12,642 |
| SEH1L | SEH1 like nucleoporin | **0,931** | 0,013057 | 29,578 | 28,349 |
| CENPT | centromere protein T | **0,931** | 0,119141 | 11,798 | 8,684 |
| NGF | nerve growth factor | **0,931** | 0,134297 | 88,846 | 39,332 |
| EXT1 | exostosin glycosyltransferase 1 | **0,931** | 0,040213 | 36,447 | 30,648 |
| PPIE | peptidylprolyl isomerase E | **0,931** | 0,010113 | 25,646 | 25,157 |
| TSC22D1-AS1 | TSC22D1 antisense RNA 1 | **0,931** | 0,648276 | 0,609 | 0,447 |
| CYB5A | cytochrome b5 type A | **0,931** | 0,192028 | 3,767 | 6,513 |
| RASD2 | RASD family member 2 | **0,931** | 0,642331 | 0,554 | 0,575 |

| | | | | | |
|---|---|---|---|---|---|
| USP3 | ubiquitin specific peptidase 3 | **0,931** | 0,129047 | 10,690 | 12,195 |
| DAP3 | death associated protein 3 | **0,931** | 0,013479 | 57,107 | 55,294 |
| ATG16L2 | autophagy related 16 like 2 | **0,930** | 0,171503 | 10,358 | 9,067 |
| BTF3 | basic transcription factor 3 | **0,930** | 0,014788 | 136,648 | 123,486 |
| MGAT2 | mannosyl (alpha-1,6-)-glycoprotein beta-1,2-N-acetylglucosaminyltransferase | **0,930** | 0,02555 | 74,998 | 53,570 |
| MALSU1 | mitochondrial assembly of ribosomal large subunit 1 | **0,930** | 0,081157 | 10,524 | 9,897 |
| UROS | uroporphyrinogen III synthase | **0,930** | 0,078897 | 10,081 | 9,322 |
| ARHGEF39 | Rho guanine nucleotide exchange factor 39 | **0,930** | 0,686506 | 0,720 | 0,128 |
| PTPN2 | protein tyrosine phosphatase, non-receptor type 2 | **0,930** | 0,015065 | 36,170 | 35,437 |
| DBN1 | drebrin 1 | **0,930** | 0,074857 | 15,398 | 9,769 |
| ARL2 | ADP ribosylation factor like GTPase 2 | **0,930** | 0,112807 | 14,069 | 10,408 |
| TXNDC17 | thioredoxin domain containing 17 | **0,930** | 0,099922 | 15,398 | 15,835 |
| ZNF185 | zinc finger protein 185 with LIM domain | **0,930** | 0,350196 | 1,994 | 2,235 |
| EIF2B3 | eukaryotic translation initiation factor 2B subunit gamma | **0,930** | 0,049105 | 15,288 | 14,558 |
| C1orf109 | chromosome 1 open reading frame 109 | **0,930** | 0,045212 | 15,620 | 17,878 |
| JAK3 | Janus kinase 3 | **0,930** | 0,195805 | 5,816 | 5,236 |
| PSMA7 | proteasome subunit alpha 7 | **0,930** | 0,027782 | 110,060 | 100,691 |
| TRIM41 | tripartite motif containing 41 | **0,930** | 0,015881 | 41,044 | 34,224 |
| NMRK1 | nicotinamide riboside kinase 1 | **0,930** | 0,047208 | 22,488 | 19,602 |
| HELB | DNA helicase B | **0,930** | 0,359295 | 2,714 | 2,682 |
| GDPD3 | glycerophosphodiester phosphodiesterase domain containing 3 | **0,930** | 0,587253 | 0,554 | 0,447 |
| SLC41A3 | solute carrier family 41 member 3 | **0,930** | 0,016994 | 40,490 | 33,777 |
| VDAC3 | voltage dependent anion channel 3 | **0,930** | 0,021454 | 38,773 | 34,607 |
| NABP1 | nucleic acid binding protein 1 | **0,930** | 0,032516 | 25,812 | 30,329 |
| LINC01647 | long intergenic non-protein coding RNA 1647 | **0,930** | 0,700498 | 0,443 | 0,575 |
| NT5E | 5'-nucleotidase ecto | **0,930** | 0,007893 | 1824,774 | 1596,311 |
| PBDC1 | polysaccharide biosynthesis domain containing 1 | **0,930** | 0,073866 | 14,623 | 14,111 |
| MTHFD1 | methylenetetrahydrofolate dehydrogenase, cyclohydrolase and formyltetrahydrofolate sy | **0,930** | 0,033319 | 28,803 | 23,305 |
| PCIF1 | PDX1 C-terminal inhibiting factor 1 | **0,930** | 0,022341 | 31,572 | 32,627 |
| ARL5A | ADP ribosylation factor like GTPase 5A | **0,930** | 0,096806 | 11,466 | 9,897 |
| FBXO9 | F-box protein 9 | **0,930** | 0,021117 | 35,837 | 29,562 |
| COX17 | COX17, cytochrome c oxidase copper chaperone | **0,930** | 0,105254 | 18,002 | 15,771 |
| NKRF | NFKB repressing factor | **0,930** | 0,134264 | 11,300 | 10,280 |

| | | | | | |
|---|---|---|---|---|---|
| GSTM3 | glutathione S-transferase mu 3 | **0,930** | 0,059575 | 19,774 | 10,854 |
| WDR5B | WD repeat domain 5B | **0,930** | 0,088777 | 14,124 | 14,494 |
| MRPS6 | mitochondrial ribosomal protein S6 | **0,930** | 0,01796 | 82,199 | 72,980 |
| FRMD6 | FERM domain containing 6 | **0,930** | 0,050042 | 267,645 | 206,618 |
| ZFPL1 | zinc finger protein like 1 | **0,930** | 0,055946 | 30,797 | 30,265 |
| POP5 | POP5 homolog, ribonuclease P/MRP subunit | **0,930** | 0,153275 | 12,186 | 10,471 |
| N4BP2 | NEDD4 binding protein 2 | **0,930** | 0,259254 | 9,693 | 7,790 |
| XPO6 | exportin 6 | **0,930** | 0,00622 | 55,390 | 46,547 |
| WDR6 | WD repeat domain 6 | **0,930** | 0,007146 | 72,893 | 73,172 |
| DTD1 | D-tyrosyl-tRNA deacylase 1 | **0,929** | 0,058602 | 19,553 | 19,474 |
| ABCC1 | ATP binding cassette subfamily C member 1 | **0,929** | 0,217807 | 8,142 | 7,407 |
| GPAA1 | glycosylphosphatidylinositol anchor attachment 1 | **0,929** | 0,022694 | 94,717 | 71,193 |
| RNF167 | ring finger protein 167 | **0,929** | 0,00503 | 48,577 | 48,462 |
| PPIH | peptidylprolyl isomerase H | **0,929** | 0,191157 | 6,314 | 5,810 |
| 1.1.1941 0:00:00 | TAM41 mitochondrial translocator assembly and maintenance homolog | **0,929** | 0,361207 | 1,662 | 2,171 |
| NPM1P27 | nucleophosmin 1 pseudogene 27 | **0,929** | 0,427883 | 2,049 | 1,213 |
| ATXN7L2 | ataxin 7 like 2 | **0,929** | 0,340154 | 2,770 | 3,320 |
| PCNX2 | pecanex homolog 2 (Drosophila) | **0,929** | 0,169383 | 5,871 | 7,598 |
| SCAND1 | SCAN domain containing 1 | **0,929** | 0,064491 | 33,677 | 30,009 |
| GSTK1 | glutathione S-transferase kappa 1 | **0,929** | 0,057322 | 12,795 | 9,450 |
| SGIP1 | SH3 domain GRB2 like endophilin interacting protein 1 | **0,929** | 0,435867 | 1,385 | 1,213 |
| AHCY | adenosylhomocysteinase | **0,929** | 0,040723 | 41,653 | 39,970 |
| COX6C | cytochrome c oxidase subunit 6C | **0,929** | 0,117784 | 28,526 | 21,198 |
| TMEM179B | transmembrane protein 179B | **0,929** | 0,033184 | 27,861 | 22,922 |
| TPM2 | tropomyosin 2 (beta) | **0,929** | 0,144935 | 23,541 | 13,281 |
| TAF6L | TATA-box binding protein associated factor 6 like | **0,929** | 0,181155 | 11,189 | 11,110 |
| STARD7-AS1 | STARD7 antisense RNA 1 | **0,929** | 0,357059 | 2,216 | 2,746 |
| LAGE3 | L antigen family member 3 | **0,929** | 0,049827 | 14,845 | 13,664 |
| LINC01816 | long intergenic non-protein coding RNA 1816 | **0,929** | 0,638342 | 0,499 | 0,511 |
| CORO1B | coronin 1B | **0,929** | 0,018307 | 46,195 | 31,861 |
| TINAGL1 | tubulointerstitial nephritis antigen like 1 | **0,929** | 0,095602 | 4,874 | 22,156 |
| WDR73 | WD repeat domain 73 | **0,929** | 0,024412 | 23,818 | 28,796 |
| TRIM29 | tripartite motif containing 29 | **0,929** | 0,528837 | 1,052 | 1,469 |

| | | | | | |
|---|---|---|---|---|---|
| GRAMD1C | GRAM domain containing 1C | **0,929** | 0,548111 | 0,886 | 0,830 |
| C3orf67 | chromosome 3 open reading frame 67 | **0,929** | 0,392506 | 1,662 | 1,405 |
| SSR4 | signal sequence receptor subunit 4 | **0,929** | 0,017484 | 141,134 | 78,216 |
| PCCB | propionyl-CoA carboxylase beta subunit | **0,929** | 0,048837 | 15,122 | 14,047 |
| ZDHHC1 | zinc finger DHHC-type containing 1 | **0,929** | 0,192482 | 5,041 | 4,469 |
| FZR1 | fizzy and cell division cycle 20 related 1 | **0,929** | 0,023043 | 33,843 | 27,455 |
| DDX51 | DEAD-box helicase 51 | **0,929** | 0,099361 | 11,853 | 14,685 |
| RRNAD1 | ribosomal RNA adenine dimethylase domain containing 1 | **0,929** | 0,019758 | 20,328 | 22,603 |
| MRPL35 | mitochondrial ribosomal protein L35 | **0,929** | 0,028974 | 16,617 | 15,962 |
| HSD17B12 | hydroxysteroid 17-beta dehydrogenase 12 | **0,929** | 0,016778 | 35,837 | 49,931 |
| DDX1 | DEAD-box helicase 1 | **0,929** | 0,00725 | 83,362 | 78,088 |
| PRPF6 | pre-mRNA processing factor 6 | **0,929** | 0,014247 | 69,293 | 64,233 |
| NOP58 | NOP58 ribonucleoprotein | **0,929** | 0,013298 | 34,508 | 40,353 |
| SCAMP2 | secretory carrier membrane protein 2 | **0,929** | 0,004171 | 93,997 | 82,494 |
| VCP | valosin containing protein | **0,929** | 0,012958 | 358,153 | 272,128 |
| CDC26 | cell division cycle 26 | **0,929** | 0,171192 | 4,764 | 4,597 |
| ENDOV | endonuclease V | **0,929** | 0,095401 | 10,192 | 11,812 |
| ZNF827 | zinc finger protein 827 | **0,929** | 0,09664 | 15,232 | 10,727 |
| RPL13 | ribosomal protein L13 | **0,929** | 0,049765 | 300,547 | 273,661 |
| FBXL15 | F-box and leucine rich repeat protein 15 | **0,929** | 0,185636 | 6,868 | 6,193 |
| GFRA2 | GDNF family receptor alpha 2 | **0,929** | 0,416275 | 2,714 | 0,702 |
| TFE3 | transcription factor binding to IGHM enhancer 3 | **0,929** | 0,007586 | 87,295 | 65,702 |
| AZI2 | 5-azacytidine induced 2 | **0,929** | 0,005113 | 37,388 | 25,093 |
| ECHDC1 | ethylmalonyl-CoA decarboxylase 1 | **0,929** | 0,007634 | 38,219 | 35,501 |
| SLCO5A1 | solute carrier organic anion transporter family member 5A1 | **0,928** | 0,487663 | 1,329 | 1,660 |
| ZNF221 | zinc finger protein 221 | **0,928** | 0,666093 | 0,554 | 1,085 |
| KLHDC4 | kelch domain containing 4 | **0,928** | 0,117457 | 12,020 | 14,302 |
| SRP72 | signal recognition particle 72 | **0,928** | 0,016763 | 84,359 | 76,492 |
| C12orf65 | chromosome 12 open reading frame 65 | **0,928** | 0,0685 | 11,687 | 11,685 |
| COG4 | component of oligomeric golgi complex 4 | **0,928** | 0,004746 | 37,998 | 30,967 |
| C19orf47 | chromosome 19 open reading frame 47 | **0,928** | 0,062121 | 10,192 | 9,322 |
| TMEM44 | transmembrane protein 44 | **0,928** | 0,165594 | 5,761 | 3,831 |
| DCBLD1 | discoidin, CUB and LCCL domain containing 1 | **0,928** | 0,065684 | 20,494 | 14,047 |

| | | | | | |
|---|---|---|---|---|---|
| CEP55 | centrosomal protein 55 | **0,928** | 0,452898 | 4,930 | 0,575 |
| FKRP | fukutin related protein | **0,928** | 0,042655 | 22,544 | 18,389 |
| UBE2G2 | ubiquitin conjugating enzyme E2 G2 | **0,928** | 0,014359 | 49,962 | 47,632 |
| ZC3H7B | zinc finger CCCH-type containing 7B | **0,928** | 0,008974 | 60,929 | 48,654 |
| GLI1 | GLI family zinc finger 1 | **0,928** | 0,579444 | 0,609 | 1,149 |
| STOML1 | stomatin like 1 | **0,928** | 0,033123 | 19,774 | 20,496 |
| RAB13 | RAB13, member RAS oncogene family | **0,928** | 0,014782 | 81,645 | 52,038 |
| WRAP53 | WD repeat containing antisense to TP53 | **0,928** | 0,109655 | 7,865 | 7,534 |
| CHST6 | carbohydrate sulfotransferase 6 | **0,928** | 0,162724 | 11,410 | 11,174 |
| LSG1 | large 60S subunit nuclear export GTPase 1 | **0,928** | 0,012152 | 26,421 | 28,605 |
| AP3D1 | adaptor related protein complex 3 delta 1 subunit | **0,928** | 0,007016 | 121,304 | 112,057 |
| TMEM168 | transmembrane protein 168 | **0,928** | 0,022884 | 18,777 | 18,133 |
| RBM45 | RNA binding motif protein 45 | **0,928** | 0,169135 | 6,924 | 6,385 |
| FAH | fumarylacetoacetate hydrolase | **0,928** | 0,090861 | 11,632 | 10,216 |
| MRPL16 | mitochondrial ribosomal protein L16 | **0,928** | 0,05152 | 18,445 | 18,133 |
| ZNF257 | zinc finger protein 257 | **0,928** | 0,677915 | 0,388 | 0,000 |
| ACOT13 | acyl-CoA thioesterase 13 | **0,928** | 0,022307 | 21,602 | 20,751 |
| BARX1-AS1 | BARX1 antisense RNA 1 (head to head) | **0,928** | 0,7278 | 0,388 | 0,319 |
| AP4M1 | adaptor related protein complex 4 mu 1 subunit | **0,928** | 0,068731 | 8,973 | 6,768 |
| FAM58A | family with sequence similarity 58 member A | **0,928** | 0,168871 | 10,247 | 7,151 |
| BAP1 | BRCA1 associated protein 1 | **0,928** | 0,004349 | 46,306 | 41,247 |
| TBC1D9 | TBC1 domain family member 9 | **0,928** | 0,135763 | 10,524 | 10,088 |
| ENDOG | endonuclease G | **0,928** | 0,161197 | 9,250 | 4,980 |
| SYNC | syncoilin, intermediate filament protein | **0,928** | 0,090951 | 19,830 | 6,513 |
| PPM1H | protein phosphatase, Mg2+/Mn2+ dependent 1H | **0,928** | 0,381206 | 1,994 | 2,682 |
| DNASE1L1 | deoxyribonuclease 1 like 1 | **0,928** | 0,009897 | 34,730 | 20,496 |
| ZNF500 | zinc finger protein 500 | **0,928** | 0,103102 | 9,250 | 9,577 |
| CENPX | centromere protein X | **0,928** | 0,086056 | 16,119 | 14,047 |
| NLRX1 | NLR family member X1 | **0,928** | 0,092655 | 10,247 | 7,534 |
| RTL8C | retrotransposon Gag like 8C | **0,928** | 0,037575 | 43,980 | 30,776 |
| UCK1 | uridine-cytidine kinase 1 | **0,928** | 0,023483 | 19,110 | 17,112 |
| TCERG1 | transcription elongation regulator 1 | **0,928** | 0,030398 | 23,652 | 27,072 |
| EFHC1 | EF-hand domain containing 1 | **0,928** | 0,041152 | 12,906 | 10,918 |

| | | | | | |
|---|---|---|---|---|---|
| MAK16 | MAK16 homolog | **0,928** | 0,026522 | 12,684 | 15,962 |
| PPP1CA | protein phosphatase 1 catalytic subunit alpha | **0,928** | 0,008671 | 68,351 | 58,167 |
| KCNH2 | potassium voltage-gated channel subfamily H member 2 | **0,928** | 0,678008 | 1,219 | 0,192 |
| GNB2 | G protein subunit beta 2 | **0,928** | 0,005348 | 139,362 | 115,377 |
| FKBP9P1 | FK506 binding protein 9 pseudogene 1 | **0,927** | 0,558729 | 1,385 | 0,255 |
| GAPDHP1 | glyceraldehyde-3-phosphate dehydrogenase pseudogene 1 | **0,927** | 0,335503 | 2,049 | 1,405 |
| SMOX | spermine oxidase | **0,927** | 0,021566 | 96,988 | 125,593 |
| ZPR1 | ZPR1 zinc finger | **0,927** | 0,007141 | 29,080 | 30,073 |
| NACAD | NAC alpha domain containing | **0,927** | 0,410424 | 2,382 | 1,469 |
| CDCP1 | CUB domain containing protein 1 | **0,927** | 0,205336 | 4,985 | 7,407 |
| C10orf76 | chromosome 10 open reading frame 76 | **0,927** | 0,014773 | 19,719 | 16,090 |
| LRRC29 | leucine rich repeat containing 29 | **0,927** | 0,429327 | 1,329 | 1,660 |
| PLXNB3 | plexin B3 | **0,927** | 0,353349 | 2,825 | 1,277 |
| DSTNP2 | destrin, actin depolymerizing factor pseudogene 2 | **0,927** | 0,19751 | 6,204 | 6,066 |
| UGP2 | UDP-glucose pyrophosphorylase 2 | **0,927** | 0,010688 | 172,596 | 122,400 |
| FOPNL | FGFR1OP N-terminal like | **0,927** | 0,61403 | 1,606 | 0,383 |
| NBPF8 | NBPF member 8 | **0,927** | 0,343806 | 3,600 | 2,171 |
| IMP3 | IMP3, U3 small nucleolar ribonucleoprotein | **0,927** | 0,018593 | 35,948 | 44,248 |
| NLN | neurolysin | **0,927** | 0,075684 | 11,743 | 9,450 |
| LYSMD1 | LysM domain containing 1 | **0,927** | 0,142063 | 5,594 | 5,044 |
| MANBAL | mannosidase beta like | **0,927** | 0,015752 | 34,453 | 32,500 |
| CEP78 | centrosomal protein 78 | **0,927** | 0,135824 | 8,142 | 7,470 |
| QDPR | quinoid dihydropteridine reductase | **0,927** | 0,093345 | 15,509 | 9,577 |
| CANX | calnexin | **0,927** | 0,303608 | 12,186 | 10,216 |
| CCNH | cyclin H | **0,927** | 0,024752 | 25,590 | 25,221 |
| ZNF688 | zinc finger protein 688 | **0,927** | 0,198111 | 7,090 | 4,597 |
| FAF1 | Fas associated factor 1 | **0,927** | 0,011561 | 19,442 | 21,454 |
| UBE2K | ubiquitin conjugating enzyme E2 K | **0,927** | 0,005565 | 61,538 | 51,718 |
| DHX35 | DEAH-box helicase 35 | **0,927** | 0,127305 | 7,312 | 9,194 |
| CWC15 | CWC15 spliceosome associated protein homolog | **0,927** | 0,022126 | 24,095 | 25,668 |
| MYH14 | myosin heavy chain 14 | **0,927** | 0,175515 | 9,472 | 7,790 |
| PSMB4 | proteasome subunit beta 4 | **0,927** | 0,008427 | 160,798 | 126,487 |
| DALRD3 | DALR anticodon binding domain containing 3 | **0,927** | 0,029263 | 15,232 | 13,217 |

| | | | | | |
|---|---|---|---|---|---|
| COX14 | COX14, cytochrome c oxidase assembly factor | **0,927** | 0,155164 | 8,973 | 7,023 |
| IARS | isoleucyl-tRNA synthetase | **0,927** | 0,012432 | 208,267 | 184,079 |
| GFPT1 | glutamine--fructose-6-phosphate transaminase 1 | **0,927** | 0,032766 | 223,776 | 180,440 |
| DDX18 | DEAD-box helicase 18 | **0,927** | 0,011477 | 51,624 | 58,742 |
| OBSL1 | obscurin like 1 | **0,927** | 0,018902 | 38,884 | 25,540 |
| TBRG4 | transforming growth factor beta regulator 4 | **0,927** | 0,005782 | 23,541 | 23,433 |
| CCDC170 | coiled-coil domain containing 170 | **0,927** | 0,29056 | 3,157 | 2,171 |
| NT5M | 5',3'-nucleotidase, mitochondrial | **0,927** | 0,613261 | 0,942 | 0,575 |
| CAMK2N1 | calcium/calmodulin dependent protein kinase II inhibitor 1 | **0,927** | 0,169245 | 6,481 | 5,555 |
| UBXN8 | UBX domain protein 8 | **0,927** | 0,107725 | 7,312 | 7,470 |
| CXXC1 | CXXC finger protein 1 | **0,927** | 0,006886 | 34,342 | 32,883 |
| ATP5SL | ATP5S like | **0,927** | 0,01489 | 19,497 | 18,261 |
| AES | amino-terminal enhancer of split | **0,927** | 0,019651 | 62,369 | 46,291 |
| NCAPD3 | non-SMC condensin II complex subunit D3 | **0,927** | 0,072341 | 14,734 | 13,728 |
| DOCK5 | dedicator of cytokinesis 5 | **0,927** | 0,080579 | 58,547 | 60,530 |
| ABT1 | activator of basal transcription 1 | **0,926** | 0,031165 | 19,497 | 18,389 |
| DZIP1L | DAZ interacting zinc finger protein 1 like | **0,926** | 0,233299 | 2,991 | 3,256 |
| KIF20B | kinesin family member 20B | **0,926** | 0,344472 | 5,816 | 3,576 |
| BCL2L1 | BCL2 like 1 | **0,926** | 0,005464 | 58,714 | 62,701 |
| HAX1 | HCLS1 associated protein X-1 | **0,926** | 0,007385 | 67,244 | 73,427 |
| C21orf59 | chromosome 21 open reading frame 59 | **0,926** | 0,081939 | 8,198 | 8,045 |
| ACSF3 | acyl-CoA synthetase family member 3 | **0,926** | 0,038669 | 11,521 | 10,280 |
| TMEM248 | transmembrane protein 248 | **0,926** | 0,00244 | 84,415 | 70,937 |
| APEX2 | apurinic/apyrimidinic endodeoxyribonuclease 2 | **0,926** | 0,078775 | 11,632 | 11,685 |
| C2orf40 | chromosome 2 open reading frame 40 | **0,926** | 0,01581 | 760,341 | 192,571 |
| SEC11C | SEC11 homolog C, signal peptidase complex subunit | **0,926** | 0,011125 | 59,267 | 52,740 |
| GYPC | glycophorin C (Gerbich blood group) | **0,926** | 0,022979 | 47,857 | 43,354 |
| UQCRQ | ubiquinol-cytochrome c reductase complex III subunit VII | **0,926** | 0,035958 | 33,733 | 32,116 |
| PDCD5 | programmed cell death 5 | **0,926** | 0,044317 | 53,618 | 42,907 |
| BUD13 | BUD13 homolog | **0,926** | 0,047593 | 12,518 | 12,387 |
| ADIRF-AS1 | ADIRF antisense RNA 1 | **0,926** | 0,235047 | 4,265 | 7,407 |
| XRCC5 | X-ray repair cross complementing 5 | **0,926** | 0,00226 | 154,982 | 142,960 |
| DYNLRB1 | dynein light chain roadblock-type 1 | **0,926** | 0,006961 | 62,092 | 52,868 |

| | | | | | |
|---|---|---|---|---|---|
| POLD4 | DNA polymerase delta 4, accessory subunit | **0,926** | 0,132994 | 5,151 | 3,512 |
| NDUFA4L2 | NDUFA4, mitochondrial complex associated like 2 | **0,926** | 0,002333 | 218,791 | 178,780 |
| SNHG3 | small nucleolar RNA host gene 3 | **0,926** | 0,110613 | 8,862 | 12,004 |
| GGA1 | golgi associated, gamma adaptin ear containing, ARF binding protein 1 | **0,926** | 0,009808 | 39,992 | 35,437 |
| SCRG1 | stimulator of chondrogenesis 1 | **0,926** | 0,016697 | 217,296 | 91,433 |
| ZBED5-AS1 | ZBED5 antisense RNA 1 | **0,926** | 0,321016 | 3,877 | 2,682 |
| ZNF503 | zinc finger protein 503 | **0,926** | 0,028086 | 20,827 | 24,263 |
| PSMB6 | proteasome subunit beta 6 | **0,926** | 0,007992 | 55,556 | 50,250 |
| DYM | dymeclin | **0,926** | 0,010999 | 27,474 | 26,881 |
| DNAJC10 | DnaJ heat shock protein family (Hsp40) member C10 | **0,926** | 0,016613 | 145,233 | 112,887 |
| TACC3 | transforming acidic coiled-coil containing protein 3 | **0,926** | 0,218313 | 10,635 | 3,065 |
| METTL17 | methyltransferase like 17 | **0,926** | 0,089982 | 10,524 | 10,982 |
| G3BP1 | G3BP stress granule assembly factor 1 | **0,926** | 0,001266 | 159,358 | 143,918 |
| APBA3 | amyloid beta precursor protein binding family A member 3 | **0,926** | 0,018581 | 25,867 | 22,667 |
| PDP1 | pyruvate dehyrogenase phosphatase catalytic subunit 1 | **0,926** | 0,111319 | 11,078 | 6,385 |
| VPS25 | vacuolar protein sorting 25 homolog | **0,926** | 0,015893 | 31,240 | 29,626 |
| PIP5KL1 | phosphatidylinositol-4-phosphate 5-kinase like 1 | **0,926** | 0,246316 | 2,326 | 3,639 |
| ENTPD6 | ectonucleoside triphosphate diphosphohydrolase 6 (putative) | **0,926** | 0,00353 | 102,250 | 69,596 |
| ITGA9-AS1 | ITGA9 antisense RNA 1 | **0,926** | 0,368024 | 1,274 | 1,852 |
| TRMT61A | tRNA methyltransferase 61A | **0,926** | 0,033791 | 18,113 | 19,985 |
| SVBP | small vasohibin binding protein | **0,925** | 0,230341 | 6,481 | 4,150 |
| ZMAT5 | zinc finger matrin-type 5 | **0,925** | 0,109335 | 7,367 | 6,449 |
| HOXA7 | homeobox A7 | **0,925** | 0,03105 | 12,851 | 12,834 |
| HHIPL1 | HHIP like 1 | **0,925** | 0,019659 | 37,555 | 21,773 |
| RNF220 | ring finger protein 220 | **0,925** | 0,006611 | 51,291 | 55,677 |
| EIF3I | eukaryotic translation initiation factor 3 subunit I | **0,925** | 0,002546 | 99,481 | 103,501 |
| PFN2 | profilin 2 | **0,925** | 0,014275 | 85,412 | 69,022 |
| ZBTB45 | zinc finger and BTB domain containing 45 | **0,925** | 0,061913 | 11,743 | 10,599 |
| MYC | MYC proto-oncogene, bHLH transcription factor | **0,925** | 0,012629 | 49,519 | 57,209 |
| TNFAIP8L3 | TNF alpha induced protein 8 like 3 | **0,925** | 0,183968 | 5,317 | 3,256 |
| ZSCAN16 | zinc finger and SCAN domain containing 16 | **0,925** | 0,405962 | 2,216 | 2,299 |
| UBAC2 | UBA domain containing 2 | **0,925** | 0,005211 | 29,800 | 28,413 |
| PRRC1 | proline rich coiled-coil 1 | **0,925** | 0,012162 | 87,240 | 68,447 |

| | | | | | |
|---|---|---|---|---|---|
| CHMP6 | charged multivesicular body protein 6 | **0,925** | 0,027153 | 20,107 | 16,409 |
| SLC25A12 | solute carrier family 25 member 12 | **0,925** | 0,058999 | 11,632 | 10,727 |
| NOL9 | nucleolar protein 9 | **0,925** | 0,135827 | 13,903 | 14,111 |
| LIX1L-AS1 | LIX1L antisense RNA 1 | **0,925** | 0,629178 | 0,443 | 0,511 |
| MXRA5 | matrix remodeling associated 5 | **0,925** | 0,232814 | 7,533 | 10,663 |
| LARS2 | leucyl-tRNA synthetase 2, mitochondrial | **0,925** | 0,091739 | 8,032 | 5,810 |
| PSMG1 | proteasome assembly chaperone 1 | **0,925** | 0,034168 | 16,672 | 13,281 |
| PHKG2 | phosphorylase kinase catalytic subunit gamma 2 | **0,925** | 0,076995 | 7,312 | 7,279 |
| EDEM2 | ER degradation enhancing alpha-mannosidase like protein 2 | **0,925** | 0,054398 | 12,352 | 10,088 |
| CARNMT1 | carnosine N-methyltransferase 1 | **0,925** | 0,13464 | 5,262 | 5,810 |
| LETM1 | leucine zipper and EF-hand containing transmembrane protein 1 | **0,925** | 0,010101 | 27,806 | 27,775 |
| NKIRAS2 | NFKB inhibitor interacting Ras like 2 | **0,925** | 0,01827 | 25,590 | 25,987 |
| ABCF3 | ATP binding cassette subfamily F member 3 | **0,925** | 0,015425 | 34,730 | 32,627 |
| SLC24A1 | solute carrier family 24 member 1 | **0,925** | 0,133194 | 10,912 | 8,875 |
| MOB3A | MOB kinase activator 3A | **0,925** | 0,090658 | 21,658 | 18,453 |
| MDFIC | MyoD family inhibitor domain containing | **0,925** | 0,016317 | 74,888 | 43,993 |
| ABCE1 | ATP binding cassette subfamily E member 1 | **0,925** | 0,007881 | 48,023 | 52,421 |
| SEC24A | SEC24 homolog A, COPII coat complex component | **0,925** | 0,035041 | 55,556 | 47,185 |
| RRAS | related RAS viral (r-ras) oncogene homolog | **0,925** | 0,012793 | 59,267 | 40,545 |
| ACAP1 | ArfGAP with coiled-coil, ankyrin repeat and PH domains 1 | **0,925** | 0,566536 | 0,554 | 0,894 |
| PBX4 | PBX homeobox 4 | **0,925** | 0,478565 | 0,942 | 0,575 |
| IRF2BP1 | interferon regulatory factor 2 binding protein 1 | **0,925** | 0,01462 | 34,342 | 32,053 |
| KIFC3 | kinesin family member C3 | **0,925** | 0,030004 | 52,399 | 44,631 |
| ZKSCAN4 | zinc finger with KRAB and SCAN domains 4 | **0,925** | 0,385559 | 2,659 | 1,915 |
| VWCE | von Willebrand factor C and EGF domains | **0,925** | 0,651725 | 0,609 | 0,319 |
| MRPL18 | mitochondrial ribosomal protein L18 | **0,925** | 0,011925 | 22,544 | 24,582 |
| LYAR | Ly1 antibody reactive | **0,924** | 0,01554 | 15,565 | 18,389 |
| RBFOX2 | RNA binding protein, fox-1 homolog 2 | **0,924** | 0,366124 | 3,656 | 2,554 |
| ZCRB1 | zinc finger CCHC-type and RNA binding motif containing 1 | **0,924** | 0,062503 | 28,747 | 24,965 |
| UQCRC1 | ubiquinol-cytochrome c reductase core protein I | **0,924** | 0,009825 | 76,272 | 63,467 |
| FAM86FP | family with sequence similarity 86 member F, pseudogene | **0,924** | 0,525966 | 0,388 | 0,894 |
| CCNY | cyclin Y | **0,924** | 0,003057 | 68,573 | 56,443 |
| EMP3 | epithelial membrane protein 3 | **0,924** | 0,007489 | 195,250 | 136,639 |

| | | | | | |
|---|---|---|---|---|---|
| SLC25A39 | solute carrier family 25 member 39 | **0,924** | 0,010975 | 42,373 | 37,799 |
| PARD6B | par-6 family cell polarity regulator beta | **0,924** | 0,499566 | 0,775 | 0,511 |
| COL13A1 | collagen type XIII alpha 1 chain | **0,924** | 0,253764 | 1,883 | 3,192 |
| TUBG1 | tubulin gamma 1 | **0,924** | 0,015391 | 17,669 | 16,984 |
| CALR | calreticulin | **0,924** | 0,008749 | 747,767 | 474,149 |
| KSR1 | kinase suppressor of ras 1 | **0,924** | 0,075777 | 7,145 | 16,984 |
| ECHS1 | enoyl-CoA hydratase, short chain 1 | **0,924** | 0,026241 | 38,441 | 34,415 |
| MPHOSPH6 | M-phase phosphoprotein 6 | **0,924** | 0,061606 | 12,075 | 10,216 |
| LINC00888 | long intergenic non-protein coding RNA 888 | **0,924** | 0,219106 | 6,758 | 5,683 |
| FAM107B | family with sequence similarity 107 member B | **0,924** | 0,001903 | 109,728 | 102,160 |
| LRRC75A-AS1 | LRRC75A antisense RNA 1 | **0,924** | 0,00705 | 105,241 | 84,282 |
| MFN2 | mitofusin 2 | **0,924** | 0,003777 | 52,510 | 49,101 |
| SCO1 | SCO1, cytochrome c oxidase assembly protein | **0,924** | 0,01389 | 28,415 | 29,882 |
| VANGL1 | VANGL planar cell polarity protein 1 | **0,924** | 0,030147 | 20,605 | 17,239 |
| RPL26L1 | ribosomal protein L26 like 1 | **0,924** | 0,108858 | 9,970 | 10,599 |
| TNIP1 | TNFAIP3 interacting protein 1 | **0,924** | 0,004123 | 363,249 | 339,937 |
| DPP9 | dipeptidyl peptidase 9 | **0,924** | 0,006228 | 51,679 | 42,907 |
| HSP90AB1 | heat shock protein 90 alpha family class B member 1 | **0,924** | 0,006064 | 413,598 | 367,775 |
| GK5 | glycerol kinase 5 (putative) | **0,924** | 0,212208 | 6,038 | 7,023 |
| FAM104B | family with sequence similarity 104 member B | **0,924** | 0,211949 | 4,708 | 4,916 |
| TIMM10B | translocase of inner mitochondrial membrane 10B | **0,924** | 0,019929 | 23,929 | 22,539 |
| MYO6 | myosin VI | **0,924** | 0,014618 | 40,989 | 23,561 |
| STN1 | STN1, CST complex subunit | **0,924** | 0,0565 | 11,078 | 9,641 |
| PUF60 | poly(U) binding splicing factor 60 | **0,924** | 0,012971 | 18,334 | 22,667 |
| TMEM250 | transmembrane protein 250 | **0,924** | 0,003881 | 51,513 | 43,354 |
| MSLN | mesothelin | **0,924** | 0,455319 | 2,382 | 0,255 |
| TACO1 | translational activator of cytochrome c oxidase I | **0,924** | 0,070944 | 11,355 | 9,450 |
| ARPC5L | actin related protein 2/3 complex subunit 5 like | **0,924** | 0,020628 | 26,476 | 23,497 |
| SIN3B | SIN3 transcription regulator family member B | **0,924** | 0,00801 | 27,529 | 25,668 |
| FOXP1 | forkhead box P1 | **0,924** | 0,010425 | 50,294 | 43,737 |
| SCAP | SREBF chaperone | **0,924** | 0,016355 | 49,076 | 51,782 |
| LINC01869 | long intergenic non-protein coding RNA 1869 | **0,924** | 0,620073 | 0,499 | 1,213 |
| SH3RF1 | SH3 domain containing ring finger 1 | **0,924** | 0,087494 | 11,964 | 11,876 |

| | | | | | |
|---|---|---|---|---|---|
| PDZD11 | PDZ domain containing 11 | **0,924** | 0,024246 | 16,562 | 13,792 |
| GTPBP4 | GTP binding protein 4 | **0,924** | 0,004247 | 52,344 | 55,741 |
| TAGLN2 | transgelin 2 | **0,923** | 0,001011 | 202,285 | 184,718 |
| BRIX1 | BRX1, biogenesis of ribosomes | **0,923** | 0,009415 | 16,728 | 22,347 |
| PARD6G-AS1 | PARD6G antisense RNA 1 | **0,923** | 0,582102 | 0,665 | 0,319 |
| LINC01024 | long intergenic non-protein coding RNA 1024 | **0,923** | 0,381045 | 1,440 | 2,490 |
| FAM213A | family with sequence similarity 213 member A | **0,923** | 0,298457 | 6,868 | 3,895 |
| RFC3 | replication factor C subunit 3 | **0,923** | 0,197365 | 3,490 | 4,342 |
| BAG1 | BCL2 associated athanogene 1 | **0,923** | 0,060334 | 60,763 | 54,719 |
| BEST4 | bestrophin 4 | **0,923** | 0,418665 | 1,329 | 1,596 |
| ING2 | inhibitor of growth family member 2 | **0,923** | 0,094766 | 9,306 | 7,343 |
| PCDHGB3 | protocadherin gamma subfamily B, 3 | **0,923** | 0,602365 | 0,443 | 0,830 |
| EXOC7 | exocyst complex component 7 | **0,923** | 0,00522 | 94,717 | 75,790 |
| SNAPC3 | small nuclear RNA activating complex polypeptide 3 | **0,923** | 0,012392 | 22,156 | 19,410 |
| TFG | TRK-fused gene | **0,923** | 0,002085 | 135,207 | 92,199 |
| MED25 | mediator complex subunit 25 | **0,923** | 0,008517 | 28,194 | 20,240 |
| TRIM5 | tripartite motif containing 5 | **0,923** | 0,177764 | 5,428 | 4,853 |
| SLC2A9 | solute carrier family 2 member 9 | **0,923** | 0,588476 | 0,222 | 0,575 |
| TMEM220-AS1 | TMEM220 antisense RNA 1 | **0,923** | 0,613616 | 0,720 | 0,319 |
| PPM1M | protein phosphatase, Mg2+/Mn2+ dependent 1M | **0,923** | 0,127616 | 7,035 | 5,300 |
| PPP2R5B | protein phosphatase 2 regulatory subunit B'beta | **0,923** | 0,018323 | 16,949 | 17,495 |
| IFT57 | intraflagellar transport 57 | **0,923** | 0,02984 | 19,276 | 19,985 |
| RBBP8 | RB binding protein 8, endonuclease | **0,923** | 0,015951 | 17,171 | 13,664 |
| SLC4A1AP | solute carrier family 4 member 1 adaptor protein | **0,923** | 0,004406 | 27,197 | 25,604 |
| TARS | threonyl-tRNA synthetase | **0,923** | 0,000716 | 131,164 | 107,268 |
| RSU1 | Ras suppressor protein 1 | **0,923** | 0,003733 | 47,912 | 43,993 |
| ARF3 | ADP ribosylation factor 3 | **0,923** | 0,001536 | 64,640 | 48,909 |
| DNAJC7 | DnaJ heat shock protein family (Hsp40) member C7 | **0,923** | 0,004291 | 43,869 | 39,012 |
| KLRA1P | killer cell lectin like receptor A1, pseudogene | **0,923** | 0,456838 | 1,052 | 0,575 |
| SCML2 | sex comb on midleg like 2 (Drosophila) | **0,923** | 0,279425 | 3,490 | 3,767 |
| FAM120C | family with sequence similarity 120C | **0,923** | 0,069786 | 12,629 | 10,854 |
| SPATA5L1 | spermatogenesis associated 5 like 1 | **0,923** | 0,040089 | 10,469 | 10,918 |
| ANG | angiogenin | **0,923** | 0,060632 | 7,312 | 3,384 |

| | | | | | |
|---|---|---|---|---|---|
| ACBD6 | acyl-CoA binding domain containing 6 | **0,923** | 0,030493 | 14,734 | 11,238 |
| TMEM87A | transmembrane protein 87A | **0,923** | 0,003808 | 68,795 | 75,598 |
| KPNB1 | karyopherin subunit beta 1 | **0,923** | 0,001715 | 130,278 | 120,165 |
| COL8A2 | collagen type VIII alpha 2 chain | **0,923** | 0,003663 | 61,538 | 52,485 |
| CHID1 | chitinase domain containing 1 | **0,923** | 0,010291 | 47,137 | 28,094 |
| PYURF | PIGY upstream reading frame | **0,923** | 0,032891 | 31,462 | 29,882 |
| AKR1C3 | aldo-keto reductase family 1 member C3 | **0,923** | 0,028569 | 10,967 | 7,151 |
| TMEM165 | transmembrane protein 165 | **0,923** | 0,003209 | 172,540 | 124,380 |
| TIPIN | TIMELESS interacting protein | **0,923** | 0,155136 | 4,320 | 5,300 |
| TAGLN | transgelin | **0,923** | 0,239864 | 4,542 | 2,809 |
| MED10 | mediator complex subunit 10 | **0,923** | 0,017896 | 28,581 | 31,861 |
| ACAD9 | acyl-CoA dehydrogenase family member 9 | **0,923** | 0,00702 | 18,999 | 15,260 |
| DSE | dermatan sulfate epimerase | **0,923** | 0,010775 | 76,328 | 78,216 |
| RPL23A | ribosomal protein L23a | **0,922** | 0,016675 | 124,406 | 105,991 |
| YKT6 | YKT6 v-SNARE homolog | **0,922** | 0,002258 | 85,855 | 74,832 |
| ARPC3 | actin related protein 2/3 complex subunit 3 | **0,922** | 0,007386 | 61,483 | 49,292 |
| CD46 | CD46 molecule | **0,922** | 0,001696 | 126,733 | 89,581 |
| CASP4 | caspase 4 | **0,922** | 0,004486 | 49,574 | 43,609 |
| YPEL3 | yippee like 3 | **0,922** | 0,02633 | 24,095 | 15,388 |
| ABHD17C | abhydrolase domain containing 17C | **0,922** | 0,067428 | 8,419 | 9,961 |
| CH17-340M24.3 | uncharacterized protein BC009467 | **0,922** | 0,368162 | 1,385 | 1,852 |
| RGS10 | regulator of G protein signaling 10 | **0,922** | 0,080684 | 13,294 | 13,919 |
| AP3S1 | adaptor related protein complex 3 sigma 1 subunit | **0,922** | 0,036547 | 17,060 | 9,641 |
| PPM1F | protein phosphatase, Mg2+/Mn2+ dependent 1F | **0,922** | 0,006879 | 24,372 | 25,732 |
| HINT2 | histidine triad nucleotide binding protein 2 | **0,922** | 0,061387 | 14,235 | 11,876 |
| GCN1 | GCN1, eIF2 alpha kinase activator homolog | **0,922** | 0,00769 | 96,047 | 93,668 |
| PARVB | parvin beta | **0,922** | 0,198345 | 3,767 | 5,108 |
| DFFA | DNA fragmentation factor subunit alpha | **0,922** | 0,011862 | 31,572 | 31,478 |
| ATP5L | ATP synthase, H+ transporting, mitochondrial Fo complex subunit G | **0,922** | 0,020425 | 48,023 | 45,525 |
| HACL1 | 2-hydroxyacyl-CoA lyase 1 | **0,922** | 0,061129 | 7,035 | 6,896 |
| UQCR11 | ubiquinol-cytochrome c reductase, complex III subunit XI | **0,922** | 0,018474 | 21,768 | 16,984 |
| MAZ | MYC associated zinc finger protein | **0,922** | 0,00893 | 52,011 | 34,670 |
| CMPK1 | cytidine/uridine monophosphate kinase 1 | **0,922** | 0,004084 | 91,615 | 74,321 |

| | | | | | |
|---|---|---|---|---|---|
| DDX59 | DEAD-box helicase 59 | **0,922** | 0,042793 | 10,856 | 7,981 |
| ECHDC3 | enoyl-CoA hydratase domain containing 3 | **0,922** | 0,24878 | 6,924 | 2,235 |
| EIF4G1 | eukaryotic translation initiation factor 4 gamma 1 | **0,922** | 0,001773 | 294,233 | 246,588 |
| RNF25 | ring finger protein 25 | **0,922** | 0,017564 | 18,777 | 18,197 |
| MCRS1 | microspherule protein 1 | **0,922** | 0,018913 | 30,354 | 30,648 |
| PA2G4 | proliferation-associated 2G4 | **0,922** | 0,002667 | 48,577 | 47,249 |
| ZNF839 | zinc finger protein 839 | **0,922** | 0,084471 | 5,927 | 6,449 |
| TRIT1 | tRNA isopentenyltransferase 1 | **0,922** | 0,059305 | 4,653 | 11,557 |
| FUNDC2 | FUN14 domain containing 2 | **0,922** | 0,011958 | 33,622 | 32,819 |
| ALKBH8 | alkB homolog 8, tRNA methyltransferase | **0,922** | 0,152155 | 4,043 | 4,853 |
| TRMT13 | tRNA methyltransferase 13 homolog | **0,922** | 0,04134 | 7,921 | 10,344 |
| NR1H3 | nuclear receptor subfamily 1 group H member 3 | **0,922** | 0,335491 | 2,160 | 2,490 |
| TVP23B | trans-golgi network vesicle protein 23 homolog B | **0,922** | 0,02734 | 39,327 | 31,606 |
| TREML2 | triggering receptor expressed on myeloid cells like 2 | **0,922** | 0,488384 | 0,665 | 0,447 |
| TEX264 | testis expressed 264 | **0,922** | 0,025798 | 27,030 | 16,856 |
| FAT1 | FAT atypical cadherin 1 | **0,922** | 0,036709 | 307,194 | 278,066 |
| ATP5S | ATP synthase, H+ transporting, mitochondrial Fo complex subunit s (factor B) | **0,922** | 0,050877 | 9,915 | 12,131 |
| COPS7B | COP9 signalosome subunit 7B | **0,922** | 0,071939 | 11,687 | 9,641 |
| PDE5A | phosphodiesterase 5A | **0,921** | 0,453738 | 2,326 | 0,319 |
| RCN1P2 | reticulocalbin 1 pseudogene 2 | **0,921** | 0,110367 | 7,090 | 0,319 |
| ECM1 | extracellular matrix protein 1 | **0,921** | 0,027402 | 29,357 | 18,516 |
| PISD | phosphatidylserine decarboxylase | **0,921** | 0,02443 | 16,008 | 15,899 |
| SUV39H2 | suppressor of variegation 3-9 homolog 2 | **0,921** | 0,117591 | 5,539 | 5,491 |
| ZNF334 | zinc finger protein 334 | **0,921** | 0,168045 | 4,376 | 4,278 |
| LMAN2 | lectin, mannose binding 2 | **0,921** | 0,010369 | 142,574 | 96,733 |
| PSMD1 | proteasome 26S subunit, non-ATPase 1 | **0,921** | 0,004545 | 100,644 | 68,830 |
| SF3B2 | splicing factor 3b subunit 2 | **0,921** | 0,003606 | 139,251 | 131,020 |
| CTBP1 | C-terminal binding protein 1 | **0,921** | 0,003251 | 79,485 | 65,382 |
| RNF7 | ring finger protein 7 | **0,921** | 0,021368 | 31,462 | 28,796 |
| PNKP | polynucleotide kinase 3'-phosphatase | **0,921** | 0,056327 | 15,786 | 18,133 |
| ZNHIT6 | zinc finger HIT-type containing 6 | **0,921** | 0,025569 | 23,098 | 20,113 |
| PDE4D | phosphodiesterase 4D | **0,921** | 0,116109 | 19,553 | 22,092 |
| RMDN3 | regulator of microtubule dynamics 3 | **0,921** | 0,041504 | 14,623 | 11,493 |

| | | | | | |
|---|---|---|---|---|---|
| MYL9 | myosin light chain 9 | **0,921** | 0,316296 | 2,825 | 1,852 |
| CCNJ | cyclin J | **0,921** | 0,228359 | 3,656 | 2,682 |
| ETAA1 | ETAA1, ATR kinase activator | **0,921** | 0,091174 | 9,970 | 10,152 |
| CCT7 | chaperonin containing TCP1 subunit 7 | **0,921** | 0,004863 | 145,067 | 144,237 |
| CBX3 | chromobox 3 | **0,921** | 0,002099 | 60,431 | 47,504 |
| ZNF584 | zinc finger protein 584 | **0,921** | 0,06616 | 7,256 | 7,854 |
| PAXIP1-AS1 | PAXIP1 antisense RNA 1 (head to head) | **0,921** | 0,288994 | 2,880 | 3,256 |
| ZNF320 | zinc finger protein 320 | **0,921** | 0,529642 | 0,775 | 0,894 |
| CFAP69 | cilia and flagella associated protein 69 | **0,921** | 0,081937 | 4,542 | 5,938 |
| TMEM261 | transmembrane protein 261 | **0,921** | 0,04785 | 20,494 | 20,113 |
| FAM212A | family with sequence similarity 212 member A | **0,921** | 0,533736 | 0,775 | 0,766 |
| CHKB-AS1 | CHKB antisense RNA 1 (head to head) | **0,921** | 0,490642 | 0,665 | 0,511 |
| COMMD5 | COMM domain containing 5 | **0,921** | 0,028546 | 15,897 | 12,578 |
| HEATR1 | HEAT repeat containing 1 | **0,921** | 0,014117 | 30,243 | 35,309 |
| TMEM177 | transmembrane protein 177 | **0,921** | 0,298959 | 1,994 | 1,852 |
| ORC5 | origin recognition complex subunit 5 | **0,921** | 0,067712 | 11,577 | 12,770 |
| PIGS | phosphatidylinositol glycan anchor biosynthesis class S | **0,921** | 0,021706 | 34,120 | 26,306 |
| PDCD11 | programmed cell death 11 | **0,921** | 0,010419 | 36,004 | 42,269 |
| TMEM41B | transmembrane protein 41B | **0,921** | 0,024845 | 26,975 | 21,134 |
| ERH | enhancer of rudimentary homolog (Drosophila) | **0,921** | 0,005787 | 66,136 | 71,129 |
| HTATIP2 | HIV-1 Tat interactive protein 2 | **0,921** | 0,021214 | 24,870 | 16,601 |
| PHF23 | PHD finger protein 23 | **0,921** | 0,006854 | 26,255 | 19,410 |
| METTL12 | methyltransferase like 12 | **0,921** | 0,161694 | 3,933 | 4,853 |
| RNF26 | ring finger protein 26 | **0,921** | 0,008928 | 23,319 | 19,921 |
| PAPSS1 | 3'-phosphoadenosine 5'-phosphosulfate synthase 1 | **0,921** | 0,001028 | 87,904 | 57,976 |
| AP4B1 | adaptor related protein complex 4 beta 1 subunit | **0,921** | 0,027334 | 14,955 | 17,750 |
| PAICS | phosphoribosylaminoimidazole carboxylase and phosphoribosylaminoimidazolesuccinocar | **0,921** | 0,003255 | 61,206 | 57,720 |
| THG1L | tRNA-histidine guanylyltransferase 1 like | **0,921** | 0,017038 | 12,241 | 15,324 |
| TCF25 | transcription factor 25 | **0,920** | 0,018443 | 44,312 | 37,927 |
| RFX3 | regulatory factor X3 | **0,920** | 0,138808 | 6,093 | 6,130 |
| SMPD4 | sphingomyelin phosphodiesterase 4 | **0,920** | 0,016685 | 27,086 | 28,222 |
| 9.syys | septin 9 | **0,920** | 0,002921 | 191,096 | 159,497 |
| PTGER2 | prostaglandin E receptor 2 | **0,920** | 0,002992 | 95,050 | 58,103 |

| | | | | | |
|---|---|---|---|---|---|
| ANP32A | acidic nuclear phosphoprotein 32 family member A | **0,920** | 0,00924 | 33,566 | 35,564 |
| SCAF1 | SR-related CTD associated factor 1 | **0,920** | 0,004884 | 92,834 | 74,960 |
| LINC00294 | long intergenic non-protein coding RNA 294 | **0,920** | 0,03266 | 12,130 | 9,705 |
| RPS20P14 | ribosomal protein S20 pseudogene 14 | **0,920** | 0,557769 | 0,388 | 0,447 |
| U2AF2 | U2 small nuclear RNA auxiliary factor 2 | **0,920** | 0,000652 | 123,852 | 113,461 |
| RUNX1T1 | RUNX1 translocation partner 1 | **0,920** | 0,47285 | 1,329 | 2,618 |
| FAM86C1 | family with sequence similarity 86 member C1 | **0,920** | 0,25105 | 3,102 | 3,001 |
| BAD | BCL2 associated agonist of cell death | **0,920** | 0,053573 | 9,638 | 4,980 |
| SH3BP5L | SH3 binding domain protein 5 like | **0,920** | 0,011608 | 24,538 | 22,475 |
| DENND1A | DENN domain containing 1A | **0,920** | 0,030122 | 12,851 | 14,685 |
| HDAC1 | histone deacetylase 1 | **0,920** | 0,004233 | 43,426 | 45,142 |
| CLIP1 | CAP-Gly domain containing linker protein 1 | **0,920** | 0,025367 | 213,584 | 193,593 |
| TXN2 | thioredoxin 2 | **0,920** | 0,029728 | 26,144 | 26,562 |
| MRPS15 | mitochondrial ribosomal protein S15 | **0,920** | 0,010989 | 17,171 | 14,749 |
| FAM155A | family with sequence similarity 155 member A | **0,920** | 0,194992 | 5,317 | 2,746 |
| LCMT1 | leucine carboxyl methyltransferase 1 | **0,920** | 0,027842 | 14,900 | 13,025 |
| SLC25A32 | solute carrier family 25 member 32 | **0,920** | 0,005211 | 41,266 | 44,503 |
| UBE3D | ubiquitin protein ligase E3D | **0,920** | 0,430346 | 1,385 | 1,149 |
| TARBP1 | TAR (HIV-1) RNA binding protein 1 | **0,920** | 0,200283 | 3,877 | 3,831 |
| ZNF33B | zinc finger protein 33B | **0,920** | 0,222514 | 5,373 | 2,873 |
| RANGAP1 | Ran GTPase activating protein 1 | **0,920** | 0,012609 | 62,757 | 51,208 |
| GART | phosphoribosylglycinamide formyltransferase, phosphoribosylglycinamide synthetase, pho | **0,920** | 0,009552 | 37,721 | 37,033 |
| NTF3 | neurotrophin 3 | **0,920** | 0,359165 | 0,831 | 1,852 |
| COMMD3 | COMM domain containing 3 | **0,920** | 0,021683 | 18,057 | 17,942 |
| YTHDF3-AS1 | YTHDF3 antisense RNA 1 (head to head) | **0,920** | 0,561595 | 0,554 | 1,149 |
| COX7A1 | cytochrome c oxidase subunit 7A1 | **0,920** | 0,063697 | 6,591 | 3,512 |
| FAM204A | family with sequence similarity 204 member A | **0,920** | 0,019798 | 18,888 | 16,920 |
| FBXW8 | F-box and WD repeat domain containing 8 | **0,920** | 0,023641 | 17,891 | 14,749 |
| TSPOAP1 | TSPO associated protein 1 | **0,920** | 0,507158 | 0,886 | 1,532 |
| GMPS | guanine monophosphate synthase | **0,920** | 0,003629 | 25,978 | 19,985 |
| HNRNPUL2 | heterogeneous nuclear ribonucleoprotein U like 2 | **0,920** | 0,020883 | 24,649 | 17,367 |
| FAM96B | family with sequence similarity 96 member B | **0,920** | 0,006403 | 31,295 | 32,691 |
| PARP10 | poly(ADP-ribose) polymerase family member 10 | **0,920** | 0,068171 | 12,130 | 12,642 |

| | | | | | |
|---|---|---|---|---|---|
| INO80E | INO80 complex subunit E | **0,919** | 0,016545 | 23,596 | 24,327 |
| FCER1G | Fc fragment of IgE receptor Ig | **0,919** | 0,280697 | 2,216 | 3,065 |
| WDR89 | WD repeat domain 89 | **0,919** | 0,009553 | 15,675 | 15,771 |
| NOP53 | NOP53 ribosome biogenesis factor | **0,919** | 0,016558 | 86,353 | 79,812 |
| ZNF444 | zinc finger protein 444 | **0,919** | 0,025592 | 17,393 | 13,217 |
| GLT8D1 | glycosyltransferase 8 domain containing 1 | **0,919** | 0,007878 | 32,514 | 23,561 |
| HOXC4 | homeobox C4 | **0,919** | 0,220123 | 3,157 | 2,171 |
| CYP19A1 | cytochrome P450 family 19 subfamily A member 1 | **0,919** | 0,273538 | 3,988 | 1,341 |
| MRPS35 | mitochondrial ribosomal protein S35 | **0,919** | 0,049752 | 14,845 | 14,366 |
| GPR108 | G protein-coupled receptor 108 | **0,919** | 0,002124 | 99,370 | 83,835 |
| C2orf82 | chromosome 2 open reading frame 82 | **0,919** | 0,067287 | 116,042 | 43,929 |
| BRD9 | bromodomain containing 9 | **0,919** | 0,035641 | 15,620 | 16,154 |
| USP5 | ubiquitin specific peptidase 5 | **0,919** | 0,004349 | 56,387 | 47,377 |
| WDFY2 | WD repeat and FYVE domain containing 2 | **0,919** | 0,070827 | 8,530 | 11,557 |
| PHTF1 | putative homeodomain transcription factor 1 | **0,919** | 0,00806 | 25,147 | 15,962 |
| AP2A1 | adaptor related protein complex 2 alpha 1 subunit | **0,919** | 0,001846 | 64,086 | 54,911 |
| PEF1 | penta-EF-hand domain containing 1 | **0,919** | 0,00407 | 62,812 | 58,486 |
| FAM129C | family with sequence similarity 129 member C | **0,919** | 0,490282 | 0,554 | 0,638 |
| MMS22L | MMS22 like, DNA repair protein | **0,919** | 0,191299 | 4,597 | 2,809 |
| IDH1 | isocitrate dehydrogenase (NADP(+)) 1, cytosolic | **0,919** | 0,014254 | 40,878 | 30,648 |
| TMEM243 | transmembrane protein 243 | **0,919** | 0,007103 | 19,276 | 19,602 |
| PPRC1 | peroxisome proliferator-activated receptor gamma, coactivator-related 1 | **0,919** | 0,005942 | 28,803 | 35,692 |
| PTH1R | parathyroid hormone 1 receptor | **0,919** | 0,31171 | 2,603 | 1,724 |
| CKAP2L | cytoskeleton associated protein 2 like | **0,919** | 0,105483 | 12,186 | 4,789 |
| HERC6 | HECT and RLD domain containing E3 ubiquitin protein ligase family member 6 | **0,919** | 0,295402 | 3,988 | 2,171 |
| RAB11B | RAB11B, member RAS oncogene family | **0,919** | 0,005065 | 70,567 | 63,084 |
| GCNT1 | glucosaminyl (N-acetyl) transferase 1, core 2 | **0,919** | 0,005107 | 39,936 | 25,923 |
| SLC16A7 | solute carrier family 16 member 7 | **0,919** | 0,029708 | 107,623 | 100,436 |
| NEK6 | NIMA related kinase 6 | **0,919** | 0,057102 | 14,900 | 9,450 |
| DNAJC17 | DnaJ heat shock protein family (Hsp40) member C17 | **0,919** | 0,073809 | 6,536 | 7,662 |
| CLDN5 | claudin 5 | **0,919** | 0,422641 | 0,554 | 0,064 |
| BCL3 | B-cell CLL/lymphoma 3 | **0,918** | 0,00267 | 50,460 | 44,823 |
| MRPS7 | mitochondrial ribosomal protein S7 | **0,918** | 0,006808 | 15,786 | 16,793 |

| | | | | | |
|---|---|---|---|---|---|
| DNASE2 | deoxyribonuclease 2, lysosomal | **0,918** | 0,019896 | 39,382 | 19,857 |
| PCNX3 | pecanex homolog 3 (Drosophila) | **0,918** | 0,003802 | 43,260 | 43,354 |
| SNRPE | small nuclear ribonucleoprotein polypeptide E | **0,918** | 0,061204 | 10,801 | 11,365 |
| WDR18 | WD repeat domain 18 | **0,918** | 0,039661 | 8,752 | 23,305 |
| URB1 | URB1 ribosome biogenesis 1 homolog (S. cerevisiae) | **0,918** | 0,00855 | 32,625 | 40,800 |
| AP1M1 | adaptor related protein complex 1 mu 1 subunit | **0,918** | 0,004461 | 51,679 | 44,376 |
| SHC1 | SHC adaptor protein 1 | **0,918** | 0,000611 | 117,704 | 83,643 |
| RAI1 | retinoic acid induced 1 | **0,918** | 0,012411 | 26,532 | 22,156 |
| JMJD8 | jumonji domain containing 8 | **0,918** | 0,006009 | 51,347 | 40,609 |
| CP | ceruloplasmin | **0,918** | 0,020055 | 186,000 | 206,235 |
| C7orf49 | chromosome 7 open reading frame 49 | **0,918** | 0,003683 | 22,821 | 21,007 |
| DPH1 | diphthamide biosynthesis 1 | **0,918** | 0,039378 | 8,696 | 10,599 |
| CHMP1A | charged multivesicular body protein 1A | **0,918** | 0,00536 | 54,227 | 45,908 |
| PDCL3 | phosducin like 3 | **0,918** | 0,068649 | 8,475 | 8,620 |
| AK1 | adenylate kinase 1 | **0,918** | 0,033108 | 13,626 | 9,386 |
| TRMT112 | tRNA methyltransferase 11-2 homolog (S. cerevisiae) | **0,918** | 0,028013 | 51,790 | 45,206 |
| PCDHB14 | protocadherin beta 14 | **0,918** | 0,253864 | 3,711 | 4,023 |
| FECH | ferrochelatase | **0,918** | 0,076244 | 13,404 | 8,811 |
| ROPN1L | rhophilin associated tail protein 1 like | **0,918** | 0,581998 | 0,665 | 0,638 |
| CLUH | clustered mitochondria homolog | **0,918** | 0,002978 | 36,059 | 36,267 |
| NAA10 | N(alpha)-acetyltransferase 10, NatA catalytic subunit | **0,918** | 0,054308 | 11,853 | 12,834 |
| OTUB1 | OTU deubiquitinase, ubiquitin aldehyde binding 1 | **0,918** | 0,005812 | 30,465 | 23,880 |
| FANCL | Fanconi anemia complementation group L | **0,918** | 0,042658 | 11,133 | 13,472 |
| TMEM69 | transmembrane protein 69 | **0,918** | 0,011008 | 18,168 | 19,857 |
| RNFT1 | ring finger protein, transmembrane 1 | **0,918** | 0,130457 | 7,367 | 6,385 |
| TTC30B | tetratricopeptide repeat domain 30B | **0,918** | 0,218245 | 3,600 | 2,937 |
| LINC01138 | long intergenic non-protein coding RNA 1138 | **0,918** | 0,141208 | 4,154 | 3,703 |
| MLH1 | mutL homolog 1 | **0,918** | 0,001141 | 39,105 | 34,926 |
| SLC39A3 | solute carrier family 39 member 3 | **0,918** | 0,029051 | 15,675 | 14,430 |
| THUMPD2 | THUMP domain containing 2 | **0,918** | 0,194049 | 4,154 | 3,512 |
| PSMD4 | proteasome 26S subunit, non-ATPase 4 | **0,918** | 0,004129 | 79,429 | 69,086 |
| EI24 | EI24, autophagy associated transmembrane protein | **0,918** | 0,001909 | 43,592 | 38,118 |
| RPL13P5 | ribosomal protein L13 pseudogene 5 | **0,918** | 0,305575 | 2,326 | 1,532 |

| | | | | | |
|---|---|---|---|---|---|
| MON1A | MON1 homolog A, secretory trafficking associated | **0,918** | 0,120192 | 9,693 | 8,109 |
| ADAMTS9 | ADAM metallopeptidase with thrombospondin type 1 motif 9 | **0,918** | 0,021142 | 71,176 | 41,247 |
| ZNF513 | zinc finger protein 513 | **0,918** | 0,053666 | 9,527 | 9,577 |
| TRAM1 | translocation associated membrane protein 1 | **0,918** | 0,003727 | 218,348 | 147,685 |
| CASP9 | caspase 9 | **0,918** | 0,038838 | 10,303 | 8,747 |
| SRSF2 | serine and arginine rich splicing factor 2 | **0,917** | 0,000536 | 56,941 | 48,973 |
| CDK2AP2 | cyclin dependent kinase 2 associated protein 2 | **0,917** | 0,011461 | 40,823 | 29,562 |
| MLLT1 | MLLT1, super elongation complex subunit | **0,917** | 0,000959 | 82,310 | 64,935 |
| HDGFP1 | heparin binding growth factor pseudogene 1 | **0,917** | 0,534384 | 1,496 | 0,064 |
| POLE3 | DNA polymerase epsilon 3, accessory subunit | **0,917** | 0,001262 | 41,432 | 40,992 |
| MYO15A | myosin XVA | **0,917** | 0,452906 | 0,886 | 0,575 |
| SMIM20 | small integral membrane protein 20 | **0,917** | 0,02369 | 11,023 | 13,855 |
| ECM2 | extracellular matrix protein 2 | **0,917** | 0,128083 | 5,151 | 2,873 |
| SSSCA1 | Sjogren syndrome/scleroderma autoantigen 1 | **0,917** | 0,038237 | 11,355 | 12,898 |
| NSUN4 | NOP2/Sun RNA methyltransferase family member 4 | **0,917** | 0,017819 | 17,060 | 15,962 |
| CXCL5 | C-X-C motif chemokine ligand 5 | **0,917** | 0,005764 | 210,095 | 299,137 |
| HHLA3 | HERV-H LTR-associating 3 | **0,917** | 0,078765 | 5,650 | 4,533 |
| GPKOW | G-patch domain and KOW motifs | **0,917** | 0,011297 | 19,830 | 19,921 |
| SLC35B2 | solute carrier family 35 member B2 | **0,917** | 0,001148 | 74,888 | 41,119 |
| ARRDC2 | arrestin domain containing 2 | **0,917** | 0,003724 | 64,086 | 69,149 |
| NAGS | N-acetylglutamate synthase | **0,917** | 0,57696 | 1,108 | 0,192 |
| HSD11B1L | hydroxysteroid 11-beta dehydrogenase 1 like | **0,917** | 0,321546 | 1,717 | 1,405 |
| MCM3 | minichromosome maintenance complex component 3 | **0,917** | 0,008023 | 25,867 | 23,688 |
| KIAA1549 | KIAA1549 | **0,917** | 0,091775 | 12,186 | 11,301 |
| C1R | complement C1r | **0,917** | 0,006479 | 137,811 | 69,916 |
| LCLAT1 | lysocardiolipin acyltransferase 1 | **0,917** | 0,128581 | 7,976 | 8,428 |
| CREB3 | cAMP responsive element binding protein 3 | **0,917** | 0,002173 | 55,999 | 45,653 |
| TTLL4 | tubulin tyrosine ligase like 4 | **0,917** | 0,002784 | 50,183 | 56,699 |
| ZNF587B | zinc finger protein 587B | **0,917** | 0,158225 | 3,323 | 3,512 |
| LRBA | LPS responsive beige-like anchor protein | **0,917** | 0,016331 | 15,952 | 18,644 |
| WTIP | WT1 interacting protein | **0,917** | 0,009498 | 21,658 | 15,899 |
| BEX2 | brain expressed X-linked 2 | **0,917** | 0,133238 | 5,927 | 5,746 |
| BABAM2 | BRISC and BRCA1 A complex member 2 | **0,917** | 0,02627 | 13,294 | 14,941 |

| | | | | | |
|---|---|---|---|---|---|
| PRELP | proline and arginine rich end leucine rich repeat protein | **0,917** | 0,001878 | 732,812 | 649,545 |
| ZNF480 | zinc finger protein 480 | **0,917** | 0,057258 | 11,632 | 6,449 |
| NUBP2 | nucleotide binding protein 2 | **0,917** | 0,004874 | 26,089 | 27,328 |
| NDFIP2 | Nedd4 family interacting protein 2 | **0,917** | 0,012215 | 39,659 | 38,118 |
| FOCAD | focadhesin | **0,917** | 0,012175 | 26,310 | 21,581 |
| IFNE | interferon epsilon | **0,917** | 0,635959 | 0,886 | 0,128 |
| DUSP1 | dual specificity phosphatase 1 | **0,917** | 0,005837 | 139,916 | 148,643 |
| RNF217 | ring finger protein 217 | **0,917** | 0,027247 | 23,430 | 15,260 |
| KCTD17 | potassium channel tetramerization domain containing 17 | **0,917** | 0,02792 | 12,407 | 10,663 |
| ZNF239 | zinc finger protein 239 | **0,916** | 0,164934 | 3,490 | 3,448 |
| HIRIP3 | HIRA interacting protein 3 | **0,916** | 0,10961 | 9,139 | 11,493 |
| TRABD2B | TraB domain containing 2B | **0,916** | 0,246671 | 3,268 | 1,852 |
| TMEM80 | transmembrane protein 80 | **0,916** | 0,115805 | 7,699 | 6,449 |
| MKS1 | Meckel syndrome, type 1 | **0,916** | 0,115434 | 6,259 | 4,725 |
| IRF3 | interferon regulatory factor 3 | **0,916** | 0,016979 | 16,783 | 15,452 |
| PLPP5 | phospholipid phosphatase 5 | **0,916** | 0,004005 | 61,151 | 61,168 |
| ESYT1 | extended synaptotagmin 1 | **0,916** | 0,00724 | 52,787 | 33,904 |
| SNAPC2 | small nuclear RNA activating complex polypeptide 2 | **0,916** | 0,025249 | 17,282 | 13,600 |
| SERP1 | stress associated endoplasmic reticulum protein 1 | **0,916** | 0,003656 | 146,784 | 137,213 |
| SLC24A3 | solute carrier family 24 member 3 | **0,916** | 0,578237 | 0,443 | 0,447 |
| UBB | ubiquitin B | **0,916** | 0,006219 | 320,044 | 275,001 |
| CXCR3 | C-X-C motif chemokine receptor 3 | **0,916** | 0,596562 | 0,609 | 0,702 |
| SNX12 | sorting nexin 12 | **0,916** | 0,00357 | 36,059 | 27,136 |
| COPG2 | coatomer protein complex subunit gamma 2 | **0,916** | 0,009512 | 19,387 | 22,028 |
| WDR13 | WD repeat domain 13 | **0,916** | 0,002052 | 55,501 | 42,524 |
| NUDT2 | nudix hydrolase 2 | **0,916** | 0,065247 | 8,696 | 9,897 |
| EVL | Enah/Vasp-like | **0,916** | 0,165235 | 4,708 | 4,023 |
| EIF5A | eukaryotic translation initiation factor 5A | **0,916** | 0,001191 | 218,791 | 179,227 |
| ZNF524 | zinc finger protein 524 | **0,916** | 0,039982 | 10,524 | 10,918 |
| PPP1R16A | protein phosphatase 1 regulatory subunit 16A | **0,916** | 0,001709 | 23,541 | 20,304 |
| SEC22A | SEC22 homolog A, vesicle trafficking protein | **0,916** | 0,066766 | 13,515 | 11,174 |
| HDAC6 | histone deacetylase 6 | **0,916** | 0,003969 | 34,176 | 23,752 |
| BRWD1-AS2 | BRWD1 antisense RNA 2 | **0,916** | 0,708581 | 0,332 | 0,192 |

| | | | | | |
|---|---|---|---|---|---|
| GSK3A | glycogen synthase kinase 3 alpha | **0,916** | 0,001129 | 41,709 | 36,267 |
| SREK1IP1 | SREK1 interacting protein 1 | **0,916** | 0,006352 | 26,200 | 23,561 |
| UCKL1 | uridine-cytidine kinase 1 like 1 | **0,916** | 0,007379 | 15,232 | 13,345 |
| POLR2H | RNA polymerase II subunit H | **0,916** | 0,022308 | 21,214 | 19,410 |
| HPRT1 | hypoxanthine phosphoribosyltransferase 1 | **0,916** | 0,075284 | 11,632 | 11,493 |
| TMTC4 | transmembrane and tetratricopeptide repeat containing 4 | **0,916** | 0,307564 | 3,102 | 3,129 |
| ZNF579 | zinc finger protein 579 | **0,916** | 0,005599 | 21,270 | 22,347 |
| DCUN1D5 | defective in cullin neddylation 1 domain containing 5 | **0,916** | 0,019501 | 18,390 | 15,643 |
| PPP6R2 | protein phosphatase 6 regulatory subunit 2 | **0,916** | 0,004801 | 37,388 | 35,117 |
| TIMM50 | translocase of inner mitochondrial membrane 50 | **0,916** | 0,001469 | 25,479 | 30,073 |
| RFT1 | RFT1 homolog | **0,916** | 0,008124 | 14,734 | 13,408 |
| HSPA5 | heat shock protein family A (Hsp70) member 5 | **0,916** | 0,045983 | 1365,589 | 1203,187 |
| DHDDS | dehydrodolichyl diphosphate synthase subunit | **0,916** | 0,008513 | 30,077 | 24,518 |
| WDR54 | WD repeat domain 54 | **0,916** | 0,040749 | 8,641 | 8,045 |
| PSMC3 | proteasome 26S subunit, ATPase 3 | **0,916** | 0,00398 | 110,559 | 93,221 |
| YBX3 | Y-box binding protein 3 | **0,916** | 0,004522 | 189,490 | 174,949 |
| KIAA0319L | KIAA0319 like | **0,916** | 0,001567 | 46,860 | 38,246 |
| PLK1 | polo like kinase 1 | **0,916** | 0,469505 | 3,545 | 0,638 |
| CSTF2 | cleavage stimulation factor subunit 2 | **0,916** | 0,010536 | 13,681 | 13,089 |
| GRIN2D | glutamate ionotropic receptor NMDA type subunit 2D | **0,916** | 0,406341 | 0,997 | 0,894 |
| TTF2 | transcription termination factor 2 | **0,916** | 0,142751 | 4,487 | 3,320 |
| USO1 | USO1 vesicle transport factor | **0,916** | 0,002911 | 130,222 | 101,904 |
| LINC00649 | long intergenic non-protein coding RNA 649 | **0,916** | 0,328517 | 1,385 | 1,469 |
| ZBTB14 | zinc finger and BTB domain containing 14 | **0,916** | 0,034524 | 9,638 | 9,131 |
| POP7 | POP7 homolog, ribonuclease P/MRP subunit | **0,916** | 0,008026 | 15,675 | 16,154 |
| NRXN2 | neurexin 2 | **0,916** | 0,655566 | 0,554 | 0,447 |
| HSPA4 | heat shock protein family A (Hsp70) member 4 | **0,916** | 0,001331 | 93,942 | 72,087 |
| ZNF77 | zinc finger protein 77 | **0,916** | 0,401236 | 1,772 | 1,915 |
| MNS1 | meiosis specific nuclear structural 1 | **0,916** | 0,430064 | 1,329 | 1,532 |
| SLIRP | SRA stem-loop interacting RNA binding protein | **0,916** | 0,028581 | 15,731 | 15,196 |
| SH3GL1 | SH3 domain containing GRB2 like 1, endophilin A2 | **0,916** | 0,00228 | 78,709 | 74,449 |
| CCL20 | C-C motif chemokine ligand 20 | **0,916** | 0,002548 | 4622,198 | 6194,460 |
| ZRANB3 | zinc finger RANBP2-type containing 3 | **0,916** | 0,221183 | 2,714 | 3,320 |

| | | | | | |
|---|---|---|---|---|---|
| DVL1 | dishevelled segment polarity protein 1 | **0,915** | 0,002936 | 40,047 | 35,309 |
| MTPAP | mitochondrial poly(A) polymerase | **0,915** | 0,01357 | 15,177 | 17,495 |
| ENTPD4 | ectonucleoside triphosphate diphosphohydrolase 4 | **0,915** | 0,001815 | 52,178 | 39,970 |
| RHBDF1 | rhomboid 5 homolog 1 | **0,915** | 0,002646 | 38,164 | 30,329 |
| SLC17A5 | solute carrier family 17 member 5 | **0,915** | 0,003801 | 30,852 | 19,730 |
| TMEM59L | transmembrane protein 59 like | **0,915** | 0,405266 | 1,440 | 0,511 |
| CMTM7 | CKLF like MARVEL transmembrane domain containing 7 | **0,915** | 0,027991 | 20,605 | 18,197 |
| KIZ | kizuna centrosomal protein | **0,915** | 0,044942 | 9,859 | 6,130 |
| RPUSD3 | RNA pseudouridylate synthase domain containing 3 | **0,915** | 0,028433 | 13,737 | 13,281 |
| COX8A | cytochrome c oxidase subunit 8A | **0,915** | 0,001583 | 43,149 | 33,649 |
| C18orf21 | chromosome 18 open reading frame 21 | **0,915** | 0,121422 | 6,758 | 5,810 |
| CTSC | cathepsin C | **0,915** | 0,023742 | 19,387 | 13,153 |
| PDLIM2 | PDZ and LIM domain 2 | **0,915** | 0,08401 | 7,644 | 5,746 |
| URM1 | ubiquitin related modifier 1 | **0,915** | 0,009415 | 34,231 | 31,031 |
| ZNF784 | zinc finger protein 784 | **0,915** | 0,132537 | 6,536 | 5,555 |
| CD2BP2 | CD2 cytoplasmic tail binding protein 2 | **0,915** | 0,005952 | 51,901 | 45,908 |
| TSSK6 | testis specific serine kinase 6 | **0,915** | 0,302637 | 1,052 | 1,341 |
| SLC7A7 | solute carrier family 7 member 7 | **0,915** | 0,060859 | 11,743 | 11,429 |
| CES2 | carboxylesterase 2 | **0,915** | 0,000969 | 43,924 | 34,670 |
| MIEF1 | mitochondrial elongation factor 1 | **0,915** | 0,004353 | 37,333 | 27,008 |
| ZNF628 | zinc finger protein 628 | **0,915** | 0,109086 | 5,539 | 4,342 |
| MILR1 | mast cell immunoglobulin like receptor 1 | **0,915** | 0,137869 | 4,154 | 2,235 |
| TMEM217 | transmembrane protein 217 | **0,915** | 0,233423 | 1,994 | 2,107 |
| ZKSCAN3 | zinc finger with KRAB and SCAN domains 3 | **0,915** | 0,208719 | 2,714 | 2,618 |
| USP16 | ubiquitin specific peptidase 16 | **0,915** | 0,009617 | 38,773 | 34,160 |
| RSG1 | REM2 and RAB like small GTPase 1 | **0,915** | 0,184505 | 3,379 | 4,533 |
| AIP | aryl hydrocarbon receptor interacting protein | **0,915** | 0,019902 | 44,091 | 38,693 |
| TNIK | TRAF2 and NCK interacting kinase | **0,915** | 0,027728 | 7,699 | 8,875 |
| ANXA6 | annexin A6 | **0,915** | 0,000754 | 82,587 | 53,123 |
| GPX1 | glutathione peroxidase 1 | **0,915** | 0,005561 | 66,247 | 50,250 |
| SLC30A5 | solute carrier family 30 member 5 | **0,915** | 0,003287 | 67,576 | 60,338 |
| XPNPEP1 | X-prolyl aminopeptidase 1 | **0,915** | 0,001662 | 27,972 | 21,070 |
| VPS33B | VPS33B, late endosome and lysosome associated | **0,914** | 0,313273 | 1,717 | 1,596 |

| | | | | | |
|---|---|---|---|---|---|
| ECD | ecdysoneless cell cycle regulator | **0,914** | 0,013788 | 23,375 | 17,814 |
| TPCN2 | two pore segment channel 2 | **0,914** | 0,021233 | 15,897 | 10,216 |
| RCC1L | RCC1 like | **0,914** | 0,006847 | 34,397 | 34,990 |
| ATXN10 | ataxin 10 | **0,914** | 0,002333 | 49,353 | 37,735 |
| ATXN7L3 | ataxin 7 like 3 | **0,914** | 0,001103 | 39,992 | 30,712 |
| RRBP1 | ribosome binding protein 1 | **0,914** | 0,005342 | 257,398 | 234,201 |
| EIF3M | eukaryotic translation initiation factor 3 subunit M | **0,914** | 0,002144 | 63,256 | 61,168 |
| ISCA2 | iron-sulfur cluster assembly 2 | **0,914** | 0,136656 | 6,481 | 3,959 |
| SAT1 | spermidine/spermine N1-acetyltransferase 1 | **0,914** | 0,004739 | 1527,439 | 1340,656 |
| UBL4A | ubiquitin like 4A | **0,914** | 0,020821 | 20,217 | 11,876 |
| ATP5H | ATP synthase, H+ transporting, mitochondrial Fo complex subunit D | **0,914** | 0,009624 | 44,423 | 37,288 |
| SELENOM | selenoprotein M | **0,914** | 0,008064 | 128,007 | 107,395 |
| UQCRH | ubiquinol-cytochrome c reductase hinge protein | **0,914** | 0,008131 | 39,881 | 32,053 |
| MESDC1 | mesoderm development candidate 1 | **0,914** | 0,001334 | 57,717 | 49,931 |
| ZNF787 | zinc finger protein 787 | **0,914** | 0,001884 | 27,252 | 23,752 |
| SPPL2B | signal peptide peptidase like 2B | **0,914** | 0,017531 | 19,553 | 20,687 |
| WIPI1 | WD repeat domain, phosphoinositide interacting 1 | **0,914** | 0,003644 | 39,881 | 23,114 |
| MESDC2 | mesoderm development candidate 2 | **0,914** | 0,000952 | 79,596 | 75,343 |
| ELMO3 | engulfment and cell motility 3 | **0,914** | 0,415222 | 0,720 | 0,894 |
| PCDHGA7 | protocadherin gamma subfamily A, 7 | **0,914** | 0,410711 | 1,329 | 1,149 |
| ATP5J | ATP synthase, H+ transporting, mitochondrial Fo complex subunit F6 | **0,914** | 0,002804 | 40,712 | 35,373 |
| DNAJC11 | DnaJ heat shock protein family (Hsp40) member C11 | **0,914** | 0,00068 | 27,252 | 25,476 |
| SYN3 | synapsin III | **0,914** | 0,45353 | 0,554 | 0,958 |
| GAS5 | growth arrest specific 5 (non-protein coding) | **0,914** | 0,00071 | 59,212 | 64,041 |
| NUDT3 | nudix hydrolase 3 | **0,914** | 0,003188 | 30,797 | 28,158 |
| UAP1 | UDP-N-acetylglucosamine pyrophosphorylase 1 | **0,914** | 0,000316 | 261,829 | 273,916 |
| STK25 | serine/threonine kinase 25 | **0,914** | 0,004593 | 40,269 | 31,670 |
| MEA1 | male-enhanced antigen 1 | **0,914** | 0,002351 | 40,933 | 37,799 |
| TSR1 | TSR1, ribosome maturation factor | **0,914** | 0,002103 | 44,368 | 44,440 |
| CAPNS1 | calpain small subunit 1 | **0,914** | 0,000956 | 189,379 | 142,704 |
| SIX2 | SIX homeobox 2 | **0,914** | 0,071307 | 9,638 | 7,215 |
| GLMN | glomulin, FKBP associated protein | **0,914** | 0,226894 | 3,157 | 3,320 |
| EIF2B4 | eukaryotic translation initiation factor 2B subunit delta | **0,914** | 0,017735 | 19,940 | 18,963 |

| | | | | | |
|---|---|---|---|---|---|
| CORIN | corin, serine peptidase | **0,914** | 0,637714 | 0,332 | 0,383 |
| FASTKD3 | FAST kinase domains 3 | **0,914** | 0,100531 | 5,151 | 5,108 |
| APIP | APAF1 interacting protein | **0,914** | 0,038159 | 10,136 | 5,938 |
| LBP | lipopolysaccharide binding protein | **0,914** | 0,086936 | 9,472 | 1,596 |
| MPDU1 | mannose-P-dolichol utilization defect 1 | **0,914** | 0,001311 | 33,234 | 28,349 |
| PYCR2 | pyrroline-5-carboxylate reductase 2 | **0,914** | 0,004547 | 36,391 | 38,310 |
| RCC1 | regulator of chromosome condensation 1 | **0,914** | 0,005362 | 22,488 | 21,454 |
| RWDD1 | RWD domain containing 1 | **0,914** | 0,032637 | 18,833 | 17,112 |
| ZNF398 | zinc finger protein 398 | **0,914** | 0,016131 | 14,014 | 13,536 |
| DGUOK | deoxyguanosine kinase | **0,914** | 0,003142 | 37,001 | 37,863 |
| RACGAP1 | Rac GTPase activating protein 1 | **0,913** | 0,057914 | 15,842 | 7,470 |
| MYO10 | myosin X | **0,913** | 0,004341 | 244,326 | 191,741 |
| FKBP8 | FK506 binding protein 8 | **0,913** | 0,01399 | 95,326 | 67,170 |
| DBNL | drebrin like | **0,913** | 0,001665 | 53,507 | 40,225 |
| ZFP64 | ZFP64 zinc finger protein | **0,913** | 0,010752 | 11,909 | 9,769 |
| SLC25A3 | solute carrier family 25 member 3 | **0,913** | 0,002385 | 169,549 | 159,944 |
| NFE2L2 | nuclear factor, erythroid 2 like 2 | **0,913** | 0,000208 | 168,054 | 150,558 |
| BICC1 | BicC family RNA binding protein 1 | **0,913** | 0,126458 | 6,868 | 7,215 |
| DDX54 | DEAD-box helicase 54 | **0,913** | 0,007365 | 44,201 | 39,587 |
| WDR61 | WD repeat domain 61 | **0,913** | 0,013705 | 26,255 | 24,838 |
| EML3 | echinoderm microtubule associated protein like 3 | **0,913** | 0,004904 | 24,649 | 19,921 |
| ADCK2 | aarF domain containing kinase 2 | **0,913** | 0,267701 | 8,475 | 5,491 |
| TBL3 | transducin beta like 3 | **0,913** | 0,027746 | 19,276 | 17,686 |
| BABAM1 | BRISC and BRCA1 A complex member 1 | **0,913** | 0,032783 | 9,804 | 8,428 |
| MXRA8 | matrix remodeling associated 8 | **0,913** | 0,158071 | 9,195 | 6,960 |
| CST2 | cystatin SA | **0,913** | 0,167405 | 0,388 | 1,532 |
| NIPSNAP2 | nipsnap homolog 2 | **0,913** | 0,036772 | 11,466 | 6,960 |
| NR1H2 | nuclear receptor subfamily 1 group H member 2 | **0,913** | 0,000964 | 87,184 | 76,556 |
| SERPINA3 | serpin family A member 3 | **0,913** | 0,312651 | 2,936 | 2,362 |
| TP53BP1 | tumor protein p53 binding protein 1 | **0,913** | 0,011291 | 56,941 | 51,399 |
| RBM28 | RNA binding motif protein 28 | **0,913** | 0,002869 | 21,990 | 23,433 |
| KBTBD6 | kelch repeat and BTB domain containing 6 | **0,913** | 0,003377 | 18,999 | 19,091 |
| USP35 | ubiquitin specific peptidase 35 | **0,913** | 0,091938 | 6,259 | 4,533 |

| | | | | | |
|---|---|---|---|---|---|
| INTS9 | integrator complex subunit 9 | **0,913** | 0,050881 | 7,644 | 9,897 |
| FCRLA | Fc receptor like A | **0,912** | 0,238074 | 2,714 | 3,767 |
| TRMT11 | tRNA methyltransferase 11 homolog | **0,912** | 0,050656 | 9,472 | 8,364 |
| FUBP1 | far upstream element binding protein 1 | **0,912** | 0,002947 | 71,896 | 63,148 |
| DLG4 | discs large MAGUK scaffold protein 4 | **0,912** | 0,165897 | 5,539 | 5,300 |
| DNAJC30 | DnaJ heat shock protein family (Hsp40) member C30 | **0,912** | 0,013488 | 14,734 | 15,579 |
| DPH5 | diphthamide biosynthesis 5 | **0,912** | 0,036785 | 9,139 | 7,279 |
| SPCS3 | signal peptidase complex subunit 3 | **0,912** | 0,002276 | 91,782 | 78,025 |
| GOSR2 | golgi SNAP receptor complex member 2 | **0,912** | 0,002842 | 34,453 | 32,819 |
| CCT5 | chaperonin containing TCP1 subunit 5 | **0,912** | 0,000469 | 120,806 | 110,205 |
| PRKD2 | protein kinase D2 | **0,912** | 0,030157 | 11,078 | 9,897 |
| PLEK2 | pleckstrin 2 | **0,912** | 0,554916 | 0,609 | 0,958 |
| PDE1A | phosphodiesterase 1A | **0,912** | 0,233713 | 1,994 | 5,810 |
| DNALI1 | dynein axonemal light intermediate chain 1 | **0,912** | 0,135299 | 4,597 | 3,703 |
| CENPW | centromere protein W | **0,912** | 0,280255 | 2,991 | 1,213 |
| ZC3HC1 | zinc finger C3HC-type containing 1 | **0,912** | 0,039976 | 10,746 | 12,515 |
| C14orf166 | chromosome 14 open reading frame 166 | **0,912** | 0,002293 | 46,251 | 44,503 |
| APBB3 | amyloid beta precursor protein binding family B member 3 | **0,912** | 0,027376 | 6,702 | 7,470 |
| TMEM55A | transmembrane protein 55A | **0,912** | 0,056285 | 13,404 | 7,981 |
| PDIA3P1 | protein disulfide isomerase family A member 3 pseudogene 1 | **0,912** | 0,075036 | 6,259 | 5,938 |
| C19orf73 | chromosome 19 open reading frame 73 | **0,912** | 0,634042 | 0,443 | 0,511 |
| ADH5 | alcohol dehydrogenase 5 (class III), chi polypeptide | **0,912** | 0,002683 | 53,618 | 35,628 |
| BMS1 | BMS1, ribosome biogenesis factor | **0,912** | 0,005927 | 37,555 | 35,309 |
| ZNF182 | zinc finger protein 182 | **0,912** | 0,166555 | 4,154 | 5,300 |
| ZNF526 | zinc finger protein 526 | **0,912** | 0,026266 | 13,958 | 11,429 |
| CKLF | chemokine like factor | **0,912** | 0,218208 | 3,046 | 1,532 |
| MCAM | melanoma cell adhesion molecule | **0,912** | 0,530701 | 0,720 | 0,702 |
| C11orf80 | chromosome 11 open reading frame 80 | **0,912** | 0,382114 | 1,883 | 0,766 |
| MBOAT2 | membrane bound O-acyltransferase domain containing 2 | **0,912** | 0,001874 | 22,932 | 20,368 |
| CARMIL1 | capping protein regulator and myosin 1 linker 1 | **0,912** | 0,056052 | 8,696 | 9,258 |
| H2AFY | H2A histone family member Y | **0,912** | 0,001719 | 40,933 | 37,991 |
| RHOT2 | ras homolog family member T2 | **0,912** | 0,002981 | 28,914 | 24,008 |
| ANAPC4 | anaphase promoting complex subunit 4 | **0,912** | 0,037459 | 11,300 | 8,556 |

| | | | | | |
|---|---|---|---|---|---|
| NDUFB3 | NADH:ubiquinone oxidoreductase subunit B3 | **0,912** | 0,020415 | 18,390 | 13,025 |
| TEAD2 | TEA domain transcription factor 2 | **0,912** | 0,128075 | 5,041 | 2,746 |
| FOXP2 | forkhead box P2 | **0,912** | 0,188269 | 4,210 | 2,554 |
| MYOZ2 | myozenin 2 | **0,912** | 0,50698 | 0,277 | 1,085 |
| RTN4 | reticulon 4 | **0,912** | 4,40E-05 | 665,901 | 499,115 |
| MX1 | MX dynamin like GTPase 1 | **0,912** | 0,151571 | 3,102 | 11,557 |
| LANCL3 | LanC like 3 | **0,911** | 0,675113 | 0,277 | 0,128 |
| OSCP1 | organic solute carrier partner 1 | **0,911** | 0,058605 | 6,536 | 4,980 |
| RPL29 | ribosomal protein L29 | **0,911** | 0,001618 | 105,961 | 86,644 |
| SSNA1 | SS nuclear autoantigen 1 | **0,911** | 0,024928 | 24,538 | 19,730 |
| TAF13 | TATA-box binding protein associated factor 13 | **0,911** | 0,000984 | 32,791 | 29,946 |
| PPA2 | pyrophosphatase (inorganic) 2 | **0,911** | 0,031089 | 12,629 | 12,068 |
| APEH | acylaminoacyl-peptide hydrolase | **0,911** | 0,009269 | 31,517 | 25,285 |
| DOCK3 | dedicator of cytokinesis 3 | **0,911** | 0,466797 | 0,554 | 0,702 |
| RPL41 | ribosomal protein L41 | **0,911** | 0,025462 | 42,983 | 48,462 |
| P3H3 | prolyl 3-hydroxylase 3 | **0,911** | 0,031172 | 31,462 | 13,281 |
| AMER1 | APC membrane recruitment protein 1 | **0,911** | 0,1832 | 4,930 | 4,406 |
| ARAF | A-Raf proto-oncogene, serine/threonine kinase | **0,911** | 0,000666 | 34,896 | 30,712 |
| EPRS | glutamyl-prolyl-tRNA synthetase | **0,911** | 0,000321 | 199,626 | 171,565 |
| C1orf112 | chromosome 1 open reading frame 112 | **0,911** | 0,182427 | 3,933 | 3,767 |
| TAPT1-AS1 | TAPT1 antisense RNA 1 (head to head) | **0,911** | 0,451778 | 1,440 | 1,149 |
| LINC02361 | long intergenic non-protein coding RNA 2361 | **0,911** | 0,406599 | 0,886 | 1,085 |
| VSIR | V-set immunoregulatory receptor | **0,911** | 0,00148 | 85,578 | 103,309 |
| IGFBP6 | insulin like growth factor binding protein 6 | **0,911** | 0,007494 | 219,234 | 207,831 |
| PCSK7 | proprotein convertase subtilisin/kexin type 7 | **0,911** | 0,009941 | 14,069 | 16,154 |
| SFXN1 | sideroflexin 1 | **0,911** | 7,00E-04 | 24,206 | 20,304 |
| ISG20L2 | interferon stimulated exonuclease gene 20 like 2 | **0,911** | 0,001487 | 19,553 | 17,750 |
| SMARCA4 | SWI/SNF related, matrix associated, actin dependent regulator of chromatin, subfamily a, | **0,911** | 0,00271 | 72,118 | 63,403 |
| NLRP1 | NLR family pyrin domain containing 1 | **0,911** | 0,026539 | 18,943 | 16,665 |
| MCM3AP-AS1 | MCM3AP antisense RNA 1 | **0,911** | 0,553129 | 0,609 | 0,766 |
| HSPA14 | heat shock protein family A (Hsp70) member 14 | **0,911** | 0,163539 | 3,490 | 3,065 |
| MARS | methionyl-tRNA synthetase | **0,911** | 0,00028 | 188,050 | 188,229 |
| LSM10 | LSM10, U7 small nuclear RNA associated | **0,911** | 0,016317 | 13,017 | 11,940 |

| | | | | | |
|---|---|---|---|---|---|
| FANCG | Fanconi anemia complementation group G | **0,911** | 0,079666 | 6,425 | 5,236 |
| SAMM50 | SAMM50 sorting and assembly machinery component | **0,911** | 0,013839 | 15,842 | 10,599 |
| HIP1R | huntingtin interacting protein 1 related | **0,911** | 0,006649 | 15,177 | 13,408 |
| FAM50B | family with sequence similarity 50 member B | **0,911** | 0,059109 | 7,755 | 10,088 |
| POC1A | POC1 centriolar protein A | **0,911** | 0,235478 | 3,268 | 2,362 |
| GSTM2 | glutathione S-transferase mu 2 | **0,911** | 0,506649 | 1,219 | 0,447 |
| BZW1 | basic leucine zipper and W2 domains 1 | **0,911** | 0,000143 | 400,139 | 328,252 |
| ZBTB8OS | zinc finger and BTB domain containing 8 opposite strand | **0,911** | 0,01213 | 11,964 | 11,812 |
| SMURF2 | SMAD specific E3 ubiquitin protein ligase 2 | **0,910** | 0,021842 | 26,200 | 24,710 |
| B4GALT1 | beta-1,4-galactosyltransferase 1 | **0,910** | 0,000547 | 149,221 | 126,614 |
| TOP1MT | topoisomerase (DNA) I, mitochondrial | **0,910** | 0,123368 | 4,874 | 6,321 |
| VASP | vasodilator-stimulated phosphoprotein | **0,910** | 0,002135 | 38,275 | 38,055 |
| OSGIN1 | oxidative stress induced growth inhibitor 1 | **0,910** | 0,045845 | 29,468 | 15,962 |
| SCG3 | secretogranin III | **0,910** | 0,633375 | 0,332 | 0,255 |
| LSR | lipolysis stimulated lipoprotein receptor | **0,910** | 0,370959 | 1,052 | 0,383 |
| RTL8A | retrotransposon Gag like 8A | **0,910** | 0,015459 | 17,005 | 12,068 |
| 6.syys | septin 6 | **0,910** | 0,052249 | 7,865 | 8,173 |
| PNPLA6 | patatin like phospholipase domain containing 6 | **0,910** | 0,002197 | 49,242 | 41,502 |
| SH3YL1 | SH3 and SYLF domain containing 1 | **0,910** | 0,031121 | 11,189 | 11,557 |
| CXCL8 | C-X-C motif chemokine ligand 8 | **0,910** | 0,000265 | 20074,281 | 22192,111 |
| FAM185A | family with sequence similarity 185 member A | **0,910** | 0,277769 | 2,105 | 1,405 |
| KCTD14 | potassium channel tetramerization domain containing 14 | **0,910** | 0,270013 | 3,434 | 1,788 |
| DNAJC18 | DnaJ heat shock protein family (Hsp40) member C18 | **0,910** | 0,099836 | 7,256 | 5,172 |
| PMM2 | phosphomannomutase 2 | **0,910** | 0,021923 | 10,967 | 9,961 |
| MRPS27 | mitochondrial ribosomal protein S27 | **0,910** | 0,000614 | 29,191 | 27,200 |
| OSGEP | O-sialoglycoprotein endopeptidase | **0,910** | 0,00879 | 12,463 | 13,153 |
| EARS2 | glutamyl-tRNA synthetase 2, mitochondrial | **0,910** | 0,036806 | 8,530 | 7,854 |
| NSUN2 | NOP2/Sun RNA methyltransferase family member 2 | **0,910** | 0,000742 | 49,076 | 45,014 |
| TEAD3 | TEA domain transcription factor 3 | **0,910** | 0,025675 | 10,635 | 5,938 |
| PRELID1 | PRELI domain containing 1 | **0,910** | 0,000951 | 59,434 | 52,357 |
| S100A2 | S100 calcium binding protein A2 | **0,910** | 0,015444 | 13,681 | 25,923 |
| HNRNPUL1 | heterogeneous nuclear ribonucleoprotein U like 1 | **0,910** | 0,000511 | 143,516 | 125,210 |
| LACTB2 | lactamase beta 2 | **0,910** | 0,05529 | 7,865 | 5,810 |

| | | | | | |
|---|---|---|---|---|---|
| ATAD3B | ATPase family, AAA domain containing 3B | **0,910** | 0,041723 | 12,518 | 12,962 |
| ZC3H12D | zinc finger CCCH-type containing 12D | **0,910** | 0,395084 | 0,554 | 0,638 |
| METTL25 | methyltransferase like 25 | **0,909** | 0,186318 | 2,603 | 2,299 |
| NAB1 | NGFI-A binding protein 1 | **0,909** | 0,004283 | 76,605 | 52,995 |
| CCDC142 | coiled-coil domain containing 142 | **0,909** | 0,103579 | 7,256 | 8,237 |
| IRGQ | immunity related GTPase Q | **0,909** | 0,052334 | 26,753 | 21,198 |
| TRADD | TNFRSF1A associated via death domain | **0,909** | 0,047421 | 13,404 | 11,493 |
| C19orf53 | chromosome 19 open reading frame 53 | **0,909** | 0,008179 | 32,348 | 35,117 |
| COMT | catechol-O-methyltransferase | **0,909** | 0,00518 | 67,299 | 56,252 |
| CDK11B | cyclin dependent kinase 11B | **0,909** | 0,012461 | 16,728 | 16,218 |
| ELOB | elongin B | **0,909** | 0,019273 | 56,110 | 44,312 |
| PTCD1 | pentatricopeptide repeat domain 1 | **0,909** | 0,245402 | 3,545 | 4,533 |
| HVCN1 | hydrogen voltage gated channel 1 | **0,909** | 0,465276 | 0,665 | 0,638 |
| GLRX3 | glutaredoxin 3 | **0,909** | 0,005146 | 28,083 | 23,688 |
| ELP5 | elongator acetyltransferase complex subunit 5 | **0,909** | 0,000632 | 24,538 | 22,475 |
| COPS4 | COP9 signalosome subunit 4 | **0,909** | 0,003082 | 25,147 | 23,561 |
| BEND3 | BEN domain containing 3 | **0,909** | 0,187265 | 2,160 | 3,384 |
| C16orf45 | chromosome 16 open reading frame 45 | **0,909** | 0,570673 | 0,609 | 0,511 |
| RHBDD3 | rhomboid domain containing 3 | **0,909** | 0,016849 | 16,506 | 13,536 |
| CD320 | CD320 molecule | **0,909** | 0,048987 | 27,474 | 14,175 |
| RBM19 | RNA binding motif protein 19 | **0,909** | 0,005109 | 22,987 | 24,901 |
| SH3D19 | SH3 domain containing 19 | **0,909** | 0,003394 | 38,939 | 32,755 |
| FBN1 | fibrillin 1 | **0,909** | 0,032382 | 143,516 | 79,174 |
| NDUFS6 | NADH:ubiquinone oxidoreductase subunit S6 | **0,909** | 0,004242 | 26,033 | 24,965 |
| INTS10 | integrator complex subunit 10 | **0,909** | 0,004299 | 36,336 | 31,478 |
| PSME3 | proteasome activator subunit 3 | **0,909** | 0,00017 | 64,197 | 53,442 |
| CDC123 | cell division cycle 123 | **0,909** | 0,00098 | 32,569 | 27,200 |
| AGPAT2 | 1-acylglycerol-3-phosphate O-acyltransferase 2 | **0,909** | 0,034075 | 16,395 | 9,961 |
| BCAR1 | BCAR1, Cas family scaffolding protein | **0,909** | 0,02201 | 11,743 | 9,833 |
| PXDC1 | PX domain containing 1 | **0,909** | 0,000295 | 52,565 | 43,673 |
| BZW2 | basic leucine zipper and W2 domains 2 | **0,909** | 0,021458 | 17,836 | 13,281 |
| NGLY1 | N-glycanase 1 | **0,909** | 0,002754 | 25,923 | 19,410 |
| PRR12 | proline rich 12 | **0,909** | 0,01451 | 24,926 | 17,814 |

| | | | | | |
|---|---|---|---|---|---|
| EPN1 | epsin 1 | **0,909** | 0,001906 | 75,829 | 65,765 |
| STRN4 | striatin 4 | **0,909** | 0,000335 | 47,802 | 40,481 |
| IMMT | inner membrane mitochondrial protein | **0,909** | 0,001016 | 44,921 | 40,289 |
| DUS1L | dihydrouridine synthase 1 like | **0,908** | 0,013337 | 13,017 | 12,068 |
| CCDC58 | coiled-coil domain containing 58 | **0,908** | 0,05727 | 5,594 | 5,427 |
| GSTP1 | glutathione S-transferase pi 1 | **0,908** | 0,008356 | 130,665 | 99,350 |
| AL161911.1 | Putative UDP-GlcNAc:betaGal beta-1,3-N-acetylglucosaminyltransferase LOC100288842 | **0,908** | 0,289369 | 2,770 | 2,490 |
| C19orf60 | chromosome 19 open reading frame 60 | **0,908** | 0,022886 | 19,774 | 15,643 |
| IER3IP1 | immediate early response 3 interacting protein 1 | **0,908** | 0,005787 | 19,996 | 18,516 |
| RPSA | ribosomal protein SA | **0,908** | 0,002546 | 176,251 | 117,037 |
| PRDX4 | peroxiredoxin 4 | **0,908** | 0,000734 | 129,336 | 63,850 |
| SCG2 | secretogranin II | **0,908** | 0,580793 | 0,609 | 0,255 |
| SLC22A23 | solute carrier family 22 member 23 | **0,908** | 0,000973 | 60,708 | 36,714 |
| CXCL16 | C-X-C motif chemokine ligand 16 | **0,908** | 0,029604 | 9,306 | 9,514 |
| QARS | glutaminyl-tRNA synthetase | **0,908** | 0,002695 | 49,851 | 32,819 |
| CAD | carbamoyl-phosphate synthetase 2, aspartate transcarbamylase, and dihydroorotase | **0,908** | 0,004725 | 30,631 | 27,392 |
| EIF2B5 | eukaryotic translation initiation factor 2B subunit epsilon | **0,908** | 0,00239 | 29,689 | 30,073 |
| NAP1L4 | nucleosome assembly protein 1 like 4 | **0,908** | 0,510684 | 0,554 | 0,000 |
| LEPROTL1 | leptin receptor overlapping transcript-like 1 | **0,908** | 0,003662 | 15,509 | 13,472 |
| ANKHD1-EIF4EBI | ANKHD1-EIF4EBP3 readthrough | **0,908** | 0,493754 | 0,609 | 0,575 |
| SERF2 | small EDRK-rich factor 2 | **0,908** | 0,005868 | 305,144 | 192,444 |
| IGSF8 | immunoglobulin superfamily member 8 | **0,908** | 0,009063 | 30,908 | 15,260 |
| RBM15B | RNA binding motif protein 15B | **0,908** | 0,000722 | 58,880 | 39,906 |
| ARG2 | arginase 2 | **0,908** | 0,058117 | 4,542 | 5,491 |
| DPAGT1 | dolichyl-phosphate N-acetylglucosaminephosphotransferase 1 | **0,908** | 0,005586 | 15,177 | 14,941 |
| KAZALD1 | Kazal type serine peptidase inhibitor domain 1 | **0,908** | 0,065271 | 7,976 | 5,363 |
| MRPL21 | mitochondrial ribosomal protein L21 | **0,908** | 0,012602 | 13,515 | 13,472 |
| NANS | N-acetylneuraminate synthase | **0,908** | 0,006961 | 42,152 | 42,652 |
| WDR83OS | WD repeat domain 83 opposite strand | **0,908** | 0,002509 | 33,179 | 30,839 |
| GPX3 | glutathione peroxidase 3 | **0,908** | 0,001414 | 351,395 | 245,694 |
| BIN1 | bridging integrator 1 | **0,908** | 0,01342 | 19,165 | 18,580 |
| KRBOX4 | KRAB box domain containing 4 | **0,908** | 0,055945 | 6,924 | 8,045 |
| DUSP19 | dual specificity phosphatase 19 | **0,908** | 0,294836 | 1,662 | 1,915 |

| | | | | | |
|---|---|---|---|---|---|
| FAM111B | family with sequence similarity 111 member B | **0,908** | 0,349231 | 2,714 | 0,638 |
| LIG3 | DNA ligase 3 | **0,908** | 0,002458 | 18,500 | 20,943 |
| CYC1 | cytochrome c1 | **0,908** | 0,001907 | 58,049 | 54,272 |
| ARPC4 | actin related protein 2/3 complex subunit 4 | **0,908** | 0,015518 | 37,610 | 33,330 |
| LYPLAL1 | lysophospholipase like 1 | **0,908** | 0,020584 | 9,472 | 7,279 |
| GORASP2 | golgi reassembly stacking protein 2 | **0,908** | 0,000766 | 97,653 | 80,706 |
| BCAS4 | breast carcinoma amplified sequence 4 | **0,908** | 0,249329 | 2,880 | 1,852 |
| FZD10 | frizzled class receptor 10 | **0,908** | 0,109318 | 4,542 | 4,086 |
| DNAAF2 | dynein axonemal assembly factor 2 | **0,908** | 0,027622 | 6,868 | 6,257 |
| CERS5 | ceramide synthase 5 | **0,907** | 0,000188 | 69,348 | 37,097 |
| COX7A2 | cytochrome c oxidase subunit 7A2 | **0,907** | 0,000564 | 68,684 | 46,227 |
| PRPF18 | pre-mRNA processing factor 18 | **0,907** | 0,10645 | 4,819 | 4,086 |
| NEDD4 | neural precursor cell expressed, developmentally down-regulated 4, E3 ubiquitin protein li | **0,907** | 0,014638 | 25,313 | 25,093 |
| ABHD11 | abhydrolase domain containing 11 | **0,907** | 0,128148 | 4,597 | 5,300 |
| TBC1D22A | TBC1 domain family member 22A | **0,907** | 0,005091 | 20,439 | 15,260 |
| SLC25A43 | solute carrier family 25 member 43 | **0,907** | 0,064659 | 10,690 | 4,533 |
| JPT2 | Jupiter microtubule associated homolog 2 | **0,907** | 0,000365 | 36,225 | 34,479 |
| GRAMD1A | GRAM domain containing 1A | **0,907** | 0,011417 | 24,593 | 10,854 |
| SCOC-AS1 | SCOC antisense RNA 1 | **0,907** | 0,584885 | 0,166 | 0,511 |
| POLR1B | RNA polymerase I subunit B | **0,907** | 0,006659 | 15,509 | 17,239 |
| RAB15 | RAB15, member RAS oncogene family | **0,907** | 0,152509 | 3,490 | 2,299 |
| MGRN1 | mahogunin ring finger 1 | **0,907** | 0,00028 | 42,207 | 31,925 |
| PSMC5 | proteasome 26S subunit, ATPase 5 | **0,907** | 0,001959 | 97,154 | 84,026 |
| ZDHHC18 | zinc finger DHHC-type containing 18 | **0,907** | 0,003783 | 21,491 | 14,302 |
| ATP5C1 | ATP synthase, H+ transporting, mitochondrial F1 complex, gamma polypeptide 1 | **0,907** | 0,001025 | 35,339 | 30,329 |
| C11orf49 | chromosome 11 open reading frame 49 | **0,907** | 0,01301 | 11,853 | 7,854 |
| EFCAB7 | EF-hand calcium binding domain 7 | **0,907** | 0,210416 | 4,930 | 3,129 |
| FAM160B2 | family with sequence similarity 160 member B2 | **0,907** | 0,004143 | 33,179 | 27,455 |
| LYRM4 | LYR motif containing 4 | **0,907** | 0,001732 | 26,421 | 29,371 |
| NOLC1 | nucleolar and coiled-body phosphoprotein 1 | **0,907** | 0,000216 | 56,498 | 64,041 |
| GOT2 | glutamic-oxaloacetic transaminase 2 | **0,907** | 0,000559 | 55,335 | 38,438 |
| NXT1 | nuclear transport factor 2 like export factor 1 | **0,907** | 0,006277 | 17,337 | 17,942 |
| ARHGAP44 | Rho GTPase activating protein 44 | **0,907** | 0,212977 | 2,216 | 2,873 |

| | | | | | |
|---|---|---|---|---|---|
| SNX17 | sorting nexin 17 | **0,907** | 0,000378 | 78,322 | 72,278 |
| PRDX2 | peroxiredoxin 2 | **0,907** | 0,005702 | 28,637 | 19,857 |
| ARFRP1 | ADP ribosylation factor related protein 1 | **0,907** | 0,004232 | 29,689 | 31,542 |
| ZNF428 | zinc finger protein 428 | **0,907** | 0,039693 | 12,407 | 9,450 |
| CCT6A | chaperonin containing TCP1 subunit 6A | **0,907** | 0,000133 | 96,933 | 85,687 |
| CDK10 | cyclin dependent kinase 10 | **0,907** | 0,037324 | 21,436 | 25,348 |
| CTDSP1 | CTD small phosphatase 1 | **0,907** | 0,001084 | 86,907 | 76,492 |
| TNFRSF12A | TNF receptor superfamily member 12A | **0,907** | 0,001336 | 232,639 | 184,782 |
| SEC23B | Sec23 homolog B, coat complex II component | **0,907** | 0,002985 | 93,222 | 74,832 |
| SPTLC3 | serine palmitoyltransferase long chain base subunit 3 | **0,907** | 0,428185 | 1,329 | 0,638 |
| PNPT1 | polyribonucleotide nucleotidyltransferase 1 | **0,907** | 0,01014 | 13,127 | 18,197 |
| POGLUT1 | protein O-glucosyltransferase 1 | **0,907** | 0,009178 | 16,783 | 11,748 |
| PKMYT1 | protein kinase, membrane associated tyrosine/threonine 1 | **0,907** | 0,515278 | 2,548 | 0,192 |
| INTS1 | integrator complex subunit 1 | **0,907** | 0,003627 | 73,890 | 86,964 |
| FCHSD2 | FCH and double SH3 domains 2 | **0,907** | 0,014317 | 24,095 | 18,261 |
| FAM234A | family with sequence similarity 234 member A | **0,907** | 0,007245 | 24,039 | 21,134 |
| PPP4C | protein phosphatase 4 catalytic subunit | **0,907** | 0,000275 | 42,318 | 33,266 |
| MAP7D1 | MAP7 domain containing 1 | **0,907** | 0,001266 | 82,199 | 62,637 |
| ANKS3 | ankyrin repeat and sterile alpha motif domain containing 3 | **0,906** | 0,018206 | 9,084 | 10,471 |
| CENPB | centromere protein B | **0,906** | 0,000324 | 89,898 | 69,149 |
| ZC3H3 | zinc finger CCCH-type containing 3 | **0,906** | 0,044031 | 8,032 | 6,321 |
| RTCB | RNA 2',3'-cyclic phosphate and 5'-OH ligase | **0,906** | 0,00268 | 64,530 | 59,380 |
| SPRYD3 | SPRY domain containing 3 | **0,906** | 0,00172 | 98,816 | 80,387 |
| PLCB1 | phospholipase C beta 1 | **0,906** | 0,097394 | 8,309 | 5,172 |
| FAN1 | FANCD2 and FANCI associated nuclease 1 | **0,906** | 0,54579 | 0,222 | 0,575 |
| BATF | basic leucine zipper ATF-like transcription factor | **0,906** | 0,306629 | 1,772 | 1,469 |
| ZNF48 | zinc finger protein 48 | **0,906** | 0,031987 | 6,481 | 6,321 |
| MTA1 | metastasis associated 1 | **0,906** | 0,002141 | 22,655 | 21,134 |
| KRT8 | keratin 8 | **0,906** | 0,060954 | 7,644 | 6,704 |
| HDHD2 | haloacid dehalogenase like hydrolase domain containing 2 | **0,906** | 0,030058 | 6,038 | 6,257 |
| TBC1D10B | TBC1 domain family member 10B | **0,906** | 0,001709 | 41,930 | 31,031 |
| ILF3-AS1 | ILF3 antisense RNA 1 (head to head) | **0,906** | 0,015082 | 18,168 | 21,581 |
| OSBPL5 | oxysterol binding protein like 5 | **0,906** | 0,00714 | 28,803 | 29,179 |

| | | | | | |
|---|---|---|---|---|---|
| EPHB2 | EPH receptor B2 | **0,906** | 0,000124 | 70,401 | 77,833 |
| ZNF620 | zinc finger protein 620 | **0,906** | 0,273234 | 1,385 | 1,405 |
| PLA2G2A | phospholipase A2 group IIA | **0,906** | 0,000761 | 351,617 | 239,629 |
| C19orf54 | chromosome 19 open reading frame 54 | **0,906** | 0,011345 | 7,921 | 9,897 |
| CDC45 | cell division cycle 45 | **0,906** | 0,47508 | 1,939 | 0,511 |
| DDX49 | DEAD-box helicase 49 | **0,906** | 0,001352 | 22,045 | 22,603 |
| PDLIM1 | PDZ and LIM domain 1 | **0,906** | 0,494135 | 0,997 | 0,638 |
| SSR1 | signal sequence receptor subunit 1 | **0,906** | 0,000355 | 204,777 | 157,326 |
| EFS | embryonal Fyn-associated substrate | **0,906** | 0,055605 | 10,635 | 7,854 |
| NAIF1 | nuclear apoptosis inducing factor 1 | **0,906** | 0,024717 | 8,253 | 5,427 |
| C18orf32 | chromosome 18 open reading frame 32 | **0,906** | 0,119802 | 3,379 | 3,384 |
| EMC10 | ER membrane protein complex subunit 10 | **0,906** | 0,000658 | 125,071 | 94,753 |
| PTGES | prostaglandin E synthase | **0,906** | 0,001132 | 117,649 | 87,091 |
| SGCA | sarcoglycan alpha | **0,906** | 0,383252 | 0,886 | 1,149 |
| MARVELD1 | MARVEL domain containing 1 | **0,906** | 9,50E-05 | 103,857 | 99,670 |
| C17orf58 | chromosome 17 open reading frame 58 | **0,906** | 0,012624 | 10,580 | 9,131 |
| TRIM7 | tripartite motif containing 7 | **0,906** | 0,277958 | 1,606 | 2,299 |
| CCDC150 | coiled-coil domain containing 150 | **0,906** | 0,720317 | 0,388 | 0,447 |
| MRPS33 | mitochondrial ribosomal protein S33 | **0,906** | 0,00149 | 26,587 | 31,542 |
| NAGK | N-acetylglucosamine kinase | **0,906** | 0,000671 | 45,974 | 41,886 |
| ZNF805 | zinc finger protein 805 | **0,906** | 0,178753 | 3,102 | 3,256 |
| TMEM259 | transmembrane protein 259 | **0,906** | 0,000153 | 118,203 | 113,270 |
| CYP2J2 | cytochrome P450 family 2 subfamily J member 2 | **0,906** | 0,606563 | 0,332 | 0,383 |
| CEACAM19 | carcinoembryonic antigen related cell adhesion molecule 19 | **0,906** | 0,158923 | 3,656 | 2,873 |
| SH2B3 | SH2B adaptor protein 3 | **0,905** | 0,033742 | 21,381 | 15,132 |
| COG6 | component of oligomeric golgi complex 6 | **0,905** | 0,007061 | 26,920 | 26,115 |
| C1orf159 | chromosome 1 open reading frame 159 | **0,905** | 0,080962 | 6,979 | 7,023 |
| SLAIN1 | SLAIN motif family member 1 | **0,905** | 0,120622 | 3,545 | 4,916 |
| SNRPB | small nuclear ribonucleoprotein polypeptides B and B1 | **0,905** | 0,005064 | 43,537 | 37,224 |
| NSUN5 | NOP2/Sun RNA methyltransferase family member 5 | **0,905** | 0,03745 | 15,066 | 14,877 |
| IRS1 | insulin receptor substrate 1 | **0,905** | 0,01641 | 37,111 | 21,454 |
| TMEM147 | transmembrane protein 147 | **0,905** | 0,014999 | 40,269 | 24,135 |
| C8orf58 | chromosome 8 open reading frame 58 | **0,905** | 0,08843 | 4,320 | 4,278 |

| | | | | | |
|---|---|---|---|---|---|
| UBL5 | ubiquitin like 5 | **0,905** | 0,002678 | 48,411 | 42,460 |
| SFT2D1 | SFT2 domain containing 1 | **0,905** | 0,003144 | 33,068 | 26,817 |
| TFB1M | transcription factor B1, mitochondrial | **0,905** | 0,021904 | 4,930 | 5,300 |
| CHST10 | carbohydrate sulfotransferase 10 | **0,905** | 0,041297 | 9,472 | 8,492 |
| CCDC137 | coiled-coil domain containing 137 | **0,905** | 0,007234 | 15,897 | 17,367 |
| MAGEF1 | MAGE family member F1 | **0,905** | 0,001042 | 30,742 | 24,965 |
| PDGFRA | platelet derived growth factor receptor alpha | **0,905** | 0,003561 | 86,132 | 97,626 |
| ALDH1L2 | aldehyde dehydrogenase 1 family member L2 | **0,905** | 0,070365 | 28,526 | 16,473 |
| MSRB2 | methionine sulfoxide reductase B2 | **0,905** | 0,142047 | 7,644 | 5,108 |
| CHD3 | chromodomain helicase DNA binding protein 3 | **0,905** | 0,048898 | 35,450 | 24,774 |
| NAT2 | N-acetyltransferase 2 | **0,905** | 0,511237 | 0,665 | 0,575 |
| PELP1 | proline, glutamate and leucine rich protein 1 | **0,905** | 0,004039 | 31,517 | 34,160 |
| DCTN3 | dynactin subunit 3 | **0,905** | 0,000919 | 24,095 | 15,771 |
| APEX1 | apurinic/apyrimidinic endodeoxyribonuclease 1 | **0,905** | 0,003343 | 43,370 | 37,161 |
| LINC-PINT | long intergenic non-protein coding RNA, p53 induced transcript | **0,905** | 0,072259 | 5,594 | 4,980 |
| MAB21L2 | mab-21 like 2 | **0,905** | 0,137856 | 3,213 | 3,256 |
| C8orf82 | chromosome 8 open reading frame 82 | **0,905** | 0,011867 | 20,827 | 10,854 |
| STK24-AS1 | STK24 antisense RNA 1 | **0,905** | 0,583984 | 0,277 | 0,575 |
| EDF1 | endothelial differentiation related factor 1 | **0,905** | 0,000745 | 161,905 | 146,089 |
| RBKS | ribokinase | **0,905** | 0,026371 | 5,816 | 7,215 |
| ATP6V1E2 | ATPase H+ transporting V1 subunit E2 | **0,905** | 0,091117 | 5,816 | 5,172 |
| FAM136A | family with sequence similarity 136 member A | **0,905** | 0,03246 | 9,416 | 7,407 |
| TIMM10 | translocase of inner mitochondrial membrane 10 | **0,905** | 0,007979 | 14,512 | 12,323 |
| SNRPF | small nuclear ribonucleoprotein polypeptide F | **0,904** | 0,012934 | 14,291 | 15,260 |
| TTC3 | tetratricopeptide repeat domain 3 | **0,904** | 0,002856 | 93,222 | 69,852 |
| C17orf62 | chromosome 17 open reading frame 62 | **0,904** | 0,002583 | 30,354 | 21,326 |
| COPB2 | coatomer protein complex subunit beta 2 | **0,904** | 0,00022 | 184,948 | 134,085 |
| CRNDE | colorectal neoplasia differentially expressed | **0,904** | 0,083224 | 9,084 | 7,662 |
| GTF3A | general transcription factor IIIA | **0,904** | 0,001369 | 30,908 | 26,753 |
| TARS2 | threonyl-tRNA synthetase 2, mitochondrial (putative) | **0,904** | 0,001251 | 15,232 | 17,176 |
| SLC22A17 | solute carrier family 22 member 17 | **0,904** | 0,008827 | 23,375 | 16,729 |
| EEF1A1P19 | eukaryotic translation elongation factor 1 alpha 1 pseudogene 19 | **0,904** | 0,561567 | 0,499 | 0,511 |
| ZWILCH | zwilch kinetochore protein | **0,904** | 0,026215 | 10,469 | 7,087 |

| | | | | | |
|---|---|---|---|---|---|
| P3H1 | prolyl 3-hydroxylase 1 | **0,904** | 0,001211 | 84,692 | 64,169 |
| AP1S1 | adaptor related protein complex 1 sigma 1 subunit | **0,904** | 0,018546 | 17,282 | 12,323 |
| PNPLA4 | patatin like phospholipase domain containing 4 | **0,904** | 0,031975 | 9,029 | 7,662 |
| SH2D1B | SH2 domain containing 1B | **0,904** | 0,358365 | 1,163 | 1,405 |
| TMED2 | transmembrane p24 trafficking protein 2 | **0,904** | 0,000364 | 234,577 | 174,246 |
| MELTF-AS1 | MELTF antisense RNA 1 | **0,904** | 0,443644 | 0,775 | 0,638 |
| TBC1D32 | TBC1 domain family member 32 | **0,904** | 0,091125 | 3,877 | 4,661 |
| NAP1L3 | nucleosome assembly protein 1 like 3 | **0,904** | 0,070607 | 8,419 | 5,044 |
| RASSF1 | Ras association domain family member 1 | **0,904** | 0,000405 | 26,033 | 21,198 |
| RN7SL2 | RNA, 7SL, cytoplasmic 2 | **0,904** | 0,357836 | 6,924 | 6,385 |
| NUDCD1 | NudC domain containing 1 | **0,904** | 0,003412 | 13,681 | 13,345 |
| FARSB | phenylalanyl-tRNA synthetase beta subunit | **0,904** | 0,000585 | 23,596 | 25,987 |
| MZT2B | mitotic spindle organizing protein 2B | **0,904** | 0,007562 | 30,686 | 15,771 |
| GRAMD1B | GRAM domain containing 1B | **0,904** | 0,185162 | 2,825 | 2,362 |
| SLC22A4 | solute carrier family 22 member 4 | **0,904** | 0,026376 | 10,358 | 11,748 |
| MYO1F | myosin IF | **0,904** | 0,632253 | 0,222 | 0,319 |
| TOMM34 | translocase of outer mitochondrial membrane 34 | **0,904** | 0,003634 | 35,560 | 40,162 |
| OST4 | oligosaccharyltransferase complex subunit 4, non-catalytic | **0,904** | 0,000611 | 92,446 | 72,278 |
| ALG12 | ALG12, alpha-1,6-mannosyltransferase | **0,904** | 0,006343 | 18,002 | 18,453 |
| KNL1 | kinetochore scaffold 1 | **0,904** | 0,280256 | 5,151 | 1,213 |
| NFATC1 | nuclear factor of activated T-cells 1 | **0,904** | 0,005916 | 24,482 | 17,303 |
| ABHD8 | abhydrolase domain containing 8 | **0,904** | 0,025815 | 10,303 | 8,875 |
| HARBI1 | harbinger transposase derived 1 | **0,904** | 0,09527 | 4,265 | 3,448 |
| CCDC106 | coiled-coil domain containing 106 | **0,904** | 0,294111 | 1,883 | 0,702 |
| TMEM57 | transmembrane protein 57 | **0,904** | 0,000731 | 31,406 | 23,752 |
| SERTAD3 | SERTA domain containing 3 | **0,904** | 0,012302 | 11,189 | 7,023 |
| UCN2 | urocortin 2 | **0,904** | 0,55723 | 0,554 | 0,383 |
| PEX2 | peroxisomal biogenesis factor 2 | **0,903** | 0,000535 | 24,095 | 20,113 |
| SF3B5 | splicing factor 3b subunit 5 | **0,903** | 0,00085 | 63,034 | 63,531 |
| MRPL15 | mitochondrial ribosomal protein L15 | **0,903** | 0,007061 | 16,949 | 16,409 |
| SMIM7 | small integral membrane protein 7 | **0,903** | 0,000361 | 34,730 | 34,862 |
| TIMP4 | TIMP metallopeptidase inhibitor 4 | **0,903** | 0,00322 | 24,815 | 18,133 |
| RIN2 | Ras and Rab interactor 2 | **0,903** | 0,00144 | 57,772 | 48,909 |

| | | | | | |
|---|---|---|---|---|---|
| THAP7-AS1 | THAP7 antisense RNA 1 | **0,903** | 0,212969 | 3,046 | 3,001 |
| ALKBH2 | alkB homolog 2, alpha-ketoglutarate dependent dioxygenase | **0,903** | 0,064263 | 9,638 | 8,939 |
| RIBC1 | RIB43A domain with coiled-coils 1 | **0,903** | 0,387887 | 1,052 | 0,958 |
| SLAMF9 | SLAM family member 9 | **0,903** | 0,247978 | 0,665 | 0,575 |
| TOR4A | torsin family 4 member A | **0,903** | 0,001216 | 82,254 | 65,829 |
| LINC00211 | long intergenic non-protein coding RNA 211 | **0,903** | 0,520359 | 0,720 | 1,022 |
| ZSWIM4 | zinc finger SWIM-type containing 4 | **0,903** | 0,003763 | 25,424 | 27,647 |
| YIPF3 | Yip1 domain family member 3 | **0,903** | 0,000145 | 101,530 | 73,619 |
| AGPAT1 | 1-acylglycerol-3-phosphate O-acyltransferase 1 | **0,903** | 0,513009 | 0,942 | 1,149 |
| NDUFA13 | NADH:ubiquinone oxidoreductase subunit A13 | **0,903** | 0,168028 | 2,880 | 2,235 |
| PRAF2 | PRA1 domain family member 2 | **0,902** | 0,0237 | 10,081 | 8,620 |
| CLTB | clathrin light chain B | **0,902** | 0,001857 | 53,895 | 42,779 |
| ZNF704 | zinc finger protein 704 | **0,902** | 0,027616 | 8,862 | 9,322 |
| STK16 | serine/threonine kinase 16 | **0,902** | 0,002742 | 16,285 | 10,663 |
| DMAP1 | DNA methyltransferase 1 associated protein 1 | **0,902** | 0,001286 | 21,547 | 23,688 |
| MRPL37 | mitochondrial ribosomal protein L37 | **0,902** | 0,004193 | 22,655 | 21,964 |
| NDUFB2 | NADH:ubiquinone oxidoreductase subunit B2 | **0,902** | 0,01297 | 23,818 | 20,304 |
| MIF4GD | MIF4G domain containing | **0,902** | 0,032886 | 5,761 | 5,938 |
| GSPT2 | G1 to S phase transition 2 | **0,902** | 0,01087 | 12,851 | 10,854 |
| PARD6A | par-6 family cell polarity regulator alpha | **0,902** | 0,194951 | 2,105 | 2,043 |
| TMEM196 | transmembrane protein 196 | **0,902** | 0,410228 | 0,111 | 1,469 |
| SMARCD3 | SWI/SNF related, matrix associated, actin dependent regulator of chromatin, subfamily d, | **0,902** | 0,315503 | 1,939 | 1,022 |
| MAP2K2 | mitogen-activated protein kinase kinase 2 | **0,902** | 0,001404 | 61,040 | 52,612 |
| THAP4 | THAP domain containing 4 | **0,902** | 0,001805 | 24,039 | 19,985 |
| CBR1 | carbonyl reductase 1 | **0,902** | 0,006772 | 20,937 | 16,218 |
| SKA2 | spindle and kinetochore associated complex subunit 2 | **0,902** | 0,012094 | 10,136 | 5,619 |
| TMEM94 | transmembrane protein 94 | **0,902** | 0,002383 | 23,430 | 21,390 |
| DPY19L3 | dpy-19 like 3 (C. elegans) | **0,902** | 0,004554 | 38,108 | 25,923 |
| AP3B1 | adaptor related protein complex 3 beta 1 subunit | **0,902** | 0,000198 | 46,362 | 33,904 |
| TTC7A | tetratricopeptide repeat domain 7A | **0,902** | 0,000755 | 28,747 | 28,669 |
| SDCCAG3 | serologically defined colon cancer antigen 3 | **0,902** | 0,003211 | 19,497 | 22,603 |
| TMEM154 | transmembrane protein 154 | **0,902** | 0,003108 | 14,069 | 20,049 |
| FER1L4 | fer-1 like family member 4, pseudogene | **0,902** | 0,505454 | 0,443 | 0,255 |

| | | | | | |
|---|---|---|---|---|---|
| GTF2H5 | general transcription factor IIH subunit 5 | **0,901** | 0,015705 | 20,661 | 19,857 |
| NDUFS5 | NADH:ubiquinone oxidoreductase subunit S5 | **0,901** | 0,047139 | 35,394 | 25,668 |
| ENTPD3-AS1 | ENTPD3 antisense RNA 1 | **0,901** | 0,182831 | 1,994 | 2,107 |
| ANAPC11 | anaphase promoting complex subunit 11 | **0,901** | 0,003635 | 31,462 | 27,519 |
| RFPL1S | RFPL1 antisense RNA 1 | **0,901** | 0,541614 | 0,443 | 0,383 |
| PXYLP1 | 2-phosphoxylose phosphatase 1 | **0,901** | 0,00086 | 62,480 | 43,546 |
| PODNL1 | podocan like 1 | **0,901** | 0,012465 | 43,592 | 13,919 |
| GINS2 | GINS complex subunit 2 | **0,901** | 0,337059 | 2,548 | 1,341 |
| AURKB | aurora kinase B | **0,901** | 0,524357 | 2,326 | 0,128 |
| PCBD1 | pterin-4 alpha-carbinolamine dehydratase 1 | **0,901** | 0,024363 | 8,973 | 7,343 |
| LPCAT1 | lysophosphatidylcholine acyltransferase 1 | **0,901** | 0,524609 | 0,443 | 0,894 |
| NCK1-AS1 | NCK1 antisense RNA 1 (head to head) | **0,901** | 0,321091 | 0,942 | 1,405 |
| FOXP3 | forkhead box P3 | **0,901** | 0,616807 | 0,443 | 0,255 |
| GLA | galactosidase alpha | **0,901** | 0,017708 | 18,445 | 9,194 |
| HNRNPA1 | heterogeneous nuclear ribonucleoprotein A1 | **0,901** | 0,000283 | 226,324 | 196,977 |
| MTHFS | methenyltetrahydrofolate synthetase | **0,901** | 0,260185 | 1,939 | 1,532 |
| SNRPD3 | small nuclear ribonucleoprotein D3 polypeptide | **0,901** | 4,90E-05 | 36,668 | 38,118 |
| USE1 | unconventional SNARE in the ER 1 | **0,901** | 0,021589 | 13,404 | 12,834 |
| NDUFC2 | NADH:ubiquinone oxidoreductase subunit C2 | **0,901** | 0,017091 | 17,005 | 13,472 |
| SGPL1 | sphingosine-1-phosphate lyase 1 | **0,901** | 0,003553 | 26,587 | 21,901 |
| PCDHGA1 | protocadherin gamma subfamily A, 1 | **0,901** | 0,440939 | 1,385 | 0,958 |
| GPN1 | GPN-loop GTPase 1 | **0,901** | 0,0025 | 13,349 | 12,642 |
| LDHD | lactate dehydrogenase D | **0,901** | 0,22946 | 1,274 | 1,341 |
| APOPT1 | apoptogenic 1, mitochondrial | **0,901** | 0,048623 | 7,976 | 6,640 |
| ZNF561-AS1 | ZNF561 antisense RNA 1 (head to head) | **0,901** | 0,291026 | 2,382 | 2,682 |
| AP2M1 | adaptor related protein complex 2 mu 1 subunit | **0,900** | 8,20E-05 | 174,147 | 126,806 |
| PIGF | phosphatidylinositol glycan anchor biosynthesis class F | **0,900** | 0,003554 | 12,906 | 15,516 |
| PCBD2 | pterin-4 alpha-carbinolamine dehydratase 2 | **0,900** | 0,051935 | 5,484 | 6,704 |
| ACOT11 | acyl-CoA thioesterase 11 | **0,900** | 0,419172 | 1,274 | 0,575 |
| KIAA1024 | KIAA1024 | **0,900** | 0,256005 | 0,886 | 1,724 |
| DPM2 | dolichyl-phosphate mannosyltransferase subunit 2, regulatory | **0,900** | 0,002024 | 18,500 | 18,644 |
| NDUFAB1 | NADH:ubiquinone oxidoreductase subunit AB1 | **0,900** | 0,001367 | 26,753 | 25,540 |
| ACOT9 | acyl-CoA thioesterase 9 | **0,900** | 0,000831 | 48,466 | 44,631 |

| | | | | | |
|---|---|---|---|---|---|
| IRAK1 | interleukin 1 receptor associated kinase 1 | **0,900** | 0,000192 | 108,177 | 83,516 |
| PPA1 | pyrophosphatase (inorganic) 1 | **0,900** | 0,016473 | 37,001 | 34,224 |
| SHMT2 | serine hydroxymethyltransferase 2 | **0,900** | 3,80E-05 | 215,966 | 164,733 |
| P2RX4 | purinergic receptor P2X 4 | **0,900** | 0,006658 | 26,200 | 17,048 |
| TIMM9 | translocase of inner mitochondrial membrane 9 | **0,900** | 0,034056 | 9,638 | 7,790 |
| NELFCD | negative elongation factor complex member C/D | **0,900** | 0,000265 | 26,089 | 34,351 |
| LRSAM1 | leucine rich repeat and sterile alpha motif containing 1 | **0,900** | 0,002589 | 13,072 | 11,174 |
| JDP2 | Jun dimerization protein 2 | **0,900** | 0,002461 | 22,987 | 16,154 |
| KIAA0141 | KIAA0141 | **0,900** | 0,000721 | 29,191 | 27,775 |
| PARP3 | poly(ADP-ribose) polymerase family member 3 | **0,900** | 0,013167 | 11,078 | 8,556 |
| NCKIPSD | NCK interacting protein with SH3 domain | **0,900** | 0,002193 | 21,436 | 16,090 |
| FUT11 | fucosyltransferase 11 | **0,900** | 0,000344 | 39,216 | 27,455 |
| COX10 | COX10, heme A:farnesyltransferase cytochrome c oxidase assembly factor | **0,900** | 0,035665 | 5,151 | 4,725 |
| ACOX3 | acyl-CoA oxidase 3, pristanoyl | **0,900** | 0,024776 | 7,644 | 5,491 |
| EHBP1 | EH domain binding protein 1 | **0,900** | 0,007384 | 28,747 | 22,028 |
| NAGPA | N-acetylglucosamine-1-phosphodiester alpha-N-acetylglucosaminidase | **0,900** | 0,025645 | 12,241 | 9,322 |
| DLAT | dihydrolipoamide S-acetyltransferase | **0,900** | 0,00255 | 30,188 | 25,157 |
| PUS7L | pseudouridylate synthase 7 like | **0,900** | 0,03104 | 10,746 | 8,556 |
| EIF2A | eukaryotic translation initiation factor 2A | **0,900** | 2,10E-05 | 57,163 | 55,805 |
| WDSUB1 | WD repeat, sterile alpha motif and U-box domain containing 1 | **0,900** | 0,043143 | 9,749 | 4,725 |
| ENPP4 | ectonucleotide pyrophosphatase/phosphodiesterase 4 (putative) | **0,900** | 0,04961 | 9,250 | 3,256 |
| E2F4 | E2F transcription factor 4 | **0,900** | 0,000353 | 32,182 | 33,266 |
| DNER | delta/notch like EGF repeat containing | **0,900** | 4,70E-05 | 146,452 | 89,581 |
| TAF11 | TATA-box binding protein associated factor 11 | **0,900** | 0,005365 | 11,189 | 9,514 |
| IL32 | interleukin 32 | **0,900** | 0,077497 | 8,475 | 4,086 |
| TMCO6 | transmembrane and coiled-coil domains 6 | **0,899** | 0,164921 | 3,434 | 3,959 |
| CHCHD2 | coiled-coil-helix-coiled-coil-helix domain containing 2 | **0,899** | 0,000565 | 78,820 | 68,575 |
| COA3 | cytochrome c oxidase assembly factor 3 | **0,899** | 0,0059 | 19,387 | 14,239 |
| PPIL3 | peptidylprolyl isomerase like 3 | **0,899** | 0,004712 | 15,177 | 13,983 |
| POLDIP2 | DNA polymerase delta interacting protein 2 | **0,899** | 0,000271 | 53,507 | 43,354 |
| DNTTIP1 | deoxynucleotidyltransferase terminal interacting protein 1 | **0,899** | 0,000986 | 24,261 | 25,221 |
| DDX56 | DEAD-box helicase 56 | **0,899** | 0,000211 | 32,348 | 37,288 |
| OAF | out at first homolog | **0,899** | 6,30E-05 | 66,302 | 56,379 |

| | | | | | |
|---|---|---|---|---|---|
| TNFRSF10A | TNF receptor superfamily member 10a | **0,899** | 0,116105 | 9,693 | 12,131 |
| P4HB | prolyl 4-hydroxylase subunit beta | **0,899** | 7,40E-05 | 1270,540 | 625,282 |
| ACAT2 | acetyl-CoA acetyltransferase 2 | **0,899** | 0,022639 | 32,071 | 19,857 |
| FSIP1 | fibrous sheath interacting protein 1 | **0,899** | 0,355524 | 0,997 | 0,958 |
| SRRM2-AS1 | SRRM2 antisense RNA 1 | **0,899** | 0,30382 | 0,997 | 0,894 |
| PPIAP22 | peptidylprolyl isomerase A pseudogene 22 | **0,899** | 0,288615 | 2,216 | 0,766 |
| ARMCX5 | armadillo repeat containing, X-linked 5 | **0,899** | 0,006312 | 9,804 | 9,322 |
| SF3A2 | splicing factor 3a subunit 2 | **0,899** | 0,00032 | 27,806 | 28,924 |
| PGLS | 6-phosphogluconolactonase | **0,899** | 0,00675 | 36,059 | 25,476 |
| PSPH | phosphoserine phosphatase | **0,899** | 0,035793 | 10,247 | 9,003 |
| ZFAND4 | zinc finger AN1-type containing 4 | **0,899** | 0,029111 | 7,201 | 7,534 |
| GALNT18 | polypeptide N-acetylgalactosaminyltransferase 18 | **0,899** | 0,00224 | 32,292 | 30,393 |
| NR2F6 | nuclear receptor subfamily 2 group F member 6 | **0,899** | 0,003808 | 17,780 | 12,962 |
| DUT | deoxyuridine triphosphatase | **0,899** | 0,003248 | 29,745 | 27,711 |
| IQGAP3 | IQ motif containing GTPase activating protein 3 | **0,899** | 0,051488 | 10,967 | 2,171 |
| EVC | EvC ciliary complex subunit 1 | **0,899** | 0,033678 | 22,876 | 18,070 |
| IL1RL2 | interleukin 1 receptor like 2 | **0,899** | 0,167598 | 1,772 | 2,107 |
| PKDCC | protein kinase domain containing, cytoplasmic | **0,899** | 0,227459 | 2,936 | 3,576 |
| R3HDM4 | R3H domain containing 4 | **0,899** | 0,000284 | 32,625 | 27,775 |
| TMEM63A | transmembrane protein 63A | **0,899** | 0,000866 | 17,725 | 17,942 |
| NPRL3 | NPR3 like, GATOR1 complex subunit | **0,899** | 0,003549 | 16,395 | 14,622 |
| CRIM1 | cysteine rich transmembrane BMP regulator 1 | **0,899** | 0,000943 | 253,188 | 144,109 |
| CLYBL | citrate lyase beta like | **0,899** | 0,458789 | 1,163 | 0,766 |
| ZNF215 | zinc finger protein 215 | **0,899** | 0,131099 | 2,714 | 2,362 |
| VPS51 | VPS51, GARP complex subunit | **0,899** | 0,002793 | 36,834 | 26,370 |
| PPCDC | phosphopantothenoylcysteine decarboxylase | **0,898** | 0,048213 | 5,871 | 6,577 |
| AKR1C1 | aldo-keto reductase family 1 member C1 | **0,898** | 0,000712 | 283,210 | 98,265 |
| POU2F3 | POU class 2 homeobox 3 | **0,898** | 0,360986 | 0,997 | 0,702 |
| PGRMC1 | progesterone receptor membrane component 1 | **0,898** | 0,000211 | 117,372 | 92,008 |
| FASTKD1 | FAST kinase domains 1 | **0,898** | 0,030417 | 8,585 | 6,513 |
| KLHL26 | kelch like family member 26 | **0,898** | 0,025 | 9,693 | 7,790 |
| ANKRD34A | ankyrin repeat domain 34A | **0,898** | 0,42931 | 0,831 | 0,830 |
| OLA1 | Obg like ATPase 1 | **0,898** | 0,002615 | 30,465 | 29,946 |

| | | | | | |
|---|---|---|---|---|---|
| SENP3 | SUMO1/sentrin/SMT3 specific peptidase 3 | **0,898** | 0,014647 | 10,247 | 9,705 |
| COPZ1 | coatomer protein complex subunit zeta 1 | **0,898** | 6,10E-05 | 77,934 | 67,042 |
| SPEG | SPEG complex locus | **0,898** | 0,374916 | 1,329 | 0,638 |
| IFITM1 | interferon induced transmembrane protein 1 | **0,898** | 0,322716 | 2,160 | 1,596 |
| ASPSCR1 | ASPSCR1, UBX domain containing tether for SLC2A4 | **0,898** | 0,006302 | 16,894 | 18,325 |
| MRPS16 | mitochondrial ribosomal protein S16 | **0,898** | 0,000199 | 41,543 | 34,734 |
| FBXO15 | F-box protein 15 | **0,898** | 0,461282 | 0,554 | 0,575 |
| DEPDC1 | DEP domain containing 1 | **0,898** | 0,513454 | 2,160 | 0,128 |
| NUS1 | NUS1 dehydrodolichyl diphosphate synthase subunit | **0,898** | 0,003041 | 36,114 | 30,137 |
| LINC01415 | long intergenic non-protein coding RNA 1415 | **0,898** | 0,418072 | 0,388 | 1,149 |
| ZFHX4 | zinc finger homeobox 4 | **0,898** | 0,107123 | 9,915 | 5,491 |
| HEATR6 | HEAT repeat containing 6 | **0,898** | 0,035277 | 9,306 | 9,961 |
| OXLD1 | oxidoreductase like domain containing 1 | **0,898** | 0,03072 | 9,859 | 7,981 |
| POC5 | POC5 centriolar protein | **0,898** | 0,042775 | 7,090 | 6,640 |
| LRRC59 | leucine rich repeat containing 59 | **0,898** | 7,20E-05 | 126,733 | 104,778 |
| SYS1 | SYS1, golgi trafficking protein | **0,898** | 0,001069 | 18,113 | 17,239 |
| PVR | poliovirus receptor | **0,898** | 6,40E-05 | 95,160 | 68,128 |
| MRPL1 | mitochondrial ribosomal protein L1 | **0,898** | 0,036779 | 7,810 | 10,024 |
| CNKSR2 | connector enhancer of kinase suppressor of Ras 2 | **0,897** | 0,512393 | 0,499 | 0,958 |
| FNBP1L | formin binding protein 1 like | **0,897** | 0,252182 | 3,822 | 2,171 |
| SLC35G2 | solute carrier family 35 member G2 | **0,897** | 0,007189 | 24,981 | 15,643 |
| DGKG | diacylglycerol kinase gamma | **0,897** | 0,054688 | 3,877 | 4,597 |
| NBDY | negative regulator of P-body association | **0,897** | 0,000313 | 33,456 | 28,860 |
| MBD2 | methyl-CpG binding domain protein 2 | **0,897** | 0,00065 | 40,158 | 27,902 |
| RFXANK | regulatory factor X associated ankyrin containing protein | **0,897** | 0,009363 | 11,743 | 10,024 |
| ARF1 | ADP ribosylation factor 1 | **0,897** | 9,00E-06 | 311,016 | 232,988 |
| KIF18A | kinesin family member 18A | **0,897** | 0,28556 | 2,216 | 1,532 |
| GPI | glucose-6-phosphate isomerase | **0,897** | 0,000177 | 66,357 | 47,951 |
| URGCP | upregulator of cell proliferation | **0,897** | 0,000199 | 50,737 | 40,481 |
| WDR76 | WD repeat domain 76 | **0,897** | 0,092039 | 4,874 | 2,235 |
| S100A4 | S100 calcium binding protein A4 | **0,897** | 0,017903 | 8,309 | 9,386 |
| CARM1 | coactivator associated arginine methyltransferase 1 | **0,897** | 0,000889 | 25,036 | 18,580 |
| ARHGEF35 | Rho guanine nucleotide exchange factor 35 | **0,897** | 0,557735 | 0,554 | 0,383 |

| | | | | | |
|---|---|---|---|---|---|
| SLC35C2 | solute carrier family 35 member C2 | **0,897** | 6,70E-05 | 42,706 | 38,310 |
| SEC31A | SEC31 homolog A, COPII coat complex component | **0,897** | 0,001443 | 188,604 | 137,213 |
| RPAP1 | RNA polymerase II associated protein 1 | **0,897** | 0,000498 | 24,870 | 20,815 |
| RACK1 | receptor for activated C kinase 1 | **0,897** | 0,000167 | 382,358 | 292,879 |
| MAP1B | microtubule associated protein 1B | **0,897** | 0,018817 | 26,089 | 25,668 |
| URB1-AS1 | URB1 antisense RNA 1 (head to head) | **0,897** | 0,128413 | 2,880 | 2,490 |
| TXNDC11 | thioredoxin domain containing 11 | **0,897** | 0,000111 | 56,886 | 46,100 |
| LINC01503 | long intergenic non-protein coding RNA 1503 | **0,897** | 0,059771 | 3,379 | 5,300 |
| SOX2-OT | SOX2 overlapping transcript | **0,897** | 0,514303 | 0,554 | 0,255 |
| QTRT2 | queuine tRNA-ribosyltransferase accessory subunit 2 | **0,897** | 0,001801 | 16,562 | 19,921 |
| MARK4 | microtubule affinity regulating kinase 4 | **0,897** | 0,006177 | 14,568 | 11,940 |
| ANTXR1 | anthrax toxin receptor 1 | **0,897** | 0,000131 | 49,962 | 47,440 |
| ZNF843 | zinc finger protein 843 | **0,897** | 0,141718 | 2,548 | 2,299 |
| C11orf68 | chromosome 11 open reading frame 68 | **0,897** | 0,000385 | 106,903 | 99,989 |
| NHLRC1 | NHL repeat containing E3 ubiquitin protein ligase 1 | **0,897** | 0,105251 | 2,382 | 3,129 |
| WIPF1 | WAS/WASL interacting protein family member 1 | **0,897** | 0,006772 | 22,932 | 15,962 |
| DRG1 | developmentally regulated GTP binding protein 1 | **0,896** | 0,001519 | 25,313 | 19,155 |
| ZNF358 | zinc finger protein 358 | **0,896** | 0,006326 | 19,110 | 9,769 |
| KIF22 | kinesin family member 22 | **0,896** | 0,005333 | 12,518 | 9,322 |
| MRPL27 | mitochondrial ribosomal protein L27 | **0,896** | 0,007609 | 21,104 | 25,604 |
| MAP3K10 | mitogen-activated protein kinase kinase kinase 10 | **0,896** | 0,010536 | 15,398 | 14,430 |
| EMP2 | epithelial membrane protein 2 | **0,896** | 4,90E-05 | 95,714 | 48,845 |
| PIGB | phosphatidylinositol glycan anchor biosynthesis class B | **0,896** | 0,01273 | 7,810 | 6,896 |
| MIR4435-2HG | MIR4435-2 host gene | **0,896** | 0,01735 | 11,743 | 6,832 |
| MINK1 | misshapen like kinase 1 | **0,896** | 0,000688 | 35,948 | 21,709 |
| FBXW5 | F-box and WD repeat domain containing 5 | **0,896** | 6,40E-05 | 86,353 | 66,659 |
| CCT6P3 | chaperonin containing TCP1 subunit 6 pseudogene 3 | **0,896** | 0,386006 | 0,499 | 0,702 |
| SEC23A | Sec23 homolog A, coat complex II component | **0,896** | 0,000838 | 95,105 | 61,168 |
| C7orf73 | chromosome 7 open reading frame 73 | **0,896** | 0,00032 | 41,432 | 26,817 |
| SLC35C1 | solute carrier family 35 member C1 | **0,896** | 0,002329 | 21,658 | 18,516 |
| CCDC124 | coiled-coil domain containing 124 | **0,896** | 0,004117 | 46,306 | 38,438 |
| COQ4 | coenzyme Q4 | **0,896** | 0,008223 | 14,180 | 14,430 |
| MRPL22 | mitochondrial ribosomal protein L22 | **0,896** | 0,001799 | 14,457 | 13,792 |

| | | | | | |
|---|---|---|---|---|---|
| RCAN1 | regulator of calcineurin 1 | **0,896** | 2,20E-05 | 314,948 | 499,561 |
| CPSF3 | cleavage and polyadenylation specific factor 3 | **0,896** | 0,00187 | 20,605 | 16,856 |
| SLC4A4 | solute carrier family 4 member 4 | **0,896** | 0,041742 | 12,297 | 4,086 |
| PDCD2 | programmed cell death 2 | **0,896** | 0,003771 | 20,384 | 21,645 |
| CNPY4 | canopy FGF signaling regulator 4 | **0,896** | 0,025958 | 8,918 | 6,704 |
| COX7B | cytochrome c oxidase subunit 7B | **0,896** | 0,000326 | 20,882 | 17,559 |
| SCFD1 | sec1 family domain containing 1 | **0,896** | 4,20E-05 | 45,586 | 35,054 |
| TMEM223 | transmembrane protein 223 | **0,896** | 0,057667 | 4,154 | 3,767 |
| EMX2OS | EMX2 opposite strand/antisense RNA | **0,896** | 0,386788 | 0,831 | 0,958 |
| SPAG16 | sperm associated antigen 16 | **0,896** | 0,028112 | 6,148 | 6,896 |
| THOC3 | THO complex 3 | **0,896** | 0,167707 | 2,160 | 2,809 |
| DDB1 | damage specific DNA binding protein 1 | **0,896** | 4,60E-05 | 137,257 | 113,142 |
| B9D2 | B9 protein domain 2 | **0,896** | 0,074988 | 4,043 | 3,256 |
| TATDN3 | TatD DNase domain containing 3 | **0,896** | 0,029152 | 7,810 | 9,514 |
| PDSS2 | decaprenyl diphosphate synthase subunit 2 | **0,896** | 0,008621 | 11,743 | 12,706 |
| IVD | isovaleryl-CoA dehydrogenase | **0,895** | 0,002643 | 29,634 | 32,053 |
| SSBP1 | single stranded DNA binding protein 1 | **0,895** | 0,02293 | 11,853 | 11,110 |
| ACP6 | acid phosphatase 6, lysophosphatidic | **0,895** | 0,119921 | 4,487 | 4,789 |
| MRPL57 | mitochondrial ribosomal protein L57 | **0,895** | 0,000894 | 20,550 | 20,560 |
| DHODH | dihydroorotate dehydrogenase (quinone) | **0,895** | 0,026186 | 6,425 | 7,470 |
| ARAP2 | ArfGAP with RhoGAP domain, ankyrin repeat and PH domain 2 | **0,895** | 0,004074 | 12,407 | 11,174 |
| SNAPC1 | small nuclear RNA activating complex polypeptide 1 | **0,895** | 0,000303 | 42,207 | 63,786 |
| PROCA1 | protein interacting with cyclin A1 | **0,895** | 0,558489 | 0,554 | 0,255 |
| MRPL9 | mitochondrial ribosomal protein L9 | **0,895** | 0,003633 | 22,101 | 20,624 |
| WDR3 | WD repeat domain 3 | **0,895** | 0,000131 | 22,932 | 25,923 |
| KRI1 | KRI1 homolog | **0,895** | 0,007435 | 19,996 | 15,388 |
| GTF2F1 | general transcription factor IIF subunit 1 | **0,895** | 3,10E-05 | 63,865 | 50,186 |
| LONP1 | lon peptidase 1, mitochondrial | **0,895** | 7,70E-05 | 86,298 | 88,687 |
| NME7 | NME/NM23 family member 7 | **0,895** | 0,002841 | 16,783 | 16,920 |
| MLX | MLX, MAX dimerization protein | **0,895** | 0,000144 | 29,024 | 26,689 |
| C10orf25 | chromosome 10 open reading frame 25 | **0,895** | 0,11953 | 2,382 | 2,362 |
| RWDD2B | RWD domain containing 2B | **0,895** | 0,006035 | 10,580 | 12,131 |
| TXNDC15 | thioredoxin domain containing 15 | **0,895** | 5,60E-05 | 77,934 | 53,315 |

| | | | | | |
|---|---|---|---|---|---|
| MRPL11 | mitochondrial ribosomal protein L11 | **0,895** | 0,007973 | 11,909 | 11,748 |
| ARL16 | ADP ribosylation factor like GTPase 16 | **0,895** | 0,010195 | 10,136 | 10,088 |
| FUOM | fucose mutarotase | **0,895** | 0,345093 | 1,662 | 0,511 |
| DPH6 | diphthamine biosynthesis 6 | **0,895** | 0,090532 | 3,434 | 3,767 |
| PRKCH | protein kinase C eta | **0,895** | 0,027501 | 5,207 | 6,385 |
| POLR3A | RNA polymerase III subunit A | **0,895** | 8,10E-05 | 33,068 | 32,180 |
| TSSC4 | tumor suppressing subtransferable candidate 4 | **0,895** | 0,002843 | 26,421 | 24,582 |
| CCDC113 | coiled-coil domain containing 113 | **0,895** | 0,0272 | 6,702 | 6,513 |
| RCN1 | reticulocalbin 1 | **0,895** | 0,000489 | 67,576 | 44,631 |
| HSPE1 | heat shock protein family E (Hsp10) member 1 | **0,895** | 0,016436 | 12,186 | 10,791 |
| FBXO24 | F-box protein 24 | **0,895** | 0,417579 | 0,277 | 0,511 |
| YDJC | YdjC chitooligosaccharide deacetylase homolog | **0,895** | 0,051587 | 6,038 | 4,597 |
| TIMMDC1 | translocase of inner mitochondrial membrane domain containing 1 | **0,894** | 0,00103 | 45,475 | 42,013 |
| ANKRD9 | ankyrin repeat domain 9 | **0,894** | 0,018933 | 10,524 | 7,854 |
| LGI4 | leucine rich repeat LGI family member 4 | **0,894** | 0,319245 | 1,052 | 1,341 |
| ACAA2 | acetyl-CoA acyltransferase 2 | **0,894** | 0,030699 | 13,626 | 10,663 |
| TAB1 | TGF-beta activated kinase 1 (MAP3K7) binding protein 1 | **0,894** | 0,000526 | 18,445 | 16,665 |
| IFT81 | intraflagellar transport 81 | **0,894** | 0,082335 | 5,927 | 3,384 |
| CUEDC1 | CUE domain containing 1 | **0,894** | 0,001142 | 25,812 | 19,666 |
| MTA2 | metastasis associated 1 family member 2 | **0,894** | 2,90E-05 | 43,370 | 34,734 |
| DYRK4 | dual specificity tyrosine phosphorylation regulated kinase 4 | **0,894** | 0,013566 | 8,973 | 8,747 |
| HSD17B11 | hydroxysteroid 17-beta dehydrogenase 11 | **0,894** | 0,179683 | 1,772 | 2,299 |
| POLR3B | RNA polymerase III subunit B | **0,894** | 0,01495 | 7,145 | 7,087 |
| NUP37 | nucleoporin 37 | **0,894** | 0,036404 | 10,192 | 9,194 |
| PSMB7 | proteasome subunit beta 7 | **0,894** | 4,80E-05 | 116,929 | 97,499 |
| SAAL1 | serum amyloid A like 1 | **0,894** | 0,079367 | 3,656 | 3,831 |
| GSS | glutathione synthetase | **0,894** | 0,000165 | 34,785 | 31,606 |
| LHX9 | LIM homeobox 9 | **0,894** | 0,394751 | 0,222 | 0,894 |
| FPGS | folylpolyglutamate synthase | **0,894** | 0,000691 | 36,391 | 31,733 |
| RPN1 | ribophorin I | **0,894** | 3,70E-05 | 236,516 | 145,705 |
| LRRC1 | leucine rich repeat containing 1 | **0,894** | 0,035389 | 8,253 | 5,427 |
| CTDNEP1 | CTD nuclear envelope phosphatase 1 | **0,894** | 0,000149 | 45,586 | 37,352 |
| GEMIN6 | gem nuclear organelle associated protein 6 | **0,894** | 0,060896 | 6,758 | 6,513 |

| | | | | | |
|---|---|---|---|---|---|
| CYP27C1 | cytochrome P450 family 27 subfamily C member 1 | **0,894** | 0,11468 | 5,761 | 5,683 |
| SLC44A3 | solute carrier family 44 member 3 | **0,894** | 0,448126 | 1,163 | 0,383 |
| HOXA6 | homeobox A6 | **0,894** | 0,333232 | 0,665 | 1,724 |
| HAGHL | hydroxyacylglutathione hydrolase-like | **0,894** | 0,178645 | 1,828 | 1,277 |
| EAF2 | ELL associated factor 2 | **0,894** | 0,127522 | 1,606 | 2,171 |
| AOC2 | amine oxidase, copper containing 2 | **0,894** | 5,90E-05 | 111,500 | 43,099 |
| ZNF853 | zinc finger protein 853 | **0,894** | 0,034425 | 6,314 | 5,300 |
| SERP2 | stress associated endoplasmic reticulum protein family member 2 | **0,894** | 0,090705 | 3,213 | 2,426 |
| SCHIP1 | schwannomin interacting protein 1 | **0,894** | 0,577126 | 0,332 | 0,383 |
| AGBL5 | ATP/GTP binding protein like 5 | **0,894** | 0,016467 | 8,918 | 5,746 |
| FUNDC1 | FUN14 domain containing 1 | **0,894** | 0,016136 | 10,358 | 8,045 |
| MEN1 | menin 1 | **0,894** | 0,000568 | 21,658 | 19,985 |
| ATG4D | autophagy related 4D cysteine peptidase | **0,894** | 0,004669 | 10,136 | 11,812 |
| C12orf10 | chromosome 12 open reading frame 10 | **0,893** | 0,000437 | 19,331 | 16,154 |
| CTU1 | cytosolic thiouridylase subunit 1 | **0,893** | 0,033158 | 6,314 | 5,683 |
| ANAPC15 | anaphase promoting complex subunit 15 | **0,893** | 0,103444 | 4,320 | 5,044 |
| ELOVL1 | ELOVL fatty acid elongase 1 | **0,893** | 2,60E-05 | 51,734 | 33,777 |
| POMC | proopiomelanocortin | **0,893** | 0,258746 | 1,440 | 0,830 |
| ATP5J2 | ATP synthase, H+ transporting, mitochondrial Fo complex subunit F2 | **0,893** | 0,008276 | 17,005 | 12,706 |
| ECSIT | ECSIT signalling integrator | **0,893** | 0,00073 | 16,395 | 12,578 |
| RAB3D | RAB3D, member RAS oncogene family | **0,893** | 0,066625 | 3,102 | 3,448 |
| EGR2 | early growth response 2 | **0,893** | 0,094032 | 1,440 | 3,065 |
| SIVA1 | SIVA1 apoptosis inducing factor | **0,893** | 0,001791 | 29,357 | 21,901 |
| NOC2L | NOC2 like nucleolar associated transcriptional repressor | **0,893** | 0,00068 | 57,329 | 60,147 |
| ACSS2 | acyl-CoA synthetase short-chain family member 2 | **0,893** | 0,012307 | 11,632 | 7,534 |
| TMEM141 | transmembrane protein 141 | **0,893** | 0,023912 | 7,533 | 6,960 |
| MED11 | mediator complex subunit 11 | **0,893** | 0,013361 | 6,979 | 6,960 |
| COLEC12 | collectin subfamily member 12 | **0,893** | 0,000902 | 32,126 | 24,008 |
| STARD13 | StAR related lipid transfer domain containing 13 | **0,893** | 0,000323 | 85,301 | 77,705 |
| LEKR1 | leucine, glutamate and lysine rich 1 | **0,893** | 0,487804 | 0,332 | 0,383 |
| BMF | Bcl2 modifying factor | **0,893** | 0,00665 | 9,306 | 4,853 |
| POLD1 | DNA polymerase delta 1, catalytic subunit | **0,893** | 0,023081 | 9,195 | 6,896 |
| TPX2 | TPX2, microtubule nucleation factor | **0,893** | 0,112505 | 13,238 | 1,213 |

| | | | | | |
|---|---|---|---|---|---|
| B3GAT3 | beta-1,3-glucuronyltransferase 3 | **0,893** | 0,009148 | 18,722 | 12,515 |
| MAP2K5 | mitogen-activated protein kinase kinase 5 | **0,893** | 0,025054 | 9,527 | 7,215 |
| SIRT6 | sirtuin 6 | **0,893** | 0,007132 | 10,303 | 9,641 |
| AKAP12 | A-kinase anchoring protein 12 | **0,893** | 0,040584 | 52,344 | 42,396 |
| TWIST2 | twist family bHLH transcription factor 2 | **0,893** | 0,031544 | 7,755 | 7,151 |
| PATZ1 | POZ/BTB and AT hook containing zinc finger 1 | **0,893** | 0,006027 | 12,297 | 10,471 |
| POLR2E | RNA polymerase II subunit E | **0,892** | 0,000431 | 47,746 | 43,418 |
| FAM131A | family with sequence similarity 131 member A | **0,892** | 0,0693 | 4,985 | 3,831 |
| PRDM15 | PR/SET domain 15 | **0,892** | 0,042678 | 5,650 | 5,108 |
| IL34 | interleukin 34 | **0,892** | 0,400471 | 0,665 | 0,192 |
| BORCS6 | BLOC-1 related complex subunit 6 | **0,892** | 0,088913 | 3,933 | 2,682 |
| GOLGA2 | golgin A2 | **0,892** | 4,70E-05 | 138,365 | 100,819 |
| CDC42EP2 | CDC42 effector protein 2 | **0,892** | 0,050257 | 8,973 | 6,640 |
| ZNF121 | zinc finger protein 121 | **0,892** | 0,013449 | 9,029 | 9,258 |
| ERLEC1 | endoplasmic reticulum lectin 1 | **0,892** | 6,00E-06 | 160,687 | 109,886 |
| TLE2 | transducin like enhancer of split 2 | **0,892** | 0,29844 | 1,274 | 2,043 |
| SEC61B | Sec61 translocon beta subunit | **0,892** | 0,000203 | 73,558 | 63,914 |
| DEPDC5 | DEP domain containing 5 | **0,892** | 0,037712 | 5,317 | 4,916 |
| NDC1 | NDC1 transmembrane nucleoporin | **0,892** | 0,008662 | 13,349 | 12,898 |
| TDRD7 | tudor domain containing 7 | **0,892** | 0,012125 | 11,410 | 9,194 |
| RORB | RAR related orphan receptor B | **0,892** | 0,530198 | 0,609 | 0,511 |
| RARB | retinoic acid receptor beta | **0,892** | 0,000284 | 13,017 | 9,577 |
| RABL6 | RAB, member RAS oncogene family like 6 | **0,892** | 0,00142 | 41,432 | 35,373 |
| ZNF865 | zinc finger protein 865 | **0,892** | 0,002395 | 17,503 | 16,409 |
| CNTF | ciliary neurotrophic factor | **0,892** | 0,559702 | 0,443 | 0,383 |
| PHB2 | prohibitin 2 | **0,892** | 0,000435 | 54,449 | 52,868 |
| DAAM2 | dishevelled associated activator of morphogenesis 2 | **0,892** | 0,045756 | 4,764 | 5,172 |
| SLC8B1 | solute carrier family 8 member B1 | **0,892** | 0,000494 | 30,520 | 30,520 |
| ME3 | malic enzyme 3 | **0,892** | 0,179933 | 1,385 | 1,724 |
| TPCN1 | two pore segment channel 1 | **0,892** | 0,00072 | 22,267 | 23,305 |
| ZNF674-AS1 | ZNF674 antisense RNA 1 (head to head) | **0,892** | 0,101835 | 2,880 | 3,320 |
| UBE2M | ubiquitin conjugating enzyme E2 M | **0,892** | 0,000187 | 55,501 | 43,290 |
| PRCC | papillary renal cell carcinoma (translocation-associated) | **0,892** | 0,000149 | 38,164 | 30,903 |

| | | | | | |
|---|---|---|---|---|---|
| KIAA1644 | KIAA1644 | **0,892** | 0,32005 | 3,600 | 2,362 |
| CNN3 | calponin 3 | **0,892** | 0,000168 | 53,507 | 47,696 |
| HNRNPCP2 | heterogeneous nuclear ribonucleoprotein C pseudogene 2 | **0,892** | 0,502636 | 0,554 | 0,702 |
| FKBP1A | FK506 binding protein 1A | **0,892** | 1,00E-04 | 48,965 | 33,649 |
| TRIM59 | tripartite motif containing 59 | **0,891** | 0,234532 | 4,099 | 2,618 |
| ARFIP2 | ADP ribosylation factor interacting protein 2 | **0,891** | 6,80E-05 | 36,724 | 31,095 |
| VWF | von Willebrand factor | **0,891** | 0,53282 | 0,499 | 0,702 |
| GCFC2 | GC-rich sequence DNA-binding factor 2 | **0,891** | 0,060528 | 5,484 | 4,086 |
| FOSL1 | FOS like 1, AP-1 transcription factor subunit | **0,891** | 9,00E-06 | 235,796 | 254,378 |
| DCAF15 | DDB1 and CUL4 associated factor 15 | **0,891** | 0,008828 | 15,066 | 13,089 |
| CDK5RAP2 | CDK5 regulatory subunit associated protein 2 | **0,891** | 2,20E-05 | 51,125 | 44,631 |
| FBLIM1 | filamin binding LIM protein 1 | **0,891** | 0,345045 | 0,554 | 0,638 |
| PCDHB13 | protocadherin beta 13 | **0,891** | 0,295829 | 1,052 | 1,915 |
| AAAS | aladin WD repeat nucleoporin | **0,891** | 0,002587 | 17,559 | 16,984 |
| UPRT | uracil phosphoribosyltransferase homolog | **0,891** | 0,004812 | 8,807 | 7,854 |
| MRPL3 | mitochondrial ribosomal protein L3 | **0,891** | 1,70E-05 | 44,257 | 42,269 |
| MPRIP | myosin phosphatase Rho interacting protein | **0,891** | 0,000423 | 86,519 | 79,557 |
| ELN | elastin | **0,891** | 0,038086 | 15,620 | 6,577 |
| IYD | iodotyrosine deiodinase | **0,891** | 0,543403 | 0,443 | 0,383 |
| THOC5 | THO complex 5 | **0,891** | 0,008238 | 14,789 | 14,494 |
| LRRC75B | leucine rich repeat containing 75B | **0,891** | 0,237982 | 2,493 | 1,085 |
| U2AF1 | U2 small nuclear RNA auxiliary factor 1 | **0,891** | 0,533496 | 0,775 | 0,638 |
| DNAAF5 | dynein axonemal assembly factor 5 | **0,891** | 0,002243 | 22,267 | 20,560 |
| MEOX2 | mesenchyme homeobox 2 | **0,891** | 0,00213 | 4,930 | 12,770 |
| CCDC144A | coiled-coil domain containing 144A | **0,891** | 0,639734 | 0,665 | 0,830 |
| WWC1 | WW and C2 domain containing 1 | **0,891** | 0,082511 | 5,927 | 2,746 |
| ERI3 | ERI1 exoribonuclease family member 3 | **0,891** | 6,70E-05 | 27,474 | 25,093 |
| PIGBOS1 | PIGB opposite strand 1 | **0,891** | 0,029457 | 5,761 | 4,533 |
| PTTG1 | pituitary tumor-transforming 1 | **0,891** | 0,095825 | 9,638 | 5,108 |
| CHEK1 | checkpoint kinase 1 | **0,891** | 0,107532 | 4,542 | 2,043 |
| MBNL1-AS1 | MBNL1 antisense RNA 1 | **0,891** | 0,053251 | 8,918 | 6,449 |
| HUS1 | HUS1 checkpoint clamp component | **0,891** | 0,007785 | 11,964 | 10,152 |
| TDRD3 | tudor domain containing 3 | **0,891** | 0,052528 | 6,204 | 6,066 |

| | | | | | |
|---|---|---|---|---|---|
| KIF4A | kinesin family member 4A | **0,891** | 0,258847 | 3,988 | 1,085 |
| RNFT2 | ring finger protein, transmembrane 2 | **0,890** | 0,348141 | 1,662 | 0,958 |
| DENND2D | DENN domain containing 2D | **0,890** | 0,069157 | 7,921 | 7,087 |
| SLC36A4 | solute carrier family 36 member 4 | **0,890** | 6,30E-05 | 30,077 | 20,560 |
| MRPL51 | mitochondrial ribosomal protein L51 | **0,890** | 0,000271 | 53,507 | 43,737 |
| CBS | cystathionine-beta-synthase | **0,890** | 0,250952 | 1,219 | 1,022 |
| SGSH | N-sulfoglucosamine sulfohydrolase | **0,890** | 6,40E-05 | 29,689 | 23,178 |
| TUBA1C | tubulin alpha 1c | **0,890** | 2,60E-05 | 90,508 | 79,238 |
| RARA | retinoic acid receptor alpha | **0,890** | 0,004219 | 10,690 | 11,493 |
| GFER | growth factor, augmenter of liver regeneration | **0,890** | 0,006935 | 11,577 | 9,003 |
| TRAF2 | TNF receptor associated factor 2 | **0,890** | 8,60E-05 | 21,713 | 23,241 |
| CES4A | carboxylesterase 4A | **0,890** | 0,433367 | 0,775 | 0,447 |
| SLC18B1 | solute carrier family 18 member B1 | **0,890** | 0,00024 | 20,162 | 16,665 |
| PRCD | photoreceptor disc component | **0,890** | 0,492829 | 0,443 | 0,319 |
| CLPTM1L | CLPTM1 like | **0,890** | 0,00293 | 16,839 | 12,387 |
| CWC27 | CWC27 spliceosome associated protein homolog | **0,890** | 0,000179 | 17,226 | 15,707 |
| ACBD4 | acyl-CoA binding domain containing 4 | **0,890** | 0,159419 | 2,493 | 2,235 |
| TCEA2 | transcription elongation factor A2 | **0,890** | 0,0121 | 11,521 | 11,685 |
| DEPDC7 | DEP domain containing 7 | **0,890** | 0,0487 | 4,874 | 4,342 |
| PSRC1 | proline and serine rich coiled-coil 1 | **0,890** | 0,301192 | 2,105 | 2,235 |
| R3HCC1 | R3H domain and coiled-coil containing 1 | **0,890** | 0,001036 | 20,605 | 18,070 |
| METTL2B | methyltransferase like 2B | **0,890** | 0,000822 | 10,635 | 12,451 |
| BHLHE22 | basic helix-loop-helix family member e22 | **0,890** | 0,307022 | 1,662 | 1,149 |
| MPG | N-methylpurine DNA glycosylase | **0,890** | 0,007362 | 22,322 | 17,686 |
| ERGIC2 | ERGIC and golgi 2 | **0,890** | 0,000339 | 37,222 | 34,798 |
| ASRGL1 | asparaginase like 1 | **0,890** | 0,2848 | 1,385 | 1,277 |
| A1BG-AS1 | A1BG antisense RNA 1 | **0,890** | 0,474889 | 1,108 | 0,255 |
| SNU13 | small nuclear ribonucleoprotein 13 | **0,890** | 6,00E-06 | 59,489 | 54,847 |
| IFT52 | intraflagellar transport 52 | **0,890** | 0,002931 | 12,795 | 9,705 |
| PNMA8A | paraneoplastic Ma antigen family member 8A | **0,889** | 0,12236 | 0,499 | 1,213 |
| MYL6B | myosin light chain 6B | **0,889** | 0,004825 | 22,267 | 14,366 |
| MAST3 | microtubule associated serine/threonine kinase 3 | **0,889** | 0,013431 | 6,758 | 6,577 |
| FBXL13 | F-box and leucine rich repeat protein 13 | **0,889** | 0,465325 | 0,443 | 0,319 |

| | | | | | |
|---|---|---|---|---|---|
| THAP3 | THAP domain containing 3 | **0,889** | 0,001986 | 8,253 | 9,577 |
| FGFBP1 | fibroblast growth factor binding protein 1 | **0,889** | 0,245403 | 1,551 | 1,979 |
| CCDC183 | coiled-coil domain containing 183 | **0,889** | 0,546352 | 0,222 | 0,192 |
| TSEN15 | tRNA splicing endonuclease subunit 15 | **0,889** | 0,000749 | 24,095 | 22,347 |
| LBHD1 | LBH domain containing 1 | **0,889** | 0,165618 | 1,717 | 1,979 |
| IMPACT | impact RWD domain protein | **0,889** | 0,000741 | 22,322 | 16,856 |
| IL17RE | interleukin 17 receptor E | **0,889** | 0,353162 | 0,775 | 1,979 |
| TELO2 | telomere maintenance 2 | **0,889** | 0,000292 | 25,036 | 22,858 |
| HRH3 | histamine receptor H3 | **0,889** | 0,520089 | 0,332 | 0,383 |
| THOP1 | thimet oligopeptidase 1 | **0,889** | 0,000648 | 21,658 | 19,985 |
| FAM225B | family with sequence similarity 225 member B (non-protein coding) | **0,889** | 0,097746 | 2,326 | 2,362 |
| PRRX2 | paired related homeobox 2 | **0,889** | 0,001397 | 28,747 | 28,860 |
| NDUFA4 | NDUFA4, mitochondrial complex associated | **0,889** | 8,60E-05 | 54,559 | 43,673 |
| TTC27 | tetratricopeptide repeat domain 27 | **0,889** | 0,009164 | 8,364 | 11,174 |
| PFKP | phosphofructokinase, platelet | **0,889** | 8,20E-05 | 75,608 | 58,550 |
| ITGA1 | integrin subunit alpha 1 | **0,889** | 8,70E-05 | 69,127 | 115,888 |
| FAM69A | family with sequence similarity 69 member A | **0,889** | 0,003163 | 18,445 | 13,472 |
| LRRC42 | leucine rich repeat containing 42 | **0,889** | 0,000897 | 15,842 | 13,217 |
| ELOVL4 | ELOVL fatty acid elongase 4 | **0,889** | 0,000261 | 19,442 | 13,153 |
| CAMKK1 | calcium/calmodulin dependent protein kinase kinase 1 | **0,889** | 0,001716 | 12,186 | 10,280 |
| PGGHG | protein-glucosylgalactosylhydroxylysine glucosidase | **0,889** | 0,09898 | 5,982 | 2,299 |
| KIF11 | kinesin family member 11 | **0,889** | 0,085626 | 6,148 | 1,979 |
| AKAP7 | A-kinase anchoring protein 7 | **0,889** | 0,159922 | 2,880 | 1,788 |
| ECI2 | enoyl-CoA delta isomerase 2 | **0,888** | 0,006501 | 16,008 | 11,812 |
| MPI | mannose phosphate isomerase | **0,888** | 0,004637 | 18,445 | 16,026 |
| PTGR2 | prostaglandin reductase 2 | **0,888** | 0,08636 | 3,102 | 2,937 |
| MRPL52 | mitochondrial ribosomal protein L52 | **0,888** | 0,008324 | 17,116 | 18,580 |
| SART1 | squamous cell carcinoma antigen recognized by T-cells 1 | **0,888** | 0,000574 | 65,416 | 57,784 |
| FAM3A | family with sequence similarity 3 member A | **0,888** | 0,000282 | 23,264 | 14,813 |
| ALG6 | ALG6, alpha-1,3-glucosyltransferase | **0,888** | 0,06368 | 4,930 | 3,959 |
| DIS3L | DIS3 like exosome 3'-5' exoribonuclease | **0,888** | 0,022777 | 16,506 | 12,195 |
| NPIPB12 | nuclear pore complex interacting protein family, member B12 | **0,888** | 0,702143 | 0,609 | 0,575 |
| SDHAF1 | succinate dehydrogenase complex assembly factor 1 | **0,888** | 0,008309 | 7,312 | 7,151 |

| | | | | | |
|---|---|---|---|---|---|
| MBNL3 | muscleblind like splicing regulator 3 | **0,888** | 0,055124 | 3,933 | 4,406 |
| KLHDC1 | kelch domain containing 1 | **0,888** | 0,052647 | 3,600 | 4,086 |
| POFUT2 | protein O-fucosyltransferase 2 | **0,888** | 9,70E-05 | 84,470 | 63,594 |
| CCT3 | chaperonin containing TCP1 subunit 3 | **0,888** | 2,00E-06 | 117,150 | 98,712 |
| CCT2 | chaperonin containing TCP1 subunit 2 | **0,888** | 8,00E-06 | 69,958 | 68,064 |
| BCCIP | BRCA2 and CDKN1A interacting protein | **0,888** | 9,40E-05 | 32,569 | 35,437 |
| SPRYD4 | SPRY domain containing 4 | **0,888** | 0,002905 | 12,130 | 12,387 |
| IRAK1BP1 | interleukin 1 receptor associated kinase 1 binding protein 1 | **0,888** | 0,142958 | 1,496 | 2,746 |
| HIBCH | 3-hydroxyisobutyryl-CoA hydrolase | **0,888** | 0,03596 | 7,810 | 5,300 |
| HIF1A | hypoxia inducible factor 1 alpha subunit | **0,888** | 3,40E-05 | 551,298 | 300,031 |
| C17orf67 | chromosome 17 open reading frame 67 | **0,888** | 0,194287 | 1,994 | 1,469 |
| CAND2 | cullin associated and neddylation dissociated 2 (putative) | **0,888** | 0,220958 | 1,606 | 1,852 |
| THY1 | Thy-1 cell surface antigen | **0,888** | 0,11648 | 2,714 | 2,171 |
| FAM225A | family with sequence similarity 225 member A (non-protein coding) | **0,888** | 0,161144 | 2,160 | 1,852 |
| UBA5 | ubiquitin like modifier activating enzyme 5 | **0,888** | 0,000983 | 23,818 | 21,581 |
| TSPAN13 | tetraspanin 13 | **0,888** | 0,035306 | 13,404 | 4,214 |
| SRBD1 | S1 RNA binding domain 1 | **0,888** | 0,002974 | 12,629 | 14,239 |
| RTTN | rotatin | **0,888** | 0,007954 | 12,020 | 12,068 |
| ARF4 | ADP ribosylation factor 4 | **0,888** | 0,000143 | 232,528 | 177,694 |
| CCDC120 | coiled-coil domain containing 120 | **0,888** | 0,122449 | 3,545 | 1,788 |
| STK11 | serine/threonine kinase 11 | **0,888** | 5,40E-05 | 42,650 | 34,798 |
| CAVIN3 | caveolae associated protein 3 | **0,888** | 0,003211 | 46,915 | 28,030 |
| HDDC3 | HD domain containing 3 | **0,888** | 0,019818 | 7,478 | 7,662 |
| PFN1 | profilin 1 | **0,888** | 3,40E-05 | 281,714 | 201,893 |
| KCNQ3 | potassium voltage-gated channel subfamily Q member 3 | **0,888** | 0,102288 | 4,099 | 3,448 |
| DHX29 | DExH-box helicase 29 | **0,887** | 0,000848 | 28,969 | 26,306 |
| C1orf53 | chromosome 1 open reading frame 53 | **0,887** | 0,382628 | 0,388 | 0,638 |
| SHKBP1 | SH3KBP1 binding protein 1 | **0,887** | 0,001136 | 30,631 | 15,452 |
| ENKD1 | enkurin domain containing 1 | **0,887** | 0,002681 | 12,906 | 11,046 |
| SIL1 | SIL1 nucleotide exchange factor | **0,887** | 0,00521 | 60,541 | 37,608 |
| CCDC71L | coiled-coil domain containing 71 like | **0,887** | 0,000136 | 58,991 | 47,377 |
| SELENOH | selenoprotein H | **0,887** | 0,013093 | 11,743 | 9,961 |
| ESAM | endothelial cell adhesion molecule | **0,887** | 0,365261 | 0,609 | 0,638 |

| | | | | | |
|---|---|---|---|---|---|
| ATAD5 | ATPase family, AAA domain containing 5 | **0,887** | 0,24332 | 1,662 | 1,724 |
| POMT1 | protein O-mannosyltransferase 1 | **0,887** | 0,000579 | 20,328 | 12,323 |
| HDAC8 | histone deacetylase 8 | **0,887** | 0,009919 | 9,029 | 8,109 |
| SNRPD2 | small nuclear ribonucleoprotein D2 polypeptide | **0,887** | 0,001714 | 51,513 | 44,631 |
| SMUG1 | single-strand-selective monofunctional uracil-DNA glycosylase 1 | **0,887** | 0,000248 | 18,390 | 13,728 |
| TRPT1 | tRNA phosphotransferase 1 | **0,887** | 0,005512 | 12,241 | 10,280 |
| LSS | lanosterol synthase | **0,887** | 0,04558 | 8,475 | 4,342 |
| CCDC24 | coiled-coil domain containing 24 | **0,887** | 0,006711 | 7,312 | 5,874 |
| OSTF1 | osteoclast stimulating factor 1 | **0,887** | 0,000174 | 32,292 | 21,007 |
| TMEM9 | transmembrane protein 9 | **0,887** | 0,000144 | 29,080 | 10,727 |
| YIPF1 | Yip1 domain family member 1 | **0,887** | 0,00098 | 21,104 | 15,643 |
| HMGB3 | high mobility group box 3 | **0,887** | 0,022166 | 8,862 | 3,895 |
| ANKRD13C | ankyrin repeat domain 13C | **0,887** | 0,001479 | 23,042 | 17,048 |
| PRRG1 | proline rich and Gla domain 1 | **0,887** | 0,022011 | 6,591 | 4,150 |
| PCGF6 | polycomb group ring finger 6 | **0,887** | 0,09407 | 3,711 | 3,703 |
| TSSC1 | tumor suppressing subtransferable candidate 1 | **0,887** | 0,003479 | 11,244 | 8,620 |
| RPL7AP50 | ribosomal protein L7a pseudogene 50 | **0,887** | 0,462226 | 0,775 | 0,128 |
| AURKAIP1 | aurora kinase A interacting protein 1 | **0,887** | 0,000369 | 43,038 | 36,969 |
| TAOK2 | TAO kinase 2 | **0,887** | 3,50E-05 | 79,042 | 65,829 |
| PSTPIP2 | proline-serine-threonine phosphatase interacting protein 2 | **0,887** | 0,007007 | 8,696 | 14,239 |
| EPM2A | EPM2A, laforin glucan phosphatase | **0,887** | 0,04483 | 4,542 | 3,767 |
| FGFBP2 | fibroblast growth factor binding protein 2 | **0,887** | 5,90E-05 | 1284,996 | 586,972 |
| ZNF775 | zinc finger protein 775 | **0,887** | 0,184194 | 3,213 | 1,852 |
| CCBE1 | collagen and calcium binding EGF domains 1 | **0,887** | 0,438419 | 0,332 | 0,319 |
| DTWD1 | DTW domain containing 1 | **0,887** | 0,002175 | 17,725 | 13,472 |
| POLRMT | RNA polymerase mitochondrial | **0,887** | 0,000276 | 26,144 | 26,306 |
| PORCN | porcupine homolog (Drosophila) | **0,887** | 0,033572 | 6,148 | 3,959 |
| DNMT1 | DNA methyltransferase 1 | **0,887** | 0,000346 | 38,718 | 34,096 |
| PFDN4 | prefoldin subunit 4 | **0,887** | 0,01319 | 13,238 | 11,238 |
| PPP5C | protein phosphatase 5 catalytic subunit | **0,887** | 1,20E-05 | 29,080 | 23,114 |
| SSU72 | SSU72 homolog, RNA polymerase II CTD phosphatase | **0,886** | 3,50E-05 | 60,209 | 51,527 |
| NID2 | nidogen 2 | **0,886** | 0,207817 | 2,770 | 1,788 |
| SLC35G1 | solute carrier family 35 member G1 | **0,886** | 0,148748 | 1,883 | 2,235 |

| | | | | | |
|---|---|---|---|---|---|
| HTRA2 | HtrA serine peptidase 2 | **0,886** | 0,000464 | 24,649 | 23,369 |
| SNX5 | sorting nexin 5 | **0,886** | 4,00E-06 | 28,415 | 25,604 |
| FBXO18 | F-box protein, helicase, 18 | **0,886** | 6,00E-06 | 52,898 | 50,378 |
| PFDN2 | prefoldin subunit 2 | **0,886** | 0,001418 | 23,929 | 26,625 |
| DIMT1 | DIM1 dimethyladenosine transferase 1 homolog | **0,886** | 0,007481 | 8,475 | 10,471 |
| ZNF219 | zinc finger protein 219 | **0,886** | 0,011532 | 11,521 | 7,790 |
| MLF2 | myeloid leukemia factor 2 | **0,886** | 1,00E-06 | 61,705 | 48,143 |
| NDUFB7 | NADH:ubiquinone oxidoreductase subunit B7 | **0,886** | 0,007958 | 31,462 | 24,646 |
| GJA1 | gap junction protein alpha 1 | **0,886** | 0,000251 | 126,622 | 103,948 |
| BANF1 | barrier to autointegration factor 1 | **0,886** | 0,000106 | 38,662 | 32,308 |
| CCR10 | C-C motif chemokine receptor 10 | **0,886** | 0,375672 | 0,831 | 1,596 |
| LDHAP7 | lactate dehydrogenase A pseudogene 7 | **0,886** | 0,266961 | 0,277 | 0,958 |
| SUCLG2 | succinate-CoA ligase GDP-forming beta subunit | **0,886** | 0,001366 | 16,562 | 16,282 |
| PIGP | phosphatidylinositol glycan anchor biosynthesis class P | **0,886** | 0,002205 | 17,171 | 14,749 |
| RPL17P50 | ribosomal protein L17 pseudogene 50 | **0,886** | 0,403943 | 0,554 | 0,319 |
| DIAPH3 | diaphanous related formin 3 | **0,886** | 0,347733 | 5,761 | 0,255 |
| ERGIC1 | endoplasmic reticulum-golgi intermediate compartment 1 | **0,886** | 6,00E-06 | 94,773 | 58,614 |
| SEC61G | Sec61 translocon gamma subunit | **0,886** | 0,000674 | 86,464 | 84,856 |
| BCL7C | BCL tumor suppressor 7C | **0,886** | 0,006816 | 20,439 | 14,749 |
| NTPCR | nucleoside-triphosphatase, cancer-related | **0,885** | 0,001783 | 15,066 | 17,623 |
| SNX21 | sorting nexin family member 21 | **0,885** | 0,002255 | 19,830 | 16,473 |
| OBSCN | obscurin, cytoskeletal calmodulin and titin-interacting RhoGEF | **0,885** | 0,137235 | 3,600 | 2,362 |
| CHIC2 | cysteine rich hydrophobic domain 2 | **0,885** | 0,00126 | 14,900 | 16,856 |
| TPH1 | tryptophan hydroxylase 1 | **0,885** | 0,476176 | 0,166 | 0,894 |
| SUGCT | succinyl-CoA:glutarate-CoA transferase | **0,885** | 0,52137 | 0,443 | 0,575 |
| TPGS1 | tubulin polyglutamylase complex subunit 1 | **0,885** | 0,007696 | 8,641 | 6,768 |
| MMACHC | methylmalonic aciduria (cobalamin deficiency) cblC type, with homocystinuria | **0,885** | 0,046457 | 3,490 | 4,406 |
| WDR74 | WD repeat domain 74 | **0,885** | 0,000722 | 16,119 | 17,559 |
| ATIC | 5-aminoimidazole-4-carboxamide ribonucleotide formyltransferase/IMP cyclohydrolase | **0,885** | 4,20E-05 | 25,147 | 25,476 |
| ITGAE | integrin subunit alpha E | **0,885** | 0,024651 | 5,207 | 4,789 |
| ABCC4 | ATP binding cassette subfamily C member 4 | **0,885** | 0,007122 | 18,722 | 8,045 |
| OIP5 | Opa interacting protein 5 | **0,885** | 0,531271 | 0,609 | 0,383 |
| UTP14A | UTP14A small subunit processome component | **0,885** | 0,000998 | 13,515 | 15,388 |

| | | | | | |
|---|---|---|---|---|---|
| PSMD13 | proteasome 26S subunit, non-ATPase 13 | **0,885** | 9,70E-05 | 82,974 | 71,512 |
| CH25H | cholesterol 25-hydroxylase | **0,885** | 0,153159 | 2,936 | 1,213 |
| MCEE | methylmalonyl-CoA epimerase | **0,885** | 0,012051 | 6,702 | 8,045 |
| FAM3C2 | family with sequence similarity 3 member C2 (pseudogene) | **0,885** | 0,110074 | 4,819 | 2,937 |
| PGAM1P8 | phosphoglycerate mutase 1 pseudogene 8 | **0,885** | 0,54124 | 0,277 | 0,383 |
| QSOX1 | quiescin sulfhydryl oxidase 1 | **0,885** | 9,00E-06 | 720,460 | 552,940 |
| DGCR11 | DiGeorge syndrome critical region gene 11 (non-protein coding) | **0,885** | 0,361258 | 0,886 | 0,894 |
| DSN1 | DSN1 homolog, MIS12 kinetochore complex component | **0,885** | 0,051225 | 4,764 | 2,809 |
| KARS | lysyl-tRNA synthetase | **0,885** | 1,20E-05 | 63,920 | 54,975 |
| ZNF346 | zinc finger protein 346 | **0,885** | 0,004183 | 7,533 | 7,726 |
| DNAAF1 | dynein axonemal assembly factor 1 | **0,885** | 0,428003 | 0,609 | 0,830 |
| JMJD4 | jumonji domain containing 4 | **0,884** | 0,005435 | 16,783 | 14,047 |
| SND1 | staphylococcal nuclease and tudor domain containing 1 | **0,884** | 6,40E-05 | 189,656 | 158,986 |
| PMS2P1 | PMS1 homolog 2, mismatch repair system component pseudogene 1 | **0,884** | 0,147366 | 3,379 | 1,788 |
| HDGF | heparin binding growth factor | **0,884** | 5,00E-05 | 293,956 | 245,311 |
| LRFN3 | leucine rich repeat and fibronectin type III domain containing 3 | **0,884** | 0,026555 | 13,072 | 7,023 |
| ARNT2 | aryl hydrocarbon receptor nuclear translocator 2 | **0,884** | 0,166014 | 1,883 | 1,532 |
| RELB | RELB proto-oncogene, NF-kB subunit | **0,884** | 3,50E-05 | 112,830 | 91,497 |
| CCDC88B | coiled-coil domain containing 88B | **0,884** | 0,396737 | 0,499 | 0,319 |
| STMN3 | stathmin 3 | **0,884** | 0,025665 | 1,717 | 4,406 |
| GLI4 | GLI family zinc finger 4 | **0,884** | 0,041511 | 4,764 | 4,469 |
| OAZ3 | ornithine decarboxylase antizyme 3 | **0,884** | 0,447129 | 0,720 | 0,575 |
| STT3A | STT3A, catalytic subunit of the oligosaccharyltransferase complex | **0,884** | 0,000103 | 278,834 | 196,466 |
| FAM110A | family with sequence similarity 110 member A | **0,884** | 0,016679 | 6,702 | 5,300 |
| ZFAND2B | zinc finger AN1-type containing 2B | **0,884** | 0,000372 | 13,571 | 9,641 |
| LARS | leucyl-tRNA synthetase | **0,884** | 7,00E-06 | 98,761 | 94,753 |
| BOC | BOC cell adhesion associated, oncogene regulated | **0,884** | 0,000639 | 35,173 | 23,050 |
| CPA4 | carboxypeptidase A4 | **0,884** | 0,008985 | 75,995 | 85,559 |
| RPTOR | regulatory associated protein of MTOR complex 1 | **0,884** | 0,002234 | 29,246 | 29,818 |
| SMURF2P1 | SMAD specific E3 ubiquitin protein ligase 2 pseudogene 1 | **0,884** | 0,326851 | 1,052 | 1,022 |
| AMOTL1 | angiomotin like 1 | **0,884** | 0,000821 | 63,422 | 56,763 |
| NAT10 | N-acetyltransferase 10 | **0,884** | 7,00E-06 | 27,861 | 27,966 |
| LINC00884 | long intergenic non-protein coding RNA 884 | **0,884** | 0,139069 | 1,440 | 1,469 |

| | | | | | |
|---|---|---|---|---|---|
| MYCBP | MYC binding protein | **0,883** | 0,019497 | 6,038 | 5,683 |
| EVC2 | EvC ciliary complex subunit 2 | **0,883** | 0,003701 | 11,244 | 9,897 |
| TMEM263 | transmembrane protein 263 | **0,883** | 3,00E-06 | 96,822 | 67,106 |
| PPIA | peptidylprolyl isomerase A | **0,883** | 1,50E-05 | 154,539 | 99,989 |
| LTV1 | LTV1 ribosome biogenesis factor | **0,883** | 0,002285 | 8,530 | 8,237 |
| MEIS3P1 | Meis homeobox 3 pseudogene 1 | **0,883** | 0,141965 | 2,382 | 2,107 |
| BBIP1 | BBSome interacting protein 1 | **0,883** | 0,000204 | 28,581 | 24,327 |
| DLD | dihydrolipoamide dehydrogenase | **0,883** | 6,50E-05 | 40,379 | 32,627 |
| RTN2 | reticulon 2 | **0,883** | 0,255592 | 1,939 | 0,766 |
| FAAP100 | Fanconi anemia core complex associated protein 100 | **0,883** | 0,006677 | 15,509 | 14,494 |
| MFSD4B | major facilitator superfamily domain containing 4B | **0,883** | 0,058275 | 2,825 | 5,810 |
| SMKR1 | small lysine rich protein 1 | **0,883** | 0,448057 | 0,554 | 0,383 |
| TRIP10 | thyroid hormone receptor interactor 10 | **0,883** | 0,000226 | 24,926 | 19,027 |
| RRM1 | ribonucleotide reductase catalytic subunit M1 | **0,883** | 0,000304 | 25,867 | 15,260 |
| HAUS1 | HAUS augmin like complex subunit 1 | **0,883** | 0,046944 | 5,761 | 4,023 |
| FGFBP3 | fibroblast growth factor binding protein 3 | **0,883** | 0,234571 | 1,274 | 1,149 |
| CBWD1 | COBW domain containing 1 | **0,883** | 0,026603 | 4,265 | 2,426 |
| GATA6 | GATA binding protein 6 | **0,883** | 0,056432 | 2,714 | 2,873 |
| NAT6 | N-acetyltransferase 6 | **0,883** | 0,07177 | 2,880 | 1,915 |
| MTIF3 | mitochondrial translational initiation factor 3 | **0,883** | 0,008656 | 10,580 | 9,450 |
| ME1 | malic enzyme 1 | **0,883** | 0,011592 | 20,162 | 9,833 |
| TALDO1 | transaldolase 1 | **0,883** | 1,10E-05 | 190,376 | 107,970 |
| HENMT1 | HEN1 methyltransferase homolog 1 | **0,882** | 0,018843 | 5,927 | 5,555 |
| INPP1 | inositol polyphosphate-1-phosphatase | **0,882** | 0,000616 | 16,949 | 12,962 |
| C12orf43 | chromosome 12 open reading frame 43 | **0,882** | 0,003899 | 11,687 | 10,408 |
| EMG1 | EMG1, N1-specific pseudouridine methyltransferase | **0,882** | 0,009438 | 10,801 | 9,386 |
| COLGALT1 | collagen beta(1-O)galactosyltransferase 1 | **0,882** | 2,10E-05 | 100,810 | 78,025 |
| MCMDC2 | minichromosome maintenance domain containing 2 | **0,882** | 0,466834 | 0,665 | 0,511 |
| SNRNP35 | small nuclear ribonucleoprotein U11/U12 subunit 35 | **0,882** | 0,000509 | 12,961 | 9,131 |
| SHISA5 | shisa family member 5 | **0,882** | 1,00E-06 | 93,942 | 81,409 |
| DHX30 | DExH-box helicase 30 | **0,882** | 7,00E-06 | 59,157 | 56,890 |
| COX18 | COX18, cytochrome c oxidase assembly factor | **0,882** | 0,007844 | 6,702 | 9,003 |
| DPY19L2P2 | DPY19L2 pseudogene 2 | **0,882** | 0,247232 | 1,385 | 0,958 |

| | | | | | |
|---|---|---|---|---|---|
| PWP1 | PWP1 homolog, endonuclein | **0,882** | 3,50E-05 | 28,692 | 18,453 |
| DPP3 | dipeptidyl peptidase 3 | **0,882** | 0,000852 | 28,471 | 22,284 |
| NQO1 | NAD(P)H quinone dehydrogenase 1 | **0,882** | 1,00E-05 | 96,656 | 33,777 |
| PRDM11 | PR/SET domain 11 | **0,882** | 0,008659 | 8,530 | 8,492 |
| FLAD1 | flavin adenine dinucleotide synthetase 1 | **0,882** | 0,018743 | 10,524 | 9,641 |
| GNG12-AS1 | GNG12 antisense RNA 1 | **0,882** | 0,519479 | 0,720 | 0,255 |
| SLC16A13 | solute carrier family 16 member 13 | **0,882** | 0,162956 | 2,770 | 1,660 |
| CHD4 | chromodomain helicase DNA binding protein 4 | **0,882** | 0,000215 | 79,208 | 54,464 |
| NSMCE1 | NSE1 homolog, SMC5-SMC6 complex component | **0,882** | 0,001488 | 15,952 | 15,452 |
| HCFC1R1 | host cell factor C1 regulator 1 | **0,882** | 0,00307 | 7,422 | 6,385 |
| DPYSL2 | dihydropyrimidinase like 2 | **0,882** | 1,00E-05 | 49,186 | 38,693 |
| ADCK1 | aarF domain containing kinase 1 | **0,882** | 0,133055 | 2,382 | 2,362 |
| ANKHD1 | ankyrin repeat and KH domain containing 1 | **0,882** | 0,276714 | 1,496 | 1,852 |
| MXRA7 | matrix remodeling associated 7 | **0,882** | 3,60E-05 | 129,281 | 33,074 |
| ATP5A1 | ATP synthase, H+ transporting, mitochondrial F1 complex, alpha subunit 1, cardiac muscle | **0,882** | 5,00E-05 | 132,604 | 91,688 |
| SPDL1 | spindle apparatus coiled-coil protein 1 | **0,882** | 0,022304 | 5,484 | 5,683 |
| SNHG11 | small nucleolar RNA host gene 11 | **0,882** | 0,009177 | 5,982 | 6,513 |
| TPRA1 | transmembrane protein adipocyte associated 1 | **0,881** | 3,20E-05 | 46,749 | 39,587 |
| PRICKLE3 | prickle planar cell polarity protein 3 | **0,881** | 0,017452 | 6,093 | 3,831 |
| SRRT | serrate, RNA effector molecule | **0,881** | 3,20E-05 | 46,749 | 48,398 |
| BET1L | Bet1 golgi vesicular membrane trafficking protein like | **0,881** | 1,00E-05 | 54,670 | 44,056 |
| SERPINB6 | serpin family B member 6 | **0,881** | 1,40E-05 | 51,125 | 41,630 |
| PKN1 | protein kinase N1 | **0,881** | 0,002709 | 72,229 | 50,569 |
| RAD23A | RAD23 homolog A, nucleotide excision repair protein | **0,881** | 3,90E-05 | 92,668 | 80,259 |
| ELP6 | elongator acetyltransferase complex subunit 6 | **0,881** | 0,001541 | 16,894 | 11,365 |
| GPT | glutamic--pyruvic transaminase | **0,881** | 0,495406 | 0,332 | 0,383 |
| ALDH5A1 | aldehyde dehydrogenase 5 family member A1 | **0,881** | 0,108476 | 2,548 | 2,235 |
| ADPRHL1 | ADP-ribosylhydrolase like 1 | **0,881** | 0,001326 | 9,029 | 8,747 |
| ELOVL5 | ELOVL fatty acid elongase 5 | **0,881** | 1,10E-05 | 59,212 | 46,163 |
| GGCT | gamma-glutamylcyclotransferase | **0,881** | 0,082432 | 3,877 | 3,448 |
| ZFP2 | ZFP2 zinc finger protein | **0,881** | 0,39702 | 0,665 | 0,958 |
| PGAM5 | PGAM family member 5, mitochondrial serine/threonine protein phosphatase | **0,881** | 0,000245 | 12,297 | 14,494 |
| RCOR2 | REST corepressor 2 | **0,881** | 0,497891 | 0,388 | 0,638 |

| | | | | | |
|---|---|---|---|---|---|
| C11orf74 | chromosome 11 open reading frame 74 | **0,881** | 0,023844 | 5,871 | 5,172 |
| EXTL1 | exostosin like glycosyltransferase 1 | **0,881** | 0,050452 | 3,988 | 2,490 |
| FAM57A | family with sequence similarity 57 member A | **0,881** | 0,000249 | 17,282 | 11,621 |
| ZNF891 | zinc finger protein 891 | **0,881** | 0,193119 | 2,493 | 2,682 |
| GJA3 | gap junction protein alpha 3 | **0,881** | 0,268589 | 1,939 | 1,085 |
| C20orf196 | chromosome 20 open reading frame 196 | **0,881** | 0,178264 | 2,160 | 1,852 |
| CORO1C | coronin 1C | **0,881** | 1,00E-05 | 66,025 | 48,590 |
| MEIS1 | Meis homeobox 1 | **0,881** | 0,047876 | 5,041 | 7,534 |
| TBC1D25 | TBC1 domain family member 25 | **0,881** | 0,002179 | 16,839 | 9,705 |
| GRIK2 | glutamate ionotropic receptor kainate type subunit 2 | **0,880** | 0,489885 | 0,942 | 0,447 |
| GLB1L3 | galactosidase beta 1 like 3 | **0,880** | 0,019774 | 7,865 | 1,852 |
| RIC8A | RIC8 guanine nucleotide exchange factor A | **0,880** | 5,00E-06 | 74,943 | 55,932 |
| RIMS2 | regulating synaptic membrane exocytosis 2 | **0,880** | 0,546897 | 0,665 | 0,383 |
| SMO | smoothened, frizzled class receptor | **0,880** | 0,002837 | 23,430 | 21,390 |
| CENPS | centromere protein S | **0,880** | 0,083697 | 2,603 | 2,426 |
| NT5DC2 | 5'-nucleotidase domain containing 2 | **0,880** | 0,002839 | 19,719 | 10,854 |
| CENPM | centromere protein M | **0,880** | 0,269023 | 1,828 | 0,766 |
| ICAM2 | intercellular adhesion molecule 2 | **0,880** | 1,50E-05 | 11,078 | 17,942 |
| ISY1 | ISY1 splicing factor homolog | **0,880** | 0,002541 | 9,749 | 10,854 |
| FXN | frataxin | **0,880** | 0,099589 | 2,437 | 3,001 |
| VIM | vimentin | **0,880** | 1,70E-05 | 2151,465 | 803,615 |
| FMC1 | formation of mitochondrial complex V assembly factor 1 homolog | **0,880** | 0,316122 | 0,997 | 1,469 |
| TNFSF12 | TNF superfamily member 12 | **0,880** | 0,146878 | 2,880 | 1,852 |
| SPATA6 | spermatogenesis associated 6 | **0,880** | 0,035708 | 4,376 | 2,490 |
| C9orf170 | chromosome 9 open reading frame 170 | **0,880** | 0,288245 | 0,720 | 1,213 |
| RABEP2 | rabaptin, RAB GTPase binding effector protein 2 | **0,880** | 0,000228 | 15,288 | 14,941 |
| CRLS1 | cardiolipin synthase 1 | **0,880** | 0,000159 | 30,188 | 24,710 |
| MED16 | mediator complex subunit 16 | **0,880** | 1,90E-05 | 26,864 | 21,007 |
| TRMT12 | tRNA methyltransferase 12 homolog | **0,880** | 0,012885 | 7,422 | 6,130 |
| CNPY2 | canopy FGF signaling regulator 2 | **0,880** | 0,001893 | 10,524 | 6,832 |
| TMEM27 | transmembrane protein 27 | **0,880** | 0,223081 | 0,831 | 1,277 |
| COPB1 | coatomer protein complex subunit beta 1 | **0,880** | 1,00E-06 | 130,056 | 91,497 |
| SEC24D | SEC24 homolog D, COPII coat complex component | **0,880** | 0,001304 | 136,869 | 90,922 |

| | | | | | |
|---|---|---|---|---|---|
| TMEM219 | transmembrane protein 219 | **0,880** | 0,00017 | 24,981 | 18,389 |
| NDUFAF2 | NADH:ubiquinone oxidoreductase complex assembly factor 2 | **0,880** | 0,0046 | 11,189 | 10,152 |
| RRP9 | ribosomal RNA processing 9, small subunit (SSU) processome component, homolog (yeast | **0,880** | 0,002015 | 14,678 | 13,983 |
| CARS | cysteinyl-tRNA synthetase | **0,880** | 0,094227 | 3,323 | 1,724 |
| NUSAP1 | nucleolar and spindle associated protein 1 | **0,880** | 0,070474 | 9,804 | 1,405 |
| SPX | spexin hormone | **0,880** | 0,006196 | 18,390 | 16,793 |
| RARS | arginyl-tRNA synthetase | **0,880** | 4,30E-05 | 57,052 | 43,290 |
| LCN2 | lipocalin 2 | **0,880** | 9,00E-06 | 1283,501 | 1748,848 |
| ZBTB7B | zinc finger and BTB domain containing 7B | **0,879** | 4,60E-05 | 31,185 | 20,496 |
| ITFG2 | integrin alpha FG-GAP repeat containing 2 | **0,879** | 0,001499 | 11,964 | 11,301 |
| RCCD1 | RCC1 domain containing 1 | **0,879** | 0,034541 | 5,373 | 3,639 |
| TMEM86B | transmembrane protein 86B | **0,879** | 0,475759 | 0,443 | 0,319 |
| PPIL1 | peptidylprolyl isomerase like 1 | **0,879** | 8,40E-05 | 22,987 | 22,986 |
| HCFC1 | host cell factor C1 | **0,879** | 9,50E-05 | 61,815 | 47,696 |
| BASP1 | brain abundant membrane attached signal protein 1 | **0,879** | 0,002481 | 5,428 | 11,174 |
| PKM | pyruvate kinase, muscle | **0,879** | 4,00E-06 | 1455,598 | 912,989 |
| C16orf95 | chromosome 16 open reading frame 95 | **0,879** | 0,52144 | 0,886 | 0,511 |
| TXNRD3 | thioredoxin reductase 3 | **0,879** | 0,068375 | 3,767 | 2,746 |
| ZNF708 | zinc finger protein 708 | **0,879** | 0,062468 | 2,825 | 3,576 |
| HEATR3 | HEAT repeat containing 3 | **0,879** | 0,037672 | 3,545 | 3,320 |
| CYP24A1 | cytochrome P450 family 24 subfamily A member 1 | **0,879** | 0,123768 | 2,880 | 2,490 |
| PGAP2 | post-GPI attachment to proteins 2 | **0,879** | 0,003602 | 9,250 | 10,791 |
| SLC35B4 | solute carrier family 35 member B4 | **0,879** | 0,000889 | 22,655 | 17,303 |
| BOLA3 | bolA family member 3 | **0,879** | 0,015309 | 9,306 | 10,791 |
| PNKD | paroxysmal nonkinesigenic dyskinesia | **0,879** | 0,001276 | 15,897 | 15,196 |
| PMVK | phosphomevalonate kinase | **0,879** | 0,002161 | 20,162 | 18,133 |
| SOCS5 | suppressor of cytokine signaling 5 | **0,879** | 2,10E-05 | 63,809 | 47,696 |
| KLHL17 | kelch like family member 17 | **0,879** | 0,004047 | 7,755 | 8,684 |
| PHLDB3 | pleckstrin homology like domain family B member 3 | **0,879** | 0,073339 | 2,659 | 3,129 |
| CREB3L4 | cAMP responsive element binding protein 3 like 4 | **0,879** | 0,007554 | 5,373 | 5,044 |
| PLLP | plasmolipin | **0,879** | 0,254712 | 1,551 | 0,766 |
| CASKIN2 | CASK interacting protein 2 | **0,878** | 0,004384 | 14,512 | 10,663 |
| ARHGEF25 | Rho guanine nucleotide exchange factor 25 | **0,878** | 0,197394 | 1,994 | 0,894 |

| | | | | | |
|---|---|---|---|---|---|
| VWA1 | von Willebrand factor A domain containing 1 | **0,878** | 0,000259 | 36,225 | 25,285 |
| ST3GAL4 | ST3 beta-galactoside alpha-2,3-sialyltransferase 4 | **0,878** | 0,00543 | 12,463 | 8,109 |
| ALKBH4 | alkB homolog 4, lysine demethylase | **0,878** | 0,009117 | 8,032 | 6,768 |
| TRAP1 | TNF receptor associated protein 1 | **0,878** | 0,000147 | 29,468 | 29,499 |
| STAMBP | STAM binding protein | **0,878** | 0,000366 | 21,048 | 18,197 |
| CELSR3 | cadherin EGF LAG seven-pass G-type receptor 3 | **0,878** | 0,193328 | 1,717 | 1,596 |
| FAM98C | family with sequence similarity 98 member C | **0,878** | 0,007315 | 6,647 | 7,279 |
| ERCC1 | ERCC excision repair 1, endonuclease non-catalytic subunit | **0,878** | 1,00E-05 | 42,761 | 42,652 |
| SMYD2 | SET and MYND domain containing 2 | **0,878** | 0,006517 | 8,641 | 4,916 |
| FBXL19-AS1 | FBXL19 antisense RNA 1 (head to head) | **0,878** | 0,064804 | 4,210 | 4,533 |
| BZW1P2 | basic leucine zipper and W2 domains 1 pseudogene 2 | **0,878** | 0,363362 | 0,886 | 0,383 |
| BARX1 | BARX homeobox 1 | **0,878** | 9,40E-05 | 26,200 | 14,813 |
| FNDC4 | fibronectin type III domain containing 4 | **0,878** | 0,005973 | 8,862 | 4,597 |
| STOML2 | stomatin like 2 | **0,878** | 1,00E-04 | 33,123 | 29,052 |
| MOCS2 | molybdenum cofactor synthesis 2 | **0,878** | 0,000266 | 18,334 | 14,239 |
| TRAF7 | TNF receptor associated factor 7 | **0,878** | 0,00013 | 35,837 | 27,775 |
| MRPL20 | mitochondrial ribosomal protein L20 | **0,878** | 7,70E-05 | 28,194 | 27,902 |
| IL17RC | interleukin 17 receptor C | **0,878** | 0,000523 | 27,806 | 23,178 |
| OGFOD2 | 2-oxoglutarate and iron dependent oxygenase domain containing 2 | **0,878** | 0,142709 | 1,274 | 1,469 |
| ABHD17A | abhydrolase domain containing 17A | **0,878** | 0,001883 | 18,057 | 13,536 |
| ORAI1 | ORAI calcium release-activated calcium modulator 1 | **0,878** | 0,435026 | 0,886 | 0,319 |
| QRSL1 | glutaminyl-tRNA synthase (glutamine-hydrolyzing)-like 1 | **0,878** | 0,004936 | 9,859 | 8,747 |
| MSS51 | MSS51 mitochondrial translational activator | **0,878** | 0,258373 | 0,831 | 0,702 |
| TMEM14B | transmembrane protein 14B | **0,878** | 0,006072 | 14,734 | 12,770 |
| PES1 | pescadillo ribosomal biogenesis factor 1 | **0,878** | 0,000156 | 51,568 | 59,508 |
| TSR3 | TSR3, acp transferase ribosome maturation factor | **0,878** | 9,30E-05 | 41,432 | 27,455 |
| TMEM144 | transmembrane protein 144 | **0,878** | 0,326612 | 0,388 | 1,022 |
| RPS26 | ribosomal protein S26 | **0,878** | 0,000121 | 56,664 | 27,583 |
| MRPL33 | mitochondrial ribosomal protein L33 | **0,878** | 0,00023 | 21,768 | 18,580 |
| METTL2A | methyltransferase like 2A | **0,878** | 0,001379 | 10,358 | 9,577 |
| GLIS1 | GLIS family zinc finger 1 | **0,877** | 0,367713 | 0,942 | 0,766 |
| CC2D1A | coiled-coil and C2 domain containing 1A | **0,877** | 0,006003 | 19,664 | 15,196 |
| PDK2 | pyruvate dehydrogenase kinase 2 | **0,877** | 0,001767 | 12,241 | 8,556 |

| | | | | | |
|---|---|---|---|---|---|
| KIAA2022 | KIAA2022 | **0,877** | 0,258315 | 1,052 | 0,830 |
| BMI1 | BMI1 proto-oncogene, polycomb ring finger | **0,877** | 3,00E-05 | 42,263 | 33,649 |
| PCDHB5 | protocadherin beta 5 | **0,877** | 0,123049 | 2,382 | 2,554 |
| HS3ST3A1 | heparan sulfate-glucosamine 3-sulfotransferase 3A1 | **0,877** | 0,001164 | 18,500 | 10,152 |
| ERCC6 | ERCC excision repair 6, chromatin remodeling factor | **0,877** | 0,009929 | 18,445 | 12,387 |
| PLK2 | polo like kinase 2 | **0,877** | 2,80E-05 | 34,840 | 17,431 |
| PLAC9 | placenta specific 9 | **0,877** | 0,000439 | 74,112 | 34,926 |
| PXMP2 | peroxisomal membrane protein 2 | **0,877** | 0,404054 | 0,388 | 0,192 |
| LDAH | lipid droplet associated hydrolase | **0,877** | 0,003252 | 7,533 | 7,790 |
| FANCB | Fanconi anemia complementation group B | **0,877** | 0,51469 | 0,609 | 0,192 |
| LINC01184 | long intergenic non-protein coding RNA 1184 | **0,877** | 0,001414 | 11,244 | 10,344 |
| NME2 | NME/NM23 nucleoside diphosphate kinase 2 | **0,877** | 0,416921 | 0,499 | 0,447 |
| TLDC1 | TBC/LysM-associated domain containing 1 | **0,877** | 4,20E-05 | 21,048 | 13,153 |
| COG7 | component of oligomeric golgi complex 7 | **0,877** | 0,001212 | 12,241 | 11,301 |
| CDPF1 | cysteine rich DPF motif domain containing 1 | **0,877** | 0,025172 | 4,210 | 4,023 |
| PPL | periplakin | **0,877** | 0,000148 | 9,195 | 17,878 |
| PIGV | phosphatidylinositol glycan anchor biosynthesis class V | **0,877** | 0,000158 | 29,745 | 28,541 |
| CTPS2 | CTP synthase 2 | **0,877** | 0,111502 | 3,213 | 1,660 |
| MZT2A | mitotic spindle organizing protein 2A | **0,877** | 0,078385 | 7,976 | 4,150 |
| FZD8 | frizzled class receptor 8 | **0,877** | 3,40E-05 | 391,442 | 375,501 |
| SSRP1 | structure specific recognition protein 1 | **0,877** | 0 | 72,949 | 72,597 |
| TIMM44 | translocase of inner mitochondrial membrane 44 | **0,877** | 4,00E-05 | 32,182 | 24,965 |
| ILF2 | interleukin enhancer binding factor 2 | **0,877** | 4,00E-06 | 88,846 | 78,599 |
| IPO5 | importin 5 | **0,877** | 2,40E-05 | 144,347 | 123,103 |
| SLC2A12 | solute carrier family 2 member 12 | **0,876** | 0,392469 | 0,388 | 0,702 |
| NFKBID | NFKB inhibitor delta | **0,876** | 0,066547 | 2,825 | 2,746 |
| DCAF13 | DDB1 and CUL4 associated factor 13 | **0,876** | 0,000286 | 24,427 | 24,518 |
| H1FX-AS1 | H1FX antisense RNA 1 | **0,876** | 0,203999 | 2,770 | 1,852 |
| GLYCTK | glycerate kinase | **0,876** | 0,008097 | 6,979 | 6,640 |
| DLG3 | discs large MAGUK scaffold protein 3 | **0,876** | 0,101309 | 2,936 | 2,682 |
| FAM216A | family with sequence similarity 216 member A | **0,876** | 0,048937 | 3,490 | 1,852 |
| PREB | prolactin regulatory element binding | **0,876** | 3,90E-05 | 51,679 | 41,949 |
| CD8B2 | CD8b2 molecule | **0,876** | 0,091048 | 1,496 | 1,852 |

| | | | | | |
|---|---|---|---|---|---|
| RPS14P8 | ribosomal protein S14 pseudogene 8 | **0,876** | 0,282289 | 1,496 | 0,511 |
| LINC01116 | long intergenic non-protein coding RNA 1116 | **0,876** | 0,005432 | 7,090 | 6,832 |
| RBFA | ribosome binding factor A (putative) | **0,876** | 0,066481 | 4,043 | 3,831 |
| FKBP7 | FK506 binding protein 7 | **0,876** | 0,027893 | 5,927 | 3,320 |
| EIF3B | eukaryotic translation initiation factor 3 subunit B | **0,876** | 7,60E-05 | 124,185 | 108,992 |
| LMCD1-AS1 | LMCD1 antisense RNA 1 (head to head) | **0,876** | 0,469245 | 0,277 | 0,192 |
| CALU | calumenin | **0,876** | 1,00E-06 | 508,759 | 287,708 |
| MFSD11 | major facilitator superfamily domain containing 11 | **0,876** | 2,20E-05 | 20,605 | 15,643 |
| SLC50A1 | solute carrier family 50 member 1 | **0,876** | 0,001842 | 10,635 | 5,746 |
| G6PC3 | glucose-6-phosphatase catalytic subunit 3 | **0,876** | 5,60E-05 | 30,908 | 25,923 |
| GEMIN2 | gem nuclear organelle associated protein 2 | **0,876** | 0,100572 | 2,936 | 3,256 |
| CTNNAL1 | catenin alpha like 1 | **0,876** | 2,50E-05 | 28,138 | 20,240 |
| RIOX2 | ribosomal oxygenase 2 | **0,876** | 0,000101 | 21,879 | 17,814 |
| MAP1LC3A | microtubule associated protein 1 light chain 3 alpha | **0,876** | 0,041802 | 4,874 | 3,959 |
| ADAT2 | adenosine deaminase, tRNA specific 2 | **0,876** | 0,227391 | 1,606 | 1,979 |
| ZNF648 | zinc finger protein 648 | **0,876** | 0,34085 | 0,443 | 0,894 |
| CCDC13 | coiled-coil domain containing 13 | **0,876** | 0,351871 | 0,720 | 0,447 |
| PRDX5 | peroxiredoxin 5 | **0,876** | 0,000317 | 120,197 | 89,390 |
| SLC29A3 | solute carrier family 29 member 3 | **0,876** | 0,019544 | 5,871 | 4,214 |
| BTBD6 | BTB domain containing 6 | **0,876** | 4,00E-06 | 28,194 | 21,517 |
| IGFBP3 | insulin like growth factor binding protein 3 | **0,875** | 8,10E-05 | 10383,440 | 6464,226 |
| TIMM23B | translocase of inner mitochondrial membrane 23 homolog B | **0,875** | 0,132212 | 2,770 | 2,299 |
| SYBU | syntabulin | **0,875** | 0,000567 | 19,165 | 20,751 |
| KIF20A | kinesin family member 20A | **0,875** | 0,377402 | 4,597 | 0,319 |
| PYM1 | PYM homolog 1, exon junction complex associated factor | **0,875** | 3,20E-05 | 15,288 | 13,983 |
| TCTA | T-cell leukemia translocation altered | **0,875** | 1,40E-05 | 28,249 | 19,602 |
| GDF10 | growth differentiation factor 10 | **0,875** | 0,164533 | 1,606 | 2,171 |
| TMEM204 | transmembrane protein 204 | **0,875** | 8,90E-05 | 25,203 | 22,347 |
| TMEM99 | transmembrane protein 99 | **0,875** | 7,40E-05 | 14,955 | 16,409 |
| NDUFB11 | NADH:ubiquinone oxidoreductase subunit B11 | **0,875** | 0,000372 | 26,033 | 18,900 |
| TRABD | TraB domain containing | **0,875** | 0,000456 | 16,119 | 11,685 |
| DAP | death associated protein | **0,875** | 0 | 204,999 | 168,372 |
| EVA1B | eva-1 homolog B | **0,875** | 0,000218 | 53,452 | 34,734 |

| | | | | | |
|---|---|---|---|---|---|
| LPL | lipoprotein lipase | **0,875** | 0,104261 | 2,991 | 10,344 |
| LDOC1 | LDOC1, regulator of NFKB signaling | **0,875** | 0,002147 | 10,856 | 11,940 |
| JUP | junction plakoglobin | **0,875** | 0,003239 | 13,127 | 14,175 |
| HECW2 | HECT, C2 and WW domain containing E3 ubiquitin protein ligase 2 | **0,875** | 0,544859 | 0,388 | 0,638 |
| PHB | prohibitin | **0,875** | 3,00E-06 | 50,239 | 46,547 |
| TOMM22 | translocase of outer mitochondrial membrane 22 | **0,875** | 0,000599 | 26,587 | 22,731 |
| CTH | cystathionine gamma-lyase | **0,874** | 0,000421 | 12,518 | 14,558 |
| ABL1 | ABL proto-oncogene 1, non-receptor tyrosine kinase | **0,874** | 8,50E-05 | 91,782 | 103,756 |
| NR4A2 | nuclear receptor subfamily 4 group A member 2 | **0,874** | 0,000556 | 25,313 | 18,261 |
| DPM3 | dolichyl-phosphate mannosyltransferase subunit 3 | **0,874** | 0,019284 | 5,871 | 4,916 |
| RARRES2 | retinoic acid receptor responder 2 | **0,874** | 0,037194 | 5,151 | 6,768 |
| LINC01140 | long intergenic non-protein coding RNA 1140 | **0,874** | 0,316383 | 0,554 | 0,894 |
| NOL12 | nucleolar protein 12 | **0,874** | 0,0836 | 1,662 | 2,235 |
| TRMT2B | tRNA methyltransferase 2 homolog B | **0,874** | 0,006337 | 8,309 | 5,427 |
| COPE | coatomer protein complex subunit epsilon | **0,874** | 3,00E-06 | 58,603 | 47,824 |
| BID | BH3 interacting domain death agonist | **0,874** | 0,002714 | 12,407 | 8,939 |
| PTPN22 | protein tyrosine phosphatase, non-receptor type 22 | **0,874** | 0,002724 | 12,075 | 22,858 |
| SPECC1L | sperm antigen with calponin homology and coiled-coil domains 1 like | **0,874** | 0,000114 | 28,803 | 22,667 |
| PIGO | phosphatidylinositol glycan anchor biosynthesis class O | **0,874** | 3,00E-06 | 32,791 | 26,817 |
| TMEM87B | transmembrane protein 87B | **0,874** | 5,00E-05 | 19,830 | 20,304 |
| TSPYL2 | TSPY like 2 | **0,874** | 9,00E-06 | 47,636 | 60,019 |
| MECR | mitochondrial trans-2-enoyl-CoA reductase | **0,874** | 0,00942 | 7,755 | 5,555 |
| CXorf40B | chromosome X open reading frame 40B | **0,874** | 0,017455 | 9,915 | 7,407 |
| CCDC61 | coiled-coil domain containing 61 | **0,874** | 0,025523 | 5,594 | 3,512 |
| SMTN | smoothelin | **0,874** | 4,00E-06 | 225,161 | 210,002 |
| FOXF2 | forkhead box F2 | **0,874** | 0,329049 | 0,942 | 0,511 |
| ILVBL | ilvB acetolactate synthase like | **0,874** | 9,00E-06 | 38,552 | 28,605 |
| NT5C | 5', 3'-nucleotidase, cytosolic | **0,874** | 0,005565 | 9,970 | 8,045 |
| VEPH1 | ventricular zone expressed PH domain containing 1 | **0,874** | 0,282143 | 1,883 | 0,830 |
| GARS | glycyl-tRNA synthetase | **0,874** | 0 | 214,914 | 179,354 |
| RBM4B | RNA binding motif protein 4B | **0,874** | 0,000202 | 11,964 | 10,727 |
| POLB | DNA polymerase beta | **0,874** | 0,010512 | 4,708 | 4,789 |
| GATA3 | GATA binding protein 3 | **0,873** | 0,198149 | 0,332 | 0,830 |

| | | | | | |
|---|---|---|---|---|---|
| ITPA | inosine triphosphatase | **0,873** | 0,000559 | 14,124 | 11,429 |
| SYVN1 | synoviolin 1 | **0,873** | 4,90E-05 | 70,179 | 61,934 |
| UNC93B1 | unc-93 homolog B1 (C. elegans) | **0,873** | 0,002104 | 14,623 | 11,429 |
| PLD1 | phospholipase D1 | **0,873** | 2,40E-05 | 37,167 | 25,987 |
| LINC01465 | long intergenic non-protein coding RNA 1465 | **0,873** | 0,026639 | 3,822 | 6,321 |
| LINC00265 | long intergenic non-protein coding RNA 265 | **0,873** | 0,347408 | 1,108 | 0,894 |
| IFT43 | intraflagellar transport 43 | **0,873** | 4,00E-06 | 17,946 | 17,367 |
| HERC4 | HECT and RLD domain containing E3 ubiquitin protein ligase 4 | **0,873** | 3,10E-05 | 40,047 | 30,776 |
| TMEM106A | transmembrane protein 106A | **0,873** | 0,000442 | 18,223 | 10,599 |
| TNFAIP8L1 | TNF alpha induced protein 8 like 1 | **0,873** | 0,588366 | 0,388 | 0,383 |
| FOXC1 | forkhead box C1 | **0,873** | 0 | 271,412 | 271,617 |
| POLR1C | RNA polymerase I subunit C | **0,873** | 0,001476 | 14,789 | 15,643 |
| MFSD3 | major facilitator superfamily domain containing 3 | **0,873** | 0,074911 | 1,883 | 2,235 |
| PTS | 6-pyruvoyltetrahydropterin synthase | **0,872** | 0,000262 | 17,171 | 16,601 |
| PDCD6IPP2 | PDCD6IP pseudogene 2 | **0,872** | 0,290244 | 0,554 | 0,383 |
| ASPM | abnormal spindle microtubule assembly | **0,872** | 0,386667 | 7,478 | 0,064 |
| PHEX | phosphate regulating endopeptidase homolog, X-linked | **0,872** | 0,266272 | 1,606 | 1,532 |
| LINC00240 | long intergenic non-protein coding RNA 240 | **0,872** | 0,356869 | 0,388 | 0,511 |
| CITED2 | Cbp/p300 interacting transactivator with Glu/Asp rich carboxy-terminal domain 2 | **0,872** | 2,90E-05 | 219,511 | 149,409 |
| FLVCR1 | feline leukemia virus subgroup C cellular receptor 1 | **0,872** | 0,015675 | 3,711 | 5,746 |
| CHMP2A | charged multivesicular body protein 2A | **0,872** | 8,30E-05 | 65,028 | 56,188 |
| UFC1 | ubiquitin-fold modifier conjugating enzyme 1 | **0,872** | 0 | 44,201 | 32,563 |
| NEK2 | NIMA related kinase 2 | **0,872** | 0,343332 | 2,603 | 0,192 |
| ATP7B | ATPase copper transporting beta | **0,872** | 0,06369 | 3,600 | 3,065 |
| NIP7 | NIP7, nucleolar pre-rRNA processing protein | **0,872** | 2,80E-05 | 20,384 | 20,304 |
| KNOP1 | lysine rich nucleolar protein 1 | **0,872** | 0,000648 | 8,585 | 9,769 |
| TCEAL7 | transcription elongation factor A like 7 | **0,872** | 0,18377 | 1,496 | 1,213 |
| XYLB | xylulokinase | **0,872** | 0,107326 | 4,265 | 3,576 |
| ARMC5 | armadillo repeat containing 5 | **0,872** | 0,000179 | 28,581 | 23,305 |
| SPAG7 | sperm associated antigen 7 | **0,872** | 6,40E-05 | 28,803 | 19,921 |
| B3GALT6 | beta-1,3-galactosyltransferase 6 | **0,872** | 0,000139 | 41,820 | 35,756 |
| TPST1 | tyrosylprotein sulfotransferase 1 | **0,872** | 4,50E-05 | 22,987 | 16,154 |
| COL5A1 | collagen type V alpha 1 chain | **0,872** | 3,60E-05 | 142,907 | 82,877 |

| | | | | | |
|---|---|---|---|---|---|
| ENO1 | enolase 1 | **0,872** | 1,00E-06 | 1333,241 | 880,362 |
| TMEM238 | transmembrane protein 238 | **0,871** | 0,456721 | 0,222 | 0,511 |
| NXPE3 | neurexophilin and PC-esterase domain family member 3 | **0,871** | 0,004047 | 13,127 | 6,257 |
| LSM7 | LSM7 homolog, U6 small nuclear RNA and mRNA degradation associated | **0,871** | 0,000333 | 12,795 | 13,153 |
| CWF19L1 | CWF19 like 1, cell cycle control (S. pombe) | **0,871** | 0,00282 | 12,961 | 11,621 |
| PPM1G | protein phosphatase, Mg2+/Mn2+ dependent 1G | **0,871** | 1,00E-06 | 49,796 | 43,482 |
| TWF2 | twinfilin actin binding protein 2 | **0,871** | 0,004355 | 13,848 | 9,003 |
| RAB36 | RAB36, member RAS oncogene family | **0,871** | 0,005588 | 8,475 | 3,448 |
| TKT | transketolase | **0,871** | 1,10E-05 | 77,934 | 42,077 |
| FIS1 | fission, mitochondrial 1 | **0,871** | 3,10E-05 | 37,111 | 28,860 |
| EXOSC5 | exosome component 5 | **0,871** | 0,006089 | 8,696 | 12,068 |
| CEP83-AS1 | CEP83 antisense RNA 1 (head to head) | **0,871** | 0,334973 | 0,609 | 0,638 |
| ORMDL2 | ORMDL sphingolipid biosynthesis regulator 2 | **0,871** | 0,000115 | 21,547 | 17,559 |
| DIRC2 | disrupted in renal carcinoma 2 | **0,871** | 0,000939 | 12,130 | 8,556 |
| ADAMTS3 | ADAM metallopeptidase with thrombospondin type 1 motif 3 | **0,871** | 0,425388 | 1,496 | 0,638 |
| C9orf43 | chromosome 9 open reading frame 43 | **0,871** | 0,256364 | 0,609 | 0,830 |
| INHBA | inhibin beta A subunit | **0,871** | 0,001146 | 992,813 | 760,644 |
| TBL2 | transducin beta like 2 | **0,871** | 0 | 36,391 | 26,881 |
| BCS1L | BCS1 homolog, ubiquinol-cytochrome c reductase complex chaperone | **0,871** | 0,000451 | 10,801 | 10,216 |
| ZMYM3 | zinc finger MYM-type containing 3 | **0,871** | 0,012818 | 6,536 | 3,639 |
| EIF3G | eukaryotic translation initiation factor 3 subunit G | **0,871** | 5,00E-06 | 51,568 | 48,462 |
| CXorf40A | chromosome X open reading frame 40A | **0,871** | 0,010694 | 6,425 | 3,129 |
| HSF2BP | heat shock transcription factor 2 binding protein | **0,871** | 0,363635 | 0,499 | 0,702 |
| CTSH | cathepsin H | **0,871** | 0,311513 | 1,717 | 1,149 |
| IRF2BPL | interferon regulatory factor 2 binding protein like | **0,871** | 1,00E-05 | 65,028 | 45,717 |
| ZDHHC4 | zinc finger DHHC-type containing 4 | **0,871** | 2,10E-05 | 22,211 | 13,792 |
| EPHB4 | EPH receptor B4 | **0,871** | 0,010516 | 10,524 | 7,151 |
| PUS1 | pseudouridylate synthase 1 | **0,871** | 0,001792 | 9,250 | 8,428 |
| DUS2 | dihydrouridine synthase 2 | **0,871** | 0,027772 | 3,434 | 3,767 |
| LSM3 | LSM3 homolog, U6 small nuclear RNA and mRNA degradation associated | **0,871** | 0,00018 | 16,451 | 13,281 |
| ZDHHC24 | zinc finger DHHC-type containing 24 | **0,871** | 0,002184 | 9,139 | 7,790 |
| IL17D | interleukin 17D | **0,870** | 0,003462 | 8,419 | 9,322 |
| FAM180A | family with sequence similarity 180 member A | **0,870** | 0,000348 | 59,600 | 57,593 |

| | | | | | |
|---|---|---|---|---|---|
| PGM1 | phosphoglucomutase 1 | **0,870** | 4,00E-06 | 76,438 | 43,354 |
| OSTC | oligosaccharyltransferase complex non-catalytic subunit | **0,870** | 2,70E-05 | 84,359 | 55,805 |
| KCNK15 | potassium two pore domain channel subfamily K member 15 | **0,870** | 0,02839 | 4,431 | 3,703 |
| GNPNAT1 | glucosamine-phosphate N-acetyltransferase 1 | **0,870** | 2,50E-05 | 59,600 | 44,503 |
| AP5S1 | adaptor related protein complex 5 sigma 1 subunit | **0,870** | 0,000153 | 15,675 | 12,451 |
| NOP14 | NOP14 nucleolar protein | **0,870** | 2,10E-05 | 28,415 | 25,285 |
| AK3 | adenylate kinase 3 | **0,870** | 7,00E-06 | 86,963 | 73,491 |
| RPL5P12 | ribosomal protein L5 pseudogene 12 | **0,870** | 0,201161 | 0,775 | 1,149 |
| TMEM218 | transmembrane protein 218 | **0,870** | 0,003918 | 8,752 | 7,534 |
| COPA | coatomer protein complex subunit alpha | **0,870** | 1,40E-05 | 251,361 | 155,028 |
| FSTL1 | follistatin like 1 | **0,870** | 0 | 1875,677 | 690,090 |
| DCHS1 | dachsous cadherin-related 1 | **0,870** | 0,000953 | 86,630 | 72,917 |
| ZDHHC16 | zinc finger DHHC-type containing 16 | **0,870** | 4,00E-06 | 21,768 | 14,302 |
| C6orf226 | chromosome 6 open reading frame 226 | **0,870** | 0,069645 | 2,770 | 2,490 |
| CCDC107 | coiled-coil domain containing 107 | **0,870** | 1,00E-06 | 22,156 | 17,878 |
| EMILIN1 | elastin microfibril interfacer 1 | **0,870** | 8,90E-05 | 181,015 | 62,764 |
| IFT20 | intraflagellar transport 20 | **0,870** | 0,000143 | 15,454 | 11,748 |
| NABP2 | nucleic acid binding protein 2 | **0,870** | 9,00E-06 | 18,057 | 17,878 |
| ARHGAP23 | Rho GTPase activating protein 23 | **0,870** | 0,236127 | 0,831 | 0,638 |
| RGMB | repulsive guidance molecule family member b | **0,870** | 2,00E-06 | 37,887 | 26,242 |
| TMEM205 | transmembrane protein 205 | **0,870** | 4,70E-05 | 23,873 | 21,773 |
| TYMS | thymidylate synthetase | **0,870** | 0,005954 | 15,897 | 4,789 |
| PCDHB12 | protocadherin beta 12 | **0,869** | 0,302103 | 0,665 | 0,958 |
| MED8 | mediator complex subunit 8 | **0,869** | 0 | 22,932 | 18,133 |
| ADM2 | adrenomedullin 2 | **0,869** | 0,00207 | 18,002 | 9,833 |
| RGL3 | ral guanine nucleotide dissociation stimulator like 3 | **0,869** | 0,063895 | 2,548 | 2,618 |
| LY96 | lymphocyte antigen 96 | **0,869** | 0,090905 | 1,994 | 1,405 |
| C20orf24 | chromosome 20 open reading frame 24 | **0,869** | 0,001132 | 9,416 | 7,981 |
| VRK2 | vaccinia related kinase 2 | **0,869** | 0,000148 | 16,340 | 12,451 |
| MGAT1 | mannosyl (alpha-1,3-)-glycoprotein beta-1,2-N-acetylglucosaminyltransferase | **0,869** | 0 | 157,087 | 106,565 |
| DUSP4 | dual specificity phosphatase 4 | **0,869** | 0,003463 | 9,472 | 10,216 |
| RRP12 | ribosomal RNA processing 12 homolog | **0,869** | 4,00E-06 | 24,206 | 26,051 |
| TRPM8 | transient receptor potential cation channel subfamily M member 8 | **0,869** | 0,040646 | 6,591 | 1,085 |

| | | | | | |
|---|---|---|---|---|---|
| G6PD | glucose-6-phosphate dehydrogenase | **0,869** | 4,70E-05 | 35,007 | 21,198 |
| COG5 | component of oligomeric golgi complex 5 | **0,869** | 0,459355 | 0,222 | 0,447 |
| GPT2 | glutamic--pyruvic transaminase 2 | **0,869** | 2,00E-06 | 36,114 | 34,479 |
| DPCD | deleted in primary ciliary dyskinesia homolog (mouse) | **0,869** | 0,001474 | 11,023 | 9,833 |
| AC011498.1 | Hepatoma-derived growth factor-related protein 2 | **0,869** | 6,20E-05 | 28,581 | 24,135 |
| ZNF710 | zinc finger protein 710 | **0,869** | 0,003919 | 5,871 | 5,427 |
| SAMD15 | sterile alpha motif domain containing 15 | **0,868** | 0,135635 | 2,382 | 1,469 |
| NDUFS8 | NADH:ubiquinone oxidoreductase core subunit S8 | **0,868** | 0,000478 | 17,891 | 13,983 |
| FARSA | phenylalanyl-tRNA synthetase alpha subunit | **0,868** | 1,00E-05 | 36,890 | 34,224 |
| GMPPA | GDP-mannose pyrophosphorylase A | **0,868** | 8,00E-06 | 42,207 | 31,286 |
| MBD3 | methyl-CpG binding domain protein 3 | **0,868** | 4,00E-06 | 52,731 | 37,224 |
| EEF2KMT | eukaryotic elongation factor 2 lysine methyltransferase | **0,868** | 0,001103 | 5,927 | 6,002 |
| HSCB | HscB mitochondrial iron-sulfur cluster cochaperone | **0,868** | 0,00132 | 6,536 | 7,790 |
| SNHG17 | small nucleolar RNA host gene 17 | **0,868** | 0,000457 | 8,142 | 14,877 |
| LMAN1 | lectin, mannose binding 1 | **0,868** | 1,40E-05 | 221,727 | 133,702 |
| SULF1 | sulfatase 1 | **0,868** | 2,20E-05 | 217,739 | 199,659 |
| TMEM198B | transmembrane protein 198B (pseudogene) | **0,868** | 0,071313 | 2,880 | 3,129 |
| PTGFR | prostaglandin F receptor | **0,868** | 0,000224 | 17,116 | 15,005 |
| CCDC89 | coiled-coil domain containing 89 | **0,868** | 0,207452 | 1,717 | 0,575 |
| ZNF410 | zinc finger protein 410 | **0,868** | 0,474808 | 0,388 | 0,128 |
| ZNF668 | zinc finger protein 668 | **0,868** | 0,002291 | 10,801 | 8,811 |
| RPF2 | ribosome production factor 2 homolog | **0,868** | 1,00E-06 | 26,200 | 25,412 |
| BCAM | basal cell adhesion molecule (Lutheran blood group) | **0,868** | 0,338841 | 1,606 | 0,319 |
| PBK | PDZ binding kinase | **0,868** | 0,229528 | 3,711 | 0,319 |
| CD8B | CD8b molecule | **0,868** | 0,365199 | 0,886 | 0,192 |
| SPNS1 | sphingolipid transporter 1 (putative) | **0,868** | 0,363078 | 0,332 | 0,447 |
| KNSTRN | kinetochore localized astrin/SPAG5 binding protein | **0,868** | 0,037225 | 4,874 | 3,001 |
| GAR1 | GAR1 ribonucleoprotein | **0,868** | 1,00E-04 | 18,223 | 18,261 |
| ZNF74 | zinc finger protein 74 | **0,867** | 0,038168 | 3,213 | 3,192 |
| FTH1P15 | ferritin heavy chain 1 pseudogene 15 | **0,867** | 0,238283 | 1,163 | 0,447 |
| NRSN2-AS1 | NRSN2 antisense RNA 1 | **0,867** | 0,258587 | 0,886 | 0,766 |
| NAT9 | N-acetyltransferase 9 (putative) | **0,867** | 5,70E-05 | 22,322 | 23,880 |
| TRAF4 | TNF receptor associated factor 4 | **0,867** | 0,000385 | 12,020 | 10,471 |

| | | | | | |
|---|---|---|---|---|---|
| CDT1 | chromatin licensing and DNA replication factor 1 | **0,867** | 0,121549 | 2,493 | 0,447 |
| MIR3142HG | MIR3142 host gene | **0,867** | 0,059704 | 6,979 | 4,023 |
| PUM3 | pumilio RNA binding family member 3 | **0,867** | 0,00011 | 18,168 | 19,730 |
| STX10 | syntaxin 10 | **0,867** | 0,00544 | 9,361 | 6,385 |
| SNHG18 | small nucleolar RNA host gene 18 | **0,867** | 0,127241 | 1,606 | 0,894 |
| DNAJC4 | DnaJ heat shock protein family (Hsp40) member C4 | **0,867** | 0,007099 | 6,647 | 5,172 |
| NDUFV2-AS1 | NDUFV2 antisense RNA 1 | **0,867** | 0,086604 | 1,662 | 2,107 |
| TMEM101 | transmembrane protein 101 | **0,867** | 2,00E-06 | 19,996 | 13,408 |
| LINC01588 | long intergenic non-protein coding RNA 1588 | **0,867** | 0,000353 | 10,967 | 11,174 |
| LINC00526 | long intergenic non-protein coding RNA 526 | **0,867** | 0,070324 | 2,271 | 1,532 |
| BNC1 | basonuclin 1 | **0,867** | 0,377594 | 0,554 | 0,383 |
| PDGFC | platelet derived growth factor C | **0,866** | 0 | 60,818 | 43,099 |
| TSTD3 | thiosulfate sulfurtransferase (rhodanese)-like domain containing 3 | **0,866** | 0,294415 | 1,163 | 0,830 |
| PNMA8B | paraneoplastic Ma antigen family member 8B | **0,866** | 0,016566 | 2,603 | 4,980 |
| MAP10 | microtubule associated protein 10 | **0,866** | 0,072619 | 3,213 | 2,299 |
| LMF2 | lipase maturation factor 2 | **0,866** | 1,90E-05 | 73,503 | 54,464 |
| ATP5G1 | ATP synthase, H+ transporting, mitochondrial Fo complex subunit C1 (subunit 9) | **0,866** | 0,002617 | 13,017 | 14,941 |
| NDUFA12 | NADH:ubiquinone oxidoreductase subunit A12 | **0,866** | 0,001837 | 16,783 | 10,854 |
| EMILIN2 | elastin microfibril interfacer 2 | **0,866** | 0,067466 | 2,770 | 2,299 |
| NIFK | nucleolar protein interacting with the FHA domain of MKI67 | **0,866** | 6,00E-06 | 22,156 | 22,475 |
| ARFGAP1 | ADP ribosylation factor GTPase activating protein 1 | **0,866** | 0 | 73,503 | 65,893 |
| PLEKHG2 | pleckstrin homology and RhoGEF domain containing G2 | **0,866** | 0,016547 | 5,428 | 3,512 |
| GLRB | glycine receptor beta | **0,866** | 0,01151 | 6,038 | 4,661 |
| ROR1 | receptor tyrosine kinase like orphan receptor 1 | **0,866** | 0,133461 | 2,825 | 1,596 |
| ANKRD36 | ankyrin repeat domain 36 | **0,866** | 0,101925 | 3,767 | 2,235 |
| HSD17B7 | hydroxysteroid 17-beta dehydrogenase 7 | **0,866** | 0,056804 | 5,207 | 3,129 |
| SNAI2 | snail family transcriptional repressor 2 | **0,865** | 6,00E-04 | 22,932 | 11,493 |
| CDKN2AIPNL | CDKN2A interacting protein N-terminal like | **0,865** | 0,000563 | 7,976 | 9,194 |
| RPL17 | ribosomal protein L17 | **0,865** | 0,050334 | 2,160 | 3,065 |
| EIF2AK1 | eukaryotic translation initiation factor 2 alpha kinase 1 | **0,865** | 6,00E-06 | 53,285 | 39,523 |
| HDAC7 | histone deacetylase 7 | **0,865** | 2,00E-04 | 13,515 | 11,046 |
| HNRNPD | heterogeneous nuclear ribonucleoprotein D | **0,865** | 0 | 99,148 | 110,652 |
| KIRREL | kin of IRRE like (Drosophila) | **0,865** | 7,20E-05 | 188,216 | 181,270 |

| | | | | | |
|---|---|---|---|---|---|
| H2AFY2 | H2A histone family member Y2 | **0,865** | 0,000921 | 8,419 | 7,023 |
| SCARF2 | scavenger receptor class F member 2 | **0,865** | 0,025442 | 12,130 | 9,577 |
| ZHX3 | zinc fingers and homeoboxes 3 | **0,865** | 0,001444 | 24,206 | 20,432 |
| EFHD2 | EF-hand domain family member D2 | **0,865** | 3,90E-05 | 21,159 | 22,603 |
| ALDH2 | aldehyde dehydrogenase 2 family (mitochondrial) | **0,865** | 2,20E-05 | 36,114 | 20,240 |
| DTD2 | D-tyrosyl-tRNA deacylase 2 (putative) | **0,865** | 0,00725 | 5,317 | 5,172 |
| KDELR2 | KDEL endoplasmic reticulum protein retention receptor 2 | **0,865** | 4,00E-06 | 195,970 | 143,279 |
| FTSJ1 | FtsJ RNA methyltransferase homolog 1 (E. coli) | **0,865** | 0,000144 | 19,442 | 11,748 |
| LOXL2 | lysyl oxidase like 2 | **0,865** | 0,000397 | 85,578 | 52,038 |
| TRUB2 | TruB pseudouridine synthase family member 2 | **0,865** | 4,00E-05 | 25,313 | 22,858 |
| TMEM18 | transmembrane protein 18 | **0,865** | 2,00E-06 | 19,830 | 17,112 |
| ZNF692 | zinc finger protein 692 | **0,865** | 0,003941 | 6,038 | 7,534 |
| C1orf122 | chromosome 1 open reading frame 122 | **0,865** | 0,00253 | 18,445 | 10,280 |
| FTSJ3 | FtsJ homolog 3 | **0,865** | 6,00E-06 | 49,353 | 52,357 |
| MRPL43 | mitochondrial ribosomal protein L43 | **0,865** | 6,00E-05 | 21,713 | 17,878 |
| UBE2E3 | ubiquitin conjugating enzyme E2 E3 | **0,865** | 3,80E-05 | 26,200 | 17,431 |
| MCTS1 | MCTS1, re-initiation and release factor | **0,865** | 0,000401 | 12,130 | 10,854 |
| CGREF1 | cell growth regulator with EF-hand domain 1 | **0,865** | 0,001614 | 9,306 | 5,363 |
| SLIT2 | slit guidance ligand 2 | **0,865** | 7,10E-05 | 22,378 | 42,460 |
| C16orf58 | chromosome 16 open reading frame 58 | **0,864** | 0 | 60,486 | 53,698 |
| FASTK | Fas activated serine/threonine kinase | **0,864** | 1,00E-06 | 41,487 | 39,459 |
| CDCA5 | cell division cycle associated 5 | **0,864** | 0,068557 | 5,041 | 1,085 |
| ADSL | adenylosuccinate lyase | **0,864** | 0,048401 | 2,659 | 2,362 |
| GEMIN4 | gem nuclear organelle associated protein 4 | **0,864** | 0,001066 | 16,174 | 16,282 |
| PINLYP | phospholipase A2 inhibitor and LY6/PLAUR domain containing | **0,864** | 0,394866 | 0,499 | 0,766 |
| TRPC4AP | transient receptor potential cation channel subfamily C member 4 associated protein | **0,864** | 1,00E-06 | 66,745 | 59,317 |
| NDUFA11 | NADH:ubiquinone oxidoreductase subunit A11 | **0,864** | 0,001319 | 12,518 | 10,982 |
| QTRT1 | queuine tRNA-ribosyltransferase catalytic subunit 1 | **0,864** | 0,005479 | 7,588 | 7,598 |
| BRIP1 | BRCA1 interacting protein C-terminal helicase 1 | **0,864** | 0,495485 | 0,831 | 0,128 |
| C22orf23 | chromosome 22 open reading frame 23 | **0,864** | 0,277194 | 0,831 | 0,766 |
| POLR1E | RNA polymerase I subunit E | **0,864** | 0,000174 | 10,967 | 10,535 |
| CD300C | CD300c molecule | **0,864** | 0,136445 | 0,997 | 1,532 |
| ALG14 | ALG14, UDP-N-acetylglucosaminyltransferase subunit | **0,864** | 0,008548 | 4,819 | 3,448 |

| | | | | | |
|---|---|---|---|---|---|
| EBNA1BP2 | EBNA1 binding protein 2 | **0,864** | 7,00E-06 | 39,881 | 43,801 |
| DOLPP1 | dolichyldiphosphatase 1 | **0,864** | 0,004495 | 8,585 | 6,768 |
| BCKDK | branched chain ketoacid dehydrogenase kinase | **0,864** | 0,000352 | 21,768 | 15,835 |
| VDR | vitamin D (1,25- dihydroxyvitamin D3) receptor | **0,864** | 1,00E-06 | 48,079 | 33,202 |
| C20orf27 | chromosome 20 open reading frame 27 | **0,864** | 0,011256 | 7,035 | 6,513 |
| ROMO1 | reactive oxygen species modulator 1 | **0,864** | 0,00031 | 24,427 | 18,453 |
| ALG5 | ALG5, dolichyl-phosphate beta-glucosyltransferase | **0,864** | 1,50E-05 | 20,827 | 21,901 |
| NDUFAF8 | NADH:ubiquinone oxidoreductase complex assembly factor 8 | **0,863** | 0,006598 | 7,090 | 7,534 |
| CSNK1E | casein kinase 1 epsilon | **0,863** | 0 | 42,429 | 43,737 |
| TTYH3 | tweety family member 3 | **0,863** | 0,021149 | 5,207 | 2,490 |
| NEIL2 | nei like DNA glycosylase 2 | **0,863** | 0,391914 | 0,332 | 0,702 |
| LAT2 | linker for activation of T-cells family member 2 | **0,863** | 0,330845 | 0,277 | 1,085 |
| MSMP | microseminoprotein, prostate associated | **0,863** | 0,001722 | 117,926 | 8,300 |
| PTPN14 | protein tyrosine phosphatase, non-receptor type 14 | **0,863** | 0,000808 | 236,848 | 218,814 |
| FLRT2 | fibronectin leucine rich transmembrane protein 2 | **0,863** | 0,026599 | 8,973 | 3,001 |
| LPAR4 | lysophosphatidic acid receptor 4 | **0,863** | 0,210079 | 2,880 | 1,660 |
| SLC1A1 | solute carrier family 1 member 1 | **0,863** | 2,00E-06 | 37,776 | 40,736 |
| CDR1 | cerebellar degeneration related protein 1 | **0,863** | 0,545703 | 21,104 | 42,652 |
| FAM20A | FAM20A, golgi associated secretory pathway pseudokinase | **0,863** | 0,022211 | 0,775 | 4,533 |
| METTL21A | methyltransferase like 21A | **0,863** | 0,015638 | 4,764 | 4,725 |
| AKIP1 | A-kinase interacting protein 1 | **0,863** | 0,002629 | 8,807 | 6,130 |
| LINC02381 | long intergenic non-protein coding RNA 2381 | **0,863** | 0,016534 | 6,813 | 4,661 |
| GNL3L | G protein nucleolar 3 like | **0,863** | 0,025616 | 10,081 | 8,939 |
| ILK | integrin linked kinase | **0,863** | 0 | 98,373 | 67,425 |
| MCTP2 | multiple C2 and transmembrane domain containing 2 | **0,863** | 0,00247 | 13,017 | 11,174 |
| PDXDC1 | pyridoxal dependent decarboxylase domain containing 1 | **0,862** | 0,31121 | 0,609 | 0,383 |
| TMED9 | transmembrane p24 trafficking protein 9 | **0,862** | 3,00E-06 | 257,010 | 182,675 |
| CCDC36 | coiled-coil domain containing 36 | **0,862** | 0,090557 | 4,930 | 1,469 |
| ERBB2 | erb-b2 receptor tyrosine kinase 2 | **0,862** | 5,40E-05 | 34,840 | 17,112 |
| HSD17B1 | hydroxysteroid 17-beta dehydrogenase 1 | **0,862** | 0,413247 | 0,111 | 0,383 |
| CREB3L2 | cAMP responsive element binding protein 3 like 2 | **0,862** | 1,70E-05 | 178,356 | 152,665 |
| SRA1 | steroid receptor RNA activator 1 | **0,862** | 1,00E-06 | 36,170 | 35,756 |
| TMEM232 | transmembrane protein 232 | **0,862** | 0,27665 | 0,886 | 1,149 |

| | | | | | |
|---|---|---|---|---|---|
| EXOC3-AS1 | EXOC3 antisense RNA 1 | **0,862** | 0,017347 | 2,825 | 3,256 |
| FBXO41 | F-box protein 41 | **0,862** | 0,273715 | 2,493 | 0,638 |
| SSR3 | signal sequence receptor subunit 3 | **0,862** | 0 | 187,883 | 160,391 |
| FBXW9 | F-box and WD repeat domain containing 9 | **0,862** | 0,083221 | 3,213 | 1,852 |
| DKC1 | dyskerin pseudouridine synthase 1 | **0,862** | 1,00E-06 | 41,543 | 37,288 |
| SNHG16 | small nucleolar RNA host gene 16 | **0,862** | 5,70E-05 | 22,045 | 15,069 |
| COPS6 | COP9 signalosome subunit 6 | **0,862** | 0 | 35,893 | 27,392 |
| KIAA1586 | KIAA1586 | **0,862** | 0,029291 | 4,210 | 4,725 |
| COTL1 | coactosin like F-actin binding protein 1 | **0,862** | 8,00E-06 | 88,015 | 80,195 |
| ABCC10 | ATP binding cassette subfamily C member 10 | **0,861** | 9,50E-05 | 12,574 | 9,514 |
| TMEM268 | transmembrane protein 268 | **0,861** | 0,012171 | 6,591 | 5,363 |
| IMPDH2 | inosine monophosphate dehydrogenase 2 | **0,861** | 5,00E-06 | 60,541 | 56,188 |
| GPR176 | G protein-coupled receptor 176 | **0,861** | 2,00E-06 | 45,531 | 27,519 |
| CC2D2A | coiled-coil and C2 domain containing 2A | **0,861** | 0,027158 | 4,930 | 2,171 |
| SLPI | secretory leukocyte peptidase inhibitor | **0,861** | 0,0011 | 15,786 | 18,963 |
| TCOF1 | treacle ribosome biogenesis factor 1 | **0,861** | 3,10E-05 | 27,252 | 28,669 |
| WRAP73 | WD repeat containing, antisense to TP73 | **0,861** | 0,00099 | 9,416 | 9,705 |
| BUB1B | BUB1 mitotic checkpoint serine/threonine kinase B | **0,861** | 0,427798 | 1,772 | 0,255 |
| HOXA10 | homeobox A10 | **0,861** | 0,000246 | 13,127 | 9,705 |
| IMP4 | IMP4 homolog, U3 small nucleolar ribonucleoprotein | **0,861** | 0 | 28,914 | 30,265 |
| TSPO | translocator protein | **0,861** | 3,00E-06 | 70,456 | 49,484 |
| SMARCA1 | SWI/SNF related, matrix associated, actin dependent regulator of chromatin, subfamily a, | **0,861** | 0,000244 | 33,179 | 16,537 |
| VCAN | versican | **0,861** | 0,021236 | 16,395 | 16,601 |
| C9orf163 | chromosome 9 open reading frame 163 | **0,861** | 0,430752 | 0,332 | 0,766 |
| SLC33A1 | solute carrier family 33 member 1 | **0,861** | 1,20E-05 | 24,593 | 17,878 |
| IL11 | interleukin 11 | **0,861** | 0,30934 | 30,852 | 4,406 |
| POLD2 | DNA polymerase delta 2, accessory subunit | **0,861** | 1,00E-06 | 52,842 | 46,994 |
| CMSS1 | cms1 ribosomal small subunit homolog (yeast) | **0,861** | 0,001009 | 11,078 | 13,855 |
| RPUSD1 | RNA pseudouridylate synthase domain containing 1 | **0,860** | 8,70E-05 | 15,232 | 9,577 |
| ATP13A1 | ATPase 13A1 | **0,860** | 4,00E-06 | 34,508 | 37,097 |
| CCDC87 | coiled-coil domain containing 87 | **0,860** | 0,116001 | 2,437 | 1,596 |
| IL17RB | interleukin 17 receptor B | **0,860** | 0,024577 | 13,183 | 7,215 |
| NDUFAF5 | NADH:ubiquinone oxidoreductase complex assembly factor 5 | **0,860** | 0,009117 | 4,043 | 2,873 |

| | | | | | |
|---|---|---|---|---|---|
| WDR43 | WD repeat domain 43 | **0,860** | 3,00E-06 | 25,590 | 28,158 |
| C1orf21 | chromosome 1 open reading frame 21 | **0,860** | 0 | 38,385 | 28,541 |
| ADORA2B | adenosine A2b receptor | **0,860** | 0,143104 | 0,831 | 0,894 |
| ADGRL1 | adhesion G protein-coupled receptor L1 | **0,860** | 0,152298 | 1,551 | 1,341 |
| MIB2 | mindbomb E3 ubiquitin protein ligase 2 | **0,860** | 2,00E-06 | 31,849 | 27,200 |
| DAZAP2P1 | DAZ associated protein 2 pseudogene 1 | **0,860** | 0,025991 | 0,055 | 2,809 |
| CATSPER2P1 | cation channel sperm associated 2 pseudogene 1 | **0,860** | 0,175338 | 0,942 | 1,149 |
| PVT1 | Pvt1 oncogene (non-protein coding) | **0,860** | 0,035847 | 1,994 | 2,682 |
| ANKRD13D | ankyrin repeat domain 13D | **0,860** | 0,002906 | 10,358 | 8,364 |
| SMAGP | small cell adhesion glycoprotein | **0,860** | 0,003933 | 5,871 | 5,491 |
| LOXL1 | lysyl oxidase like 1 | **0,859** | 0,000104 | 18,611 | 10,854 |
| LANCL2 | LanC like 2 | **0,859** | 0,000235 | 10,746 | 8,364 |
| SELP | selectin P | **0,859** | 0,108934 | 1,219 | 1,469 |
| SYNGR2 | synaptogyrin 2 | **0,859** | 1,60E-05 | 19,940 | 14,749 |
| CCDC12 | coiled-coil domain containing 12 | **0,859** | 2,30E-05 | 14,457 | 12,515 |
| MORC4 | MORC family CW-type zinc finger 4 | **0,859** | 0,00195 | 10,746 | 6,513 |
| RASIP1 | Ras interacting protein 1 | **0,859** | 0,036671 | 4,487 | 1,532 |
| ATP8B1 | ATPase phospholipid transporting 8B1 | **0,859** | 0,011436 | 4,874 | 3,512 |
| ERCC8 | ERCC excision repair 8, CSA ubiquitin ligase complex subunit | **0,859** | 0,038991 | 3,600 | 2,299 |
| FIBIN | fin bud initiation factor homolog (zebrafish) | **0,859** | 0 | 37,998 | 48,271 |
| RPL13AP5 | ribosomal protein L13a pseudogene 5 | **0,859** | 0,100524 | 1,717 | 1,149 |
| CTGF | connective tissue growth factor | **0,859** | 0,001415 | 1402,479 | 1734,610 |
| BEND7 | BEN domain containing 7 | **0,859** | 0,026149 | 3,600 | 3,512 |
| PRKCZ-AS1 | PRKCZ antisense RNA 1 | **0,859** | 0,214834 | 0,554 | 0,575 |
| TMEM39A | transmembrane protein 39A | **0,859** | 2,10E-05 | 66,025 | 54,975 |
| COL1A2 | collagen type I alpha 2 chain | **0,859** | 0,00177 | 27,197 | 58,550 |
| SYNPO | synaptopodin | **0,859** | 0,000351 | 39,715 | 33,074 |
| ACP2 | acid phosphatase 2, lysosomal | **0,859** | 9,00E-06 | 34,674 | 30,967 |
| ALG8 | ALG8, alpha-1,3-glucosyltransferase | **0,858** | 0,00088 | 14,512 | 12,195 |
| IKZF2 | IKAROS family zinc finger 2 | **0,858** | 0,049925 | 3,213 | 2,426 |
| LAMTOR2 | late endosomal/lysosomal adaptor, MAPK and MTOR activator 2 | **0,858** | 0,002616 | 13,404 | 9,194 |
| SAC3D1 | SAC3 domain containing 1 | **0,858** | 0,011626 | 5,982 | 4,661 |
| CBX8 | chromobox 8 | **0,858** | 0,022438 | 3,268 | 1,213 |

| | | | | | |
|---|---|---|---|---|---|
| MOSPD3 | motile sperm domain containing 3 | **0,858** | 0,001473 | 8,087 | 5,427 |
| PITRM1-AS1 | PITRM1 antisense RNA 1 | **0,858** | 0,328158 | 0,942 | 0,638 |
| MANF | mesencephalic astrocyte derived neurotrophic factor | **0,858** | 0,000111 | 78,432 | 58,359 |
| SLC22A5 | solute carrier family 22 member 5 | **0,858** | 0,002845 | 3,656 | 5,491 |
| BAIAP2L2 | BAI1 associated protein 2 like 2 | **0,858** | 0,306825 | 0,277 | 0,575 |
| URB2 | URB2 ribosome biogenesis 2 homolog (S. cerevisiae) | **0,857** | 0,000512 | 10,856 | 12,578 |
| PTK6 | protein tyrosine kinase 6 | **0,857** | 0,437407 | 0,055 | 0,383 |
| CKAP4 | cytoskeleton associated protein 4 | **0,857** | 0 | 284,428 | 176,736 |
| SNCA | synuclein alpha | **0,857** | 0,318106 | 2,493 | 0,383 |
| CCL7 | C-C motif chemokine ligand 7 | **0,857** | 0,065948 | 3,268 | 2,299 |
| SLC12A9 | solute carrier family 12 member 9 | **0,857** | 0,000481 | 12,186 | 7,151 |
| SPHK2 | sphingosine kinase 2 | **0,857** | 4,00E-06 | 13,958 | 13,025 |
| MCRIP2 | MAPK regulated corepressor interacting protein 2 | **0,857** | 0,01237 | 5,761 | 3,065 |
| BACE2 | beta-site APP-cleaving enzyme 2 | **0,857** | 0 | 56,498 | 50,952 |
| CHRNE | cholinergic receptor nicotinic epsilon subunit | **0,857** | 0,051748 | 1,274 | 0,830 |
| LDLRAD3 | low density lipoprotein receptor class A domain containing 3 | **0,857** | 0 | 36,502 | 28,094 |
| TMEM158 | transmembrane protein 158 (gene/pseudogene) | **0,857** | 8,00E-06 | 72,949 | 47,185 |
| PAN3-AS1 | PAN3 antisense RNA 1 | **0,857** | 0,166866 | 0,942 | 1,213 |
| FOXF1 | forkhead box F1 | **0,857** | 0,164538 | 1,385 | 1,085 |
| LINC00116 | long intergenic non-protein coding RNA 116 | **0,857** | 0,005138 | 7,588 | 7,726 |
| RRP36 | ribosomal RNA processing 36 | **0,857** | 5,80E-05 | 18,943 | 16,346 |
| SRSF9 | serine and arginine rich splicing factor 9 | **0,857** | 3,00E-06 | 54,504 | 41,055 |
| KDM8 | lysine demethylase 8 | **0,857** | 0,129731 | 2,105 | 1,852 |
| NECAB3 | N-terminal EF-hand calcium binding protein 3 | **0,857** | 0,023833 | 3,323 | 2,235 |
| TBX4 | T-box 4 | **0,857** | 0,001908 | 8,973 | 8,428 |
| ZNF860 | zinc finger protein 860 | **0,857** | 0,08741 | 2,493 | 3,831 |
| TMEM25 | transmembrane protein 25 | **0,857** | 0,013561 | 6,370 | 3,256 |
| GDPGP1 | GDP-D-glucose phosphorylase 1 | **0,857** | 0,146036 | 0,831 | 0,766 |
| DCPS | decapping enzyme, scavenger | **0,856** | 0,004056 | 4,985 | 5,300 |
| PTOV1 | prostate tumor overexpressed 1 | **0,856** | 1,60E-05 | 30,797 | 21,901 |
| ORAI2 | ORAI calcium release-activated calcium modulator 2 | **0,856** | 0 | 34,563 | 21,390 |
| PSMB5 | proteasome subunit beta 5 | **0,856** | 0 | 64,640 | 46,930 |
| PHPT1 | phosphohistidine phosphatase 1 | **0,856** | 5,00E-06 | 26,255 | 19,793 |

| | | | | | |
|---|---|---|---|---|---|
| AGAP2-AS1 | AGAP2 antisense RNA 1 | **0,856** | 0,016748 | 6,758 | 3,129 |
| WDR4 | WD repeat domain 4 | **0,856** | 0,006985 | 7,478 | 7,087 |
| LINC00893 | long intergenic non-protein coding RNA 893 | **0,856** | 0,268354 | 0,831 | 0,766 |
| NF2 | neurofibromin 2 | **0,856** | 2,00E-06 | 26,421 | 15,899 |
| PRR34-AS1 | PRR34 antisense RNA 1 | **0,856** | 0,115281 | 2,271 | 1,277 |
| SGSM2 | small G protein signaling modulator 2 | **0,856** | 0,005594 | 11,521 | 7,917 |
| SMIM10 | small integral membrane protein 10 | **0,856** | 0,003003 | 8,198 | 7,662 |
| FKBP2 | FK506 binding protein 2 | **0,856** | 3,50E-05 | 56,443 | 34,926 |
| FOXN3-AS1 | FOXN3 antisense RNA 1 | **0,856** | 0,111969 | 1,551 | 1,788 |
| PTDSS2 | phosphatidylserine synthase 2 | **0,856** | 0,035785 | 2,825 | 1,660 |
| TRAPPC1 | trafficking protein particle complex 1 | **0,856** | 1,00E-06 | 16,395 | 10,854 |
| TWNK | twinkle mtDNA helicase | **0,856** | 0,000151 | 9,416 | 12,004 |
| GNB1L | G protein subunit beta 1 like | **0,856** | 0,101864 | 1,939 | 2,618 |
| TMUB1 | transmembrane and ubiquitin like domain containing 1 | **0,856** | 0,000872 | 20,162 | 12,578 |
| MIR635 | microRNA 635 | **0,856** | 0,275191 | 0,775 | 0,319 |
| GALK1 | galactokinase 1 | **0,856** | 0,033346 | 3,323 | 1,852 |
| GEMIN7 | gem nuclear organelle associated protein 7 | **0,855** | 0,002636 | 7,533 | 6,449 |
| TMCC1-AS1 | TMCC1 antisense RNA 1 (head to head) | **0,855** | 0,302041 | 0,665 | 0,575 |
| NEMP2 | nuclear envelope integral membrane protein 2 | **0,855** | 0,005546 | 5,207 | 3,767 |
| NKX2-5 | NK2 homeobox 5 | **0,855** | 0,356297 | 0,222 | 0,511 |
| MID1 | midline 1 | **0,855** | 0,047631 | 4,874 | 4,150 |
| ANKRD36B | ankyrin repeat domain 36B | **0,855** | 0,333334 | 1,385 | 0,830 |
| COX16 | COX16, cytochrome c oxidase assembly homolog | **0,855** | 0,229469 | 0,720 | 0,830 |
| BATF2 | basic leucine zipper ATF-like transcription factor 2 | **0,855** | 0,054701 | 2,160 | 3,192 |
| RIPOR3 | RIPOR family member 3 | **0,855** | 0,006236 | 5,816 | 1,341 |
| NUFIP1 | NUFIP1, FMR1 interacting protein 1 | **0,855** | 0,002481 | 5,927 | 5,491 |
| PIGU | phosphatidylinositol glycan anchor biosynthesis class U | **0,855** | 0,000111 | 11,300 | 11,621 |
| JAK2 | Janus kinase 2 | **0,855** | 0 | 68,407 | 41,439 |
| ZWINT | ZW10 interacting kinetochore protein | **0,855** | 0,014751 | 5,262 | 2,746 |
| MRPL28 | mitochondrial ribosomal protein L28 | **0,855** | 0 | 30,908 | 22,092 |
| TSC22D4 | TSC22 domain family member 4 | **0,855** | 4,00E-05 | 53,396 | 38,118 |
| RTL3 | retrotransposon Gag like 3 | **0,855** | 0,037337 | 3,102 | 2,746 |
| DNM3OS | DNM3 opposite strand/antisense RNA | **0,855** | 0,017112 | 4,764 | 3,256 |

| | | | | | |
|---|---|---|---|---|---|
| TRAPPC12 | trafficking protein particle complex 12 | **0,855** | 0,002254 | 8,696 | 7,023 |
| GNL2 | G protein nucleolar 2 | **0,855** | 0 | 46,029 | 38,629 |
| S1PR2 | sphingosine-1-phosphate receptor 2 | **0,855** | 0,101555 | 2,216 | 1,022 |
| CYGB | cytoglobin | **0,854** | 0,009922 | 4,708 | 2,235 |
| COASY | Coenzyme A synthase | **0,854** | 0 | 34,176 | 26,689 |
| INSIG2 | insulin induced gene 2 | **0,854** | 0 | 86,187 | 47,696 |
| SLC39A1 | solute carrier family 39 member 1 | **0,854** | 0 | 91,449 | 66,787 |
| SMYD5 | SMYD family member 5 | **0,854** | 0,001399 | 14,014 | 15,707 |
| PUSL1 | pseudouridylate synthase-like 1 | **0,854** | 0,029989 | 5,539 | 5,746 |
| CTBP1-AS2 | CTBP1 antisense RNA 2 (head to head) | **0,854** | 0,000148 | 16,008 | 12,451 |
| FITM2 | fat storage inducing transmembrane protein 2 | **0,854** | 2,00E-06 | 17,614 | 15,132 |
| APRT | adenine phosphoribosyltransferase | **0,854** | 0,000107 | 18,334 | 14,558 |
| ANKRD13B | ankyrin repeat domain 13B | **0,854** | 0,015104 | 3,711 | 3,576 |
| PEX5L | peroxisomal biogenesis factor 5 like | **0,854** | 0,443672 | 1,496 | 0,511 |
| APOLD1 | apolipoprotein L domain containing 1 | **0,854** | 0,017392 | 4,874 | 3,767 |
| ITGBL1 | integrin subunit beta like 1 | **0,854** | 0,000127 | 11,909 | 9,514 |
| RPL4P4 | ribosomal protein L4 pseudogene 4 | **0,854** | 0,354614 | 0,831 | 0,447 |
| HYAL2 | hyaluronoglucosaminidase 2 | **0,854** | 0,00169 | 13,958 | 11,493 |
| KRT17 | keratin 17 | **0,854** | 0,095166 | 11,687 | 7,854 |
| TKFC | triokinase and FMN cyclase | **0,854** | 1,10E-05 | 19,940 | 15,260 |
| ARSI | arylsulfatase family member I | **0,854** | 0,153601 | 1,274 | 1,596 |
| MCFD2 | multiple coagulation factor deficiency 2 | **0,854** | 0 | 300,436 | 214,983 |
| ZNF491 | zinc finger protein 491 | **0,854** | 0,342316 | 0,665 | 0,511 |
| CDK2AP1 | cyclin dependent kinase 2 associated protein 1 | **0,854** | 1,00E-06 | 40,102 | 19,347 |
| NACAP1 | nascent polypeptide associated complex alpha subunit pseudogene 1 | **0,854** | 0,317097 | 0,720 | 0,383 |
| GLIPR1 | GLI pathogenesis related 1 | **0,854** | 2,00E-06 | 37,001 | 23,944 |
| PGAP3 | post-GPI attachment to proteins 3 | **0,854** | 4,40E-05 | 10,856 | 6,130 |
| ZBTB47 | zinc finger and BTB domain containing 47 | **0,854** | 0,004472 | 13,294 | 5,619 |
| SEC61A1 | Sec61 translocon alpha 1 subunit | **0,853** | 0 | 447,940 | 326,784 |
| NDUFB10 | NADH:ubiquinone oxidoreductase subunit B10 | **0,853** | 1,70E-05 | 27,972 | 16,665 |
| FANCA | Fanconi anemia complementation group A | **0,853** | 0,105356 | 2,991 | 1,085 |
| EFEMP2 | EGF containing fibulin like extracellular matrix protein 2 | **0,853** | 0 | 105,463 | 49,867 |
| AGAP9 | ArfGAP with GTPase domain, ankyrin repeat and PH domain 9 | **0,853** | 0,055442 | 1,329 | 1,724 |

| | | | | | |
|---|---|---|---|---|---|
| MYL12A | myosin light chain 12A | **0,853** | 0 | 126,179 | 82,877 |
| PEMT | phosphatidylethanolamine N-methyltransferase | **0,853** | 0,000357 | 10,801 | 9,577 |
| STAT4 | signal transducer and activator of transcription 4 | **0,853** | 0,058662 | 4,708 | 2,171 |
| RBM42 | RNA binding motif protein 42 | **0,853** | 1,80E-05 | 40,933 | 31,733 |
| PFKM | phosphofructokinase, muscle | **0,853** | 9,00E-06 | 20,051 | 14,877 |
| RBM3 | RNA binding motif (RNP1, RRM) protein 3 | **0,853** | 0 | 82,365 | 56,443 |
| CTXN1 | cortexin 1 | **0,853** | 0,06544 | 1,883 | 0,830 |
| OGFOD3 | 2-oxoglutarate and iron dependent oxygenase domain containing 3 | **0,853** | 3,60E-05 | 18,943 | 11,238 |
| BHLHE40-AS1 | BHLHE40 antisense RNA 1 | **0,853** | 0,27143 | 0,609 | 0,638 |
| SWI5 | SWI5 homologous recombination repair protein | **0,853** | 0 | 22,378 | 19,985 |
| CD226 | CD226 molecule | **0,853** | 0,404972 | 0,665 | 0,702 |
| PKNOX2 | PBX/knotted 1 homeobox 2 | **0,853** | 0,001793 | 3,877 | 5,555 |
| HOXA5 | homeobox A5 | **0,853** | 0,009653 | 3,545 | 4,150 |
| TMEM258 | transmembrane protein 258 | **0,853** | 0 | 51,513 | 40,736 |
| PI4KAP2 | phosphatidylinositol 4-kinase alpha pseudogene 2 | **0,852** | 0,153918 | 1,385 | 1,532 |
| MVB12A | multivesicular body subunit 12A | **0,852** | 0,000424 | 10,690 | 9,194 |
| SNF8 | SNF8, ESCRT-II complex subunit | **0,852** | 3,80E-05 | 28,415 | 22,667 |
| PLEKHA2 | pleckstrin homology domain containing A2 | **0,852** | 0,066364 | 1,496 | 1,149 |
| POMK | protein-O-mannose kinase | **0,852** | 0,272443 | 1,108 | 1,277 |
| RFTN1 | raftlin, lipid raft linker 1 | **0,852** | 0,014235 | 3,157 | 3,001 |
| MRPS28 | mitochondrial ribosomal protein S28 | **0,852** | 0,009759 | 4,043 | 3,831 |
| TRDMT1 | tRNA aspartic acid methyltransferase 1 | **0,852** | 0,009878 | 3,988 | 3,384 |
| GNL3 | G protein nucleolar 3 | **0,852** | 1,00E-06 | 42,983 | 37,033 |
| ALDH7A1 | aldehyde dehydrogenase 7 family member A1 | **0,852** | 0,001978 | 6,314 | 4,533 |
| UTP20 | UTP20, small subunit processome component | **0,852** | 0,000109 | 14,734 | 16,409 |
| ARL4D | ADP ribosylation factor like GTPase 4D | **0,852** | 0,001437 | 11,853 | 7,151 |
| TSEN2 | tRNA splicing endonuclease subunit 2 | **0,852** | 0,00769 | 5,096 | 5,236 |
| ABCB6 | ATP binding cassette subfamily B member 6 (Langereis blood group) | **0,852** | 0,259207 | 0,609 | 0,511 |
| FAM92A | family with sequence similarity 92 member A | **0,852** | 0,005931 | 7,201 | 4,725 |
| ALKBH7 | alkB homolog 7 | **0,852** | 0,000723 | 18,500 | 10,344 |
| ACO1 | aconitase 1 | **0,851** | 0 | 89,677 | 74,194 |
| MRPL2 | mitochondrial ribosomal protein L2 | **0,851** | 0,000176 | 15,454 | 15,388 |
| WDR77 | WD repeat domain 77 | **0,851** | 2,60E-05 | 17,393 | 14,685 |

| | | | | | |
|---|---|---|---|---|---|
| GSTT2B | glutathione S-transferase theta 2B (gene/pseudogene) | **0,851** | 0,342128 | 0,665 | 0,128 |
| FOXC2-AS1 | FOXC2 antisense RNA 1 | **0,851** | 0,302443 | 0,499 | 0,638 |
| LINC00910 | long intergenic non-protein coding RNA 910 | **0,851** | 0,12769 | 1,883 | 1,149 |
| RNF121 | ring finger protein 121 | **0,851** | 1,00E-06 | 20,494 | 15,196 |
| NHS | NHS actin remodeling regulator | **0,851** | 0,072168 | 3,102 | 1,532 |
| C8orf46 | chromosome 8 open reading frame 46 | **0,851** | 0,040239 | 3,046 | 1,979 |
| DYNC1I2P1 | dynein cytoplasmic 1 intermediate chain 2 pseudogene 1 | **0,851** | 0,413645 | 0,388 | 0,319 |
| CDR2 | cerebellar degeneration related protein 2 | **0,851** | 0,054989 | 2,382 | 1,085 |
| PRMT6 | protein arginine methyltransferase 6 | **0,851** | 0,000143 | 12,075 | 9,577 |
| SLC48A1 | solute carrier family 48 member 1 | **0,850** | 0,000231 | 9,859 | 7,854 |
| SCYL1 | SCY1 like pseudokinase 1 | **0,850** | 0 | 69,072 | 46,036 |
| KLHL35 | kelch like family member 35 | **0,850** | 0,048654 | 2,105 | 1,213 |
| LINC01010 | long intergenic non-protein coding RNA 1010 | **0,850** | 0,383605 | 1,052 | 0,447 |
| GGACT | gamma-glutamylamine cyclotransferase | **0,850** | 0,110973 | 1,772 | 1,022 |
| TMEM45A | transmembrane protein 45A | **0,850** | 0 | 72,561 | 40,162 |
| FLJ37453 | uncharacterized LOC729614 | **0,850** | 0,038408 | 4,265 | 4,533 |
| NSUN5P2 | NOP2/Sun RNA methyltransferase family member 5 pseudogene 2 | **0,850** | 0,055073 | 2,603 | 2,554 |
| HNRNPA1P48 | heterogeneous nuclear ribonucleoprotein A1 pseudogene 48 | **0,850** | 0,311825 | 0,997 | 1,277 |
| EFCAB11 | EF-hand calcium binding domain 11 | **0,850** | 0,02524 | 2,770 | 2,362 |
| WNK4 | WNK lysine deficient protein kinase 4 | **0,850** | 0,006889 | 6,924 | 2,554 |
| MRPL48 | mitochondrial ribosomal protein L48 | **0,850** | 0,010083 | 6,204 | 3,703 |
| SHROOM3 | shroom family member 3 | **0,850** | 0,004571 | 7,865 | 5,619 |
| TIMM17B | translocase of inner mitochondrial membrane 17B | **0,850** | 3,00E-06 | 16,562 | 11,876 |
| ZBTB11-AS1 | ZBTB11 antisense RNA 1 | **0,849** | 0,001064 | 5,373 | 3,639 |
| OPA3 | OPA3, outer mitochondrial membrane lipid metabolism regulator | **0,849** | 1,00E-06 | 25,036 | 18,963 |
| GMIP | GEM interacting protein | **0,849** | 0,015557 | 5,207 | 4,214 |
| EPHA4 | EPH receptor A4 | **0,849** | 0,001008 | 11,466 | 11,238 |
| CCDC181 | coiled-coil domain containing 181 | **0,849** | 0,026992 | 2,880 | 2,362 |
| IL4R | interleukin 4 receptor | **0,849** | 0,011831 | 3,877 | 3,512 |
| SNHG15 | small nucleolar RNA host gene 15 | **0,849** | 0,000481 | 9,361 | 15,388 |
| ATOX1 | antioxidant 1 copper chaperone | **0,849** | 9,90E-05 | 19,553 | 19,410 |
| PCDH12 | protocadherin 12 | **0,849** | 0,000262 | 4,265 | 12,004 |
| CHCHD5 | coiled-coil-helix-coiled-coil-helix domain containing 5 | **0,848** | 2,10E-05 | 10,247 | 8,684 |

| | | | | | |
|---|---|---|---|---|---|
| RERG | RAS like estrogen regulated growth inhibitor | **0,848** | 3,00E-06 | 32,016 | 30,009 |
| HIGD2A | HIG1 hypoxia inducible domain family member 2A | **0,848** | 0,000176 | 15,011 | 9,131 |
| TCIRG1 | T-cell immune regulator 1, ATPase H+ transporting V0 subunit a3 | **0,848** | 1,00E-06 | 32,680 | 22,284 |
| TMA7 | translation machinery associated 7 homolog | **0,848** | 0,001465 | 22,433 | 18,580 |
| CCDC138 | coiled-coil domain containing 138 | **0,848** | 0,173199 | 0,886 | 0,958 |
| OSR1 | odd-skipped related transciption factor 1 | **0,848** | 0,321167 | 0,332 | 0,511 |
| CAPN15 | calpain 15 | **0,848** | 2,00E-06 | 28,858 | 30,073 |
| C19orf24 | chromosome 19 open reading frame 24 | **0,848** | 0,000112 | 15,786 | 11,557 |
| B9D1 | B9 domain containing 1 | **0,848** | 0,000115 | 9,582 | 6,832 |
| CXCL1 | C-X-C motif chemokine ligand 1 | **0,848** | 0 | 1507,720 | 1188,757 |
| CCDC144B | coiled-coil domain containing 144B (pseudogene) | **0,848** | 0,232333 | 0,665 | 1,660 |
| MRPS11 | mitochondrial ribosomal protein S11 | **0,848** | 2,30E-05 | 14,346 | 10,918 |
| IL18R1 | interleukin 18 receptor 1 | **0,848** | 0,024948 | 2,382 | 3,129 |
| ARAP3 | ArfGAP with RhoGAP domain, ankyrin repeat and PH domain 3 | **0,848** | 0,017212 | 3,933 | 3,448 |
| ESPNL | espin-like | **0,848** | 0,362752 | 0,000 | 0,958 |
| RTL8B | retrotransposon Gag like 8B | **0,848** | 0,000603 | 6,647 | 5,363 |
| KRT18 | keratin 18 | **0,848** | 0,000705 | 8,696 | 8,939 |
| TRAFD1 | TRAF-type zinc finger domain containing 1 | **0,848** | 1,00E-06 | 32,736 | 24,582 |
| UBXN6 | UBX domain protein 6 | **0,848** | 0 | 80,537 | 52,229 |
| MEI1 | meiotic double-stranded break formation protein 1 | **0,847** | 0,000221 | 10,967 | 9,322 |
| STS | steroid sulfatase | **0,847** | 0 | 92,003 | 58,167 |
| TP53I13 | tumor protein p53 inducible protein 13 | **0,847** | 3,00E-06 | 28,858 | 18,836 |
| NUDT1 | nudix hydrolase 1 | **0,847** | 0,01439 | 3,379 | 3,320 |
| METTL26 | methyltransferase like 26 | **0,847** | 0,000367 | 15,565 | 7,343 |
| FLYWCH2 | FLYWCH family member 2 | **0,847** | 0,000153 | 8,973 | 6,385 |
| ACTR8 | ARP8 actin-related protein 8 homolog | **0,847** | 9,00E-06 | 21,935 | 17,559 |
| IFRD2 | interferon related developmental regulator 2 | **0,847** | 0 | 35,505 | 31,286 |
| SLC35E1 | solute carrier family 35 member E1 | **0,847** | 0 | 79,706 | 61,871 |
| GPRC5C | G protein-coupled receptor class C group 5 member C | **0,847** | 0,000502 | 12,961 | 9,450 |
| OCIAD2 | OCIA domain containing 2 | **0,847** | 0,023007 | 2,714 | 1,979 |
| CUEDC2 | CUE domain containing 2 | **0,847** | 0,00014 | 18,833 | 11,685 |
| FOXM1 | forkhead box M1 | **0,847** | 0,046792 | 9,527 | 0,702 |
| KCTD21-AS1 | KCTD21 antisense RNA 1 | **0,847** | 0,162308 | 0,499 | 1,469 |

| | | | | | |
|---|---|---|---|---|---|
| MB21D2 | Mab-21 domain containing 2 | **0,847** | 0,004876 | 4,487 | 3,895 |
| GALNT2 | polypeptide N-acetylgalactosaminyltransferase 2 | **0,847** | 0 | 488,874 | 262,295 |
| KRT10 | keratin 10 | **0,847** | 2,00E-06 | 38,995 | 33,138 |
| CENPH | centromere protein H | **0,847** | 0,090782 | 3,877 | 1,724 |
| PART1 | prostate androgen-regulated transcript 1 (non-protein coding) | **0,846** | 0,144023 | 0,775 | 0,894 |
| NAA38 | N(alpha)-acetyltransferase 38, NatC auxiliary subunit | **0,846** | 0,000346 | 9,915 | 7,343 |
| GHDC | GH3 domain containing | **0,846** | 1,00E-06 | 21,325 | 18,708 |
| RHOBTB1 | Rho related BTB domain containing 1 | **0,846** | 0,045945 | 2,936 | 1,149 |
| TIPARP-AS1 | TIPARP antisense RNA 1 | **0,846** | 0,329094 | 0,166 | 0,766 |
| GRWD1 | glutamate rich WD repeat containing 1 | **0,846** | 3,00E-06 | 24,870 | 22,156 |
| TGFB1I1 | transforming growth factor beta 1 induced transcript 1 | **0,846** | 3,00E-06 | 21,104 | 13,472 |
| JTB | jumping translocation breakpoint | **0,846** | 0 | 54,061 | 32,947 |
| WDR34 | WD repeat domain 34 | **0,846** | 0 | 31,351 | 22,667 |
| SFN | stratifin | **0,846** | 0,086209 | 3,213 | 1,405 |
| TIMM13 | translocase of inner mitochondrial membrane 13 | **0,846** | 6,80E-05 | 14,401 | 11,812 |
| FXYD5 | FXYD domain containing ion transport regulator 5 | **0,846** | 0 | 202,673 | 114,674 |
| FNTB | farnesyltransferase, CAAX box, beta | **0,846** | 0,003464 | 3,656 | 3,192 |
| UFSP1 | UFM1 specific peptidase 1 (inactive) | **0,845** | 0,122787 | 0,997 | 1,022 |
| DGKA | diacylglycerol kinase alpha | **0,845** | 0,003884 | 5,262 | 4,214 |
| GAS1 | growth arrest specific 1 | **0,845** | 0,000461 | 14,512 | 9,961 |
| NOL6 | nucleolar protein 6 | **0,845** | 0 | 31,074 | 34,670 |
| MAGIX | MAGI family member, X-linked | **0,845** | 0,076164 | 1,496 | 1,469 |
| CHRDL2 | chordin like 2 | **0,845** | 1,00E-06 | 26,920 | 19,538 |
| CCDC85B | coiled-coil domain containing 85B | **0,845** | 0,000412 | 132,936 | 125,848 |
| HOXA3 | homeobox A3 | **0,844** | 0,121556 | 0,831 | 0,894 |
| PPP1R13L | protein phosphatase 1 regulatory subunit 13 like | **0,844** | 6,00E-06 | 24,759 | 23,944 |
| HOXC8 | homeobox C8 | **0,844** | 0,061844 | 2,271 | 1,022 |
| ST8SIA1 | ST8 alpha-N-acetyl-neuraminide alpha-2,8-sialyltransferase 1 | **0,844** | 0,009502 | 4,210 | 5,363 |
| FRMPD4 | FERM and PDZ domain containing 4 | **0,844** | 0,267156 | 0,886 | 0,319 |
| EEF1E1 | eukaryotic translation elongation factor 1 epsilon 1 | **0,844** | 0,008221 | 5,650 | 5,427 |
| ALOXE3 | arachidonate lipoxygenase 3 | **0,844** | 0,026427 | 3,157 | 3,895 |
| POLE4 | DNA polymerase epsilon 4, accessory subunit | **0,844** | 1,90E-05 | 15,620 | 11,365 |
| C7orf31 | chromosome 7 open reading frame 31 | **0,844** | 0,041376 | 2,326 | 1,660 |

| | | | | | |
|---|---|---|---|---|---|
| MYL6 | myosin light chain 6 | **0,844** | 3,00E-06 | 218,791 | 131,531 |
| FKBP10 | FK506 binding protein 10 | **0,844** | 0 | 335,554 | 137,788 |
| PINK1 | PTEN induced putative kinase 1 | **0,844** | 0 | 107,789 | 64,041 |
| PFKFB4 | 6-phosphofructo-2-kinase/fructose-2,6-biphosphatase 4 | **0,843** | 5,70E-05 | 9,915 | 5,044 |
| EFNA5 | ephrin A5 | **0,843** | 1,60E-05 | 11,632 | 12,068 |
| CENPV | centromere protein V | **0,843** | 0,341734 | 0,665 | 0,830 |
| RBM34 | RNA binding motif protein 34 | **0,843** | 0,298505 | 0,609 | 0,702 |
| ANO7 | anoctamin 7 | **0,843** | 0,238137 | 0,831 | 0,766 |
| STK17A | serine/threonine kinase 17a | **0,843** | 0 | 103,358 | 77,003 |
| IRX3 | iroquois homeobox 3 | **0,843** | 0,082531 | 1,828 | 0,319 |
| RFPL3S | RFPL3 antisense | **0,843** | 0,256672 | 0,277 | 0,511 |
| ZDHHC12 | zinc finger DHHC-type containing 12 | **0,843** | 0,015889 | 6,425 | 2,809 |
| SDHAF3 | succinate dehydrogenase complex assembly factor 3 | **0,843** | 0,000461 | 6,038 | 4,789 |
| SLC39A13 | solute carrier family 39 member 13 | **0,843** | 0 | 65,083 | 36,331 |
| GLIS3 | GLIS family zinc finger 3 | **0,843** | 1,70E-05 | 50,017 | 50,314 |
| BMPER | BMP binding endothelial regulator | **0,842** | 0,03625 | 1,385 | 2,554 |
| MYPOP | Myb related transcription factor, partner of profilin | **0,842** | 9,50E-05 | 9,306 | 5,746 |
| PRR7 | proline rich 7, synaptic | **0,842** | 0,066303 | 1,440 | 0,830 |
| TMEM216 | transmembrane protein 216 | **0,842** | 2,00E-06 | 13,681 | 14,239 |
| PAPPA | pappalysin 1 | **0,842** | 0,000506 | 233,303 | 185,037 |
| MYBBP1A | MYB binding protein 1a | **0,842** | 0 | 27,750 | 26,242 |
| MRPL4 | mitochondrial ribosomal protein L4 | **0,842** | 6,00E-06 | 18,168 | 17,048 |
| FAIM2 | Fas apoptotic inhibitory molecule 2 | **0,842** | 0,152277 | 0,166 | 0,638 |
| GOLGA3 | golgin A3 | **0,842** | 0 | 125,237 | 101,330 |
| NIPSNAP1 | nipsnap homolog 1 | **0,842** | 0,003842 | 5,761 | 3,129 |
| PELI3 | pellino E3 ubiquitin protein ligase family member 3 | **0,842** | 0,010858 | 3,877 | 2,043 |
| MRPL54 | mitochondrial ribosomal protein L54 | **0,842** | 9,00E-06 | 15,454 | 12,323 |
| TMCO4 | transmembrane and coiled-coil domains 4 | **0,842** | 0,001478 | 7,035 | 5,044 |
| C6orf132 | chromosome 6 open reading frame 132 | **0,842** | 0,093381 | 0,997 | 1,213 |
| TMA16 | translation machinery associated 16 homolog | **0,842** | 1,30E-05 | 11,743 | 10,216 |
| C19orf70 | chromosome 19 open reading frame 70 | **0,842** | 0,000989 | 13,127 | 11,748 |
| GPX7 | glutathione peroxidase 7 | **0,842** | 0,011728 | 4,431 | 1,660 |
| ITPRIP | inositol 1,4,5-trisphosphate receptor interacting protein | **0,841** | 0 | 203,448 | 220,282 |

| | | | | | |
|---|---|---|---|---|---|
| ORC1 | origin recognition complex subunit 1 | **0,841** | 0,142646 | 1,717 | 0,702 |
| AKR1E2 | aldo-keto reductase family 1 member E2 | **0,841** | 0,074594 | 2,049 | 1,022 |
| CMIP | c-Maf inducing protein | **0,841** | 4,00E-06 | 23,042 | 27,328 |
| SLC7A6 | solute carrier family 7 member 6 | **0,841** | 0,000214 | 29,634 | 22,794 |
| SPATA7 | spermatogenesis associated 7 | **0,841** | 0,08315 | 1,606 | 1,341 |
| MRPL17 | mitochondrial ribosomal protein L17 | **0,841** | 0 | 31,572 | 31,861 |
| HOXA9 | homeobox A9 | **0,841** | 0,00032 | 8,198 | 7,790 |
| LCTL | lactase like | **0,841** | 0,328908 | 1,108 | 0,192 |
| SUV39H1 | suppressor of variegation 3-9 homolog 1 | **0,841** | 0,013824 | 3,711 | 2,809 |
| CCDC110 | coiled-coil domain containing 110 | **0,840** | 0,323978 | 0,554 | 0,383 |
| SREBF1 | sterol regulatory element binding transcription factor 1 | **0,840** | 0 | 25,701 | 24,901 |
| PUDP | pseudouridine 5'-phosphatase | **0,840** | 0,000183 | 12,186 | 8,620 |
| SYNGR3 | synaptogyrin 3 | **0,840** | 0,029341 | 4,542 | 1,724 |
| SNAI3-AS1 | SNAI3 antisense RNA 1 | **0,840** | 0,010059 | 3,102 | 2,490 |
| BNIP1 | BCL2 interacting protein 1 | **0,840** | 2,60E-05 | 7,478 | 8,492 |
| TRIP6 | thyroid hormone receptor interactor 6 | **0,840** | 3,00E-06 | 54,449 | 34,160 |
| RANBP1 | RAN binding protein 1 | **0,840** | 1,00E-06 | 23,541 | 19,985 |
| LIMK1 | LIM domain kinase 1 | **0,840** | 0,000117 | 14,734 | 10,663 |
| PTGS2 | prostaglandin-endoperoxide synthase 2 | **0,840** | 6,00E-06 | 950,384 | 497,518 |
| USP21 | ubiquitin specific peptidase 21 | **0,840** | 2,60E-05 | 11,355 | 8,939 |
| HYKK | hydroxylysine kinase | **0,840** | 0,248667 | 0,775 | 0,766 |
| HPF1 | histone PARylation factor 1 | **0,840** | 1,20E-05 | 9,084 | 7,981 |
| GPR180 | G protein-coupled receptor 180 | **0,839** | 8,00E-06 | 20,993 | 13,025 |
| FAM173A | family with sequence similarity 173 member A | **0,839** | 0,000129 | 9,582 | 7,470 |
| ZNF528-AS1 | ZNF528 antisense RNA 1 | **0,839** | 0,25182 | 0,942 | 1,341 |
| CYB561 | cytochrome b561 | **0,839** | 5,80E-05 | 18,888 | 12,706 |
| RABAC1 | Rab acceptor 1 | **0,839** | 0,000216 | 19,940 | 11,685 |
| POP1 | POP1 homolog, ribonuclease P/MRP subunit | **0,839** | 0,001324 | 8,309 | 7,087 |
| C3orf18 | chromosome 3 open reading frame 18 | **0,839** | 7,40E-05 | 7,090 | 4,980 |
| SCRN2 | secernin 2 | **0,839** | 0,000928 | 6,702 | 2,809 |
| IFT27 | intraflagellar transport 27 | **0,839** | 1,00E-06 | 15,177 | 14,366 |
| MACC1 | MACC1, MET transcriptional regulator | **0,839** | 0,098619 | 0,831 | 1,213 |
| FCRLB | Fc receptor like B | **0,839** | 0,171634 | 0,499 | 0,575 |

| | | | | | |
|---|---|---|---|---|---|
| CHKB | choline kinase beta | **0,839** | 0,214602 | 0,665 | 1,085 |
| FIBP | FGF1 intracellular binding protein | **0,839** | 0 | 36,170 | 34,096 |
| PECR | peroxisomal trans-2-enoyl-CoA reductase | **0,839** | 0,040188 | 2,437 | 3,959 |
| LINC00941 | long intergenic non-protein coding RNA 941 | **0,838** | 0,402182 | 0,443 | 0,383 |
| RPS6KA4 | ribosomal protein S6 kinase A4 | **0,838** | 4,70E-05 | 16,119 | 11,493 |
| SIPA1L3 | signal induced proliferation associated 1 like 3 | **0,838** | 0 | 38,441 | 33,138 |
| BRSK1 | BR serine/threonine kinase 1 | **0,838** | 0,298311 | 0,499 | 0,638 |
| CCDC74B | coiled-coil domain containing 74B | **0,838** | 0,303163 | 0,775 | 0,064 |
| CUL7 | cullin 7 | **0,838** | 0 | 61,982 | 48,717 |
| FAT4 | FAT atypical cadherin 4 | **0,838** | 0,000325 | 18,556 | 14,685 |
| CD163L1 | CD163 molecule like 1 | **0,838** | 0,273207 | 0,499 | 0,447 |
| FAM21EP | family with sequence similarity 21 member E, pseudogene | **0,838** | 0,257535 | 0,554 | 0,447 |
| MBOAT7 | membrane bound O-acyltransferase domain containing 7 | **0,838** | 0,301564 | 0,332 | 0,638 |
| CD38 | CD38 molecule | **0,838** | 0,003612 | 5,650 | 4,278 |
| DBP | D-box binding PAR bZIP transcription factor | **0,838** | 0,001237 | 3,323 | 1,788 |
| TMED1 | transmembrane p24 trafficking protein 1 | **0,838** | 0 | 32,459 | 22,156 |
| ZDHHC8 | zinc finger DHHC-type containing 8 | **0,838** | 0 | 26,476 | 16,984 |
| MAB21L1 | mab-21 like 1 | **0,837** | 0,157527 | 1,551 | 1,085 |
| GAREM2 | GRB2 associated regulator of MAPK1 subtype 2 | **0,837** | 0,000106 | 8,198 | 6,321 |
| LINC00623 | long intergenic non-protein coding RNA 623 | **0,837** | 0,037533 | 1,551 | 1,341 |
| PDE7B | phosphodiesterase 7B | **0,837** | 0,03296 | 2,880 | 2,107 |
| EXOSC4 | exosome component 4 | **0,837** | 0,000238 | 10,026 | 6,896 |
| TES | testin LIM domain protein | **0,837** | 0 | 63,145 | 50,952 |
| RPP40 | ribonuclease P/MRP subunit p40 | **0,837** | 0,007144 | 3,157 | 3,831 |
| TMEM214 | transmembrane protein 214 | **0,837** | 0 | 83,695 | 59,827 |
| PREX2 | phosphatidylinositol-3,4,5-trisphosphate dependent Rac exchange factor 2 | **0,837** | 0,294393 | 0,166 | 0,702 |
| TRIM16L | tripartite motif containing 16 like | **0,837** | 0,011233 | 4,265 | 1,788 |
| ATP5G2 | ATP synthase, H+ transporting, mitochondrial Fo complex subunit C2 (subunit 9) | **0,837** | 0 | 41,930 | 32,180 |
| POLR3D | RNA polymerase III subunit D | **0,837** | 0 | 34,730 | 29,116 |
| PDE7A | phosphodiesterase 7A | **0,837** | 0,000286 | 12,740 | 8,173 |
| DESI1 | desumoylating isopeptidase 1 | **0,837** | 0 | 32,957 | 25,540 |
| HMMR | hyaluronan mediated motility receptor | **0,837** | 0,121745 | 5,151 | 0,830 |
| KDELC1 | KDEL motif containing 1 | **0,836** | 0,012355 | 5,761 | 4,023 |

| | | | | | |
|---|---|---|---|---|---|
| UQCC3 | ubiquinol-cytochrome c reductase complex assembly factor 3 | **0,836** | 0,000189 | 7,035 | 6,321 |
| CTSW | cathepsin W | **0,836** | 4,80E-05 | 5,982 | 5,363 |
| TNC | tenascin C | **0,836** | 2,00E-06 | 559,773 | 841,861 |
| C3AR1 | complement C3a receptor 1 | **0,836** | 0,014035 | 3,711 | 6,193 |
| TMEM208 | transmembrane protein 208 | **0,836** | 6,00E-06 | 20,882 | 20,496 |
| ZNF726 | zinc finger protein 726 | **0,836** | 0,364084 | 0,609 | 0,192 |
| NOB1 | NIN1/PSMD8 binding protein 1 homolog | **0,836** | 0 | 24,926 | 26,817 |
| KCNAB3 | potassium voltage-gated channel subfamily A regulatory beta subunit 3 | **0,836** | 0,342238 | 0,222 | 0,383 |
| PCYOX1L | prenylcysteine oxidase 1 like | **0,836** | 0,00076 | 4,597 | 3,959 |
| PDCD2L | programmed cell death 2 like | **0,836** | 0,087144 | 1,606 | 1,660 |
| BMS1P1 | BMS1, ribosome biogenesis factor pseudogene 1 | **0,836** | 0,180422 | 0,775 | 0,830 |
| SLC25A1 | solute carrier family 25 member 1 | **0,836** | 2,00E-06 | 32,237 | 17,367 |
| B4GALT7 | beta-1,4-galactosyltransferase 7 | **0,836** | 6,00E-06 | 27,197 | 18,389 |
| CEND1 | cell cycle exit and neuronal differentiation 1 | **0,836** | 0,204527 | 0,886 | 1,022 |
| HIPK2 | homeodomain interacting protein kinase 2 | **0,835** | 0,001526 | 110,005 | 63,339 |
| IFITM3 | interferon induced transmembrane protein 3 | **0,835** | 0 | 43,869 | 40,928 |
| TBC1D31 | TBC1 domain family member 31 | **0,835** | 0,063791 | 2,603 | 2,554 |
| ZNF876P | zinc finger protein 876, pseudogene | **0,835** | 0,233462 | 0,775 | 1,277 |
| FBXL19 | F-box and leucine rich repeat protein 19 | **0,835** | 0,000143 | 10,856 | 7,151 |
| STON1 | stonin 1 | **0,835** | 0,000354 | 6,924 | 4,725 |
| EPG5 | ectopic P-granules autophagy protein 5 homolog | **0,835** | 1,20E-05 | 106,571 | 81,792 |
| EDARADD | EDAR associated death domain | **0,835** | 0,265875 | 0,554 | 0,128 |
| CST1 | cystatin SN | **0,835** | 0,000204 | 0,665 | 1,022 |
| PFAS | phosphoribosylformylglycinamidine synthase | **0,835** | 0,000189 | 7,699 | 7,854 |
| TOB2P1 | transducer of ERBB2, 2 pseudogene 1 | **0,835** | 0,169422 | 1,108 | 0,766 |
| MRTO4 | MRT4 homolog, ribosome maturation factor | **0,835** | 0 | 24,593 | 26,434 |
| PLPPR2 | phospholipid phosphatase related 2 | **0,835** | 4,00E-06 | 28,803 | 20,432 |
| RAD54L | RAD54 like (S. cerevisiae) | **0,835** | 0,395132 | 0,997 | 0,064 |
| ACTB | actin beta | **0,835** | 0 | 840,712 | 608,298 |
| BUB1 | BUB1 mitotic checkpoint serine/threonine kinase | **0,835** | 0,099535 | 5,816 | 0,766 |
| C12orf75 | chromosome 12 open reading frame 75 | **0,834** | 0,000967 | 12,186 | 4,086 |
| LINC01002 | long intergenic non-protein coding RNA 1002 | **0,834** | 0,237746 | 0,554 | 0,255 |
| PAGR1 | PAXIP1 associated glutamate rich protein 1 | **0,834** | 0,005664 | 3,046 | 2,426 |

| | | | | | |
|---|---|---|---|---|---|
| PTOV1-AS1 | PTOV1 antisense RNA 1 | **0,834** | 0,056707 | 1,551 | 1,532 |
| EIF6 | eukaryotic translation initiation factor 6 | **0,834** | 0 | 43,924 | 38,693 |
| CSNK1G2 | casein kinase 1 gamma 2 | **0,834** | 0 | 36,558 | 22,922 |
| GBGT1 | globoside alpha-1,3-N-acetylgalactosaminyltransferase 1 (FORS blood group) | **0,834** | 0,016506 | 3,102 | 2,554 |
| NOP16 | NOP16 nucleolar protein | **0,834** | 0,000752 | 12,684 | 14,239 |
| SRPRB | SRP receptor beta subunit | **0,834** | 0 | 72,284 | 66,787 |
| TEAD4 | TEA domain transcription factor 4 | **0,834** | 0,000328 | 11,577 | 5,555 |
| STAMBPL1 | STAM binding protein like 1 | **0,834** | 0,00405 | 5,041 | 4,342 |
| SSR2 | signal sequence receptor subunit 2 | **0,834** | 0 | 154,649 | 103,118 |
| SKIDA1 | SKI/DACH domain containing 1 | **0,834** | 0,330666 | 0,609 | 0,255 |
| PCDHB7 | protocadherin beta 7 | **0,834** | 0,074631 | 1,496 | 1,277 |
| VSIG2 | V-set and immunoglobulin domain containing 2 | **0,834** | 0,319433 | 0,443 | 0,638 |
| ANAPC13 | anaphase promoting complex subunit 13 | **0,834** | 8,00E-06 | 34,508 | 26,817 |
| SLC27A5 | solute carrier family 27 member 5 | **0,834** | 0,250266 | 0,942 | 0,766 |
| SBK3 | SH3 domain binding kinase family member 3 | **0,833** | 0,116506 | 0,997 | 1,149 |
| C19orf48 | chromosome 19 open reading frame 48 | **0,833** | 0 | 12,851 | 16,218 |
| LMF1 | lipase maturation factor 1 | **0,833** | 0,000129 | 9,416 | 6,066 |
| MDH1B | malate dehydrogenase 1B | **0,833** | 0,232772 | 0,720 | 0,319 |
| ARF4-AS1 | ARF4 antisense RNA 1 | **0,833** | 0,073649 | 1,052 | 1,277 |
| FAM98A | family with sequence similarity 98 member A | **0,833** | 0 | 56,830 | 46,100 |
| CA12 | carbonic anhydrase 12 | **0,833** | 3,40E-05 | 12,075 | 25,859 |
| TBX6 | T-box 6 | **0,833** | 0,260532 | 0,277 | 0,638 |
| C16orf71 | chromosome 16 open reading frame 71 | **0,833** | 0,024765 | 1,939 | 1,277 |
| CCDC102A | coiled-coil domain containing 102A | **0,833** | 0,001349 | 4,819 | 2,937 |
| BTBD2 | BTB domain containing 2 | **0,833** | 0 | 28,027 | 16,729 |
| FAAP20 | Fanconi anemia core complex associated protein 20 | **0,833** | 3,00E-06 | 24,316 | 14,494 |
| PREX1 | phosphatidylinositol-3,4,5-trisphosphate dependent Rac exchange factor 1 | **0,833** | 0 | 99,979 | 58,295 |
| HM13 | histocompatibility minor 13 | **0,833** | 0 | 159,191 | 121,634 |
| ARMCX2 | armadillo repeat containing, X-linked 2 | **0,833** | 0 | 50,017 | 33,585 |
| GAMT | guanidinoacetate N-methyltransferase | **0,833** | 0,300593 | 0,665 | 0,383 |
| FAM227A | family with sequence similarity 227 member A | **0,833** | 0,181253 | 0,942 | 1,341 |
| CHST12 | carbohydrate sulfotransferase 12 | **0,833** | 0 | 27,363 | 15,579 |
| MTHFD1L | methylenetetrahydrofolate dehydrogenase (NADP+ dependent) 1-like | **0,833** | 0 | 62,923 | 56,379 |

| | | | | | |
|---|---|---|---|---|---|
| ALDH1B1 | aldehyde dehydrogenase 1 family member B1 | **0,833** | 2,70E-05 | 9,693 | 8,364 |
| PARL | presenilin associated rhomboid like | **0,832** | 0,003232 | 6,259 | 6,193 |
| TOLLIP-AS1 | TOLLIP antisense RNA 1 (head to head) | **0,832** | 0,1189 | 0,775 | 1,085 |
| OLMALINC | oligodendrocyte maturation-associated long intergenic non-coding RNA | **0,832** | 0,002153 | 6,259 | 4,533 |
| RAB34 | RAB34, member RAS oncogene family | **0,832** | 0 | 44,368 | 28,413 |
| SYDE1 | synapse defective Rho GTPase homolog 1 | **0,832** | 0 | 63,976 | 37,863 |
| TFPI | tissue factor pathway inhibitor | **0,832** | 0 | 54,449 | 21,007 |
| GMPPB | GDP-mannose pyrophosphorylase B | **0,832** | 0 | 50,017 | 42,205 |
| STC1 | stanniocalcin 1 | **0,832** | 0,066122 | 1,939 | 0,575 |
| SDR16C5 | short chain dehydrogenase/reductase family 16C member 5 | **0,832** | 0,217962 | 0,332 | 0,255 |
| ZNF485 | zinc finger protein 485 | **0,831** | 0,049063 | 1,551 | 1,596 |
| MAMDC4 | MAM domain containing 4 | **0,831** | 0,000194 | 8,142 | 7,981 |
| PTPMT1 | protein tyrosine phosphatase, mitochondrial 1 | **0,831** | 0,003757 | 4,265 | 3,256 |
| LMCD1 | LIM and cysteine rich domains 1 | **0,831** | 4,80E-05 | 10,192 | 11,238 |
| PRMT5 | protein arginine methyltransferase 5 | **0,831** | 0 | 44,091 | 39,587 |
| HMBS | hydroxymethylbilane synthase | **0,831** | 0,003195 | 6,979 | 5,938 |
| JOSD2 | Josephin domain containing 2 | **0,831** | 2,00E-06 | 21,602 | 13,153 |
| C11orf24 | chromosome 11 open reading frame 24 | **0,830** | 0 | 34,785 | 21,198 |
| DNAH5 | dynein axonemal heavy chain 5 | **0,830** | 0,015195 | 2,825 | 3,639 |
| TWIST1 | twist family bHLH transcription factor 1 | **0,830** | 0,008664 | 3,157 | 1,915 |
| KLHL34 | kelch like family member 34 | **0,830** | 0,108515 | 0,886 | 0,638 |
| COL8A1 | collagen type VIII alpha 1 chain | **0,830** | 0,164311 | 0,443 | 0,958 |
| ALG1 | ALG1, chitobiosyldiphosphodolichol beta-mannosyltransferase | **0,830** | 4,00E-06 | 13,681 | 12,898 |
| CDK5 | cyclin dependent kinase 5 | **0,830** | 0,007346 | 3,656 | 2,362 |
| ZBED3 | zinc finger BED-type containing 3 | **0,830** | 0,148331 | 1,274 | 0,766 |
| BSCL2 | BSCL2, seipin lipid droplet biogenesis associated | **0,830** | 0,143292 | 0,997 | 1,085 |
| CHCHD4 | coiled-coil-helix-coiled-coil-helix domain containing 4 | **0,829** | 4,00E-06 | 9,195 | 9,386 |
| ANXA8L1 | annexin A8 like 1 | **0,829** | 0,311238 | 0,720 | 0,511 |
| AFAP1L1 | actin filament associated protein 1 like 1 | **0,829** | 0,293951 | 0,554 | 0,383 |
| OXNAD1 | oxidoreductase NAD binding domain containing 1 | **0,829** | 0,001317 | 5,761 | 4,023 |
| GALNT5 | polypeptide N-acetylgalactosaminyltransferase 5 | **0,829** | 0,004061 | 7,035 | 2,937 |
| EPHA2 | EPH receptor A2 | **0,829** | 0 | 40,435 | 70,363 |
| METAP1D | methionyl aminopeptidase type 1D, mitochondrial | **0,829** | 0,00952 | 2,603 | 3,512 |

| | | | | | |
|---|---|---|---|---|---|
| PPP1R35 | protein phosphatase 1 regulatory subunit 35 | **0,829** | 0,001049 | 7,976 | 4,597 |
| XKR8 | XK related 8 | **0,829** | 0,000275 | 11,521 | 5,619 |
| KIAA1211L | KIAA1211 like | **0,829** | 0,161554 | 0,554 | 0,638 |
| TRAM2 | translocation associated membrane protein 2 | **0,829** | 0 | 129,391 | 120,038 |
| MMP24-AS1 | MMP24 antisense RNA 1 | **0,829** | 4,00E-06 | 35,117 | 21,773 |
| LIMD2 | LIM domain containing 2 | **0,829** | 0,025601 | 2,991 | 1,532 |
| EEF1AKMT3 | EEF1A lysine methyltransferase 3 | **0,828** | 0,00335 | 6,038 | 4,342 |
| ZNF503-AS2 | ZNF503 antisense RNA 2 | **0,828** | 0,039336 | 3,046 | 2,937 |
| NT5C3B | 5'-nucleotidase, cytosolic IIIB | **0,828** | 0 | 23,153 | 11,812 |
| CCDC150P1 | coiled-coil domain containing 150 pseudogene 1 | **0,828** | 0,053515 | 1,108 | 1,532 |
| LPXN | leupaxin | **0,828** | 0 | 24,704 | 16,346 |
| ENC1 | ectodermal-neural cortex 1 | **0,828** | 8,10E-05 | 21,658 | 16,282 |
| CYSLTR1 | cysteinyl leukotriene receptor 1 | **0,828** | 0,172314 | 0,942 | 0,447 |
| TXNRD2 | thioredoxin reductase 2 | **0,828** | 4,00E-06 | 12,130 | 12,068 |
| LINC02432 | long intergenic non-protein coding RNA 2432 | **0,828** | 0,187927 | 0,443 | 0,447 |
| AMDHD1 | amidohydrolase domain containing 1 | **0,828** | 0,074122 | 1,108 | 1,915 |
| COA4 | cytochrome c oxidase assembly factor 4 homolog | **0,828** | 0 | 22,322 | 20,879 |
| SFXN2 | sideroflexin 2 | **0,828** | 0,024912 | 1,551 | 3,256 |
| EPB41L4A-AS2 | EPB41L4A antisense RNA 2 (head to head) | **0,827** | 0,225505 | 0,277 | 0,447 |
| DTYMK | deoxythymidylate kinase | **0,827** | 0,00016 | 12,574 | 11,174 |
| TRIM6 | tripartite motif containing 6 | **0,827** | 0,275616 | 0,443 | 0,575 |
| VEGFA | vascular endothelial growth factor A | **0,827** | 0 | 436,419 | 370,840 |
| CHPF2 | chondroitin polymerizing factor 2 | **0,827** | 0 | 89,178 | 46,419 |
| SLC16A2 | solute carrier family 16 member 2 | **0,827** | 0,000444 | 15,011 | 5,491 |
| ABHD14A | abhydrolase domain containing 14A | **0,827** | 0,179893 | 0,942 | 0,638 |
| PLEKHG5 | pleckstrin homology and RhoGEF domain containing G5 | **0,827** | 0,26962 | 0,443 | 0,319 |
| CYP27A1 | cytochrome P450 family 27 subfamily A member 1 | **0,826** | 1,00E-06 | 45,918 | 28,477 |
| BBOF1 | basal body orientation factor 1 | **0,826** | 0,000585 | 7,090 | 5,300 |
| TXNL4A | thioredoxin like 4A | **0,826** | 0 | 31,572 | 24,391 |
| MYO1B | myosin IB | **0,826** | 0 | 56,553 | 46,291 |
| SLC25A10 | solute carrier family 25 member 10 | **0,826** | 0,145339 | 1,052 | 0,702 |
| RPL13AP25 | ribosomal protein L13a pseudogene 25 | **0,826** | 0,075569 | 2,160 | 0,702 |
| COPG1 | coatomer protein complex subunit gamma 1 | **0,826** | 0 | 184,283 | 117,037 |

| | | | | | |
|---|---|---|---|---|---|
| HOMER3 | homer scaffolding protein 3 | **0,826** | 1,30E-05 | 10,580 | 6,768 |
| TMEM160 | transmembrane protein 160 | **0,826** | 0,002047 | 4,708 | 2,299 |
| LINC02175 | long intergenic non-protein coding RNA 2175 | **0,825** | 0,305376 | 0,443 | 0,447 |
| LYSMD2 | LysM domain containing 2 | **0,825** | 9,50E-05 | 5,650 | 6,385 |
| ALDOC | aldolase, fructose-bisphosphate C | **0,825** | 0 | 41,709 | 36,905 |
| LMO2 | LIM domain only 2 | **0,825** | 0,001557 | 1,717 | 4,916 |
| COLCA2 | colorectal cancer associated 2 | **0,825** | 0,328482 | 0,166 | 0,511 |
| MRPL55 | mitochondrial ribosomal protein L55 | **0,825** | 8,80E-05 | 9,804 | 7,279 |
| GRK5 | G protein-coupled receptor kinase 5 | **0,824** | 0 | 43,758 | 46,994 |
| ARRB2 | arrestin beta 2 | **0,824** | 0,042033 | 1,772 | 1,596 |
| MAP4K2 | mitogen-activated protein kinase kinase kinase kinase 2 | **0,824** | 0,006115 | 3,268 | 1,724 |
| PDIA5 | protein disulfide isomerase family A member 5 | **0,824** | 7,00E-06 | 15,842 | 8,300 |
| C21orf2 | chromosome 21 open reading frame 2 | **0,824** | 6,50E-05 | 9,195 | 6,960 |
| GGN | gametogenetin | **0,824** | 0,110978 | 1,274 | 0,511 |
| DUS3L | dihydrouridine synthase 3 like | **0,823** | 0 | 7,422 | 10,791 |
| LRP5 | LDL receptor related protein 5 | **0,823** | 0 | 13,460 | 12,770 |
| VILL | villin like | **0,823** | 0,001275 | 7,312 | 4,980 |
| RUVBL1 | RuvB like AAA ATPase 1 | **0,823** | 0 | 19,664 | 19,793 |
| CCDC86 | coiled-coil domain containing 86 | **0,823** | 1,00E-06 | 14,789 | 13,345 |
| SEC13 | SEC13 homolog, nuclear pore and COPII coat complex component | **0,823** | 0 | 73,281 | 59,891 |
| ZNF462 | zinc finger protein 462 | **0,823** | 0,000465 | 9,416 | 9,961 |
| PLEKHG3 | pleckstrin homology and RhoGEF domain containing G3 | **0,822** | 1,00E-06 | 8,364 | 10,663 |
| KIAA1958 | KIAA1958 | **0,822** | 0,014137 | 2,603 | 1,915 |
| RRAS2 | related RAS viral (r-ras) oncogene homolog 2 | **0,822** | 0 | 33,899 | 24,327 |
| LOXL1-AS1 | LOXL1 antisense RNA 1 | **0,822** | 0,012176 | 2,659 | 1,660 |
| ARHGAP35 | Rho GTPase activating protein 35 | **0,822** | 0 | 112,054 | 57,146 |
| DHX37 | DEAH-box helicase 37 | **0,822** | 0 | 14,734 | 13,345 |
| RCL1 | RNA terminal phosphate cyclase like 1 | **0,822** | 4,00E-06 | 23,042 | 13,983 |
| ZNF414 | zinc finger protein 414 | **0,822** | 4,70E-05 | 6,093 | 6,066 |
| FAM86DP | family with sequence similarity 86 member D, pseudogene | **0,822** | 0,000246 | 7,367 | 7,534 |
| HOXD-AS2 | HOXD cluster antisense RNA 2 | **0,822** | 0,060046 | 1,772 | 1,022 |
| PSEN2 | presenilin 2 | **0,822** | 0,000625 | 8,253 | 5,746 |
| BEX3 | brain expressed X-linked 3 | **0,822** | 0,000704 | 9,804 | 3,320 |

| | | | | | |
|---|---|---|---|---|---|
| RN7SL832P | RNA, 7SL, cytoplasmic 832, pseudogene | **0,822** | 0,155661 | 1,052 | 1,277 |
| PRKAR2A-AS1 | PRKAR2A antisense RNA 1 | **0,822** | 0,032238 | 1,496 | 1,532 |
| TMEM17 | transmembrane protein 17 | **0,822** | 0,000563 | 4,985 | 2,362 |
| MMP19 | matrix metallopeptidase 19 | **0,821** | 0,070718 | 0,886 | 1,788 |
| CXCL3 | C-X-C motif chemokine ligand 3 | **0,821** | 0 | 215,135 | 192,060 |
| APOO | apolipoprotein O | **0,821** | 4,00E-06 | 17,005 | 10,854 |
| GPATCH4 | G-patch domain containing 4 | **0,821** | 0 | 22,544 | 19,538 |
| TBKBP1 | TBK1 binding protein 1 | **0,821** | 1,80E-05 | 12,463 | 9,514 |
| SNX22 | sorting nexin 22 | **0,821** | 0,277869 | 1,274 | 1,788 |
| DDX12P | DEAD/H-box helicase 12, pseudogene | **0,821** | 0,103722 | 0,775 | 0,575 |
| LRRC61 | leucine rich repeat containing 61 | **0,821** | 0,016413 | 3,379 | 3,512 |
| CCDC184 | coiled-coil domain containing 184 | **0,821** | 0,000285 | 5,816 | 3,831 |
| ATAD3A | ATPase family, AAA domain containing 3A | **0,821** | 1,40E-05 | 14,845 | 12,323 |
| LIPA | lipase A, lysosomal acid type | **0,821** | 1,40E-05 | 12,075 | 9,067 |
| PAQR7 | progestin and adipoQ receptor family member 7 | **0,821** | 0,000337 | 4,764 | 3,129 |
| AP2S1 | adaptor related protein complex 2 sigma 1 subunit | **0,821** | 0 | 27,197 | 15,771 |
| FBN2 | fibrillin 2 | **0,821** | 0,025752 | 2,659 | 1,341 |
| VASH1-AS1 | VASH1 antisense RNA 1 | **0,820** | 0,095757 | 0,831 | 0,383 |
| JMJD1C-AS1 | JMJD1C antisense RNA 1 | **0,820** | 0,07517 | 1,717 | 1,085 |
| SLC11A1 | solute carrier family 11 member 1 | **0,820** | 0,256429 | 0,277 | 0,383 |
| GADD45GIP1 | GADD45G interacting protein 1 | **0,820** | 2,00E-06 | 29,911 | 26,178 |
| CDCA3 | cell division cycle associated 3 | **0,820** | 0,075526 | 3,102 | 1,596 |
| ZGPAT | zinc finger CCCH-type and G-patch domain containing | **0,820** | 0,012784 | 3,545 | 2,746 |
| C5orf38 | chromosome 5 open reading frame 38 | **0,820** | 0,176688 | 0,277 | 0,575 |
| NT5DC1 | 5'-nucleotidase domain containing 1 | **0,820** | 6,10E-05 | 11,023 | 6,002 |
| POLR2J4 | RNA polymerase II subunit J4, pseudogene | **0,820** | 0,16963 | 0,665 | 0,447 |
| SLC25A20 | solute carrier family 25 member 20 | **0,820** | 0,000161 | 7,865 | 4,406 |
| FAM207A | family with sequence similarity 207 member A | **0,820** | 5,80E-05 | 10,192 | 8,045 |
| SMCO4 | single-pass membrane protein with coiled-coil domains 4 | **0,820** | 0,001044 | 5,262 | 3,959 |
| PDE4A | phosphodiesterase 4A | **0,820** | 0,003422 | 5,373 | 2,043 |
| BEST3 | bestrophin 3 | **0,819** | 0,120373 | 0,222 | 0,702 |
| HSD17B10 | hydroxysteroid 17-beta dehydrogenase 10 | **0,819** | 0 | 27,529 | 20,432 |
| MFAP4 | microfibrillar associated protein 4 | **0,819** | 0,059107 | 1,440 | 0,830 |

| | | | | | |
|---|---|---|---|---|---|
| KDELR1 | KDEL endoplasmic reticulum protein retention receptor 1 | **0,819** | 0 | 102,084 | 58,806 |
| EXOSC2 | exosome component 2 | **0,819** | 3,50E-05 | 11,743 | 12,642 |
| NUDT22 | nudix hydrolase 22 | **0,819** | 5,30E-05 | 11,632 | 6,385 |
| PLIN3 | perilipin 3 | **0,819** | 0 | 81,257 | 43,929 |
| C4orf48 | chromosome 4 open reading frame 48 | **0,819** | 0,000673 | 8,475 | 5,619 |
| CDKN2B-AS1 | CDKN2B antisense RNA 1 | **0,819** | 0,252022 | 0,886 | 0,255 |
| TBX5 | T-box 5 | **0,819** | 0,105124 | 0,499 | 1,469 |
| CENPK | centromere protein K | **0,818** | 0,021978 | 2,880 | 1,724 |
| CAMTA2 | calmodulin binding transcription activator 2 | **0,818** | 0 | 49,796 | 40,545 |
| SSH1 | slingshot protein phosphatase 1 | **0,818** | 0 | 116,042 | 111,418 |
| FBXO5 | F-box protein 5 | **0,818** | 0,000493 | 6,148 | 4,214 |
| CHEK2 | checkpoint kinase 2 | **0,818** | 0,000139 | 4,265 | 5,363 |
| SLC35A2 | solute carrier family 35 member A2 | **0,818** | 0 | 36,225 | 24,071 |
| LINC00160 | long intergenic non-protein coding RNA 160 | **0,818** | 0,058494 | 0,886 | 0,830 |
| MIR31HG | MIR31 host gene | **0,818** | 0,016233 | 4,487 | 1,532 |
| TRIM46 | tripartite motif containing 46 | **0,818** | 0,09896 | 0,775 | 0,575 |
| MTMR9LP | myotubularin related protein 9-like, pseudogene | **0,818** | 0,094432 | 0,720 | 0,575 |
| NUDT6 | nudix hydrolase 6 | **0,818** | 0,014066 | 2,770 | 3,192 |
| PMF1 | polyamine modulated factor 1 | **0,817** | 0,000208 | 3,933 | 4,342 |
| TSPAN4 | tetraspanin 4 | **0,817** | 0 | 98,539 | 55,932 |
| FZD9 | frizzled class receptor 9 | **0,817** | 0 | 85,079 | 36,267 |
| ROBO3 | roundabout guidance receptor 3 | **0,817** | 0,015929 | 1,606 | 2,171 |
| PLPP7 | phospholipid phosphatase 7 (inactive) | **0,817** | 0,002782 | 4,099 | 2,171 |
| CIRBP-AS1 | CIRBP antisense RNA 1 | **0,817** | 0,056015 | 0,886 | 0,766 |
| RAB38 | RAB38, member RAS oncogene family | **0,817** | 1,80E-05 | 8,752 | 6,321 |
| MAP2K6 | mitogen-activated protein kinase kinase 6 | **0,816** | 0,019634 | 1,274 | 2,746 |
| IKBIP | IKBKB interacting protein | **0,816** | 0 | 34,730 | 18,070 |
| KLHDC8B | kelch domain containing 8B | **0,816** | 0,013939 | 3,434 | 1,852 |
| RNF215 | ring finger protein 215 | **0,816** | 0 | 21,935 | 19,410 |
| DOK6 | docking protein 6 | **0,816** | 0,092914 | 2,049 | 0,383 |
| ZNF652 | zinc finger protein 652 | **0,816** | 9,00E-05 | 8,364 | 5,108 |
| MUS81 | MUS81 structure-specific endonuclease subunit | **0,816** | 0 | 19,719 | 15,196 |
| EIF2AK4 | eukaryotic translation initiation factor 2 alpha kinase 4 | **0,816** | 9,00E-06 | 17,669 | 11,365 |

| | | | | | |
|---|---|---|---|---|---|
| MLST8 | MTOR associated protein, LST8 homolog | **0,816** | 8,00E-06 | 15,343 | 12,706 |
| CDK18 | cyclin dependent kinase 18 | **0,816** | 0,098999 | 0,665 | 0,383 |
| CCDC8 | coiled-coil domain containing 8 | **0,816** | 0,000163 | 7,090 | 5,427 |
| MOGS | mannosyl-oligosaccharide glucosidase | **0,816** | 0 | 53,341 | 35,947 |
| SIGIRR | single Ig and TIR domain containing | **0,815** | 6,00E-04 | 4,210 | 3,703 |
| ARHGAP29 | Rho GTPase activating protein 29 | **0,815** | 0 | 22,211 | 16,218 |
| ARHGEF33 | Rho guanine nucleotide exchange factor 33 | **0,815** | 0,098932 | 0,831 | 0,575 |
| C6orf58 | chromosome 6 open reading frame 58 | **0,815** | 0,033952 | 2,659 | 1,788 |
| ARMC6 | armadillo repeat containing 6 | **0,815** | 0 | 12,075 | 10,535 |
| GTF3C6 | general transcription factor IIIC subunit 6 | **0,815** | 0 | 28,471 | 22,475 |
| C15orf52 | chromosome 15 open reading frame 52 | **0,815** | 0,065331 | 2,105 | 0,255 |
| CCDC136 | coiled-coil domain containing 136 | **0,815** | 0,078635 | 2,271 | 1,469 |
| TMED3 | transmembrane p24 trafficking protein 3 | **0,814** | 0 | 77,879 | 44,312 |
| PRORSD1P | prolyl-tRNA synthetase associated domain containing 1, pseudogene | **0,814** | 0,077369 | 1,274 | 1,085 |
| FSCN1 | fascin actin-bundling protein 1 | **0,814** | 3,00E-06 | 37,665 | 27,902 |
| DOCK9-AS2 | DOCK9 antisense RNA 2 (head to head) | **0,814** | 0,117742 | 0,775 | 0,638 |
| MT-TI | mitochondrially encoded tRNA isoleucine | **0,814** | 0,267421 | 0,332 | 0,511 |
| ECT2 | epithelial cell transforming 2 | **0,814** | 6,00E-06 | 17,503 | 8,237 |
| ST7-AS1 | ST7 antisense RNA 1 | **0,814** | 0,141402 | 1,274 | 0,958 |
| PSMG3 | proteasome assembly chaperone 3 | **0,814** | 5,00E-06 | 7,312 | 7,534 |
| CDH3 | cadherin 3 | **0,813** | 0,00181 | 2,437 | 2,107 |
| FBXO27 | F-box protein 27 | **0,813** | 0,026214 | 2,326 | 1,405 |
| MRPS34 | mitochondrial ribosomal protein S34 | **0,813** | 2,00E-06 | 19,165 | 13,536 |
| RPS19BP1 | ribosomal protein S19 binding protein 1 | **0,813** | 0 | 33,400 | 24,263 |
| TADA2A | transcriptional adaptor 2A | **0,813** | 0,396502 | 0,554 | 0,319 |
| POLE2 | DNA polymerase epsilon 2, accessory subunit | **0,813** | 0,220934 | 0,609 | 0,319 |
| MYDGF | myeloid derived growth factor | **0,813** | 0 | 162,626 | 85,431 |
| NHP2 | NHP2 ribonucleoprotein | **0,813** | 1,00E-06 | 19,940 | 16,090 |
| MRPL24 | mitochondrial ribosomal protein L24 | **0,812** | 0 | 16,119 | 16,218 |
| IGDCC4 | immunoglobulin superfamily DCC subclass member 4 | **0,812** | 0,093694 | 2,437 | 1,149 |
| PLEKHA5 | pleckstrin homology domain containing A5 | **0,812** | 0,000133 | 8,142 | 4,469 |
| FEZ1 | fasciculation and elongation protein zeta 1 | **0,812** | 1,60E-05 | 13,903 | 7,662 |
| PANX2 | pannexin 2 | **0,812** | 2,00E-06 | 15,952 | 6,768 |

| | | | | | |
|---|---|---|---|---|---|
| MPST | mercaptopyruvate sulfurtransferase | **0,811** | 2,60E-05 | 9,749 | 6,960 |
| ZBTB8A | zinc finger and BTB domain containing 8A | **0,811** | 0,114259 | 1,551 | 1,022 |
| MTFP1 | mitochondrial fission process 1 | **0,811** | 0,257439 | 0,111 | 0,128 |
| HYAL3 | hyaluronoglucosaminidase 3 | **0,811** | 0,014856 | 1,828 | 1,213 |
| ALYREF | Aly/REF export factor | **0,811** | 0 | 18,279 | 16,090 |
| FAM53A | family with sequence similarity 53 member A | **0,811** | 0,056617 | 1,163 | 1,149 |
| ADORA1 | adenosine A1 receptor | **0,811** | 6,00E-06 | 6,148 | 11,174 |
| LMNB1 | lamin B1 | **0,811** | 0,011222 | 3,767 | 1,469 |
| FKBP14 | FK506 binding protein 14 | **0,811** | 0 | 32,791 | 22,539 |
| NFIB | nuclear factor I B | **0,810** | 0 | 20,993 | 16,026 |
| LINC00545 | long intergenic non-protein coding RNA 545 | **0,810** | 0,05088 | 1,329 | 1,405 |
| KIF7 | kinesin family member 7 | **0,810** | 0,001786 | 4,431 | 2,873 |
| EXO1 | exonuclease 1 | **0,810** | 0,195814 | 1,108 | 0,319 |
| PCDHB3 | protocadherin beta 3 | **0,810** | 0,089324 | 1,440 | 0,447 |
| NKX3-2 | NK3 homeobox 2 | **0,810** | 4,10E-05 | 8,419 | 5,044 |
| SLC46A1 | solute carrier family 46 member 1 | **0,810** | 1,10E-05 | 10,192 | 10,344 |
| EIF3EP1 | eukaryotic translation initiation factor 3 subunit E pseudogene 1 | **0,810** | 0,250769 | 0,388 | 0,383 |
| PEX11G | peroxisomal biogenesis factor 11 gamma | **0,810** | 0,038558 | 2,049 | 1,213 |
| TARBP2 | TARBP2, RISC loading complex RNA binding subunit | **0,810** | 4,00E-06 | 7,312 | 4,789 |
| SNCG | synuclein gamma | **0,809** | 0,110261 | 0,554 | 0,638 |
| SERPINH1 | serpin family H member 1 | **0,809** | 0 | 59,157 | 34,479 |
| GAS6 | growth arrest specific 6 | **0,809** | 0 | 118,313 | 50,186 |
| ANLN | anillin actin binding protein | **0,809** | 0,01759 | 21,104 | 1,405 |
| SP2-AS1 | SP2 antisense RNA 1 | **0,809** | 0,021414 | 1,496 | 1,469 |
| RABEPK | Rab9 effector protein with kelch motifs | **0,809** | 1,30E-05 | 8,973 | 6,385 |
| YIF1B | Yip1 interacting factor homolog B, membrane trafficking protein | **0,808** | 0 | 40,490 | 24,582 |
| EREG | epiregulin | **0,808** | 0 | 261,774 | 227,880 |
| CAPN1 | calpain 1 | **0,808** | 2,00E-06 | 18,777 | 8,300 |
| C3orf33 | chromosome 3 open reading frame 33 | **0,808** | 0,22594 | 0,388 | 0,319 |
| MGMT | O-6-methylguanine-DNA methyltransferase | **0,808** | 7,20E-05 | 3,933 | 4,023 |
| MAFG-AS1 | MAFG antisense RNA 1 (head to head) | **0,808** | 0,018166 | 1,828 | 1,915 |
| REP15 | RAB15 effector protein | **0,808** | 0,045812 | 1,108 | 1,085 |
| DPH2 | DPH2 homolog | **0,807** | 0 | 10,912 | 11,493 |

| | | | | | |
|---|---|---|---|---|---|
| ZNF521 | zinc finger protein 521 | **0,807** | 5,00E-06 | 24,704 | 14,558 |
| OPLAH | 5-oxoprolinase (ATP-hydrolysing) | **0,807** | 0,000158 | 9,859 | 5,300 |
| RAB42 | RAB42, member RAS oncogene family | **0,807** | 0,079016 | 1,440 | 0,255 |
| HOXA1 | homeobox A1 | **0,807** | 0,080937 | 1,385 | 0,830 |
| FABP5 | fatty acid binding protein 5 | **0,807** | 0,014094 | 3,268 | 1,660 |
| SLC17A9 | solute carrier family 17 member 9 | **0,807** | 6,80E-05 | 6,370 | 3,448 |
| ETV4 | ETS variant 4 | **0,806** | 0,036405 | 1,939 | 1,022 |
| FAM86EP | family with sequence similarity 86 member E, pseudogene | **0,806** | 0,004121 | 1,496 | 2,299 |
| NME4 | NME/NM23 nucleoside diphosphate kinase 4 | **0,806** | 0,011633 | 3,988 | 1,724 |
| DMTN | dematin actin binding protein | **0,806** | 0,213465 | 0,499 | 0,575 |
| NCLN | nicalin | **0,806** | 0 | 64,197 | 51,718 |
| TTC4P1 | tetratricopeptide repeat domain 4 pseudogene 1 | **0,806** | 0,253626 | 0,388 | 0,064 |
| PABPC4L | poly(A) binding protein cytoplasmic 4 like | **0,806** | 0,357012 | 0,609 | 0,894 |
| MEX3A | mex-3 RNA binding family member A | **0,806** | 0,110975 | 0,720 | 0,766 |
| FAM198B | family with sequence similarity 198 member B | **0,806** | 0,018147 | 1,496 | 1,724 |
| AGMAT | agmatinase | **0,806** | 0,240187 | 0,443 | 0,255 |
| SLC4A8 | solute carrier family 4 member 8 | **0,806** | 0,141977 | 1,717 | 0,766 |
| CDC20 | cell division cycle 20 | **0,805** | 0,019718 | 6,204 | 0,766 |
| C9orf172 | chromosome 9 open reading frame 172 | **0,805** | 0,00432 | 2,548 | 1,405 |
| LIPG | lipase G, endothelial type | **0,805** | 0,019184 | 3,213 | 1,724 |
| XYLT2 | xylosyltransferase 2 | **0,805** | 0 | 11,466 | 6,768 |
| FAM114A1 | family with sequence similarity 114 member A1 | **0,805** | 0 | 101,807 | 45,844 |
| SWSAP1 | SWIM-type zinc finger 7 associated protein 1 | **0,805** | 0,078644 | 1,219 | 0,766 |
| ADD3 | adducin 3 | **0,804** | 0 | 47,691 | 19,793 |
| LINC01134 | long intergenic non-protein coding RNA 1134 | **0,804** | 0,014298 | 2,603 | 1,979 |
| LIPT2 | lipoyl(octanoyl) transferase 2 (putative) | **0,804** | 0,166626 | 0,554 | 0,192 |
| PXN-AS1 | PXN antisense RNA 1 | **0,804** | 0,015641 | 1,385 | 1,149 |
| THSD1 | thrombospondin type 1 domain containing 1 | **0,804** | 0,002871 | 2,049 | 2,618 |
| SNHG20 | small nucleolar RNA host gene 20 | **0,804** | 0,011055 | 1,385 | 1,085 |
| TRIM52-AS1 | TRIM52 antisense RNA 1 (head to head) | **0,804** | 0,00186 | 3,323 | 3,129 |
| MTX1 | metaxin 1 | **0,804** | 0,004398 | 3,711 | 3,001 |
| MEDAG | mesenteric estrogen dependent adipogenesis | **0,804** | 0 | 135,706 | 146,982 |
| CHCHD6 | coiled-coil-helix-coiled-coil-helix domain containing 6 | **0,804** | 0,012072 | 2,936 | 1,469 |

| | | | | | |
|---|---|---|---|---|---|
| MGST2 | microsomal glutathione S-transferase 2 | **0,803** | 0,206189 | 0,388 | 0,575 |
| LNP1 | leukemia NUP98 fusion partner 1 | **0,803** | 0,003043 | 3,213 | 2,171 |
| UHRF1 | ubiquitin like with PHD and ring finger domains 1 | **0,803** | 0,061575 | 2,991 | 0,511 |
| CNKSR1 | connector enhancer of kinase suppressor of Ras 1 | **0,803** | 0,06338 | 0,665 | 1,341 |
| PITPNM1 | phosphatidylinositol transfer protein membrane associated 1 | **0,803** | 1,50E-05 | 18,943 | 9,003 |
| GRK3 | G protein-coupled receptor kinase 3 | **0,803** | 0,000153 | 6,702 | 5,044 |
| KPTN | kaptin, actin binding protein | **0,803** | 0,003145 | 3,213 | 2,937 |
| NDUFV2P1 | NADH:ubiquinone oxidoreductase core subunit V2 pseudogene 1 | **0,803** | 0,148878 | 0,720 | 0,319 |
| LINC00665 | long intergenic non-protein coding RNA 665 | **0,803** | 0,017425 | 1,606 | 1,213 |
| SC5D | sterol-C5-desaturase | **0,803** | 0 | 94,828 | 70,810 |
| SNRPA | small nuclear ribonucleoprotein polypeptide A | **0,803** | 0 | 20,494 | 19,219 |
| TRAIP | TRAF interacting protein | **0,803** | 0,031479 | 1,496 | 0,575 |
| RAB7B | RAB7B, member RAS oncogene family | **0,803** | 0,239369 | 1,052 | 1,022 |
| MAGOHB | mago homolog B, exon junction complex core component | **0,803** | 1,00E-05 | 6,425 | 5,044 |
| PPIB | peptidylprolyl isomerase B | **0,803** | 0 | 338,102 | 218,430 |
| PLEKHN1 | pleckstrin homology domain containing N1 | **0,803** | 0,000373 | 0,775 | 5,938 |
| DRAP1 | DR1 associated protein 1 | **0,802** | 0 | 135,318 | 117,228 |
| AGR2 | anterior gradient 2, protein disulphide isomerase family member | **0,802** | 4,00E-06 | 18,943 | 0,064 |
| TRIP13 | thyroid hormone receptor interactor 13 | **0,802** | 0,035821 | 2,271 | 0,638 |
| ZDHHC9 | zinc finger DHHC-type containing 9 | **0,801** | 0 | 27,806 | 17,239 |
| STAG3 | stromal antigen 3 | **0,801** | 0,220655 | 0,609 | 0,319 |
| MRPS17 | mitochondrial ribosomal protein S17 | **0,801** | 0,001386 | 2,659 | 2,746 |
| TIPARP | TCDD inducible poly(ADP-ribose) polymerase | **0,801** | 1,20E-05 | 52,399 | 26,881 |
| FKBP11 | FK506 binding protein 11 | **0,801** | 0 | 14,014 | 5,172 |
| GPR173 | G protein-coupled receptor 173 | **0,800** | 0,032931 | 0,997 | 1,085 |
| WDR12 | WD repeat domain 12 | **0,800** | 0 | 20,051 | 16,920 |
| NEXN | nexilin F-actin binding protein | **0,800** | 0,022343 | 3,268 | 0,638 |
| POLR3H | RNA polymerase III subunit H | **0,800** | 0 | 31,129 | 25,093 |
| SPAG4 | sperm associated antigen 4 | **0,800** | 0,008734 | 1,385 | 1,085 |
| EEF1A1P9 | eukaryotic translation elongation factor 1 alpha 1 pseudogene 9 | **0,800** | 0,02629 | 1,274 | 0,958 |
| NUBP1 | nucleotide binding protein 1 | **0,800** | 0 | 15,731 | 13,281 |
| BIRC5 | baculoviral IAP repeat containing 5 | **0,800** | 0,051656 | 6,314 | 0,255 |
| NPB | neuropeptide B | **0,800** | 0,064022 | 0,720 | 0,064 |

| | | | | | |
|---|---|---|---|---|---|
| RBM44 | RNA binding motif protein 44 | **0,799** | 0,288138 | 0,277 | 0,383 |
| LINC01852 | long intergenic non-protein coding RNA 1852 | **0,799** | 0,002883 | 1,828 | 2,426 |
| PEX10 | peroxisomal biogenesis factor 10 | **0,799** | 0 | 22,433 | 13,345 |
| SCFD2 | sec1 family domain containing 2 | **0,799** | 7,00E-05 | 4,819 | 4,533 |
| HNRNPA3P6 | heterogeneous nuclear ribonucleoprotein A3 pseudogene 6 | **0,799** | 0,246414 | 0,554 | 0,575 |
| SLC19A3 | solute carrier family 19 member 3 | **0,799** | 0 | 11,466 | 16,409 |
| TTC25 | tetratricopeptide repeat domain 25 | **0,799** | 0,00095 | 3,822 | 2,426 |
| PRPF40B | pre-mRNA processing factor 40 homolog B | **0,799** | 4,00E-06 | 7,201 | 7,151 |
| B4GALT2 | beta-1,4-galactosyltransferase 2 | **0,799** | 0 | 37,001 | 19,091 |
| TRMT1 | tRNA methyltransferase 1 | **0,799** | 0 | 13,460 | 12,706 |
| HSPBP1 | HSPA (Hsp70) binding protein 1 | **0,799** | 0 | 23,929 | 20,368 |
| LMO7 | LIM domain 7 | **0,799** | 0,000416 | 11,078 | 3,129 |
| MTHFD2L | methylenetetrahydrofolate dehydrogenase (NADP+ dependent) 2-like | **0,798** | 0 | 17,946 | 18,070 |
| C17orf53 | chromosome 17 open reading frame 53 | **0,798** | 0,087351 | 1,052 | 0,702 |
| NLE1 | notchless homolog 1 | **0,798** | 4,00E-06 | 9,250 | 10,216 |
| COQ3 | coenzyme Q3, methyltransferase | **0,798** | 0,006588 | 2,216 | 1,915 |
| HOXA10-AS | HOXA10 antisense RNA | **0,797** | 0,08098 | 1,717 | 1,596 |
| PAQR9-AS1 | PAQR9 antisense RNA 1 | **0,797** | 0,29698 | 0,222 | 0,511 |
| DGAT2 | diacylglycerol O-acyltransferase 2 | **0,797** | 0,001715 | 2,936 | 2,107 |
| SLC35F2 | solute carrier family 35 member F2 | **0,797** | 0,000139 | 4,597 | 3,703 |
| PCYT1B | phosphate cytidylyltransferase 1, choline, beta | **0,797** | 0,002513 | 3,600 | 2,554 |
| SUSD1 | sushi domain containing 1 | **0,797** | 7,00E-06 | 6,924 | 2,682 |
| GNB3 | G protein subunit beta 3 | **0,797** | 0,06742 | 0,997 | 0,830 |
| TRIQK | triple QxxK/R motif containing | **0,797** | 0 | 18,999 | 8,684 |
| ENG | endoglin | **0,797** | 0 | 133,158 | 89,454 |
| CLIC3 | chloride intracellular channel 3 | **0,797** | 0,00108 | 3,711 | 2,682 |
| LINC01269 | long intergenic non-protein coding RNA 1269 | **0,796** | 0,350359 | 0,665 | 0,447 |
| MRGPRX3 | MAS related GPR family member X3 | **0,796** | 0,000425 | 6,813 | 4,214 |
| LINC00856 | long intergenic non-protein coding RNA 856 | **0,796** | 0,171511 | 0,665 | 0,766 |
| CAMKMT | calmodulin-lysine N-methyltransferase | **0,795** | 0,038263 | 1,883 | 1,085 |
| CDK1 | cyclin dependent kinase 1 | **0,795** | 0,043846 | 6,425 | 0,128 |
| PDZK1IP1 | PDZK1 interacting protein 1 | **0,795** | 0 | 37,721 | 38,246 |
| HIC1 | HIC ZBTB transcriptional repressor 1 | **0,795** | 2,20E-05 | 3,656 | 5,427 |

| | | | | | |
|---|---|---|---|---|---|
| SAMD14 | sterile alpha motif domain containing 14 | **0,795** | 0,076857 | 1,274 | 0,383 |
| CD34 | CD34 molecule | **0,795** | 0,000847 | 2,991 | 2,809 |
| MPP3 | membrane palmitoylated protein 3 | **0,795** | 0,033329 | 2,548 | 1,341 |
| HEPH | hephaestin | **0,795** | 0,207747 | 0,831 | 0,255 |
| TRIM16 | tripartite motif containing 16 | **0,794** | 0,013573 | 1,717 | 1,277 |
| SGSM3 | small G protein signaling modulator 3 | **0,794** | 0,085412 | 0,443 | 0,766 |
| B3GNT9 | UDP-GlcNAc:betaGal beta-1,3-N-acetylglucosaminyltransferase 9 | **0,794** | 0 | 17,005 | 9,131 |
| PRR22 | proline rich 22 | **0,794** | 0,165081 | 0,665 | 0,511 |
| GCKR | glucokinase regulator | **0,794** | 0,165613 | 0,499 | 0,383 |
| CHST14 | carbohydrate sulfotransferase 14 | **0,794** | 0 | 21,048 | 11,429 |
| SULT1A1 | sulfotransferase family 1A member 1 | **0,794** | 0,235677 | 0,720 | 0,511 |
| IL1B | interleukin 1 beta | **0,794** | 0 | 475,469 | 717,162 |
| METTL1 | methyltransferase like 1 | **0,793** | 0,000381 | 5,373 | 6,513 |
| RPS13P2 | ribosomal protein S13 pseudogene 2 | **0,793** | 0,269077 | 0,443 | 0,064 |
| CARD8-AS1 | CARD8 antisense RNA 1 | **0,793** | 0,003901 | 1,994 | 1,724 |
| ZNF608 | zinc finger protein 608 | **0,793** | 0,000282 | 8,309 | 5,874 |
| HLA-DQB1 | major histocompatibility complex, class II, DQ beta 1 | **0,793** | 0,094931 | 0,388 | 0,894 |
| RRS1 | ribosome biogenesis regulator homolog | **0,793** | 0 | 14,678 | 17,878 |
| MEG9 | maternally expressed 9 (non-protein coding) | **0,793** | 0,079033 | 1,772 | 0,575 |
| TTC4 | tetratricopeptide repeat domain 4 | **0,793** | 0,052572 | 0,886 | 0,894 |
| ZNF837 | zinc finger protein 837 | **0,793** | 0,019599 | 0,886 | 1,149 |
| PCDHB18P | protocadherin beta 18 pseudogene | **0,792** | 0,260681 | 0,609 | 0,702 |
| AKR1B1 | aldo-keto reductase family 1 member B | **0,792** | 0 | 309,576 | 125,529 |
| SMAD3 | SMAD family member 3 | **0,792** | 0 | 133,324 | 105,416 |
| HOXA11 | homeobox A11 | **0,792** | 4,00E-06 | 12,629 | 7,470 |
| PITX1 | paired like homeodomain 1 | **0,792** | 0 | 79,208 | 58,997 |
| IKBKE | inhibitor of nuclear factor kappa B kinase subunit epsilon | **0,791** | 5,40E-05 | 5,262 | 3,639 |
| TFDP1 | transcription factor Dp-1 | **0,791** | 0 | 28,304 | 15,388 |
| TREX2 | three prime repair exonuclease 2 | **0,791** | 0,23765 | 0,388 | 0,638 |
| EFNA4 | ephrin A4 | **0,791** | 0,035948 | 1,994 | 1,085 |
| KCNE5 | potassium voltage-gated channel subfamily E regulatory subunit 5 | **0,790** | 0,048356 | 1,329 | 1,405 |
| ZFP69 | ZFP69 zinc finger protein | **0,790** | 0,084843 | 1,329 | 0,766 |
| LRRC6 | leucine rich repeat containing 6 | **0,790** | 0,019692 | 0,997 | 0,702 |

| | | | | | |
|---|---|---|---|---|---|
| TMEM151A | transmembrane protein 151A | **0,790** | 0,043546 | 1,163 | 1,469 |
| PCDHGA6 | protocadherin gamma subfamily A, 6 | **0,790** | 0,000847 | 3,379 | 2,362 |
| CPPED1 | calcineurin like phosphoesterase domain containing 1 | **0,790** | 0 | 24,815 | 14,302 |
| LINC00115 | long intergenic non-protein coding RNA 115 | **0,790** | 0,367098 | 0,388 | 0,192 |
| CYTOR | cytoskeleton regulator RNA | **0,790** | 0 | 22,876 | 15,579 |
| AHR | aryl hydrocarbon receptor | **0,790** | 0 | 28,858 | 13,408 |
| HAAO | 3-hydroxyanthranilate 3,4-dioxygenase | **0,789** | 0,0542 | 1,108 | 0,894 |
| FAM163A | family with sequence similarity 163 member A | **0,789** | 0,290849 | 0,111 | 0,447 |
| C8orf37 | chromosome 8 open reading frame 37 | **0,789** | 0,002735 | 2,437 | 1,724 |
| TFR2 | transferrin receptor 2 | **0,789** | 0,001198 | 2,770 | 2,873 |
| CXCL6 | C-X-C motif chemokine ligand 6 | **0,788** | 0 | 368,566 | 217,600 |
| SMIM3 | small integral membrane protein 3 | **0,788** | 0 | 19,276 | 12,068 |
| DM1-AS | DM1 locus antisense RNA | **0,788** | 0,077188 | 0,775 | 0,702 |
| LNX1 | ligand of numb-protein X 1 | **0,788** | 0,002745 | 3,988 | 5,044 |
| FHL2 | four and a half LIM domains 2 | **0,788** | 0 | 68,518 | 49,420 |
| ICAM3 | intercellular adhesion molecule 3 | **0,787** | 0,000453 | 4,210 | 2,043 |
| LIMD1-AS1 | LIMD1 antisense RNA 1 | **0,787** | 0,197782 | 0,499 | 0,702 |
| LINC01600 | long intergenic non-protein coding RNA 1600 | **0,787** | 0,253556 | 0,388 | 0,128 |
| TPBGL | trophoblast glycoprotein like | **0,787** | 0,21925 | 0,332 | 0,255 |
| RUSC2 | RUN and SH3 domain containing 2 | **0,787** | 0 | 55,612 | 35,628 |
| ST6GALNAC4 | ST6 N-acetylgalactosaminide alpha-2,6-sialyltransferase 4 | **0,787** | 0 | 32,292 | 25,221 |
| SUPT3H | SPT3 homolog, SAGA and STAGA complex component | **0,787** | 0,023442 | 3,323 | 2,043 |
| ATP5D | ATP synthase, H+ transporting, mitochondrial F1 complex, delta subunit | **0,786** | 0 | 22,987 | 17,623 |
| VLDLR-AS1 | VLDLR antisense RNA 1 | **0,786** | 0,017376 | 1,662 | 1,213 |
| LURAP1L | leucine rich adaptor protein 1 like | **0,786** | 0 | 19,719 | 9,577 |
| LGR4 | leucine rich repeat containing G protein-coupled receptor 4 | **0,785** | 0 | 54,227 | 38,246 |
| YIF1A | Yip1 interacting factor homolog A, membrane trafficking protein | **0,785** | 0 | 61,871 | 47,121 |
| GSC | goosecoid homeobox | **0,785** | 0,244501 | 1,108 | 0,319 |
| TGFA | transforming growth factor alpha | **0,785** | 0 | 26,089 | 23,497 |
| AVPI1 | arginine vasopressin induced 1 | **0,785** | 0 | 35,173 | 28,285 |
| ELMO1 | engulfment and cell motility 1 | **0,784** | 2,00E-06 | 6,647 | 5,938 |
| CA13 | carbonic anhydrase 13 | **0,784** | 0,000965 | 2,991 | 2,299 |
| SLC16A4 | solute carrier family 16 member 4 | **0,784** | 0,000152 | 5,705 | 2,937 |

| | | | | | |
|---|---|---|---|---|---|
| EML1 | echinoderm microtubule associated protein like 1 | **0,784** | 0 | 38,330 | 22,411 |
| LINC01909 | long intergenic non-protein coding RNA 1909 | **0,784** | 0,020692 | 1,163 | 0,830 |
| AXL | AXL receptor tyrosine kinase | **0,784** | 0 | 262,660 | 135,170 |
| ARMC9 | armadillo repeat containing 9 | **0,783** | 0 | 37,111 | 17,942 |
| SYNGR1 | synaptogyrin 1 | **0,783** | 0,012621 | 2,770 | 0,958 |
| ANXA11 | annexin A11 | **0,783** | 0 | 148,113 | 88,751 |
| TEX41 | testis expressed 41 (non-protein coding) | **0,783** | 2,00E-06 | 4,265 | 4,597 |
| LAPTM5 | lysosomal protein transmembrane 5 | **0,783** | 1,30E-05 | 9,029 | 3,959 |
| CASP1 | caspase 1 | **0,783** | 0,08839 | 1,163 | 0,958 |
| SFXN4 | sideroflexin 4 | **0,783** | 8,00E-06 | 7,422 | 5,172 |
| CYB561D2 | cytochrome b561 family member D2 | **0,783** | 1,00E-06 | 9,638 | 7,087 |
| NTHL1 | nth like DNA glycosylase 1 | **0,782** | 0,001819 | 3,711 | 1,724 |
| KLF7 | Kruppel like factor 7 | **0,782** | 0 | 8,032 | 6,066 |
| SLC12A4 | solute carrier family 12 member 4 | **0,782** | 0 | 70,567 | 40,545 |
| NSUN7 | NOP2/Sun RNA methyltransferase family member 7 | **0,782** | 0,141908 | 0,554 | 0,255 |
| PRRX1 | paired related homeobox 1 | **0,781** | 0 | 162,404 | 101,330 |
| RCN3 | reticulocalbin 3 | **0,781** | 0 | 286,589 | 100,627 |
| STEAP1B | STEAP family member 1B | **0,781** | 0,060321 | 1,163 | 0,447 |
| E2F5 | E2F transcription factor 5 | **0,781** | 0,006483 | 2,216 | 1,788 |
| BMP5 | bone morphogenetic protein 5 | **0,781** | 0,000236 | 6,370 | 3,256 |
| FAM171A1 | family with sequence similarity 171 member A1 | **0,781** | 0,000115 | 6,758 | 1,469 |
| PRPH2 | peripherin 2 | **0,781** | 0,120276 | 0,554 | 0,894 |
| ETNK2 | ethanolamine kinase 2 | **0,780** | 0,005718 | 2,825 | 1,979 |
| ZNF341 | zinc finger protein 341 | **0,780** | 0,004063 | 1,440 | 1,724 |
| ZNF593 | zinc finger protein 593 | **0,780** | 0 | 10,801 | 10,280 |
| IFITM2 | interferon induced transmembrane protein 2 | **0,780** | 0 | 12,297 | 8,811 |
| DCTPP1 | dCTP pyrophosphatase 1 | **0,780** | 1,00E-06 | 9,582 | 6,832 |
| NDP | NDP, norrin cystine knot growth factor | **0,780** | 0 | 47,082 | 43,482 |
| TNFRSF9 | TNF receptor superfamily member 9 | **0,779** | 0 | 24,261 | 13,217 |
| CDC42EP1 | CDC42 effector protein 1 | **0,779** | 1,20E-05 | 4,819 | 4,789 |
| IL1RL1 | interleukin 1 receptor like 1 | **0,779** | 0 | 17,337 | 19,027 |
| RMDN2 | regulator of microtubule dynamics 2 | **0,779** | 0,003589 | 2,437 | 1,405 |
| B4GAT1 | beta-1,4-glucuronyltransferase 1 | **0,779** | 0,000564 | 8,032 | 5,044 |

| | | | | | |
|---|---|---|---|---|---|
| PICK1 | protein interacting with PRKCA 1 | **0,779** | 1,00E-06 | 6,813 | 5,363 |
| FANCC | Fanconi anemia complementation group C | **0,778** | 8,90E-05 | 4,542 | 3,065 |
| NOG | noggin | **0,778** | 0,000369 | 3,323 | 2,490 |
| C17orf100 | chromosome 17 open reading frame 100 | **0,777** | 0,002796 | 2,160 | 1,788 |
| ALDH18A1 | aldehyde dehydrogenase 18 family member A1 | **0,777** | 0 | 61,815 | 44,631 |
| C2orf73 | chromosome 2 open reading frame 73 | **0,777** | 0,264682 | 0,222 | 0,255 |
| PDHB | pyruvate dehydrogenase (lipoamide) beta | **0,777** | 0 | 22,322 | 11,621 |
| GPX8 | glutathione peroxidase 8 (putative) | **0,777** | 0 | 91,172 | 43,035 |
| KAT2A | lysine acetyltransferase 2A | **0,776** | 3,00E-06 | 6,868 | 5,746 |
| PCDHB11 | protocadherin beta 11 | **0,776** | 0,077723 | 1,662 | 1,022 |
| PTGER1 | prostaglandin E receptor 1 | **0,776** | 0,117576 | 0,222 | 0,319 |
| ALG3 | ALG3, alpha-1,3- mannosyltransferase | **0,776** | 0 | 25,147 | 23,816 |
| SRGAP1 | SLIT-ROBO Rho GTPase activating protein 1 | **0,775** | 0 | 13,183 | 14,749 |
| PRELID2 | PRELI domain containing 2 | **0,775** | 0,00053 | 2,991 | 2,362 |
| CCDC65 | coiled-coil domain containing 65 | **0,775** | 0,066127 | 0,720 | 0,766 |
| EPB41L3 | erythrocyte membrane protein band 4.1 like 3 | **0,775** | 1,00E-06 | 26,753 | 32,244 |
| ESCO2 | establishment of sister chromatid cohesion N-acetyltransferase 2 | **0,775** | 0,047324 | 3,268 | 0,511 |
| ALPK2 | alpha kinase 2 | **0,775** | 0,016725 | 0,997 | 1,085 |
| CLPP | caseinolytic mitochondrial matrix peptidase proteolytic subunit | **0,774** | 0 | 22,655 | 16,218 |
| KLK10 | kallikrein related peptidase 10 | **0,774** | 0,000892 | 4,210 | 2,554 |
| PRMT1 | protein arginine methyltransferase 1 | **0,774** | 0 | 53,230 | 36,841 |
| CREB3L1 | cAMP responsive element binding protein 3 like 1 | **0,774** | 0,000568 | 7,422 | 2,554 |
| ST3GAL5 | ST3 beta-galactoside alpha-2,3-sialyltransferase 5 | **0,774** | 0,053423 | 0,609 | 0,575 |
| PDF | peptide deformylase (mitochondrial) | **0,773** | 0,001022 | 2,105 | 1,724 |
| LINC02104 | long intergenic non-protein coding RNA 2104 | **0,773** | 0,036136 | 0,997 | 0,958 |
| NUF2 | NUF2, NDC80 kinetochore complex component | **0,773** | 0,050852 | 2,714 | 0,128 |
| USP43 | ubiquitin specific peptidase 43 | **0,773** | 0,106965 | 1,440 | 0,447 |
| PBX1 | PBX homeobox 1 | **0,772** | 0 | 10,580 | 5,874 |
| RARA-AS1 | RARA antisense RNA 1 | **0,772** | 0,009727 | 1,662 | 0,958 |
| PHGDH | phosphoglycerate dehydrogenase | **0,772** | 0 | 136,648 | 86,133 |
| AK7 | adenylate kinase 7 | **0,772** | 0,044432 | 0,554 | 0,575 |
| DAW1 | dynein assembly factor with WD repeats 1 | **0,772** | 0,050685 | 0,332 | 0,383 |
| RNF152 | ring finger protein 152 | **0,772** | 2,00E-05 | 12,906 | 8,811 |

| | | | | | |
|---|---|---|---|---|---|
| INO80C | INO80 complex subunit C | **0,772** | 0 | 9,250 | 7,279 |
| CXCL2 | C-X-C motif chemokine ligand 2 | **0,771** | 0 | 193,201 | 235,223 |
| CCDC74A | coiled-coil domain containing 74A | **0,771** | 0,024112 | 1,606 | 0,702 |
| NME1 | NME/NM23 nucleoside diphosphate kinase 1 | **0,771** | 0 | 9,693 | 7,917 |
| LNCSRLR | lncRNA sorafenib resistance in renal cell carcinoma associated | **0,771** | 0,054747 | 0,665 | 0,383 |
| CD14 | CD14 molecule | **0,771** | 0 | 11,410 | 18,836 |
| ALOX12 | arachidonate 12-lipoxygenase, 12S type | **0,771** | 0,018053 | 0,942 | 1,022 |
| IQCD | IQ motif containing D | **0,770** | 0,025006 | 1,329 | 0,958 |
| ACSS3 | acyl-CoA synthetase short-chain family member 3 | **0,770** | 0,005514 | 1,828 | 1,469 |
| FLJ31104 | uncharacterized LOC441072 | **0,769** | 0,006491 | 1,163 | 1,852 |
| F3 | coagulation factor III, tissue factor | **0,769** | 0,00022 | 3,656 | 2,937 |
| PMEPA1 | prostate transmembrane protein, androgen induced 1 | **0,768** | 1,00E-06 | 82,033 | 59,061 |
| ARID3A | AT-rich interaction domain 3A | **0,768** | 7,90E-05 | 5,539 | 3,129 |
| TOMM40 | translocase of outer mitochondrial membrane 40 | **0,768** | 0 | 23,652 | 23,624 |
| NCCRP1 | non-specific cytotoxic cell receptor protein 1 homolog (zebrafish) | **0,768** | 0,000793 | 6,813 | 0,511 |
| FOXCUT | FOXC1 upstream transcript (non-protein coding) | **0,767** | 0,196426 | 0,609 | 0,638 |
| KCTD16 | potassium channel tetramerization domain containing 16 | **0,767** | 0,026188 | 1,219 | 0,958 |
| FAM19A3 | family with sequence similarity 19 member A3, C-C motif chemokine like | **0,767** | 0,022799 | 0,942 | 0,702 |
| FAM71E1 | family with sequence similarity 71 member E1 | **0,766** | 0,131509 | 0,388 | 0,192 |
| TNFSF11 | TNF superfamily member 11 | **0,766** | 0,004762 | 1,717 | 1,022 |
| FUZ | fuzzy planar cell polarity protein | **0,766** | 0 | 9,527 | 7,726 |
| SEC14L5 | SEC14 like lipid binding 5 | **0,766** | 0,091583 | 0,277 | 0,447 |
| ASTN2 | astrotactin 2 | **0,766** | 0,036082 | 0,554 | 0,575 |
| 9.maalis | membrane associated ring-CH-type finger 9 | **0,766** | 7,00E-06 | 6,979 | 4,597 |
| LCAT | lecithin-cholesterol acyltransferase | **0,765** | 0,00011 | 3,600 | 2,362 |
| POLR2J3 | RNA polymerase II subunit J3 | **0,765** | 0,152913 | 0,443 | 0,192 |
| ABCF2 | ATP binding cassette subfamily F member 2 | **0,765** | 0 | 13,903 | 9,194 |
| GPAT2 | glycerol-3-phosphate acyltransferase 2, mitochondrial | **0,765** | 0,034044 | 4,210 | 1,852 |
| PPAN | peter pan homolog (Drosophila) | **0,765** | 0,001056 | 1,772 | 2,043 |
| KCNIP2 | potassium voltage-gated channel interacting protein 2 | **0,765** | 0,164523 | 0,720 | 0,383 |
| MDFI | MyoD family inhibitor | **0,764** | 0 | 12,186 | 6,321 |
| HOXC10 | homeobox C10 | **0,764** | 0 | 20,605 | 12,642 |
| SCARA5 | scavenger receptor class A member 5 | **0,764** | 0,010033 | 1,606 | 7,023 |

| | | | | | |
|---|---|---|---|---|---|
| ALMS1-IT1 | ALMS1 intronic transcript 1 | **0,764** | 0,088944 | 0,665 | 0,192 |
| CCDC151 | coiled-coil domain containing 151 | **0,764** | 0,25976 | 0,222 | 0,255 |
| ATP1B1 | ATPase Na+/K+ transporting subunit beta 1 | **0,764** | 0 | 319,823 | 210,641 |
| DLX4 | distal-less homeobox 4 | **0,763** | 0,008474 | 0,775 | 1,022 |
| THBD | thrombomodulin | **0,763** | 0 | 72,727 | 94,306 |
| HUNK | hormonally up-regulated Neu-associated kinase | **0,763** | 0,126852 | 0,942 | 0,383 |
| SPDYA | speedy/RINGO cell cycle regulator family member A | **0,763** | 0,253821 | 0,443 | 0,128 |
| SIX5 | SIX homeobox 5 | **0,763** | 0 | 28,304 | 18,006 |
| EHD3 | EH domain containing 3 | **0,763** | 0 | 10,413 | 4,980 |
| DHRS3 | dehydrogenase/reductase 3 | **0,762** | 0 | 24,704 | 26,689 |
| GPR161 | G protein-coupled receptor 161 | **0,762** | 1,70E-05 | 6,204 | 4,853 |
| SYNJ2 | synaptojanin 2 | **0,762** | 0 | 153,431 | 109,311 |
| FABP4 | fatty acid binding protein 4 | **0,762** | 0,029339 | 1,219 | 0,894 |
| RBMS3 | RNA binding motif single stranded interacting protein 3 | **0,761** | 0 | 12,297 | 7,662 |
| TMPO-AS1 | TMPO antisense RNA 1 | **0,761** | 0,119533 | 1,385 | 0,192 |
| TEKT4P2 | tektin 4 pseudogene 2 | **0,761** | 0,024866 | 0,554 | 0,702 |
| CA9 | carbonic anhydrase 9 | **0,761** | 0,000665 | 2,714 | 0,702 |
| MIRLET7BHG | MIRLET7B host gene | **0,760** | 0,000362 | 4,431 | 3,639 |
| NPM3 | nucleophosmin/nucleoplasmin 3 | **0,760** | 6,00E-05 | 3,767 | 2,618 |
| FAM20C | FAM20C, golgi associated secretory pathway kinase | **0,760** | 0 | 161,241 | 68,639 |
| NAPA-AS1 | NAPA antisense RNA 1 | **0,759** | 0,00049 | 1,772 | 1,724 |
| CCNJL | cyclin J like | **0,759** | 0,001003 | 2,160 | 0,958 |
| OR7E14P | olfactory receptor family 7 subfamily E member 14 pseudogene | **0,759** | 0,016327 | 0,609 | 0,830 |
| SERPIND1 | serpin family D member 1 | **0,759** | 0 | 4,154 | 7,981 |
| ACOX2 | acyl-CoA oxidase 2 | **0,758** | 0,089809 | 0,443 | 0,383 |
| TENM1 | teneurin transmembrane protein 1 | **0,758** | 0,001381 | 3,323 | 3,129 |
| TEX21P | testis expressed 21, pseudogene | **0,758** | 0,102108 | 0,332 | 0,511 |
| PET117 | PET117 homolog | **0,758** | 0,142567 | 0,388 | 0,192 |
| CCDC81 | coiled-coil domain containing 81 | **0,758** | 0,223006 | 0,000 | 0,383 |
| YIPF2 | Yip1 domain family member 2 | **0,758** | 0 | 38,552 | 23,624 |
| TGFBR2 | transforming growth factor beta receptor 2 | **0,758** | 0 | 168,331 | 158,922 |
| L3HYPDH | trans-L-3-hydroxyproline dehydratase | **0,757** | 3,00E-06 | 6,758 | 3,639 |
| TOP2A | topoisomerase (DNA) II alpha | **0,757** | 1,50E-05 | 23,098 | 1,022 |

| | | | | | |
|---|---|---|---|---|---|
| ADAMTS1 | ADAM metallopeptidase with thrombospondin type 1 motif 1 | **0,757** | 0 | 71,786 | 34,351 |
| CCNA2 | cyclin A2 | **0,756** | 0,005031 | 4,930 | 0,766 |
| FST | follistatin | **0,756** | 0 | 260,666 | 116,143 |
| WWC2-AS2 | WWC2 antisense RNA 2 | **0,756** | 0,010083 | 1,329 | 1,149 |
| TLCD1 | TLC domain containing 1 | **0,756** | 2,00E-05 | 7,201 | 4,278 |
| PEAR1 | platelet endothelial aggregation receptor 1 | **0,755** | 0,108854 | 0,222 | 0,447 |
| PITPNC1 | phosphatidylinositol transfer protein, cytoplasmic 1 | **0,755** | 0 | 22,765 | 25,732 |
| SHROOM2 | shroom family member 2 | **0,754** | 0,185578 | 0,443 | 0,064 |
| LDLRAD2 | low density lipoprotein receptor class A domain containing 2 | **0,754** | 0,081659 | 1,052 | 0,702 |
| LETM2 | leucine zipper and EF-hand containing transmembrane protein 2 | **0,754** | 0 | 10,524 | 9,258 |
| BYSL | bystin like | **0,754** | 0 | 17,226 | 16,154 |
| OR7E38P | olfactory receptor family 7 subfamily E member 38 pseudogene | **0,754** | 0,069618 | 0,609 | 0,575 |
| DKK1 | dickkopf WNT signaling pathway inhibitor 1 | **0,754** | 0,09944 | 0,332 | 0,128 |
| CAMK2B | calcium/calmodulin dependent protein kinase II beta | **0,753** | 0,184929 | 0,277 | 0,064 |
| AREG | amphiregulin | **0,753** | 0,002782 | 1,606 | 1,596 |
| ADRA1B | adrenoceptor alpha 1B | **0,753** | 0,125813 | 0,332 | 0,383 |
| NTN4 | netrin 4 | **0,753** | 0 | 304,535 | 157,582 |
| FZD10-AS1 | FZD10 antisense RNA 1 (head to head) | **0,752** | 0,003401 | 0,886 | 0,830 |
| MLKL | mixed lineage kinase domain like pseudokinase | **0,752** | 0 | 13,404 | 10,344 |
| RPS6KA2 | ribosomal protein S6 kinase A2 | **0,752** | 0 | 86,575 | 48,462 |
| CFP | complement factor properdin | **0,752** | 0,212303 | 0,554 | 0,319 |
| SLC9A3R2 | SLC9A3 regulator 2 | **0,751** | 0 | 97,542 | 31,031 |
| CLCF1 | cardiotrophin like cytokine factor 1 | **0,751** | 0 | 56,664 | 51,335 |
| INSIG1 | insulin induced gene 1 | **0,751** | 0 | 237,624 | 109,694 |
| MYD88 | myeloid differentiation primary response 88 | **0,751** | 0 | 13,958 | 7,662 |
| PPP1R14BP3 | protein phosphatase 1 regulatory inhibitor subunit 14B pseudogene 3 | **0,750** | 0,003921 | 1,606 | 0,575 |
| FAM109B | family with sequence similarity 109 member B | **0,750** | 0,006168 | 1,939 | 1,085 |
| SNHG25 | small nucleolar RNA host gene 25 | **0,750** | 0,089985 | 0,222 | 0,511 |
| MIER2 | MIER family member 2 | **0,750** | 0 | 13,017 | 8,173 |
| SMAD6 | SMAD family member 6 | **0,749** | 0 | 27,197 | 15,452 |
| MYBL1 | MYB proto-oncogene like 1 | **0,749** | 0 | 19,331 | 10,216 |
| GAS6-AS2 | GAS6 antisense RNA 2 (head to head) | **0,749** | 0,00945 | 1,939 | 0,894 |
| FGF1 | fibroblast growth factor 1 | **0,749** | 0,000496 | 50,571 | 33,904 |

| | | | | | |
|---|---|---|---|---|---|
| SREBF2 | sterol regulatory element binding transcription factor 2 | **0,749** | 0 | 150,218 | 82,111 |
| SLC2A10 | solute carrier family 2 member 10 | **0,749** | 0 | 29,246 | 8,556 |
| NR2F1-AS1 | NR2F1 antisense RNA 1 | **0,748** | 0,026193 | 0,942 | 0,447 |
| CTF1 | cardiotrophin 1 | **0,748** | 0,002983 | 1,883 | 2,043 |
| SHISA9 | shisa family member 9 | **0,748** | 0,002418 | 1,274 | 1,341 |
| ASH1L-AS1 | ASH1L antisense RNA 1 | **0,747** | 0,022735 | 0,499 | 0,447 |
| KNDC1 | kinase non-catalytic C-lobe domain containing 1 | **0,746** | 0,024609 | 0,609 | 0,255 |
| IGFBP1 | insulin like growth factor binding protein 1 | **0,746** | 0 | 36,059 | 45,078 |
| HMGCR | 3-hydroxy-3-methylglutaryl-CoA reductase | **0,746** | 0 | 89,511 | 58,295 |
| TET1 | tet methylcytosine dioxygenase 1 | **0,745** | 0,226444 | 0,443 | 0,192 |
| SMOC1 | SPARC related modular calcium binding 1 | **0,745** | 0 | 280,440 | 118,888 |
| FAR2 | fatty acyl-CoA reductase 2 | **0,744** | 0,003274 | 3,434 | 2,107 |
| MVK | mevalonate kinase | **0,744** | 0 | 7,865 | 5,619 |
| CNN2 | calponin 2 | **0,744** | 0 | 7,478 | 5,555 |
| WFDC10B | WAP four-disulfide core domain 10B | **0,744** | 0,090487 | 0,222 | 0,192 |
| MYOF | myoferlin | **0,744** | 0 | 170,657 | 90,795 |
| NFIA | nuclear factor I A | **0,744** | 5,00E-06 | 7,588 | 5,236 |
| SCARB1 | scavenger receptor class B member 1 | **0,744** | 0 | 25,424 | 18,644 |
| PXDN | peroxidasin | **0,744** | 0 | 123,465 | 46,738 |
| ZNF512B | zinc finger protein 512B | **0,744** | 0 | 7,201 | 3,192 |
| FGF7 | fibroblast growth factor 7 | **0,744** | 0,166988 | 0,055 | 0,383 |
| HJURP | Holliday junction recognition protein | **0,743** | 0,005942 | 5,594 | 0,255 |
| PRDM1 | PR/SET domain 1 | **0,743** | 0,000227 | 1,662 | 1,341 |
| EPB41L4B | erythrocyte membrane protein band 4.1 like 4B | **0,743** | 0,000156 | 5,207 | 2,490 |
| MAGEH1 | MAGE family member H1 | **0,742** | 0 | 19,940 | 11,812 |
| COL7A1 | collagen type VII alpha 1 chain | **0,742** | 3,00E-06 | 62,203 | 22,539 |
| ISOC2 | isochorismatase domain containing 2 | **0,742** | 0 | 9,638 | 6,960 |
| SLC38A6 | solute carrier family 38 member 6 | **0,742** | 0 | 7,755 | 6,449 |
| STK32B | serine/threonine kinase 32B | **0,741** | 0,001227 | 1,606 | 1,213 |
| PAFAH1B3 | platelet activating factor acetylhydrolase 1b catalytic subunit 3 | **0,741** | 0,007189 | 1,163 | 1,469 |
| HYAL1 | hyaluronoglucosaminidase 1 | **0,741** | 0 | 6,204 | 9,322 |
| FOXO3B | forkhead box O3B pseudogene | **0,741** | 0,013225 | 1,052 | 0,766 |
| MAMLD1 | mastermind like domain containing 1 | **0,741** | 1,20E-05 | 6,702 | 6,832 |

| | | | | | |
|---|---|---|---|---|---|
| ANKEF1 | ankyrin repeat and EF-hand domain containing 1 | **0,740** | 0,006309 | 2,548 | 0,511 |
| ZBED6CL | ZBED6 C-terminal like | **0,739** | 0,021232 | 0,886 | 0,383 |
| MAGEL2 | MAGE family member L2 | **0,739** | 0,008667 | 0,665 | 0,575 |
| B3GALNT1 | beta-1,3-N-acetylgalactosaminyltransferase 1 (globoside blood group) | **0,739** | 1,00E-06 | 6,038 | 5,108 |
| PIEZO2 | piezo type mechanosensitive ion channel component 2 | **0,738** | 0 | 6,536 | 5,108 |
| LIN7B | lin-7 homolog B, crumbs cell polarity complex component | **0,738** | 0,008267 | 1,772 | 1,085 |
| TPM4 | tropomyosin 4 | **0,738** | 0 | 102,306 | 40,162 |
| NINJ2 | ninjurin 2 | **0,738** | 0,035533 | 0,499 | 0,511 |
| RBPMS-AS1 | RBPMS antisense RNA 1 | **0,738** | 0,008551 | 0,166 | 0,830 |
| TMEM178B | transmembrane protein 178B | **0,737** | 0,007219 | 1,939 | 1,149 |
| RIN1 | Ras and Rab interactor 1 | **0,736** | 0 | 25,036 | 19,730 |
| SLC25A15 | solute carrier family 25 member 15 | **0,736** | 9,00E-05 | 2,437 | 1,532 |
| COL3A1 | collagen type III alpha 1 chain | **0,736** | 0 | 1005,276 | 427,858 |
| RAPGEF3 | Rap guanine nucleotide exchange factor 3 | **0,736** | 1,00E-06 | 3,379 | 2,554 |
| NCEH1 | neutral cholesterol ester hydrolase 1 | **0,736** | 0 | 17,282 | 9,705 |
| SPSB4 | splA/ryanodine receptor domain and SOCS box containing 4 | **0,736** | 0 | 11,964 | 11,301 |
| HEG1 | heart development protein with EGF like domains 1 | **0,735** | 0 | 69,515 | 49,101 |
| MVB12B | multivesicular body subunit 12B | **0,735** | 0,001849 | 1,883 | 1,788 |
| TEX14 | testis expressed 14, intercellular bridge forming factor | **0,735** | 0,005288 | 0,443 | 1,469 |
| RBPMS | RNA binding protein with multiple splicing | **0,734** | 0 | 3,988 | 8,747 |
| FJX1 | four jointed box 1 | **0,734** | 0 | 21,990 | 15,707 |
| EHF | ETS homologous factor | **0,734** | 0,033695 | 0,831 | 0,255 |
| EIF4EBP1 | eukaryotic translation initiation factor 4E binding protein 1 | **0,734** | 0 | 35,117 | 19,985 |
| SRM | spermidine synthase | **0,733** | 0 | 44,534 | 33,074 |
| C19orf57 | chromosome 19 open reading frame 57 | **0,733** | 0,012379 | 1,329 | 0,702 |
| EPGN | epithelial mitogen | **0,733** | 0,095309 | 0,831 | 0,575 |
| NTNG1 | netrin G1 | **0,733** | 0,019888 | 3,268 | 0,447 |
| SEMA3A | semaphorin 3A | **0,733** | 0 | 108,509 | 96,158 |
| HOXA11-AS | HOXA11 antisense RNA | **0,732** | 0,002063 | 1,662 | 1,213 |
| SPTY2D1-AS1 | SPTY2D1 antisense RNA 1 | **0,732** | 0,067846 | 0,665 | 1,022 |
| CMBL | carboxymethylenebutenolidase homolog | **0,732** | 0,000896 | 4,542 | 2,299 |
| HSD17B7P2 | hydroxysteroid 17-beta dehydrogenase 7 pseudogene 2 | **0,732** | 0,051787 | 0,665 | 0,192 |
| CFAP58-AS1 | CFAP58 antisense RNA 1 (head to head) | **0,731** | 4,00E-06 | 3,933 | 3,512 |

| | | | | | |
|---|---|---|---|---|---|
| STARD4 | StAR related lipid transfer domain containing 4 | **0,731** | 0 | 15,897 | 5,236 |
| FDXACB1 | ferredoxin-fold anticodon binding domain containing 1 | **0,730** | 0,022742 | 0,997 | 0,766 |
| LRRN3 | leucine rich repeat neuronal 3 | **0,730** | 0 | 6,924 | 11,493 |
| ARHGAP6 | Rho GTPase activating protein 6 | **0,729** | 0,114346 | 0,443 | 0,575 |
| NAT14 | N-acetyltransferase 14 (putative) | **0,729** | 0,000255 | 2,105 | 1,724 |
| ZFP69B | ZFP69 zinc finger protein B | **0,728** | 0,000169 | 2,825 | 2,171 |
| AOX1 | aldehyde oxidase 1 | **0,728** | 0 | 7,533 | 4,023 |
| SLC22A3 | solute carrier family 22 member 3 | **0,727** | 0,071959 | 0,222 | 0,128 |
| DDX52 | DExD-box helicase 52 | **0,727** | 0,016356 | 1,329 | 0,319 |
| C17orf107 | chromosome 17 open reading frame 107 | **0,727** | 0 | 8,198 | 6,513 |
| LINC00342 | long intergenic non-protein coding RNA 342 | **0,726** | 0,005806 | 2,770 | 1,149 |
| FAAHP1 | fatty acid amide hydrolase pseudogene 1 | **0,726** | 0,093608 | 0,222 | 0,447 |
| TMEM121 | transmembrane protein 121 | **0,725** | 0,00576 | 1,606 | 0,702 |
| FASN | fatty acid synthase | **0,725** | 0 | 119,920 | 51,335 |
| SIX4 | SIX homeobox 4 | **0,725** | 0 | 19,054 | 14,813 |
| CCNB2 | cyclin B2 | **0,725** | 0,003543 | 4,099 | 0,255 |
| SIX1 | SIX homeobox 1 | **0,725** | 0 | 53,341 | 43,226 |
| LINC01119 | long intergenic non-protein coding RNA 1119 | **0,724** | 0,122942 | 0,388 | 0,128 |
| ACTG1 | actin gamma 1 | **0,723** | 0 | 530,915 | 246,780 |
| KIF23 | kinesin family member 23 | **0,723** | 4,20E-05 | 6,370 | 0,958 |
| LINC00886 | long intergenic non-protein coding RNA 886 | **0,722** | 0,006334 | 1,496 | 0,575 |
| AL365205.1 | Prickle-like protein 4 | **0,722** | 0,037995 | 0,609 | 0,383 |
| LRRC45 | leucine rich repeat containing 45 | **0,722** | 0,002347 | 2,714 | 1,724 |
| C8orf88 | chromosome 8 open reading frame 88 | **0,721** | 0 | 13,903 | 7,023 |
| ERCC2 | ERCC excision repair 2, TFIIH core complex helicase subunit | **0,721** | 1,00E-06 | 8,364 | 4,342 |
| RRM2 | ribonucleotide reductase regulatory subunit M2 | **0,719** | 3,00E-06 | 11,632 | 1,213 |
| SH3RF2 | SH3 domain containing ring finger 2 | **0,719** | 0 | 10,081 | 4,533 |
| NSDHL | NAD(P) dependent steroid dehydrogenase-like | **0,718** | 0 | 18,611 | 11,046 |
| MRPL12 | mitochondrial ribosomal protein L12 | **0,718** | 0 | 4,819 | 3,320 |
| FAM43B | family with sequence similarity 43 member B | **0,718** | 0 | 6,038 | 6,002 |
| PID1 | phosphotyrosine interaction domain containing 1 | **0,718** | 0 | 352,780 | 165,052 |
| PSTPIP1 | proline-serine-threonine phosphatase interacting protein 1 | **0,718** | 6,00E-06 | 1,440 | 2,299 |
| KDELR3 | KDEL endoplasmic reticulum protein retention receptor 3 | **0,718** | 0 | 23,707 | 9,961 |

| | | | | | |
|---|---|---|---|---|---|
| UBE2C | ubiquitin conjugating enzyme E2 C | **0,717** | 0,013638 | 4,597 | 0,128 |
| RFLNA | refilin A | **0,717** | 0 | 3,711 | 4,023 |
| RPH3AL | rabphilin 3A like (without C2 domains) | **0,717** | 0,002616 | 1,496 | 0,447 |
| AMOTL2 | angiomotin like 2 | **0,717** | 0 | 154,428 | 118,825 |
| SCN8A | sodium voltage-gated channel alpha subunit 8 | **0,717** | 0,000879 | 2,049 | 1,085 |
| DLX6-AS1 | DLX6 antisense RNA 1 | **0,716** | 0,020928 | 1,939 | 0,702 |
| C2orf70 | chromosome 2 open reading frame 70 | **0,716** | 0,073695 | 0,277 | 0,319 |
| RFXAP | regulatory factor X associated protein | **0,716** | 0,000375 | 1,883 | 1,852 |
| RBMS2 | RNA binding motif single stranded interacting protein 2 | **0,716** | 0 | 41,321 | 25,987 |
| SLC1A5 | solute carrier family 1 member 5 | **0,716** | 0 | 247,428 | 190,400 |
| ZNF511 | zinc finger protein 511 | **0,715** | 2,00E-06 | 4,431 | 2,426 |
| TCP11 | t-complex 11 | **0,715** | 0,043166 | 0,609 | 0,511 |
| NR2F1 | nuclear receptor subfamily 2 group F member 1 | **0,714** | 0,002138 | 2,049 | 0,319 |
| KLRG1 | killer cell lectin like receptor G1 | **0,714** | 0,034544 | 0,332 | 0,511 |
| IL12RB2 | interleukin 12 receptor subunit beta 2 | **0,714** | 0,043599 | 0,665 | 1,469 |
| P2RY6 | pyrimidinergic receptor P2Y6 | **0,713** | 0,000227 | 1,440 | 1,085 |
| IL12A | interleukin 12A | **0,712** | 0,014981 | 0,554 | 0,383 |
| PRSS23 | protease, serine 23 | **0,712** | 0 | 265,263 | 123,103 |
| TEX26 | testis expressed 26 | **0,712** | 0,102113 | 0,222 | 0,064 |
| ARHGDIB | Rho GDP dissociation inhibitor beta | **0,712** | 0 | 7,478 | 1,915 |
| PANK1 | pantothenate kinase 1 | **0,711** | 0,022 | 1,108 | 0,830 |
| PDCD1LG2 | programmed cell death 1 ligand 2 | **0,711** | 0 | 12,186 | 8,684 |
| KCNK6 | potassium two pore domain channel subfamily K member 6 | **0,711** | 0 | 15,731 | 7,726 |
| MAN1B1-AS1 | MAN1B1 antisense RNA 1 (head to head) | **0,710** | 5,70E-05 | 2,659 | 1,532 |
| RPGRIP1L | RPGRIP1 like | **0,710** | 0 | 7,976 | 5,108 |
| DISC1 | disrupted in schizophrenia 1 | **0,709** | 7,00E-06 | 3,268 | 2,618 |
| CLDN1 | claudin 1 | **0,708** | 3,00E-05 | 5,594 | 3,448 |
| RSPO3 | R-spondin 3 | **0,708** | 0 | 148,944 | 49,228 |
| TRAK1 | trafficking kinesin protein 1 | **0,707** | 0 | 9,582 | 6,002 |
| ARHGEF19 | Rho guanine nucleotide exchange factor 19 | **0,707** | 0 | 7,201 | 5,619 |
| WISP1 | WNT1 inducible signaling pathway protein 1 | **0,707** | 0 | 8,696 | 16,537 |
| SLC16A1-AS1 | SLC16A1 antisense RNA 1 | **0,705** | 0,09479 | 0,166 | 0,000 |
| IDI1 | isopentenyl-diphosphate delta isomerase 1 | **0,705** | 0 | 72,949 | 38,374 |

| | | | | | |
|---|---|---|---|---|---|
| RAET1E | retinoic acid early transcript 1E | **0,705** | 0,042316 | 1,108 | 0,319 |
| TNFSF10 | TNF superfamily member 10 | **0,704** | 2,00E-06 | 1,606 | 2,426 |
| STC2 | stanniocalcin 2 | **0,703** | 0 | 184,228 | 101,968 |
| MRPS24 | mitochondrial ribosomal protein S24 | **0,702** | 0 | 8,198 | 4,406 |
| CATSPER1 | cation channel sperm associated 1 | **0,702** | 0,013322 | 0,775 | 0,702 |
| AURKA | aurora kinase A | **0,701** | 0,000345 | 4,210 | 0,575 |
| WNT5B | Wnt family member 5B | **0,701** | 0 | 27,197 | 6,002 |
| EME1 | essential meiotic structure-specific endonuclease 1 | **0,701** | 0,033211 | 1,108 | 0,766 |
| EVA1A | eva-1 homolog A, regulator of programmed cell death | **0,701** | 0 | 6,148 | 2,107 |
| MN1 | MN1 proto-oncogene, transcriptional regulator | **0,700** | 0 | 25,535 | 17,942 |
| HCST | hematopoietic cell signal transducer | **0,700** | 0,031075 | 0,665 | 0,255 |
| ZNF69 | zinc finger protein 69 | **0,700** | 0,003196 | 1,440 | 0,447 |
| PPP1R14B | protein phosphatase 1 regulatory inhibitor subunit 14B | **0,700** | 0 | 36,447 | 16,729 |
| GALNT3 | polypeptide N-acetylgalactosaminyltransferase 3 | **0,699** | 8,00E-06 | 3,213 | 1,149 |
| NEURL1 | neuralized E3 ubiquitin protein ligase 1 | **0,698** | 0,017755 | 0,831 | 0,766 |
| SPOCD1 | SPOC domain containing 1 | **0,698** | 0 | 4,210 | 5,619 |
| LINC01715 | long intergenic non-protein coding RNA 1715 | **0,697** | 0,010662 | 0,499 | 0,702 |
| ARHGAP18 | Rho GTPase activating protein 18 | **0,697** | 1,40E-05 | 2,326 | 1,660 |
| TBX2 | T-box 2 | **0,697** | 0,001465 | 2,603 | 0,319 |
| P3H4 | prolyl 3-hydroxylase family member 4 (non-enzymatic) | **0,697** | 0 | 61,095 | 27,392 |
| C9orf3 | chromosome 9 open reading frame 3 | **0,696** | 0 | 96,047 | 54,336 |
| H3F3AP4 | H3 histone, family 3A, pseudogene 4 | **0,695** | 0,029109 | 0,277 | 0,319 |
| PDE10A | phosphodiesterase 10A | **0,694** | 2,10E-05 | 8,585 | 3,448 |
| SPIN2B | spindlin family member 2B | **0,693** | 0,049557 | 0,942 | 0,511 |
| MTCP1 | mature T-cell proliferation 1 | **0,693** | 0,121418 | 0,443 | 0,447 |
| ID1 | inhibitor of DNA binding 1, HLH protein | **0,693** | 0 | 42,983 | 17,431 |
| C14orf1 | chromosome 14 open reading frame 1 | **0,692** | 0 | 23,541 | 12,004 |
| SLC25A5-AS1 | SLC25A5 antisense RNA 1 | **0,691** | 0,001836 | 0,775 | 1,405 |
| RGS19 | regulator of G protein signaling 19 | **0,691** | 0,000322 | 3,877 | 1,022 |
| AHRR | aryl-hydrocarbon receptor repressor | **0,690** | 0 | 5,761 | 2,171 |
| LINC00852 | long intergenic non-protein coding RNA 852 | **0,690** | 0,064943 | 0,277 | 0,064 |
| LINC00857 | long intergenic non-protein coding RNA 857 | **0,689** | 1,00E-06 | 3,157 | 2,362 |
| TMEM71 | transmembrane protein 71 | **0,689** | 8,10E-05 | 1,108 | 1,085 |

| | | | | | |
|---|---|---|---|---|---|
| MYOSLID | myocardin-induced smooth muscle lncRNA, inducer of differentiation | **0,689** | 0,005803 | 0,775 | 0,702 |
| NYNRIN | NYN domain and retroviral integrase containing | **0,688** | 2,00E-06 | 4,154 | 2,426 |
| SCD | stearoyl-CoA desaturase | **0,687** | 0 | 204,888 | 86,900 |
| GPR68 | G protein-coupled receptor 68 | **0,687** | 0 | 38,773 | 20,560 |
| DPY19L2P1 | DPY19L2 pseudogene 1 | **0,687** | 0,038699 | 0,831 | 0,575 |
| GPR39 | G protein-coupled receptor 39 | **0,686** | 0 | 3,379 | 2,171 |
| LINC00471 | long intergenic non-protein coding RNA 471 | **0,686** | 0,014958 | 0,609 | 0,319 |
| RIN3 | Ras and Rab interactor 3 | **0,685** | 0 | 16,285 | 9,897 |
| TBXAS1 | thromboxane A synthase 1 | **0,685** | 0,073607 | 0,775 | 0,638 |
| CLUHP3 | clustered mitochondria homolog pseudogene 3 | **0,684** | 0 | 2,936 | 2,043 |
| TTK | TTK protein kinase | **0,683** | 0,014789 | 2,326 | 0,128 |
| MKI67 | marker of proliferation Ki-67 | **0,682** | 0,000265 | 16,285 | 0,766 |
| PNPLA3 | patatin like phospholipase domain containing 3 | **0,682** | 0,084862 | 0,942 | 0,192 |
| SLC38A10 | solute carrier family 38 member 10 | **0,682** | 0 | 193,976 | 108,992 |
| CARD9 | caspase recruitment domain family member 9 | **0,680** | 7,20E-05 | 2,216 | 0,702 |
| PRR5L | proline rich 5 like | **0,679** | 0,000434 | 2,437 | 0,830 |
| NFE2L3 | nuclear factor, erythroid 2 like 3 | **0,679** | 0,025009 | 0,609 | 0,319 |
| PARPBP | PARP1 binding protein | **0,679** | 0,004605 | 0,997 | 0,575 |
| STXBP2 | syntaxin binding protein 2 | **0,679** | 0,058407 | 0,332 | 0,128 |
| KRT7 | keratin 7 | **0,679** | 0,002788 | 0,554 | 1,085 |
| STAG3L3 | stromal antigen 3-like 3 (pseudogene) | **0,678** | 0,036172 | 0,499 | 0,192 |
| SPATA24 | spermatogenesis associated 24 | **0,677** | 0,000137 | 1,385 | 0,766 |
| PROSER2 | proline and serine rich 2 | **0,673** | 0 | 28,803 | 14,239 |
| LINC02015 | long intergenic non-protein coding RNA 2015 | **0,673** | 0 | 8,364 | 2,043 |
| MAGED1 | MAGE family member D1 | **0,672** | 0 | 70,456 | 22,858 |
| CYP26B1 | cytochrome P450 family 26 subfamily B member 1 | **0,672** | 0 | 32,569 | 19,347 |
| CAP2 | CAP, adenylate cyclase-associated protein, 2 (yeast) | **0,670** | 3,60E-05 | 2,271 | 0,575 |
| PDCD4-AS1 | PDCD4 antisense RNA 1 | **0,670** | 0,009097 | 0,499 | 0,255 |
| PALMD | palmdelphin | **0,670** | 0 | 44,478 | 26,945 |
| SULT1C4 | sulfotransferase family 1C member 4 | **0,668** | 0,000236 | 1,828 | 1,022 |
| HNRNPA1L2 | heterogeneous nuclear ribonucleoprotein A1-like 2 | **0,667** | 4,00E-06 | 1,883 | 1,469 |
| INSC | inscuteable homolog (Drosophila) | **0,667** | 0,009026 | 0,665 | 0,575 |
| FGF14 | fibroblast growth factor 14 | **0,667** | 0,040724 | 0,277 | 0,447 |

| | | | | | |
|---|---|---|---|---|---|
| RHOXF1-AS1 | RHOXF1 antisense RNA 1 | **0,667** | 0,000407 | 1,828 | 0,638 |
| MYLK4 | myosin light chain kinase family member 4 | **0,666** | 0,045616 | 0,388 | 0,064 |
| GNG11 | G protein subunit gamma 11 | **0,666** | 0 | 24,372 | 17,431 |
| MYLK3 | myosin light chain kinase 3 | **0,666** | 0,009866 | 0,499 | 0,383 |
| BACH1-IT2 | BACH1 intronic transcript 2 | **0,666** | 0,044975 | 0,443 | 0,319 |
| SAMD12 | sterile alpha motif domain containing 12 | **0,666** | 0,014656 | 0,942 | 0,192 |
| EFEMP1 | EGF containing fibulin like extracellular matrix protein 1 | **0,666** | 0 | 76,217 | 40,162 |
| CSF3 | colony stimulating factor 3 | **0,665** | 0 | 18,279 | 11,748 |
| HOXA4 | homeobox A4 | **0,661** | 0,00087 | 1,440 | 0,575 |
| TGFB2 | transforming growth factor beta 2 | **0,661** | 0 | 59,434 | 37,927 |
| DTX4 | deltex E3 ubiquitin ligase 4 | **0,661** | 0,021378 | 0,609 | 0,255 |
| DOK1 | docking protein 1 | **0,659** | 0 | 49,463 | 14,558 |
| KRT18P59 | keratin 18 pseudogene 59 | **0,658** | 0,009256 | 1,329 | 0,702 |
| PLA2G4A | phospholipase A2 group IVA | **0,658** | 0 | 55,999 | 18,516 |
| SV2A | synaptic vesicle glycoprotein 2A | **0,657** | 0 | 7,755 | 3,192 |
| UBE2QL1 | ubiquitin conjugating enzyme E2 Q family like 1 | **0,657** | 0 | 3,046 | 3,256 |
| ADAMTS5 | ADAM metallopeptidase with thrombospondin type 1 motif 5 | **0,655** | 0 | 39,992 | 10,280 |
| KDR | kinase insert domain receptor | **0,655** | 0,001767 | 1,108 | 0,255 |
| DBNDD2 | dysbindin domain containing 2 | **0,655** | 0,008426 | 0,775 | 0,319 |
| GPR89A | G protein-coupled receptor 89A | **0,655** | 2,40E-05 | 1,662 | 0,830 |
| IL7R | interleukin 7 receptor | **0,651** | 0,001097 | 1,496 | 0,575 |
| DHCR24 | 24-dehydrocholesterol reductase | **0,649** | 0 | 65,139 | 39,778 |
| LINC00565 | long intergenic non-protein coding RNA 565 | **0,647** | 7,40E-05 | 1,994 | 0,575 |
| MVD | mevalonate diphosphate decarboxylase | **0,646** | 0 | 62,203 | 32,244 |
| COQ2 | coenzyme Q2, polyprenyltransferase | **0,646** | 0 | 6,038 | 4,469 |
| CABLES1 | Cdk5 and Abl enzyme substrate 1 | **0,643** | 0 | 6,924 | 6,193 |
| PLAU | plasminogen activator, urokinase | **0,643** | 0 | 9,306 | 5,236 |
| AMPD2 | adenosine monophosphate deaminase 2 | **0,642** | 0 | 36,391 | 16,856 |
| PTGIS | prostaglandin I2 synthase | **0,641** | 3,00E-06 | 0,499 | 1,277 |
| HMGCS1 | 3-hydroxy-3-methylglutaryl-CoA synthase 1 | **0,640** | 0 | 77,103 | 41,247 |
| TM7SF2 | transmembrane 7 superfamily member 2 | **0,640** | 0 | 2,770 | 1,149 |
| SLC19A1 | solute carrier family 19 member 1 | **0,640** | 1,00E-06 | 2,049 | 1,022 |
| SQLE | squalene epoxidase | **0,639** | 0 | 92,834 | 32,883 |

| | | | | | |
|---|---|---|---|---|---|
| LOXL3 | lysyl oxidase like 3 | **0,638** | 0 | 114,048 | 22,092 |
| BCL11A | B-cell CLL/lymphoma 11A | **0,636** | 0,00029 | 1,274 | 0,511 |
| FAM218A | family with sequence similarity 218 member A | **0,635** | 0,006411 | 0,665 | 0,255 |
| TSKU | tsukushi, small leucine rich proteoglycan | **0,633** | 0 | 20,273 | 8,875 |
| MELK | maternal embryonic leucine zipper kinase | **0,633** | 6,10E-05 | 4,542 | 0,319 |
| PDE3A | phosphodiesterase 3A | **0,631** | 0,000663 | 1,939 | 0,575 |
| FLVCR1-AS1 | FLVCR1 antisense RNA 1 (head to head) | **0,630** | 0,001294 | 1,385 | 0,638 |
| ATOH8 | atonal bHLH transcription factor 8 | **0,628** | 0 | 12,795 | 4,406 |
| HOXC11 | homeobox C11 | **0,628** | 8,10E-05 | 1,440 | 0,575 |
| TMEM26 | transmembrane protein 26 | **0,627** | 0,072843 | 0,443 | 0,447 |
| MAMSTR | MEF2 activating motif and SAP domain containing transcriptional regulator | **0,626** | 0,018123 | 0,775 | 0,064 |
| LINC01114 | long intergenic non-protein coding RNA 1114 | **0,625** | 0,007336 | 0,332 | 0,255 |
| NEURL1B | neuralized E3 ubiquitin protein ligase 1B | **0,625** | 0,011176 | 1,717 | 0,383 |
| SLC6A9 | solute carrier family 6 member 9 | **0,623** | 0 | 23,929 | 11,557 |
| MMAB | methylmalonic aciduria (cobalamin deficiency) cblB type | **0,623** | 0 | 12,407 | 8,045 |
| MRGPRF | MAS related GPR family member F | **0,623** | 0 | 4,985 | 2,746 |
| NANOS3 | nanos C2HC-type zinc finger 3 | **0,621** | 0,030996 | 0,222 | 0,383 |
| GNA14 | G protein subunit alpha 14 | **0,620** | 0 | 1,329 | 1,213 |
| TICRR | TOPBP1 interacting checkpoint and replication regulator | **0,620** | 0,009333 | 0,499 | 0,319 |
| C6orf223 | chromosome 6 open reading frame 223 | **0,616** | 0,013175 | 0,720 | 0,319 |
| LINC01915 | long intergenic non-protein coding RNA 1915 | **0,615** | 0,010581 | 0,554 | 0,064 |
| GDF6 | growth differentiation factor 6 | **0,614** | 0 | 135,429 | 47,121 |
| CPM | carboxypeptidase M | **0,611** | 0 | 16,008 | 6,193 |
| PYCR1 | pyrroline-5-carboxylate reductase 1 | **0,611** | 0 | 44,312 | 20,560 |
| NKILA | NF-kappaB interacting lncRNA | **0,609** | 0 | 1,440 | 1,022 |
| ST6GALNAC6 | ST6 N-acetylgalactosaminide alpha-2,6-sialyltransferase 6 | **0,608** | 0,002634 | 0,388 | 0,192 |
| LINC00519 | long intergenic non-protein coding RNA 519 | **0,606** | 0,036218 | 0,499 | 0,447 |
| FADS1 | fatty acid desaturase 1 | **0,605** | 0 | 28,692 | 9,322 |
| TGFBI | transforming growth factor beta induced | **0,604** | 0 | 190,819 | 62,892 |
| CYP51A1 | cytochrome P450 family 51 subfamily A member 1 | **0,599** | 0 | 6,204 | 2,873 |
| SLC8A1 | solute carrier family 8 member A1 | **0,597** | 0 | 10,469 | 4,661 |
| CYR61 | cysteine rich angiogenic inducer 61 | **0,595** | 0 | 398,920 | 158,092 |
| CYSRT1 | cysteine rich tail 1 | **0,593** | 0,000344 | 0,831 | 0,575 |

| | | | | | |
|---|---|---|---|---|---|
| EBP | emopamil binding protein (sterol isomerase) | **0,593** | 0 | 8,032 | 2,873 |
| EIF4A1P10 | eukaryotic translation initiation factor 4A1 pseudogene 10 | **0,592** | 0,010489 | 0,222 | 0,319 |
| DSCAM | DS cell adhesion molecule | **0,591** | 0,004658 | 0,720 | 0,830 |
| CCL2 | C-C motif chemokine ligand 2 | **0,589** | 0 | 184,726 | 172,267 |
| ESM1 | endothelial cell specific molecule 1 | **0,582** | 0 | 883,861 | 850,225 |
| CYP1B1-AS1 | CYP1B1 antisense RNA 1 | **0,582** | 0,000122 | 1,496 | 0,447 |
| FDPS | farnesyl diphosphate synthase | **0,581** | 0 | 72,395 | 29,179 |
| RAMP1 | receptor activity modifying protein 1 | **0,579** | 0 | 7,201 | 1,469 |
| NEGR1 | neuronal growth regulator 1 | **0,579** | 0 | 42,540 | 12,834 |
| AJUBA | ajuba LIM protein | **0,578** | 0 | 31,351 | 8,364 |
| LINC00707 | long intergenic non-protein coding RNA 707 | **0,577** | 0 | 7,090 | 2,618 |
| SLC38A5 | solute carrier family 38 member 5 | **0,576** | 0 | 4,043 | 1,085 |
| LINC02407 | long intergenic non-protein coding RNA 2407 | **0,573** | 0 | 0,775 | 0,702 |
| ST6GALNAC5 | ST6 N-acetylgalactosaminide alpha-2,6-sialyltransferase 5 | **0,572** | 0 | 3,213 | 4,023 |
| FLRT1 | fibronectin leucine rich transmembrane protein 1 | **0,572** | 0,000137 | 0,388 | 0,511 |
| ESRP2 | epithelial splicing regulatory protein 2 | **0,568** | 0,006671 | 0,222 | 0,255 |
| CPNE5 | copine 5 | **0,559** | 0,001532 | 0,332 | 0,447 |
| GPR3 | G protein-coupled receptor 3 | **0,557** | 0,00853 | 0,332 | 0,255 |
| ADAMTS6 | ADAM metallopeptidase with thrombospondin type 1 motif 6 | **0,554** | 0 | 16,617 | 6,832 |
| NDC80 | NDC80, kinetochore complex component | **0,542** | 1,50E-05 | 3,102 | 0,128 |
| HAS1 | hyaluronan synthase 1 | **0,541** | 0,000154 | 0,499 | 0,511 |
| EBLN1 | endogenous Bornavirus-like nucleoprotein 1 | **0,540** | 0,004247 | 0,332 | 0,383 |
| IL6 | interleukin 6 | **0,539** | 0 | 1280,011 | 557,856 |
| PACSIN3 | protein kinase C and casein kinase substrate in neurons 3 | **0,535** | 0 | 7,921 | 1,660 |
| IER3 | immediate early response 3 | **0,535** | 0 | 1,219 | 0,894 |
| CDA | cytidine deaminase | **0,532** | 0 | 3,268 | 1,213 |
| SLC14A1 | solute carrier family 14 member 1 (Kidd blood group) | **0,527** | 0 | 2,825 | 0,575 |
| WFDC21P | WAP four-disulfide core domain 21, pseudogene | **0,525** | 0,000967 | 0,499 | 0,128 |
| HAL | histidine ammonia-lyase | **0,523** | 0 | 1,163 | 1,085 |
| HDAC10 | histone deacetylase 10 | **0,522** | 0,000391 | 0,609 | 0,128 |
| GGT5 | gamma-glutamyltransferase 5 | **0,487** | 0,000114 | 1,551 | 0,383 |
| CIDEA | cell death-inducing DFFA-like effector a | **0,474** | 1,00E-06 | 1,828 | 0,511 |
| RIPK4 | receptor interacting serine/threonine kinase 4 | **0,465** | 0 | 8,087 | 2,554 |

| | | | | | |
|---|---|---|---|---|---|
| MSMO1 | methylsterol monooxygenase 1 | **0,463** | 0 | 122,135 | 37,033 |
| TMEM97 | transmembrane protein 97 | **0,460** | 0 | 13,737 | 4,789 |
| HRCT1 | histidine rich carboxyl terminus 1 | **0,459** | 4,40E-05 | 1,108 | 0,255 |
| LDB2 | LIM domain binding 2 | **0,445** | 1,90E-05 | 0,277 | 0,192 |
| SHISA3 | shisa family member 3 | **0,441** | 0 | 2,714 | 0,958 |
| GDF5 | growth differentiation factor 5 | **0,419** | 0 | 9,582 | 1,724 |
| DHCR7 | 7-dehydrocholesterol reductase | **0,407** | 0 | 48,466 | 11,429 |
| LDLR | low density lipoprotein receptor | **0,400** | 0 | 90,231 | 22,475 |
| CYP1A1 | cytochrome P450 family 1 subfamily A member 1 | **0,379** | 0,000354 | 0,609 | 0,319 |
| ALDH3A1 | aldehyde dehydrogenase 3 family member A1 | **0,375** | 0 | 4,874 | 1,915 |
| FADS2 | fatty acid desaturase 2 | **0,364** | 0 | 31,240 | 4,980 |
| IGFBP4 | insulin like growth factor binding protein 4 | **0,341** | 0 | 147,892 | 38,501 |
| CYP1B1 | cytochrome P450 family 1 subfamily B member 1 | **0,337** | 0 | 1191,775 | 134,915 |
