# Supplementary material for: Regulation of gene expression by MF63, a selective inhibitor of microsomal PGE synthase 1 (mPGES1) in human osteoarthritic chondrocytes
Source: Br J Pharmacol. 2020 Aug 10;177(18):4134–46. doi: 10.1111/bph.15142 (PMC7443472; doi:10.1111/bph.15142)
Supplement: Supplementary file 2 — Table S2. Supporting information [file BPH-177-4134-s002.pdf]

Gene	Description	FC	adj. P	Mean (ktrl)	Mean (MF)
G0S2	G0/G1 switch 2	**1,526**	4,05E-10	24,732	41,545
MT1L	metallothionein 1L, pseudogene	**1,485**	1,04E-04	24,175	64,906
PXDNL	peroxidasin like	**1,464**	1,35E-04	1,883	3,657
WNT7B	Wnt family member 7B	**1,376**	4,95E-04	4,084	6,367
PPARGC1A	PPARG coactivator 1 alpha	**1,376**	1,27E-03	2,809	4,640
SLC30A2	solute carrier family 30 member 2	**1,366**	1,71E-03	2,326	5,250
ARRB1	arrestin beta 1	**1,347**	1,81E-04	16,349	22,636
CMYA5	cardiomyopathy associated 5	**1,347**	6,57E-04	4,274	6,327
SORL1	sortilin related receptor 1	**1,338**	2,25E-05	22,300	31,262
CCRL2	C-C motif chemokine receptor like 2	**1,338**	5,08E-04	3,587	5,209
FAM189A2	family with sequence similarity 189 member A2	**1,338**	2,82E-03	2,061	3,225
CCDC18-AS1	CCDC18 antisense RNA 1	**1,320**	2,07E-04	7,470	10,337
PGPEP1	pyroglutamyl-peptidase I	**1,320**	6,25E-04	14,865	20,577
MT1M	metallothionein 1M	**1,320**	4,18E-03	97,019	218,563
FAM78B	family with sequence similarity 78 member B	**1,320**	4,47E-03	2,641	3,995
FBXO32	F-box protein 32	**1,320**	7,43E-03	41,910	71,747
CBLB	Cbl proto-oncogene B	**1,310**	9,82E-05	28,388	38,278
EGR2	early growth response 2	**1,310**	1,54E-03	2,937	4,150
MT1F	metallothionein 1F	**1,310**	4,77E-03	111,620	252,098
RN7SL2	RNA, 7SL, cytoplasmic 2	**1,301**	1,11E-03	10,414	14,130
MT1H	metallothionein 1H	**1,301**	4,57E-03	96,128	240,268
NEAT1	nuclear paraspeckle assembly transcript 1 (non-protein coding)	**1,301**	5,25E-03	660,239	957,291
GEM	GTP binding protein overexpressed in skeletal muscle	**1,301**	5,37E-03	56,332	82,051
FAM19A2	family with sequence similarity 19 member A2, C-C motif chemok	**1,301**	7,54E-03	2,110	3,109
SCUBE1	signal peptide, CUB domain and EGF like domain containing 1	**1,292**	3,54E-05	337,041	428,122
BCL2	BCL2, apoptosis regulator	**1,292**	4,82E-05	63,173	84,111
AHNAK2	AHNAK nucleoprotein 2	**1,292**	5,94E-05	308,108	400,165
SYT11	synaptotagmin 11	**1,292**	9,06E-05	13,473	18,163
RORC	RAR related orphan receptor C	**1,292**	3,26E-04	13,359	17,929
FAXDC2	fatty acid hydroxylase domain containing 2	**1,292**	3,79E-04	19,779	26,131
FAT3	FAT atypical cadherin 3	**1,292**	3,85E-04	6,825	9,325

CHDH	choline dehydrogenase	**1,292**	3,99E-04	19,412	25,867
KIFC2	kinesin family member C2	**1,292**	1,20E-03	6,248	8,496
GALM	galactose mutarotase	**1,292**	1,71E-03	12,779	17,869
SLC30A1	solute carrier family 30 member 1	**1,292**	4,75E-03	150,732	210,331
GALNT15	polypeptide N-acetylgalactosaminyltransferase 15	**1,292**	4,91E-03	234,168	327,316
CDKN2B	cyclin dependent kinase inhibitor 2B	**1,292**	9,86E-03	71,405	108,596
FAM160A1	family with sequence similarity 160 member A1	**1,283**	1,03E-03	8,492	11,571
ZMIZ1-AS1	ZMIZ1 antisense RNA 1	**1,283**	2,47E-03	5,934	8,063
BACH2	BTB domain and CNC homolog 2	**1,283**	9,92E-03	2,169	3,113
NFKBIZ	NFKB inhibitor zeta	**1,275**	3,63E-05	31,136	41,174
COL14A1	collagen type XIV alpha 1 chain	**1,275**	7,16E-05	52,570	70,434
GBP2	guanylate binding protein 2	**1,275**	2,86E-04	20,729	27,332
ANKRD6	ankyrin repeat domain 6	**1,275**	2,94E-04	9,866	13,059
NR4A2	nuclear receptor subfamily 4 group A member 2	**1,275**	2,96E-04	10,681	14,033
DLX3	distal-less homeobox 3	**1,275**	6,08E-04	23,972	32,313
UAP1L1	UDP-N-acetylglucosamine pyrophosphorylase 1 like 1	**1,275**	8,02E-04	16,426	21,681
RASSF8-AS1	RASSF8 antisense RNA 1	**1,275**	8,64E-04	9,194	12,164
C1QTNF6	C1q and TNF related 6	**1,275**	1,06E-03	5,736	7,691
ELF3	E74 like ETS transcription factor 3	**1,275**	4,71E-03	10,334	13,496
SCARA5	scavenger receptor class A member 5	**1,275**	7,13E-03	3,600	5,053
KDM7A	lysine demethylase 7A	**1,275**	8,02E-03	46,366	64,969
IFIT3	interferon induced protein with tetratricopeptide repeats 3	**1,275**	8,86E-03	24,135	33,280
MT1E	metallothionein 1E	**1,275**	1,24E-02	399,287	813,026
IFIT2	interferon induced protein with tetratricopeptide repeats 2	**1,275**	1,31E-02	1,995	2,926
RAPGEFL1	Rap guanine nucleotide exchange factor like 1	**1,275**	1,54E-02	2,612	3,753
SLITRK4	SLIT and NTRK like family member 4	**1,275**	1,68E-02	8,275	12,968
YPEL2	yippee like 2	**1,266**	2,56E-05	30,962	39,890
ZNF385C	zinc finger protein 385C	**1,266**	6,74E-05	22,343	29,081
BMF	Bcl2 modifying factor	**1,266**	2,28E-04	35,782	45,575
IL6R	interleukin 6 receptor	**1,266**	2,30E-04	31,471	40,610
KLF15	Kruppel like factor 15	**1,266**	1,61E-03	17,437	22,918
ACCS	1-aminocyclopropane-1-carboxylate synthase homolog (inactive)	**1,266**	1,71E-03	5,603	7,397
METTL7A	methyltransferase like 7A	**1,266**	3,01E-03	162,036	209,143

IFIT1	interferon induced protein with tetratricopeptide repeats 1	**1,266**	3,05E-03	6,655	9,203
GRIN2C	glutamate ionotropic receptor NMDA type subunit 2C	**1,266**	6,71E-03	12,632	16,462
RASGEF1B	RasGEF domain family member 1B	**1,266**	9,03E-03	2,891	4,013
CD1D	CD1d molecule	**1,266**	1,25E-02	5,288	6,796
RPARP-AS1	RPARP antisense RNA 1	**1,266**	1,40E-02	1,637	2,318
MT2A	metallothionein 2A	**1,266**	1,60E-02	2019,404	3827,661
SNED1	sushi, nidogen and EGF like domains 1	**1,257**	2,33E-04	138,145	176,989
SCUBE3	signal peptide, CUB domain and EGF like domain containing 3	**1,257**	2,65E-04	34,128	46,137
A2M	alpha-2-macroglobulin	**1,257**	2,85E-04	1221,108	1397,438
TRIB1	tribbles pseudokinase 1	**1,257**	1,27E-03	32,286	42,151
KLHL24	kelch like family member 24	**1,257**	1,36E-03	25,121	32,513
PSD4	pleckstrin and Sec7 domain containing 4	**1,257**	2,05E-03	14,845	18,890
DAPK2	death associated protein kinase 2	**1,257**	2,16E-03	14,732	19,418
FAM49A	family with sequence similarity 49 member A	**1,257**	2,81E-03	21,737	28,937
LMO2	LIM domain only 2	**1,257**	4,86E-03	5,032	6,936
ZNF667	zinc finger protein 667	**1,257**	7,05E-03	3,696	4,990
LINC01089	long intergenic non-protein coding RNA 1089	**1,257**	7,33E-03	3,018	4,053
SQSTM1	sequestosome 1	**1,257**	7,70E-03	76,628	100,278
SH3BP5	SH3 domain binding protein 5	**1,257**	8,02E-03	79,795	106,405
NEDD4L	neural precursor cell expressed, developmentally down-regulated	**1,257**	1,02E-02	7,925	10,933
MT1X	metallothionein 1X	**1,257**	1,45E-02	223,105	476,544
SALL2	spalt like transcription factor 2	**1,257**	2,42E-02	1,791	2,590
GDF15	growth differentiation factor 15	**1,257**	2,70E-02	20,230	29,875
THRA	thyroid hormone receptor, alpha	**1,248**	2,50E-05	53,428	67,780
ARID1B	AT-rich interaction domain 1B	**1,248**	1,94E-04	100,948	127,027
ITGA6	integrin subunit alpha 6	**1,248**	4,59E-04	65,436	83,421
LYST	lysosomal trafficking regulator	**1,248**	4,63E-04	15,176	19,470
C3	complement C3	**1,248**	1,84E-03	9,542	12,705
TRANK1	tetratricopeptide repeat and ankyrin repeat containing 1	**1,248**	2,15E-03	12,853	16,649
RGS2	regulator of G protein signaling 2	**1,248**	8,54E-03	181,066	239,049
PPM1L	protein phosphatase, Mg2+/Mn2+ dependent 1L	**1,248**	9,24E-03	6,910	9,327
AMDHD2	amidohydrolase domain containing 2	**1,248**	9,29E-03	10,583	13,907
SPP1	secreted phosphoprotein 1	**1,248**	1,12E-02	3343,297	4516,805

IVNS1ABP	influenza virus NS1A binding protein	**1,248**	1,25E-02	812,054	1101,255
LINC01139	long intergenic non-protein coding RNA 1139	**1,248**	1,31E-02	4,315	5,639
SOD2	superoxide dismutase 2	**1,248**	2,82E-02	930,541	1301,915
CTSF	cathepsin F	**1,240**	1,14E-04	64,160	80,445
FRY	FRY microtubule binding protein	**1,240**	1,23E-04	147,007	184,589
PTPRM	protein tyrosine phosphatase, receptor type M	**1,240**	1,96E-04	172,043	216,290
BCL6	B-cell CLL/lymphoma 6	**1,240**	4,31E-04	127,279	160,066
OBSCN	obscurin, cytoskeletal calmodulin and titin-interacting RhoGEF	**1,240**	1,06E-03	17,447	21,764
ZBTB7C	zinc finger and BTB domain containing 7C	**1,240**	1,76E-03	13,506	17,256
DNAH11	dynein axonemal heavy chain 11	**1,240**	5,91E-03	12,096	15,419
WDFY3-AS2	WDFY3 antisense RNA 2	**1,240**	6,38E-03	11,935	15,294
IRS2	insulin receptor substrate 2	**1,240**	1,10E-02	77,586	101,321
PMP22	peripheral myelin protein 22	**1,240**	1,53E-02	290,157	383,000
DDIT4L	DNA damage inducible transcript 4 like	**1,240**	2,52E-02	50,549	67,009
C11orf96	chromosome 11 open reading frame 96	**1,240**	2,77E-02	36,687	50,870
MT-ND5	mitochondrially encoded NADH:ubiquinone oxidoreductase core s	**1,231**	2,07E-05	825,802	1028,895
FLNB	filamin B	**1,231**	4,49E-05	126,401	155,959
JADE2	jade family PHD finger 2	**1,231**	1,79E-04	43,881	54,487
C14orf132	chromosome 14 open reading frame 132	**1,231**	7,42E-04	55,591	69,425
RNF19B	ring finger protein 19B	**1,231**	7,53E-04	45,996	57,398
SSTR5	somatostatin receptor 5	**1,231**	8,12E-04	20,171	25,572
ST6GAL1	ST6 beta-galactoside alpha-2,6-sialyltransferase 1	**1,231**	1,09E-03	83,032	102,417
CREBRF	CREB3 regulatory factor	**1,231**	1,39E-03	50,242	63,067
FOXN3	forkhead box N3	**1,231**	1,71E-03	46,326	58,276
ENPP2	ectonucleotide pyrophosphatase/phosphodiesterase 2	**1,231**	1,76E-03	63,209	76,390
FZD4	frizzled class receptor 4	**1,231**	2,51E-03	14,028	17,961
RRAGD	Ras related GTP binding D	**1,231**	3,87E-03	24,097	30,389
PARD3B	par-3 family cell polarity regulator beta	**1,231**	4,61E-03	6,433	8,137
SDAD1P1	SDA1 domain containing 1 pseudogene 1	**1,231**	8,27E-03	5,597	7,163
VIT	vitrin	**1,231**	9,36E-03	6,138	7,991
RANBP3L	RAN binding protein 3 like	**1,231**	9,72E-03	13,198	15,743
TLR5	toll like receptor 5	**1,231**	9,83E-03	5,844	7,509
PNPLA7	patatin like phospholipase domain containing 7	**1,231**	1,05E-02	6,773	8,945

PCDHGA4	protocadherin gamma subfamily A, 4	**1,231**	1,94E-02	4,158	5,343
DLGAP1-AS1	DLGAP1 antisense RNA 1	**1,231**	2,07E-02	4,149	5,491
DACT1	dishevelled binding antagonist of beta catenin 1	**1,231**	2,33E-02	23,463	31,906
LRRC2	leucine rich repeat containing 2	**1,231**	2,44E-02	4,094	5,404
NGFR	nerve growth factor receptor	**1,231**	3,53E-02	2,182	2,936
SYT17	synaptotagmin 17	**1,231**	3,54E-02	2,531	3,497
THSD7A	thrombospondin type 1 domain containing 7A	**1,231**	3,55E-02	1,889	2,650
KLHDC9	kelch domain containing 9	**1,231**	3,73E-02	1,894	2,601
SRGAP3	SLIT-ROBO Rho GTPase activating protein 3	**1,231**	3,78E-02	1,791	2,463
CD79A	CD79a molecule	**1,231**	4,18E-02	1,526	2,180
IGF2R	insulin like growth factor 2 receptor	**1,223**	8,95E-05	320,772	396,244
TNFRSF11B	TNF receptor superfamily member 11b	**1,223**	1,15E-04	1525,094	1896,253
ABHD14B	abhydrolase domain containing 14B	**1,223**	1,81E-04	21,261	26,368
NBL1	neuroblastoma 1, DAN family BMP antagonist	**1,223**	2,23E-04	88,539	109,732
DENND3	DENN domain containing 3	**1,223**	7,84E-04	29,816	36,906
DISP1	dispatched RND transporter family member 1	**1,223**	8,64E-04	13,884	17,192
FOXO1	forkhead box O1	**1,223**	8,64E-04	57,073	70,991
SLC27A1	solute carrier family 27 member 1	**1,223**	9,50E-04	23,213	28,956
AKAP6	A-kinase anchoring protein 6	**1,223**	1,15E-03	10,118	12,668
CBX7	chromobox 7	**1,223**	1,17E-03	87,810	108,781
TACSTD2	tumor associated calcium signal transducer 2	**1,223**	1,57E-03	10,519	13,020
MXI1	MAX interactor 1, dimerization protein	**1,223**	2,18E-03	36,289	44,678
ATP9A	ATPase phospholipid transporting 9A (putative)	**1,223**	2,27E-03	52,909	65,122
GPNMB	glycoprotein nmb	**1,223**	4,73E-03	31,445	40,562
BMP2	bone morphogenetic protein 2	**1,223**	8,29E-03	451,775	557,252
BPI	bactericidal/permeability-increasing protein	**1,223**	1,30E-02	7,584	9,819
PLEKHB1	pleckstrin homology domain containing B1	**1,223**	1,41E-02	8,013	10,247
GSDMB	gasdermin B	**1,223**	1,52E-02	3,837	5,011
N4BP2L1	NEDD4 binding protein 2 like 1	**1,223**	2,17E-02	4,542	5,924
TTN	titin	**1,223**	2,83E-02	4,829	6,478
SYT13	synaptotagmin 13	**1,223**	2,83E-02	3,900	4,958
RETREG1	reticulophagy regulator 1	**1,223**	2,85E-02	118,691	153,659
CYFIP2	cytoplasmic FMR1 interacting protein 2	**1,223**	2,93E-02	2,943	3,912

PPIL6	peptidylprolyl isomerase like 6	**1,223**	3,27E-02	2,409	3,168
FAM84A	family with sequence similarity 84 member A	**1,223**	3,85E-02	4,139	5,569
AZIN1-AS1	AZIN1 antisense RNA 1	**1,223**	4,01E-02	1,942	2,615
VPS37D	VPS37D, ESCRT-I subunit	**1,223**	4,58E-02	1,939	2,642
NUMA1	nuclear mitotic apparatus protein 1	**1,214**	4,60E-05	217,098	264,434
ANGPTL2	angiopoietin like 2	**1,214**	8,66E-05	940,112	1138,671
MTUS1	microtubule associated scaffold protein 1	**1,214**	1,96E-04	144,628	176,844
CDH13	cadherin 13	**1,214**	1,96E-04	21,460	26,537
ZCCHC14	zinc finger CCHC-type containing 14	**1,214**	2,02E-04	82,109	101,061
DUSP6	dual specificity phosphatase 6	**1,214**	4,65E-04	51,109	63,638
CCDC149	coiled-coil domain containing 149	**1,214**	6,69E-04	35,066	42,979
TGFBR3	transforming growth factor beta receptor 3	**1,214**	7,53E-04	94,376	115,591
FTH1	ferritin heavy chain 1	**1,214**	7,53E-04	1509,012	1829,749
P4HA3	prolyl 4-hydroxylase subunit alpha 3	**1,214**	8,12E-04	32,445	41,014
EFNA1	ephrin A1	**1,214**	1,54E-03	21,075	26,001
PKD2	polycystin 2, transient receptor potential cation channel	**1,214**	1,54E-03	126,298	155,652
HLF	HLF, PAR bZIP transcription factor	**1,214**	1,59E-03	18,126	22,042
ANKRD10	ankyrin repeat domain 10	**1,214**	2,51E-03	46,055	57,230
SYNE3	spectrin repeat containing nuclear envelope family member 3	**1,214**	3,49E-03	43,752	54,954
JUP	junction plakoglobin	**1,214**	3,63E-03	14,283	17,844
PGM2L1	phosphoglucomutase 2 like 1	**1,214**	5,79E-03	18,582	23,201
C14orf28	chromosome 14 open reading frame 28	**1,214**	7,54E-03	8,325	10,428
SLC25A29	solute carrier family 25 member 29	**1,214**	9,43E-03	9,124	11,528
ACSF2	acyl-CoA synthetase family member 2	**1,214**	1,23E-02	8,806	10,987
HECW1	HECT, C2 and WW domain containing E3 ubiquitin protein ligase 1	**1,214**	1,36E-02	6,645	8,430
FN3K	fructosamine 3 kinase	**1,214**	1,45E-02	4,953	6,291
BDNF-AS	BDNF antisense RNA	**1,214**	1,58E-02	4,447	5,598
WHRN	whirlin	**1,214**	1,90E-02	3,983	5,095
ZMYND12	zinc finger MYND-type containing 12	**1,214**	2,08E-02	3,584	4,574
FNIP2	folliculin interacting protein 2	**1,214**	2,49E-02	103,150	129,171
PRKAR2B	protein kinase cAMP-dependent type II regulatory subunit beta	**1,214**	2,87E-02	5,311	6,747
MT1XP1	metallothionein 1X pseudogene 1	**1,214**	3,23E-02	2,173	4,474
TMEM176A	transmembrane protein 176A	**1,214**	3,56E-02	2,627	3,473

TMEM140	transmembrane protein 140	**1,214**	4,18E-02	3,041	4,088
COLGALT2	collagen beta(1-O)galactosyltransferase 2	**1,206**	7,19E-05	234,455	287,286
C1RL	complement C1r subcomponent like	**1,206**	1,24E-04	39,777	48,939
CALCOCO1	calcium binding and coiled-coil domain 1	**1,206**	1,72E-04	71,273	86,746
DDR2	discoidin domain receptor tyrosine kinase 2	**1,206**	2,23E-04	390,774	477,145
ZNF608	zinc finger protein 608	**1,206**	2,30E-04	21,102	25,783
PBXIP1	PBX homeobox interacting protein 1	**1,206**	5,46E-04	110,582	133,852
SOD3	superoxide dismutase 3	**1,206**	6,02E-04	1585,896	1926,237
CEMIP	cell migration inducing hyaluronan binding protein	**1,206**	6,69E-04	68,470	84,843
ZNF84	zinc finger protein 84	**1,206**	7,06E-04	18,663	22,731
ADAMTSL2	ADAMTS like 2	**1,206**	7,89E-04	114,328	139,819
CDO1	cysteine dioxygenase type 1	**1,206**	8,02E-04	166,129	197,060
APOD	apolipoprotein D	**1,206**	8,47E-04	171,341	204,160
FLCN	folliculin	**1,206**	8,65E-04	25,537	31,159
TPP1	tripeptidyl peptidase 1	**1,206**	1,39E-03	112,530	136,294
LVRN	laeverin	**1,206**	2,40E-03	50,749	59,754
PARP14	poly(ADP-ribose) polymerase family member 14	**1,206**	2,60E-03	14,884	18,383
ZNF358	zinc finger protein 358	**1,206**	4,04E-03	26,913	33,127
ZNF181	zinc finger protein 181	**1,206**	5,50E-03	12,669	15,701
ARID5A	AT-rich interaction domain 5A	**1,206**	5,65E-03	11,498	14,176
SPHK1	sphingosine kinase 1	**1,206**	8,50E-03	14,265	17,721
ZNF135	zinc finger protein 135	**1,206**	1,40E-02	4,863	6,123
CFAP54	cilia and flagella associated protein 54	**1,206**	1,41E-02	7,258	9,011
MYOC	myocilin	**1,206**	2,00E-02	4,706	5,932
MT1G	metallothionein 1G	**1,206**	2,38E-02	872,555	2239,921
KANTR	KDM5C adjacent transcript	**1,206**	2,46E-02	5,213	6,529
ZNF204P	zinc finger protein 204, pseudogene	**1,206**	2,54E-02	5,013	6,286
LRRC37A3	leucine rich repeat containing 37 member A3	**1,206**	2,76E-02	3,848	4,909
KBTBD3	kelch repeat and BTB domain containing 3	**1,206**	3,09E-02	3,681	4,667
IFIH1	interferon induced with helicase C domain 1	**1,206**	3,12E-02	3,203	4,089
NLRC5	NLR family CARD domain containing 5	**1,206**	3,73E-02	3,331	4,296
BCL2L11	BCL2 like 11	**1,206**	4,83E-02	3,708	4,803
FAM20A	FAM20A, golgi associated secretory pathway pseudokinase	**1,206**	4,83E-02	5,421	6,577

PC	pyruvate carboxylase	**1,197**	2,48E-04	92,918	111,352
ATXN1	ataxin 1	**1,197**	2,85E-04	40,727	49,167
TSC22D1	TSC22 domain family member 1	**1,197**	3,01E-04	742,881	905,278
FYCO1	FYVE and coiled-coil domain containing 1	**1,197**	4,46E-04	46,444	56,056
BCAT1	branched chain amino acid transaminase 1	**1,197**	5,03E-04	580,317	702,031
ISLR	immunoglobulin superfamily containing leucine rich repeat	**1,197**	7,44E-04	543,952	654,732
MT-CYB	mitochondrially encoded cytochrome b	**1,197**	7,63E-04	703,254	852,357
CTSB	cathepsin B	**1,197**	8,70E-04	195,336	232,904
NUPR1	nuclear protein 1, transcriptional regulator	**1,197**	1,03E-03	198,512	240,985
HHIPL2	HHIP like 2	**1,197**	1,72E-03	33,739	40,631
JMY	junction mediating and regulatory protein, p53 cofactor	**1,197**	1,80E-03	22,641	27,581
KIAA0355	KIAA0355	**1,197**	1,81E-03	13,220	16,050
SOX4	SRY-box 4	**1,197**	2,10E-03	113,499	139,678
ALDH1A2	aldehyde dehydrogenase 1 family member A2	**1,197**	2,84E-03	137,982	162,339
STON1	stonin 1	**1,197**	3,39E-03	12,412	15,124
SLC5A3	solute carrier family 5 member 3	**1,197**	3,51E-03	316,453	377,386
COQ8A	coenzyme Q8A	**1,197**	4,09E-03	14,165	17,312
SLC6A6	solute carrier family 6 member 6	**1,197**	4,23E-03	16,039	19,366
WDR19	WD repeat domain 19	**1,197**	5,44E-03	21,753	26,453
TSHZ1	teashirt zinc finger homeobox 1	**1,197**	6,13E-03	20,504	25,083
DPP4	dipeptidyl peptidase 4	**1,197**	6,98E-03	36,782	45,084
IFI16	interferon gamma inducible protein 16	**1,197**	7,05E-03	25,742	31,139
GPR137B	G protein-coupled receptor 137B	**1,197**	7,31E-03	11,233	13,937
STARD9	StAR related lipid transfer domain containing 9	**1,197**	1,38E-02	9,196	11,276
DIO3	iodothyronine deiodinase 3	**1,197**	1,61E-02	5,748	7,169
SOX9-AS1	SOX9 antisense RNA 1	**1,197**	1,88E-02	4,647	5,761
LINC00702	long intergenic non-protein coding RNA 702	**1,197**	2,25E-02	16,143	19,362
CX3CL1	C-X3-C motif chemokine ligand 1	**1,197**	2,25E-02	4,539	5,632
DIO3OS	DIO3 opposite strand/antisense RNA (head to head)	**1,197**	2,71E-02	5,333	6,652
PCLO	piccolo presynaptic cytomatrix protein	**1,197**	2,75E-02	6,133	7,611
ZMAT1	zinc finger matrin-type 1	**1,197**	3,26E-02	4,101	5,112
HMCN1	hemicentin 1	**1,197**	3,60E-02	4,941	6,065
AKR1C2	aldo-keto reductase family 1 member C2	**1,197**	3,82E-02	78,534	85,696

ZBTB16	zinc finger and BTB domain containing 16	**1,197**	3,91E-02	4,248	5,322
TNFRSF1B	TNF receptor superfamily member 1B	**1,197**	4,98E-02	4,353	5,554
MT-CO1	mitochondrially encoded cytochrome c oxidase I	**1,189**	9,34E-05	1621,801	1954,853
HDAC9	histone deacetylase 9	**1,189**	1,41E-04	32,542	38,757
MAN2A2	mannosidase alpha class 2A member 2	**1,189**	2,30E-04	54,800	65,683
LAMB3	laminin subunit beta 3	**1,189**	2,36E-04	235,604	281,509
HMGA1	high mobility group AT-hook 1	**1,189**	2,76E-04	240,751	292,046
INPP4A	inositol polyphosphate-4-phosphatase type I A	**1,189**	2,85E-04	32,910	39,412
SSTR5-AS1	SSTR5 antisense RNA 1	**1,189**	4,46E-04	41,472	49,765
TPD52L1	tumor protein D52 like 1	**1,189**	5,49E-04	223,244	264,975
IFITM1	interferon induced transmembrane protein 1	**1,189**	6,08E-04	29,932	35,872
SORT1	sortilin 1	**1,189**	8,51E-04	130,383	152,291
ENPP1	ectonucleotide pyrophosphatase/phosphodiesterase 1	**1,189**	8,54E-04	404,790	484,635
VAT1	vesicle amine transport 1	**1,189**	1,05E-03	411,099	494,736
NTRK2	neurotrophic receptor tyrosine kinase 2	**1,189**	1,15E-03	30,237	37,135
SLC7A8	solute carrier family 7 member 8	**1,189**	1,24E-03	34,620	42,255
OLFM1	olfactomedin 1	**1,189**	1,75E-03	64,861	78,136
F13A1	coagulation factor XIII A chain	**1,189**	2,12E-03	360,473	421,388
PCED1A	PC-esterase domain containing 1A	**1,189**	2,22E-03	18,112	21,800
CHI3L1	chitinase 3 like 1	**1,189**	2,38E-03	10285,154	12171,156
GPX3	glutathione peroxidase 3	**1,189**	3,52E-03	1187,078	1397,463
PCDH9	protocadherin 9	**1,189**	4,31E-03	18,853	23,218
MPZL2	myelin protein zero like 2	**1,189**	5,32E-03	24,744	30,227
ARHGEF3	Rho guanine nucleotide exchange factor 3	**1,189**	7,29E-03	17,646	21,181
RNF145	ring finger protein 145	**1,189**	8,74E-03	80,156	96,723
RAB40B	RAB40B, member RAS oncogene family	**1,189**	9,30E-03	11,574	14,257
ARHGAP32	Rho GTPase activating protein 32	**1,189**	1,07E-02	10,400	12,666
APOL3	apolipoprotein L3	**1,189**	1,17E-02	18,474	22,719
TSPAN14	tetraspanin 14	**1,189**	1,19E-02	15,728	19,225
UBE2L6	ubiquitin conjugating enzyme E2 L6	**1,189**	1,42E-02	9,961	12,075
L3MBTL3	l(3)mbt-like 3 (Drosophila)	**1,189**	1,53E-02	7,248	8,808
BCAS4	breast carcinoma amplified sequence 4	**1,189**	1,73E-02	6,466	7,922
AKR1C1	aldo-keto reductase family 1 member C1	**1,189**	1,80E-02	20,316	25,418

GRIN2A	glutamate ionotropic receptor NMDA type subunit 2A	**1,189**	1,84E-02	7,605	9,180
MZF1	myeloid zinc finger 1	**1,189**	1,92E-02	11,604	14,093
AVIL	advillin	**1,189**	2,87E-02	5,219	6,445
LYRM9	LYR motif containing 9	**1,189**	3,00E-02	7,063	8,731
C14orf93	chromosome 14 open reading frame 93	**1,189**	3,30E-02	5,535	6,829
AQP7	aquaporin 7	**1,189**	3,57E-02	6,563	7,981
MMP1	matrix metallopeptidase 1	**1,189**	3,90E-02	699,767	901,253
IL17RD	interleukin 17 receptor D	**1,189**	4,25E-02	5,189	6,530
HSF4	heat shock transcription factor 4	**1,189**	4,71E-02	4,462	5,485
ITGA10	integrin subunit alpha 10	**1,181**	1,18E-04	594,942	704,977
MT-ND4	mitochondrially encoded NADH:ubiquinone oxidoreductase core s	**1,181**	1,94E-04	1975,881	2352,305
SSBP3	single stranded DNA binding protein 3	**1,181**	2,72E-04	34,480	41,009
VPS37B	VPS37B, ESCRT-I subunit	**1,181**	4,25E-04	73,604	88,189
GSN	gelsolin	**1,181**	4,49E-04	656,925	776,182
UTRN	utrophin	**1,181**	4,86E-04	106,504	126,043
PAN2	PAN2 poly(A) specific ribonuclease subunit	**1,181**	7,47E-04	30,595	36,429
SPATA13	spermatogenesis associated 13	**1,181**	7,53E-04	33,587	39,692
CYTL1	cytokine like 1	**1,181**	8,60E-04	313,304	375,403
MANBA	mannosidase beta	**1,181**	1,24E-03	61,830	73,620
NRP2	neuropilin 2	**1,181**	1,28E-03	262,736	316,700
MGP	matrix Gla protein	**1,181**	1,57E-03	11525,452	13560,800
CA5B	carbonic anhydrase 5B	**1,181**	1,62E-03	41,683	50,256
PLEKHM3	pleckstrin homology domain containing M3	**1,181**	1,69E-03	13,928	16,667
EPS8	epidermal growth factor receptor pathway substrate 8	**1,181**	1,80E-03	557,734	660,891
GAB2	GRB2 associated binding protein 2	**1,181**	1,81E-03	24,325	29,167
EZH1	enhancer of zeste 1 polycomb repressive complex 2 subunit	**1,181**	2,32E-03	30,632	36,677
GABARAPL1	GABA type A receptor associated protein like 1	**1,181**	2,61E-03	37,895	45,071
NOVA1	NOVA alternative splicing regulator 1	**1,181**	3,43E-03	81,445	96,177
CALHM2	calcium homeostasis modulator 2	**1,181**	3,50E-03	39,654	47,808
ZFYVE26	zinc finger FYVE-type containing 26	**1,181**	3,63E-03	20,732	24,692
KLF12	Kruppel like factor 12	**1,181**	4,47E-03	17,748	21,202
SELENOP	selenoprotein P	**1,181**	7,86E-03	47,091	56,016
PCDHGB6	protocadherin gamma subfamily B, 6	**1,181**	8,45E-03	12,257	14,599

CD27-AS1	CD27 antisense RNA 1	**1,181**	8,54E-03	12,359	14,884
COL4A3BP	collagen type IV alpha 3 binding protein	**1,181**	8,66E-03	37,177	44,515
PUS10	pseudouridylate synthase 10	**1,181**	1,20E-02	22,607	26,754
TSPOAP1	TSPO associated protein 1	**1,181**	1,43E-02	7,625	9,260
AR	androgen receptor	**1,181**	1,50E-02	9,684	11,743
PCDHGB7	protocadherin gamma subfamily B, 7	**1,181**	1,56E-02	16,978	20,234
RIPOR3	RIPOR family member 3	**1,181**	1,85E-02	10,222	11,862
CARF	calcium responsive transcription factor	**1,181**	2,55E-02	8,272	10,033
PCDHGA5	protocadherin gamma subfamily A, 5	**1,181**	2,75E-02	6,381	7,753
TMEM198B	transmembrane protein 198B (pseudogene)	**1,181**	3,19E-02	5,388	6,584
TDRP	testis development related protein	**1,181**	3,29E-02	5,324	6,457
TMEM200A	transmembrane protein 200A	**1,181**	3,71E-02	4,890	6,010
SWT1	SWT1, RNA endoribonuclease homolog	**1,181**	3,94E-02	5,503	6,744
QRICH2	glutamine rich 2	**1,181**	4,60E-02	4,938	5,984
SCARA3	scavenger receptor class A member 3	**1,173**	1,35E-04	633,061	747,392
IQSEC2	IQ motif and Sec7 domain 2	**1,173**	6,64E-04	25,104	29,525
MT-ND4L	mitochondrially encoded NADH:ubiquinone oxidoreductase core s	**1,173**	8,47E-04	194,564	232,463
CLSTN3	calsyntenin 3	**1,173**	9,30E-04	24,849	29,307
PTPRD	protein tyrosine phosphatase, receptor type D	**1,173**	1,05E-03	37,937	44,581
RAB11FIP4	RAB11 family interacting protein 4	**1,173**	1,23E-03	76,896	91,038
SESTD1	SEC14 and spectrin domain containing 1	**1,173**	1,54E-03	63,842	75,846
THNSL2	threonine synthase like 2	**1,173**	1,68E-03	20,400	24,124
EPB41L1	erythrocyte membrane protein band 4.1 like 1	**1,173**	2,11E-03	82,211	96,249
NR1D1	nuclear receptor subfamily 1 group D member 1	**1,173**	2,24E-03	101,463	119,506
FBXO44	F-box protein 44	**1,173**	2,50E-03	20,596	24,471
EIF4B	eukaryotic translation initiation factor 4B	**1,173**	2,68E-03	245,317	289,855
LINC00963	long intergenic non-protein coding RNA 963	**1,173**	3,20E-03	30,507	36,058
ABCA3	ATP binding cassette subfamily A member 3	**1,173**	3,90E-03	17,824	21,169
EPHX2	epoxide hydrolase 2	**1,173**	4,04E-03	19,291	22,965
SLC40A1	solute carrier family 40 member 1	**1,173**	4,23E-03	59,809	71,326
PTGS1	prostaglandin-endoperoxide synthase 1	**1,173**	5,02E-03	38,232	45,627
RAB29	RAB29, member RAS oncogene family	**1,173**	5,04E-03	38,682	45,848
SH3GLB2	SH3 domain containing GRB2 like, endophilin B2	**1,173**	5,18E-03	25,867	30,751

SP4	Sp4 transcription factor	**1,173**	7,70E-03	14,072	16,675
TMEM255B	transmembrane protein 255B	**1,173**	8,02E-03	35,104	40,356
TRIM66	tripartite motif containing 66	**1,173**	8,07E-03	14,671	17,408
BMP6	bone morphogenetic protein 6	**1,173**	8,23E-03	69,765	84,743
BDH2	3-hydroxybutyrate dehydrogenase 2	**1,173**	1,04E-02	11,849	14,082
LIFR	LIF receptor alpha	**1,173**	1,07E-02	61,208	72,619
FOXO4	forkhead box O4	**1,173**	1,31E-02	10,541	12,496
HS1BP3	HCLS1 binding protein 3	**1,173**	1,34E-02	21,108	25,123
SLC37A2	solute carrier family 37 member 2	**1,173**	1,37E-02	37,216	41,678
PLCD4	phospholipase C delta 4	**1,173**	1,71E-02	9,089	10,821
AQP3	aquaporin 3 (Gill blood group)	**1,173**	1,71E-02	15,233	17,731
CDH19	cadherin 19	**1,173**	2,01E-02	15,125	18,049
ZBTB26	zinc finger and BTB domain containing 26	**1,173**	2,27E-02	8,082	9,635
ZNF771	zinc finger protein 771	**1,173**	2,72E-02	6,873	8,318
STK32A	serine/threonine kinase 32A	**1,173**	3,17E-02	7,233	8,750
AUH	AU RNA binding methylglutaconyl-CoA hydratase	**1,173**	3,19E-02	11,119	13,455
MIR4697HG	MIR4697 host gene	**1,173**	3,95E-02	5,440	6,642
CARD6	caspase recruitment domain family member 6	**1,173**	4,84E-02	8,958	10,587
PLXNB1	plexin B1	**1,165**	7,19E-05	98,804	114,781
COLEC12	collectin subfamily member 12	**1,165**	1,37E-04	106,720	125,218
MT-ATP8	mitochondrially encoded ATP synthase 8	**1,165**	3,02E-04	75,529	88,460
ROR2	receptor tyrosine kinase like orphan receptor 2	**1,165**	3,84E-04	75,063	87,845
CHAD	chondroadherin	**1,165**	4,52E-04	591,508	685,277
PLBD2	phospholipase B domain containing 2	**1,165**	6,02E-04	161,475	187,145
AKAP13	A-kinase anchoring protein 13	**1,165**	6,74E-04	60,985	71,273
CLU	clusterin	**1,165**	7,47E-04	11080,688	12883,133
ITIH6	inter-alpha-trypsin inhibitor heavy chain family member 6	**1,165**	1,37E-03	330,329	387,563
GAA	glucosidase alpha, acid	**1,165**	1,79E-03	99,188	114,490
CD55	CD55 molecule (Cromer blood group)	**1,165**	1,84E-03	1703,166	1969,236
PSAP	prosaposin	**1,165**	1,85E-03	1884,375	2178,779
TRIM2	tripartite motif containing 2	**1,165**	2,06E-03	25,297	29,541
NEBL	nebulette	**1,165**	2,16E-03	414,689	486,054
AGPAT4	1-acylglycerol-3-phosphate O-acyltransferase 4	**1,165**	2,26E-03	25,150	29,442

KLHL21	kelch like family member 21	**1,165**	2,30E-03	148,443	174,594
ERMAP	erythroblast membrane associated protein (Scianna blood group)	**1,165**	3,12E-03	19,222	22,650
USP6NL	USP6 N-terminal like	**1,165**	3,26E-03	55,847	65,342
FLYWCH1	FLYWCH-type zinc finger 1	**1,165**	3,48E-03	38,513	45,353
TWSG1	twisted gastrulation BMP signaling modulator 1	**1,165**	3,77E-03	347,661	410,248
IGF2BP2	insulin like growth factor 2 mRNA binding protein 2	**1,165**	3,91E-03	14,360	16,888
SYNM	synemin	**1,165**	4,01E-03	166,148	193,720
PLA2R1	phospholipase A2 receptor 1	**1,165**	4,82E-03	31,202	36,731
FNDC1	fibronectin type III domain containing 1	**1,165**	5,00E-03	86,812	104,175
SERPINE2	serpin family E member 2	**1,165**	6,23E-03	5548,332	6498,285
TRIM56	tripartite motif containing 56	**1,165**	6,42E-03	54,778	64,245
CTSD	cathepsin D	**1,165**	7,59E-03	988,585	1129,857
GLB1L	galactosidase beta 1 like	**1,165**	1,00E-02	16,842	19,800
CTC1	CST telomere replication complex component 1	**1,165**	1,01E-02	13,003	15,346
FZD7	frizzled class receptor 7	**1,165**	1,10E-02	29,489	34,850
HNMT	histamine N-methyltransferase	**1,165**	1,15E-02	39,727	46,663
TMOD1	tropomodulin 1	**1,165**	1,24E-02	13,216	15,659
SMIM29	small integral membrane protein 29	**1,165**	1,40E-02	18,150	21,427
PSMG3-AS1	PSMG3 antisense RNA 1 (head to head)	**1,165**	2,15E-02	11,643	13,750
PLCG2	phospholipase C gamma 2	**1,165**	2,24E-02	21,642	25,432
ANG	angiogenin	**1,165**	2,83E-02	10,345	12,273
KIAA1324L	KIAA1324 like	**1,165**	3,07E-02	12,759	15,166
PIWIL4	piwi like RNA-mediated gene silencing 4	**1,165**	3,57E-02	11,912	14,148
MMP10	matrix metallopeptidase 10	**1,165**	3,61E-02	7,544	9,010
WDR27	WD repeat domain 27	**1,165**	3,70E-02	8,289	9,933
AK9	adenylate kinase 9	**1,165**	3,90E-02	8,066	9,666
TOP1MT	topoisomerase (DNA) I, mitochondrial	**1,165**	4,06E-02	9,745	11,577
PPOX	protoporphyrinogen oxidase	**1,165**	4,50E-02	8,913	10,481
PRRT3	proline rich transmembrane protein 3	**1,165**	4,52E-02	6,231	7,380
LINC00847	long intergenic non-protein coding RNA 847	**1,165**	4,88E-02	7,595	9,122
BOC	BOC cell adhesion associated, oncogene regulated	**1,157**	7,16E-05	111,628	130,617
CHD2	chromodomain helicase DNA binding protein 2	**1,157**	4,64E-04	90,804	105,140
VCAM1	vascular cell adhesion molecule 1	**1,157**	7,06E-04	855,761	987,784

CD276	CD276 molecule	**1,157**	8,02E-04	159,851	186,551
PHF3	PHD finger protein 3	**1,157**	8,64E-04	79,862	92,561
IDS	iduronate 2-sulfatase	**1,157**	1,27E-03	121,141	140,532
EDIL3	EGF like repeats and discoidin domains 3	**1,157**	1,38E-03	1164,582	1358,900
PTPRS	protein tyrosine phosphatase, receptor type S	**1,157**	1,39E-03	76,786	89,627
RNF130	ring finger protein 130	**1,157**	1,65E-03	132,895	154,433
CPAMD8	C3 and PZP like, alpha-2-macroglobulin domain containing 8	**1,157**	2,13E-03	73,890	85,188
ZHX2	zinc fingers and homeoboxes 2	**1,157**	2,23E-03	46,896	54,542
ZBTB44	zinc finger and BTB domain containing 44	**1,157**	2,70E-03	25,539	29,832
MAGI1	membrane associated guanylate kinase, WW and PDZ domain cor	**1,157**	2,88E-03	42,537	48,970
MT-RNR2	mitochondrially encoded 16S RNA	**1,157**	3,00E-03	2553,281	3020,968
KALRN	kalirin, RhoGEF kinase	**1,157**	3,26E-03	38,448	45,208
TCF7L2	transcription factor 7 like 2	**1,157**	3,71E-03	54,166	63,384
ADGRA3	adhesion G protein-coupled receptor A3	**1,157**	4,47E-03	36,828	43,068
EFL1	elongation factor like GTPase 1	**1,157**	4,62E-03	33,358	39,044
PPARA	peroxisome proliferator activated receptor alpha	**1,157**	4,82E-03	38,975	45,375
KIAA0040	KIAA0040	**1,157**	5,05E-03	101,565	118,734
NDRG1	N-myc downstream regulated 1	**1,157**	5,43E-03	561,935	648,319
ZFYVE1	zinc finger FYVE-type containing 1	**1,157**	5,45E-03	21,440	25,075
MINDY1	MINDY lysine 48 deubiquitinase 1	**1,157**	5,54E-03	19,371	22,459
FCGRT	Fc fragment of IgG receptor and transporter	**1,157**	5,67E-03	76,878	89,796
ABCB9	ATP binding cassette subfamily B member 9	**1,157**	5,78E-03	38,551	45,125
NOP53	NOP53 ribosome biogenesis factor	**1,157**	6,17E-03	115,296	134,600
HECA	hdc homolog, cell cycle regulator	**1,157**	6,30E-03	47,863	55,629
CDK19	cyclin dependent kinase 19	**1,157**	7,61E-03	21,566	25,182
RETREG2	reticulophagy regulator family member 2	**1,157**	7,86E-03	99,363	116,167
PIK3IP1	phosphoinositide-3-kinase interacting protein 1	**1,157**	1,00E-02	19,510	22,929
GLMP	glycosylated lysosomal membrane protein	**1,157**	1,01E-02	45,053	52,134
INPP4B	inositol polyphosphate-4-phosphatase type II B	**1,157**	1,02E-02	17,398	20,276
VWA5A	von Willebrand factor A domain containing 5A	**1,157**	1,10E-02	29,270	34,019
CDH11	cadherin 11	**1,157**	1,15E-02	104,860	125,614
ZC3H6	zinc finger CCCH-type containing 6	**1,157**	1,20E-02	18,097	21,123
KDSR	3-ketodihydrosphingosine reductase	**1,157**	1,43E-02	104,815	122,425

SNN	stannin	**1,157**	1,90E-02	18,582	21,891
LEPR	leptin receptor	**1,157**	1,99E-02	12,137	14,536
SORBS2	sorbin and SH3 domain containing 2	**1,157**	2,15E-02	15,650	18,765
ADAMTS6	ADAM metallopeptidase with thrombospondin type 1 motif 6	**1,157**	2,22E-02	16,365	19,334
ING4	inhibitor of growth family member 4	**1,157**	2,23E-02	15,621	18,314
GRAMD4	GRAM domain containing 4	**1,157**	2,46E-02	40,399	47,801
SERPINI1	serpin family I member 1	**1,157**	2,55E-02	42,389	49,525
RAI2	retinoic acid induced 2	**1,157**	2,59E-02	10,394	12,195
CPEB3	cytoplasmic polyadenylation element binding protein 3	**1,157**	2,69E-02	8,110	9,587
TPT1-AS1	TPT1 antisense RNA 1	**1,157**	2,83E-02	9,815	11,549
RNASET2	ribonuclease T2	**1,157**	2,99E-02	9,192	10,839
KIAA1614	KIAA1614	**1,157**	3,31E-02	11,422	13,356
BANK1	B-cell scaffold protein with ankyrin repeats 1	**1,157**	3,44E-02	15,122	17,803
MYPOP	Myb related transcription factor, partner of profilin	**1,157**	3,48E-02	7,236	8,544
ZNF524	zinc finger protein 524	**1,157**	3,76E-02	13,794	16,271
THUMPD3-AS1	THUMPD3 antisense RNA 1	**1,157**	4,45E-02	6,632	7,807
RNPC3	RNA binding region (RNP1, RRM) containing 3	**1,157**	4,68E-02	5,760	6,871
CAPS	calcyphosine	**1,149**	2,50E-04	58,411	67,183
IQSEC1	IQ motif and Sec7 domain 1	**1,149**	6,08E-04	186,267	215,014
CRTAC1	cartilage acidic protein 1	**1,149**	1,01E-03	1031,826	1186,350
MT-ATP6	mitochondrially encoded ATP synthase 6	**1,149**	1,13E-03	684,661	790,588
PARM1	prostate androgen-regulated mucin-like protein 1	**1,149**	1,27E-03	54,670	62,649
ITGB5	integrin subunit beta 5	**1,149**	1,31E-03	2234,874	2593,839
TSC1	tuberous sclerosis 1	**1,149**	1,38E-03	33,170	38,204
SEMA3E	semaphorin 3E	**1,149**	1,51E-03	103,575	119,412
SCIN	scinderin	**1,149**	1,58E-03	261,106	301,390
SLC1A1	solute carrier family 1 member 1	**1,149**	1,60E-03	75,754	87,431
STAT6	signal transducer and activator of transcription 6	**1,149**	1,81E-03	155,915	179,567
CTSK	cathepsin K	**1,149**	1,91E-03	124,016	142,590
ADAMTSL3	ADAMTS like 3	**1,149**	2,26E-03	111,147	127,851
RNF24	ring finger protein 24	**1,149**	2,65E-03	102,572	118,168
RERE	arginine-glutamic acid dipeptide repeats	**1,149**	3,50E-03	77,622	89,639
F5	coagulation factor V	**1,149**	3,50E-03	28,092	32,586

LGALS3	galectin 3	**1,149**	3,50E-03	375,419	430,952
TGFBR2	transforming growth factor beta receptor 2	**1,149**	4,07E-03	433,021	498,785
FAM83G	family with sequence similarity 83 member G	**1,149**	4,10E-03	41,370	47,946
MAPRE2	microtubule associated protein RP/EB family member 2	**1,149**	4,80E-03	128,344	146,640
VGLL4	vestigial like family member 4	**1,149**	4,85E-03	134,313	155,654
SLC12A6	solute carrier family 12 member 6	**1,149**	4,90E-03	31,925	37,046
KIAA1109	KIAA1109	**1,149**	4,91E-03	52,217	60,417
SIK2	salt inducible kinase 2	**1,149**	5,50E-03	40,949	47,428
GRN	granulin precursor	**1,149**	5,54E-03	310,480	355,605
IGBP1	immunoglobulin binding protein 1	**1,149**	5,74E-03	38,209	44,459
RSRP1	arginine and serine rich protein 1	**1,149**	6,13E-03	37,364	43,320
GNPDA1	glucosamine-6-phosphate deaminase 1	**1,149**	6,24E-03	58,626	67,604
CLK1	CDC like kinase 1	**1,149**	6,40E-03	79,848	92,360
KMT2A	lysine methyltransferase 2A	**1,149**	6,71E-03	65,487	76,165
LAMA4	laminin subunit alpha 4	**1,149**	6,98E-03	76,657	89,214
ARMCX6	armadillo repeat containing, X-linked 6	**1,149**	7,16E-03	15,719	18,250
FNIP1	folliculin interacting protein 1	**1,149**	7,47E-03	32,259	37,241
RCAN3	RCAN family member 3	**1,149**	7,52E-03	22,413	26,125
TNRC6B	trinucleotide repeat containing 6B	**1,149**	9,34E-03	26,520	30,875
MAPK8IP3	mitogen-activated protein kinase 8 interacting protein 3	**1,149**	9,63E-03	45,647	52,782
SLC25A23	solute carrier family 25 member 23	**1,149**	1,03E-02	19,804	23,044
ARHGAP42	Rho GTPase activating protein 42	**1,149**	1,12E-02	23,073	26,631
TRIM38	tripartite motif containing 38	**1,149**	1,15E-02	29,341	33,873
OSBPL1A	oxysterol binding protein like 1A	**1,149**	1,23E-02	32,440	37,577
GAREM1	GRB2 associated regulator of MAPK1 subtype 1	**1,149**	1,27E-02	34,482	39,523
NME3	NME/NM23 nucleoside diphosphate kinase 3	**1,149**	1,43E-02	17,778	20,794
ZKSCAN8	zinc finger with KRAB and SCAN domains 8	**1,149**	1,58E-02	26,026	30,255
DENND4C	DENN domain containing 4C	**1,149**	1,71E-02	29,334	33,817
PHYH	phytanoyl-CoA 2-hydroxylase	**1,149**	1,88E-02	24,299	28,038
RBPMS2	RNA binding protein with multiple splicing 2	**1,149**	1,95E-02	15,852	18,592
YPEL3	yippee like 3	**1,149**	2,08E-02	27,790	32,059
SLC9A7	solute carrier family 9 member A7	**1,149**	2,10E-02	19,598	22,603
COL18A1	collagen type XVIII alpha 1 chain	**1,149**	2,25E-02	18,266	21,215

AMPD3	adenosine monophosphate deaminase 3	**1,149**	2,33E-02	13,925	16,386
DNAJC4	DnaJ heat shock protein family (Hsp40) member C4	**1,149**	2,69E-02	11,177	13,012
PSMD5-AS1	PSMD5 antisense RNA 1 (head to head)	**1,149**	3,09E-02	48,321	44,516
CNNM2	cyclin and CBS domain divalent metal cation transport mediator 2	**1,149**	3,09E-02	11,129	12,975
CCDC28A	coiled-coil domain containing 28A	**1,149**	3,11E-02	11,056	12,896
DNASE1	deoxyribonuclease 1	**1,149**	3,52E-02	12,136	14,169
ACADS	acyl-CoA dehydrogenase, C-2 to C-3 short chain	**1,149**	3,64E-02	12,212	14,212
ZFP28	ZFP28 zinc finger protein	**1,149**	3,73E-02	7,858	9,200
COA5	cytochrome c oxidase assembly factor 5	**1,149**	3,83E-02	13,166	15,429
ZBTB18	zinc finger and BTB domain containing 18	**1,149**	3,95E-02	23,964	28,137
ZNF581	zinc finger protein 581	**1,149**	3,95E-02	10,023	11,819
ZNF224	zinc finger protein 224	**1,149**	4,06E-02	9,540	11,178
HINFP	histone H4 transcription factor	**1,149**	4,18E-02	9,002	10,569
TTLL3	tubulin tyrosine ligase like 3	**1,149**	4,24E-02	8,709	10,175
MIPOL1	mirror-image polydactyly 1	**1,149**	4,95E-02	11,893	13,763
DDX17	DEAD-box helicase 17	**1,141**	7,07E-04	311,363	357,242
PLD3	phospholipase D family member 3	**1,141**	1,51E-03	317,315	362,233
ZKSCAN1	zinc finger with KRAB and SCAN domains 1	**1,141**	1,80E-03	86,173	98,943
ABCA2	ATP binding cassette subfamily A member 2	**1,141**	2,87E-03	82,447	94,652
APP	amyloid beta precursor protein	**1,141**	3,23E-03	553,876	632,107
SPPL3	signal peptide peptidase like 3	**1,141**	3,42E-03	51,349	58,916
PCYOX1	prenylcysteine oxidase 1	**1,141**	3,48E-03	177,446	203,156
SAMD4A	sterile alpha motif domain containing 4A	**1,141**	3,76E-03	160,566	183,935
BCR	BCR, RhoGEF and GTPase activating protein	**1,141**	4,07E-03	48,757	56,035
LAG3	lymphocyte activating 3	**1,141**	4,77E-03	50,663	58,012
SLC25A37	solute carrier family 25 member 37	**1,141**	5,65E-03	174,811	199,312
PSD3	pleckstrin and Sec7 domain containing 3	**1,141**	5,79E-03	205,251	234,365
CFLAR	CASP8 and FADD like apoptosis regulator	**1,141**	5,98E-03	30,880	35,512
CLDN12	claudin 12	**1,141**	6,63E-03	54,321	62,396
GJA1	gap junction protein alpha 1	**1,141**	7,57E-03	168,933	193,802
THBS4	thrombospondin 4	**1,141**	8,41E-03	55,144	64,220
FUCA1	fucosidase, alpha-L- 1, tissue	**1,141**	9,41E-03	47,046	54,041
ALDH6A1	aldehyde dehydrogenase 6 family member A1	**1,141**	9,80E-03	27,299	31,325

MAOB	monoamine oxidase B	**1,141**	1,08E-02	92,033	105,066
MMP2	matrix metallopeptidase 2	**1,141**	1,08E-02	34,066	40,163
SLC35E2B	solute carrier family 35 member E2B	**1,141**	1,11E-02	34,982	40,929
LRIG1	leucine rich repeats and immunoglobulin like domains 1	**1,141**	1,12E-02	20,788	23,908
SYF2	SYF2 pre-mRNA splicing factor	**1,141**	1,17E-02	49,341	56,534
MAML2	mastermind like transcriptional coactivator 2	**1,141**	1,28E-02	29,355	33,818
ZNF844	zinc finger protein 844	**1,141**	1,48E-02	17,241	19,877
PNRC1	proline rich nuclear receptor coactivator 1	**1,141**	1,50E-02	63,170	72,265
TYMP	thymidine phosphorylase	**1,141**	1,60E-02	28,565	32,438
SOBP	sine oculis binding protein homolog	**1,141**	1,63E-02	41,988	48,077
FOXA3	forkhead box A3	**1,141**	1,84E-02	14,419	16,698
DSG2	desmoglein 2	**1,141**	1,85E-02	59,401	65,653
FOS	Fos proto-oncogene, AP-1 transcription factor subunit	**1,141**	1,93E-02	19,713	22,683
FAM210B	family with sequence similarity 210 member B	**1,141**	2,29E-02	61,681	70,880
KLC4	kinesin light chain 4	**1,141**	2,38E-02	15,499	17,831
RFTN2	raftlin family member 2	**1,141**	2,71E-02	28,028	32,005
DUSP22	dual specificity phosphatase 22	**1,141**	2,84E-02	40,193	46,698
IRF2	interferon regulatory factor 2	**1,141**	2,87E-02	14,129	16,320
SPSB3	splA/ryanodine receptor domain and SOCS box containing 3	**1,141**	3,14E-02	22,188	25,507
AP1S2	adaptor related protein complex 1 sigma 2 subunit	**1,141**	3,31E-02	19,664	22,804
GID4	GID complex subunit 4 homolog	**1,141**	3,57E-02	15,605	18,002
SAV1	salvador family WW domain containing protein 1	**1,141**	3,95E-02	103,467	113,823
AGT	angiotensinogen	**1,141**	4,03E-02	17,468	19,636
ASB13	ankyrin repeat and SOCS box containing 13	**1,141**	4,06E-02	17,429	20,020
RTL5	retrotransposon Gag like 5	**1,141**	4,47E-02	12,210	14,065
TLR4	toll like receptor 4	**1,141**	4,55E-02	9,228	10,671
DCHS1	dachsous cadherin-related 1	**1,133**	2,48E-04	182,747	207,358
AHNAK	AHNAK nucleoprotein	**1,133**	7,49E-04	2549,306	2907,806
NAB2	NGFI-A binding protein 2	**1,133**	8,19E-04	71,126	80,739
SULF2	sulfatase 2	**1,133**	1,19E-03	232,432	263,762
SERPING1	serpin family G member 1	**1,133**	1,23E-03	232,623	267,160
S100A1	S100 calcium binding protein A1	**1,133**	1,28E-03	537,543	613,035
ARHGAP1	Rho GTPase activating protein 1	**1,133**	1,35E-03	160,639	182,632

ANKH	ANKH inorganic pyrophosphate transport regulator	**1,133**	1,37E-03	1086,550	1233,177
CTDSP2	CTD small phosphatase 2	**1,133**	1,57E-03	272,433	308,146
PLEKHA4	pleckstrin homology domain containing A4	**1,133**	1,84E-03	73,309	83,469
CILP	cartilage intermediate layer protein	**1,133**	2,25E-03	1469,697	1645,038
SPOCK1	SPARC/osteonectin, cwcv and kazal like domains proteoglycan 1	**1,133**	2,61E-03	205,197	229,254
TRPS1	transcriptional repressor GATA binding 1	**1,133**	2,88E-03	177,717	202,210
NORAD	non-coding RNA activated by DNA damage	**1,133**	3,02E-03	458,839	520,745
HIP1	huntingtin interacting protein 1	**1,133**	3,52E-03	76,482	86,942
SARAF	store-operated calcium entry associated regulatory factor	**1,133**	4,01E-03	229,693	259,796
WBP2	WW domain binding protein 2	**1,133**	4,31E-03	148,638	168,180
SEMA4C	semaphorin 4C	**1,133**	5,04E-03	49,015	55,646
CCPG1	cell cycle progression 1	**1,133**	5,28E-03	118,351	133,436
SRPX2	sushi repeat containing protein, X-linked 2	**1,133**	5,64E-03	260,052	295,202
MBD6	methyl-CpG binding domain protein 6	**1,133**	6,24E-03	29,002	33,189
SERTAD2	SERTA domain containing 2	**1,133**	6,27E-03	159,291	180,936
SPRY2	sprouty RTK signaling antagonist 2	**1,133**	6,42E-03	113,388	129,205
GAS7	growth arrest specific 7	**1,133**	7,67E-03	90,170	103,880
USF2	upstream transcription factor 2, c-fos interacting	**1,133**	8,13E-03	92,168	104,705
ZNF385D	zinc finger protein 385D	**1,133**	8,39E-03	125,450	140,521
DIP2C	disco interacting protein 2 homolog C	**1,133**	9,14E-03	63,107	72,096
ICAM1	intercellular adhesion molecule 1	**1,133**	9,44E-03	139,436	157,005
AFF1	AF4/FMR2 family member 1	**1,133**	1,12E-02	55,742	63,265
RGCC	regulator of cell cycle	**1,133**	1,15E-02	343,413	391,383
TOM1	target of myb1 membrane trafficking protein	**1,133**	1,15E-02	54,749	61,963
SPEN	spen family transcriptional repressor	**1,133**	1,19E-02	72,702	82,663
MNT	MAX network transcriptional repressor	**1,133**	1,19E-02	20,764	23,733
MTHFR	methylenetetrahydrofolate reductase	**1,133**	1,23E-02	25,611	29,230
PTPRU	protein tyrosine phosphatase, receptor type U	**1,133**	1,28E-02	45,422	51,621
CDH23	cadherin related 23	**1,133**	1,41E-02	23,998	27,316
LCN2	lipocalin 2	**1,133**	1,43E-02	101,673	114,623
LTA4H	leukotriene A4 hydrolase	**1,133**	1,56E-02	65,724	74,913
C2orf68	chromosome 2 open reading frame 68	**1,133**	1,59E-02	21,268	24,311
MAN1A1	mannosidase alpha class 1A member 1	**1,133**	1,74E-02	144,111	164,995

KIAA1549	KIAA1549	**1,133**	1,92E-02	28,187	32,095
THBS3	thrombospondin 3	**1,133**	2,07E-02	18,727	21,369
ABCC5	ATP binding cassette subfamily C member 5	**1,133**	2,29E-02	16,045	18,430
AOC3	amine oxidase, copper containing 3	**1,133**	2,30E-02	22,505	25,641
GPRASP1	G protein-coupled receptor associated sorting protein 1	**1,133**	2,44E-02	29,728	34,290
DPYD	dihydropyrimidine dehydrogenase	**1,133**	2,48E-02	26,293	30,013
SPSB1	splA/ryanodine receptor domain and SOCS box containing 1	**1,133**	2,48E-02	51,560	58,714
ATG2A	autophagy related 2A	**1,133**	2,71E-02	18,774	21,442
HLA-B	major histocompatibility complex, class I, B	**1,133**	2,83E-02	18,698	21,358
FIG4	FIG4 phosphoinositide 5-phosphatase	**1,133**	2,83E-02	16,548	18,778
PRICKLE1	prickle planar cell polarity protein 1	**1,133**	3,02E-02	19,402	22,136
ECHDC2	enoyl-CoA hydratase domain containing 2	**1,133**	3,17E-02	18,661	21,375
TFPI2	tissue factor pathway inhibitor 2	**1,133**	3,33E-02	18,323	20,887
NMT2	N-myristoyltransferase 2	**1,133**	3,44E-02	20,253	23,090
RIPK2	receptor interacting serine/threonine kinase 2	**1,133**	3,61E-02	19,223	22,059
VEGFB	vascular endothelial growth factor B	**1,133**	3,88E-02	38,420	43,483
LETMD1	LETM1 domain containing 1	**1,133**	3,94E-02	18,433	21,184
USP30	ubiquitin specific peptidase 30	**1,133**	4,07E-02	11,997	13,700
C15orf40	chromosome 15 open reading frame 40	**1,133**	4,27E-02	12,773	14,629
CLK4	CDC like kinase 4	**1,133**	4,46E-02	11,292	12,916
RUBCNL	RUN and cysteine rich domain containing beclin 1 interacting prot	**1,133**	5,00E-02	18,697	21,435
FURIN	furin, paired basic amino acid cleaving enzyme	**1,125**	2,07E-04	222,225	250,750
MEF2D	myocyte enhancer factor 2D	**1,125**	6,19E-04	99,681	112,025
PPDPF	pancreatic progenitor cell differentiation and proliferation factor	**1,125**	6,32E-04	274,238	310,103
CUL7	cullin 7	**1,125**	7,06E-04	96,822	109,254
LTBP3	latent transforming growth factor beta binding protein 3	**1,125**	1,75E-03	792,726	895,201
TNS3	tensin 3	**1,125**	1,84E-03	490,922	554,228
FGD5	FYVE, RhoGEF and PH domain containing 5	**1,125**	2,22E-03	49,398	55,469
PRG4	proteoglycan 4	**1,125**	2,25E-03	766,261	885,148
MELTF	melanotransferrin	**1,125**	2,60E-03	773,896	870,918
TPT1	tumor protein, translationally-controlled 1	**1,125**	2,65E-03	1229,802	1384,451
NEO1	neogenin 1	**1,125**	2,67E-03	92,173	103,953
ZNF592	zinc finger protein 592	**1,125**	3,52E-03	44,179	49,842

UBR4	ubiquitin protein ligase E3 component n-recognin 4	**1,125**	3,54E-03	129,409	145,976
MTATP6P1	mitochondrially encoded ATP synthase 6 pseudogene 1	**1,125**	4,98E-03	148,053	168,072
FBXL7	F-box and leucine rich repeat protein 7	**1,125**	5,84E-03	48,217	54,515
GIGYF1	GRB10 interacting GYF protein 1	**1,125**	5,84E-03	49,708	56,108
FRZB	frizzled-related protein	**1,125**	5,98E-03	242,680	276,167
H6PD	hexose-6-phosphate dehydrogenase/glucose 1-dehydrogenase	**1,125**	6,24E-03	137,930	155,503
EIF4G3	eukaryotic translation initiation factor 4 gamma 3	**1,125**	6,40E-03	113,943	128,065
PPP3CA	protein phosphatase 3 catalytic subunit alpha	**1,125**	6,50E-03	328,729	371,461
C1S	complement C1s	**1,125**	7,29E-03	692,936	776,656
MAGI2-AS3	MAGI2 antisense RNA 3	**1,125**	9,40E-03	53,664	59,912
ASH1L	ASH1 like histone lysine methyltransferase	**1,125**	9,41E-03	87,587	99,405
MOXD1	monooxygenase DBH like 1	**1,125**	1,00E-02	324,346	366,622
ABTB2	ankyrin repeat and BTB domain containing 2	**1,125**	1,01E-02	47,371	53,426
PLEKHM2	pleckstrin homology and RUN domain containing M2	**1,125**	1,08E-02	75,759	85,710
CD109	CD109 molecule	**1,125**	1,12E-02	402,243	453,791
HECTD4	HECT domain E3 ubiquitin protein ligase 4	**1,125**	1,20E-02	28,234	31,704
CRISPLD1	cysteine rich secretory protein LCCL domain containing 1	**1,125**	1,24E-02	38,310	43,594
CHMP4B	charged multivesicular body protein 4B	**1,125**	1,25E-02	144,710	163,945
DSP	desmoplakin	**1,125**	1,31E-02	57,428	64,807
MINDY2	MINDY lysine 48 deubiquitinase 2	**1,125**	1,31E-02	41,876	47,207
MKRN1	makorin ring finger protein 1	**1,125**	1,36E-02	63,647	71,965
ITIH5	inter-alpha-trypsin inhibitor heavy chain family member 5	**1,125**	1,37E-02	36,235	40,835
GNS	glucosamine (N-acetyl)-6-sulfatase	**1,125**	1,43E-02	218,369	244,846
ACVR1	activin A receptor type 1	**1,125**	1,47E-02	163,509	184,742
SBSPON	somatomedin B and thrombospondin type 1 domain containing	**1,125**	1,48E-02	32,704	36,893
PLBD1	phospholipase B domain containing 1	**1,125**	1,57E-02	29,729	33,831
PHC2	polyhomeotic homolog 2	**1,125**	1,63E-02	112,517	127,422
PCDHGA2	protocadherin gamma subfamily A, 2	**1,125**	1,68E-02	27,283	31,031
DHPS	deoxyhypusine synthase	**1,125**	1,71E-02	27,834	31,537
SP100	SP100 nuclear antigen	**1,125**	1,84E-02	48,661	55,245
KCTD21	potassium channel tetramerization domain containing 21	**1,125**	1,97E-02	17,740	20,094
GABRA4	gamma-aminobutyric acid type A receptor alpha4 subunit	**1,125**	2,01E-02	67,354	76,632
ZNF397	zinc finger protein 397	**1,125**	2,38E-02	28,696	32,587

AKAP1	A-kinase anchoring protein 1	**1,125**	2,43E-02	40,889	45,353
INSR	insulin receptor	**1,125**	2,48E-02	35,815	40,648
AFTPH	aftiphilin	**1,125**	2,49E-02	48,840	54,985
LINC00346	long intergenic non-protein coding RNA 346	**1,125**	2,50E-02	16,813	18,974
DDIT3	DNA damage inducible transcript 3	**1,125**	2,51E-02	32,022	36,095
GM2A	GM2 ganglioside activator	**1,125**	2,60E-02	38,067	42,935
STEAP2	STEAP2 metalloreductase	**1,125**	2,72E-02	33,375	37,595
NFIL3	nuclear factor, interleukin 3 regulated	**1,125**	2,77E-02	57,714	65,241
VRK3	vaccinia related kinase 3	**1,125**	2,93E-02	20,128	22,827
CHD6	chromodomain helicase DNA binding protein 6	**1,125**	2,93E-02	23,434	26,516
WSB1	WD repeat and SOCS box containing 1	**1,125**	3,10E-02	71,707	80,500
SAP30L	SAP30 like	**1,125**	3,95E-02	35,065	39,716
ZNF407	zinc finger protein 407	**1,125**	4,01E-02	13,779	15,610
SLC26A4	solute carrier family 26 member 4	**1,125**	4,11E-02	40,244	45,524
ARHGEF10L	Rho guanine nucleotide exchange factor 10 like	**1,125**	4,16E-02	25,347	28,631
ZADH2	zinc binding alcohol dehydrogenase domain containing 2	**1,125**	4,46E-02	25,537	28,925
SATB1	SATB homeobox 1	**1,125**	4,77E-02	15,905	17,958
MBTPS1	membrane bound transcription factor peptidase, site 1	**1,117**	7,23E-04	223,218	248,300
GLG1	golgi glycoprotein 1	**1,117**	1,17E-03	501,748	562,130
LRP10	LDL receptor related protein 10	**1,117**	1,40E-03	870,989	976,334
KIF13B	kinesin family member 13B	**1,117**	1,76E-03	175,386	195,065
OS9	OS9, endoplasmic reticulum lectin	**1,117**	1,87E-03	477,116	531,284
TNS1	tensin 1	**1,117**	2,28E-03	161,424	180,873
TIMP2	TIMP metallopeptidase inhibitor 2	**1,117**	3,23E-03	942,099	1049,114
CDON	cell adhesion associated, oncogene regulated	**1,117**	3,83E-03	223,725	250,807
EEF2	eukaryotic translation elongation factor 2	**1,117**	3,91E-03	1871,732	2097,855
CNBP	CCHC-type zinc finger nucleic acid binding protein	**1,117**	4,92E-03	312,763	350,609
HDAC5	histone deacetylase 5	**1,117**	6,40E-03	81,201	90,808
MYCBP2	MYC binding protein 2, E3 ubiquitin protein ligase	**1,117**	6,46E-03	61,485	69,097
CYBRD1	cytochrome b reductase 1	**1,117**	6,56E-03	593,971	666,237
KDM3B	lysine demethylase 3B	**1,117**	7,11E-03	51,915	58,373
ITGAV	integrin subunit alpha V	**1,117**	7,16E-03	235,093	262,741
LAMC1	laminin subunit gamma 1	**1,117**	7,70E-03	173,936	194,522

FN1	fibronectin 1	**1,117**	7,76E-03	102943,562	113693,412
OGT	O-linked N-acetylglucosamine (GlcNAc) transferase	**1,117**	7,86E-03	103,086	115,509
SMOC1	SPARC related modular calcium binding 1	**1,117**	8,27E-03	239,894	269,421
GAS1	growth arrest specific 1	**1,117**	8,33E-03	181,232	204,635
PAM	peptidylglycine alpha-amidating monooxygenase	**1,117**	9,14E-03	588,026	653,746
ENOSF1	enolase superfamily member 1	**1,117**	9,14E-03	30,417	34,167
MT-CO2	mitochondrially encoded cytochrome c oxidase II	**1,117**	9,40E-03	903,751	1011,493
PRMT2	protein arginine methyltransferase 2	**1,117**	9,62E-03	107,758	120,990
CELSR1	cadherin EGF LAG seven-pass G-type receptor 1	**1,117**	1,09E-02	78,962	88,754
PNISR	PNN interacting serine and arginine rich protein	**1,117**	1,13E-02	50,033	56,140
PRKAB2	protein kinase AMP-activated non-catalytic subunit beta 2	**1,117**	1,16E-02	23,929	26,873
GHR	growth hormone receptor	**1,117**	1,20E-02	35,108	39,369
CAMKK1	calcium/calmodulin dependent protein kinase kinase 1	**1,117**	1,23E-02	26,044	29,086
ZFP36	ZFP36 ring finger protein	**1,117**	1,23E-02	60,083	67,227
C12orf57	chromosome 12 open reading frame 57	**1,117**	1,24E-02	95,814	107,790
FTL	ferritin light chain	**1,117**	1,30E-02	2062,035	2248,449
CSRP1	cysteine and glycine rich protein 1	**1,117**	1,30E-02	163,851	182,854
KMT2E	lysine methyltransferase 2E	**1,117**	1,44E-02	72,402	80,639
ZBTB40	zinc finger and BTB domain containing 40	**1,117**	1,47E-02	28,552	31,980
NTN1	netrin 1	**1,117**	1,50E-02	421,870	470,784
DNASE2	deoxyribonuclease 2, lysosomal	**1,117**	1,50E-02	63,029	70,405
SLC3A2	solute carrier family 3 member 2	**1,117**	1,55E-02	216,282	242,935
ST5	suppression of tumorigenicity 5	**1,117**	1,56E-02	43,145	48,445
RETREG3	reticulophagy regulator family member 3	**1,117**	1,59E-02	40,966	45,754
CYSTM1	cysteine rich transmembrane module containing 1	**1,117**	1,70E-02	40,265	45,256
CD14	CD14 molecule	**1,117**	1,75E-02	76,599	85,578
BTD	biotinidase	**1,117**	1,78E-02	22,555	25,350
KIAA2026	KIAA2026	**1,117**	1,85E-02	48,781	54,489
GAB1	GRB2 associated binding protein 1	**1,117**	1,85E-02	46,888	52,402
HEXA	hexosaminidase subunit alpha	**1,117**	1,93E-02	89,441	100,256
BICRAL	BRD4 interacting chromatin remodelling complex associated prot	**1,117**	1,94E-02	30,676	34,429
TF	transferrin	**1,117**	2,00E-02	105,770	118,017
XIST	X inactive specific transcript (non-protein coding)	**1,117**	2,28E-02	211,945	241,702

ARSA	arylsulfatase A	**1,117**	2,30E-02	39,867	44,356
C19orf12	chromosome 19 open reading frame 12	**1,117**	2,42E-02	33,982	38,169
TOLLIP	toll interacting protein	**1,117**	2,55E-02	82,690	92,676
TBC1D14	TBC1 domain family member 14	**1,117**	2,65E-02	35,364	39,620
PACS2	phosphofurin acidic cluster sorting protein 2	**1,117**	2,65E-02	61,497	68,760
CPQ	carboxypeptidase Q	**1,117**	2,69E-02	54,714	61,035
ZNF766	zinc finger protein 766	**1,117**	2,89E-02	18,103	20,326
TMEM25	transmembrane protein 25	**1,117**	2,93E-02	26,205	29,417
ZNF740	zinc finger protein 740	**1,117**	2,93E-02	24,831	27,952
MORC3	MORC family CW-type zinc finger 3	**1,117**	2,97E-02	28,203	31,657
FOXP1	forkhead box P1	**1,117**	3,07E-02	42,725	48,224
CD164	CD164 molecule	**1,117**	3,24E-02	470,365	524,029
RECQL5	RecQ like helicase 5	**1,117**	3,27E-02	19,501	21,932
PRDM11	PR/SET domain 11	**1,117**	3,70E-02	15,625	17,610
RSAD1	radical S-adenosyl methionine domain containing 1	**1,117**	3,73E-02	29,062	32,729
ZNF32	zinc finger protein 32	**1,117**	3,76E-02	17,960	20,207
CGNL1	cingulin like 1	**1,117**	3,83E-02	39,513	44,379
ULK1	unc-51 like autophagy activating kinase 1	**1,117**	3,99E-02	27,413	30,977
SIRT5	sirtuin 5	**1,117**	4,01E-02	13,673	15,425
ECSIT	ECSIT signalling integrator	**1,117**	4,21E-02	18,290	20,588
NAGPA	N-acetylglucosamine-1-phosphodiester alpha-N-acetylglucosamin	**1,117**	4,27E-02	15,753	17,764
ARID5B	AT-rich interaction domain 5B	**1,117**	4,65E-02	38,835	43,668
SPON1	spondin 1	**1,117**	4,65E-02	27,123	31,176
RGS10	regulator of G protein signaling 10	**1,117**	4,66E-02	21,687	24,340
TRNP1	TMF1-regulated nuclear protein 1	**1,117**	4,67E-02	19,778	22,116
CSPG4	chondroitin sulfate proteoglycan 4	**1,110**	9,61E-04	1789,020	1999,182
ACAN	aggrecan	**1,110**	1,38E-03	11285,035	12490,758
FXYD6	FXYD domain containing ion transport regulator 6	**1,110**	1,92E-03	194,939	216,647
WWTR1	WW domain containing transcription regulator 1	**1,110**	3,78E-03	468,260	518,502
PACSIN2	protein kinase C and casein kinase substrate in neurons 2	**1,110**	6,15E-03	125,544	138,985
MFGE8	milk fat globule-EGF factor 8 protein	**1,110**	6,24E-03	2110,818	2328,589
SOX9	SRY-box 9	**1,110**	6,93E-03	251,649	280,147
MT-ND1	mitochondrially encoded NADH:ubiquinone oxidoreductase core s	**1,110**	7,40E-03	407,384	454,645

CNPY3	canopy FGF signaling regulator 3	**1,110**	7,64E-03	73,030	81,240
AKAP9	A-kinase anchoring protein 9	**1,110**	7,90E-03	124,460	137,331
CLMP	CXADR like membrane protein	**1,110**	8,13E-03	596,250	663,590
PRRC2B	proline rich coiled-coil 2B	**1,110**	9,95E-03	232,171	258,531
MXD4	MAX dimerization protein 4	**1,110**	1,00E-02	78,571	86,942
TM9SF2	transmembrane 9 superfamily member 2	**1,110**	1,01E-02	319,223	353,080
MIR100HG	mir-100-let-7a-2 cluster host gene	**1,110**	1,04E-02	187,150	209,181
LTBP1	latent transforming growth factor beta binding protein 1	**1,110**	1,08E-02	324,134	358,315
ZNFX1	zinc finger NFX1-type containing 1	**1,110**	1,08E-02	87,213	96,660
ARMCX1	armadillo repeat containing, X-linked 1	**1,110**	1,08E-02	60,383	67,551
WDTC1	WD and tetratricopeptide repeats 1	**1,110**	1,09E-02	60,650	67,259
GUSB	glucuronidase beta	**1,110**	1,10E-02	59,420	66,594
GOLGB1	golgin B1	**1,110**	1,10E-02	135,974	151,077
SPTBN1	spectrin beta, non-erythrocytic 1	**1,110**	1,18E-02	269,702	300,085
AMFR	autocrine motility factor receptor	**1,110**	1,25E-02	130,756	145,069
MAVS	mitochondrial antiviral signaling protein	**1,110**	1,26E-02	95,329	106,177
SVIL	supervillin	**1,110**	1,30E-02	97,693	108,871
AOC2	amine oxidase, copper containing 2	**1,110**	1,30E-02	165,289	184,369
CHRDL2	chordin like 2	**1,110**	1,36E-02	39,502	44,308
ZNF532	zinc finger protein 532	**1,110**	1,44E-02	81,789	90,917
SUMF1	sulfatase modifying factor 1	**1,110**	1,53E-02	59,452	65,830
MFSD10	major facilitator superfamily domain containing 10	**1,110**	1,54E-02	52,534	58,782
SUSD5	sushi domain containing 5	**1,110**	1,57E-02	267,901	296,341
CPD	carboxypeptidase D	**1,110**	1,57E-02	175,802	196,115
PTPRE	protein tyrosine phosphatase, receptor type E	**1,110**	1,64E-02	70,580	77,519
LIMCH1	LIM and calponin homology domains 1	**1,110**	1,66E-02	113,474	125,903
EPAS1	endothelial PAS domain protein 1	**1,110**	1,79E-02	346,785	386,198
NPDC1	neural proliferation, differentiation and control 1	**1,110**	1,83E-02	57,298	63,779
FOXK1	forkhead box K1	**1,110**	1,99E-02	56,160	62,778
EIF3L	eukaryotic translation initiation factor 3 subunit L	**1,110**	2,08E-02	88,384	98,501
GPRC5C	G protein-coupled receptor class C group 5 member C	**1,110**	2,18E-02	108,531	120,283
STX7	syntaxin 7	**1,110**	2,25E-02	83,234	92,145
OSMR	oncostatin M receptor	**1,110**	2,26E-02	484,869	533,895

CSGALNACT2	chondroitin sulfate N-acetylgalactosaminyltransferase 2	**1,110**	2,40E-02	61,882	69,431
BHLHE41	basic helix-loop-helix family member e41	**1,110**	2,41E-02	64,204	70,771
PRDM2	PR/SET domain 2	**1,110**	2,42E-02	33,687	37,401
KCTD2	potassium channel tetramerization domain containing 2	**1,110**	2,44E-02	46,761	52,176
NOMO2	NODAL modulator 2	**1,110**	2,54E-02	33,699	37,388
SLC39A14	solute carrier family 39 member 14	**1,110**	2,60E-02	1742,417	1940,794
ANXA4	annexin A4	**1,110**	2,64E-02	113,156	126,194
IDUA	iduronidase, alpha-L-	**1,110**	2,68E-02	22,228	24,839
DPP7	dipeptidyl peptidase 7	**1,110**	2,82E-02	90,923	101,452
C8orf82	chromosome 8 open reading frame 82	**1,110**	2,86E-02	31,484	35,288
PIAS1	protein inhibitor of activated STAT 1	**1,110**	2,95E-02	32,903	36,625
ANKRD50	ankyrin repeat domain 50	**1,110**	2,99E-02	81,121	89,240
CD302	CD302 molecule	**1,110**	3,06E-02	26,337	29,261
SNHG14	small nucleolar RNA host gene 14	**1,110**	3,06E-02	34,977	38,963
HDAC4	histone deacetylase 4	**1,110**	3,10E-02	27,112	30,371
DST	dystonin	**1,110**	3,11E-02	404,634	450,353
TCF4	transcription factor 4	**1,110**	3,17E-02	117,099	129,099
RFX1	regulatory factor X1	**1,110**	3,45E-02	19,548	21,803
MED12	mediator complex subunit 12	**1,110**	3,46E-02	32,243	35,816
NRIP1	nuclear receptor interacting protein 1	**1,110**	3,57E-02	50,105	55,907
ZFC3H1	zinc finger C3H1-type containing	**1,110**	3,59E-02	31,180	34,987
RETSAT	retinol saturase	**1,110**	3,85E-02	30,551	33,955
EP300	E1A binding protein p300	**1,110**	3,89E-02	58,372	64,912
CTSL	cathepsin L	**1,110**	3,98E-02	491,574	550,487
PRICKLE2	prickle planar cell polarity protein 2	**1,110**	4,39E-02	37,480	41,727
ZBTB1	zinc finger and BTB domain containing 1	**1,110**	4,49E-02	34,632	38,732
VPS13A	vacuolar protein sorting 13 homolog A	**1,110**	4,62E-02	22,275	24,818
TSPYL4	TSPY like 4	**1,110**	4,71E-02	25,981	28,811
SRRM2	serine/arginine repetitive matrix 2	**1,102**	2,12E-03	278,068	306,479
CLSTN1	calsyntenin 1	**1,102**	3,30E-03	383,294	422,847
LRP1	LDL receptor related protein 1	**1,102**	3,55E-03	2503,486	2752,361
FGFBP2	fibroblast growth factor binding protein 2	**1,102**	5,64E-03	1428,143	1580,448
TIMP3	TIMP metallopeptidase inhibitor 3	**1,102**	7,05E-03	900,833	996,365

LAMP1	lysosomal associated membrane protein 1	**1,102**	7,52E-03	483,357	531,141
APLP2	amyloid beta precursor like protein 2	**1,102**	8,81E-03	778,022	857,757
SMARCA2	SWI/SNF related, matrix associated, actin dependent regulator of	**1,102**	9,63E-03	126,317	139,788
HAPLN1	hyaluronan and proteoglycan link protein 1	**1,102**	1,00E-02	1983,517	2200,170
TIMP1	TIMP metallopeptidase inhibitor 1	**1,102**	1,09E-02	1560,032	1743,856
MAN2B2	mannosidase alpha class 2B member 2	**1,102**	1,21E-02	105,539	116,344
GLT8D2	glycosyltransferase 8 domain containing 2	**1,102**	1,21E-02	53,248	58,871
CTSA	cathepsin A	**1,102**	1,40E-02	178,556	196,491
KIDINS220	kinase D interacting substrate 220	**1,102**	1,53E-02	99,529	110,231
JMJD1C	jumonji domain containing 1C	**1,102**	1,61E-02	59,934	66,333
FGFR2	fibroblast growth factor receptor 2	**1,102**	1,74E-02	127,349	139,999
LDB1	LIM domain binding 1	**1,102**	1,91E-02	32,605	36,124
C1R	complement C1r	**1,102**	1,94E-02	222,492	243,978
ATMIN	ATM interactor	**1,102**	1,94E-02	52,626	58,396
RB1CC1	RB1 inducible coiled-coil 1	**1,102**	2,03E-02	62,404	68,733
STAT5B	signal transducer and activator of transcription 5B	**1,102**	2,25E-02	54,987	60,717
MEGF8	multiple EGF like domains 8	**1,102**	2,38E-02	117,674	130,591
PDPN	podoplanin	**1,102**	2,45E-02	488,756	536,118
ZFAND5	zinc finger AN1-type containing 5	**1,102**	2,48E-02	234,034	258,065
ABHD2	abhydrolase domain containing 2	**1,102**	2,55E-02	242,727	268,013
AFDN	afadin, adherens junction formation factor	**1,102**	2,57E-02	66,771	73,473
THRB	thyroid hormone receptor beta	**1,102**	2,69E-02	49,452	54,669
NFATC1	nuclear factor of activated T-cells 1	**1,102**	2,84E-02	67,750	74,682
RAPGEF1	Rap guanine nucleotide exchange factor 1	**1,102**	2,99E-02	51,247	56,844
LRRC8A	leucine rich repeat containing 8 family member A	**1,102**	3,06E-02	88,573	98,192
RBL2	RB transcriptional corepressor like 2	**1,102**	3,11E-02	75,130	82,589
TMX4	thioredoxin related transmembrane protein 4	**1,102**	3,20E-02	170,769	188,182
ZNF496	zinc finger protein 496	**1,102**	3,29E-02	27,471	30,273
A4GALT	alpha 1,4-galactosyltransferase (P blood group)	**1,102**	3,35E-02	42,331	46,880
ITM2B	integral membrane protein 2B	**1,102**	3,46E-02	900,660	991,569
VPS13C	vacuolar protein sorting 13 homolog C	**1,102**	3,46E-02	42,828	47,528
SCPEP1	serine carboxypeptidase 1	**1,102**	3,46E-02	87,775	96,755
HEXB	hexosaminidase subunit beta	**1,102**	3,47E-02	170,542	189,028

PNPLA2	patatin like phospholipase domain containing 2	**1,102**	3,47E-02	119,984	131,899
BTBD2	BTB domain containing 2	**1,102**	3,54E-02	60,426	66,925
ERAP1	endoplasmic reticulum aminopeptidase 1	**1,102**	3,65E-02	39,787	44,053
BRI3	brain protein I3	**1,102**	3,73E-02	162,047	179,189
CILP2	cartilage intermediate layer protein 2	**1,102**	4,04E-02	152,291	171,082
GGA2	golgi associated, gamma adaptin ear containing, ARF binding prot	**1,102**	4,08E-02	48,614	53,709
GPX4	glutathione peroxidase 4	**1,102**	4,17E-02	196,062	215,594
CYB5D2	cytochrome b5 domain containing 2	**1,102**	4,19E-02	43,880	48,662
KIF13A	kinesin family member 13A	**1,102**	4,25E-02	54,589	59,956
PALD1	phosphatase domain containing, paladin 1	**1,102**	4,67E-02	24,546	26,958
USP20	ubiquitin specific peptidase 20	**1,102**	4,74E-02	20,486	22,662
CIC	capicua transcriptional repressor	**1,094**	3,63E-03	129,936	142,409
NUCB1	nucleobindin 1	**1,094**	4,89E-03	401,527	437,671
ATP8B2	ATPase phospholipid transporting 8B2	**1,094**	6,21E-03	346,178	378,900
S100B	S100 calcium binding protein B	**1,094**	6,77E-03	106,493	116,954
PIGT	phosphatidylinositol glycan anchor biosynthesis class T	**1,094**	8,02E-03	157,747	172,454
ARFGEF2	ADP ribosylation factor guanine nucleotide exchange factor 2	**1,094**	9,29E-03	120,094	131,013
ITM2C	integral membrane protein 2C	**1,094**	9,76E-03	333,941	365,318
WDR6	WD repeat domain 6	**1,094**	1,13E-02	125,287	137,251
SECISBP2L	SECIS binding protein 2 like	**1,094**	1,18E-02	87,108	95,628
COL6A2	collagen type VI alpha 2 chain	**1,094**	1,23E-02	3939,489	4329,266
UBR5	ubiquitin protein ligase E3 component n-recognin 5	**1,094**	1,32E-02	107,829	118,518
LRPAP1	LDL receptor related protein associated protein 1	**1,094**	1,36E-02	166,342	182,111
DYNC1H1	dynein cytoplasmic 1 heavy chain 1	**1,094**	1,43E-02	302,183	330,966
HTRA1	HtrA serine peptidase 1	**1,094**	1,44E-02	1079,352	1184,251
MMP3	matrix metallopeptidase 3	**1,094**	1,53E-02	11316,899	12444,860
XPC	XPC complex subunit, DNA damage recognition and repair factor	**1,094**	1,58E-02	45,377	49,682
TNK2	tyrosine kinase non receptor 2	**1,094**	1,61E-02	66,048	72,290
IQGAP1	IQ motif containing GTPase activating protein 1	**1,094**	1,66E-02	607,684	667,776
NR1H2	nuclear receptor subfamily 1 group H member 2	**1,094**	1,72E-02	85,712	93,535
ITFG1	integrin alpha FG-GAP repeat containing 1	**1,094**	1,77E-02	127,855	140,225
CSGALNACT1	chondroitin sulfate N-acetylgalactosaminyltransferase 1	**1,094**	1,87E-02	255,224	277,886
GLI3	GLI family zinc finger 3	**1,094**	1,99E-02	67,037	73,330

LPIN2	lipin 2	**1,094**	2,15E-02	112,252	122,711
MT-CO3	mitochondrially encoded cytochrome c oxidase III	**1,094**	2,38E-02	735,419	802,813
PPFIBP1	PPFIA binding protein 1	**1,094**	2,43E-02	262,565	288,264
WSCD2	WSC domain containing 2	**1,094**	2,45E-02	70,379	77,087
CST3	cystatin C	**1,094**	2,45E-02	270,017	294,378
FBXO2	F-box protein 2	**1,094**	2,61E-02	106,876	115,399
PTPN18	protein tyrosine phosphatase, non-receptor type 18	**1,094**	2,62E-02	43,370	47,366
PEX5	peroxisomal biogenesis factor 5	**1,094**	2,65E-02	53,916	59,362
EGR1	early growth response 1	**1,094**	2,69E-02	92,104	102,192
TMEM59	transmembrane protein 59	**1,094**	2,76E-02	364,933	399,194
PCOLCE2	procollagen C-endopeptidase enhancer 2	**1,094**	2,78E-02	1534,894	1701,047
CPNE2	copine 2	**1,094**	2,82E-02	62,037	67,704
CD47	CD47 molecule	**1,094**	2,83E-02	93,974	103,123
MAN2A1	mannosidase alpha class 2A member 1	**1,094**	2,93E-02	85,268	93,752
ZNF106	zinc finger protein 106	**1,094**	3,07E-02	65,835	71,920
TMEM246	transmembrane protein 246	**1,094**	3,15E-02	30,879	34,055
CC2D1B	coiled-coil and C2 domain containing 1B	**1,094**	3,31E-02	58,714	64,114
PDLIM5	PDZ and LIM domain 5	**1,094**	3,44E-02	478,561	519,792
TRIM4	tripartite motif containing 4	**1,094**	3,61E-02	47,695	52,685
ST3GAL1	ST3 beta-galactoside alpha-2,3-sialyltransferase 1	**1,094**	3,65E-02	84,755	92,824
CAMK2D	calcium/calmodulin dependent protein kinase II delta	**1,094**	3,67E-02	134,419	148,335
ZER1	zyg-11 related cell cycle regulator	**1,094**	3,78E-02	47,493	52,060
KCNQ5	potassium voltage-gated channel subfamily Q member 5	**1,094**	3,81E-02	137,400	149,762
ZSCAN18	zinc finger and SCAN domain containing 18	**1,094**	3,82E-02	36,445	39,893
ASAH1	N-acylsphingosine amidohydrolase 1	**1,094**	3,85E-02	117,127	129,069
PCGF5	polycomb group ring finger 5	**1,094**	3,96E-02	72,678	79,786
LDLRAD4	low density lipoprotein receptor class A domain containing 4	**1,094**	4,09E-02	37,481	41,150
IGF1R	insulin like growth factor 1 receptor	**1,094**	4,14E-02	45,453	49,981
BTG2	BTG anti-proliferation factor 2	**1,094**	4,17E-02	36,513	40,255
WDR48	WD repeat domain 48	**1,094**	4,29E-02	36,107	39,651
NOTCH2	notch 2	**1,094**	4,30E-02	550,355	604,830
CLIP2	CAP-Gly domain containing linker protein 2	**1,094**	4,34E-02	72,074	79,465
IER5L	immediate early response 5 like	**1,094**	4,37E-02	155,768	170,774

MT-ND3	mitochondrially encoded NADH:ubiquinone oxidoreductase core s	**1,094**	4,40E-02	252,732	278,355
SIK3	SIK family kinase 3	**1,094**	4,55E-02	29,905	32,879
IFNAR1	interferon alpha and beta receptor subunit 1	**1,094**	4,80E-02	80,746	88,330
HAPLN3	hyaluronan and proteoglycan link protein 3	**1,094**	4,85E-02	45,023	49,242
ATF2	activating transcription factor 2	**1,094**	4,91E-02	34,654	38,049
OGFR	opioid growth factor receptor	**1,094**	4,94E-02	35,623	39,043
CD9	CD9 molecule	**1,094**	5,00E-02	230,431	252,595
RARG	retinoic acid receptor gamma	**1,087**	3,14E-03	281,522	306,988
NFE2L1	nuclear factor, erythroid 2 like 1	**1,087**	4,83E-03	772,504	838,825
WWP2	WW domain containing E3 ubiquitin protein ligase 2	**1,087**	8,68E-03	652,804	706,647
SUN2	Sad1 and UNC84 domain containing 2	**1,087**	1,07E-02	352,757	381,394
HECTD1	HECT domain E3 ubiquitin protein ligase 1	**1,087**	1,24E-02	130,259	141,371
CRTAP	cartilage associated protein	**1,087**	1,36E-02	433,575	471,387
MXRA8	matrix remodeling associated 8	**1,087**	1,46E-02	379,155	411,041
ZBTB7A	zinc finger and BTB domain containing 7A	**1,087**	1,54E-02	90,668	98,462
TUSC3	tumor suppressor candidate 3	**1,087**	1,72E-02	143,539	156,259
ATXN7L3B	ataxin 7 like 3B	**1,087**	1,75E-02	69,616	75,871
CD99L2	CD99 molecule like 2	**1,087**	1,89E-02	141,214	154,071
TIAM2	T-cell lymphoma invasion and metastasis 2	**1,087**	2,02E-02	113,506	123,614
SRSF5	serine and arginine rich splicing factor 5	**1,087**	2,06E-02	106,280	115,868
QSOX1	quiescin sulfhydryl oxidase 1	**1,087**	2,06E-02	655,034	707,187
SLC38A1	solute carrier family 38 member 1	**1,087**	2,33E-02	195,001	211,945
SERINC1	serine incorporator 1	**1,087**	2,33E-02	530,863	575,659
ORMDL3	ORMDL sphingolipid biosynthesis regulator 3	**1,087**	2,45E-02	91,546	99,525
NCK2	NCK adaptor protein 2	**1,087**	3,15E-02	125,520	135,964
MPHOSPH8	M-phase phosphoprotein 8	**1,087**	3,21E-02	66,473	72,165
NCOR1	nuclear receptor corepressor 1	**1,087**	3,33E-02	96,263	104,813
TMED4	transmembrane p24 trafficking protein 4	**1,087**	3,34E-02	105,598	114,919
NT5E	5'-nucleotidase ecto	**1,087**	3,44E-02	2278,484	2485,426
UBE2H	ubiquitin conjugating enzyme E2 H	**1,087**	3,95E-02	73,759	80,313
GIGYF2	GRB10 interacting GYF protein 2	**1,087**	4,02E-02	66,920	73,114
ISCU	iron-sulfur cluster assembly enzyme	**1,087**	4,21E-02	53,219	58,048
SMARCC2	SWI/SNF related, matrix associated, actin dependent regulator of	**1,087**	4,23E-02	71,243	77,510

SERPINA1	serpin family A member 1	**1,087**	4,35E-02	418,465	452,418
SCAMP1	secretory carrier membrane protein 1	**1,087**	4,49E-02	75,469	82,013
DAZAP2	DAZ associated protein 2	**1,087**	4,68E-02	127,897	139,107
SH3KBP1	SH3 domain containing kinase binding protein 1	**1,087**	4,68E-02	40,878	44,595
TANC1	tetratricopeptide repeat, ankyrin repeat and coiled-coil containin	**1,087**	4,85E-02	37,566	40,962
SPATA20	spermatogenesis associated 20	**1,087**	4,98E-02	45,749	49,694
DPYSL3	dihydropyrimidinase like 3	**1,079**	1,76E-02	150,034	162,308
IL13RA1	interleukin 13 receptor subunit alpha 1	**1,079**	1,94E-02	288,388	312,745
CAPN2	calpain 2	**1,079**	2,11E-02	326,818	353,739
STAT1	signal transducer and activator of transcription 1	**1,079**	2,68E-02	115,823	125,571
BSG	basigin (Ok blood group)	**1,079**	2,70E-02	451,538	488,094
FMOD	fibromodulin	**1,079**	3,09E-02	4089,316	4391,372
TMED10	transmembrane p24 trafficking protein 10	**1,079**	3,25E-02	568,465	613,717
LTBP4	latent transforming growth factor beta binding protein 4	**1,079**	3,36E-02	97,492	106,215
PEBP1	phosphatidylethanolamine binding protein 1	**1,079**	3,66E-02	207,036	223,626
ADARB1	adenosine deaminase, RNA specific B1	**1,079**	3,73E-02	102,171	110,185
CUX1	cut like homeobox 1	**1,079**	3,97E-02	102,842	111,199
BMPR2	bone morphogenetic protein receptor type 2	**1,079**	4,24E-02	65,210	70,457
RPS4X	ribosomal protein S4, X-linked	**1,079**	4,49E-02	658,106	717,113
PRRC2C	proline rich coiled-coil 2C	**1,079**	4,72E-02	159,515	171,858
NR2C2	nuclear receptor subfamily 2 group C member 2	**1,079**	4,83E-02	60,082	64,699
MSL1	male specific lethal 1 homolog	**1,079**	4,85E-02	85,184	92,291
MIA3	MIA family member 3, ER export factor	**1,079**	4,85E-02	73,059	79,025
GRINA	glutamate ionotropic receptor NMDA type subunit associated pro	**1,079**	5,00E-02	114,767	123,398
CYB5R3	cytochrome b5 reductase 3	**1,072**	1,95E-02	681,798	727,386
TMCO3	transmembrane and coiled-coil domains 3	**1,072**	2,11E-02	301,676	323,002
MAN1B1	mannosidase alpha class 1B member 1	**1,072**	2,36E-02	168,538	180,076
C2orf40	chromosome 2 open reading frame 40	**1,072**	3,02E-02	1541,842	1682,795
BCAP31	B-cell receptor-associated protein 31	**1,072**	3,10E-02	182,106	194,755
SUMF2	sulfatase modifying factor 2	**1,072**	3,61E-02	213,311	228,881
ADAM15	ADAM metallopeptidase domain 15	**1,072**	3,73E-02	180,278	193,184
MSI2	musashi RNA binding protein 2	**1,072**	3,99E-02	111,016	119,369
ITGA5	integrin subunit alpha 5	**1,072**	4,55E-02	542,737	583,167

EBLN3P	endogenous Bornavirus-like nucleoprotein 3, pseudogene	**1,072**	4,58E-02	59,497	63,908
IL1R1	interleukin 1 receptor type 1	**1,072**	4,65E-02	149,105	160,295
EXT2	exostosin glycosyltransferase 2	**1,072**	4,74E-02	328,274	351,068
DNAJB2	DnaJ heat shock protein family (Hsp40) member B2	**1,072**	4,83E-02	94,284	101,229
TTC3	tetratricopeptide repeat domain 3	**1,072**	4,85E-02	250,697	268,190
SERINC3	serine incorporator 3	**1,072**	5,00E-02	230,003	246,346
DDOST	dolichyl-diphosphooligosaccharide--protein glycosyltransferase nc	**1,064**	2,15E-02	374,974	399,303
PRKCSH	protein kinase C substrate 80K-H	**1,064**	2,33E-02	329,863	352,140
VSIR	V-set immunoregulatory receptor	**1,064**	3,43E-02	168,697	179,860
SLC29A1	solute carrier family 29 member 1 (Augustine blood group)	**1,064**	4,52E-02	373,082	397,024
SLC39A6	solute carrier family 39 member 6	**1,064**	4,54E-02	214,326	228,638
GANAB	glucosidase II alpha subunit	**1,057**	3,91E-02	705,402	743,909
LAMB2	laminin subunit beta 2	**1,050**	4,90E-02	515,375	541,330
HSP90AA1	heat shock protein 90 alpha family class A member 1	**-1,057**	3,42E-02	1052,986	994,177
KHDRBS1	KH RNA binding domain containing, signal transduction associatec	**-1,064**	3,82E-02	189,077	176,985
CD46	CD46 molecule	**-1,064**	4,22E-02	202,761	190,403
DNAJA1	DnaJ heat shock protein family (Hsp40) member A1	**-1,064**	4,62E-02	140,886	131,528
VIM	vimentin	**-1,064**	4,63E-02	5136,614	4797,515
HMGB1	high mobility group box 1	**-1,064**	4,92E-02	132,053	123,822
YWHAH	tyrosine 3-monooxygenase/tryptophan 5-monooxygenase activat	**-1,064**	5,00E-02	166,112	155,406
HNRNPK	heterogeneous nuclear ribonucleoprotein K	**-1,072**	1,31E-02	433,171	401,916
HNRNPA0	heterogeneous nuclear ribonucleoprotein A0	**-1,072**	2,00E-02	155,775	145,118
PTP4A2	protein tyrosine phosphatase type IVA, member 2	**-1,072**	2,11E-02	150,653	139,719
GAPDH	glyceraldehyde-3-phosphate dehydrogenase	**-1,072**	2,40E-02	3408,852	3179,739
TRAM1	translocation associated membrane protein 1	**-1,072**	3,04E-02	306,188	285,175
ALKBH5	alkB homolog 5, RNA demethylase	**-1,072**	3,10E-02	105,034	97,616
MYL12B	myosin light chain 12B	**-1,072**	3,28E-02	261,084	242,717
COX7A2	cytochrome c oxidase subunit 7A2	**-1,072**	3,41E-02	94,429	87,860
ADRM1	adhesion regulating molecule 1	**-1,072**	3,44E-02	90,081	83,462
GSPT1	G1 to S phase transition 1	**-1,072**	3,61E-02	155,554	144,626
BUB3	BUB3, mitotic checkpoint protein	**-1,072**	4,17E-02	78,220	72,275
U2AF2	U2 small nuclear RNA auxiliary factor 2	**-1,072**	4,25E-02	154,300	143,620
JTB	jumping translocation breakpoint	**-1,072**	4,45E-02	102,055	95,180

PARK7	Parkinsonism associated deglycase	**-1,072**	4,60E-02	193,362	179,327
HNRNPDL	heterogeneous nuclear ribonucleoprotein D like	**-1,072**	4,83E-02	167,371	155,325
YWHAB	tyrosine 3-monooxygenase/tryptophan 5-monooxygenase activat	**-1,079**	1,09E-02	218,467	202,136
GNAI3	G protein subunit alpha i3	**-1,079**	1,31E-02	97,762	89,897
EIF2S2	eukaryotic translation initiation factor 2 subunit beta	**-1,079**	1,42E-02	111,031	102,421
RBBP7	RB binding protein 7, chromatin remodeling factor	**-1,079**	2,22E-02	93,607	86,214
MORF4L2	mortality factor 4 like 2	**-1,079**	2,68E-02	279,477	258,880
UBQLN1	ubiquilin 1	**-1,079**	2,68E-02	131,566	121,490
VCP	valosin containing protein	**-1,079**	2,69E-02	359,999	332,014
TM4SF1	transmembrane 4 L six family member 1	**-1,079**	3,10E-02	518,370	477,478
CENPB	centromere protein B	**-1,079**	3,10E-02	154,399	142,589
FAM120A	family with sequence similarity 120A	**-1,079**	3,13E-02	181,588	166,746
VDAC1	voltage dependent anion channel 1	**-1,079**	3,13E-02	143,087	131,693
PPP2CB	protein phosphatase 2 catalytic subunit beta	**-1,079**	3,28E-02	122,742	113,311
BCL2L1	BCL2 like 1	**-1,079**	3,48E-02	96,574	88,862
DDX1	DEAD-box helicase 1	**-1,079**	3,69E-02	158,048	145,598
PRDX3	peroxiredoxin 3	**-1,079**	3,69E-02	104,000	95,820
GLIPR1	GLI pathogenesis related 1	**-1,079**	3,70E-02	137,257	126,298
ANXA2	annexin A2	**-1,079**	3,76E-02	999,439	919,219
HACD3	3-hydroxyacyl-CoA dehydratase 3	**-1,079**	3,94E-02	82,443	76,177
SLC25A3	solute carrier family 25 member 3	**-1,079**	4,03E-02	246,564	228,132
PPP1CA	protein phosphatase 1 catalytic subunit alpha	**-1,079**	4,09E-02	93,375	85,992
ATXN10	ataxin 10	**-1,079**	4,11E-02	86,482	80,054
LARS	leucyl-tRNA synthetase	**-1,079**	4,17E-02	119,299	110,160
CIB1	calcium and integrin binding 1	**-1,079**	4,18E-02	73,699	67,824
SEC24D	SEC24 homolog D, COPII coat complex component	**-1,079**	4,22E-02	178,388	163,979
DENR	density regulated re-initiation and release factor	**-1,079**	4,24E-02	50,610	46,549
EIF1AX	eukaryotic translation initiation factor 1A, X-linked	**-1,079**	4,35E-02	53,593	49,324
MCMBP	minichromosome maintenance complex binding protein	**-1,079**	4,44E-02	75,797	70,029
DR1	down-regulator of transcription 1	**-1,079**	4,50E-02	58,103	53,554
GFPT1	glutamine--fructose-6-phosphate transaminase 1	**-1,079**	4,67E-02	203,268	187,277
ARHGDIA	Rho GDP dissociation inhibitor alpha	**-1,079**	4,83E-02	486,277	449,317
STIP1	stress induced phosphoprotein 1	**-1,079**	4,91E-02	96,990	89,264

ANXA1	annexin A1	**-1,087**	9,72E-03	852,755	784,520
XRCC5	X-ray repair cross complementing 5	**-1,087**	1,08E-02	249,912	229,208
USP14	ubiquitin specific peptidase 14	**-1,087**	1,08E-02	95,303	87,502
SAR1B	secretion associated Ras related GTPase 1B	**-1,087**	1,22E-02	72,844	66,655
LDHB	lactate dehydrogenase B	**-1,087**	1,28E-02	140,046	128,373
EIF3B	eukaryotic translation initiation factor 3 subunit B	**-1,087**	1,40E-02	159,400	145,643
EFEMP1	EGF containing fibulin like extracellular matrix protein 1	**-1,087**	1,47E-02	427,922	394,612
UBE2K	ubiquitin conjugating enzyme E2 K	**-1,087**	1,64E-02	93,116	85,230
KPNA1	karyopherin subunit alpha 1	**-1,087**	1,64E-02	66,788	61,129
PPIF	peptidylprolyl isomerase F	**-1,087**	1,85E-02	50,972	46,473
XRCC6	X-ray repair cross complementing 6	**-1,087**	2,26E-02	253,870	232,631
CCT8	chaperonin containing TCP1 subunit 8	**-1,087**	2,35E-02	126,930	116,526
LONP1	lon peptidase 1, mitochondrial	**-1,087**	2,51E-02	84,546	77,439
SRRT	serrate, RNA effector molecule	**-1,087**	2,55E-02	83,216	76,164
WDR1	WD repeat domain 1	**-1,087**	2,57E-02	287,159	263,194
PRPF40A	pre-mRNA processing factor 40 homolog A	**-1,087**	2,62E-02	73,680	67,367
COPB1	coatomer protein complex subunit beta 1	**-1,087**	2,83E-02	193,750	177,880
PSMB5	proteasome subunit beta 5	**-1,087**	2,95E-02	98,235	90,035
G3BP1	G3BP stress granule assembly factor 1	**-1,087**	3,09E-02	139,209	126,944
DBNL	drebrin like	**-1,087**	3,24E-02	129,448	118,664
FKBP1A	FK506 binding protein 1A	**-1,087**	3,41E-02	86,118	78,882
TMX3	thioredoxin related transmembrane protein 3	**-1,087**	3,61E-02	87,161	79,544
EI24	EI24, autophagy associated transmembrane protein	**-1,087**	3,65E-02	67,724	61,982
ARPC3	actin related protein 2/3 complex subunit 3	**-1,087**	3,73E-02	97,355	89,391
MRPL42	mitochondrial ribosomal protein L42	**-1,087**	4,03E-02	38,288	34,992
MRPS16	mitochondrial ribosomal protein S16	**-1,087**	4,19E-02	53,337	48,866
MPC2	mitochondrial pyruvate carrier 2	**-1,087**	4,34E-02	46,779	42,709
RAB34	RAB34, member RAS oncogene family	**-1,087**	4,38E-02	97,703	89,308
DESI2	desumoylating isopeptidase 2	**-1,087**	4,46E-02	41,460	38,084
CCAR2	cell cycle and apoptosis regulator 2	**-1,087**	4,53E-02	56,022	51,366
GALNT18	polypeptide N-acetylgalactosaminyltransferase 18	**-1,087**	4,62E-02	102,054	92,809
UFD1	ubiquitin recognition factor in ER associated degradation 1	**-1,087**	4,67E-02	54,162	49,543
SEC23A	Sec23 homolog A, coat complex II component	**-1,087**	4,73E-02	168,916	154,599

HSPA4	heat shock protein family A (Hsp70) member 4	**-1,087**	4,74E-02	115,438	105,890
EIF5B	eukaryotic translation initiation factor 5B	**-1,087**	4,80E-02	132,995	121,553
FTSJ3	FtsJ homolog 3	**-1,087**	4,98E-02	54,256	49,494
CALM2	calmodulin 2	**-1,094**	4,21E-03	461,686	420,843
PGK1	phosphoglycerate kinase 1	**-1,094**	4,96E-03	727,542	663,017
CLIC4	chloride intracellular channel 4	**-1,094**	5,43E-03	933,748	849,693
UBE3C	ubiquitin protein ligase E3C	**-1,094**	8,04E-03	101,049	91,572
PPIA	peptidylprolyl isomerase A	**-1,094**	9,69E-03	318,363	288,989
YWHAG	tyrosine 3-monooxygenase/tryptophan 5-monooxygenase activat	**-1,094**	9,69E-03	151,521	137,804
ZNF207	zinc finger protein 207	**-1,094**	1,09E-02	91,401	83,036
IMMT	inner membrane mitochondrial protein	**-1,094**	1,12E-02	69,655	63,151
FAM177A1	family with sequence similarity 177 member A1	**-1,094**	1,15E-02	83,399	75,854
C1QBP	complement C1q binding protein	**-1,094**	1,18E-02	73,953	67,034
CBFB	core-binding factor beta subunit	**-1,094**	1,20E-02	58,483	53,200
CAVIN1	caveolae associated protein 1	**-1,094**	1,36E-02	1022,570	926,946
SRP54	signal recognition particle 54	**-1,094**	1,42E-02	78,496	71,600
ZC3H15	zinc finger CCCH-type containing 15	**-1,094**	1,45E-02	61,566	56,075
PSMA7	proteasome subunit alpha 7	**-1,094**	1,45E-02	156,785	142,186
PFKP	phosphofructokinase, platelet	**-1,094**	1,60E-02	159,341	145,644
AP2M1	adaptor related protein complex 2 mu 1 subunit	**-1,094**	1,72E-02	377,107	344,019
PITPNB	phosphatidylinositol transfer protein beta	**-1,094**	1,82E-02	64,393	58,590
MSN	moesin	**-1,094**	1,84E-02	626,830	567,086
MSH6	mutS homolog 6	**-1,094**	1,85E-02	69,603	62,882
PTPN11	protein tyrosine phosphatase, non-receptor type 11	**-1,094**	1,86E-02	135,057	122,698
HSPA8	heat shock protein family A (Hsp70) member 8	**-1,094**	1,89E-02	863,874	782,389
SLC39A1	solute carrier family 39 member 1	**-1,094**	2,25E-02	158,713	144,240
ERH	enhancer of rudimentary homolog (Drosophila)	**-1,094**	2,27E-02	91,005	82,760
WDR41	WD repeat domain 41	**-1,094**	2,43E-02	53,494	48,472
AGPS	alkylglycerone phosphate synthase	**-1,094**	2,44E-02	40,455	36,692
APPL2	adaptor protein, phosphotyrosine interacting with PH domain anc	**-1,094**	2,46E-02	77,023	70,060
ROCK2	Rho associated coiled-coil containing protein kinase 2	**-1,094**	2,56E-02	97,244	87,954
HDAC2	histone deacetylase 2	**-1,094**	2,76E-02	64,613	58,787
NCAPD2	non-SMC condensin I complex subunit D2	**-1,094**	2,87E-02	61,691	55,756

SMS	spermine synthase	**-1,094**	2,93E-02	45,780	41,585
PSMC2	proteasome 26S subunit, ATPase 2	**-1,094**	2,96E-02	114,002	103,915
PHF19	PHD finger protein 19	**-1,094**	3,09E-02	70,045	63,568
GRPEL1	GrpE like 1, mitochondrial	**-1,094**	3,13E-02	35,889	32,572
NUP43	nucleoporin 43	**-1,094**	3,13E-02	32,450	29,376
NUTF2	nuclear transport factor 2	**-1,094**	3,13E-02	53,566	48,477
GTF2A2	general transcription factor IIA subunit 2	**-1,094**	3,16E-02	39,902	36,427
LMF2	lipase maturation factor 2	**-1,094**	3,28E-02	112,453	102,409
PSMC6	proteasome 26S subunit, ATPase 6	**-1,094**	3,36E-02	52,814	48,018
COL1A2	collagen type I alpha 2 chain	**-1,094**	3,55E-02	634,543	579,851
TAOK2	TAO kinase 2	**-1,094**	3,58E-02	72,708	65,850
PDCD10	programmed cell death 10	**-1,094**	3,68E-02	34,155	31,106
NUP88	nucleoporin 88	**-1,094**	3,82E-02	35,131	31,826
BCCIP	BRCA2 and CDKN1A interacting protein	**-1,094**	3,95E-02	49,649	44,943
PHPT1	phosphohistidine phosphatase 1	**-1,094**	4,06E-02	43,910	39,713
CKAP4	cytoskeleton associated protein 4	**-1,094**	4,09E-02	396,563	359,081
C8orf33	chromosome 8 open reading frame 33	**-1,094**	4,10E-02	49,018	44,663
FZD1	frizzled class receptor 1	**-1,094**	4,11E-02	52,659	47,494
IMPDH2	inosine monophosphate dehydrogenase 2	**-1,094**	4,16E-02	113,211	102,668
ST6GALNAC4	ST6 N-acetylgalactosaminide alpha-2,6-sialyltransferase 4	**-1,094**	4,17E-02	73,403	66,681
TWISTNB	TWIST neighbor	**-1,094**	4,46E-02	47,167	42,550
RHOD	ras homolog family member D	**-1,094**	4,63E-02	40,234	36,295
CFAP20	cilia and flagella associated protein 20	**-1,094**	4,69E-02	30,441	27,559
PDHB	pyruvate dehydrogenase (lipoamide) beta	**-1,094**	4,91E-02	44,804	40,611
SRP9	signal recognition particle 9	**-1,102**	3,14E-03	95,165	85,956
PSMB2	proteasome subunit beta 2	**-1,102**	4,10E-03	79,615	71,906
ACSL4	acyl-CoA synthetase long-chain family member 4	**-1,102**	4,82E-03	94,050	85,277
EIF4G2	eukaryotic translation initiation factor 4 gamma 2	**-1,102**	6,45E-03	1062,329	957,326
PPA1	pyrophosphatase (inorganic) 1	**-1,102**	6,71E-03	86,455	78,191
POMP	proteasome maturation protein	**-1,102**	7,29E-03	67,952	61,336
HNRNPA3	heterogeneous nuclear ribonucleoprotein A3	**-1,102**	7,70E-03	186,657	168,352
TARS	threonyl-tRNA synthetase	**-1,102**	8,88E-03	118,647	107,237
PA2G4	proliferation-associated 2G4	**-1,102**	9,45E-03	72,410	65,014

PHB	prohibitin	**-1,102**	9,75E-03	91,718	82,418
LMNA	lamin A/C	**-1,102**	1,24E-02	752,549	675,402
DPY19L1	dpy-19 like 1	**-1,102**	1,35E-02	80,100	72,306
PSMD13	proteasome 26S subunit, non-ATPase 13	**-1,102**	1,38E-02	112,218	101,097
POLDIP2	DNA polymerase delta interacting protein 2	**-1,102**	1,45E-02	81,846	73,869
SLC25A32	solute carrier family 25 member 32	**-1,102**	1,58E-02	40,337	36,216
LAPTM4B	lysosomal protein transmembrane 4 beta	**-1,102**	1,59E-02	70,930	63,714
CBX5	chromobox 5	**-1,102**	1,59E-02	271,400	244,648
DLD	dihydrolipoamide dehydrogenase	**-1,102**	1,60E-02	57,408	51,816
C1GALT1	core 1 synthase, glycoprotein-N-acetylgalactosamine 3-beta-galac	**-1,102**	1,60E-02	57,477	52,056
AP1B1	adaptor related protein complex 1 beta 1 subunit	**-1,102**	1,70E-02	75,772	68,482
SLC9A6	solute carrier family 9 member A6	**-1,102**	1,70E-02	45,701	41,240
SSRP1	structure specific recognition protein 1	**-1,102**	1,74E-02	118,595	106,602
TMED2	transmembrane p24 trafficking protein 2	**-1,102**	1,76E-02	292,496	263,533
PTTG1IP	PTTG1 interacting protein	**-1,102**	1,77E-02	1766,775	1588,086
ETF1	eukaryotic translation termination factor 1	**-1,102**	1,85E-02	111,893	101,147
NUP62	nucleoporin 62	**-1,102**	1,85E-02	50,202	45,169
PSMC5	proteasome 26S subunit, ATPase 5	**-1,102**	2,00E-02	135,646	122,493
BANF1	barrier to autointegration factor 1	**-1,102**	2,06E-02	83,410	74,875
ETFA	electron transfer flavoprotein alpha subunit	**-1,102**	2,08E-02	55,829	50,290
TPST1	tyrosylprotein sulfotransferase 1	**-1,102**	2,10E-02	45,351	40,858
AKT1	AKT serine/threonine kinase 1	**-1,102**	2,16E-02	177,965	161,130
FKBP3	FK506 binding protein 3	**-1,102**	2,18E-02	43,415	39,102
SLC25A39	solute carrier family 25 member 39	**-1,102**	2,18E-02	64,437	57,849
ZDHHC9	zinc finger DHHC-type containing 9	**-1,102**	2,42E-02	57,523	52,039
USP5	ubiquitin specific peptidase 5	**-1,102**	2,49E-02	106,761	96,256
UBL7	ubiquitin like 7	**-1,102**	2,66E-02	26,914	24,265
CDC42EP3	CDC42 effector protein 3	**-1,102**	2,72E-02	142,283	128,033
POLR2L	RNA polymerase II subunit L	**-1,102**	2,75E-02	109,726	98,456
FEZ2	fasciculation and elongation protein zeta 2	**-1,102**	2,79E-02	40,964	37,023
CS	citrate synthase	**-1,102**	2,89E-02	60,208	54,055
ELOC	elongin C	**-1,102**	2,91E-02	47,850	43,108
SYNJ2	synaptojanin 2	**-1,102**	2,92E-02	60,478	54,294

ATP2A2	ATPase sarcoplasmic/endoplasmic reticulum Ca2+ transporting 2	**-1,102**	2,93E-02	273,538	246,481
ZNF217	zinc finger protein 217	**-1,102**	3,09E-02	27,299	24,591
ADCY6	adenylate cyclase 6	**-1,102**	3,15E-02	36,331	32,638
WDR5	WD repeat domain 5	**-1,102**	3,23E-02	38,041	34,083
COPS4	COP9 signalosome subunit 4	**-1,102**	3,36E-02	45,903	41,445
CUL2	cullin 2	**-1,102**	3,54E-02	30,906	27,937
SNAI1	snail family transcriptional repressor 1	**-1,102**	3,54E-02	31,899	28,620
SLC7A1	solute carrier family 7 member 1	**-1,102**	3,65E-02	116,396	104,802
CHD3	chromodomain helicase DNA binding protein 3	**-1,102**	3,66E-02	62,274	55,888
PIAS3	protein inhibitor of activated STAT 3	**-1,102**	3,73E-02	40,737	36,698
CPSF2	cleavage and polyadenylation specific factor 2	**-1,102**	3,73E-02	54,428	49,054
MARS	methionyl-tRNA synthetase	**-1,102**	3,76E-02	152,301	136,967
DNAJB4	DnaJ heat shock protein family (Hsp40) member B4	**-1,102**	4,03E-02	29,831	26,824
CAPN15	calpain 15	**-1,102**	4,22E-02	37,403	33,526
INHBA	inhibin beta A subunit	**-1,102**	4,27E-02	703,337	635,593
UBA6	ubiquitin like modifier activating enzyme 6	**-1,102**	4,55E-02	36,531	33,023
GOSR2	golgi SNAP receptor complex member 2	**-1,102**	4,59E-02	41,057	36,909
PTDSS1	phosphatidylserine synthase 1	**-1,102**	4,81E-02	60,137	54,152
LSG1	large 60S subunit nuclear export GTPase 1	**-1,102**	5,00E-02	31,838	28,737
ACTR2	ARP2 actin related protein 2 homolog	**-1,110**	2,26E-03	218,371	195,268
SET	SET nuclear proto-oncogene	**-1,110**	2,65E-03	233,973	208,639
SMAD6	SMAD family member 6	**-1,110**	3,63E-03	103,482	92,694
S100A10	S100 calcium binding protein A10	**-1,110**	3,83E-03	298,629	266,702
CCT7	chaperonin containing TCP1 subunit 7	**-1,110**	3,95E-03	192,703	173,179
SYNCRIP	synaptotagmin binding cytoplasmic RNA interacting protein	**-1,110**	4,28E-03	144,857	129,292
PSMD2	proteasome 26S subunit, non-ATPase 2	**-1,110**	4,36E-03	205,053	183,979
KIF5B	kinesin family member 5B	**-1,110**	5,64E-03	177,098	158,005
NSUN2	NOP2/Sun RNA methyltransferase family member 2	**-1,110**	5,79E-03	62,910	56,275
MCFD2	multiple coagulation factor deficiency 2	**-1,110**	7,54E-03	293,823	262,399
CETN2	centrin 2	**-1,110**	8,02E-03	59,781	53,713
KPNA4	karyopherin subunit alpha 4	**-1,110**	8,14E-03	63,640	56,863
CALU	calumenin	**-1,110**	8,31E-03	763,391	684,679
PSMD1	proteasome 26S subunit, non-ATPase 1	**-1,110**	9,15E-03	139,100	125,041

GPC1	glypican 1	**-1,110**	1,12E-02	294,819	267,272
ZNF598	zinc finger protein 598	**-1,110**	1,12E-02	29,115	25,947
NCL	nucleolin	**-1,110**	1,19E-02	465,388	415,185
RSPRY1	ring finger and SPRY domain containing 1	**-1,110**	1,22E-02	54,063	48,511
ALDH9A1	aldehyde dehydrogenase 9 family member A1	**-1,110**	1,28E-02	49,242	44,150
PWP1	PWP1 homolog, endonuclein	**-1,110**	1,31E-02	51,933	46,566
PSMD12	proteasome 26S subunit, non-ATPase 12	**-1,110**	1,36E-02	59,069	52,796
GARS	glycyl-tRNA synthetase	**-1,110**	1,37E-02	218,618	195,898
SLC38A2	solute carrier family 38 member 2	**-1,110**	1,40E-02	576,399	519,182
PDCD5	programmed cell death 5	**-1,110**	1,41E-02	51,139	45,777
COPE	coatomer protein complex subunit epsilon	**-1,110**	1,45E-02	67,107	60,396
UBE2V2	ubiquitin conjugating enzyme E2 V2	**-1,110**	1,49E-02	48,599	43,618
PDK1	pyruvate dehydrogenase kinase 1	**-1,110**	1,51E-02	35,380	31,666
RBM8A	RNA binding motif protein 8A	**-1,110**	1,53E-02	69,605	62,504
FAM98A	family with sequence similarity 98 member A	**-1,110**	1,58E-02	75,598	67,918
SLC1A5	solute carrier family 1 member 5	**-1,110**	1,87E-02	362,629	325,484
PSMA3	proteasome subunit alpha 3	**-1,110**	1,96E-02	47,438	42,614
NUP107	nucleoporin 107	**-1,110**	2,01E-02	29,113	25,921
SUZ12	SUZ12 polycomb repressive complex 2 subunit	**-1,110**	2,06E-02	28,411	25,485
SLC9A3R2	SLC9A3 regulator 2	**-1,110**	2,11E-02	167,892	150,404
EIF2S1	eukaryotic translation initiation factor 2 subunit alpha	**-1,110**	2,24E-02	88,393	79,204
SRPRB	SRP receptor beta subunit	**-1,110**	2,33E-02	96,794	86,776
PSMB6	proteasome subunit beta 6	**-1,110**	2,47E-02	84,399	75,815
WDR61	WD repeat domain 61	**-1,110**	2,64E-02	51,882	46,598
PEA15	phosphoprotein enriched in astrocytes 15	**-1,110**	2,69E-02	170,049	152,158
ERBIN	erbb2 interacting protein	**-1,110**	2,84E-02	47,776	42,798
HERC3	HECT and RLD domain containing E3 ubiquitin protein ligase 3	**-1,110**	2,87E-02	39,431	35,370
COMMD4	COMM domain containing 4	**-1,110**	3,05E-02	30,486	27,338
PRMT1	protein arginine methyltransferase 1	**-1,110**	3,11E-02	77,519	69,549
NHP2	NHP2 ribonucleoprotein	**-1,110**	3,17E-02	56,791	50,755
COMMD2	COMM domain containing 2	**-1,110**	3,19E-02	31,405	28,159
TWF1	twinfilin actin binding protein 1	**-1,110**	3,35E-02	38,191	34,086
SLC35A2	solute carrier family 35 member A2	**-1,110**	3,47E-02	40,686	36,371

RUVBL2	RuvB like AAA ATPase 2	**-1,110**	3,51E-02	62,937	56,125
TTPAL	alpha tocopherol transfer protein like	**-1,110**	3,60E-02	25,318	22,638
FIBP	FGF1 intracellular binding protein	**-1,110**	3,73E-02	50,262	45,070
RTCA	RNA 3'-terminal phosphate cyclase	**-1,110**	3,82E-02	35,126	31,327
HAUS2	HAUS augmin like complex subunit 2	**-1,110**	3,97E-02	24,632	21,911
PTGER2	prostaglandin E receptor 2	**-1,110**	4,01E-02	27,878	24,823
SPRY4	sprouty RTK signaling antagonist 4	**-1,110**	4,05E-02	37,703	33,506
GDF10	growth differentiation factor 10	**-1,110**	4,07E-02	84,115	74,189
TIMM50	translocase of inner mitochondrial membrane 50	**-1,110**	4,23E-02	30,772	27,520
ENG	endoglin	**-1,110**	4,30E-02	649,597	585,974
TMEM208	transmembrane protein 208	**-1,110**	4,67E-02	26,810	23,839
AIFM2	apoptosis inducing factor, mitochondria associated 2	**-1,110**	4,74E-02	24,086	21,525
PRPF4	pre-mRNA processing factor 4	**-1,110**	4,84E-02	34,574	30,833
TATDN2	TatD DNase domain containing 2	**-1,110**	4,88E-02	17,240	15,368
DEK	DEK proto-oncogene	**-1,117**	1,29E-03	155,590	138,454
SF3B3	splicing factor 3b subunit 3	**-1,117**	1,92E-03	129,258	115,216
DEGS1	delta 4-desaturase, sphingolipid 1	**-1,117**	2,75E-03	105,169	93,614
LDHA	lactate dehydrogenase A	**-1,117**	2,99E-03	1091,257	975,634
ARF6	ADP ribosylation factor 6	**-1,117**	3,39E-03	110,326	97,616
DSTN	destrin, actin depolymerizing factor	**-1,117**	3,50E-03	608,446	542,424
CNN3	calponin 3	**-1,117**	4,34E-03	223,118	198,100
EMP3	epithelial membrane protein 3	**-1,117**	4,97E-03	223,640	198,430
HNRNPM	heterogeneous nuclear ribonucleoprotein M	**-1,117**	5,25E-03	249,607	220,354
ARHGAP29	Rho GTPase activating protein 29	**-1,117**	5,89E-03	47,934	42,778
HIGD1A	HIG1 hypoxia inducible domain family member 1A	**-1,117**	6,08E-03	32,605	28,881
PPP2CA	protein phosphatase 2 catalytic subunit alpha	**-1,117**	6,14E-03	98,364	87,624
STRAP	serine/threonine kinase receptor associated protein	**-1,117**	6,26E-03	117,399	104,486
RARS	arginyl-tRNA synthetase	**-1,117**	7,89E-03	80,130	70,980
SMAD3	SMAD family member 3	**-1,117**	8,02E-03	102,840	91,465
HNRNPD	heterogeneous nuclear ribonucleoprotein D	**-1,117**	8,08E-03	175,225	155,671
NSD2	nuclear receptor binding SET domain protein 2	**-1,117**	8,14E-03	65,708	58,040
C5orf15	chromosome 5 open reading frame 15	**-1,117**	9,51E-03	128,229	114,150
THBS1	thrombospondin 1	**-1,117**	9,64E-03	1421,405	1271,807

MYL6	myosin light chain 6	**-1,117**	9,80E-03	458,027	406,698
CCT2	chaperonin containing TCP1 subunit 2	**-1,117**	9,85E-03	83,965	74,083
LYPLA1	lysophospholipase I	**-1,117**	1,09E-02	31,218	27,786
IKBIP	IKBKB interacting protein	**-1,117**	1,11E-02	79,863	70,965
DAZAP1	DAZ associated protein 1	**-1,117**	1,12E-02	86,421	76,652
SMAD7	SMAD family member 7	**-1,117**	1,12E-02	45,238	40,338
SEC61G	Sec61 translocon gamma subunit	**-1,117**	1,24E-02	87,001	77,226
DOCK5	dedicator of cytokinesis 5	**-1,117**	1,28E-02	76,658	68,260
IARS	isoleucyl-tRNA synthetase	**-1,117**	1,48E-02	221,940	198,280
AP2S1	adaptor related protein complex 2 sigma 1 subunit	**-1,117**	1,55E-02	70,793	62,906
MIDN	midnolin	**-1,117**	1,56E-02	69,661	62,325
SEC24A	SEC24 homolog A, COPII coat complex component	**-1,117**	1,57E-02	65,776	58,240
NAT10	N-acetyltransferase 10	**-1,117**	1,69E-02	43,461	38,441
TRA2A	transformer 2 alpha homolog	**-1,117**	1,75E-02	41,828	37,040
HM13	histocompatibility minor 13	**-1,117**	1,88E-02	187,275	166,707
ZNF503	zinc finger protein 503	**-1,117**	1,95E-02	56,215	49,619
IDH3A	isocitrate dehydrogenase 3 (NAD(+)) alpha	**-1,117**	2,00E-02	38,204	34,000
TSR1	TSR1, ribosome maturation factor	**-1,117**	2,00E-02	51,734	45,804
GTF2H3	general transcription factor IIH subunit 3	**-1,117**	2,11E-02	28,170	24,964
EIF6	eukaryotic translation initiation factor 6	**-1,117**	2,19E-02	58,771	51,918
NACC1	nucleus accumbens associated 1	**-1,117**	2,25E-02	47,031	41,935
FTSJ1	FtsJ RNA methyltransferase homolog 1 (E. coli)	**-1,117**	2,26E-02	24,847	22,105
CDK5RAP2	CDK5 regulatory subunit associated protein 2	**-1,117**	2,42E-02	66,915	59,200
LRRC42	leucine rich repeat containing 42	**-1,117**	2,53E-02	25,117	22,253
PRMT5	protein arginine methyltransferase 5	**-1,117**	2,53E-02	64,328	56,748
CNOT10	CCR4-NOT transcription complex subunit 10	**-1,117**	2,53E-02	21,944	19,372
TNFRSF10D	TNF receptor superfamily member 10d	**-1,117**	2,69E-02	111,404	99,461
EIF3J	eukaryotic translation initiation factor 3 subunit J	**-1,117**	2,76E-02	62,547	55,385
SLC35B4	solute carrier family 35 member B4	**-1,117**	2,79E-02	37,116	32,977
PPA2	pyrophosphatase (inorganic) 2	**-1,117**	2,83E-02	27,199	24,200
TP53I13	tumor protein p53 inducible protein 13	**-1,117**	2,92E-02	64,497	57,415
CPSF3	cleavage and polyadenylation specific factor 3	**-1,117**	2,99E-02	23,627	20,987
UTP6	UTP6, small subunit processome component	**-1,117**	3,03E-02	39,511	35,007

CHCHD7	coiled-coil-helix-coiled-coil-helix domain containing 7	**-1,117**	3,03E-02	20,467	18,183
DDX56	DEAD-box helicase 56	**-1,117**	3,09E-02	41,525	36,840
PLCB3	phospholipase C beta 3	**-1,117**	3,29E-02	26,538	23,343
MPRIP	myosin phosphatase Rho interacting protein	**-1,117**	3,34E-02	181,480	160,849
SLC25A17	solute carrier family 25 member 17	**-1,117**	3,52E-02	19,833	17,644
ABI3BP	ABI family member 3 binding protein	**-1,117**	3,54E-02	376,292	327,700
SPRYD7	SPRY domain containing 7	**-1,117**	3,58E-02	23,694	20,911
CPT1A	carnitine palmitoyltransferase 1A	**-1,117**	3,60E-02	49,361	43,534
PSAT1	phosphoserine aminotransferase 1	**-1,117**	3,68E-02	92,940	81,928
UQCC2	ubiquinol-cytochrome c reductase complex assembly factor 2	**-1,117**	3,77E-02	27,991	24,820
SIGMAR1	sigma non-opioid intracellular receptor 1	**-1,117**	3,87E-02	57,307	50,403
SULF1	sulfatase 1	**-1,117**	3,94E-02	724,095	649,548
MANF	mesencephalic astrocyte derived neurotrophic factor	**-1,117**	4,35E-02	75,564	66,465
DAAM2	dishevelled associated activator of morphogenesis 2	**-1,117**	4,37E-02	58,369	51,708
GPX8	glutathione peroxidase 8 (putative)	**-1,117**	4,38E-02	114,663	102,015
IMPDH1	inosine monophosphate dehydrogenase 1	**-1,117**	4,68E-02	42,860	37,727
NASP	nuclear autoantigenic sperm protein	**-1,117**	4,83E-02	39,616	34,988
USP1	ubiquitin specific peptidase 1	**-1,125**	8,65E-04	71,341	63,064
SRSF1	serine and arginine rich splicing factor 1	**-1,125**	9,93E-04	151,302	133,139
ENO1	enolase 1	**-1,125**	1,38E-03	2826,540	2508,706
TMEM165	transmembrane protein 165	**-1,125**	1,64E-03	106,915	94,855
EIF4A3	eukaryotic translation initiation factor 4A3	**-1,125**	1,71E-03	68,259	60,189
CFL1	cofilin 1	**-1,125**	2,50E-03	427,725	376,058
CYC1	cytochrome c1	**-1,125**	2,65E-03	72,702	63,939
IRAK1	interleukin 1 receptor associated kinase 1	**-1,125**	2,67E-03	163,196	143,779
KDELR2	KDEL endoplasmic reticulum protein retention receptor 2	**-1,125**	2,77E-03	304,316	269,265
PSMA4	proteasome subunit alpha 4	**-1,125**	3,25E-03	76,360	67,081
PSMD11	proteasome 26S subunit, non-ATPase 11	**-1,125**	3,44E-03	85,340	75,424
GCLM	glutamate-cysteine ligase modifier subunit	**-1,125**	3,63E-03	61,047	54,133
PSME3	proteasome activator subunit 3	**-1,125**	3,94E-03	96,589	84,841
PSMC3	proteasome 26S subunit, ATPase 3	**-1,125**	3,95E-03	164,806	145,867
PDXK	pyridoxal kinase	**-1,125**	4,10E-03	85,822	76,025
PRPS1	phosphoribosyl pyrophosphate synthetase 1	**-1,125**	5,14E-03	54,913	48,301

AURKAIP1	aurora kinase A interacting protein 1	**-1,125**	6,11E-03	67,315	59,475
MRPL24	mitochondrial ribosomal protein L24	**-1,125**	6,15E-03	50,386	44,153
CCT3	chaperonin containing TCP1 subunit 3	**-1,125**	6,58E-03	166,209	146,918
HMGB2	high mobility group box 2	**-1,125**	6,89E-03	119,178	103,948
LSM4	LSM4 homolog, U6 small nuclear RNA and mRNA degradation ass	**-1,125**	8,02E-03	60,229	53,108
GFM1	G elongation factor mitochondrial 1	**-1,125**	8,13E-03	42,169	37,061
SEH1L	SEH1 like nucleoporin	**-1,125**	8,13E-03	40,551	35,689
DCAF13	DDB1 and CUL4 associated factor 13	**-1,125**	8,73E-03	41,307	36,263
NOP14	NOP14 nucleolar protein	**-1,125**	9,11E-03	38,230	33,638
ODC1	ornithine decarboxylase 1	**-1,125**	9,61E-03	200,016	175,730
UBE2N	ubiquitin conjugating enzyme E2 N	**-1,125**	9,62E-03	62,252	54,910
MSH2	mutS homolog 2	**-1,125**	9,68E-03	40,627	35,622
CCND3	cyclin D3	**-1,125**	9,69E-03	70,662	62,130
NEMP1	nuclear envelope integral membrane protein 1	**-1,125**	1,01E-02	26,242	23,089
SOCS5	suppressor of cytokine signaling 5	**-1,125**	1,06E-02	73,915	65,054
NRAS	NRAS proto-oncogene, GTPase	**-1,125**	1,07E-02	54,910	48,535
FUS	FUS RNA binding protein	**-1,125**	1,07E-02	298,370	260,994
EIF4G1	eukaryotic translation initiation factor 4 gamma 1	**-1,125**	1,18E-02	405,713	357,946
SLC31A1	solute carrier family 31 member 1	**-1,125**	1,45E-02	66,011	58,040
SEC13	SEC13 homolog, nuclear pore and COPII coat complex component	**-1,125**	1,47E-02	117,281	103,668
PANK3	pantothenate kinase 3	**-1,125**	1,51E-02	40,501	35,536
NUP93	nucleoporin 93	**-1,125**	1,56E-02	24,182	21,244
SGMS2	sphingomyelin synthase 2	**-1,125**	1,61E-02	84,691	74,520
COA4	cytochrome c oxidase assembly factor 4 homolog	**-1,125**	1,63E-02	30,957	27,324
TIMM17B	translocase of inner mitochondrial membrane 17B	**-1,125**	1,71E-02	21,744	19,147
DHX29	DExH-box helicase 29	**-1,125**	1,76E-02	37,979	33,521
YIF1B	Yip1 interacting factor homolog B, membrane trafficking protein	**-1,125**	1,84E-02	80,155	70,944
PPM1G	protein phosphatase, Mg2+/Mn2+ dependent 1G	**-1,125**	1,84E-02	72,951	63,838
NF2	neurofibromin 2	**-1,125**	1,84E-02	38,106	33,371
SFXN1	sideroflexin 1	**-1,125**	1,94E-02	34,732	30,763
IMP4	IMP4 homolog, U3 small nucleolar ribonucleoprotein	**-1,125**	2,08E-02	47,533	41,785
ARMC5	armadillo repeat containing 5	**-1,125**	2,25E-02	30,590	26,982
MPDU1	mannose-P-dolichol utilization defect 1	**-1,125**	2,38E-02	40,463	35,567

GRWD1	glutamate rich WD repeat containing 1	**-1,125**	2,38E-02	33,309	29,166
COPZ2	coatomer protein complex subunit zeta 2	**-1,125**	2,43E-02	85,543	74,526
LAP3	leucine aminopeptidase 3	**-1,125**	2,44E-02	41,219	36,550
TAF13	TATA-box binding protein associated factor 13	**-1,125**	2,56E-02	27,446	23,938
GPT2	glutamic--pyruvic transaminase 2	**-1,125**	2,60E-02	59,803	52,336
MDFI	MyoD family inhibitor	**-1,125**	2,62E-02	97,399	85,978
ASCC3	activating signal cointegrator 1 complex subunit 3	**-1,125**	2,85E-02	53,090	46,805
RWDD2B	RWD domain containing 2B	**-1,125**	3,09E-02	17,631	15,511
MAGOH	mago homolog, exon junction complex core component	**-1,125**	3,09E-02	22,888	20,145
POLR1C	RNA polymerase I subunit C	**-1,125**	3,66E-02	18,801	16,535
KRT10	keratin 10	**-1,125**	3,89E-02	45,703	40,410
NPR2	natriuretic peptide receptor 2	**-1,125**	3,92E-02	152,412	134,935
NUDT15	nudix hydrolase 15	**-1,125**	3,95E-02	16,319	14,421
CNN2	calponin 2	**-1,125**	4,18E-02	45,669	40,704
MTMR1	myotubularin related protein 1	**-1,125**	4,49E-02	15,373	13,440
FARSA	phenylalanyl-tRNA synthetase alpha subunit	**-1,125**	4,67E-02	46,139	40,250
LMCD1	LIM and cysteine rich domains 1	**-1,125**	4,67E-02	19,404	17,185
CYB561	cytochrome b561	**-1,125**	5,00E-02	25,715	22,492
CAPZA1	capping actin protein of muscle Z-line alpha subunit 1	**-1,133**	1,66E-04	137,481	120,632
HNRNPA2B1	heterogeneous nuclear ribonucleoprotein A2/B1	**-1,133**	6,69E-04	642,112	561,985
JPT2	Jupiter microtubule associated homolog 2	**-1,133**	1,23E-03	76,344	67,229
TPM3	tropomyosin 3	**-1,133**	1,25E-03	279,201	242,515
TPI1	triosephosphate isomerase 1	**-1,133**	1,30E-03	415,924	365,019
RTN4	reticulon 4	**-1,133**	1,39E-03	799,290	701,927
SNRPB	small nuclear ribonucleoprotein polypeptides B and B1	**-1,133**	1,39E-03	62,330	54,530
TKT	transketolase	**-1,133**	1,81E-03	138,980	123,004
MTHFD2	methylenetetrahydrofolate dehydrogenase (NADP+ dependent) 2	**-1,133**	1,94E-03	185,423	161,855
RHOA	ras homolog family member A	**-1,133**	2,26E-03	543,458	476,033
TIMM17A	translocase of inner mitochondrial membrane 17A	**-1,133**	2,35E-03	58,641	51,149
ARF4	ADP ribosylation factor 4	**-1,133**	3,88E-03	248,087	216,673
TXNDC9	thioredoxin domain containing 9	**-1,133**	4,31E-03	34,008	29,766
MAP7D1	MAP7 domain containing 1	**-1,133**	4,49E-03	92,525	81,093
CSK	CSK, non-receptor tyrosine kinase	**-1,133**	4,87E-03	55,101	48,194

MLH1	mutL homolog 1	**-1,133**	5,45E-03	53,555	46,789
OGFOD1	2-oxoglutarate and iron dependent oxygenase domain containing	**-1,133**	6,24E-03	41,436	36,101
YKT6	YKT6 v-SNARE homolog	**-1,133**	6,64E-03	133,858	116,966
NCAPH2	non-SMC condensin II complex subunit H2	**-1,133**	7,61E-03	42,236	36,902
DLAT	dihydrolipoamide S-acetyltransferase	**-1,133**	8,02E-03	39,247	34,296
KIF22	kinesin family member 22	**-1,133**	8,02E-03	33,491	29,148
PDCD6	programmed cell death 6	**-1,133**	9,08E-03	76,488	66,796
GOLT1B	golgi transport 1B	**-1,133**	9,11E-03	40,875	35,846
GLRX3	glutaredoxin 3	**-1,133**	9,39E-03	45,563	39,871
MRPS35	mitochondrial ribosomal protein S35	**-1,133**	9,80E-03	28,867	25,339
PFDN2	prefoldin subunit 2	**-1,133**	1,09E-02	28,527	24,971
MRPS15	mitochondrial ribosomal protein S15	**-1,133**	1,11E-02	30,168	26,422
TMSB4X	thymosin beta 4, X-linked	**-1,133**	1,15E-02	87,757	75,181
ZW10	zw10 kinetochore protein	**-1,133**	1,15E-02	22,581	19,689
HSD17B12	hydroxysteroid 17-beta dehydrogenase 12	**-1,133**	1,16E-02	63,981	56,332
NDC1	NDC1 transmembrane nucleoporin	**-1,133**	1,30E-02	28,419	24,864
SUGP2	SURP and G-patch domain containing 2	**-1,133**	1,36E-02	47,454	41,390
VCL	vinculin	**-1,133**	1,36E-02	157,198	136,788
UTP11	UTP11, small subunit processome component homolog (S. cerevis	**-1,133**	1,41E-02	22,909	20,039
PLPPR2	phospholipid phosphatase related 2	**-1,133**	1,79E-02	46,337	40,578
GSS	glutathione synthetase	**-1,133**	1,79E-02	38,274	33,481
DKC1	dyskerin pseudouridine synthase 1	**-1,133**	1,88E-02	55,127	47,993
PITPNM1	phosphatidylinositol transfer protein membrane associated 1	**-1,133**	2,00E-02	50,448	43,918
CNEP1R1	CTD nuclear envelope phosphatase 1 regulatory subunit 1	**-1,133**	2,01E-02	19,363	16,895
ITPA	inosine triphosphatase	**-1,133**	2,08E-02	20,020	17,407
HYAL2	hyaluronoglucosaminidase 2	**-1,133**	2,14E-02	33,700	29,473
ELP6	elongator acetyltransferase complex subunit 6	**-1,133**	2,17E-02	20,852	18,208
TRAF4	TNF receptor associated factor 4	**-1,133**	2,25E-02	29,824	26,028
HPF1	histone PARylation factor 1	**-1,133**	2,26E-02	18,659	16,336
CYB5B	cytochrome b5 type B	**-1,133**	2,29E-02	73,033	63,574
NOLC1	nucleolar and coiled-body phosphoprotein 1	**-1,133**	2,35E-02	61,196	53,476
MYOF	myoferlin	**-1,133**	2,38E-02	274,131	238,398
MRPL22	mitochondrial ribosomal protein L22	**-1,133**	2,46E-02	18,147	15,818

PGAM5	PGAM family member 5, mitochondrial serine/threonine protein	**-1,133**	2,48E-02	19,303	16,761
DCTPP1	dCTP pyrophosphatase 1	**-1,133**	2,55E-02	25,494	22,063
C4orf48	chromosome 4 open reading frame 48	**-1,133**	2,63E-02	20,202	17,698
WDR12	WD repeat domain 12	**-1,133**	2,69E-02	21,073	18,341
DOHH	deoxyhypusine hydroxylase	**-1,133**	2,76E-02	18,449	16,091
DESI1	desumoylating isopeptidase 1	**-1,133**	2,93E-02	34,021	29,695
CENPX	centromere protein X	**-1,133**	2,97E-02	25,147	21,784
AXIN2	axin 2	**-1,133**	2,98E-02	22,689	19,682
FAM126A	family with sequence similarity 126 member A	**-1,133**	2,98E-02	23,017	19,980
TPCN2	two pore segment channel 2	**-1,133**	3,00E-02	24,998	21,903
BDKRB2	bradykinin receptor B2	**-1,133**	3,09E-02	16,305	14,185
OPA3	OPA3, outer mitochondrial membrane lipid metabolism regulator	**-1,133**	3,11E-02	22,805	19,830
ZNF593	zinc finger protein 593	**-1,133**	3,20E-02	13,534	11,714
PALMD	palmdelphin	**-1,133**	3,22E-02	29,945	25,525
NUP160	nucleoporin 160	**-1,133**	3,25E-02	16,696	14,571
THOC6	THO complex 6	**-1,133**	3,25E-02	16,288	14,075
PLA2G4A	phospholipase A2 group IVA	**-1,133**	3,31E-02	98,148	86,130
MFSD12	major facilitator superfamily domain containing 12	**-1,133**	3,42E-02	27,926	24,251
ATP13A2	ATPase 13A2	**-1,133**	3,87E-02	67,974	59,063
QTRT2	queuine tRNA-ribosyltransferase accessory subunit 2	**-1,133**	3,91E-02	15,258	13,227
RUVBL1	RuvB like AAA ATPase 1	**-1,133**	3,96E-02	30,543	26,416
NDUFS3	NADH:ubiquinone oxidoreductase core subunit S3	**-1,133**	4,17E-02	14,912	13,001
BIRC2	baculoviral IAP repeat containing 2	**-1,141**	1,08E-04	98,583	85,889
ACSL3	acyl-CoA synthetase long-chain family member 3	**-1,141**	3,63E-04	108,594	94,580
TXNL4A	thioredoxin like 4A	**-1,141**	1,71E-03	54,658	47,520
SFPQ	splicing factor proline and glutamine rich	**-1,141**	1,71E-03	180,032	155,769
PRELID1	PRELI domain containing 1	**-1,141**	1,71E-03	82,308	71,823
PITX1	paired like homeodomain 1	**-1,141**	2,07E-03	151,496	133,250
MAPRE1	microtubule associated protein RP/EB family member 1	**-1,141**	2,22E-03	101,571	88,044
MAEA	macrophage erythroblast attacher	**-1,141**	2,26E-03	68,548	59,655
ICMT	isoprenylcysteine carboxyl methyltransferase	**-1,141**	2,61E-03	124,736	107,687
VBP1	VHL binding protein 1	**-1,141**	2,84E-03	35,038	30,316
DDX39A	DExD-box helicase 39A	**-1,141**	3,26E-03	59,774	51,699

HAT1	histone acetyltransferase 1	**-1,141**	3,57E-03	51,538	44,620
SNRPA	small nuclear ribonucleoprotein polypeptide A	**-1,141**	3,63E-03	40,897	35,331
ILF2	interleukin enhancer binding factor 2	**-1,141**	4,23E-03	120,932	104,416
TOPBP1	topoisomerase (DNA) II binding protein 1	**-1,141**	4,61E-03	29,662	25,731
H2AFY	H2A histone family member Y	**-1,141**	5,13E-03	68,735	59,507
FH	fumarate hydratase	**-1,141**	5,18E-03	43,331	37,688
PSMA5	proteasome subunit alpha 5	**-1,141**	5,28E-03	69,642	60,492
TPM2	tropomyosin 2 (beta)	**-1,141**	6,37E-03	131,024	113,344
ABCD3	ATP binding cassette subfamily D member 3	**-1,141**	6,57E-03	40,120	34,920
EFHD2	EF-hand domain family member D2	**-1,141**	7,33E-03	25,368	21,894
MRPL47	mitochondrial ribosomal protein L47	**-1,141**	8,04E-03	25,075	21,719
STYX	serine/threonine/tyrosine interacting protein	**-1,141**	9,26E-03	23,992	20,872
C1orf122	chromosome 1 open reading frame 122	**-1,141**	9,72E-03	35,651	31,017
TRIB3	tribbles pseudokinase 3	**-1,141**	1,09E-02	75,877	67,023
SNRPD1	small nuclear ribonucleoprotein D1 polypeptide	**-1,141**	1,37E-02	36,743	31,760
FIBIN	fin bud initiation factor homolog (zebrafish)	**-1,141**	1,40E-02	166,808	146,760
STK38L	serine/threonine kinase 38 like	**-1,141**	1,45E-02	128,056	110,943
C20orf27	chromosome 20 open reading frame 27	**-1,141**	1,70E-02	22,943	19,830
COPS3	COP9 signalosome subunit 3	**-1,141**	1,78E-02	45,852	39,610
SSX2IP	SSX family member 2 interacting protein	**-1,141**	1,84E-02	19,535	16,982
GMPPB	GDP-mannose pyrophosphorylase B	**-1,141**	1,91E-02	58,156	49,978
ARPC5L	actin related protein 2/3 complex subunit 5 like	**-1,141**	2,15E-02	30,560	26,425
RRP15	ribosomal RNA processing 15 homolog	**-1,141**	2,29E-02	30,274	26,106
RFC2	replication factor C subunit 2	**-1,141**	2,33E-02	23,696	20,412
MYC	MYC proto-oncogene, bHLH transcription factor	**-1,141**	2,57E-02	32,445	27,848
THAP9-AS1	THAP9 antisense RNA 1	**-1,141**	2,91E-02	20,817	17,876
TIMM23	translocase of inner mitochondrial membrane 23	**-1,141**	2,91E-02	28,554	24,769
GLDN	gliomedin	**-1,141**	3,02E-02	282,612	247,651
ATG9A	autophagy related 9A	**-1,141**	3,03E-02	20,964	18,103
STOML1	stomatin like 1	**-1,141**	3,09E-02	39,358	33,773
SLC25A20	solute carrier family 25 member 20	**-1,141**	3,22E-02	18,550	15,993
CHTF18	chromosome transmission fidelity factor 18	**-1,141**	4,04E-02	11,831	10,129
HSPA14	heat shock protein family A (Hsp70) member 14	**-1,141**	4,54E-02	11,035	10,091

KNTC1	kinetochore associated 1	**-1,141**	4,72E-02	19,927	17,043
MATN3	matrilin 3	**-1,149**	2,85E-04	204,190	177,357
WDR34	WD repeat domain 34	**-1,149**	8,33E-04	72,057	62,036
SDHA	succinate dehydrogenase complex flavoprotein subunit A	**-1,149**	8,47E-04	114,945	98,465
MRPL20	mitochondrial ribosomal protein L20	**-1,149**	1,36E-03	56,466	48,612
TES	testin LIM domain protein	**-1,149**	1,86E-03	77,610	67,267
SLC7A5	solute carrier family 7 member 5	**-1,149**	2,60E-03	140,029	120,829
GADD45GIP1	GADD45G interacting protein 1	**-1,149**	2,84E-03	67,392	57,895
TRA2B	transformer 2 beta homolog	**-1,149**	3,14E-03	105,566	91,286
CCT5	chaperonin containing TCP1 subunit 5	**-1,149**	3,32E-03	148,411	127,060
MICALL1	MICAL like 1	**-1,149**	3,52E-03	37,801	32,488
SLC7A11	solute carrier family 7 member 11	**-1,149**	3,82E-03	52,215	44,800
TRUB1	TruB pseudouridine synthase family member 1	**-1,149**	3,83E-03	29,105	25,078
TCTA	T-cell leukemia translocation altered	**-1,149**	3,95E-03	66,142	56,811
DYNLL1	dynein light chain LC8-type 1	**-1,149**	4,00E-03	173,264	148,864
PHACTR4	phosphatase and actin regulator 4	**-1,149**	5,09E-03	47,052	40,335
GAS2L1	growth arrest specific 2 like 1	**-1,149**	5,30E-03	26,463	22,670
CUEDC1	CUE domain containing 1	**-1,149**	5,64E-03	31,761	27,235
TNPO1	transportin 1	**-1,149**	6,06E-03	126,234	108,693
KCTD5	potassium channel tetramerization domain containing 5	**-1,149**	6,08E-03	35,765	30,819
RANGAP1	Ran GTPase activating protein 1	**-1,149**	6,37E-03	85,388	72,476
UCHL5	ubiquitin C-terminal hydrolase L5	**-1,149**	6,77E-03	25,698	22,096
TP53INP2	tumor protein p53 inducible nuclear protein 2	**-1,149**	6,83E-03	30,791	26,616
EMC8	ER membrane protein complex subunit 8	**-1,149**	7,00E-03	17,478	15,028
MCTS1	MCTS1, re-initiation and release factor	**-1,149**	7,70E-03	23,818	20,392
ATP10A	ATPase phospholipid transporting 10A (putative)	**-1,149**	7,78E-03	67,000	57,373
DDAH1	dimethylarginine dimethylaminohydrolase 1	**-1,149**	7,93E-03	135,255	116,694
ARSJ	arylsulfatase family member J	**-1,149**	8,14E-03	112,260	96,295
NAA25	N(alpha)-acetyltransferase 25, NatB auxiliary subunit	**-1,149**	8,14E-03	21,374	18,335
TRMT6	tRNA methyltransferase 6	**-1,149**	8,88E-03	23,200	19,914
TRMT61A	tRNA methyltransferase 61A	**-1,149**	1,11E-02	26,027	22,290
TASP1	taspase 1	**-1,149**	1,13E-02	18,091	15,436
RPF2	ribosome production factor 2 homolog	**-1,149**	1,25E-02	30,492	26,090

MAGED1	MAGE family member D1	**-1,149**	1,36E-02	317,870	275,756
KDELR3	KDEL endoplasmic reticulum protein retention receptor 3	**-1,149**	1,43E-02	107,293	92,439
TBC1D2	TBC1 domain family member 2	**-1,149**	1,53E-02	21,715	18,587
MRTO4	MRT4 homolog, ribosome maturation factor	**-1,149**	1,56E-02	36,584	31,170
NSMF	NMDA receptor synaptonuclear signaling and neuronal migration	**-1,149**	1,59E-02	21,269	18,132
MICAL2	microtubule associated monooxygenase, calponin and LIM domai	**-1,149**	1,85E-02	55,770	47,879
PFAS	phosphoribosylformylglycinamidine synthase	**-1,149**	1,86E-02	17,415	14,909
INTS7	integrator complex subunit 7	**-1,149**	1,87E-02	16,208	13,898
TMEM104	transmembrane protein 104	**-1,149**	1,94E-02	34,491	29,580
SERPINB9	serpin family B member 9	**-1,149**	2,01E-02	13,777	11,842
FAM180A	family with sequence similarity 180 member A	**-1,149**	2,15E-02	32,197	27,927
PDE7B	phosphodiesterase 7B	**-1,149**	2,22E-02	21,477	18,323
GCLC	glutamate-cysteine ligase catalytic subunit	**-1,149**	2,30E-02	22,431	19,124
LRWD1	leucine rich repeats and WD repeat domain containing 1	**-1,149**	2,33E-02	19,257	16,327
FADS3	fatty acid desaturase 3	**-1,149**	2,38E-02	66,346	57,164
NAV1	neuron navigator 1	**-1,149**	2,50E-02	157,068	134,392
NREP	neuronal regeneration related protein	**-1,149**	3,06E-02	50,931	43,476
CARNMT1	carnosine N-methyltransferase 1	**-1,149**	3,34E-02	12,006	10,213
CEP78	centrosomal protein 78	**-1,149**	3,44E-02	17,497	14,935
ARL13B	ADP ribosylation factor like GTPase 13B	**-1,149**	3,54E-02	11,151	9,541
POP1	POP1 homolog, ribonuclease P/MRP subunit	**-1,149**	3,68E-02	10,291	8,719
TTC39B	tetratricopeptide repeat domain 39B	**-1,149**	3,89E-02	10,709	9,145
PWWP2A	PWWP domain containing 2A	**-1,149**	3,91E-02	11,956	10,159
RAD18	RAD18, E3 ubiquitin protein ligase	**-1,149**	4,75E-02	10,243	8,664
DUT	deoxyuridine triphosphatase	**-1,157**	5,94E-05	82,235	70,259
PARP1	poly(ADP-ribose) polymerase 1	**-1,157**	3,61E-04	93,861	80,157
SLBP	stem-loop binding protein	**-1,157**	6,22E-04	52,268	44,767
ACTR3	ARP3 actin related protein 3 homolog	**-1,157**	8,02E-04	231,896	198,847
AXL	AXL receptor tyrosine kinase	**-1,157**	8,60E-04	346,656	297,270
POLD2	DNA polymerase delta 2, accessory subunit	**-1,157**	9,50E-04	108,144	92,318
MEDAG	mesenteric estrogen dependent adipogenesis	**-1,157**	1,23E-03	53,924	46,238
MYL12A	myosin light chain 12A	**-1,157**	1,51E-03	206,362	177,138
ABCE1	ATP binding cassette subfamily E member 1	**-1,157**	1,78E-03	64,048	54,550

PTBP1	polypyrimidine tract binding protein 1	**-1,157**	2,26E-03	217,519	185,126
LGALS1	galectin 1	**-1,157**	2,26E-03	295,548	257,027
GTPBP4	GTP binding protein 4	**-1,157**	2,61E-03	57,703	49,134
STMN1	stathmin 1	**-1,157**	2,83E-03	56,830	48,736
DNAJC2	DnaJ heat shock protein family (Hsp40) member C2	**-1,157**	3,51E-03	24,884	21,274
PNP	purine nucleoside phosphorylase	**-1,157**	3,52E-03	81,242	69,072
PSMG1	proteasome assembly chaperone 1	**-1,157**	3,52E-03	37,342	32,047
EWSR1	EWS RNA binding protein 1	**-1,157**	3,52E-03	148,782	127,009
NOP58	NOP58 ribonucleoprotein	**-1,157**	3,63E-03	51,686	43,839
NUP58	nucleoporin 58	**-1,157**	3,71E-03	44,848	38,201
POSTN	periostin	**-1,157**	4,23E-03	72,971	63,446
ACTN1	actinin alpha 1	**-1,157**	4,83E-03	234,563	201,574
MRPL17	mitochondrial ribosomal protein L17	**-1,157**	4,88E-03	57,898	49,279
FAM107B	family with sequence similarity 107 member B	**-1,157**	5,49E-03	57,393	48,289
G6PC3	glucose-6-phosphatase catalytic subunit 3	**-1,157**	5,98E-03	50,892	43,599
CDCA4	cell division cycle associated 4	**-1,157**	6,00E-03	25,461	21,520
TMEM41B	transmembrane protein 41B	**-1,157**	6,41E-03	26,563	22,666
BAIAP2L1	BAI1 associated protein 2 like 1	**-1,157**	8,08E-03	58,908	50,482
SNRPA1	small nuclear ribonucleoprotein polypeptide A'	**-1,157**	8,13E-03	22,404	19,177
LIPA	lipase A, lysosomal acid type	**-1,157**	8,50E-03	18,501	15,755
TOR3A	torsin family 3 member A	**-1,157**	8,83E-03	34,304	29,003
MASTL	microtubule associated serine/threonine kinase like	**-1,157**	9,75E-03	21,140	17,791
ADRB2	adrenoceptor beta 2	**-1,157**	9,76E-03	30,959	26,300
RFWD3	ring finger and WD repeat domain 3	**-1,157**	1,07E-02	35,181	29,978
NAA10	N(alpha)-acetyltransferase 10, NatA catalytic subunit	**-1,157**	1,11E-02	17,073	14,533
HPRT1	hypoxanthine phosphoribosyltransferase 1	**-1,157**	1,25E-02	27,721	23,395
DPP3	dipeptidyl peptidase 3	**-1,157**	1,31E-02	27,325	23,217
WDR43	WD repeat domain 43	**-1,157**	1,34E-02	30,353	25,776
MCU	mitochondrial calcium uniporter	**-1,157**	1,36E-02	16,950	14,317
B3GALNT1	beta-1,3-N-acetylgalactosaminyltransferase 1 (globoside blood gr	**-1,157**	1,37E-02	21,777	18,516
NOL6	nucleolar protein 6	**-1,157**	1,49E-02	45,910	39,017
RIN1	Ras and Rab interactor 1	**-1,157**	1,50E-02	28,585	24,279
LMBRD2	LMBR1 domain containing 2	**-1,157**	1,52E-02	33,150	28,025

SSH1	slingshot protein phosphatase 1	**-1,157**	1,74E-02	77,345	65,539
KCNK6	potassium two pore domain channel subfamily K member 6	**-1,157**	1,99E-02	27,598	23,598
PUSL1	pseudouridylate synthase-like 1	**-1,157**	2,01E-02	14,109	12,000
ACER3	alkaline ceramidase 3	**-1,157**	2,02E-02	15,055	12,794
PPIL1	peptidylprolyl isomerase like 1	**-1,157**	2,12E-02	26,606	22,575
RABEPK	Rab9 effector protein with kelch motifs	**-1,157**	2,33E-02	16,877	14,330
PUM3	pumilio RNA binding family member 3	**-1,157**	2,87E-02	22,451	18,952
NAP1L5	nucleosome assembly protein 1 like 5	**-1,157**	3,00E-02	9,576	8,158
KLHDC8B	kelch domain containing 8B	**-1,157**	3,07E-02	9,071	7,620
ZMYND19	zinc finger MYND-type containing 19	**-1,157**	3,38E-02	16,248	13,722
REEP4	receptor accessory protein 4	**-1,157**	3,57E-02	15,531	12,978
PRTFDC1	phosphoribosyl transferase domain containing 1	**-1,157**	4,29E-02	7,573	6,394
APOO	apolipoprotein O	**-1,157**	4,44E-02	9,508	8,015
TRIM59	tripartite motif containing 59	**-1,157**	4,46E-02	8,369	7,079
CCDC167	coiled-coil domain containing 167	**-1,157**	4,97E-02	8,653	7,330
H2AFZ	H2A histone family member Z	**-1,165**	2,65E-05	139,440	118,189
NIPA2	non imprinted in Prader-Willi/Angelman syndrome 2	**-1,165**	7,47E-04	54,255	46,170
DDX21	DExD-box helicase 21	**-1,165**	1,39E-03	105,331	88,943
GLS	glutaminase	**-1,165**	1,51E-03	245,731	208,709
FARSB	phenylalanyl-tRNA synthetase beta subunit	**-1,165**	2,61E-03	52,230	44,272
SMC2	structural maintenance of chromosomes 2	**-1,165**	2,98E-03	45,529	38,688
PIP5K1A	phosphatidylinositol-4-phosphate 5-kinase type 1 alpha	**-1,165**	3,43E-03	49,224	41,594
KLF10	Kruppel like factor 10	**-1,165**	3,56E-03	68,480	57,620
SNRNP40	small nuclear ribonucleoprotein U5 subunit 40	**-1,165**	4,97E-03	26,466	22,245
PIGU	phosphatidylinositol glycan anchor biosynthesis class U	**-1,165**	4,99E-03	21,125	17,883
NDUFS8	NADH:ubiquinone oxidoreductase core subunit S8	**-1,165**	6,07E-03	23,987	20,335
FSCN1	fascin actin-bundling protein 1	**-1,165**	6,15E-03	219,660	184,864
ACLY	ATP citrate lyase	**-1,165**	7,39E-03	238,290	202,723
STEAP4	STEAP4 metalloreductase	**-1,165**	7,40E-03	75,033	63,845
IGFBP3	insulin like growth factor binding protein 3	**-1,165**	7,70E-03	3955,606	3394,093
SLC25A1	solute carrier family 25 member 1	**-1,165**	8,02E-03	69,303	59,223
SERPINH1	serpin family H member 1	**-1,165**	9,62E-03	298,286	251,222
ABCF2	ATP binding cassette subfamily F member 2	**-1,165**	1,01E-02	0,000	0,000

CMSS1	cms1 ribosomal small subunit homolog (yeast)	**-1,165**	1,04E-02	21,090	17,724
DCUN1D5	defective in cullin neddylation 1 domain containing 5	**-1,165**	1,16E-02	21,109	17,847
TUBB2B	tubulin beta 2B class IIb	**-1,165**	1,19E-02	26,595	22,231
FIGNL1	fidgetin like 1	**-1,165**	1,32E-02	12,969	10,881
SNRNP25	small nuclear ribonucleoprotein U11/U12 subunit 25	**-1,165**	1,37E-02	16,952	14,311
FKBP14	FK506 binding protein 14	**-1,165**	1,78E-02	52,887	44,419
GPATCH4	G-patch domain containing 4	**-1,165**	2,00E-02	32,666	27,263
MED27	mediator complex subunit 27	**-1,165**	2,22E-02	19,597	16,359
DSN1	DSN1 homolog, MIS12 kinetochore complex component	**-1,165**	2,36E-02	13,567	11,287
CDC7	cell division cycle 7	**-1,165**	2,46E-02	10,123	8,519
ARNTL2	aryl hydrocarbon receptor nuclear translocator like 2	**-1,165**	2,56E-02	16,205	13,483
CKS2	CDC28 protein kinase regulatory subunit 2	**-1,165**	2,69E-02	12,004	9,974
RAB30	RAB30, member RAS oncogene family	**-1,165**	2,94E-02	9,302	7,827
HMGB3	high mobility group box 3	**-1,165**	3,24E-02	10,593	8,716
TRDMT1	tRNA aspartic acid methyltransferase 1	**-1,165**	3,24E-02	8,933	7,519
TMEM26	transmembrane protein 26	**-1,165**	4,17E-02	13,969	11,861
SHROOM3	shroom family member 3	**-1,165**	4,45E-02	22,294	18,614
CENPL	centromere protein L	**-1,165**	4,50E-02	9,448	7,775
MPV17L2	MPV17 mitochondrial inner membrane protein like 2	**-1,165**	4,67E-02	8,919	7,441
RRM1	ribonucleotide reductase catalytic subunit M1	**-1,173**	9,34E-05	85,945	72,233
UAP1	UDP-N-acetylglucosamine pyrophosphorylase 1	**-1,173**	9,67E-05	233,113	195,761
RAN	RAN, member RAS oncogene family	**-1,173**	1,29E-04	246,914	206,751
GADD45B	growth arrest and DNA damage inducible beta	**-1,173**	1,81E-04	116,026	99,319
PSMD6	proteasome 26S subunit, non-ATPase 6	**-1,173**	4,85E-04	56,328	47,550
SRSF3	serine and arginine rich splicing factor 3	**-1,173**	9,50E-04	191,318	161,096
FOXC2	forkhead box C2	**-1,173**	1,76E-03	41,674	34,900
ELOVL5	ELOVL fatty acid elongase 5	**-1,173**	1,84E-03	82,741	69,597
SRF	serum response factor	**-1,173**	1,90E-03	40,202	33,831
RNF144B	ring finger protein 144B	**-1,173**	2,07E-03	46,277	38,670
C19orf48	chromosome 19 open reading frame 48	**-1,173**	2,10E-03	27,165	22,790
MRPL3	mitochondrial ribosomal protein L3	**-1,173**	2,17E-03	73,409	62,061
IFRD2	interferon related developmental regulator 2	**-1,173**	2,37E-03	52,765	44,315
SLC16A3	solute carrier family 16 member 3	**-1,173**	2,71E-03	247,797	210,619

NAA15	N(alpha)-acetyltransferase 15, NatA auxiliary subunit	**-1,173**	3,28E-03	39,959	33,582
TRAM2	translocation associated membrane protein 2	**-1,173**	3,62E-03	415,498	348,523
COL2A1	collagen type II alpha 1 chain	**-1,173**	3,74E-03	17921,647	15141,588
CLTB	clathrin light chain B	**-1,173**	3,95E-03	96,541	80,719
NCBP1	nuclear cap binding protein subunit 1	**-1,173**	4,77E-03	45,331	38,155
NIFK	nucleolar protein interacting with the FHA domain of MKI67	**-1,173**	5,06E-03	27,412	22,920
PDGFC	platelet derived growth factor C	**-1,173**	5,25E-03	76,826	65,603
GPR180	G protein-coupled receptor 180	**-1,173**	5,25E-03	29,987	25,286
BAG2	BCL2 associated athanogene 2	**-1,173**	5,43E-03	18,779	15,737
PDLIM2	PDZ and LIM domain 2	**-1,173**	5,44E-03	35,991	29,835
PHTF1	putative homeodomain transcription factor 1	**-1,173**	5,49E-03	19,978	16,635
ASPM	abnormal spindle microtubule assembly	**-1,173**	5,88E-03	19,774	16,373
SLC38A6	solute carrier family 38 member 6	**-1,173**	5,98E-03	21,051	17,801
SLC12A4	solute carrier family 12 member 4	**-1,173**	6,10E-03	103,925	87,786
NT5DC3	5'-nucleotidase domain containing 3	**-1,173**	6,79E-03	17,265	14,465
RASD1	ras related dexamethasone induced 1	**-1,173**	7,02E-03	29,891	25,425
POMT2	protein O-mannosyltransferase 2	**-1,173**	7,52E-03	16,751	13,950
PNPT1	polyribonucleotide nucleotidyltransferase 1	**-1,173**	9,22E-03	22,125	18,496
CDR2L	cerebellar degeneration related protein 2 like	**-1,173**	9,64E-03	10,316	8,564
TUBA4A	tubulin alpha 4a	**-1,173**	9,71E-03	30,141	25,105
PSRC1	proline and serine rich coiled-coil 1	**-1,173**	9,86E-03	11,405	9,558
TSC22D3	TSC22 domain family member 3	**-1,173**	1,01E-02	75,954	63,471
PODXL	podocalyxin like	**-1,173**	1,09E-02	43,185	35,338
EPG5	ectopic P-granules autophagy protein 5 homolog	**-1,173**	1,13E-02	129,779	108,911
BRCA1	BRCA1, DNA repair associated	**-1,173**	1,71E-02	22,285	18,383
CYB5R2	cytochrome b5 reductase 2	**-1,173**	2,06E-02	14,250	11,821
RGS19	regulator of G protein signaling 19	**-1,173**	2,26E-02	8,592	7,137
MRPL12	mitochondrial ribosomal protein L12	**-1,173**	2,84E-02	9,995	8,278
DHODH	dihydroorotate dehydrogenase (quinone)	**-1,173**	3,19E-02	8,131	6,771
KCNE4	potassium voltage-gated channel subfamily E regulatory subunit 4	**-1,173**	3,56E-02	8,465	6,933
SAPCD2	suppressor APC domain containing 2	**-1,173**	3,71E-02	8,014	6,545
MOCOS	molybdenum cofactor sulfurase	**-1,173**	4,11E-02	11,316	9,168
CCND1	cyclin D1	**-1,173**	4,28E-02	336,046	276,798

CD3EAP	CD3e molecule associated protein	**-1,173**	4,91E-02	9,527	7,756
IMPA1	inositol monophosphatase 1	**-1,181**	4,82E-05	37,367	31,436
TAGLN2	transgelin 2	**-1,181**	7,19E-05	168,012	140,696
SRSF2	serine and arginine rich splicing factor 2	**-1,181**	2,27E-04	133,746	111,141
SLC25A5	solute carrier family 25 member 5	**-1,181**	2,85E-04	120,420	100,937
GLO1	glyoxalase I	**-1,181**	3,02E-04	108,035	90,524
SDC1	syndecan 1	**-1,181**	4,14E-04	85,792	70,729
MCM3	minichromosome maintenance complex component 3	**-1,181**	8,47E-04	84,443	70,031
PPP1R14B	protein phosphatase 1 regulatory inhibitor subunit 14B	**-1,181**	1,03E-03	59,246	48,869
CCT6A	chaperonin containing TCP1 subunit 6A	**-1,181**	1,20E-03	159,816	133,369
CITED2	Cbp/p300 interacting transactivator with Glu/Asp rich carboxy-ter	**-1,181**	1,41E-03	205,697	174,329
TIMM10B	translocase of inner mitochondrial membrane 10B	**-1,181**	1,60E-03	23,145	19,335
ME1	malic enzyme 1	**-1,181**	1,66E-03	37,033	31,138
EIF4EBP1	eukaryotic translation initiation factor 4E binding protein 1	**-1,181**	1,74E-03	49,531	41,107
YIF1A	Yip1 interacting factor homolog A, membrane trafficking protein	**-1,181**	1,78E-03	89,496	75,034
SLC31A2	solute carrier family 31 member 2	**-1,181**	2,26E-03	34,025	28,326
POLD1	DNA polymerase delta 1, catalytic subunit	**-1,181**	2,65E-03	19,539	16,208
SCARF2	scavenger receptor class F member 2	**-1,181**	2,76E-03	55,022	45,911
ANKRD9	ankyrin repeat domain 9	**-1,181**	3,51E-03	16,742	13,893
KPNB1	karyopherin subunit beta 1	**-1,181**	3,52E-03	227,368	189,072
SDC4	syndecan 4	**-1,181**	3,65E-03	244,067	202,505
MTSS1L	MTSS1L, I-BAR domain containing	**-1,181**	3,74E-03	75,418	62,789
GTF3C6	general transcription factor IIIC subunit 6	**-1,181**	3,90E-03	46,382	38,553
CCDC137	coiled-coil domain containing 137	**-1,181**	4,08E-03	19,448	16,102
IQGAP3	IQ motif containing GTPase activating protein 3	**-1,181**	5,04E-03	34,336	28,926
RAB23	RAB23, member RAS oncogene family	**-1,181**	5,69E-03	20,409	16,832
RRP36	ribosomal RNA processing 36	**-1,181**	5,72E-03	33,925	28,054
SNRPF	small nuclear ribonucleoprotein polypeptide F	**-1,181**	5,79E-03	20,845	17,239
CABLES1	Cdk5 and Abl enzyme substrate 1	**-1,181**	6,93E-03	20,423	16,752
NCAPD3	non-SMC condensin II complex subunit D3	**-1,181**	7,34E-03	30,671	25,488
PNO1	partner of NOB1 homolog	**-1,181**	7,70E-03	24,728	20,507
ARMC6	armadillo repeat containing 6	**-1,181**	8,13E-03	23,425	19,539
ISOC2	isochorismatase domain containing 2	**-1,181**	8,35E-03	27,376	22,817

CENPF	centromere protein F	**-1,181**	8,50E-03	39,355	33,323
PCYT2	phosphate cytidylyltransferase 2, ethanolamine	**-1,181**	8,65E-03	36,362	29,971
RRS1	ribosome biogenesis regulator homolog	**-1,181**	9,03E-03	14,202	11,707
ALG3	ALG3, alpha-1,3- mannosyltransferase	**-1,181**	9,11E-03	34,043	28,308
C11orf24	chromosome 11 open reading frame 24	**-1,181**	1,00E-02	68,051	56,760
BTG3	BTG anti-proliferation factor 3	**-1,181**	1,06E-02	18,356	15,166
CHSY3	chondroitin sulfate synthase 3	**-1,181**	1,08E-02	28,062	23,081
SCFD2	sec1 family domain containing 2	**-1,181**	1,26E-02	20,129	16,675
RAB15	RAB15, member RAS oncogene family	**-1,181**	1,28E-02	9,744	7,968
SLC5A6	solute carrier family 5 member 6	**-1,181**	1,36E-02	18,188	15,021
CACNB1	calcium voltage-gated channel auxiliary subunit beta 1	**-1,181**	1,65E-02	10,425	8,525
TOE1	target of EGR1, member 1 (nuclear)	**-1,181**	1,65E-02	13,967	11,526
MRAP2	melanocortin 2 receptor accessory protein 2	**-1,181**	1,73E-02	44,191	35,874
SLC17A9	solute carrier family 17 member 9	**-1,181**	1,75E-02	22,905	18,835
CAPN5	calpain 5	**-1,181**	2,14E-02	9,579	7,876
SLC19A1	solute carrier family 19 member 1	**-1,181**	2,55E-02	8,157	6,710
SNHG15	small nucleolar RNA host gene 15	**-1,181**	2,69E-02	7,634	6,258
TIGAR	TP53 induced glycolysis regulatory phosphatase	**-1,181**	2,85E-02	7,794	6,440
PTGFR	prostaglandin F receptor	**-1,181**	3,32E-02	6,133	4,994
CENPE	centromere protein E	**-1,181**	3,43E-02	10,965	8,778
KIF14	kinesin family member 14	**-1,181**	4,27E-02	7,819	6,464
NAT14	N-acetyltransferase 14 (putative)	**-1,181**	4,38E-02	6,423	5,242
CSE1L	chromosome segregation 1 like	**-1,189**	8,66E-05	98,067	81,236
STK17A	serine/threonine kinase 17a	**-1,189**	1,94E-04	130,917	108,125
UGP2	UDP-glucose pyrophosphorylase 2	**-1,189**	2,94E-04	271,830	226,395
SAE1	SUMO1 activating enzyme subunit 1	**-1,189**	4,96E-04	97,507	80,263
NTN4	netrin 4	**-1,189**	5,07E-04	529,885	436,154
NT5DC2	5'-nucleotidase domain containing 2	**-1,189**	5,35E-04	82,982	69,070
TUBB4B	tubulin beta 4B class IVb	**-1,189**	6,80E-04	274,877	227,648
ACTG1	actin gamma 1	**-1,189**	7,24E-04	2050,123	1690,664
UGDH	UDP-glucose 6-dehydrogenase	**-1,189**	9,70E-04	398,084	329,489
LIG1	DNA ligase 1	**-1,189**	1,23E-03	21,930	17,903
TMEM45A	transmembrane protein 45A	**-1,189**	1,39E-03	159,142	131,914

SLC9A3R1	SLC9A3 regulator 1	**-1,189**	1,42E-03	66,443	54,559
RANBP1	RAN binding protein 1	**-1,189**	2,15E-03	57,005	46,802
PTTG1	pituitary tumor-transforming 1	**-1,189**	2,38E-03	14,290	11,766
NOP56	NOP56 ribonucleoprotein	**-1,189**	2,97E-03	72,269	59,421
SLC25A25	solute carrier family 25 member 25	**-1,189**	3,18E-03	13,004	10,727
RRP9	ribosomal RNA processing 9, small subunit (SSU) processome com	**-1,189**	3,26E-03	18,325	15,067
GCNT1	glucosaminyl (N-acetyl) transferase 1, core 2	**-1,189**	3,71E-03	71,623	61,311
AACS	acetoacetyl-CoA synthetase	**-1,189**	3,76E-03	40,646	34,321
SDF2L1	stromal cell derived factor 2 like 1	**-1,189**	4,30E-03	23,455	19,319
GOT1	glutamic-oxaloacetic transaminase 1	**-1,189**	4,93E-03	45,373	37,199
ATAD3A	ATPase family, AAA domain containing 3A	**-1,189**	5,27E-03	30,473	24,837
EHD4	EH domain containing 4	**-1,189**	5,43E-03	37,044	30,568
ACSL1	acyl-CoA synthetase long-chain family member 1	**-1,189**	5,59E-03	30,356	25,187
SCML1	sex comb on midleg like 1 (Drosophila)	**-1,189**	6,26E-03	12,297	10,174
CRISPLD2	cysteine rich secretory protein LCCL domain containing 2	**-1,189**	8,43E-03	24,225	19,781
CHAC1	ChaC glutathione specific gamma-glutamylcyclotransferase 1	**-1,189**	9,02E-03	27,719	22,845
HMBS	hydroxymethylbilane synthase	**-1,189**	1,37E-02	11,780	9,612
CENPH	centromere protein H	**-1,189**	1,43E-02	12,063	9,736
SYNC	syncoilin, intermediate filament protein	**-1,189**	1,85E-02	11,998	9,699
SH3RF2	SH3 domain containing ring finger 2	**-1,189**	3,00E-02	7,620	6,178
KIF18B	kinesin family member 18B	**-1,189**	3,88E-02	8,476	7,037
RCSD1	RCSD domain containing 1	**-1,189**	4,36E-02	8,723	7,021
PSMD14	proteasome 26S subunit, non-ATPase 14	**-1,197**	2,23E-04	64,910	53,693
CCDC85B	coiled-coil domain containing 85B	**-1,197**	3,34E-04	100,868	82,601
CORO1C	coronin 1C	**-1,197**	5,34E-04	109,168	89,166
EIF5A	eukaryotic translation initiation factor 5A	**-1,197**	8,93E-04	260,944	213,261
NIP7	NIP7, nucleolar pre-rRNA processing protein	**-1,197**	1,15E-03	23,602	19,300
WEE1	WEE1 G2 checkpoint kinase	**-1,197**	1,26E-03	21,747	17,823
RCL1	RNA terminal phosphate cyclase like 1	**-1,197**	2,25E-03	27,693	22,748
CYTH3	cytohesin 3	**-1,197**	2,75E-03	45,435	36,646
FOXM1	forkhead box M1	**-1,197**	3,04E-03	41,926	34,791
GNPNAT1	glucosamine-phosphate N-acetyltransferase 1	**-1,197**	3,95E-03	49,353	40,007
BOP1	block of proliferation 1	**-1,197**	5,18E-03	30,021	24,228

FLRT2	fibronectin leucine rich transmembrane protein 2	**-1,197**	6,71E-03	16,162	13,072
SLC6A9	solute carrier family 6 member 9	**-1,197**	8,13E-03	28,969	23,413
FJX1	four jointed box 1	**-1,197**	8,14E-03	15,495	12,485
CDC42EP1	CDC42 effector protein 1	**-1,197**	9,47E-03	29,942	24,157
PRADC1	protease associated domain containing 1	**-1,197**	1,18E-02	19,560	15,879
MAGOHB	mago homolog B, exon junction complex core component	**-1,197**	1,45E-02	15,158	12,273
GINS3	GINS complex subunit 3	**-1,197**	1,55E-02	7,768	6,154
SUV39H2	suppressor of variegation 3-9 homolog 2	**-1,197**	1,74E-02	7,787	6,276
PSTPIP2	proline-serine-threonine phosphatase interacting protein 2	**-1,197**	2,01E-02	12,273	9,915
S1PR3	sphingosine-1-phosphate receptor 3	**-1,197**	2,25E-02	8,283	6,655
MYO5C	myosin VC	**-1,197**	2,44E-02	16,849	13,749
MARS2	methionyl-tRNA synthetase 2, mitochondrial	**-1,197**	3,62E-02	3,812	3,006
COL5A1	collagen type V alpha 1 chain	**-1,197**	3,95E-02	815,117	640,789
ZGRF1	zinc finger GRF-type containing 1	**-1,197**	4,42E-02	4,597	3,559
UCHL3	ubiquitin C-terminal hydrolase L3	**-1,197**	4,50E-02	4,450	3,527
DTYMK	deoxythymidylate kinase	**-1,206**	2,40E-04	30,342	24,703
EBNA1BP2	EBNA1 binding protein 2	**-1,206**	4,67E-04	55,365	44,904
CDK2	cyclin dependent kinase 2	**-1,206**	7,23E-04	33,859	27,244
FRMD6	FERM domain containing 6	**-1,206**	1,36E-03	142,194	114,922
BYSL	bystin like	**-1,206**	1,54E-03	19,682	15,913
NANS	N-acetylneuraminate synthase	**-1,206**	1,84E-03	64,698	52,882
KIF20B	kinesin family member 20B	**-1,206**	2,37E-03	14,683	11,816
MPHOSPH6	M-phase phosphoprotein 6	**-1,206**	2,71E-03	12,519	10,172
IDH2	isocitrate dehydrogenase (NADP(+)) 2, mitochondrial	**-1,206**	3,02E-03	42,748	34,137
SIX4	SIX homeobox 4	**-1,206**	3,50E-03	37,342	29,728
KLF2	Kruppel like factor 2	**-1,206**	3,52E-03	37,542	30,135
LRRC15	leucine rich repeat containing 15	**-1,206**	4,44E-03	91,480	72,832
KNSTRN	kinetochore localized astrin/SPAG5 binding protein	**-1,206**	5,19E-03	11,909	9,811
YRDC	yrdC N6-threonylcarbamoyltransferase domain containing	**-1,206**	5,28E-03	13,617	11,025
MKI67	marker of proliferation Ki-67	**-1,206**	5,50E-03	59,239	47,792
BOLA3	bolA family member 3	**-1,206**	5,65E-03	16,795	13,426
SUV39H1	suppressor of variegation 3-9 homolog 1	**-1,206**	6,15E-03	8,684	6,994
SPDL1	spindle apparatus coiled-coil protein 1	**-1,206**	6,27E-03	9,774	7,867

EXOSC9	exosome component 9	**-1,206**	6,45E-03	18,354	14,616
ADAMTS2	ADAM metallopeptidase with thrombospondin type 1 motif 2	**-1,206**	6,79E-03	18,815	15,030
SNHG17	small nucleolar RNA host gene 17	**-1,206**	7,70E-03	9,719	7,838
DCLRE1B	DNA cross-link repair 1B	**-1,206**	8,14E-03	12,638	10,216
NFATC2	nuclear factor of activated T-cells 2	**-1,206**	9,15E-03	18,318	14,692
ATAD3B	ATPase family, AAA domain containing 3B	**-1,206**	9,84E-03	17,439	14,030
TLE4	transducin like enhancer of split 4	**-1,206**	1,02E-02	8,389	6,635
RFC4	replication factor C subunit 4	**-1,206**	1,19E-02	14,397	11,440
FAM43B	family with sequence similarity 43 member B	**-1,206**	1,27E-02	10,436	8,357
CHAF1B	chromatin assembly factor 1 subunit B	**-1,206**	1,30E-02	9,127	7,299
CCNF	cyclin F	**-1,206**	1,59E-02	7,071	5,516
C1orf112	chromosome 1 open reading frame 112	**-1,206**	1,79E-02	6,543	5,201
CSF1	colony stimulating factor 1	**-1,206**	1,88E-02	511,555	403,608
PDE5A	phosphodiesterase 5A	**-1,206**	1,93E-02	18,536	15,046
SPAG1	sperm associated antigen 1	**-1,206**	2,36E-02	5,318	4,196
LIMS2	LIM zinc finger domain containing 2	**-1,206**	2,70E-02	5,868	4,695
CCDC18	coiled-coil domain containing 18	**-1,206**	2,93E-02	5,577	4,342
CDK5R1	cyclin dependent kinase 5 regulatory subunit 1	**-1,206**	4,71E-02	3,477	2,673
MCM7	minichromosome maintenance complex component 7	**-1,214**	4,82E-05	109,064	88,507
TMPO	thymopoietin	**-1,214**	1,40E-04	67,075	53,553
TPM4	tropomyosin 4	**-1,214**	1,96E-04	272,145	218,132
PAICS	phosphoribosylaminoimidazole carboxylase and phosphoribosylai	**-1,214**	2,07E-04	108,859	87,814
NCAPG2	non-SMC condensin II complex subunit G2	**-1,214**	2,16E-04	35,023	27,861
C12orf75	chromosome 12 open reading frame 75	**-1,214**	2,48E-04	55,213	45,065
RBMS2	RNA binding motif single stranded interacting protein 2	**-1,214**	3,02E-04	81,575	65,975
PRSS23	protease, serine 23	**-1,214**	4,60E-04	581,744	463,982
LRRC59	leucine rich repeat containing 59	**-1,214**	6,08E-04	188,477	151,681
SELENOI	selenoprotein I	**-1,214**	6,67E-04	31,044	25,054
TIMELESS	timeless circadian clock	**-1,214**	6,94E-04	32,750	26,386
CHCHD3	coiled-coil-helix-coiled-coil-helix domain containing 3	**-1,214**	7,53E-04	22,092	17,793
EZR	ezrin	**-1,214**	8,93E-04	79,833	64,003
RPS6KA4	ribosomal protein S6 kinase A4	**-1,214**	9,59E-04	40,677	32,471
SLC38A10	solute carrier family 38 member 10	**-1,214**	1,23E-03	284,201	230,186

TUBB2A	tubulin beta 2A class IIa	**-1,214**	1,25E-03	56,140	45,253
LSP1	lymphocyte-specific protein 1	**-1,214**	1,38E-03	233,877	189,205
NUDCD1	NudC domain containing 1	**-1,214**	1,41E-03	19,630	15,622
SEMA3C	semaphorin 3C	**-1,214**	1,64E-03	95,880	77,709
LYAR	Ly1 antibody reactive	**-1,214**	2,75E-03	17,488	13,903
OMD	osteomodulin	**-1,214**	2,75E-03	79,422	67,124
SMTN	smoothelin	**-1,214**	2,81E-03	67,453	53,393
MYO1B	myosin IB	**-1,214**	2,84E-03	77,195	61,344
JPT1	Jupiter microtubule associated homolog 1	**-1,214**	2,96E-03	18,963	14,995
CLN6	ceroid-lipofuscinosis, neuronal 6, late infantile, variant	**-1,214**	3,26E-03	15,078	12,129
PYCR1	pyrroline-5-carboxylate reductase 1	**-1,214**	3,71E-03	117,246	94,719
RBL1	RB transcriptional corepressor like 1	**-1,214**	4,15E-03	17,579	13,941
HEATR3	HEAT repeat containing 3	**-1,214**	5,97E-03	9,279	7,329
VDR	vitamin D (1,25- dihydroxyvitamin D3) receptor	**-1,214**	6,77E-03	25,234	20,186
LBR	lamin B receptor	**-1,214**	7,79E-03	10,010	7,947
HYLS1	HYLS1, centriolar and ciliogenesis associated	**-1,214**	8,72E-03	7,786	6,132
ESPL1	extra spindle pole bodies like 1, separase	**-1,214**	9,69E-03	12,874	10,244
SPIN4	spindlin family member 4	**-1,214**	9,87E-03	6,878	5,463
FSTL1	follistatin like 1	**-1,214**	1,14E-02	799,955	631,530
TGM2	transglutaminase 2	**-1,214**	1,36E-02	666,529	529,574
DLGAP5	DLG associated protein 5	**-1,214**	1,43E-02	9,901	7,810
TRPV2	transient receptor potential cation channel subfamily V member [illegible]	**-1,214**	1,47E-02	7,929	6,245
TTK	TTK protein kinase	**-1,214**	1,64E-02	7,664	6,038
METRNL	meteorin like, glial cell differentiation regulator	**-1,214**	1,97E-02	6,044	4,784
COL5A3	collagen type V alpha 3 chain	**-1,214**	2,13E-02	6,274	4,868
NCLN	nicalin	**-1,214**	2,15E-02	131,484	102,312
MTBP	MDM2 binding protein	**-1,214**	2,70E-02	3,835	2,982
COL12A1	collagen type XII alpha 1 chain	**-1,214**	3,13E-02	989,919	769,478
MSX1	msh homeobox 1	**-1,214**	3,69E-02	3,596	2,666
TNFRSF12A	TNF receptor superfamily member 12A	**-1,214**	3,77E-02	170,756	130,464
TRIM6	tripartite motif containing 6	**-1,214**	4,35E-02	3,660	2,728
ZNF587B	zinc finger protein 587B	**-1,214**	4,80E-02	2,742	2,054
LOX	lysyl oxidase	**-1,223**	7,19E-05	193,848	155,373

CDKN2C	cyclin dependent kinase inhibitor 2C	**-1,223**	8,66E-05	47,942	38,596
SNHG1	small nucleolar RNA host gene 1	**-1,223**	1,14E-04	21,352	17,216
MCM6	minichromosome maintenance complex component 6	**-1,223**	1,94E-04	82,560	65,716
PFN1	profilin 1	**-1,223**	2,51E-04	449,700	359,458
SMC4	structural maintenance of chromosomes 4	**-1,223**	2,94E-04	78,956	62,727
RBM3	RNA binding motif (RNP1, RRM) protein 3	**-1,223**	3,88E-04	197,946	158,515
PALLD	palladin, cytoskeletal associated protein	**-1,223**	4,23E-04	70,261	55,554
DBI	diazepam binding inhibitor, acyl-CoA binding protein	**-1,223**	4,82E-04	84,805	68,291
WDR77	WD repeat domain 77	**-1,223**	8,47E-04	24,131	19,195
JAK2	Janus kinase 2	**-1,223**	1,06E-03	70,336	56,325
PENK	proenkephalin	**-1,223**	1,06E-03	159,962	127,602
SRM	spermidine synthase	**-1,223**	1,79E-03	129,893	104,275
ALDH1B1	aldehyde dehydrogenase 1 family member B1	**-1,223**	1,94E-03	18,385	14,520
MAP1B	microtubule associated protein 1B	**-1,223**	2,17E-03	38,349	29,940
NOP16	NOP16 nucleolar protein	**-1,223**	3,32E-03	16,925	13,414
UCK2	uridine-cytidine kinase 2	**-1,223**	3,95E-03	19,340	15,100
ZNF469	zinc finger protein 469	**-1,223**	5,18E-03	30,344	24,175
WDR4	WD repeat domain 4	**-1,223**	5,84E-03	10,304	8,037
CLIC3	chloride intracellular channel 3	**-1,223**	7,15E-03	15,278	12,155
XRCC3	X-ray repair cross complementing 3	**-1,223**	9,26E-03	10,645	8,341
MGLL	monoglyceride lipase	**-1,223**	1,27E-02	224,858	175,000
SLC25A15	solute carrier family 25 member 15	**-1,223**	1,30E-02	6,647	5,175
C16orf87	chromosome 16 open reading frame 87	**-1,223**	1,52E-02	5,236	4,024
GPR68	G protein-coupled receptor 68	**-1,223**	1,83E-02	8,307	6,447
TLCD1	TLC domain containing 1	**-1,223**	2,16E-02	5,651	4,299
DDIAS	DNA damage induced apoptosis suppressor	**-1,223**	3,32E-02	4,794	3,651
ASPN	asporin	**-1,223**	3,78E-02	41,714	31,708
LCTL	lactase like	**-1,223**	3,94E-02	4,636	3,413
TFDP1	transcription factor Dp-1	**-1,231**	4,82E-05	82,182	65,147
H2AFX	H2A histone family member X	**-1,231**	9,66E-05	54,997	42,936
CTNNAL1	catenin alpha like 1	**-1,231**	1,26E-04	47,756	37,791
SRSF7	serine and arginine rich splicing factor 7	**-1,231**	1,67E-04	73,765	58,878
TUBB6	tubulin beta 6 class V	**-1,231**	3,71E-04	229,206	181,271

DNMT1	DNA methyltransferase 1	**-1,231**	7,07E-04	89,600	70,507
TUBG1	tubulin gamma 1	**-1,231**	7,13E-04	32,387	25,653
PROSER2	proline and serine rich 2	**-1,231**	7,43E-04	17,684	13,996
CENPO	centromere protein O	**-1,231**	7,53E-04	22,551	17,895
P3H2	prolyl 3-hydroxylase 2	**-1,231**	7,58E-04	1060,487	854,894
COTL1	coactosin like F-actin binding protein 1	**-1,231**	7,63E-04	61,936	48,817
PUS1	pseudouridylate synthase 1	**-1,231**	9,93E-04	12,479	9,853
MCM2	minichromosome maintenance complex component 2	**-1,231**	1,06E-03	48,693	38,084
LTV1	LTV1 ribosome biogenesis factor	**-1,231**	1,11E-03	14,710	11,599
EPHA2	EPH receptor A2	**-1,231**	2,10E-03	49,680	38,897
CDKN3	cyclin dependent kinase inhibitor 3	**-1,231**	2,21E-03	10,623	8,353
ABHD5	abhydrolase domain containing 5	**-1,231**	3,23E-03	12,747	9,958
GMNN	geminin, DNA replication inhibitor	**-1,231**	4,41E-03	11,758	9,143
HYAL1	hyaluronoglucosaminidase 1	**-1,231**	4,68E-03	39,463	30,872
FABP5	fatty acid binding protein 5	**-1,231**	5,65E-03	6,498	5,040
INO80C	INO80 complex subunit C	**-1,231**	6,30E-03	8,864	6,866
CHAF1A	chromatin assembly factor 1 subunit A	**-1,231**	6,41E-03	16,056	12,512
NEK2	NIMA related kinase 2	**-1,231**	6,93E-03	7,984	6,093
ENPEP	glutamyl aminopeptidase	**-1,231**	7,18E-03	6,451	4,907
PRR11	proline rich 11	**-1,231**	7,67E-03	7,664	5,918
FAM83D	family with sequence similarity 83 member D	**-1,231**	8,02E-03	7,210	5,677
CARD9	caspase recruitment domain family member 9	**-1,231**	8,04E-03	7,411	5,696
NEXN	nexilin F-actin binding protein	**-1,231**	9,61E-03	7,481	5,822
TEX30	testis expressed 30	**-1,231**	9,76E-03	7,244	5,607
SIX1	SIX homeobox 1	**-1,231**	1,07E-02	149,755	114,448
POLE2	DNA polymerase epsilon 2, accessory subunit	**-1,231**	2,85E-02	4,435	3,283
OSR1	odd-skipped related transciption factor 1	**-1,231**	3,57E-02	3,316	2,312
BLM	Bloom syndrome RecQ like helicase	**-1,231**	3,78E-02	4,470	3,192
MND1	meiotic nuclear divisions 1	**-1,231**	3,98E-02	2,587	1,837
DRAP1	DR1 associated protein 1	**-1,240**	1,67E-04	124,226	97,349
ALYREF	Aly/REF export factor	**-1,240**	2,48E-04	66,342	52,033
VEGFA	vascular endothelial growth factor A	**-1,240**	2,85E-04	280,556	224,717
HNRNPAB	heterogeneous nuclear ribonucleoprotein A/B	**-1,240**	3,71E-04	126,040	98,506

B4GALT1	beta-1,4-galactosyltransferase 1	**-1,240**	5,60E-04	96,591	75,867
DNAJC9	DnaJ heat shock protein family (Hsp40) member C9	**-1,240**	7,63E-04	27,756	21,462
CDCA3	cell division cycle associated 3	**-1,240**	1,00E-03	14,699	11,534
MCM8	minichromosome maintenance 8 homologous recombination rep	**-1,240**	1,19E-03	15,168	11,811
PART1	prostate androgen-regulated transcript 1 (non-protein coding)	**-1,240**	1,38E-03	21,604	16,898
RUSC2	RUN and SH3 domain containing 2	**-1,240**	1,71E-03	56,517	44,408
BCL2L12	BCL2 like 12	**-1,240**	2,12E-03	8,122	6,326
EHD1	EH domain containing 1	**-1,240**	2,47E-03	54,561	42,657
SAC3D1	SAC3 domain containing 1	**-1,240**	2,84E-03	14,092	10,712
PGP	phosphoglycolate phosphatase	**-1,240**	2,84E-03	14,731	11,372
CENPK	centromere protein K	**-1,240**	3,98E-03	8,858	6,976
BUB1B	BUB1 mitotic checkpoint serine/threonine kinase B	**-1,240**	4,20E-03	10,854	8,418
RELT	RELT, TNF receptor	**-1,240**	5,28E-03	9,205	7,099
E2F1	E2F transcription factor 1	**-1,240**	5,75E-03	26,052	19,787
ADSL	adenylosuccinate lyase	**-1,240**	5,84E-03	6,346	4,877
FAM111B	family with sequence similarity 111 member B	**-1,240**	7,94E-03	14,941	11,477
TROAP	trophinin associated protein	**-1,240**	8,14E-03	6,399	4,908
NCAPH	non-SMC condensin I complex subunit H	**-1,240**	9,15E-03	7,415	5,687
ALDOC	aldolase, fructose-bisphosphate C	**-1,240**	1,11E-02	72,100	55,621
FGF1	fibroblast growth factor 1	**-1,240**	1,23E-02	133,358	101,064
PVR	poliovirus receptor	**-1,240**	1,48E-02	135,431	104,660
ABCC1	ATP binding cassette subfamily C member 1	**-1,240**	3,19E-02	3,053	2,180
EBP	emopamil binding protein (sterol isomerase)	**-1,240**	3,28E-02	14,655	8,244
MTFR2	mitochondrial fission regulator 2	**-1,240**	3,38E-02	2,307	1,638
HRCT1	histidine rich carboxyl terminus 1	**-1,240**	3,47E-02	33,649	23,769
CIDEA	cell death-inducing DFFA-like effector a	**-1,240**	3,57E-02	2,528	1,812
NUDT8	nudix hydrolase 8	**-1,240**	3,66E-02	4,122	2,867
PCNA	proliferating cell nuclear antigen	**-1,248**	4,68E-05	95,881	74,127
EXOSC8	exosome component 8	**-1,248**	6,31E-05	21,995	17,232
CYCS	cytochrome c, somatic	**-1,248**	1,23E-04	67,780	52,892
PTS	6-pyruvoyltetrahydropterin synthase	**-1,248**	1,72E-04	22,958	18,075
TPM1	tropomyosin 1 (alpha)	**-1,248**	2,05E-04	117,155	91,095
NEGR1	neuronal growth regulator 1	**-1,248**	2,85E-04	45,876	36,004

ADAM12	ADAM metallopeptidase domain 12	**-1,248**	6,08E-04	51,321	39,725
POLA2	DNA polymerase alpha 2, accessory subunit	**-1,248**	6,25E-04	17,429	13,414
SPAG5	sperm associated antigen 5	**-1,248**	1,15E-03	21,890	17,039
ADAMTS5	ADAM metallopeptidase with thrombospondin type 1 motif 5	**-1,248**	1,23E-03	61,870	48,711
CCNB1	cyclin B1	**-1,248**	1,26E-03	28,819	22,193
C4orf46	chromosome 4 open reading frame 46	**-1,248**	1,66E-03	7,940	6,103
LOXL1	lysyl oxidase like 1	**-1,248**	1,71E-03	56,535	43,311
FLVCR1	feline leukemia virus subgroup C cellular receptor 1	**-1,248**	1,82E-03	10,417	8,029
GPR176	G protein-coupled receptor 176	**-1,248**	3,08E-03	16,631	12,739
CREB3L1	cAMP responsive element binding protein 3 like 1	**-1,248**	3,21E-03	20,618	15,875
NDC80	NDC80, kinetochore complex component	**-1,248**	3,46E-03	10,162	7,688
ACTB	actin beta	**-1,248**	3,97E-03	1980,245	1507,217
INAFM2	InaF motif containing 2	**-1,248**	4,61E-03	8,692	6,670
FOSL1	FOS like 1, AP-1 transcription factor subunit	**-1,248**	8,08E-03	123,955	93,414
ENC1	ectodermal-neural cortex 1	**-1,248**	8,36E-03	8,291	6,309
LOXL3	lysyl oxidase like 3	**-1,248**	8,50E-03	659,492	513,139
PRR5L	proline rich 5 like	**-1,248**	1,03E-02	4,794	3,642
FADS2	fatty acid desaturase 2	**-1,248**	1,09E-02	108,646	42,986
TK1	thymidine kinase 1	**-1,248**	1,48E-02	43,579	32,714
PDSS1	decaprenyl diphosphate synthase subunit 1	**-1,248**	1,80E-02	3,855	2,803
AMOTL2	angiomotin like 2	**-1,248**	2,03E-02	99,776	72,363
HOXC8	homeobox C8	**-1,248**	2,38E-02	3,019	2,162
RTN4R	reticulon 4 receptor	**-1,248**	3,13E-02	2,368	1,631
NGF	nerve growth factor	**-1,248**	3,19E-02	12,082	8,493
RACGAP1	Rac GTPase activating protein 1	**-1,257**	8,66E-05	48,453	37,514
LMNB2	lamin B2	**-1,257**	8,66E-05	112,546	86,482
SKA2	spindle and kinetochore associated complex subunit 2	**-1,257**	2,48E-04	25,723	19,857
MCM5	minichromosome maintenance complex component 5	**-1,257**	3,91E-04	74,006	56,809
RASSF1	Ras association domain family member 1	**-1,257**	4,04E-04	49,013	37,296
RBBP8	RB binding protein 8, endonuclease	**-1,257**	4,63E-04	23,410	18,001
RMI1	RecQ mediated genome instability 1	**-1,257**	5,29E-04	12,114	9,406
PIMREG	PICALM interacting mitotic regulator	**-1,257**	7,49E-04	18,482	14,221
LRR1	leucine rich repeat protein 1	**-1,257**	1,25E-03	9,313	7,058

IGFBP1	insulin like growth factor binding protein 1	**-1,257**	1,63E-03	17,912	13,425
FANCD2	Fanconi anemia complementation group D2	**-1,257**	1,71E-03	10,654	8,029
CCNB2	cyclin B2	**-1,257**	2,23E-03	11,761	9,012
GEN1	GEN1, Holliday junction 5' flap endonuclease	**-1,257**	2,65E-03	7,714	5,836
BARD1	BRCA1 associated RING domain 1	**-1,257**	3,23E-03	11,409	8,376
STK17B	serine/threonine kinase 17b	**-1,257**	3,76E-03	8,941	6,651
COQ2	coenzyme Q2, polyprenyltransferase	**-1,257**	6,81E-03	7,857	5,888
CDA	cytidine deaminase	**-1,257**	1,02E-02	6,161	4,813
CENPI	centromere protein I	**-1,257**	1,05E-02	5,172	3,793
PLPP1	phospholipid phosphatase 1	**-1,257**	1,34E-02	58,312	42,365
ADCY7	adenylate cyclase 7	**-1,257**	1,97E-02	19,455	13,876
PDCD1LG2	programmed cell death 1 ligand 2	**-1,257**	2,26E-02	2,808	1,995
AJUBA	ajuba LIM protein	**-1,257**	2,29E-02	41,658	29,255
SLC8A1	solute carrier family 8 member A1	**-1,257**	2,61E-02	39,718	27,031
TUBA1C	tubulin alpha 1c	**-1,266**	2,43E-05	195,166	148,802
TUBA1B	tubulin alpha 1b	**-1,266**	8,58E-05	588,351	448,398
NUSAP1	nucleolar and spindle associated protein 1	**-1,266**	9,34E-05	33,955	26,428
RDH11	retinol dehydrogenase 11 (all-trans/9-cis/11-cis)	**-1,266**	2,78E-04	62,378	48,012
CDK1	cyclin dependent kinase 1	**-1,266**	3,41E-04	27,335	21,003
MMS22L	MMS22 like, DNA repair protein	**-1,266**	7,89E-04	11,313	8,490
RFC3	replication factor C subunit 3	**-1,266**	8,53E-04	14,116	10,547
CYTOR	cytoskeleton regulator RNA	**-1,266**	8,90E-04	18,063	13,863
SLC25A22	solute carrier family 25 member 22	**-1,266**	9,59E-04	29,598	22,305
AURKA	aurora kinase A	**-1,266**	2,51E-03	10,071	7,494
MYL9	myosin light chain 9	**-1,266**	2,74E-03	23,890	17,653
SKA3	spindle and kinetochore associated complex subunit 3	**-1,266**	2,84E-03	9,430	7,023
DSCC1	DNA replication and sister chromatid cohesion 1	**-1,266**	3,23E-03	6,674	5,016
TONSL	tonsoku like, DNA repair protein	**-1,266**	3,78E-03	6,887	5,121
NKD1	naked cuticle homolog 1	**-1,266**	3,86E-03	12,302	9,219
FANCA	Fanconi anemia complementation group A	**-1,266**	6,15E-03	9,400	7,061
LSS	lanosterol synthase	**-1,266**	7,79E-03	10,366	7,559
NSDHL	NAD(P) dependent steroid dehydrogenase-like	**-1,266**	2,09E-02	20,042	13,740
PKP1	plakophilin 1	**-1,266**	2,10E-02	2,764	1,581

LIF	LIF, interleukin 6 family cytokine	**-1,266**	2,31E-02	25,450	16,960
CKB	creatine kinase B	**-1,275**	2,56E-05	30,734	23,020
CKAP2	cytoskeleton associated protein 2	**-1,275**	4,68E-05	36,730	28,425
KPNA2	karyopherin subunit alpha 2	**-1,275**	8,08E-05	40,288	30,732
MCM4	minichromosome maintenance complex component 4	**-1,275**	1,29E-04	66,607	50,620
GTSE1	G2 and S-phase expressed 1	**-1,275**	2,01E-04	14,138	10,635
KIF20A	kinesin family member 20A	**-1,275**	2,28E-04	23,288	18,356
KNL1	kinetochore scaffold 1	**-1,275**	3,44E-04	15,586	11,813
SLC7A6	solute carrier family 7 member 6	**-1,275**	4,35E-04	28,813	21,777
SIX2	SIX homeobox 2	**-1,275**	6,08E-04	29,858	22,093
DONSON	downstream neighbor of SON	**-1,275**	6,70E-04	11,702	8,716
NUF2	NUF2, NDC80 kinetochore complex component	**-1,275**	7,87E-04	9,275	7,005
CKAP2L	cytoskeleton associated protein 2 like	**-1,275**	8,65E-04	18,415	13,868
BUB1	BUB1 mitotic checkpoint serine/threonine kinase	**-1,275**	9,09E-04	22,226	17,150
RTL3	retrotransposon Gag like 3	**-1,275**	3,87E-03	14,157	10,313
CENPM	centromere protein M	**-1,275**	4,25E-03	6,512	4,798
MYEOV	myeloma overexpressed	**-1,275**	4,92E-03	5,003	3,583
ATP1B1	ATPase Na+/K+ transporting subunit beta 1	**-1,275**	5,06E-03	259,774	188,467
HMGCS1	3-hydroxy-3-methylglutaryl-CoA synthase 1	**-1,275**	6,91E-03	95,727	38,108
LDLR	low density lipoprotein receptor	**-1,275**	7,48E-03	82,104	33,372
MSMO1	methylsterol monooxygenase 1	**-1,275**	7,79E-03	99,504	41,653
ACAT2	acetyl-CoA acetyltransferase 2	**-1,275**	8,02E-03	58,206	24,146
H19	H19, imprinted maternally expressed transcript (non-protein codi	**-1,275**	8,08E-03	149,200	104,603
CDC42EP2	CDC42 effector protein 2	**-1,275**	1,21E-02	2,985	2,085
1.maalis	membrane associated ring-CH-type finger 1	**-1,275**	1,83E-02	2,842	2,773
DHFR	dihydrofolate reductase	**-1,283**	7,19E-05	18,424	13,818
HMMR	hyaluronan mediated motility receptor	**-1,283**	9,19E-05	21,119	16,208
ZWILCH	zwilch kinetochore protein	**-1,283**	1,14E-04	26,364	19,890
RRAS2	related RAS viral (r-ras) oncogene homolog 2	**-1,283**	1,29E-04	23,891	17,807
WNT5B	Wnt family member 5B	**-1,283**	2,18E-04	20,836	15,790
KIF4A	kinesin family member 4A	**-1,283**	2,48E-04	13,600	10,081
PLK1	polo like kinase 1	**-1,283**	2,82E-04	13,966	10,483
NME1	NME/NM23 nucleoside diphosphate kinase 1	**-1,283**	4,94E-04	30,366	22,958

ASF1B	anti-silencing function 1B histone chaperone	**-1,283**	5,03E-04	14,477	10,618
WDR76	WD repeat domain 76	**-1,283**	6,08E-04	19,237	14,219
SNAPC1	small nuclear RNA activating complex polypeptide 1	**-1,283**	7,44E-04	40,864	30,224
WDR62	WD repeat domain 62	**-1,283**	7,49E-04	12,269	9,160
WDHD1	WD repeat and HMG-box DNA binding protein 1	**-1,283**	9,86E-04	13,351	9,979
FANCI	Fanconi anemia complementation group I	**-1,283**	1,11E-03	28,013	20,744
FBXO5	F-box protein 5	**-1,283**	1,36E-03	11,889	8,728
POC1A	POC1 centriolar protein A	**-1,283**	1,48E-03	10,053	7,314
TGFBI	transforming growth factor beta induced	**-1,283**	1,82E-03	177,311	132,556
CENPW	centromere protein W	**-1,283**	2,40E-03	6,577	4,682
RCAN1	regulator of calcineurin 1	**-1,283**	3,40E-03	152,376	108,415
EXO1	exonuclease 1	**-1,283**	5,20E-03	6,104	4,382
TYMS	thymidylate synthetase	**-1,283**	5,46E-03	80,911	57,483
SREBF2	sterol regulatory element binding transcription factor 2	**-1,283**	6,46E-03	168,705	119,768
NXPH3	neurexophilin 3	**-1,283**	7,67E-03	7,141	5,005
LMO7	LIM domain 7	**-1,283**	7,86E-03	61,923	43,789
SQLE	squalene epoxidase	**-1,283**	8,84E-03	87,564	41,633
COL1A1	collagen type I alpha 1 chain	**-1,283**	1,18E-02	70,589	45,844
CPA4	carboxypeptidase A4	**-1,283**	1,39E-02	41,583	27,917
FADS1	fatty acid desaturase 1	**-1,283**	1,43E-02	67,154	38,268
ADAMTS1	ADAM metallopeptidase with thrombospondin type 1 motif 1	**-1,283**	1,50E-02	193,339	112,696
MVK	mevalonate kinase	**-1,283**	1,58E-02	18,263	11,493
CENPN	centromere protein N	**-1,292**	1,41E-04	15,462	11,402
CDK6	cyclin dependent kinase 6	**-1,292**	1,72E-04	26,879	20,136
TPX2	TPX2, microtubule nucleation factor	**-1,292**	2,94E-04	55,300	41,612
RPL22L1	ribosomal protein L22 like 1	**-1,292**	3,91E-04	29,137	21,655
GINS4	GINS complex subunit 4	**-1,292**	4,30E-04	11,063	8,038
PRIM1	primase (DNA) subunit 1	**-1,292**	6,08E-04	8,563	6,295
PTGS2	prostaglandin-endoperoxide synthase 2	**-1,292**	6,28E-04	33,407	25,202
UBE2C	ubiquitin conjugating enzyme E2 C	**-1,292**	1,16E-03	13,301	9,547
CLCF1	cardiotrophin like cytokine factor 1	**-1,292**	1,22E-03	19,515	14,306
KIF2C	kinesin family member 2C	**-1,292**	2,05E-03	10,186	7,417
RAD54L	RAD54 like (S. cerevisiae)	**-1,292**	4,11E-03	6,335	4,469

DOK1	docking protein 1	**-1,292**	5,74E-03	256,642	183,685
STC2	stanniocalcin 2	**-1,292**	5,84E-03	111,956	80,790
INSIG1	insulin induced gene 1	**-1,292**	6,74E-03	128,036	56,512
DHCR7	7-dehydrocholesterol reductase	**-1,292**	6,86E-03	79,824	38,097
MMAB	methylmalonic aciduria (cobalamin deficiency) cblB type	**-1,292**	9,64E-03	26,933	18,101
HMGCR	3-hydroxy-3-methylglutaryl-CoA reductase	**-1,292**	9,68E-03	85,965	45,026
TMEM97	transmembrane protein 97	**-1,292**	1,16E-02	14,819	8,143
CYP1B1	cytochrome P450 family 1 subfamily B member 1	**-1,292**	1,17E-02	445,910	295,886
TOP2A	topoisomerase (DNA) II alpha	**-1,301**	1,20E-05	90,400	69,042
KIF11	kinesin family member 11	**-1,301**	8,66E-05	26,868	19,775
MRGPRF	MAS related GPR family member F	**-1,301**	1,02E-04	33,060	24,391
MAD2L1	mitotic arrest deficient 2 like 1	**-1,301**	2,18E-04	12,505	9,314
FEN1	flap structure-specific endonuclease 1	**-1,301**	3,97E-04	21,438	15,431
CDCA8	cell division cycle associated 8	**-1,301**	7,53E-04	14,216	10,336
STIL	SCL/TAL1 interrupting locus	**-1,301**	8,02E-04	9,048	6,491
PGF	placental growth factor	**-1,301**	3,87E-03	7,107	5,037
SCD	stearoyl-CoA desaturase	**-1,301**	5,30E-03	246,135	106,668
SC5D	sterol-C5-desaturase	**-1,301**	5,50E-03	61,000	41,543
GDF5	growth differentiation factor 5	**-1,301**	9,03E-03	50,244	30,358
FSTL3	follistatin like 3	**-1,301**	9,11E-03	13,405	8,865
SPAAR	small regulatory polypeptide of amino acid response	**-1,301**	9,44E-03	4,102	2,618
MYBL1	MYB proto-oncogene like 1	**-1,301**	9,49E-03	51,927	32,096
FASN	fatty acid synthase	**-1,301**	9,55E-03	122,275	70,750
CDC20	cell division cycle 20	**-1,310**	1,29E-05	29,706	22,243
NCAPG	non-SMC condensin I complex subunit G	**-1,310**	6,81E-05	18,835	13,782
TACC3	transforming acidic coiled-coil containing protein 3	**-1,310**	7,82E-05	40,052	29,413
PSMC3IP	PSMC3 interacting protein	**-1,310**	8,58E-05	15,430	11,292
RECQL4	RecQ like helicase 4	**-1,310**	2,23E-04	16,531	12,080
ERCC2	ERCC excision repair 2, TFIIH core complex helicase subunit	**-1,310**	4,63E-04	16,707	12,059
TINAGL1	tubulointerstitial nephritis antigen like 1	**-1,310**	6,13E-04	16,776	12,158
NDP	NDP, norrin cystine knot growth factor	**-1,310**	7,53E-04	8,585	6,104
ORC1	origin recognition complex subunit 1	**-1,310**	1,01E-03	6,655	4,669
KLF7	Kruppel like factor 7	**-1,310**	1,40E-03	10,474	7,273

TRIP13	thyroid hormone receptor interactor 13	**-1,310**	1,56E-03	14,371	10,172
PCLAF	PCNA clamp associated factor	**-1,310**	1,60E-03	5,510	3,886
SLC38A5	solute carrier family 38 member 5	**-1,310**	2,36E-03	10,876	7,487
IDI1	isopentenyl-diphosphate delta isomerase 1	**-1,310**	5,98E-03	74,035	35,477
CDC6	cell division cycle 6	**-1,310**	7,71E-03	22,592	13,838
ECT2	epithelial cell transforming 2	**-1,320**	1,77E-05	24,452	17,911
ANLN	anillin actin binding protein	**-1,320**	4,60E-05	78,591	59,043
CENPU	centromere protein U	**-1,320**	1,62E-04	18,486	13,135
CTPS1	CTP synthase 1	**-1,320**	5,09E-04	19,381	13,610
PLK4	polo like kinase 4	**-1,320**	8,60E-04	8,291	5,750
ST6GALNAC5	ST6 N-acetylgalactosaminide alpha-2,6-sialyltransferase 5	**-1,320**	1,09E-03	7,182	5,089
HHIP	hedgehog interacting protein	**-1,320**	1,51E-03	9,312	6,179
FHL2	four and a half LIM domains 2	**-1,320**	1,71E-03	150,118	103,735
RHOB	ras homolog family member B	**-1,320**	4,68E-03	72,966	46,194
XRCC2	X-ray repair cross complementing 2	**-1,320**	5,06E-03	2,927	1,900
DHCR24	24-dehydrocholesterol reductase	**-1,320**	5,65E-03	190,938	96,029
UHRF1	ubiquitin like with PHD and ring finger domains 1	**-1,320**	5,98E-03	22,449	14,151
FDPS	farnesyl diphosphate synthase	**-1,320**	6,08E-03	89,925	45,306
ZNF367	zinc finger protein 367	**-1,329**	1,29E-05	77,244	56,023
UBE2S	ubiquitin conjugating enzyme E2 S	**-1,329**	1,89E-05	23,533	16,766
BIRC5	baculoviral IAP repeat containing 5	**-1,329**	5,94E-05	28,075	20,231
CHEK1	checkpoint kinase 1	**-1,329**	1,13E-04	13,064	9,222
RAD51AP1	RAD51 associated protein 1	**-1,329**	4,94E-04	6,961	4,912
TAGLN	transgelin	**-1,329**	8,19E-04	16,698	10,993
TMPO-AS1	TMPO antisense RNA 1	**-1,329**	1,80E-03	3,710	2,545
IGFBP4	insulin like growth factor binding protein 4	**-1,329**	3,15E-03	75,299	52,380
MVD	mevalonate diphosphate decarboxylase	**-1,329**	4,28E-03	50,719	23,715
PLAU	plasminogen activator, urokinase	**-1,329**	4,77E-03	13,397	8,191
ELN	elastin	**-1,329**	4,98E-03	59,018	31,875
SHISA3	shisa family member 3	**-1,338**	3,54E-05	32,413	22,596
EZH2	enhancer of zeste 2 polycomb repressive complex 2 subunit	**-1,338**	7,19E-05	13,084	9,197
PKMYT1	protein kinase, membrane associated tyrosine/threonine 1	**-1,338**	2,18E-04	17,861	12,499
GINS2	GINS complex subunit 2	**-1,338**	2,84E-04	14,821	10,265

SFN	stratifin	**-1,338**	4,67E-04	16,002	10,743
PLK2	polo like kinase 2	**-1,338**	5,49E-04	12,088	8,442
ESCO2	establishment of sister chromatid cohesion N-acetyltransferase 2	**-1,338**	7,11E-04	7,812	5,399
GINS1	GINS complex subunit 1	**-1,338**	8,02E-04	6,921	4,600
CLSPN	claspin	**-1,338**	8,47E-04	13,801	9,430
SLC4A4	solute carrier family 4 member 4	**-1,338**	2,84E-03	4,361	2,842
SEMA7A	semaphorin 7A (John Milton Hagen blood group)	**-1,338**	4,57E-03	25,163	14,092
PAQR4	progestin and adipoQ receptor family member 4	**-1,347**	2,54E-06	25,229	18,106
SHCBP1	SHC binding and spindle associated 1	**-1,347**	1,23E-04	27,202	19,222
CDC25A	cell division cycle 25A	**-1,347**	8,65E-04	8,542	5,726
CHRNA5	cholinergic receptor nicotinic alpha 5 subunit	**-1,347**	1,84E-03	2,670	1,710
EVA1A	eva-1 homolog A, regulator of programmed cell death	**-1,347**	1,98E-03	3,841	2,458
HYAL3	hyaluronoglucosaminidase 3	**-1,347**	2,19E-03	2,920	1,857
P2RY6	pyrimidinergic receptor P2Y6	**-1,347**	2,35E-03	19,886	12,961
DEPDC1B	DEP domain containing 1B	**-1,347**	2,47E-03	2,669	1,685
STARD4	StAR related lipid transfer domain containing 4	**-1,347**	3,16E-03	17,267	8,413
PBK	PDZ binding kinase	**-1,357**	3,34E-05	24,275	17,416
HJURP	Holliday junction recognition protein	**-1,357**	3,48E-05	18,281	13,002
KIF23	kinesin family member 23	**-1,357**	3,63E-05	27,608	19,681
AURKB	aurora kinase B	**-1,357**	8,20E-05	10,398	7,153
CAP2	CAP, adenylate cyclase-associated protein, 2 (yeast)	**-1,357**	6,64E-04	12,991	8,706
NANOS1	nanos C2HC-type zinc finger 1	**-1,357**	1,11E-03	9,152	6,657
ATAD2	ATPase family, AAA domain containing 2	**-1,366**	1,20E-05	42,482	29,653
DEPDC1	DEP domain containing 1	**-1,366**	3,34E-05	10,575	7,404
CCNE2	cyclin E2	**-1,366**	4,96E-04	6,437	4,192
CDT1	chromatin licensing and DNA replication factor 1	**-1,376**	4,42E-05	17,190	11,542
ORC6	origin recognition complex subunit 6	**-1,376**	2,43E-04	7,781	5,072
SNAI3-AS1	SNAI3 antisense RNA 1	**-1,376**	6,28E-04	4,588	2,941
ESM1	endothelial cell specific molecule 1	**-1,376**	1,06E-03	4,187	2,392
THY1	Thy-1 cell surface antigen	**-1,385**	3,05E-06	82,852	56,722
LMNB1	lamin B1	**-1,385**	4,09E-06	18,004	12,251
CEP55	centrosomal protein 55	**-1,385**	8,51E-06	24,877	16,897
MELK	maternal embryonic leucine zipper kinase	**-1,385**	8,15E-05	16,589	11,275

AP1S3	adaptor related protein complex 1 sigma 3 subunit	**-1,385**	7,47E-04	4,167	2,584
ZWINT	ZW10 interacting kinetochore protein	**-1,395**	2,54E-06	24,917	16,931
DIAPH3	diaphanous related formin 3	**-1,395**	2,54E-06	22,502	15,569
CCNA2	cyclin A2	**-1,395**	4,68E-06	23,744	16,202
ANGPTL4	angiopoietin like 4	**-1,395**	1,24E-05	37,483	25,982
CDC45	cell division cycle 45	**-1,395**	1,65E-04	11,677	7,470
MEST	mesoderm specific transcript	**-1,395**	1,01E-03	3,767	2,138
FHOD3	formin homology 2 domain containing 3	**-1,404**	8,51E-04	3,423	1,872
UBE2T	ubiquitin conjugating enzyme E2 T	**-1,414**	1,20E-05	12,320	8,148
ARSI	arylsulfatase family member I	**-1,414**	9,34E-05	9,519	5,906
CDCA5	cell division cycle associated 5	**-1,424**	8,51E-08	22,204	14,853
MCM10	minichromosome maintenance 10 replication initiation factor	**-1,454**	2,27E-05	8,213	4,968
HSD17B7	hydroxysteroid 17-beta dehydrogenase 7	**-1,454**	6,53E-05	6,538	3,872
CCL2	C-C motif chemokine ligand 2	**-1,454**	1,80E-04	6,239	3,326
RRM2	ribonucleotide reductase regulatory subunit M2	**-1,464**	1,74E-07	37,810	23,959
HELLS	helicase, lymphoid-specific	**-1,474**	4,09E-06	13,072	7,875
