# Supplementary material for: Regulation of gene expression by MF63, a selective inhibitor of microsomal PGE synthase 1 (mPGES1) in human osteoarthritic chondrocytes
Source: Br J Pharmacol. 2020 Aug 10;177(18):4134–46. doi: 10.1111/bph.15142 (PMC7443472; doi:10.1111/bph.15142)
Supplement: Supplementary file 3 — Table S3. Supporting information [file BPH-177-4134-s003.pdf]

Gene	Name	Mean (ktrl)	Mean (IL1)	FC	Adj. p
IL1B	interleukin 1 beta	0,1	560,9	**3477,01**	0
CXCL8	C-X-C motif chemokine ligand 8	6,4	21281,4	**2384,14**	0
CXCL1	C-X-C motif chemokine ligand 1	0,6	1778,1	**2316,90**	0
CXCL6	C-X-C motif chemokine ligand 6	0,1	422,2	**1976,80**	0
IL6	interleukin 6	0,5	938,0	**1505,18**	0
CXCL5	C-X-C motif chemokine ligand 5	0,2	278,7	**1363,99**	0
CXCL3	C-X-C motif chemokine ligand 3	0,1	208,4	**1168,27**	0
IL36RN	interleukin 36 receptor antagonist	0,1	116,8	**1118,95**	0
CXCL2	C-X-C motif chemokine ligand 2	0,2	271,4	**1067,69**	0
FCAMR	Fc fragment of IgA and IgM receptor	0,0	36,2	**895,64**	0
CCL20	C-C motif chemokine ligand 20	6,9	6645,3	**751,42**	0
IL36G	interleukin 36, gamma	0,0	29,0	**697,96**	0
SGPP2	sphingosine-1-phosphate phosphatase 2	0,1	49,2	**544,13**	0
SELE	selectin E	0,1	40,5	**479,65**	0
SERPINB7	serpin family B member 7	0,2	129,3	**477,90**	0
CSF3	colony stimulating factor 3	0,0	19,4	**370,43**	0
EREG	epiregulin	0,3	199,1	**333,93**	0
VNN1	vanin 1	0,3	95,0	**312,17**	0
CSF2	colony stimulating factor 2	0,0	5,1	**255,49**	0
ESM1	endothelial cell specific molecule 1	4,2	1175,8	**232,98**	0
VNN3	vanin 3	0,0	10,4	**210,51**	0
C15orf48	chromosome 15 open reading frame 48	1,5	357,2	**196,48**	0
UNC13A	unc-13 homolog A	0,0	9,1	**172,82**	0
IL1A	interleukin 1 alpha	0,0	3,2	**169,06**	0
MSC	musculin	0,3	43,6	**148,09**	0
CXCL10	C-X-C motif chemokine ligand 10	0,0	9,4	**136,76**	0
TNFSF18	TNF superfamily member 18	0,1	11,1	**134,48**	0
MRGPRX3	MAS related GPR family member X3	0,0	5,0	**124,59**	0
KCNMB1	potassium calcium-activated channel subfamily M regulatory beta sub	0,1	12,5	**120,91**	0
LCE3E	late cornified envelope 3E	0,0	7,8	**115,41**	0
GPR84	G protein-coupled receptor 84	0,1	24,1	**111,31**	0
NOD2	nucleotide binding oligomerization domain containing 2	0,5	68,8	**109,37**	0

IL1RN	interleukin 1 receptor antagonist	0,1	23,3	**108,46**	0
ICAM4	intercellular adhesion molecule 4 (Landsteiner-Wiener blood group)	0,2	46,5	**104,05**	0
BIRC3	baculoviral IAP repeat containing 3	7,5	1063,9	**103,53**	0
PI3	peptidase inhibitor 3	0,0	11,1	**98,71**	0
NOS2	nitric oxide synthase 2	9,7	991,5	**91,05**	0
GPR37L1	G protein-coupled receptor 37 like 1	0,2	22,9	**89,16**	0
ADORA2A	adenosine A2a receptor	0,0	4,6	**84,44**	0
ELOVL7	ELOVL fatty acid elongase 7	0,3	34,3	**82,95**	0
MIR3142HG	MIR3142 host gene	0,0	4,5	**81,46**	0
AREG	amphiregulin	0,0	2,3	**75,95**	0
COL22A1	collagen type XXII alpha 1 chain	0,0	1,6	**75,26**	0
XIRP1	xin actin binding repeat containing 1	0,0	5,6	**74,17**	0
SPRR2E	small proline rich protein 2E	0,0	3,2	**73,49**	0
ADORA2BP1	adenosine A2b receptor pseudogene 1	0,0	3,7	**70,09**	0
POU2F2	POU class 2 homeobox 2	1,0	102,1	**69,47**	0
AIRE	autoimmune regulator	0,0	4,1	**69,39**	0
PTX3	pentraxin 3	4,9	457,0	**64,07**	0
PILRA	paired immunoglobin like type 2 receptor alpha	0,4	33,2	**62,73**	0
NEFM	neurofilament medium	0,2	13,2	**59,73**	0
HMGN2P46	high mobility group nucleosomal binding domain 2 pseudogene 46	0,2	15,5	**55,89**	0
SAA2	serum amyloid A2	0,4	26,5	**55,28**	0
LCE3D	late cornified envelope 3D	0,0	1,6	**55,18**	0
ICOSLG	inducible T-cell costimulator ligand	0,1	10,0	**54,09**	0
SERPINB2	serpin family B member 2	0,0	6,7	**53,95**	0
DHRS2	dehydrogenase/reductase 2	0,3	27,3	**53,67**	0
NCCRP1	non-specific cytotoxic cell receptor protein 1 homolog (zebrafish)	0,0	3,1	**53,14**	0
BCL2A1	BCL2 related protein A1	1,6	113,8	**52,33**	0
KRT16	keratin 16	7,6	544,4	**51,91**	0
SAA1	serum amyloid A1	3,4	134,8	**49,59**	0
LINC02005	long intergenic non-protein coding RNA 2005	0,0	1,4	**48,48**	0
EBI3	Epstein-Barr virus induced 3	0,0	1,4	**46,72**	0
IDO1	indoleamine 2,3-dioxygenase 1	0,0	1,3	**42,44**	0
IL36B	interleukin 36, beta	0,4	23,2	**41,09**	0

CD300E	CD300e molecule	0,1	6,6	**40,50**	0
LRRC55	leucine rich repeat containing 55	0,0	0,8	**40,05**	0
IL24	interleukin 24	0,0	2,3	**38,99**	0
TNFRSF9	TNF receptor superfamily member 9	0,3	18,9	**38,94**	0
HSD11B1	hydroxysteroid 11-beta dehydrogenase 1	11,1	534,1	**38,79**	0
NPPC	natriuretic peptide C	0,0	2,8	**38,62**	0
PGLYRP2	peptidoglycan recognition protein 2	0,1	4,9	**37,05**	0
C4orf26	chromosome 4 open reading frame 26			**35,47**	0
G0S2	G0/G1 switch 2	24,7	1102,7	**33,60**	0
LUCAT1	lung cancer associated transcript 1 (non-protein coding)	0,2	10,7	**32,83**	0
CCL2	C-C motif chemokine ligand 2	6,2	276,0	**32,81**	0
TNFAIP6	TNF alpha induced protein 6	17,4	945,8	**32,63**	0
IL32	interleukin 32	0,2	8,1	**32,48**	0
CPXM1	carboxypeptidase X, M14 family member 1	0,0	3,0	**32,24**	2,00E-06
LIF	LIF, interleukin 6 family cytokine	25,5	978,5	**31,81**	0
RSPO3	R-spondin 3	1,6	81,0	**31,78**	0
TMEM132A	transmembrane protein 132A	2,0	73,3	**31,59**	0
TNFRSF1B	TNF receptor superfamily member 1B	4,4	193,6	**31,18**	0
SLC28A3	solute carrier family 28 member 3	0,2	8,6	**28,94**	0
CYP24A1	cytochrome P450 family 24 subfamily A member 1	0,1	2,6	**28,62**	0
C3	complement C3	9,5	211,3	**27,50**	0
SLC7A2	solute carrier family 7 member 2	209,5	8205,0	**27,17**	0
TNFAIP2	TNF alpha induced protein 2	23,5	848,0	**26,34**	0
IGF1	insulin like growth factor 1	0,2	7,4	**26,14**	0
IRAK2	interleukin 1 receptor associated kinase 2	4,8	162,8	**25,42**	0
TNFAIP3	TNF alpha induced protein 3	5,3	171,4	**25,32**	0
CCL7	C-C motif chemokine ligand 7	0,1	5,3	**25,00**	0
TREML3P	triggering receptor expressed on myeloid cells like 3, pseudogene	0,1	2,8	**24,17**	0
RASGRP1	RAS guanyl releasing protein 1	0,2	6,3	**23,68**	1,00E-06
ICAM1	intercellular adhesion molecule 1	139,4	4644,2	**23,30**	0
MAFA	MAF bZIP transcription factor A	0,1	3,0	**22,22**	0
HIST1H2BG	histone cluster 1 H2B family member g	0,1	2,3	**21,30**	0
RAET1L	retinoic acid early transcript 1L	0,2	4,8	**21,29**	0

C1QTNF1	C1q and TNF related 1	6,2	189,7	**20,77**	0
IVL	involucrin	0,1	1,9	**20,63**	0
SLC18B1	solute carrier family 18 member B1	0,8	22,0	**19,90**	0
IL23A	interleukin 23 subunit alpha	0,4	15,0	**19,82**	0
PDZK1IP1	PDZK1 interacting protein 1	1,6	48,8	**19,80**	0
IL17C	interleukin 17C	0,0	1,9	**19,72**	0
CX3CL1	C-X3-C motif chemokine ligand 1	4,5	97,8	**19,65**	0
PLAT	plasminogen activator, tissue type	1,8	47,0	**19,06**	0
ZC3H12A	zinc finger CCCH-type containing 12A	6,3	168,8	**18,91**	0
AMIGO2	adhesion molecule with Ig like domain 2	3,1	79,5	**17,78**	0
ITGAX	integrin subunit alpha X	0,5	10,8	**17,71**	0
C3AR1	complement C3a receptor 1	0,1	3,5	**17,65**	0
MARCKS	myristoylated alanine rich protein kinase C substrate	11,0	256,5	**17,57**	0
SLAMF8	SLAM family member 8	0,2	4,3	**17,29**	0
SLIT2	slit guidance ligand 2	1,4	32,1	**17,21**	0
TRAF1	TNF receptor associated factor 1	12,9	291,0	**16,91**	0
AKR1B15	aldo-keto reductase family 1 member B15	0,0	0,6	**16,69**	2,00E-06
NFKBIA	NFKB inhibitor alpha	24,5	565,2	**16,68**	0
RND1	Rho family GTPase 1	1,7	39,2	**16,36**	0
KRT17	keratin 17	0,6	14,1	**16,29**	0
SLCO2B1	solute carrier organic anion transporter family member 2B1	0,1	1,4	**16,12**	0
DSCAM	DS cell adhesion molecule	0,1	0,8	**16,08**	3,00E-06
SOD2	superoxide dismutase 2	930,5	20577,6	**15,96**	0
TNFAIP8	TNF alpha induced protein 8	6,1	126,6	**15,90**	0
KDR	kinase insert domain receptor	0,0	1,0	**15,85**	0
CD274	CD274 molecule	1,4	28,4	**15,59**	0
MSC-AS1	MSC antisense RNA 1	0,4	9,6	**15,46**	0
CXCR4	C-X-C motif chemokine receptor 4	0,0	1,0	**15,45**	0
CD38	CD38 molecule	0,1	3,8	**15,39**	8,00E-06
ADORA2A-AS	ADORA2A antisense RNA 1	0,0	0,8	**15,17**	0
ARC	activity regulated cytoskeleton associated protein	2,8	65,5	**15,11**	0
GDNF	glial cell derived neurotrophic factor	0,2	3,0	**14,81**	0
LRRN3	leucine rich repeat neuronal 3	0,5	8,7	**14,78**	0

MT3	metallothionein 3	0,1	1,9	**14,72**	0
IL7R	interleukin 7 receptor	0,0	0,9	**14,57**	9,00E-06
SBSN	suprabasin	1,1	17,9	**14,24**	0
IL4I1	interleukin 4 induced 1	0,6	12,8	**14,12**	0
HIST2H2BE	histone cluster 2 H2B family member e	3,0	56,2	**14,04**	0
KYNU	kynureninase	0,5	9,2	**14,01**	0
TFPI2	tissue factor pathway inhibitor 2	18,3	343,9	**13,91**	0
WNT5A	Wnt family member 5A	2,5	44,7	**13,88**	0
TMEM200C	transmembrane protein 200C	0,1	1,0	**13,86**	6,00E-06
GRB7	growth factor receptor bound protein 7	0,1	2,2	**13,45**	0
CYP7A1	cytochrome P450 family 7 subfamily A member 1	0,0	0,9	**13,25**	2,80E-05
SLCO5A1	solute carrier organic anion transporter family member 5A1	0,1	1,7	**13,07**	1,00E-06
TLR2	toll like receptor 2	5,9	106,2	**12,96**	0
RELB	RELB proto-oncogene, NF-kB subunit	6,7	118,5	**12,94**	0
POU3F1	POU class 3 homeobox 1	0,1	1,9	**12,92**	6,00E-06
LAMB3	laminin subunit beta 3	235,6	3512,9	**12,77**	0
MFSD2A	major facilitator superfamily domain containing 2A	1,5	23,5	**12,57**	0
LCN2	lipocalin 2	101,7	1612,6	**12,44**	0
ABCA12	ATP binding cassette subfamily A member 12	0,2	2,5	**12,31**	0
VGF	VGF nerve growth factor inducible	0,6	10,1	**12,11**	0
MMP12	matrix metallopeptidase 12	0,1	2,1	**12,09**	0
FLG	filaggrin	0,1	1,3	**11,88**	0
CXCR3	C-X-C motif chemokine receptor 3	0,0	0,7	**11,73**	0
CTSS	cathepsin S	7,0	113,9	**11,67**	0
IRF4	interferon regulatory factor 4	1,4	17,8	**11,64**	0
KRT14	keratin 14	0,2	3,4	**11,59**	0
CFAP161	cilia and flagella associated protein 161	0,0	0,9	**11,57**	0
PCDH19	protocadherin 19	0,2	3,3	**11,55**	0
WWC1	WW and C2 domain containing 1	0,5	7,1	**11,52**	0
CD83	CD83 molecule	2,9	40,4	**11,47**	0
ZP3	zona pellucida glycoprotein 3	0,7	6,4	**11,40**	0
MARCH3	membrane associated ring-CH-type finger 3	3,0	42,5	**11,28**	0
ASCL2	achaete-scute family bHLH transcription factor 2	0,1	2,6	**11,18**	0

VNN2	vanin 2	1,0	14,0	**11,11**	0
MMP1	matrix metallopeptidase 1	699,8	9785,7	**10,98**	2,00E-06
PTGES2-AS1	PTGES2 antisense RNA 1 (head to head)	32,6	1,9	**10,98**	0
TREM1	triggering receptor expressed on myeloid cells 1	3,4	44,3	**10,96**	0
IL19	interleukin 19	0,0	0,7	**10,95**	0
ICAM5	intercellular adhesion molecule 5	0,3	4,4	**10,86**	0
ESPN	espin	0,1	2,3	**10,86**	0
MYH16	myosin heavy chain 16 pseudogene	0,2	1,9	**10,84**	0
MAST4	microtubule associated serine/threonine kinase family member 4	16,9	212,8	**10,77**	0
PTGS2	prostaglandin-endoperoxide synthase 2	33,4	565,0	**10,76**	0
STRA6	stimulated by retinoic acid 6	0,2	1,9	**10,69**	0
IER3	immediate early response 3	0,1	1,3	**10,52**	0
RND3	Rho family GTPase 3	53,5	673,3	**10,08**	0
CLEC2B	C-type lectin domain family 2 member B	0,2	2,9	**10,06**	0
BDKRB1	bradykinin receptor B1	2,5	31,8	**10,00**	0
SOX17	SRY-box 17	0,2	3,5	**9,91**	0
IL15RA	interleukin 15 receptor subunit alpha	3,0	34,6	**9,74**	0
LINC01909	long intergenic non-protein coding RNA 1909	0,1	1,4	**9,70**	0
SERPIND1	serpin family D member 1	0,6	7,0	**9,70**	0
HIST2H4A	histone cluster 2 H4 family member a	0,1	0,9	**9,62**	0
BAIAP3	BAI1 associated protein 3	0,3	4,1	**9,19**	0
OXTR	oxytocin receptor	0,2	2,6	**9,19**	0
STAT4	signal transducer and activator of transcription 4	0,1	2,5	**9,18**	4,20E-05
PIM2	Pim-2 proto-oncogene, serine/threonine kinase	4,3	48,7	**9,14**	0
FLJ31104	uncharacterized LOC441072		1,9	**9,11**	0
CSF1	colony stimulating factor 1	511,6	5413,4	**9,04**	0
S1PR1	sphingosine-1-phosphate receptor 1	0,7	8,2	**8,93**	0
TMEM178B	transmembrane protein 178B	0,1	1,7	**8,89**	7,00E-06
MTMR7	myotubularin related protein 7	1,5	16,2	**8,87**	0
CD82	CD82 molecule	52,7	557,4	**8,74**	0
KIAA1217	KIAA1217	1,5	18,6	**8,71**	0
SH2D1B	SH2 domain containing 1B	0,2	1,6	**8,70**	0
CFAP58-AS1	CFAP58 antisense RNA 1 (head to head)	0,3	2,0	**8,66**	0

DUSP5	dual specificity phosphatase 5	6,1	63,4	**8,64**	0
SCO2	SCO2, cytochrome c oxidase assembly protein	0,2	2,2	**8,59**	0
WTAPP1	Wilms tumor 1 associated protein pseudogene 1	2,6	34,4	**8,49**	0
MT1A	metallothionein 1A	4,1	51,2	**8,43**	0
LPL	lipoprotein lipase	0,4	4,7	**8,41**	0
SSTR2	somatostatin receptor 2	4,8	51,2	**8,32**	0
SLC13A3	solute carrier family 13 member 3	1,3	13,2	**8,29**	0
AKR1C2	aldo-keto reductase family 1 member C2	78,5	751,4	**8,28**	0
SMPD3	sphingomyelin phosphodiesterase 3	0,2	2,2	**8,22**	0
EPPK1	epiplakin 1	0,2	1,8	**8,18**	0
NKX3-1	NK3 homeobox 1	15,9	166,6	**8,18**	0
PAX8	paired box 8	2,6	17,5	**8,13**	0
RBM47	RNA binding motif protein 47	0,6	6,9	**8,09**	0
ELF3	E74 like ETS transcription factor 3	10,3	106,9	**8,07**	0
CHRNB2	cholinergic receptor nicotinic beta 2 subunit	0,1	0,7	**8,06**	0
RRAD	RRAD, Ras related glycolysis inhibitor and calcium channel regulator	1,0	13,1	**8,06**	0
APOL3	apolipoprotein L3	18,5	185,8	**8,02**	0
TNN	tenascin N	0,1	1,6	**7,96**	5,00E-06
DLGAP1-AS2	DLGAP1 antisense RNA 2	1,9	19,4	**7,92**	0
GBP1	guanylate binding protein 1	2,0	19,4	**7,86**	0
HMGA2	high mobility group AT-hook 2	2,0	20,6	**7,84**	0
IL12A	interleukin 12A	0,1	0,9	**7,84**	0
CH25H	cholesterol 25-hydroxylase	0,3	2,2	**7,81**	0
CLGN	calmegin	0,8	7,2	**7,74**	0
C6orf58	chromosome 6 open reading frame 58	0,1	1,8	**7,73**	4,00E-06
LINC02015	long intergenic non-protein coding RNA 2015	0,5	5,7	**7,63**	0
TYMP	thymidine phosphorylase	28,6	275,1	**7,57**	0
MXD1	MAX dimerization protein 1	22,2	205,2	**7,56**	0
MRPS24	mitochondrial ribosomal protein S24	0,7	7,4	**7,55**	0
GPR158	G protein-coupled receptor 158	0,1	1,2	**7,53**	6,00E-06
MME	membrane metalloendopeptidase	0,7	5,8	**7,52**	0
MMP3	matrix metallopeptidase 3	11316,9	109522,4	**7,50**	1,20E-05
FCMR	Fc fragment of IgM receptor	0,9	9,1	**7,49**	0

INA	internexin neuronal intermediate filament protein alpha	0,1	0,9	**7,48**	1,60E-05
IRF1	interferon regulatory factor 1	9,1	82,0	**7,46**	0
ITGAM	integrin subunit alpha M	0,4	3,5	**7,34**	0
HBEGF	heparin binding EGF like growth factor	7,5	65,6	**7,33**	0
SAT1	spermidine/spermine N1-acetyltransferase 1	183,3	1685,7	**7,32**	0
STC1	stanniocalcin 1	0,1	1,9	**7,26**	1,20E-05
ELF3-AS1	ELF3 antisense RNA 1	0,3	3,5	**7,25**	0
GCH1	GTP cyclohydrolase 1	30,2	280,1	**7,20**	0
MAFF	MAF bZIP transcription factor F	52,3	458,7	**7,18**	0
CYP7B1	cytochrome P450 family 7 subfamily B member 1	2,9	25,4	**7,15**	0
PRLR	prolactin receptor	0,3	1,9	**7,15**	2,00E-06
CFAP58	cilia and flagella associated protein 58	0,3	2,0	**7,13**	0
CDKN2B	cyclin dependent kinase inhibitor 2B	71,4	599,4	**7,09**	0
HAL	histidine ammonia-lyase	0,2	1,7	**7,09**	0
HAP1	huntingtin associated protein 1	0,1	1,4	**7,08**	0
LRP1B	LDL receptor related protein 1B	0,4	2,7	**7,02**	1,60E-05
DRAM1	DNA damage regulated autophagy modulator 1	15,8	143,1	**7,01**	0
CD70	CD70 molecule	0,1	1,0	**7,01**	3,00E-05
CBX2	chromobox 2	0,1	1,2	**6,99**	0
EHF	ETS homologous factor	0,1	0,6	**6,97**	4,00E-06
HES6	hes family bHLH transcription factor 6	1,8	15,8	**6,94**	0
IL2RG	interleukin 2 receptor subunit gamma	1,5	12,5	**6,89**	0
GPR39	G protein-coupled receptor 39	0,7	4,9	**6,84**	0
SLC22A23	solute carrier family 22 member 23	5,1	41,7	**6,83**	0
IL11	interleukin 11	1,2	11,9	**6,82**	1,00E-06
TMEM71	transmembrane protein 71	0,2	1,7	**6,82**	0
KRT7	keratin 7	0,1	1,0	**6,80**	2,00E-06
CYP3A5	cytochrome P450 family 3 subfamily A member 5	5,7	53,1	**6,79**	0
AMPD3	adenosine monophosphate deaminase 3	13,9	118,2	**6,78**	0
ISG20	interferon stimulated exonuclease gene 20	8,9	73,9	**6,78**	0
KCNC3	potassium voltage-gated channel subfamily C member 3	1,4	12,3	**6,71**	0
FGR	FGR proto-oncogene, Src family tyrosine kinase	0,5	3,4	**6,69**	0
AHCYL2	adenosylhomocysteinase like 2	19,7	155,5	**6,68**	0

ERN1	endoplasmic reticulum to nucleus signaling 1	38,2	297,7	**6,64**	0
HIST2H2BF	histone cluster 2 H2B family member f	0,1	1,3	**6,61**	7,80E-05
ITPRIP	inositol 1,4,5-trisphosphate receptor interacting protein	30,1	223,1	**6,59**	0
IL10RB-AS1	IL10RB antisense RNA 1 (head to head)	0,7	1,3	**6,59**	1,60E-05
LINC00513	long intergenic non-protein coding RNA 513	0,1	1,0	**6,51**	3,00E-06
PCED1B	PC-esterase domain containing 1B	0,2	2,2	**6,49**	3,00E-06
SLC30A2	solute carrier family 30 member 2	2,3	16,1	**6,49**	0
DOCK10	dedicator of cytokinesis 10	0,2	1,8	**6,48**	1,10E-05
DUSP6	dual specificity phosphatase 6	51,1	416,6	**6,45**	0
NLRP3	NLR family pyrin domain containing 3	0,1	0,6	**6,44**	0
NFKBIZ	NFKB inhibitor zeta	31,1	255,6	**6,34**	0
EPHB2	EPH receptor B2	10,4	71,2	**6,34**	0
GRHL1	grainyhead like transcription factor 1	0,7	4,7	**6,33**	1,00E-06
WIPF3	WAS/WASL interacting protein family member 3	0,6	4,5	**6,28**	0
TAC1	tachykinin precursor 1	1,9	11,1	**6,23**	0
KRT15	keratin 15	0,2	1,2	**6,17**	0
JHDM1D-AS1	JHDM1D antisense RNA 1 (head to head)			**6,17**	0
SLC7A7	solute carrier family 7 member 7	1,1	9,5	**6,15**	0
LRRC38	leucine rich repeat containing 38	4,5	33,5	**6,11**	0
CA12	carbonic anhydrase 12	3,0	20,4	**6,08**	0
GSAP	gamma-secretase activating protein	3,1	24,3	**6,06**	0
GFPT2	glutamine-fructose-6-phosphate transaminase 2	52,4	403,7	**6,05**	0
SEMA3C	semaphorin 3C	95,9	750,6	**6,05**	0
STX11	syntaxin 11	3,3	24,2	**5,99**	0
NFKB2	nuclear factor kappa B subunit 2	37,6	279,6	**5,98**	0
BMPR1B	bone morphogenetic protein receptor type 1B	0,7	4,7	**5,97**	0
C11orf96	chromosome 11 open reading frame 96	36,7	258,4	**5,93**	0
ALOXE3	arachidonate lipoxygenase 3	0,4	3,0	**5,92**	0
TRIM36	tripartite motif containing 36	2,0	14,1	**5,91**	0
LINC01588	long intergenic non-protein coding RNA 1588	1,9	13,0	**5,81**	0
TRAF3IP3	TRAF3 interacting protein 3	0,1	0,9	**5,80**	1,30E-05
ZSWIM4	zinc finger SWIM-type containing 4	3,9	27,1	**5,77**	0
F3	coagulation factor III, tissue factor	0,5	3,0	**5,76**	1,00E-06

SLC22A1	solute carrier family 22 member 1	0,2	1,4	**5,75**	0
PGBD5	piggyBac transposable element derived 5	0,8	4,1	**5,74**	0
KLF5	Kruppel like factor 5	13,9	91,3	**5,66**	0
PMAIP1	phorbol-12-myristate-13-acetate-induced protein 1	1,5	9,6	**5,65**	0
KIF1A	kinesin family member 1A	2,5	7,2	**5,65**	4,00E-06
DTX2	deltex E3 ubiquitin ligase 2	3,0	20,2	**5,64**	0
FGF18	fibroblast growth factor 18	3,0	22,5	**5,51**	0
FERMT1	fermitin family member 1	5,8	30,0	**5,44**	0
CCNA1	cyclin A1	0,3	2,2	**5,42**	1,00E-06
AKR1C1	aldo-keto reductase family 1 member C1	20,3	167,5	**5,41**	0
SLC25A25-AS	SLC25A25 antisense RNA 1	1,2	7,7	**5,34**	0
KCNJ2-AS1	KCNJ2 antisense RNA 1 (head to head)	0,3	2,1	**5,34**	0
AGTR1	angiotensin II receptor type 1	0,4	2,8	**5,32**	0
FTH1P20	ferritin heavy chain 1 pseudogene 20	0,2	1,0	**5,32**	1,00E-06
PAQR5	progestin and adipoQ receptor family member 5	0,2	0,9	**5,31**	0
AQP9	aquaporin 9	0,3	2,0	**5,31**	9,70E-05
NDP	NDP, norrin cystine knot growth factor	8,6	48,1	**5,31**	0
IFNAR2	interferon alpha and beta receptor subunit 2	5,0	31,6	**5,30**	0
HIST1H2BC	histone cluster 1 H2B family member c	1,9	12,2	**5,28**	0
LINC01004	long intergenic non-protein coding RNA 1004	0,7	4,7	**5,25**	0
STOX1	storkhead box 1	0,3	2,0	**5,24**	0
FCRLA	Fc receptor like A	0,7	4,1	**5,23**	0
PDE4B	phosphodiesterase 4B	64,7	410,7	**5,22**	0
KCNE5	potassium voltage-gated channel subfamily E regulatory subunit 5	0,2	1,3	**5,21**	0
TNIP3	TNFAIP3 interacting protein 3	0,1	1,0	**5,19**	3,50E-05
HIST1H2BE	histone cluster 1 H2B family member e	0,3	2,0	**5,18**	1,00E-06
ABCA13	ATP binding cassette subfamily A member 13	2,8	14,7	**5,14**	1,00E-06
ATP2B1	ATPase plasma membrane Ca2+ transporting 1	97,3	590,1	**5,14**	0
HIST1H2BJ	histone cluster 1 H2B family member j	0,8	4,9	**5,12**	0
ULBP1	UL16 binding protein 1	3,6	21,6	**5,11**	0
SDK1	sidekick cell adhesion molecule 1	1,7	9,4	**5,11**	0
HIST1H2BK	histone cluster 1 H2B family member k	4,1	24,3	**5,11**	0
GJB2	gap junction protein beta 2	2,5	12,7	**5,10**	0

VWC2	von Willebrand factor C domain containing 2	4,9	27,4	**5,10**	0
ALOX12P2	arachidonate 12-lipoxygenase pseudogene 2	0,1	0,7	**5,07**	4,00E-06
SYP	synaptophysin	1,1	6,1	**5,07**	0
FCER1G	Fc fragment of IgE receptor Ig	0,4	2,6	**5,06**	0
ST3GAL1	ST3 beta-galactoside alpha-2,3-sialyltransferase 1	84,8	502,0	**5,06**	0
PPP1R15A	protein phosphatase 1 regulatory subunit 15A	84,4	474,2	**5,06**	0
TIFA	TRAF interacting protein with forkhead associated domain	12,2	69,7	**5,03**	0
RIPK2	receptor interacting serine/threonine kinase 2	19,2	113,6	**5,03**	0
LINC01750	long intergenic non-protein coding RNA 1750	0,1	0,7	**5,03**	0
NCF2	neutrophil cytosolic factor 2	0,3	1,1	**5,01**	0
HIST1H2BD	histone cluster 1 H2B family member d	2,7	16,0	**5,00**	0
HIC1	HIC ZBTB transcriptional repressor 1	1,3	7,5	**5,00**	0
ADAMTSL4-A	ADAMTSL4 antisense RNA 1	0,3	1,8	**4,99**	9,00E-06
BEST4	bestrophin 4	0,3	1,9	**4,95**	2,00E-06
MMP9	matrix metallopeptidase 9	0,3	1,4	**4,95**	3,40E-05
TCHH	trichohyalin	0,3	1,8	**4,94**	0
GDF15	growth differentiation factor 15	20,2	102,7	**4,93**	0
JUN	Jun proto-oncogene, AP-1 transcription factor subunit	67,4	356,6	**4,93**	0
CCRL2	C-C motif chemokine receptor like 2	3,6	19,1	**4,92**	0
PROX1	prospero homeobox 1	0,1	0,7	**4,92**	4,20E-05
WTAP	WT1 associated protein	67,6	402,2	**4,92**	0
CCDC168	coiled-coil domain containing 168	0,1	0,7	**4,88**	2,00E-06
S100A9	S100 calcium binding protein A9	2,6	15,3	**4,87**	0
NFKBIE	NFKB inhibitor epsilon	6,2	35,8	**4,87**	0
FTH1	ferritin heavy chain 1	1509,0	8432,3	**4,83**	1,00E-06
DDN	dendrin	0,1	0,5	**4,83**	0
PLK3	polo like kinase 3	11,2	61,6	**4,82**	0
ENTPD7	ectonucleoside triphosphate diphosphohydrolase 7	13,2	74,6	**4,80**	0
IP6K3	inositol hexakisphosphate kinase 3	0,2	1,4	**4,80**	9,90E-05
KCNK12	potassium two pore domain channel subfamily K member 12	0,1	0,6	**4,80**	0
ANKRD33B	ankyrin repeat domain 33B	0,1	0,6	**4,80**	8,20E-05
PDCD1LG2	programmed cell death 1 ligand 2	2,8	13,9	**4,78**	0
LINC01426	long intergenic non-protein coding RNA 1426	3,0	17,0	**4,77**	0

KCNJ2	potassium voltage-gated channel subfamily J member 2	1,4	7,9	**4,76**	1,00E-06
ABCC3	ATP binding cassette subfamily C member 3	7,3	40,8	**4,76**	0
CITED4	Cbp/p300 interacting transactivator with Glu/Asp rich carboxy-termin	9,4	51,1	**4,75**	0
SYNPO2	synaptopodin 2	1,0	5,8	**4,70**	1,10E-05
N4BP3	NEDD4 binding protein 3	1,1	6,7	**4,66**	0
ARHGAP24	Rho GTPase activating protein 24	4,9	28,5	**4,64**	0
FBXO32	F-box protein 32	41,9	214,0	**4,63**	0
ROPN1L	rhophilin associated tail protein 1 like	0,1	0,6	**4,63**	1,00E-06
LAMC2	laminin subunit gamma 2	5,1	26,2	**4,63**	0
SEMA4D	semaphorin 4D	2,9	15,3	**4,61**	0
IER5	immediate early response 5	33,5	170,1	**4,61**	0
PTGES	prostaglandin E synthase	17,4	98,8	**4,60**	0
FTH1P15	ferritin heavy chain 1 pseudogene 15	0,1	0,9	**4,59**	6,00E-06
MAK	male germ cell associated kinase	0,1	0,6	**4,59**	4,50E-05
LINC01465	long intergenic non-protein coding RNA 1465	1,4	7,1	**4,58**	0
FTH1P23	ferritin heavy chain 1 pseudogene 23	1,5	7,9	**4,58**	0
SLFN5	schlafen family member 5	24,4	126,9	**4,56**	0
LHFPL3-AS2	LHFPL3 antisense RNA 2	0,3	1,8	**4,55**	4,00E-06
TMEM158	transmembrane protein 158 (gene/pseudogene)	15,6	64,7	**4,53**	0
PSD4	pleckstrin and Sec7 domain containing 4	14,8	79,7	**4,50**	0
ZNF697	zinc finger protein 697	4,6	24,4	**4,47**	0
CST1	cystatin SN	3,6	10,2	**4,46**	0,00306
PEG10	paternally expressed 10	3,8	23,4	**4,44**	1,00E-06
PNRC1	proline rich nuclear receptor coactivator 1	63,2	355,2	**4,44**	0
CLEC2D	C-type lectin domain family 2 member D	4,5	22,4	**4,43**	0
LIPG	lipase G, endothelial type	0,4	2,6	**4,43**	0
SGK1	serum/glucocorticoid regulated kinase 1	52,1	298,1	**4,42**	0
DOCK4	dedicator of cytokinesis 4	1,2	6,6	**4,42**	2,00E-06
LINC01869	long intergenic non-protein coding RNA 1869	0,2	0,9	**4,42**	0
SOCS1	suppressor of cytokine signaling 1	1,4	6,3	**4,41**	0
SLCO4A1	solute carrier organic anion transporter family member 4A1	2,8	13,0	**4,41**	2,00E-06
CST2	cystatin SA	1,3	4,2	**4,40**	0,000116
DLGAP1-AS1	DLGAP1 antisense RNA 1	4,1	21,4	**4,39**	0

GBP3	guanylate binding protein 3	13,3	65,0	**4,38**	0
ATP2B1-AS1	ATP2B1 antisense RNA 1	3,4	17,4	**4,35**	0
HIST1H1E	histone cluster 1 H1 family member e	0,3	1,3	**4,34**	3,80E-05
SLC11A2	solute carrier family 11 member 2	52,3	263,8	**4,33**	0
PITPNC1	phosphatidylinositol transfer protein, cytoplasmic 1	5,8	25,9	**4,32**	0
SDC4	syndecan 4	244,1	1290,9	**4,32**	1,00E-06
CTSV	cathepsin V	0,5	2,2	**4,30**	0
BDKRB2	bradykinin receptor B2	16,3	79,3	**4,30**	0
CLDN4	claudin 4	0,2	1,2	**4,29**	0
TRERF1	transcriptional regulating factor 1	5,0	25,9	**4,25**	1,00E-06
NGF	nerve growth factor	12,1	60,6	**4,25**	0
SYNGR3	synaptogyrin 3	0,4	2,9	**4,24**	1,30E-05
FAM43A	family with sequence similarity 43 member A	8,6	39,1	**4,24**	0
RAB27B	RAB27B, member RAS oncogene family	0,2	1,0	**4,22**	0,000869
CDH2	cadherin 2	0,3	1,7	**4,22**	3,00E-06
PAPPA	pappalysin 1	32,3	169,7	**4,22**	2,00E-06
SEMA3F	semaphorin 3F	1,0	5,6	**4,21**	0
FTH1P8	ferritin heavy chain 1 pseudogene 8	1,2	6,4	**4,19**	0
LINC02407	long intergenic non-protein coding RNA 2407		1,8	**4,15**	5,00E-06
LINC02454	long intergenic non-protein coding RNA 2454	0,2	0,9	**4,13**	3,00E-06
RAC2	ras-related C3 botulinum toxin substrate 2 (rho family, small GTP bind	1,2	4,3	**4,12**	0
FGF22	fibroblast growth factor 22	0,2	1,0	**4,11**	1,80E-05
ENKUR	enkurin, TRPC channel interacting protein	2,8	12,0	**4,11**	0
LINC00884	long intergenic non-protein coding RNA 884	0,4	1,8	**4,10**	1,00E-06
L3MBTL4	l(3)mbt-like 4 (Drosophila)	1,5	7,1	**4,10**	7,00E-06
FAM222A	family with sequence similarity 222 member A	4,4	18,8	**4,10**	0
TGIF1	TGFB induced factor homeobox 1	35,0	158,1	**4,09**	0
BCL11A	B-cell CLL/lymphoma 11A	0,2	1,0	**4,08**	2,00E-06
FTH1P12	ferritin heavy chain 1 pseudogene 12	0,4	1,6	**4,07**	0
FAM19A3	family with sequence similarity 19 member A3, C-C motif chemokine l	0,3	1,5	**4,05**	5,60E-05
MIR155HG	MIR155 host gene	0,6	2,5	**4,05**	0
HIST1H2AG	histone cluster 1 H2A family member g	0,5	2,3	**4,04**	0
GPR68	G protein-coupled receptor 68	8,3	38,3	**4,04**	0

IGFBP1	insulin like growth factor binding protein 1	17,9	67,7	**4,03**	0
ABCB1	ATP binding cassette subfamily B member 1	0,6	2,7	**4,03**	0
ZMIZ1-AS1	ZMIZ1 antisense RNA 1	5,9	24,0	**4,01**	0
CST4	cystatin S	0,9	2,5	**4,00**	0,001209
STRIP2	striatin interacting protein 2	3,6	15,2	**3,99**	0
TACSTD2	tumor associated calcium signal transducer 2	10,5	52,3	**3,99**	0
HIST1H4H	histone cluster 1 H4 family member h	2,4	11,4	**3,98**	0
HAS3	hyaluronan synthase 3	1,5	7,4	**3,97**	1,00E-06
PLEK2	pleckstrin 2	0,2	0,9	**3,96**	0
CYP2J2	cytochrome P450 family 2 subfamily J member 2	0,1	0,5	**3,96**	0
RPTN	repetin	0,2	0,9	**3,96**	0
HS3ST3B1	heparan sulfate-glucosamine 3-sulfotransferase 3B1	8,5	36,6	**3,93**	0
ANGPTL4	angiopoietin like 4	37,5	148,0	**3,93**	0
ASNS	asparagine synthetase (glutamine-hydrolyzing)	1,4	6,3	**3,92**	1,00E-06
MGST1	microsomal glutathione S-transferase 1	7,7	32,6	**3,92**	0
ELOVL3	ELOVL fatty acid elongase 3	0,2	1,2	**3,92**	0
NPTX2	neuronal pentraxin 2	5,6	21,8	**3,91**	0
SLC2A1	solute carrier family 2 member 1	139,4	632,0	**3,88**	0
TNFRSF10B	TNF receptor superfamily member 10b	52,9	217,2	**3,88**	0
XDH	xanthine dehydrogenase	0,4	1,6	**3,87**	4,00E-06
AXIN2	axin 2	22,7	89,8	**3,86**	0
OSBP2	oxysterol binding protein 2	1,1	4,1	**3,86**	0
RASGEF1B	RasGEF domain family member 1B	2,9	13,6	**3,86**	0
LINC00160	long intergenic non-protein coding RNA 160	0,3	1,3	**3,86**	0
NGFR	nerve growth factor receptor	2,2	9,8	**3,85**	0
CDCP1	CUB domain containing protein 1	2,2	8,0	**3,83**	0
AGRN	agrin	23,3	106,9	**3,83**	0
BEST1	bestrophin 1	18,1	79,7	**3,82**	0
PLA2G4C	phospholipase A2 group IVC	5,4	22,3	**3,81**	0
AP3B2	adaptor related protein complex 3 beta 2 subunit	0,5	2,7	**3,78**	2,00E-06
FAM110B	family with sequence similarity 110 member B	10,4	44,3	**3,78**	0
RARRES1	retinoic acid receptor responder 1	1,2	4,8	**3,78**	0
BNC1	basonuclin 1	0,1	0,7	**3,76**	1,00E-05

A4GALT	alpha 1,4-galactosyltransferase (P blood group)	42,3	180,1	**3,75**	0
ACSL4	acyl-CoA synthetase long-chain family member 4	94,0	418,8	**3,74**	0
TNFRSF21	TNF receptor superfamily member 21	1,3	4,5	**3,73**	6,00E-06
KDM7A	lysine demethylase 7A	46,4	196,6	**3,73**	0
MT2A	metallothionein 2A	2019,4	8236,1	**3,73**	4,40E-05
HMGA1	high mobility group AT-hook 1	240,8	891,7	**3,72**	0
SEMA3G	semaphorin 3G	0,4	1,7	**3,71**	6,00E-06
SLC19A2	solute carrier family 19 member 2	5,1	21,3	**3,70**	0
SUSD6	sushi domain containing 6	36,2	151,8	**3,70**	0
TEX14	testis expressed 14, intercellular bridge forming factor	0,3	1,2	**3,70**	0
TNFSF9	TNF superfamily member 9	1,5	6,3	**3,70**	0
LACC1	laccase domain containing 1	6,3	25,1	**3,67**	0
DLL4	delta like canonical Notch ligand 4	0,2	0,9	**3,66**	1,30E-05
RIPK4	receptor interacting serine/threonine kinase 4	1,6	6,7	**3,65**	0
OSGIN2	oxidative stress induced growth inhibitor family member 2	31,3	127,9	**3,65**	0
ADGRV1	adhesion G protein-coupled receptor V1	0,3	1,1	**3,64**	8,40E-05
DLGAP1	DLG associated protein 1	0,5	1,6	**3,63**	0
ID2	inhibitor of DNA binding 2, HLH protein	127,0	524,1	**3,63**	0
PAPPA2	pappalysin 2	5,4	23,5	**3,63**	5,30E-05
C9orf16	chromosome 9 open reading frame 16	24,2	102,9	**3,61**	0
CYLD	CYLD lysine 63 deubiquitinase	47,2	191,7	**3,59**	0
BDNF	brain derived neurotrophic factor	1,2	4,6	**3,58**	1,00E-06
FTH1P3	ferritin heavy chain 1 pseudogene 3	0,2	0,7	**3,58**	1,00E-06
C6orf132	chromosome 6 open reading frame 132	0,3	1,3	**3,57**	6,70E-05
LINC00623	long intergenic non-protein coding RNA 623	0,5	1,8	**3,56**	1,00E-06
MT1JP	metallothionein 1J, pseudogene	0,3	1,1	**3,55**	8,10E-05
DUSP2	dual specificity phosphatase 2	0,8	3,4	**3,55**	0,000286
HIST1H3H	histone cluster 1 H3 family member h	0,2	0,9	**3,54**	2,50E-05
BACH1	BTB domain and CNC homolog 1	18,3	78,9	**3,53**	0
PAX8-AS1	PAX8 antisense RNA 1	20,5	50,9	**3,52**	1,00E-06
COL7A1	collagen type VII alpha 1 chain	10,7	40,1	**3,51**	0
SLC16A6	solute carrier family 16 member 6	0,2	0,8	**3,51**	0,000376
TNIP1	TNFAIP3 interacting protein 1	95,8	388,7	**3,51**	0

SP6	Sp6 transcription factor	1,3	5,0	**3,50**	7,00E-06
LINC00310	long intergenic non-protein coding RNA 310	0,6	2,1	**3,49**	0
IFIH1	interferon induced with helicase C domain 1	3,2	13,4	**3,47**	2,70E-05
SMTN	smoothelin	67,5	239,4	**3,47**	0
CLMN	calmin	2,4	9,2	**3,46**	0
LRRC49	leucine rich repeat containing 49	6,1	24,2	**3,45**	0
LSMEM1	leucine rich single-pass membrane protein 1	0,6	2,3	**3,44**	0
FTH1P16	ferritin heavy chain 1 pseudogene 16	0,2	0,8	**3,43**	2,00E-06
TLE1	transducin like enhancer of split 1	7,3	24,6	**3,43**	0
ADAMTS9	ADAM metallopeptidase with thrombospondin type 1 motif 9	8,5	35,3	**3,43**	5,00E-06
UPP1	uridine phosphorylase 1	37,5	134,9	**3,43**	0
PIM1	Pim-1 proto-oncogene, serine/threonine kinase	16,2	58,2	**3,43**	0
NEDD4L	neural precursor cell expressed, developmentally down-regulated 4-li	7,9	31,3	**3,42**	4,00E-06
PRDM1	PR/SET domain 1	0,6	2,4	**3,42**	1,00E-06
SLC29A4	solute carrier family 29 member 4	0,2	0,9	**3,42**	0,001833
BMP2	bone morphogenetic protein 2	451,8	1588,6	**3,41**	5,10E-05
QPCT	glutaminyl-peptide cyclotransferase	0,7	2,4	**3,40**	1,00E-06
SHB	SH2 domain containing adaptor protein B	1,9	6,3	**3,40**	0
CAV1	caveolin 1	175,9	648,9	**3,40**	0
TG	thyroglobulin	0,4	1,4	**3,39**	0
PDZD2	PDZ domain containing 2	4,5	15,9	**3,37**	3,00E-06
FGF2	fibroblast growth factor 2	242,5	901,4	**3,35**	1,00E-06
SEMA6D	semaphorin 6D	8,7	33,8	**3,34**	0
DNAJB9	DnaJ heat shock protein family (Hsp40) member B9	67,7	263,5	**3,34**	0
DDX58	DExD/H-box helicase 58	7,1	25,2	**3,33**	0
GPRC5A	G protein-coupled receptor class C group 5 member A	24,4	94,5	**3,33**	0
NRP2	neuropilin 2	262,7	976,5	**3,32**	2,70E-05
ACKR3	atypical chemokine receptor 3	37,5	134,0	**3,32**	0
HOXD11	homeobox D11	0,2	0,7	**3,32**	0,000131
BARX2	BARX homeobox 2	14,9	54,9	**3,32**	0
BID	BH3 interacting domain death agonist	3,2	11,6	**3,31**	1,00E-06
LINC01239	long intergenic non-protein coding RNA 1239	0,6	2,2	**3,31**	0
ALPK2	alpha kinase 2	0,4	1,2	**3,30**	5,90E-05

TRIM72	tripartite motif containing 72	0,2	0,8	**3,29**	0,000711
LPP	LIM domain containing preferred translocation partner in lipoma	100,8	376,0	**3,28**	5,00E-06
ATF3	activating transcription factor 3	7,8	29,2	**3,28**	0
CPED1	cadherin like and PC-esterase domain containing 1	10,5	37,5	**3,27**	0
RFX8	RFX family member 8, lacking RFX DNA binding domain	2,0	6,3	**3,26**	0
SQSTM1	sequestosome 1	76,6	257,6	**3,26**	0
DIO3	iodothyronine deiodinase 3	5,7	21,7	**3,25**	1,00E-05
NR4A3	nuclear receptor subfamily 4 group A member 3	3,5	14,0	**3,24**	4,00E-06
TRIB1	tribbles pseudokinase 1	32,3	111,6	**3,24**	0
KHDRBS3	KH RNA binding domain containing, signal transduction associated 3	1,0	3,1	**3,24**	1,00E-06
AFDN-AS1	AFDN antisense RNA 1 (head to head)	66,8	159,9	**3,24**	2,00E-06
GLP2R	glucagon like peptide 2 receptor	1,9	6,3	**3,23**	2,00E-06
PTPN12	protein tyrosine phosphatase, non-receptor type 12	62,2	222,0	**3,23**	0
EGFR	epidermal growth factor receptor	51,0	189,9	**3,23**	2,00E-06
RHBDL2	rhomboid like 2	0,7	2,4	**3,22**	4,00E-06
TTLL4	tubulin tyrosine ligase like 4	16,4	56,7	**3,22**	0
PCDH17	protocadherin 17	0,3	0,9	**3,22**	0,001026
EFNB2	ephrin B2	5,9	20,3	**3,21**	1,00E-06
LINC01134	long intergenic non-protein coding RNA 1134	0,6	2,3	**3,21**	5,00E-06
MAP3K8	mitogen-activated protein kinase kinase kinase 8	19,7	74,8	**3,20**	0
MT1E	metallothionein 1E	399,3	1365,0	**3,19**	3,00E-06
SH3PXD2B	SH3 and PX domains 2B	21,1	78,5	**3,19**	1,00E-06
MTSS1	MTSS1, I-BAR domain containing	1,8	6,8	**3,19**	1,40E-05
CLIC2	chloride intracellular channel 2	3,2	10,2	**3,18**	0
ATP2C2-AS1	ATP2C2 antisense RNA 1	0,2	0,6	**3,18**	5,00E-06
MMP13	matrix metallopeptidase 13	106,0	398,9	**3,18**	7,70E-05
FZD10	frizzled class receptor 10	1,7	6,5	**3,18**	2,00E-06
GBP2	guanylate binding protein 2	20,7	83,6	**3,18**	0
GATM	glycine amidinotransferase	1,0	3,3	**3,18**	4,00E-06
DAW1	dynein assembly factor with WD repeats 1	0,3	0,9	**3,18**	0,001054
WFDC3	WAP four-disulfide core domain 3	0,6	2,2	**3,18**	1,90E-05
CLCF1	cardiotrophin like cytokine factor 1	19,5	64,0	**3,18**	0
PLPP3	phospholipid phosphatase 3	56,5	221,0	**3,17**	4,00E-06

C5orf56	chromosome 5 open reading frame 56	2,2	7,7	**3,17**	0
ALCAM	activated leukocyte cell adhesion molecule	9,1	32,1	**3,17**	1,00E-06
ZNF341-AS1	ZNF341 antisense RNA 1	0,6	2,1	**3,16**	3,50E-05
HMCN2	hemicentin 2	0,2	0,7	**3,16**	0,002306
ATP13A3	ATPase 13A3	84,3	298,2	**3,16**	1,00E-06
KLHL21	kelch like family member 21	148,4	492,0	**3,16**	1,00E-06
ADTRP	androgen dependent TFPI regulating protein	11,2	26,5	**3,16**	0,001508
AKR1C3	aldo-keto reductase family 1 member C3	3,4	12,2	**3,15**	4,00E-06
NXPE2	neurexophilin and PC-esterase domain family member 2	1,0	3,6	**3,15**	0,000177
MAP2	microtubule associated protein 2	0,5	1,2	**3,15**	0,001086
ODF3L1	outer dense fiber of sperm tails 3 like 1	0,6	1,9	**3,15**	1,00E-06
AHR	aryl hydrocarbon receptor	7,1	23,8	**3,15**	0
MT1P3	metallothionein 1 pseudogene 3	0,7	2,5	**3,14**	0
CDKN1A	cyclin dependent kinase inhibitor 1A	111,8	375,8	**3,13**	0
HIVEP2	human immunodeficiency virus type I enhancer binding protein 2	63,9	219,7	**3,13**	6,00E-06
ODF3B	outer dense fiber of sperm tails 3B	4,1	15,1	**3,13**	0
TPPP	tubulin polymerization promoting protein	0,9	3,0	**3,13**	0,000152
CPEB2	cytoplasmic polyadenylation element binding protein 2	14,2	47,9	**3,12**	0
MAP3K7CL	MAP3K7 C-terminal like	4,4	15,6	**3,12**	0
CSPG5	chondroitin sulfate proteoglycan 5	0,8	2,7	**3,12**	1,20E-05
RBM43	RNA binding motif protein 43	10,7	39,3	**3,11**	2,00E-06
NAMPTP1	nicotinamide phosphoribosyltransferase pseudogene 1	6,4	25,5	**3,11**	1,00E-06
MMP10	matrix metallopeptidase 10	7,5	24,3	**3,10**	0
SLC6A12	solute carrier family 6 member 12	0,5	1,7	**3,10**	2,00E-04
IGFBP3	insulin like growth factor binding protein 3	3955,6	12026,3	**3,09**	0,000561
BTN2A2	butyrophilin subfamily 2 member A2	3,0	11,0	**3,09**	3,00E-06
FOSL1	FOS like 1, AP-1 transcription factor subunit	124,0	335,7	**3,09**	0
RNF19B	ring finger protein 19B	46,0	148,7	**3,09**	0
SOX11	SRY-box 11	1,8	6,0	**3,08**	3,00E-06
GATA6	GATA binding protein 6	1,8	6,0	**3,08**	0,000494
ESAM	endothelial cell adhesion molecule	0,3	0,9	**3,07**	0,000323
DGAT2	diacylglycerol O-acyltransferase 2	0,9	3,4	**3,07**	4,60E-05
ABCA4	ATP binding cassette subfamily A member 4	0,6	2,1	**3,07**	8,90E-05

GRM4	glutamate metabotropic receptor 4	0,3	0,7	**3,06**	0,000342
SSSCA1-AS1	SSSCA1 antisense RNA 1 (head to head)	0,6	1,9	**3,06**	0
ADAMTS4	ADAM metallopeptidase with thrombospondin type 1 motif 4	0,7	2,7	**3,05**	0,000381
NCK1	NCK adaptor protein 1	24,6	78,3	**3,05**	0
AKAP12	A-kinase anchoring protein 12	28,8	76,2	**3,04**	0
BACH1-IT1	BACH1 intronic transcript 1	0,2	0,7	**3,03**	0,000411
IL7	interleukin 7	0,2	0,5	**3,03**	0,000169
MAP1LC3B2	microtubule associated protein 1 light chain 3 beta 2	0,2	0,5	**3,03**	1,20E-05
FRMD4A	FERM domain containing 4A	1,9	8,2	**3,03**	0,001398
TEX41	testis expressed 41 (non-protein coding)	1,8	5,8	**3,02**	0
IRX2	iroquois homeobox 2	0,8	2,3	**3,02**	0
SLC25A28	solute carrier family 25 member 28	19,1	62,0	**3,02**	0
SQOR	sulfide quinone oxidoreductase	9,0	29,6	**3,01**	0
COLEC10	collectin subfamily member 10	0,1	0,5	**3,00**	0,000143
PSMD6-AS2	PSMD6 antisense RNA 2	0,2	0,5	**3,00**	6,30E-05
PDE10A	phosphodiesterase 10A	1,4	4,2	**3,00**	1,80E-05
EPB41L4B	erythrocyte membrane protein band 4.1 like 4B	0,9	2,7	**3,00**	2,50E-05
LINC02062	long intergenic non-protein coding RNA 2062	0,2	0,6	**2,99**	8,00E-06
PIK3R3	phosphoinositide-3-kinase regulatory subunit 3	6,4	17,9	**2,99**	0
PARP8	poly(ADP-ribose) polymerase family member 8	1,3	4,3	**2,99**	1,30E-05
ABLIM2	actin binding LIM protein family member 2	0,3	0,8	**2,98**	0,000939
ORM1	orosomucoid 1	1,3	2,1	**2,98**	0,007827
PLK2	polo like kinase 2	12,1	40,3	**2,97**	0,000262
BTN3A1	butyrophilin subfamily 3 member A1	4,2	15,0	**2,97**	6,00E-06
CDHR2	cadherin related family member 2	0,2	0,8	**2,97**	0,001297
GBP4	guanylate binding protein 4	1,6	5,9	**2,96**	5,50E-05
PRKAR2B	protein kinase cAMP-dependent type II regulatory subunit beta	5,3	17,9	**2,95**	6,00E-06
C17orf49	chromosome 17 open reading frame 49	0,5	1,3	**2,95**	0,000237
MEDAG	mesenteric estrogen dependent adipogenesis	53,9	177,7	**2,95**	0
LINC01705	long intergenic non-protein coding RNA 1705	0,5	1,6	**2,94**	8,00E-06
PLD1	phospholipase D1	9,3	30,6	**2,94**	0
CHST2	carbohydrate sulfotransferase 2	28,0	85,4	**2,93**	0
CNNM4	cyclin and CBS domain divalent metal cation transport mediator 4	13,9	42,8	**2,93**	0

SLC39A14	solute carrier family 39 member 14	1742,4	5911,0	**2,93**	0,000401
NAMPT	nicotinamide phosphoribosyltransferase	158,1	583,5	**2,92**	1,50E-05
IFIT2	interferon induced protein with tetratricopeptide repeats 2	2,0	6,5	**2,92**	8,00E-06
CSN1S1	casein alpha s1	0,2	0,7	**2,92**	0,00292
INPP5J	inositol polyphosphate-5-phosphatase J	0,4	1,1	**2,92**	3,30E-05
RCAN1	regulator of calcineurin 1	152,4	421,4	**2,92**	0
CBR3	carbonyl reductase 3	11,4	33,8	**2,92**	0
ACSL3	acyl-CoA synthetase long-chain family member 3	108,6	336,4	**2,91**	0
ARRDC3	arrestin domain containing 3	183,9	592,9	**2,91**	2,00E-06
EML5	echinoderm microtubule associated protein like 5		1,0	**2,91**	0,004139
DMXL2	Dmx like 2	20,0	64,9	**2,89**	3,00E-06
PDZRN3	PDZ domain containing ring finger 3	21,4	67,1	**2,88**	3,00E-06
DUSP4	dual specificity phosphatase 4	6,3	17,0	**2,88**	0
KCNJ14	potassium voltage-gated channel subfamily J member 14	0,5	1,5	**2,87**	1,00E-06
MMP19	matrix metallopeptidase 19	0,5	1,3	**2,86**	2,60E-05
GALNT9	polypeptide N-acetylgalactosaminyltransferase 9	4,1	12,7	**2,86**	1,00E-06
EML2-AS1	EML2 antisense RNA 1	0,4	1,1	**2,86**	6,10E-05
DRC3	dynein regulatory complex subunit 3	1,2	3,6	**2,85**	4,50E-05
DDIT4	DNA damage inducible transcript 4	91,1	269,9	**2,85**	0
ZNRF1	zinc and ring finger 1	12,9	38,8	**2,83**	0
SLC15A3	solute carrier family 15 member 3	3,7	11,7	**2,83**	1,50E-05
SLPI	secretory leukocyte peptidase inhibitor	7,5	17,5	**2,83**	0
MAML2	mastermind like transcriptional coactivator 2	29,4	94,8	**2,82**	1,00E-06
SLC13A4	solute carrier family 13 member 4	0,4	0,9	**2,82**	6,00E-06
CACNA1G	calcium voltage-gated channel subunit alpha1 G	0,2	0,7	**2,82**	4,50E-05
CTSW	cathepsin W	3,1	8,1	**2,82**	1,00E-06
PSMB10	proteasome subunit beta 10	2,4	8,3	**2,82**	3,00E-06
MTHFD2L	methylenetetrahydrofolate dehydrogenase (NADP+ dependent) 2-like	5,1	16,5	**2,81**	0
SCNN1D	sodium channel epithelial 1 delta subunit	1,7	4,8	**2,80**	0
MT1M	metallothionein 1M	97,0	291,7	**2,80**	1,00E-06
FICD	FIC domain containing	32,5	98,3	**2,80**	0
GPR35	G protein-coupled receptor 35	0,4	1,3	**2,78**	1,80E-05
TLL2	tolloid like 2	0,5	1,4	**2,78**	1,00E-06

KDM6B	lysine demethylase 6B	14,0	42,9	**2,77**	0
SLC25A34	solute carrier family 25 member 34	0,7	1,9	**2,77**	6,90E-05
BBC3	BCL2 binding component 3	3,2	10,1	**2,77**	0
MAPKBP1	mitogen-activated protein kinase binding protein 1	16,2	45,8	**2,77**	0
NTF3	neurotrophin 3	0,8	2,2	**2,76**	0,001295
PTGFR	prostaglandin F receptor	6,1	17,4	**2,76**	0
MLLT11	myeloid/lymphoid or mixed-lineage leukemia; translocated to, 11	16,6	46,7	**2,76**	0
HLX	H2.0 like homeobox	1,4	4,4	**2,75**	3,00E-06
NFKB1	nuclear factor kappa B subunit 1	39,9	118,6	**2,75**	0
GADD45G	growth arrest and DNA damage inducible gamma	4,2	11,4	**2,75**	0
ZNF267	zinc finger protein 267	6,5	18,5	**2,75**	0
SLC43A2	solute carrier family 43 member 2	0,7	2,4	**2,75**	0,000106
UCN2	urocortin 2	0,3	0,7	**2,75**	1,60E-05
BTN3A3	butyrophilin subfamily 3 member A3	0,8	2,8	**2,75**	0,000494
SPSB4	splA/ryanodine receptor domain and SOCS box containing 4	4,0	11,4	**2,74**	0
TRAF3IP2	TRAF3 interacting protein 2	42,0	126,1	**2,74**	0
PHLDA1	pleckstrin homology like domain family A member 1	161,1	432,5	**2,74**	0
SPHK1	sphingosine kinase 1	14,3	36,5	**2,74**	0
TMPRSS4	transmembrane protease, serine 4	0,8	2,0	**2,74**	0,000214
SMOX	spermine oxidase	50,5	139,9	**2,73**	0
BTG2	BTG anti-proliferation factor 2	36,5	110,6	**2,73**	0
MAP2K3	mitogen-activated protein kinase kinase 3	57,4	160,3	**2,73**	0
C9orf163	chromosome 9 open reading frame 163	0,1	0,4	**2,73**	0,000424
MDM2	MDM2 proto-oncogene	74,4	198,9	**2,73**	0
PARP12	poly(ADP-ribose) polymerase family member 12	6,3	18,4	**2,72**	0
EN2	engrailed homeobox 2	0,2	0,5	**2,72**	0,000246
HIF1A-AS2	HIF1A antisense RNA 2	1,0	3,0	**2,72**	2,30E-05
CHST11	carbohydrate sulfotransferase 11	27,9	71,1	**2,72**	0
LURAP1L	leucine rich adaptor protein 1 like	5,4	15,0	**2,72**	1,00E-06
HIST1H2BN	histone cluster 1 H2B family member n	0,5	1,1	**2,72**	1,00E-06
CBR3-AS1	CBR3 antisense RNA 1	1,5	4,0	**2,71**	2,00E-06
CCDC71L	coiled-coil domain containing 71 like	25,3	75,7	**2,71**	0
TRIM25	tripartite motif containing 25	30,6	85,4	**2,71**	0

LINC01176	long intergenic non-protein coding RNA 1176	0,3	0,7	**2,71**	4,20E-05
FHDC1	FH2 domain containing 1	7,4	22,0	**2,71**	6,00E-06
NPPA-AS1	NPPA antisense RNA 1	0,0	0,0	**2,70**	1,70E-05
PRSS54	protease, serine 54	0,2	0,6	**2,70**	0,000627
TXNL4B	thioredoxin like 4B	8,2	21,0	**2,69**	0
HIST1H2AC	histone cluster 1 H2A family member c	16,6	46,0	**2,68**	0
LAMA1	laminin subunit alpha 1	3,9	12,3	**2,67**	5,00E-05
IRF8	interferon regulatory factor 8	0,5	1,3	**2,67**	7,00E-06
CEBPA	CCAAT/enhancer binding protein alpha	0,3	0,9	**2,66**	4,00E-05
TNFRSF11B	TNF receptor superfamily member 11b	1525,1	4260,7	**2,66**	0,000241
MT1L	metallothionein 1L, pseudogene	24,2	75,2	**2,66**	0
PTPRH	protein tyrosine phosphatase, receptor type H	0,5	1,1	**2,66**	1,70E-05
FBXO24	F-box protein 24	0,3	0,7	**2,65**	1,50E-05
RAB3A	RAB3A, member RAS oncogene family	0,9	2,4	**2,65**	1,40E-05
ZEB2	zinc finger E-box binding homeobox 2	28,6	87,9	**2,65**	1,50E-05
SH2D5	SH2 domain containing 5	1,2	2,9	**2,65**	1,50E-05
CYB5R2	cytochrome b5 reductase 2	14,2	41,3	**2,65**	0
TRPV3	transient receptor potential cation channel subfamily V member 3	0,6	1,7	**2,65**	0,00089
PCDH1	protocadherin 1	7,3	19,2	**2,65**	0
CEL	carboxyl ester lipase	0,4	0,9	**2,64**	3,00E-06
STS	steroid sulfatase	23,5	68,0	**2,64**	0
UCKL1-AS1	UCKL1 antisense RNA 1	0,3	1,0	**2,64**	0,000275
PRKAG2-AS1	PRKAG2 antisense RNA 1	1,0	2,8	**2,63**	1,00E-06
CCDC154	coiled-coil domain containing 154	0,4	1,3	**2,63**	0,000104
AKR1B1	aldo-keto reductase family 1 member B	60,2	194,3	**2,63**	1,00E-06
RELT	RELT, TNF receptor	9,2	23,5	**2,63**	0
ERAP2	endoplasmic reticulum aminopeptidase 2	11,1	26,2	**2,63**	0,001692
PHACTR1	phosphatase and actin regulator 1	4,9	14,0	**2,62**	1,00E-06
C3orf52	chromosome 3 open reading frame 52	0,9	2,5	**2,61**	1,50E-05
SLCO3A1	solute carrier organic anion transporter family member 3A1	23,4	62,9	**2,61**	0
TNFRSF10D	TNF receptor superfamily member 10d	111,4	282,1	**2,61**	0
ADM	adrenomedullin	78,3	213,8	**2,61**	1,00E-06
BTG3	BTG anti-proliferation factor 3	18,4	46,9	**2,61**	0

MID1	midline 1	1,2	3,2	**2,61**	0,000381
SPX	spexin hormone	7,2	18,2	**2,60**	0
ISM2	isthmin 2	0,7	1,4	**2,60**	3,00E-06
SERPINA1	serpin family A member 1	418,5	1226,9	**2,60**	5,40E-05
FGFBP1	fibroblast growth factor binding protein 1	1,0	2,2	**2,60**	0,007143
SLC9A1	solute carrier family 9 member A1	32,5	84,0	**2,60**	0
WARS	tryptophanyl-tRNA synthetase	188,7	497,1	**2,60**	5,00E-06
JPH1	junctophilin 1	1,1	2,1	**2,59**	0,002156
HS6ST3	heparan sulfate 6-O-sulfotransferase 3	0,3	0,8	**2,59**	7,20E-05
CEBPD	CCAAT/enhancer binding protein delta	139,9	382,5	**2,58**	0
ID2-AS1	ID2 antisense RNA 1 (head to head)	0,8	2,2	**2,58**	0,000101
TXNIP	thioredoxin interacting protein	116,2	305,6	**2,58**	0
NR6A1	nuclear receptor subfamily 6 group A member 1	0,3	0,7	**2,58**	0,000219
RSAD2	radical S-adenosyl methionine domain containing 2	0,5	1,4	**2,57**	0,00287
CASZ1	castor zinc finger 1	0,9	2,2	**2,57**	2,90E-05
SYNJ2	synaptojanin 2	60,5	160,5	**2,57**	3,00E-06
PPP1R14C	protein phosphatase 1 regulatory inhibitor subunit 14C	25,1	60,6	**2,57**	0
ABL2	ABL proto-oncogene 2, non-receptor tyrosine kinase	40,1	110,5	**2,56**	1,70E-05
GPR176	G protein-coupled receptor 176	16,6	41,9	**2,56**	0
NPTX1	neuronal pentraxin 1	0,4	1,0	**2,56**	0,000215
CDC42EP2	CDC42 effector protein 2	3,0	8,2	**2,55**	3,90E-05
TRIM9	tripartite motif containing 9	2,1	5,5	**2,55**	1,00E-06
RPSAP52	ribosomal protein SA pseudogene 52	0,3	0,9	**2,55**	0,003026
GLDC	glycine decarboxylase	0,2	0,6	**2,55**	0,000642
GPRIN1	G protein regulated inducer of neurite outgrowth 1	1,3	3,3	**2,55**	0,000163
GALNT12	polypeptide N-acetylgalactosaminyltransferase 12	8,4	21,2	**2,54**	0
IZUMO4	IZUMO family member 4	0,5	1,3	**2,54**	2,70E-05
CDKN2A	cyclin dependent kinase inhibitor 2A	2,7	6,0	**2,54**	1,00E-06
IRAK3	interleukin 1 receptor associated kinase 3	20,4	52,7	**2,54**	0
TMEM171	transmembrane protein 171	2,6	6,8	**2,54**	1,00E-06
CNOT6LP1	CCR4-NOT transcription complex subunit 6-like pseudogene 1	0,4	1,0	**2,53**	0,008586
ACVR1C	activin A receptor type 1C	0,3	0,7	**2,53**	0,000843
TSPAN33	tetraspanin 33	1,5	4,2	**2,53**	0,000307

KIAA1614-AS	KIAA1614 antisense RNA 1	0,4	1,0	**2,53**	0,000339
NKIRAS1	NFKB inhibitor interacting Ras like 1	15,7	38,4	**2,52**	0
MET	MET proto-oncogene, receptor tyrosine kinase	127,2	327,2	**2,52**	9,00E-06
ZNF217	zinc finger protein 217	27,3	69,1	**2,52**	0
VCAM1	vascular cell adhesion molecule 1	855,8	2492,6	**2,52**	0,001448
MILR1	mast cell immunoglobulin like receptor 1	1,5	3,5	**2,52**	0,000215
LYPD3	LY6/PLAUR domain containing 3	0,7	2,3	**2,52**	0,000577
IL10RA	interleukin 10 receptor subunit alpha	0,4	1,0	**2,52**	0,00026
IGFBP4	insulin like growth factor binding protein 4	75,3	209,2	**2,51**	0
NANOS1	nanos C2HC-type zinc finger 1	9,2	20,3	**2,51**	0
PID1	phosphotyrosine interaction domain containing 1	104,6	279,3	**2,51**	1,00E-06
TLR1	toll like receptor 1	2,7	6,4	**2,51**	2,00E-06
CLSTN2	calsyntenin 2	0,8	1,9	**2,50**	9,00E-06
CHD7	chromodomain helicase DNA binding protein 7	6,5	15,2	**2,50**	2,00E-06
ZC3H12C	zinc finger CCCH-type containing 12C	12,1	31,2	**2,50**	6,00E-06
LYN	LYN proto-oncogene, Src family tyrosine kinase	19,7	50,6	**2,50**	0
NEK10	NIMA related kinase 10	0,6	1,3	**2,50**	2,00E-05
ABTB2	ankyrin repeat and BTB domain containing 2	47,4	119,5	**2,50**	0
UBE2H	ubiquitin conjugating enzyme E2 H	73,8	186,2	**2,49**	0
MT1G	metallothionein 1G	872,6	2106,6	**2,49**	0,00068
ASPRV1	aspartic peptidase, retroviral-like 1	1,8	4,2	**2,49**	3,00E-06
FAR2	fatty acyl-CoA reductase 2	1,2	3,1	**2,49**	1,30E-05
DNAAF1	dynein axonemal assembly factor 1	0,2	0,6	**2,48**	0,000353
HS6ST1	heparan sulfate 6-O-sulfotransferase 1	11,3	27,6	**2,48**	0
TICAM1	toll like receptor adaptor molecule 1	16,5	40,4	**2,48**	0
MT1P1	metallothionein 1 pseudogene 1	0,2	0,6	**2,48**	0,004161
RASL11B	RAS like family 11 member B	0,3	0,9	**2,48**	0,008669
VAMP1	vesicle associated membrane protein 1	4,5	11,1	**2,47**	0
MEIG1	meiosis/spermiogenesis associated 1	0,2	0,5	**2,47**	0,000215
GABARAPL1	GABA type A receptor associated protein like 1	37,9	96,6	**2,47**	0
KIAA1683	KIAA1683			**2,47**	8,60E-05
ABCA1	ATP binding cassette subfamily A member 1	1,3	3,8	**2,47**	0,000789
SPOCD1	SPOC domain containing 1	3,5	7,4	**2,46**	0,000125

SLC3A2	solute carrier family 3 member 2	216,3	536,4	**2,46**	2,00E-06
MGAT5B	mannosyl (alpha-1,6-)-glycoprotein beta-1,6-N-acetyl-glucosaminyltra	0,3	0,7	**2,46**	0,003213
LINC00707	long intergenic non-protein coding RNA 707	2,1	5,5	**2,46**	0,00059
IQCD	IQ motif containing D	0,7	1,6	**2,45**	5,60E-05
YJEFN3	YjeF N-terminal domain containing 3	0,8	1,8	**2,45**	1,00E-06
GPR146	G protein-coupled receptor 146	2,6	6,8	**2,45**	1,00E-06
CCDC17	coiled-coil domain containing 17	0,5	1,2	**2,44**	0,000138
LINC01336	long intergenic non-protein coding RNA 1336		0,5	**2,44**	0,002885
AFDN	afadin, adherens junction formation factor	66,8	159,9	**2,44**	1,00E-06
EHD1	EH domain containing 1	54,6	129,1	**2,44**	0
CSRNP1	cysteine and serine rich nuclear protein 1	30,0	75,0	**2,43**	0
PARP14	poly(ADP-ribose) polymerase family member 14	14,9	39,4	**2,43**	1,70E-05
ITGB2-AS1	ITGB2 antisense RNA 1	0,9	2,3	**2,43**	4,00E-06
MAFG	MAF bZIP transcription factor G	35,2	86,9	**2,43**	0
SLC7A11	solute carrier family 7 member 11	52,2	131,1	**2,43**	0
NEDD9	neural precursor cell expressed, developmentally down-regulated 9	3,7	9,8	**2,43**	2,90E-05
PAG1	phosphoprotein membrane anchor with glycosphingolipid microdoma	4,1	10,1	**2,43**	1,20E-05
TMEM217	transmembrane protein 217	0,9	2,3	**2,43**	0,000648
TBC1D8	TBC1 domain family member 8	2,5	6,4	**2,43**	4,20E-05
PSEN1	presenilin 1	47,3	120,7	**2,43**	0
NCOA7	nuclear receptor coactivator 7	24,3	59,0	**2,43**	0
BHLHE22	basic helix-loop-helix family member e22	0,3	1,0	**2,42**	0,000822
MYOZ2	myozenin 2	0,3	0,7	**2,42**	0,003318
ISLR2	immunoglobulin superfamily containing leucine rich repeat 2	0,4	0,9	**2,42**	5,70E-05
SERPINB8	serpin family B member 8	15,8	38,9	**2,42**	1,00E-06
SIK3	SIK family kinase 3	29,9	75,0	**2,42**	1,00E-06
PLEKHH1	pleckstrin homology, MyTH4 and FERM domain containing H1	0,5	1,3	**2,41**	3,00E-05
IYD	iodotyrosine deiodinase	0,2	0,5	**2,41**	0,002154
SYN3	synapsin III	0,4	0,9	**2,41**	0,003259
AIF1L	allograft inflammatory factor 1 like	0,2	0,5	**2,41**	0,010214
PELI1	pellino E3 ubiquitin protein ligase 1	24,6	60,2	**2,41**	1,00E-06
RTN1	reticulon 1	1,1	2,6	**2,41**	7,00E-06
SLC25A37	solute carrier family 25 member 37	174,8	444,1	**2,40**	1,40E-05

CLDN1	claudin 1	2,0	5,1	**2,40**	0,000218
ULBP2	UL16 binding protein 2	9,2	21,0	**2,40**	0
RELA	RELA proto-oncogene, NF-kB subunit	90,3	225,6	**2,40**	3,00E-06
PIK3IP1	phosphoinositide-3-kinase interacting protein 1	19,5	49,5	**2,40**	0
PANX1	pannexin 1	13,3	33,7	**2,39**	1,00E-06
SMIM29	small integral membrane protein 29	18,2	39,9	**2,39**	0
HILPDA	hypoxia inducible lipid droplet associated	9,9	23,7	**2,39**	0
CCDC150P1	coiled-coil domain containing 150 pseudogene 1			**2,39**	0,006963
AC003002.1	Trafficking protein particle complex subunit 2B	0,0	0,0	**2,38**	0,000133
MIR9-3HG	MIR9-3 host gene	0,2	0,4	**2,38**	0,004546
MT1XP1	metallothionein 1X pseudogene 1	2,2	4,6	**2,38**	0,000169
BTN2A3P	butyrophilin subfamily 2 member A3, pseudogene	1,3	3,7	**2,38**	0,00137
MGAT4A	mannosyl (alpha-1,3-)-glycoprotein beta-1,4-N-acetylglucosaminyltrar	4,8	11,6	**2,38**	3,00E-06
GUCA1B	guanylate cyclase activator 1B	0,5	1,0	**2,37**	0,000808
BTC	betacellulin	2,7	6,3	**2,37**	1,00E-06
GPAT3	glycerol-3-phosphate acyltransferase 3	8,7	20,0	**2,37**	0
CCDC163	coiled-coil domain containing 163	2,5	5,0	**2,37**	6,00E-06
PARD6B	par-6 family cell polarity regulator beta	0,5	1,0	**2,37**	2,50E-05
TMEM191A	transmembrane protein 191A (pseudogene)	0,6	1,3	**2,37**	0,000409
PRDM2	PR/SET domain 2	33,7	86,1	**2,36**	7,00E-06
NPIPA1	nuclear pore complex interacting protein family member A1	0,2	0,6	**2,36**	0,006238
AZIN2	antizyme inhibitor 2	4,1	9,5	**2,36**	0
FYN	FYN proto-oncogene, Src family tyrosine kinase	12,0	30,6	**2,36**	0
HK2	hexokinase 2	49,7	123,3	**2,35**	1,00E-06
MESDC1	mesoderm development candidate 1			**2,35**	0
RNF144B	ring finger protein 144B	46,3	112,4	**2,34**	0
PI4KAP1	phosphatidylinositol 4-kinase alpha pseudogene 1	0,6	1,0	**2,34**	0,000689
CYBA	cytochrome b-245 alpha chain	34,9	82,3	**2,34**	0
SDCBP	syndecan binding protein	124,2	296,1	**2,33**	1,00E-06
GHRLOS	ghrelin opposite strand/antisense RNA	0,3	0,8	**2,33**	0,000503
LINC02280	long intergenic non-protein coding RNA 2280	0,2	0,5	**2,32**	0,001266
BMP6	bone morphogenetic protein 6	69,8	160,1	**2,32**	1,00E-06
RHBDF2	rhomboid 5 homolog 2	26,0	60,9	**2,32**	0

ZFP36L1	ZFP36 ring finger protein like 1	140,2	331,2	**2,32**	4,00E-06
PTGES2	prostaglandin E synthase 2	32,6	70,7	**2,32**	0
SLC26A1	solute carrier family 26 member 1	0,5	1,5	**2,31**	0,00499
CFAP45	cilia and flagella associated protein 45	0,3	0,6	**2,31**	0,001039
DDIT3	DNA damage inducible transcript 3	32,0	68,4	**2,31**	0
FRMD6-AS1	FRMD6 antisense RNA 1	0,9	2,2	**2,31**	2,10E-05
CFAP69	cilia and flagella associated protein 69	2,5	6,0	**2,30**	2,60E-05
PDE7A	phosphodiesterase 7A	3,6	9,4	**2,30**	1,20E-05
ARRDC4	arrestin domain containing 4	18,5	43,3	**2,30**	0
PRKAG2	protein kinase AMP-activated non-catalytic subunit gamma 2	21,1	46,8	**2,30**	0
SYT9	synaptotagmin 9	0,3	0,5	**2,30**	0,00427
HRH1	histamine receptor H1	98,0	220,0	**2,30**	3,00E-06
ARHGAP42	Rho GTPase activating protein 42	23,1	56,3	**2,30**	1,90E-05
DYRK3	dual specificity tyrosine phosphorylation regulated kinase 3	12,9	30,9	**2,30**	9,00E-06
TSPAN10	tetraspanin 10	1,5	2,8	**2,30**	0,001291
FLNC	filamin C	1,2	2,4	**2,29**	2,00E-05
NOCT	nocturnin	6,7	16,1	**2,29**	4,00E-06
AMZ1	archaelysin family metallopeptidase 1	0,3	0,8	**2,29**	0,008478
RASSF8-AS1	RASSF8 antisense RNA 1	9,2	21,5	**2,28**	2,00E-06
SLC41A2	solute carrier family 41 member 2	16,7	41,2	**2,28**	3,00E-06
TMEM81	transmembrane protein 81	1,7	3,8	**2,28**	1,10E-05
MT1H	metallothionein 1H	96,1	208,7	**2,28**	1,00E-05
KMT2E	lysine methyltransferase 2E	72,4	174,2	**2,28**	1,70E-05
ANTXR2	anthrax toxin receptor 2	55,1	112,1	**2,28**	1,00E-06
FAM126A	family with sequence similarity 126 member A	23,0	54,7	**2,28**	1,00E-06
PDE2A	phosphodiesterase 2A	0,8	1,7	**2,27**	0,002204
TENM3	teneurin transmembrane protein 3	5,5	11,2	**2,27**	0,000349
KLHL29	kelch like family member 29	31,1	71,4	**2,27**	1,00E-06
NECTIN1	nectin cell adhesion molecule 1	15,2	34,2	**2,27**	1,00E-06
ADGRG1	adhesion G protein-coupled receptor G1	10,4	22,2	**2,27**	9,40E-05
PTGIR	prostaglandin I2 (prostacyclin) receptor (IP)	4,0	10,0	**2,26**	1,80E-05
MTCL1	microtubule crosslinking factor 1	4,1	9,3	**2,26**	3,80E-05
PTGER2	prostaglandin E receptor 2	27,9	68,5	**2,26**	1,00E-06

CIDECP	cell death-inducing DFFA-like effector c pseudogene		10,6	**2,26**	0
HOMER1	homer scaffolding protein 1	3,2	7,6	**2,26**	1,00E-05
SIPA1L2	signal induced proliferation associated 1 like 2	29,0	66,7	**2,25**	6,00E-06
MAP1LC3B	microtubule associated protein 1 light chain 3 beta	88,6	197,5	**2,25**	0
P2RX7	purinergic receptor P2X 7	1,4	2,8	**2,25**	0,000707
ENC1	ectodermal-neural cortex 1	8,3	17,6	**2,25**	1,70E-05
CTH	cystathionine gamma-lyase	6,1	13,2	**2,25**	0
TOM1	target of myb1 membrane trafficking protein	54,7	123,5	**2,24**	0
PALM2	paralemmin 2	1,1	2,7	**2,24**	0,001524
LRGUK	leucine rich repeats and guanylate kinase domain containing	0,2	0,5	**2,24**	0,002504
WNT9A	Wnt family member 9A	3,8	7,9	**2,24**	8,00E-06
KCND3	potassium voltage-gated channel subfamily D member 3	2,9	7,0	**2,24**	9,70E-05
AGTRAP	angiotensin II receptor associated protein	46,4	104,5	**2,24**	0
CD226	CD226 molecule	0,2	0,6	**2,23**	0,009739
DGKI	diacylglycerol kinase iota	0,7	1,7	**2,23**	0,008996
GAL	galanin and GMAP prepropeptide	0,6	1,2	**2,23**	0,001343
APBA3	amyloid beta precursor protein binding family A member 3	13,8	30,7	**2,23**	0
FAM160A1	family with sequence similarity 160 member A1	8,5	19,7	**2,23**	0,000116
BASP1	brain abundant membrane attached signal protein 1	4,3	9,7	**2,23**	0,000578
STAM-AS1	STAM antisense RNA 1 (head to head)	0,3	0,6	**2,23**	0,004287
RASSF4	Ras association domain family member 4	4,1	8,7	**2,22**	0
ADGRB2	adhesion G protein-coupled receptor B2	0,7	1,3	**2,22**	0,000536
MARCKSL1	MARCKS like 1	3,9	10,0	**2,22**	5,30E-05
ADAM8	ADAM metallopeptidase domain 8	0,8	1,9	**2,22**	6,20E-05
USP44	ubiquitin specific peptidase 44	0,6	1,4	**2,22**	7,40E-05
CPA4	carboxypeptidase A4	41,6	94,1	**2,21**	1,00E-06
TBX3	T-box 3	2,0	5,2	**2,21**	0,001583
STARD5	StAR related lipid transfer domain containing 5	0,9	2,0	**2,21**	0,000104
SLC2A3	solute carrier family 2 member 3	17,6	38,5	**2,21**	0
TIAM2	T-cell lymphoma invasion and metastasis 2	113,5	256,6	**2,21**	3,10E-05
MYOSLID	myocardin-induced smooth muscle lncRNA, inducer of differentiation	0,5	1,0	**2,21**	0,000843
ANKRD6	ankyrin repeat domain 6	9,9	22,0	**2,20**	0
DARS-AS1	DARS antisense RNA 1	1,2	2,8	**2,20**	3,00E-05

ZNF385A	zinc finger protein 385A	61,9	135,6	**2,20**	6,00E-06
MAATS1	MYCBP associated and testis expressed 1	2,4	4,9	**2,20**	2,30E-05
UBTD2	ubiquitin domain containing 2	56,0	126,3	**2,20**	1,00E-06
CBARP	CACN beta subunit associated regulatory protein	4,6	9,2	**2,19**	4,50E-05
LAMP3	lysosomal associated membrane protein 3	2,3	4,3	**2,19**	3,00E-05
BCL2L11	BCL2 like 11	3,7	9,0	**2,19**	9,50E-05
LYZ	lysozyme	1,3	2,7	**2,19**	0,000173
PLCXD2	phosphatidylinositol specific phospholipase C X domain containing 2	0,2	0,4	**2,19**	0,030367
TINCR	tissue differentiation-inducing non-protein coding RNA	0,5	1,1	**2,18**	0,000177
NBPF19	NBPF member 19	4,1	9,4	**2,18**	0,000287
HELZ2	helicase with zinc finger 2	8,8	20,4	**2,18**	0,000185
KCTD16	potassium channel tetramerization domain containing 16	0,6	1,4	**2,18**	0,002902
ATG2A	autophagy related 2A	18,8	41,4	**2,18**	1,00E-06
MEIS1	Meis homeobox 1	2,8	5,7	**2,18**	0,000142
BTN3A2	butyrophilin subfamily 3 member A2	2,1	5,0	**2,18**	5,00E-04
LAT2	linker for activation of T-cells family member 2	0,4	0,7	**2,18**	0,001827
RNF122	ring finger protein 122	0,4	0,9	**2,18**	0,002104
STEAP2	STEAP2 metalloreductase	33,4	78,0	**2,18**	2,00E-06
RELN	reelin	2,0	2,9	**2,18**	0,006714
PKIB	cAMP-dependent protein kinase inhibitor beta	0,3	0,6	**2,17**	0,001099
ALDH4A1	aldehyde dehydrogenase 4 family member A1	3,0	6,4	**2,17**	2,10E-05
ARHGAP26	Rho GTPase activating protein 26	4,5	9,6	**2,17**	3,90E-05
VEGFA	vascular endothelial growth factor A	280,6	597,0	**2,17**	0,000117
LZTS3	leucine zipper tumor suppressor family member 3	3,5	7,6	**2,17**	1,00E-06
SLC22A4	solute carrier family 22 member 4	5,7	12,2	**2,17**	1,40E-05
DEPDC7	DEP domain containing 7	2,3	4,8	**2,17**	1,50E-05
CCDC157	coiled-coil domain containing 157	2,0	3,9	**2,16**	7,00E-06
EPB41L3	erythrocyte membrane protein band 4.1 like 3	14,6	33,9	**2,16**	3,00E-06
LGALS3BP	galectin 3 binding protein	0,4	0,7	**2,16**	0,000587
PPP3CC	protein phosphatase 3 catalytic subunit gamma	13,8	30,2	**2,16**	0
TRAF2	TNF receptor associated factor 2	13,6	27,2	**2,16**	1,00E-06
S100P	S100 calcium binding protein P	1,5	3,2	**2,16**	2,70E-05
NCMAP	non-compact myelin associated protein	0,5	0,8	**2,16**	0,001142

NXT2	nuclear transport factor 2 like export factor 2	11,5	24,2	**2,16**	1,00E-06
SIRPA	signal regulatory protein alpha	135,6	304,7	**2,16**	2,70E-05
VPS37A	VPS37A, ESCRT-I subunit	29,3	62,7	**2,16**	0
IGFBP7	insulin like growth factor binding protein 7	101,9	207,8	**2,15**	1,00E-06
FAM107B	family with sequence similarity 107 member B	57,4	113,4	**2,15**	4,00E-06
CLIC6	chloride intracellular channel 6	21,4	46,9	**2,15**	1,00E-06
PTHLH	parathyroid hormone like hormone	1,1	2,2	**2,15**	3,00E-05
CHST7	carbohydrate sulfotransferase 7	3,0	6,7	**2,15**	5,50E-05
MAN1A1	mannosidase alpha class 1A member 1	144,1	386,1	**2,15**	0,000194
ATG7	autophagy related 7	23,3	49,3	**2,15**	0
GCNT4	glucosaminyl (N-acetyl) transferase 4, core 2	3,9	8,7	**2,15**	0,000128
CD44	CD44 molecule (Indian blood group)	624,0	1398,2	**2,14**	0,000696
C11orf68	chromosome 11 open reading frame 68	63,1	126,5	**2,14**	0
TMEM27	transmembrane protein 27			**2,14**	0,000549
TNKS1BP1	tankyrase 1 binding protein 1	130,0	281,0	**2,14**	1,90E-05
GCNT3	glucosaminyl (N-acetyl) transferase 3, mucin type	1,7	3,1	**2,14**	4,00E-04
TRMO	tRNA methyltransferase O	8,5	17,2	**2,14**	0
FAM83B	family with sequence similarity 83 member B	1,5	3,1	**2,14**	0,008838
MMP2	matrix metallopeptidase 2	34,1	69,1	**2,13**	0
PLAUR	plasminogen activator, urokinase receptor	107,8	220,4	**2,13**	2,00E-06
CASP4	caspase 4	23,8	52,1	**2,13**	0
ITGB2	integrin subunit beta 2	15,5	30,2	**2,13**	0
IL34	interleukin 34	0,5	1,1	**2,13**	0,004866
KCNK3	potassium two pore domain channel subfamily K member 3	0,3	0,7	**2,13**	0,008573
KCNQ1OT1	KCNQ1 opposite strand/antisense transcript 1 (non-protein coding)	5,7	12,9	**2,13**	5,70E-05
LINC01137	long intergenic non-protein coding RNA 1137	3,7	8,2	**2,12**	2,10E-05
PELI2	pellino E3 ubiquitin protein ligase family member 2	2,1	4,3	**2,12**	3,30E-05
KIAA1211L	KIAA1211 like	0,5	0,7	**2,12**	0,003784
LYG1	lysozyme g1	0,4	0,6	**2,12**	0,000623
SNHG19	small nucleolar RNA host gene 19	4,1	8,4	**2,12**	1,00E-06
RGMB	repulsive guidance molecule family member b	14,9	32,2	**2,12**	0
ATG4D	autophagy related 4D cysteine peptidase	6,5	12,2	**2,12**	0
UBR5-AS1	UBR5 antisense RNA 1	0,4	0,8	**2,12**	0,001427

RUNX1	runt related transcription factor 1	100,6	222,1	**2,11**	2,80E-05
LINC01772	long intergenic non-protein coding RNA 1772	0,4	0,8	**2,11**	0,001969
SLC25A45	solute carrier family 25 member 45	5,5	11,0	**2,11**	1,00E-06
C5orf66	chromosome 5 open reading frame 66	0,6	1,3	**2,11**	0,000735
EFNB1	ephrin B1	13,1	27,8	**2,11**	0
HERC6	HECT and RLD domain containing E3 ubiquitin protein ligase family me	1,4	2,7	**2,11**	0,002703
NFE2L2	nuclear factor, erythroid 2 like 2	82,9	170,4	**2,11**	2,00E-06
LINC02257	long intergenic non-protein coding RNA 2257	0,2	0,4	**2,11**	0,005276
PIGA	phosphatidylinositol glycan anchor biosynthesis class A	6,2	13,7	**2,11**	1,50E-05
B3GNT2	UDP-GlcNAc:betaGal beta-1,3-N-acetylglucosaminyltransferase 2	9,9	21,0	**2,11**	1,00E-06
ZC3H12D	zinc finger CCCH-type containing 12D	0,6	1,0	**2,11**	0,000292
USP49	ubiquitin specific peptidase 49	3,9	7,7	**2,11**	1,80E-05
CACUL1	CDK2 associated cullin domain 1	35,6	71,6	**2,11**	0
TMIE	transmembrane inner ear	1,2	2,4	**2,10**	0,000168
PCDH12	protocadherin 12	4,7	9,1	**2,10**	0,000116
BCL6	B-cell CLL/lymphoma 6	127,3	275,6	**2,10**	3,20E-05
FST	follistatin	115,4	249,8	**2,10**	3,90E-05
AEN	apoptosis enhancing nuclease	26,1	51,8	**2,10**	0
FAM86FP	family with sequence similarity 86 member F, pseudogene	0,4	0,9	**2,10**	0,002398
ARRDC2	arrestin domain containing 2	32,3	68,5	**2,10**	0
GNG11	G protein subunit gamma 11	10,5	21,2	**2,10**	3,00E-06
ZNF165	zinc finger protein 165	0,7	1,4	**2,09**	0,000566
MAFB	MAF bZIP transcription factor B	96,8	220,0	**2,09**	1,50E-05
RBPJ	recombination signal binding protein for immunoglobulin kappa J regi	71,2	148,3	**2,09**	3,00E-06
CHKB-AS1	CHKB antisense RNA 1 (head to head)	0,5	0,8	**2,09**	0,000846
HDX	highly divergent homeobox	0,2	0,6	**2,09**	0,020657
RASGRP3	RAS guanyl releasing protein 3	0,3	0,7	**2,09**	0,044966
ERO1B	endoplasmic reticulum oxidoreductase 1 beta	10,7	25,9	**2,09**	2,80E-05
ARID3A	AT-rich interaction domain 3A	2,2	4,7	**2,09**	0,000453
TWIST2	twist family bHLH transcription factor 2	4,3	8,4	**2,08**	4,00E-06
GTPBP2	GTP binding protein 2	55,2	110,9	**2,08**	1,00E-06
TP53RK	TP53 regulating kinase	17,6	35,0	**2,08**	0
STK10	serine/threonine kinase 10	25,6	49,2	**2,08**	0

NCAM2	neural cell adhesion molecule 2	1,0	2,0	**2,08**	0,003622
GLIS3	GLIS family zinc finger 3	22,4	47,4	**2,07**	3,00E-05
CCBE1	collagen and calcium binding EGF domains 1	0,3	0,5	**2,07**	0,001605
LINC01686	long intergenic non-protein coding RNA 1686	0,9	2,0	**2,07**	0,00068
SLC43A3	solute carrier family 43 member 3	53,7	110,1	**2,07**	4,00E-06
HMOX1	heme oxygenase 1	475,9	924,8	**2,07**	0,000684
SIRT4	sirtuin 4	0,4	0,6	**2,07**	0,001287
NOXRED1	NADP dependent oxidoreductase domain containing 1	0,3	0,6	**2,07**	0,004766
PCBP3	poly(rC) binding protein 3	1,9	4,3	**2,07**	0,00032
BET1	Bet1 golgi vesicular membrane trafficking protein	15,1	28,8	**2,07**	0
RABGEF1	RAB guanine nucleotide exchange factor 1	2,8	4,9	**2,07**	6,00E-06
LRFN5	leucine rich repeat and fibronectin type III domain containing 5	0,8	1,3	**2,07**	0,018204
ADIRF-AS1	ADIRF antisense RNA 1	2,4	4,7	**2,07**	5,00E-05
DNER	delta/notch like EGF repeat containing	68,2	136,2	**2,06**	5,80E-05
SIRPB1	signal regulatory protein beta 1	0,9	2,0	**2,06**	0,027521
GK	glycerol kinase	2,3	4,7	**2,06**	0,000177
MT1X	metallothionein 1X	223,1	429,8	**2,06**	0,000574
SLC39A8	solute carrier family 39 member 8	66,1	143,8	**2,06**	3,00E-06
NRG1	neuregulin 1	0,3	0,7	**2,06**	0,017248
ITGA2	integrin subunit alpha 2	7,0	13,5	**2,06**	4,00E-06
EFCAB2	EF-hand calcium binding domain 2	6,7	12,1	**2,05**	0
ADD3	adducin 3	16,5	33,6	**2,05**	1,00E-06
RAI14	retinoic acid induced 14	22,8	47,0	**2,05**	2,10E-05
LONRF3	LON peptidase N-terminal domain and ring finger 3	2,8	5,5	**2,05**	0,001063
KCNK5	potassium two pore domain channel subfamily K member 5	53,5	90,7	**2,05**	0
ABCB9	ATP binding cassette subfamily B member 9	38,6	74,3	**2,05**	4,00E-06
SPATA17	spermatogenesis associated 17	1,2	1,9	**2,05**	1,90E-05
DNAH5	dynein axonemal heavy chain 5	1,7	3,7	**2,05**	0,000697
ISOC1	isochorismatase domain containing 1	13,0	24,1	**2,05**	0
MAFK	MAF bZIP transcription factor K	75,1	155,0	**2,04**	1,50E-05
CCDC85B	coiled-coil domain containing 85B	100,9	179,1	**2,04**	1,00E-06
PARD6A	par-6 family cell polarity regulator alpha	1,3	2,6	**2,04**	0,000359
MCC	mutated in colorectal cancers	12,6	28,9	**2,04**	0,000251

CTNS	cystinosin, lysosomal cystine transporter	14,5	28,4	**2,04**	1,00E-06
IFNGR2	interferon gamma receptor 2	3,4	7,6	**2,04**	0,001
ACRBP	acrosin binding protein	0,4	0,8	**2,04**	0,005865
SLC31A2	solute carrier family 31 member 2	34,0	65,8	**2,04**	5,00E-06
UGCG	UDP-glucose ceramide glucosyltransferase	56,7	120,4	**2,04**	4,00E-06
ZNF620	zinc finger protein 620	0,8	1,6	**2,03**	0,000538
BBS12	Bardet-Biedl syndrome 12	9,3	18,0	**2,03**	4,00E-06
SELENOK	selenoprotein K	46,7	95,7	**2,03**	1,00E-06
GAL3ST1	galactose-3-O-sulfotransferase 1	0,3	0,6	**2,03**	0,001772
LIX1L-AS1	LIX1L antisense RNA 1	0,2	0,5	**2,03**	0,004728
JARID2	jumonji and AT-rich interaction domain containing 2	6,5	13,2	**2,03**	3,00E-05
MIR100HG	mir-100-let-7a-2 cluster host gene	187,2	365,4	**2,03**	9,80E-05
CD58	CD58 molecule	9,6	17,9	**2,03**	0
MORC3	MORC family CW-type zinc finger 3	28,2	58,2	**2,03**	3,00E-06
ECE1	endothelin converting enzyme 1	35,8	71,7	**2,03**	2,00E-06
TMEM86B	transmembrane protein 86B	0,3	0,6	**2,03**	0,019311
HSPA4L	heat shock protein family A (Hsp70) member 4 like	5,8	11,0	**2,02**	4,80E-05
TMEM140	transmembrane protein 140	3,0	5,9	**2,02**	0,001278
CD163L1	CD163 molecule like 1	0,5	0,9	**2,02**	0,00157
OSCP1	organic solute carrier partner 1	3,5	6,8	**2,02**	1,40E-05
SRPK1	SRSF protein kinase 1	29,5	56,5	**2,02**	5,00E-06
SPOCK2	SPARC/osteonectin, cwcv and kazal like domains proteoglycan 2	0,3	0,5	**2,01**	0,009675
TMC7	transmembrane channel like 7	4,3	8,2	**2,01**	0,000784
AMTN	amelotin	12,1	24,3	**2,01**	0,000271
CETN4P	centrin 4, pseudogene	0,6	1,2	**2,01**	0,004071
PYGM	glycogen phosphorylase, muscle associated	0,3	0,7	**2,01**	0,00946
GET4	golgi to ER traffic protein 4	1,7	3,5	**2,01**	0,000776
MEI1	meiotic double-stranded break formation protein 1	8,1	18,3	**2,01**	3,00E-05
ZC2HC1C	zinc finger C2HC-type containing 1C	1,5	2,6	**2,01**	6,20E-05
KIAA1958	KIAA1958	1,2	2,5	**2,01**	0,005055
CAPN10-AS1	CAPN10 antisense RNA 1 (head to head)	12,2	14,1	**2,01**	0,002161
TSPAN1	tetraspanin 1	6,6	12,1	**2,01**	4,00E-06
TP53BP2	tumor protein p53 binding protein 2	24,6	50,8	**2,01**	1,50E-05

PLGRKT	plasminogen receptor with a C-terminal lysine	9,9	19,4	**2,00**	0
RET	ret proto-oncogene	0,8	1,9	**2,00**	0,007626
LINC01768	long intergenic non-protein coding RNA 1768	0,2	0,4	**2,00**	0,010545
TRIM62	tripartite motif containing 62	3,7	8,3	**2,00**	0,00084
XBP1	X-box binding protein 1	246,3	468,7	**2,00**	3,20E-05
STARD13	StAR related lipid transfer domain containing 13	42,3	85,2	**2,00**	2,00E-06
CSNK1E	casein kinase 1 epsilon	28,1	52,9	**2,00**	0
SMPD2	sphingomyelin phosphodiesterase 2	6,3	11,6	**2,00**	2,00E-06
B3GNT5	UDP-GlcNAc:betaGal beta-1,3-N-acetylglucosaminyltransferase 5	13,1	25,4	**2,00**	2,90E-05
ZDHHC11B	zinc finger DHHC-type containing 11B	0,3	0,6	**2,00**	0,03198
MYLIP	myosin regulatory light chain interacting protein	7,5	14,2	**2,00**	2,00E-06
SLC35E3	solute carrier family 35 member E3	18,3	35,0	**2,00**	0
GLIPR2	GLI pathogenesis related 2	2,2	3,7	**2,00**	4,10E-05
PANX2	pannexin 2	6,2	11,3	**1,99**	0,000112
UBE2QL1	ubiquitin conjugating enzyme E2 Q family like 1	3,0	5,2	**1,99**	0,002622
RAPGEFL1	Rap guanine nucleotide exchange factor like 1	2,6	5,5	**1,99**	0,000526
ONECUT2	one cut homeobox 2	0,6	1,3	**1,99**	0,005707
PPL	periplakin	9,4	20,7	**1,99**	0,000595
CACHD1	cache domain containing 1	23,9	47,5	**1,99**	9,00E-06
USP53	ubiquitin specific peptidase 53	67,9	136,8	**1,99**	0,000272
DAGLB	diacylglycerol lipase beta	9,8	18,6	**1,99**	0
CEBPB	CCAAT/enhancer binding protein beta	161,3	324,2	**1,98**	3,60E-05
SMG1P7	SMG1P7, nonsense mediated mRNA decay associated PI3K related kir	0,9	1,7	**1,98**	9,60E-05
TAF1A	TATA-box binding protein associated factor, RNA polymerase I subuni	9,2	17,4	**1,98**	5,00E-06
SLAIN1	SLAIN motif family member 1	2,0	4,0	**1,98**	0,000516
ERAP1	endoplasmic reticulum aminopeptidase 1	39,8	81,6	**1,98**	0,000192
ANKRD12	ankyrin repeat domain 12	83,8	167,7	**1,98**	2,10E-05
EGLN3	egl-9 family hypoxia inducible factor 3	16,0	30,1	**1,98**	2,00E-06
SNHG26	small nucleolar RNA host gene 26	0,7	1,4	**1,98**	0,001965
DISP2	dispatched RND transporter family member 2	1,1	2,0	**1,97**	0,006913
MYH3	myosin heavy chain 3	1,7	2,9	**1,97**	0,000388
FAM46C	family with sequence similarity 46 member C			**1,97**	6,70E-05
UBALD2	UBA like domain containing 2	36,2	65,2	**1,97**	0

HOXD1	homeobox D1	0,4	0,6	**1,97**	0,002507
FAM189A2	family with sequence similarity 189 member A2	2,1	4,1	**1,97**	0,00292
TRH	thyrotropin releasing hormone	0,2	0,4	**1,97**	0,022626
COX19	COX19, cytochrome c oxidase assembly factor	7,4	13,7	**1,97**	2,20E-05
SLC12A6	solute carrier family 12 member 6	31,9	60,7	**1,97**	3,00E-06
RTTN	rotatin	7,2	13,1	**1,97**	1,00E-06
HERC5	HECT and RLD domain containing E3 ubiquitin protein ligase 5	3,5	5,7	**1,97**	5,00E-05
MIS12	MIS12, kinetochore complex component	20,3	37,7	**1,97**	1,00E-06
ZNF622	zinc finger protein 622	33,8	60,8	**1,97**	0
IL18R1	interleukin 18 receptor 1	1,4	2,9	**1,97**	0,004134
IL1RAP	interleukin 1 receptor accessory protein	14,3	25,0	**1,97**	3,00E-06
KDM4D	lysine demethylase 4D	1,6	2,7	**1,97**	0,000751
HAVCR2	hepatitis A virus cellular receptor 2	0,4	0,6	**1,97**	0,002753
LARP6	La ribonucleoprotein domain family member 6	42,0	79,8	**1,96**	0
SERTAD1	SERTA domain containing 1	33,7	62,6	**1,96**	0
RBM48	RNA binding motif protein 48	9,2	17,2	**1,96**	6,00E-06
PACERR	PTGS2 antisense NFKB1 complex-mediated expression regulator RNA	0,2	0,5	**1,96**	0,036858
HSPA13	heat shock protein family A (Hsp70) member 13	85,4	162,9	**1,96**	1,80E-05
SNHG15	small nucleolar RNA host gene 15	7,6	13,4	**1,96**	1,00E-06
CDKN2B-AS1	CDKN2B antisense RNA 1	0,3	0,6	**1,96**	0,011968
ARNT2	aryl hydrocarbon receptor nuclear translocator 2	0,9	1,6	**1,96**	0,004053
ANKHD1-EIF⁴	ANKHD1-EIF4EBP3 readthrough	0,3	0,7	**1,95**	0,002595
TTLL9	tubulin tyrosine ligase like 9	0,3	0,5	**1,95**	0,008643
GCLM	glutamate-cysteine ligase modifier subunit	61,0	112,9	**1,95**	1,50E-05
TMEM186	transmembrane protein 186	8,9	16,1	**1,95**	1,90E-05
KBTBD8	kelch repeat and BTB domain containing 8	4,5	8,6	**1,95**	0,000503
SLC46A3	solute carrier family 46 member 3	2,8	4,9	**1,95**	2,80E-05
TIGAR	TP53 induced glycolysis regulatory phosphatase	7,8	14,2	**1,95**	8,00E-06
PHLDB2	pleckstrin homology like domain family B member 2	1,1	2,2	**1,95**	0,036839
ZFAND2A	zinc finger AN1-type containing 2A	14,3	26,4	**1,95**	1,90E-05
ALMS1-IT1	ALMS1 intronic transcript 1	0,4	0,7	**1,95**	0,018343
ZFAND6	zinc finger AN1-type containing 6	33,8	64,2	**1,95**	0
EPHB1	EPH receptor B1	1,7	3,0	**1,95**	0,003863

SLC19A3	solute carrier family 19 member 3	8,7	17,3	**1,95**	1,80E-05
SERTAD2	SERTA domain containing 2	159,3	310,7	**1,95**	0,000605
VHL	von Hippel-Lindau tumor suppressor	26,8	49,7	**1,95**	1,00E-06
C4orf32	chromosome 4 open reading frame 32			**1,95**	1,50E-05
PIGV	phosphatidylinositol glycan anchor biosynthesis class V	14,5	27,8	**1,94**	0
HS3ST3A1	heparan sulfate-glucosamine 3-sulfotransferase 3A1	8,7	16,2	**1,94**	1,50E-05
MSX1	msh homeobox 1	3,6	5,7	**1,94**	0,003409
KRT18P59	keratin 18 pseudogene 59	0,4	0,9	**1,94**	0,037753
STEAP1	STEAP family member 1	30,2	62,4	**1,94**	8,00E-06
TRIM56	tripartite motif containing 56	54,8	104,2	**1,94**	1,20E-05
NR1D1	nuclear receptor subfamily 1 group D member 1	101,5	194,5	**1,94**	0,000206
TREML2	triggering receptor expressed on myeloid cells like 2	0,6	1,2	**1,94**	0,022612
FNDC3B	fibronectin type III domain containing 3B	307,8	600,5	**1,94**	0,000789
EPHX1	epoxide hydrolase 1	20,0	36,0	**1,94**	0
OSER1	oxidative stress responsive serine rich 1	33,0	61,1	**1,93**	0
KCNK1	potassium two pore domain channel subfamily K member 1	28,0	52,2	**1,93**	1,00E-06
RPS4XP16	ribosomal protein S4X pseudogene 16	0,3	0,6	**1,93**	0,007652
CHRNA10	cholinergic receptor nicotinic alpha 10 subunit	0,5	0,8	**1,93**	0,002205
CFAP57	cilia and flagella associated protein 57	0,3	0,5	**1,93**	0,008666
STK40	serine/threonine kinase 40	60,9	111,4	**1,93**	7,00E-06
KANSL3	KAT8 regulatory NSL complex subunit 3	48,1	88,7	**1,93**	5,00E-06
PPFIBP2	PPFIA binding protein 2	11,3	30,0	**1,93**	0,01556
IL10RB	interleukin 10 receptor subunit beta	0,7	1,3	**1,93**	0,00506
ABCC2	ATP binding cassette subfamily C member 2	0,7	1,4	**1,93**	0,003152
MSS51	MSS51 mitochondrial translational activator	0,6	1,1	**1,92**	0,001373
SMIM3	small integral membrane protein 3	14,2	25,0	**1,92**	1,00E-06
TTC39C	tetratricopeptide repeat domain 39C	5,6	10,1	**1,92**	3,00E-05
PARM1	prostate androgen-regulated mucin-like protein 1	54,7	105,7	**1,92**	2,10E-05
SMIM13	small integral membrane protein 13	8,6	15,7	**1,92**	2,80E-05
GOLGA2P7	golgin A2 pseudogene 7	0,5	0,8	**1,92**	0,007625
NFE2L3	nuclear factor, erythroid 2 like 3	0,2	0,5	**1,92**	0,017112
P2RY11	purinergic receptor P2Y11	1,0	1,7	**1,92**	0,000367
NPC1	NPC intracellular cholesterol transporter 1	113,7	208,9	**1,92**	0,00012

RFPL3S	RFPL3 antisense	0,3	0,6	**1,92**	0,005203
IFNAR1	interferon alpha and beta receptor subunit 1	80,7	150,1	**1,92**	4,80E-05
BIRC2	baculoviral IAP repeat containing 2	98,6	181,3	**1,92**	1,90E-05
ACVR2A	activin A receptor type 2A	7,4	13,4	**1,92**	2,00E-05
SGIP1	SH3 domain GRB2 like endophilin interacting protein 1	1,4	2,2	**1,92**	0,001056
PROSER2	proline and serine rich 2	17,7	30,5	**1,92**	2,00E-06
CNTN4	contactin 4	0,5	0,9	**1,91**	0,012034
DNAJC3-AS1	DNAJC3 antisense RNA 1 (head to head)	191,5	230,1	**1,91**	0,000498
KIAA1324	KIAA1324	0,8	1,2	**1,91**	0,003084
MBP	myelin basic protein	10,1	17,9	**1,91**	0,000922
MYC	MYC proto-oncogene, bHLH transcription factor	32,4	59,7	**1,91**	1,20E-05
BRF2	BRF2, RNA polymerase III transcription initiation factor subunit	21,5	37,7	**1,91**	0
MAPK8IP2	mitogen-activated protein kinase 8 interacting protein 2	4,2	7,8	**1,91**	0,000455
TDRKH	tudor and KH domain containing	3,8	7,5	**1,91**	0,000517
AVIL	advillin	5,2	9,9	**1,91**	0,000202
DEDD2	death effector domain containing 2	18,2	33,7	**1,91**	1,00E-06
ZSWIM6	zinc finger SWIM-type containing 6	13,5	24,9	**1,91**	1,20E-05
EFR3B	EFR3 homolog B	5,4	9,3	**1,90**	0,000269
HNMT	histamine N-methyltransferase	39,7	70,0	**1,90**	0
KMT2E-AS1	KMT2E antisense RNA 1 (head to head)	1,2	2,6	**1,90**	0,005233
PTPRJ	protein tyrosine phosphatase, receptor type J	19,2	35,8	**1,90**	4,00E-05
PIK3CD	phosphatidylinositol-4,5-bisphosphate 3-kinase catalytic subunit delta	7,5	14,4	**1,90**	9,00E-05
KDM3A	lysine demethylase 3A	33,7	61,9	**1,90**	7,00E-06
ALDH3A1	aldehyde dehydrogenase 3 family member A1	4,1	5,2	**1,90**	0,002614
RASSF5	Ras association domain family member 5	4,2	8,0	**1,90**	0,000898
AQP11	aquaporin 11	0,3	0,6	**1,90**	0,01869
DNAJC6	DnaJ heat shock protein family (Hsp40) member C6	6,3	11,0	**1,90**	2,40E-05
TBCC	tubulin folding cofactor C	17,5	31,5	**1,90**	1,00E-06
AMMECR1	Alport syndrome, mental retardation, midface hypoplasia and elliptoc	6,2	11,1	**1,90**	5,70E-05
CCDC181	coiled-coil domain containing 181	1,3	2,4	**1,89**	0,002038
NMB	neuromedin B	9,2	17,4	**1,89**	1,90E-05
DNASE1L2	deoxyribonuclease 1 like 2	0,5	1,0	**1,89**	0,005463
VDR	vitamin D (1,25- dihydroxyvitamin D3) receptor	25,2	45,7	**1,89**	2,00E-05

C19orf38	chromosome 19 open reading frame 38	0,8	1,2	**1,89**	0,001567
PLAC8L1	PLAC8 like 1	0,2	0,4	**1,89**	0,014964
C22orf23	chromosome 22 open reading frame 23	0,6	1,0	**1,89**	0,001039
OSTM1	osteopetrosis associated transmembrane protein 1	43,8	77,4	**1,89**	1,50E-05
EMP1	epithelial membrane protein 1	305,7	528,8	**1,89**	0,000932
BPGM	bisphosphoglycerate mutase	22,8	44,9	**1,89**	3,30E-05
FEM1C	fem-1 homolog C	25,7	49,3	**1,88**	1,30E-05
TTC41P	tetratricopeptide repeat domain 41, pseudogene	0,5	0,9	**1,88**	0,006663
CCDC170	coiled-coil domain containing 170	1,9	3,2	**1,88**	0,006392
FLRT1	fibronectin leucine rich transmembrane protein 1	0,5	0,8	**1,88**	0,008051
UNC13D	unc-13 homolog D	3,3	5,5	**1,88**	0,000155
FLCN	folliculin	25,5	45,6	**1,88**	9,00E-06
SLC25A25	solute carrier family 25 member 25	13,0	21,7	**1,88**	1,00E-06
BEND7	BEN domain containing 7	2,3	3,7	**1,88**	0,000618
OGFOD2	2-oxoglutarate and iron dependent oxygenase domain containing 2	0,9	1,6	**1,88**	0,004372
HIST4H4	histone cluster 4 H4	0,5	0,8	**1,87**	0,016306
PDE4D	phosphodiesterase 4D	14,5	24,9	**1,87**	5,00E-06
EPHA4	EPH receptor A4	7,1	12,2	**1,87**	1,00E-05
ACTN2	actinin alpha 2	0,5	0,8	**1,87**	0,005982
CAV2	caveolin 2	54,0	98,6	**1,87**	7,00E-06
KCTD13	potassium channel tetramerization domain containing 13	8,7	15,2	**1,87**	3,00E-06
CFLAR	CASP8 and FADD like apoptosis regulator	30,9	57,6	**1,87**	1,30E-05
HIST1H1C	histone cluster 1 H1 family member c	35,5	63,7	**1,87**	0
SLC4A4	solute carrier family 4 member 4	4,4	7,3	**1,87**	0,001022
NDRG1	N-myc downstream regulated 1	561,9	1029,3	**1,87**	0,001667
LEAP2	liver enriched antimicrobial peptide 2	0,5	0,9	**1,87**	0,005286
CRYGS	crystallin gamma S	0,7	1,2	**1,87**	0,00302
EPAS1	endothelial PAS domain protein 1	346,8	659,9	**1,87**	0,001437
STK33	serine/threonine kinase 33	0,2	0,3	**1,87**	0,034733
DKFZP434I07	uncharacterized protein DKFZP434I0714			**1,87**	0,000345
KRT8P33	keratin 8 pseudogene 33	0,8	1,3	**1,87**	0,008198
DPF3	double PHD fingers 3	0,4	1,0	**1,86**	0,029295
TLE4	transducin like enhancer of split 4	8,4	15,8	**1,86**	0,000125

TMEM51	transmembrane protein 51	0,7	1,5	**1,86**	0,009791
FOSL2	FOS like 2, AP-1 transcription factor subunit	119,1	209,9	**1,86**	0,000123
MINPP1	multiple inositol-polyphosphate phosphatase 1	30,8	54,3	**1,86**	3,00E-06
NEB	nebulin	0,7	1,3	**1,86**	0,012167
CCDC183-AS:	CCDC183 antisense RNA 1	1,8	3,2	**1,86**	0,003602
STXBP5-AS1	STXBP5 antisense RNA 1	4,4	7,4	**1,86**	0,000563
CDH3	cadherin 3	1,7	3,0	**1,86**	0,019114
LFNG	LFNG O-fucosylpeptide 3-beta-N-acetylglucosaminyltransferase	7,2	13,9	**1,86**	0,000827
IL6ST	interleukin 6 signal transducer	335,8	627,6	**1,86**	0,00209
ARHGEF2	Rho/Rac guanine nucleotide exchange factor 2	209,2	364,1	**1,85**	0,000177
IFNGR1	interferon gamma receptor 1	40,1	71,5	**1,85**	1,00E-06
CCNL1	cyclin L1	25,7	44,7	**1,85**	0
METRNL	meteorin like, glial cell differentiation regulator	6,0	10,6	**1,85**	0,000329
B9D2	B9 protein domain 2	2,9	5,3	**1,85**	0,000334
CP	ceruloplasmin	133,7	229,7	**1,85**	0,002023
STC2	stanniocalcin 2	112,0	202,8	**1,85**	0,000209
LINC01569	long intergenic non-protein coding RNA 1569	0,9	1,6	**1,85**	0,004903
CNBD2	cyclic nucleotide binding domain containing 2	1,4	2,1	**1,85**	0,000286
NPIPB2	nuclear pore complex interacting protein family, member B2	2,0	3,4	**1,85**	0,00259
ARIH2OS	ariadne homolog 2 opposite strand	0,9	1,4	**1,85**	0,001651
MEOX2	mesenchyme homeobox 2	7,0	12,8	**1,85**	0,020544
ZSWIM1	zinc finger SWIM-type containing 1	6,5	11,1	**1,85**	9,60E-05
SYT11	synaptotagmin 11	13,5	24,7	**1,84**	0,000183
TRIB3	tribbles pseudokinase 3	75,9	128,1	**1,84**	6,00E-06
IMPG2	interphotoreceptor matrix proteoglycan 2	0,3	0,7	**1,84**	0,01752
BOLA1	bolA family member 1	9,0	14,9	**1,84**	3,00E-05
C1orf162	chromosome 1 open reading frame 162	0,5	0,7	**1,84**	0,025389
VSIG2	V-set and immunoglobulin domain containing 2	0,3	0,5	**1,84**	0,006659
ZPLD1	zona pellucida like domain containing 1	1,1	1,7	**1,84**	0,001246
MSX2	msh homeobox 2	1,9	3,0	**1,84**	0,000538
LINC00472	long intergenic non-protein coding RNA 472	0,5	1,0	**1,84**	0,004836
SRGN	serglycin	86,1	165,6	**1,84**	1,20E-05
MOB3B	MOB kinase activator 3B	2,8	4,5	**1,84**	0,001116

AVPI1	arginine vasopressin induced 1	21,0	38,9	**1,84**	6,00E-06
SLC22A15	solute carrier family 22 member 15	11,5	20,0	**1,84**	0,000377
IRF2	interferon regulatory factor 2	14,1	25,8	**1,83**	1,00E-06
PCSK5	proprotein convertase subtilisin/kexin type 5	5,3	9,0	**1,83**	0,000762
PLCXD3	phosphatidylinositol specific phospholipase C X domain containing 3	0,8	1,6	**1,83**	0,022369
NXF1	nuclear RNA export factor 1	43,8	76,4	**1,83**	2,00E-06
STIM2	stromal interaction molecule 2	9,3	15,1	**1,83**	1,00E-06
ZNF222	zinc finger protein 222	1,8	3,2	**1,83**	0,006324
FUT8-AS1	FUT8 antisense RNA 1	0,4	0,7	**1,83**	0,005956
C14orf79	chromosome 14 open reading frame 79			**1,83**	8,00E-06
SH3D21	SH3 domain containing 21	14,0	22,9	**1,83**	2,20E-05
PHC2	polyhomeotic homolog 2	112,5	185,4	**1,83**	2,30E-05
PTGER4	prostaglandin E receptor 4	5,9	11,5	**1,83**	0,000927
ERCC6	ERCC excision repair 6, chromatin remodeling factor	8,1	14,7	**1,83**	0,000518
IL20RB	interleukin 20 receptor subunit beta	0,6	1,0	**1,83**	0,004934
PLLP	plasmolipin	0,6	1,2	**1,82**	0,014426
OSGIN1	oxidative stress induced growth inhibitor 1	11,9	20,7	**1,82**	0,000111
SNHG1	small nucleolar RNA host gene 1	21,4	33,4	**1,82**	1,00E-06
KDM4A-AS1	KDM4A antisense RNA 1	0,4	0,7	**1,82**	0,007911
LARGE1	LARGE xylosyl- and glucuronyltransferase 1	6,8	12,4	**1,82**	0,000145
SPRY1	sprouty RTK signaling antagonist 1	0,4	0,7	**1,82**	0,017116
IFNE	interferon epsilon	0,3	0,5	**1,82**	0,031533
BNIP1	BCL2 interacting protein 1	7,3	11,3	**1,82**	3,00E-06
RHOB	ras homolog family member B	73,0	127,2	**1,82**	0,00042
APLF	aprataxin and PNKP like factor	2,7	4,8	**1,82**	0,000438
LRRN1	leucine rich repeat neuronal 1	1,9	2,8	**1,82**	0,042082
MT1F	metallothionein 1F	111,6	178,8	**1,82**	0,000426
CATSPER3	cation channel sperm associated 3	0,4	0,5	**1,82**	0,016286
EIF4A3	eukaryotic translation initiation factor 4A3	68,3	110,4	**1,82**	3,00E-06
EIF1	eukaryotic translation initiation factor 1	536,8	896,4	**1,82**	0,001032
RASSF8	Ras association domain family member 8	57,7	108,6	**1,82**	0,000453
JMJD7-PLA2(	JMJD7-PLA2G4B readthrough	1,4	2,3	**1,82**	0,001175
RNF139-AS1	RNF139 antisense RNA 1 (head to head)	0,7	1,3	**1,81**	0,002173

SCN4A	sodium voltage-gated channel alpha subunit 4	0,3	0,4	**1,81**	0,021063
ZFYVE16	zinc finger FYVE-type containing 16	38,8	67,1	**1,81**	0,00014
CFP	complement factor properdin	0,3	0,4	**1,81**	0,037927
TSC22D1-AS:	TSC22D1 antisense RNA 1	0,2	0,5	**1,81**	0,045911
DDIT4L	DNA damage inducible transcript 4 like	50,5	86,6	**1,81**	6,90E-05
AK3	adenylate kinase 3	39,0	69,1	**1,81**	5,20E-05
COTL1	coactosin like F-actin binding protein 1	61,9	95,0	**1,81**	9,00E-06
LINC01481	long intergenic non-protein coding RNA 1481	0,1	0,9	**1,81**	0,00623
ZNFX1	zinc finger NFX1-type containing 1	87,2	156,2	**1,81**	0,000489
TMEM205	transmembrane protein 205	19,6	31,4	**1,81**	0
ATP6V1C2	ATPase H+ transporting V1 subunit C2	1,3	2,4	**1,81**	0,003866
SLC33A1	solute carrier family 33 member 1	16,8	28,8	**1,81**	4,00E-06
CEBPB-AS1	CEBPB antisense RNA 1	1,9	3,1	**1,81**	0,000859
MAMDC4	MAM domain containing 4	7,3	11,7	**1,81**	0,000109
POU3F3	POU class 3 homeobox 3	2,2	3,6	**1,81**	0,000286
AIFM2	apoptosis inducing factor, mitochondria associated 2	24,1	41,4	**1,81**	7,00E-06
LINC01138	long intergenic non-protein coding RNA 1138	2,2	3,9	**1,81**	0,000784
SIRT7	sirtuin 7	11,0	17,4	**1,80**	1,00E-06
ELMSAN1	ELM2 and Myb/SANT domain containing 1	18,9	32,2	**1,80**	2,80E-05
FAM161B	family with sequence similarity 161 member B	13,8	23,1	**1,80**	4,90E-05
EPHA2	EPH receptor A2	49,7	73,3	**1,80**	3,80E-05
MPV17L2	MPV17 mitochondrial inner membrane protein like 2	8,9	16,1	**1,80**	2,90E-05
DNHD1	dynein heavy chain domain 1	7,8	13,6	**1,80**	0,000378
ZBTB49	zinc finger and BTB domain containing 49	3,4	5,6	**1,80**	0,000841
ITGB3	integrin subunit beta 3	6,4	9,4	**1,80**	0,001836
NUMB	NUMB, endocytic adaptor protein	75,4	131,9	**1,80**	7,40E-05
CDC42SE1	CDC42 small effector 1	98,6	167,0	**1,80**	6,50E-05
CCR10	C-C motif chemokine receptor 10	0,9	1,3	**1,80**	0,001508
TSPYL2	TSPY like 2	40,8	69,5	**1,80**	6,00E-06
ZNF774	zinc finger protein 774	4,8	8,2	**1,80**	0,000262
LINC00857	long intergenic non-protein coding RNA 857	2,6	4,1	**1,80**	0,000232
DCXR	dicarbonyl and L-xylulose reductase	25,6	43,6	**1,80**	3,00E-06
CCDC87	coiled-coil domain containing 87	0,7	1,4	**1,80**	0,032316

ZNF497	zinc finger protein 497	0,6	0,9	**1,80**	0,015083
PDXP	pyridoxal phosphatase	1,7	3,1	**1,80**	0,001357
COMMD3	COMM domain containing 3	14,2	22,9	**1,80**	2,00E-06
NR2F1	nuclear receptor subfamily 2 group F member 1	0,5	1,2	**1,80**	0,039044
VWCE	von Willebrand factor C and EGF domains	0,3	0,6	**1,79**	0,041922
PTPN2	protein tyrosine phosphatase, non-receptor type 2	21,7	37,7	**1,79**	3,00E-06
FRMD6	FERM domain containing 6	142,2	250,3	**1,79**	0,000538
LACTB	lactamase beta	11,5	19,2	**1,79**	7,00E-06
C3orf58	chromosome 3 open reading frame 58		29,2	**1,79**	1,20E-05
KCNN3	potassium calcium-activated channel subfamily N member 3	1,2	2,7	**1,79**	0,045207
CYP1B1	cytochrome P450 family 1 subfamily B member 1	445,9	779,6	**1,79**	0,009071
NBPF10	NBPF member 10	2,9	5,3	**1,79**	0,009565
ZFAS1	ZNFX1 antisense RNA 1	21,6	33,7	**1,79**	0
SH3BP5-AS1	SH3BP5 antisense RNA 1	3,7	6,6	**1,79**	0,001508
AMPH	amphiphysin	1,3	2,4	**1,79**	0,017996
CHKA	choline kinase alpha	10,5	18,0	**1,79**	2,90E-05
CDKN1C	cyclin dependent kinase inhibitor 1C	0,5	0,8	**1,79**	0,009108
MCL1	MCL1, BCL2 family apoptosis regulator	233,1	390,2	**1,79**	0,000377
SFRP1	secreted frizzled related protein 1	2,4	3,9	**1,78**	0,001651
CHCHD7	coiled-coil-helix-coiled-coil-helix domain containing 7	20,5	35,7	**1,78**	3,00E-06
TTC25	tetratricopeptide repeat domain 25	2,5	4,2	**1,78**	0,002406
PPP2R1B	protein phosphatase 2 scaffold subunit Abeta	24,0	40,4	**1,78**	1,20E-05
TAF4B	TATA-box binding protein associated factor 4b	4,4	7,6	**1,78**	0,00121
ING3	inhibitor of growth family member 3	9,3	14,9	**1,78**	9,60E-05
TET3	tet methylcytosine dioxygenase 3	8,7	14,5	**1,78**	0,00063
LINC01578	long intergenic non-protein coding RNA 1578	55,6	87,8	**1,78**	2,00E-06
MAPK8	mitogen-activated protein kinase 8	20,2	33,9	**1,78**	6,00E-06
APOO	apolipoprotein O	9,5	15,9	**1,78**	4,70E-05
JAG2	jagged 2	1,3	2,1	**1,78**	0,002744
STARD8	StAR related lipid transfer domain containing 8	3,8	4,8	**1,78**	0,005203
ACSL1	acyl-CoA synthetase long-chain family member 1	30,4	54,6	**1,78**	4,00E-06
STX6	syntaxin 6	23,3	37,4	**1,78**	2,00E-06
HINFP	histone H4 transcription factor	9,0	15,2	**1,78**	5,90E-05

CBLN3	cerebellin 3 precursor	4,2	6,6	**1,78**	0,003869
NAV2	neuron navigator 2	49,0	78,9	**1,78**	0,003457
PGBD4	piggyBac transposable element derived 4	5,1	8,5	**1,78**	0,000544
MYLK3	myosin light chain kinase 3	0,3	0,6	**1,78**	0,023577
DUSP10	dual specificity phosphatase 10	7,0	12,5	**1,77**	6,00E-05
RGS16	regulator of G protein signaling 16	31,1	58,8	**1,77**	0,000798
SLC30A1	solute carrier family 30 member 1	150,7	271,5	**1,77**	0,001165
SAMD5	sterile alpha motif domain containing 5	3,4	6,0	**1,77**	0,004355
BCL3	B-cell CLL/lymphoma 3	30,0	54,5	**1,77**	2,00E-05
TMEM9B-AS	TMEM9B antisense RNA 1	1,6	2,4	**1,77**	0,001209
FAM117A	family with sequence similarity 117 member A	5,7	9,7	**1,77**	6,40E-05
TOB1-AS1	TOB1 antisense RNA 1	0,4	0,7	**1,77**	0,035523
PHEX	phosphate regulating endopeptidase homolog, X-linked	1,4	2,0	**1,77**	0,002097
MTG1	mitochondrial ribosome associated GTPase 1	2,2	3,6	**1,77**	0,001722
RASL11A	RAS like family 11 member A	2,1	4,2	**1,77**	0,009337
TMEM79	transmembrane protein 79	3,5	5,1	**1,77**	0,000145
ARF4-AS1	ARF4 antisense RNA 1	0,9	1,5	**1,77**	0,003522
TXNRD1	thioredoxin reductase 1	82,9	140,6	**1,77**	9,00E-05
SSH1	slingshot protein phosphatase 1	77,3	126,9	**1,77**	0,000225
ZMIZ2	zinc finger MIZ-type containing 2	38,7	64,8	**1,77**	0,000125
ZKSCAN5	zinc finger with KRAB and SCAN domains 5	15,3	25,5	**1,77**	0,000101
FMNL1	formin like 1	1,1	1,9	**1,77**	0,031982
CRY1	cryptochrome circadian clock 1	24,6	38,0	**1,76**	4,00E-06
SOCS6	suppressor of cytokine signaling 6	17,4	28,9	**1,76**	8,00E-06
AMOTL2	angiomotin like 2	99,8	163,6	**1,76**	0,000344
CPXM2	carboxypeptidase X, M14 family member 2	25,5	40,8	**1,76**	0,000308
PPM1J	protein phosphatase, Mg2+/Mn2+ dependent 1J	1,0	1,5	**1,76**	0,002221
KLHL25	kelch like family member 25	4,3	6,9	**1,76**	7,00E-04
FOXN2	forkhead box N2	13,4	22,8	**1,76**	0,000343
RAB24	RAB24, member RAS oncogene family	3,4	5,4	**1,76**	0,000379
THAP10	THAP domain containing 10	4,0	5,7	**1,76**	0,00055
MAOB	monoamine oxidase B	92,0	152,9	**1,76**	3,40E-05
EAF1	ELL associated factor 1	21,3	36,1	**1,76**	0,000118

SLC5A3	solute carrier family 5 member 3	316,5	512,3	**1,75**	0,001578
ABCC1	ATP binding cassette subfamily C member 1	3,1	4,7	**1,75**	0,006877
ATP6V0A4	ATPase H+ transporting V0 subunit a4	0,3	0,4	**1,75**	0,01742
VPS37C	VPS37C, ESCRT-I subunit	32,8	53,2	**1,75**	4,00E-06
TMEM198	transmembrane protein 198	5,8	8,6	**1,75**	0,000121
WDR97	WD repeat domain 97	0,7	1,1	**1,75**	0,020183
SMURF1	SMAD specific E3 ubiquitin protein ligase 1	27,7	44,7	**1,75**	5,20E-05
TMEM170A	transmembrane protein 170A	10,3	17,1	**1,75**	2,30E-05
CCDC30	coiled-coil domain containing 30	1,7	2,4	**1,75**	0,000927
TAF3	TATA-box binding protein associated factor 3	17,2	26,5	**1,75**	4,00E-06
SLC25A33	solute carrier family 25 member 33	6,5	10,3	**1,75**	3,40E-05
MKNK2	MAP kinase interacting serine/threonine kinase 2	94,6	152,0	**1,75**	8,00E-06
GCC1	GRIP and coiled-coil domain containing 1	25,6	41,8	**1,75**	2,10E-05
CHKB	choline kinase beta	0,5	0,8	**1,75**	0,003553
FZD5	frizzled class receptor 5	10,3	17,1	**1,75**	0,000128
RASAL2-AS1	RASAL2 antisense RNA 1	1,0	1,7	**1,75**	0,010307
N4BP2L1	NEDD4 binding protein 2 like 1	4,5	7,4	**1,75**	0,000523
HIPK2	homeodomain interacting protein kinase 2	49,2	86,7	**1,75**	0,001573
BCDIN3D	BCDIN3 domain containing RNA methyltransferase	7,5	12,5	**1,75**	9,90E-05
GEM	GTP binding protein overexpressed in skeletal muscle	56,3	90,9	**1,74**	0,00011
NINJ1	ninjurin 1	65,9	108,9	**1,74**	0,000144
AKIRIN2	akirin 2	38,9	57,9	**1,74**	1,00E-06
HIVEP1	human immunodeficiency virus type I enhancer binding protein 1	21,5	41,7	**1,74**	0,005794
INTS6-AS1	INTS6 antisense RNA 1	1,2	2,0	**1,74**	0,001965
PNRC2	proline rich nuclear receptor coactivator 2	65,3	106,9	**1,74**	4,20E-05
TRAF3	TNF receptor associated factor 3	38,6	63,8	**1,74**	7,00E-05
DOK3	docking protein 3	0,9	1,4	**1,74**	0,006724
LINC01600	long intergenic non-protein coding RNA 1600	0,2	0,4	**1,74**	0,029676
WASH2P	WAS protein family homolog 2 pseudogene	1,5	2,2	**1,74**	0,00186
RICTOR	RPTOR independent companion of MTOR complex 2	25,3	45,3	**1,74**	0,000689
LMTK2	lemur tyrosine kinase 2	16,7	27,8	**1,74**	0,000951
LINC01605	long intergenic non-protein coding RNA 1605	1,5	2,4	**1,74**	0,007029
KLF4	Kruppel like factor 4	85,9	138,2	**1,74**	4,80E-05

GTF2B	general transcription factor IIB	19,8	32,1	**1,74**	2,00E-06
ZFAT	zinc finger and AT-hook domain containing	7,2	11,3	**1,74**	0,000164
APOL2	apolipoprotein L2	30,4	51,8	**1,74**	0,000152
CLIP1	CAP-Gly domain containing linker protein 1	136,3	223,9	**1,74**	0,001393
SLC9A8	solute carrier family 9 member A8	25,1	40,6	**1,73**	3,30E-05
ACO1	aconitase 1	49,7	83,5	**1,73**	2,40E-05
CNOT4	CCR4-NOT transcription complex subunit 4	18,7	31,4	**1,73**	6,70E-05
C17orf100	chromosome 17 open reading frame 100	1,3	2,2	**1,73**	0,012527
KSR1	kinase suppressor of ras 1	6,6	10,4	**1,73**	0,000196
TMEM189	transmembrane protein 189	14,1	22,3	**1,73**	7,00E-06
STAC3	SH3 and cysteine rich domain 3	0,6	0,8	**1,73**	0,01332
CLDN15	claudin 15	3,0	4,8	**1,73**	0,000376
TFPI	tissue factor pathway inhibitor	27,1	41,5	**1,73**	6,20E-05
GTF2IRD1P1	GTF2I repeat domain containing 1 pseudogene 1	0,3	0,4	**1,73**	0,038502
RHOF	ras homolog family member F, filopodia associated	7,4	11,3	**1,73**	0,000355
KLHL15	kelch like family member 15	20,6	32,1	**1,73**	1,50E-05
TAX1BP3	Tax1 binding protein 3	5,3	8,2	**1,73**	0,000409
FZD10-AS1	FZD10 antisense RNA 1 (head to head)	1,1	6,5	**1,73**	0,006379
ZFYVE27	zinc finger FYVE-type containing 27	22,1	34,5	**1,73**	0
RAP1B	RAP1B, member of RAS oncogene family	35,9	57,4	**1,73**	1,60E-05
SMAD7	SMAD family member 7	45,2	69,4	**1,73**	4,00E-06
LINC00174	long intergenic non-protein coding RNA 174	3,9	6,8	**1,73**	0,00496
TUBB4B	tubulin beta 4B class IVb	274,9	391,8	**1,72**	0,0021
LYST	lysosomal trafficking regulator	15,2	26,4	**1,72**	0,001692
MEGF10	multiple EGF like domains 10	8,7	13,7	**1,72**	0,003145
FAM118B	family with sequence similarity 118 member B	8,5	13,1	**1,72**	0,000102
SLC25A19	solute carrier family 25 member 19	4,1	6,4	**1,72**	0,000161
NFE2L1	nuclear factor, erythroid 2 like 1	772,5	1260,2	**1,72**	0,010273
TMEM184B	transmembrane protein 184B	96,9	151,3	**1,72**	2,30E-05
ALG2	ALG2, alpha-1,3/1,6-mannosyltransferase	45,7	72,4	**1,72**	6,00E-06
SRRM2-AS1	SRRM2 antisense RNA 1	0,9	1,2	**1,72**	0,008191
GPATCH2	G-patch domain containing 2	9,5	15,0	**1,72**	0,000159
GRAMD3	GRAM domain containing 3			**1,72**	3,20E-05

PICART1	p53-inducible cancer-associated RNA transcript 1	0,3	0,4	**1,72**	0,042461
LYRM4	LYR motif containing 4	18,8	30,0	**1,72**	8,00E-06
FOSB	FosB proto-oncogene, AP-1 transcription factor subunit	5,1	7,5	**1,72**	0,000485
PCF11	PCF11 cleavage and polyadenylation factor subunit	43,5	68,0	**1,72**	3,00E-05
ACER2	alkaline ceramidase 2	0,7	0,9	**1,72**	0,006659
DNAL4	dynein axonemal light chain 4	10,4	16,2	**1,72**	3,70E-05
BCL7A	BCL tumor suppressor 7A	0,7	1,2	**1,72**	0,022347
LINC01003	long intergenic non-protein coding RNA 1003	4,2	6,5	**1,72**	0,000399
LAMTOR5-AS	LAMTOR5 antisense RNA 1	0,9	1,4	**1,72**	0,008012
DOCK3	dedicator of cytokinesis 3	0,5	0,8	**1,72**	0,032462
DEPTOR	DEP domain containing MTOR interacting protein	0,7	1,2	**1,71**	0,012363
IGSF9B	immunoglobulin superfamily member 9B	3,6	6,0	**1,71**	0,003349
CHMP5	charged multivesicular body protein 5	63,2	96,8	**1,71**	6,00E-06
SERPINE2	serpin family E member 2	5548,3	9362,9	**1,71**	0,04362
C20orf196	chromosome 20 open reading frame 196			**1,71**	0,001937
NABP1	nucleic acid binding protein 1	17,7	29,2	**1,71**	1,40E-05
CEP85L	centrosomal protein 85 like	6,2	10,0	**1,71**	0,001937
ASIC3	acid sensing ion channel subunit 3	0,4	0,6	**1,71**	0,013403
KCTD11	potassium channel tetramerization domain containing 11	17,9	28,6	**1,71**	2,70E-05
OSMR-AS1	OSMR antisense RNA 1 (head to head)	1,0	1,6	**1,71**	0,004062
STARD10	StAR related lipid transfer domain containing 10	20,7	33,8	**1,71**	1,60E-05
MARS2	methionyl-tRNA synthetase 2, mitochondrial	3,8	5,7	**1,71**	0,001546
ZNF337-AS1	ZNF337 antisense RNA 1	1,5	2,1	**1,71**	0,002823
WNT11	Wnt family member 11	0,9	1,2	**1,71**	0,030742
UBE2O	ubiquitin conjugating enzyme E2 O	40,8	64,2	**1,71**	1,80E-05
ABL1	ABL proto-oncogene 1, non-receptor tyrosine kinase	59,3	96,7	**1,71**	0,000202
CXCL16	C-X-C motif chemokine ligand 16	7,1	11,7	**1,71**	0,00269
ANK1	ankyrin 1	0,3	0,5	**1,71**	0,042158
ZNF649	zinc finger protein 649	4,9	8,0	**1,71**	0,003146
BTG1	BTG anti-proliferation factor 1	511,1	813,0	**1,70**	0,007345
IER2	immediate early response 2	67,4	100,3	**1,70**	1,30E-05
BSN	bassoon presynaptic cytomatrix protein	3,0	4,7	**1,70**	0,018774
UFM1	ubiquitin fold modifier 1	75,9	117,3	**1,70**	2,70E-05

ODC1	ornithine decarboxylase 1	200,0	311,0	**1,70**	0,000351
SLC6A8	solute carrier family 6 member 8	55,4	95,7	**1,70**	1,40E-05
ADPRM	ADP-ribose/CDP-alcohol diphosphatase, manganese dependent	4,7	7,4	**1,70**	0,000629
RPS6KA2	ribosomal protein S6 kinase A2	44,8	72,9	**1,70**	7,80E-05
DOPEY2	dopey family member 2			**1,70**	0,005289
CLDND1	claudin domain containing 1	36,9	60,5	**1,70**	1,10E-05
ACTA2	actin, alpha 2, smooth muscle, aorta	1,5	1,9	**1,70**	0,001386
SPTBN5	spectrin beta, non-erythrocytic 5	1,1	1,4	**1,70**	0,026231
SIRT1	sirtuin 1	20,9	34,3	**1,70**	0,000253
TESK2	testis-specific kinase 2	7,8	12,3	**1,70**	0,000145
MYL5	myosin light chain 5	5,9	8,3	**1,70**	7,70E-05
RIOK3	RIO kinase 3	71,5	109,8	**1,70**	8,50E-05
NR4A2	nuclear receptor subfamily 4 group A member 2	10,7	19,4	**1,70**	0,001307
RGS5	regulator of G-protein signaling 5	3,3	2,7	**1,70**	0,031019
FSTL1	follistatin like 1	800,0	1379,1	**1,70**	0,013618
POPDC2	popeye domain containing 2	0,8	1,2	**1,70**	0,016113
ZHX2	zinc fingers and homeoboxes 2	46,9	75,4	**1,69**	0,000254
LPAR1	lysophosphatidic acid receptor 1	106,8	182,6	**1,69**	0,000708
POC1B-AS1	POC1B antisense RNA 1	1,1	1,8	**1,69**	0,014257
TRAPPC5	trafficking protein particle complex 5	0,4	0,7	**1,69**	0,013769
COQ10B	coenzyme Q10B	34,6	55,2	**1,69**	2,10E-05
C8orf76	chromosome 8 open reading frame 76	1,1	1,8	**1,69**	0,008051
PSMA6	proteasome subunit alpha 6	3,3	5,2	**1,69**	0,000982
MTF1	metal regulatory transcription factor 1	19,3	32,6	**1,69**	0,000639
MRPL38	mitochondrial ribosomal protein L38	1,1	1,6	**1,69**	0,003408
ZNF829	zinc finger protein 829	3,2	5,2	**1,69**	0,003603
TRIM11	tripartite motif containing 11	19,4	29,3	**1,69**	7,00E-06
TUT1	terminal uridylyl transferase 1, U6 snRNA-specific	6,8	10,5	**1,69**	0,000164
GSK3B	glycogen synthase kinase 3 beta	47,7	78,3	**1,69**	0,000226
ZNF597	zinc finger protein 597	5,0	7,8	**1,69**	0,001769
ALDOA	aldolase, fructose-bisphosphate A	7,5	11,2	**1,69**	0,000352
C19orf54	chromosome 19 open reading frame 54	7,7	11,4	**1,69**	0,00021
JAGN1	jagunal homolog 1	26,7	39,7	**1,68**	1,00E-06

IPMK	inositol polyphosphate multikinase	5,2	8,6	**1,68**	0,001605
PDRG1	p53 and DNA damage regulated 1	14,6	21,9	**1,68**	4,70E-05
LINC02104	long intergenic non-protein coding RNA 2104	0,6	1,0	**1,68**	0,010377
SEMA4B	semaphorin 4B	24,7	38,4	**1,68**	0,001006
ZNF143	zinc finger protein 143	12,6	20,0	**1,68**	9,80E-05
LINC-PINT	long intergenic non-protein coding RNA, p53 induced transcript	3,7	7,6	**1,68**	0,001469
SRGAP3	SLIT-ROBO Rho GTPase activating protein 3	1,8	2,4	**1,68**	0,0033
SCARNA13	small Cajal body-specific RNA 13	0,3	0,5	**1,68**	0,030415
TBL1X	transducin beta like 1X-linked	29,2	49,5	**1,68**	0,001019
RNASEK	ribonuclease K	0,9	1,7	**1,68**	0,006663
SIGLEC15	sialic acid binding Ig like lectin 15	1,2	1,5	**1,68**	0,004015
LRRC46	leucine rich repeat containing 46	0,6	1,0	**1,68**	0,014611
TNFSF10	TNF superfamily member 10	1,6	2,7	**1,68**	0,018098
CCDC130	coiled-coil domain containing 130	16,4	25,1	**1,68**	2,10E-05
SAMD15	sterile alpha motif domain containing 15	1,0	1,7	**1,68**	0,010256
SIK2	salt inducible kinase 2	40,9	66,2	**1,68**	0,001179
RNF181	ring finger protein 181	35,3	51,9	**1,68**	5,00E-06
FBXL19-AS1	FBXL19 antisense RNA 1 (head to head)	2,8	4,5	**1,68**	0,006501
RNF149	ring finger protein 149	25,0	40,0	**1,67**	1,90E-05
PNPLA8	patatin like phospholipase domain containing 8	39,8	61,5	**1,67**	1,00E-05
GALNT13	polypeptide N-acetylgalactosaminyltransferase 13	2,2	3,3	**1,67**	0,003866
SPRY4	sprouty RTK signaling antagonist 4	37,7	56,9	**1,67**	0,000124
SFR1	SWI5 dependent homologous recombination repair protein 1	10,6	15,7	**1,67**	6,10E-05
ITPR1	inositol 1,4,5-trisphosphate receptor type 1	11,2	18,4	**1,67**	0,003706
USP12	ubiquitin specific peptidase 12	42,3	72,0	**1,67**	0,00016
SMS	spermine synthase	45,8	64,6	**1,67**	1,00E-06
EP300	E1A binding protein p300	58,4	99,0	**1,67**	0,006131
WHRN	whirlin	4,0	5,9	**1,67**	0,001135
ELL	elongation factor for RNA polymerase II	18,6	28,4	**1,67**	4,70E-05
CRTAC1	cartilage acidic protein 1	1031,8	1572,6	**1,67**	0,014354
CNNM2	cyclin and CBS domain divalent metal cation transport mediator 2	11,1	17,0	**1,67**	0,000144
ARL4A	ADP ribosylation factor like GTPase 4A	2,8	4,2	**1,67**	0,006134
CLK4	CDC like kinase 4	11,3	17,7	**1,67**	0,00012

NOTCH2	notch 2	550,4	907,2	**1,67**	0,030674
TOB2P1	transducer of ERBB2, 2 pseudogene 1	0,4	0,7	**1,67**	0,041143
RFK	riboflavin kinase	14,5	22,7	**1,67**	8,60E-05
KDM5C	lysine demethylase 5C	92,8	141,5	**1,67**	0,001042
PI4K2A	phosphatidylinositol 4-kinase type 2 alpha	27,1	42,7	**1,67**	1,90E-05
FLJ37453	uncharacterized LOC729614		4,5	**1,67**	0,000817
USP54	ubiquitin specific peptidase 54	8,8	13,6	**1,67**	0,000869
ANKRD37	ankyrin repeat domain 37	8,4	13,4	**1,67**	0,000312
GORAB	golgin, RAB6 interacting	20,6	30,8	**1,67**	7,00E-06
MAP4K4	mitogen-activated protein kinase kinase kinase kinase 4	153,9	225,7	**1,67**	0,000556
UBA6-AS1	UBA6 antisense RNA 1 (head to head)	9,1	14,2	**1,66**	0,000459
PNPLA2	patatin like phospholipase domain containing 2	120,0	193,6	**1,66**	0,000132
SLC4A5	solute carrier family 4 member 5	0,5	0,8	**1,66**	0,016873
GNAI1	G protein subunit alpha i1	5,7	8,5	**1,66**	0,000406
ITGA5	integrin subunit alpha 5	542,7	780,3	**1,66**	0,016187
ETV5	ETS variant 5	51,4	78,2	**1,66**	2,80E-05
SORCS2	sortilin related VPS10 domain containing receptor 2	1,1	2,0	**1,66**	0,041641
WFDC2	WAP four-disulfide core domain 2	0,9	1,1	**1,66**	0,013559
ISG15	ISG15 ubiquitin-like modifier	2,9	4,2	**1,66**	0,012791
ITSN2	intersectin 2	35,6	56,8	**1,66**	0,000379
ITPR3	inositol 1,4,5-trisphosphate receptor type 3	198,9	309,3	**1,66**	0,008309
ZNF10	zinc finger protein 10	3,6	5,3	**1,66**	0,001922
BRICD5	BRICHOS domain containing 5	1,3	1,9	**1,66**	0,004712
PDLIM7	PDZ and LIM domain 7	60,0	91,1	**1,66**	0,000752
B2M	beta-2-microglobulin	42,2	63,3	**1,66**	2,00E-06
LSM6	LSM6 homolog, U6 small nuclear RNA and mRNA degradation associa	11,3	16,5	**1,66**	7,30E-05
SYNE1	spectrin repeat containing nuclear envelope protein 1	118,9	181,9	**1,66**	0,00462
CMIP	c-Maf inducing protein	16,2	24,7	**1,66**	0,000639
GTDC1	glycosyltransferase like domain containing 1	14,5	22,3	**1,65**	3,60E-05
MIPEPP3	mitochondrial intermediate peptidase pseudogene 3	0,8	1,1	**1,65**	0,017884
SLC35F2	solute carrier family 35 member F2	4,3	5,7	**1,65**	0,002353
NDUFV2-AS1	NDUFV2 antisense RNA 1	1,5	2,3	**1,65**	0,003576
FAM180A	family with sequence similarity 180 member A	32,2	52,3	**1,65**	0,00031

HEXIM1	hexamethylene bisacetamide inducible 1	57,3	90,6	**1,65**	0,000128
LIME1	Lck interacting transmembrane adaptor 1	0,8	1,1	**1,65**	0,010448
PAWR	pro-apoptotic WT1 regulator	1,5	2,5	**1,65**	0,04028
IRGQ	immunity related GTPase Q	15,9	24,6	**1,65**	8,20E-05
IGHMBP2	immunoglobulin mu binding protein 2	17,1	25,2	**1,65**	6,20E-05
LINC01125	long intergenic non-protein coding RNA 1125			**1,65**	0,012636
CSGALNACT2	chondroitin sulfate N-acetylgalactosaminyltransferase 2	61,9	96,4	**1,65**	0,00016
RN7SL2	RNA, 7SL, cytoplasmic 2	10,4	16,4	**1,65**	0,034499
GTF2IRD2B	GTF2I repeat domain containing 2B	2,3	3,2	**1,65**	0,001897
WHAMM	WAS protein homolog associated with actin, golgi membranes and mi	18,3	27,3	**1,65**	3,80E-05
CATSPER2	cation channel sperm associated 2	1,4	2,1	**1,65**	0,006695
ATP1B2	ATPase Na+/K+ transporting subunit beta 2	2,0	3,4	**1,65**	0,010004
UNKL	unkempt family like zinc finger	38,6	58,2	**1,65**	8,30E-05
KLHL5	kelch like family member 5	21,6	33,5	**1,65**	0,000348
MT1DP	metallothionein 1D, pseudogene	1,0	1,7	**1,65**	0,033538
C20orf204	chromosome 20 open reading frame 204	0,4	0,7	**1,65**	0,035735
MIEN1	migration and invasion enhancer 1	19,1	28,9	**1,65**	1,10E-05
LGALSL	galectin like	19,8	29,8	**1,65**	4,10E-05
ICAM2	intercellular adhesion molecule 2	16,7	23,0	**1,65**	3,70E-05
DDX28	DEAD-box helicase 28	6,4	9,0	**1,65**	0,000411
EFL1	elongation factor like GTPase 1	33,4	52,4	**1,65**	0,000141
SF3B5	splicing factor 3b subunit 5	55,4	80,9	**1,65**	2,70E-05
IFT43	intraflagellar transport 43	15,1	21,8	**1,65**	7,00E-06
KCNG1	potassium voltage-gated channel modifier subfamily G member 1	6,4	9,0	**1,64**	0,002104
DAPK2	death associated protein kinase 2	14,7	23,0	**1,64**	7,40E-05
RNF113A	ring finger protein 113A	11,5	16,5	**1,64**	0,000251
BNIP3L	BCL2 interacting protein 3 like	185,7	293,2	**1,64**	0,001389
CHI3L2	chitinase 3 like 2	1206,8	1711,4	**1,64**	0,028113
JAM2	junctional adhesion molecule 2	3,5	5,3	**1,64**	0,005093
NCEH1	neutral cholesterol ester hydrolase 1	9,4	14,1	**1,64**	0,001591
FLJ20021	uncharacterized LOC90024		19,2	**1,64**	0,000197
KANSL1L	KAT8 regulatory NSL complex subunit 1 like	19,1	29,6	**1,64**	0,000401
GPR108	G protein-coupled receptor 108	67,1	103,5	**1,64**	8,80E-05

C5orf22	chromosome 5 open reading frame 22	12,1	18,2	**1,64**	0,000387
GEMIN8P4	gem nuclear organelle associated protein 8 pseudogene 4	0,6	0,8	**1,64**	0,03112
SDCBP2	syndecan binding protein 2	1,8	2,6	**1,64**	0,008969
ADSS	adenylosuccinate synthase	83,6	123,0	**1,64**	9,90E-05
FAM155A	family with sequence similarity 155 member A	2,1	3,2	**1,64**	0,03888
PLAGL2	PLAG1 like zinc finger 2	12,6	19,2	**1,64**	0,000602
THG1L	tRNA-histidine guanylyltransferase 1 like	11,4	16,1	**1,64**	3,30E-05
ALOX12	arachidonate 12-lipoxygenase, 12S type	1,0	1,3	**1,64**	0,020535
SGK494	uncharacterized serine/threonine-protein kinase SgK494		2,2	**1,64**	0,009177
ATP6V0A1	ATPase H+ transporting V0 subunit a1	58,6	90,1	**1,64**	0,000258
PIWIL2	piwi like RNA-mediated gene silencing 2	3,3	4,5	**1,64**	0,002354
GGT1	gamma-glutamyltransferase 1	0,7	0,9	**1,64**	0,013377
PAIP2B	poly(A) binding protein interacting protein 2B	3,0	4,4	**1,64**	0,002737
TFIP11	tuftelin interacting protein 11	33,0	50,5	**1,64**	3,80E-05
GPATCH3	G-patch domain containing 3	8,6	12,8	**1,64**	0,000168
PGBD2	piggyBac transposable element derived 2	3,9	5,7	**1,64**	0,003461
TSGA10	testis specific 10	1,7	2,6	**1,64**	0,006009
CRY2	cryptochrome circadian clock 2	26,5	40,7	**1,64**	7,40E-05
ZNF674	zinc finger protein 674	3,5	5,1	**1,64**	0,002879
SUCO	SUN domain containing ossification factor	65,3	99,1	**1,64**	0,00037
LINC00240	long intergenic non-protein coding RNA 240	0,4	0,6	**1,64**	0,027625
KLHL35	kelch like family member 35	2,1	3,1	**1,63**	0,012048
SLC7A5	solute carrier family 7 member 5	140,0	218,9	**1,63**	0,002827
FNIP1	folliculin interacting protein 1	32,3	49,4	**1,63**	0,001381
IL1RL1	interleukin 1 receptor like 1	17,7	24,2	**1,63**	0,000282
SH2D4A	SH2 domain containing 4A	25,7	39,2	**1,63**	0,00021
SLC4A7	solute carrier family 4 member 7	32,1	47,3	**1,63**	4,70E-05
GZF1	GDNF inducible zinc finger protein 1	16,8	25,6	**1,63**	3,00E-05
DZANK1	double zinc ribbon and ankyrin repeat domains 1	2,6	3,9	**1,63**	0,005883
ZFYVE26	zinc finger FYVE-type containing 26	20,7	32,0	**1,63**	0,004499
ZNF574	zinc finger protein 574	15,7	23,1	**1,63**	0,00011
TCEA1	transcription elongation factor A1	59,2	86,2	**1,63**	1,50E-05
SMURF2	SMAD specific E3 ubiquitin protein ligase 2	19,0	28,3	**1,63**	0,000181

CLDN12	claudin 12	54,3	83,3	**1,63**	0,000379
ZBTB11	zinc finger and BTB domain containing 11	24,1	35,5	**1,63**	9,70E-05
FADD	Fas associated via death domain	34,4	49,4	**1,63**	3,00E-05
NBPF14	NBPF member 14	7,3	11,1	**1,63**	0,005071
FAM225A	family with sequence similarity 225 member A (non-protein coding)	2,6	3,6	**1,63**	0,003864
WASH3P	WAS protein family homolog 3 pseudogene	1,3	1,8	**1,63**	0,030132
ZMAT1	zinc finger matrin-type 1	4,1	6,0	**1,63**	0,002202
ZNF460	zinc finger protein 460	1,9	2,9	**1,63**	0,009652
GATA3	GATA binding protein 3	1,0	1,3	**1,63**	0,035367
CYP2T1P	cytochrome P450 family 2 subfamily T member 1, pseudogene	2,4	3,6	**1,63**	0,004157
HLA-B	major histocompatibility complex, class I, B	18,7	31,2	**1,63**	0,008051
CCND1	cyclin D1	336,0	437,6	**1,62**	0,010025
THUMPD3-A	THUMPD3 antisense RNA 1	6,6	9,6	**1,62**	0,000148
SAMD9	sterile alpha motif domain containing 9	7,4	12,4	**1,62**	0,016712
INHBE	inhibin beta E subunit	0,9	1,4	**1,62**	0,037861
IQUB	IQ motif and ubiquitin domain containing	0,4	0,5	**1,62**	0,045353
PTP4A2	protein tyrosine phosphatase type IVA, member 2	150,7	221,0	**1,62**	0,000561
SH3TC2	SH3 domain and tetratricopeptide repeats 2	4,6	5,5	**1,62**	0,003032
C18orf25	chromosome 18 open reading frame 25	17,2	26,3	**1,62**	0,000379
CADM1	cell adhesion molecule 1	3,0	5,3	**1,62**	0,010813
SLC25A44	solute carrier family 25 member 44	17,2	24,0	**1,62**	2,90E-05
SULT1C4	sulfotransferase family 1C member 4	1,1	1,7	**1,62**	0,02114
TMEM38B	transmembrane protein 38B	20,0	30,7	**1,62**	0,000204
MICALL1	MICAL like 1	37,8	56,3	**1,62**	0,000505
ZBTB40	zinc finger and BTB domain containing 40	28,6	43,7	**1,62**	0,001509
ZNF420	zinc finger protein 420	5,3	8,1	**1,62**	0,003284
KCTD5	potassium channel tetramerization domain containing 5	35,8	49,4	**1,62**	1,50E-05
STXBP6	syntaxin binding protein 6	1,0	1,3	**1,62**	0,022727
CCNO	cyclin O	7,0	9,0	**1,62**	0,00075
MYO10	myosin X	121,9	198,2	**1,62**	0,00391
IGSF3	immunoglobulin superfamily member 3	16,6	26,1	**1,62**	0,002092
NPTN-IT1	NPTN intronic transcript 1	1,2	1,9	**1,62**	0,045677
SENP8	SUMO/sentrin peptidase family member, NEDD8 specific	7,0	10,3	**1,62**	0,000806

TMEM44	transmembrane protein 44	4,9	6,8	**1,62**	0,001436
ZBTB1	zinc finger and BTB domain containing 1	34,6	51,1	**1,62**	0,000114
RCE1	Ras converting CAAX endopeptidase 1	10,3	14,0	**1,62**	8,50E-05
KLKB1	kallikrein B1	0,7	0,9	**1,62**	0,022486
JMJD1C	jumonji domain containing 1C	59,9	93,5	**1,62**	0,005992
RRNAD1	ribosomal RNA adenine dimethylase domain containing 1	17,5	25,3	**1,62**	2,10E-05
ABHD17B	abhydrolase domain containing 17B	6,0	8,4	**1,61**	0,000422
BTN2A1	butyrophilin subfamily 2 member A1	27,5	42,7	**1,61**	0,000428
RBBP6	RB binding protein 6, ubiquitin ligase	48,1	70,0	**1,61**	2,70E-05
ZCCHC2	zinc finger CCHC-type containing 2	8,6	13,1	**1,61**	0,001068
PPM1D	protein phosphatase, Mg2+/Mn2+ dependent 1D	12,9	19,0	**1,61**	0,000411
RAD51-AS1	RAD51 antisense RNA 1 (head to head)	1,5	2,1	**1,61**	0,005258
LOXL4	lysyl oxidase like 4	231,8	357,8	**1,61**	0,010915
TMEM99	transmembrane protein 99	14,1	19,4	**1,61**	3,70E-05
NOP56	NOP56 ribonucleoprotein	72,3	101,7	**1,61**	0,000392
GCNA	germ cell nuclear acidic peptidase	1,5	2,5	**1,61**	0,011629
TRPV1	transient receptor potential cation channel subfamily V member 1	2,2	3,5	**1,61**	0,019272
PRDM10	PR/SET domain 10	8,9	12,4	**1,61**	0,000701
ATP6V1B2	ATPase H+ transporting V1 subunit B2	99,8	144,2	**1,60**	0,000597
DCUN1D2	defective in cullin neddylation 1 domain containing 2	7,3	10,9	**1,60**	0,00059
DNAJB6	DnaJ heat shock protein family (Hsp40) member B6	60,4	91,7	**1,60**	0,000387
SNX33	sorting nexin 33	75,3	112,4	**1,60**	0,000538
ZNF18	zinc finger protein 18	5,4	7,8	**1,60**	0,001814
MARK1	microtubule affinity regulating kinase 1	3,3	5,0	**1,60**	0,018069
CD59	CD59 molecule (CD59 blood group)	709,6	1030,3	**1,60**	0,011934
LCA5L	LCA5L, lebercilin like	1,9	2,7	**1,60**	0,009189
C8orf88	chromosome 8 open reading frame 88	7,9	11,7	**1,60**	0,001007
RBM14	RNA binding motif protein 14	38,4	57,7	**1,60**	5,00E-05
ZNF79	zinc finger protein 79	5,1	7,7	**1,60**	0,005144
ZMYND8	zinc finger MYND-type containing 8	23,3	31,6	**1,60**	4,70E-05
AP3M2	adaptor related protein complex 3 mu 2 subunit	11,0	15,4	**1,60**	0,000322
IFIT3	interferon induced protein with tetratricopeptide repeats 3	24,1	36,1	**1,60**	0,001078
DNAJC1	DnaJ heat shock protein family (Hsp40) member C1	55,5	78,9	**1,60**	3,20E-05

RRAS2	related RAS viral (r-ras) oncogene homolog 2	23,9	34,7	**1,60**	0,000212
PAXBP1-AS1	PAXBP1 antisense RNA 1	1,8	2,4	**1,60**	0,006346
PKMP3	pyruvate kinase, muscle pseudogene 3	0,9	1,2	**1,60**	0,017476
ANXA5	annexin A5	625,6	889,1	**1,60**	0,011435
SPATA5L1	spermatogenesis associated 5 like 1	8,2	12,5	**1,60**	0,001287
OARD1	O-acyl-ADP-ribose deacylase 1	17,5	24,1	**1,60**	3,80E-05
ALDH1A3	aldehyde dehydrogenase 1 family member A3	58,6	84,6	**1,60**	0,000443
ZBTB43	zinc finger and BTB domain containing 43	15,7	23,7	**1,60**	0,001283
NCK1-AS1	NCK1 antisense RNA 1 (head to head)	24,6	78,3	**1,60**	0,044679
FAM53C	family with sequence similarity 53 member C	27,4	40,6	**1,60**	3,20E-05
TMEM222	transmembrane protein 222	34,4	47,6	**1,59**	2,20E-05
ARL8B	ADP ribosylation factor like GTPase 8B	77,7	109,1	**1,59**	0,00015
SNIP1	Smad nuclear interacting protein 1	19,3	28,9	**1,59**	0,000233
FBXL20	F-box and leucine rich repeat protein 20	13,6	21,0	**1,59**	0,001004
VEGFC	vascular endothelial growth factor C	9,6	14,6	**1,59**	0,013032
CREBBP	CREB binding protein	67,3	102,1	**1,59**	0,00681
CDC26	cell division cycle 26	4,5	6,1	**1,59**	0,000625
STXBP1	syntaxin binding protein 1	54,6	80,8	**1,59**	6,40E-05
VPS13A	vacuolar protein sorting 13 homolog A	22,3	35,0	**1,59**	0,007289
STAM	signal transducing adaptor molecule	24,1	34,6	**1,59**	0,000313
FAM221A	family with sequence similarity 221 member A	1,8	2,3	**1,59**	0,005612
CCDC174	coiled-coil domain containing 174	16,1	23,4	**1,59**	0,000379
FXR2	FMR1 autosomal homolog 2	43,4	62,9	**1,59**	6,40E-05
ATE1-AS1	ATE1 antisense RNA 1	3,5	5,0	**1,59**	0,002832
FAS	Fas cell surface death receptor	6,1	8,5	**1,59**	0,003702
ZNF24	zinc finger protein 24	58,6	86,3	**1,59**	0,00106
YPEL5	yippee like 5	71,1	103,0	**1,59**	0,000153
USP36	ubiquitin specific peptidase 36	39,0	57,4	**1,59**	7,00E-04
ARHGAP39	Rho GTPase activating protein 39	5,7	8,2	**1,59**	0,002571
FMNL3	formin like 3	29,9	45,3	**1,59**	0,00284
PARS2	prolyl-tRNA synthetase 2, mitochondrial (putative)	4,0	5,6	**1,59**	0,002488
NR2F2	nuclear receptor subfamily 2 group F member 2	2,5	4,3	**1,59**	0,004179
IBA57	IBA57 homolog, iron-sulfur cluster assembly	8,1	10,9	**1,59**	0,000368

CLK3	CDC like kinase 3	30,6	42,6	**1,59**	3,90E-05
PPTC7	PTC7 protein phosphatase homolog	16,0	23,8	**1,59**	0,000356
ELMO3	engulfment and cell motility 3	0,8	1,1	**1,59**	0,03171
CPAMD8	C3 and PZP like, alpha-2-macroglobulin domain containing 8	73,9	109,1	**1,59**	0,001154
APOL1	apolipoprotein L1	17,3	26,4	**1,59**	0,002103
NRIP3	nuclear receptor interacting protein 3	2,0	2,7	**1,58**	0,008176
PIWIL4	piwi like RNA-mediated gene silencing 4	11,9	16,8	**1,58**	0,000218
RSRP1	arginine and serine rich protein 1	37,4	55,8	**1,58**	1,40E-05
CEBPG	CCAAT/enhancer binding protein gamma	44,3	64,3	**1,58**	6,80E-05
C16orf71	chromosome 16 open reading frame 71	1,9	2,6	**1,58**	0,008554
MKLN1-AS	MKLN1 antisense RNA	2,2	2,8	**1,58**	0,005479
DCBLD2	discoidin, CUB and LCCL domain containing 2	28,4	40,2	**1,58**	0,000323
UBE2Z	ubiquitin conjugating enzyme E2 Z	116,4	167,6	**1,58**	0,001046
TMEM199	transmembrane protein 199	12,9	18,5	**1,58**	0,00017
ADAMTS9-AS	ADAMTS9 antisense RNA 2	1,5	2,3	**1,58**	0,013426
SELENOS	selenoprotein S	98,7	140,6	**1,58**	0,000484
SRXN1	sulfiredoxin 1	2,0	3,1	**1,58**	0,026549
ZNF782	zinc finger protein 782	3,4	5,2	**1,58**	0,014973
SLC35E4	solute carrier family 35 member E4	9,0	12,7	**1,58**	0,001251
SNRNP27	small nuclear ribonucleoprotein U4/U6.U5 subunit 27	19,9	28,6	**1,58**	3,40E-05
DMRTA1	DMRT like family A1	11,1	15,1	**1,58**	0,000658
CCNH	cyclin H	19,2	27,2	**1,58**	6,50E-05
CALCA	calcitonin related polypeptide alpha	0,6	0,8	**1,58**	0,020323
TEFM	transcription elongation factor, mitochondrial	6,4	9,0	**1,58**	0,001269
ALOX12-AS1	ALOX12 antisense RNA 1	2,3	3,3	**1,58**	0,016807
PLEKHM1P1	pleckstrin homology and RUN domain containing M1 pseudogene 1	8,7	12,9	**1,58**	0,001668
SNHG17	small nucleolar RNA host gene 17	9,7	12,9	**1,58**	0,00169
PTPRM	protein tyrosine phosphatase, receptor type M	172,0	247,2	**1,58**	0,003262
LINC01715	long intergenic non-protein coding RNA 1715	0,8	1,0	**1,58**	0,016745
TMEM55B	transmembrane protein 55B			**1,58**	1,10E-05
ETHE1	ETHE1, persulfide dioxygenase	10,8	16,5	**1,57**	0,000952
TMEM165	transmembrane protein 165	106,9	161,2	**1,57**	0,000631
NOSIP	nitric oxide synthase interacting protein	28,4	39,8	**1,57**	6,20E-05

DNAJC16	DnaJ heat shock protein family (Hsp40) member C16	25,0	38,3	**1,57**	0,002332
CDKL3	cyclin dependent kinase like 3	0,7	0,9	**1,57**	0,03334
C6orf62	chromosome 6 open reading frame 62	217,4	316,0	**1,57**	0,002861
FZD7	frizzled class receptor 7	29,5	43,5	**1,57**	4,20E-05
JAK1	Janus kinase 1	126,5	185,4	**1,57**	0,001651
HELB	DNA helicase B	2,4	3,7	**1,57**	0,017532
TOB1	transducer of ERBB2, 1	43,2	59,7	**1,57**	1,90E-05
AMD1	adenosylmethionine decarboxylase 1	104,4	149,2	**1,57**	0,000718
CRYBB2P1	crystallin beta B2 pseudogene 1	7,7	10,8	**1,57**	0,002796
IFRD1	interferon related developmental regulator 1	56,5	79,1	**1,57**	0,000152
RNF103	ring finger protein 103	35,5	52,4	**1,57**	0,000117
EIF4A1	eukaryotic translation initiation factor 4A1	1,5	2,1	**1,57**	0,019464
EMSY	EMSY, BRCA2 interacting transcriptional repressor	16,0	24,4	**1,57**	0,005884
MYEOV	myeloma overexpressed	5,0	5,8	**1,57**	0,044244
CRAMP1	cramped chromatin regulator homolog 1	10,3	15,2	**1,57**	0,001131
MREG	melanoregulin	8,4	12,1	**1,57**	0,005094
ZFHX2	zinc finger homeobox 2	0,6	0,8	**1,57**	0,039522
GDPD1	glycerophosphodiester phosphodiesterase domain containing 1	0,6	0,7	**1,57**	0,030762
CRELD1	cysteine rich with EGF like domains 1	23,0	29,5	**1,57**	0,001251
TMEM39A	transmembrane protein 39A	54,0	76,0	**1,57**	0,000132
ZNF841	zinc finger protein 841	9,8	14,6	**1,57**	0,001512
FOXC2	forkhead box C2	41,7	55,6	**1,57**	0,000406
MOCS3	molybdenum cofactor synthesis 3	9,7	12,9	**1,57**	0,000604
ZNF175	zinc finger protein 175	9,5	13,5	**1,57**	0,003295
URGCP	upregulator of cell proliferation	33,0	48,0	**1,57**	0,000176
GXYLT1	glucoside xylosyltransferase 1	25,4	36,7	**1,57**	0,000346
HOXC11	homeobox C11	0,9	1,2	**1,57**	0,021666
RELL1	RELT like 1	5,6	8,3	**1,57**	0,012088
ZSCAN5A	zinc finger and SCAN domain containing 5A	2,8	4,0	**1,57**	0,005153
TNFRSF12A	TNF receptor superfamily member 12A	170,8	233,2	**1,56**	0,003288
AARS	alanyl-tRNA synthetase	180,3	249,4	**1,56**	0,002395
TLR6	toll like receptor 6	6,5	9,4	**1,56**	0,01556
S100A16	S100 calcium binding protein A16	58,3	77,9	**1,56**	0,015218

LINC01619	long intergenic non-protein coding RNA 1619	0,9	1,2	**1,56**	0,018091
ZNF394	zinc finger protein 394	12,8	18,2	**1,56**	0,000314
LINC00649	long intergenic non-protein coding RNA 649	1,1	1,6	**1,56**	0,011339
ACAP1	ArfGAP with coiled-coil, ankyrin repeat and PH domains 1	0,8	1,0	**1,56**	0,019107
GDAP1	ganglioside induced differentiation associated protein 1	15,2	20,8	**1,56**	0,000751
ZNHIT2	zinc finger HIT-type containing 2	6,8	9,7	**1,56**	0,00218
SLC7A1	solute carrier family 7 member 1	116,4	165,3	**1,56**	0,003702
ADPRHL2	ADP-ribosylhydrolase like 2	30,2	40,6	**1,56**	5,70E-05
ZNF611	zinc finger protein 611	6,5	9,7	**1,56**	0,006712
TLR3	toll like receptor 3	6,6	10,0	**1,56**	0,01278
WFIKKN1	WAP, follistatin/kazal, immunoglobulin, kunitz and netrin domain con	0,6	0,7	**1,56**	0,041021
LBX2-AS1	LBX2 antisense RNA 1	3,2	4,2	**1,56**	0,016109
SHROOM4	shroom family member 4	7,2	10,5	**1,56**	0,004295
SLC2A1-AS1	SLC2A1 antisense RNA 1	0,9	1,1	**1,56**	0,018505
AMMECR1L	AMMECR1 like	29,0	41,7	**1,56**	0,000647
B4GALT1	beta-1,4-galactosyltransferase 1	96,6	136,7	**1,56**	0,001574
CCDC65	coiled-coil domain containing 65	0,6	0,9	**1,56**	0,04264
ZNF670	zinc finger protein 670	2,4	3,4	**1,56**	0,012911
CBX4	chromobox 4	15,0	21,0	**1,56**	4,50E-05
PPP1R3F	protein phosphatase 1 regulatory subunit 3F	10,1	14,4	**1,56**	0,000661
TTC1	tetratricopeptide repeat domain 1	54,5	73,1	**1,56**	4,00E-05
STX5	syntaxin 5	33,3	48,1	**1,56**	9,70E-05
FUT4	fucosyltransferase 4	26,1	37,3	**1,56**	0,000455
S1PR3	sphingosine-1-phosphate receptor 3	8,3	11,1	**1,55**	0,001694
ZC3H6	zinc finger CCCH-type containing 6	18,1	25,8	**1,55**	0,000422
KLHL28	kelch like family member 28	15,8	23,0	**1,55**	0,000741
HPS5	HPS5, biogenesis of lysosomal organelles complex 2 subunit 2	21,8	30,0	**1,55**	0,000176
HEG1	heart development protein with EGF like domains 1	35,9	54,4	**1,55**	0,002058
GOLT1B	golgi transport 1B	40,9	57,8	**1,55**	0,000138
AMY2B	amylase, alpha 2B (pancreatic)	1,3	1,7	**1,55**	0,013204
FAM200A	family with sequence similarity 200 member A	6,6	9,5	**1,55**	0,001421
MITD1	microtubule interacting and trafficking domain containing 1	11,5	15,3	**1,55**	0,000178
KLF12	Kruppel like factor 12	17,7	27,2	**1,55**	0,006371

UBALD1	UBA like domain containing 1	10,6	14,9	**1,55**	0,000454
SNAPC1	small nuclear RNA activating complex polypeptide 1	40,9	56,8	**1,55**	9,60E-05
TMEM2	transmembrane protein 2			**1,55**	0,000986
BACE2	beta-site APP-cleaving enzyme 2	45,2	64,0	**1,55**	0,00091
RWDD2A	RWD domain containing 2A	7,2	10,9	**1,55**	0,001874
ANKRD42	ankyrin repeat domain 42	14,2	19,2	**1,55**	0,000497
UNC5B	unc-5 netrin receptor B	89,1	126,0	**1,55**	0,000416
BOD1L1	biorientation of chromosomes in cell division 1 like 1	60,2	87,2	**1,55**	0,0045
TMTC2	transmembrane and tetratricopeptide repeat containing 2	4,0	5,1	**1,55**	0,03144
ZSCAN9	zinc finger and SCAN domain containing 9	5,2	7,0	**1,55**	0,003628
TAGLN2	transgelin 2	168,0	220,2	**1,54**	0,003128
RAB29	RAB29, member RAS oncogene family	38,7	54,4	**1,54**	0,000279
CD74	CD74 molecule	4,2	4,8	**1,54**	0,034942
MIR17HG	miR-17-92a-1 cluster host gene	0,5	0,7	**1,54**	0,038971
PLEKHA7	pleckstrin homology domain containing A7	5,9	7,3	**1,54**	0,001836
GTF2IP13	general transcription factor IIi pseudogene 13	2,7	3,9	**1,54**	0,0157
C2CD3	C2 calcium dependent domain containing 3	14,9	20,8	**1,54**	0,006186
HIC2	HIC ZBTB transcriptional repressor 2	2,1	3,2	**1,54**	0,033084
TMEM154	transmembrane protein 154	12,6	17,0	**1,54**	0,000613
POR	cytochrome p450 oxidoreductase	36,9	51,6	**1,54**	5,60E-05
CSRNP2	cysteine and serine rich nuclear protein 2	28,4	39,5	**1,54**	0,000479
RBBP5	RB binding protein 5, histone lysine methyltransferase complex subun	20,9	29,3	**1,54**	0,00075
ADCY10P1	adenylate cyclase 10, soluble pseudogene 1	3,1	4,3	**1,54**	0,006348
NMI	N-myc and STAT interactor	10,5	15,7	**1,54**	0,001272
THAP5	THAP domain containing 5	31,5	43,5	**1,54**	0,000203
BCL2L13	BCL2 like 13	48,7	66,4	**1,54**	0,000384
SEC23B	Sec23 homolog B, coat complex II component	73,0	102,2	**1,54**	0,000941
LRRC37B	leucine rich repeat containing 37B	4,1	6,2	**1,54**	0,018315
SH3RF3-AS1	SH3RF3 antisense RNA 1	1,0	1,5	**1,54**	0,040913
RORA	RAR related orphan receptor A	30,9	46,3	**1,54**	0,001079
MUL1	mitochondrial E3 ubiquitin protein ligase 1	43,4	58,9	**1,54**	7,40E-05
ANAPC10	anaphase promoting complex subunit 10	8,2	10,0	**1,54**	0,000703
SRSF12	serine and arginine rich splicing factor 12	1,5	2,0	**1,54**	0,015083

LPIN2	lipin 2	112,3	153,8	**1,54**	0,001661
ZFP3	ZFP3 zinc finger protein	6,5	9,4	**1,54**	0,006013
SLC15A4	solute carrier family 15 member 4	42,6	64,4	**1,54**	0,00075
KATNBL1	katanin regulatory subunit B1 like 1	7,5	11,7	**1,54**	0,00372
ANGPTL1	angiopoietin like 1	7,5	11,8	**1,54**	0,021973
RHOG	ras homolog family member G	32,7	45,6	**1,54**	0,000153
ABHD17C	abhydrolase domain containing 17C	5,0	7,5	**1,54**	0,00317
PER1	period circadian clock 1		39,2	**1,54**	0,001187
BICRA	BRD4 interacting chromatin remodelling complex associated protein	9,0	12,9	**1,54**	0,002542
ZKSCAN8	zinc finger with KRAB and SCAN domains 8	26,0	39,4	**1,54**	0,012101
NIPA1	non imprinted in Prader-Willi/Angelman syndrome 1	14,1	20,7	**1,54**	0,004215
FBXO30	F-box protein 30	34,4	52,6	**1,54**	0,001885
LRRC37A3	leucine rich repeat containing 37 member A3	3,8	4,9	**1,53**	0,00617
ZNF586	zinc finger protein 586	2,3	3,2	**1,53**	0,017854
PPP1R15B	protein phosphatase 1 regulatory subunit 15B	39,1	55,6	**1,53**	0,001395
ITPRIPL2	inositol 1,4,5-trisphosphate receptor interacting protein like 2	136,1	189,4	**1,53**	0,003421
PACSIN2	protein kinase C and casein kinase substrate in neurons 2	125,5	172,7	**1,53**	0,001508
FBRS	fibrosin	55,7	80,8	**1,53**	0,000846
CHIC2	cysteine rich hydrophobic domain 2	12,8	17,4	**1,53**	0,000278
WDR89	WD repeat domain 89	13,4	18,1	**1,53**	0,00042
ZXDB	zinc finger, X-linked, duplicated B	8,8	13,2	**1,53**	0,004163
R3HCC1L	R3H domain and coiled-coil containing 1 like	14,3	19,5	**1,53**	0,000807
CIRBP-AS1	CIRBP antisense RNA 1	0,9	1,2	**1,53**	0,041247
ZFP37	ZFP37 zinc finger protein	6,1	8,4	**1,53**	0,002248
HARBI1	harbinger transposase derived 1	3,1	4,3	**1,53**	0,00696
GCC2	GRIP and coiled-coil domain containing 2	42,5	60,9	**1,53**	0,001494
SLC6A6	solute carrier family 6 member 6	16,0	23,7	**1,53**	0,004053
DENND5A	DENN domain containing 5A	102,0	147,4	**1,53**	0,004241
SARNP	SAP domain containing ribonucleoprotein	1,0	1,3	**1,53**	0,030336
NBPF20	NBPF member 20	1,8	2,6	**1,53**	0,039139
RB1CC1	RB1 inducible coiled-coil 1	62,4	91,3	**1,53**	0,003221
ZNF674-AS1	ZNF674 antisense RNA 1 (head to head)	2,4	3,3	**1,53**	0,015151
CD34	CD34 molecule	2,6	3,3	**1,52**	0,040222

E2F6	E2F transcription factor 6	6,5	8,4	**1,52**	0,002613
ZNF317	zinc finger protein 317	20,1	27,3	**1,52**	0,000903
PRRC2C	proline rich coiled-coil 2C	159,5	234,0	**1,52**	0,009226
POPDC3	popeye domain containing 3	4,4	6,8	**1,52**	0,002617
GTF2IRD2	GTF2I repeat domain containing 2	1,3	1,9	**1,52**	0,019129
ACOT7	acyl-CoA thioesterase 7	39,1	50,9	**1,52**	0,003518
LONRF2	LON peptidase N-terminal domain and ring finger 2	7,7	10,2	**1,52**	0,00419
PIM3	Pim-3 proto-oncogene, serine/threonine kinase	111,6	163,8	**1,52**	0,002092
EPC2	enhancer of polycomb homolog 2	23,3	32,2	**1,52**	0,001105
LRP5L	LDL receptor related protein 5 like	1,7	2,1	**1,52**	0,015442
PHF8	PHD finger protein 8	23,9	32,7	**1,52**	0,000539
RPGR	retinitis pigmentosa GTPase regulator	6,9	9,6	**1,52**	0,00489
MANF	mesencephalic astrocyte derived neurotrophic factor	75,6	109,0	**1,52**	0,014239
FJX1	four jointed box 1	15,5	21,7	**1,52**	0,000537
SP140L	SP140 nuclear body protein like	6,7	8,5	**1,52**	0,005121
SLC25A22	solute carrier family 25 member 22	29,6	36,4	**1,52**	0,00145
SC5D	sterol-C5-desaturase	61,0	82,5	**1,52**	0,00021
ZCCHC8	zinc finger CCHC-type containing 8	19,3	27,3	**1,52**	0,00201
MON1B	MON1 homolog B, secretory trafficking associated	44,8	61,4	**1,52**	0,000263
AKIRIN1	akirin 1	38,4	52,5	**1,52**	0,000191
ZFAND3	zinc finger AN1-type containing 3	89,9	122,1	**1,52**	0,00082
DOCK8	dedicator of cytokinesis 8	14,3	21,2	**1,52**	0,026303
TJAP1	tight junction associated protein 1	24,5	33,0	**1,52**	0,000268
DCAF12L2	DDB1 and CUL4 associated factor 12 like 2	1,2	1,6	**1,52**	0,044638
TMEM170B	transmembrane protein 170B	4,6	6,8	**1,52**	0,009799
YRDC	yrdC N6-threonylcarbamoyltransferase domain containing	13,6	18,7	**1,52**	0,000429
JMY	junction mediating and regulatory protein, p53 cofactor	22,6	32,9	**1,52**	0,006287
THAP2	THAP domain containing 2	7,9	11,1	**1,52**	0,001844
CHD1	chromodomain helicase DNA binding protein 1	29,6	42,8	**1,52**	0,003563
INSIG2	insulin induced gene 2	45,9	68,3	**1,52**	0,001512
GNPDA2	glucosamine-6-phosphate deaminase 2	23,1	32,6	**1,52**	0,001113
WDR27	WD repeat domain 27	8,3	10,8	**1,52**	0,005419
CLMP	CXADR like membrane protein	596,3	810,6	**1,52**	0,03252

GOT1	glutamic-oxaloacetic transaminase 1	45,4	55,0	**1,52**	0,000142
ARAP2	ArfGAP with RhoGAP domain, ankyrin repeat and PH domain 2	8,1	12,3	**1,52**	0,047106
GOLGA7B	golgin A7 family member B	1,6	2,2	**1,52**	0,023962
WDR19	WD repeat domain 19	21,8	30,1	**1,52**	0,001179
TTC30A	tetratricopeptide repeat domain 30A	11,4	15,4	**1,52**	0,003706
CHMP4B	charged multivesicular body protein 4B	144,7	193,8	**1,51**	0,001959
ZNF784	zinc finger protein 784	4,7	6,4	**1,51**	0,004287
MCOLN1	mucolipin 1	16,7	22,6	**1,51**	0,000497
IGFBP5	insulin like growth factor binding protein 5	139,4	181,2	**1,51**	0,044389
RIMKLA	ribosomal modification protein rimK like family member A	5,8	8,2	**1,51**	0,004225
TIGD1	tigger transposable element derived 1	3,1	3,9	**1,51**	0,007285
RFFL	ring finger and FYVE like domain containing E3 ubiquitin protein ligase	20,5	27,5	**1,51**	0,000625
CHMP7	charged multivesicular body protein 7	26,2	35,9	**1,51**	0,000242
SLC9A3-AS1	SLC9A3 antisense RNA 1	11,1	15,4	**1,51**	0,001374
UBN2	ubinuclein 2	13,7	20,7	**1,51**	0,01332
MKL1	megakaryoblastic leukemia (translocation) 1			**1,51**	0,001844
DRAP1	DR1 associated protein 1	124,2	156,0	**1,51**	0,003825
ZNF720	zinc finger protein 720	9,1	12,2	**1,51**	0,001101
LINC00641	long intergenic non-protein coding RNA 641	6,5	9,0	**1,51**	0,01245
AP5Z1	adaptor related protein complex 5 zeta 1 subunit	27,8	36,3	**1,51**	0,000141
JUNB	JunB proto-oncogene, AP-1 transcription factor subunit	142,9	210,4	**1,51**	0,00804
WDR25	WD repeat domain 25	10,7	13,7	**1,51**	0,00105
CASP9	caspase 9	7,5	10,4	**1,51**	0,002372
ZUFSP	zinc finger with UFM1 specific peptidase domain			**1,51**	0,006974
UAP1	UDP-N-acetylglucosamine pyrophosphorylase 1	233,1	298,9	**1,51**	0,006741
CGRRF1	cell growth regulator with ring finger domain 1	13,6	17,7	**1,51**	0,000355
ZBTB17	zinc finger and BTB domain containing 17	21,2	28,4	**1,51**	0,000288
ENO2	enolase 2	65,7	90,5	**1,51**	0,003383
TVP23B	trans-golgi network vesicle protein 23 homolog B	31,4	41,8	**1,51**	0,000156
MB21D2	Mab-21 domain containing 2	3,0	4,1	**1,51**	0,018012
MOB2	MOB kinase activator 2	18,6	24,9	**1,51**	0,000188
TGFA	transforming growth factor alpha	20,6	26,9	**1,51**	0,000247
LIMD2	LIM domain containing 2	2,1	2,8	**1,51**	0,031006

GIPR	gastric inhibitory polypeptide receptor	4,3	6,2	**1,51**	0,013544
MTERF1	mitochondrial transcription termination factor 1	5,7	7,8	**1,51**	0,003507
DAAM1	dishevelled associated activator of morphogenesis 1	14,1	20,0	**1,51**	0,004353
QRICH2	glutamine rich 2	4,9	6,7	**1,51**	0,004682
LRRC8B	leucine rich repeat containing 8 family member B	2,5	3,5	**1,51**	0,031644
CLK1	CDC like kinase 1	79,8	109,6	**1,51**	0,00124
NELFA	negative elongation factor complex member A	19,3	25,9	**1,51**	0,000679
ARFGAP3	ADP ribosylation factor GTPase activating protein 3	105,9	139,4	**1,51**	0,001092
SNX25	sorting nexin 25	15,2	22,0	**1,51**	0,001474
CMTM8	CKLF like MARVEL transmembrane domain containing 8	1,6	1,8	**1,50**	0,020507
RASD1	ras related dexamethasone induced 1	29,9	48,3	**1,50**	0,01494
ATP1B1	ATPase Na+/K+ transporting subunit beta 1	259,8	335,1	**1,50**	0,018444
ARFGEF3	ARFGEF family member 3	2,7	3,6	**1,50**	0,034301
ZNF417	zinc finger protein 417	3,8	4,9	**1,50**	0,009919
SAFB2	scaffold attachment factor B2	40,3	54,6	**1,50**	0,000392
SDAD1P1	SDA1 domain containing 1 pseudogene 1	5,6	8,1	**1,50**	0,008894
METTL22	methyltransferase like 22	8,9	11,5	**1,50**	0,001948
RUNDC1	RUN domain containing 1	23,7	31,9	**1,50**	0,000684
TOR4A	torsin family 4 member A	63,1	81,7	**1,50**	0,000997
ZNF616	zinc finger protein 616	5,7	7,7	**1,50**	0,010021
ZNF582	zinc finger protein 582	2,4	3,5	**1,50**	0,02579
EXT1	exostosin glycosyltransferase 1	25,3	33,5	**1,50**	0,000422
UBAP1	ubiquitin associated protein 1	56,4	74,9	**1,50**	0,000175
FZD3	frizzled class receptor 3	2,1	2,8	**1,50**	0,019453
DIO3OS	DIO3 opposite strand/antisense RNA (head to head)	5,3	8,1	**1,50**	0,010359
SARS	seryl-tRNA synthetase	248,1	320,7	**1,50**	0,005971
IRS2	insulin receptor substrate 2	77,6	104,2	**1,50**	0,009126
FOXO3	forkhead box O3	54,5	76,0	**1,50**	0,003569
ZNF181	zinc finger protein 181	12,7	17,2	**1,50**	0,004587
RAB32	RAB32, member RAS oncogene family	24,4	32,5	**1,50**	0,000564
TUBE1	tubulin epsilon 1	15,7	21,1	**1,50**	0,000958
CREM	cAMP responsive element modulator	21,8	28,4	**1,50**	0,000148
GPR137	G protein-coupled receptor 137	20,9	29,3	**1,50**	0,001217

ZNF701	zinc finger protein 701	4,4	6,1	**1,50**	0,012682
ZNF280C	zinc finger protein 280C	3,5	4,6	**1,50**	0,018741
MRPS10	mitochondrial ribosomal protein S10	19,9	25,4	**1,50**	0,000669
PVT1	Pvt1 oncogene (non-protein coding)	1,9	2,5	**1,50**	0,036606
ARL4C	ADP ribosylation factor like GTPase 4C	14,9	18,8	**1,50**	0,000557
LINC01006	long intergenic non-protein coding RNA 1006	2,0	2,6	**1,50**	0,037794
ATG101	autophagy related 101	36,2	46,8	**1,50**	0,000174
ZCCHC6	zinc finger CCHC-type containing 6			**1,50**	0,005066
MIDN	midnolin	69,7	96,5	**1,50**	0,00129
AREL1	apoptosis resistant E3 ubiquitin protein ligase 1	26,4	35,3	**1,49**	0,000786
PTRH1	peptidyl-tRNA hydrolase 1 homolog	8,2	10,7	**1,49**	0,006418
C21orf91	chromosome 21 open reading frame 91	8,9	11,9	**1,49**	0,0068
NR1D2	nuclear receptor subfamily 1 group D member 2	87,1	123,9	**1,49**	0,006146
RRM2B	ribonucleotide reductase regulatory TP53 inducible subunit M2B	46,0	62,0	**1,49**	0,002642
SLC38A9	solute carrier family 38 member 9	9,3	12,8	**1,49**	0,003047
ZNF333	zinc finger protein 333	11,3	15,3	**1,49**	0,003425
RPP38	ribonuclease P/MRP subunit p38	8,0	10,1	**1,49**	0,001094
ULBP3	UL16 binding protein 3	1,8	2,2	**1,49**	0,035522
PAPD5	poly(A) RNA polymerase D5, non-canonical			**1,49**	0,003055
C7orf43	chromosome 7 open reading frame 43	7,6	10,4	**1,49**	0,004465
TPRA1	transmembrane protein adipocyte associated 1	35,9	47,0	**1,49**	0,000156
AC008560.1	Uncharacterized protein FLJ26957			**1,49**	0,037173
MGA	MGA, MAX dimerization protein	38,4	55,4	**1,49**	0,014791
TTI2	TELO2 interacting protein 2	10,6	13,7	**1,49**	0,000696
SDE2	SDE2 telomere maintenance homolog	25,0	32,6	**1,49**	0,000761
IST1	IST1, ESCRT-III associated factor	101,6	131,7	**1,49**	0,001213
RILPL2	Rab interacting lysosomal protein like 2	33,3	44,5	**1,49**	5,50E-05
EVI2B	ecotropic viral integration site 2B	1,9	2,4	**1,49**	0,025895
EMC6	ER membrane protein complex subunit 6	17,1	21,6	**1,49**	0,000198
RBM33	RNA binding motif protein 33	28,6	38,6	**1,49**	0,001386
ZNF653	zinc finger protein 653	3,4	4,8	**1,49**	0,018051
SPEN	spen family transcriptional repressor	72,7	104,4	**1,49**	0,023069
CACNA1A	calcium voltage-gated channel subunit alpha1 A	1,6	1,9	**1,49**	0,036947

YPEL1	yippee like 1	3,4	4,6	**1,49**	0,031099
NIPAL1	NIPA like domain containing 1	3,8	4,4	**1,49**	0,012211
MRPS33	mitochondrial ribosomal protein S33	27,3	34,0	**1,49**	5,40E-05
H1FX	H1 histone family member X	107,8	149,2	**1,49**	0,002139
ZNF281	zinc finger protein 281	12,6	18,2	**1,49**	0,005555
ENDOV	endonuclease V	11,3	13,8	**1,49**	0,000907
SEMA6C	semaphorin 6C	6,5	9,0	**1,49**	0,006211
SHISA2	shisa family member 2	4,9	6,5	**1,49**	0,036839
CHEK2	checkpoint kinase 2	5,0	6,2	**1,48**	0,01008
DNAH9	dynein axonemal heavy chain 9	1,5	1,6	**1,48**	0,022924
ZNF84	zinc finger protein 84	18,7	25,4	**1,48**	0,003956
CRKL	CRK like proto-oncogene, adaptor protein	69,6	94,1	**1,48**	0,001893
KIF27	kinesin family member 27	3,2	4,3	**1,48**	0,024663
MED7	mediator complex subunit 7	13,3	17,4	**1,48**	0,000797
DNAJB5	DnaJ heat shock protein family (Hsp40) member B5	5,2	7,4	**1,48**	0,009806
PALMD	palmdelphin	29,9	41,7	**1,48**	0,005671
TMOD3	tropomodulin 3	85,4	115,3	**1,48**	0,003772
COG1	component of oligomeric golgi complex 1	26,7	34,6	**1,48**	0,000261
MAN2B1	mannosidase alpha class 2B member 1	28,8	39,1	**1,48**	0,000375
AKT1S1	AKT1 substrate 1	76,1	104,3	**1,48**	0,001508
EML6	echinoderm microtubule associated protein like 6	1,9	2,2	**1,48**	0,025763
PNO1	partner of NOB1 homolog	24,7	32,5	**1,48**	0,00115
DCP2	decapping mRNA 2	16,6	21,2	**1,48**	0,000981
TAF5	TATA-box binding protein associated factor 5	6,6	8,7	**1,48**	0,01167
FBXO48	F-box protein 48	2,3	3,3	**1,48**	0,035362
COL13A1	collagen type XIII alpha 1 chain	3,0	3,6	**1,48**	0,036256
TAF8	TATA-box binding protein associated factor 8	13,6	18,2	**1,48**	0,002841
GAS5	growth arrest specific 5 (non-protein coding)	54,2	68,2	**1,48**	0,000176
KDM6A	lysine demethylase 6A	25,1	34,7	**1,48**	0,010828
OSER1-AS1	OSER1 antisense RNA 1 (head to head)	33,0	61,1	**1,48**	0,001026
NECAP1	NECAP endocytosis associated 1	27,6	35,2	**1,48**	0,000381
ACAD8	acyl-CoA dehydrogenase family member 8	15,4	20,4	**1,48**	0,001079
ZNF202	zinc finger protein 202	8,9	11,4	**1,48**	0,002829

FAM69A	family with sequence similarity 69 member A		11,0	**1,48**	0,011979
ABTB1	ankyrin repeat and BTB domain containing 1	26,4	36,0	**1,48**	0,001122
WDR73	WD repeat domain 73	22,7	28,8	**1,48**	0,00015
ZNF799	zinc finger protein 799	2,0	2,6	**1,48**	0,03493
MARK3	microtubule affinity regulating kinase 3	41,7	55,1	**1,48**	0,000339
ZNF587B	zinc finger protein 587B	2,7	3,5	**1,48**	0,018356
NUP58	nucleoporin 58	44,8	57,5	**1,48**	0,001636
EPS8L2	EPS8 like 2	115,2	147,6	**1,48**	0,002403
PML	promyelocytic leukemia	39,1	53,1	**1,48**	0,000604
TNRC6B	trinucleotide repeat containing 6B	26,5	36,2	**1,48**	0,006991
LRIG1	leucine rich repeats and immunoglobulin like domains 1	20,8	30,6	**1,48**	0,027491
EAF2	ELL associated factor 2	2,5	2,9	**1,48**	0,043147
VRK3	vaccinia related kinase 3	20,1	26,6	**1,48**	0,000607
ZNF710	zinc finger protein 710	4,5	5,8	**1,48**	0,010585
SNHG11	small nucleolar RNA host gene 11	6,2	7,6	**1,47**	0,003662
TPRN	taperin	9,7	12,6	**1,47**	0,005155
DHRS7B	dehydrogenase/reductase 7B	14,7	18,7	**1,47**	0,001089
USP42	ubiquitin specific peptidase 42	11,0	14,5	**1,47**	0,002725
HSDL1	hydroxysteroid dehydrogenase like 1	24,0	31,9	**1,47**	0,000918
SDHAF2	succinate dehydrogenase complex assembly factor 2	6,7	8,6	**1,47**	0,003773
CDKN2AIP	CDKN2A interacting protein	22,4	28,3	**1,47**	0,000474
ZNF213	zinc finger protein 213	17,1	22,8	**1,47**	0,002075
ZNF227	zinc finger protein 227	8,8	11,7	**1,47**	0,007414
TYW5	tRNA-yW synthesizing protein 5	5,2	6,9	**1,47**	0,015847
LCMT2	leucine carboxyl methyltransferase 2	17,4	22,5	**1,47**	0,001088
UTP11	UTP11, small subunit processome component homolog (S. cerevisiae)	22,9	28,4	**1,47**	0,000688
SP1	Sp1 transcription factor	64,4	87,2	**1,47**	0,006226
SNHG20	small nucleolar RNA host gene 20	1,5	1,8	**1,47**	0,026055
RLF	rearranged L-myc fusion	25,2	34,3	**1,47**	0,003184
OPTN	optineurin	98,8	136,4	**1,47**	0,004467
KAT5	lysine acetyltransferase 5	45,7	58,4	**1,47**	0,000265
PPP2R2A	protein phosphatase 2 regulatory subunit Balpha	38,3	49,4	**1,47**	0,000569
PHF5A	PHD finger protein 5A	19,9	24,0	**1,47**	0,00017

CCNK	cyclin K	43,7	56,0	**1,47**	0,000583
OTUD1	OTU deubiquitinase 1	11,6	15,5	**1,47**	0,004428
ZSCAN21	zinc finger and SCAN domain containing 21	7,3	9,2	**1,47**	0,003472
ATXN1	ataxin 1	40,7	55,2	**1,47**	0,001467
ZNF487	zinc finger protein 487	1,9	2,2	**1,47**	0,018029
KDM2A	lysine demethylase 2A	112,7	147,1	**1,47**	0,004658
GCDH	glutaryl-CoA dehydrogenase	15,1	20,5	**1,47**	0,001036
SEC61G	Sec61 translocon gamma subunit	87,0	111,6	**1,47**	0,003317
RPP25L	ribonuclease P/MRP subunit p25 like	10,2	12,9	**1,47**	0,004667
ZNF26	zinc finger protein 26	7,7	10,1	**1,47**	0,008305
BAMBI	BMP and activin membrane bound inhibitor	33,8	43,8	**1,47**	0,000295
SEC22B	SEC22 homolog B, vesicle trafficking protein (gene/pseudogene)	70,1	92,7	**1,47**	0,001835
LPXN	leupaxin	18,1	23,5	**1,47**	0,001395
GNG2	G protein subunit gamma 2	8,7	8,5	**1,47**	0,010459
MAP1S	microtubule associated protein 1S	21,4	29,2	**1,47**	0,003272
MRPS22	mitochondrial ribosomal protein S22	27,9	34,8	**1,47**	0,000264
SRA1	steroid receptor RNA activator 1	38,9	46,5	**1,47**	0,000316
PPP1R2	protein phosphatase 1 regulatory inhibitor subunit 2	30,6	38,7	**1,47**	0,000234
MFSD6	major facilitator superfamily domain containing 6	9,1	11,5	**1,47**	0,008615
CWF19L2	CWF19 like 2, cell cycle control (S. pombe)	15,7	19,4	**1,46**	0,000913
EVA1A	eva-1 homolog A, regulator of programmed cell death	3,8	5,1	**1,46**	0,033596
DUSP1	dual specificity phosphatase 1	110,0	155,2	**1,46**	0,004396
LIN52	lin-52 DREAM MuvB core complex component	13,7	16,8	**1,46**	0,001128
BBIP1	BBSome interacting protein 1	24,0	31,1	**1,46**	0,000426
PQLC2	PQ loop repeat containing 2	9,4	11,6	**1,46**	0,003271
MAIP1	matrix AAA peptidase interacting protein 1	8,2	10,1	**1,46**	0,002915
STRIP1	striatin interacting protein 1	24,8	32,0	**1,46**	0,001044
MED13	mediator complex subunit 13	45,7	63,9	**1,46**	0,016779
SMG9	SMG9, nonsense mediated mRNA decay factor	37,8	46,9	**1,46**	0,002413
VLDLR-AS1	VLDLR antisense RNA 1	1,4	1,9	**1,46**	0,046095
FAF2	Fas associated factor family member 2	59,1	77,1	**1,46**	0,001783
H3F3B	H3 histone family member 3B	434,5	548,9	**1,46**	0,02229
SLC8B1	solute carrier family 8 member B1	27,7	35,8	**1,46**	0,001228

ST20	suppressor of tumorigenicity 20	2,0	2,6	**1,46**	0,037927
INSR	insulin receptor	35,8	48,4	**1,46**	0,005286
DNTTIP2	deoxynucleotidyltransferase terminal interacting protein 2	47,7	63,7	**1,46**	0,001583
PEX12	peroxisomal biogenesis factor 12	19,3	24,2	**1,46**	0,000419
ULK4	unc-51 like kinase 4	3,2	3,9	**1,46**	0,040204
ACVR1	activin A receptor type 1	163,5	213,3	**1,46**	0,007049
TNFRSF10A	TNF receptor superfamily member 10a	4,8	6,5	**1,46**	0,033755
PCBP1-AS1	PCBP1 antisense RNA 1	15,6	19,4	**1,46**	0,000454
RAB3IP	RAB3A interacting protein	3,8	4,8	**1,46**	0,016988
TUSC2	tumor suppressor candidate 2	20,5	26,4	**1,46**	0,000166
TDP2	tyrosyl-DNA phosphodiesterase 2	35,3	46,2	**1,46**	0,001057
C2orf42	chromosome 2 open reading frame 42	9,0	11,3	**1,46**	0,003705
SNRK	SNF related kinase	15,8	20,8	**1,46**	0,003896
SOX13	SRY-box 13	3,4	4,1	**1,46**	0,015408
HNRNPC	heterogeneous nuclear ribonucleoprotein C (C1/C2)	237,9	293,2	**1,46**	0,007208
CKMT2-AS1	CKMT2 antisense RNA 1	2,8	3,8	**1,46**	0,042458
KIAA1755	KIAA1755	45,9	55,8	**1,46**	0,01104
GAS7	growth arrest specific 7	90,2	111,8	**1,46**	0,012461
ADAM17	ADAM metallopeptidase domain 17	34,6	45,4	**1,46**	0,001043
DISC1	disrupted in schizophrenia 1	2,9	4,0	**1,46**	0,023945
ZNF473	zinc finger protein 473	8,6	10,8	**1,46**	0,005918
ASB16-AS1	ASB16 antisense RNA 1	5,4	7,0	**1,46**	0,010562
SLC9A5	solute carrier family 9 member A5	1,0	1,2	**1,46**	0,035979
SCNM1	sodium channel modifier 1	16,2	19,9	**1,46**	0,000645
PLCG2	phospholipase C gamma 2	21,6	27,4	**1,46**	0,001305
XPR1	xenotropic and polytropic retrovirus receptor 1	26,3	35,4	**1,46**	0,003472
GRIPAP1	GRIP1 associated protein 1	33,6	45,0	**1,46**	0,001093
GPATCH2L	G-patch domain containing 2 like	29,1	38,1	**1,46**	0,007473
GRPEL1	GrpE like 1, mitochondrial	35,9	46,6	**1,46**	0,000585
ZNF77	zinc finger protein 77	1,9	2,4	**1,46**	0,023985
METTL15	methyltransferase like 15	8,7	11,0	**1,46**	0,005694
TBK1	TANK binding kinase 1	21,7	30,3	**1,46**	0,002299
IRX5	iroquois homeobox 5	2,7	3,2	**1,46**	0,035342

DOK5	docking protein 5	3,3	4,5	**1,46**	0,025933
RAB21	RAB21, member RAS oncogene family	89,5	115,1	**1,45**	0,002604
TCEANC	transcription elongation factor A N-terminal and central domain conta	2,1	2,7	**1,45**	0,031054
MRFAP1L1	Morf4 family associated protein 1 like 1	56,4	72,2	**1,45**	0,000834
POLG	DNA polymerase gamma, catalytic subunit	47,6	63,0	**1,45**	0,00122
CHTOP	chromatin target of PRMT1	68,6	86,9	**1,45**	0,000426
ADPRHL1	ADP-ribosylhydrolase like 1	6,6	9,1	**1,45**	0,015853
SLC25A53	solute carrier family 25 member 53	2,0	2,8	**1,45**	0,04707
RAPGEF2	Rap guanine nucleotide exchange factor 2	61,8	83,7	**1,45**	0,013504
TIGD6	tigger transposable element derived 6	7,3	9,2	**1,45**	0,004287
CCDC82	coiled-coil domain containing 82	24,3	33,1	**1,45**	0,003635
CASP3	caspase 3	12,3	16,2	**1,45**	0,003518
SUPT4H1	SPT4 homolog, DSIF elongation factor subunit	46,0	57,8	**1,45**	0,00019
DLGAP4	DLG associated protein 4	108,2	143,5	**1,45**	0,005369
ELK3	ELK3, ETS transcription factor	82,8	109,5	**1,45**	0,002232
FAM83G	family with sequence similarity 83 member G	41,4	52,5	**1,45**	0,00183
DGKE	diacylglycerol kinase epsilon	8,1	10,6	**1,45**	0,012683
RTN3	reticulon 3	94,2	124,8	**1,45**	0,002151
COQ8A	coenzyme Q8A	14,2	18,4	**1,45**	0,000665
GYPC	glycophorin C (Gerbich blood group)	46,8	58,4	**1,45**	0,003216
SYN1	synapsin I	3,3	4,3	**1,45**	0,030644
TOP1	topoisomerase (DNA) I	59,2	77,3	**1,45**	0,001042
TMX2	thioredoxin related transmembrane protein 2	59,8	72,3	**1,45**	0,00044
FOS	Fos proto-oncogene, AP-1 transcription factor subunit	19,7	25,5	**1,45**	0,000969
FEM1A	fem-1 homolog A	30,8	39,0	**1,45**	0,000822
PSMA3-AS1	PSMA3 antisense RNA 1	16,5	21,2	**1,45**	0,000857
CA5BP1	carbonic anhydrase 5B pseudogene 1	6,5	8,3	**1,45**	0,006147
UBE2B	ubiquitin conjugating enzyme E2 B	73,3	94,7	**1,45**	0,001197
SCUBE3	signal peptide, CUB domain and EGF like domain containing 3	34,1	41,5	**1,45**	0,049436
CABLES2	Cdk5 and Abl enzyme substrate 2	7,8	10,4	**1,45**	0,014051
ZNF322	zinc finger protein 322	3,7	4,6	**1,45**	0,017502
CCDC96	coiled-coil domain containing 96	2,1	2,6	**1,45**	0,035019
TAF13	TATA-box binding protein associated factor 13	27,4	34,4	**1,45**	0,000308

EPC1	enhancer of polycomb homolog 1	28,4	36,8	**1,45**	0,001806
ZNF319	zinc finger protein 319	13,2	17,1	**1,45**	0,003103
MARS	methionyl-tRNA synthetase	152,3	191,3	**1,45**	0,006594
CLP1	cleavage and polyadenylation factor I subunit 1	8,4	10,5	**1,45**	0,007234
SCAND1	SCAN domain containing 1	31,1	39,0	**1,45**	0,000656
DEAF1	DEAF1, transcription factor	16,2	20,7	**1,45**	0,001257
STK36	serine/threonine kinase 36	12,1	15,2	**1,45**	0,002841
C1orf132	chromosome 1 open reading frame 132			**1,45**	0,045896
NGDN	neuroguidin	20,2	25,1	**1,44**	0,000465
CBX8	chromobox 8	2,6	3,1	**1,44**	0,026019
UBE2D3	ubiquitin conjugating enzyme E2 D3	233,5	296,1	**1,44**	0,011349
CCSAP	centriole, cilia and spindle associated protein	6,6	7,8	**1,44**	0,008969
MOSPD1	motile sperm domain containing 1	11,1	13,9	**1,44**	0,002926
FAM133B	family with sequence similarity 133 member B	6,9	8,5	**1,44**	0,005867
PMP22	peripheral myelin protein 22	290,2	380,1	**1,44**	0,022989
CNNM3	cyclin and CBS domain divalent metal cation transport mediator 3	21,7	28,3	**1,44**	0,001165
ORAOV1	oral cancer overexpressed 1			**1,44**	0,003843
NPL	N-acetylneuraminate pyruvate lyase	2,0	2,9	**1,44**	0,041093
TVP23C	trans-golgi network vesicle protein 23 homolog C	4,3	5,6	**1,44**	0,034997
PEAK1	pseudopodium enriched atypical kinase 1	20,7	27,4	**1,44**	0,013295
SELENOM	selenoprotein M	112,6	147,3	**1,44**	0,003028
UCHL1	ubiquitin C-terminal hydrolase L1	7,1	8,1	**1,44**	0,006153
RBM38	RNA binding motif protein 38	5,7	7,0	**1,44**	0,010709
RAPGEF6	Rap guanine nucleotide exchange factor 6	5,4	6,7	**1,44**	0,007696
STK32C	serine/threonine kinase 32C	6,1	7,3	**1,44**	0,011144
RNF207	ring finger protein 207	6,5	8,2	**1,44**	0,013666
EVA1C	eva-1 homolog C	3,7	4,5	**1,44**	0,014006
GAN	gigaxonin	7,2	8,8	**1,44**	0,011781
CNBP	CCHC-type zinc finger nucleic acid binding protein	312,8	387,8	**1,44**	0,017387
MMADHC	methylmalonic aciduria and homocystinuria, cblD type	93,2	116,0	**1,44**	0,001808
LINC00638	long intergenic non-protein coding RNA 638	2,6	3,1	**1,44**	0,022834
SMYD4	SET and MYND domain containing 4	11,5	14,5	**1,44**	0,006843
ATF4	activating transcription factor 4	441,6	567,7	**1,44**	0,031397

RBM18	RNA binding motif protein 18	26,1	33,7	**1,44**	0,000684
SLC31A1	solute carrier family 31 member 1	66,0	82,9	**1,44**	0,000874
ZNF350	zinc finger protein 350	7,0	8,9	**1,44**	0,005198
TMEM44-AS:	TMEM44 antisense RNA 1	3,0	3,7	**1,44**	0,033933
UBIAD1	UbiA prenyltransferase domain containing 1	17,7	22,8	**1,44**	0,002071
RRS1	ribosome biogenesis regulator homolog	14,2	18,1	**1,44**	0,006
TMF1	TATA element modulatory factor 1	87,0	112,7	**1,44**	0,006033
RPP14	ribonuclease P/MRP subunit p14	11,7	14,1	**1,44**	0,003166
CNTRL	centriolin	11,7	15,0	**1,44**	0,026451
DDI2	DNA damage inducible 1 homolog 2	24,4	32,4	**1,44**	0,00855
DACT3	dishevelled binding antagonist of beta catenin 3	5,2	6,5	**1,44**	0,010343
PIGH	phosphatidylinositol glycan anchor biosynthesis class H	18,6	23,8	**1,44**	0,000723
PIAS4	protein inhibitor of activated STAT 4	18,4	22,9	**1,44**	0,000636
ZNF195	zinc finger protein 195	8,0	10,2	**1,44**	0,007694
PISD	phosphatidylserine decarboxylase	17,0	20,9	**1,44**	0,002688
DIP2B	disco interacting protein 2 homolog B	25,2	33,6	**1,44**	0,008425
UBE2D1	ubiquitin conjugating enzyme E2 D1	12,5	15,8	**1,44**	0,00479
ZBTB5	zinc finger and BTB domain containing 5	13,8	17,8	**1,44**	0,005921
GCAT	glycine C-acetyltransferase	14,2	18,7	**1,43**	0,001128
EGLN1	egl-9 family hypoxia inducible factor 1	106,2	141,6	**1,43**	0,005555
BANP	BTG3 associated nuclear protein	11,9	14,3	**1,43**	0,002932
CATSPERG	cation channel sperm associated auxiliary subunit gamma	1,3	1,6	**1,43**	0,044288
RNF111	ring finger protein 111	26,5	35,1	**1,43**	0,008456
CLCN6	chloride voltage-gated channel 6	21,5	28,9	**1,43**	0,007618
SLC38A1	solute carrier family 38 member 1	195,0	261,5	**1,43**	0,022615
PEX16	peroxisomal biogenesis factor 16	15,6	19,9	**1,43**	0,001609
BBS4	Bardet-Biedl syndrome 4	24,1	28,8	**1,43**	0,00184
POLDIP3	DNA polymerase delta interacting protein 3	117,3	143,3	**1,43**	0,003273
ARHGAP12	Rho GTPase activating protein 12	21,4	27,2	**1,43**	0,002071
METTL18	methyltransferase like 18	5,6	7,3	**1,43**	0,020544
MAP2K7	mitogen-activated protein kinase kinase 7	36,3	46,1	**1,43**	0,000392
CMTM6	CKLF like MARVEL transmembrane domain containing 6	40,9	49,6	**1,43**	0,001392
SPRTN	SprT-like N-terminal domain	9,9	12,1	**1,43**	0,007623

CEP112	centrosomal protein 112	15,9	19,0	**1,43**	0,001887
TAF12	TATA-box binding protein associated factor 12	21,9	26,4	**1,43**	0,000366
SAFB	scaffold attachment factor B	58,3	73,1	**1,43**	0,002044
EXOC8	exocyst complex component 8	20,0	25,7	**1,43**	0,004962
PAN2	PAN2 poly(A) specific ribonuclease subunit	30,6	39,4	**1,43**	0,001109
INAFM2	InaF motif containing 2	8,7	11,3	**1,43**	0,008341
CREBRF	CREB3 regulatory factor	50,2	67,4	**1,43**	0,017659
NFXL1	nuclear transcription factor, X-box binding like 1	14,5	18,7	**1,43**	0,006865
CHM	CHM, Rab escort protein 1	29,8	38,2	**1,43**	0,01249
OTUD4	OTU deubiquitinase 4	26,7	35,7	**1,43**	0,016998
HSPA6	heat shock protein family A (Hsp70) member 6	3,4	3,9	**1,43**	0,032941
BCL9L	B-cell CLL/lymphoma 9 like	73,2	97,5	**1,43**	0,008218
PDPN	podoplanin	488,8	586,0	**1,43**	0,036171
CLHC1	clathrin heavy chain linker domain containing 1	1,4	1,4	**1,43**	0,041143
FAM206A	family with sequence similarity 206 member A	11,1	13,9	**1,43**	0,003134
LINC00869	long intergenic non-protein coding RNA 869	1,2	1,6	**1,43**	0,04655
MCAT	malonyl-CoA-acyl carrier protein transacylase	9,3	11,7	**1,43**	0,006493
MORN1	MORN repeat containing 1	5,2	6,6	**1,43**	0,016201
GRK6	G protein-coupled receptor kinase 6	18,4	22,4	**1,43**	0,002313
SCAND2P	SCAN domain containing 2 pseudogene	9,0	11,3	**1,43**	0,00613
OGDH	oxoglutarate dehydrogenase	150,9	191,6	**1,43**	0,010092
NXT1	nuclear transport factor 2 like export factor 1	17,9	21,6	**1,43**	0,001676
INO80C	INO80 complex subunit C	8,9	10,8	**1,43**	0,011228
GTF2A1	general transcription factor IIA subunit 1	57,0	73,7	**1,43**	0,004
NUP35	nucleoporin 35	13,2	16,5	**1,43**	0,007189
FAM160A2	family with sequence similarity 160 member A2	17,6	22,3	**1,42**	0,002143
SYS1	SYS1, golgi trafficking protein	15,9	20,2	**1,42**	0,002796
TRIM8	tripartite motif containing 8	97,6	129,0	**1,42**	0,007649
UTP3	UTP3, small subunit processome component homolog (S. cerevisiae)	36,0	45,3	**1,42**	0,000602
LINC00511	long intergenic non-protein coding RNA 511	35,7	40,1	**1,42**	0,002614
FNDC1	fibronectin type III domain containing 1	86,8	113,4	**1,42**	0,028443
PPFIA1	PTPRF interacting protein alpha 1	57,9	75,4	**1,42**	0,005111
RBMS3	RNA binding motif single stranded interacting protein 3	9,6	11,9	**1,42**	0,033374

TMEM136	transmembrane protein 136	10,6	12,8	**1,42**	0,004826
NAB1	NGFI-A binding protein 1	44,7	63,5	**1,42**	0,01036
MZF1	myeloid zinc finger 1	11,6	15,8	**1,42**	0,011022
C19orf44	chromosome 19 open reading frame 44	7,9	9,8	**1,42**	0,007234
FAM110A	family with sequence similarity 110 member A	6,1	7,0	**1,42**	0,03867
BAZ1A	bromodomain adjacent to zinc finger domain 1A	26,6	34,3	**1,42**	0,002507
GOLPH3L	golgi phosphoprotein 3 like	31,5	40,5	**1,42**	0,00277
SOCS3	suppressor of cytokine signaling 3	74,6	101,8	**1,42**	0,004764
SLC41A1	solute carrier family 41 member 1	54,5	70,5	**1,42**	0,004903
SNW1	SNW domain containing 1	53,3	64,9	**1,42**	0,00156
TUFT1	tuftelin 1	6,6	8,2	**1,42**	0,012045
RNF168	ring finger protein 168	23,4	28,1	**1,42**	0,001218
OXSM	3-oxoacyl-ACP synthase, mitochondrial	9,4	11,0	**1,42**	0,003433
ZNF721	zinc finger protein 721	11,0	13,8	**1,42**	0,005873
TULP3	tubby like protein 3	42,8	53,3	**1,42**	0,00202
PATL1	PAT1 homolog 1, processing body mRNA decay factor	31,8	41,2	**1,42**	0,006518
SNHG12	small nucleolar RNA host gene 12	8,7	9,8	**1,42**	0,008573
MRPL14	mitochondrial ribosomal protein L14	30,9	37,1	**1,42**	6,00E-04
VLDLR	very low density lipoprotein receptor	56,6	75,8	**1,42**	0,00584
DNAJC5	DnaJ heat shock protein family (Hsp40) member C5	100,0	125,8	**1,42**	0,005258
TRIM23	tripartite motif containing 23	25,6	33,9	**1,42**	0,006066
PPARD	peroxisome proliferator activated receptor delta	39,1	48,9	**1,42**	0,00149
MTMR14	myotubularin related protein 14	17,5	21,1	**1,42**	0,001018
QPCTL	glutaminyl-peptide cyclotransferase like	12,2	15,6	**1,42**	0,005636
THAP7	THAP domain containing 7	24,9	30,3	**1,42**	0,002038
UBN1	ubinuclein 1	48,6	59,6	**1,42**	0,004644
DHRS13	dehydrogenase/reductase 13	6,5	8,3	**1,42**	0,034424
ZNF396	zinc finger protein 396	4,8	6,1	**1,42**	0,0311
DAZAP2	DAZ associated protein 2	127,9	160,0	**1,42**	0,005571
TMED5	transmembrane p24 trafficking protein 5	52,8	68,6	**1,42**	0,001875
C10orf35	chromosome 10 open reading frame 35			**1,42**	0,025154
UNC119B	unc-119 lipid binding chaperone B	81,7	101,5	**1,42**	0,003457
WDR78	WD repeat domain 78	5,9	7,1	**1,41**	0,011686

CCDC134	coiled-coil domain containing 134	4,4	4,9	**1,41**	0,0256
TMEM115	transmembrane protein 115	36,9	46,7	**1,41**	0,001195
COG3	component of oligomeric golgi complex 3	34,3	43,1	**1,41**	0,002349
NECTIN3	nectin cell adhesion molecule 3	19,4	24,5	**1,41**	0,003409
RBMXL1	RNA binding motif protein, X-linked like 1	12,6	16,2	**1,41**	0,007512
EPS15L1	epidermal growth factor receptor pathway substrate 15 like 1	24,6	31,3	**1,41**	0,002019
FBXO42	F-box protein 42	21,4	26,2	**1,41**	0,000501
GTF2E1	general transcription factor IIE subunit 1	8,5	10,5	**1,41**	0,009121
SPATA2	spermatogenesis associated 2	12,3	16,0	**1,41**	0,005588
ASB6	ankyrin repeat and SOCS box containing 6	35,7	44,7	**1,41**	0,001761
RRP12	ribosomal RNA processing 12 homolog	22,8	27,7	**1,41**	0,00173
ACTR6	ARP6 actin-related protein 6 homolog	15,4	18,6	**1,41**	0,002428
FTX	FTX transcript, XIST regulator (non-protein coding)	12,9	17,5	**1,41**	0,011333
CCNYL1	cyclin Y like 1	23,6	28,4	**1,41**	0,000836
MORC2	MORC family CW-type zinc finger 2	35,9	45,0	**1,41**	0,00131
S100A3	S100 calcium binding protein A3	5,0	6,4	**1,41**	0,038735
USP13	ubiquitin specific peptidase 13 (isopeptidase T-3)	21,8	27,0	**1,41**	0,003534
TMEM67	transmembrane protein 67	13,7	17,0	**1,41**	0,00619
MTHFSD	methenyltetrahydrofolate synthetase domain containing	10,5	13,1	**1,41**	0,007282
SNX16	sorting nexin 16	7,7	9,8	**1,41**	0,014381
C1orf74	chromosome 1 open reading frame 74	4,7	5,6	**1,41**	0,020085
FRYL	FRY like transcription coactivator	36,1	47,2	**1,41**	0,027316
JMJD6	arginine demethylase and lysine hydroxylase	21,6	27,2	**1,41**	0,004414
ZNF529	zinc finger protein 529	7,2	9,1	**1,41**	0,018353
SLC16A13	solute carrier family 16 member 13	2,0	2,4	**1,41**	0,048033
LRIG2	leucine rich repeats and immunoglobulin like domains 2	17,0	20,7	**1,41**	0,003802
PELO	pelota homolog (Drosophila)	53,4	64,6	**1,41**	0,001438
CCDC93	coiled-coil domain containing 93	40,1	51,0	**1,41**	0,007735
SOX9	SRY-box 9	251,6	309,8	**1,41**	0,02368
SPATA2L	spermatogenesis associated 2 like	5,1	6,3	**1,41**	0,02793
PGM3	phosphoglucomutase 3	79,8	103,3	**1,41**	0,008573
CBLL1	Cbl proto-oncogene like 1	12,7	15,9	**1,41**	0,00952
C19orf25	chromosome 19 open reading frame 25	18,8	22,8	**1,41**	0,003036

ATL2	atlastin GTPase 2	19,6	24,2	**1,41**	0,007299
SLC25A32	solute carrier family 25 member 32	40,3	48,5	**1,41**	0,000806
PPWD1	peptidylprolyl isomerase domain and WD repeat containing 1	22,8	28,0	**1,41**	0,003059
FAM234B	family with sequence similarity 234 member B	4,8	5,6	**1,41**	0,019665
GPKOW	G-patch domain and KOW motifs	19,5	23,4	**1,41**	0,002205
ZNF302	zinc finger protein 302	29,7	37,3	**1,41**	0,002908
GDPD5	glycerophosphodiester phosphodiesterase domain containing 5	19,7	25,4	**1,40**	0,048027
MEPCE	methylphosphate capping enzyme	58,0	71,4	**1,40**	0,0023
SLC25A14	solute carrier family 25 member 14	7,1	7,9	**1,40**	0,009991
REXO1	RNA exonuclease 1 homolog	22,9	28,1	**1,40**	0,003206
RAE1	ribonucleic acid export 1	22,7	26,5	**1,40**	0,000888
TMEM128	transmembrane protein 128	24,7	29,2	**1,40**	0,000617
RFX2	regulatory factor X2	4,6	5,5	**1,40**	0,02697
WDR24	WD repeat domain 24	13,0	16,1	**1,40**	0,005402
TXNDC9	thioredoxin domain containing 9	34,0	40,5	**1,40**	0,001357
ANKRD10	ankyrin repeat domain 10	46,1	58,9	**1,40**	0,005963
GCLC	glutamate-cysteine ligase catalytic subunit	22,4	27,0	**1,40**	0,002552
MRM3	mitochondrial rRNA methyltransferase 3	11,4	13,7	**1,40**	0,006593
ZNF791	zinc finger protein 791	31,0	39,1	**1,40**	0,003062
TP53INP1	tumor protein p53 inducible nuclear protein 1	9,8	12,1	**1,40**	0,009316
SPAG1	sperm associated antigen 1	5,3	6,8	**1,40**	0,031405
TEX10	testis expressed 10	18,6	22,7	**1,40**	0,004456
MKRN1	makorin ring finger protein 1	63,6	76,0	**1,40**	0,001274
PHF20	PHD finger protein 20	46,3	59,4	**1,40**	0,004502
KLHL24	kelch like family member 24	25,1	33,3	**1,40**	0,014272
DHX58	DExH-box helicase 58	4,1	5,0	**1,40**	0,035158
CFAP44	cilia and flagella associated protein 44	3,2	4,2	**1,40**	0,047584
CCDC97	coiled-coil domain containing 97	30,6	37,7	**1,40**	0,001955
MAPK8IP1	mitogen-activated protein kinase 8 interacting protein 1	10,1	12,5	**1,40**	0,017331
ATXN2L	ataxin 2 like	70,2	88,2	**1,40**	0,008669
LRIF1	ligand dependent nuclear receptor interacting factor 1	17,1	21,8	**1,40**	0,009781
PPM1B	protein phosphatase, Mg2+/Mn2+ dependent 1B	24,3	29,2	**1,40**	0,001163
PSMD12	proteasome 26S subunit, non-ATPase 12	59,1	72,0	**1,40**	0,002992

LGMN	legumain	144,6	183,5	**1,40**	0,007911
ZNF549	zinc finger protein 549	4,3	5,4	**1,40**	0,049732
KLF3	Kruppel like factor 3	92,4	112,6	**1,40**	0,009106
ULK1	unc-51 like autophagy activating kinase 1	27,4	37,1	**1,40**	0,003528
LINC00526	long intergenic non-protein coding RNA 526	2,1	2,4	**1,40**	0,042458
ASL	argininosuccinate lyase	26,0	31,7	**1,40**	0,003458
EZH1	enhancer of zeste 1 polycomb repressive complex 2 subunit	30,6	38,3	**1,40**	0,002967
LONP1	lon peptidase 1, mitochondrial	84,5	101,4	**1,40**	0,004348
MFSD9	major facilitator superfamily domain containing 9	4,8	6,0	**1,40**	0,025531
CHMP1B	charged multivesicular body protein 1B	45,8	59,6	**1,40**	0,003194
LINC00963	long intergenic non-protein coding RNA 963	30,5	38,0	**1,40**	0,000873
PSTK	phosphoseryl-tRNA kinase	2,0	2,4	**1,40**	0,043305
GRK5	G protein-coupled receptor kinase 5	44,9	55,6	**1,40**	0,004401
RPA2	replication protein A2	39,3	45,1	**1,39**	0,002224
CYP27A1	cytochrome P450 family 27 subfamily A member 1	40,1	52,8	**1,39**	0,006769
C16orf91	chromosome 16 open reading frame 91	7,3	8,8	**1,39**	0,011785
9.syys	septin 9			**1,39**	0,018343
ZSCAN25	zinc finger and SCAN domain containing 25	10,0	12,1	**1,39**	0,010611
SS18	SS18, nBAF chromatin remodeling complex subunit	61,9	75,9	**1,39**	0,003407
SPPL3	signal peptide peptidase like 3	51,3	64,7	**1,39**	0,003127
THAP3	THAP domain containing 3	10,0	11,8	**1,39**	0,01015
MAD2L1BP	MAD2L1 binding protein	17,7	21,5	**1,39**	0,003596
TBC1D12	TBC1 domain family member 12	12,1	15,1	**1,39**	0,007618
SNRNP40	small nuclear ribonucleoprotein U5 subunit 40	26,5	30,1	**1,39**	0,001099
CMC1	C-X9-C motif containing 1	12,8	14,4	**1,39**	0,00353
GSK3A	glycogen synthase kinase 3 alpha	34,8	42,3	**1,39**	0,000798
STXBP3	syntaxin binding protein 3	47,7	59,4	**1,39**	0,002654
YARS	tyrosyl-tRNA synthetase	108,3	131,4	**1,39**	0,007643
ZNF644	zinc finger protein 644	31,9	40,3	**1,39**	0,006976
TRIM33	tripartite motif containing 33	28,7	36,4	**1,39**	0,013204
TMEM87A	transmembrane protein 87A	63,7	75,3	**1,39**	0,002753
RNMT	RNA guanine-7 methyltransferase	30,5	38,8	**1,39**	0,005308
PLEKHA3	pleckstrin homology domain containing A3	33,9	43,0	**1,39**	0,003158

HSPA9	heat shock protein family A (Hsp70) member 9	329,1	391,8	**1,39**	0,033297
NBN	nibrin	48,3	57,2	**1,39**	0,003661
FAM13A	family with sequence similarity 13 member A	20,5	25,2	**1,39**	0,018189
IP6K2	inositol hexakisphosphate kinase 2	26,8	33,0	**1,39**	0,002821
CREB1	cAMP responsive element binding protein 1	37,5	46,0	**1,39**	0,004674
ASS1	argininosuccinate synthase 1	150,7	208,1	**1,39**	0,023438
NAPB	NSF attachment protein beta	6,5	7,7	**1,39**	0,016675
EID2B	EP300 interacting inhibitor of differentiation 2B	3,3	4,1	**1,39**	0,038942
CTTNBP2NL	CTTNBP2 N-terminal like	22,0	27,4	**1,39**	0,020863
PSMA3	proteasome subunit alpha 3	47,4	57,4	**1,39**	0,002188
ZNF446	zinc finger protein 446	6,9	8,2	**1,39**	0,015163
TBC1D17	TBC1 domain family member 17	46,8	58,7	**1,39**	0,001986
SCAMP2	secretory carrier membrane protein 2	79,9	96,9	**1,39**	0,003468
BNIP3	BCL2 interacting protein 3	152,1	191,1	**1,39**	0,037317
ITGA1	integrin subunit alpha 1	77,9	102,3	**1,39**	0,031202
CYTOR	cytoskeleton regulator RNA	18,1	23,1	**1,39**	0,010141
BLOC1S3	biogenesis of lysosomal organelles complex 1 subunit 3	12,1	15,3	**1,39**	0,01049
MFSD4B	major facilitator superfamily domain containing 4B	4,1	4,4	**1,39**	0,025607
DCP1A	decapping mRNA 1A	31,5	38,1	**1,38**	0,004986
SIPA1L3	signal induced proliferation associated 1 like 3	27,3	33,4	**1,38**	0,013217
PAXBP1	PAX3 and PAX7 binding protein 1	13,6	16,6	**1,38**	0,009913
SNX11	sorting nexin 11	11,7	13,7	**1,38**	0,00482
MRPS18A	mitochondrial ribosomal protein S18A	22,9	26,8	**1,38**	0,001398
S100A11	S100 calcium binding protein A11	207,9	256,6	**1,38**	0,026164
ASAH2B	N-acylsphingosine amidohydrolase 2B	2,3	2,5	**1,38**	0,048153
KDM4B	lysine demethylase 4B	31,0	38,1	**1,38**	0,002005
GSTO1	glutathione S-transferase omega 1	105,7	128,7	**1,38**	0,012079
ZNF696	zinc finger protein 696	7,1	8,7	**1,38**	0,027401
H1F0	H1 histone family member 0	171,2	211,4	**1,38**	0,015143
LIPT1	lipoyltransferase 1	3,2	3,7	**1,38**	0,036408
KHNYN	KH and NYN domain containing	53,1	67,1	**1,38**	0,005403
SMG8	SMG8, nonsense mediated mRNA decay factor	14,8	18,3	**1,38**	0,009749
CCDC57	coiled-coil domain containing 57	11,6	14,2	**1,38**	0,013238

TBC1D10A	TBC1 domain family member 10A	8,8	10,7	**1,38**	0,009683
AAR2	AAR2 splicing factor homolog	30,1	35,4	**1,38**	0,001695
TPST2	tyrosylprotein sulfotransferase 2	11,9	15,6	**1,38**	0,028762
PARP10	poly(ADP-ribose) polymerase family member 10	9,9	12,1	**1,38**	0,016245
KIAA0907	KIAA0907			**1,38**	0,002145
TTN-AS1	TTN antisense RNA 1	4,9	5,9	**1,38**	0,044113
TWNK	twinkle mtDNA helicase	9,6	11,4	**1,38**	0,021171
ARMT1	acidic residue methyltransferase 1	19,1	23,8	**1,38**	0,005921
SYVN1	synoviolin 1	67,8	86,8	**1,38**	0,01666
INSIG1	insulin induced gene 1	128,0	167,1	**1,38**	0,047127
ORMDL1	ORMDL sphingolipid biosynthesis regulator 1	24,6	28,7	**1,38**	0,001684
PRICKLE2	prickle planar cell polarity protein 2	37,5	47,8	**1,38**	0,010585
ZNF160	zinc finger protein 160	22,7	28,1	**1,38**	0,009126
C18orf8	chromosome 18 open reading frame 8			**1,38**	0,004414
LRWD1	leucine rich repeats and WD repeat domain containing 1	19,3	21,4	**1,38**	0,010836
TBC1D22B	TBC1 domain family member 22B	8,7	11,0	**1,38**	0,029191
TOPORS	TOP1 binding arginine/serine rich protein	31,0	38,4	**1,38**	0,00649
HMGCL	3-hydroxymethyl-3-methylglutaryl-CoA lyase	37,8	44,2	**1,38**	0,001328
TERF2IP	TERF2 interacting protein	98,1	117,2	**1,38**	0,007357
ALG13	ALG13, UDP-N-acetylglucosaminyltransferase subunit	18,1	21,9	**1,38**	0,002933
AAGAB	alpha- and gamma-adaptin binding protein	34,3	39,7	**1,38**	0,001706
TMEM127	transmembrane protein 127	73,2	87,6	**1,38**	0,004736
NEDD4	neural precursor cell expressed, developmentally down-regulated 4, E	22,7	27,9	**1,38**	0,011083
MYO1E	myosin IE	56,7	66,1	**1,38**	0,003879
ABI1	abl interactor 1	67,6	83,5	**1,38**	0,006422
PPP1R12C	protein phosphatase 1 regulatory subunit 12C	60,5	71,3	**1,38**	0,003922
WDR20	WD repeat domain 20	16,8	21,2	**1,38**	0,007148
TMEM216	transmembrane protein 216	16,2	18,1	**1,38**	0,002836
ZBTB48	zinc finger and BTB domain containing 48	8,4	9,9	**1,38**	0,010601
KIRREL	kin of IRRE like (Drosophila)			**1,38**	0,034851
B4GALT3	beta-1,4-galactosyltransferase 3	23,1	26,5	**1,38**	0,00353
MSANTD3	Myb/SANT DNA binding domain containing 3	34,3	43,0	**1,37**	0,002785
MYNN	myoneurin	18,0	21,5	**1,37**	0,008434

CLCN7	chloride voltage-gated channel 7	51,3	63,5	**1,37**	0,008915
RPS6KA3	ribosomal protein S6 kinase A3	71,2	92,6	**1,37**	0,012141
ENTPD4	ectonucleoside triphosphate diphosphohydrolase 4	35,4	43,2	**1,37**	0,005879
CYCS	cytochrome c, somatic	67,8	77,7	**1,37**	0,005224
TUG1	taurine up-regulated 1 (non-protein coding)	98,1	120,9	**1,37**	0,033106
MGEA5	meningioma expressed antigen 5 (hyaluronidase)			**1,37**	0,016656
GGA3	golgi associated, gamma adaptin ear containing, ARF binding protein 3	24,9	31,0	**1,37**	0,006775
UBE2W	ubiquitin conjugating enzyme E2 W	18,7	22,4	**1,37**	0,00465
AHI1	Abelson helper integration site 1	34,9	42,1	**1,37**	0,005007
ORC5	origin recognition complex subunit 5	11,0	12,7	**1,37**	0,00928
RPP40	ribonuclease P/MRP subunit p40	4,8	5,1	**1,37**	0,031155
ZNF425	zinc finger protein 425	6,4	7,5	**1,37**	0,022799
GON4L	gon-4 like	19,5	24,4	**1,37**	0,030621
TMEM69	transmembrane protein 69	17,2	20,6	**1,37**	0,005187
SCAF4	SR-related CTD associated factor 4	27,7	34,3	**1,37**	0,007694
ZBTB26	zinc finger and BTB domain containing 26	8,1	10,0	**1,37**	0,024102
UPF3B	UPF3 regulator of nonsense transcripts homolog B (yeast)	14,2	17,2	**1,37**	0,00867
TAB3	TGF-beta activated kinase 1 and MAP3K7 binding protein 3	19,4	25,8	**1,37**	0,034225
NBR1	NBR1, autophagy cargo receptor	181,2	221,7	**1,37**	0,031943
TMEM138	transmembrane protein 138	13,5	16,4	**1,37**	0,01089
RAB14	RAB14, member RAS oncogene family	110,1	130,2	**1,37**	0,011695
MYO5B	myosin VB	52,3	66,0	**1,37**	0,040729
ZNF646	zinc finger protein 646	17,5	21,8	**1,37**	0,02145
FAM76B	family with sequence similarity 76 member B	9,6	11,8	**1,37**	0,015992
C1orf109	chromosome 1 open reading frame 109	16,6	18,9	**1,37**	0,004012
HMGXB4	HMG-box containing 4	25,9	30,3	**1,37**	0,008058
LAMTOR3	late endosomal/lysosomal adaptor, MAPK and MTOR activator 3	30,6	36,8	**1,37**	0,002714
PTBP2	polypyrimidine tract binding protein 2	9,5	11,2	**1,37**	0,019906
MTERF3	mitochondrial transcription termination factor 3	10,9	12,6	**1,37**	0,010599
KLF9	Kruppel like factor 9	18,2	23,5	**1,37**	0,00554
ERCC4	ERCC excision repair 4, endonuclease catalytic subunit	13,2	16,5	**1,37**	0,044057
JUND	JunD proto-oncogene, AP-1 transcription factor subunit	90,5	111,7	**1,37**	0,008618
RIT1	Ras like without CAAX 1	11,5	14,3	**1,36**	0,015318

TXLNG	taxilin gamma	14,9	18,3	**1,36**	0,020779
EPB41L4A-AS	EPB41L4A antisense RNA 1	15,1	18,0	**1,36**	0,009107
WDR66	WD repeat domain 66	6,0	7,2	**1,36**	0,041278
MAGOH	mago homolog, exon junction complex core component	22,9	25,8	**1,36**	0,001576
PSMD7	proteasome 26S subunit, non-ATPase 7	73,8	86,8	**1,36**	0,007623
RHBDD2	rhomboid domain containing 2	37,9	44,4	**1,36**	0,002854
SIAH2	siah E3 ubiquitin protein ligase 2	36,1	43,8	**1,36**	0,001383
GINS3	GINS complex subunit 3	7,8	8,5	**1,36**	0,035295
GADD45A	growth arrest and DNA damage inducible alpha	52,6	61,9	**1,36**	0,002389
FLVCR2	feline leukemia virus subgroup C cellular receptor family member 2	4,3	5,2	**1,36**	0,047552
PPM1A	protein phosphatase, Mg2+/Mn2+ dependent 1A	40,8	50,5	**1,36**	0,002803
ZNF638	zinc finger protein 638	76,1	92,0	**1,36**	0,016389
AGFG1	ArfGAP with FG repeats 1	56,2	67,8	**1,36**	0,007904
RAB8B	RAB8B, member RAS oncogene family	28,8	35,5	**1,36**	0,010576
TTC39B	tetratricopeptide repeat domain 39B	10,7	12,7	**1,36**	0,022545
PEF1	penta-EF-hand domain containing 1	59,7	69,2	**1,36**	0,003891
TRIM32	tripartite motif containing 32	19,9	23,6	**1,36**	0,01254
RNF146	ring finger protein 146	35,9	43,4	**1,36**	0,003912
GLTP	glycolipid transfer protein	88,5	103,5	**1,36**	0,011223
BLZF1	basic leucine zipper nuclear factor 1	43,9	51,5	**1,36**	0,00499
PHKG2	phosphorylase kinase catalytic subunit gamma 2	8,9	9,4	**1,36**	0,012342
DNAJC30	DnaJ heat shock protein family (Hsp40) member C30	15,1	17,1	**1,36**	0,004542
NFYA	nuclear transcription factor Y subunit alpha	32,6	39,3	**1,36**	0,011038
KIF21A	kinesin family member 21A	12,7	14,4	**1,36**	0,011478
CLINT1	clathrin interactor 1	109,1	127,9	**1,36**	0,01424
ZNF213-AS1	ZNF213 antisense RNA 1 (head to head)	4,6	5,3	**1,36**	0,033872
CASP7	caspase 7	13,5	15,8	**1,36**	0,020945
TSC1	tuberous sclerosis 1	33,2	40,2	**1,36**	0,014085
STYXL1	serine/threonine/tyrosine interacting like 1	21,2	23,5	**1,36**	0,003221
MKLN1	muskelin 1	58,8	74,2	**1,36**	0,015741
CPEB3	cytoplasmic polyadenylation element binding protein 3	8,1	8,8	**1,36**	0,02819
CPEB4	cytoplasmic polyadenylation element binding protein 4	60,9	75,5	**1,36**	0,019872
LRCH1	leucine rich repeats and calponin homology domain containing 1	17,2	21,5	**1,36**	0,024618

EMC3	ER membrane protein complex subunit 3	66,3	80,6	**1,36**	0,006402
TBP	TATA-box binding protein	9,4	11,1	**1,36**	0,021127
UVSSA	UV stimulated scaffold protein A	19,8	24,4	**1,36**	0,023603
CHST15	carbohydrate sulfotransferase 15	32,0	37,2	**1,36**	0,027471
PTP4A1	protein tyrosine phosphatase type IVA, member 1	126,0	156,3	**1,36**	0,01649
PTRH2	peptidyl-tRNA hydrolase 2	13,5	15,6	**1,36**	0,012954
YWHAG	tyrosine 3-monooxygenase/tryptophan 5-monooxygenase activation	151,5	179,4	**1,36**	0,025543
PREB	prolactin regulatory element binding	49,9	58,6	**1,36**	0,009219
ZCCHC7	zinc finger CCHC-type containing 7	18,2	21,7	**1,36**	0,00859
ZNF771	zinc finger protein 771	6,9	8,0	**1,36**	0,022492
TPRKB	TP53RK binding protein	15,6	17,1	**1,36**	0,006017
SKIL	SKI like proto-oncogene	47,3	58,8	**1,35**	0,015075
ANKRA2	ankyrin repeat family A member 2	9,1	10,9	**1,35**	0,013748
MBD6	methyl-CpG binding domain protein 6	29,0	35,0	**1,35**	0,005286
SCO1	SCO1, cytochrome c oxidase assembly protein	26,7	30,4	**1,35**	0,005943
HOXC9	homeobox C9	4,9	6,1	**1,35**	0,03747
EDRF1	erythroid differentiation regulatory factor 1	12,4	13,9	**1,35**	0,012063
ZNF596	zinc finger protein 596	4,7	5,6	**1,35**	0,041259
PPP1R8	protein phosphatase 1 regulatory subunit 8	40,8	46,4	**1,35**	0,003347
CUL1	cullin 1	57,3	66,8	**1,35**	0,006124
CTDP1	CTD phosphatase subunit 1	7,1	8,7	**1,35**	0,03496
ATP6V0B	ATPase H+ transporting V0 subunit b	59,8	69,4	**1,35**	0,006121
ALAS1	5'-aminolevulinate synthase 1	26,5	31,5	**1,35**	0,00232
SLC30A7	solute carrier family 30 member 7	32,7	39,6	**1,35**	0,00684
FBXO46	F-box protein 46	6,6	8,1	**1,35**	0,039597
LYAR	Ly1 antibody reactive	17,5	18,9	**1,35**	0,008016
MBD1	methyl-CpG binding domain protein 1	48,2	56,4	**1,35**	0,006569
PEX14	peroxisomal biogenesis factor 14	17,6	19,7	**1,35**	0,00695
THAP9-AS1	THAP9 antisense RNA 1	20,8	23,2	**1,35**	0,004031
C9orf85	chromosome 9 open reading frame 85	7,3	8,4	**1,35**	0,025374
FAM193B	family with sequence similarity 193 member B	20,5	24,5	**1,35**	0,009763
COG8	component of oligomeric golgi complex 8	5,7	6,7	**1,35**	0,043984
CCDC22	coiled-coil domain containing 22	12,7	15,0	**1,35**	0,016114

MAPK6	mitogen-activated protein kinase 6	83,3	100,5	**1,35**	0,019158
PLPP5	phospholipid phosphatase 5	62,3	73,3	**1,35**	0,011122
ZFPL1	zinc finger protein like 1	30,9	35,9	**1,35**	0,005266
SPCS2	signal peptidase complex subunit 2	35,6	41,2	**1,35**	0,008073
KDSR	3-ketodihydrosphingosine reductase	104,8	124,8	**1,35**	0,01931
PTPN1	protein tyrosine phosphatase, non-receptor type 1	43,4	51,9	**1,35**	0,00559
USP37	ubiquitin specific peptidase 37	14,3	16,1	**1,35**	0,014426
HTR7P1	5-hydroxytryptamine receptor 7 pseudogene 1	5,4	6,5	**1,35**	0,047028
ZSWIM8	zinc finger SWIM-type containing 8	70,1	85,6	**1,35**	0,015962
PSTPIP2	proline-serine-threonine phosphatase interacting protein 2	12,3	14,0	**1,35**	0,026165
TMEM206	transmembrane protein 206	3,5	4,5	**1,35**	0,048835
CNOT6	CCR4-NOT transcription complex subunit 6	18,1	21,8	**1,35**	0,011046
PLRG1	pleiotropic regulator 1	60,9	71,1	**1,35**	0,007177
PPIL4	peptidylprolyl isomerase like 4	31,1	36,8	**1,35**	0,008104
CCDC9	coiled-coil domain containing 9	26,3	29,5	**1,35**	0,008191
TMEM209	transmembrane protein 209	22,2	24,6	**1,35**	0,008865
YIPF4	Yip1 domain family member 4	50,4	57,6	**1,35**	0,003998
PLEKHM2	pleckstrin homology and RUN domain containing M2	75,8	91,9	**1,35**	0,010386
AK9	adenylate kinase 9	8,1	9,4	**1,35**	0,027369
TIPRL	TOR signaling pathway regulator	37,4	42,4	**1,35**	0,002992
RSBN1	round spermatid basic protein 1	17,8	21,5	**1,35**	0,015771
POLR3A	RNA polymerase III subunit A	27,7	33,1	**1,34**	0,010813
GRINA	glutamate ionotropic receptor NMDA type subunit associated protein	114,8	138,2	**1,34**	0,016245
GABPB1-AS1	GABPB1 antisense RNA 1	14,1	17,6	**1,34**	0,016123
ANKRD11	ankyrin repeat domain 11	117,3	139,5	**1,34**	0,026152
OTUD6B-AS1	OTUD6B antisense RNA 1 (head to head)	40,3	46,9	**1,34**	0,006547
CDK2AP2	cyclin dependent kinase 2 associated protein 2	41,0	51,1	**1,34**	0,043469
MORN4	MORN repeat containing 4	11,1	12,2	**1,34**	0,02378
UTP15	UTP15, small subunit processome component	12,1	13,8	**1,34**	0,020141
GLRX2	glutaredoxin 2	20,9	22,6	**1,34**	0,006236
CASC3	cancer susceptibility 3	83,3	98,5	**1,34**	0,014202
MAP3K10	mitogen-activated protein kinase kinase kinase 10	14,3	16,6	**1,34**	0,017252
MED20	mediator complex subunit 20	9,4	10,8	**1,34**	0,017214

FGFR1OP	FGFR1 oncogene partner	7,9	9,1	**1,34**	0,01901
ESCO1	establishment of sister chromatid cohesion N-acetyltransferase 1	13,6	15,7	**1,34**	0,01742
ZNF335	zinc finger protein 335	19,9	24,2	**1,34**	0,025902
ZNF562	zinc finger protein 562	18,8	22,6	**1,34**	0,022263
PRKCE	protein kinase C epsilon	6,4	7,3	**1,34**	0,037927
SNRPD1	small nuclear ribonucleoprotein D1 polypeptide	36,7	39,2	**1,34**	0,00307
NHLRC2	NHL repeat containing 2	29,8	35,5	**1,34**	0,027751
NDFIP2	Nedd4 family interacting protein 2	36,2	41,4	**1,34**	0,004885
ATP6V1C1	ATPase H+ transporting V1 subunit C1	47,6	55,3	**1,34**	0,007837
PXK	PX domain containing serine/threonine kinase like	40,7	45,8	**1,34**	0,01613
UBXN1	UBX domain protein 1	43,5	49,8	**1,34**	0,003655
ISY1	ISY1 splicing factor homolog	12,6	13,2	**1,34**	0,015579
MED19	mediator complex subunit 19	14,6	16,5	**1,34**	0,013765
LINS1	lines homolog 1	6,4	7,3	**1,34**	0,033464
SLC25A4	solute carrier family 25 member 4	13,7	15,7	**1,34**	0,016291
CENPBD1P1	CENPB DNA-binding domains containing 1 pseudogene 1	19,8	23,4	**1,34**	0,011329
SRF	serum response factor	40,2	47,3	**1,34**	0,007889
LMLN	leishmanolysin like peptidase	14,5	17,7	**1,34**	0,046307
WFS1	wolframin ER transmembrane glycoprotein	58,3	68,2	**1,33**	0,013029
P2RX4	purinergic receptor P2X 4	19,9	23,6	**1,33**	0,011278
ATG13	autophagy related 13	58,8	68,1	**1,33**	0,009422
SPOP	speckle type BTB/POZ protein	53,4	58,3	**1,33**	0,003341
C20orf24	chromosome 20 open reading frame 24			**1,33**	0,023773
UVRAG	UV radiation resistance associated	16,1	18,4	**1,33**	0,02006
ADK	adenosine kinase	83,5	94,0	**1,33**	0,011659
DUSP12	dual specificity phosphatase 12	18,1	20,4	**1,33**	0,009294
RABIF	RAB interacting factor	11,2	13,6	**1,33**	0,020601
PCGF1	polycomb group ring finger 1	11,3	13,1	**1,33**	0,010923
LHFPL2	lipoma HMGIC fusion partner-like 2	108,0	133,3	**1,33**	0,040774
NUP188	nucleoporin 188	41,1	45,6	**1,33**	0,016433
LRRC23	leucine rich repeat containing 23	8,8	9,3	**1,33**	0,02395
PI4KB	phosphatidylinositol 4-kinase beta	48,1	56,9	**1,33**	0,009604
POLR3K	RNA polymerase III subunit K	9,2	9,8	**1,33**	0,031998

RAB35	RAB35, member RAS oncogene family	44,0	51,9	**1,33**	0,00827
FCF1	FCF1 rRNA-processing protein	51,9	58,4	**1,33**	0,004348
ASNSD1	asparagine synthetase domain containing 1	42,8	48,1	**1,33**	0,003249
C12orf65	chromosome 12 open reading frame 65	11,3	13,0	**1,33**	0,017402
PPIP5K1	diphosphoinositol pentakisphosphate kinase 1	8,2	8,8	**1,33**	0,034157
MED10	mediator complex subunit 10	31,7	36,1	**1,33**	0,009765
MED26	mediator complex subunit 26	5,7	6,8	**1,33**	0,043844
MED6	mediator complex subunit 6	15,2	16,9	**1,33**	0,009375
BTBD19	BTB domain containing 19	7,5	8,8	**1,33**	0,04479
DHFR2	dihydrofolate reductase 2	15,1	17,6	**1,33**	0,026602
EDF1	endothelial differentiation related factor 1	168,0	189,1	**1,33**	0,031855
C22orf46	chromosome 22 open reading frame 46	32,1	36,3	**1,33**	0,011192
UBOX5	U-box domain containing 5	8,9	9,5	**1,33**	0,022804
RNF220	ring finger protein 220	48,2	54,7	**1,33**	0,004691
TPM1	tropomyosin 1 (alpha)	117,2	131,2	**1,33**	0,049931
TMEM11	transmembrane protein 11	19,2	21,3	**1,33**	0,006963
WDR53	WD repeat domain 53	6,3	7,0	**1,33**	0,032027
IKBKB	inhibitor of nuclear factor kappa B kinase subunit beta	22,2	26,7	**1,33**	0,016197
ZNF212	zinc finger protein 212	10,6	12,5	**1,33**	0,026451
TSG101	tumor susceptibility 101	76,8	87,5	**1,33**	0,008759
TXNRD2	thioredoxin reductase 2	14,6	15,7	**1,33**	0,01686
SRP19	signal recognition particle 19	14,9	17,0	**1,33**	0,020961
ZNF706	zinc finger protein 706	25,8	28,4	**1,33**	0,003028
NCOA5	nuclear receptor coactivator 5	29,4	34,1	**1,33**	0,012887
SLC35C2	solute carrier family 35 member C2	39,9	45,7	**1,33**	0,005093
RBM23	RNA binding motif protein 23	46,8	53,1	**1,33**	0,005955
COL4A3BP	collagen type IV alpha 3 binding protein	37,2	43,5	**1,33**	0,020004
CEP95	centrosomal protein 95	13,2	14,7	**1,33**	0,018101
WSB1	WD repeat and SOCS box containing 1	71,7	84,9	**1,33**	0,017113
SENP5	SUMO1/sentrin specific peptidase 5	26,6	30,8	**1,33**	0,009346
VCPKMT	valosin containing protein lysine methyltransferase	5,0	5,7	**1,33**	0,041709
TALDO1	transaldolase 1	131,3	149,2	**1,33**	0,024894
ZXDC	ZXD family zinc finger C	25,2	29,9	**1,33**	0,018558

XRRA1	X-ray radiation resistance associated 1	18,0	19,6	**1,33**	0,018505
TMEM50A	transmembrane protein 50A	89,6	106,1	**1,33**	0,013153
HERPUD2	HERPUD family member 2	32,4	36,7	**1,33**	0,00586
TEPSIN	TEPSIN, adaptor related protein complex 4 accessory protein	9,9	11,8	**1,33**	0,032823
ENTPD5	ectonucleoside triphosphate diphosphohydrolase 5	25,9	30,4	**1,33**	0,018566
RP9	retinitis pigmentosa 9 (autosomal dominant)	6,4	7,1	**1,33**	0,042884
HELQ	helicase, POLQ-like	12,4	14,4	**1,33**	0,044232
TIMM17A	translocase of inner mitochondrial membrane 17A	58,6	66,1	**1,33**	0,009947
MIER3	MIER family member 3	14,1	16,5	**1,33**	0,032002
ZNF271P	zinc finger protein 271, pseudogene	24,5	28,7	**1,32**	0,015603
PRKRIP1	PRKR interacting protein 1 (IL11 inducible)	18,5	19,9	**1,32**	0,006896
EDEM1	ER degradation enhancing alpha-mannosidase like protein 1	57,5	68,1	**1,32**	0,021773
CHAC1	ChaC glutathione specific gamma-glutamylcyclotransferase 1	27,7	32,2	**1,32**	0,011034
MTG2	mitochondrial ribosome associated GTPase 2	23,4	26,2	**1,32**	0,008229
HCCS	holocytochrome c synthase	14,5	17,4	**1,32**	0,016044
MRPL50	mitochondrial ribosomal protein L50	19,2	21,9	**1,32**	0,013057
MYPOP	Myb related transcription factor, partner of profilin	7,2	8,8	**1,32**	0,046571
EXOSC3	exosome component 3	12,8	14,1	**1,32**	0,021997
RHEB	Ras homolog enriched in brain	88,7	100,2	**1,32**	0,011968
SELENOI	selenoprotein I	31,0	35,9	**1,32**	0,034318
ZNF787	zinc finger protein 787	25,6	29,5	**1,32**	0,009964
C12orf29	chromosome 12 open reading frame 29	17,3	19,4	**1,32**	0,011224
SLC35A5	solute carrier family 35 member A5	53,2	61,8	**1,32**	0,010868
TTC17	tetratricopeptide repeat domain 17	56,0	66,7	**1,32**	0,018364
KTI12	KTI12 chromatin associated homolog	7,6	8,5	**1,32**	0,038207
CCNT2	cyclin T2	24,8	29,6	**1,32**	0,019833
TBRG1	transforming growth factor beta regulator 1	26,8	31,4	**1,32**	0,008569
CNDP2	carnosine dipeptidase 2	48,8	54,6	**1,32**	0,011822
ARL6IP1	ADP ribosylation factor like GTPase 6 interacting protein 1	51,6	51,4	**1,32**	0,021679
CAPN7	calpain 7	41,5	47,9	**1,32**	0,012493
ALKBH1	alkB homolog 1, histone H2A dioxygenase	8,0	9,3	**1,32**	0,037794
RPL36AL	ribosomal protein L36a like	94,1	107,8	**1,32**	0,022486
BTBD9	BTB domain containing 9	15,0	17,3	**1,32**	0,022196

IPPK	inositol-pentakisphosphate 2-kinase	11,2	12,5	**1,32**	0,022268
SRC	SRC proto-oncogene, non-receptor tyrosine kinase	25,0	28,1	**1,32**	0,019351
ANAPC13	anaphase promoting complex subunit 13	34,0	35,8	**1,32**	0,003284
NMT1	N-myristoyltransferase 1	104,2	118,6	**1,32**	0,022161
RNF44	ring finger protein 44	20,0	23,7	**1,32**	0,014836
BCR	BCR, RhoGEF and GTPase activating protein	48,8	56,7	**1,32**	0,017867
CFAP20	cilia and flagella associated protein 20	30,4	32,9	**1,32**	0,003621
SETD4	SET domain containing 4	10,5	11,5	**1,32**	0,020924
CELF1	CUGBP Elav-like family member 1	40,5	46,9	**1,32**	0,011435
PUM1	pumilio RNA binding family member 1	59,5	70,4	**1,32**	0,028832
ZNF687	zinc finger protein 687	22,4	26,4	**1,32**	0,014575
ATG4B	autophagy related 4B cysteine peptidase	35,5	40,2	**1,32**	0,006972
TEX2	testis expressed 2	32,8	37,7	**1,32**	0,016064
PDE8A	phosphodiesterase 8A	34,4	39,2	**1,32**	0,008011
TRIM21	tripartite motif containing 21	10,8	12,4	**1,32**	0,033374
GTPBP10	GTP binding protein 10	14,0	16,4	**1,32**	0,02495
ARL8A	ADP ribosylation factor like GTPase 8A	35,7	42,1	**1,32**	0,009831
MRPL54	mitochondrial ribosomal protein L54	20,3	20,9	**1,32**	0,019625
DOHH	deoxyhypusine hydroxylase	18,4	20,2	**1,32**	0,016448
C17orf80	chromosome 17 open reading frame 80	19,3	21,8	**1,32**	0,013125
CSTF2	cleavage stimulation factor subunit 2	12,6	13,8	**1,32**	0,032888
CAAP1	caspase activity and apoptosis inhibitor 1	13,5	15,2	**1,32**	0,024618
CCNDBP1	cyclin D1 binding protein 1	40,6	45,8	**1,32**	0,008709
HAGH	hydroxyacylglutathione hydrolase	26,3	30,0	**1,32**	0,012689
OTUD7B	OTU deubiquitinase 7B	42,0	48,0	**1,32**	0,021342
ZNF2	zinc finger protein 2	5,8	6,2	**1,32**	0,04226
FZD1	frizzled class receptor 1	52,7	57,8	**1,32**	0,011613
DNTTIP1	deoxynucleotidyltransferase terminal interacting protein 1	23,0	26,3	**1,32**	0,009434
GGA1	golgi associated, gamma adaptin ear containing, ARF binding protein [illegible]	34,5	40,7	**1,32**	0,011327
ACAD10	acyl-CoA dehydrogenase family member 10	10,1	11,5	**1,32**	0,03024
NRBF2	nuclear receptor binding factor 2	24,9	28,0	**1,31**	0,005289
PDCD5	programmed cell death 5	51,1	58,3	**1,31**	0,010695
CCS	copper chaperone for superoxide dismutase	19,9	22,3	**1,31**	0,006663

ATP6V1H	ATPase H+ transporting V1 subunit H	48,4	54,6	**1,31**	0,012683
ALS2	ALS2, alsin Rho guanine nucleotide exchange factor	17,7	19,8	**1,31**	0,024964
ZRANB1	zinc finger RANBP2-type containing 1	34,8	40,2	**1,31**	0,008437
C1orf43	chromosome 1 open reading frame 43	194,8	216,0	**1,31**	0,039908
UPF2	UPF2 regulator of nonsense transcripts homolog (yeast)	53,5	60,7	**1,31**	0,017791
SLC12A2	solute carrier family 12 member 2	18,2	20,6	**1,31**	0,028284
LYSMD3	LysM domain containing 3	32,9	39,5	**1,31**	0,021309
CAPN10	calpain 10	12,2	14,1	**1,31**	0,029063
LEMD2	LEM domain containing 2	58,9	64,1	**1,31**	0,008107
IFT27	intraflagellar transport 27	16,4	17,8	**1,31**	0,017785
NPHP4	nephrocystin 4	14,6	16,1	**1,31**	0,023834
UMAD1	UBAP1-MVB12-associated (UMA) domain containing 1	19,6	22,0	**1,31**	0,011225
CDK12	cyclin dependent kinase 12	38,6	46,9	**1,31**	0,040894
ZNF226	zinc finger protein 226	8,3	9,3	**1,31**	0,04529
PLPP6	phospholipid phosphatase 6	10,3	11,9	**1,31**	0,025933
FAM219A	family with sequence similarity 219 member A	33,7	39,0	**1,31**	0,017947
SEC14L1	SEC14 like lipid binding 1	62,8	72,3	**1,31**	0,028076
ZBTB6	zinc finger and BTB domain containing 6	16,2	18,8	**1,31**	0,025368
PSMB1	proteasome subunit beta 1	148,9	159,7	**1,31**	0,029367
FOXO4	forkhead box O4	10,5	12,1	**1,31**	0,028431
PXN	paxillin	66,1	74,0	**1,31**	0,025262
PANK3	pantothenate kinase 3	40,5	48,6	**1,31**	0,028721
ZNF800	zinc finger protein 800	15,8	18,4	**1,31**	0,028348
UBXN11	UBX domain protein 11	15,6	17,1	**1,31**	0,023228
ETNK1	ethanolamine kinase 1	32,0	37,1	**1,31**	0,010248
TFE3	transcription factor binding to IGHM enhancer 3	74,3	83,4	**1,31**	0,014568
RANBP10	RAN binding protein 10	20,8	23,9	**1,31**	0,019771
FAM91A1	family with sequence similarity 91 member A1	29,0	34,4	**1,31**	0,025357
DERL2	derlin 2	46,6	51,9	**1,31**	0,014037
HAUS3	HAUS augmin like complex subunit 3	10,6	11,9	**1,31**	0,038598
ALKBH3	alkB homolog 3, alpha-ketoglutaratedependent dioxygenase	15,2	16,4	**1,31**	0,016029
ZNF44	zinc finger protein 44	9,7	10,6	**1,31**	0,031572
WDR37	WD repeat domain 37	14,2	16,7	**1,31**	0,027932

MRPS6	mitochondrial ribosomal protein S6	115,8	123,4	**1,31**	0,049931
QTRT2	queuine tRNA-ribosyltransferase accessory subunit 2	15,3	17,3	**1,31**	0,044045
SMIM20	small integral membrane protein 20	15,1	15,7	**1,31**	0,023593
ZNF740	zinc finger protein 740	24,8	28,2	**1,31**	0,012015
PRPF38A	pre-mRNA processing factor 38A	36,1	39,8	**1,31**	0,010395
SUPT6H	SPT6 homolog, histone chaperone	108,6	123,5	**1,31**	0,042019
ARMC10	armadillo repeat containing 10	7,2	7,8	**1,31**	0,048329
PSMA4	proteasome subunit alpha 4	76,4	84,4	**1,31**	0,014491
CNOT9	CCR4-NOT transcription complex subunit 9	42,6	46,6	**1,31**	0,009332
ELMOD3	ELMO domain containing 3	10,6	11,7	**1,31**	0,022189
TERF2	telomeric repeat binding factor 2	26,4	29,7	**1,30**	0,016906
PRUNE1	prune exopolyphosphatase 1	19,2	21,3	**1,30**	0,018244
COA6	cytochrome c oxidase assembly factor 6	8,9	9,6	**1,30**	0,037794
TMEM164	transmembrane protein 164	12,0	14,1	**1,30**	0,03493
HTATIP2	HIV-1 Tat interactive protein 2	19,9	22,6	**1,30**	0,022653
ELAC1	elaC ribonuclease Z 1	6,9	7,4	**1,30**	0,045499
NATD1	N-acetyltransferase domain containing 1	12,0	13,8	**1,30**	0,048271
IP6K1	inositol hexakisphosphate kinase 1	56,4	66,1	**1,30**	0,015603
NIF3L1	NGG1 interacting factor 3 like 1	15,5	17,8	**1,30**	0,028005
FIZ1	FLT3 interacting zinc finger 1	15,8	17,4	**1,30**	0,015101
RHBDD1	rhomboid domain containing 1	27,6	30,8	**1,30**	0,010869
FASTKD5	FAST kinase domains 5	13,1	15,0	**1,30**	0,027328
MECP2	methyl-CpG binding protein 2	63,3	73,0	**1,30**	0,027577
CDK5RAP1	CDK5 regulatory subunit associated protein 1	11,4	12,3	**1,30**	0,029899
BSDC1	BSD domain containing 1	68,2	78,0	**1,30**	0,020141
PNP	purine nucleoside phosphorylase	81,2	87,2	**1,30**	0,0494
CCDC107	coiled-coil domain containing 107	23,6	25,7	**1,30**	0,013137
TYK2	tyrosine kinase 2	42,5	47,7	**1,30**	0,01346
ZBTB25	zinc finger and BTB domain containing 25	14,0	16,4	**1,30**	0,048271
PXDC1	PX domain containing 1	54,4	60,4	**1,30**	0,012837
ABHD3	abhydrolase domain containing 3	11,1	11,4	**1,30**	0,044286
CCNT1	cyclin T1	30,8	36,3	**1,30**	0,042158
BRAP	BRCA1 associated protein	21,0	23,8	**1,30**	0,017599

NUDT9	nudix hydrolase 9	30,1	32,9	**1,30**	0,008573
SRP54	signal recognition particle 54	78,5	89,4	**1,30**	0,037079
CRIPT	CXXC repeat containing interactor of PDZ3 domain	19,8	22,5	**1,30**	0,018656
SEC16A	SEC16 homolog A, endoplasmic reticulum export factor	104,1	118,9	**1,30**	0,048021
PC	pyruvate carboxylase	92,9	106,7	**1,30**	0,041511
LLPH	LLP homolog, long-term synaptic facilitation	15,0	16,6	**1,30**	0,018122
ZNF830	zinc finger protein 830	14,3	15,4	**1,30**	0,017062
ARID4B	AT-rich interaction domain 4B	45,3	52,5	**1,30**	0,022126
CTR9	CTR9 homolog, Paf1/RNA polymerase II complex component	58,1	65,0	**1,30**	0,018356
ZSCAN32	zinc finger and SCAN domain containing 32	9,0	10,4	**1,30**	0,045617
PLEKHB2	pleckstrin homology domain containing B2	92,0	103,2	**1,30**	0,033427
CRTC2	CREB regulated transcription coactivator 2	21,6	24,4	**1,30**	0,01613
C2orf49	chromosome 2 open reading frame 49	23,2	26,2	**1,30**	0,013407
FASTKD2	FAST kinase domains 2	25,8	27,5	**1,30**	0,015797
MDM4	MDM4, p53 regulator	21,5	24,5	**1,30**	0,015114
TMEM68	transmembrane protein 68	11,8	12,8	**1,30**	0,027619
DET1	de-etiolated homolog 1 (Arabidopsis)	9,3	10,5	**1,30**	0,041173
TSN	translin	99,0	104,3	**1,30**	0,021236
SNN	stannin	18,6	21,0	**1,30**	0,031393
GIGYF1	GRB10 interacting GYF protein 1	49,7	57,9	**1,30**	0,01872
RHBDD3	rhomboid domain containing 3	15,2	16,7	**1,30**	0,01975
FKRP	fukutin related protein	19,7	21,8	**1,30**	0,018915
RNF114	ring finger protein 114	52,3	59,0	**1,29**	0,01245
LEMD3	LEM domain containing 3	38,9	45,0	**1,29**	0,021754
DNAJB2	DnaJ heat shock protein family (Hsp40) member B2	94,3	102,6	**1,29**	0,02101
PDE12	phosphodiesterase 12	22,5	24,6	**1,29**	0,021592
C11orf71	chromosome 11 open reading frame 71	9,5	10,5	**1,29**	0,038532
WDR47	WD repeat domain 47	21,8	25,4	**1,29**	0,046555
TNRC6C	trinucleotide repeat containing 6C	26,7	30,1	**1,29**	0,032544
SPOUT1	SPOUT domain containing methyltransferase 1	27,1	28,7	**1,29**	0,011346
SH3KBP1	SH3 domain containing kinase binding protein 1	40,9	43,0	**1,29**	0,020543
GMEB2	glucocorticoid modulatory element binding protein 2	22,0	25,0	**1,29**	0,031536
NRF1	nuclear respiratory factor 1	11,6	12,9	**1,29**	0,042748

ZC3H10	zinc finger CCCH-type containing 10	10,4	12,4	**1,29**	0,043608
DYRK1A	dual specificity tyrosine phosphorylation regulated kinase 1A	44,1	50,0	**1,29**	0,026231
RPIA	ribose 5-phosphate isomerase A	9,0	9,4	**1,29**	0,039139
MAPK8IP3	mitogen-activated protein kinase 8 interacting protein 3	45,6	52,4	**1,29**	0,013049
CDAN1	codanin 1	14,1	15,0	**1,29**	0,039334
EIF1B	eukaryotic translation initiation factor 1B	34,4	38,1	**1,29**	0,010501
ILF3-AS1	ILF3 antisense RNA 1 (head to head)	184,1	132,0	**1,29**	0,01613
C6orf203	chromosome 6 open reading frame 203	11,4	12,1	**1,29**	0,028573
TRIM13	tripartite motif containing 13	22,9	26,3	**1,29**	0,019133
PRR13	proline rich 13	28,9	31,3	**1,29**	0,008652
YAE1D1	Yae1 domain containing 1			**1,29**	0,040561
TMEM47	transmembrane protein 47	96,5	107,0	**1,29**	0,037362
FDX1	ferredoxin 1	10,9	11,4	**1,29**	0,034864
DCBLD1	discoidin, CUB and LCCL domain containing 1	15,3	17,6	**1,29**	0,037042
ZNF282	zinc finger protein 282	26,9	29,3	**1,29**	0,012685
CUL3	cullin 3	58,9	65,1	**1,29**	0,019269
WIPI2	WD repeat domain, phosphoinositide interacting 2	56,4	65,8	**1,29**	0,018655
AKAP8L	A-kinase anchoring protein 8 like	31,5	35,4	**1,29**	0,015268
ZNF75A	zinc finger protein 75a	9,2	10,1	**1,29**	0,041469
SBF1	SET binding factor 1	103,0	114,5	**1,29**	0,03742
STK11IP	serine/threonine kinase 11 interacting protein	11,8	12,6	**1,29**	0,036849
MRPS30	mitochondrial ribosomal protein S30	22,9	24,9	**1,29**	0,042855
CSTB	cystatin B	125,4	140,6	**1,28**	0,037624
NSFL1C	NSFL1 cofactor	78,3	86,4	**1,28**	0,023163
PEX2	peroxisomal biogenesis factor 2	23,3	24,3	**1,28**	0,015252
IRF2BP2	interferon regulatory factor 2 binding protein 2	72,4	76,7	**1,28**	0,016704
CFAP36	cilia and flagella associated protein 36	35,2	38,6	**1,28**	0,018486
BRD8	bromodomain containing 8	42,9	46,5	**1,28**	0,016988
DPF2	double PHD fingers 2	37,7	42,2	**1,28**	0,015077
BAG1	BCL2 associated athanogene 1	51,3	58,9	**1,28**	0,020402
ARMC5	armadillo repeat containing 5	30,6	32,2	**1,28**	0,014821
TBCA	tubulin folding cofactor A	43,5	46,5	**1,28**	0,01167
ARHGEF10L	Rho guanine nucleotide exchange factor 10 like	25,3	28,4	**1,28**	0,01719

C12orf49	chromosome 12 open reading frame 49	37,7	41,4	**1,28**	0,019675
ZDHHC5	zinc finger DHHC-type containing 5	88,5	97,2	**1,28**	0,040887
NR1H2	nuclear receptor subfamily 1 group H member 2	85,7	95,9	**1,28**	0,03707
PPP1R16A	protein phosphatase 1 regulatory subunit 16A	23,5	26,1	**1,28**	0,022092
MIGA2	mitoguardin 2	17,8	19,3	**1,28**	0,024663
PDXK	pyridoxal kinase	85,8	90,9	**1,28**	0,032261
UBE2E1	ubiquitin conjugating enzyme E2 E1	59,7	68,3	**1,28**	0,029195
SECISBP2	SECIS binding protein 2	29,8	34,1	**1,28**	0,021736
TMEM33	transmembrane protein 33	46,2	51,0	**1,28**	0,014675
VMP1	vacuole membrane protein 1	89,4	98,5	**1,28**	0,026152
RETREG3	reticulophagy regulator family member 3	41,0	46,8	**1,28**	0,018173
NCBP2-AS2	NCBP2 antisense RNA 2 (head to head)	10,0	10,3	**1,28**	0,037938
GPN2	GPN-loop GTPase 2	20,2	21,5	**1,28**	0,016014
FAM104A	family with sequence similarity 104 member A	30,9	33,0	**1,28**	0,012873
TARS2	threonyl-tRNA synthetase 2, mitochondrial (putative)	17,4	18,0	**1,28**	0,028217
RNF139	ring finger protein 139	35,0	38,0	**1,28**	0,011104
RAP2B	RAP2B, member of RAS oncogene family	14,6	14,7	**1,28**	0,043505
SNRPB2	small nuclear ribonucleoprotein polypeptide B2	43,4	46,0	**1,28**	0,013594
TARS	threonyl-tRNA synthetase	118,6	131,5	**1,28**	0,046415
C16orf70	chromosome 16 open reading frame 70	12,3	12,7	**1,28**	0,036477
MTPAP	mitochondrial poly(A) polymerase	14,9	16,3	**1,27**	0,047828
ATP6V1G1	ATPase H+ transporting V1 subunit G1	82,5	88,0	**1,27**	0,024397
CSK	CSK, non-receptor tyrosine kinase	55,1	58,3	**1,27**	0,028066
OTUB1	OTU deubiquitinase, ubiquitin aldehyde binding 1	28,5	31,8	**1,27**	0,016222
ZNF627	zinc finger protein 627	11,7	12,6	**1,27**	0,038123
C6orf120	chromosome 6 open reading frame 120	35,3	38,7	**1,27**	0,020582
SLU7	SLU7 homolog, splicing factor	56,7	62,7	**1,27**	0,024547
TERF1	telomeric repeat binding factor 1	20,6	22,6	**1,27**	0,032676
CHMP2B	charged multivesicular body protein 2B	43,8	48,2	**1,27**	0,022857
DHX8	DEAH-box helicase 8	41,4	45,4	**1,27**	0,040879
NME7	NME/NM23 family member 7	19,1	20,0	**1,27**	0,029651
BAX	BCL2 associated X, apoptosis regulator	31,5	32,6	**1,27**	0,016577
DNAJC25	DnaJ heat shock protein family (Hsp40) member C25	21,8	24,6	**1,27**	0,03116

AP5S1	adaptor related protein complex 5 sigma 1 subunit	15,7	16,6	**1,27**	0,027264
ACOT9	acyl-CoA thioesterase 9	41,0	45,0	**1,27**	0,028255
ZNF263	zinc finger protein 263	23,3	25,2	**1,27**	0,020381
FBXW5	F-box and WD repeat domain containing 5	81,2	89,0	**1,27**	0,039227
FAM222B	family with sequence similarity 222 member B	17,7	19,8	**1,27**	0,032131
TFB2M	transcription factor B2, mitochondrial	10,7	10,7	**1,27**	0,043462
WASF3	WAS protein family member 3	15,4	16,9	**1,27**	0,046614
VPS37B	VPS37B, ESCRT-I subunit	73,6	76,4	**1,27**	0,037344
NECAP2	NECAP endocytosis associated 2	53,8	57,1	**1,27**	0,023937
AHCTF1	AT-hook containing transcription factor 1	34,0	37,7	**1,27**	0,048271
ZDHHC17	zinc finger DHHC-type containing 17	15,0	16,8	**1,27**	0,047943
CNIH1	cornichon family AMPA receptor auxiliary protein 1	116,7	123,8	**1,27**	0,03873
PRDM4	PR/SET domain 4	36,2	41,1	**1,27**	0,02598
TRMT6	tRNA methyltransferase 6	23,2	23,9	**1,27**	0,029191
DHPS	deoxyhypusine synthase	27,8	29,8	**1,27**	0,02395
XAB2	XPA binding protein 2	40,6	44,1	**1,27**	0,024102
BORCS7	BLOC-1 related complex subunit 7	14,9	16,2	**1,27**	0,03454
SFT2D1	SFT2 domain containing 1	31,6	34,1	**1,27**	0,020472
ZNF655	zinc finger protein 655	35,2	40,2	**1,27**	0,035317
KAT8	lysine acetyltransferase 8	20,6	23,2	**1,27**	0,029028
RNPS1	RNA binding protein with serine rich domain 1	63,3	68,3	**1,27**	0,025278
PGRMC1	progesterone receptor membrane component 1	95,0	106,0	**1,27**	0,042011
UBL7	ubiquitin like 7	26,9	28,0	**1,27**	0,017242
ABHD13	abhydrolase domain containing 13	16,2	18,0	**1,27**	0,034528
RSPRY1	ring finger and SPRY domain containing 1	54,1	59,9	**1,27**	0,029732
UBE2N	ubiquitin conjugating enzyme E2 N	62,3	64,6	**1,27**	0,028255
NBDY	negative regulator of P-body association	34,3	36,7	**1,27**	0,019403
ARHGEF11	Rho guanine nucleotide exchange factor 11	40,3	44,6	**1,27**	0,041058
NCKIPSD	NCK interacting protein with SH3 domain	22,0	23,6	**1,27**	0,028255
LZTFL1	leucine zipper transcription factor like 1	26,9	30,5	**1,27**	0,035979
RAB11B	RAB11B, member RAS oncogene family	77,5	84,3	**1,27**	0,042158
INIP	INTS3 and NABP interacting protein	28,1	30,2	**1,27**	0,025139
WDR33	WD repeat domain 33	49,3	54,1	**1,27**	0,033921

TMEM184C	transmembrane protein 184C	31,8	35,1	**1,27**	0,024874
SUGP1	SURP and G-patch domain containing 1		21,1	**1,27**	0,035295
TRAPPC3	trafficking protein particle complex 3	52,2	54,4	**1,26**	0,018189
MAK16	MAK16 homolog	15,5	15,9	**1,26**	0,038113
BRD1	bromodomain containing 1	25,6	28,1	**1,26**	0,027939
TAF11	TATA-box binding protein associated factor 11	11,3	12,0	**1,26**	0,045766
TOMM70	translocase of outer mitochondrial membrane 70	80,3	85,1	**1,26**	0,04383
AFTPH	aftiphilin	48,8	54,1	**1,26**	0,034871
MAU2	MAU2 sister chromatid cohesion factor	49,7	54,6	**1,26**	0,03598
TMEM185B	transmembrane protein 185B	27,1	29,6	**1,26**	0,033596
GNPNAT1	glucosamine-phosphate N-acetyltransferase 1	49,4	54,5	**1,26**	0,03581
TMUB2	transmembrane and ubiquitin like domain containing 2	26,9	29,0	**1,26**	0,026029
LRRC58	leucine rich repeat containing 58	53,2	58,6	**1,26**	0,045847
TOMM34	translocase of outer mitochondrial membrane 34	43,6	41,8	**1,26**	0,037324
ATP6V0E1	ATPase H+ transporting V0 subunit e1	102,0	106,7	**1,26**	0,04707
AAMP	angio associated migratory cell protein	71,8	77,0	**1,26**	0,037927
AP4B1	adaptor related protein complex 4 beta 1 subunit	15,9	16,6	**1,26**	0,043687
RAB22A	RAB22A, member RAS oncogene family	43,5	47,7	**1,26**	0,027647
ORC4	origin recognition complex subunit 4	20,2	22,3	**1,26**	0,043859
ZNF397	zinc finger protein 397	28,7	31,5	**1,26**	0,042505
PNKD	paroxysmal nonkinesigenic dyskinesia	18,7	18,8	**1,26**	0,029161
RNF215	ring finger protein 215	23,2	24,1	**1,26**	0,035011
LMBR1L	limb development membrane protein 1 like	11,8	13,2	**1,26**	0,041814
CCDC59	coiled-coil domain containing 59	20,9	21,8	**1,26**	0,037025
GABPB2	GA binding protein transcription factor beta subunit 2	20,3	21,9	**1,26**	0,038322
NOLC1	nucleolar and coiled-body phosphoprotein 1	61,2	64,6	**1,26**	0,03598
BTF3L4	basic transcription factor 3 like 4	38,4	41,0	**1,26**	0,021853
RBCK1	RANBP2-type and C3HC4-type zinc finger containing 1	52,9	58,6	**1,26**	0,041493
SLC35F6	solute carrier family 35 member F6	38,5	40,8	**1,26**	0,024331
BUD31	BUD31 homolog	52,5	53,0	**1,26**	0,03329
MLXIP	MLX interacting protein	18,2	20,0	**1,26**	0,04293
PAFAH2	platelet activating factor acetylhydrolase 2	14,9	15,3	**1,26**	0,039666
DHX34	DExH-box helicase 34	18,9	20,7	**1,26**	0,046599

ZNF7	zinc finger protein 7	13,6	14,1	**1,26**	0,047381
UTP14C	UTP14, small subunit processome component homolog C (S. cerevisia	38,8	42,0	**1,26**	0,034629
CCDC71	coiled-coil domain containing 71	17,5	18,1	**1,26**	0,035107
USF1	upstream transcription factor 1	19,9	20,9	**1,26**	0,031572
LUC7L	LUC7 like	26,0	29,2	**1,26**	0,031458
CNST	consortin, connexin sorting protein	21,6	24,0	**1,26**	0,046555
NCBP2	nuclear cap binding protein subunit 2	49,3	52,4	**1,26**	0,025461
RRP1	ribosomal RNA processing 1	19,2	20,1	**1,26**	0,038046
CDC34	cell division cycle 34	27,3	29,3	**1,26**	0,027856
HAX1	HCLS1 associated protein X-1	81,7	83,7	**1,25**	0,041648
SWI5	SWI5 homologous recombination repair protein	27,4	27,6	**1,25**	0,043618
WDR74	WD repeat domain 74	19,4	20,0	**1,25**	0,037105
MSL1	male specific lethal 1 homolog	85,2	91,5	**1,25**	0,049162
ZNF384	zinc finger protein 384	41,9	45,6	**1,25**	0,032489
ZFYVE1	zinc finger FYVE-type containing 1	21,4	23,6	**1,25**	0,046191
SLC4A1AP	solute carrier family 4 member 1 adaptor protein	27,1	28,2	**1,25**	0,032566
HMGXB3	HMG-box containing 3	34,2	36,9	**1,25**	0,031992
AGFG2	ArfGAP with FG repeats 2	38,1	40,8	**1,25**	0,047378
YTHDF2	YTH N6-methyladenosine RNA binding protein 2	47,7	50,9	**1,25**	0,030985
SLC35A4	solute carrier family 35 member A4	70,9	75,3	**1,25**	0,045942
TIMM21	translocase of inner mitochondrial membrane 21	21,3	20,9	**1,25**	0,040313
SP2	Sp2 transcription factor	18,6	20,3	**1,25**	0,041405
POLR2F	RNA polymerase II subunit F	32,6	34,2	**1,25**	0,035371
GPN3	GPN-loop GTPase 3	19,3	19,7	**1,25**	0,032307
ZCCHC3	zinc finger CCHC-type containing 3	23,2	23,5	**1,25**	0,025849
USP20	ubiquitin specific peptidase 20	20,5	22,2	**1,25**	0,033193
NUDT16L1	nudix hydrolase 16 like 1	20,4	20,5	**1,25**	0,04488
AFG3L2	AFG3 like matrix AAA peptidase subunit 2	41,5	42,5	**1,25**	0,026643
MESDC2	mesoderm development candidate 2			**1,25**	0,048425
TSSC4	tumor suppressing subtransferable candidate 4	27,9	28,8	**1,25**	0,031202
TANK	TRAF family member associated NFKB activator	41,2	46,9	**1,25**	0,039579
NEPRO	nucleolus and neural progenitor protein	25,7	27,4	**1,25**	0,030043
UBE2E2	ubiquitin conjugating enzyme E2 E2	17,5	18,7	**1,25**	0,047943

RPRD1B	regulation of nuclear pre-mRNA domain containing 1B	41,3	43,1	**1,25**	0,03267
VEZF1	vascular endothelial zinc finger 1	46,2	50,3	**1,25**	0,045463
PDE6D	phosphodiesterase 6D	15,2	16,7	**1,25**	0,049379
EMC8	ER membrane protein complex subunit 8	17,5	17,9	**1,25**	0,048363
MTO1	mitochondrial tRNA translation optimization 1	25,1	24,8	**1,25**	0,031173
COQ9	coenzyme Q9	23,3	23,7	**1,25**	0,033464
SPTY2D1	SPT2 chromatin protein domain containing 1	51,3	55,5	**1,25**	0,042945
HARS2	histidyl-tRNA synthetase 2, mitochondrial	24,7	25,6	**1,25**	0,028145
ANAPC16	anaphase promoting complex subunit 16	69,0	72,6	**1,25**	0,045096
STK4	serine/threonine kinase 4	25,2	25,9	**1,25**	0,042825
TLE3	transducin like enhancer of split 3	31,9	32,9	**1,25**	0,041405
MRPL27	mitochondrial ribosomal protein L27	24,7	25,5	**1,25**	0,043469
UNC50	unc-50 inner nuclear membrane RNA binding protein	33,3	34,4	**1,25**	0,022702
MED28	mediator complex subunit 28	27,1	28,5	**1,25**	0,033845
PIGQ	phosphatidylinositol glycan anchor biosynthesis class Q	23,4	23,4	**1,24**	0,036307
ARFGAP2	ADP ribosylation factor GTPase activating protein 2	43,0	44,4	**1,24**	0,027967
ASPSCR1	ASPSCR1, UBX domain containing tether for SLC2A4	20,4	20,4	**1,24**	0,037798
GTF3C5	general transcription factor IIIC subunit 5	33,1	34,3	**1,24**	0,038263
PPP4R2	protein phosphatase 4 regulatory subunit 2	47,5	51,9	**1,24**	0,04094
CXXC1	CXXC finger protein 1	34,9	36,5	**1,24**	0,034997
LSM14B	LSM family member 14B	31,0	32,4	**1,24**	0,027111
MRPS2	mitochondrial ribosomal protein S2	22,8	23,7	**1,24**	0,045564
ARL6IP6	ADP ribosylation factor like GTPase 6 interacting protein 6	26,3	26,8	**1,24**	0,049346
ARFIP1	ADP ribosylation factor interacting protein 1	36,0	37,6	**1,24**	0,026643
RNF185	ring finger protein 185	36,4	39,3	**1,24**	0,038063
TRAPPC6B	trafficking protein particle complex 6B	30,3	31,5	**1,24**	0,033219
SMAD4	SMAD family member 4	43,1	46,7	**1,24**	0,032471
BROX	BRO1 domain and CAAX motif containing	46,6	50,1	**1,24**	0,039934
KMT5B	lysine methyltransferase 5B	36,9	40,5	**1,24**	0,046789
CCNL2	cyclin L2	66,0	70,6	**1,24**	0,044882
LYRM2	LYR motif containing 2	30,6	31,2	**1,24**	0,029994
MARK2	microtubule affinity regulating kinase 2	28,0	30,2	**1,24**	0,045034
LIN7C	lin-7 homolog C, crumbs cell polarity complex component	29,2	30,1	**1,24**	0,039579

GTPBP4	GTP binding protein 4	57,7	58,3	**1,23**	0,049048
SAT2	spermidine/spermine N1-acetyltransferase family member 2	24,0	24,2	**1,23**	0,034881
RNF41	ring finger protein 41	30,1	32,4	**1,23**	0,032833
FGFR1OP2	FGFR1 oncogene partner 2	32,1	33,9	**1,23**	0,033683
VSTM4	V-set and transmembrane domain containing 4	33,1	34,0	**1,23**	0,049788
METTL16	methyltransferase like 16	35,9	37,5	**1,23**	0,048217
TIMM44	translocase of inner mitochondrial membrane 44	30,6	32,3	**1,23**	0,037037
UBE2A	ubiquitin conjugating enzyme E2 A	61,2	61,4	**1,23**	0,040291
FKBP15	FK506 binding protein 15	44,2	47,0	**1,23**	0,04948
CMTR1	cap methyltransferase 1	38,8	39,4	**1,23**	0,041101
SMIM7	small integral membrane protein 7	39,7	39,5	**1,23**	0,033123
VPS26A	VPS26, retromer complex component A	62,6	65,4	**1,23**	0,044861
ZNF672	zinc finger protein 672	26,9	28,1	**1,22**	0,047586
ALG5	ALG5, dolichyl-phosphate beta-glucosyltransferase	25,3	25,0	**1,22**	0,046062
GOLGA7	golgin A7	43,6	45,7	**1,22**	0,049085
IRF2BP1	interferon regulatory factor 2 binding protein 1	36,2	36,1	**1,22**	0,043977
ZSCAN18	zinc finger and SCAN domain containing 18	36,4	36,2	**1,22**	0,046706
BCAS2	BCAS2, pre-mRNA processing factor	38,3	38,0	**1,21**	0,049243
UBQLN2	ubiquilin 2	56,5	37,3	**0,82**	0,04799
PRKD3	protein kinase D3	43,5	28,0	**0,82**	0,044875
GNPAT	glyceronephosphate O-acyltransferase	40,5	25,4	**0,82**	0,047955
MRPS27	mitochondrial ribosomal protein S27	47,7	29,7	**0,82**	0,043165
RARS2	arginyl-tRNA synthetase 2, mitochondrial	31,6	19,8	**0,82**	0,045564
IDE	insulin degrading enzyme	41,9	27,6	**0,82**	0,044374
PHF2	PHD finger protein 2	47,7	30,9	**0,82**	0,047827
PHF10	PHD finger protein 10	49,3	31,8	**0,82**	0,038893
TPP2	tripeptidyl peptidase 2	39,5	24,7	**0,82**	0,049617
ANKRD40	ankyrin repeat domain 40	51,4	33,9	**0,82**	0,032565
AZI2	5-azacytidine induced 2	50,5	33,0	**0,81**	0,035545
RHOT1	ras homolog family member T1	27,8	18,2	**0,81**	0,049934
PIAS3	protein inhibitor of activated STAT 3	40,7	25,5	**0,81**	0,035979
SNAPC3	small nuclear RNA activating complex polypeptide 3	33,0	20,9	**0,81**	0,042966
COG4	component of oligomeric golgi complex 4	60,1	37,3	**0,81**	0,034034

PTPN9	protein tyrosine phosphatase, non-receptor type 9	46,7	29,4	**0,81**	0,034104
NUP133	nucleoporin 133	32,7	20,4	**0,81**	0,035751
PPM1F	protein phosphatase, Mg2+/Mn2+ dependent 1F	47,4	28,6	**0,81**	0,033445
AHDC1	AT-hook DNA binding motif containing 1	43,3	27,1	**0,81**	0,034733
USP6NL	USP6 N-terminal like	55,8	35,2	**0,81**	0,045327
VPS35	VPS35, retromer complex component	114,6	72,3	**0,81**	0,049093
CYP4V2	cytochrome P450 family 4 subfamily V member 2	37,7	23,4	**0,81**	0,034482
ITSN1	intersectin 1	39,0	25,2	**0,81**	0,039082
POLDIP2	DNA polymerase delta interacting protein 2	81,8	50,1	**0,80**	0,0424
BIN1	bridging integrator 1	31,3	19,3	**0,80**	0,047237
NT5C2	5'-nucleotidase, cytosolic II	72,4	45,1	**0,80**	0,029296
IMMT	inner membrane mitochondrial protein	69,7	42,2	**0,80**	0,030005
CAMSAP1	calmodulin regulated spectrin associated protein 1	33,6	21,4	**0,80**	0,025656
ZFAND1	zinc finger AN1-type containing 1	26,4	15,7	**0,80**	0,042926
RCC1	regulator of chromosome condensation 1	38,1	22,9	**0,80**	0,032462
DNAJC7	DnaJ heat shock protein family (Hsp40) member C7	68,9	41,8	**0,80**	0,027539
JAK2	Janus kinase 2	70,3	48,3	**0,80**	0,047549
SENP6	SUMO1/sentrin specific peptidase 6	73,7	46,7	**0,80**	0,033482
OXR1	oxidation resistance 1	38,6	22,8	**0,80**	0,032171
TRAFD1	TRAF-type zinc finger domain containing 1	50,8	32,5	**0,80**	0,038309
DERA	deoxyribose-phosphate aldolase	23,1	14,0	**0,80**	0,044984
GTF2IRD1	GTF2I repeat domain containing 1	33,8	19,9	**0,80**	0,045353
NCBP1	nuclear cap binding protein subunit 1	45,3	26,4	**0,80**	0,025607
ZCRB1	zinc finger CCHC-type and RNA binding motif containing 1	52,4	31,5	**0,80**	0,048444
ICE2	interactor of little elongation complex ELL subunit 2	28,4	17,3	**0,80**	0,030059
PREP	prolyl endopeptidase	30,8	19,3	**0,80**	0,032565
SNX21	sorting nexin family member 21	28,6	17,9	**0,80**	0,043994
PTPRG	protein tyrosine phosphatase, receptor type G	55,1	37,6	**0,80**	0,043469
ANKMY2	ankyrin repeat and MYND domain containing 2	24,4	15,2	**0,80**	0,044769
POMGNT1	protein O-linked mannose N-acetylglucosaminyltransferase 1 (beta 1,	83,2	51,4	**0,80**	0,037007
PCMT1	protein-L-isoaspartate (D-aspartate) O-methyltransferase	101,9	58,8	**0,80**	0,046685
ENDOD1	endonuclease domain containing 1	70,7	44,0	**0,80**	0,026874
RPE	ribulose-5-phosphate-3-epimerase	35,5	21,5	**0,80**	0,025933

TSR2	TSR2, ribosome maturation factor	42,1	24,0	**0,80**	0,035935
NDUFA5	NADH:ubiquinone oxidoreductase subunit A5	45,6	27,3	**0,80**	0,031028
SCFD1	sec1 family domain containing 1	74,6	45,2	**0,80**	0,034733
GPR180	G protein-coupled receptor 180	30,0	18,7	**0,80**	0,026422
RAB11A	RAB11A, member RAS oncogene family	83,4	49,5	**0,79**	0,035281
CEP250	centrosomal protein 250	29,2	17,1	**0,79**	0,028511
MYO9A	myosin IXA	63,3	39,7	**0,79**	0,022379
USP39	ubiquitin specific peptidase 39	36,9	22,0	**0,79**	0,021405
BRD7	bromodomain containing 7	50,5	30,2	**0,79**	0,02388
UBP1	upstream binding protein 1 (LBP-1a)	74,1	45,2	**0,79**	0,025327
NIT2	nitrilase family member 2	32,0	19,5	**0,79**	0,028807
STAG1	stromal antigen 1	83,6	51,1	**0,79**	0,024163
ARSJ	arylsulfatase family member J	112,3	68,1	**0,79**	0,04181
TBC1D9B	TBC1 domain family member 9B	38,5	23,4	**0,79**	0,024122
LIX1L	limb and CNS expressed 1 like	40,5	24,0	**0,79**	0,022806
GLRX3	glutaredoxin 3	45,6	26,8	**0,79**	0,028183
SLC25A12	solute carrier family 25 member 12	14,4	9,1	**0,79**	0,048193
YEATS2	YEATS domain containing 2	42,1	25,9	**0,79**	0,02357
ST6GAL1	ST6 beta-galactoside alpha-2,6-sialyltransferase 1	83,0	52,5	**0,79**	0,027317
DBR1	debranching RNA lariats 1	17,5	10,7	**0,79**	0,044999
CSNK1G2	casein kinase 1 gamma 2	54,6	33,5	**0,79**	0,035148
FTO	FTO, alpha-ketoglutarate dependent dioxygenase	48,5	29,1	**0,79**	0,019455
TKFC	triokinase and FMN cyclase	33,3	20,2	**0,79**	0,031686
GPS1	G protein pathway suppressor 1	56,9	33,7	**0,79**	0,026019
TUBGCP3	tubulin gamma complex associated protein 3	17,9	10,7	**0,79**	0,041247
NUDT3	nudix hydrolase 3	55,1	33,7	**0,79**	0,02562
THOC7	THO complex 7	42,3	25,1	**0,79**	0,02711
MYO5A	myosin VA	57,9	36,9	**0,79**	0,02574
PRMT2	protein arginine methyltransferase 2	107,8	63,4	**0,79**	0,049496
CRYZL1	crystallin zeta like 1	17,9	11,1	**0,79**	0,041696
TSC2	tuberous sclerosis 2	64,1	39,5	**0,79**	0,020425
PTPN21	protein tyrosine phosphatase, non-receptor type 21		26,4	**0,79**	0,029785
TMEM126B	transmembrane protein 126B	24,1	14,1	**0,79**	0,042237

UROS	uroporphyrinogen III synthase	15,6	9,1	**0,79**	0,049442
UBAP2	ubiquitin associated protein 2	28,0	16,9	**0,79**	0,0289
MBIP	MAP3K12 binding inhibitory protein 1	18,4	11,3	**0,79**	0,034431
SYAP1	synapse associated protein 1	55,1	34,1	**0,79**	0,024408
MTR	5-methyltetrahydrofolate-homocysteine methyltransferase	50,2	31,4	**0,79**	0,031764
TMEM106B	transmembrane protein 106B	72,5	44,4	**0,79**	0,03116
UBXN2A	UBX domain protein 2A	22,4	13,5	**0,79**	0,031846
PEX13	peroxisomal biogenesis factor 13	53,7	32,0	**0,79**	0,020586
KARS	lysyl-tRNA synthetase	101,6	61,2	**0,79**	0,027471
MFSD14C	major facilitator superfamily domain containing 14C	13,8	8,3	**0,79**	0,04592
NPRL3	NPR3 like, GATOR1 complex subunit	32,5	18,4	**0,79**	0,030059
ZEB1	zinc finger E-box binding homeobox 1	94,8	60,0	**0,79**	0,040019
FAM20B	FAM20B, glycosaminoglycan xylosylkinase	107,8	64,9	**0,79**	0,024471
TOP2B	topoisomerase (DNA) II beta	123,2	76,0	**0,79**	0,038598
ELOVL5	ELOVL fatty acid elongase 5	82,7	47,5	**0,79**	0,03001
EARS2	glutamyl-tRNA synthetase 2, mitochondrial	17,1	9,9	**0,79**	0,047755
TMEM94	transmembrane protein 94	39,2	23,5	**0,79**	0,017516
ARHGAP10	Rho GTPase activating protein 10	25,7	15,9	**0,79**	0,024281
GLOD4	glyoxalase domain containing 4	49,3	28,7	**0,79**	0,015119
ETFA	electron transfer flavoprotein alpha subunit	55,8	31,9	**0,78**	0,02272
NAA50	N(alpha)-acetyltransferase 50, NatE catalytic subunit	119,9	72,0	**0,78**	0,029445
NISCH	nischarin	67,1	39,5	**0,78**	0,012583
CCT6A	chaperonin containing TCP1 subunit 6A	159,8	92,6	**0,78**	0,048833
FBXL7	F-box and leucine rich repeat protein 7	48,2	29,0	**0,78**	0,014616
TCHP	trichoplein keratin filament binding	22,5	13,2	**0,78**	0,026068
MUT	methylmalonyl-CoA mutase		26,1	**0,78**	0,01761
FPGT	fucose-1-phosphate guanylyltransferase	13,7	8,1	**0,78**	0,038698
KCTD10	potassium channel tetramerization domain containing 10	138,9	80,3	**0,78**	0,047584
RUFY2	RUN and FYVE domain containing 2	19,6	12,1	**0,78**	0,032486
GPR157	G protein-coupled receptor 157	12,9	7,7	**0,78**	0,045953
P4HTM	prolyl 4-hydroxylase, transmembrane	43,2	26,0	**0,78**	0,03086
LIMK2	LIM domain kinase 2	14,8	8,7	**0,78**	0,042419
NEK9	NIMA related kinase 9	78,3	47,1	**0,78**	0,019732

LRSAM1	leucine rich repeat and sterile alpha motif containing 1	22,7	14,0	**0,78**	0,022161
PKIG	cAMP-dependent protein kinase inhibitor gamma	32,2	20,0	**0,78**	0,018105
HDAC5	histone deacetylase 5	81,2	48,6	**0,78**	0,017655
MYL6B	myosin light chain 6B	36,2	21,7	**0,78**	0,043099
COPS7B	COP9 signalosome subunit 7B	15,6	9,6	**0,78**	0,029186
MAP4K5	mitogen-activated protein kinase kinase kinase kinase 5	60,0	37,1	**0,78**	0,017938
PLPPR2	phospholipid phosphatase related 2	46,3	28,2	**0,78**	0,046369
MAN2A2	mannosidase alpha class 2A member 2	54,8	33,1	**0,78**	0,012232
MPI	mannose phosphate isomerase	34,7	21,2	**0,78**	0,034031
PER3	period circadian clock 3	60,4	36,8	**0,78**	0,023871
LMBRD1	LMBR1 domain containing 1	90,4	54,6	**0,78**	0,029514
BCKDK	branched chain ketoacid dehydrogenase kinase	39,9	23,8	**0,78**	0,035177
CHD1L	chromodomain helicase DNA binding protein 1 like	50,0	28,7	**0,78**	0,024031
DOCK1	dedicator of cytokinesis 1	74,6	45,0	**0,78**	0,019376
NELFB	negative elongation factor complex member B	78,9	47,3	**0,78**	0,031054
PRPSAP2	phosphoribosyl pyrophosphate synthetase associated protein 2	25,8	15,5	**0,78**	0,033123
TIMM13	translocase of inner mitochondrial membrane 13	24,9	14,2	**0,78**	0,046533
HSD17B10	hydroxysteroid 17-beta dehydrogenase 10	42,9	24,7	**0,78**	0,023534
SPIN1	spindlin 1	86,7	52,0	**0,78**	0,019341
SNX6	sorting nexin 6	59,8	36,3	**0,78**	0,010343
CDC16	cell division cycle 16	52,2	31,3	**0,78**	0,011686
HECTD2	HECT domain E3 ubiquitin protein ligase 2	25,8	15,4	**0,78**	0,023108
DLD	dihydrolipoamide dehydrogenase	57,4	34,5	**0,78**	0,011781
LONP2	lon peptidase 2, peroxisomal	60,8	36,2	**0,78**	0,015473
DTX3	deltex E3 ubiquitin ligase 3	18,6	11,1	**0,78**	0,041327
C19orf12	chromosome 19 open reading frame 12	34,0	20,9	**0,78**	0,012464
MRPL19	mitochondrial ribosomal protein L19	25,0	14,2	**0,78**	0,025508
SRRT	serrate, RNA effector molecule	83,2	49,3	**0,78**	0,018716
TBC1D22A	TBC1 domain family member 22A	35,9	21,1	**0,78**	0,026403
ANKRD27	ankyrin repeat domain 27	40,6	25,6	**0,78**	0,013694
PDCL	phosducin like	27,0	15,9	**0,78**	0,031306
C1orf216	chromosome 1 open reading frame 216	30,9	18,8	**0,78**	0,018576
KRT18	keratin 18	18,9	10,9	**0,78**	0,041785

TYW3	tRNA-yW synthesizing protein 3 homolog	33,8	19,9	**0,78**	0,017719
BTBD1	BTB domain containing 1	60,7	36,3	**0,78**	0,010636
MED13L	mediator complex subunit 13 like	103,1	64,5	**0,78**	0,037362
CORO1B	coronin 1B	65,3	39,9	**0,78**	0,018985
GNA11	G protein subunit alpha 11	115,9	68,6	**0,78**	0,02495
ZNF532	zinc finger protein 532	81,8	52,6	**0,78**	0,030762
PTPDC1	protein tyrosine phosphatase domain containing 1	17,2	10,1	**0,78**	0,035342
NCOR2	nuclear receptor corepressor 2	114,2	68,3	**0,78**	0,020829
BCAP29	B-cell receptor associated protein 29	27,3	15,8	**0,78**	0,022126
KIAA0586	KIAA0586	13,9	8,5	**0,78**	0,049936
PXYLP1	2-phosphoxylose phosphatase 1	100,8	59,6	**0,78**	0,026019
BCL9	B-cell CLL/lymphoma 9	12,4	7,2	**0,78**	0,043123
SNX12	sorting nexin 12	55,8	33,4	**0,78**	0,017211
MIS18BP1	MIS18 binding protein 1	17,7	9,7	**0,78**	0,029593
URI1	URI1, prefoldin like chaperone	71,5	42,2	**0,78**	0,016408
IFT57	intraflagellar transport 57	32,2	18,7	**0,78**	0,018576
KPNA3	karyopherin subunit alpha 3	41,1	25,1	**0,78**	0,008576
ATP9B	ATPase phospholipid transporting 9B (putative)	22,4	13,1	**0,78**	0,020567
PSME3	proteasome activator subunit 3	96,6	56,9	**0,78**	0,026343
RBX1	ring-box 1	45,6	26,5	**0,77**	0,028556
DNAJC13	DnaJ heat shock protein family (Hsp40) member C13	69,5	42,8	**0,77**	0,027811
FYTTD1	forty-two-three domain containing 1	101,8	60,2	**0,77**	0,021044
ASAH1	N-acylsphingosine amidohydrolase 1	117,1	70,3	**0,77**	0,042888
MMP24-AS1	MMP24 antisense RNA 1	1,6	1,0	**0,77**	0,039199
POLH	DNA polymerase eta	15,0	8,8	**0,77**	0,028556
IQSEC2	IQ motif and Sec7 domain 2	25,1	15,3	**0,77**	0,025935
AGO1	argonaute 1, RISC catalytic component	33,2	19,9	**0,77**	0,019158
COMMD6	COMM domain containing 6	41,3	23,9	**0,77**	0,034779
ZNF362	zinc finger protein 362	20,7	12,7	**0,77**	0,023397
RAI1	retinoic acid induced 1	43,0	26,1	**0,77**	0,008648
PIGX	phosphatidylinositol glycan anchor biosynthesis class X	23,0	12,9	**0,77**	0,041877
SLC4A2	solute carrier family 4 member 2	173,1	103,2	**0,77**	0,039037
PPP4R1	protein phosphatase 4 regulatory subunit 1	56,1	35,1	**0,77**	0,008411

NDUFB3	NADH:ubiquinone oxidoreductase subunit B3	31,0	17,5	**0,77**	0,040887
KCNC4	potassium voltage-gated channel subfamily C member 4	16,1	10,4	**0,77**	0,0365
RPL22	ribosomal protein L22	69,1	39,3	**0,77**	0,029488
MTA2	metastasis associated 1 family member 2	70,7	41,1	**0,77**	0,011824
SSFA2	sperm specific antigen 2			**0,77**	0,031015
PLA2R1	phospholipase A2 receptor 1	31,2	19,3	**0,77**	0,012239
INTS13	integrator complex subunit 13	26,5	15,4	**0,77**	0,023649
COPS4	COP9 signalosome subunit 4	45,9	26,4	**0,77**	0,011676
TMA16	translation machinery associated 16 homolog	18,8	11,2	**0,77**	0,026676
FOCAD	focadhesin	39,2	23,5	**0,77**	0,012026
PIGS	phosphatidylinositol glycan anchor biosynthesis class S	58,3	33,3	**0,77**	0,028255
DNAJC21	DnaJ heat shock protein family (Hsp40) member C21	44,0	26,8	**0,77**	0,008669
TDRD7	tudor domain containing 7	17,3	10,4	**0,77**	0,024033
LYPLA1	lysophospholipase I	31,2	19,0	**0,77**	0,013959
BICRAL	BRD4 interacting chromatin remodelling complex associated protein l	30,7	19,3	**0,77**	0,029998
ITGB3BP	integrin subunit beta 3 binding protein	10,7	6,1	**0,77**	0,049891
FAM160B2	family with sequence similarity 160 member B2	54,1	32,1	**0,77**	0,012021
ACTN1	actinin alpha 1	234,6	135,1	**0,77**	0,04144
NFU1	NFU1 iron-sulfur cluster scaffold	29,2	16,2	**0,77**	0,016921
FITM2	fat storage inducing transmembrane protein 2	30,0	17,3	**0,77**	0,017471
AMOTL1	angiomotin like 1	105,6	61,9	**0,77**	0,022958
KHDRBS1	KH RNA binding domain containing, signal transduction associated 1	189,1	106,3	**0,77**	0,043768
CSE1L	chromosome segregation 1 like	98,1	54,0	**0,77**	0,016042
RHNO1	RAD9-HUS1-RAD1 interacting nuclear orphan 1	16,7	9,3	**0,77**	0,048791
SLC12A4	solute carrier family 12 member 4	103,9	61,2	**0,77**	0,027809
TCF12	transcription factor 12	53,7	33,3	**0,77**	0,016094
SLC25A5	solute carrier family 25 member 5	120,4	68,7	**0,77**	0,038912
ANAPC4	anaphase promoting complex subunit 4	19,0	11,2	**0,77**	0,018297
HMGN2	high mobility group nucleosomal binding domain 2	119,2	64,0	**0,77**	0,024862
LPGAT1	lysophosphatidylglycerol acyltransferase 1	67,7	40,9	**0,77**	0,0064
ELAC2	elaC ribonuclease Z 2	46,0	27,1	**0,77**	0,011832
PKP4	plakophilin 4	31,9	18,4	**0,77**	0,012506
AGO4	argonaute 4, RISC catalytic component	14,1	8,9	**0,77**	0,023593

MN1	MN1 proto-oncogene, transcriptional regulator	38,2	23,8	**0,77**	0,005565
CRADD	CASP2 and RIPK1 domain containing adaptor with death domain	12,2	7,3	**0,77**	0,037575
COPRS	coordinator of PRMT5 and differentiation stimulator	53,1	30,7	**0,77**	0,021199
CENPB	centromere protein B	154,4	88,1	**0,77**	0,040586
ADH5	alcohol dehydrogenase 5 (class III), chi polypeptide	94,1	53,6	**0,77**	0,019868
LRRC40	leucine rich repeat containing 40	16,6	9,9	**0,77**	0,023914
PSEN2	presenilin 2	12,7	7,3	**0,77**	0,043782
ADGRA3	adhesion G protein-coupled receptor A3	36,8	21,9	**0,77**	0,036339
MECR	mitochondrial trans-2-enoyl-CoA reductase	13,0	7,3	**0,77**	0,049555
POFUT2	protein O-fucosyltransferase 2	150,6	86,1	**0,77**	0,037703
RCL1	RNA terminal phosphate cyclase like 1	27,7	18,5	**0,77**	0,014313
7.syys	septin 7			**0,77**	0,029187
TIA1	TIA1 cytotoxic granule associated RNA binding protein	33,2	19,9	**0,77**	0,006649
PIP5K1C	phosphatidylinositol-4-phosphate 5-kinase type 1 gamma	58,7	35,7	**0,77**	0,006182
CEP170B	centrosomal protein 170B	60,1	37,0	**0,77**	0,010491
PACS2	phosphofurin acidic cluster sorting protein 2	61,5	36,3	**0,77**	0,010682
PON2	paraoxonase 2	34,4	20,5	**0,77**	0,014381
UNC45A	unc-45 myosin chaperone A	57,3	33,9	**0,77**	0,018357
NEK11	NIMA related kinase 11	12,7	7,2	**0,77**	0,031405
SLC25A13	solute carrier family 25 member 13	25,3	15,2	**0,77**	0,013945
VSIG10	V-set and immunoglobulin domain containing 10	21,9	12,3	**0,77**	0,033126
LINC00094	long intergenic non-protein coding RNA 94			**0,77**	0,014168
USP48	ubiquitin specific peptidase 48	51,0	28,9	**0,77**	0,007703
RTCA	RNA 3'-terminal phosphate cyclase	35,1	20,1	**0,77**	0,013032
DZIP3	DAZ interacting zinc finger protein 3	18,5	10,8	**0,77**	0,031393
SLC30A9	solute carrier family 30 member 9	64,4	37,8	**0,77**	0,008959
CMAS	cytidine monophosphate N-acetylneuraminic acid synthetase	47,2	26,2	**0,76**	0,016064
TEAD1	TEA domain transcription factor 1	75,5	44,8	**0,76**	0,021912
SPTLC2	serine palmitoyltransferase long chain base subunit 2	34,9	20,3	**0,76**	0,012636
TUBG1	tubulin gamma 1	32,4	17,4	**0,76**	0,029278
NHLRC3	NHL repeat containing 3	18,7	10,7	**0,76**	0,023773
DYM	dymeclin	50,7	29,3	**0,76**	0,008367
CD2AP	CD2 associated protein	26,2	16,3	**0,76**	0,015545

ANAPC5	anaphase promoting complex subunit 5	113,3	64,2	**0,76**	0,021499
RTL8A	retrotransposon Gag like 8A	24,0	14,7	**0,76**	0,017396
FAM175A	family with sequence similarity 175 member A			**0,76**	0,040567
FAM3A	family with sequence similarity 3 member A	38,1	22,5	**0,76**	0,026531
GATD1	glutamine amidotransferase like class 1 domain containing 1	58,3	33,6	**0,76**	0,013498
CSMD2	CUB and Sushi multiple domains 2	29,8	17,1	**0,76**	0,036511
EIF4B	eukaryotic translation initiation factor 4B	245,3	142,9	**0,76**	0,040477
LIFR	LIF receptor alpha	61,2	40,2	**0,76**	0,013945
ELOVL6	ELOVL fatty acid elongase 6	14,3	7,9	**0,76**	0,032464
SHKBP1	SH3KBP1 binding protein 1	34,7	21,6	**0,76**	0,010882
ITFG1	integrin alpha FG-GAP repeat containing 1	127,9	75,2	**0,76**	0,02234
FCHSD1	FCH and double SH3 domains 1	20,1	12,1	**0,76**	0,024628
PTGR1	prostaglandin reductase 1	41,5	23,2	**0,76**	0,013676
PPP2R5A	protein phosphatase 2 regulatory subunit B'alpha	63,4	36,2	**0,76**	0,014903
RRP15	ribosomal RNA processing 15 homolog	30,3	17,1	**0,76**	0,023427
AP1B1	adaptor related protein complex 1 beta 1 subunit	75,8	43,4	**0,76**	0,008841
VPS41	VPS41, HOPS complex subunit	49,4	28,7	**0,76**	0,007151
ZNF71	zinc finger protein 71	7,8	4,7	**0,76**	0,043408
HMGN1	high mobility group nucleosome binding domain 1	66,4	37,5	**0,76**	0,013218
RNF141	ring finger protein 141	31,8	17,6	**0,76**	0,027155
GRK3	G protein-coupled receptor kinase 3	8,2	4,9	**0,76**	0,046432
LAMTOR2	late endosomal/lysosomal adaptor, MAPK and MTOR activator 2	22,5	13,0	**0,76**	0,025042
PRRC2B	proline rich coiled-coil 2B	232,2	139,2	**0,76**	0,044447
DLG1	discs large MAGUK scaffold protein 1	74,9	43,5	**0,76**	0,012476
MFF	mitochondrial fission factor	36,7	21,1	**0,76**	0,01666
NONO	non-POU domain containing, octamer-binding	182,3	100,9	**0,76**	0,025933
TMEM175	transmembrane protein 175	18,1	10,0	**0,76**	0,044491
EFNA1	ephrin A1	21,1	13,5	**0,76**	0,023108
DDX1	DEAD-box helicase 1	158,0	86,4	**0,76**	0,028887
SCRN3	secernin 3	17,3	9,8	**0,76**	0,020483
RCBTB1	RCC1 and BTB domain containing protein 1	22,4	13,2	**0,76**	0,028736
NAA40	N(alpha)-acetyltransferase 40, NatD catalytic subunit	24,1	13,3	**0,76**	0,016721
FBXL17	F-box and leucine rich repeat protein 17	31,5	18,2	**0,76**	0,017756

DDB1	damage specific DNA binding protein 1	227,4	131,1	**0,76**	0,029521
LINC00346	long intergenic non-protein coding RNA 346	16,8	9,8	**0,76**	0,015294
ARHGEF12	Rho guanine nucleotide exchange factor 12	141,7	85,8	**0,76**	0,023111
SLC35D1	solute carrier family 35 member D1	41,5	24,1	**0,76**	0,009217
FAM49A	family with sequence similarity 49 member A	21,7	12,9	**0,76**	0,009781
TMEM43	transmembrane protein 43	240,4	140,6	**0,76**	0,035285
SUCLG1	succinate-CoA ligase alpha subunit	38,7	20,9	**0,76**	0,011862
FAM208A	family with sequence similarity 208 member A		38,8	**0,76**	0,009437
TMEM246	transmembrane protein 246	30,9	16,9	**0,76**	0,009389
UCHL5	ubiquitin C-terminal hydrolase L5	25,7	14,2	**0,76**	0,015513
STOML2	stomatin like 2	60,4	33,7	**0,76**	0,013325
POLR1E	RNA polymerase I subunit E	19,0	10,8	**0,76**	0,016811
COPB1	coatomer protein complex subunit beta 1	193,7	112,7	**0,76**	0,031311
NOVA1	NOVA alternative splicing regulator 1	81,4	50,1	**0,76**	0,010576
SORBS3	sorbin and SH3 domain containing 3	144,0	81,6	**0,76**	0,028899
CD276	CD276 molecule	159,9	94,8	**0,76**	0,019725
GRSF1	G-rich RNA sequence binding factor 1	87,5	51,0	**0,76**	0,010373
FH	fumarate hydratase	43,3	24,3	**0,76**	0,009576
YWHAB	tyrosine 3-monooxygenase/tryptophan 5-monooxygenase activation	218,5	124,3	**0,76**	0,039725
CCND3	cyclin D3	70,7	38,4	**0,76**	0,01901
DYRK2	dual specificity tyrosine phosphorylation regulated kinase 2	28,8	16,7	**0,76**	0,014512
UTP20	UTP20, small subunit processome component	26,4	15,6	**0,76**	0,040155
COQ8B	coenzyme Q8B	26,2	15,3	**0,76**	0,021516
MAGOHB	mago homolog B, exon junction complex core component	15,2	7,7	**0,76**	0,039709
SORBS1	sorbin and SH3 domain containing 1	21,4	14,2	**0,76**	0,03382
EFNA5	ephrin A5	24,4	13,3	**0,76**	0,015141
KPNB1	karyopherin subunit beta 1	227,4	125,6	**0,76**	0,029406
C2CD5	C2 calcium dependent domain containing 5	29,9	17,0	**0,76**	0,009189
MAGEH1	MAGE family member H1	35,4	19,9	**0,76**	0,035362
EPN2	epsin 2	47,7	26,9	**0,76**	0,004926
YPEL2	yippee like 2	31,0	18,5	**0,76**	0,012585
IPO7	importin 7	139,4	81,5	**0,76**	0,017535
MGLL	monoglyceride lipase	224,9	125,3	**0,76**	0,04226

PHB	prohibitin	91,7	52,0	**0,76**	0,017485
CAPZB	capping actin protein of muscle Z-line beta subunit	157,4	88,7	**0,76**	0,028857
ZNF219	zinc finger protein 219	16,4	10,0	**0,75**	0,016099
RFC2	replication factor C subunit 2	23,7	11,6	**0,75**	0,032465
TRADD	TNFRSF1A associated via death domain	23,6	14,0	**0,75**	0,04481
PNPLA4	patatin like phospholipase domain containing 4	13,7	7,8	**0,75**	0,04162
ATF7IP	activating transcription factor 7 interacting protein	32,4	18,6	**0,75**	0,023593
SIVA1	SIVA1 apoptosis inducing factor	58,8	32,9	**0,75**	0,028161
APEX1	apurinic/apyrimidinic endodeoxyribonuclease 1	80,8	45,3	**0,75**	0,016446
TSEN15	tRNA splicing endonuclease subunit 15	41,0	23,1	**0,75**	0,010128
ATP6AP1	ATPase H+ transporting accessory protein 1	197,9	115,3	**0,75**	0,034238
PRDM11	PR/SET domain 11	15,6	8,5	**0,75**	0,030919
FAM149B1	family with sequence similarity 149 member B1	28,7	17,0	**0,75**	0,005463
POMT1	protein O-mannosyltransferase 1	30,4	17,2	**0,75**	0,009856
ARMCX3	armadillo repeat containing, X-linked 3	74,3	43,7	**0,75**	0,015837
PHYH	phytanoyl-CoA 2-hydroxylase	24,3	12,8	**0,75**	0,036936
TANC1	tetratricopeptide repeat, ankyrin repeat and coiled-coil containing 1	37,6	22,6	**0,75**	0,022428
COA3	cytochrome c oxidase assembly factor 3	32,4	18,0	**0,75**	0,009948
GANC	glucosidase alpha, neutral C	14,9	8,5	**0,75**	0,021175
ERBB2	erb-b2 receptor tyrosine kinase 2	54,3	30,8	**0,75**	0,01392
COPS2	COP9 signalosome subunit 2	92,0	53,3	**0,75**	0,007911
SMARCD2	SWI/SNF related, matrix associated, actin dependent regulator of chro	41,9	23,6	**0,75**	0,009817
STOML1	stomatin like 1	39,4	21,9	**0,75**	0,011832
GIPC1	GIPC PDZ domain containing family member 1	53,0	31,1	**0,75**	0,009675
GLUD1	glutamate dehydrogenase 1	92,6	52,3	**0,75**	0,011723
GRHPR	glyoxylate and hydroxypyruvate reductase	51,7	29,3	**0,75**	0,011439
GALNT11	polypeptide N-acetylgalactosaminyltransferase 11	72,6	41,7	**0,75**	0,007709
DAB2IP	DAB2 interacting protein	50,1	27,1	**0,75**	0,00496
TOR3A	torsin family 3 member A	34,3	17,7	**0,75**	0,014575
DPY19L1	dpy-19 like 1	80,1	45,0	**0,75**	0,010722
SMARCAL1	SWI/SNF related, matrix associated, actin dependent regulator of chro	25,1	14,3	**0,75**	0,008825
EIF3H	eukaryotic translation initiation factor 3 subunit H	141,7	81,6	**0,75**	0,01568
LINC01116	long intergenic non-protein coding RNA 1116	14,9	7,9	**0,75**	0,041247

CDC123	cell division cycle 123	54,4	30,7	**0,75**	0,005583
TMEM255B	transmembrane protein 255B	35,1	20,0	**0,75**	0,010678
SERPINB6	serpin family B member 6	95,2	53,0	**0,75**	0,015443
KDM1A	lysine demethylase 1A	50,1	28,4	**0,75**	0,002614
CS	citrate synthase	60,2	34,4	**0,75**	0,002952
RPGRIP1L	RPGRIP1 like	12,3	6,7	**0,75**	0,042461
DRG1	developmentally regulated GTP binding protein 1	43,1	23,6	**0,75**	0,008405
HOXA11	homeobox A11	21,8	12,4	**0,75**	0,047881
SETBP1	SET binding protein 1	27,3	15,5	**0,75**	0,016064
AKTIP	AKT interacting protein	24,5	13,5	**0,75**	0,018291
KDELR1	KDEL endoplasmic reticulum protein retention receptor 1	172,4	97,5	**0,75**	0,037785
EVI5L	ecotropic viral integration site 5 like	17,4	10,0	**0,75**	0,032056
ERP29	endoplasmic reticulum protein 29	122,1	69,8	**0,75**	0,020435
COX5A	cytochrome c oxidase subunit 5A	49,4	27,8	**0,75**	0,010189
DPP7	dipeptidyl peptidase 7	90,9	53,5	**0,75**	0,012719
EXTL2	exostosin like glycosyltransferase 2	35,5	19,3	**0,75**	0,006014
EIF2S3	eukaryotic translation initiation factor 2 subunit gamma	85,0	48,5	**0,75**	0,00844
NR2C2AP	nuclear receptor 2C2 associated protein	13,9	7,3	**0,75**	0,029073
FAM122B	family with sequence similarity 122B	29,3	15,5	**0,75**	0,013543
NCSTN	nicastrin	120,8	67,9	**0,75**	0,012195
PRDM5	PR/SET domain 5	12,6	7,3	**0,75**	0,029583
ARSK	arylsulfatase family member K	13,2	7,2	**0,75**	0,028076
PBXIP1	PBX homeobox interacting protein 1	110,6	68,9	**0,75**	0,007926
ECHS1	enoyl-CoA hydratase, short chain 1	71,7	39,5	**0,75**	0,014121
TPCN2	two pore segment channel 2	25,0	14,2	**0,75**	0,012585
GLT8D1	glycosyltransferase 8 domain containing 1	54,3	31,1	**0,75**	0,009683
ACAD9	acyl-CoA dehydrogenase family member 9	33,5	18,4	**0,75**	0,004201
AGPAT2	1-acylglycerol-3-phosphate O-acyltransferase 2	24,9	14,0	**0,74**	0,014144
PTPN4	protein tyrosine phosphatase, non-receptor type 4	15,5	9,1	**0,74**	0,036947
H2AFY	H2A histone family member Y	68,7	37,4	**0,74**	0,004984
INO80E	INO80 complex subunit E	48,6	26,1	**0,74**	0,011182
CWC27	CWC27 spliceosome associated protein homolog	31,7	17,5	**0,74**	0,005868
TPM3	tropomyosin 3	279,2	150,9	**0,74**	0,033845

GJA1	gap junction protein alpha 1	168,9	112,8	**0,74**	0,038478
HLTF	helicase like transcription factor	44,9	24,0	**0,74**	0,007573
COMMD10	COMM domain containing 10	14,9	8,2	**0,74**	0,018041
SH2B3	SH2B adaptor protein 3	32,2	18,1	**0,74**	0,002978
ZNF680	zinc finger protein 680	8,9	5,0	**0,74**	0,02916
USP46	ubiquitin specific peptidase 46	23,3	13,7	**0,74**	0,006518
TMED3	transmembrane p24 trafficking protein 3	137,2	76,0	**0,74**	0,034424
ABCA7	ATP binding cassette subfamily A member 7	8,0	4,9	**0,74**	0,029192
POLR3B	RNA polymerase III subunit B	12,6	6,8	**0,74**	0,024102
MYD88	myeloid differentiation primary response 88	19,5	11,7	**0,74**	0,010779
OSBPL5	oxysterol binding protein like 5	70,3	37,6	**0,74**	0,00731
CTSA	cathepsin A	178,6	102,4	**0,74**	0,021835
SNX18	sorting nexin 18	56,6	31,5	**0,74**	0,013889
DCAF6	DDB1 and CUL4 associated factor 6	62,0	35,0	**0,74**	0,002798
RIOX2	ribosomal oxygenase 2	37,0	21,1	**0,74**	0,010923
LRBA	LPS responsive beige-like anchor protein	28,6	16,7	**0,74**	0,022806
TUSC3	tumor suppressor candidate 3	143,5	84,1	**0,74**	0,026851
CTNNAL1	catenin alpha like 1	47,8	24,6	**0,74**	0,005055
BZW2	basic leucine zipper and W2 domains 2	27,5	15,2	**0,74**	0,019491
CDC42EP4	CDC42 effector protein 4	56,8	32,2	**0,74**	0,003472
TUBB6	tubulin beta 6 class V	229,2	117,5	**0,74**	0,037624
LRPPRC	leucine rich pentatricopeptide repeat containing	88,1	50,0	**0,74**	0,007578
ARL10	ADP ribosylation factor like GTPase 10	43,6	25,6	**0,74**	0,006545
HPF1	histone PARylation factor 1	18,7	9,8	**0,74**	0,021595
WDR6	WD repeat domain 6	125,3	70,9	**0,74**	0,008459
SPRYD7	SPRY domain containing 7	23,7	12,7	**0,74**	0,011181
STUB1	STIP1 homology and U-box containing protein 1	66,2	36,8	**0,74**	0,013851
AGPS	alkylglycerone phosphate synthase	40,5	23,3	**0,74**	0,00433
DISP1	dispatched RND transporter family member 1	13,9	7,4	**0,74**	0,048444
HSD17B4	hydroxysteroid 17-beta dehydrogenase 4	70,3	40,5	**0,74**	0,010256
RFNG	RFNG O-fucosylpeptide 3-beta-N-acetylglucosaminyltransferase	43,2	24,0	**0,74**	0,013503
CDCA4	cell division cycle associated 4	25,5	12,9	**0,74**	0,013067
C4orf46	chromosome 4 open reading frame 46	7,9	3,9	**0,74**	0,041219

MCTS1	MCTS1, re-initiation and release factor	23,8	12,7	**0,74**	0,017387
RPA1	replication protein A1	106,5	56,3	**0,74**	0,005188
PRPS1	phosphoribosyl pyrophosphate synthetase 1	54,9	28,3	**0,74**	0,005918
HMGB2	high mobility group box 2	119,2	59,1	**0,74**	0,015963
PRAF2	PRA1 domain family member 2	18,1	10,3	**0,74**	0,020842
MAGI2-AS3	MAGI2 antisense RNA 3	53,7	31,6	**0,74**	0,003917
PITPNM2	phosphatidylinositol transfer protein membrane associated 2	23,0	12,5	**0,74**	0,010741
PARP2	poly(ADP-ribose) polymerase 2	18,4	9,4	**0,74**	0,015059
GCA	grancalcin	6,1	3,4	**0,74**	0,047084
QSER1	glutamine and serine rich 1	43,2	25,9	**0,74**	0,003028
PCSK7	proprotein convertase subtilisin/kexin type 7	28,3	15,0	**0,74**	0,005438
B4GAT1	beta-1,4-glucuronyltransferase 1	11,3	6,4	**0,74**	0,0292
RANBP6	RAN binding protein 6	32,5	18,5	**0,74**	0,002795
IL17RB	interleukin 17 receptor B	13,7	8,9	**0,74**	0,049252
NDUFAF5	NADH:ubiquinone oxidoreductase complex assembly factor 5	7,9	4,2	**0,74**	0,046394
SREBF1	sterol regulatory element binding transcription factor 1	58,8	31,0	**0,74**	0,016691
SCLT1	sodium channel and clathrin linker 1	6,5	3,4	**0,74**	0,037362
SERPING1	serpin family G member 1	232,6	138,1	**0,74**	0,028255
NDUFC2	NADH:ubiquinone oxidoreductase subunit C2	29,0	16,0	**0,74**	0,022984
2.syys	septin 2			**0,74**	0,030574
GLO1	glyoxalase I	108,0	58,7	**0,74**	0,00719
ZNF430	zinc finger protein 430	7,1	4,1	**0,74**	0,034659
TCTN3	tectonic family member 3	69,8	38,1	**0,74**	0,006419
RSU1	Ras suppressor protein 1	83,5	47,3	**0,74**	0,006406
MRPL37	mitochondrial ribosomal protein L37	48,1	25,4	**0,74**	0,012124
NIM1K	NIM1 serine/threonine protein kinase	13,7	7,1	**0,74**	0,040887
CHPT1	choline phosphotransferase 1	58,7	32,4	**0,74**	0,005347
ACSS2	acyl-CoA synthetase short-chain family member 2	17,0	9,4	**0,74**	0,023447
NDUFB1	NADH:ubiquinone oxidoreductase subunit B1	25,1	13,2	**0,74**	0,032291
SH3GLB1	SH3 domain containing GRB2 like, endophilin B1	122,1	68,4	**0,74**	0,007623
SH2B1	SH2B adaptor protein 1	31,7	18,5	**0,74**	0,003462
FAR1	fatty acyl-CoA reductase 1	44,5	25,2	**0,74**	0,00265
GK5	glycerol kinase 5 (putative)	8,8	5,2	**0,74**	0,027756

GPRASP2	G protein-coupled receptor associated sorting protein 2	31,5	17,6	**0,74**	0,005747
ST13	ST13, Hsp70 interacting protein	107,4	59,5	**0,74**	0,005946
INPP5A	inositol polyphosphate-5-phosphatase A	24,1	12,4	**0,74**	0,01261
ZC3H7B	zinc finger CCCH-type containing 7B	90,1	51,7	**0,74**	0,004481
MED14	mediator complex subunit 14	62,0	37,2	**0,74**	0,005957
ZNF438	zinc finger protein 438	9,7	5,6	**0,74**	0,017354
NOL8	nucleolar protein 8	28,2	16,3	**0,74**	0,004654
C11orf1	chromosome 11 open reading frame 1	9,9	5,2	**0,74**	0,04289
ATP5H	ATP synthase, H+ transporting, mitochondrial Fo complex subunit D			**0,74**	0,013857
HNRNPUL1	heterogeneous nuclear ribonucleoprotein U like 1	259,9	141,1	**0,74**	0,016267
CASKIN2	CASK interacting protein 2	21,5	12,3	**0,74**	0,005433
TUBA1C	tubulin alpha 1c	195,2	94,8	**0,73**	0,037362
TSHZ1	teashirt zinc finger homeobox 1	20,5	11,0	**0,73**	0,012034
HEBP2	heme binding protein 2	32,7	17,0	**0,73**	0,015104
HDDC2	HD domain containing 2	64,7	34,2	**0,73**	0,009993
HLCS	holocarboxylase synthetase	30,3	16,3	**0,73**	0,002531
RANBP9	RAN binding protein 9	89,7	50,9	**0,73**	0,003075
C12orf4	chromosome 12 open reading frame 4	15,1	7,7	**0,73**	0,013498
RPL23AP82	ribosomal protein L23a pseudogene 82	6,4	3,7	**0,73**	0,028255
CCDC85C	coiled-coil domain containing 85C	30,8	16,5	**0,73**	0,005084
CDK14	cyclin dependent kinase 14	28,6	15,7	**0,73**	0,00969
HSPH1	heat shock protein family H (Hsp110) member 1	91,7	52,6	**0,73**	0,004287
NDUFAF8	NADH:ubiquinone oxidoreductase complex assembly factor 8	18,1	9,1	**0,73**	0,038722
SRSF1	serine and arginine rich splicing factor 1	151,3	80,5	**0,73**	0,008478
TRIM3	tripartite motif containing 3	11,0	6,4	**0,73**	0,016067
MLLT10	myeloid/lymphoid or mixed-lineage leukemia; translocated to, 10	21,3	12,0	**0,73**	0,004222
RNF130	ring finger protein 130	132,9	73,8	**0,73**	0,0157
WASHC5	WASH complex subunit 5	53,2	29,4	**0,73**	0,002622
FUT10	fucosyltransferase 10	6,5	3,6	**0,73**	0,035371
ZNF512	zinc finger protein 512	14,3	7,9	**0,73**	0,016089
UBE2L6	ubiquitin conjugating enzyme E2 L6	10,0	6,1	**0,73**	0,015776
GTPBP3	GTP binding protein 3 (mitochondrial)	12,1	6,8	**0,73**	0,019119
ZDHHC4	zinc finger DHHC-type containing 4	34,6	19,5	**0,73**	0,01426

HDAC4	histone deacetylase 4	27,1	15,9	**0,73**	0,003921
PROCR	protein C receptor	72,2	42,2	**0,73**	0,011629
SLC25A46	solute carrier family 25 member 46	37,5	20,8	**0,73**	0,004145
GSTP1	glutathione S-transferase pi 1	225,5	120,9	**0,73**	0,035707
ZNF250	zinc finger protein 250	7,5	4,2	**0,73**	0,029998
HIP1R	huntingtin interacting protein 1 related	26,1	14,7	**0,73**	0,003961
CAPRIN1	cell cycle associated protein 1	201,0	113,2	**0,73**	0,011822
SIGMAR1	sigma non-opioid intracellular receptor 1	57,3	30,5	**0,73**	0,008156
SEC31A	SEC31 homolog A, COPII coat complex component	317,3	178,0	**0,73**	0,024783
C3orf18	chromosome 3 open reading frame 18	15,5	7,9	**0,73**	0,019313
APBB1	amyloid beta precursor protein binding family B member 1	42,7	24,3	**0,73**	0,006412
UCP2	uncoupling protein 2	11,9	7,0	**0,73**	0,039579
CPE	carboxypeptidase E	194,1	110,9	**0,73**	0,023039
FAF1	Fas associated factor 1	43,8	22,5	**0,73**	0,00177
ICK	intestinal cell kinase	23,6	12,8	**0,73**	0,006085
SENP7	SUMO1/sentrin specific peptidase 7	16,6	9,6	**0,73**	0,013117
SDC3	syndecan 3	177,6	98,1	**0,73**	0,015669
FBXO9	F-box protein 9	66,0	35,8	**0,73**	0,003472
RFC5	replication factor C subunit 5	11,9	6,0	**0,73**	0,014836
SIM2	single-minded family bHLH transcription factor 2	16,9	9,3	**0,73**	0,029028
PM20D2	peptidase M20 domain containing 2	24,0	14,2	**0,73**	0,003468
TEX264	testis expressed 264	43,8	23,7	**0,73**	0,004683
RAB4A	RAB4A, member RAS oncogene family	25,6	14,0	**0,73**	0,004855
CHSY1	chondroitin sulfate synthase 1	94,5	50,6	**0,73**	0,003229
GLB1L	galactosidase beta 1 like	16,8	9,2	**0,73**	0,017386
CYFIP1	cytoplasmic FMR1 interacting protein 1	66,9	36,2	**0,73**	0,004786
MYADM	myeloid associated differentiation marker	200,2	113,2	**0,73**	0,027487
UROD	uroporphyrinogen decarboxylase	39,3	21,1	**0,73**	0,007041
MEIS3P1	Meis homeobox 3 pseudogene 1	4,4	2,5	**0,73**	0,034744
PTPRU	protein tyrosine phosphatase, receptor type U	45,4	25,7	**0,73**	0,005762
STRN	striatin	44,5	25,4	**0,73**	0,003013
IQCE	IQ motif containing E	30,6	17,3	**0,73**	0,001412
SAMM50	SAMM50 sorting and assembly machinery component	29,5	15,9	**0,73**	0,004466

ANP32A	acidic nuclear phosphoprotein 32 family member A	63,9	34,8	**0,73**	0,002901
ZNF514	zinc finger protein 514	9,2	5,6	**0,73**	0,013372
TRAPPC12	trafficking protein particle complex 12	13,1	7,6	**0,73**	0,010134
KLHL22	kelch like family member 22	17,4	9,1	**0,73**	0,017814
GUK1	guanylate kinase 1	131,0	71,2	**0,73**	0,03149
R3HDM1	R3H domain containing 1	15,7	8,7	**0,73**	0,00951
PARP16	poly(ADP-ribose) polymerase family member 16	11,5	6,5	**0,73**	0,017097
ITGB1BP1	integrin subunit beta 1 binding protein 1	82,1	44,2	**0,73**	0,008671
CYB5R1	cytochrome b5 reductase 1	84,1	44,8	**0,73**	0,007732
CNPY2	canopy FGF signaling regulator 2	18,3	10,0	**0,73**	0,019671
TTLL5	tubulin tyrosine ligase like 5	17,2	9,3	**0,73**	0,011223
ADAM15	ADAM metallopeptidase domain 15	180,3	100,0	**0,73**	0,033595
LPCAT3	lysophosphatidylcholine acyltransferase 3	20,9	11,0	**0,73**	0,012046
EGFLAM	EGF like, fibronectin type III and laminin G domains	35,5	19,4	**0,73**	0,004825
CERS2	ceramide synthase 2	287,8	161,2	**0,73**	0,021304
THYN1	thymocyte nuclear protein 1	20,5	10,7	**0,73**	0,017676
ANKRD9	ankyrin repeat domain 9	16,7	9,5	**0,73**	0,047323
CORO1C	coronin 1C	109,2	58,8	**0,73**	0,007513
PRKDC	protein kinase, DNA-activated, catalytic polypeptide	180,1	103,5	**0,73**	0,022486
SAMD4A	sterile alpha motif domain containing 4A	160,6	92,0	**0,73**	0,012507
PCBD1	pterin-4 alpha-carbinolamine dehydratase 1	16,2	9,0	**0,73**	0,014185
ATPAF1	ATP synthase mitochondrial F1 complex assembly factor 1	48,4	25,1	**0,73**	0,003714
DDX46	DEAD-box helicase 46	66,7	37,0	**0,73**	0,00139
MALT1	MALT1 paracaspase	35,2	19,4	**0,72**	0,000851
IL27RA	interleukin 27 receptor subunit alpha	9,4	4,6	**0,72**	0,039934
MBNL2	muscleblind like splicing regulator 2	79,4	45,9	**0,72**	0,012591
INTS7	integrator complex subunit 7	16,2	8,2	**0,72**	0,009094
MVB12A	multivesicular body subunit 12A	23,1	12,1	**0,72**	0,011327
DIAPH2	diaphanous related formin 2	13,8	8,7	**0,72**	0,008429
HAUS6	HAUS augmin like complex subunit 6	11,4	6,3	**0,72**	0,011716
SLC25A40	solute carrier family 25 member 40	16,6	8,5	**0,72**	0,020902
SNRNP48	small nuclear ribonucleoprotein U11/U12 subunit 48	17,7	9,5	**0,72**	0,009034
CMTM7	CKLF like MARVEL transmembrane domain containing 7	48,0	24,9	**0,72**	0,011241

ATP5C1	ATP synthase, H+ transporting, mitochondrial F1 complex, gamma polypeptide 1			**0,72**	0,00419
NLRP1	NLR family pyrin domain containing 1	36,1	18,9	**0,72**	0,006705
RRAGB	Ras related GTP binding B	8,2	4,2	**0,72**	0,031976
DNMT3A	DNA methyltransferase 3 alpha	14,2	7,3	**0,72**	0,013145
FOXO1	forkhead box O1	57,1	31,7	**0,72**	0,003299
TBCB	tubulin folding cofactor B	89,0	48,6	**0,72**	0,012743
MRPS34	mitochondrial ribosomal protein S34	40,4	21,2	**0,72**	0,013269
PDCD4	programmed cell death 4	119,3	61,2	**0,72**	0,009398
ZNF675	zinc finger protein 675	4,3	2,4	**0,72**	0,035371
GNS	glucosamine (N-acetyl)-6-sulfatase	218,4	120,3	**0,72**	0,014525
ERV3-1	endogenous retrovirus group 3 member 1, envelope	7,2	4,1	**0,72**	0,032384
C15orf61	chromosome 15 open reading frame 61	6,3	3,5	**0,72**	0,041173
ALDH3A2	aldehyde dehydrogenase 3 family member A2	110,0	58,0	**0,72**	0,008048
CA13	carbonic anhydrase 13	5,2	2,7	**0,72**	0,047434
C1orf112	chromosome 1 open reading frame 112	6,5	3,4	**0,72**	0,030377
SERPINB9	serpin family B member 9	13,8	8,0	**0,72**	0,01837
PRMT7	protein arginine methyltransferase 7	16,8	8,7	**0,72**	0,017803
PRKAR2A	protein kinase cAMP-dependent type II regulatory subunit alpha	76,4	42,3	**0,72**	0,001016
UBAC1	UBA domain containing 1	24,9	13,0	**0,72**	0,008705
RAB31	RAB31, member RAS oncogene family	39,1	21,7	**0,72**	0,006637
SNRPA1	small nuclear ribonucleoprotein polypeptide A'	22,4	11,8	**0,72**	0,008199
METTL5	methyltransferase like 5	29,7	15,3	**0,72**	0,011245
REXO2	RNA exonuclease 2	77,0	40,9	**0,72**	0,008241
KIF13A	kinesin family member 13A	54,6	30,8	**0,72**	0,001976
NUCKS1	nuclear casein kinase and cyclin dependent kinase substrate 1	258,4	130,6	**0,72**	0,013054
ZNF74	zinc finger protein 74	7,3	3,8	**0,72**	0,031439
CIC	capicua transcriptional repressor	129,9	73,2	**0,72**	0,003413
RAPGEF3	Rap guanine nucleotide exchange factor 3	6,9	3,7	**0,72**	0,018721
PRCP	prolylcarboxypeptidase	73,5	40,9	**0,72**	0,003557
GADD45GIP1	GADD45G interacting protein 1	67,4	35,0	**0,72**	0,007735
COPG2	coatomer protein complex subunit gamma 2	36,1	19,4	**0,72**	0,001304
LIPA	lipase A, lysosomal acid type	18,5	10,0	**0,72**	0,010251
ZMPSTE24	zinc metallopeptidase STE24	95,6	51,4	**0,72**	0,003975

MOCS2	molybdenum cofactor synthesis 2	32,1	17,0	**0,72**	0,003353
ITGAE	integrin subunit alpha E	10,1	5,4	**0,72**	0,033596
QTRT1	queuine tRNA-ribosyltransferase catalytic subunit 1	16,7	8,7	**0,72**	0,010809
RAB3IL1	RAB3A interacting protein like 1	22,6	12,7	**0,72**	0,001965
PRKCD	protein kinase C delta	35,0	19,6	**0,72**	0,003449
PIGB	phosphatidylinositol glycan anchor biosynthesis class B	14,4	7,5	**0,72**	0,011784
ANAPC1	anaphase promoting complex subunit 1	10,8	5,9	**0,72**	0,027579
KCNQ5	potassium voltage-gated channel subfamily Q member 5	137,4	75,1	**0,72**	0,008206
SMARCA2	SWI/SNF related, matrix associated, actin dependent regulator of chro	126,3	69,0	**0,72**	0,003595
SSH2	slingshot protein phosphatase 2	8,8	4,5	**0,72**	0,029676
PPP5C	protein phosphatase 5 catalytic subunit	51,1	27,1	**0,72**	0,001063
EEA1	early endosome antigen 1	46,5	26,9	**0,72**	0,001411
OLFM1	olfactomedin 1	64,9	33,1	**0,72**	0,013952
DTD2	D-tyrosyl-tRNA deacylase 2 (putative)	9,8	5,0	**0,72**	0,026959
LNP1	leukemia NUP98 fusion partner 1	6,3	3,3	**0,72**	0,039884
POLD2	DNA polymerase delta 2, accessory subunit	108,1	55,4	**0,72**	0,006308
DONSON	downstream neighbor of SON	11,7	5,6	**0,72**	0,024145
TACC2	transforming acidic coiled-coil containing protein 2	39,6	21,0	**0,72**	0,00079
PAICS	phosphoribosylaminoimidazole carboxylase and phosphoribosylaminc	108,9	56,3	**0,72**	0,005559
NDN	necdin, MAGE family member	75,4	39,7	**0,71**	0,002614
PAPLN	papilin, proteoglycan like sulfated glycoprotein	5,4	2,9	**0,71**	0,022754
ATRAID	all-trans retinoic acid induced differentiation factor	149,5	80,6	**0,71**	0,010289
GYG1	glycogenin 1	75,8	41,5	**0,71**	0,003275
DZIP1	DAZ interacting zinc finger protein 1	27,8	15,0	**0,71**	0,002088
STX7	syntaxin 7	83,2	46,2	**0,71**	0,002276
CTTN	cortactin	255,3	137,4	**0,71**	0,010816
LAYN	layilin	9,2	4,6	**0,71**	0,025389
NF2	neurofibromin 2	38,1	20,6	**0,71**	0,000944
CROCC	ciliary rootlet coiled-coil, rootletin	17,1	9,3	**0,71**	0,010844
ZNF32	zinc finger protein 32	18,0	9,7	**0,71**	0,006328
GPRASP1	G protein-coupled receptor associated sorting protein 1	29,7	15,5	**0,71**	0,010747
TBC1D2	TBC1 domain family member 2	21,7	11,4	**0,71**	0,006715
ZNF358	zinc finger protein 358	26,9	15,7	**0,71**	0,004353

CHST12	carbohydrate sulfotransferase 12	45,0	24,7	**0,71**	0,00295
LRFN3	leucine rich repeat and fibronectin type III domain containing 3	18,1	10,4	**0,71**	0,00693
THRA	thyroid hormone receptor, alpha	53,4	29,0	**0,71**	0,001325
EML1	echinoderm microtubule associated protein like 1	50,7	28,4	**0,71**	0,001305
NEGR1	neuronal growth regulator 1	45,9	26,6	**0,71**	0,001626
HSPD1	heat shock protein family D (Hsp60) member 1	191,1	101,0	**0,71**	0,011223
KANTR	KDM5C adjacent transcript	5,2	3,0	**0,71**	0,027068
NUCB1	nucleobindin 1	401,5	226,9	**0,71**	0,03001
TNPO2	transportin 2	70,1	37,5	**0,71**	0,00053
PTPN20	protein tyrosine phosphatase, non-receptor type 20	5,5	2,8	**0,71**	0,026602
FCGRT	Fc fragment of IgG receptor and transporter	76,9	43,3	**0,71**	0,007178
CARNMT1	carnosine N-methyltransferase 1	12,0	6,1	**0,71**	0,020544
ACLY	ATP citrate lyase	238,3	129,9	**0,71**	0,013878
DMKN	dermokine	35,0	20,7	**0,71**	0,016087
RPL39L	ribosomal protein L39 like	8,3	3,6	**0,71**	0,025763
TCEA2	transcription elongation factor A2	26,0	13,3	**0,71**	0,015218
FBXO5	F-box protein 5	11,9	5,7	**0,71**	0,008615
MRPS28	mitochondrial ribosomal protein S28	7,2	3,8	**0,71**	0,025154
SEMA4C	semaphorin 4C	49,0	27,7	**0,71**	0,00161
ALDH18A1	aldehyde dehydrogenase 18 family member A1	124,6	65,1	**0,71**	0,004365
TCF25	transcription factor 25	84,8	44,6	**0,71**	0,004817
HOXC4	homeobox C4	4,9	2,7	**0,71**	0,03256
LDB1	LIM domain binding 1	32,6	17,9	**0,71**	0,001087
COBLL1	cordon-bleu WH2 repeat protein like 1	77,0	42,5	**0,71**	0,004071
ANKRD44	ankyrin repeat domain 44	5,4	3,2	**0,71**	0,016501
NUBPL	nucleotide binding protein like	12,5	6,1	**0,71**	0,024181
EFCAB11	EF-hand calcium binding domain 11	5,3	2,7	**0,71**	0,025656
PRKACA	protein kinase cAMP-activated catalytic subunit alpha	74,4	38,8	**0,71**	0,001024
LPP-AS2	LPP antisense RNA 2	3,6	2,0	**0,71**	0,039482
CCDC18	coiled-coil domain containing 18	5,6	2,7	**0,71**	0,040879
WDYHV1	WDYHV motif containing 1	9,4	5,2	**0,71**	0,017785
HMBS	hydroxymethylbilane synthase	11,8	6,3	**0,71**	0,029348
POLE	DNA polymerase epsilon, catalytic subunit	21,7	10,8	**0,71**	0,007701

MXD4	MAX dimerization protein 4	78,6	43,8	**0,71**	0,005043
MAP1B	microtubule associated protein 1B	38,3	19,8	**0,71**	0,03237
TSEN2	tRNA splicing endonuclease subunit 2	9,1	4,9	**0,71**	0,015676
KCTD20	potassium channel tetramerization domain containing 20	81,5	44,9	**0,71**	0,000706
LEPR	leptin receptor	12,1	6,4	**0,71**	0,015971
NCLN	nicalin	131,5	66,7	**0,71**	0,01613
CPQ	carboxypeptidase Q	54,7	30,6	**0,71**	0,002151
TOP1MT	topoisomerase (DNA) I, mitochondrial	9,7	5,4	**0,71**	0,034357
PFKP	phosphofructokinase, platelet	159,3	83,9	**0,71**	0,008429
PCDHGB6	protocadherin gamma subfamily B, 6	12,3	6,9	**0,71**	0,005082
GGCT	gamma-glutamylcyclotransferase	7,1	3,7	**0,71**	0,041469
IARS2	isoleucyl-tRNA synthetase 2, mitochondrial	115,7	61,1	**0,71**	0,001823
RPSA	ribosomal protein SA	321,0	171,8	**0,71**	0,033285
TSHZ3	teashirt zinc finger homeobox 3	7,8	4,6	**0,71**	0,011816
PRKRA	protein activator of interferon induced protein kinase EIF2AK2	19,4	10,4	**0,71**	0,009538
KCNMA1	potassium calcium-activated channel subfamily M alpha 1	580,5	306,6	**0,71**	0,044288
RNF14	ring finger protein 14	51,6	26,2	**0,71**	0,001509
NDUFB10	NADH:ubiquinone oxidoreductase subunit B10	51,0	26,9	**0,71**	0,004912
VCAN	versican	26,5	12,8	**0,71**	0,03252
AGAP2-AS1	AGAP2 antisense RNA 1	9,7	5,1	**0,71**	0,010251
FARSB	phenylalanyl-tRNA synthetase beta subunit	52,2	26,9	**0,71**	0,004984
COG6	component of oligomeric golgi complex 6	51,8	26,2	**0,71**	0,003009
TRNP1	TMF1-regulated nuclear protein 1	19,8	10,6	**0,71**	0,038404
ZNF788	zinc finger family member 788			**0,71**	0,014337
NUP85	nucleoporin 85	27,1	13,7	**0,71**	0,002253
ARMCX2	armadillo repeat containing, X-linked 2	84,9	45,5	**0,71**	0,002614
CAD	carbamoyl-phosphate synthetase 2, aspartate transcarbamylase, and	59,9	31,5	**0,71**	0,001168
TBC1D2B	TBC1 domain family member 2B	37,7	20,4	**0,71**	0,000677
TBC1D14	TBC1 domain family member 14	35,4	19,6	**0,71**	0,001266
IL13RA1	interleukin 13 receptor subunit alpha 1	288,4	153,9	**0,71**	0,018774
NT5C3B	5'-nucleotidase, cytosolic IIIB	33,0	17,6	**0,71**	0,001006
APOLD1	apolipoprotein L domain containing 1	9,2	4,3	**0,71**	0,0212
PRRG1	proline rich and Gla domain 1	9,9	5,4	**0,71**	0,012031

MTA1	metastasis associated 1	44,5	23,5	**0,71**	0,001041
FECH	ferrochelatase	18,5	10,2	**0,71**	0,002754
SHC1	SHC adaptor protein 1	223,2	118,8	**0,71**	0,012521
HDAC7	histone deacetylase 7	25,3	13,8	**0,71**	0,004758
JAK3	Janus kinase 3	11,6	6,5	**0,71**	0,021179
TRAK2	trafficking kinesin protein 2	40,6	21,7	**0,70**	0,000348
TTC7B	tetratricopeptide repeat domain 7B	8,3	4,7	**0,70**	0,015063
WDFY3-AS2	WDFY3 antisense RNA 2	11,9	5,7	**0,70**	0,020714
TMEM45A	transmembrane protein 45A	159,1	81,0	**0,70**	0,044747
H2AFY2	H2A histone family member Y2	16,3	8,2	**0,70**	0,003549
PMM1	phosphomannomutase 1	37,0	18,8	**0,70**	0,018936
SEC22C	SEC22 homolog C, vesicle trafficking protein	40,8	21,7	**0,70**	0,000777
INPP4A	inositol polyphosphate-4-phosphatase type I A	32,9	17,6	**0,70**	0,001384
CFDP1	craniofacial development protein 1	27,8	14,0	**0,70**	0,002317
PEX10	peroxisomal biogenesis factor 10	39,3	20,5	**0,70**	0,001848
LMNB2	lamin B2	112,5	50,0	**0,70**	0,005392
GNAI2	G protein subunit alpha i2	479,3	252,6	**0,70**	0,036121
MAN2B2	mannosidase alpha class 2B member 2	105,5	58,8	**0,70**	0,001398
ALG14	ALG14, UDP-N-acetylglucosaminyltransferase subunit	10,2	5,1	**0,70**	0,02402
COMMD2	COMM domain containing 2	31,4	16,1	**0,70**	0,001405
MED27	mediator complex subunit 27	19,6	9,3	**0,70**	0,009479
PLA2G15	phospholipase A2 group XV	34,9	17,9	**0,70**	0,005153
AMPD2	adenosine monophosphate deaminase 2	63,0	33,7	**0,70**	0,001474
PSMD1	proteasome 26S subunit, non-ATPase 1	139,1	74,7	**0,70**	0,003275
PRICKLE3	prickle planar cell polarity protein 3	7,8	4,6	**0,70**	0,007049
SGSH	N-sulfoglucosamine sulfohydrolase	59,7	31,2	**0,70**	0,003273
DTYMK	deoxythymidylate kinase	30,3	13,8	**0,70**	0,007944
WDR61	WD repeat domain 61	51,9	26,0	**0,70**	0,002227
USF2	upstream transcription factor 2, c-fos interacting	92,2	48,6	**0,70**	0,002241
N4BP2	NEDD4 binding protein 2	15,2	8,2	**0,70**	0,009997
ADAMTS6	ADAM metallopeptidase with thrombospondin type 1 motif 6	16,4	10,2	**0,70**	0,005776
PCDHGA3	protocadherin gamma subfamily A, 3	7,4	4,0	**0,70**	0,018784
GPR27	G protein-coupled receptor 27	9,9	4,5	**0,70**	0,022347

MAN1B1	mannosidase alpha class 1B member 1	168,5	85,8	**0,70**	0,010813
TCF3	transcription factor 3	43,7	21,4	**0,70**	0,002481
RFTN2	raftlin family member 2	28,0	14,9	**0,70**	0,00942
AES	amino-terminal enhancer of split	123,0	64,5	**0,70**	0,002796
TOMM40L	translocase of outer mitochondrial membrane 40 like	15,9	8,2	**0,70**	0,006147
RIN3	Ras and Rab interactor 3	27,6	13,8	**0,70**	0,01034
ATP5G3	ATP synthase, H+ transporting, mitochondrial Fo complex subunit C3 (subunit 9)			**0,70**	0,00419
FKBP1A	FK506 binding protein 1A	86,1	45,3	**0,70**	0,003034
CPM	carboxypeptidase M	30,1	15,4	**0,70**	0,01778
PYROXD2	pyridine nucleotide-disulphide oxidoreductase domain 2	3,4	1,8	**0,70**	0,034293
ARHGEF28	Rho guanine nucleotide exchange factor 28	12,2	6,8	**0,70**	0,010844
KCTD3	potassium channel tetramerization domain containing 3	40,9	22,5	**0,70**	0,000295
VANGL1	VANGL planar cell polarity protein 1	34,8	17,3	**0,70**	0,000625
LRRC8D	leucine rich repeat containing 8 family member D	21,8	10,9	**0,70**	0,012901
KPNA2	karyopherin subunit alpha 2	40,3	18,0	**0,70**	0,021257
CARF	calcium responsive transcription factor	8,3	4,4	**0,70**	0,029816
MYH14	myosin heavy chain 14	17,3	9,1	**0,70**	0,011809
PRIMPOL	primase and DNA directed polymerase	13,4	6,6	**0,70**	0,006847
PPP1R3E	protein phosphatase 1 regulatory subunit 3E	10,2	5,0	**0,70**	0,018623
LANCL1	LanC like 1	57,7	29,3	**0,70**	0,000763
SCCPDH	saccharopine dehydrogenase (putative)	45,1	22,7	**0,70**	0,000874
SNRNP25	small nuclear ribonucleoprotein U11/U12 subunit 25	17,0	8,3	**0,70**	0,032316
PRPSAP1	phosphoribosyl pyrophosphate synthetase associated protein 1	54,2	27,8	**0,70**	0,0029
TBC1D19	TBC1 domain family member 19	6,9	3,5	**0,70**	0,013589
FIG4	FIG4 phosphoinositide 5-phosphatase	16,5	9,0	**0,70**	0,002981
EHBP1L1	EH domain binding protein 1 like 1	198,0	104,3	**0,70**	0,014821
TCFL5	transcription factor like 5	18,6	9,3	**0,70**	0,012198
CAMK2D	calcium/calmodulin dependent protein kinase II delta	134,4	69,8	**0,70**	0,002773
APOPT1	apoptogenic 1, mitochondrial	15,5	7,9	**0,70**	0,010885
ALKBH7	alkB homolog 7	35,2	19,1	**0,70**	0,010809
RRAGA	Ras related GTP binding A	114,9	59,7	**0,70**	0,003161
SLC26A2	solute carrier family 26 member 2	85,5	45,1	**0,70**	0,003309
JTB	jumping translocation breakpoint	102,1	53,0	**0,70**	0,007369

PIGK	phosphatidylinositol glycan anchor biosynthesis class K	71,5	37,1	**0,70**	0,00199
SUN2	Sad1 and UNC84 domain containing 2	352,8	181,7	**0,70**	0,019307
TFDP2	transcription factor Dp-2	26,6	13,9	**0,70**	0,002132
COX7B	cytochrome c oxidase subunit 7B	41,9	21,4	**0,70**	0,004693
MRPL12	mitochondrial ribosomal protein L12	10,0	5,2	**0,70**	0,020697
NINL	ninein like	5,5	2,8	**0,70**	0,01997
GPAA1	glycosylphosphatidylinositol anchor attachment 1	177,6	92,8	**0,70**	0,009844
AFMID	arylformamidase	8,8	4,1	**0,70**	0,021942
PDK3	pyruvate dehydrogenase kinase 3	14,0	7,2	**0,70**	0,006725
PPP2R5C	protein phosphatase 2 regulatory subunit B'gamma	81,0	41,1	**0,70**	0,001224
SLC39A13	solute carrier family 39 member 13	111,6	60,7	**0,69**	0,005032
HADHA	hydroxyacyl-CoA dehydrogenase/3-ketoacyl-CoA thiolase/enoyl-CoA l	159,8	82,8	**0,69**	0,003102
POGLUT1	protein O-glucosyltransferase 1	29,2	15,2	**0,69**	0,000806
POFUT1	protein O-fucosyltransferase 1	129,6	64,8	**0,69**	0,001837
SAP30L	SAP30 like	35,1	18,9	**0,69**	0,001089
RNASEH2B	ribonuclease H2 subunit B	12,4	6,3	**0,69**	0,005203
ECM1	extracellular matrix protein 1	55,2	29,4	**0,69**	0,00837
DCAF17	DDB1 and CUL4 associated factor 17	20,3	10,9	**0,69**	0,003204
LNPEP	leucyl and cystinyl aminopeptidase	107,2	57,7	**0,69**	0,001611
SKAP2	src kinase associated phosphoprotein 2	21,7	11,8	**0,69**	0,045896
RABEPK	Rab9 effector protein with kelch motifs	16,9	8,4	**0,69**	0,007768
CASP2	caspase 2	20,8	10,4	**0,69**	0,001221
VPS51	VPS51, GARP complex subunit	62,2	33,8	**0,69**	0,002373
PPP1R3C	protein phosphatase 1 regulatory subunit 3C	454,7	214,1	**0,69**	0,031018
PSIP1	PC4 and SFRS1 interacting protein 1	71,1	35,7	**0,69**	0,000818
RFWD3	ring finger and WD repeat domain 3	35,2	17,4	**0,69**	0,000602
SLC25A36	solute carrier family 25 member 36	72,7	38,9	**0,69**	0,000461
JMJD8	jumonji domain containing 8	99,3	51,1	**0,69**	0,003475
ATXN10	ataxin 10	86,5	44,0	**0,69**	0,001386
SIL1	SIL1 nucleotide exchange factor	113,9	58,4	**0,69**	0,008012
RAB11FIP4	RAB11 family interacting protein 4	76,9	36,9	**0,69**	0,002491
RHOXF1-AS1	RHOXF1 antisense RNA 1	4,5	2,3	**0,69**	0,042015
IRAK4	interleukin 1 receptor associated kinase 4	21,5	11,2	**0,69**	0,001145

USP24	ubiquitin specific peptidase 24	77,1	43,0	**0,69**	0,001855
CBX1	chromobox 1	101,7	50,3	**0,69**	0,000654
MAP4	microtubule associated protein 4	403,4	212,5	**0,69**	0,012229
ATP6V0E2	ATPase H+ transporting V0 subunit e2	6,8	3,1	**0,69**	0,04226
SLC39A6	solute carrier family 39 member 6	214,3	111,3	**0,69**	0,004436
FOXRED2	FAD dependent oxidoreductase domain containing 2	27,3	13,8	**0,69**	0,003475
CMTM3	CKLF like MARVEL transmembrane domain containing 3	52,9	26,4	**0,69**	0,002228
IER5L	immediate early response 5 like	155,8	77,2	**0,69**	0,010857
YIF1B	Yip1 interacting factor homolog B, membrane trafficking protein	80,2	41,0	**0,69**	0,012211
STAU2	staufen double-stranded RNA binding protein 2	11,7	6,3	**0,69**	0,003612
TBC1D24	TBC1 domain family member 24	20,6	10,8	**0,69**	0,002113
SPATA7	spermatogenesis associated 7	2,8	1,4	**0,69**	0,036813
DGKD	diacylglycerol kinase delta	21,3	11,1	**0,69**	0,00105
RBMS2	RNA binding motif single stranded interacting protein 2	81,6	41,7	**0,69**	0,002903
GRK4	G protein-coupled receptor kinase 4	5,1	2,5	**0,69**	0,016451
JPT2	Jupiter microtubule associated homolog 2	76,3	37,4	**0,69**	0,000488
GOLIM4	golgi integral membrane protein 4	175,2	90,4	**0,69**	0,00427
SLC10A3	solute carrier family 10 member 3	63,3	31,9	**0,69**	0,002544
TMEM109	transmembrane protein 109	112,0	56,8	**0,69**	0,002337
UBASH3B	ubiquitin associated and SH3 domain containing B	47,2	24,7	**0,69**	0,000399
BANF1	barrier to autointegration factor 1	83,4	40,7	**0,69**	0,003116
BFSP1	beaded filament structural protein 1	5,7	2,9	**0,69**	0,046456
CD164	CD164 molecule	470,4	237,4	**0,69**	0,021899
MAPK9	mitogen-activated protein kinase 9	39,8	20,2	**0,69**	0,000943
PLBD2	phospholipase B domain containing 2	161,5	91,8	**0,69**	0,002595
QARS	glutaminyl-tRNA synthetase	81,1	42,9	**0,69**	0,001247
KCNN4	potassium calcium-activated channel subfamily N member 4	13,0	6,3	**0,69**	0,014042
CMPK1	cytidine/uridine monophosphate kinase 1	167,4	85,8	**0,69**	0,003769
GOT2	glutamic-oxaloacetic transaminase 2	89,1	45,2	**0,69**	0,001003
ADCY6	adenylate cyclase 6	36,3	19,6	**0,69**	0,000353
VPS16	VPS16, CORVET/HOPS core subunit	21,4	11,0	**0,69**	0,001154
CEP152	centrosomal protein 152	6,6	3,1	**0,69**	0,0118
TMUB1	transmembrane and ubiquitin like domain containing 1	38,2	19,9	**0,69**	0,002507

CAB39	calcium binding protein 39	183,0	89,3	**0,69**	0,00391
MAGT1	magnesium transporter 1	175,8	91,4	**0,69**	0,008109
C7orf31	chromosome 7 open reading frame 31	4,3	2,2	**0,69**	0,028981
MRI1	methylthioribose-1-phosphate isomerase 1	19,3	9,8	**0,69**	0,003522
PLAGL1	PLAG1 like zinc finger 1	78,5	39,1	**0,69**	0,001924
GPD2	glycerol-3-phosphate dehydrogenase 2	96,1	52,4	**0,69**	0,000784
GNAZ	G protein subunit alpha z	5,9	3,1	**0,69**	0,023994
PCDHGA11	protocadherin gamma subfamily A, 11	10,1	5,3	**0,69**	0,012301
ANKRD13A	ankyrin repeat domain 13A	32,6	17,3	**0,69**	0,000749
ZNF519	zinc finger protein 519	3,6	1,8	**0,69**	0,028281
HS3ST1	heparan sulfate-glucosamine 3-sulfotransferase 1	10,6	4,8	**0,69**	0,044113
SEPHS1	selenophosphate synthetase 1	44,9	22,6	**0,69**	0,00075
NAXE	NAD(P)HX epimerase	41,3	20,0	**0,69**	0,003028
ALDH2	aldehyde dehydrogenase 2 family (mitochondrial)	50,6	27,1	**0,69**	0,002507
DNPH1	2'-deoxynucleoside 5'-phosphate N-hydrolase 1	12,3	6,1	**0,69**	0,025098
CNPY3	canopy FGF signaling regulator 3	73,0	37,9	**0,69**	0,003405
DOCK7	dedicator of cytokinesis 7	63,8	35,4	**0,69**	0,002595
VCL	vinculin	157,2	79,5	**0,69**	0,002439
MSH2	mutS homolog 2	40,6	19,4	**0,69**	0,000987
RIMKLB	ribosomal modification protein rimK like family member B	9,6	5,0	**0,69**	0,005863
PHOSPHO2	phosphatase, orphan 2	1,7	1,1	**0,69**	0,036662
SHOX2	short stature homeobox 2	7,2	3,8	**0,69**	0,010742
ZFP90	ZFP90 zinc finger protein	32,4	16,5	**0,69**	0,000366
ACOT4	acyl-CoA thioesterase 4	6,1	3,0	**0,69**	0,016807
EBF1	early B-cell factor 1	3,9	2,3	**0,69**	0,029583
DSC2	desmocollin 2	7,7	4,0	**0,69**	0,014616
EPS15	epidermal growth factor receptor pathway substrate 15	31,9	16,9	**0,69**	0,000468
WDFY1	WD repeat and FYVE domain containing 1	86,9	43,8	**0,68**	0,000337
BPNT1	3'(2'), 5'-bisphosphate nucleotidase 1	23,3	11,0	**0,68**	0,002486
TDP1	tyrosyl-DNA phosphodiesterase 1	6,9	3,6	**0,68**	0,006284
ECT2	epithelial cell transforming 2	24,5	11,3	**0,68**	0,001269
ADD1	adducin 1	207,2	107,0	**0,68**	0,002593
AUTS2	AUTS2, activator of transcription and developmental regulator	169,4	92,3	**0,68**	0,00399

C17orf67	chromosome 17 open reading frame 67	2,8	1,5	**0,68**	0,046685
TRAF4	TNF receptor associated factor 4	29,8	14,6	**0,68**	0,00575
C16orf62	chromosome 16 open reading frame 62			**0,68**	0,000344
ATAD3A	ATPase family, AAA domain containing 3A	30,5	14,5	**0,68**	0,007643
AKAP7	A-kinase anchoring protein 7	4,5	2,2	**0,68**	0,021757
FKBP11	FK506 binding protein 11	21,9	12,2	**0,68**	0,020535
ACBD6	acyl-CoA binding domain containing 6	25,0	12,9	**0,68**	0,000945
LRRK1	leucine rich repeat kinase 1	20,6	10,8	**0,68**	0,001787
STAT1	signal transducer and activator of transcription 1	115,8	61,0	**0,68**	0,000836
ZNF737	zinc finger protein 737		1,4	**0,68**	0,028827
SPOCK1	SPARC/osteonectin, cwcv and kazal like domains proteoglycan 1	205,2	113,2	**0,68**	0,017615
AKIP1	A-kinase interacting protein 1	16,9	8,6	**0,68**	0,004937
UGDH	UDP-glucose 6-dehydrogenase	398,1	202,1	**0,68**	0,010912
RANGRF	RAN guanine nucleotide release factor	9,4	4,7	**0,68**	0,00827
CISD1	CDGSH iron sulfur domain 1	16,8	8,1	**0,68**	0,006246
NSUN5P2	NOP2/Sun RNA methyltransferase family member 5 pseudogene 2	4,6	2,4	**0,68**	0,016881
SOCS2-AS1	SOCS2 antisense RNA 1	4,4	2,6	**0,68**	0,01891
CKS2	CDC28 protein kinase regulatory subunit 2	12,0	5,1	**0,68**	0,015683
PATZ1	POZ/BTB and AT hook containing zinc finger 1	22,9	11,6	**0,68**	0,000946
MELTF-AS1	MELTF antisense RNA 1	1,4	0,7	**0,68**	0,048036
ZRANB3	zinc finger RANBP2-type containing 3	5,5	2,7	**0,68**	0,010644
CHAC2	ChaC cation transport regulator homolog 2	7,1	3,6	**0,68**	0,014813
SUCLA2	succinate-CoA ligase ADP-forming beta subunit	40,3	20,1	**0,68**	0,000373
SMAD1	SMAD family member 1	7,3	3,8	**0,68**	0,014621
ERF	ETS2 repressor factor	98,4	48,9	**0,68**	0,002608
PLEKHJ1	pleckstrin homology domain containing J1	18,8	9,7	**0,68**	0,008051
ICMT	isoprenylcysteine carboxyl methyltransferase	124,7	60,4	**0,68**	0,000823
ATP1A1	ATPase Na+/K+ transporting subunit alpha 1	543,2	270,9	**0,68**	0,016267
XPO1	exportin 1	175,5	89,4	**0,68**	0,001897
RPL10A	ribosomal protein L10a	305,7	156,4	**0,68**	0,016424
ZNF480	zinc finger protein 480	12,3	7,2	**0,68**	0,005065
MBD2	methyl-CpG binding domain protein 2	72,5	37,7	**0,68**	0,001411
GAS6	growth arrest specific 6	167,2	84,6	**0,68**	0,004947

SLC44A1	solute carrier family 44 member 1	32,0	16,5	**0,68**	0,002401
PCDHGB2	protocadherin gamma subfamily B, 2	3,0	1,5	**0,68**	0,02649
FAM171A1	family with sequence similarity 171 member A1	8,7	4,5	**0,68**	0,011771
NUTF2	nuclear transport factor 2	53,6	27,3	**0,68**	0,001468
ADAT2	adenosine deaminase, tRNA specific 2	3,7	1,8	**0,68**	0,031857
TXNDC15	thioredoxin domain containing 15	156,0	76,7	**0,68**	0,006694
MLF2	myeloid leukemia factor 2	125,1	62,5	**0,68**	0,004208
SLC22A17	solute carrier family 22 member 17	48,2	23,8	**0,68**	0,002688
S100A6	S100 calcium binding protein A6	682,1	353,9	**0,68**	0,034942
NEK6	NIMA related kinase 6	26,4	14,4	**0,68**	0,002398
LNPK	lunapark, ER junction formation factor	58,5	29,3	**0,68**	0,000181
AP3B1	adaptor related protein complex 3 beta 1 subunit	81,5	40,6	**0,68**	0,000412
MCCC1	methylcrotonoyl-CoA carboxylase 1	14,4	6,8	**0,68**	0,008142
GPR153	G protein-coupled receptor 153	12,1	6,5	**0,68**	0,016187
RDX	radixin	91,4	44,7	**0,68**	0,000312
ATF7IP2	activating transcription factor 7 interacting protein 2	6,6	3,2	**0,68**	0,016913
CAMKK1	calcium/calmodulin dependent protein kinase kinase 1	26,0	13,5	**0,68**	0,001122
ZNF254	zinc finger protein 254	3,9	2,1	**0,68**	0,01985
MR1	major histocompatibility complex, class I-related	38,4	18,5	**0,68**	0,000831
KHDC1	KH homology domain containing 1	2,8	1,4	**0,68**	0,040578
AMOT	angiomotin	8,3	4,0	**0,68**	0,014755
MAGIX	MAGI family member, X-linked	4,5	2,0	**0,68**	0,045899
STON1	stonin 1	12,4	6,7	**0,68**	0,026692
MGST3	microsomal glutathione S-transferase 3	71,9	35,1	**0,68**	0,000925
SCPEP1	serine carboxypeptidase 1	87,8	45,8	**0,68**	0,002901
SNX4	sorting nexin 4	26,6	13,8	**0,68**	0,000337
TMEM220	transmembrane protein 220	9,2	4,4	**0,68**	0,025752
CTSK	cathepsin K	124,0	61,3	**0,68**	0,004586
IDNK	IDNK, gluconokinase	4,6	2,4	**0,68**	0,010511
MYDGF	myeloid derived growth factor	316,5	164,6	**0,68**	0,027539
ARID1B	AT-rich interaction domain 1B	100,9	53,8	**0,68**	0,001164
CCDC102A	coiled-coil domain containing 102A	11,0	5,3	**0,68**	0,011122
TRAPPC9	trafficking protein particle complex 9	18,1	8,9	**0,68**	0,002419

DCLRE1B	DNA cross-link repair 1B	12,6	5,9	**0,68**	0,004466
C7orf73	chromosome 7 open reading frame 73			**0,68**	0,001168
DPY19L4	dpy-19 like 4 (C. elegans)	54,6	28,5	**0,68**	7,90E-05
RABAC1	Rab acceptor 1	43,9	22,2	**0,68**	0,007268
SNRPN	small nuclear ribonucleoprotein polypeptide N	13,4	6,2	**0,68**	0,010676
BBS7	Bardet-Biedl syndrome 7	16,1	8,4	**0,68**	0,001573
CEP70	centrosomal protein 70	12,2	5,7	**0,68**	0,00489
AR	androgen receptor	9,7	5,3	**0,68**	0,007639
MRPS15	mitochondrial ribosomal protein S15	30,2	15,3	**0,67**	0,001036
RANBP1	RAN binding protein 1	57,0	26,4	**0,67**	0,001987
NBPF3	NBPF member 3	3,5	1,9	**0,67**	0,047423
ALDH1B1	aldehyde dehydrogenase 1 family member B1	18,4	9,4	**0,67**	0,006083
C14orf37	chromosome 14 open reading frame 37			**0,67**	0,012205
SPACA9	sperm acrosome associated 9	12,2	5,9	**0,67**	0,010091
HAPLN3	hyaluronan and proteoglycan link protein 3	45,0	24,0	**0,67**	0,000707
CENPL	centromere protein L	9,4	4,1	**0,67**	0,021579
MTMR1	myotubularin related protein 1	15,4	7,4	**0,67**	0,001291
ADAMTS2	ADAM metallopeptidase with thrombospondin type 1 motif 2	18,8	10,1	**0,67**	0,01136
RAI2	retinoic acid induced 2	10,4	5,3	**0,67**	0,011448
RBMX	RNA binding motif protein, X-linked	124,5	58,7	**0,67**	0,000605
NDC1	NDC1 transmembrane nucleoporin	28,4	13,5	**0,67**	0,000367
RTN2	reticulon 2	3,1	1,6	**0,67**	0,047549
RNLS	renalase, FAD dependent amine oxidase	5,5	2,5	**0,67**	0,017719
HSDL2	hydroxysteroid dehydrogenase like 2	35,1	17,0	**0,67**	0,001371
TMEM54	transmembrane protein 54	29,7	14,3	**0,67**	0,00662
MFSD13A	major facilitator superfamily domain containing 13A	1,8	1,0	**0,67**	0,028841
EIF2AK2	eukaryotic translation initiation factor 2 alpha kinase 2	44,1	22,8	**0,67**	0,000103
ERI1	exoribonuclease 1	4,7	2,2	**0,67**	0,016187
PPIP5K2	diphosphoinositol pentakisphosphate kinase 2	55,4	27,5	**0,67**	0,000131
SAAL1	serum amyloid A like 1	7,6	3,6	**0,67**	0,005671
GMPS	guanine monophosphate synthase	48,7	23,5	**0,67**	0,000119
PGPEP1	pyroglutamyl-peptidase I	14,9	7,4	**0,67**	0,005153
SUMF1	sulfatase modifying factor 1	59,5	30,5	**0,67**	0,00101

PKNOX2	PBX/knotted 1 homeobox 2	12,9	5,7	**0,67**	0,014434
METTL8	methyltransferase like 8	11,1	5,6	**0,67**	0,001977
NAAA	N-acylethanolamine acid amidase	29,4	14,8	**0,67**	0,006111
ZNF569	zinc finger protein 569	9,3	4,7	**0,67**	0,00352
ABHD15	abhydrolase domain containing 15	18,8	9,7	**0,67**	0,004308
SLC35B4	solute carrier family 35 member B4	37,1	18,6	**0,67**	0,000353
SFXN2	sideroflexin 2	6,0	2,7	**0,67**	0,019868
FAM89B	family with sequence similarity 89 member B	9,7	5,5	**0,67**	0,019455
A2M	alpha-2-macroglobulin	1221,1	640,4	**0,67**	0,037737
ERLIN1	ER lipid raft associated 1	84,5	39,5	**0,67**	0,000339
ADAM22	ADAM metallopeptidase domain 22	2,9	1,5	**0,67**	0,038423
MYOF	myoferlin	274,1	140,8	**0,67**	0,012375
ADSL	adenylosuccinate lyase	6,3	3,0	**0,67**	0,010562
MYO18A	myosin XVIIIA	42,6	22,1	**0,67**	0,000288
HAUS4	HAUS augmin like complex subunit 4	8,3	4,3	**0,67**	0,007016
TMEM147	transmembrane protein 147	72,9	36,7	**0,67**	0,001771
JAM3	junctional adhesion molecule 3	64,3	31,4	**0,67**	0,000159
CKLF	chemokine like factor	5,0	2,6	**0,67**	0,009455
ZFP30	ZFP30 zinc finger protein	6,1	3,2	**0,67**	0,012895
CBX3	chromobox 3	118,3	57,2	**0,67**	0,000764
MFSD3	major facilitator superfamily domain containing 3	5,3	2,7	**0,67**	0,040924
HSP90AA1	heat shock protein 90 alpha family class A member 1	1053,0	494,0	**0,67**	0,036782
SAE1	SUMO1 activating enzyme subunit 1	97,5	43,3	**0,67**	0,003593
LINC00342	long intergenic non-protein coding RNA 342	2,3	1,6	**0,67**	0,029296
MPV17	MPV17, mitochondrial inner membrane protein	41,0	19,7	**0,67**	0,000842
LRRC28	leucine rich repeat containing 28	9,4	4,6	**0,67**	0,007931
THUMPD2	THUMP domain containing 2	7,8	3,6	**0,67**	0,015778
DIO2	iodothyronine deiodinase 2	29,6	18,0	**0,67**	0,022989
KBTBD3	kelch repeat and BTB domain containing 3	3,7	2,0	**0,67**	0,013113
LSM11	LSM11, U7 small nuclear RNA associated	7,0	3,5	**0,67**	0,00651
CD46	CD46 molecule	202,8	104,7	**0,67**	0,002063
BMPR2	bone morphogenetic protein receptor type 2	65,2	34,9	**0,67**	0,000116
GJC2	gap junction protein gamma 2	2,5	1,4	**0,67**	0,025188

RFC4	replication factor C subunit 4	14,4	6,1	**0,67**	0,008575
CENPN	centromere protein N	15,5	7,1	**0,67**	0,007345
HSPA14	heat shock protein family A (Hsp70) member 14	11,0	12,4	**0,67**	0,009099
FAM168A	family with sequence similarity 168 member A	66,9	33,5	**0,67**	3,60E-05
MLLT1	MLLT1, super elongation complex subunit	173,2	85,0	**0,67**	0,001106
CAMK1D	calcium/calmodulin dependent protein kinase ID	55,0	27,7	**0,67**	0,000108
ABI2	abl interactor 2	61,0	29,3	**0,67**	0,000145
PCOLCE	procollagen C-endopeptidase enhancer	116,3	66,0	**0,67**	0,010772
ZNF516	zinc finger protein 516	15,7	7,9	**0,67**	0,004522
HOXC10	homeobox C10	39,8	19,4	**0,67**	0,001101
DCTN1	dynactin subunit 1	146,2	73,2	**0,67**	0,000451
DPT	dermatopontin	19,2	10,2	**0,67**	0,01008
BMS1P10	BMS1, ribosome biogenesis factor pseudogene 10	1,8	0,9	**0,67**	0,046377
ZNF561-AS1	ZNF561 antisense RNA 1 (head to head)	5,8	2,8	**0,67**	0,022754
ZBED6CL	ZBED6 C-terminal like	1,6	0,8	**0,67**	0,045463
HOXD8	homeobox D8	22,0	11,0	**0,67**	0,002237
PDK2	pyruvate dehydrogenase kinase 2	24,1	11,8	**0,67**	0,00276
TMEM219	transmembrane protein 219	52,0	25,6	**0,67**	0,002
ROR2	receptor tyrosine kinase like orphan receptor 2	75,1	38,6	**0,67**	0,00059
ZNF69	zinc finger protein 69	3,5	1,7	**0,67**	0,032982
XYLB	xylulokinase	6,4	3,5	**0,67**	0,017735
ARHGAP5-AS	ARHGAP5 antisense RNA 1 (head to head)	8,0	3,5	**0,67**	0,030584
FBXL8	F-box and leucine rich repeat protein 8	2,7	1,4	**0,67**	0,046859
ACADM	acyl-CoA dehydrogenase, C-4 to C-12 straight chain	42,1	20,0	**0,67**	0,000177
FHL3	four and a half LIM domains 3	4,6	2,2	**0,67**	0,029028
MPP6	membrane palmitoylated protein 6	27,2	12,4	**0,67**	0,001057
MKX	mohawk homeobox	10,6	4,7	**0,67**	0,036828
SRI	sorcin	59,1	28,0	**0,67**	0,001461
EXOC4	exocyst complex component 4	78,9	38,3	**0,67**	0,000125
C11orf24	chromosome 11 open reading frame 24	68,1	31,8	**0,67**	0,000751
ATP8B2	ATPase phospholipid transporting 8B2	346,2	173,6	**0,67**	0,00432
FLVCR1	feline leukemia virus subgroup C cellular receptor 1	10,4	4,9	**0,67**	0,003986
DCUN1D4	defective in cullin neddylation 1 domain containing 4	33,3	16,3	**0,67**	9,90E-05

TMEM30A	transmembrane protein 30A	360,9	173,1	**0,67**	0,006089
FKBP5	FK506 binding protein 5	25,8	12,4	**0,67**	0,000562
WTIP	WT1 interacting protein	48,5	23,2	**0,67**	0,000583
IFT52	intraflagellar transport 52	26,1	12,6	**0,67**	0,002132
PDP1	pyruvate dehyrogenase phosphatase catalytic subunit 1	15,9	8,4	**0,67**	0,001668
ACTRT3	actin related protein T3	3,0	1,4	**0,67**	0,038893
ATP2B4	ATPase plasma membrane Ca2+ transporting 4	186,8	98,2	**0,67**	0,002906
KIAA1324L	KIAA1324 like	12,8	6,7	**0,66**	0,000922
EXOC5	exocyst complex component 5	47,8	24,1	**0,66**	7,70E-05
FAM161A	family with sequence similarity 161 member A	4,1	2,0	**0,66**	0,009716
MBOAT1	membrane bound O-acyltransferase domain containing 1	11,6	5,8	**0,66**	0,004579
YIPF2	Yip1 domain family member 2	92,0	42,3	**0,66**	0,003582
SEC23A	Sec23 homolog A, coat complex II component	168,9	83,9	**0,66**	0,001009
AACS	acetoacetyl-CoA synthetase	40,6	19,8	**0,66**	0,000454
CLCN4	chloride voltage-gated channel 4	36,1	17,9	**0,66**	0,000466
LCLAT1	lysocardiolipin acyltransferase 1	14,9	7,2	**0,66**	0,002125
DPH5	diphthamide biosynthesis 5	16,6	8,0	**0,66**	0,00085
CEP57L1	centrosomal protein 57 like 1	9,8	4,6	**0,66**	0,006373
MYL12B	myosin light chain 12B	261,1	128,0	**0,66**	0,005093
PRDX6	peroxiredoxin 6	173,9	93,1	**0,66**	0,002992
PLEKHG3	pleckstrin homology and RhoGEF domain containing G3	27,3	12,2	**0,66**	0,001028
EPHB3	EPH receptor B3	42,6	19,0	**0,66**	0,003741
SYTL2	synaptotagmin like 2	6,1	2,8	**0,66**	0,020526
FAM58A	family with sequence similarity 58 member A			**0,66**	0,001438
SLC16A5	solute carrier family 16 member 5	2,9	1,3	**0,66**	0,020842
RBM41	RNA binding motif protein 41	6,9	3,6	**0,66**	0,008012
HPRT1	hypoxanthine phosphoribosyltransferase 1	27,7	12,2	**0,66**	0,0023
BCKDHB	branched chain keto acid dehydrogenase E1 subunit beta	24,1	11,3	**0,66**	0,003653
ST6GALNAC4	ST6 N-acetylgalactosaminide alpha-2,6-sialyltransferase 4	73,4	36,8	**0,66**	0,007505
STX8	syntaxin 8	20,8	9,8	**0,66**	0,002632
FUT8	fucosyltransferase 8	19,8	9,1	**0,66**	0,00147
C19orf70	chromosome 19 open reading frame 70		15,9	**0,66**	0,008758
CAMK1	calcium/calmodulin dependent protein kinase I	10,0	4,7	**0,66**	0,007944

B4GALNT1	beta-1,4-N-acetyl-galactosaminyltransferase 1	8,6	4,3	**0,66**	0,017113
RBFA	ribosome binding factor A (putative)	9,5	4,6	**0,66**	0,007722
FOXN3	forkhead box N3	46,3	23,9	**0,66**	0,001208
USP5	ubiquitin specific peptidase 5	106,8	50,6	**0,66**	0,000386
SEMA5A	semaphorin 5A	3,1	1,7	**0,66**	0,021263
ADRB2	adrenoceptor beta 2	31,0	13,3	**0,66**	0,004188
RPL4	ribosomal protein L4	768,2	380,4	**0,66**	0,023015
GCNT1	glucosaminyl (N-acetyl) transferase 1, core 2	71,6	34,5	**0,66**	0,001688
VWA8	von Willebrand factor A domain containing 8	22,5	10,7	**0,66**	0,000582
SNX5	sorting nexin 5	68,5	31,3	**0,66**	0,000377
PCDHGA5	protocadherin gamma subfamily A, 5	6,4	3,3	**0,66**	0,003709
TTLL11	tubulin tyrosine ligase like 11	5,5	2,7	**0,66**	0,006673
SCAMP5	secretory carrier membrane protein 5	5,2	2,2	**0,66**	0,018429
TTC38	tetratricopeptide repeat domain 38	18,7	9,3	**0,66**	0,000481
MSRB3	methionine sulfoxide reductase B3	70,7	33,9	**0,66**	0,00016
CAPN2	calpain 2	326,8	157,5	**0,66**	0,002992
TRPM8	transient receptor potential cation channel subfamily M member 8	7,5	3,7	**0,66**	0,034609
WDR41	WD repeat domain 41	53,5	23,9	**0,66**	0,000732
AKT1	AKT serine/threonine kinase 1	178,0	87,4	**0,66**	0,001262
MCRIP2	MAPK regulated corepressor interacting protein 2	11,0	5,6	**0,66**	0,00472
TMEM135	transmembrane protein 135	9,8	4,7	**0,66**	0,004946
SYCP2	synaptonemal complex protein 2	5,0	2,5	**0,66**	0,006606
ZC2HC1A	zinc finger C2HC-type containing 1A	8,4	4,3	**0,66**	0,003569
C20orf194	chromosome 20 open reading frame 194	18,1	9,7	**0,66**	0,000309
DCAKD	dephospho-CoA kinase domain containing	28,2	13,9	**0,66**	0,000416
TWIST1	twist family bHLH transcription factor 1	6,5	3,5	**0,66**	0,023925
ORAI2	ORAI calcium release-activated calcium modulator 2	68,7	32,7	**0,66**	0,000389
MPPE1	metallophosphoesterase 1	16,6	7,9	**0,66**	0,002586
SPATS2	spermatogenesis associated serine rich 2	18,8	8,8	**0,66**	0,001308
AIG1	androgen induced 1	59,7	27,7	**0,66**	0,00024
TMEM192	transmembrane protein 192	30,1	14,2	**0,66**	0,000718
HABP4	hyaluronan binding protein 4	47,9	22,0	**0,66**	0,000254
TSPAN4	tetraspanin 4	211,7	98,6	**0,66**	0,007119

TK2	thymidine kinase 2, mitochondrial	50,8	23,5	**0,66**	0,000432
UBE2E3	ubiquitin conjugating enzyme E2 E3	53,1	25,3	**0,66**	0,000602
CD27-AS1	CD27 antisense RNA 1	12,4	5,7	**0,66**	0,003983
ANOS1	anosmin 1	35,9	19,1	**0,66**	0,019371
GPR161	G protein-coupled receptor 161	9,5	4,7	**0,66**	0,004013
PCDHGA1	protocadherin gamma subfamily A, 1	1,5	0,8	**0,66**	0,04273
CAPNS1	calpain small subunit 1	386,7	185,1	**0,66**	0,008758
TSPAN5	tetraspanin 5	49,1	24,2	**0,66**	0,000412
PGM2	phosphoglucomutase 2	65,3	30,0	**0,65**	0,000157
SRSF2	serine and arginine rich splicing factor 2	133,7	61,7	**0,65**	0,001673
HSP90AB1	heat shock protein 90 alpha family class B member 1	797,1	386,0	**0,65**	0,015218
ZHX3	zinc fingers and homeoboxes 3	54,2	25,4	**0,65**	0,000616
KYAT1	kynurenine aminotransferase 1	3,7	1,8	**0,65**	0,011047
SLC46A1	solute carrier family 46 member 1	22,2	10,6	**0,65**	0,000968
DOK4	docking protein 4	33,5	15,9	**0,65**	0,002132
FGF14-AS2	FGF14 antisense RNA 2	5,5	2,7	**0,65**	0,0311
RAB34	RAB34, member RAS oncogene family	97,7	44,3	**0,65**	0,000961
CENPT	centromere protein T	24,2	11,2	**0,65**	0,001131
SEMA3B	semaphorin 3B	23,7	12,1	**0,65**	0,021236
TRAPPC1	trafficking protein particle complex 1	35,1	16,6	**0,65**	0,000818
C19orf60	chromosome 19 open reading frame 60			**0,65**	0,002409
ATP5D	ATP synthase, H+ transporting, mitochondrial F1 complex, delta subunit			**0,65**	0,001986
SH3D19	SH3 domain containing 19	73,4	35,3	**0,65**	0,000164
LDLRAP1	low density lipoprotein receptor adaptor protein 1	50,6	24,7	**0,65**	0,000201
NDUFB2	NADH:ubiquinone oxidoreductase subunit B2	49,0	23,1	**0,65**	0,002439
MFHAS1	malignant fibrous histiocytoma amplified sequence 1	1,3	0,7	**0,65**	0,046739
SARM1	sterile alpha and TIR motif containing 1	20,2	9,9	**0,65**	0,00052
OAZ2	ornithine decarboxylase antizyme 2	73,3	36,8	**0,65**	0,000607
RCAN3	RCAN family member 3	22,4	10,8	**0,65**	0,003472
MANBA	mannosidase beta	61,8	31,3	**0,65**	8,10E-05
ZNF736	zinc finger protein 736	6,3	3,6	**0,65**	0,010882
LINC01963	long intergenic non-protein coding RNA 1963	6,1	2,8	**0,65**	0,013679
MRC2	mannose receptor C type 2		268,9	**0,65**	0,008841

PCDHGA12	protocadherin gamma subfamily A, 12	3,7	1,6	**0,65**	0,030687
WASHC3	WASH complex subunit 3	35,1	16,7	**0,65**	0,000335
PPA2	pyrophosphatase (inorganic) 2	27,2	12,5	**0,65**	0,000722
GPRC5B	G protein-coupled receptor class C group 5 member B	26,0	12,0	**0,65**	0,002976
CAPG	capping actin protein, gelsolin like	127,7	63,2	**0,65**	0,001417
CHCHD6	coiled-coil-helix-coiled-coil-helix domain containing 6	6,5	2,9	**0,65**	0,018119
PTTG1	pituitary tumor-transforming 1	14,3	5,9	**0,65**	0,01752
B3GAT3	beta-1,3-glucuronyltransferase 3	35,7	17,1	**0,65**	0,002355
FEN1	flap structure-specific endonuclease 1	21,4	8,5	**0,65**	0,006941
RBBP7	RB binding protein 7, chromatin remodeling factor	93,6	44,0	**0,65**	0,000616
PCDHB2	protocadherin beta 2	4,3	2,1	**0,65**	0,016987
IMPACT	impact RWD domain protein	37,6	18,2	**0,65**	0,000158
LAMB2	laminin subunit beta 2	515,4	262,9	**0,65**	0,006576
PYGO1	pygopus family PHD finger 1	11,8	5,5	**0,65**	0,004527
NADK2	NAD kinase 2, mitochondrial	38,9	18,4	**0,65**	0,000194
SH3BGRL	SH3 domain binding glutamate rich protein like	80,5	38,2	**0,65**	0,00037
CERNA1	competing endogenous lncRNA 1 for miR-4707-5p and miR-4767	0,9	0,4	**0,65**	0,040053
C8orf82	chromosome 8 open reading frame 82	31,5	16,3	**0,65**	0,000807
HOMER3	homer scaffolding protein 3	20,6	9,6	**0,65**	0,001806
SMIM14	small integral membrane protein 14	332,5	163,6	**0,65**	0,002472
PCYT2	phosphate cytidylyltransferase 2, ethanolamine	36,4	17,8	**0,65**	0,005082
DGKG	diacylglycerol kinase gamma	8,7	4,6	**0,65**	0,001394
PELI3	pellino E3 ubiquitin protein ligase family member 3	7,1	3,3	**0,65**	0,012437
ZFP36L2	ZFP36 ring finger protein like 2	196,7	95,7	**0,65**	0,000774
ROCK2	Rho associated coiled-coil containing protein kinase 2	97,2	45,6	**0,65**	0,000271
ANGEL1	angel homolog 1	13,6	6,8	**0,65**	0,00215
ANK2	ankyrin 2	10,1	4,6	**0,65**	0,007771
HACD3	3-hydroxyacyl-CoA dehydratase 3	82,4	37,9	**0,65**	0,000116
MID2	midline 2	14,9	7,4	**0,65**	0,000939
ANXA11	annexin A11	305,3	145,2	**0,65**	0,003708
FUCA2	fucosidase, alpha-L- 2, plasma	135,9	63,5	**0,65**	0,000715
C8orf37	chromosome 8 open reading frame 37	4,5	2,2	**0,65**	0,006084
NEO1	neogenin 1	92,2	42,3	**0,65**	0,000804

CD81	CD81 molecule	599,8	308,6	**0,65**	0,02246
POLA1	DNA polymerase alpha 1, catalytic subunit	19,7	8,9	**0,65**	8,00E-05
STIM1	stromal interaction molecule 1	39,3	18,7	**0,65**	0,000345
GRIN2A	glutamate ionotropic receptor NMDA type subunit 2A	7,6	4,5	**0,65**	0,009519
CTDSPL	CTD small phosphatase like	66,9	30,8	**0,65**	0,00237
HDAC9	histone deacetylase 9	32,5	15,2	**0,65**	0,001898
CAMK2G	calcium/calmodulin dependent protein kinase II gamma	23,1	11,1	**0,65**	0,000377
PSMG4	proteasome assembly chaperone 4	12,4	5,5	**0,65**	0,005187
FGFR1	fibroblast growth factor receptor 1	585,2	267,7	**0,65**	0,009251
ATP5G2	ATP synthase, H+ transporting, mitochondrial Fo complex subunit C2 (subunit 9)			**0,65**	0,001324
ZNF25	zinc finger protein 25	25,3	12,4	**0,65**	0,000138
CHPF2	chondroitin polymerizing factor 2	149,0	73,0	**0,65**	0,000947
TRUB1	TruB pseudouridine synthase family member 1	29,1	13,7	**0,65**	0,000395
MYL6	myosin light chain 6	458,0	215,8	**0,65**	0,013594
PIP4K2A	phosphatidylinositol-5-phosphate 4-kinase type 2 alpha	50,3	24,4	**0,65**	3,30E-05
CHID1	chitinase domain containing 1	86,6	42,1	**0,65**	0,000455
SIMC1	SUMO interacting motifs containing 1	6,0	2,7	**0,65**	0,005549
CSPG4P13	chondroitin sulfate proteoglycan 4 pseudogene 13	7,4	3,7	**0,65**	0,009437
FUCA1	fucosidase, alpha-L- 1, tissue	47,0	22,1	**0,65**	0,000517
FANCC	Fanconi anemia complementation group C	7,8	3,7	**0,64**	0,002841
GOLGA2P5	golgin A2 pseudogene 5	2,0	1,0	**0,64**	0,015538
NKX3-2	NK3 homeobox 2	15,6	7,1	**0,64**	0,005943
MRPS35	mitochondrial ribosomal protein S35	28,9	13,4	**0,64**	0,000387
CKS1B	CDC28 protein kinase regulatory subunit 1B	13,8	6,0	**0,64**	0,005369
PRRT3-AS1	PRRT3 antisense RNA 1	2,5	1,3	**0,64**	0,035768
ZDHHC8	zinc finger DHHC-type containing 8	47,9	23,2	**0,64**	6,10E-05
PCDHGA2	protocadherin gamma subfamily A, 2	27,3	13,4	**0,64**	0,000874
SERPINA5	serpin family A member 5	169,5	86,8	**0,64**	0,001016
PCYOX1L	prenylcysteine oxidase 1 like	10,3	4,7	**0,64**	0,006068
TBC1D8B	TBC1 domain family member 8B	24,4	11,5	**0,64**	0,000289
ESD	esterase D	95,1	45,1	**0,64**	9,60E-05
PROS1	protein S (alpha)	40,9	20,1	**0,64**	0,000241
ABHD6	abhydrolase domain containing 6	11,9	5,6	**0,64**	0,002493

CDC25B	cell division cycle 25B	49,9	22,4	**0,64**	0,000746
IL17RD	interleukin 17 receptor D	5,2	2,4	**0,64**	0,006898
GLB1	galactosidase beta 1	68,5	33,4	**0,64**	0,000186
SAC3D1	SAC3 domain containing 1	14,1	5,9	**0,64**	0,018189
SOX4	SRY-box 4	113,5	55,3	**0,64**	0,000282
MAB21L2	mab-21 like 2	6,2	3,0	**0,64**	0,011727
PTPN13	protein tyrosine phosphatase, non-receptor type 13	24,3	11,5	**0,64**	0,004286
RALGDS	ral guanine nucleotide dissociation stimulator	4,3	2,1	**0,64**	0,014381
ZNF415	zinc finger protein 415	3,5	1,7	**0,64**	0,026449
CILP2	cartilage intermediate layer protein 2	152,3	84,6	**0,64**	0,004984
DNAJC9	DnaJ heat shock protein family (Hsp40) member C9	27,8	11,6	**0,64**	0,000235
RFC1	replication factor C subunit 1	52,2	24,2	**0,64**	1,70E-05
NSUN5P1	NOP2/Sun RNA methyltransferase family member 5 pseudogene 1	4,0	1,7	**0,64**	0,021303
TMCO4	transmembrane and coiled-coil domains 4	11,9	5,7	**0,64**	0,000796
MMAB	methylmalonic aciduria (cobalamin deficiency) cblB type	26,9	12,6	**0,64**	0,009435
MVK	mevalonate kinase	18,3	8,4	**0,64**	0,017429
C11orf63	chromosome 11 open reading frame 63			**0,64**	0,001417
EXD2	exonuclease 3'-5' domain containing 2	24,9	12,2	**0,64**	0,000147
WNT5B	Wnt family member 5B	20,8	10,9	**0,64**	0,010695
RABGAP1L	RAB GTPase activating protein 1 like	26,4	12,2	**0,64**	0,000457
GPHN	gephyrin	13,1	5,7	**0,64**	0,001154
SLC38A2	solute carrier family 38 member 2	576,4	284,5	**0,64**	0,005868
VAMP5	vesicle associated membrane protein 5	16,3	7,6	**0,64**	0,001528
POLR2J4	RNA polymerase II subunit J4, pseudogene	0,9	1,2	**0,64**	0,02906
TWISTNB	TWIST neighbor	47,2	22,9	**0,64**	8,40E-05
SULF2	sulfatase 2	232,4	114,2	**0,64**	0,000694
CALM3	calmodulin 3	293,5	133,1	**0,64**	0,003472
MCM7	minichromosome maintenance complex component 7	109,1	45,1	**0,64**	0,000392
ALG8	ALG8, alpha-1,3-glucosyltransferase	31,7	14,6	**0,64**	0,000177
HOXA3	homeobox A3	2,6	1,2	**0,64**	0,009633
IAH1	isoamyl acetate-hydrolyzing esterase 1 homolog	24,2	11,3	**0,64**	0,000447
COX6C	cytochrome c oxidase subunit 6C	55,0	26,8	**0,64**	0,002383
RARA	retinoic acid receptor alpha	32,2	14,9	**0,64**	0,000689

MPRIP	myosin phosphatase Rho interacting protein	181,5	84,1	**0,64**	0,000353
RNPEP	arginyl aminopeptidase	77,7	35,4	**0,64**	0,000228
ARHGAP35	Rho GTPase activating protein 35	167,8	80,4	**0,64**	0,001531
RUFY3	RUN and FYVE domain containing 3	18,5	9,6	**0,64**	0,000625
LTBR	lymphotoxin beta receptor	123,3	60,0	**0,64**	0,000532
SIRT5	sirtuin 5	13,7	6,2	**0,64**	0,000541
TYRO3	TYRO3 protein tyrosine kinase	19,9	8,4	**0,64**	0,005671
TARBP1	TAR (HIV-1) RNA binding protein 1	8,5	4,0	**0,64**	0,00692
SLC9A6	solute carrier family 9 member A6	45,7	22,0	**0,64**	2,90E-05
MVP	major vault protein	234,8	116,1	**0,64**	0,000644
TUBGCP4	tubulin gamma complex associated protein 4	18,7	8,9	**0,64**	0,000373
SRSF7	serine and arginine rich splicing factor 7	73,8	30,3	**0,64**	0,000463
SUCLG2-AS1	SUCLG2 antisense RNA 1 (head to head)	1,6	0,8	**0,64**	0,049956
INPP4B	inositol polyphosphate-4-phosphatase type II B	17,4	8,4	**0,64**	0,003266
NACC2	NACC family member 2	47,1	21,8	**0,64**	4,40E-05
FAM57A	family with sequence similarity 57 member A	36,0	16,3	**0,64**	0,000381
COL4A2	collagen type IV alpha 2 chain	17,4	7,9	**0,64**	0,000455
DYNLL1	dynein light chain LC8-type 1	173,3	78,0	**0,64**	0,003048
WDR34	WD repeat domain 34	72,1	31,8	**0,64**	0,000509
APEH	acylaminoacyl-peptide hydrolase	61,9	28,3	**0,64**	5,60E-05
MYO6	myosin VI	67,1	31,7	**0,64**	2,10E-05
SIGIRR	single Ig and TIR domain containing	12,8	5,8	**0,64**	0,021477
CEMIP	cell migration inducing hyaluronan binding protein	68,5	34,8	**0,64**	0,001106
MTA3	metastasis associated 1 family member 3	20,0	9,3	**0,64**	0,000244
SNX7	sorting nexin 7	21,5	10,9	**0,64**	0,000172
MUC1	mucin 1, cell surface associated	13,0	7,4	**0,64**	0,006068
FANCG	Fanconi anemia complementation group G	13,5	5,6	**0,64**	0,001668
TCEAL9	transcription elongation factor A like 9	74,8	35,4	**0,64**	0,000254
PUSL1	pseudouridylate synthase-like 1	14,1	6,0	**0,64**	0,002937
BEX4	brain expressed X-linked 4	44,1	20,4	**0,64**	0,000219
SH3BP2	SH3 domain binding protein 2	106,8	48,6	**0,64**	0,000486
ZNF607	zinc finger protein 607	2,8	1,3	**0,64**	0,026536
RAD51D	RAD51 paralog D	13,8	6,3	**0,64**	0,000493

DSP	desmoplakin	57,4	25,9	**0,64**	0,048132
STK25	serine/threonine kinase 25	85,9	39,3	**0,64**	0,000192
LINC00998	long intergenic non-protein coding RNA 998			**0,64**	0,000378
LYPLAL1	lysophospholipase like 1	19,9	8,7	**0,63**	0,003393
CPT1C	carnitine palmitoyltransferase 1C	9,1	3,8	**0,63**	0,007618
DSG2	desmoglein 2	59,4	27,5	**0,63**	0,011968
KATNAL1	katanin catalytic subunit A1 like 1	35,5	16,9	**0,63**	3,60E-05
TRAPPC6A	trafficking protein particle complex 6A	6,4	3,0	**0,63**	0,006645
ARMCX4	armadillo repeat containing, X-linked 4	15,1	6,6	**0,63**	0,002302
BLVRB	biliverdin reductase B	40,9	19,1	**0,63**	0,000278
IL17D	interleukin 17D	25,8	11,6	**0,63**	0,001863
COL17A1	collagen type XVII alpha 1 chain	1,0	0,6	**0,63**	0,036947
SRPX2	sushi repeat containing protein, X-linked 2	260,1	122,3	**0,63**	0,003575
MEST	mesoderm specific transcript	3,8	1,7	**0,63**	0,031168
CDKN3	cyclin dependent kinase inhibitor 3	10,6	4,1	**0,63**	0,032833
KCTD15	potassium channel tetramerization domain containing 15	30,6	13,6	**0,63**	0,000584
MINK1	misshapen like kinase 1	62,7	29,2	**0,63**	3,20E-05
LSM3	LSM3 homolog, U6 small nuclear RNA and mRNA degradation associa	39,1	17,5	**0,63**	0,001245
ST3GAL6	ST3 beta-galactoside alpha-2,3-sialyltransferase 6	2,1	0,9	**0,63**	0,022754
RNF212	ring finger protein 212	5,5	2,4	**0,63**	0,011968
IVNS1ABP	influenza virus NS1A binding protein	812,1	351,9	**0,63**	0,028511
VKORC1	vitamin K epoxide reductase complex subunit 1	38,6	18,2	**0,63**	0,002401
TRIOBP	TRIO and F-actin binding protein	84,8	40,5	**0,63**	0,000108
NIPSNAP2	nipsnap homolog 2	22,5	10,5	**0,63**	0,00045
AAED1	AhpC/TSA antioxidant enzyme domain containing 1			**0,63**	0,000199
ZHX1	zinc fingers and homeoboxes 1	38,4	17,5	**0,63**	5,20E-05
VAMP8	vesicle associated membrane protein 8	13,1	5,9	**0,63**	0,003299
APOBEC3F	apolipoprotein B mRNA editing enzyme catalytic subunit 3F	2,2	0,9	**0,63**	0,027652
ZDHHC9	zinc finger DHHC-type containing 9	57,5	27,1	**0,63**	9,80E-05
PLEKHA4	pleckstrin homology domain containing A4	73,3	34,0	**0,63**	0,000149
SLC17A5	solute carrier family 17 member 5	55,7	25,9	**0,63**	7,80E-05
HDAC10	histone deacetylase 10	1,0	0,5	**0,63**	0,038502
PFAS	phosphoribosylformylglycinamidine synthase	17,4	7,9	**0,63**	0,00047

PMVK	phosphomevalonate kinase	51,0	22,1	**0,63**	0,000401
C14orf1	chromosome 14 open reading frame 1			**0,63**	0,003073
DYNC1I1	dynein cytoplasmic 1 intermediate chain 1	19,9	9,2	**0,63**	0,000738
SYDE2	synapse defective Rho GTPase homolog 2	4,2	2,1	**0,63**	0,022767
HSPB11	heat shock protein family B (small) member 11	13,6	6,3	**0,63**	0,001218
DANCR	differentiation antagonizing non-protein coding RNA	22,3	10,5	**0,63**	0,001428
BVES	blood vessel epicardial substance	20,8	10,2	**0,63**	0,001705
LSS	lanosterol synthase	10,4	5,1	**0,63**	0,004823
FAM13B	family with sequence similarity 13 member B	71,9	33,8	**0,63**	1,20E-05
PRKCA	protein kinase C alpha	192,1	83,0	**0,63**	0,001307
PPP1R7	protein phosphatase 1 regulatory subunit 7	53,6	23,5	**0,63**	0,000223
HNRNPLP2	heterogeneous nuclear ribonucleoprotein L pseudogene 2	0,8	0,4	**0,63**	0,033126
GRIN2D	glutamate ionotropic receptor NMDA type subunit 2D	2,7	1,3	**0,63**	0,01238
TSPAN3	tetraspanin 3	260,4	120,8	**0,63**	0,00258
DUSP19	dual specificity phosphatase 19	3,3	1,5	**0,63**	0,012337
SLC47A1	solute carrier family 47 member 1	9,9	4,4	**0,63**	0,002522
KIF1BP	KIF1 binding protein	93,5	41,5	**0,63**	5,40E-05
SPATS2L	spermatogenesis associated serine rich 2 like	106,5	46,0	**0,63**	0,000242
LMF1	lipase maturation factor 1	18,1	8,5	**0,63**	0,000122
RARG	retinoic acid receptor gamma	281,5	120,4	**0,63**	0,004301
NUMBL	NUMB like, endocytic adaptor protein	29,0	14,0	**0,63**	5,10E-05
GLUL	glutamate-ammonia ligase	181,8	90,4	**0,63**	0,003812
GRAMD1A	GRAM domain containing 1A	32,4	16,5	**0,63**	1,60E-05
NCAPD3	non-SMC condensin II complex subunit D3	30,7	13,2	**0,63**	2,70E-05
ATG4C	autophagy related 4C cysteine peptidase	10,3	4,7	**0,63**	0,001229
PPA1	pyrophosphatase (inorganic) 1	86,5	39,4	**0,63**	0,001818
BBS9	Bardet-Biedl syndrome 9	17,1	7,7	**0,63**	0,000359
UBL7-AS1	UBL7 antisense RNA 1 (head to head)	1,7	0,9	**0,63**	0,023799
ITM2C	integral membrane protein 2C	333,9	158,1	**0,63**	0,005514
CC2D1A	coiled-coil and C2 domain containing 1A	37,6	17,0	**0,63**	7,60E-05
MAP3K14	mitogen-activated protein kinase kinase kinase 14	15,4	7,1	**0,63**	0,000678
TTC28	tetratricopeptide repeat domain 28	31,1	15,1	**0,62**	0,000191
TRDMT1	tRNA aspartic acid methyltransferase 1	8,9	4,1	**0,62**	0,000684

C1orf21	chromosome 1 open reading frame 21	73,3	34,3	**0,62**	0,000152
XXYLT1	xyloside xylosyltransferase 1	16,0	7,0	**0,62**	0,000751
SMARCC2	SWI/SNF related, matrix associated, actin dependent regulator of chro	71,2	33,9	**0,62**	4,00E-06
DHCR7	7-dehydrocholesterol reductase	79,8	39,8	**0,62**	0,014585
ARMCX6	armadillo repeat containing, X-linked 6	15,7	7,2	**0,62**	0,000682
PAQR8	progestin and adipoQ receptor family member 8	11,2	5,2	**0,62**	0,004152
LDOC1	LDOC1, regulator of NFKB signaling	28,5	11,9	**0,62**	0,000444
SNHG14	small nucleolar RNA host gene 14	35,0	16,2	**0,62**	0,000149
SUMO3	small ubiquitin-like modifier 3	118,8	52,2	**0,62**	0,000598
PBX3	PBX homeobox 3	16,8	8,0	**0,62**	0,00021
SPTBN4	spectrin beta, non-erythrocytic 4	2,3	1,0	**0,62**	0,011055
PHKB	phosphorylase kinase regulatory subunit beta	56,1	25,1	**0,62**	1,50E-05
B4GALNT3	beta-1,4-N-acetyl-galactosaminyltransferase 3	137,4	59,1	**0,62**	0,000869
HOOK2	hook microtubule tethering protein 2	23,0	10,3	**0,62**	0,000174
ERI2	ERI1 exoribonuclease family member 2	11,5	5,1	**0,62**	0,000565
LAPTM4B	lysosomal protein transmembrane 4 beta	70,9	32,3	**0,62**	0,00027
TMEM106C	transmembrane protein 106C	89,0	35,9	**0,62**	0,000313
TPST1	tyrosylprotein sulfotransferase 1	45,4	20,0	**0,62**	0,000115
RFX7	regulatory factor X7	15,8	7,5	**0,62**	0,000294
HIRIP3	HIRA interacting protein 3	24,6	10,2	**0,62**	0,00069
ZBED5-AS1	ZBED5 antisense RNA 1	7,9	3,7	**0,62**	0,004456
ZNF843	zinc finger protein 843	5,9	2,6	**0,62**	0,007694
TOPBP1	topoisomerase (DNA) II binding protein 1	29,7	12,9	**0,62**	4,00E-06
NAA38	N(alpha)-acetyltransferase 38, NatC auxiliary subunit	25,4	11,0	**0,62**	0,002286
PEX11B	peroxisomal biogenesis factor 11 beta	18,5	8,4	**0,62**	0,000586
PRKACB	protein kinase cAMP-activated catalytic subunit beta	40,4	17,8	**0,62**	9,10E-05
C16orf95	chromosome 16 open reading frame 95	0,9	0,4	**0,62**	0,038542
WBSCR17	Williams-Beuren syndrome chromosome region 17			**0,62**	0,005928
GALNT7	polypeptide N-acetylgalactosaminyltransferase 7	64,9	30,4	**0,62**	9,00E-06
RSRC1	arginine and serine rich coiled-coil 1	22,5	10,3	**0,62**	5,30E-05
ALKBH8	alkB homolog 8, tRNA methyltransferase	10,3	4,4	**0,62**	0,00258
PAPSS2	3'-phosphoadenosine 5'-phosphosulfate synthase 2	319,9	140,3	**0,62**	0,001759
DLEU1	deleted in lymphocytic leukemia 1	2,9	1,3	**0,62**	0,022016

UBTF	upstream binding transcription factor, RNA polymerase I	89,7	39,6	**0,62**	6,80E-05
C11orf80	chromosome 11 open reading frame 80	2,3	1,1	**0,62**	0,008988
RAP1GDS1	Rap1 GTPase-GDP dissociation stimulator 1	38,1	16,7	**0,62**	4,60E-05
KIAA0930	KIAA0930	58,1	26,4	**0,62**	7,10E-05
ANO8	anoctamin 8	8,8	3,9	**0,62**	0,006879
DNALI1	dynein axonemal light intermediate chain 1	9,2	4,1	**0,62**	0,001631
KCTD14	potassium channel tetramerization domain containing 14	5,7	2,7	**0,62**	0,003044
ALDH1L2	aldehyde dehydrogenase 1 family member L2	46,1	22,8	**0,62**	5,60E-05
STEAP3	STEAP3 metalloreductase	213,4	99,7	**0,62**	0,000544
CBR1	carbonyl reductase 1	46,2	19,4	**0,62**	0,000147
DMTN	dematin actin binding protein	1,3	0,5	**0,62**	0,042369
CAND2	cullin associated and neddylation dissociated 2 (putative)	5,0	1,9	**0,62**	0,015683
KIF13B	kinesin family member 13B	175,4	76,7	**0,62**	0,000202
TTC37	tetratricopeptide repeat domain 37	81,4	37,2	**0,62**	2,30E-05
RARB	retinoic acid receptor beta	43,8	19,5	**0,62**	0,000123
GPI	glucose-6-phosphate isomerase	149,4	68,8	**0,62**	0,001149
ATIC	5-aminoimidazole-4-carboxamide ribonucleotide formyltransferase/IM	59,6	26,2	**0,62**	0,000172
SIAE	sialic acid acetylesterase	22,4	10,1	**0,62**	0,000454
RWDD1	RWD domain containing 1	44,3	19,5	**0,62**	0,000675
PTOV1	prostate tumor overexpressed 1	68,3	30,6	**0,62**	0,000739
RMDN1	regulator of microtubule dynamics 1	43,5	19,0	**0,61**	1,20E-05
LIN9	lin-9 DREAM MuvB core complex component	7,0	2,9	**0,61**	0,003146
AP1S1	adaptor related protein complex 1 sigma 1 subunit	36,9	16,5	**0,61**	0,00084
SHE	Src homology 2 domain containing E	2,9	1,2	**0,61**	0,012222
CRYZ	crystallin zeta	51,9	23,5	**0,61**	0,000498
BCAM	basal cell adhesion molecule (Lutheran blood group)	1,5	0,7	**0,61**	0,039359
ARHGAP18	Rho GTPase activating protein 18	5,2	2,2	**0,61**	0,004752
PAM	peptidylglycine alpha-amidating monooxygenase	588,0	269,9	**0,61**	0,00317
SPP1	secreted phosphoprotein 1	3343,3	1598,2	**0,61**	0,044622
SKP2	S-phase kinase associated protein 2	13,0	5,3	**0,61**	0,00048
HNRNPA3	heterogeneous nuclear ribonucleoprotein A3	186,7	81,2	**0,61**	0,000386
SRSF3	serine and arginine rich splicing factor 3	191,3	79,8	**0,61**	0,000753
PRKN	parkin RBR E3 ubiquitin protein ligase	1,4	0,5	**0,61**	0,040582

HSBP1L1	heat shock factor binding protein 1 like 1	5,1	2,0	**0,61**	0,02377
DPY19L3	dpy-19 like 3 (C. elegans)	73,4	33,8	**0,61**	8,00E-06
ZNF385D	zinc finger protein 385D	125,5	55,5	**0,61**	6,20E-05
CRLF1	cytokine receptor like factor 1	116,5	54,3	**0,61**	0,002411
NEIL2	nei like DNA glycosylase 2	1,4	0,6	**0,61**	0,022343
EIF2D	eukaryotic translation initiation factor 2D	34,0	14,7	**0,61**	0,000199
PRPS2	phosphoribosyl pyrophosphate synthetase 2	18,4	7,9	**0,61**	0,001619
CA5B	carbonic anhydrase 5B	41,7	16,4	**0,61**	0,000199
ZGRF1	zinc finger GRF-type containing 1	4,6	1,8	**0,61**	0,007717
BAIAP2-AS1	BAIAP2 antisense RNA 1 (head to head)	22,7	21,2	**0,61**	0,000448
VAMP4	vesicle associated membrane protein 4	19,4	8,1	**0,61**	0,001116
CCL28	C-C motif chemokine ligand 28	13,1	5,8	**0,61**	0,000108
FHL2	four and a half LIM domains 2	150,1	66,8	**0,61**	0,000946
SERGEF	secretion regulating guanine nucleotide exchange factor	9,6	4,6	**0,61**	0,006079
APPL2	adaptor protein, phosphotyrosine interacting with PH domain and leu	77,0	33,7	**0,61**	9,00E-06
DUSP7	dual specificity phosphatase 7	53,1	23,0	**0,61**	2,10E-05
GLMP	glycosylated lysosomal membrane protein	45,1	20,2	**0,61**	3,90E-05
CKAP5	cytoskeleton associated protein 5	115,9	52,0	**0,61**	5,70E-05
TBC1D5	TBC1 domain family member 5	62,6	29,2	**0,61**	1,90E-05
PGF	placental growth factor	7,1	2,9	**0,61**	0,020824
TM7SF3	transmembrane 7 superfamily member 3	71,8	32,1	**0,61**	1,30E-05
TMEM87B	transmembrane protein 87B	50,2	22,0	**0,61**	3,70E-05
C2CD2	C2 calcium dependent domain containing 2	42,3	19,6	**0,61**	1,00E-06
HTRA1	HtrA serine peptidase 1	1079,4	537,9	**0,61**	0,011138
CHSY3	chondroitin sulfate synthase 3	28,1	12,3	**0,61**	0,000377
BGN	biglycan	1736,6	826,3	**0,61**	0,020417
THBS3	thrombospondin 3	18,7	9,0	**0,61**	0,001827
BLVRA	biliverdin reductase A	36,1	15,8	**0,61**	0,000236
RDH10	retinol dehydrogenase 10	43,2	20,2	**0,61**	9,10E-05
CROT	carnitine O-octanoyltransferase	17,2	7,7	**0,61**	0,000219
NFIC	nuclear factor I C	235,0	103,9	**0,61**	0,000202
TCEAL1	transcription elongation factor A like 1	15,0	6,7	**0,61**	0,000305
RNASET2	ribonuclease T2	9,2	4,3	**0,61**	0,00741

HVCN1	hydrogen voltage gated channel 1	2,2	0,7	**0,61**	0,020444
SH3BP4	SH3 domain binding protein 4	30,1	13,7	**0,61**	5,70E-05
ZNF626	zinc finger protein 626	6,4	2,9	**0,61**	0,001258
DENND3	DENN domain containing 3	29,8	14,3	**0,61**	3,00E-06
TP53I13	tumor protein p53 inducible protein 13	64,5	28,1	**0,61**	0,000539
SERP2	stress associated endoplasmic reticulum protein family member 2	7,8	3,2	**0,61**	0,002605
PLA2G12A	phospholipase A2 group XIIA	17,9	7,4	**0,61**	0,000633
ZNF257	zinc finger protein 257	1,1	0,4	**0,61**	0,025198
PCDHGB4	protocadherin gamma subfamily B, 4	7,4	3,7	**0,61**	0,003301
PLEK	pleckstrin	1,7	0,8	**0,61**	0,032261
SUMF2	sulfatase modifying factor 2	213,3	96,3	**0,61**	0,000443
FAM210B	family with sequence similarity 210 member B	61,7	27,2	**0,61**	1,50E-05
TWF2	twinfilin actin binding protein 2	28,4	12,9	**0,61**	0,000836
CDC14B	cell division cycle 14B	6,7	2,8	**0,61**	0,011474
RBL2	RB transcriptional corepressor like 2	75,1	33,9	**0,61**	9,00E-06
DDR2	discoidin domain receptor tyrosine kinase 2	390,8	176,9	**0,61**	0,002989
THRB	thyroid hormone receptor beta	49,5	21,2	**0,61**	7,70E-05
ABLIM1	actin binding LIM protein 1	12,9	6,2	**0,61**	0,000175
BCAT2	branched chain amino acid transaminase 2	20,4	9,0	**0,61**	0,000105
LLGL1	LLGL1, scribble cell polarity complex component	45,5	19,9	**0,61**	1,80E-05
RPS26	ribosomal protein S26	89,7	42,2	**0,61**	0,000239
TPD52	tumor protein D52	46,7	21,1	**0,61**	1,20E-05
LINC01770	long intergenic non-protein coding RNA 1770	1,0	0,4	**0,61**	0,029335
MPND	MPN domain containing	9,7	4,8	**0,61**	0,005612
CTHRC1	collagen triple helix repeat containing 1	20,3	8,4	**0,61**	0,007604
ZNF185	zinc finger protein 185 with LIM domain	7,6	2,9	**0,61**	0,003151
TANC2	tetratricopeptide repeat, ankyrin repeat and coiled-coil containing 2	11,0	5,3	**0,61**	0,001966
MSRB2	methionine sulfoxide reductase B2	17,3	7,4	**0,61**	0,001009
CEP78	centrosomal protein 78	17,5	7,0	**0,61**	0,000343
TMEM9	transmembrane protein 9	55,3	25,6	**0,61**	0,000693
BARX1-AS1	BARX1 antisense RNA 1 (head to head)	86,1	23,6	**0,61**	0,044258
HNRNPA1	heterogeneous nuclear ribonucleoprotein A1	446,8	195,5	**0,61**	0,00139
IQCA1	IQ motif containing with AAA domain 1	12,3	5,2	**0,61**	0,00083

FAM86C2P	family with sequence similarity 86 member C2, pseudogene	2,7	1,1	**0,61**	0,008655
LRP11	LDL receptor related protein 11	97,1	41,5	**0,61**	2,10E-05
MIIP	migration and invasion inhibitory protein	15,8	6,5	**0,60**	0,002452
AP1G2	adaptor related protein complex 1 gamma 2 subunit	17,7	8,0	**0,60**	4,10E-05
C15orf41	chromosome 15 open reading frame 41	3,4	1,5	**0,60**	0,001926
CLN6	ceroid-lipofuscinosis, neuronal 6, late infantile, variant	15,1	6,5	**0,60**	0,000175
GM2A	GM2 ganglioside activator	38,1	17,3	**0,60**	8,00E-04
COX16	COX16, cytochrome c oxidase assembly homolog	2,1	1,0	**0,60**	0,018418
PWP1	PWP1 homolog, endonuclein	51,9	23,4	**0,60**	1,00E-05
LRRCC1	leucine rich repeat and coiled-coil centrosomal protein 1	15,8	6,7	**0,60**	0,000479
DNMT1	DNA methyltransferase 1	89,6	35,2	**0,60**	7,00E-06
ZFYVE28	zinc finger FYVE-type containing 28	11,2	5,2	**0,60**	0,000902
NUDT14	nudix hydrolase 14	6,6	3,0	**0,60**	0,006725
FIS1	fission, mitochondrial 1	87,2	37,5	**0,60**	0,000374
DNAJC18	DnaJ heat shock protein family (Hsp40) member C18	13,8	6,1	**0,60**	0,000656
CMPK2	cytidine/uridine monophosphate kinase 2	1,7	0,8	**0,60**	0,00743
BCL7C	BCL tumor suppressor 7C	48,6	21,1	**0,60**	0,000332
VPS26B	VPS26, retromer complex component B	48,0	21,2	**0,60**	6,00E-06
IGF2	insulin like growth factor 2	4,7	2,4	**0,60**	0,00213
ARHGDIA	Rho GDP dissociation inhibitor alpha	486,3	213,9	**0,60**	0,00191
ATG10	autophagy related 10	8,8	3,7	**0,60**	0,001016
C1orf122	chromosome 1 open reading frame 122	35,7	15,8	**0,60**	0,000203
SEC31B	SEC31 homolog B, COPII coat complex component	2,2	0,9	**0,60**	0,014791
TP53TG1	TP53 target 1 (non-protein coding)	7,2	2,6	**0,60**	0,016147
OGFOD3	2-oxoglutarate and iron dependent oxygenase domain containing 3	40,6	18,1	**0,60**	0,000197
CENPJ	centromere protein J	7,9	3,2	**0,60**	0,000993
EIF3EP1	eukaryotic translation initiation factor 3 subunit E pseudogene 1	0,9	0,4	**0,60**	0,032158
PRIM2	primase (DNA) subunit 2	8,0	3,2	**0,60**	0,000898
DNASE2	deoxyribonuclease 2, lysosomal	63,0	29,1	**0,60**	8,70E-05
TDRD3	tudor domain containing 3	13,4	5,7	**0,60**	0,000413
SETDB2	SET domain bifurcated 2	12,7	5,5	**0,60**	0,001406
C2orf82	chromosome 2 open reading frame 82			**0,60**	0,012341
PALLD	palladin, cytoskeletal associated protein	70,3	27,5	**0,60**	0,000182

DVL1	dishevelled segment polarity protein 1	93,6	40,8	**0,60**	0,00013
PPT1	palmitoyl-protein thioesterase 1	210,4	91,2	**0,60**	0,000238
CALD1	caldesmon 1	149,8	68,1	**0,60**	3,30E-05
ECI2	enoyl-CoA delta isomerase 2	36,1	15,4	**0,60**	8,70E-05
MRPL48	mitochondrial ribosomal protein L48	14,4	6,3	**0,60**	0,001769
THSD4	thrombospondin type 1 domain containing 4	19,9	8,8	**0,60**	0,000514
SGSM2	small G protein signaling modulator 2	24,5	10,8	**0,60**	1,90E-05
AP2M1	adaptor related protein complex 2 mu 1 subunit	377,1	161,4	**0,60**	0,000823
SOCS2	suppressor of cytokine signaling 2	43,3	20,4	**0,60**	4,80E-05
KLHL23	kelch like family member 23	6,7	3,0	**0,60**	0,000335
NDNF	neuron derived neurotrophic factor	37,3	16,8	**0,60**	1,00E-06
IFFO1	intermediate filament family orphan 1	19,7	8,3	**0,60**	0,000506
SAP30	Sin3A associated protein 30	40,0	16,3	**0,60**	0,00015
ZNF714	zinc finger protein 714	2,8	1,1	**0,60**	0,006362
SLC16A9	solute carrier family 16 member 9	4,1	1,5	**0,60**	0,001844
HAND2	heart and neural crest derivatives expressed 2	2,0	0,8	**0,60**	0,038726
ULK2	unc-51 like autophagy activating kinase 2	20,7	9,2	**0,60**	0,000103
TMOD1	tropomodulin 1	13,2	6,4	**0,60**	0,005402
NT5DC3	5'-nucleotidase domain containing 3	17,3	7,2	**0,60**	0,000114
HACE1	HECT domain and ankyrin repeat containing E3 ubiquitin protein ligas	15,3	7,0	**0,60**	4,50E-05
HNRNPUL2	heterogeneous nuclear ribonucleoprotein U like 2	47,9	20,8	**0,60**	2,40E-05
FLRT2	fibronectin leucine rich transmembrane protein 2	16,2	6,4	**0,60**	0,003165
PRKX	protein kinase, X-linked	3,7	1,7	**0,60**	0,014056
UBE2Q2	ubiquitin conjugating enzyme E2 Q2	82,1	36,0	**0,60**	4,00E-06
KCNK15	potassium two pore domain channel subfamily K member 15	8,5	3,4	**0,60**	0,004164
HAUS5	HAUS augmin like complex subunit 5	13,8	5,6	**0,60**	0,000212
CDC42EP3	CDC42 effector protein 3	142,3	56,1	**0,60**	0,000583
C1R	complement C1r	222,5	104,2	**0,60**	0,000791
PPIA	peptidylprolyl isomerase A	318,4	139,1	**0,60**	0,001436
PRG4	proteoglycan 4	766,3	385,6	**0,60**	0,003656
EBP	emopamil binding protein (sterol isomerase)	14,7	6,3	**0,60**	0,017713
TMCO3	transmembrane and coiled-coil domains 3	301,7	131,4	**0,59**	0,000501
KLHL13	kelch like family member 13	7,3	2,9	**0,59**	0,003875

TRNAU1AP	tRNA selenocysteine 1 associated protein 1	16,8	7,0	**0,59**	0,000225
COLGALT1	collagen beta(1-O)galactosyltransferase 1	235,9	96,9	**0,59**	0,00079
PLXNB3	plexin B3	6,2	2,6	**0,59**	0,000868
CA9	carbonic anhydrase 9	4,7	2,4	**0,59**	0,013759
SLC35B2	solute carrier family 35 member B2	172,6	72,4	**0,59**	0,000685
LTBP2	latent transforming growth factor beta binding protein 2	1129,7	519,8	**0,59**	0,00538
SOGA1	suppressor of glucose, autophagy associated 1	42,0	18,2	**0,59**	4,70E-05
TRAM2-AS1	TRAM2 antisense RNA 1 (head to head)	15,7	6,7	**0,59**	0,000225
MBNL3	muscleblind like splicing regulator 3	8,5	3,7	**0,59**	0,004602
FBXL22	F-box and leucine rich repeat protein 22	0,5	0,3	**0,59**	0,03867
DDX43	DEAD-box helicase 43	2,2	0,9	**0,59**	0,009756
NAV1	neuron navigator 1	157,1	67,5	**0,59**	8,30E-05
CYP26B1	cytochrome P450 family 26 subfamily B member 1	55,3	26,6	**0,59**	3,00E-06
BMP4	bone morphogenetic protein 4	5,0	2,1	**0,59**	0,003295
HIBCH	3-hydroxyisobutyryl-CoA hydrolase	13,9	6,1	**0,59**	0,000119
NIPAL2	NIPA like domain containing 2	12,3	5,4	**0,59**	0,000595
TRIP6	thyroid hormone receptor interactor 6	122,4	51,4	**0,59**	9,80E-05
PLAC9	placenta specific 9	137,9	62,6	**0,59**	0,001937
SMYD2	SET and MYND domain containing 2	16,7	7,4	**0,59**	0,000116
BTBD2	BTB domain containing 2	60,4	25,8	**0,59**	2,90E-05
ERCC8	ERCC excision repair 8, CSA ubiquitin ligase complex subunit	6,7	2,9	**0,59**	0,000652
ARSG	arylsulfatase G	11,5	4,6	**0,59**	0,001908
SGCE	sarcoglycan epsilon	16,8	6,9	**0,59**	0,001605
CARD19	caspase recruitment domain family member 19	37,6	17,4	**0,59**	0,000116
VASH1	vasohibin 1	5,6	2,3	**0,59**	0,008581
TIMP4	TIMP metallopeptidase inhibitor 4	54,3	23,5	**0,59**	0,000144
HAUS1	HAUS augmin like complex subunit 1	13,3	5,2	**0,59**	0,00025
SNX22	sorting nexin 22	3,5	1,5	**0,59**	0,046786
IKBIP	IKBKB interacting protein	79,9	33,5	**0,59**	0,000177
AP2B1	adaptor related protein complex 2 beta 1 subunit	227,3	92,7	**0,59**	5,80E-05
NQO1	NAD(P)H quinone dehydrogenase 1	139,1	52,4	**0,59**	0,003173
PARP3	poly(ADP-ribose) polymerase family member 3	23,8	10,2	**0,59**	1,30E-05
ABRACL	ABRA C-terminal like	15,3	6,6	**0,59**	0,00082

ACTB	actin beta	1980,2	820,6	**0,59**	0,02209
DYSF	dysferlin	31,9	13,5	**0,59**	0,000914
CAMKMT	calmodulin-lysine N-methyltransferase	3,2	1,4	**0,59**	0,004662
ZDHHC15	zinc finger DHHC-type containing 15	6,4	2,7	**0,59**	0,001493
CBX6	chromobox 6	119,7	50,2	**0,59**	1,70E-05
APPL1	adaptor protein, phosphotyrosine interacting with PH domain and leu	70,5	29,8	**0,59**	3,00E-06
ADGRG2	adhesion G protein-coupled receptor G2	56,3	26,7	**0,59**	1,00E-06
LASP1	LIM and SH3 protein 1	247,8	107,3	**0,59**	0,000355
CRYL1	crystallin lambda 1	18,7	7,2	**0,59**	0,000637
DUBR	DPPA2 upstream binding RNA	4,1	1,7	**0,59**	0,003449
ANKRD13B	ankyrin repeat domain 13B	8,0	3,4	**0,59**	0,000339
ZNF738	zinc finger protein 738	5,5	2,0	**0,59**	0,004494
MAPKAPK3	mitogen-activated protein kinase-activated protein kinase 3	58,1	23,2	**0,59**	6,20E-05
VASH1-AS1	VASH1 antisense RNA 1	2,1	0,9	**0,59**	0,009285
LINC00632	long intergenic non-protein coding RNA 632	1,2	0,5	**0,59**	0,012488
TMEM237	transmembrane protein 237	34,3	14,3	**0,59**	6,00E-05
TRAF5	TNF receptor associated factor 5	2,3	1,1	**0,59**	0,006384
NAGLU	N-acetyl-alpha-glucosaminidase	73,3	30,7	**0,59**	0,000123
CPT1A	carnitine palmitoyltransferase 1A	49,4	22,0	**0,59**	1,10E-05
NPM3	nucleophosmin/nucleoplasmin 3	11,1	4,4	**0,59**	0,004103
ESYT2	extended synaptotagmin 2	209,1	90,5	**0,59**	6,60E-05
VPS37D	VPS37D, ESCRT-I subunit	1,9	0,8	**0,59**	0,019049
KIAA1614	KIAA1614	11,4	5,1	**0,59**	0,000201
TBCK	TBC1 domain containing kinase	35,6	14,7	**0,59**	1,30E-05
AHNAK	AHNAK nucleoprotein	2549,3	1190,8	**0,59**	0,015491
2.maalis	mitochondrial amidoxime reducing component 2			**0,59**	0,000556
DCHS1	dachsous cadherin-related 1	182,7	78,0	**0,59**	2,80E-05
STK17B	serine/threonine kinase 17b	8,9	3,9	**0,59**	0,002243
EZH2	enhancer of zeste 2 polycomb repressive complex 2 subunit	13,1	4,6	**0,59**	0,001508
SCARA3	scavenger receptor class A member 3	633,1	250,6	**0,59**	0,002604
DUSP23	dual specificity phosphatase 23	10,7	4,3	**0,59**	0,005007
KLF15	Kruppel like factor 15	17,4	6,7	**0,59**	0,00372
STK39	serine/threonine kinase 39	46,9	18,2	**0,59**	5,10E-05

GTF3A	general transcription factor IIIA	74,1	31,3	**0,59**	8,80E-05
MIR4435-2H	MIR4435-2 host gene	21,3	9,2	**0,59**	0,000101
AGBL5	ATP/GTP binding protein like 5	16,5	7,1	**0,59**	7,40E-05
CTSD	cathepsin D	988,6	409,9	**0,59**	0,009795
RTCA-AS1	RTCA antisense RNA 1	0,9	0,4	**0,59**	0,020143
METTL26	methyltransferase like 26	29,8	12,8	**0,59**	0,000239
CCDC88A	coiled-coil domain containing 88A	110,9	46,9	**0,59**	3,80E-05
HGSNAT	heparan-alpha-glucosaminide N-acetyltransferase	73,9	32,3	**0,59**	5,00E-06
NT5M	5',3'-nucleotidase, mitochondrial	2,3	1,0	**0,59**	0,022345
LINC01117	long intergenic non-protein coding RNA 1117	1,1	0,5	**0,58**	0,006984
TXNRD3	thioredoxin reductase 3	7,9	3,2	**0,58**	0,00105
PCDHGA4	protocadherin gamma subfamily A, 4	4,2	1,7	**0,58**	0,003084
DPYSL2	dihydropyrimidinase like 2	122,7	51,8	**0,58**	0,000111
ARHGEF1	Rho guanine nucleotide exchange factor 1	135,8	54,4	**0,58**	9,10E-05
PLEKHH2	pleckstrin homology, MyTH4 and FERM domain containing H2	23,0	9,4	**0,58**	7,60E-05
ORC6	origin recognition complex subunit 6	7,8	2,7	**0,58**	0,005474
DUT	deoxyuridine triphosphatase	82,2	31,4	**0,58**	9,00E-05
SLC16A2	solute carrier family 16 member 2	19,0	9,2	**0,58**	8,50E-05
MYH10	myosin heavy chain 10	46,3	18,5	**0,58**	3,00E-06
IGSF8	immunoglobulin superfamily member 8	47,3	22,1	**0,58**	9,10E-05
ABCB6	ATP binding cassette subfamily B member 6 (Langereis blood group)	1,8	0,6	**0,58**	0,031015
HOXC-AS2	HOXC cluster antisense RNA 2	0,8	0,4	**0,58**	0,008815
RUSC1-AS1	RUSC1 antisense RNA 1	1,5	0,8	**0,58**	0,008463
ARRB2	arrestin beta 2	5,1	1,9	**0,58**	0,004118
ZNF462	zinc finger protein 462	20,5	8,8	**0,58**	0,001592
PAQR7	progestin and adipoQ receptor family member 7	12,8	5,3	**0,58**	0,001343
TKT	transketolase	139,0	58,2	**0,58**	0,000109
ANAPC15	anaphase promoting complex subunit 15	13,1	5,0	**0,58**	0,001915
DCTN3	dynactin subunit 3	53,9	21,5	**0,58**	1,90E-05
IL31RA	interleukin 31 receptor A	1,5	0,6	**0,58**	0,03708
UNG	uracil DNA glycosylase	31,1	12,2	**0,58**	1,60E-05
BST1	bone marrow stromal cell antigen 1	1,0	0,4	**0,58**	0,033842
ZFP2	ZFP2 zinc finger protein	1,6	0,6	**0,58**	0,012796

TCN2	transcobalamin 2	3,1	1,3	**0,58**	0,003459
CAMKK2	calcium/calmodulin dependent protein kinase kinase 2	36,9	15,5	**0,58**	3,00E-06
PDE1B	phosphodiesterase 1B	16,6	6,4	**0,58**	0,000922
HMGN3	high mobility group nucleosomal binding domain 3	30,9	11,9	**0,58**	0,000292
PRR5	proline rich 5	7,8	3,3	**0,58**	0,005861
USP35	ubiquitin specific peptidase 35	11,6	5,2	**0,58**	2,80E-05
PENK	proenkephalin	160,0	72,5	**0,58**	0,033458
F8	coagulation factor VIII	17,3	7,1	**0,58**	3,70E-05
PCDHGB7	protocadherin gamma subfamily B, 7	17,0	7,2	**0,58**	0,001217
TMEM8B	transmembrane protein 8B	33,7	12,8	**0,58**	0,000493
TRABD2B	TraB domain containing 2B	5,2	2,3	**0,58**	0,001211
STAT5B	signal transducer and activator of transcription 5B	55,0	22,9	**0,58**	1,00E-06
SLFN12	schlafen family member 12	1,2	0,6	**0,58**	0,010688
CLIP3	CAP-Gly domain containing linker protein 3	17,9	8,1	**0,58**	0,000228
ZNF665	zinc finger protein 665	1,9	0,8	**0,58**	0,00337
KLF8	Kruppel like factor 8	4,7	2,2	**0,58**	9,00E-04
PDHB	pyruvate dehydrogenase (lipoamide) beta	44,8	18,7	**0,58**	3,00E-05
ANKRD36B	ankyrin repeat domain 36B	1,4	0,8	**0,58**	0,00434
PCSK1N	proprotein convertase subtilisin/kexin type 1 inhibitor	12,3	5,9	**0,58**	0,027539
LRR1	leucine rich repeat protein 1	9,3	3,4	**0,58**	0,001287
TICRR	TOPBP1 interacting checkpoint and replication regulator	1,5	0,4	**0,58**	0,012437
BATF	basic leucine zipper ATF-like transcription factor	4,4	1,8	**0,58**	0,000885
RHOBTB2	Rho related BTB domain containing 2	30,8	12,3	**0,58**	2,20E-05
DDHD2	DDHD domain containing 2	32,7	13,2	**0,58**	1,20E-05
HNRNPA2B1	heterogeneous nuclear ribonucleoprotein A2/B1	642,1	257,0	**0,58**	0,001255
CERS4	ceramide synthase 4	12,0	5,3	**0,58**	0,001147
MICAL2	microtubule associated monooxygenase, calponin and LIM domain co	55,8	23,9	**0,58**	6,60E-05
ZNF702P	zinc finger protein 702, pseudogene	1,0	0,4	**0,58**	0,047271
SCMH1	sex comb on midleg homolog 1 (Drosophila)	42,5	17,8	**0,58**	2,00E-06
SLC41A3	solute carrier family 41 member 3	88,0	36,7	**0,58**	3,00E-05
EIF2AK1	eukaryotic translation initiation factor 2 alpha kinase 1	113,7	46,5	**0,58**	2,10E-05
DMD	dystrophin	0,9	0,4	**0,58**	0,019282
ARHGEF9	Cdc42 guanine nucleotide exchange factor 9	14,1	5,6	**0,58**	0,000257

ADCK2	aarF domain containing kinase 2	12,1	5,4	**0,58**	0,000597
PROB1	proline rich basic protein 1	4,1	1,6	**0,58**	0,002754
LINC00933	long intergenic non-protein coding RNA 933	0,7	0,3	**0,58**	0,009947
ZNF853	zinc finger protein 853	16,3	7,0	**0,58**	1,20E-05
ADGRA2	adhesion G protein-coupled receptor A2	2,8	1,2	**0,58**	0,006312
RASSF7	Ras association domain family member 7	0,6	0,3	**0,58**	0,028603
MICAL1	microtubule associated monooxygenase, calponin and LIM domain co	34,4	14,8	**0,58**	1,00E-06
SELENOW	selenoprotein W	58,2	23,8	**0,58**	0,000132
RPL4P4	ribosomal protein L4 pseudogene 4	1,1	0,5	**0,58**	0,010055
ARL6IP5	ADP ribosylation factor like GTPase 6 interacting protein 5	328,9	134,7	**0,58**	0,000489
ELOVL1	ELOVL fatty acid elongase 1	108,4	45,3	**0,58**	9,70E-05
C3orf70	chromosome 3 open reading frame 70	12,2	4,8	**0,58**	0,00067
XKR8	XK related 8	20,4	8,9	**0,58**	6,40E-05
RCOR3	REST corepressor 3	32,7	13,6	**0,58**	5,00E-06
CD300C	CD300c molecule	4,8	1,6	**0,58**	0,011206
PLOD1	procollagen-lysine,2-oxoglutarate 5-dioxygenase 1	312,8	136,4	**0,58**	0,000866
C9orf66	chromosome 9 open reading frame 66	1,0	0,4	**0,58**	0,034138
SLC25A27	solute carrier family 25 member 27	3,0	1,3	**0,58**	0,006232
NSD2	nuclear receptor binding SET domain protein 2	65,7	25,5	**0,58**	0
PLPP1	phospholipid phosphatase 1	58,3	21,0	**0,58**	0,002298
MRPL24	mitochondrial ribosomal protein L24	50,4	19,7	**0,58**	0,000165
ICAM3	intercellular adhesion molecule 3	9,7	4,0	**0,58**	0,00184
FOXP4-AS1	FOXP4 antisense RNA 1	0,5	0,2	**0,58**	0,031028
HES1	hes family bHLH transcription factor 1	21,0	8,0	**0,58**	0,000395
AGA	aspartylglucosaminidase	22,1	9,1	**0,58**	0,000246
PABPC1P4	poly(A) binding protein cytoplasmic 1 pseudogene 4	6,3	2,8	**0,58**	0,000147
LOX	lysyl oxidase	193,8	78,2	**0,58**	0,00029
CCDC15	coiled-coil domain containing 15	4,7	1,6	**0,58**	0,002754
FBXO27	F-box protein 27	6,1	2,3	**0,58**	0,003406
PCNT	pericentrin	52,8	22,4	**0,58**	0
IDH3G	isocitrate dehydrogenase 3 (NAD(+)) gamma	36,7	15,1	**0,58**	2,30E-05
PSMC3IP	PSMC3 interacting protein	15,4	5,9	**0,57**	0,000235
RUNX1T1	RUNX1 translocation partner 1	5,2	2,0	**0,57**	0,0064

CMAHP	cytidine monophospho-N-acetylneuraminic acid hydroxylase, pseudog	141,4	56,2	**0,57**	0,000322
MACROD2	MACRO domain containing 2	0,7	0,3	**0,57**	0,018357
SPNS2	sphingolipid transporter 2		2,4	**0,57**	0,008972
MSRB1	methionine sulfoxide reductase B1	28,1	11,8	**0,57**	0,000238
PNMA8B	paraneoplastic Ma antigen family member 8B	9,7	4,0	**0,57**	0,003545
SLITRK4	SLIT and NTRK like family member 4	8,3	3,7	**0,57**	0,002221
LAMA2	laminin subunit alpha 2	4,2	1,6	**0,57**	0,01021
MGMT	O-6-methylguanine-DNA methyltransferase	16,8	6,4	**0,57**	0,001522
PCDHB9	protocadherin beta 9	2,6	1,1	**0,57**	0,001301
VOPP1	vesicular, overexpressed in cancer, prosurvival protein 1	56,7	23,6	**0,57**	1,70E-05
NRN1	neuritin 1	227,0	92,5	**0,57**	0,00025
INPPL1	inositol polyphosphate phosphatase like 1	89,8	38,2	**0,57**	1,00E-06
ZNF90	zinc finger protein 90	1,0	0,4	**0,57**	0,00912
LTF	lactotransferrin	1,9	0,8	**0,57**	0,007532
GMDS	GDP-mannose 4,6-dehydratase	62,5	24,4	**0,57**	0,000113
TM4SF19-AS	TM4SF19 antisense RNA 1	0,5	0,2	**0,57**	0,025856
SUV39H1	suppressor of variegation 3-9 homolog 1	8,7	3,3	**0,57**	5,00E-04
TGFB1I1	transforming growth factor beta 1 induced transcript 1	55,9	22,9	**0,57**	1,40E-05
UBE2Q2P1	ubiquitin conjugating enzyme E2 Q2 pseudogene 1	1,1	0,5	**0,57**	0,008677
SGTB	small glutamine rich tetratricopeptide repeat containing beta	27,3	11,6	**0,57**	2,40E-05
PLBD1	phospholipase B domain containing 1	29,7	13,1	**0,57**	2,80E-05
ZBTB8A	zinc finger and BTB domain containing 8A	3,2	1,3	**0,57**	0,002051
ZNF503	zinc finger protein 503	56,2	0,0	**0,57**	4,20E-05
PPP1R3B	protein phosphatase 1 regulatory subunit 3B	0,9	0,4	**0,57**	0,008356
CPS1	carbamoyl-phosphate synthase 1	8,5	3,5	**0,57**	0,000215
ANXA6	annexin A6	186,8	75,6	**0,57**	0,000128
ZNF681	zinc finger protein 681	2,5	1,1	**0,57**	0,002557
CHD5	chromodomain helicase DNA binding protein 5	1,5	0,5	**0,57**	0,031173
CALR	calreticulin	1945,1	812,4	**0,57**	0,024137
DDIAS	DNA damage induced apoptosis suppressor	4,8	1,7	**0,57**	0,005744
PSMG1	proteasome assembly chaperone 1	37,3	15,0	**0,57**	9,00E-06
ITGA10	integrin subunit alpha 10	594,9	253,9	**0,57**	0,000985
NDRG4	NDRG family member 4	3,5	1,2	**0,57**	0,022656

TMEM143	transmembrane protein 143	6,5	2,5	**0,57**	0,00168
NET1	neuroepithelial cell transforming 1	41,7	16,2	**0,57**	6,30E-05
ACAA2	acetyl-CoA acyltransferase 2	27,0	11,2	**0,57**	3,40E-05
TPK1	thiamin pyrophosphokinase 1	1,5	0,6	**0,57**	0,011104
CAVIN3	caveolae associated protein 3	125,3	51,8	**0,57**	0,001009
RAET1E-AS1	RAET1E antisense RNA 1	0,0	0,4	**0,57**	0,011527
CCDC6	coiled-coil domain containing 6	99,4	40,9	**0,57**	0
LMO4	LIM domain only 4	134,5	57,0	**0,57**	8,00E-05
PARP4	poly(ADP-ribose) polymerase family member 4	53,4	23,0	**0,57**	1,00E-06
ERMP1	endoplasmic reticulum metallopeptidase 1	27,6	11,5	**0,57**	5,00E-06
TUBA3FP	tubulin alpha 3f pseudogene	0,7	0,3	**0,57**	0,024914
GCKR	glucokinase regulator	1,4	0,6	**0,57**	0,008026
GDPD3	glycerophosphodiester phosphodiesterase domain containing 3	1,7	0,7	**0,57**	0,012541
ADAM12	ADAM metallopeptidase domain 12	51,3	19,2	**0,57**	7,00E-06
NR2F6	nuclear receptor subfamily 2 group F member 6	42,8	17,5	**0,57**	4,30E-05
HOXD3	homeobox D3	0,8	0,4	**0,57**	0,048899
ISOC2	isochorismatase domain containing 2	27,4	10,9	**0,57**	0,001222
ZC3H8	zinc finger CCCH-type containing 8	15,3	6,0	**0,57**	0,000275
TRIB2	tribbles pseudokinase 2	45,1	17,2	**0,57**	7,30E-05
TSPO	translocator protein	188,7	78,5	**0,57**	0,000774
PCDHGC5	protocadherin gamma subfamily C, 5	1,9	0,7	**0,57**	0,005043
TMOD2	tropomodulin 2	9,2	4,5	**0,57**	0,000191
TMEM173	transmembrane protein 173	84,6	32,4	**0,57**	4,40E-05
LURAP1	leucine rich adaptor protein 1	6,1	2,4	**0,57**	0,000539
WNK2	WNK lysine deficient protein kinase 2	5,9	2,3	**0,57**	0,001363
LYVE1	lymphatic vessel endothelial hyaluronan receptor 1	24,4	10,1	**0,57**	0,000954
C20orf96	chromosome 20 open reading frame 96	4,1	1,6	**0,57**	0,001509
NOD1	nucleotide binding oligomerization domain containing 1	7,1	2,8	**0,57**	0,001237
SCP2	sterol carrier protein 2	78,5	31,1	**0,57**	9,00E-06
TRAIP	TRAF interacting protein	3,7	1,3	**0,57**	0,00395
CDK5	cyclin dependent kinase 5	8,3	3,3	**0,57**	0,000226
RPARP-AS1	RPARP antisense RNA 1	1,6	0,7	**0,57**	0,007393
C17orf53	chromosome 17 open reading frame 53	2,5	0,9	**0,57**	0,005348

PLEKHG2	pleckstrin homology and RhoGEF domain containing G2	10,5	4,5	**0,57**	8,60E-05
SNCA	synuclein alpha	2,5	0,9	**0,57**	0,020863
SORBS2	sorbin and SH3 domain containing 2	15,7	6,4	**0,57**	5,10E-05
NAALADL1	N-acetylated alpha-linked acidic dipeptidase like 1	10,5	4,2	**0,57**	0,000567
GPSM2	G protein signaling modulator 2	37,9	14,2	**0,57**	1,50E-05
NTN4	netrin 4	529,9	215,7	**0,57**	0,001554
PPP1R9B	protein phosphatase 1 regulatory subunit 9B	54,4	22,2	**0,57**	2,00E-06
SP2-AS1	SP2 antisense RNA 1	4,0	1,6	**0,57**	0,002953
TDRD9	tudor domain containing 9	7,9	3,5	**0,57**	0,008575
HYAL2	hyaluronoglucosaminidase 2	33,7	13,4	**0,57**	0,000188
LGALS1	galectin 1	295,5	127,9	**0,57**	0,000569
C16orf86	chromosome 16 open reading frame 86	2,7	1,1	**0,57**	0,006714
NACAD	NAC alpha domain containing	4,7	2,2	**0,57**	0,000101
SYNE2	spectrin repeat containing nuclear envelope protein 2	14,4	6,2	**0,57**	0,00503
MPP1	membrane palmitoylated protein 1	19,7	7,6	**0,57**	0,000131
DSTN	destrin, actin depolymerizing factor	608,4	236,8	**0,57**	0,002584
PEBP1	phosphatidylethanolamine binding protein 1	207,0	82,0	**0,57**	0,000404
WISP3	WNT1 inducible signaling pathway protein 3		29,9	**0,56**	8,20E-05
TBC1D1	TBC1 domain family member 1	151,6	61,9	**0,56**	4,00E-06
PPP1R36	protein phosphatase 1 regulatory subunit 36	1,3	0,4	**0,56**	0,016349
UBE3D	ubiquitin protein ligase E3D	2,5	1,1	**0,56**	0,003643
PLEKHG4	pleckstrin homology and RhoGEF domain containing G4	15,3	5,4	**0,56**	0,000143
FAM136A	family with sequence similarity 136 member A	21,9	8,6	**0,56**	6,10E-05
SPIN2B	spindlin family member 2B	1,8	0,7	**0,56**	0,020336
SPATA6	spermatogenesis associated 6	8,3	3,5	**0,56**	9,80E-05
C9orf24	chromosome 9 open reading frame 24	0,6	0,3	**0,56**	0,040048
MYCBPAP	MYCBP associated protein	1,4	0,5	**0,56**	0,014619
RASGEF1A	RasGEF domain family member 1A	2,3	0,8	**0,56**	0,007349
CEP126	centrosomal protein 126	23,5	9,0	**0,56**	2,20E-05
CENPV	centromere protein V	1,4	0,5	**0,56**	0,020217
RMI1	RecQ mediated genome instability 1	12,1	4,7	**0,56**	0,000116
GMPR	guanosine monophosphate reductase	2,1	0,8	**0,56**	0,012733
WWOX	WW domain containing oxidoreductase	10,0	3,7	**0,56**	0,00147

PPIAP22	peptidylprolyl isomerase A pseudogene 22	3,7	1,6	**0,56**	0,002743
CROCCP3	ciliary rootlet coiled-coil, rootletin pseudogene 3	1,9	0,9	**0,56**	0,000961
KIAA1644	KIAA1644			**0,56**	0,011466
PPFIA4	PTPRF interacting protein alpha 4	31,8	13,4	**0,56**	0,000454
VAV2	vav guanine nucleotide exchange factor 2	50,2	19,1	**0,56**	5,00E-06
CHMP4C	charged multivesicular body protein 4C	8,3	2,9	**0,56**	0,000776
CTTNBP2	cortactin binding protein 2	3,5	1,3	**0,56**	0,001841
RUSC1	RUN and SH3 domain containing 1	28,1	11,8	**0,56**	4,30E-05
OPHN1	oligophrenin 1	15,8	6,0	**0,56**	0,000208
TIMELESS	timeless circadian clock	32,7	11,8	**0,56**	1,30E-05
GLI2	GLI family zinc finger 2	8,3	3,1	**0,56**	0,000422
COPS6	COP9 signalosome subunit 6	86,6	33,6	**0,56**	1,20E-05
KIF22	kinesin family member 22	33,5	11,8	**0,56**	1,70E-05
TEX9	testis expressed 9	10,4	3,8	**0,56**	0,001615
PXDNL	peroxidasin like	1,9	0,8	**0,56**	0,008045
IFT122	intraflagellar transport 122	28,9	11,5	**0,56**	2,40E-05
ARHGAP29	Rho GTPase activating protein 29	47,9	19,7	**0,56**	2,00E-06
TMEM100	transmembrane protein 100	2,3	0,8	**0,56**	0,014354
BCAS4	breast carcinoma amplified sequence 4	6,5	2,6	**0,56**	0,003709
SPAG8	sperm associated antigen 8	1,0	0,3	**0,56**	0,028293
GNB3	G protein subunit beta 3	2,5	1,0	**0,56**	0,003825
FAM120A	family with sequence similarity 120A	181,6	71,8	**0,56**	1,00E-05
TGFB1	transforming growth factor beta 1		120,7	**0,56**	0,000246
CREB5	cAMP responsive element binding protein 5	56,6	22,9	**0,56**	0
WEE1	WEE1 G2 checkpoint kinase	21,7	8,9	**0,56**	1,50E-05
PRR5L	proline rich 5 like	4,8	1,8	**0,56**	0,004244
EMP2	epithelial membrane protein 2	216,5	88,1	**0,56**	3,30E-05
CUEDC2	CUE domain containing 2	39,6	16,3	**0,56**	3,20E-05
CDH13	cadherin 13	21,5	8,5	**0,56**	1,10E-05
ANKS1B	ankyrin repeat and sterile alpha motif domain containing 1B	2,5	1,1	**0,56**	0,0035
IQCH-AS1	IQCH antisense RNA 1	3,0	1,2	**0,56**	0,003938
CCDC102B	coiled-coil domain containing 102B	0,9	0,3	**0,56**	0,017048
ZIC4	Zic family member 4	0,8	0,3	**0,56**	0,04268

SUCLG2	succinate-CoA ligase GDP-forming beta subunit	44,4	17,2	**0,56**	6,00E-06
FAM8A1	family with sequence similarity 8 member A1	51,8	20,4	**0,56**	1,00E-06
HSP90AA2P	heat shock protein 90 alpha family class A member 2, pseudogene	0,9	0,3	**0,56**	0,03144
ATP5A1	ATP synthase, H+ transporting, mitochondrial F1 complex, alpha subunit 1, cardiac muscle			**0,56**	8,30E-05
PKDCC	protein kinase domain containing, cytoplasmic	9,9	3,6	**0,56**	0,000258
BZW1	basic leucine zipper and W2 domains 1	1053,6	423,1	**0,56**	0,002551
TGIF2	TGFB induced factor homeobox 2	6,8	2,6	**0,56**	0,000658
TCTA	T-cell leukemia translocation altered	66,1	25,7	**0,56**	1,20E-05
LSR	lipolysis stimulated lipoprotein receptor	3,1	1,3	**0,56**	0,006909
SPIN4	spindlin family member 4	6,9	2,5	**0,56**	0,00028
TRAM1L1	translocation associated membrane protein 1-like 1	2,5	0,9	**0,56**	0,004307
MT-TQ	mitochondrially encoded tRNA glutamine	0,9	0,3	**0,56**	0,026892
FAM216A	family with sequence similarity 216 member A	7,2	3,0	**0,56**	0,000159
ZFP36	ZFP36 ring finger protein	60,1	25,7	**0,56**	1,00E-06
NKD1	naked cuticle homolog 1	12,3	4,7	**0,56**	0,003736
KMT5A	lysine methyltransferase 5A	39,4	15,0	**0,56**	6,00E-06
PQLC3	PQ loop repeat containing 3	22,1	8,6	**0,55**	0,000565
FRG1BP	FSHD region gene 1 family member B, pseudogene	2,9	1,2	**0,55**	0,004433
GSN	gelsolin	656,9	276,8	**0,55**	0,001748
LIN7B	lin-7 homolog B, crumbs cell polarity complex component	3,9	1,4	**0,55**	0,002319
COL8A2	collagen type VIII alpha 2 chain	201,7	77,9	**0,55**	0,000108
DAGLA	diacylglycerol lipase alpha	3,1	1,1	**0,55**	0,001741
ZCCHC24	zinc finger CCHC-type containing 24	73,5	30,3	**0,55**	8,00E-06
SKIDA1	SKI/DACH domain containing 1	1,1	0,5	**0,55**	0,012651
CYTL1	cytokine like 1	313,3	121,9	**0,55**	0,005312
PWAR5	Prader Willi/Angelman region RNA 5	1,5	0,8	**0,55**	0,02956
XYLT2	xylosyltransferase 2	27,4	10,6	**0,55**	3,00E-06
CD151	CD151 molecule (Raph blood group)	596,5	241,1	**0,55**	0,001914
OSBPL9	oxysterol binding protein like 9	72,8	28,6	**0,55**	1,00E-06
CAVIN2	caveolae associated protein 2	0,5	0,2	**0,55**	0,026857
GNAL	G protein subunit alpha L	9,0	3,6	**0,55**	0,000577
TLE2	transducin like enhancer of split 2	4,3	1,7	**0,55**	0,000815
ZNF724	zinc finger protein 724	0,6	0,2	**0,55**	0,018774

LAG3	lymphocyte activating 3	50,7	21,0	**0,55**	0,000482
ZIC1	Zic family member 1	2,6	1,0	**0,55**	0,001433
PRDM6	PR/SET domain 6	3,9	1,6	**0,55**	0,010214
NUDT1	nudix hydrolase 1	12,8	4,6	**0,55**	0,00197
RGS19	regulator of G protein signaling 19	8,6	3,5	**0,55**	0,000364
C10orf10	chromosome 10 open reading frame 10			**0,55**	0,000585
MIR621	microRNA 621	0,5	0,2	**0,55**	0,025389
EHHADH	enoyl-CoA hydratase and 3-hydroxyacyl CoA dehydrogenase	10,4	4,3	**0,55**	0,000442
BMPR1A	bone morphogenetic protein receptor type 1A	29,4	11,8	**0,55**	1,00E-06
TCEAL4	transcription elongation factor A like 4	89,1	34,8	**0,55**	6,00E-06
SH3RF1	SH3 domain containing ring finger 1	32,1	12,0	**0,55**	0
NECAB3	N-terminal EF-hand calcium binding protein 3	6,9	2,8	**0,55**	0,000688
PGM1	phosphoglucomutase 1	179,2	71,8	**0,55**	8,70E-05
PLIN3	perilipin 3	205,8	82,7	**0,55**	0,000379
TRIM47	tripartite motif containing 47		30,9	**0,55**	3,00E-06
ANO7	anoctamin 7	1,7	0,7	**0,55**	0,001909
CDKL1	cyclin dependent kinase like 1	19,1	6,6	**0,55**	0,000144
DIS3L	DIS3 like exosome 3'-5' exoribonuclease	27,5	11,2	**0,55**	1,20E-05
DTNB	dystrobrevin beta	7,0	2,9	**0,55**	0,000406
SLC25A10	solute carrier family 25 member 10	2,6	0,9	**0,55**	0,006606
PYCR1	pyrroline-5-carboxylate reductase 1	117,2	44,2	**0,55**	0,000315
PXMP4	peroxisomal membrane protein 4	10,0	3,8	**0,55**	0,000121
C9orf3	chromosome 9 open reading frame 3	244,2	98,0	**0,55**	0,000315
MAGEL2	MAGE family member L2	2,8	1,1	**0,55**	0,00237
PRADC1	protease associated domain containing 1	19,6	7,1	**0,55**	0,001769
TCEAL3	transcription elongation factor A like 3	38,9	14,3	**0,55**	7,00E-05
ELMO1	engulfment and cell motility 1	26,6	10,4	**0,55**	8,00E-05
RHBDL3	rhomboid like 3	0,7	0,3	**0,55**	0,032039
DNAH14	dynein axonemal heavy chain 14	4,2	1,7	**0,55**	0,000621
C17orf107	chromosome 17 open reading frame 107	32,8	11,7	**0,55**	0,000567
IDH2	isocitrate dehydrogenase (NADP(+)) 2, mitochondrial	42,7	15,7	**0,55**	0,000476
FOXP4	forkhead box P4	117,3	46,8	**0,55**	1,00E-06
FBN1	fibrillin 1	229,4	94,2	**0,55**	2,50E-05

NME2	NME/NM23 nucleoside diphosphate kinase 2	1,3	0,5	**0,55**	0,01074
C4orf48	chromosome 4 open reading frame 48	20,2	7,8	**0,55**	0,000491
BTBD11	BTB domain containing 11	17,6	7,2	**0,55**	0,000345
MEF2A	myocyte enhancer factor 2A	191,5	71,4	**0,55**	8,00E-06
CDON	cell adhesion associated, oncogene regulated	223,7	87,8	**0,55**	3,00E-05
PI4K2B	phosphatidylinositol 4-kinase type 2 beta	14,5	5,5	**0,55**	8,60E-05
PKDREJ	polycystin family receptor for egg jelly	0,6	0,2	**0,55**	0,028142
CYP11A1	cytochrome P450 family 11 subfamily A member 1	2,2	0,8	**0,55**	0,002143
CPPED1	calcineurin like phosphoesterase domain containing 1	50,2	19,7	**0,55**	2,00E-06
KAT2A	lysine acetyltransferase 2A	17,8	7,0	**0,55**	0,000103
HCAR1	hydroxycarboxylic acid receptor 1	1,1	0,5	**0,55**	0,004202
HYI	hydroxypyruvate isomerase (putative)	25,1	9,1	**0,55**	0,000231
MAP1A	microtubule associated protein 1A	36,4	14,5	**0,55**	1,50E-05
TTLL7	tubulin tyrosine ligase like 7	18,9	7,3	**0,55**	1,20E-05
ARHGEF17	Rho guanine nucleotide exchange factor 17	108,5	41,9	**0,55**	2,00E-06
FAM228B	family with sequence similarity 228 member B	6,8	2,9	**0,55**	0,000127
TIMP2	TIMP metallopeptidase inhibitor 2	942,1	379,9	**0,55**	0,001901
CCDC74A	coiled-coil domain containing 74A	2,9	1,1	**0,55**	0,007559
ZNF575	zinc finger protein 575	2,8	1,1	**0,54**	0,003528
MAGI3	membrane associated guanylate kinase, WW and PDZ domain contair	7,6	2,9	**0,54**	5,30E-05
SORT1	sortilin 1	130,4	49,9	**0,54**	4,40E-05
TTF2	transcription termination factor 2	11,7	4,2	**0,54**	3,60E-05
CRTAP	cartilage associated protein	433,6	173,9	**0,54**	0,000238
PARP9	poly(ADP-ribose) polymerase family member 9	28,0	11,3	**0,54**	0
LINC01114	long intergenic non-protein coding RNA 1114	1,2	0,5	**0,54**	0,006388
SLC2A10	solute carrier family 2 member 10	43,8	18,3	**0,54**	6,00E-06
GALNT2	polypeptide N-acetylgalactosaminyltransferase 2	973,4	389,0	**0,54**	0,001829
DSC3	desmocollin 3	29,4	10,7	**0,54**	0,000982
SGCB	sarcoglycan beta	131,7	52,1	**0,54**	1,00E-06
MVB12B	multivesicular body subunit 12B	4,1	1,5	**0,54**	0,000806
LINC01301	long intergenic non-protein coding RNA 1301	1,3	0,5	**0,54**	0,003602
EEPD1	endonuclease/exonuclease/phosphatase family domain containing 1	4,2	1,6	**0,54**	0,002656
DLC1	DLC1 Rho GTPase activating protein	23,4	9,7	**0,54**	4,00E-06

ENPEP	glutamyl aminopeptidase	6,5	2,0	**0,54**	0,004414
KREMEN1	kringle containing transmembrane protein 1	24,6	11,6	**0,54**	0,000142
MCM8	minichromosome maintenance 8 homologous recombination repair fa	15,2	5,0	**0,54**	1,20E-05
TNC	tenascin C	1690,6	623,9	**0,54**	0,004043
DUSP15	dual specificity phosphatase 15	3,7	1,3	**0,54**	0,006157
METRN	meteorin, glial cell differentiation regulator	63,0	23,2	**0,54**	0,000841
C9orf142	chromosome 9 open reading frame 142			**0,54**	0,000241
TUBBP1	tubulin beta pseudogene 1	0,6	0,2	**0,54**	0,013345
FBXO2	F-box protein 2	106,9	38,9	**0,54**	0,00038
TFEB	transcription factor EB	10,7	3,8	**0,54**	0,000577
BEND5	BEN domain containing 5	1,9	0,8	**0,54**	0,008068
CAPS	calcyphosine	58,4	21,7	**0,54**	0,000117
STX2	syntaxin 2	32,6	12,9	**0,54**	1,00E-06
KLF17	Kruppel like factor 17	0,5	0,2	**0,54**	0,0204
PLS3	plastin 3	213,2	81,2	**0,54**	1,90E-05
MZT2B	mitotic spindle organizing protein 2B	67,8	26,9	**0,54**	9,50E-05
KDELC1	KDEL motif containing 1	11,2	4,5	**0,54**	5,70E-05
PTK6	protein tyrosine kinase 6	1,1	0,4	**0,54**	0,025933
KIAA1549	KIAA1549	28,2	10,6	**0,54**	2,00E-06
NCS1	neuronal calcium sensor 1	26,1	9,5	**0,54**	0,000113
LHPP	phospholysine phosphohistidine inorganic pyrophosphate phosphatas	13,1	4,7	**0,54**	0,000369
ITGB5	integrin subunit beta 5	2234,9	849,2	**0,54**	0,005983
CCDC74B	coiled-coil domain containing 74B	1,2	0,5	**0,54**	0,012418
ISPD	isoprenoid synthase domain containing	1,6	0,6	**0,54**	0,001943
ARL4D	ADP ribosylation factor like GTPase 4D	23,2	9,1	**0,54**	3,90E-05
ADCY5	adenylate cyclase 5	4,9	1,6	**0,54**	0,00496
TSKU	tsukushi, small leucine rich proteoglycan	37,4	14,6	**0,54**	9,00E-06
STEAP1B	STEAP family member 1B	3,5	1,4	**0,54**	0,004804
HOTAIR	HOX transcript antisense RNA	0,6	0,2	**0,54**	0,039757
CARM1	coactivator associated arginine methyltransferase 1	62,6	23,4	**0,54**	1,00E-06
CD302	CD302 molecule	26,3	10,3	**0,54**	5,50E-05
CTSZ	cathepsin Z	181,0	77,4	**0,54**	8,20E-05
MPST	mercaptopyruvate sulfurtransferase	27,8	10,4	**0,54**	0,000121

CD109	CD109 molecule	402,2	155,8	**0,54**	9,40E-05
ZNF608	zinc finger protein 608	21,1	8,6	**0,54**	5,60E-05
PCDHB4	protocadherin beta 4	16,9	6,6	**0,54**	0,000135
CCDC136	coiled-coil domain containing 136	3,0	1,3	**0,54**	0,000794
TRIQK	triple QxxK/R motif containing	41,0	16,4	**0,54**	3,00E-06
HYLS1	HYLS1, centriolar and ciliogenesis associated	7,8	2,8	**0,54**	0,000368
SLC25A15	solute carrier family 25 member 15	6,6	2,3	**0,54**	0,001001
ZNF710-AS1	ZNF710 antisense RNA 1	1,7	0,7	**0,54**	0,007502
SRM	spermidine synthase	129,9	49,9	**0,54**	0,000136
CORO2A	coronin 2A	0,6	0,2	**0,54**	0,025902
MELTF	melanotransferrin	773,9	312,2	**0,54**	0,00122
FGF13	fibroblast growth factor 13	4,4	2,0	**0,54**	0,001664
LDHB	lactate dehydrogenase B	140,0	51,0	**0,54**	4,10E-05
FAM114A1	family with sequence similarity 114 member A1	227,2	88,5	**0,54**	3,80E-05
AGL	amylo-alpha-1, 6-glucosidase, 4-alpha-glucanotransferase	15,7	6,2	**0,54**	1,70E-05
ATAD5	ATPase family, AAA domain containing 5	3,6	1,2	**0,54**	0,00083
SLC12A9	solute carrier family 12 member 9	25,2	9,9	**0,54**	2,50E-05
KNSTRN	kinetochore localized astrin/SPAG5 binding protein	11,9	3,8	**0,54**	0,00048
GAS2L1	growth arrest specific 2 like 1	26,5	9,4	**0,54**	3,40E-05
TBC1D4	TBC1 domain family member 4	31,3	12,0	**0,54**	0
ACAT1	acetyl-CoA acetyltransferase 1	43,0	15,6	**0,54**	5,00E-06
CES4A	carboxylesterase 4A	1,5	0,6	**0,54**	0,007102
ADCY2	adenylate cyclase 2	38,1	13,6	**0,54**	1,00E-06
ANTXR1	anthrax toxin receptor 1	116,4	43,6	**0,54**	2,00E-06
MOXD1	monooxygenase DBH like 1	324,3	120,6	**0,54**	0,000162
HNRNPA1P4	heterogeneous nuclear ribonucleoprotein A1 pseudogene 48	2,8	1,1	**0,54**	0,010655
IL1RL2	interleukin 1 receptor like 2	5,9	2,1	**0,54**	0,000326
SNX32	sorting nexin 32	0,7	0,3	**0,54**	0,01354
FAM72B	family with sequence similarity 72 member B	0,6	0,2	**0,54**	0,02741
ALAD	aminolevulinate dehydratase	38,5	13,8	**0,53**	1,10E-05
LMNA	lamin A/C	752,5	274,8	**0,53**	0,000481
PDE6B	phosphodiesterase 6B	0,7	0,2	**0,53**	0,011355
NOL4L	nucleolar protein 4 like	12,6	4,8	**0,53**	1,10E-05

DSN1	DSN1 homolog, MIS12 kinetochore complex component	13,6	4,6	**0,53**	9,10E-05
CASP6	caspase 6	6,3	2,4	**0,53**	0,000161
RNF128	ring finger protein 128, E3 ubiquitin protein ligase	3,0	1,0	**0,53**	0,02705
LOXL1-AS1	LOXL1 antisense RNA 1	6,5	2,4	**0,53**	0,000612
NHP2	NHP2 ribonucleoprotein	56,8	21,1	**0,53**	0,000123
RBM3	RNA binding motif (RNP1, RRM) protein 3	197,9	71,6	**0,53**	8,20E-05
FKBP8	FK506 binding protein 8	246,2	94,8	**0,53**	0,000264
HEATR3	HEAT repeat containing 3	9,3	3,4	**0,53**	0,000425
FAM20C	FAM20C, golgi associated secretory pathway kinase	340,2	135,4	**0,53**	0,000497
SH3PXD2A	SH3 and PX domains 2A	43,2	14,9	**0,53**	3,00E-06
ZNF793-AS1	ZNF793 antisense RNA 1 (head to head)	1,1	0,4	**0,53**	0,008776
MAGEE1	MAGE family member E1	7,1	2,6	**0,53**	0,000261
SCNN1A	sodium channel epithelial 1 alpha subunit	6,8	2,4	**0,53**	0,006379
ZNF835	zinc finger protein 835	0,7	0,3	**0,53**	0,007273
CHD3	chromodomain helicase DNA binding protein 3	62,3	24,6	**0,53**	0
CENPW	centromere protein W	6,6	2,0	**0,53**	0,001658
LINGO1	leucine rich repeat and Ig domain containing 1	5,2	1,9	**0,53**	0,002083
VEGFB	vascular endothelial growth factor B	38,4	14,3	**0,53**	0,000114
CLVS2	clavesin 2	9,9	3,5	**0,53**	0,000756
STK38L	serine/threonine kinase 38 like	128,1	48,4	**0,53**	1,00E-06
ABCB10	ATP binding cassette subfamily B member 10	13,1	5,0	**0,53**	5,00E-06
CNTLN	centlein	15,9	5,9	**0,53**	2,00E-06
NTN1	netrin 1	421,9	162,2	**0,53**	4,90E-05
CYTH3	cytohesin 3	45,4	16,2	**0,53**	4,00E-06
ACCS	1-aminocyclopropane-1-carboxylate synthase homolog (inactive)	5,6	2,2	**0,53**	0,000145
PLEKHH3	pleckstrin homology, MyTH4 and FERM domain containing H3	23,1	8,4	**0,53**	1,10E-05
TRPC1	transient receptor potential cation channel subfamily C member 1	17,4	6,6	**0,53**	1,60E-05
TMTC4	transmembrane and tetratricopeptide repeat containing 4	5,1	2,2	**0,53**	6,20E-05
CERK	ceramide kinase	139,4	51,1	**0,53**	3,00E-06
ZDHHC12	zinc finger DHHC-type containing 12	11,7	4,6	**0,53**	0,000114
PLCB3	phospholipase C beta 3	26,5	10,0	**0,53**	2,50E-05
FAM131A	family with sequence similarity 131 member A	10,9	4,2	**0,53**	1,90E-05
ATP9A	ATPase phospholipid transporting 9A (putative)	52,9	21,2	**0,53**	0

TICAM2	toll like receptor adaptor molecule 2	1,0	0,4	**0,53**	0,008411
GYPE	glycophorin E (MNS blood group)	0,9	0,3	**0,53**	0,006038
NAT14	N-acetyltransferase 14 (putative)	6,4	2,2	**0,53**	0,00124
NME1	NME/NM23 nucleoside diphosphate kinase 1	30,4	11,0	**0,53**	0,000513
THEMIS2	thymocyte selection associated family member 2	1,5	0,5	**0,53**	0,006935
GSC	goosecoid homeobox	1,5	0,7	**0,53**	0,0038
HOXD10	homeobox D10	1,4	0,5	**0,53**	0,031572
MKL2	MKL1/myocardin like 2			**0,53**	5,00E-06
MRVI1	murine retrovirus integration site 1 homolog	0,6	0,2	**0,53**	0,012872
RECK	reversion inducing cysteine rich protein with kazal motifs	65,6	24,1	**0,53**	3,00E-06
SPATA20	spermatogenesis associated 20	45,7	16,7	**0,53**	3,00E-06
TRPS1	transcriptional repressor GATA binding 1	177,7	67,8	**0,53**	1,00E-06
TUBA4A	tubulin alpha 4a	30,1	10,0	**0,53**	2,50E-05
CD99L2	CD99 molecule like 2	141,2	53,7	**0,53**	7,00E-06
SMIM10L2A	small integral membrane protein 10 like 2A	3,3	1,2	**0,53**	0,002661
TGFB2-AS1	TGFB2 antisense RNA 1 (head to head)	0,6	0,2	**0,53**	0,011346
LINC00968	long intergenic non-protein coding RNA 968	1,4	0,7	**0,53**	0,013666
COL5A3	collagen type V alpha 3 chain	6,3	2,3	**0,53**	0,006359
TCEAL8	transcription elongation factor A like 8	58,7	22,1	**0,53**	3,00E-06
TMEM105	transmembrane protein 105	0,5	0,2	**0,53**	0,046481
PLP2	proteolipid protein 2	271,6	101,1	**0,53**	3,00E-05
DIP2C	disco interacting protein 2 homolog C	63,1	23,1	**0,53**	0
TMEM160	transmembrane protein 160	10,6	4,1	**0,53**	0,000727
BLMH	bleomycin hydrolase	19,0	7,2	**0,53**	5,00E-06
MYO1C	myosin IC	349,6	133,3	**0,53**	1,10E-05
KIF1C	kinesin family member 1C	158,2	59,6	**0,53**	3,00E-06
RAB42	RAB42, member RAS oncogene family	2,5	1,0	**0,53**	0,002787
PTPA	protein phosphatase 2 phosphatase activator	195,7	68,9	**0,53**	7,80E-05
STAG3	stromal antigen 3	1,1	0,4	**0,53**	0,002332
FGFRL1	fibroblast growth factor receptor-like 1	885,7	324,1	**0,53**	0,00093
CCNB1IP1	cyclin B1 interacting protein 1	18,0	6,7	**0,52**	3,40E-05
PCDHB10	protocadherin beta 10	5,5	2,2	**0,52**	9,20E-05
ARL2	ADP ribosylation factor like GTPase 2	36,0	13,1	**0,52**	1,90E-05

GNAS	GNAS complex locus	841,1	319,4	**0,52**	0,001057
C18orf54	chromosome 18 open reading frame 54	8,5	3,0	**0,52**	1,60E-05
PRELID3A	PRELI domain containing 3A	4,0	1,5	**0,52**	0,000488
FANCB	Fanconi anemia complementation group B	1,2	0,4	**0,52**	0,009659
PUS10	pseudouridylate synthase 10	22,6	7,8	**0,52**	4,50E-05
RPS6KA4	ribosomal protein S6 kinase A4	40,7	14,6	**0,52**	1,20E-05
KIFAP3	kinesin associated protein 3	64,2	23,8	**0,52**	0
SCART1	scavenger receptor family member expressed on T-cells 1	1,5	0,6	**0,52**	0,00406
PCSK6	proprotein convertase subtilisin/kexin type 6	4,9	1,7	**0,52**	0,001312
PWWP2B	PWWP domain containing 2B	11,5	4,2	**0,52**	9,30E-05
NNT	nicotinamide nucleotide transhydrogenase	33,0	11,9	**0,52**	0
DLEU2	deleted in lymphocytic leukemia 2 (non-protein coding)	4,8	1,7	**0,52**	0,000228
ZNHIT6	zinc finger HIT-type containing 6	56,8	20,9	**0,52**	0
SETMAR	SET domain and mariner transposase fusion gene	8,9	3,2	**0,52**	2,10E-05
PLD2	phospholipase D2	8,6	3,3	**0,52**	0,000103
APOBEC3G	apolipoprotein B mRNA editing enzyme catalytic subunit 3G	3,4	1,4	**0,52**	0,00237
RASA4CP	RAS p21 protein activator 4C, pseudogene	0,6	0,3	**0,52**	0,043712
SLC9A3R2	SLC9A3 regulator 2	167,9	64,0	**0,52**	4,70E-05
ANKRD50	ankyrin repeat domain 50	81,1	33,1	**0,52**	0
DBNL	drebrin like	129,4	47,4	**0,52**	4,00E-06
STX10	syntaxin 10	24,2	9,2	**0,52**	0,000313
SMC2	structural maintenance of chromosomes 2	45,5	16,2	**0,52**	0
NFIB	nuclear factor I B	50,2	18,4	**0,52**	0
MUM1	melanoma associated antigen (mutated) 1		16,6	**0,52**	1,00E-06
BTBD6	BTB domain containing 6	77,9	28,2	**0,52**	2,10E-05
RNF217	ring finger protein 217	56,7	20,5	**0,52**	2,00E-06
SLC25A1	solute carrier family 25 member 1	69,3	27,3	**0,52**	0,000242
IRS1	insulin receptor substrate 1	75,5	28,8	**0,52**	4,00E-06
P3H4	prolyl 3-hydroxylase family member 4 (non-enzymatic)	148,5	54,6	**0,52**	5,70E-05
CHST14	carbohydrate sulfotransferase 14	44,7	16,4	**0,52**	1,00E-06
CHST10	carbohydrate sulfotransferase 10	26,6	9,6	**0,52**	6,00E-06
MFAP3L	microfibrillar associated protein 3 like	37,1	13,6	**0,52**	3,40E-05
PLCG1	phospholipase C gamma 1	69,3	26,1	**0,52**	0

DEPDC4	DEP domain containing 4	0,5	0,2	**0,52**	0,013723
PTPN3	protein tyrosine phosphatase, non-receptor type 3	19,2	6,8	**0,52**	1,00E-06
FAM92A	family with sequence similarity 92 member A	18,1	6,4	**0,52**	4,60E-05
AOC2	amine oxidase, copper containing 2	165,3	66,6	**0,52**	3,40E-05
MAZ	MYC associated zinc finger protein	133,2	46,3	**0,52**	2,90E-05
TTC3P1	tetratricopeptide repeat domain 3 pseudogene 1	9,1	3,5	**0,52**	9,00E-06
CFI	complement factor I	2,0	0,7	**0,52**	0,01049
MCTS2P	malignant T-cell amplified sequence 2, pseudogene	0,6	0,3	**0,52**	0,017386
ERCC2	ERCC excision repair 2, TFIIH core complex helicase subunit	16,7	6,2	**0,52**	0,000104
DLX6	distal-less homeobox 6	7,6	2,6	**0,52**	0,000704
RAVER2	ribonucleoprotein, PTB binding 2	7,1	2,5	**0,52**	0,000123
TEAD4	TEA domain transcription factor 4	24,2	9,1	**0,52**	1,30E-05
KRT8	keratin 8	32,1	10,7	**0,52**	0,000176
CD320	CD320 molecule	68,8	27,0	**0,52**	0,000111
BOLA3-AS1	BOLA3 antisense RNA 1 (head to head)	4,7	1,7	**0,52**	0,000162
TMEM196	transmembrane protein 196	3,6	1,4	**0,52**	0,007578
JAKMIP3	Janus kinase and microtubule interacting protein 3	0,6	0,2	**0,52**	0,020183
SMYD3	SET and MYND domain containing 3	7,1	2,3	**0,52**	0,002106
UBL4A	ubiquitin like 4A	49,9	17,8	**0,52**	1,00E-06
MT-TT	mitochondrially encoded tRNA threonine	0,6	0,2	**0,52**	0,040132
FGF14	fibroblast growth factor 14	1,6	0,5	**0,52**	0,016339
RILPL1	Rab interacting lysosomal protein like 1	24,0	8,8	**0,52**	3,00E-06
TTC3	tetratricopeptide repeat domain 3	250,7	90,6	**0,51**	6,00E-06
SAMHD1	SAM and HD domain containing deoxynucleoside triphosphate tripho:	6,3	2,4	**0,51**	0,00011
CHAF1A	chromatin assembly factor 1 subunit A	16,1	5,1	**0,51**	1,40E-05
MXD3	MAX dimerization protein 3	9,0	2,7	**0,51**	0,00126
PDGFRL	platelet derived growth factor receptor like	59,3	20,3	**0,51**	4,60E-05
PLXNA3	plexin A3	96,1	37,6	**0,51**	2,00E-06
DCTPP1	dCTP pyrophosphatase 1	25,5	9,2	**0,51**	2,10E-05
SLC6A16	solute carrier family 6 member 16	1,1	0,4	**0,51**	0,004764
CRAT	carnitine O-acetyltransferase	60,6	22,3	**0,51**	0
FKBP10	FK506 binding protein 10	659,9	255,6	**0,51**	0,000389
SNAI1	snail family transcriptional repressor 1	31,9	10,2	**0,51**	0,000892

PRSS23	protease, serine 23	581,7	202,6	**0,51**	0,000795
SMC4	structural maintenance of chromosomes 4	79,0	24,4	**0,51**	0
HOXA13	homeobox A13	10,7	3,8	**0,51**	6,50E-05
DLG4	discs large MAGUK scaffold protein 4	14,0	5,0	**0,51**	1,00E-06
LPCAT4	lysophosphatidylcholine acyltransferase 4	13,8	4,7	**0,51**	0,000501
LMTK3	lemur tyrosine kinase 3		0,2	**0,51**	0,014873
HFE	hemochromatosis	18,1	6,3	**0,51**	1,20E-05
PRRX1	paired related homeobox 1	369,5	135,6	**0,51**	2,60E-05
SMO	smoothened, frizzled class receptor	61,0	20,7	**0,51**	5,00E-06
DHCR24	24-dehydrocholesterol reductase	190,9	65,4	**0,51**	0,001673
RPS6KL1	ribosomal protein S6 kinase like 1	0,9	0,4	**0,51**	0,003071
KIAA1211	KIAA1211	9,9	3,1	**0,51**	0,001357
MINDY1	MINDY lysine 48 deubiquitinase 1	19,4	7,0	**0,51**	1,00E-06
POLD1	DNA polymerase delta 1, catalytic subunit	19,5	6,8	**0,51**	4,00E-06
PMEL	premelanosome protein	1,3	0,6	**0,51**	0,001376
LPIN3	lipin 3	14,5	5,1	**0,51**	1,00E-06
NPDC1	neural proliferation, differentiation and control 1	57,3	21,8	**0,51**	1,10E-05
AP2S1	adaptor related protein complex 2 sigma 1 subunit	70,8	25,7	**0,51**	2,60E-05
ITPKB	inositol-trisphosphate 3-kinase B	13,7	4,6	**0,51**	1,00E-06
RGS12	regulator of G protein signaling 12	16,7	6,1	**0,51**	5,00E-06
CCDC78	coiled-coil domain containing 78	0,8	0,2	**0,51**	0,018051
HERC3	HECT and RLD domain containing E3 ubiquitin protein ligase 3	39,4	14,1	**0,51**	0
ARHGEF35	Rho guanine nucleotide exchange factor 35	1,1	0,4	**0,51**	0,028762
DTWD1	DTW domain containing 1	44,4	16,5	**0,51**	7,00E-06
NNAT	neuronatin	1,1	0,3	**0,51**	0,035177
SMARCE1	SWI/SNF related, matrix associated, actin dependent regulator of chrc	7,5	2,8	**0,51**	1,30E-05
ACTBP2	actin, beta pseudogene 2	0,7	0,3	**0,51**	0,008356
PDLIM5	PDZ and LIM domain 5	478,6	175,4	**0,51**	1,30E-05
PCDHGA9	protocadherin gamma subfamily A, 9	5,8	2,1	**0,51**	2,90E-05
LDLRAD3	low density lipoprotein receptor class A domain containing 3	88,9	31,5	**0,51**	7,00E-06
KCNK2	potassium two pore domain channel subfamily K member 2	4,6	2,0	**0,51**	3,60E-05
LIMA1	LIM domain and actin binding 1	165,5	56,7	**0,51**	8,00E-06
ZNF503-AS1	ZNF503 antisense RNA 1	2,6	0,8	**0,51**	0,001117

XAF1	XIAP associated factor 1	4,3	1,5	**0,51**	0,000221
HOXA10	homeobox A10	35,2	12,2	**0,51**	2,00E-06
GPC4	glypican 4	4,8	1,7	**0,51**	0,003297
MCOLN3	mucolipin 3	13,1	4,1	**0,51**	0,000426
E2F7	E2F transcription factor 7	1,7	0,6	**0,51**	0,009513
ADA	adenosine deaminase	0,7	0,3	**0,51**	0,010688
CNKSR2	connector enhancer of kinase suppressor of Ras 2	1,9	0,6	**0,51**	0,005498
FAAH	fatty acid amide hydrolase	8,0	2,6	**0,51**	0,000264
LINC00482	long intergenic non-protein coding RNA 482	0,8	0,4	**0,51**	0,012088
PPIC	peptidylprolyl isomerase C	72,3	24,4	**0,50**	1,60E-05
CHD4	chromodomain helicase DNA binding protein 4	162,3	59,4	**0,50**	0
SDK2	sidekick cell adhesion molecule 2	197,8	66,9	**0,50**	7,00E-06
FGF1	fibroblast growth factor 1	133,4	45,0	**0,50**	2,20E-05
KNDC1	kinase non-catalytic C-lobe domain containing 1	2,2	0,8	**0,50**	0,001622
ICA1L	islet cell autoantigen 1 like	6,0	2,0	**0,50**	2,00E-04
8.syys	septin 8			**0,50**	0
COL15A1	collagen type XV alpha 1 chain	588,6	195,1	**0,50**	0,000972
PACS1	phosphofurin acidic cluster sorting protein 1	66,2	23,8	**0,50**	0
CORIN	corin, serine peptidase	0,9	0,4	**0,50**	0,002475
PHLPP1	PH domain and leucine rich repeat protein phosphatase 1	26,0	9,1	**0,50**	0
RCCD1	RCC1 domain containing 1	13,0	4,3	**0,50**	8,30E-05
MIR1282	microRNA 1282	0,5	0,2	**0,50**	0,02395
ZNF391	zinc finger protein 391	4,2	1,5	**0,50**	0,000368
GNG7	G protein subunit gamma 7	7,8	2,5	**0,50**	0,001012
PTGFRN	prostaglandin F2 receptor inhibitor	63,9	21,5	**0,50**	0
GSE1	Gse1 coiled-coil protein	34,7	11,7	**0,50**	1,00E-06
GPX7	glutathione peroxidase 7	9,5	3,3	**0,50**	0,000106
SV2A	synaptic vesicle glycoprotein 2A	10,8	4,2	**0,50**	2,00E-06
PAPSS1	3'-phosphoadenosine 5'-phosphosulfate synthase 1	240,0	80,6	**0,50**	1,20E-05
NME5	NME/NM23 family member 5	2,5	0,8	**0,50**	0,002104
EIF4E3	eukaryotic translation initiation factor 4E family member 3	26,2	9,0	**0,50**	3,00E-06
TBCD	tubulin folding cofactor D	27,8	9,3	**0,50**	1,00E-06
AOX1	aldehyde oxidase 1	13,7	5,5	**0,50**	0,000375

RNF180	ring finger protein 180	3,6	1,3	**0,50**	0,000787
NT5DC1	5'-nucleotidase domain containing 1	28,0	9,6	**0,50**	7,00E-06
PRKD1	protein kinase D1	24,2	8,4	**0,50**	2,00E-06
PARD3B	par-3 family cell polarity regulator beta	6,4	2,4	**0,50**	7,30E-05
SYTL3	synaptotagmin like 3	2,4	0,9	**0,50**	0,001009
PARVB	parvin beta	14,2	4,9	**0,50**	5,90E-05
RYR1	ryanodine receptor 1	2,0	0,6	**0,50**	0,003836
CD1D	CD1d molecule	5,3	1,6	**0,50**	0,003025
CSDC2	cold shock domain containing C2	5,4	1,8	**0,50**	0,003066
PRKCQ	protein kinase C theta	4,9	1,7	**0,50**	0,001378
ADORA1	adenosine A1 receptor	32,0	10,7	**0,50**	0,000165
AQP1	aquaporin 1 (Colton blood group)	1,5	0,5	**0,50**	0,00266
KIFC2	kinesin family member C2	6,2	2,2	**0,50**	0,000262
KRBOX1	KRAB box domain containing 1	0,8	0,3	**0,50**	0,017996
MPDZ	multiple PDZ domain crumbs cell polarity complex component	31,2	11,7	**0,50**	0
ABCC4	ATP binding cassette subfamily C member 4	27,3	10,7	**0,50**	0
FAM167B	family with sequence similarity 167 member B	1,2	0,4	**0,50**	0,011731
PATJ	PATJ, crumbs cell polarity complex component	19,8	6,9	**0,50**	0
P4HA3	prolyl 4-hydroxylase subunit alpha 3	32,4	11,6	**0,50**	8,00E-06
FIGN	fidgetin, microtubule severing factor	5,1	1,9	**0,50**	4,00E-05
ZMYND10	zinc finger MYND-type containing 10	3,0	0,7	**0,50**	0,018581
MPP7	membrane palmitoylated protein 7	9,5	3,2	**0,50**	0,00014
SLC25A20	solute carrier family 25 member 20	18,6	6,1	**0,50**	2,00E-05
PCDHB11	protocadherin beta 11	2,5	0,9	**0,50**	0,000479
BANK1	B-cell scaffold protein with ankyrin repeats 1	15,1	5,0	**0,50**	0,000207
IMPA2	inositol monophosphatase 2	6,3	2,0	**0,50**	0,000587
SLC38A5	solute carrier family 38 member 5	10,9	4,0	**0,50**	0,000205
KIAA0513	KIAA0513	31,9	10,2	**0,50**	2,00E-06
C14orf93	chromosome 14 open reading frame 93	5,5	2,0	**0,50**	1,50E-05
SFT2D2	SFT2 domain containing 2	92,6	31,5	**0,50**	1,00E-06
RGS22	regulator of G protein signaling 22	1,6	0,4	**0,50**	0,041913
CDCA7L	cell division cycle associated 7 like	15,8	5,3	**0,50**	2,40E-05
SLC17A9	solute carrier family 17 member 9	22,9	7,9	**0,50**	0,000513

LINC01152	long intergenic non-protein coding RNA 1152	3,3	1,1	**0,50**	0,000342
CCDC89	coiled-coil domain containing 89	3,4	1,2	**0,49**	0,000194
ZNF726	zinc finger protein 726	1,2	0,4	**0,49**	0,00327
TMPO	thymopoietin	67,1	20,5	**0,49**	0
SLC43A1	solute carrier family 43 member 1	6,8	2,2	**0,49**	0,000177
ME3	malic enzyme 3	6,0	1,9	**0,49**	0,000641
MYZAP	myocardial zonula adherens protein	0,6	0,2	**0,49**	0,01672
GLS	glutaminase	245,7	84,1	**0,49**	2,20E-05
C21orf58	chromosome 21 open reading frame 58	4,1	1,2	**0,49**	0,000434
TNXB	tenascin XB	2,4	0,9	**0,49**	0,000202
TRIM65	tripartite motif containing 65	19,4	6,8	**0,49**	1,00E-06
TRPM4	transient receptor potential cation channel subfamily M member 4	8,1	3,0	**0,49**	2,10E-05
ACAN	aggrecan	11285,0	3792,2	**0,49**	0,012034
CCDC180	coiled-coil domain containing 180	0,5	0,2	**0,49**	0,006816
SGO2	shugoshin 2	13,1	3,6	**0,49**	3,50E-05
EDARADD	EDAR associated death domain	2,0	0,6	**0,49**	0,00062
PPM1M	protein phosphatase, Mg2+/Mn2+ dependent 1M	20,3	6,6	**0,49**	3,00E-05
MPP2	membrane palmitoylated protein 2	5,4	1,8	**0,49**	0,000429
PCDHB3	protocadherin beta 3	2,5	0,9	**0,49**	0,000501
FABP5	fatty acid binding protein 5	6,5	2,3	**0,49**	0,000885
GALNT18	polypeptide N-acetylgalactosaminyltransferase 18	102,1	31,8	**0,49**	6,50E-05
AGMAT	agmatinase	1,3	0,4	**0,49**	0,000842
CHAF1B	chromatin assembly factor 1 subunit B	9,1	2,7	**0,49**	0,000215
ZC3HAV1L	zinc finger CCCH-type containing, antiviral 1 like	3,7	1,3	**0,49**	0,000319
MPPED2	metallophosphoesterase domain containing 2	5,7	1,9	**0,49**	9,00E-04
TRAM2	translocation associated membrane protein 2	415,5	136,5	**0,49**	2,00E-05
AHNAK2	AHNAK nucleoprotein 2	308,1	117,5	**0,49**	1,00E-06
ATAD2	ATPase family, AAA domain containing 2	42,5	13,0	**0,49**	0
DNASE1L1	deoxyribonuclease 1 like 1	73,3	26,6	**0,49**	1,00E-06
MCM2	minichromosome maintenance complex component 2	48,7	13,8	**0,49**	1,00E-06
SLC2A9	solute carrier family 2 member 9	1,6	0,6	**0,49**	0,002215
C16orf59	chromosome 16 open reading frame 59			**0,49**	0,010908
AP3S1	adaptor related protein complex 3 sigma 1 subunit	44,7	15,2	**0,49**	7,00E-06

LRRC20	leucine rich repeat containing 20	10,7	3,2	**0,49**	0,000586
ALDH5A1	aldehyde dehydrogenase 5 family member A1	7,5	2,5	**0,49**	2,80E-05
HDAC11	histone deacetylase 11	18,9	6,4	**0,49**	1,60E-05
PHACTR2	phosphatase and actin regulator 2	52,8	18,3	**0,49**	0
KANK2	KN motif and ankyrin repeat domains 2	132,5	44,9	**0,49**	0
GTF2I	general transcription factor IIi	37,9	13,5	**0,49**	0
FAM19A5	family with sequence similarity 19 member A5, C-C motif chemokine l	2,6	0,9	**0,49**	0,012822
TTLL3	tubulin tyrosine ligase like 3	8,7	2,8	**0,49**	2,10E-05
MISP3	MISP family member 3	2,4	0,8	**0,49**	0,001224
TMEM98	transmembrane protein 98	67,8	22,9	**0,49**	9,00E-06
VWA5A	von Willebrand factor A domain containing 5A	29,3	9,8	**0,49**	2,00E-06
NGEF	neuronal guanine nucleotide exchange factor	126,4	43,2	**0,49**	2,20E-05
CABLES1	Cdk5 and Abl enzyme substrate 1	20,4	6,2	**0,49**	0,000176
PITPNM1	phosphatidylinositol transfer protein membrane associated 1	50,4	17,4	**0,49**	2,00E-06
GLIS2	GLIS family zinc finger 2	0,9	0,3	**0,49**	0,033531
SKA2	spindle and kinetochore associated complex subunit 2	25,7	7,7	**0,49**	1,00E-06
UTRN	utrophin	106,5	38,2	**0,49**	0
ETFB	electron transfer flavoprotein beta subunit	25,5	8,3	**0,49**	1,00E-05
SLC27A5	solute carrier family 27 member 5	3,0	1,0	**0,49**	0,000475
KIAA1161	KIAA1161			**0,49**	1,00E-06
PTMA	prothymosin, alpha	485,7	160,3	**0,49**	0,000104
PTPRD-AS1	PTPRD antisense RNA 1	1,8	0,5	**0,49**	0,009739
PASK	PAS domain containing serine/threonine kinase	5,9	1,9	**0,49**	9,00E-06
FILIP1	filamin A interacting protein 1	1,9	0,8	**0,48**	4,70E-05
ADCY9	adenylate cyclase 9	30,0	10,2	**0,48**	0
AADAT	aminoadipate aminotransferase	6,9	2,1	**0,48**	4,90E-05
DLX5	distal-less homeobox 5	19,4	6,0	**0,48**	0,000104
EML3	echinoderm microtubule associated protein like 3	70,3	23,5	**0,48**	2,00E-06
MNS1	meiosis specific nuclear structural 1	4,3	1,3	**0,48**	0,000323
FHOD1	formin homology 2 domain containing 1	20,4	6,4	**0,48**	2,50E-05
SULF1	sulfatase 1	724,1	263,9	**0,48**	7,70E-05
TSPAN6	tetraspanin 6	98,4	31,2	**0,48**	8,00E-06
RGCC	regulator of cell cycle	343,4	107,7	**0,48**	0,000297

FNDC4	fibronectin type III domain containing 4	21,1	7,3	**0,48**	2,40E-05
PLBD1-AS1	PLBD1 antisense RNA 1	1,1	0,4	**0,48**	0,003866
MMP16	matrix metallopeptidase 16	21,6	6,3	**0,48**	7,60E-05
BACE1	beta-secretase 1	76,1	24,7	**0,48**	0
SELENOP	selenoprotein P	47,1	14,7	**0,48**	2,00E-06
PARP1	poly(ADP-ribose) polymerase 1	93,9	30,7	**0,48**	0
FRMPD4	FERM and PDZ domain containing 4	2,3	0,7	**0,48**	0,000401
DPY19L2P2	DPY19L2 pseudogene 2	4,0	1,2	**0,48**	6,90E-05
TMEM117	transmembrane protein 117	6,3	1,9	**0,48**	9,80E-05
CD9	CD9 molecule	230,4	77,1	**0,48**	3,30E-05
DNM1	dynamin 1	45,5	14,3	**0,48**	0
UAP1L1	UDP-N-acetylglucosamine pyrophosphorylase 1 like 1	16,4	5,4	**0,48**	2,60E-05
AQP3	aquaporin 3 (Gill blood group)	15,2	5,7	**0,48**	9,90E-05
RAG1	recombination activating 1	1,2	0,4	**0,48**	0,003138
ZNF793	zinc finger protein 793	5,4	1,8	**0,48**	6,00E-06
ALDH1A2	aldehyde dehydrogenase 1 family member A2	138,0	46,7	**0,48**	1,00E-06
ZBTB16	zinc finger and BTB domain containing 16	4,2	1,5	**0,48**	0,000185
ALYREF	Aly/REF export factor	66,3	20,1	**0,48**	1,70E-05
LINC01140	long intergenic non-protein coding RNA 1140	2,2	0,7	**0,48**	0,000446
AKAP1	A-kinase anchoring protein 1	40,9	12,8	**0,48**	5,00E-06
DMBT1	deleted in malignant brain tumors 1	0,6	0,3	**0,48**	0,003485
MCM3	minichromosome maintenance complex component 3	84,4	24,6	**0,48**	0
SCRN2	secernin 2	16,3	5,3	**0,48**	2,20E-05
LBP	lipopolysaccharide binding protein	22,8	10,0	**0,48**	0,000397
ACAP3	ArfGAP with coiled-coil, ankyrin repeat and PH domains 3	27,3	9,0	**0,48**	1,70E-05
GBGT1	globoside alpha-1,3-N-acetylgalactosaminyltransferase 1 (FORS blood	9,4	3,0	**0,48**	7,20E-05
POC1A	POC1 centriolar protein A	10,1	2,7	**0,48**	1,20E-05
GAL3ST4	galactose-3-O-sulfotransferase 4	0,7	0,2	**0,48**	0,002201
RTL8C	retrotransposon Gag like 8C	122,9	42,1	**0,48**	7,00E-06
SYTL1	synaptotagmin like 1	0,9	0,2	**0,48**	0,042404
CXXC5	CXXC finger protein 5	111,7	37,6	**0,48**	4,00E-06
USP18	ubiquitin specific peptidase 18	1,1	0,3	**0,48**	0,005336
LRP8	LDL receptor related protein 8	97,5	34,8	**0,48**	0

CTF1	cardiotrophin 1	6,3	2,0	**0,48**	0,00036
SHF	Src homology 2 domain containing F	2,8	0,9	**0,48**	0,000592
PLSCR4	phospholipid scramblase 4	36,6	12,6	**0,48**	0
MAP4K2	mitogen-activated protein kinase kinase kinase kinase 2	9,7	3,0	**0,48**	2,10E-05
EPB41L2	erythrocyte membrane protein band 4.1 like 2	125,6	40,4	**0,48**	0
VWDE	von Willebrand factor D and EGF domains	3,0	1,2	**0,48**	0,002231
FCHSD2	FCH and double SH3 domains 2	62,8	20,7	**0,48**	0
ENPP6	ectonucleotide pyrophosphatase/phosphodiesterase 6	1,2	0,4	**0,48**	0,000159
NUAK1	NUAK family kinase 1	23,3	8,0	**0,48**	0
HSF4	heat shock transcription factor 4	4,5	1,4	**0,48**	0,002104
ZNF521	zinc finger protein 521	61,4	20,9	**0,48**	0
MTHFD1	methylenetetrahydrofolate dehydrogenase, cyclohydrolase and formy	77,5	25,2	**0,48**	0
KNTC1	kinetochore associated 1	19,9	5,7	**0,48**	0
GINS4	GINS complex subunit 4	11,1	3,2	**0,48**	0,000295
ACKR2	atypical chemokine receptor 2	2,4	0,9	**0,48**	0,000313
LINC02449	long intergenic non-protein coding RNA 2449	0,8	0,2	**0,48**	0,003475
P2RY2	purinergic receptor P2Y2	7,7	2,7	**0,48**	0,000402
KATNAL2	katanin catalytic subunit A1 like 2	2,7	0,8	**0,47**	0,001393
MATN2	matrilin 2	54,3	18,6	**0,47**	0
MYBL1	MYB proto-oncogene like 1	51,9	15,6	**0,47**	3,00E-06
C9orf40	chromosome 9 open reading frame 40	7,7	2,4	**0,47**	2,40E-05
IFI16	interferon gamma inducible protein 16	25,7	8,1	**0,47**	5,00E-06
DPY19L2P1	DPY19L2 pseudogene 1	1,8	0,6	**0,47**	0,001406
GRIN2C	glutamate ionotropic receptor NMDA type subunit 2C	12,6	3,8	**0,47**	0,008051
ZWILCH	zwilch kinetochore protein	26,4	8,0	**0,47**	0
KLHDC8B	kelch domain containing 8B	9,1	2,7	**0,47**	3,30E-05
C20orf27	chromosome 20 open reading frame 27	22,9	7,6	**0,47**	1,00E-04
DGKA	diacylglycerol kinase alpha	16,5	5,2	**0,47**	1,00E-06
DAB2	DAB2, clathrin adaptor protein	336,3	115,1	**0,47**	2,00E-06
RFC3	replication factor C subunit 3	14,1	3,8	**0,47**	1,00E-05
PCDHGC3	protocadherin gamma subfamily C, 3	162,5	52,4	**0,47**	0
ID4	inhibitor of DNA binding 4, HLH protein	69,9	23,4	**0,47**	1,00E-06
PTMAP4	prothymosin, alpha pseudogene 4	2,0	0,7	**0,47**	9,40E-05

GPC1	glypican 1	294,8	105,0	**0,47**	1,70E-05
CNTD1	cyclin N-terminal domain containing 1	1,0	0,3	**0,47**	0,001445
TMEM107	transmembrane protein 107	7,0	2,4	**0,47**	2,00E-05
SCAPER	S-phase cyclin A associated protein in the ER	14,6	4,9	**0,47**	0
PLCD1	phospholipase C delta 1	152,6	48,0	**0,47**	1,10E-05
SELENON	selenoprotein N	69,1	22,5	**0,47**	0
ZNF618	zinc finger protein 618	4,7	1,5	**0,47**	0,000131
BARD1	BRCA1 associated RING domain 1	11,4	3,4	**0,47**	2,20E-05
RAB27A	RAB27A, member RAS oncogene family	69,9	21,8	**0,47**	0
PHKA1	phosphorylase kinase regulatory subunit alpha 1	10,8	3,4	**0,47**	3,00E-06
SFTPD	surfactant protein D	1,7	0,7	**0,47**	0,022087
KY	kyphoscoliosis peptidase	0,7	0,2	**0,47**	0,003762
ARHGAP1	Rho GTPase activating protein 1	160,6	51,9	**0,47**	0
FOXCUT	FOXC1 upstream transcript (non-protein coding)	1,0	0,4	**0,47**	0,000412
FNIP2	folliculin interacting protein 2	103,2	37,2	**0,47**	0
PDLIM1	PDZ and LIM domain 1	2,0	0,7	**0,47**	4,50E-05
HR	HR, lysine demethylase and nuclear receptor corepressor	30,3	8,6	**0,47**	7,20E-05
NLN	neurolysin	31,7	10,4	**0,47**	1,00E-06
SLC2A4RG	SLC2A4 regulator	88,0	27,0	**0,47**	6,50E-05
PARD6G	par-6 family cell polarity regulator gamma	2,7	0,9	**0,47**	0,000468
SOX15	SRY-box 15	0,7	0,2	**0,47**	0,004657
RAB15	RAB15, member RAS oncogene family	9,7	3,0	**0,47**	0,000115
LRRC17	leucine rich repeat containing 17	1,4	0,3	**0,47**	0,004654
PIFO	primary cilia formation	5,5	1,9	**0,47**	0,000422
PFKM	phosphofructokinase, muscle	50,5	16,5	**0,47**	0
ARHGEF16	Rho guanine nucleotide exchange factor 16	1,1	0,3	**0,47**	0,003073
AGR2	anterior gradient 2, protein disulphide isomerase family member	22,2	7,7	**0,47**	0,001401
GPR155	G protein-coupled receptor 155	11,6	3,8	**0,47**	5,00E-06
LRIG3	leucine rich repeats and immunoglobulin like domains 3	60,7	20,0	**0,47**	0
NME3	NME/NM23 nucleoside diphosphate kinase 3	17,8	5,6	**0,47**	9,50E-05
B3GNT8	UDP-GlcNAc:betaGal beta-1,3-N-acetylglucosaminyltransferase 8	1,0	0,3	**0,47**	0,00774
FRY	FRY microtubule binding protein	147,0	46,2	**0,47**	0
ARVCF	armadillo repeat gene deleted in velocardiofacial syndrome	10,9	3,5	**0,47**	6,10E-05

BIVM	basic, immunoglobulin-like variable motif containing	9,1	2,9	**0,47**	1,00E-05
ZNF492	zinc finger protein 492	0,4	0,2	**0,47**	0,016511
MDFIC	MyoD family inhibitor domain containing	179,3	59,4	**0,47**	0
ETV2	ETS variant 2	0,8	0,3	**0,47**	0,004567
PPP1R26-AS1	PPP1R26 antisense RNA 1	0,5	0,2	**0,47**	0,001679
MEX3A	mex-3 RNA binding family member A	2,8	0,9	**0,47**	8,30E-05
FOXA3	forkhead box A3	14,4	4,4	**0,47**	4,00E-06
ZNF273	zinc finger protein 273	3,9	1,2	**0,46**	4,30E-05
PDGFRA	platelet derived growth factor receptor alpha	241,1	78,9	**0,46**	0
LYNX1	Ly6/neurotoxin 1	114,4	37,1	**0,46**	3,00E-05
LYL1	LYL1, basic helix-loop-helix family member	0,7	0,2	**0,46**	0,000906
PRR7	proline rich 7, synaptic	5,5	1,7	**0,46**	0,001428
GSTK1	glutathione S-transferase kappa 1	39,0	12,4	**0,46**	2,00E-06
STAC2	SH3 and cysteine rich domain 2	4,1	1,3	**0,46**	0,004233
MYO3A	myosin IIIA	0,9	0,4	**0,46**	0,005559
LPCAT1	lysophosphatidylcholine acyltransferase 1	2,3	0,6	**0,46**	0,00053
BEGAIN	brain enriched guanylate kinase associated	7,3	2,1	**0,46**	4,50E-05
ARHGEF6	Rac/Cdc42 guanine nucleotide exchange factor 6	15,7	5,4	**0,46**	1,00E-06
ESPNL	espin-like	2,4	0,6	**0,46**	0,008252
ERGIC1	endoplasmic reticulum-golgi intermediate compartment 1	252,7	81,8	**0,46**	5,00E-06
FAM102B	family with sequence similarity 102 member B	11,6	3,3	**0,46**	5,00E-06
LRP4	LDL receptor related protein 4	19,8	6,3	**0,46**	7,00E-06
SOX5	SRY-box 5	194,7	67,3	**0,46**	0
HHIPL2	HHIP like 2	33,7	10,9	**0,46**	5,00E-05
ITIH5	inter-alpha-trypsin inhibitor heavy chain family member 5	36,2	12,3	**0,46**	1,00E-06
CFAP221	cilia and flagella associated protein 221	0,8	0,2	**0,46**	0,00763
CCDC34	coiled-coil domain containing 34	11,7	3,1	**0,46**	5,00E-06
FAM81A	family with sequence similarity 81 member A	3,3	0,9	**0,46**	0,004233
DBN1	drebrin 1	50,9	15,5	**0,46**	1,00E-06
REEP2	receptor accessory protein 2	5,3	1,6	**0,46**	6,80E-05
MAGED2	MAGE family member D2	603,3	184,5	**0,46**	5,20E-05
RAD54B	RAD54 homolog B (S. cerevisiae)	0,5	0,1	**0,46**	0,006529
TMEM144	transmembrane protein 144	3,7	0,9	**0,46**	0,001423

EPDR1	ependymin related 1	16,3	5,3	**0,46**	1,00E-06
RGMA	repulsive guidance molecule family member a	47,8	14,4	**0,46**	0
SCN8A	sodium voltage-gated channel alpha subunit 8	5,2	1,7	**0,46**	0,000338
ITGA6	integrin subunit alpha 6	65,4	19,7	**0,46**	0
TMEM19	transmembrane protein 19	30,0	9,7	**0,46**	0
MCM4	minichromosome maintenance complex component 4	66,6	18,4	**0,46**	0
B4GALT2	beta-1,4-galactosyltransferase 2	119,2	37,5	**0,46**	9,00E-06
C4BPA	complement component 4 binding protein alpha	2,6	0,9	**0,46**	0,00788
CHDH	choline dehydrogenase	19,4	6,2	**0,46**	0
CARMIL1	capping protein regulator and myosin 1 linker 1	29,1	9,0	**0,46**	0
WDHD1	WD repeat and HMG-box DNA binding protein 1	13,4	3,5	**0,46**	4,00E-06
ANKRD45	ankyrin repeat domain 45	0,6	0,2	**0,46**	0,041529
FXYD6	FXYD domain containing ion transport regulator 6	194,9	64,7	**0,46**	2,00E-06
PLIN2	perilipin 2	41,2	12,6	**0,46**	0
PALM	paralemmin	4,1	1,1	**0,46**	0,000992
KLHL34	kelch like family member 34	3,3	1,0	**0,46**	0,001087
KIF24	kinesin family member 24	4,4	1,3	**0,46**	5,70E-05
PTMAP5	prothymosin, alpha pseudogene 5	5,9	1,9	**0,46**	0,00012
SLC25A43	solute carrier family 25 member 43	21,5	7,3	**0,46**	0
KIF26A	kinesin family member 26A	15,5	4,8	**0,46**	0
PHF19	PHD finger protein 19	70,0	20,7	**0,46**	0
PRIM1	primase (DNA) subunit 1	8,6	2,2	**0,46**	4,20E-05
B3GLCT	beta 3-glucosyltransferase	30,0	9,3	**0,46**	0
CORO6	coronin 6	1,4	0,3	**0,45**	0,007732
NUDT17	nudix hydrolase 17	3,1	0,8	**0,45**	0,000215
KCNT2	potassium sodium-activated channel subfamily T member 2	7,2	2,3	**0,45**	9,30E-05
RECQL4	RecQ like helicase 4	16,5	4,4	**0,45**	4,00E-05
LINC00882	long intergenic non-protein coding RNA 882	0,9	0,3	**0,45**	0,001061
GPAT2	glycerol-3-phosphate acyltransferase 2, mitochondrial	7,2	2,3	**0,45**	6,10E-05
ABCA2	ATP binding cassette subfamily A member 2	82,4	25,9	**0,45**	0
CD14	CD14 molecule	76,6	21,0	**0,45**	1,40E-05
CDNF	cerebral dopamine neurotrophic factor	3,1	0,9	**0,45**	0,000264
SLC25A35	solute carrier family 25 member 35	2,7	0,8	**0,45**	0,001237

COL6A3	collagen type VI alpha 3 chain	3671,0	1158,5	**0,45**	0,000585
PRDX2	peroxiredoxin 2	88,8	28,1	**0,45**	5,00E-06
CDC25A	cell division cycle 25A	8,5	2,5	**0,45**	2,00E-06
SHROOM3	shroom family member 3	22,3	6,8	**0,45**	0
IDH1	isocitrate dehydrogenase (NADP(+)) 1, cytosolic	99,3	31,2	**0,45**	5,00E-06
NFATC1	nuclear factor of activated T-cells 1	67,8	21,6	**0,45**	0
IMPDH1	inosine monophosphate dehydrogenase 1	42,9	12,4	**0,45**	1,00E-06
1.maalis	mitochondrial amidoxime reducing component 1	2,8	1,9	**0,45**	0,003232
DNA2	DNA replication helicase/nuclease 2	3,8	1,2	**0,45**	3,10E-05
PRELP	proline and arginine rich end leucine rich repeat protein	2931,1	891,3	**0,45**	0,00124
TRPV2	transient receptor potential cation channel subfamily V member 2	7,9	2,3	**0,45**	1,60E-05
HAPLN1	hyaluronan and proteoglycan link protein 1	1983,5	618,0	**0,45**	0,000539
ZBTB42	zinc finger and BTB domain containing 42		1,7	**0,45**	2,30E-05
HSD17B14	hydroxysteroid 17-beta dehydrogenase 14	6,5	1,8	**0,45**	0,000647
MRAS	muscle RAS oncogene homolog	40,0	12,2	**0,45**	0
RPL22L1	ribosomal protein L22 like 1	29,1	9,1	**0,45**	1,00E-06
IL1R1	interleukin 1 receptor type 1	149,1	45,2	**0,45**	0
PYGL	glycogen phosphorylase L	81,3	24,4	**0,45**	1,00E-06
GRIK3	glutamate ionotropic receptor kainate type subunit 3	4,9	1,4	**0,45**	8,20E-05
GPX3	glutathione peroxidase 3	1187,1	366,9	**0,45**	0,000341
FBLIM1	filamin binding LIM protein 1	2,7	0,9	**0,45**	3,00E-05
ELFN1	extracellular leucine rich repeat and fibronectin type III domain contai	1,0	0,3	**0,45**	0,023212
RTKN	rhotekin	44,5	12,7	**0,45**	0
TGFB2	transforming growth factor beta 2	183,9	55,0	**0,45**	1,00E-06
ZNF33B	zinc finger protein 33B	10,8	3,5	**0,45**	3,50E-05
CPNE3	copine 3	181,3	55,6	**0,45**	0
ANKEF1	ankyrin repeat and EF-hand domain containing 1	3,3	1,1	**0,45**	0,000461
JPH3	junctophilin 3	1,2	0,3	**0,45**	0,00181
MFSD7	major facilitator superfamily domain containing 7			**0,45**	9,00E-06
BOK	BOK, BCL2 family apoptosis regulator	88,8	27,8	**0,45**	0
ME1	malic enzyme 1	37,0	11,7	**0,45**	1,00E-05
FAM83D	family with sequence similarity 83 member D	7,2	1,9	**0,45**	0,002684
EIF2AK4	eukaryotic translation initiation factor 2 alpha kinase 4	41,6	12,9	**0,45**	0

PHTF2	putative homeodomain transcription factor 2	104,0	30,7	**0,45**	0
CDH24	cadherin 24	3,2	0,9	**0,45**	0,000407
PCDHB13	protocadherin beta 13	4,2	1,3	**0,45**	1,40E-05
MAP3K12	mitogen-activated protein kinase kinase kinase 12	9,3	2,8	**0,45**	7,00E-06
CLYBL	citrate lyase beta like	2,5	0,9	**0,45**	0,000148
SMIM5	small integral membrane protein 5	2,2	0,5	**0,45**	0,002812
1.syys	septin 1	2,8	1,9	**0,44**	0,008073
PDK4	pyruvate dehydrogenase kinase 4	12,5	3,2	**0,44**	0,000103
F5	coagulation factor V	28,1	10,3	**0,44**	0,005121
WWP2	WW domain containing E3 ubiquitin protein ligase 2	652,8	180,1	**0,44**	0,000187
PRX	periaxin	7,3	2,1	**0,44**	1,50E-05
LINC01521	long intergenic non-protein coding RNA 1521	2,2	0,7	**0,44**	0,000233
KCNA1	potassium voltage-gated channel subfamily A member 1	5,3	1,6	**0,44**	0,000128
FKBP9	FK506 binding protein 9	136,7	44,4	**0,44**	0
NOTCH3	notch 3	13,9	4,2	**0,44**	0,002688
ATP5F1P5	ATP synthase, H+ transporting, mitochondrial Fo complex subunit B1 pseudogene 5			**0,44**	0,001589
CPNE2	copine 2	62,0	18,3	**0,44**	0
KLHL3	kelch like family member 3	5,2	1,6	**0,44**	6,10E-05
FIBIN	fin bud initiation factor homolog (zebrafish)	166,8	50,4	**0,44**	1,00E-06
GRTP1	growth hormone regulated TBC protein 1	3,1	1,0	**0,44**	0,008101
TCF4	transcription factor 4	117,1	42,0	**0,44**	0
JDP2	Jun dimerization protein 2	63,0	19,0	**0,44**	0
RTL8B	retrotransposon Gag like 8B	23,9	7,2	**0,44**	4,00E-06
NAGA	alpha-N-acetylgalactosaminidase	49,4	15,8	**0,44**	0
CDK2	cyclin dependent kinase 2	33,9	9,2	**0,44**	0
MAPRE2	microtubule associated protein RP/EB family member 2	128,3	38,0	**0,44**	0
WLS	wntless Wnt ligand secretion mediator	91,6	25,5	**0,44**	0
TNIK	TRAF2 and NCK interacting kinase	33,8	9,6	**0,44**	0
ZNF488	zinc finger protein 488	0,6	0,2	**0,44**	0,012463
PRR16	proline rich 16	1,8	0,6	**0,44**	0,005572
PPARG	peroxisome proliferator activated receptor gamma	0,8	0,3	**0,44**	0,00047
ADSSL1	adenylosuccinate synthase like 1	26,2	7,1	**0,44**	1,20E-05
PTMS	parathymosin	253,9	76,2	**0,44**	2,50E-05

SLC16A8	solute carrier family 16 member 8	0,5	0,2	**0,44**	0,024173
SYNPO	synaptopodin	149,0	42,6	**0,44**	0
OBSL1	obscurin like 1	128,6	36,6	**0,44**	1,00E-06
DBP	D-box binding PAR bZIP transcription factor	13,7	4,4	**0,44**	1,00E-06
BAD	BCL2 associated agonist of cell death	26,2	8,1	**0,44**	4,00E-06
FBXO44	F-box protein 44	20,6	6,3	**0,44**	3,00E-06
PCYOX1	prenylcysteine oxidase 1	177,4	55,3	**0,44**	1,00E-06
CIB2	calcium and integrin binding family member 2	7,6	2,3	**0,44**	2,00E-06
EDIL3	EGF like repeats and discoidin domains 3	1164,6	387,4	**0,44**	3,60E-05
TMEM14A	transmembrane protein 14A	23,2	6,5	**0,44**	4,20E-05
C5	complement C5	12,1	3,3	**0,44**	1,00E-06
ISLR	immunoglobulin superfamily containing leucine rich repeat	544,0	173,8	**0,44**	0,000438
ANGPTL7	angiopoietin like 7	11,2	3,7	**0,44**	2,70E-05
TPGS2	tubulin polyglutamylase complex subunit 2	62,5	19,2	**0,44**	0
PNPLA7	patatin like phospholipase domain containing 7	6,8	1,9	**0,44**	9,80E-05
APLN	apelin	2,9	0,8	**0,44**	0,000349
ACSS3	acyl-CoA synthetase short-chain family member 3	5,8	1,8	**0,43**	3,00E-06
LRRC15	leucine rich repeat containing 15	91,5	28,2	**0,43**	3,80E-05
TRIM7	tripartite motif containing 7	6,3	1,7	**0,43**	1,40E-05
MTMR9LP	myotubularin related protein 9-like, pseudogene	3,1	0,9	**0,43**	6,70E-05
HADH	hydroxyacyl-CoA dehydrogenase	47,7	13,5	**0,43**	0
PARPBP	PARP1 binding protein	3,6	0,8	**0,43**	0,000968
SELL	selectin L	3,8	1,1	**0,43**	0,000251
FBLN1	fibulin 1	19,4	5,8	**0,43**	2,10E-05
FAM213A	family with sequence similarity 213 member A			**0,43**	4,00E-06
OXCT1	3-oxoacid CoA-transferase 1	67,6	19,2	**0,43**	0
GNB4	G protein subunit beta 4	57,8	16,6	**0,43**	0
UACA	uveal autoantigen with coiled-coil domains and ankyrin repeats	165,1	45,0	**0,43**	0
EHD3	EH domain containing 3	29,0	8,2	**0,43**	1,00E-06
CNN2	calponin 2	45,7	12,3	**0,43**	1,00E-05
MORC4	MORC family CW-type zinc finger 4	27,5	7,9	**0,43**	0
NRBP2	nuclear receptor binding protein 2	20,8	6,8	**0,43**	0
SPINT2	serine peptidase inhibitor, Kunitz type 2	96,9	30,1	**0,43**	5,00E-06

ARHGEF3	Rho guanine nucleotide exchange factor 3	17,6	5,0	**0,43**	8,00E-06
PWAR6	Prader Willi/Angelman region RNA 6		0,5	**0,43**	0,000357
TUBA1A	tubulin alpha 1a	69,6	21,3	**0,43**	0
LGI4	leucine rich repeat LGI family member 4	4,8	1,2	**0,43**	0,000266
MAPK10	mitogen-activated protein kinase 10	5,1	1,5	**0,43**	0,000101
COL25A1	collagen type XXV alpha 1 chain	0,8	0,2	**0,43**	0,001728
PRKG1	protein kinase, cGMP-dependent, type I	18,0	5,0	**0,43**	1,00E-05
ESYT1	extended synaptotagmin 1	155,4	43,7	**0,43**	0
FADS1	fatty acid desaturase 1	67,2	19,7	**0,43**	0
BAHCC1	BAH domain and coiled-coil containing 1	23,2	7,6	**0,43**	0
MTSS1L	MTSS1L, I-BAR domain containing	75,4	21,9	**0,43**	0
ABCA8	ATP binding cassette subfamily A member 8	4,6	1,2	**0,43**	0,000539
SORD	sorbitol dehydrogenase	2,8	0,7	**0,43**	0,001094
ANXA4	annexin A4	113,2	32,7	**0,43**	0
PXMP2	peroxisomal membrane protein 2	1,9	0,5	**0,43**	0,001967
WIPF1	WAS/WASL interacting protein family member 1	70,6	20,1	**0,43**	0
ALDH7A1	aldehyde dehydrogenase 7 family member A1	19,9	5,8	**0,43**	1,00E-06
GAP43	growth associated protein 43	0,7	0,2	**0,43**	0,001789
ZNF792	zinc finger protein 792	2,3	0,6	**0,43**	0,000281
GALT	galactose-1-phosphate uridylyltransferase	8,2	2,4	**0,43**	1,60E-05
FBXO43	F-box protein 43	0,9	0,2	**0,43**	0,001622
DDO	D-aspartate oxidase	1,4	0,5	**0,43**	4,90E-05
HLF	HLF, PAR bZIP transcription factor	18,1	5,4	**0,43**	3,00E-06
GCHFR	GTP cyclohydrolase I feedback regulator	3,0	0,7	**0,43**	0,002221
RBL1	RB transcriptional corepressor like 1	17,6	4,8	**0,43**	0
ZNF491	zinc finger protein 491	1,5	0,5	**0,42**	0,000209
CKAP2L	cytoskeleton associated protein 2 like	18,4	5,2	**0,42**	0
EPYC	epiphycan	2,6	0,7	**0,42**	0,001674
USP2	ubiquitin specific peptidase 2	3,0	1,0	**0,42**	3,00E-05
RPL23AP49	ribosomal protein L23a pseudogene 49	0,7	0,2	**0,42**	0,001229
MYOM3	myomesin 3	0,6	0,2	**0,42**	0,000889
RHOJ	ras homolog family member J	13,6	3,6	**0,42**	1,20E-05
CNTNAP3B	contactin associated protein-like 3B	1,3	0,3	**0,42**	0,00012

IGDCC4	immunoglobulin superfamily DCC subclass member 4	3,5	1,2	**0,42**	1,70E-05
TNFAIP8L1	TNF alpha induced protein 8 like 1	1,5	0,3	**0,42**	0,003181
SUSD5	sushi domain containing 5	267,9	80,1	**0,42**	0
DLX4	distal-less homeobox 4	5,9	1,5	**0,42**	2,80E-05
NRK	Nik related kinase	30,7	9,2	**0,42**	0
NOV	nephroblastoma overexpressed		7,6	**0,42**	1,70E-05
FKBP1B	FK506 binding protein 1B	1,1	0,3	**0,42**	0,004736
CST6	cystatin E/M	48,0	16,7	**0,42**	5,00E-05
MMS22L	MMS22 like, DNA repair protein	11,3	3,1	**0,42**	0
KDELC2	KDEL motif containing 2	169,2	47,6	**0,42**	0
PCDHB14	protocadherin beta 14	11,2	3,2	**0,42**	2,00E-06
ZC4H2	zinc finger C4H2-type containing	5,1	1,4	**0,42**	1,10E-05
CLLU1	chronic lymphocytic leukemia up-regulated 1	1,0	0,3	**0,42**	0,002983
MDH1B	malate dehydrogenase 1B	2,1	0,6	**0,42**	0,000216
OSBPL1A	oxysterol binding protein like 1A	32,4	9,1	**0,42**	0
ANO7L1	anoctamin 7 like 1	0,7	0,2	**0,42**	0,008073
CHRD	chordin	5,8	1,9	**0,42**	2,80E-05
MATN3	matrilin 3	204,2	59,2	**0,42**	1,40E-05
ADAMTS1	ADAM metallopeptidase with thrombospondin type 1 motif 1	193,3	56,8	**0,42**	9,00E-06
PLXDC2	plexin domain containing 2	512,2	145,8	**0,42**	3,00E-06
SH3TC1	SH3 domain and tetratricopeptide repeats 1	65,9	17,1	**0,42**	1,00E-06
HCFC2	host cell factor C2	31,2	9,0	**0,42**	0
FBLN2	fibulin 2	8,2	2,1	**0,42**	0,000293
CDO1	cysteine dioxygenase type 1	166,1	45,1	**0,42**	3,00E-05
PODXL2	podocalyxin like 2	5,9	1,8	**0,42**	0,000155
ITGA9	integrin subunit alpha 9	10,1	2,9	**0,42**	4,00E-06
ATL1	atlastin GTPase 1	18,9	5,6	**0,42**	0
RAMP1	receptor activity modifying protein 1	19,5	5,2	**0,42**	0,000609
CACNA1H	calcium voltage-gated channel subunit alpha1 H	5,4	1,3	**0,42**	0,014299
TSC22D1	TSC22 domain family member 1	742,9	199,6	**0,42**	4,10E-05
DBNDD1	dysbindin domain containing 1	1,3	0,3	**0,42**	0,001494
TLL1	tolloid like 1	0,8	0,3	**0,42**	0,001681
GALNT10	polypeptide N-acetylgalactosaminyltransferase 10	262,7	72,8	**0,42**	0

VIM	vimentin	5136,6	1583,9	**0,42**	0,000939
ADGRG6	adhesion G protein-coupled receptor G6	115,4	31,0	**0,42**	0
MCMDC2	minichromosome maintenance domain containing 2	1,4	0,4	**0,42**	0,000267
TGFB3	transforming growth factor beta 3	9,1	2,3	**0,42**	5,00E-06
BICC1	BicC family RNA binding protein 1	24,3	6,7	**0,42**	0
NUDT16P1	nudix hydrolase 16 pseudogene 1	7,6	2,3	**0,42**	2,00E-06
DZIP1L	DAZ interacting zinc finger protein 1 like	12,8	3,4	**0,42**	1,00E-06
FAM109B	family with sequence similarity 109 member B			**0,42**	8,00E-06
MZT2A	mitotic spindle organizing protein 2A	19,2	5,7	**0,41**	1,00E-06
PTN	pleiotrophin	5,5	1,7	**0,41**	0,000368
SLC16A4	solute carrier family 16 member 4	16,9	4,8	**0,41**	0
PTK7	protein tyrosine kinase 7 (inactive)	58,2	15,9	**0,41**	0
ZMYM3	zinc finger MYM-type containing 3	19,2	5,4	**0,41**	0
C4orf33	chromosome 4 open reading frame 33	5,6	1,5	**0,41**	6,00E-06
TTYH3	tweety family member 3	11,5	3,6	**0,41**	0
FAM47E	family with sequence similarity 47 member E	2,3	0,7	**0,41**	5,30E-05
BZW1P2	basic leucine zipper and W2 domains 1 pseudogene 2	1,9	0,6	**0,41**	1,40E-05
SLC40A1	solute carrier family 40 member 1	59,8	16,4	**0,41**	0
MLPH	melanophilin	8,6	2,3	**0,41**	0
ZFP69	ZFP69 zinc finger protein	2,8	0,8	**0,41**	3,70E-05
TMEM30B	transmembrane protein 30B	30,5	7,9	**0,41**	0
CTPS2	CTP synthase 2	8,2	2,3	**0,41**	2,00E-06
ADAMTSL2	ADAMTS like 2	114,3	31,2	**0,41**	2,40E-05
PODXL	podocalyxin like	43,2	11,0	**0,41**	5,00E-06
RGS14	regulator of G protein signaling 14	2,5	0,6	**0,41**	0,001032
ZNF815P	zinc finger protein 815, pseudogene	0,9	0,3	**0,41**	0,001086
GPT	glutamic--pyruvic transaminase	2,0	0,5	**0,41**	0,001597
AUNIP	aurora kinase A and ninein interacting protein	1,0	0,2	**0,41**	0,008701
SSBP2	single stranded DNA binding protein 2	26,5	6,9	**0,41**	0
ISYNA1	inositol-3-phosphate synthase 1	12,4	3,4	**0,41**	2,10E-05
PRKAR1B	protein kinase cAMP-dependent type I regulatory subunit beta	13,4	3,5	**0,41**	1,10E-05
NID1	nidogen 1	8,2	2,2	**0,41**	0
MAP3K20	mitogen-activated protein kinase kinase kinase 20	86,0	24,7	**0,41**	0

NFASC	neurofascin	25,7	7,2	**0,41**	0
RASGRF2	Ras protein specific guanine nucleotide releasing factor 2	1,0	0,3	**0,41**	0,002495
NDRG3	NDRG family member 3	23,1	6,0	**0,41**	1,00E-06
PRR29	proline rich 29	1,1	0,3	**0,41**	0,001019
PLA2G4A	phospholipase A2 group IVA	98,1	31,0	**0,41**	0
PCDHB7	protocadherin beta 7	6,5	1,7	**0,41**	1,00E-06
HOXA10-AS	HOXA10 antisense RNA	5,2	1,4	**0,41**	5,70E-05
MEGF6	multiple EGF like domains 6	89,9	24,8	**0,41**	0
DKFZp779M(	uncharacterized DKFZp779M0652		0,1	**0,41**	0,011893
STIL	SCL/TAL1 interrupting locus	9,0	2,2	**0,41**	1,00E-06
FGD1	FYVE, RhoGEF and PH domain containing 1	18,9	5,0	**0,41**	1,00E-06
AEBP1	AE binding protein 1	1147,0	352,8	**0,41**	0,000123
LOXL1	lysyl oxidase like 1	56,5	15,2	**0,41**	1,00E-06
KIF5A	kinesin family member 5A	0,8	0,2	**0,41**	0,01714
C14orf159	chromosome 14 open reading frame 159			**0,41**	5,00E-06
CLEC11A	C-type lectin domain containing 11A	19,7	5,6	**0,41**	3,00E-06
S100A1	S100 calcium binding protein A1	537,5	147,8	**0,40**	0,000254
TASP1	taspase 1	18,1	4,6	**0,40**	2,00E-06
BARX1	BARX homeobox 1	86,1	23,6	**0,40**	1,00E-06
LINC01503	long intergenic non-protein coding RNA 1503	17,5	5,1	**0,40**	3,70E-05
NIPSNAP3A	nipsnap homolog 3A	25,4	6,9	**0,40**	1,00E-06
ARHGEF25	Rho guanine nucleotide exchange factor 25	5,2	1,4	**0,40**	0,000177
GPR162	G protein-coupled receptor 162	2,1	0,6	**0,40**	3,30E-05
HECW1	HECT, C2 and WW domain containing E3 ubiquitin protein ligase 1	6,6	1,8	**0,40**	3,00E-06
NAP1L3	nucleosome assembly protein 1 like 3	25,8	6,7	**0,40**	0
AK1	adenylate kinase 1	46,4	12,8	**0,40**	0
SCX	scleraxis bHLH transcription factor	8,6	2,5	**0,40**	0,001874
FXYD2	FXYD domain containing ion transport regulator 2	0,7	0,3	**0,40**	0,000316
CRACR2A	calcium release activated channel regulator 2A	0,7	0,2	**0,40**	0,001585
KIAA1524	KIAA1524			**0,40**	0
SLC38A3	solute carrier family 38 member 3	36,7	9,4	**0,40**	6,00E-06
DNAJC22	DnaJ heat shock protein family (Hsp40) member C22	6,8	1,6	**0,40**	1,00E-05
ATAD3C	ATPase family, AAA domain containing 3C	2,6	0,8	**0,40**	7,00E-06

GSTM4	glutathione S-transferase mu 4	11,0	2,8	**0,40**	3,40E-05
MUM1L1	MUM1 like 1		0,6	**0,40**	0,000544
CLEC18B	C-type lectin domain family 18 member B	1,5	0,3	**0,40**	0,006408
IQSEC1	IQ motif and Sec7 domain 1	186,3	47,1	**0,40**	0
MRPL23	mitochondrial ribosomal protein L23	5,0	1,4	**0,40**	0,000703
CTSC	cathepsin C	85,0	23,1	**0,40**	3,00E-06
RGS2	regulator of G protein signaling 2	181,1	51,0	**0,40**	0
C1orf145	chromosome 1 open reading frame 145			**0,40**	0,003825
PAK1	p21 (RAC1) activated kinase 1	18,4	4,7	**0,40**	0
C12orf60	chromosome 12 open reading frame 60	49,2	13,1	**0,40**	7,00E-06
ADAMTSL3	ADAMTS like 3	111,1	29,3	**0,40**	0
CGREF1	cell growth regulator with EF-hand domain 1	34,6	9,1	**0,40**	1,00E-06
ACSF2	acyl-CoA synthetase family member 2	8,8	2,4	**0,40**	3,40E-05
BRCA1	BRCA1, DNA repair associated	22,3	5,1	**0,40**	0
CYP27C1	cytochrome P450 family 27 subfamily C member 1	20,1	5,3	**0,40**	0
ASIC1	acid sensing ion channel subunit 1	2,0	0,5	**0,40**	0,002175
COL14A1	collagen type XIV alpha 1 chain	52,6	13,3	**0,40**	0
BMF	Bcl2 modifying factor	35,8	9,2	**0,40**	0
ETNK2	ethanolamine kinase 2	11,4	2,8	**0,40**	2,00E-06
FNBP1L	formin binding protein 1 like	8,6	2,4	**0,40**	1,00E-06
PDIA5	protein disulfide isomerase family A member 5	55,6	15,3	**0,40**	5,00E-06
CRIM1	cysteine rich transmembrane BMP regulator 1	602,3	165,2	**0,40**	2,00E-06
NTHL1	nth like DNA glycosylase 1	13,5	3,5	**0,40**	1,40E-05
SLC25A23	solute carrier family 25 member 23	19,8	5,3	**0,40**	0
SFXN3	sideroflexin 3	71,1	18,7	**0,40**	0
NBL1	neuroblastoma 1, DAN family BMP antagonist	88,5	21,8	**0,40**	8,00E-06
FOXP2	forkhead box P2	13,2	3,5	**0,40**	0
LY6K	lymphocyte antigen 6 family member K	1,6	0,3	**0,40**	0,010644
SALL2	spalt like transcription factor 2	1,8	0,7	**0,39**	5,00E-06
SNTA1	syntrophin alpha 1	15,0	3,8	**0,39**	1,70E-05
CDK18	cyclin dependent kinase 18	3,7	0,9	**0,39**	9,30E-05
MYO1D	myosin ID	656,8	166,3	**0,39**	1,00E-06
NEURL1	neuralized E3 ubiquitin protein ligase 1	3,1	0,8	**0,39**	1,00E-04

SSPN	sarcospan	34,9	8,7	**0,39**	1,00E-06
FAM198A	family with sequence similarity 198 member A		0,4	**0,39**	9,20E-05
LMOD3	leiomodin 3	1,1	0,2	**0,39**	0,002139
TRIM59	tripartite motif containing 59	8,4	2,2	**0,39**	0
CCDC80	coiled-coil domain containing 80	1079,1	298,2	**0,39**	2,60E-05
C11orf95	chromosome 11 open reading frame 95	44,4	11,9	**0,39**	0
TMEM56	transmembrane protein 56	3,6	0,7	**0,39**	0,000535
NFAM1	NFAT activating protein with ITAM motif 1	0,7	0,2	**0,39**	0,000346
PCDHB5	protocadherin beta 5	7,6	2,1	**0,39**	0
CDHR3	cadherin related family member 3	0,6	0,1	**0,39**	0,002207
MYBPH	myosin binding protein H	9,8	2,6	**0,39**	2,00E-06
HTRA3	HtrA serine peptidase 3	45,4	12,5	**0,39**	7,00E-06
TPRG1	tumor protein p63 regulated 1	5,1	1,3	**0,39**	2,00E-06
RTL3	retrotransposon Gag like 3	14,2	3,2	**0,39**	8,00E-05
SLC8A1	solute carrier family 8 member A1	39,7	10,0	**0,39**	5,00E-06
MFAP4	microfibrillar associated protein 4	5,4	1,5	**0,39**	8,20E-05
CRABP2	cellular retinoic acid binding protein 2	3,4	0,7	**0,39**	0,001108
FHIT	fragile histidine triad	2,2	0,6	**0,39**	2,00E-06
CHEK1	checkpoint kinase 1	13,1	3,0	**0,39**	0
STMN3	stathmin 3	21,3	5,7	**0,39**	0,000563
TEAD2	TEA domain transcription factor 2	19,0	5,2	**0,39**	0
MALL	mal, T-cell differentiation protein like	23,5	5,8	**0,39**	0
KIAA1456	KIAA1456			**0,39**	0,000755
KCND1	potassium voltage-gated channel subfamily D member 1	1,2	0,3	**0,39**	0,000618
CENPO	centromere protein O	22,6	5,3	**0,39**	0
LDHD	lactate dehydrogenase D	7,7	1,8	**0,39**	9,20E-05
CD8B2	CD8b2 molecule	8,5	2,2	**0,39**	4,00E-06
MGST2	microsomal glutathione S-transferase 2	1,9	0,4	**0,39**	0,002267
PHYHD1	phytanoyl-CoA dioxygenase domain containing 1	7,4	1,8	**0,39**	5,00E-06
PCDHB6	protocadherin beta 6	7,1	2,0	**0,39**	3,00E-06
STOM	stomatin	188,7	50,7	**0,39**	0
HSD17B6	hydroxysteroid 17-beta dehydrogenase 6	0,8	0,1	**0,39**	0,002373
SIX2	SIX homeobox 2	29,9	7,8	**0,39**	1,00E-06

MCM6	minichromosome maintenance complex component 6	82,6	18,7	**0,39**	0
TPM4	tropomyosin 4	272,1	69,4	**0,39**	0
SCIN	scinderin	261,1	62,2	**0,39**	1,00E-06
CAPN1	calpain 1	52,3	14,4	**0,39**	0
SNX10	sorting nexin 10	2,7	0,6	**0,38**	1,40E-05
GAB1	GRB2 associated binding protein 1	46,9	12,1	**0,38**	0
IBSP	integrin binding sialoprotein	53,2	17,6	**0,38**	0,001794
RAMP3	receptor activity modifying protein 3	1,7	0,4	**0,38**	0,001654
ARSE	arylsulfatase E (chondrodysplasia punctata 1)	2,1	0,5	**0,38**	0,000568
BOC	BOC cell adhesion associated, oncogene regulated	111,6	27,6	**0,38**	0
AP1S3	adaptor related protein complex 1 sigma 3 subunit	4,2	1,1	**0,38**	4,00E-06
CCDC28B	coiled-coil domain containing 28B	4,7	1,1	**0,38**	1,50E-05
NEXN	nexilin F-actin binding protein	7,5	2,1	**0,38**	7,00E-06
ADAMTS5	ADAM metallopeptidase with thrombospondin type 1 motif 5	61,9	21,3	**0,38**	0
RNF144A	ring finger protein 144A	7,6	1,9	**0,38**	3,00E-06
HYAL1	hyaluronoglucosaminidase 1	39,5	9,0	**0,38**	4,00E-06
RAB9B	RAB9B, member RAS oncogene family	0,8	0,2	**0,38**	0,003205
ADIRF	adipogenesis regulatory factor	180,9	49,4	**0,38**	7,40E-05
TONSL	tonsoku like, DNA repair protein	6,9	1,6	**0,38**	1,00E-05
SSH3	slingshot protein phosphatase 3	21,3	5,6	**0,38**	0
SIPA1L1	signal induced proliferation associated 1 like 1	158,0	39,0	**0,38**	0
CARD9	caspase recruitment domain family member 9	7,4	1,9	**0,38**	4,90E-05
TUBB2A	tubulin beta 2A class IIa	56,1	12,9	**0,38**	8,00E-06
CRYBG1	crystallin beta-gamma domain containing 1	5,4	1,3	**0,38**	0,000714
ST3GAL5	ST3 beta-galactoside alpha-2,3-sialyltransferase 5	3,9	0,9	**0,38**	0,000114
KIF3C	kinesin family member 3C	15,7	4,0	**0,38**	0
PRRT4	proline rich transmembrane protein 4	0,7	0,2	**0,38**	0,000607
ZNF512B	zinc finger protein 512B	22,5	5,6	**0,38**	0
MAPK12	mitogen-activated protein kinase 12	9,9	2,4	**0,38**	2,00E-06
SGCA	sarcoglycan alpha	5,1	1,3	**0,38**	0,000263
MAN1C1	mannosidase alpha class 1C member 1	12,4	3,3	**0,38**	3,00E-06
IQSEC3	IQ motif and Sec7 domain 3	0,7	0,2	**0,38**	0,000582
EMILIN1	elastin microfibril interfacer 1	463,3	130,7	**0,38**	1,50E-05

EBPL	emopamil binding protein like	18,3	4,7	**0,38**	1,10E-05
SPAG5	sperm associated antigen 5	21,9	4,6	**0,38**	0
GLIPR1	GLI pathogenesis related 1	137,3	35,5	**0,38**	0
PCDHB16	protocadherin beta 16	22,7	6,1	**0,38**	0
RAPGEF5	Rap guanine nucleotide exchange factor 5	11,4	2,8	**0,38**	6,00E-06
ATP8B1	ATPase phospholipid transporting 8B1	15,2	4,0	**0,38**	0
FABP3	fatty acid binding protein 3	20,0	4,1	**0,38**	2,10E-05
LRP3	LDL receptor related protein 3	36,1	9,0	**0,38**	0
ERFE	erythroferrone	25,9	6,4	**0,38**	2,00E-06
GPR88	G protein-coupled receptor 88	146,3	43,4	**0,38**	0
FAM78B	family with sequence similarity 78 member B	2,6	0,6	**0,38**	3,00E-06
TFDP1	transcription factor Dp-1	82,2	20,1	**0,38**	0
NUDT8	nudix hydrolase 8	4,1	1,0	**0,37**	0,000411
PCDHB8	protocadherin beta 8	0,8	0,2	**0,37**	0,003452
KIF20B	kinesin family member 20B	14,7	3,3	**0,37**	0
TNFSF12	TNF superfamily member 12	8,8	2,2	**0,37**	1,40E-05
NCAPG2	non-SMC condensin II complex subunit G2	35,0	7,9	**0,37**	0
SLC19A1	solute carrier family 19 member 1	8,2	1,9	**0,37**	4,00E-06
RXRA	retinoid X receptor alpha	147,9	35,3	**0,37**	0
LXN	latexin	7,4	1,8	**0,37**	3,00E-06
LIG1	DNA ligase 1	21,9	4,8	**0,37**	2,00E-06
PCDHB15	protocadherin beta 15	6,2	1,8	**0,37**	0
STEAP4	STEAP4 metalloreductase	75,0	24,2	**0,37**	0
TGFBR3L	transforming growth factor beta receptor 3 like	1,8	0,3	**0,37**	0,001376
PTGER3	prostaglandin E receptor 3	20,8	5,0	**0,37**	3,00E-06
GARNL3	GTPase activating Rap/RanGAP domain like 3	3,5	0,8	**0,37**	0,000313
PIP5KL1	phosphatidylinositol-4-phosphate 5-kinase like 1	11,6	2,9	**0,37**	5,00E-06
CLSPN	claspin	13,8	3,0	**0,37**	3,00E-06
KAZN	kazrin, periplakin interacting protein	7,4	1,7	**0,37**	0,000149
LIMCH1	LIM and calponin homology domains 1	113,5	27,3	**0,37**	0
THBS1	thrombospondin 1	1421,4	336,9	**0,37**	9,00E-06
CFAP54	cilia and flagella associated protein 54	7,3	1,7	**0,37**	1,00E-06
LPAR4	lysophosphatidic acid receptor 4	9,2	2,2	**0,37**	0

SPTSSB	serine palmitoyltransferase small subunit B	6,8	1,5	**0,37**	3,10E-05
ECHDC3	enoyl-CoA hydratase domain containing 3	12,6	3,6	**0,37**	8,00E-06
PDLIM2	PDZ and LIM domain 2	36,0	8,1	**0,37**	0
FER1L4	fer-1 like family member 4, pseudogene	2,1	0,5	**0,37**	1,30E-05
CYBRD1	cytochrome b reductase 1	594,0	141,7	**0,37**	2,00E-06
IFITM3	interferon induced transmembrane protein 3	237,6	60,7	**0,37**	1,00E-06
GLI3	GLI family zinc finger 3	67,0	17,2	**0,37**	0
TUBA1B	tubulin alpha 1b	588,4	128,6	**0,37**	3,00E-06
KAT2B	lysine acetyltransferase 2B	33,5	8,1	**0,37**	0
CNPY4	canopy FGF signaling regulator 4	29,8	7,0	**0,37**	0
KCNG2	potassium voltage-gated channel modifier subfamily G member 2	1,7	0,4	**0,37**	0,000212
MCM5	minichromosome maintenance complex component 5	74,0	16,0	**0,37**	0
PCDHB12	protocadherin beta 12	3,0	0,7	**0,37**	4,00E-05
DEPDC1B	DEP domain containing 1B	2,7	0,5	**0,37**	2,70E-05
TNFAIP8L3	TNF alpha induced protein 8 like 3	18,0	4,3	**0,37**	1,50E-05
ABHD14A	abhydrolase domain containing 14A	3,5	0,9	**0,37**	0,000215
NAALADL2	N-acetylated alpha-linked acidic dipeptidase like 2	5,4	1,4	**0,36**	3,00E-06
CA11	carbonic anhydrase 11	6,8	1,5	**0,36**	1,80E-05
ETV1	ETS variant 1	13,4	3,2	**0,36**	0
PHLDB1	pleckstrin homology like domain family B member 1	172,3	40,6	**0,36**	0
FAXDC2	fatty acid hydroxylase domain containing 2	19,8	4,4	**0,36**	2,00E-06
CNTFR	ciliary neurotrophic factor receptor	21,1	5,3	**0,36**	1,00E-06
DEF6	DEF6, guanine nucleotide exchange factor	4,3	0,9	**0,36**	1,70E-05
GDF7	growth differentiation factor 7	2,0	0,5	**0,36**	3,00E-06
NEIL1	nei like DNA glycosylase 1	7,3	1,6	**0,36**	1,10E-05
MAP2K6	mitogen-activated protein kinase kinase 6	18,5	3,5	**0,36**	2,00E-05
TAGLN	transgelin	16,7	3,3	**0,36**	4,00E-05
RCOR2	REST corepressor 2	2,2	0,4	**0,36**	0,000629
ANGPTL5	angiopoietin like 5	8,6	2,1	**0,36**	2,20E-05
ITGBL1	integrin subunit beta like 1	69,8	17,5	**0,36**	4,00E-06
EFHD1	EF-hand domain family member D1	56,4	12,5	**0,36**	1,00E-06
PPP1R1C	protein phosphatase 1 regulatory inhibitor subunit 1C	0,6	0,1	**0,36**	0,000422
HPDL	4-hydroxyphenylpyruvate dioxygenase like	1,4	0,2	**0,36**	0,003668

CNN3	calponin 3	223,1	51,9	**0,36**	0
EPHB4	EPH receptor B4	33,1	7,9	**0,36**	0
B3GALNT1	beta-1,3-N-acetylgalactosaminyltransferase 1 (globoside blood group)	21,8	5,1	**0,36**	0
GOLM1	golgi membrane protein 1	610,8	155,4	**0,36**	1,00E-06
NXPE3	neurexophilin and PC-esterase domain family member 3	33,6	8,2	**0,36**	0
C9orf50	chromosome 9 open reading frame 50	2,3	0,4	**0,36**	9,20E-05
LHX9	LIM homeobox 9	2,6	0,7	**0,36**	0
CAT	catalase	59,4	14,3	**0,36**	0
SSBP4	single stranded DNA binding protein 4	61,7	14,2	**0,36**	0
6.syys	septin 6			**0,36**	0
GALNT16	polypeptide N-acetylgalactosaminyltransferase 16	12,4	3,2	**0,36**	5,00E-06
KCNIP3	potassium voltage-gated channel interacting protein 3	3,9	1,0	**0,36**	0,000106
LDLRAD4	low density lipoprotein receptor class A domain containing 4	37,5	8,2	**0,36**	0
VIT	vitrin	6,1	1,4	**0,36**	5,00E-06
KLHDC9	kelch domain containing 9	1,9	0,5	**0,36**	4,10E-05
S100A10	S100 calcium binding protein A10	298,6	74,8	**0,36**	2,00E-06
CDC42EP1	CDC42 effector protein 1	29,9	6,3	**0,36**	3,00E-06
GSTA4	glutathione S-transferase alpha 4	36,4	8,0	**0,36**	0
HIP1	huntingtin interacting protein 1	76,5	16,8	**0,36**	0
TMEM121	transmembrane protein 121	7,1	1,6	**0,36**	7,20E-05
TCEAL5	transcription elongation factor A like 5	1,2	0,2	**0,36**	0,00087
CERCAM	cerebral endothelial cell adhesion molecule	153,5	34,9	**0,36**	0
OSBPL7	oxysterol binding protein like 7		1,5	**0,36**	1,00E-05
TRO	trophinin	13,7	3,2	**0,36**	0
PICSAR	P38 inhibited cutaneous squamous cell carcinoma associated lincRNA	1,0	0,2	**0,36**	0,001287
NREP	neuronal regeneration related protein	50,9	12,1	**0,36**	0
PALD1	phosphatase domain containing, paladin 1	24,5	5,3	**0,36**	0
LINC02298	long intergenic non-protein coding RNA 2298	0,9	0,1	**0,36**	0,001186
B3GNT9	UDP-GlcNAc:betaGal beta-1,3-N-acetylglucosaminyltransferase 9	68,2	15,2	**0,36**	0
IGFBP2	insulin like growth factor binding protein 2	27,1	4,8	**0,35**	0,000266
PDE11A	phosphodiesterase 11A	1,9	0,3	**0,35**	0,000187
MRGPRF	MAS related GPR family member F	33,1	6,9	**0,35**	8,00E-06
LZTS2	leucine zipper tumor suppressor 2	85,3	19,7	**0,35**	0

NRSN2	neurensin 2	47,5	11,1	**0,35**	0
TCF7	transcription factor 7 (T-cell specific, HMG-box)	9,5	2,0	**0,35**	2,60E-05
ACTG1	actin gamma 1	2050,1	489,6	**0,35**	0,000135
KCNE4	potassium voltage-gated channel subfamily E regulatory subunit 4	8,5	2,1	**0,35**	1,60E-05
RRM1	ribonucleotide reductase catalytic subunit M1	85,9	18,8	**0,35**	0
ASF1B	anti-silencing function 1B histone chaperone	14,5	2,8	**0,35**	1,10E-05
ADAMTSL4	ADAMTS like 4	12,8	3,2	**0,35**	0
MYLK	myosin light chain kinase	12,6	2,7	**0,35**	5,00E-06
UMODL1	uromodulin like 1	0,8	0,3	**0,35**	0,003165
UBE2T	ubiquitin conjugating enzyme E2 T	12,3	2,4	**0,35**	4,00E-06
FBXL2	F-box and leucine rich repeat protein 2	13,8	3,3	**0,35**	0
NCALD	neurocalcin delta	0,7	0,1	**0,35**	0,00129
TRIM14	tripartite motif containing 14	10,1	2,1	**0,35**	0
ATP2A3	ATPase sarcoplasmic/endoplasmic reticulum Ca2+ transporting 3	3,9	0,9	**0,35**	3,00E-05
PRKCQ-AS1	PRKCQ antisense RNA 1	4,5	1,0	**0,35**	5,70E-05
ARPIN	actin-related protein 2/3 complex inhibitor	26,1	6,1	**0,35**	0
MGP	matrix Gla protein	11525,5	2736,0	**0,35**	0,000825
HHIPL1	HHIP like 1	103,7	24,9	**0,35**	0
CEP128	centrosomal protein 128	3,9	0,8	**0,35**	1,00E-06
ENPP1	ectonucleotide pyrophosphatase/phosphodiesterase 1	404,8	93,3	**0,35**	1,00E-06
DLG3	discs large MAGUK scaffold protein 3	11,0	2,4	**0,35**	0
APOBEC3B	apolipoprotein B mRNA editing enzyme catalytic subunit 3B	4,4	1,1	**0,35**	2,10E-05
SCFD2	sec1 family domain containing 2	20,1	4,5	**0,35**	0
PRCD	photoreceptor disc component	2,5	0,5	**0,35**	3,30E-05
CAB39L	calcium binding protein 39 like	51,0	10,8	**0,35**	0
NINJ2	ninjurin 2	3,1	0,7	**0,35**	0,000219
CAMK2B	calcium/calmodulin dependent protein kinase II beta	2,2	0,5	**0,35**	7,00E-06
TET1	tet methylcytosine dioxygenase 1	2,1	0,5	**0,35**	4,60E-05
A2M-AS1	A2M antisense RNA 1 (head to head)	2,7	0,6	**0,35**	0,000426
VAT1	vesicle amine transport 1	411,1	92,7	**0,35**	0
COL10A1	collagen type X alpha 1 chain	418,9	126,9	**0,35**	0,000227
PLEKHG5	pleckstrin homology and RhoGEF domain containing G5	2,4	0,5	**0,35**	4,00E-06
PABPC4L	poly(A) binding protein cytoplasmic 4 like	1,6	0,4	**0,35**	0,000182

SNED1	sushi, nidogen and EGF like domains 1	138,1	33,9	**0,35**	0
HAUS7	HAUS augmin like complex subunit 7	1,1	0,3	**0,35**	6,70E-05
MAPK3	mitogen-activated protein kinase 3	81,7	19,7	**0,34**	0
GPNMB	glycoprotein nmb	31,4	7,3	**0,34**	0
OSR1	odd-skipped related transciption factor 1	3,3	0,7	**0,34**	0,002522
DDAH1	dimethylarginine dimethylaminohydrolase 1	135,3	29,4	**0,34**	0
ANO1	anoctamin 1	4,7	0,9	**0,34**	2,10E-05
LYPD5	LY6/PLAUR domain containing 5	2,0	0,4	**0,34**	1,50E-05
FAM212A	family with sequence similarity 212 member A			**0,34**	1,80E-05
HMGCLL1	3-hydroxymethyl-3-methylglutaryl-CoA lyase like 1	0,8	0,1	**0,34**	0,000886
GPC6	glypican 6	731,4	162,4	**0,34**	1,00E-06
SHISA4	shisa family member 4	64,2	14,3	**0,34**	2,00E-06
IL12RB2	interleukin 12 receptor subunit beta 2	4,1	0,9	**0,34**	1,00E-05
BLM	Bloom syndrome RecQ like helicase	4,5	0,9	**0,34**	2,00E-05
HAAO	3-hydroxyanthranilate 3,4-dioxygenase	4,1	0,9	**0,34**	2,00E-06
ITM2A	integral membrane protein 2A	85,1	23,1	**0,34**	0
NUPR2	nuclear protein 2, transcriptional regulator	0,6	0,1	**0,34**	0,007441
FKBP7	FK506 binding protein 7	25,5	5,3	**0,34**	0
LTBP1	latent transforming growth factor beta binding protein 1	324,1	77,4	**0,34**	1,00E-06
APOBEC3C	apolipoprotein B mRNA editing enzyme catalytic subunit 3C	16,6	3,6	**0,34**	3,00E-06
SNHG18	small nucleolar RNA host gene 18	6,9	1,5	**0,34**	7,00E-06
OIP5	Opa interacting protein 5	2,5	0,4	**0,34**	0,000331
CREB3L1	cAMP responsive element binding protein 3 like 1	20,6	4,7	**0,34**	0
SMARCA1	SWI/SNF related, matrix associated, actin dependent regulator of chrc	101,6	23,0	**0,34**	0
SSX2IP	SSX family member 2 interacting protein	19,5	3,9	**0,34**	0
OBSCN	obscurin, cytoskeletal calmodulin and titin-interacting RhoGEF	17,4	3,8	**0,34**	0
ZNF107	zinc finger protein 107	7,5	1,7	**0,34**	0
ADCY1	adenylate cyclase 1	4,6	1,0	**0,34**	0,000117
C10orf105	chromosome 10 open reading frame 105	14,1	3,3	**0,34**	9,00E-06
LRRC3	leucine rich repeat containing 3	2,0	0,5	**0,34**	0,000356
S100A4	S100 calcium binding protein A4	50,8	10,0	**0,34**	5,00E-06
LRRC45	leucine rich repeat containing 45	10,2	2,1	**0,34**	0
NXNL2	nucleoredoxin-like 2	0,9	0,2	**0,34**	0,000405

KIF6	kinesin family member 6	1,0	0,3	**0,34**	8,00E-06
PDZD7	PDZ domain containing 7	1,3	0,3	**0,34**	0,004901
PRAG1	PEAK1 related kinase activating pseudokinase 1	0,9	0,2	**0,34**	0,001334
DPYSL3	dihydropyrimidinase like 3	150,0	32,1	**0,34**	0
MAP3K6	mitogen-activated protein kinase kinase kinase 6	30,3	6,7	**0,34**	0
MTMR11	myotubularin related protein 11	20,9	4,7	**0,34**	0
DOK7	docking protein 7	9,2	1,5	**0,34**	6,30E-05
ITGB4	integrin subunit beta 4	3,4	0,8	**0,34**	1,00E-06
LMO3	LIM domain only 3	0,8	0,2	**0,34**	0,001553
SHMT1	serine hydroxymethyltransferase 1	4,3	1,0	**0,33**	2,00E-06
MAD2L1	mitotic arrest deficient 2 like 1	12,5	2,2	**0,33**	0
CISH	cytokine inducible SH2 containing protein	2,4	0,6	**0,33**	3,00E-06
COLEC12	collectin subfamily member 12	106,7	24,4	**0,33**	4,00E-06
SAMD1	sterile alpha motif domain containing 1	33,4	7,5	**0,33**	0
SOX6	SRY-box 6	17,3	3,4	**0,33**	0
CCDC8	coiled-coil domain containing 8	33,8	7,1	**0,33**	0
CENPK	centromere protein K	8,9	1,8	**0,33**	0
STAB1	stabilin 1	11,5	2,7	**0,33**	0
CENPP	centromere protein P	9,8	2,2	**0,33**	0
COL9A1	collagen type IX alpha 1 chain	45,1	10,3	**0,33**	1,50E-05
PCDHGC4	protocadherin gamma subfamily C, 4	1,5	0,3	**0,33**	1,10E-05
KIAA1841	KIAA1841	17,9	4,1	**0,33**	0
DIRAS1	DIRAS family GTPase 1	12,8	2,8	**0,33**	2,00E-06
EMILIN3	elastin microfibril interfacer 3	7,8	1,4	**0,33**	4,10E-05
F13A1	coagulation factor XIII A chain	360,5	72,2	**0,33**	1,00E-06
HRASLS5	HRAS like suppressor family member 5	0,7	0,1	**0,33**	0,000687
CHI3L1	chitinase 3 like 1	10285,2	2272,8	**0,33**	0,000143
MBNL1-AS1	MBNL1 antisense RNA 1	43,9	9,2	**0,33**	0
FAT4	FAT atypical cadherin 4	69,7	15,7	**0,33**	0
PBX1	PBX homeobox 1	29,8	6,6	**0,33**	0
CLDN23	claudin 23	0,7	0,2	**0,33**	0,0177
PTPRF	protein tyrosine phosphatase, receptor type F	72,8	15,5	**0,33**	0
LRP5	LDL receptor related protein 5	61,3	12,2	**0,32**	0

FMO5	flavin containing monooxygenase 5	2,2	0,5	**0,32**	2,00E-06
MAMSTR	MEF2 activating motif and SAP domain containing transcriptional regu	2,8	0,5	**0,32**	0,000399
DIXDC1	DIX domain containing 1	75,8	15,0	**0,32**	0
SNAI2	snail family transcriptional repressor 2	71,2	15,1	**0,32**	0
EFS	embryonal Fyn-associated substrate	46,1	9,1	**0,32**	0
OGN	osteoglycin	390,0	87,7	**0,32**	6,00E-06
SMOC2	SPARC related modular calcium binding 2	1434,7	340,0	**0,32**	2,50E-05
GPER1	G protein-coupled estrogen receptor 1	20,4	4,0	**0,32**	7,00E-06
EPB41L1	erythrocyte membrane protein band 4.1 like 1	82,2	16,3	**0,32**	0
SLC29A1	solute carrier family 29 member 1 (Augustine blood group)	373,1	77,7	**0,32**	0
DHFR	dihydrofolate reductase	18,4	3,4	**0,32**	0
PAFAH1B3	platelet activating factor acetylhydrolase 1b catalytic subunit 3	10,5	2,1	**0,32**	1,60E-05
ADCY7	adenylate cyclase 7	19,5	3,8	**0,32**	0
NPAS3	neuronal PAS domain protein 3	6,1	1,2	**0,32**	3,00E-06
ITIH6	inter-alpha-trypsin inhibitor heavy chain family member 6	330,3	72,3	**0,32**	0
FAM133A	family with sequence similarity 133 member A	1,5	0,3	**0,32**	6,40E-05
TMTC1	transmembrane and tetratricopeptide repeat containing 1	267,6	54,7	**0,32**	0
SULT1A1	sulfotransferase family 1A member 1	1,7	0,4	**0,32**	1,00E-06
COL8A1	collagen type VIII alpha 1 chain	4,5	0,9	**0,32**	1,00E-06
HAS2-AS1	HAS2 antisense RNA 1	4,2	0,9	**0,32**	1,00E-06
FGFR2	fibroblast growth factor receptor 2	127,3	24,6	**0,32**	0
KCNJ12	potassium voltage-gated channel subfamily J member 12	8,9	2,2	**0,32**	1,00E-06
GCK	glucokinase	1,1	0,2	**0,32**	0,001227
ENPP2	ectonucleotide pyrophosphatase/phosphodiesterase 2	63,2	13,7	**0,32**	0
MPO	myeloperoxidase	0,7	0,1	**0,32**	0,001076
ZBTB47	zinc finger and BTB domain containing 47	38,7	8,5	**0,32**	0
CYSLTR1	cysteinyl leukotriene receptor 1	3,7	0,7	**0,32**	5,70E-05
ADAM21	ADAM metallopeptidase domain 21	1,5	0,4	**0,32**	6,50E-05
KIF7	kinesin family member 7	20,6	4,0	**0,32**	0
PHOSPHO1	phosphoethanolamine/phosphocholine phosphatase	6,1	0,9	**0,32**	0,017645
PNMA8A	paraneoplastic Ma antigen family member 8A	9,0	2,2	**0,32**	0,000313
PAQR4	progestin and adipoQ receptor family member 4	25,2	4,7	**0,32**	0
RND2	Rho family GTPase 2	0,9	0,2	**0,32**	0,000489

KIF4A	kinesin family member 4A	13,6	2,2	**0,32**	0
TP53I11	tumor protein p53 inducible protein 11	7,4	1,4	**0,31**	0
TUB	tubby bipartite transcription factor	15,2	3,0	**0,31**	0
LGR6	leucine rich repeat containing G protein-coupled receptor 6	2,4	0,4	**0,31**	0,000147
TMEM200A	transmembrane protein 200A	4,9	1,1	**0,31**	5,00E-06
LBH	limb bud and heart development	184,1	36,3	**0,31**	0
PLCB4	phospholipase C beta 4	4,7	0,9	**0,31**	8,00E-06
SLC44A2	solute carrier family 44 member 2	291,2	58,4	**0,31**	0
CD52	CD52 molecule	1,0	0,3	**0,31**	0,00068
KIF14	kinesin family member 14	7,8	1,5	**0,31**	0
CASC10	cancer susceptibility 10	5,4	1,0	**0,31**	3,00E-06
EVL	Enah/Vasp-like	17,0	3,6	**0,31**	0
GPSM1	G protein signaling modulator 1	114,3	21,5	**0,31**	0
TBX4	T-box 4	45,8	8,6	**0,31**	0
LINC02095	long intergenic non-protein coding RNA 2095			**0,31**	0,001368
GLT8D2	glycosyltransferase 8 domain containing 2	53,2	10,4	**0,31**	0
FBXO41	F-box protein 41	6,5	1,4	**0,31**	0
WWTR1	WW domain containing transcription regulator 1	468,3	90,8	**0,31**	0
NAT2	N-acetyltransferase 2	2,5	0,5	**0,31**	1,00E-05
C1orf167	chromosome 1 open reading frame 167	1,6	0,3	**0,31**	0,000167
IHH	indian hedgehog	1,7	0,1	**0,31**	0,022806
ADGRL1	adhesion G protein-coupled receptor L1	8,1	1,6	**0,31**	3,00E-06
MTUS1	microtubule associated scaffold protein 1	144,6	29,6	**0,31**	0
LZTS1	leucine zipper tumor suppressor 1	19,5	3,3	**0,31**	5,00E-06
CRIP2	cysteine rich protein 2	207,6	42,0	**0,31**	1,00E-06
ARSI	arylsulfatase family member I	9,5	1,7	**0,31**	1,00E-06
ADCY3	adenylate cyclase 3	81,3	15,2	**0,31**	0
BEND6	BEN domain containing 6	20,4	4,1	**0,31**	0
GHR	growth hormone receptor	35,1	7,2	**0,31**	0
TPD52L1	tumor protein D52 like 1	223,2	40,2	**0,31**	0
LRRN4CL	LRRN4 C-terminal like	24,1	4,4	**0,30**	0
ATP10A	ATPase phospholipid transporting 10A (putative)	67,0	11,5	**0,30**	0
LMO7-AS1	LMO7 antisense RNA 1	1,0	0,2	**0,30**	0,000291

PDE4A	phosphodiesterase 4A		3,2	**0,30**	0
FNDC10	fibronectin type III domain containing 10	4,7	1,0	**0,30**	1,30E-05
RNF112	ring finger protein 112	2,2	0,4	**0,30**	0
SERPINH1	serpin family H member 1	298,3	54,0	**0,30**	0
TCEAL2	transcription elongation factor A like 2	32,9	5,9	**0,30**	0
NFATC2	nuclear factor of activated T-cells 2	18,3	3,1	**0,30**	0
FAM110D	family with sequence similarity 110 member D	0,6	0,2	**0,30**	0,00371
SRPX	sushi repeat containing protein, X-linked	6,2	1,2	**0,30**	2,20E-05
MACROD1	MACRO domain containing 1	11,6	2,1	**0,30**	3,00E-06
FAIM2	Fas apoptotic inhibitory molecule 2	4,5	0,9	**0,30**	9,50E-05
RASL12	RAS like family 12	3,9	0,7	**0,30**	1,00E-06
SCARF2	scavenger receptor class F member 2	55,0	10,1	**0,30**	0
COL12A1	collagen type XII alpha 1 chain	989,9	205,5	**0,30**	0
DNM3OS	DNM3 opposite strand/antisense RNA	21,6	4,0	**0,30**	0
MYOZ3	myozenin 3	1,0	0,1	**0,30**	0,009877
NOG	noggin	21,7	4,9	**0,30**	0
KDELR3	KDEL endoplasmic reticulum protein retention receptor 3	107,3	20,5	**0,30**	0
RAB7B	RAB7B, member RAS oncogene family	3,0	0,6	**0,30**	5,00E-06
PRRT2	proline rich transmembrane protein 2	0,7	0,2	**0,30**	5,00E-06
ATP8B3	ATPase phospholipid transporting 8B3	1,4	0,2	**0,30**	0,000194
TBXA2R	thromboxane A2 receptor	4,2	0,8	**0,30**	6,00E-06
ANGPT1	angiopoietin 1	0,6	0,1	**0,30**	0,000907
CLIC3	chloride intracellular channel 3	15,3	2,8	**0,30**	3,00E-06
HCFC1R1	host cell factor C1 regulator 1	45,4	8,3	**0,30**	0
CCNE2	cyclin E2	6,4	1,0	**0,30**	0
PITPNM3	PITPNM family member 3	5,0	0,8	**0,30**	4,00E-06
PDGFRB	platelet derived growth factor receptor beta	61,5	10,9	**0,30**	0
MGAT3	mannosyl (beta-1,4-)-glycoprotein beta-1,4-N-acetylglucosaminyltrans	4,8	1,1	**0,30**	1,00E-04
SLC44A3	solute carrier family 44 member 3	3,6	0,7	**0,30**	5,00E-06
LAMB1	laminin subunit beta 1	7,5	1,5	**0,30**	2,00E-06
RNF217-AS1	RNF217 antisense RNA 1 (head to head)	1,7	0,3	**0,30**	4,00E-06
CC2D2A	coiled-coil and C2 domain containing 2A	19,5	3,8	**0,30**	0
PRKCZ	protein kinase C zeta	62,8	10,6	**0,30**	0

CACNB3	calcium voltage-gated channel auxiliary subunit beta 3	7,5	1,4	**0,30**	1,00E-06
VWA1	von Willebrand factor A domain containing 1	203,8	40,6	**0,29**	1,00E-06
DACT1	dishevelled binding antagonist of beta catenin 1	23,5	3,9	**0,29**	1,00E-06
PODNL1	podocan like 1	108,5	23,1	**0,29**	0
ZBED3	zinc finger BED-type containing 3	5,5	1,0	**0,29**	0
HHIP-AS1	HHIP antisense RNA 1	1,1	0,1	**0,29**	0,001894
E2F1	E2F transcription factor 1	26,1	4,4	**0,29**	1,00E-06
CLEC18A	C-type lectin domain family 18 member A	1,5	0,3	**0,29**	0,001261
TSPAN11	tetraspanin 11	8,4	1,7	**0,29**	0
LRRC37A7P	leucine rich repeat containing 37 member A7, pseudogene	2,4	0,4	**0,29**	1,00E-06
DENND1B	DENN domain containing 1B	10,7	2,0	**0,29**	0
KCNJ15	potassium voltage-gated channel subfamily J member 15	6,4	1,3	**0,29**	0
RSPO2	R-spondin 2	129,0	25,1	**0,29**	0
CDK2AP1	cyclin dependent kinase 2 associated protein 1	172,6	32,5	**0,29**	0
TCEAL6	transcription elongation factor A like 6	3,5	0,6	**0,29**	6,60E-05
MIR99AHG	mir-99a-let-7c cluster host gene	10,6	1,8	**0,29**	0
RACGAP1	Rac GTPase activating protein 1	48,5	8,2	**0,29**	0
THBS2	thrombospondin 2	59,6	10,6	**0,29**	0
STK32B	serine/threonine kinase 32B	10,4	1,6	**0,29**	1,00E-06
NT5DC2	5'-nucleotidase domain containing 2	83,0	16,3	**0,29**	0
LINC00654	long intergenic non-protein coding RNA 654	5,1	0,9	**0,29**	0
SLC4A3	solute carrier family 4 member 3	6,7	1,1	**0,29**	2,00E-06
NPR2	natriuretic peptide receptor 2	152,4	26,4	**0,29**	0
GRIA2	glutamate ionotropic receptor AMPA type subunit 2	1,4	0,2	**0,29**	0,002317
JAZF1	JAZF zinc finger 1	32,7	6,2	**0,29**	0
LINC00702	long intergenic non-protein coding RNA 702	16,1	3,3	**0,29**	0
EPHX2	epoxide hydrolase 2	19,3	3,3	**0,29**	0
PHYHIP	phytanoyl-CoA 2-hydroxylase interacting protein	2,0	0,4	**0,29**	2,00E-05
CCDC106	coiled-coil domain containing 106	8,3	1,5	**0,29**	0
PREX2	phosphatidylinositol-3,4,5-trisphosphate dependent Rac exchange fac	3,3	0,5	**0,29**	4,40E-05
CCDC68	coiled-coil domain containing 68	2,1	0,3	**0,29**	2,80E-05
FSCN1	fascin actin-bundling protein 1	219,7	38,8	**0,29**	0
NIPSNAP3B	nipsnap homolog 3B	4,5	0,7	**0,29**	3,00E-06

SELENBP1	selenium binding protein 1	23,7	4,4	**0,29**	2,00E-06
BMPER	BMP binding endothelial regulator	18,7	2,9	**0,29**	0
WDR76	WD repeat domain 76	19,2	3,1	**0,29**	0
FRMD4B	FERM domain containing 4B	72,2	13,4	**0,29**	0
ADAM19	ADAM metallopeptidase domain 19	2,1	0,4	**0,29**	1,90E-05
RHOBTB3	Rho related BTB domain containing 3	127,3	22,8	**0,29**	0
FGFR3	fibroblast growth factor receptor 3	198,9	32,4	**0,29**	1,60E-05
COL1A2	collagen type I alpha 2 chain	634,5	121,2	**0,29**	1,00E-06
ENG	endoglin	649,6	117,1	**0,29**	0
ANKRD35	ankyrin repeat domain 35	15,5	2,8	**0,28**	0
TMEM25	transmembrane protein 25	26,2	4,8	**0,28**	0
SLC22A3	solute carrier family 22 member 3	3,0	0,5	**0,28**	1,00E-05
BIRC7	baculoviral IAP repeat containing 7	1,0	0,2	**0,28**	0,003753
AKAP6	A-kinase anchoring protein 6	10,1	2,0	**0,28**	0
KAZALD1	Kazal type serine peptidase inhibitor domain 1	42,0	7,1	**0,28**	0
CHST6	carbohydrate sulfotransferase 6	56,4	9,7	**0,28**	0
SPARC	secreted protein acidic and cysteine rich	2859,9	558,7	**0,28**	1,10E-05
EPHA3	EPH receptor A3	7,5	1,1	**0,28**	7,60E-05
COL5A2	collagen type V alpha 2 chain	799,3	150,9	**0,28**	0
SEMA3E	semaphorin 3E	103,6	18,7	**0,28**	0
TCF7L1	transcription factor 7 like 1	7,1	1,2	**0,28**	0
ZNF365	zinc finger protein 365	5,2	1,0	**0,28**	0
TSPOAP1	TSPO associated protein 1	7,6	1,2	**0,28**	0
XRCC2	X-ray repair cross complementing 2	2,9	0,6	**0,28**	0
P3H3	prolyl 3-hydroxylase 3	131,2	24,0	**0,28**	0
11.syys	septin 11			**0,28**	0
FRMPD3	FERM and PDZ domain containing 3	1,6	0,3	**0,28**	1,60E-05
NCAM1	neural cell adhesion molecule 1	18,0	2,8	**0,28**	0
GSG2	germ cell associated 2, haspin			**0,28**	1,90E-05
ORC1	origin recognition complex subunit 1	6,7	1,0	**0,28**	0
PDE3B	phosphodiesterase 3B	6,7	1,1	**0,28**	0
SRRM3	serine/arginine repetitive matrix 3	1,5	0,2	**0,28**	0,000169
ECM2	extracellular matrix protein 2	33,1	6,0	**0,28**	0

MEF2C	myocyte enhancer factor 2C	50,8	8,1	**0,28**	0
SPATA9	spermatogenesis associated 9	0,7	0,1	**0,28**	0,000243
ALPL	alkaline phosphatase, liver/bone/kidney	1,4	0,2	**0,28**	0,002936
PQLC2L	PQ loop repeat containing 2 like	0,9	0,1	**0,28**	9,00E-06
MND1	meiotic nuclear divisions 1	2,6	0,3	**0,28**	6,00E-06
ADARB1	adenosine deaminase, RNA specific B1	102,2	17,5	**0,28**	0
ANKH	ANKH inorganic pyrophosphate transport regulator	1086,5	177,7	**0,28**	0
AURKA	aurora kinase A	10,1	1,6	**0,28**	0
FBLN7	fibulin 7	38,2	7,4	**0,28**	0
RMI2	RecQ mediated genome instability 2	7,6	1,2	**0,28**	5,00E-06
SLC30A4	solute carrier family 30 member 4	22,0	3,8	**0,27**	0
TSPAN2	tetraspanin 2	21,6	3,7	**0,27**	0
LINC01703	long intergenic non-protein coding RNA 1703	0,7	0,1	**0,27**	2,00E-06
ARMC4	armadillo repeat containing 4	0,9	0,1	**0,27**	4,00E-06
LINC01915	long intergenic non-protein coding RNA 1915	3,8	0,6	**0,27**	6,00E-06
SMAD6	SMAD family member 6	103,5	18,1	**0,27**	0
STK26	serine/threonine kinase 26	246,0	39,8	**0,27**	0
S100B	S100 calcium binding protein B	106,5	18,1	**0,27**	0
DSCC1	DNA replication and sister chromatid cohesion 1	6,7	1,0	**0,27**	1,00E-06
WDR62	WD repeat domain 62	12,3	1,9	**0,27**	0
LRRTM2	leucine rich repeat transmembrane neuronal 2	0,7	0,1	**0,27**	0,000588
TRIL	TLR4 interactor with leucine rich repeats	0,9	0,1	**0,27**	1,80E-05
ADAMTS3	ADAM metallopeptidase with thrombospondin type 1 motif 3	3,9	0,7	**0,27**	0
PACSIN3	protein kinase C and casein kinase substrate in neurons 3	29,7	5,0	**0,27**	0
SPON2	spondin 2	18,1	2,9	**0,27**	3,00E-06
POSTN	periostin	73,0	17,8	**0,27**	4,30E-05
CENPH	centromere protein H	12,1	2,1	**0,27**	0
NDRG2	NDRG family member 2	37,4	5,6	**0,27**	0
FANCA	Fanconi anemia complementation group A	9,4	1,6	**0,27**	0
C12orf75	chromosome 12 open reading frame 75	55,2	9,3	**0,27**	0
TSPAN32	tetraspanin 32	2,3	0,4	**0,27**	0,000217
NRP1	neuropilin 1	166,3	25,7	**0,27**	0
COPZ2	coatomer protein complex subunit zeta 2	85,5	13,9	**0,27**	0

CALHM2	calcium homeostasis modulator 2	39,7	6,6	**0,27**	0
SLC7A8	solute carrier family 7 member 8	34,6	5,4	**0,27**	0
MAP7D3	MAP7 domain containing 3	143,6	24,1	**0,27**	0
SORL1	sortilin related receptor 1	22,3	3,4	**0,27**	0
CCNB1	cyclin B1	28,8	3,9	**0,27**	0
SOX8	SRY-box 8	90,6	13,9	**0,27**	1,00E-06
CDCA8	cell division cycle associated 8	14,2	2,0	**0,27**	0
GRAMD2	GRAM domain containing 2			**0,27**	5,00E-06
CAP2	CAP, adenylate cyclase-associated protein, 2 (yeast)	13,0	2,2	**0,27**	2,00E-06
CRYAB	crystallin alpha B	151,9	23,4	**0,26**	1,00E-06
GABRB1	gamma-aminobutyric acid type A receptor beta1 subunit	3,0	0,5	**0,26**	3,00E-05
SPTLC3	serine palmitoyltransferase long chain base subunit 3	5,7	1,0	**0,26**	7,00E-06
MAGED1	MAGE family member D1	317,9	53,0	**0,26**	0
DMPK	dystrophia myotonica protein kinase	97,4	15,8	**0,26**	0
CARD10	caspase recruitment domain family member 10	0,7	0,1	**0,26**	0,000121
SNTB2	syntrophin beta 2	80,6	13,2	**0,26**	0
SPTB	spectrin beta, erythrocytic	2,2	0,3	**0,26**	5,80E-05
LIPC	lipase C, hepatic type	0,8	0,1	**0,26**	9,30E-05
ELN	elastin	59,0	9,4	**0,26**	1,00E-06
CASP12	caspase 12 (gene/pseudogene)	1,6	0,2	**0,26**	1,00E-06
GINS2	GINS complex subunit 2	14,8	2,0	**0,26**	1,00E-06
TSPAN13	tetraspanin 13	47,9	7,3	**0,26**	1,00E-06
TMEM200B	transmembrane protein 200B	20,5	3,2	**0,26**	0
SPON1	spondin 1	27,1	6,1	**0,26**	1,80E-05
ZNF704	zinc finger protein 704	46,1	7,5	**0,26**	0
ZNF204P	zinc finger protein 204, pseudogene	5,0	0,9	**0,26**	0
ARRB1	arrestin beta 1	16,3	2,8	**0,26**	0
NEBL	nebulette	414,7	63,5	**0,26**	0
ANKRD65	ankyrin repeat domain 65	5,5	0,9	**0,26**	3,00E-06
PIEZO2	piezo type mechanosensitive ion channel component 2	39,5	5,9	**0,26**	0
UNC5C	unc-5 netrin receptor C	30,0	4,7	**0,26**	0
KIF2C	kinesin family member 2C	10,2	1,9	**0,26**	0
ARHGAP28	Rho GTPase activating protein 28	7,2	1,2	**0,26**	0

ARHGEF39	Rho guanine nucleotide exchange factor 39	3,1	0,4	**0,26**	0
DKK1	dickkopf WNT signaling pathway inhibitor 1	4,5	0,5	**0,26**	0,000241
CCDC158	coiled-coil domain containing 158	0,8	0,1	**0,26**	0
IQGAP3	IQ motif containing GTPase activating protein 3	34,3	5,3	**0,26**	0
GALK1	galactokinase 1	16,5	2,6	**0,26**	0
RTKN2	rhotekin 2	0,7	0,1	**0,26**	0,000403
EFEMP1	EGF containing fibulin like extracellular matrix protein 1	427,9	71,2	**0,26**	0
PRTFDC1	phosphoribosyl transferase domain containing 1	7,6	1,2	**0,25**	0
SPOCK3	SPARC/osteonectin, cwcv and kazal like domains proteoglycan 3	5,8	1,1	**0,25**	6,80E-05
FADS2	fatty acid desaturase 2	108,6	19,6	**0,25**	1,00E-05
BAALC	brain and acute leukemia, cytoplasmic	22,0	3,6	**0,25**	0
GALNT8	polypeptide N-acetylgalactosaminyltransferase 8	36,6	5,3	**0,25**	0
CKAP2	cytoskeleton associated protein 2	36,7	5,5	**0,25**	0
RAMP2	receptor activity modifying protein 2	2,0	0,3	**0,25**	6,70E-05
CENPM	centromere protein M	6,5	1,0	**0,25**	0
CYR61	cysteine rich angiogenic inducer 61		388,9	**0,25**	1,00E-05
SYNGR1	synaptogyrin 1	13,3	2,2	**0,25**	0
RAD51AP1	RAD51 associated protein 1	7,0	0,9	**0,25**	0
TRIM6	tripartite motif containing 6	3,7	0,5	**0,25**	4,10E-05
CYP19A1	cytochrome P450 family 19 subfamily A member 1	16,1	2,4	**0,25**	2,00E-06
NUP210	nucleoporin 210	2,3	0,3	**0,25**	0
SV2B	synaptic vesicle glycoprotein 2B	2,2	0,3	**0,25**	0
COX7A1	cytochrome c oxidase subunit 7A1	42,4	6,9	**0,25**	0
CMKLR1	chemerin chemokine-like receptor 1	86,4	12,9	**0,25**	0
ACP5	acid phosphatase 5, tartrate resistant	3,1	0,5	**0,25**	0
IL11RA	interleukin 11 receptor subunit alpha	12,0	1,9	**0,25**	3,00E-06
HSPB7	heat shock protein family B (small) member 7	9,8	1,4	**0,25**	1,00E-06
SMARCD3	SWI/SNF related, matrix associated, actin dependent regulator of chro	9,9	1,4	**0,25**	0
EME1	essential meiotic structure-specific endonuclease 1	4,5	0,6	**0,25**	0
ISM1	isthmin 1	450,9	66,6	**0,25**	0
COL5A1	collagen type V alpha 1 chain	815,1	123,3	**0,25**	0
HPN	hepsin	1,0	0,2	**0,25**	0
DLX6-AS1	DLX6 antisense RNA 1	8,9	1,4	**0,25**	0

FUOM	fucose mutarotase	8,0	1,3	**0,25**	1,00E-06
GALM	galactose mutarotase	12,8	1,9	**0,25**	0
PLXND1	plexin D1	57,8	8,7	**0,25**	0
PGGHG	protein-glucosylgalactosylhydroxylysine glucosidase	18,4	3,1	**0,25**	0
FLNB	filamin B	126,4	18,4	**0,25**	0
PLCD3	phospholipase C delta 3	183,9	26,9	**0,25**	0
CD79B	CD79b molecule	5,8	0,8	**0,25**	1,00E-06
CD4	CD4 molecule	5,7	0,9	**0,25**	4,00E-06
CMBL	carboxymethylenebutenolidase homolog	17,5	2,8	**0,24**	0
LRRC34	leucine rich repeat containing 34	2,0	0,3	**0,24**	1,00E-06
PLCE1	phospholipase C epsilon 1	13,5	2,2	**0,24**	1,00E-06
COL27A1	collagen type XXVII alpha 1 chain	257,8	34,4	**0,24**	0
SEMA3D	semaphorin 3D	90,4	12,8	**0,24**	0
GDF5	growth differentiation factor 5	50,2	7,6	**0,24**	0
GSTM2	glutathione S-transferase mu 2	3,8	0,6	**0,24**	0
ENAH	enabled homolog (Drosophila)	237,1	36,8	**0,24**	0
RFLNA	refilin A	48,2	5,8	**0,24**	3,00E-06
RAET1E	retinoic acid early transcript 1E	4,2	0,6	**0,24**	0
TAS1R1	taste 1 receptor member 1	0,8	0,1	**0,24**	0,000752
REXO5	RNA exonuclease 5	6,2	0,7	**0,24**	0
ABCB4	ATP binding cassette subfamily B member 4	3,8	0,6	**0,24**	1,20E-05
LRRN2	leucine rich repeat neuronal 2	13,0	2,0	**0,24**	0
IFITM2	interferon induced transmembrane protein 2	88,7	13,0	**0,24**	0
GAS2L3	growth arrest specific 2 like 3	5,0	0,7	**0,24**	0
CRACR2B	calcium release activated channel regulator 2B	14,7	1,8	**0,24**	0
MEF2C-AS1	MEF2C antisense RNA 1	1,4	0,2	**0,24**	2,00E-06
TPM2	tropomyosin 2 (beta)	131,0	20,2	**0,24**	0
HELLS	helicase, lymphoid-specific	13,1	1,7	**0,23**	0
ZWINT	ZW10 interacting kinetochore protein	24,9	3,2	**0,23**	0
PTGS1	prostaglandin-endoperoxide synthase 1	38,2	6,1	**0,23**	0
CYGB	cytoglobin	31,1	4,1	**0,23**	0
CACTIN-AS1	CACTIN antisense RNA 1	0,7	0,1	**0,23**	5,00E-06
TMPO-AS1	TMPO antisense RNA 1	3,7	0,6	**0,23**	0

MALRD1	MAM and LDL receptor class A domain containing 1	0,6	0,1	**0,23**	0,000295
CCDC110	coiled-coil domain containing 110	2,7	0,4	**0,23**	0
ANGPT2	angiopoietin 2	4,0	0,5	**0,23**	0
CARNS1	carnosine synthase 1	2,5	0,3	**0,23**	2,60E-05
TUBB2B	tubulin beta 2B class IIb	26,6	3,3	**0,23**	0
NID2	nidogen 2	11,1	1,5	**0,23**	1,00E-06
ARHGEF4	Rho guanine nucleotide exchange factor 4	2,3	0,3	**0,23**	3,60E-05
ACOT11	acyl-CoA thioesterase 11	6,1	0,9	**0,23**	0
ACADL	acyl-CoA dehydrogenase, long chain	3,9	0,6	**0,23**	0
POLE2	DNA polymerase epsilon 2, accessory subunit	4,4	0,5	**0,23**	0
CLEC3A	C-type lectin domain family 3 member A	39,8	5,8	**0,23**	3,00E-06
EFNB3	ephrin B3	1,8	0,3	**0,23**	5,10E-05
SLC22A11	solute carrier family 22 member 11	0,7	0,1	**0,23**	0,001485
ARHGAP33	Rho GTPase activating protein 33	7,0	1,0	**0,23**	0
PSRC1	proline and serine rich coiled-coil 1	11,4	1,5	**0,23**	0
P2RY6	pyrimidinergic receptor P2Y6	19,9	3,2	**0,22**	3,00E-06
PDZK1	PDZ domain containing 1	1,1	0,1	**0,22**	7,00E-06
RIMS1	regulating synaptic membrane exocytosis 1	4,5	0,6	**0,22**	1,00E-06
FES	FES proto-oncogene, tyrosine kinase	6,7	0,8	**0,22**	0
SCN1B	sodium voltage-gated channel beta subunit 1	52,2	7,1	**0,22**	0
ROM1	retinal outer segment membrane protein 1	16,9	2,3	**0,22**	0
GINS1	GINS complex subunit 1	6,9	0,8	**0,22**	0
RUBCNL	RUN and cysteine rich domain containing beclin 1 interacting protein l	18,7	2,4	**0,22**	0
TMEM176A	transmembrane protein 176A	2,6	0,3	**0,22**	6,90E-05
FAM149A	family with sequence similarity 149 member A	4,4	0,5	**0,22**	1,50E-05
GRID2IP	Grid2 interacting protein	1,2	0,1	**0,22**	2,00E-06
SYNE3	spectrin repeat containing nuclear envelope family member 3	43,8	5,8	**0,22**	0
TNS2	tensin 2	173,7	22,5	**0,22**	0
SCUBE1	signal peptide, CUB domain and EGF like domain containing 1	337,0	46,8	**0,22**	0
CDCA3	cell division cycle associated 3	14,7	1,7	**0,22**	0
MDFI	MyoD family inhibitor	97,4	12,0	**0,22**	0
MYO5C	myosin VC	16,8	2,1	**0,22**	0
LMNB1	lamin B1	18,0	2,0	**0,21**	0

LNX1	ligand of numb-protein X 1	37,8	4,7	**0,21**	0
HOGA1	4-hydroxy-2-oxoglutarate aldolase 1	1,3	0,2	**0,21**	3,00E-06
ERG	ERG, ETS transcription factor	104,1	12,4	**0,21**	0
INSC	inscuteable homolog (Drosophila)	5,9	0,6	**0,21**	1,00E-06
EFCAB1	EF-hand calcium binding domain 1	0,8	0,2	**0,21**	0
NFIA	nuclear factor I A	50,3	6,3	**0,21**	0
ABHD1	abhydrolase domain containing 1	0,5	0,0	**0,21**	6,10E-05
SEPT5	septin 5			**0,21**	0
TLR5	toll like receptor 5	5,8	0,8	**0,21**	0
MARCH1	membrane associated ring-CH-type finger 1	2,8	1,9	**0,21**	7,00E-06
CCDC85A	coiled-coil domain containing 85A	16,7	2,1	**0,21**	0
RAC3	ras-related C3 botulinum toxin substrate 3 (rho family, small GTP bind	3,0	0,3	**0,21**	7,00E-06
PAX9	paired box 9	0,8	0,1	**0,21**	0,000219
C1QTNF2	C1q and TNF related 2	1,7	0,2	**0,21**	1,00E-06
E2F8	E2F transcription factor 8	2,0	0,3	**0,21**	0
TMEM37	transmembrane protein 37	1,0	0,1	**0,21**	0,000201
PRPH	peripherin	1,1	0,1	**0,21**	5,20E-05
MTUS2	microtubule associated scaffold protein 2	0,8	0,1	**0,21**	7,00E-06
PIMREG	PICALM interacting mitotic regulator	18,5	2,1	**0,21**	0
TACC3	transforming acidic coiled-coil containing protein 3	40,1	4,8	**0,20**	0
CDH11	cadherin 11	104,9	13,4	**0,20**	0
SELP	selectin P	14,0	1,7	**0,20**	0
NIPSNAP1	nipsnap homolog 1	30,8	3,7	**0,20**	0
KIF11	kinesin family member 11	26,9	2,9	**0,20**	0
TMC6	transmembrane channel like 6	2,9	0,3	**0,20**	8,00E-06
HSPB2	heat shock protein family B (small) member 2	0,8	0,1	**0,20**	5,00E-06
F2	coagulation factor II, thrombin	1,7	0,2	**0,20**	0
PCBP4	poly(rC) binding protein 4	14,9	2,0	**0,20**	0
GPD1	glycerol-3-phosphate dehydrogenase 1	4,5	0,5	**0,20**	0
PSTPIP1	proline-serine-threonine phosphatase interacting protein 1	26,3	2,9	**0,20**	0
STK32A	serine/threonine kinase 32A	7,2	0,7	**0,20**	1,00E-06
CENPU	centromere protein U	18,5	2,0	**0,20**	0
ZNF385B	zinc finger protein 385B	35,0	4,3	**0,20**	0

CENPE	centromere protein E	11,0	1,3	**0,20**	0
MTFR2	mitochondrial fission regulator 2	2,3	0,3	**0,20**	6,00E-06
TMEM176B	transmembrane protein 176B	2,8	0,4	**0,20**	1,60E-05
SNCG	synuclein gamma	10,6	1,2	**0,19**	0
PDE7B	phosphodiesterase 7B	21,5	2,4	**0,19**	0
GRIK4	glutamate ionotropic receptor kainate type subunit 4	0,7	0,1	**0,19**	1,20E-05
KCNE3	potassium voltage-gated channel subfamily E regulatory subunit 3	0,9	0,1	**0,19**	0,00037
MYL3	myosin light chain 3	1,5	0,2	**0,19**	6,20E-05
DOK1	docking protein 1	256,6	29,8	**0,19**	0
COMTD1	catechol-O-methyltransferase domain containing 1	7,0	0,7	**0,19**	0
GPRC5C	G protein-coupled receptor class C group 5 member C	108,5	12,1	**0,19**	0
MYL9	myosin light chain 9	23,9	2,4	**0,19**	0
CDCA5	cell division cycle associated 5	22,2	2,3	**0,19**	0
SDC1	syndecan 1	85,8	10,3	**0,19**	0
RIBC2	RIB43A domain with coiled-coils 2	1,9	0,2	**0,19**	0
SCN2A	sodium voltage-gated channel alpha subunit 2	0,6	0,0	**0,19**	0,00026
ANGPTL2	angiopoietin like 2	940,1	99,4	**0,19**	0
CDKN2C	cyclin dependent kinase inhibitor 2C	47,9	5,2	**0,18**	0
C2CD6	C2 calcium dependent domain containing 6	0,8	0,1	**0,18**	6,00E-06
SGO1	shugoshin 1	3,2	0,3	**0,18**	0
EXO1	exonuclease 1	6,1	0,6	**0,18**	0
RORC	RAR related orphan receptor C	13,4	1,2	**0,18**	0
GYG2	glycogenin 2	1,2	0,1	**0,18**	2,30E-05
SUSD4	sushi domain containing 4	1,3	0,1	**0,18**	0,000896
SBSPON	somatomedin B and thrombospondin type 1 domain containing	32,7	3,2	**0,18**	0
SGCD	sarcoglycan delta	92,5	9,8	**0,18**	0
DMC1	DNA meiotic recombinase 1	3,5	0,3	**0,18**	0
ADA2	adenosine deaminase 2	2,8	0,4	**0,18**	0,000104
ESCO2	establishment of sister chromatid cohesion N-acetyltransferase 2	7,8	1,0	**0,18**	0
TTC22	tetratricopeptide repeat domain 22	3,2	0,2	**0,18**	6,00E-06
PRC1	protein regulator of cytokinesis 1	1,9	0,2	**0,18**	5,00E-06
CYP39A1	cytochrome P450 family 39 subfamily A member 1	2,6	0,3	**0,18**	2,00E-06
TYMS	thymidylate synthetase	80,9	8,1	**0,18**	0

CSRNP3	cysteine and serine rich nuclear protein 3	1,2	0,1	**0,18**	7,30E-05
FANCI	Fanconi anemia complementation group I	28,0	3,1	**0,18**	0
SFN	stratifin	16,0	1,6	**0,18**	0
RRM2	ribonucleotide reductase regulatory subunit M2	37,8	4,8	**0,17**	0
NCAPH	non-SMC condensin I complex subunit H	7,4	0,8	**0,17**	0
SARDH	sarcosine dehydrogenase	4,1	0,4	**0,17**	0
DAAM2	dishevelled associated activator of morphogenesis 2	58,4	5,6	**0,17**	0
MAF	MAF bZIP transcription factor	168,7	17,9	**0,17**	0
LINC00517	long intergenic non-protein coding RNA 517	0,7	0,0	**0,17**	2,80E-05
FHL1	four and a half LIM domains 1	398,6	36,7	**0,17**	0
SOX12	SRY-box 12	13,4	1,5	**0,17**	0
FHOD3	formin homology 2 domain containing 3	3,4	0,2	**0,17**	2,00E-06
SLC16A14	solute carrier family 16 member 14	1,4	0,1	**0,17**	0,000117
PLK1	polo like kinase 1	14,0	1,2	**0,17**	0
GTSE1	G2 and S-phase expressed 1	14,1	1,4	**0,17**	0
LINC00900	long intergenic non-protein coding RNA 900	1,3	0,1	**0,16**	1,00E-06
PLEKHB1	pleckstrin homology domain containing B1	8,0	0,9	**0,16**	0
FGF7	fibroblast growth factor 7	4,4	0,4	**0,16**	3,00E-06
SYT2	synaptotagmin 2	0,7	0,0	**0,16**	0,000422
SLC14A1	solute carrier family 14 member 1 (Kidd blood group)	14,2	1,5	**0,16**	0
GAMT	guanidinoacetate N-methyltransferase	6,1	0,6	**0,16**	0
TROAP	trophinin associated protein	6,4	0,6	**0,16**	0
CDCA7	cell division cycle associated 7	1,6	0,1	**0,16**	0
MYBL2	MYB proto-oncogene like 2	3,7	0,3	**0,16**	0
GPM6B	glycoprotein M6B	6,4	0,6	**0,16**	0
COL2A1	collagen type II alpha 1 chain	17921,6	1974,9	**0,16**	1,00E-05
POLQ	DNA polymerase theta	4,5	0,4	**0,16**	0
SMAD9	SMAD family member 9	31,8	2,9	**0,16**	0
COL11A1	collagen type XI alpha 1 chain	2404,8	225,2	**0,16**	0
GALNT5	polypeptide N-acetylgalactosaminyltransferase 5	46,3	4,3	**0,16**	0
DTL	denticleless E3 ubiquitin protein ligase homolog	17,1	1,6	**0,16**	0
IFITM1	interferon induced transmembrane protein 1	29,9	2,8	**0,16**	0
CENPF	centromere protein F	39,4	4,6	**0,16**	0

LOXL3	lysyl oxidase like 3	659,5	66,3	**0,16**	0
CRISPLD1	cysteine rich secretory protein LCCL domain containing 1	38,3	2,8	**0,16**	0
PRR11	proline rich 11	7,7	1,0	**0,16**	0
CDT1	chromatin licensing and DNA replication factor 1	17,2	1,6	**0,16**	0
F2R	coagulation factor II thrombin receptor	1,4	0,1	**0,16**	0
AQP7	aquaporin 7	6,6	0,5	**0,15**	0
KNL1	kinetochore scaffold 1	15,6	1,6	**0,15**	0
BRIP1	BRCA1 interacting protein C-terminal helicase 1	3,7	0,3	**0,15**	0
CENPA	centromere protein A	3,4	0,3	**0,15**	4,00E-06
ARHGDIB	Rho GDP dissociation inhibitor beta	68,1	6,1	**0,15**	0
STMN1	stathmin 1	56,8	6,4	**0,15**	0
SUSD3	sushi domain containing 3	1,4	0,1	**0,15**	0
OMD	osteomodulin	79,4	7,8	**0,15**	0
NME4	NME/NM23 nucleoside diphosphate kinase 4	34,6	3,2	**0,15**	0
SSC5D	scavenger receptor cysteine rich family member with 5 domains	38,1	3,8	**0,15**	0
RHOBTB1	Rho related BTB domain containing 1	21,3	1,9	**0,15**	0
EXTL1	exostosin like glycosyltransferase 1	49,8	4,4	**0,15**	0
ERCC6L	ERCC excision repair 6 like, spindle assembly checkpoint helicase	4,9	0,5	**0,15**	0
NYNRIN	NYN domain and retroviral integrase containing	30,5	2,7	**0,15**	0
PDGFD	platelet derived growth factor D	47,3	3,7	**0,15**	0
ITPRIPL1	inositol 1,4,5-trisphosphate receptor interacting protein-like 1	3,0	0,2	**0,15**	0
PTH1R	parathyroid hormone 1 receptor	22,7	2,0	**0,15**	0
GABRA4	gamma-aminobutyric acid type A receptor alpha4 subunit	67,4	6,2	**0,15**	0
LMO7	LIM domain 7	61,9	5,3	**0,15**	0
CCNB2	cyclin B2	11,8	1,2	**0,15**	0
TRIP13	thyroid hormone receptor interactor 13	14,4	1,2	**0,15**	0
ITGA11	integrin subunit alpha 11	499,3	46,6	**0,15**	0
ATOH8	atonal bHLH transcription factor 8	131,1	11,1	**0,15**	0
RAD54L	RAD54 like (S. cerevisiae)	6,3	0,5	**0,15**	0
BEX3	brain expressed X-linked 3	68,4	6,2	**0,15**	0
HSPB1	heat shock protein family B (small) member 1	134,4	10,6	**0,15**	0
NUSAP1	nucleolar and spindle associated protein 1	34,0	3,3	**0,15**	0
HACD4	3-hydroxyacyl-CoA dehydratase 4	11,0	1,1	**0,14**	0

ZNF367	zinc finger protein 367	77,2	6,2	**0,14**	0
CYP1A1	cytochrome P450 family 1 subfamily A member 1	9,2	0,6	**0,14**	0,000176
CYS1	cystin 1	26,1	2,0	**0,14**	0
CMTM5	CKLF like MARVEL transmembrane domain containing 5	0,8	0,1	**0,14**	3,00E-05
GAS1RR	GAS1 adjacent regulatory RNA	1,3	0,1	**0,14**	0
UROC1	urocanate hydratase 1	1,8	0,1	**0,14**	9,00E-06
PCLAF	PCNA clamp associated factor	5,5	0,3	**0,14**	0
MXRA5Y	matrix remodeling associated 5, Y-linked (pseudogene)	1,0	0,1	**0,14**	1,00E-06
CENPI	centromere protein I	5,2	0,5	**0,14**	0
MXRA5	matrix remodeling associated 5	133,8	10,3	**0,14**	0
TMEM119	transmembrane protein 119	1,3	0,1	**0,14**	0
SMIM1	small integral membrane protein 1 (Vel blood group)	1,3	0,1	**0,13**	0
NEURL1B	neuralized E3 ubiquitin protein ligase 1B	7,0	0,6	**0,13**	0
UHRF1	ubiquitin like with PHD and ring finger domains 1	22,4	1,7	**0,13**	0
CDC45	cell division cycle 45	11,7	0,9	**0,13**	0
IFITM10	interferon induced transmembrane protein 10	13,0	0,9	**0,13**	0
LCTL	lactase like	4,6	0,4	**0,13**	0
ID1	inhibitor of DNA binding 1, HLH protein	288,8	26,2	**0,13**	0
KIF23	kinesin family member 23	27,6	2,3	**0,13**	0
RGN	regucalcin	4,1	0,3	**0,13**	0
SHC4	SHC adaptor protein 4	20,4	1,5	**0,13**	0
REM1	RRAD and GEM like GTPase 1	1,3	0,1	**0,13**	0
ALX4	ALX homeobox 4	2,7	0,2	**0,13**	0
MCM10	minichromosome maintenance 10 replication initiation factor	8,2	0,6	**0,13**	0
CDC25C	cell division cycle 25C	2,4	0,2	**0,13**	1,00E-06
CIT	citron rho-interacting serine/threonine kinase	9,2	1,0	**0,13**	0
TK1	thymidine kinase 1	43,6	3,7	**0,13**	0
FAM111B	family with sequence similarity 111 member B	14,9	1,2	**0,13**	0
NUF2	NUF2, NDC80 kinetochore complex component	9,3	0,8	**0,13**	0
ATRNL1	attractin like 1	23,6	1,8	**0,13**	0
SYT8	synaptotagmin 8	3,1	0,2	**0,13**	0
GFAP	glial fibrillary acidic protein	1,5	0,1	**0,12**	0,001291
COL1A1	collagen type I alpha 1 chain	70,6	6,0	**0,12**	0

LINC01478	long intergenic non-protein coding RNA 1478	1,1	0,1	**0,12**	3,50E-05
RTN4RL1	reticulon 4 receptor like 1	22,9	1,5	**0,12**	0
PLK4	polo like kinase 4	8,3	0,6	**0,12**	0
P2RX6	purinergic receptor P2X 6	4,9	0,3	**0,12**	0
ADGRG4	adhesion G protein-coupled receptor G4	1,2	0,1	**0,12**	4,00E-06
GSTM5	glutathione S-transferase mu 5	7,8	0,6	**0,12**	0
PDE5A	phosphodiesterase 5A	18,5	1,2	**0,12**	0
FAM46B	family with sequence similarity 46 member B			**0,12**	0
NCAPG	non-SMC condensin I complex subunit G	18,8	1,4	**0,11**	0
PTGIS	prostaglandin I2 synthase	40,0	3,2	**0,11**	1,00E-06
MELK	maternal embryonic leucine zipper kinase	16,6	1,4	**0,11**	0
NDC80	NDC80, kinetochore complex component	10,2	0,9	**0,11**	0
TTK	TTK protein kinase	7,7	0,7	**0,11**	0
C10orf107	chromosome 10 open reading frame 107			**0,11**	0
LINC00092	long intergenic non-protein coding RNA 92	0,8	0,0	**0,11**	6,00E-06
CCNA2	cyclin A2	23,7	1,7	**0,11**	0
SHISA3	shisa family member 3	32,4	2,1	**0,11**	0
UBE2C	ubiquitin conjugating enzyme E2 C	13,3	1,3	**0,11**	0
GAS1	growth arrest specific 1	181,2	11,9	**0,11**	0
LVRN	laeverin	50,7	2,6	**0,11**	0
TOX	thymocyte selection associated high mobility group box	2,2	0,1	**0,11**	0
C1QTNF8	C1q and TNF related 8	29,0	1,8	**0,10**	0
GLDN	gliomedin	282,6	17,4	**0,10**	0
KIF12	kinesin family member 12	0,6	0,0	**0,10**	1,70E-05
TPX2	TPX2, microtubule nucleation factor	55,3	4,0	**0,10**	0
HJURP	Holliday junction recognition protein	18,3	1,5	**0,10**	0
AURKB	aurora kinase B	10,4	0,8	**0,10**	0
MSMP	microseminoprotein, prostate associated	449,3	38,0	**0,10**	0
HSPB6	heat shock protein family B (small) member 6	23,1	1,2	**0,10**	0
SLC2A12	solute carrier family 2 member 12	11,4	0,5	**0,10**	0
NEK2	NIMA related kinase 2	8,0	0,6	**0,10**	0
GPRIN3	GPRIN family member 3	1,1	0,1	**0,10**	0
CDK1	cyclin dependent kinase 1	27,3	2,0	**0,10**	0

OLFML3	olfactomedin like 3	3,3	0,2	**0,09**	0
TNNT3	troponin T3, fast skeletal type	5,1	0,2	**0,09**	0
LINC01229	long intergenic non-protein coding RNA 1229	1,0	0,0	**0,09**	0
BUB1	BUB1 mitotic checkpoint serine/threonine kinase	22,2	1,5	**0,09**	0
HMMR	hyaluronan mediated motility receptor	21,1	1,6	**0,09**	0
ZFHX4-AS1	ZFHX4 antisense RNA 1	1,2	0,0	**0,09**	0,000411
SYT13	synaptotagmin 13	3,9	0,2	**0,09**	0
RIMBP2	RIMS binding protein 2	1,1	0,1	**0,09**	2,00E-06
NEIL3	nei like DNA glycosylase 3	2,7	0,1	**0,09**	0
SKA3	spindle and kinetochore associated complex subunit 3	9,4	0,7	**0,09**	0
SPC24	SPC24, NDC80 kinetochore complex component	6,1	0,4	**0,09**	0
PART1	prostate androgen-regulated transcript 1 (non-protein coding)	21,6	1,0	**0,09**	0
SSTR5-AS1	SSTR5 antisense RNA 1	41,5	2,0	**0,09**	0
CDC20	cell division cycle 20	29,7	1,7	**0,09**	0
KIF18B	kinesin family member 18B	8,5	0,6	**0,09**	0
ANLN	anillin actin binding protein	78,6	5,3	**0,09**	0
MRAP2	melanocortin 2 receptor accessory protein 2	44,2	1,7	**0,09**	0
DEPDC1	DEP domain containing 1	10,6	0,7	**0,09**	0
SSTR5	somatostatin receptor 5	20,2	0,9	**0,08**	0
ASPN	asporin	41,7	1,8	**0,08**	1,00E-06
THY1	Thy-1 cell surface antigen	82,9	3,7	**0,08**	0
SLC26A4	solute carrier family 26 member 4	40,2	2,0	**0,08**	0
OLFML2B	olfactomedin like 2B	10,2	0,4	**0,08**	0
ROPN1B	rhophilin associated tail protein 1B	0,8	0,0	**0,08**	7,00E-06
BUB1B	BUB1 mitotic checkpoint serine/threonine kinase B	10,9	0,6	**0,08**	0
FOXM1	forkhead box M1	41,9	2,7	**0,08**	0
WFDC1	WAP four-disulfide core domain 1	2,5	0,1	**0,08**	1,00E-06
SHCBP1	SHC binding and spindle associated 1	27,2	1,6	**0,08**	0
MAMDC2	MAM domain containing 2	1,8	0,1	**0,08**	0
CEP55	centrosomal protein 55	24,9	1,4	**0,08**	0
PKMYT1	protein kinase, membrane associated tyrosine/threonine 1	17,9	0,9	**0,08**	0
MFAP2	microfibrillar associated protein 2	3,6	0,2	**0,08**	0
NXPH3	neurexophilin 3	7,1	0,2	**0,08**	0

MKI67	marker of proliferation Ki-67	59,2	4,0	**0,08**	0
SPEG	SPEG complex locus	24,3	0,9	**0,07**	0
HHIP	hedgehog interacting protein	9,3	0,4	**0,07**	0
DLGAP5	DLG associated protein 5	9,9	0,7	**0,07**	0
SRPK3	SRSF protein kinase 3	2,7	0,1	**0,07**	0
ANK3	ankyrin 3	138,1	4,8	**0,07**	0
C1QTNF7	C1q and TNF related 7	2,3	0,1	**0,07**	3,40E-05
MXRA8	matrix remodeling associated 8	379,2	13,6	**0,07**	0
TOP2A	topoisomerase (DNA) II alpha	90,4	5,6	**0,07**	0
RANBP3L	RAN binding protein 3 like	13,2	0,4	**0,06**	0
RCAN2	regulator of calcineurin 2	26,0	0,8	**0,06**	0
DIAPH3	diaphanous related formin 3	22,5	1,3	**0,06**	0
SPAAR	small regulatory polypeptide of amino acid response	4,1	0,1	**0,06**	0
HTRA4	HtrA serine peptidase 4	1,0	0,0	**0,05**	1,10E-05
ASPM	abnormal spindle microtubule assembly	19,8	1,3	**0,05**	0
ATP8B4	ATPase phospholipid transporting 8B4 (putative)	4,3	0,1	**0,05**	0
WSCD2	WSC domain containing 2	70,4	1,7	**0,05**	0
GDF10	growth differentiation factor 10	84,1	2,1	**0,05**	0
TMEM26	transmembrane protein 26	14,0	0,3	**0,05**	0
FAM13C	family with sequence similarity 13 member C	1,4	0,0	**0,05**	0
KIF20A	kinesin family member 20A	23,3	1,0	**0,05**	0
BIRC5	baculoviral IAP repeat containing 5	28,1	1,4	**0,05**	0
PBK	PDZ binding kinase	24,3	1,0	**0,05**	0
CAPN6	calpain 6	5,1	0,1	**0,05**	0
SCN2B	sodium voltage-gated channel beta subunit 2	12,4	0,3	**0,04**	0
PGAM2	phosphoglycerate mutase 2	2,3	0,1	**0,04**	0,001228
H19	H19, imprinted maternally expressed transcript (non-protein coding)	149,2	2,2	**0,03**	0
HRCT1	histidine rich carboxyl terminus 1	33,6	0,6	**0,03**	0
LSP1	lymphocyte-specific protein 1	233,9	2,7	**0,02**	0
SAMD11	sterile alpha motif domain containing 11	42,5	0,5	**0,02**	0
LINC00163	long intergenic non-protein coding RNA 163	5,5	0,0	**0,02**	0
ACTC1	actin, alpha, cardiac muscle 1	26,2	0,2	**0,02**	0
