# Supplementary material for: Regulation of gene expression by MF63, a selective inhibitor of microsomal PGE synthase 1 (mPGES1) in human osteoarthritic chondrocytes
Source: Br J Pharmacol. 2020 Aug 10;177(18):4134–46. doi: 10.1111/bph.15142 (PMC7443472; doi:10.1111/bph.15142)
Supplement: Supplementary file 4 — Table S4. Supporting information [file BPH-177-4134-s004.pdf]

Gene	Name	mean (ctrl)	mean (IL1)	mean (IL1+MF63)	FC (IL1 vs ctrl)	adj. p (IL1 vs ctrl)	FC (IL1+MF63 vs IL1)	adj. p (IL1 vs ctrl)
IL1B	Interleukin 1 beta	0,1	560,9	435,4	**3477,01**	< 0.0001	**0,79**	< 0.0001
IL6	Interleukin 6	0,5	938,0	519,5	**1505,18**	< 0.0001	**0,54**	< 0.0001
IL1A	Interleukin 1 alpha	0,0	3,2	4,3	**169,06**	< 0.0001	**1,31**	0,00041
TNFSF18	TNF superfamily member 18	0,1	11,1	14,6	**134,48**	< 0.0001	**1,31**	3,30E-05
NOS2	Nitric oxide synthase 2	9,7	991,5	1610,4	**91,05**	< 0.0001	**1,64**	< 0.0001
CCL2	C-C motif chemokine ligand 2	6,2	276,0	164,1	**32,81**	< 0.0001	**0,59**	< 0.0001
SOX9	SRY-box 9	251,6	309,8	344,7	**1,41**	0,024	**1,11**	0,0017
COL2A1	Collagen type II alpha 1 chain	17921,6	1974,9	2107,2	**0,16**	0,00001	**1,07**	0,13
MMP12	Matrix metallopeptidase 12	0,1	2,1	2,4	**12,09**	< 0.0001	**1,15**	0,17
MMP1	Matrix metallopeptidase 1	699,8	9785,7	12184,4	**10,98**	2,00E-06	**1,26**	< 0.0001
MMP3	Matrix metallopeptidase 3	11316,9	109522,4	114571,3	**7,50**	1,20E-05	**1,04**	0,33
MMP9	Matrix metallopeptidase 9	0,3	1,4	2,1	**4,95**	0,000034	**1,47**	0,0054
MMP13	Matrix metallopeptidase 13	106,0	398,9	383,5	**3,18**	0,000077	**0,95**	0,12
DUSP1	Dual specificity phosphatase 1	110,0	155,2	141,9	**1,46**	0,0044	**0,92**	0,026
KLF9	Kruppel like factor 9	18,2	23,5	27,8	**1,37**	0,0055	**1,17**	7,20E-05
FOXO3	Forkhead box O3	54,5	76,0	90,0	**1,50**	0,0036	**1,19**	< 0.0001
SOD2	Superoxide dismutase 2	930,5	20577,6	23296,0	**15,96**	< 0.0001	**1,13**	0,00018
CX3CL1	C-X3-C motif chemokine ligand 1	4,5	97,8	106,8	**19,65**	< 0.0001	**1,14**	0,0083
PTGS2	Prostaglandin-endoperoxide synthase 2	33,4	565,0	477,6	**10,76**	< 0.0001	**0,84**	7,10E-05
PTGES2	Prostaglandin E synthase 2	32,6	70,7	68,1	**2,32**	< 0.0001	**0,97**	0,39
